# Identification and Replication of Loci Involved in Camptothecin-Induced Cytotoxicity Using CEPH Pedigrees

**DOI:** 10.1371/journal.pone.0017561

**Published:** 2011-05-05

**Authors:** Venita Gresham Watson, Alison Motsinger-Reif, Nicholas E. Hardison, Eric J. Peters, Tammy M. Havener, Lorraine Everitt, James Todd Auman, Daniel L. Comins, Howard L. McLeod

**Affiliations:** 1 Division of Pharmacotherapy and Experimental Therapeutics, Institute for Pharmacogenomics and Individualized Therapy, Eshelman School of Pharmacy, University of North Carolina at Chapel Hill, Chapel Hill, North Carolina, United States of America; 2 Department of Statistics, Bioinformatics Research Center, North Carolina State University, Raleigh, North Carolina, United States of America; 3 Department of Chemistry, North Carolina State University, Raleigh, North Carolina, United States of America; Texas A&M University, United States of America

## Abstract

To date, the Centre d'Etude Polymorphism Humain (CEPH) cell line model has only been used as a pharmacogenomic tool to evaluate which genes are responsible for the disparity in response to a *single* drug. The purpose of this study was demonstrate the model's ability to establish a specific pattern of quantitative trait loci (QTL) related to a shared mechanism for multiple structurally related drugs, the camptothecins, which are Topoisomerase 1 inhibitors. A simultaneous screen of six camptothecin analogues for *in vitro* sensitivity in the CEPH cell lines resulted in cytotoxicity profiles and orders of potency which were in agreement with the literature. For all camptothecins studied, heritability estimates for cytotoxic response averaged 23.1±2.6%. Nonparametric linkage analysis was used to identify a relationship between genetic markers and response to the camptothecins. Ten QTLs on chromosomes 1, 3, 5, 6, 11, 12, 16 and 20 were identified as shared by all six camptothecin analogues. In a separate validation experiment, nine of the ten QTLs were replicated at the significant and suggestive levels using three additional camptothecin analogues. To further refine this list of QTLs, another validation study was undertaken and seven of the nine QTLs were independently replicated for all nine camptothecin analogues. This is the first study using the CEPH cell lines that demonstrates that a specific pattern of QTLs could be established for a class of drugs which share a mechanism of action. Moreover, it is the first study to report replication of linkage results for drug-induced cytotoxicity using this model. The QTLs, which have been identified as shared by all camptothecins and replicated across multiple datasets, are of considerable interest; they harbor genes related to the shared mechanism of action for the camptothecins, which are responsible for variation in response.

## Introduction

Prior to the 1990s, the phenotypic-based drug discovery approach dominated the pharmaceutical industry. In this approach, small molecules were screened against cells, tissues, or even whole organisms for their ability to enhance or suppress a specific phenotype desired in humans. The apparent advantages of this method over the existing target-based drug discovery paradigm have resulted in a renewed interest in phenotypic screening. One of the greatest advantages of this approach is that it enables the discovery of novel therapeutic targets for a disease. Drugs are screened for a biological effect rather than perturbation of a single molecular target, linking chemistry with biology and driving the serendipitous discovery of numerous structures with novel mechanisms of action (MOA).

Despite the recent revival in phenotypic screening, there are noteworthy limitations which can create a considerable bottleneck in the drug discovery process. Mechanism elucidation following the identification of hits remains the most important weakness. A number of methods are being developed and optimized for mechanism elucidation; however, they are fraught with limitations which have been reviewed extensively elsewhere [1]. Since the typical phenotypic screening methods are unable to suggest key information about the mechanism of biologically active drugs, there is no way to distinguish between them other than by potency. Without a clear understanding of MOA, problems arise in lead optimization, drug safety, and efficacy. Structure activity relationship (SAR) studies for lead optimization become quite complicated with phenotypic screens. Binding to an unknown target can be influenced by cell absorption and transport, additional protein binding, secondary target interactions, drug metabolism, etc. These sites of drug loss can vary significantly within a series of structurally related drugs. Most current methods of mechanism elucidation are also unable to account for or convey changes in mechanism (ie primary and secondary targets) with changes in structure. As a result SAR patterns become difficult to interpret and use during lead optimization. Finally, when mechanism is unclear our ability to assess the risk of mechanism based toxicity, side effects associated with secondary targets, or lapses in efficacy is also quite limited.

Genetic and genomic methods which screen all possible targets of drugs of interest are being developed to surmount issues associated with target identification following phenotypic screens. These methods which simultaneously screen drugs for a desired biological effect and provide information about molecular targets and SAR patterns are rising as powerful tools in drug discovery and development. Some of the most prominent examples of this approach use the budding yeast *Saccharomyces cerevisiae* [2], [3] or human cancer cell lines [4], [5] as *in vitro* model systems. In both cases, inconsistencies in data between humans and the model are a significant drawback. An ideal genomic strategy would investigate drug activity in a normal healthy human model. Recently, an *ex vivo* familial genetic strategy involving lymphoblastoid cell lines (LCLs) derived from Centre d'Etude du Polymorphisme Humain (CEPH) reference pedigrees was employed to quantify the impact of genetics on drug response and to identify quantitative trait loci (QTLs) harboring genes critical to drug action [6], [7]. Here we asked whether this *ex vivo* familial genetics model could be used to establish specific patterns of QTLs related to a shared mechanism for a class of structurally related drugs.

The camptothecins were chosen as a model class of drugs to investigate for a number of reasons. Extensive efforts in medicinal chemistry have led to the generation of a large number of camptothecin derivatives. Two of these, topotecan and irinotecan, are being used in the clinic as antitumor agents, and many are in preclinical and clinical development. In spite of the identification of a number of analogs with improved therapeutic activity, (intrinsic and acquired) resistance and toxicity remain major limitations to camptothecin therapy. While extensively studied, the mechanisms of resistance and toxicity remain unclear [8]. In addition, though it is firmly established that the key molecular target of all of the camptothecins is Topoisomerase 1 (Top1), the post target interaction events responsible for antitumor activity are vague [9]. It is reasonable to suggest that a clearer understanding of the biochemical cascade associated with camptothecin cytotoxicity might lend answers to the questions surrounding mechanisms of activity, toxicity, and resistance. To this end, the CEPH model system was used to a) assess variation in response to the camptothecins across normal healthy human LCLs, b) evaluate the genetic contribution to variation in response and c) establish a pattern of multiple QTLs common to a class of drugs suggesting a shared mechanism of action.

## Results

### Variation in Camptothecin-Induced Cytotoxicity

**Figure 1:**
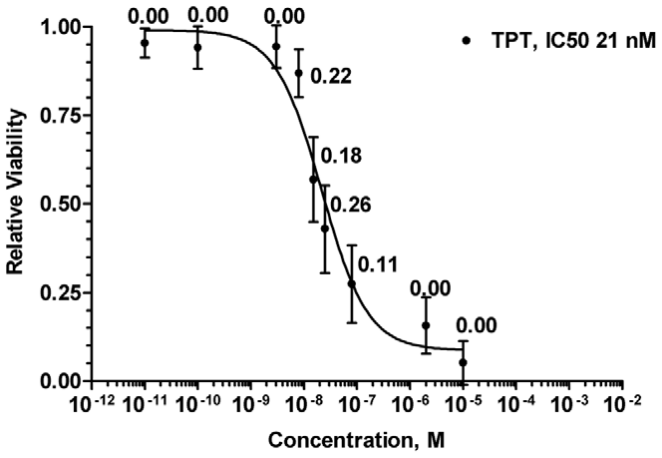
Representative dose–response curve for camptothecin analogues. Data points represent the overall population mean (n = 126) for growth inhibition relative to untreated controls at each concentration of topotecan. Vertical bars represent the standard deviation for cell viability across the population. Numbers are the growth-rate adjusted heritability estimates for each concentration. IC50 represents overall population IC50.

Sensitivity to the camptothecins was assessed in 125 lymphoblastoid cell lines derived from 14 CEPH pedigrees. Cells were exposed to increasing concentrations of each camptothecin (9 concentrations per drug) for 96 h and growth inhibition relative to vehicle control was determined. Variation in response to each camptothecin within and between the CEPH pedigrees was observed (Figure 1, Supplementary Figure S1). For example, 9AC, which had the widest range of IC50s, concentration required to inhibit growth by 50%, had a population mean IC50 of 93 nM and the IC50 ranged from 7 nM to 4 uM. Boxplots illustrating variation in cytotoxic response across the entire CEPH population for each drug are supplied in Dataset S3. Boxplots illustrating intra- and inter-family variability in response are provided in Dataset S4. Both the order of potency and IC50s in the CEPH cell lines are consistent with literature values in cancer cell lines such as the NCI60 cell line panel (NCI Developmental Therapeutics Human Tumor Cell Line Screening data, http://dtp.nci.nih.gov/dtpstandard/InvivoSummary/index.jsp [10].

The data was also used to identify individuals and/or families which were hypersensitive or resistant to the camptothecins. Further genetic and genomic studies with these individuals might lend insight into mechanisms of activity and resistance. A hierarchal clustering analysis of z-score transformed logIC50 values (where IC50 is the concentration required to inhibit viability by 50%) was performed keeping family structure intact or clustering on both drugs and family (Figure S2). The clusters matched the overall potency (SN38<CPT<9NC<TPT<9AC<CPT11) in the cell lines studied. CPT11 is most divergent from the other camptothecins studied (Figure S2). Since CPT11 is the prodrug of SN38 and requires submicromolar concentrations for effective cell kill, IC50s across the panel of CEPH cell lines are considerably higher for CPT11 than other camptothecins investigated. Of note, there are individuals who are sensitive to some but not all camptothecins and whole families which are resistant or sensitive to all camptothecins. For example, pedigree 1408 appears resistant to all camptothecins with the exception of 9AC. All but two members of pedigree 1362 are sensitive to all camptothecins; two offspring (11982 and 11983) are resistant to all camptothecins.

### Heritability Analysis

Heritability was estimated to quantify the impact of genetic factors on the cytotoxic response to each of the camptothecins at each concentration. There is a known correlation between cellular sensitivity to many chemotherapeutic agents and growth rate [11], [12]. As a control, heritability was calculated for growth rate in the presence of vehicle. The heritability estimate for growth rate was low (1.60%) which suggests that environmental factors play a much larger role than genetics in growth rate. For each camptothecin, the growth-rate adjusted heritability estimates at each concentration are featured in Figures 1 and S1. Heritability estimates at the asymptotes of the sigmoidal dose-response curve are low as there is little to no variability in cytotoxic response at these points. For all camptothecins studied heritability estimates averaged 23.1±2.6% for concentrations within the linear portion of the sigmoid curve. Since heritability estimates were approximately 20% for all camptothecins this reinforces the idea that inherited genetic variation is an important determinant of the cytotoxic response to camptothecins. The heritability associated with the cytotoxicity of these drugs is analogous to heritabilities reported for other common human phenotypes such as systolic and diastolic blood pressures [13], and for the cytotoxic response to daunorubicin in CEPH cell lines [14].

### Genome-Wide Linkage Analysis

Nonparametric linkage analysis was performed using mean growth inhibition (relative to a vehicle control) at each concentration for each camptothecin, which is referred to as the drug-dose phenotype. A complete set of QTL maps for each camptothecin, by chromosome can be found in Dataset S5. For each drug-dose phenotype statistically significant logarithm of odds (LOD) score thresholds corresponding to a p-values less than or equal to 0.05 were determined using gene-dropping permutations under the null hypothesis that no linkage exists. Regions of the genome referred to as quantitative trait loci (QTLs) were considered significant if the highest LOD score in the region was greater than or equal to the predetermined LOD score cut-offs for each drug-dose combination on a given chromosome. The mean LOD score cut-off across all phenotypes and chromosomes indicating significant linkage was 1.37 (range: 0.83–1.72). Additionally, cutoffs for suggestive linkage were determined for each drug-dose combination for an alpha of 0.05 for each chromosome. A region identified as significant in one drug-dose phenotype was considered replicated in another drug-dose phenotype if the maximum LOD score in that region surpassed the significant or suggestive LOD score threshold. The mean LOD score cut-off across all phenotypes and chromosomes indicating suggestive linkage was 0.59 (range: 0.41–0.72).

**Figure 2:**
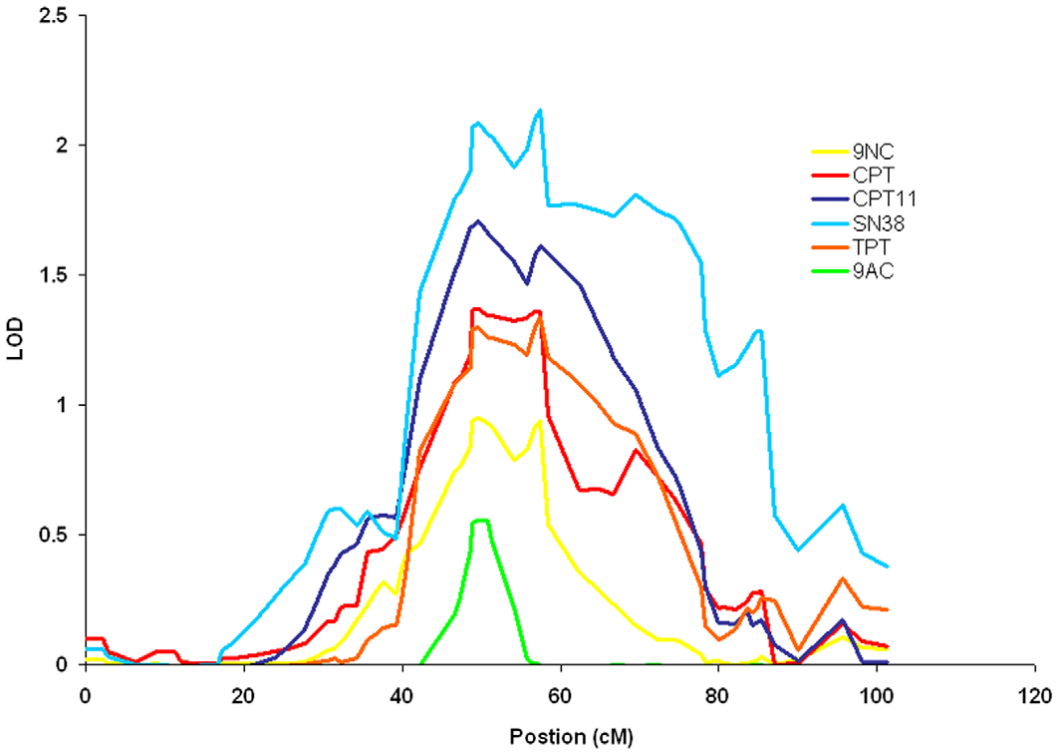
QTL shared across all camptothecins on chromosome 20. The QTL on chromosome 20 contains the gene for Top1, the sole molecular target of all camptothecins. Each drug is represented by a different color. Multiple concentrations for each drug were identified as significant and suggestive at this location. The drug-dose combinations with the highest LOD scores are represented here.

**Figure 3:**
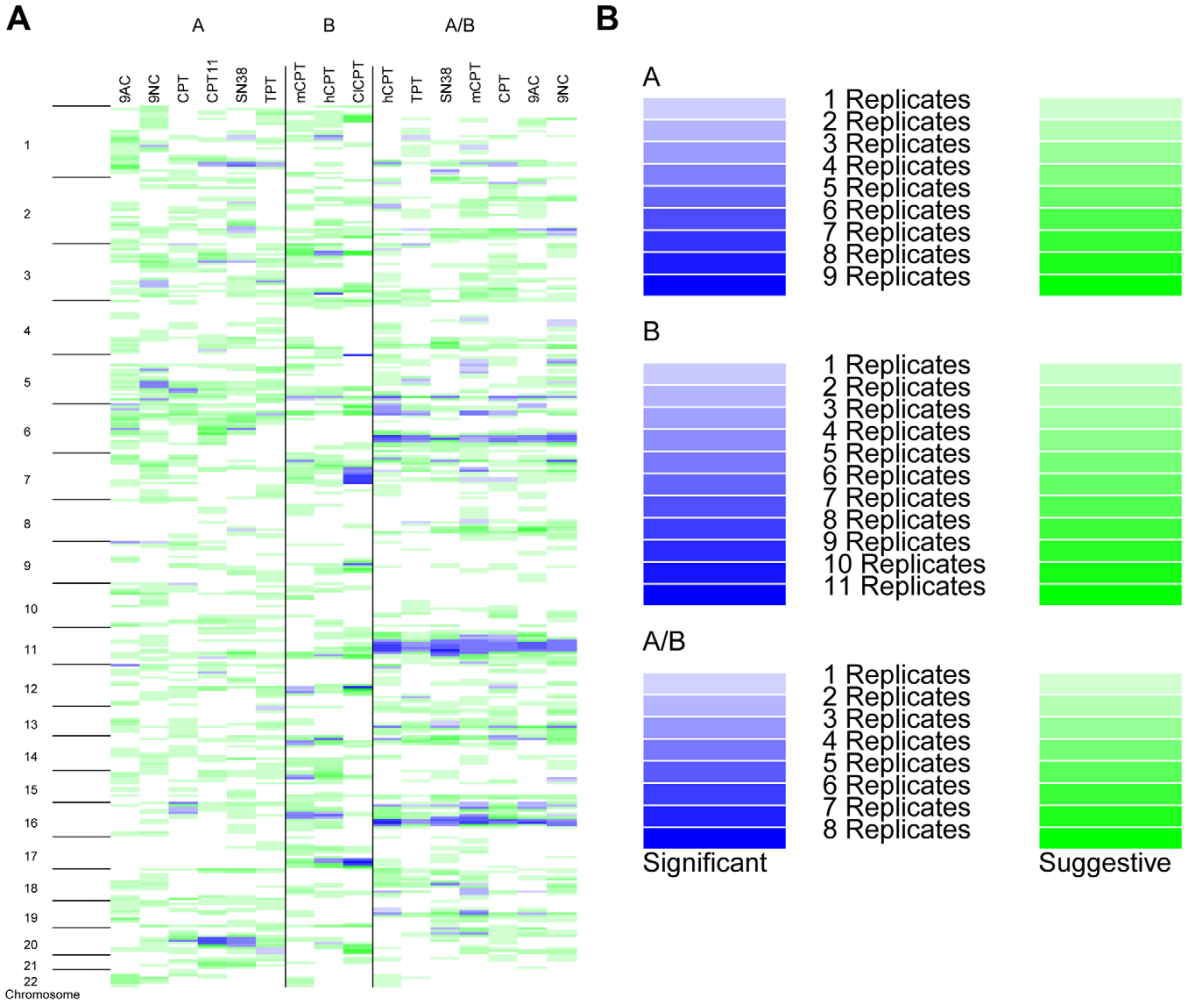
Genome wide pattern of QTLs for the camptothecins. **A.** Group A contains camptothecin analogues used in primary screen. Camptothecin analogues in Group B were used in the validation screen. Camptothecins in Group A/B were rerun from the primary and secondary screens. Each chromosome was partitioned into 10 cM regions. **B.** Each drug-dose combination that resulted in a significant QTL (LOD>threshold value) is indicated in blue. Intensity of the shading indicates the number of doses replicating that QTL at either the suggestive or significant level. Regions which also had a suggestive QTL (LOD>suggestive threshold) are indicated in green with color intensity referring to the number of doses replicating this peak.

To establish a pattern of QTLs significant to a class of drugs, regions of the genome which were overrepresented across the camptothecins were examined. Ten linkage peaks were initially identified as significant in a given drug-dose combination and replicated in all of the camptothecins at a number of concentrations (Table 1, Dataset S2). This implies that the same linkage regions influence the cytotoxic response to all camptothecins over a broad range of concentrations. The highest LOD score with genomic significance (2.13) was observed with the 8.0 nM SN38 phenotype and was located on chromosome 20 between 42 and 101 cM (20p12.1–20q13.32), and presumably associated with Top1 (56 cM, 20q12–q13.1), the primary target of the camptothecins. All camptothecin analogues studied (at multiple concentration for each drug) had a peak at chromosome 20 centered around 50 cM (Figure 2, Dataset S2). Unlike the other significant linkage peaks, the QTL on chromosome 6 from 0 to 29 cM is only associated with higher concentrations of the camptothecins which result in greater than 80% growth inhibition. Figure 3 illustrates significant and suggestive QTLs identified in one camptothecin which were replicated in other camptothecins. The results of a sign test (p<0.5) indicated there was a significant overrepresentation of overlapping QTLs compared to the null hypothesis that QTLs were randomly distributed across the genome amongst all drugs.

**Table 1:** QTLs shared by camptothecin analogues.

Chr	Peak Start (cM)	Peak End (cM)	LOD^b^
**1** a	**229**	**252**	**1.855**
3^d^	48	78	1.682
3^d^	148	180	1.638
**5**	**125**	**194**	**1.709**
**6**	**0**	**29**	**1.528**
**6**	**42**	**65**	**1.652**
**11**	**115**	**131**	**1.352**
12^c^	0	6	1.705
**16**	**0**	**75**	**1.345**
**20**	**42**	**101**	**2.134**

a Bolded QTLs were shared across all three validation sets.

b Maximum LOD score observed in this region.

c QTLs which were not replicated in Camptothecin Group B.

d QTLs which were not replicated in Camptothecin Group A/B.

Moreover, notable distinctions between significant QTLs associated with camptothecin analogues have been observed and are summarized in Figure 3. For example, TPT is the only camptothecin with a linkage peak extending from 0 to 19.6 cM on chromosome 13 (LOD = 1.365) (Table S3). Interestingly, 9NC is considered to be the prodrug of 9AC and there is one linkage peak which was identified exclusively in these drugs on chromosome 5 [15] (Table S3). Chromosome 1 has two QTLs centered at 70 and 129 cM respectively which are shared exclusively by camptothecins possessing a nitrogen bearing substituent on carbon 9: 9AC, 9NC, and TPT (Table S3). No peaks were identified which were unique solely to SN38 and its prodrug CPT11. However, a QTL on chromosome 4 is only present in CPT11 and 9AC. Regions suggested to influence the cytotoxic response to CPT11 were not always replicated in SN38 or vice versa. This was also observed for 9AC and 9NC. This is unsurprising since for example, the prodrug CPT11 must undergo activation by carboxylesterases (CESs) to the active SN38 and SN38 is not subsequently metabolized by CES. Only suggestive QTLs for CPT11 where located on chromosome 16 from 1–69 cM; CES1 and CES2 are centered around 73 cM on chromosome 16. Finally, to compare the overall QTL patterns a similarity matrix was constructed using a binary assessment of peaks present at either the significant or suggestive level for each camptothecin (summary list of peaks used for similarity matrix in Dataset S2). R squared correlations (r^2^) are bound by 0 and 1 and the greater the value the more related the pattern are to each other (Table S1). The majority of the correlations are above 0.5, indicating a strong association between overall QTL patterns for the camptothecins and suggests similar mechanisms of action. The highest correlations (highest degree of similarity) are between the 9AC and 9NC, CPT11 and CPT, and CPT11 and SN38. While the biological profile of CPT11 appears different from the remaining camptothecins, the genomic profile of TPT appears most distinct.

### Independent Validation of Shared QTLs

Ten QTLs were identified as shared across multiple drug-dose combinations of six camptothecin analogues (Camptothecin Group A: 9AC, 9NC, CPT, CPT11, SN38, and TPT). We next asked whether QTLs identified as shared among all camptothecins in Group A could be replicated independently in a set of 3 additional but distinct camptothecin analogues (Camptothecin Group B: mCPT, hCPT, ClCPT). In a separate validation experiment, the same 14 CEPH pedigrees were exposed to a dosing spectrum of Group B. Variation in sensitivity to this set of camptothecins was then used to calculate heritability estimates at each drug-dose phenotype. Just as with Group A, heritability estimates were highest for doses in the linear portion of the sigmoid curve. Growth rate adjusted heritability estimates for mCPT, hCPT, and ClCPT at these doses were comparable to estimates for the analogues belonging to Camptothecin Group A. The highest heritability estimates for mCPT, hCPT, and ClCPT were 20.2%, 18.7%, and 20.7% respectively. Linkage analysis, peak prioritization, and peak replication assessment were repeated with this second set of camptothecins. Nine of the ten QTLs identified as characteristic of camptothecin activity in Group A were subsequently validated in multiple doses of mCPT, hCPT, and ClCPT (Figure 3, Table 1). While no concentrations of mCPT or hCPT possessed the shared QTL on chromosome 6 from 0–29 cM, seven of the eleven doses of ClCPT possessed this shared QTL. Variation in response across the broad dosing spectrum for mCPT, hCPT, and ClCPT was not linked to the QTL on chromosome 12 from 0–6 cM.

This list of QTLs shared by the camptothecins was further refined by performing a third validation study with seven of the nine drugs from our initial study (Camptothecin Group A/B: mCPT, hCPT, 9AC, 9NC, SN38, CPT, TPT). Since Group A/B was evaluated using the same concentrations and in the same panel of CEPH cell lines, we consider this a technical replicate of our previously studies. Any QTLs which could not be replicated at the significant or suggestive level for all camptothecins within this separate validation step were excluded from further analysis.

Seven of the nine QTLs were replicated at the significant and suggestive level for Group A/B (Table 1, Figure 3). The peak on chromosome 16 was identified as significant in multiple concentrations of CPT just as previously reported. In fact, this QTL was replicated at the significance level in all camptothecins (for n≥1 concentrations) from Group A/B. The QTL on chromosome 11 was only present in two concentrations of CPT11 when studying Group A. In this replication step, it was present in all camptothecins at the significance level. Multiple concentrations of both SN38 and CPT11 (Group A) had QTLs on chromosome 20 which surpassed the significance LOD score thresholds in our earlier work. The QTL on chromosome 20 was replicated at the significance level in multiple doses of SN38 and at the suggestive level of all other camptothecin analogues in the Group A/B. CPT11 was not included in Camptothecin Group A/B. The QTL on chromosome 5, which was significant in multiple concentrations of analogues from Groups A and B, was also replicated at the significance level for multiple drug-dose combinations of Group A/B.

### Comparison to Topoisomerase 2 Inhibitors

To illustrate class specific patterns could be established, the same cell lines were phenotyped for sensitivity to the Topoisomerase 2 (Top2) inhibitors, etoposide and teniposide. Genetics plays a greater role in cytotoxic response to the Top2 inhibitors compared to Top1 inhibitors. The maximum heritability estimates for a Top1 inhibitor (topotecan, TPT) was 25.9%, compared to 42.4 and 32.9% for etoposide and teniposide respectively. IC50s were used to visualize patterns of sensitivity and resistance when comparing cytotoxic response to the camptothecins across the entire CEPH cell lines population. We chose another mode of comparison between the Top1 and Top2 inhibitors since IC50s could not be obtained for more than 80% of the cell lines treated with teniposide. Hierarchal clustering using the dose which yields a population mean growth inhibition of 50% for each drug reveals that overall patterns of sensitivity and resistance between the Top1 and Top2 inhibitors are indeed distinct and form two clusters corresponding to differences in mechanism (Figure S3). The same is true for the dose which yields a population mean growth inhibition of 40 and 60%.

Linkage analysis was performed using cell viability at each drug-dose combination of the Top2 inhibitors. Four QTLs present on chromosomes 6, 12, 13, and 18 were identified as significant and replicated (considered replicated if LOD>suggestive threshold) in both Top2 inhibitors at multiple dosages. This pattern of QTLs for the Top2 inhibitors was quite distinct from those established for the camptothecins (Figure 3). Unlike the camptothecins, no QTLs located on chromosome 17 and 3 (chromosomes that carry topoisomerase II alpha and beta genes, the targets of these inhibitors) were found. This may not be surprising. An earlier linkage analysis study of 5-fluoruracil toxicity in CEPH cell lines failed to identify a significant linkage peak on chromosome 18 around thymidylate synthetase (TYMS), the presumed primary target of 5FU [6]. In a subsequent association study using the same LCL samples and HAPMAP SNP data rather than the microsatellite data used for linkage analysis, SNPs variants encompassing the TYMS gene were subsequently identified as significantly associated with 5FU cytotoxicity in the CEPH cell lines [7]. The genotype density improved when going from the microsatellite markers used in the preliminary linkage analysis study of 5FU to the SNP data available for HAPMAP cell lines; the HAPMAP SNP genotype data enabled the detection of an association between 5-FU cytotoxicity and TYMS.

## Discussion

Early models for chemogenomic studies have used cancer cell lines [4], [5], mutant yeast strains [2], [3], and rodents [16], [17]. The biggest limitation with these systems is that the data does not always correlate to humans. For example, some mammalian targets are absent in yeast and vice versa. Targets which produce a desired phenotype in rodents may not exhibit the same phenotype in man [18]. In addition, cancer cell lines can differ morphologically and genetically from primary tissues [19].

This is one of the first genomic studies to use a healthy human cell line model to identify class specific pharmacological and genomic profiles. While cancer cell line panels such as the NCI60 are prepared from 4–5 cell lines of a given tissue origin, this study use a large collection of cell lines of the same type. Just as genetic heterogeneity across the cancer cell lines has been used to stratify drugs by mechanistic class, natural genetic variation in the CEPH cell lines can be used to identify a class specific profile for the camptothecins [4], [20]. In fact, heritability analysis demonstrates that 23.1±2.6% of human variation in sensitivity to the camptothecins is due to genetic components. Not only were these heritability estimates consistent across multiple concentrations, but they were consistent across multiple camptothecins analogues and experiments. Moreover, linkage results for camptothecin-induced cytotoxicity were replicated across multiple datasets. This finding reflects the advantage of performing linkage analysis using the CEPH cell lines over human subjects; cell lines can be grown & treated under identical conditions and experiments can be repeated multiple times with the same individuals. This is the first study to report replication of linkage results for drug-induced cytotoxicity using the CEPH cell lines.

Using this system to investigate drugs within a structural class and sharing the same mechanism one would expect a pattern of QTLs related to the cytotoxic activity of all drugs within that class. Furthermore, one would expect this pattern of QTLs to be reproducible across multiple CEPH phenotyping experiments. Indeed, ten QTLs across seven chromosomes were replicated in the first six camptothecin analogues studied suggesting a pattern of QTLs associated with a general and shared mechanism of action. We consider the fact that these QTLs were replicated across multiple analogues and doses within the first screen a form of internal validation. In a separate phenotyping experiment using three additional camptothecins, nine of those ten QTLs were again independently replicated. This list was further refined to seven QTLs which were replicated across multiple drug-dose combinations in a total three different screens. Finally, both the biological and genomic profiles generated in CEPH for the camptothecins and the Topoisomerase 2 inhibitiors, etoposide and teniposide were very distinct. Hierarchal clustering on biological data generated two clusters in agreement with the two distinct mechanisms of action. Moreover, the overall pattern of shared QTLs differed significantly between the two groups; no QTLs were present on the same chromosomes for the two classes.

Figure 3 highlights regions which might contain genes that contribute to the cytotoxic activity of all of the camptothecins. There are thousands of candidate genes for follow-up under the QTLs shared by all nine camptothecin analogues alone. Identifying which of these genes are critical to camptothecin-induced cytotoxicity can be a challenging and time-consuming process. To maximize success, a tiered approach is recommended when choosing QTLs for further investigation. QTLs shared by all nine camptothecins are considered the most promising (Table 1). QTLs shared by the first set of six camptothecins should be investigated next, followed by the QTLs identified as significant and shared by all three camptothecins in the validation set. Those significant QTLs which have been identified as unique to 1 or more drugs but are not replicated even at the suggestive level in all camptothecins should be considered next. Examples of this class include the QTLs on chromosome 1 at 70 and 129 cM, and the linkage peak on chromosome 13 (0–19 cM) that is observed solely with the 10 nM TPT phenotype. Finally, since the average LOD score threshold for a suggestive QTL is 0.59, suggestive QTLs present in all 9 camptothecins at multiple doses should be pursued last.

Using these prioritization criteria, of the QTLs identified in this study, the region on chromosome 20 is considered the most important for follow-up investigations. We used the functional annotation clustering tool from the web-accessible program Database for Annotation, Visualization, and Integrated Discovery (DAVID) to identify over-represented gene ontology terms (GO) and KEGG pathways for genes under each of the shared QTLs, including chromosome 20 [21], [22] (Table S2). The presence of Top1, the sole molecular target of all camptothecins, in this region is encouraging. Top1 expression levels have previously been correlated with cellular sensitivity to camptothecins; low levels of Top1 confer resistance to cancer cell lines such as lymphomas [9]. Smirnov et al. performed microarray experiments to measure human gene expression levels in CEPH [23] (data accessible at NCBI GEO database [24], accession GSE12626). Baseline measures of Top1 gene expression varied as much as 2 fold in this dataset. (Limited overlap between cell lines used in the studies prevented direct association analysis in the current study.) Admittedly, since linkage analysis produced a broad QTL spanning hundreds of genes, it cannot be assumed that a single gene under this QTL is influencing the activity of these drugs. Bcl-xl, is another promising gene within this region. Down-regulation of Bcl-xl, which inhibits apoptosis, has been shown to enhance cytotoxic response to the camptothecins [25], [26]. Association studies could be used to fine map this and other QTLs and pinpoint genes associated with drug response; however, limited statistical power prevents us from doing so here.

Observing significant or suggestive LOD scores for a given drug across a number of doses has been previously reported as replication and suggestive of a shared genetic component contributing to the cytotoxic effect at all concentrations [6], [14]. The same regions of interest were not identified as significant or suggestive for all drug-dose combinations of the camptothecins. In fact, some QTLs were apparent only in the higher concentrations of the camptothecins. For example, the QTL on chromosome 6 from 0 to 29 cM is only associated which is shared by all of the camptothecins was only significant and replicated at the highest concentrations of each analogue. The overrepresented GO terms and their associated genes under this QTL are listed in Table S2. One plausible explanation for changes in patterns of observed QTLs with differences in dose might be different mechanisms of action predominating at different concentrations. It has been reported that the anticancer activity of the camptothecins can switch from a replication-dependent to transcription-dependent process solely at higher concentrations in normal lymphoblasts and other highly proliferative cell lines [9]. Also different DNA repair, cell cycle checkpoint, and cell-death signaling pathways have been implicated following DNA damage at different doses [27], [28]. Without a doubt, there a number of complex mechanisms associated with the cytotoxic activity of the camptothecins that can occur simultaneously or selectively given certain intracellular conditions [29]. Work is ongoing to identify the conditions that dictate which pathways are preferred and why.

We have demonstrated that specific patterns of biological response and QTLs could be established for a class of structurally related drugs. When examining a drug class, slight changes in structure also resulted in differences in patterns of QTLs associated with cytotoxic response and drug action. To confirm the ability of this model to stratify drugs based on mechanistic of action, future studies should be undertaken with drugs from additional mechanistic classes. Biological and genomic profiling should again reveal patterns which are chemical and class specific. Moreover, as the ultimate goal of this research is correlate biological response to genes involved in drug action, work is needed to pinpoint the genes under these QTLs which are influencing response. Thousands of genes are present in the seven QTLs shared by all of the camptothecins. Recently, RNA interference (RNAi) screens in model organisms and human cell lines have successfully identified genes that modulate cell growth, apoptosis, chemoresistance, and chemosensitivity [30]–[34]. Large scale RNAi in the form of high throughput screens (HTS) using small interfering RNAs (siRNA) can be used to systematically screen all genes under the shared QTL. Known and novel genes whose loss of function confers alterations in sensitivity to the camptothecins can be identified. Taken together, these results lay the groundwork for using the *ex vivo* familial genetic strategy in CEPH cell lines for mechanism elucidation and drug development efforts.

## Materials and Methods

### Cell Lines

The CEPH cell lines are a set of immortalized lymphoblastoid cell lines collected from normal, healthy human volunteers which can be purchased from Corriell Cell Repositories (Camden, NJ). This collection is unique because the cell lines are established from large multigenerational families and every individual within the families has been genotyped, which enables investigators to perform genetics & pharmacogenomic analyses [6], [35], [36]. For the purposes of this study, all CEPH cell lines from the following family identification numbers were used (http://ccr.coriell.org/sections/collections/nigms/cephfamilies.aspx?PgId=49): 35, 45, 1334, 1340, 1341, 1345, 1350, 1362, 1408, 1420, 1447, 1451, 1454, 1459, 1463. The cells were cultured in RPMI 1640 supplemented with 10% fetal bovine serum at 37°C in humidified air containing 5% CO_2_ and passaged 2–3 times per week. Exponentially growing lymphoblastoid cell lines at passages 3–7 were used for experimentation.

### Drugs

The following camptothecin analogues (referred to as Camptothecin Group A) were purchased from LKT Labs (St Paul, MN): camptothecin (CPT), irinotecan (CPT11), 7-ethyl-10-hydroxycamptothecin (SN38), topotecan (TPT), 9-aminocamptothecin (9AC) and 9-nitrocamptothecin (9NC). Dr. Daniel Comins (North Carolina State University, Raleigh, NC) kindly provided the members of Camptothecin Group B: 10-methoxycamptothecin (mCPT), 10-hydroxycamptothecin (hCPT), and 7-chlorocamptothecin (ClCPT). All camptothecins were prepared in 10 mM working solutions of dimethyl sulfoxide (DMSO) (Sigma-Aldrich, St Louis, MO). Since camptothecins have a labile lactone form that exists in a pH dependent equilibrium with the inactive carboxy form (present at basic pH), drugs were serially diluted in citrate-phosphate buffer at pH 3. Final concentrations of DMSO were 0.1% in all experiments.

### Cytotoxicity Profiling

The cytotoxic effect of each panel of camptothecins was determined by using the nontoxic colorimetric-based assay, alamar blue [6]. Plates (384 well,Corning, Corning, NY) were preloaded with vehicle (citrate-PBS, 0.1% DMSO), 10% DMSO, and increasing concentrations of each drug (n = 9 concentrations per drug). Each plate contained 6 replicates for each drug-dose combination. Cells were then plated at a density of 4000 cells in 45 ul. Following 72 h incubation, 5 ul alamar blue was added. Fluorescence was read (Ex 535 nm and Em 595 nm) using a DTX880 plate reader (Beckman Coulter) at 96 h drug exposure. Raw fluorescence values for each set of replicates of a drug-dose combination were considered outliers if there was more than a ten-fold increase or decrease in the fluorescence signal of a single replicate. Growth inhibition relative to untreated controls was determined according to the manufacturer's protocol. The final percent growth inhibition at each concentration was averaged from six replicates of two independently plated experiments (n = 12). Additionally, growth rate in vehicle was calculated as previously described [12]. The IC50 (the dose needed to inhibit growth by 50%), was calculated based on a sigmoidal dose-response curve using the nls package in R (www.r-project.org) [37].

### Hierarchical Clustering

LogIC50s for each cell line-drug combination were z-score transformed prior to clustering. The data was loaded into Cluster 3.0 (http://bonsai.ims.u-tokyo.ac.jp/~mdehoon/software/cluster/) and clustered using uncentered correlation and complete linkage. To stabilize clusters, a self organizing map (SOM) was calculated using 100,000 iterations for cell lines and 20,000 iterations for drugs. Clusters were visualized using Java TreeView.

To compare the Top1 inhibitors to the Top2 inhibitors, the concentration closest to yielding a population mean of 50% was selected for each drug from the boxplot results provided in Dataset S3. Hierarchical clustering analysis of cytotoxic response to the Top1 and Top2 inhibitors was performed using the percent growth inhibition for each cell line at the concentration closest to yielding a population mean of 50% was selected for each drug.

### Heritability Analysis

Heritability estimates of the proportion of variation in cytotoxic response due to inherited factors were calculated using variance components analysis using MERLIN 1.1.2 (http://www.sph.umich.edu/csg/abecasis/Merlin/index.html) [38]. The degree of heritability associated with growth rate in vehicle was also calculated, and the heritability calculation for each drug-dose combination was adjusted using growth rate as a covariate in the variance components analysis [38].

### Genotype Data and Error Checking

Genotype data for each cell line were downloaded from V10 of the CEPH database (ftp://ftp.cephb.fr/ceph_genotype_db/ceph_db/Ver_10/mkr/) [39] using error checked markers. Genetic map information was downloaded from the Marshfield database (http://research.marshfieldclinic.org/genetics) [40]. Error checking for Mendelian incompatibility, misspecified relationships and unlikely recombinations was performed, as previously described [40]. A combined total of 8269 single nucleotide polymorphisms (SNPs) and microsatellite markers were used for linkage analysis.

### Genome-Wide linkage Analysis

Drug-dose combinations were considered the phenotypes of interest for linkage analysis (n = 54). For each phenotype, non-parametric linkage analysis was performed using MERLIN which constructs a likelihood ratio test for linkage based on inheritance vectors. For quantitative traits, scores used to calculate the likelihood ratio test are defined as follows:
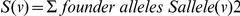


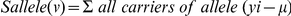
where S(ν) is the score for each inheritance vector, Sallele(ν) is the score for each founder allele, yi is the phenotype for each individual, μ is the mean phenotype for the population, and ν is the list of individuals who carry a specific founder allele such that the score for each inheritance vector is the summation of the squared score for each founder allele, and the score for each found allele is the sum of square deviation from all individuals that carry that allele. For each phenotype of interest, QTL maps were generated by displaying the logarithm of odds (LOD) scores from the likelihood ratio tests across each chromosome. The LOD score is a statistical estimate of linkage; it is the ratio of the likelihood that a chromosomal region is linked to the phenotype of interest over the likelihood that it is not. A LOD score of three indicates 1000 to 1 odds that the region is linked.

### Peak Identification

Guidelines for interpreting LOD scores have suggested viewing LOD scores of 2.2 as suggestive and 3.6 as significant [41]. However, since such a categorization is inexact, the data in this study was used to dictate at which threshold results would no longer be considered due to chance and most likely occur as a result of linkage. For each drug-dose phenotype, gene-dropping permutations were conducted using Merlin to get a distribution of LOD scores which would occur under the null hypothesis of no linkage to the observed drug-dose phenotypes [38]. Marker data were simulated under the null hypothesis of no linkage or association to the observed phenotypes while retaining the same pedigree structures, maps, marker allele frequencies, and missing data patterns. Ten thousand replicates were simulated for each of the 54 phenotypes, resulting in a total of 54,000 simulated datasets. Linkage analysis was conducted as described above for each replicate set. Based on these simulations, permutation distributions were generated across the chromosomes for each drug-dose phenotype and then used to determine genome-wide LOD score cut-offs corresponding to p-values less than or equal to .05 for each phenotype. Additionally, cutoffs for suggestive linkage were determined for each drug-dose combination for an alpha of 0.05 for each chromosome. A complete list of LOD score significant and suggestive cut-offs can be found in Dataset S1. QTLs observed for a drug-dose phenotype were considered significant if the highest LOD score in that region surpassed the significance LOD score threshold for that drug-dose phenotype. QTLs observed for a drug-dose phenotype were considered suggestive if the highest LOD score in that region surpassed the suggestive LOD score threshold for that drug-dose phenotype on that chromosome. Dataset S2 contains a list of QTLs identified as significant for drug-dose phenotypes as well as a those QTLs which are replicated within the panel of camptothecins.

## Supporting Information

Figure S1 **Dose–response curve for camptothecin analogues.** Data points represent the overall population mean (n = 126) for growth inhbition relative to untreated controls at each dose. Vertical bars represent the standard deviation for cell viability across the population. Numbers are the growth-rate adjusted heritability estimates for each concentration. IC50 represents overall population IC50.
(PDF)

Figure S2 **Hierarchal clustering of log transformed IC50s for camptothecins in CEPH cell lines.** Log IC50s were z-score transformed. Clustering based on drugs holding family structure intact. Yellow color indicates positive Z-scores (resistance), blue color indicates negative Z-scores (sensitive), black color indicates Z-score = 0 (median resistance value). The brighter the color the greater the value from 0, with max brightness set at 2.5. Black and white bar indicates family structure (n = 14 pedigrees).
(PDF)

Figure S3 **Differences in biological activity between Top1 and Top2 inhibitors in CEPH cell lines.** Hierarchal clustering of z-score transformed mean cell viabilities at the dose which yields population mean IC50. Clustered on both drugs and cell lines. Yellow color indicates positive Z-scores (resistance), blue color indicates negative Z-scores (sensitive), black color indicates Z-score = 0 (median resistance value). The brighter the color the greater the value from 0, with max brightness set at 2.5.
(PDF)

Table S1 **Similarity matrix of overall QTL patterns for each camptothecin.** To compare the overall QTL patterns between each of the camptothecin analogues a similarity matrix was constructed using a binary assessment of peaks present at either the significant or suggestive level for each camptothecin R squared correlations (r^2^) are bound by 0 and 1 and the greater the value the more related the overall QTL patterns are to each other.
(DOCX)

Table S2 **Genes under QTLs shared by camptothecins.** The functional annotation clustering tool from the web-accessible program Database for Annotation, Visualization, and Integrated Discovery (DAVID) was used to identify over-represented gene ontology terms (GO) and KEGG pathways for genes under each of the shared QTLs on chromosomes 1, 5, 11, 16 and 20. Genes of interest are listed by chromosomal location and then gene ontology term. The bolded gene names in Table S1 have previously been associated with camptothecin activity in yeast and/or mammalian cell lines.
(DOCX)

Table S3 **Genes under QTLs of interest for the camptothecins.** DAVID was used to identify over-represented gene ontology terms (GO) and KEGG pathways for genes located under the QTL on chromosome 1 for which a linkage with CPT bearing a nitrogen atom in position 9 is reported. Genes of interest are also listed for the QTL observed on chromosome 13 correlating uniquely with topotecan treatment and on chromosome 5 which is in linkage only with CPT11, 9AC and 9NC.
(DOCX)

Dataset S1 **LOD score thresholds.** **Gene** dropping permutations were used to identify LOD score thresholds for significant and suggestive linkage. LOD score cut-offs corresponding to a genome-wide p-values less than or equal to .05 for each drug at each dose were found, and used to define significant LOD score peaks. LOD score cut-offs corresponding to a genome-wide p-values less than or equal to .05 for each drug at each dose were found, and used to define significant LOD score peaks. LOD score cutoffs for suggestive peaks were defined as the minimum LOD score to achieve a p-value of 0.05 at each chromosome for each drug-dose phenotype. Significant and suggestive peak thresholds for each drug-dose combination on each chromosome as generated by permutation analysis are listed.
(XLS)

Dataset S2 **Details of significant, suggestive, and replicating peaks for camptothecins.** Tab 1 lists all significant QTLs by chromosome. The chromosome, the beginning and end of all peaks in centiMorgan (cM) units, and the peak LOD score in the region are listed. Additionally, drug-dose combinations which replicate the significant QTLs (LOD score>suggestive peak threshold at that location) are listed along with their maximum LOD score in that region (tab 2). As before, the chromosome, the beginning and end of all peaks in centiMorgan (cM) units, and the peak LOD score in the region are listed for each drug-dose combination. Drug-dose combinations with a significant peak are indicated in bold.
(XLS)

Dataset S3 **Boxplots illustrating variance in cell viability across the entire CEPH population (n = 125) for each drug.** Line represents mean phenotypic response, whiskers box represents upper and lower quartiles, and whiskers are 1.5*IQR. Outliers (circles) are individuals whose mean viability is greater than 1.5*IQR.
(PDF)

Dataset S4 **Boxplots illustrating intra- and inter-family variance in cell viability of each drug and dose across CEPH families.** Line represents mean phenotypic response, whiskers box represents upper and lower quartiles, and whiskers are 1.5*IQR. Outliers (circles) are individuals whose mean viability is greater than 1.5*IQR.
(PDF)

Dataset S5 **Genomewide QTL plots for each drug and dose.** For each drug and dose, LOD scores are shown across each chromosome.
(PDF)
