# Supplementary figures and images for: Identification and Replication of Loci Involved in Camptothecin-Induced Cytotoxicity Using CEPH Pedigrees
Source: PLoS One. 2011 May 5;6(5):e17561. doi: 10.1371/journal.pone.0017561 (PMC3088663; doi:10.1371/journal.pone.0017561)

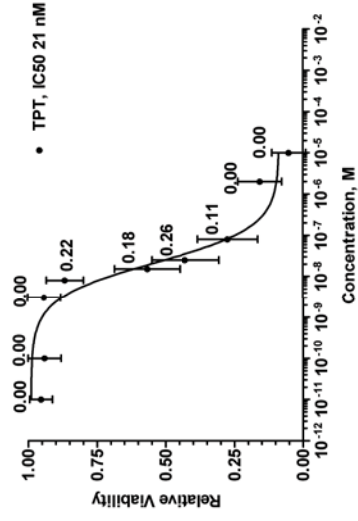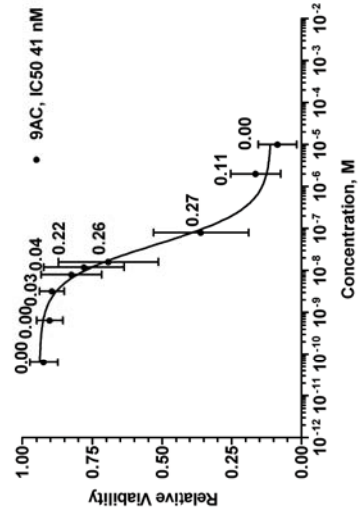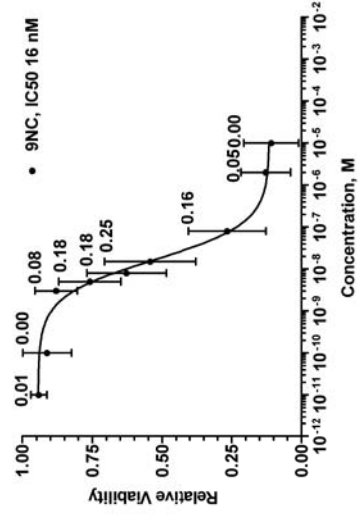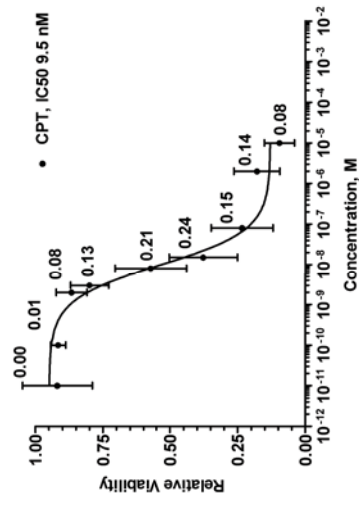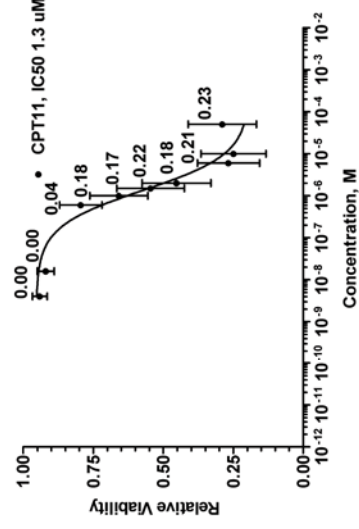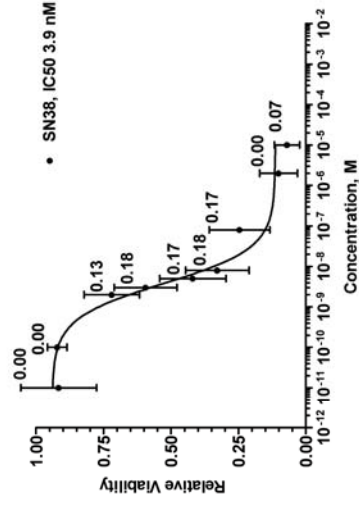

Supplement: Figure S1 — Dose–response curve for camptothecin analogues. Data points represent the overall population mean (n = 126) for growth inhbition relative to untreated controls at each dose. Vertical bars represent the standard deviation for cell viability across the population. Numbers are the growth-rate adjusted heritability estimates for each concentration. IC50 represents overall population IC50. (PDF) [file pone.0017561.s001.pdf]

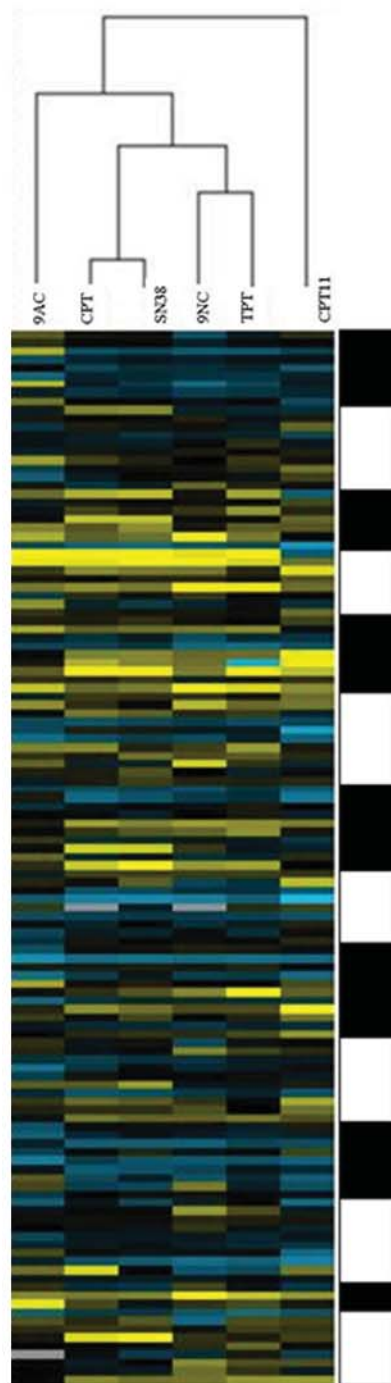

Supplement: Figure S2 — Hierarchal clustering of log transformed IC50s for camptothecins in CEPH cell lines. Log IC50s were z-score transformed. Clustering based on drugs holding family structure intact. Yellow color indicates positive Z-scores (resistance), blue color indicates negative Z-scores (sensitive), black color indicates Z-score = 0 (median resistance value). The brighter the color the greater the value from 0, with max brightness set at 2.5. Black and white bar indicates family structure (n = 14 pedigrees). (PDF) [file pone.0017561.s002.pdf]

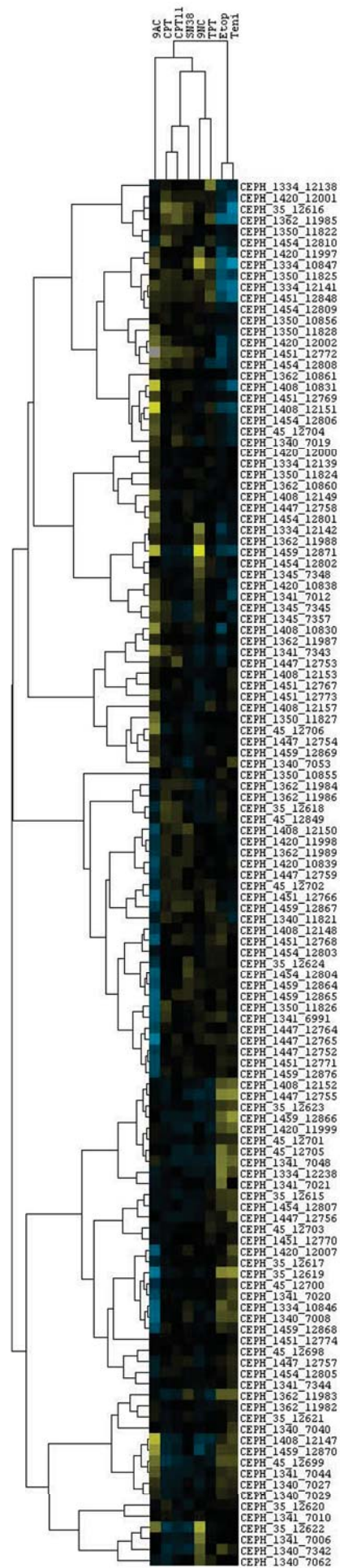

Supplement: Figure S3 — Differences in biological activity between Top1 and Top2 inhibitors in CEPH cell lines. Hierarchal clustering of z-score transformed mean cell viabilities at the dose which yields population mean IC50. Clustered on both drugs and cell lines. Yellow color indicates positive Z-scores (resistance), blue color indicates negative Z-scores (sensitive), black color indicates Z-score = 0 (median resistance value). The brighter the color the greater the value from 0, with max brightness set at 2.5. (PDF) [file pone.0017561.s003.pdf]

# Drug 9AC

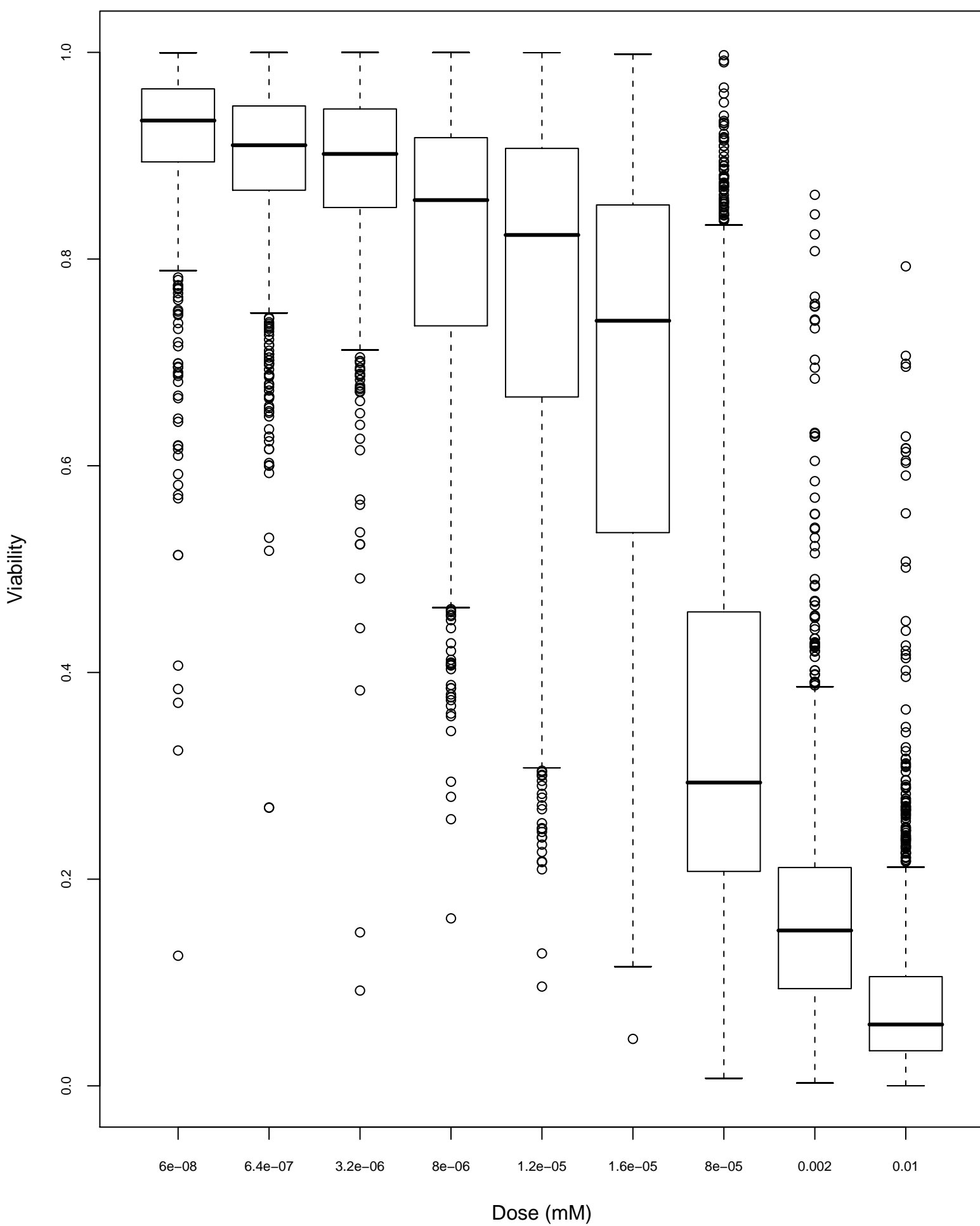

# Drug 9NC

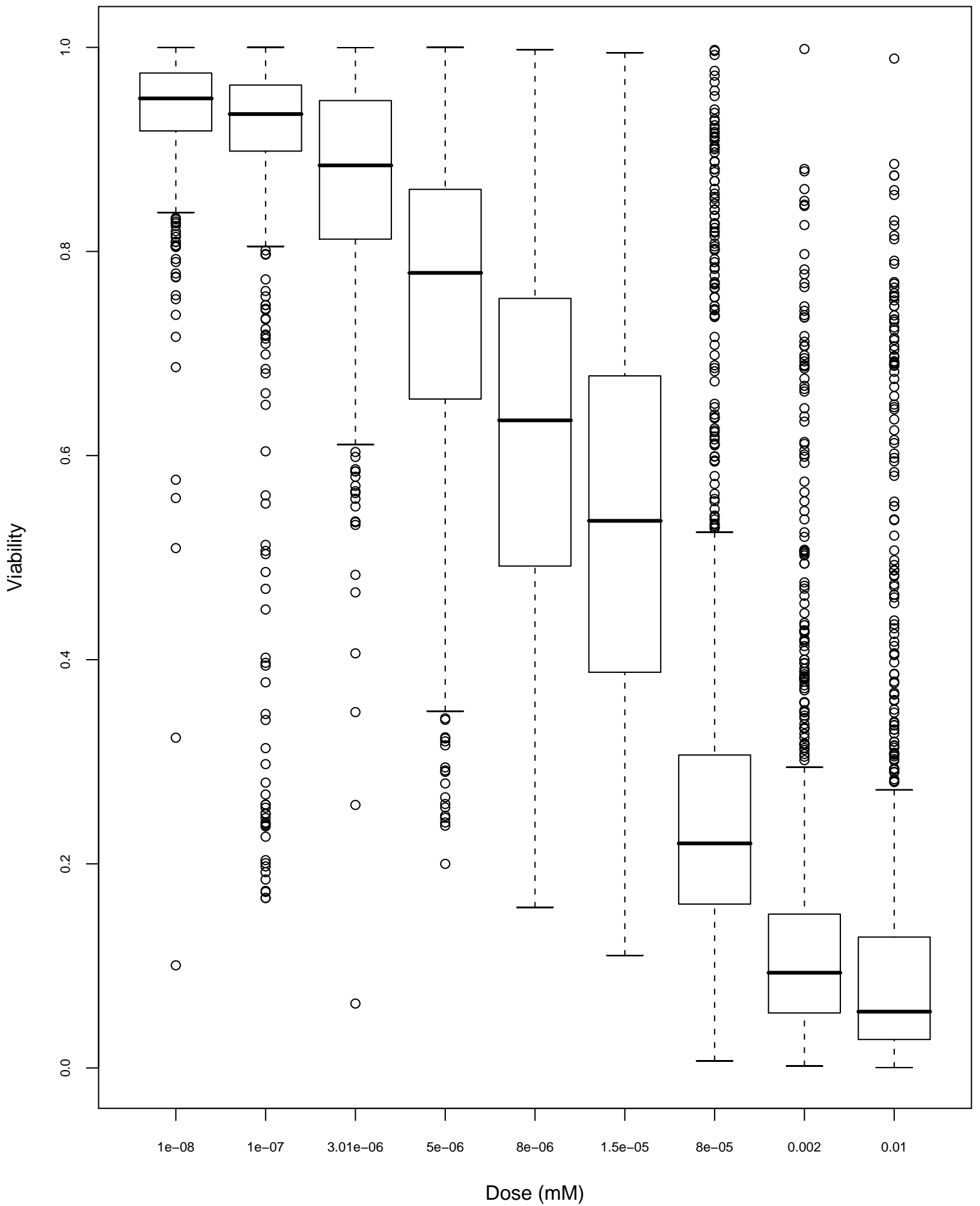

# Drug CPT

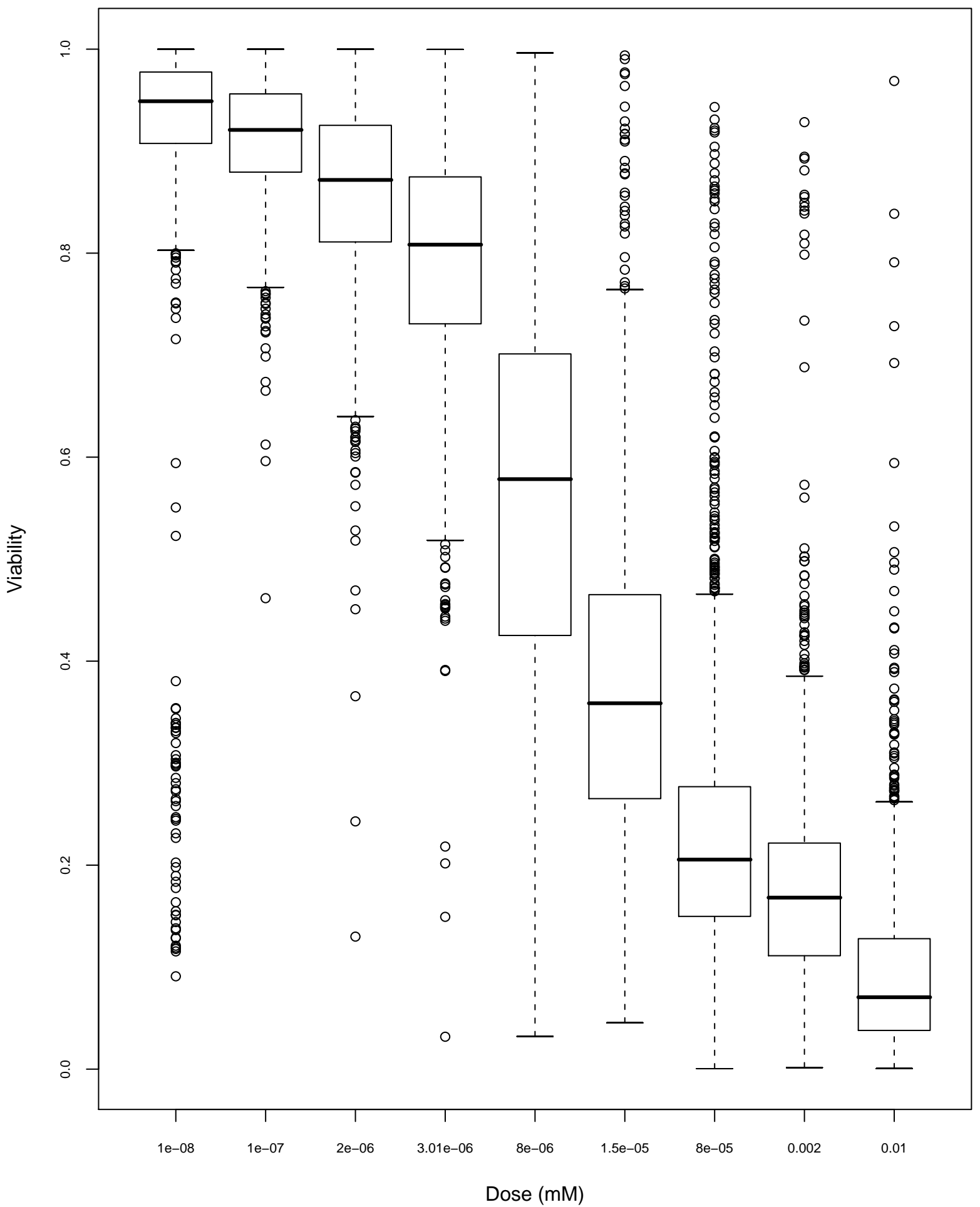

# Drug CPT11

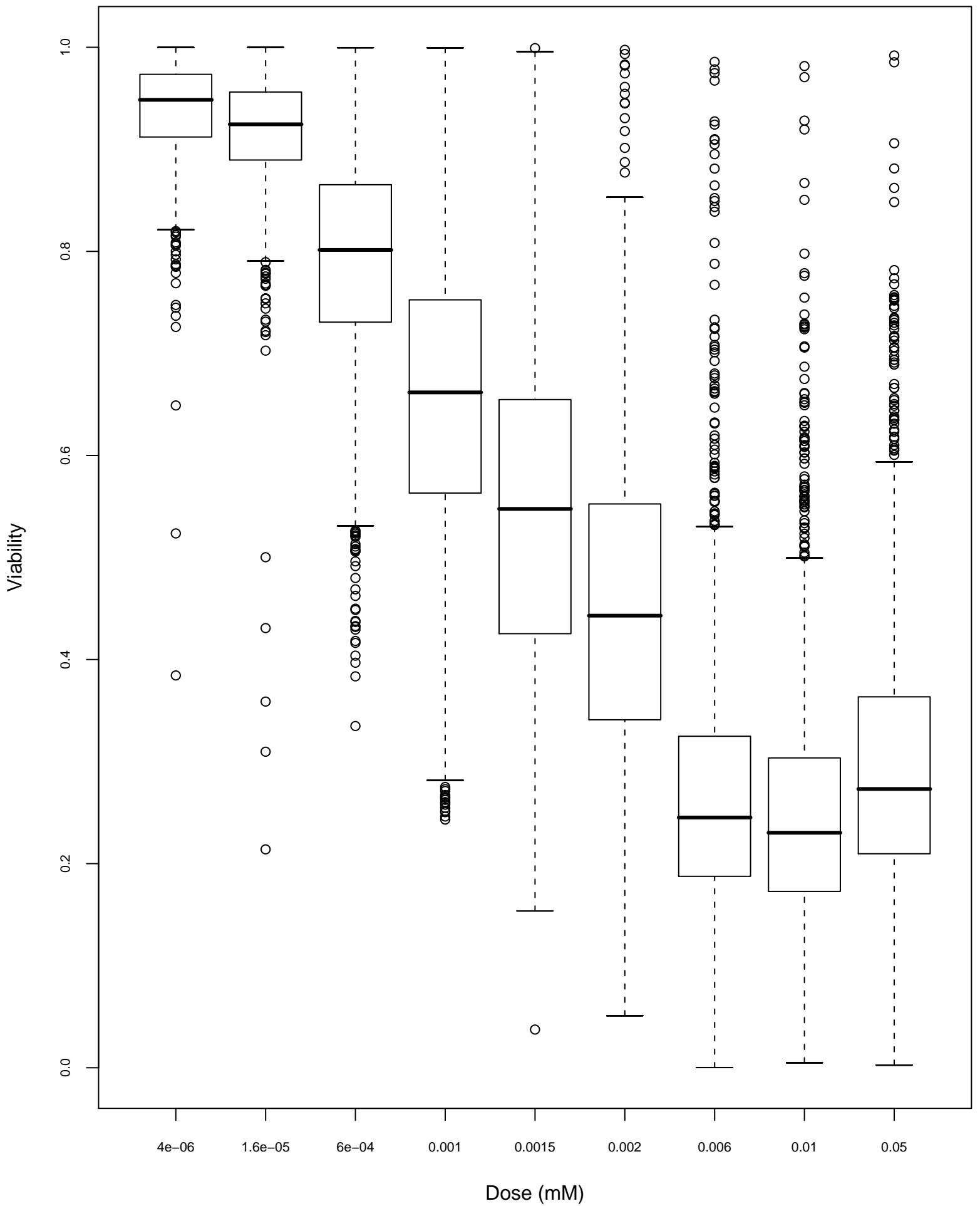

Drug SN38

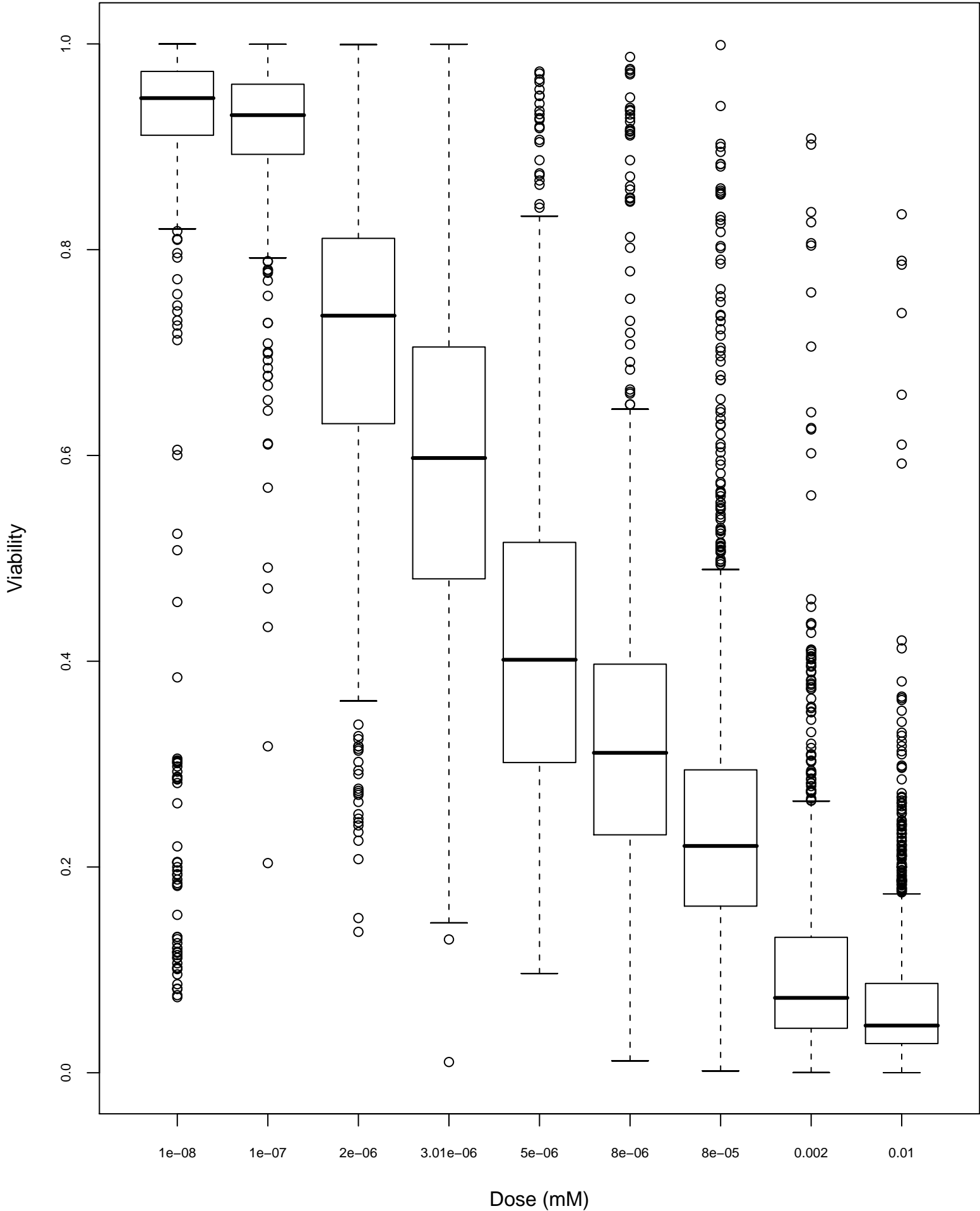

# Drug TPT

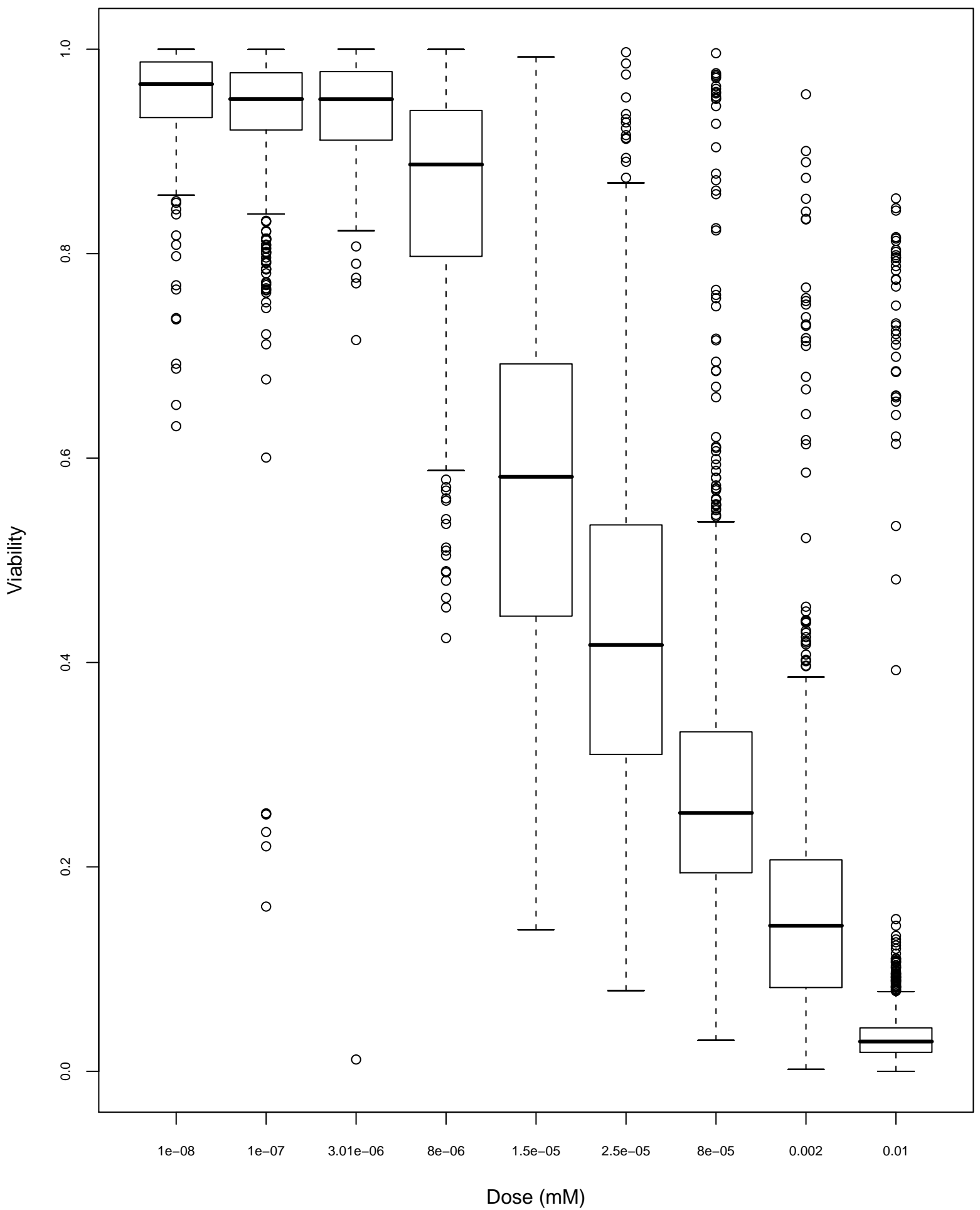

# Drug CICPT

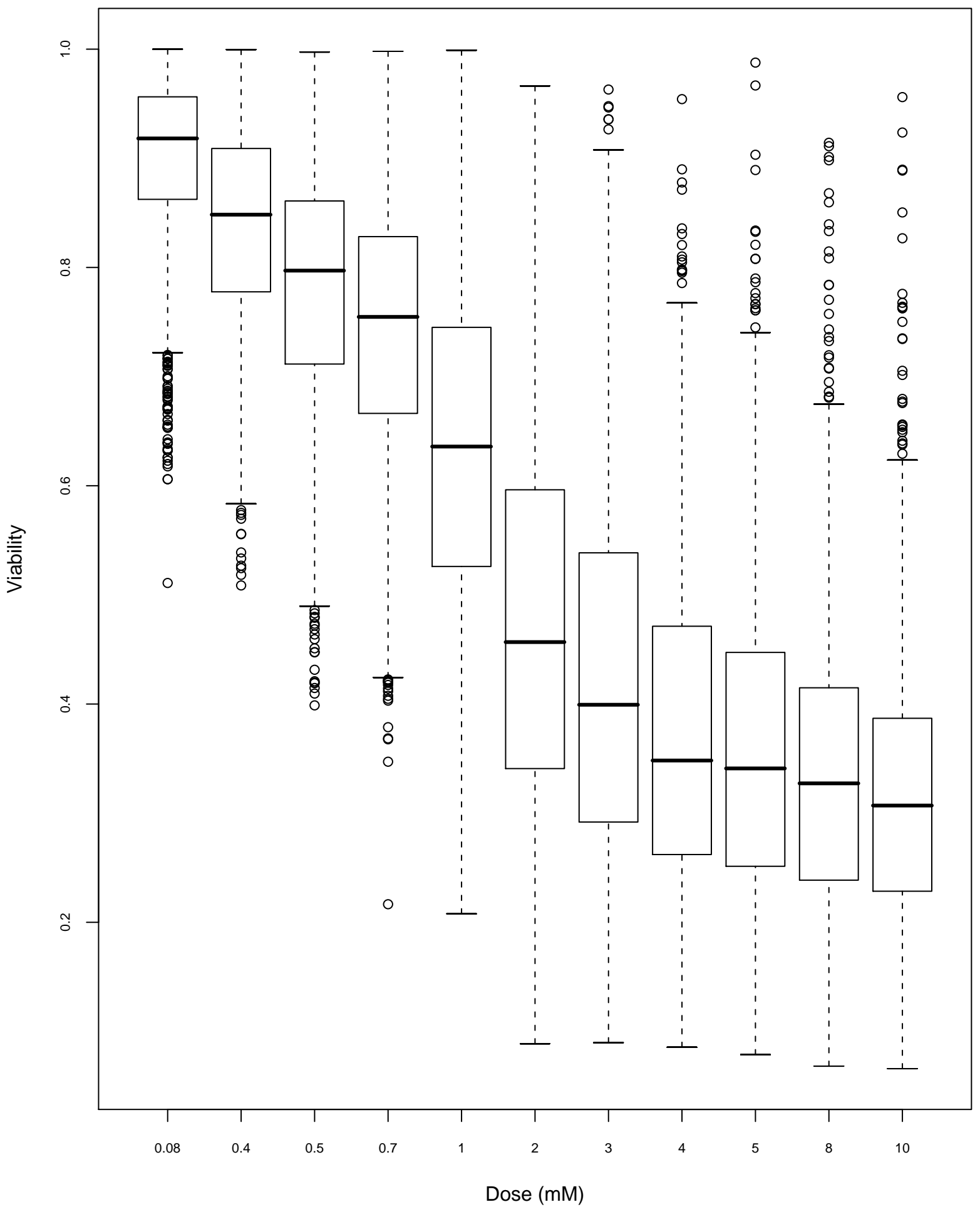

# Drug hCPT

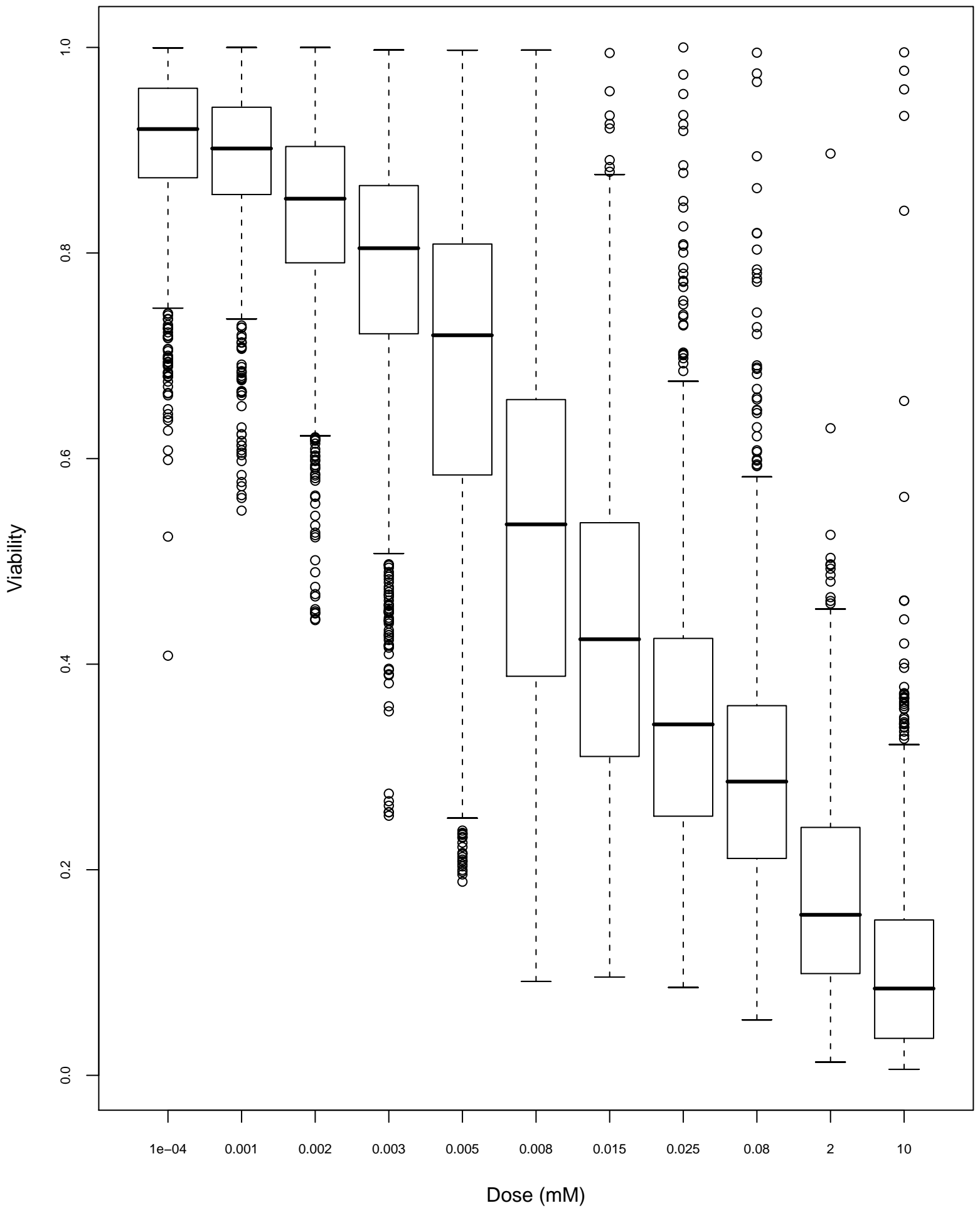

# Drug mCPT

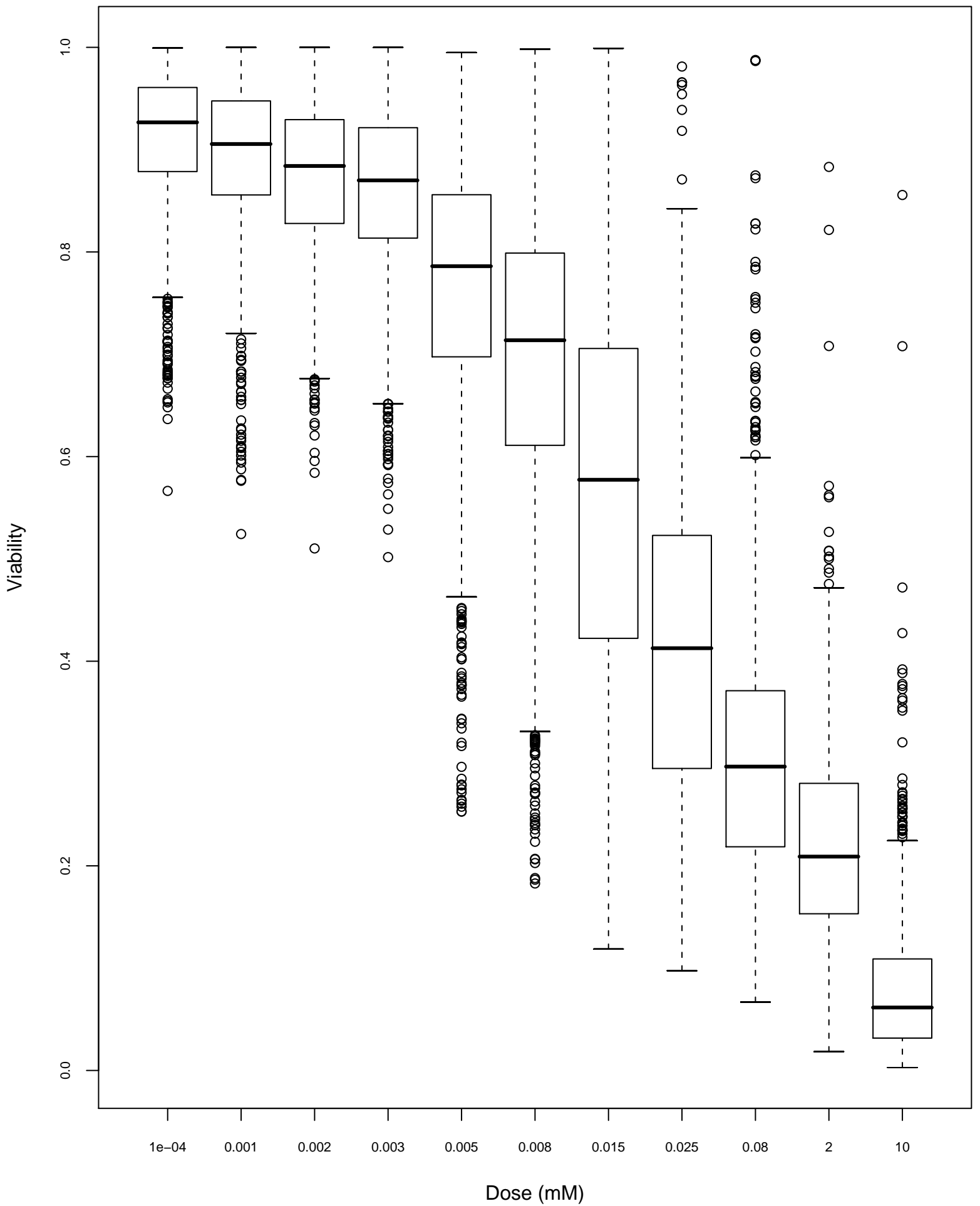

Supplement: Dataset S3 — Boxplots illustrating variance in cell viability across the entire CEPH population (n = 125) for each drug. Line represents mean phenotypic response, whiskers box represents upper and lower quartiles, and whiskers are 1.5*IQR. Outliers (circles) are individuals whose mean viability is greater than 1.5*IQR. (PDF) [file pone.0017561.s009.pdf]
