# Supplementary material for: Identification and Replication of Loci Involved in Camptothecin-Induced Cytotoxicity Using CEPH Pedigrees
Source: PLoS One. 2011 May 5;6(5):e17561. doi: 10.1371/journal.pone.0017561 (PMC3088663; doi:10.1371/journal.pone.0017561)
Supplement: Table S1 — Similarity matrix of overall QTL patterns for each camptothecin. To compare the overall QTL patterns between each of the camptothecin analogues a similarity matrix was constructed using a binary assessment of peaks present at either the significant or suggestive level for each camptothecin R squared correlations (r2) are bound by 0 and 1 and the greater the value the more related the overall QTL patterns are to each other. (DOCX) [file pone.0017561.s004.docx]

Table S1: Similarity matrix of overall QTL patterns for each camptothecin.

| r^2^ | 9AC | 9NC | CPT | CPT11 | SN38 | TPT |
| --- | --- | --- | --- | --- | --- | --- |
| 9AC | 1.000 | 0.724 | 0.517 | 0.483 | 0.552 | 0.483 |
| 9NC |  | 1.000 | 0.517 | 0.483 | 0.621 | 0.483 |
| CPT |  |  | 1.000 | 0.759 | 0.690 | 0.517 |
| CPT11 |  |  |  | 1.000 | 0.655 | 0.517 |
| SN38 |  |  |  |  | 1.000 | 0.586 |
| TPT |  |  |  |  |  | 1.000 |

*Constructed from binary evaluation of presence/absence of significant QTLs
