# Supplementary material for: Identification and Replication of Loci Involved in Camptothecin-Induced Cytotoxicity Using CEPH Pedigrees
Source: PLoS One. 2011 May 5;6(5):e17561. doi: 10.1371/journal.pone.0017561 (PMC3088663; doi:10.1371/journal.pone.0017561)
Supplement: Table S2 — Genes under QTLs shared by camptothecins. The functional annotation clustering tool from the web-accessible program Database for Annotation, Visualization, and Integrated Discovery (DAVID) was used to identify over-represented gene ontology terms (GO) and KEGG pathways for genes under each of the shared QTLs on chromosomes 1, 5, 11, 16 and 20. Genes of interest are listed by chromosomal location and then gene ontology term. The bolded gene names in Table S1 have previously been associated with camptothecin activity in yeast and/or mammalian cell lines. (DOCX) [file pone.0017561.s005.docx]

Table S2: Genes under QTLs shared by camptothecins *

| chr | GO terms | | | | GENES |
| --- | --- | --- | --- | --- | --- |
| 1 | GO:0016055 | Wnt receptor signaling pathway | **WNT3A, WNT10A, WNT6, WNT9A,** | | |
|  | GO:0006974 | response to DNA damage stimulus | TNP1, **NHEJ1,** C1orf124, **PARP1,** **BARD1,** DTL, **XRCC5,** | | |
|  | GO:0003677 | DNA binding | MIXL1, BATF3, PROX1, TAF1A, SP110, ZNF142, ESRRG, **HIST3H3,** IKZF2, SMARCAL1, **HIST3H2BB,** ZNF678, TSNAX, TAF5L, LBR, **HIST3H2A,** FEV, RCOR3, TIGD1, **PAX3,** HLX, SP100, ATF3, SP140, NCL, | | |
|  | GO:0006350 | transcription | KCNH1, RCOR3, PTMA, HLX, EGLN1, IHH, STK36 | | |
|  | GO:0006915 | apoptosis | COL4A3, **CUL3**, SCG2, IL8RB, **AGT**, INHA, INPP5D, SPATA3, **TP53BP2,** PECR, PSEN2, **TGFB2,** TRAF5, | | |
|  | GO:0030154 | cell differentiation | USH2A, ERBB4, MREG, NGEF, EFHD1, ITPKB, ACTA1, SERPINE2, OBSCN, SCG2, COL4A4, DNER, SPEG, LEFTY2, | | |
| 5 | GO:0030154 | cell differentiation | NRG2, NME5, ISL1, NAIP, HSPA9, DND1, **MAP3K1,** CYFIP2, ZNF346, AGGF1, UNC5A, TSSK1B, CTNNA1, NPM1, DRD1, SLIT3, PDLIM7, FST, POU4F3, PURA, F2R, AP3B1, **PIK3R1,** PRKAA1, NKX2-5, GPR98, CD74, PRDM6, NEUROG1, PPP2CB, NR2F1, JMY, MEF2C, CSF2, GZMA, MAP1B, ADRB2, FGF10, VDAC1, **UBE2B,** IL12B, CARD6, CARTPT, ATG12, IL3, MRPS30, SEMA6A, SMAD5, EFNA5, HDAC3, SMN2, PROP1, AFF4, ERAP1, C9, GDNF, SPINK5, EGR1, PPP2R2B, DDX41, PRLR, FGF1, HAND1, SFXN1, HNRPAB, HSD17B4, RASA1, IL4, ACSL6, FGF18, CD14, DBN1, GFRA3, | | |
|  | GO:0006915 | apoptosis | PROP1, PPP2CB, ADRB2, | | |
|  | GO:0008283 | cell proliferation | FABP6, **SKP2,** APC, CNOT8, TGFBI, B4GALT7, DAB2, IL9, HTR1A, L6ST, ADRA1B, SPOCK1, PPAP2A, LIFR, FGF1, CSF1R, HBEGF, | | |
|  | GO:0006512 | ubiquitin cycle | **FBXL17,** **FBXO4,** RNF14, TRIM23, **SKP1,** ENC1, CDC23, RNF145, TSPAN17, ERCC8, **FBXO38,** MARCH3, KLHL3, **FBXW11,** **UBE2D2**, **FBXL21,** | | |
|  | GO:0000079 | regulation of cyclin-dependent protein kinase activity | **CDC25C, CDK7, CCNG1,** CCNH, | | |
| chr | GO terms | | | | GENES |
| 5 | GO:0006974 | response to DNA damage stimulus | CCNO, POLK, **RAD17,** PTTG1, **XRCC4,** **RAD50,** GTF2H2, MSH3, | | |
|  | GO:0043122 | regulation of NF-kappaB cascade | F2R, PLK2, CXXC5, NDFIP1, TICAM2, | | |
|  | GO:0007049 | cell cycle | PAM, MCC, IRF1, CETN3, NIPBL, DUSP1, **CCNB1,** PFDN1, ERBB2IP, SEPT8, CDC20B, **RASA1** | | |
|  | GO:0003682 | chromatin binding | NSD1, **H2AFY**, CHD1, | | |
| 6 | GO:0006915 | apoptosis | MAK, GCM2, LY86, TUBB2A, EDN1, SERPINB9, EEF1E1, TXNDC5, TUBB2B, **FOXF2**, RIPK1, **FOXC1**, DSP, BMP6, | | |
|  | GO:0006350 | transcription | FARS2, PRPF4B, RPP40, SIRT5, RREB1, CDYL, HIVEP1, TFAP2A, **FOXQ1**, | | |
|  | GO:0065004 | protein-DNA complex assembly | HIST1H2BJ, HIST1H2AC, HIST1H1C, **HIST1H2AA, HIST1H2AD**, HIST1H2BM, HIST1H1A, HIST1H1E, HIST1H2AM, HIST1H2AE, HIST1H3J, HIST1H2BL, HIST1H1B, HIST1H2BB, HIST1H2APS4, HIST1H2BK, HIST1H2BN, HIST1H1T, HIST1H2BH, HIST1H2BA, HIST1H1D, HIST1H4G, | | |
|  | GO:0008134 | transcription factor binding | BAT5, CFB, HSD17B8, SRF, PFDN6, RING1, TTRAP, ABT1, TRERF1, DDAH2, ZBTB9, C6orf27, HMGA1, ZBTB22, MDFI, PPARD, RXRB, DOM3Z, | | |
| 11 | GO:0050896 | response to stimulus | TIRAP,CHEK1,OR10G4,MFRP,BSX,C1QTNF5,OR10G7,OR10G9,PVRL1, POU2F3, R8A1,ROBO3,HSPA8,CRTAM,OR10S1,OR8G5OR6T1,OR4D5, OR6M1,EI24,OR8B8, OR8B12,OR8B2,OR8B3,OR8D1,OR8B4,OR8D2,OR8D4, PATE4,OR6X1 | | |
|  | GO:0030154 | cell differentiation | PVRL1,POU2F3,**DDX25**,CDON,ROBO4,TIRAPROBO3,THY1,FEZ1 | | |
|  | GO:0022402 | cell cycle process | HEPACAM,HEPN1,TBRG1,**CHK1** | | |
|  | GO:0048522 | positive regulation of cellular process | BSX,EI24,CRTAM,POU2F3,CBL,ARHGEF12,THY1 | | |
| chr | GO terms | | | | GENES |
| 11 | GO:0003677 | DNA binding | PKNOX2,BSX,POU2F3,TRIM29,CBL,TBRG1,ZNF202 | | |
| 16 | GO:0005524 | ATP binding | ERN2, PDPK1, **PLK1,** ATP2A1, NOD2, ATP6V0C, CREBBP, DNAH3, **TAOK2,** NME3, **PRKCB1,** NUBP2, KIFC3, WDR51B, NUBP1, PHKG2, RAB26, CIITA, EEF2K, MYH11, EARS2, PKMYT1, SEPHS2, TRAP1, BCKDK, CHTF18, NLRC5, ACSM1, | | |
|  | GO:0007049 | cell cycle | AXIN1, CORO1A, CYLD, CP110, SEPT1, CSNK2A2, DNAJA2, **RBL2,** TSC2, PAPD5, PRM2, KATNB1, PRM1, SIAH1, **CCNF**, GSPT1, **MAPK3,** | | |
|  | GO:0051092 | activation of NF-kappaB  transcription factor | PYCARD, NLRC3 | | |
|  | GO:0006974 | response to DNA damage stimulus | C16ORF35, NTHL1, SMG1, ERCC4, KCTD13, KIF22, MPG, GIYD2, | | |
|  | GO:0042493 | response to drug | ABCA3, **ABCC6,** **ABCC1,** **MVP,** | | |
|  | GO:0030154 | cell differentiation | SOX8, TRAF7, NTN2L, CACNA1H, TBX6, SALL1, IRX5, GNAO1, NUPR1, BBS2, METRN, TNP2, MT3, MKL2, MYST1, IL27, **NDRG4,** | | |
|  | GO:0008219 | cell death | CLN3, TNFRSF12A, CIAPIN1, DNAJA3, LITAF, PDIA2, EMP2, BFAR, DNASE1, SPN, | | |
|  | GO:0006512 | ubiquitin cycle | RAB40C, TCEB2, SOCS1, AMFR, **FBXL16**, STUB1, RNF40, USP7, **FBXL19,** AKTIP | | |
|  | GO:0003677 | DNA binding | ZNF646, GTF3C1, ZNF213, HN1L, ORC6L, ZNF174, CARHSP1, MAZ, UBN1, IRX3, ALG1, IRX6, ZNF597, ZNF75A, ZNF434, FUS, SRCAP, TFAP4, CHD9, ZNF263, POLR2C, DNASE1L2, ZNF500, ZNF267, POLR2K, MRPL28, ZNF319, ZNF205, E4F1, | | |
|  | GO:0006350 | transcription | RPUSD1, NUDT21, TUFM, RPL3L, EIF3C, TRAF7, RSL1D1, RNPS1, RPS2, TBL3, POLR3E, ZNF720, C16ORF33, RPS15A, SRRM2, | | |
|  | GO:0010467 | gene expression | ZNF200, RRN3, | | |
| chr | GO terms | | | GENES | |
| 20 | GO:0003677 | DNA binding | MYBL2, TAF4, HBF4A, CTCFL, SALL4, SYCP2, VSX1, PHF0, CHD5,**TOP1**, CST1, TFAP23, ZNF341, SPO11, ZHX3, ZNF337, TCEA2, SOX18, INSM1, TGIF2, TCFL5, CST2, FOXA2, ZNF334, ZBP1, GZF1, MYT1, ZNFX1, FKHL18, SNA1, NKX2-2, MAFB, L3MBTL, SCNAD1, TSHZ2, GMEB2, E2F1, PRIC285, ADNP, GATA5, PAX1, RTEL1, ZFP64, RBPJL, ZNF217, PLAGL2, RP11-227D2.4, CBFA2T2, WISP2, ZNF335, DNMT3B, HMG1L1, CEPB | | |
|  | GO:0030154 | cell differentiation | NKX2-2, SPATA2, MAFB, BCL2L1, SCAND1, MYBL2, NCO86, DIDO1, PARD6B, CD40, E2F1, ELMO2, RPS21, NEURL2, PTGIS, TOP1, TFPA2C, EEF1A2, PAX1, LAMA5, EYA2, TGM2, CDH4, TNFRS6B, PLAGL2, SERINC3, SNTA1, SOX18, CDK5RAP1, NDRG3, CTNNBL1, INSM1, STK4, TCFL5, FOXA2, GDF5, CEBPB, ZNF313, BMP7, MYT1, TMEM189-UBE2V1, BIRC7, MMP9 | | |
|  | GO:0008219 | cell death | | TGMS, **BCL2**L1, TNFRSF6B, SCAND1, MYBL2, PLAGL2, SERINC3, CTNNBL1, DIDO1, CD40, STK4, E2F1, ELMO2, GDF5, PTGIS, CEBPB, TOP1, EEF1A2, BIRC7, MMP9 | |
|  | GO:0007049 | cell cycle | | DSN1, CEP250, RBL1, CTCFL, TPX2, MAPRE1, UBE2C, CABLES2, AURKA | |
|  | GO:0006350 | transcription | | RBM39, C20ORF20, TAF4, POFUT1, NCOA3, ZNF341, TH1L, TCEA2, ADRM1, , ZNFX1, FKHL18, L3MBTL, XRN2, TSHZ2, GMEB2, ASLX1, PRIC285, ZMYND8, ADNP, GATA5, ZFP64, ZBTB46, ZNNF217, ID1, NFATC2, HMG1L1, SLA2 | |
|  | GO:0040007 | regulation of growth | | OGFR, C20ORF10, GINS1, GHRH, ITCH | |
|  | GO:0050896 | response to stimulus | | ASIP, ADA, PLUNC, SGK2, PROCR, VAPB, DEFB123, WFDC12, DEFB124, GNAS, TOP1, CHRNA4, TGM2, DEFB118, CD93, NTSR1, DEFB115, BPI, GSS, DEFB116, DEFB121, C20ORF185, CST7, LBP, THBD, DYNLRB1, SAMHD1, DEFB119, CST11, LIME1 | |

*Gene names in bold indicate genes which have previously been associated with camptothecin-induced cytotoxicity.
