# Supplementary material for: Identification and Replication of Loci Involved in Camptothecin-Induced Cytotoxicity Using CEPH Pedigrees
Source: PLoS One. 2011 May 5;6(5):e17561. doi: 10.1371/journal.pone.0017561 (PMC3088663; doi:10.1371/journal.pone.0017561)
Supplement: Table S3 — Genes under QTLs of interest for the camptothecins. DAVID was used to identify over-represented gene ontology terms (GO) and KEGG pathways for genes located under the QTL on chromosome 1 for which a linkage with CPT bearing a nitrogen atom in position 9 is reported. Genes of interest are also listed for the QTL observed on chromosome 13 correlating uniquely with topotecan treatment and on chromosome 5 which is in linkage only with CPT11, 9AC and 9NC. (DOCX) [file pone.0017561.s006.docx]

Table S3: Genes under QTLs of interest for the camptothecins

| Chr | Location (cM) | GO terms | | Genes |
| --- | --- | --- | --- | --- |
| 1 | 102-159 | GO:0006396 | RNA processing | TARS2, EIF2C1, LMO4, YBX1, FUBP1, RBM8A, WDR77, PTBP2, DDX20, KHDRBS1, YARS, PTGER3, ZCCHC11, MAGOH, SARS, PRPF3, GTF2B, TTF2, TAF13, SERBP1, CELF3, SNRNP40, PRPF38B, EIF2C3, EIF2C4, PRPF38A, PABPC4, TYW3, WARS2, SF3B4, RRAGC, ARNT, MOV10, UTP11L, PARS2, RPS7P4, RPL5, SCNM1, SFRS11, BCAS2, RTCD1, ELAVL4, RPF1, LOC100131261, SF3A3, CCDC76, PPIE, PPIH, SFPQ, THRAP3, ZRANB2, WDR3, LSM10, RNPC3, FOXE3, TRIT1 |
|  |  | GO:0065004 | protein-DNA complex assembly | MEAF6, HIST2H2AA3, HIST2H2AA4, DMAP1, HIST2H4A, SYCP1, VPS72, HIST2H4B, HIST2H2AB, CDCA8, CHD1L, HIST2H2AC, PRMT6, LOC646817, MYSM1, SETDB1, HIST2H3A, RBBP4, NASP, MSH4, RAD54L, HIST2H3C, HIST2H3D, HDAC1, HIST2H2BD, HIST2H2BE, HIST2H2BF, BRDT, KDM4A |
|  |  | GO:0010608 | regulation of gene expression | EIF2C1, ZCCHC11, MAGOH, BARHL2, MKNK1, YBX1, MOV10, RBM8A, SERBP1, PUM1, EIF2B3, EIF2C3, EIF2C4 |
|  |  |  |  | JUN, GSTM1, FOXD3, FOXO6, BCLX2L15, JAK1 |
| 5 | 54-74 | GO:0042127 | regulation of cell proliferation | NDUFS4, HTR1A, OSMR, IL6ST, LIFR, ITGA2, FGF10, PPAP2A, IL31RA |
|  |  | GO:0007242 | intracellular signaling cascade | FYB, PLCXD3, RAB3C, PTGER4, IL6ST, ITGA1, CDK7, TRIM23, IL31RA, NDUFS4, HTR1A, MAP3K1, PRKAA1, DEPDC1B, PPAP2A, PIK3R1, RAD17, GHR |
|  |  | GO:0016567 | protein ubiquitination | ERCC8, MAP3K1, FBXO4, TRIM23 |
|  |  | GO:0017076 | nucleotide binding | ACTBL2, HCN1, MGC42105, SKIV2L2, CDK7, TRIM23, DDX4, MAST4, DHX29, PLK2, MAP3K1, PRKAA1, TAF9, RAD17, KIF2A |
|  |  | GO:0042981 | regulation of apoptosis | MAP3K1, ITGA1, TAF9, CARD6, GDNF, IL31RA, GHR |
|  |  | GO:0010628 | positive regulation of gene expression | CENPK, ISL1, IL31RA |
| chr | location | GO terms | | GENES |
| 13 | 0-19.6 | GO:0006350 | positive regulation of transcription | RNF6, GSX1, CDX2, POLR1D, GTF3A, CDK8, SAP18  ATP8A2, CDX2, FGF9, FLT1, FLT3, GJB6, PDX1 |
| 13 | 0-19.6 | GO:0003677 | DNA binding | PARP4 |
|  |  | GO:0016887 | ATPase activity | ATP5EP2, ATP12A, ATP8A2 |
