# Supplementary material for: Identification and Replication of Loci Involved in Camptothecin-Induced Cytotoxicity Using CEPH Pedigrees
Source: PLoS One. 2011 May 5;6(5):e17561. doi: 10.1371/journal.pone.0017561 (PMC3088663; doi:10.1371/journal.pone.0017561)
Supplement: Dataset S4 — Boxplots illustrating intra- and inter-family variance in cell viability of each drug and dose across CEPH families. Line represents mean phenotypic response, whiskers box represents upper and lower quartiles, and whiskers are 1.5*IQR. Outliers (circles) are individuals whose mean viability is greater than 1.5*IQR. (PDF) [file pone.0017561.s010.pdf]

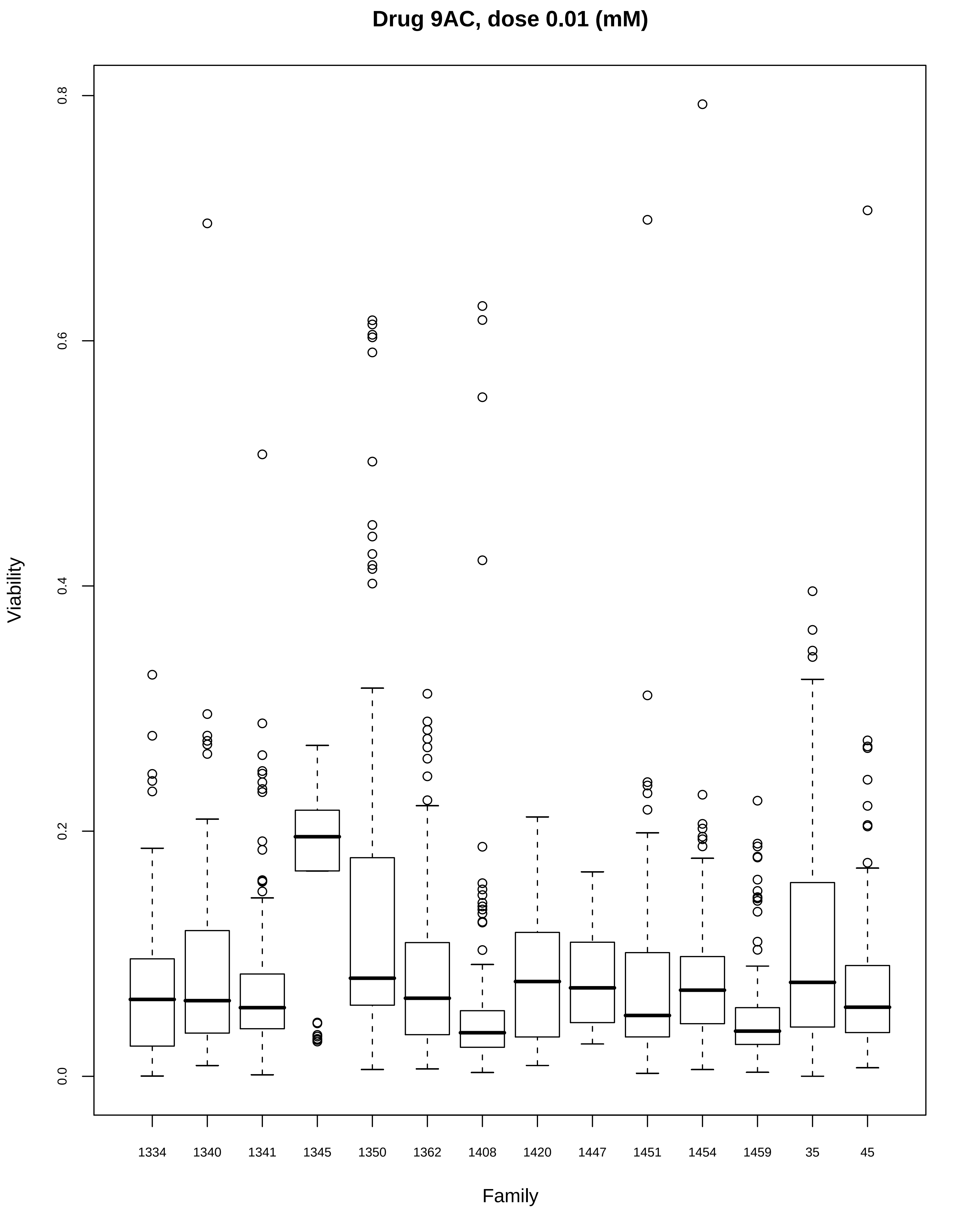

Drug 9AC, dose 0.01 (mM)
Viability
0.0
0.2
0.4
0.6
0.8
1334
1340
1341
1345
1350
1362
1408
1420
1447
1451
1454
1459
35
45
Family

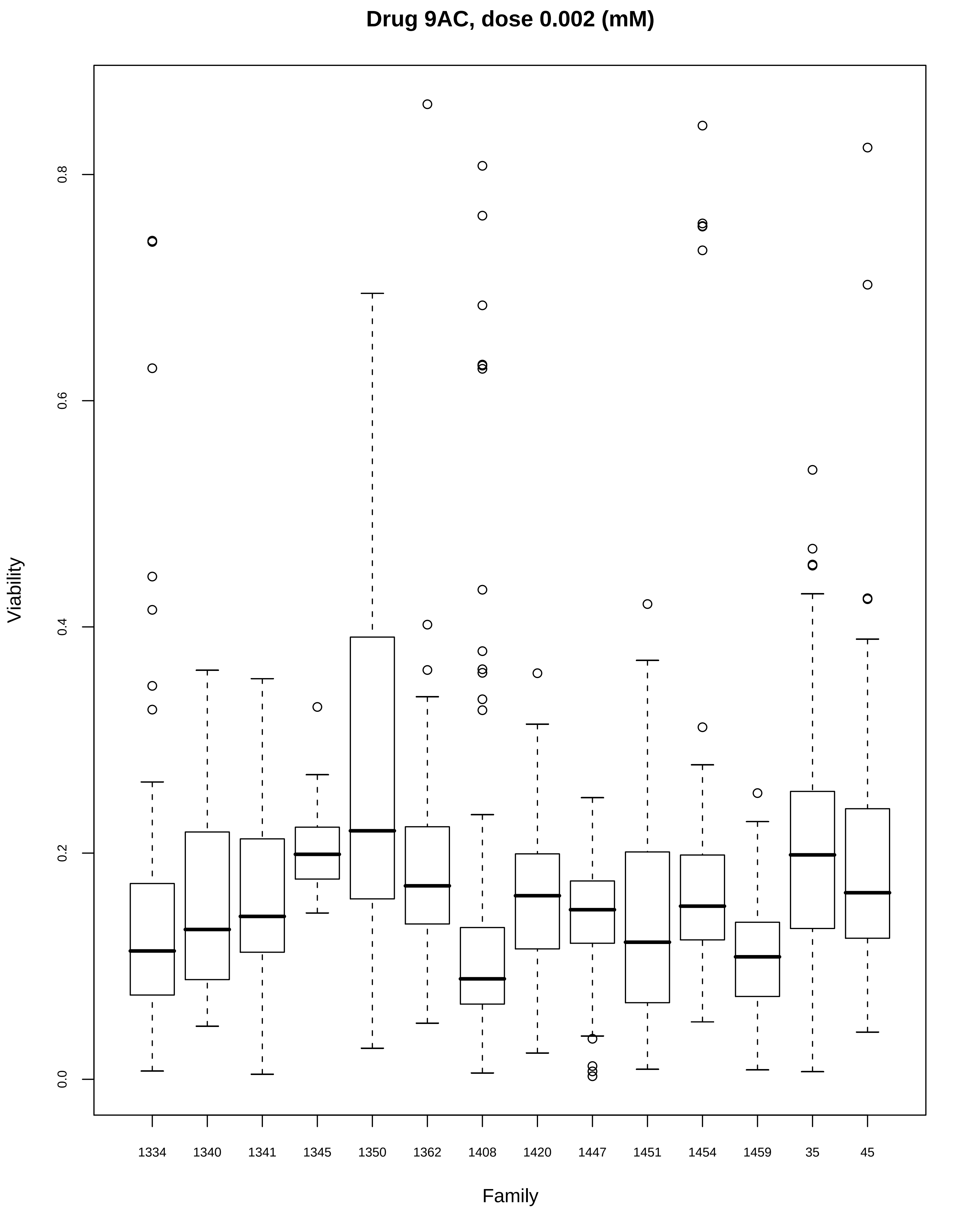

Drug 9AC, dose 0.002 (mM)
Viability
0.0
0.2
0.4
0.6
0.8
Family
1334
1340
1341
1345
1350
1362
1408
1420
1447
1451
1454
1459
35
45

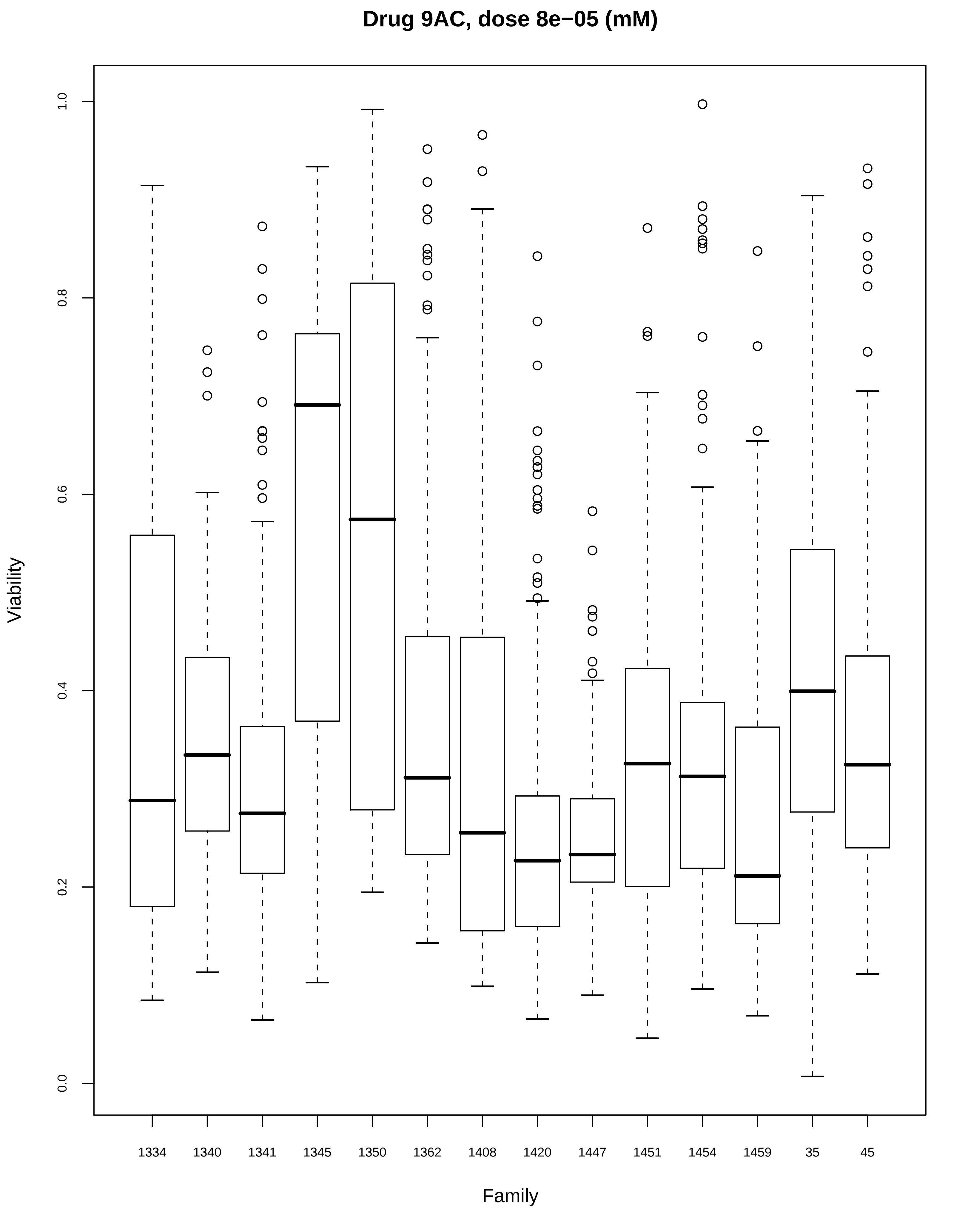
Drug 9AC, dose 8e−05 (mM)
Viability
0.0
0.2
0.4
0.6
0.8
1.0
Family
1334
1340
1341
1345
1350
1362
1408
1420
1447
1451
1454
1459
35
45

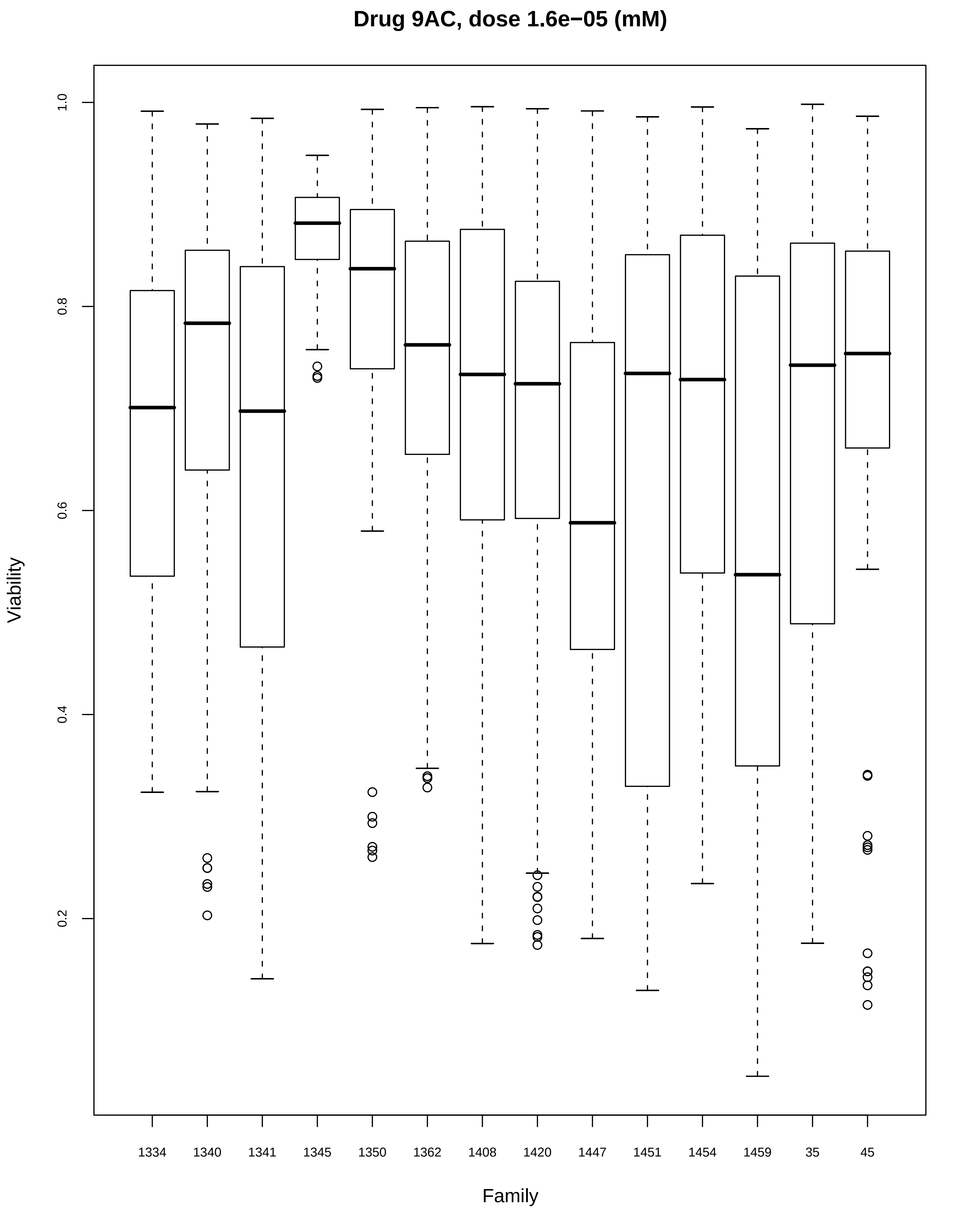
Drug 9AC, dose 1.6e−05 (mM)
Viability
1.0
0.8
0.6
0.4
0.2
Family
1334
1340
1341
1345
1350
1362
1408
1420
1447
1451
1454
1459
35
45

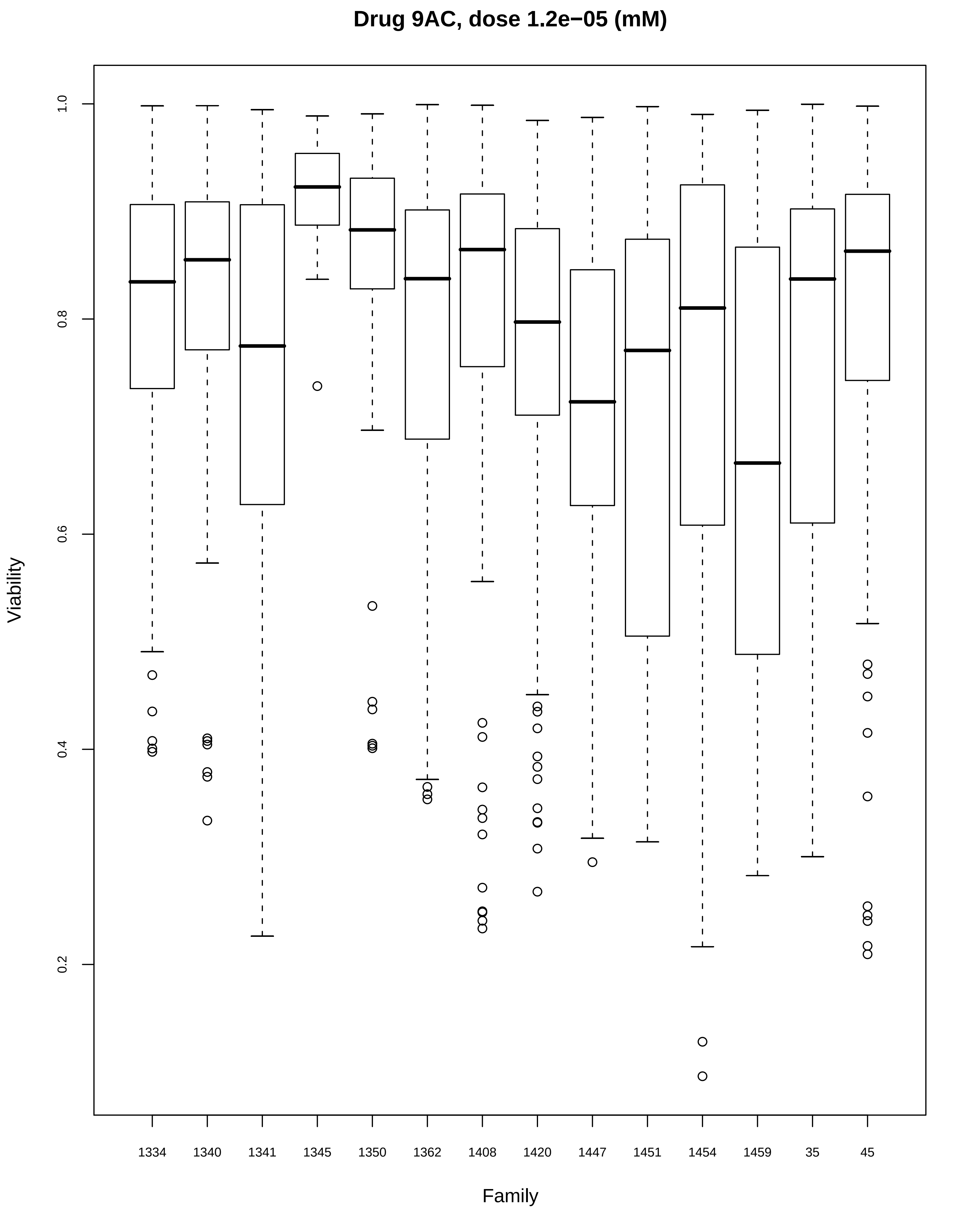
Drug 9AC, dose 1.2e−05 (mM)
Viability
1.0
0.8
0.6
0.4
0.2
1334
1340
1341
1345
1350
1362
1408
1420
1447
1451
1454
1459
35
45
Family

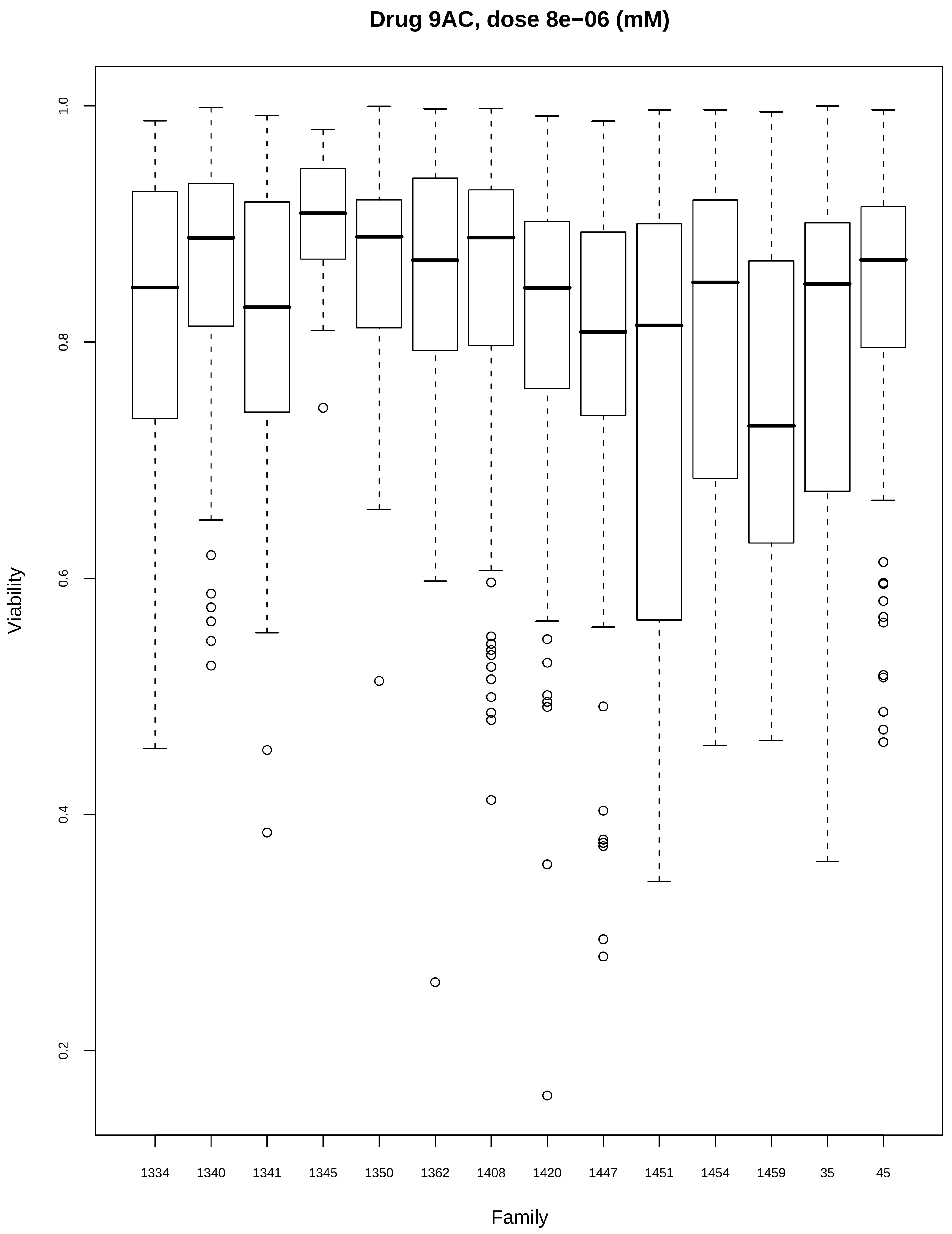
Drug 9AC, dose 8e−06 (mM)
Viability
1.0
0.8
0.6
0.4
0.2
1334
1340
1341
1345
1350
1362
1408
1420
1447
1451
1454
1459
35
45
Family

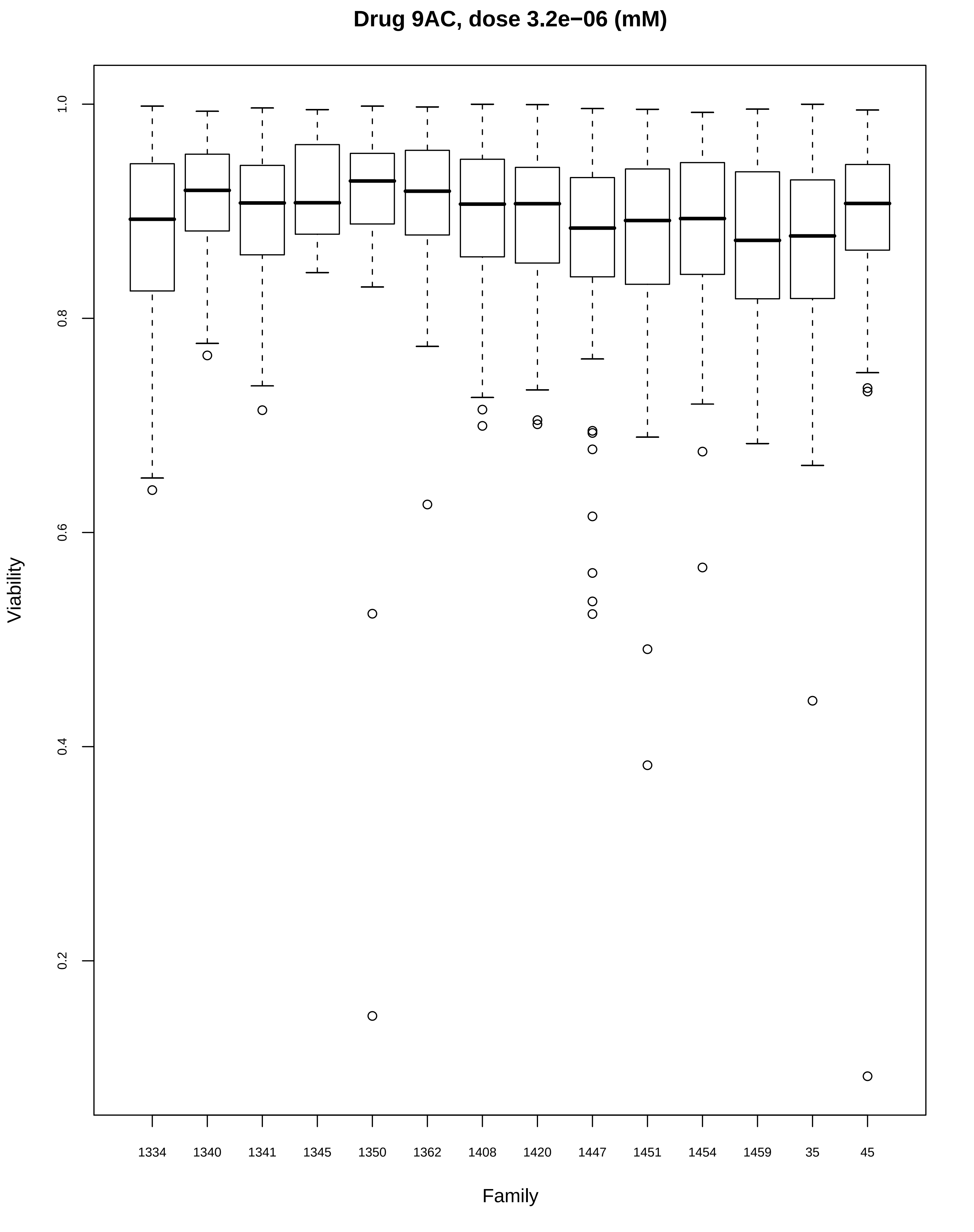
Drug 9AC, dose 3.2e−06 (mM)
Viability
1.0
0.8
0.6
0.4
0.2
1334
1340
1341
1345
1350
1362
1408
1420
1447
1451
1454
1459
35
45
Family

Drug 9AC, dose 6.4e−07 (mM)

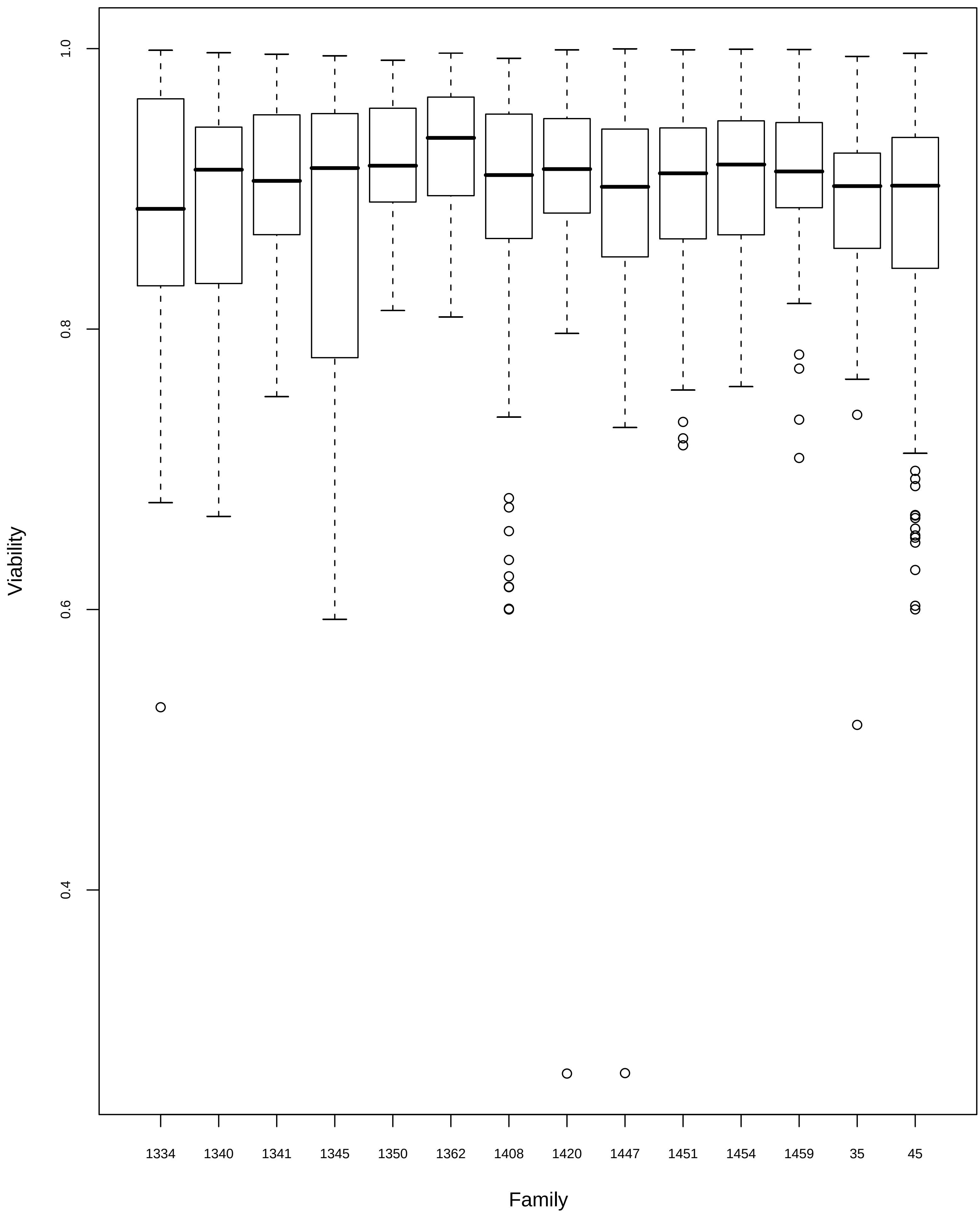

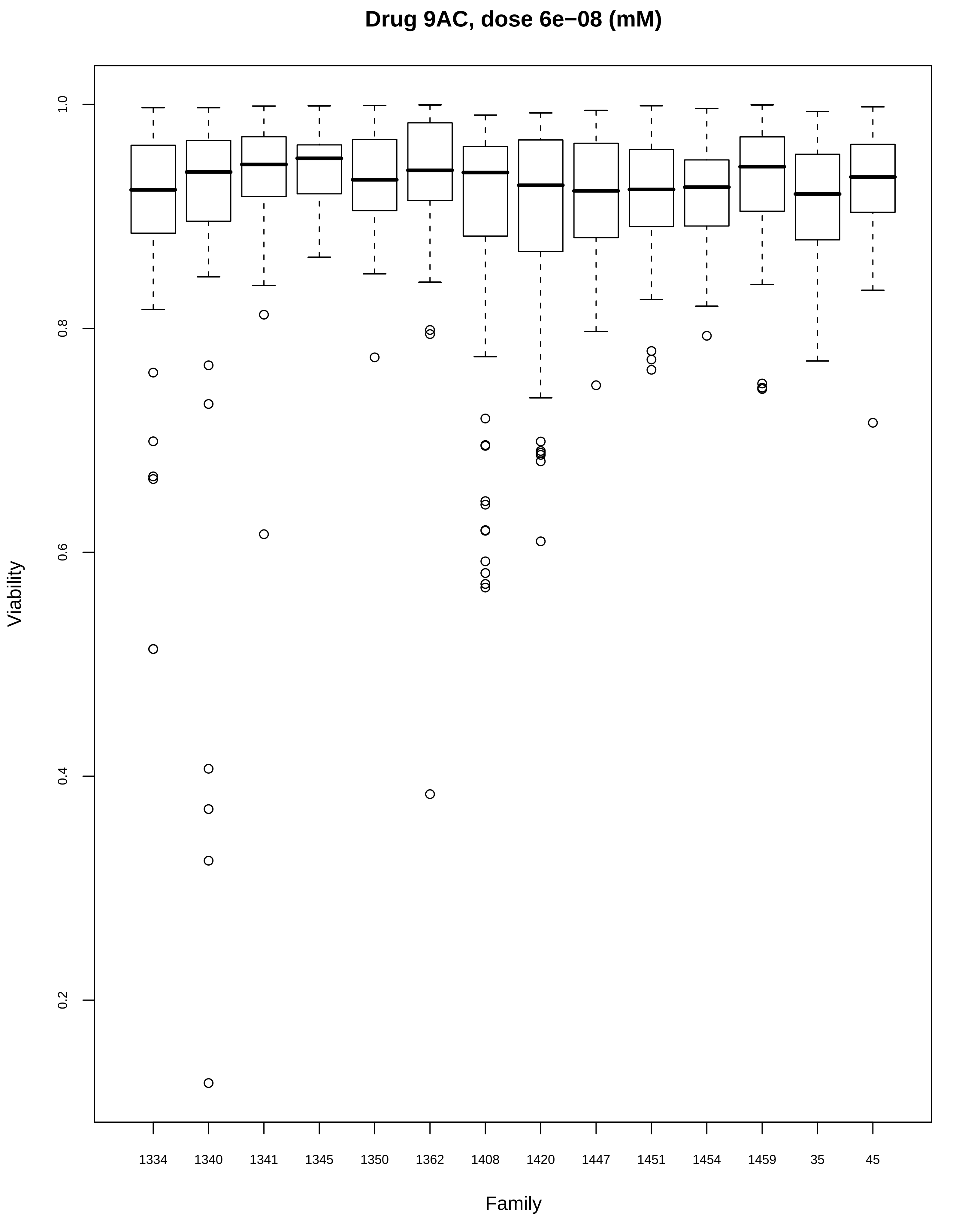
Drug 9AC, dose 6e−08 (mM)
Viability
1.0
0.8
0.6
0.4
0.2
Family
1334
1340
1341
1345
1350
1362
1408
1420
1447
1451
1454
1459
35
45

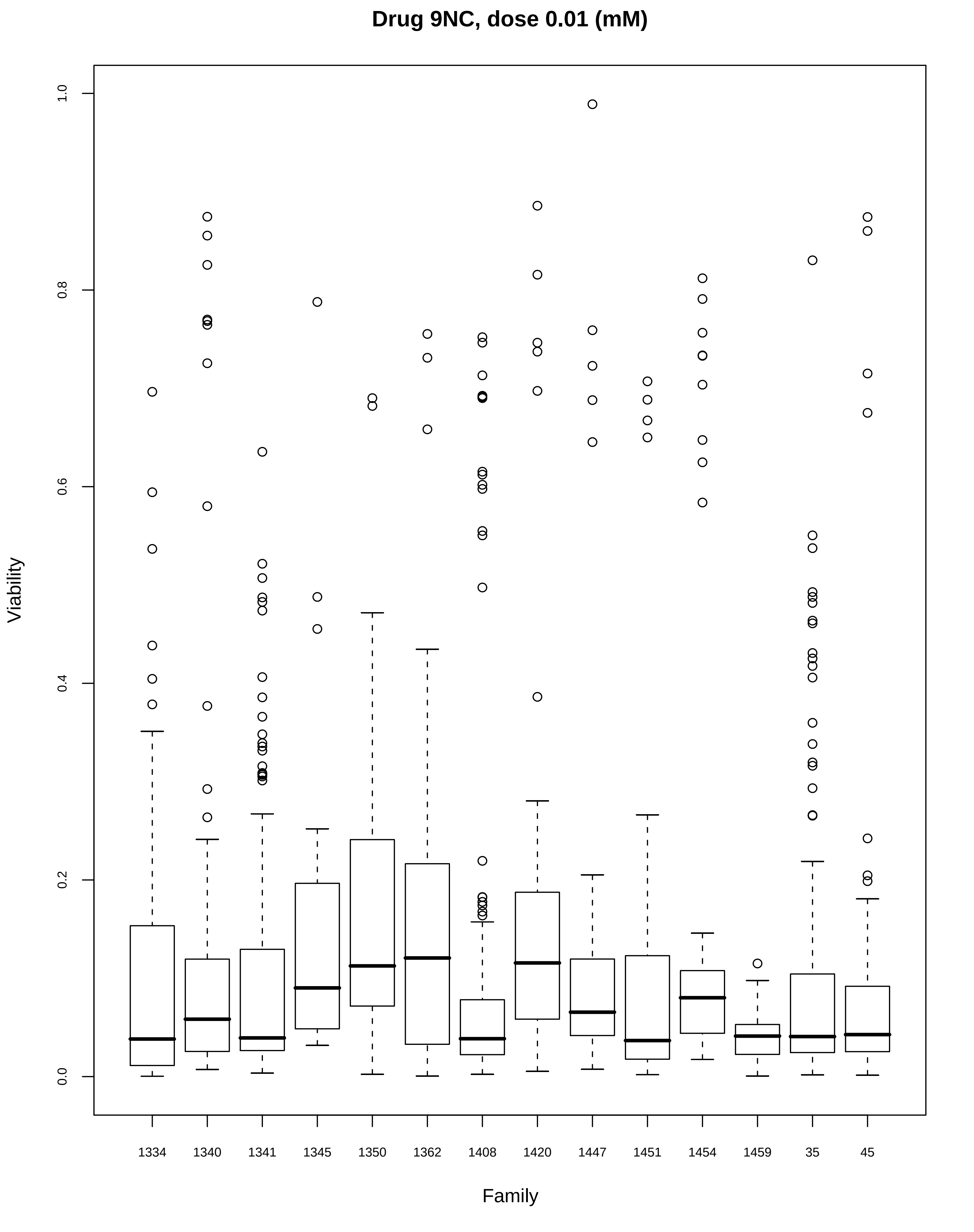
Drug 9NC, dose 0.01 (mM)
Viability
0.0
0.2
0.4
0.6
0.8
1.0
Family
1334
1340
1341
1345
1350
1362
1408
1420
1447
1451
1454
1459
35
45

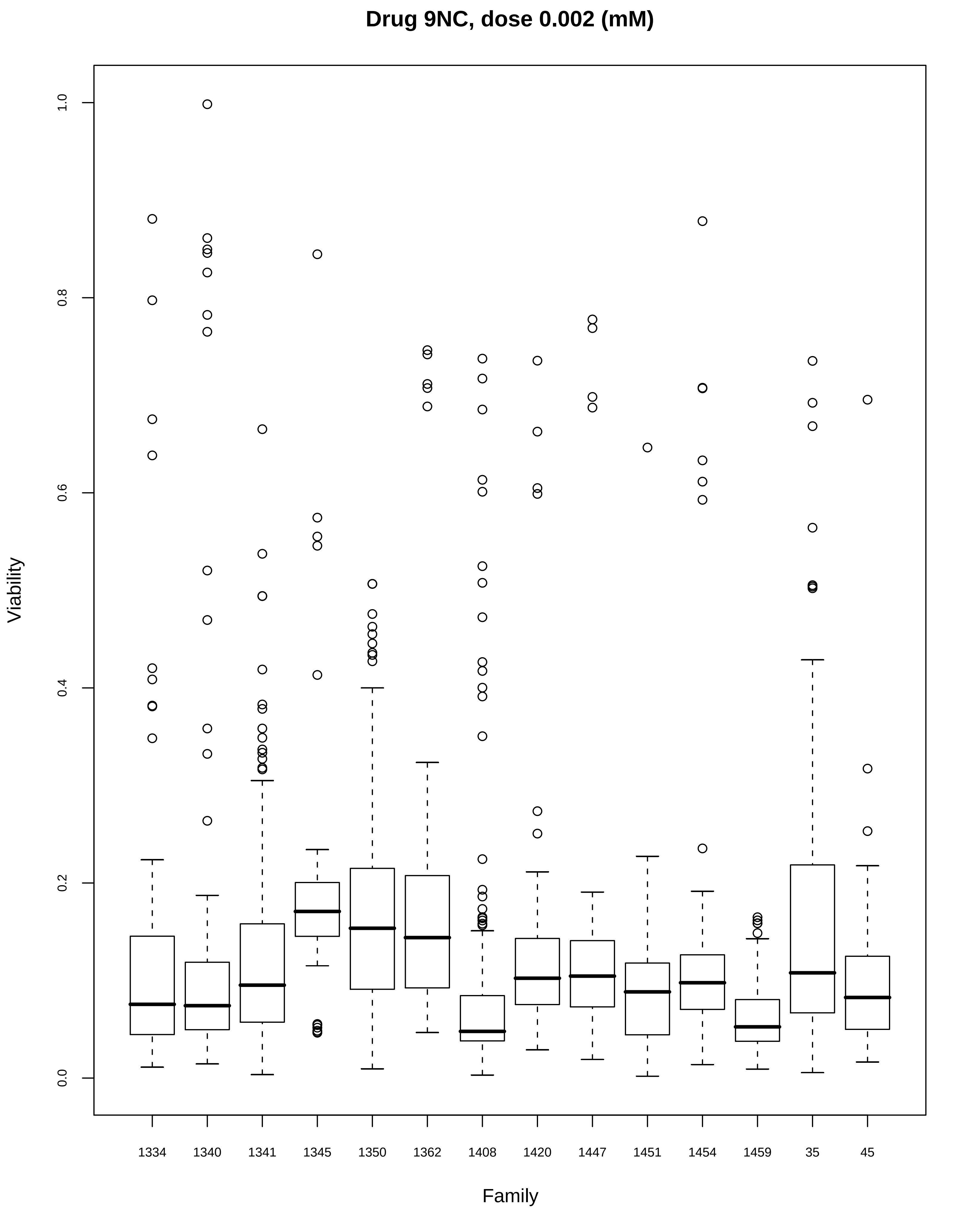

Drug 9NC, dose 0.002 (mM)
Viability
0.0
0.2
0.4
0.6
0.8
1.0
1334
1340
1341
1345
1350
1362
1408
1420
1447
1451
1454
1459
35
45
Family

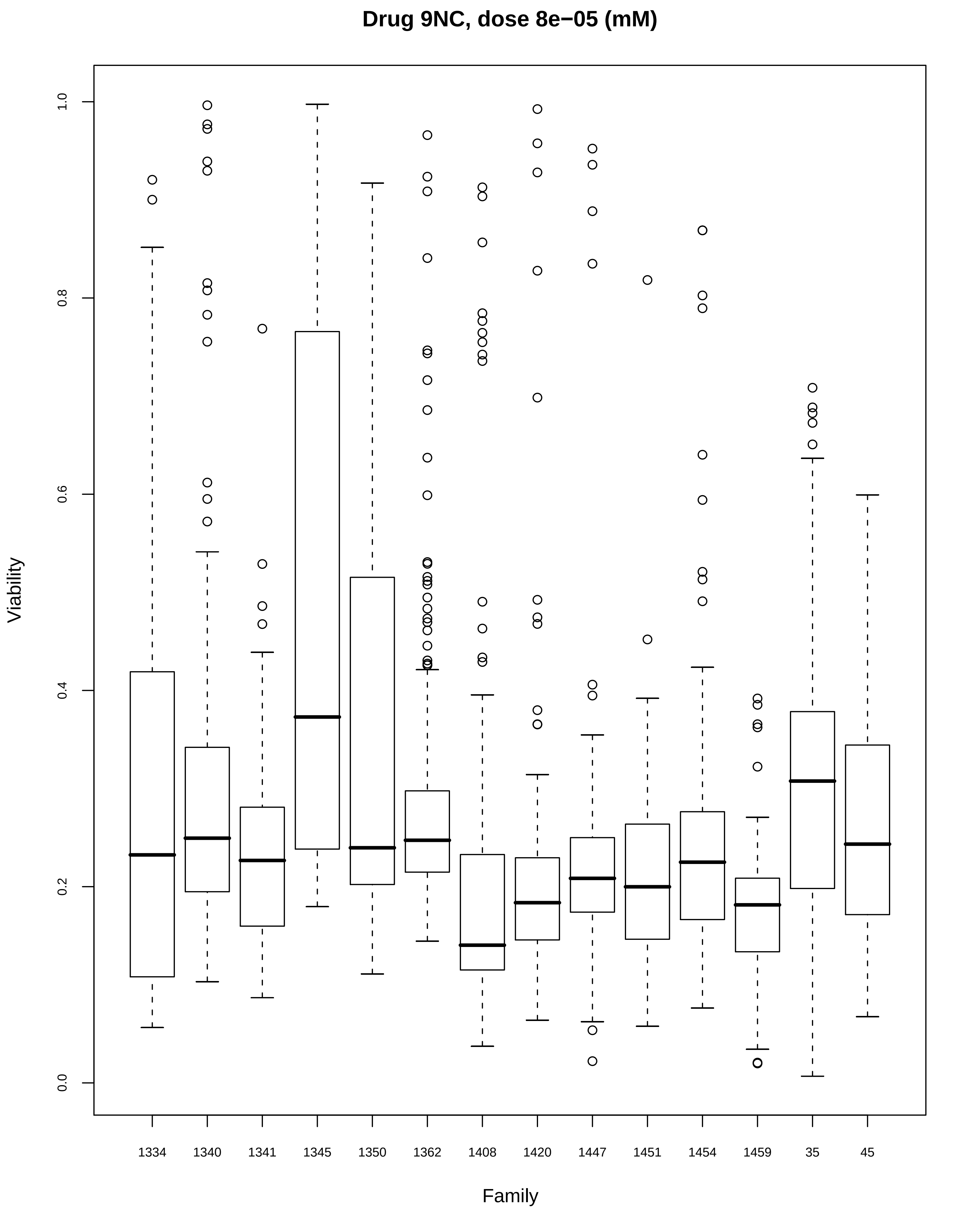

Drug 9NC, dose 8e−05 (mM)
Viability
0.0
0.2
0.4
0.6
0.8
1.0
1334
1340
1341
1345
1350
1362
1408
1420
1447
1451
1454
1459
35
45
Family

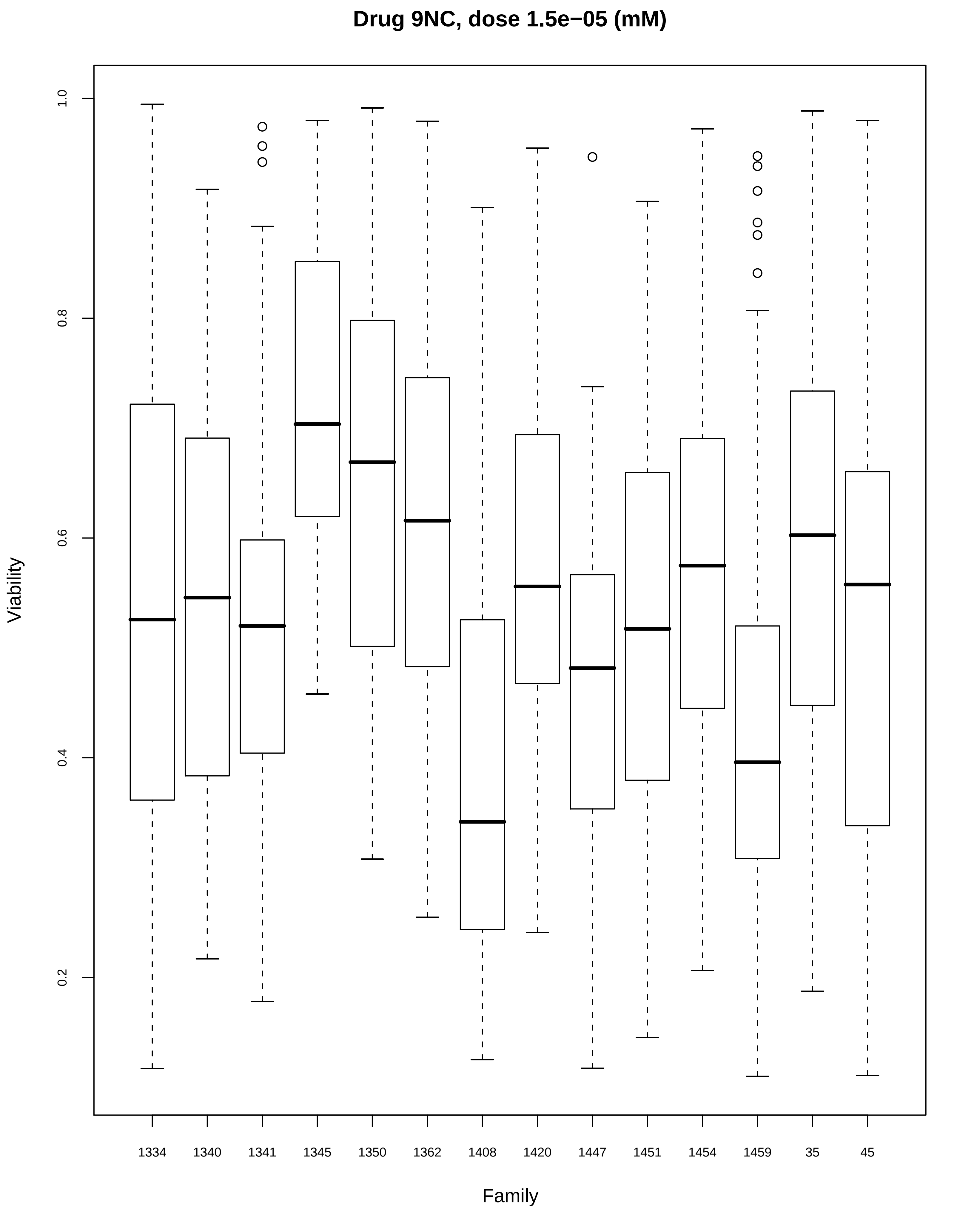

Drug 9NC, dose 1.5e−05 (mM)
Viability
0.2
0.4
0.6
0.8
1.0
Family
1334
1340
1341
1345
1350
1362
1408
1420
1447
1451
1454
1459
35
45

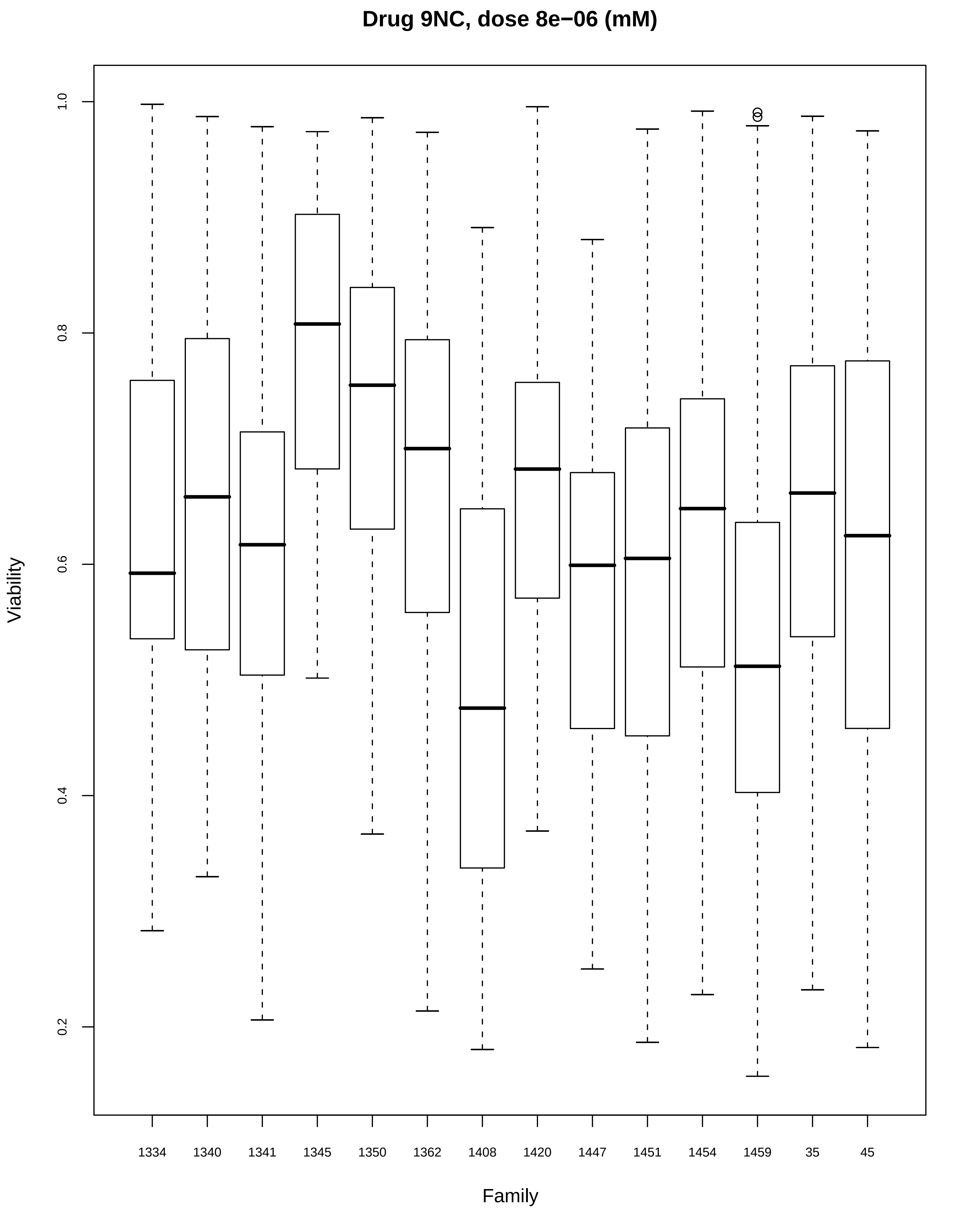
Drug 9NC, dose 8e−06 (mM)
Viability
0.2
0.4
0.6
0.8
1.0
1334
1340
1341
1345
1350
1362
1408
1420
1447
1451
1454
1459
35
45
Family

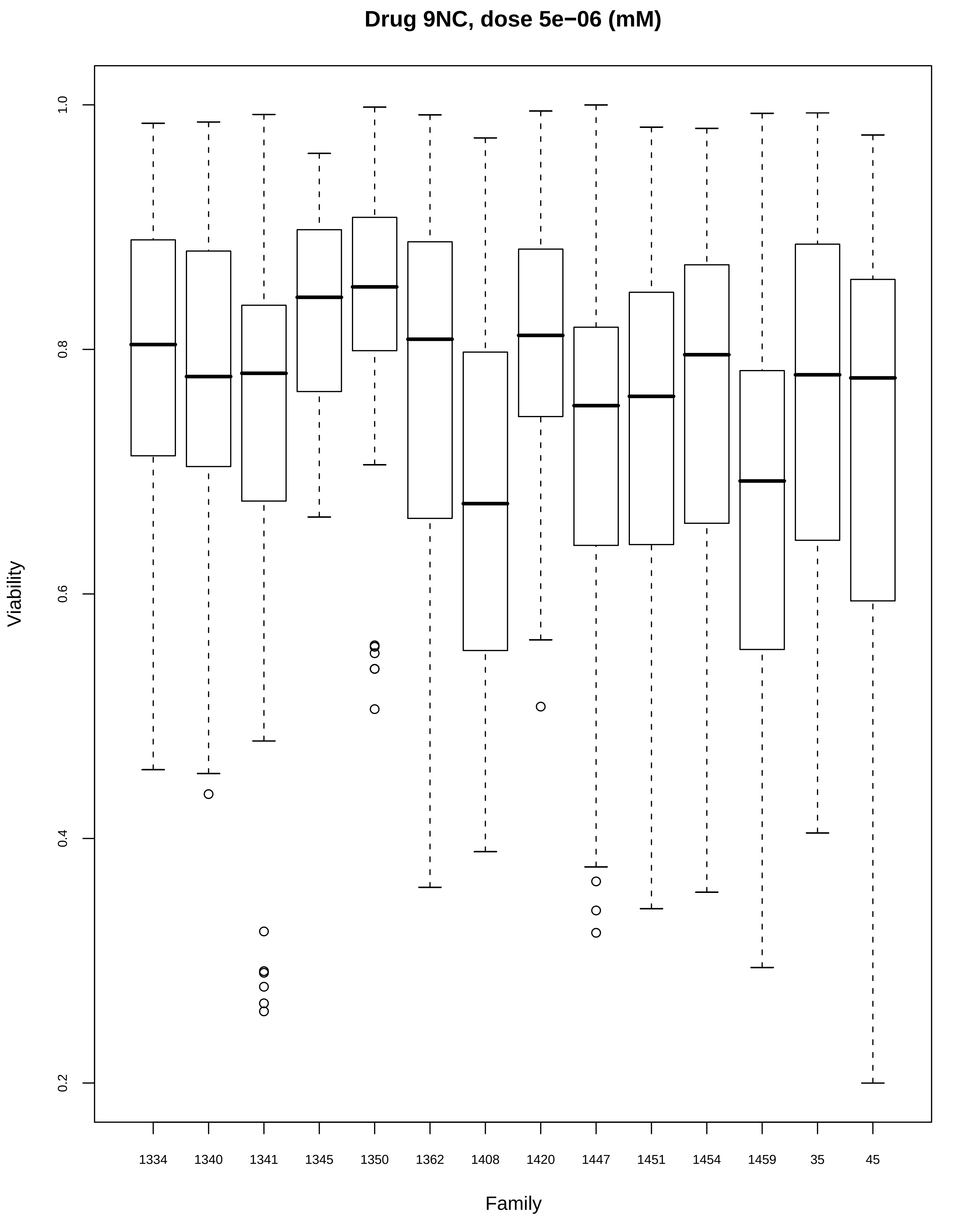

Drug 9NC, dose 5e−06 (mM)
Viability
1.0
0.8
0.6
0.4
0.2
Family
1334
1340
1341
1345
1350
1362
1408
1420
1447
1451
1454
1459
35
45

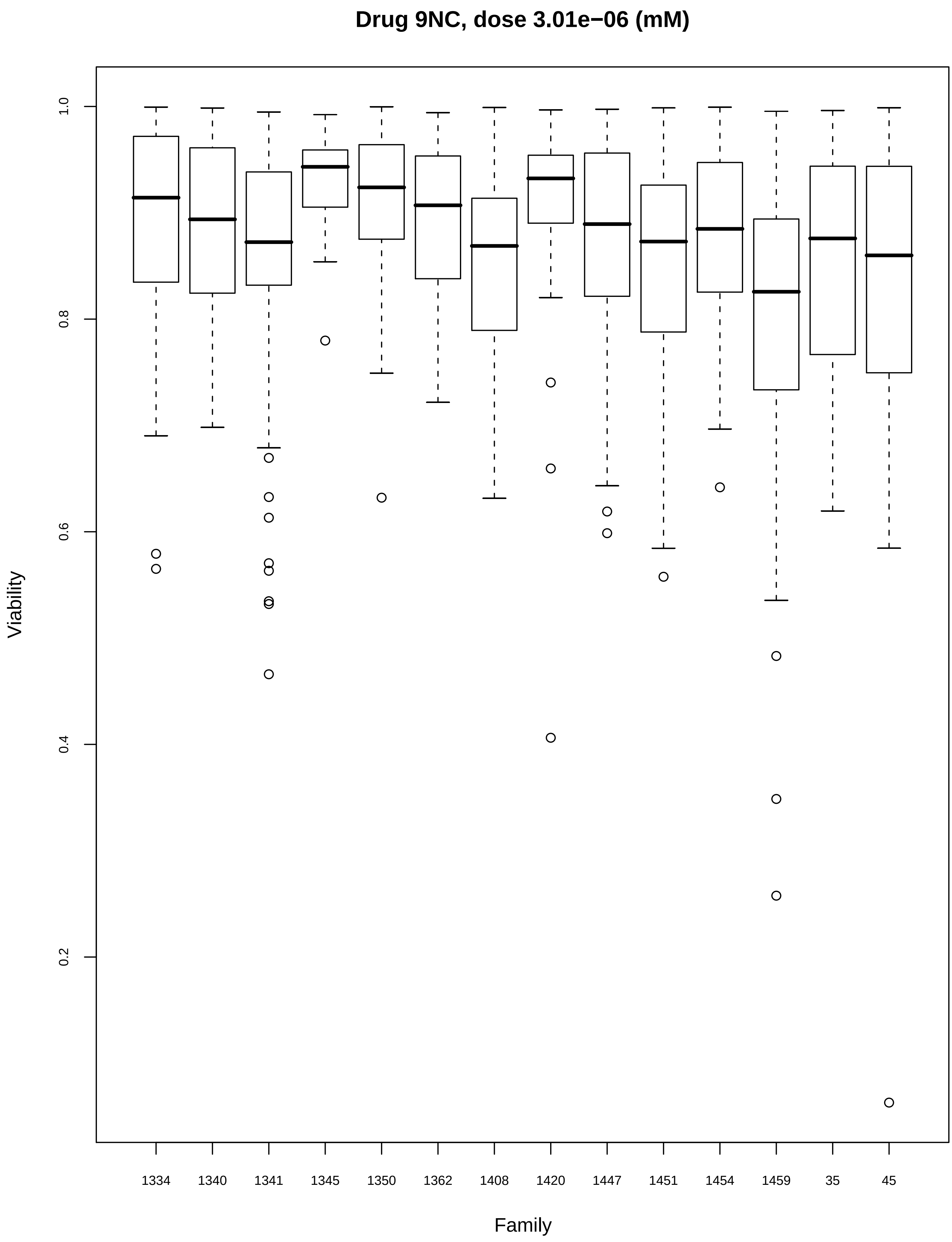
Drug 9NC, dose 3.01e−06 (mM)
Viability
1.0
0.8
0.6
0.4
0.2
Family
1334
1340
1341
1345
1350
1362
1408
1420
1447
1451
1454
1459
35
45

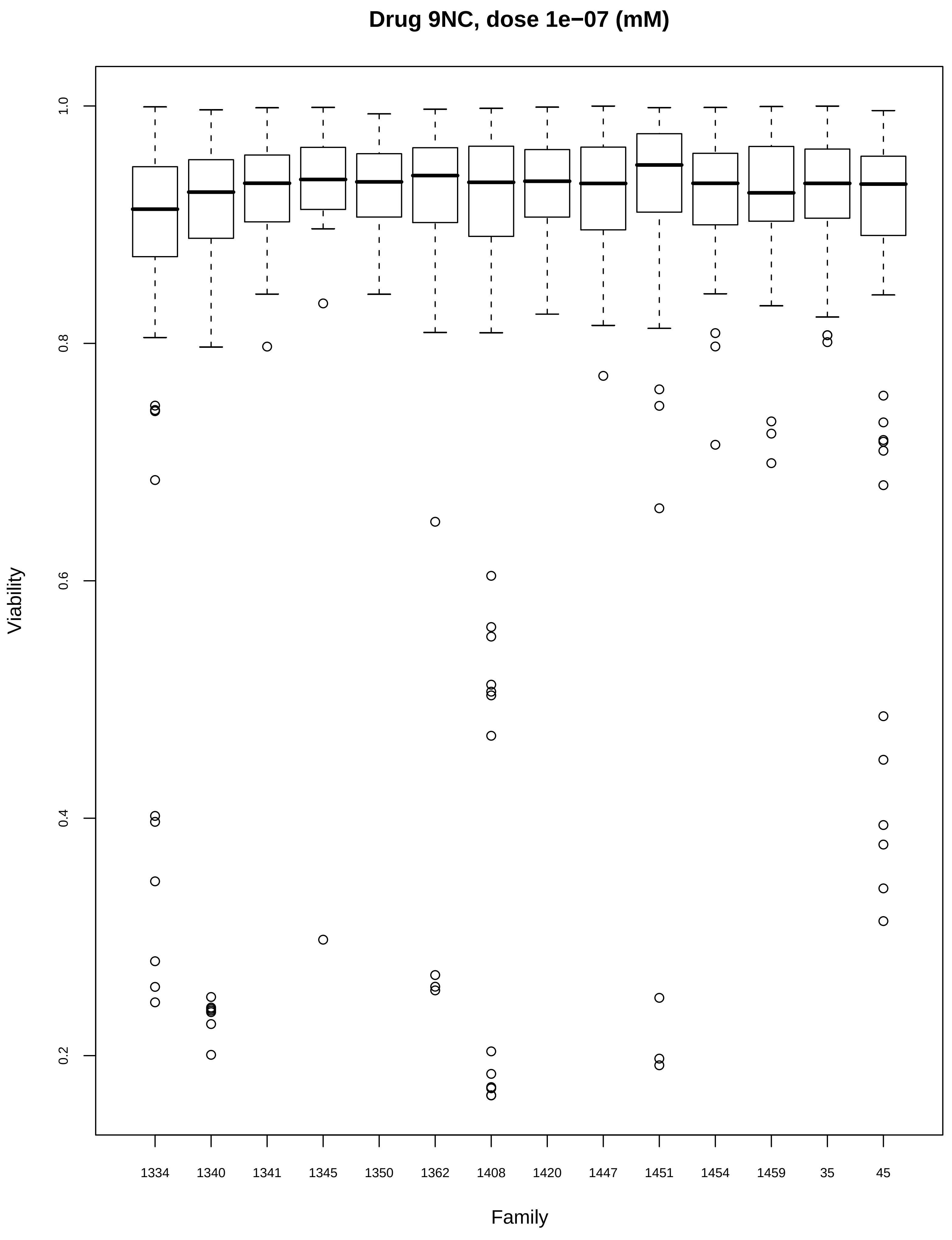
Drug 9NC, dose 1e−07 (mM)
Viability
1.0
0.8
0.6
0.4
0.2
1334
1340
1341
1345
1350
1362
1408
1420
1447
1451
1454
1459
35
45
Family

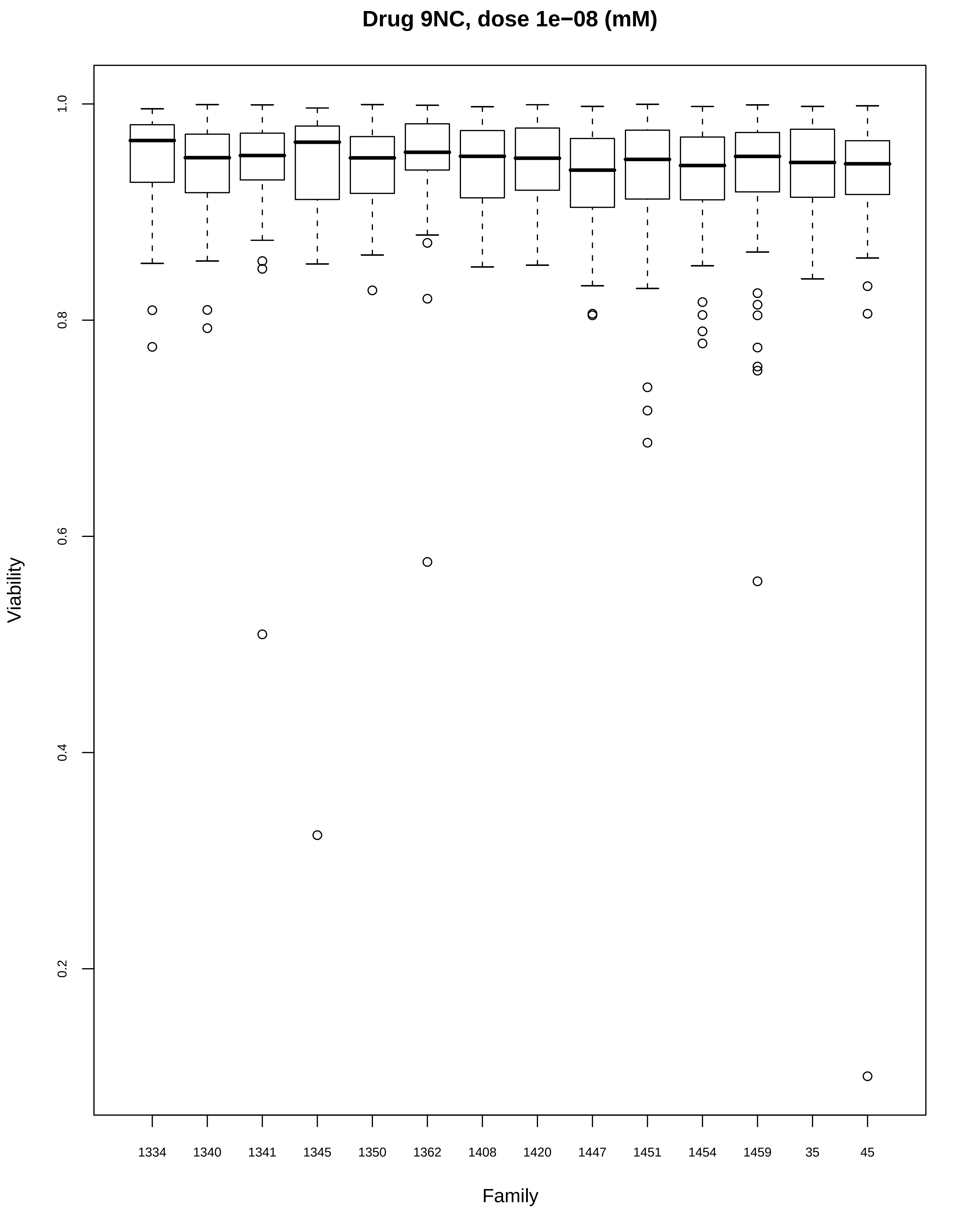
Drug 9NC, dose 1e−08 (mM)
Viability
0.2
0.4
0.6
0.8
1.0
1334
1340
1341
1345
1350
1362
1408
1420
1447
1451
1454
1459
35
45
Family

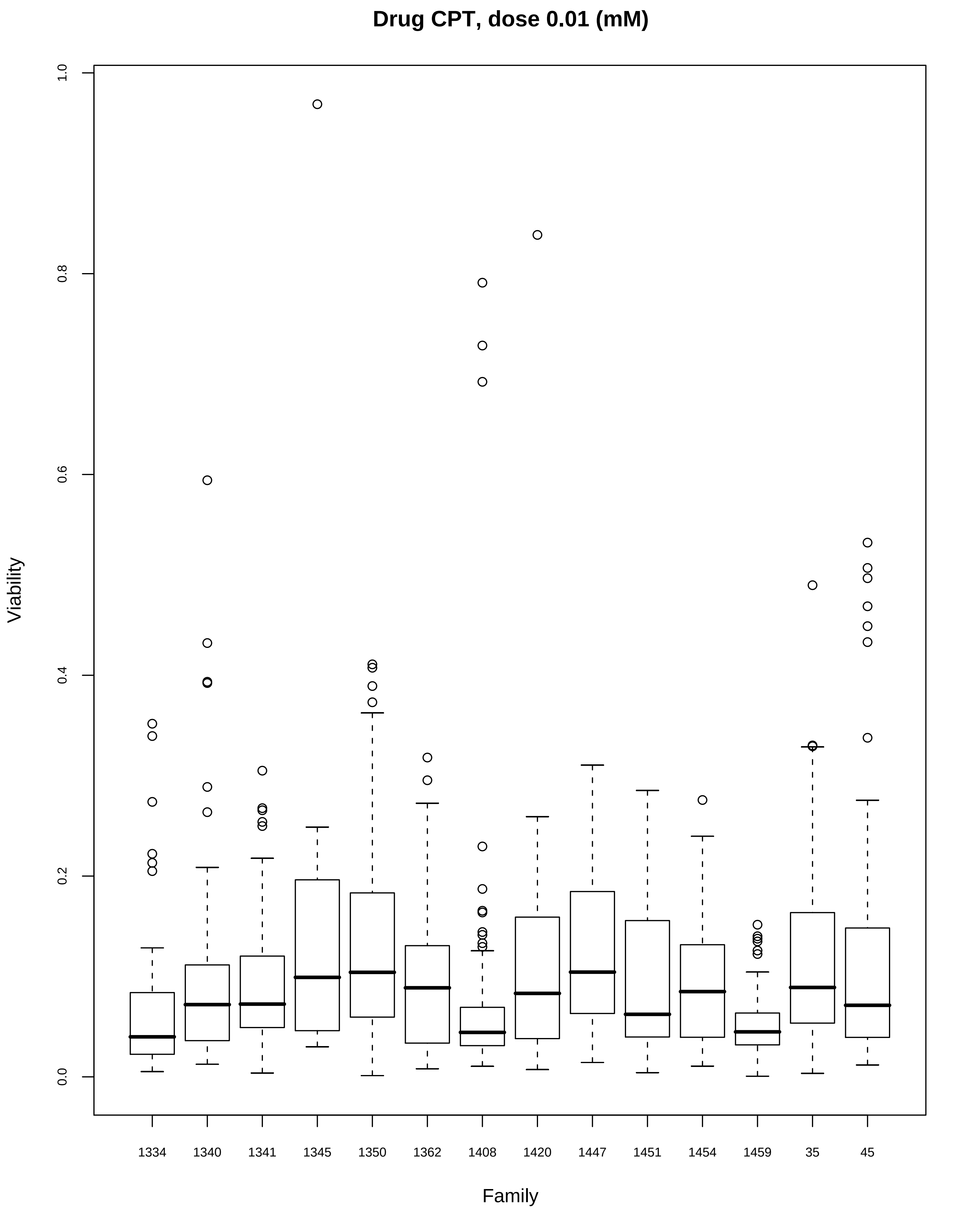
Drug CPT, dose 0.01 (mM)
Viability
0.0
0.2
0.4
0.6
0.8
1.0
1334
1340
1341
1345
1350
1362
1408
1420
1447
1451
1454
1459
35
45
Family

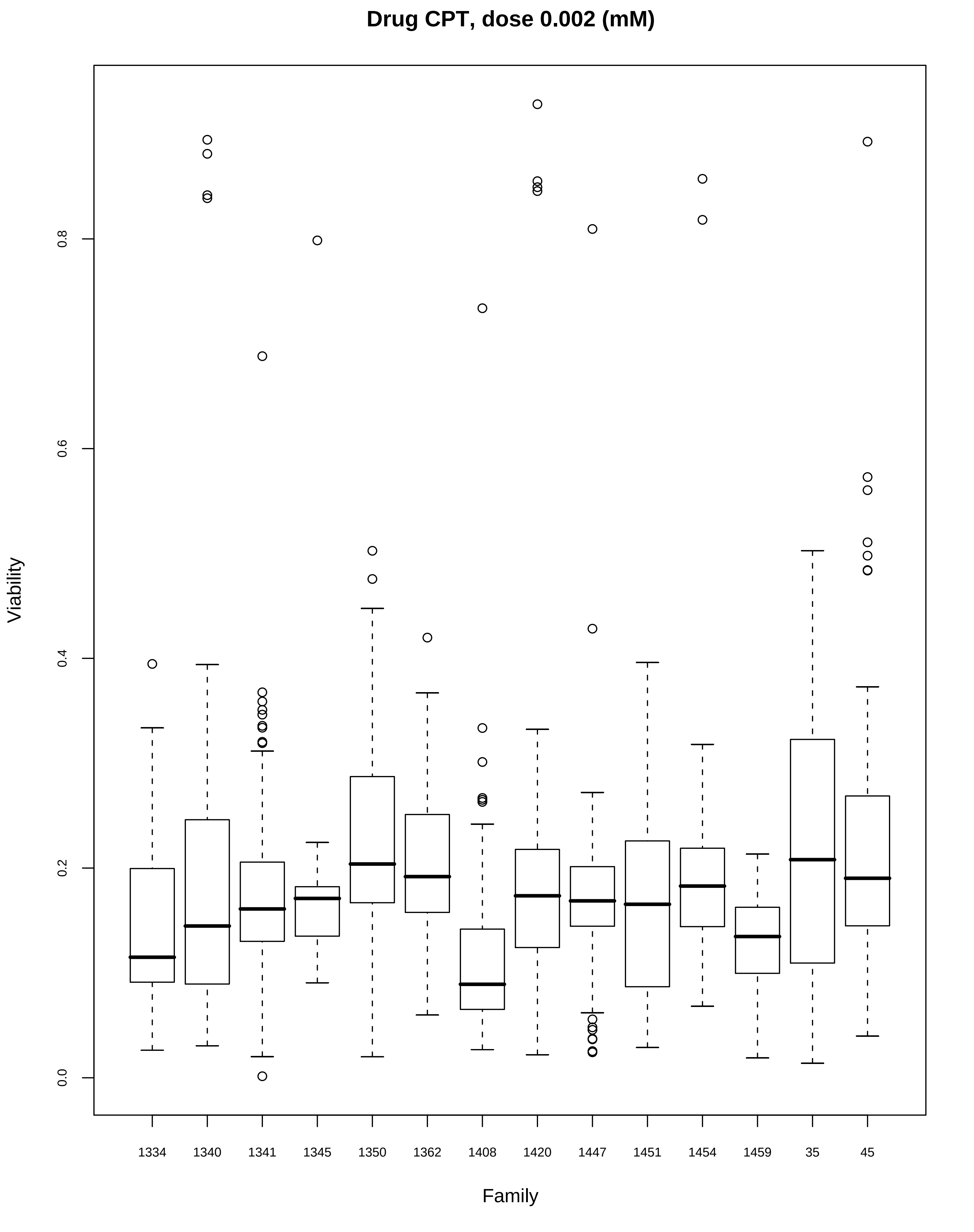
Drug CPT, dose 0.002 (mM)
Viability
0.0
0.2
0.4
0.6
0.8
1334
1340
1341
1345
1350
1362
1408
1420
1447
1451
1454
1459
35
45
Family

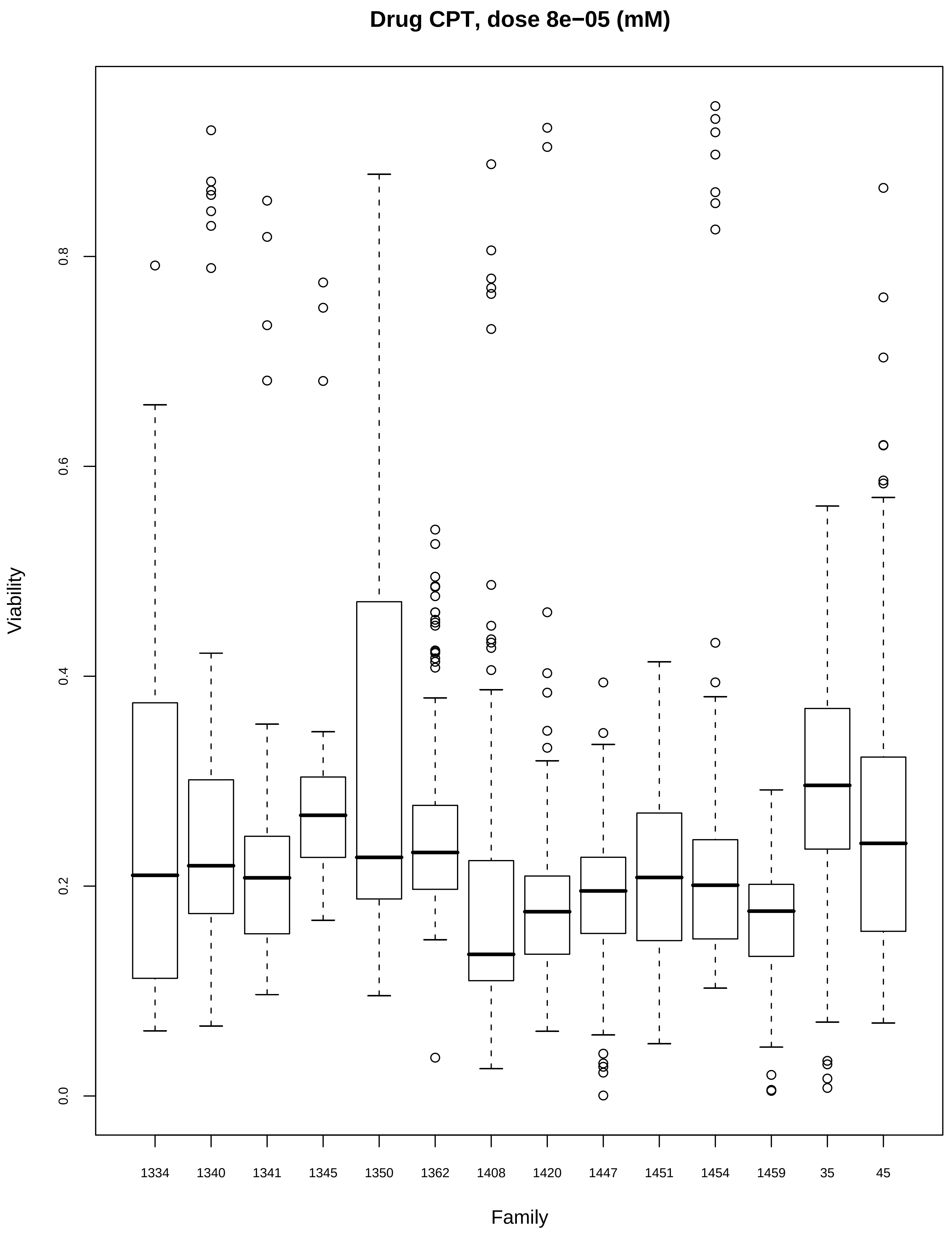
Drug CPT, dose 8e−05 (mM)
Viability
0.0
0.2
0.4
0.6
0.8
Family
1334
1340
1341
1345
1350
1362
1408
1420
1447
1451
1454
1459
35
45

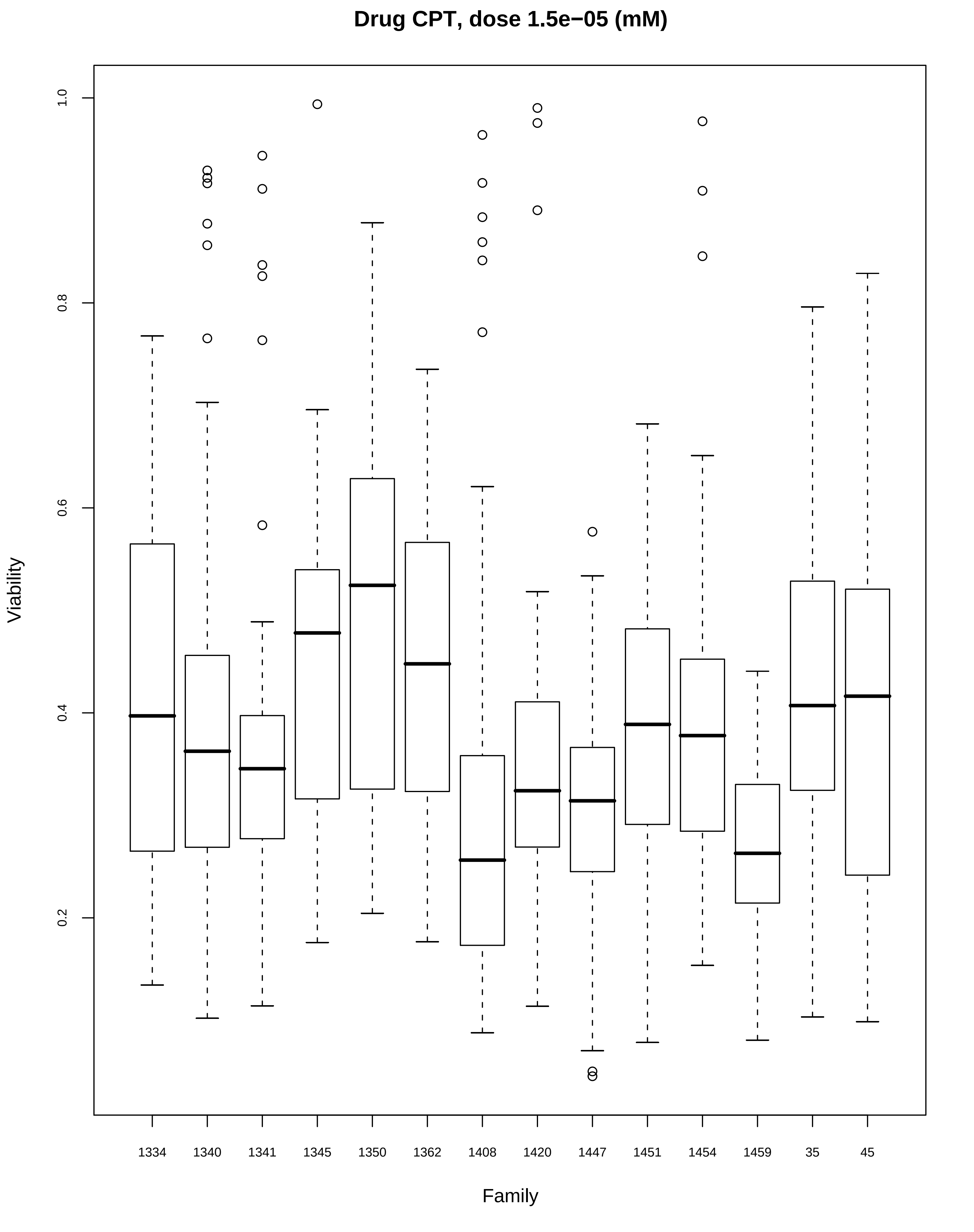

Drug CPT, dose 1.5e−05 (mM)
Viability
0.2
0.4
0.6
0.8
1.0
1334
1340
1341
1345
1350
1362
1408
1420
1447
1451
1454
1459
35
45
Family

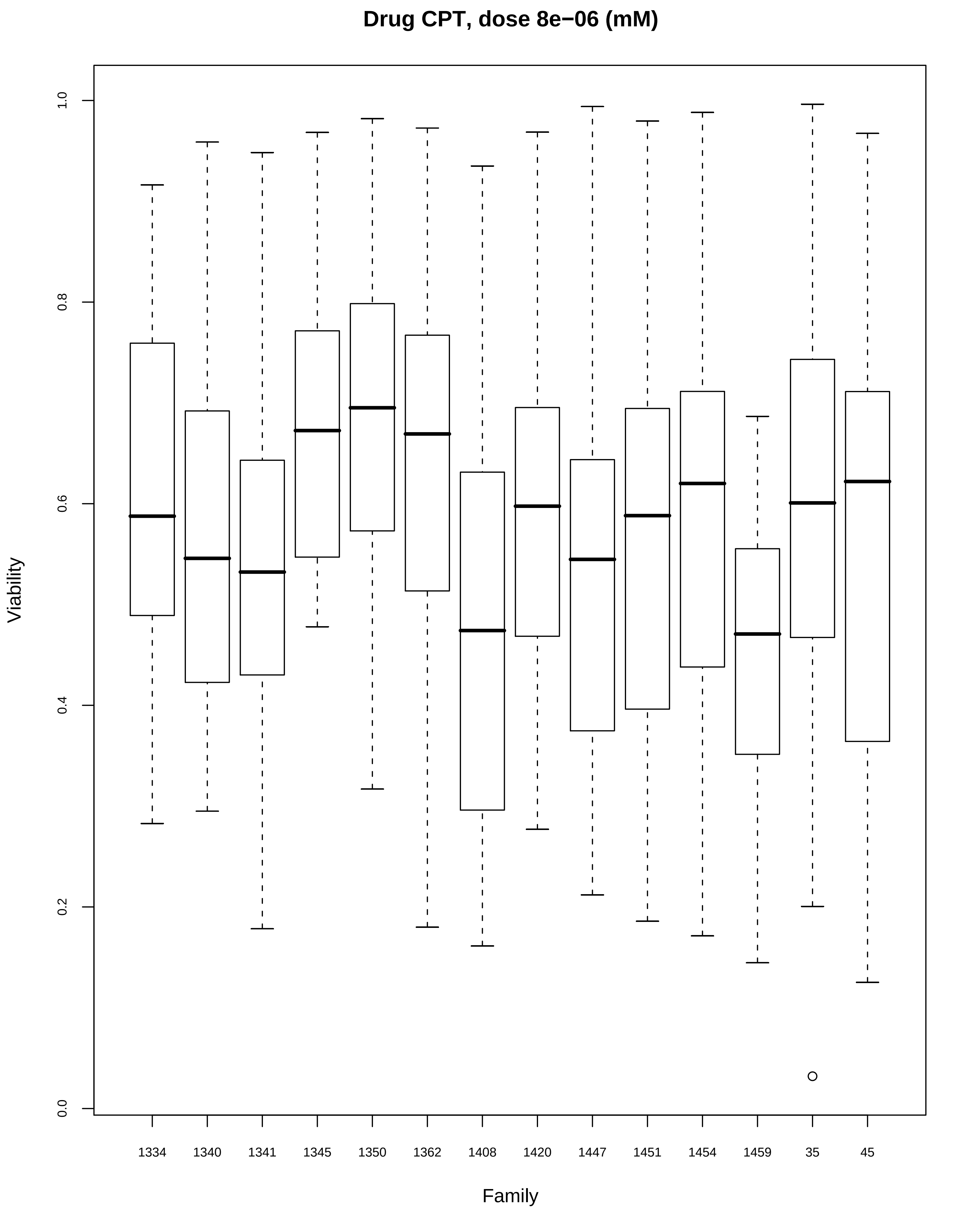
Drug CPT, dose 8e−06 (mM)
Viability
0.0
0.2
0.4
0.6
0.8
1.0
1334
1340
1341
1345
1350
1362
1408
1420
1447
1451
1454
1459
35
45
Family

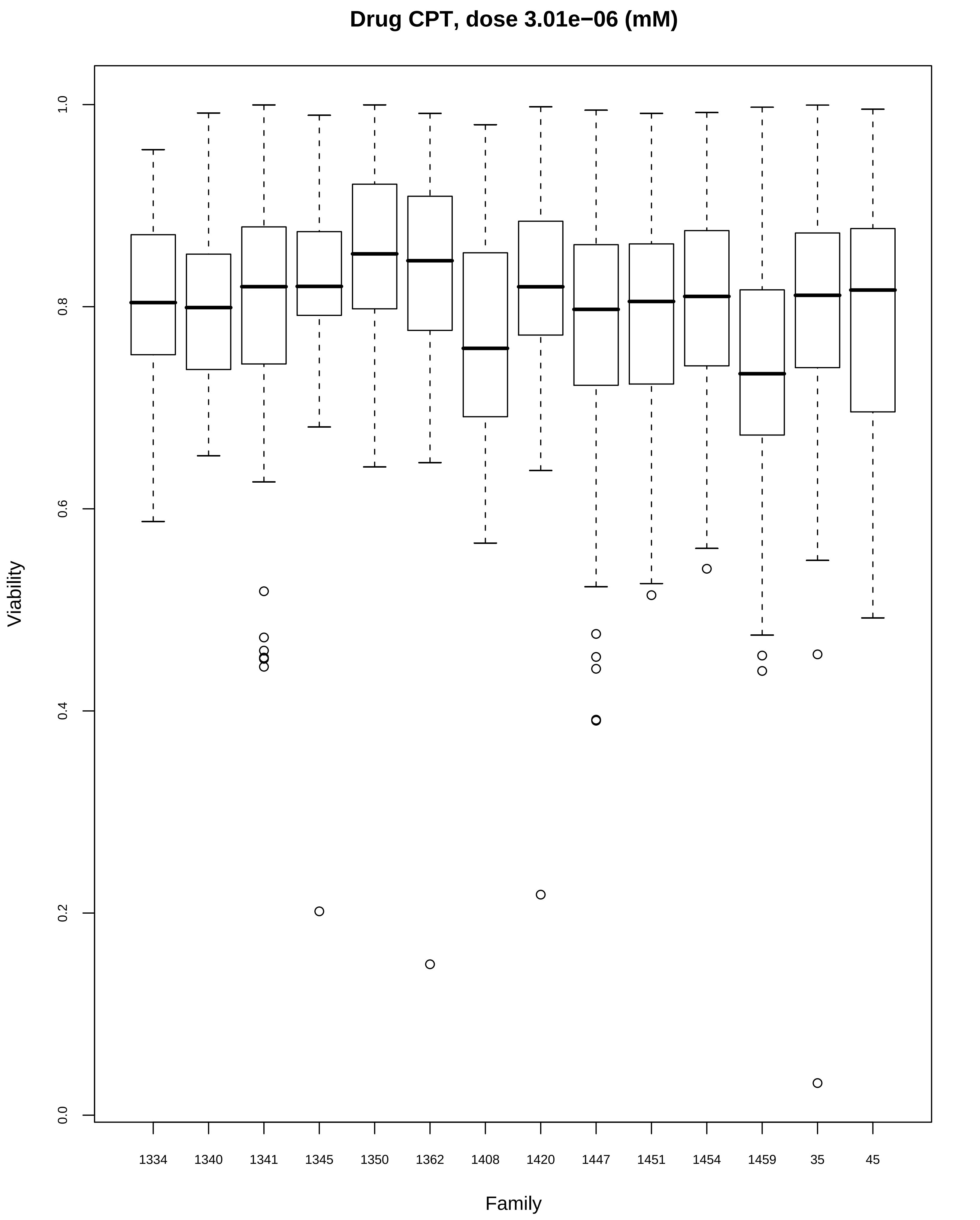
Drug CPT, dose 3.01e−06 (mM)
Viability
0.0
0.2
0.4
0.6
0.8
1.0
1334
1340
1341
1345
1350
1362
1408
1420
1447
1451
1454
1459
35
45
Family

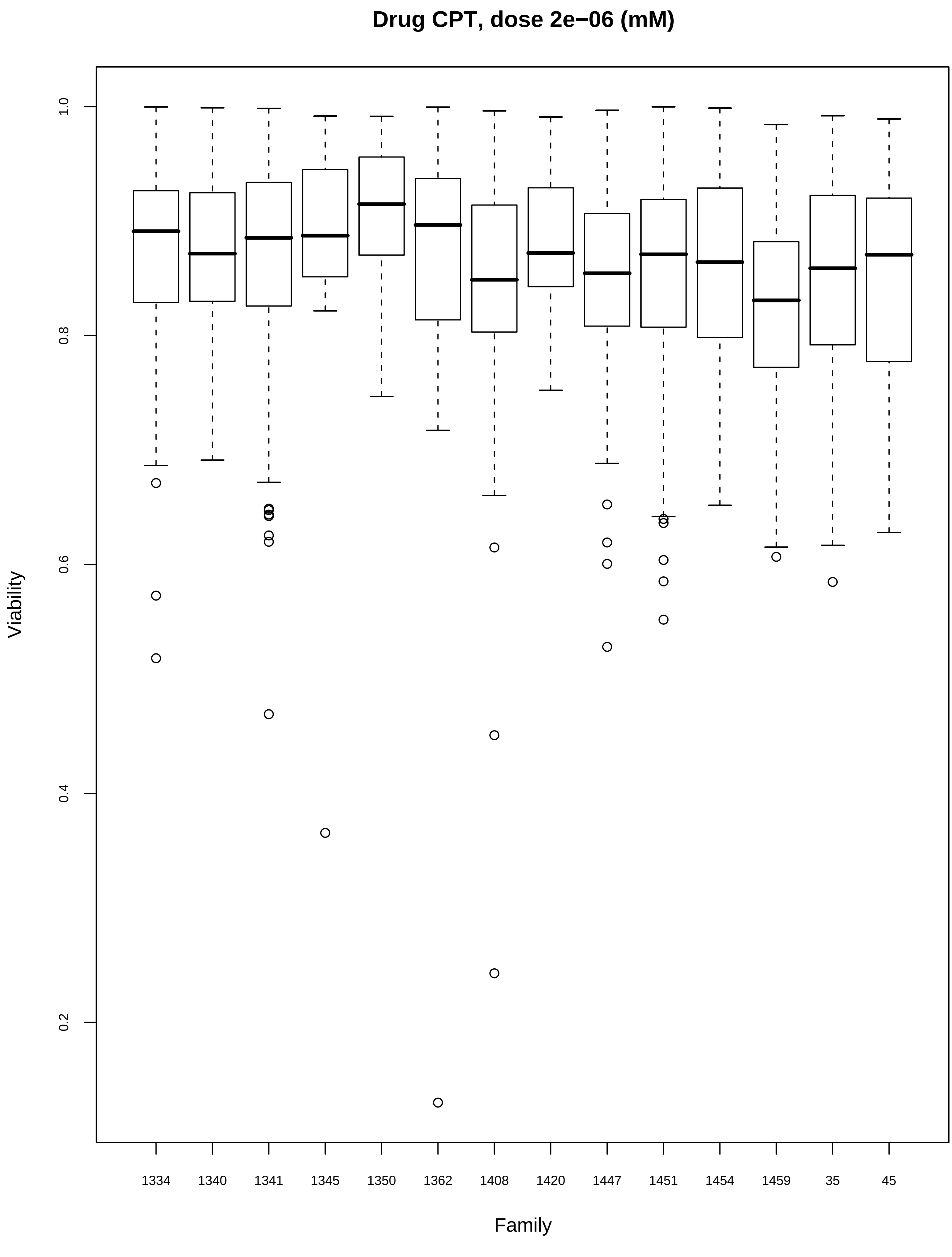

Drug CPT, dose 2e−06 (mM)
Viability
0.2
0.4
0.6
0.8
1.0
1334
1340
1341
1345
1350
1362
1408
1420
1447
1451
1454
1459
35
45
Family

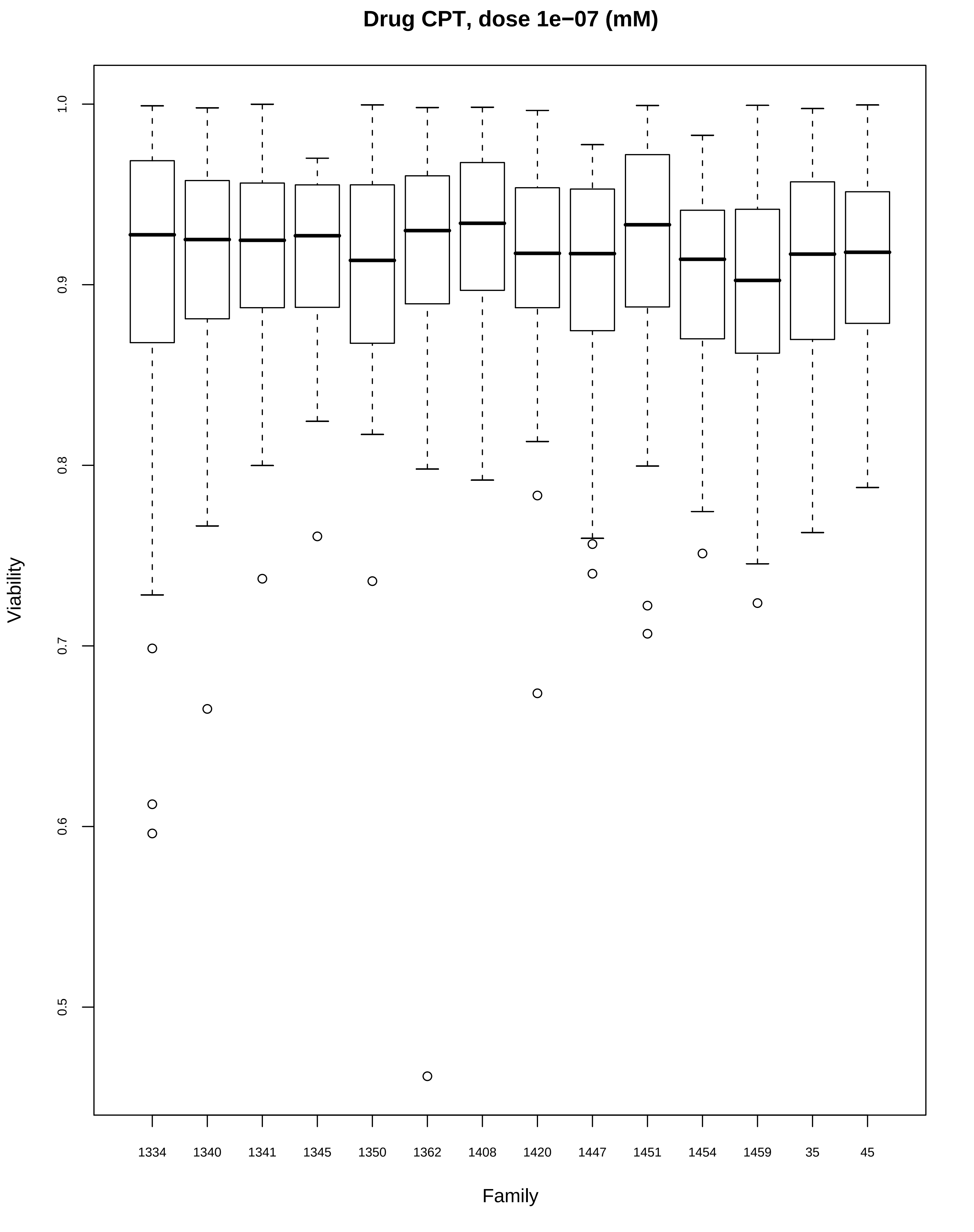

Drug CPT, dose 1e−07 (mM)
Viability
Family
0.5
0.6
0.7
0.8
0.9
1.0
1334
1340
1341
1345
1350
1362
1408
1420
1447
1451
1454
1459
35
45

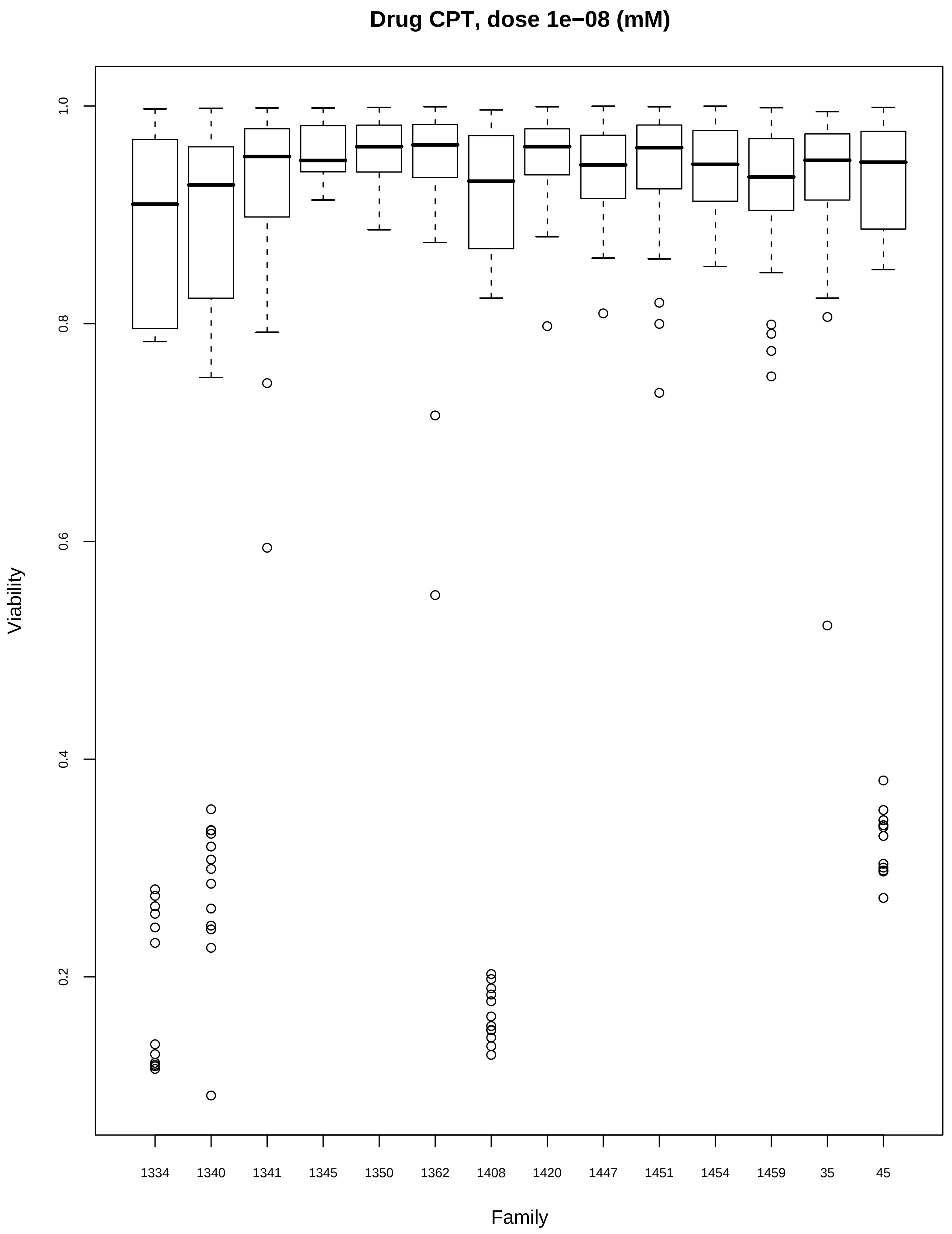
Drug CPT, dose 1e−08 (mM)
Viability
Family
0.2
0.4
0.6
0.8
1.0
1334
1340
1341
1345
1350
1362
1408
1420
1447
1451
1454
1459
35
45

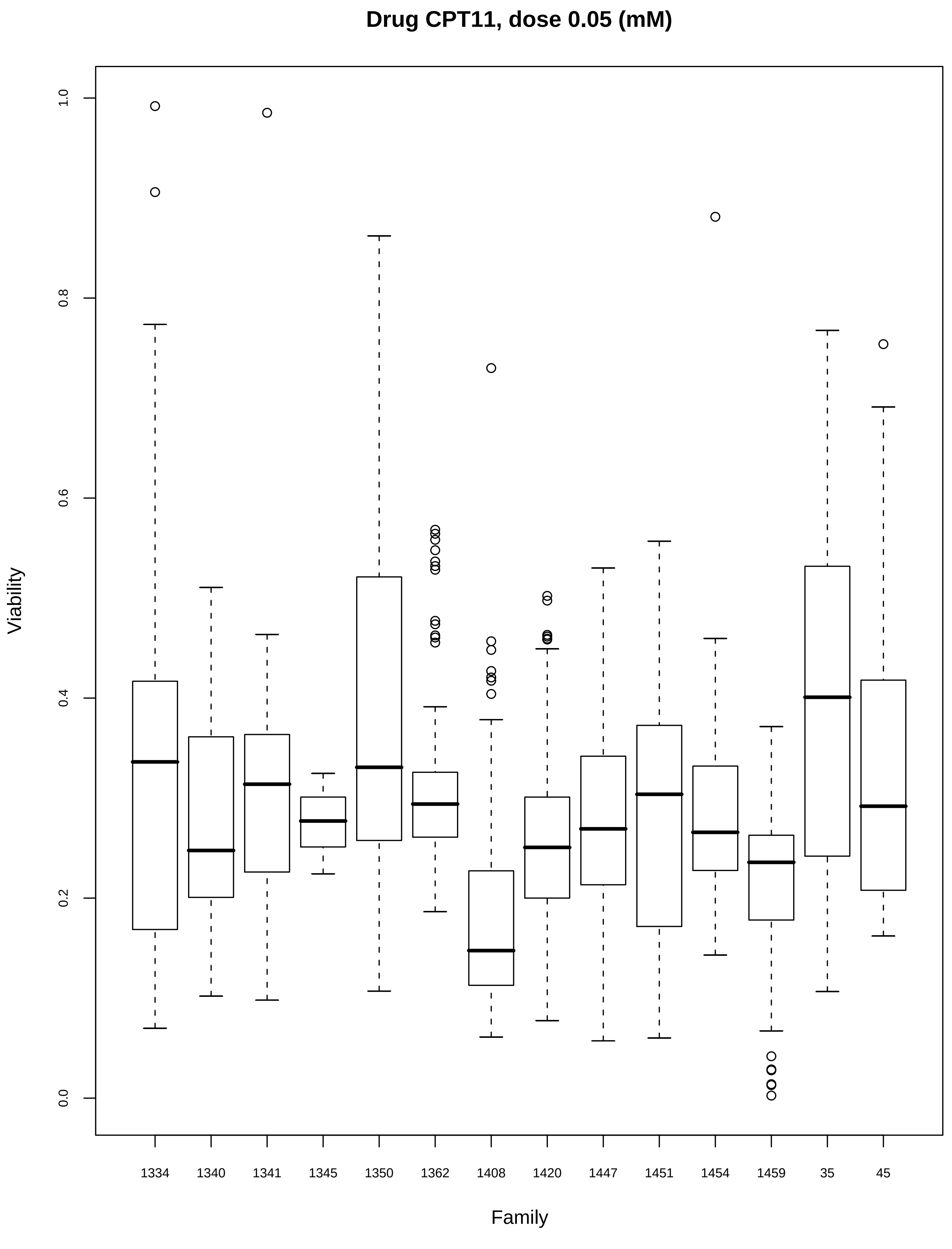
Drug CPT11, dose 0.05 (mM)
Viability
0.0
0.2
0.4
0.6
0.8
1.0
1334
1340
1341
1345
1350
1362
1408
1420
1447
1451
1454
1459
35
45
Family

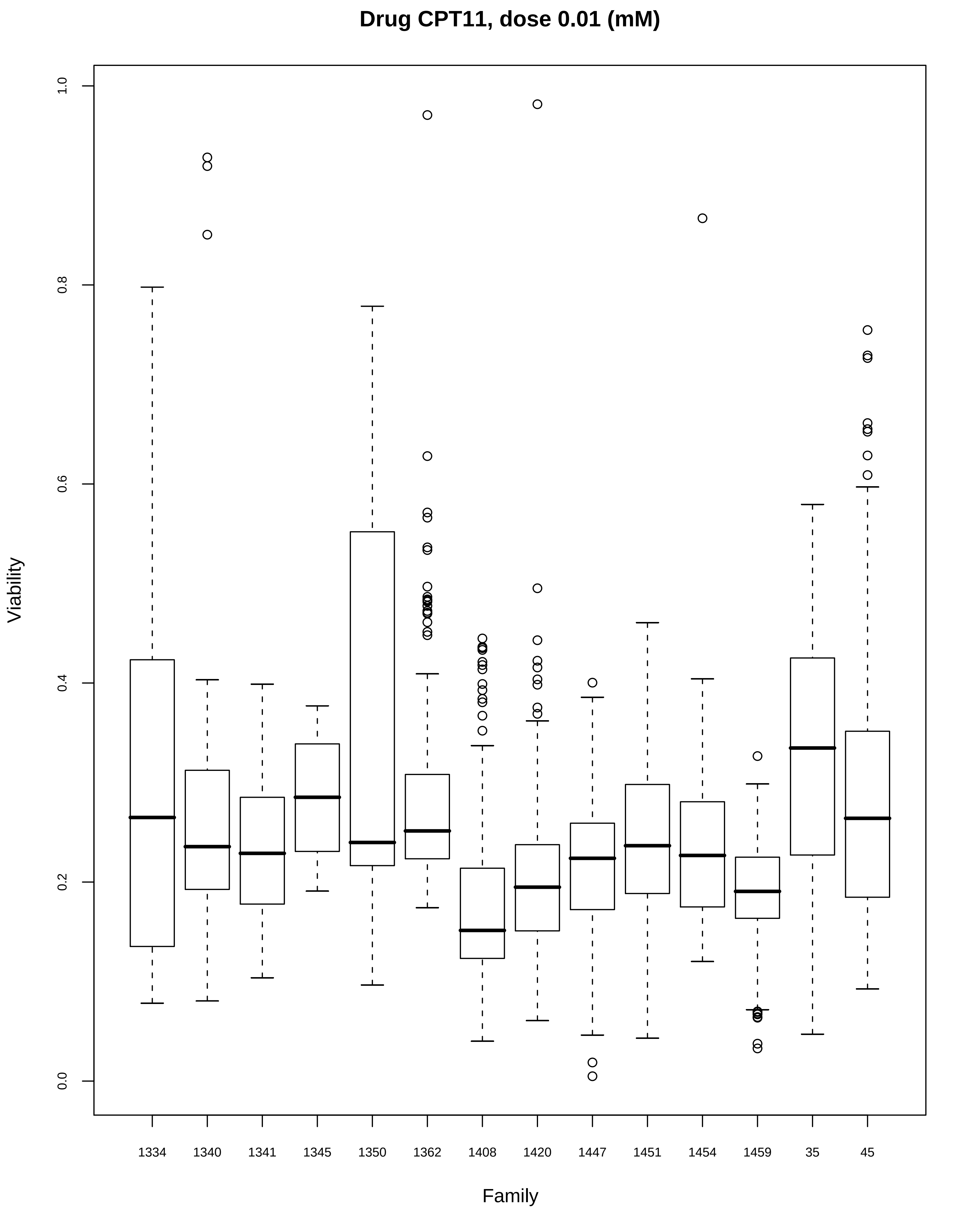
Drug CPT11, dose 0.01 (mM)
Viability
0.0
0.2
0.4
0.6
0.8
1.0
1334
1340
1341
1345
1350
1362
1408
1420
1447
1451
1454
1459
35
45
Family

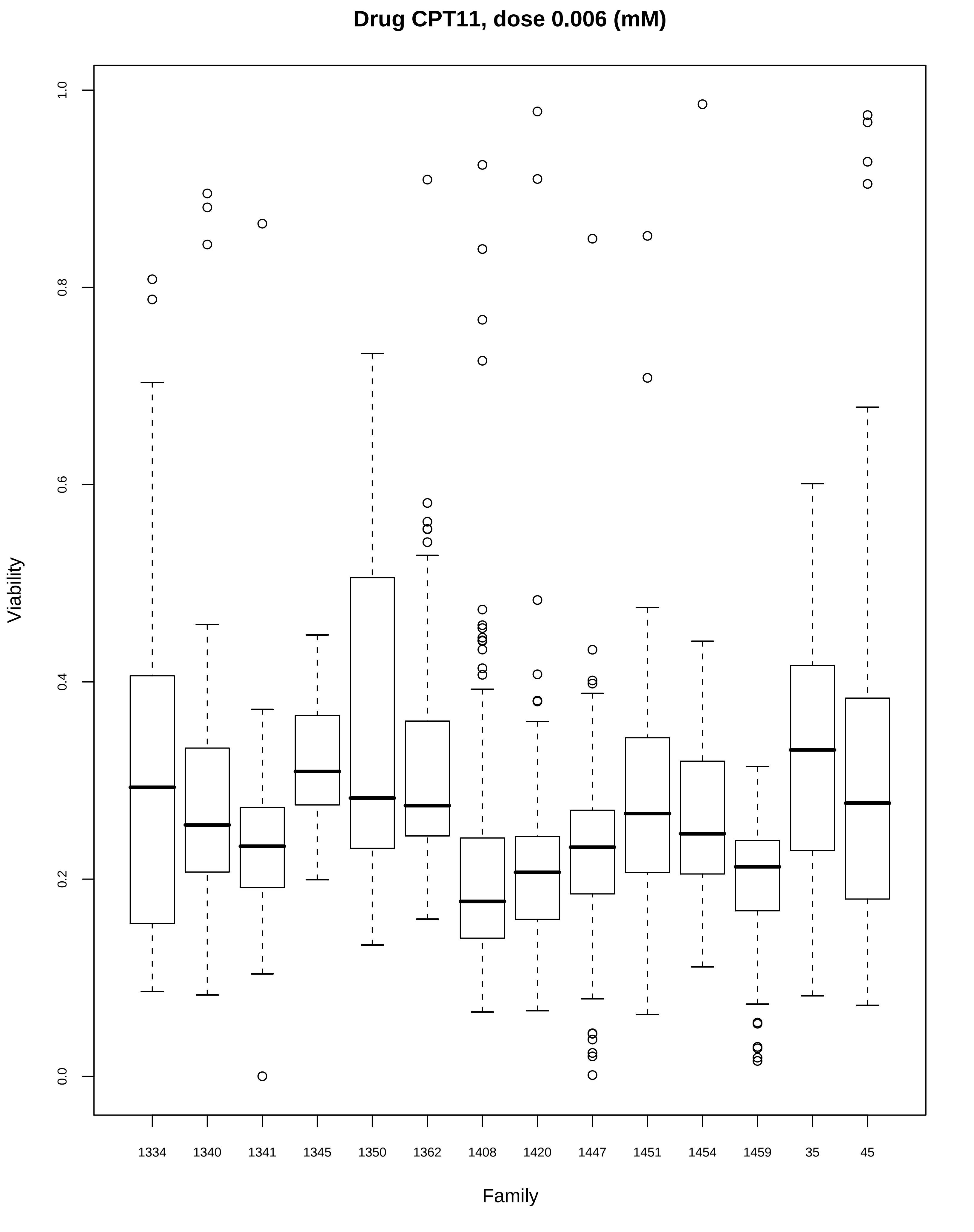
Drug CPT11, dose 0.006 (mM)
Viability
0.0
0.2
0.4
0.6
0.8
1.0
1334
1340
1341
1345
1350
1362
1408
1420
1447
1451
1454
1459
35
45
Family

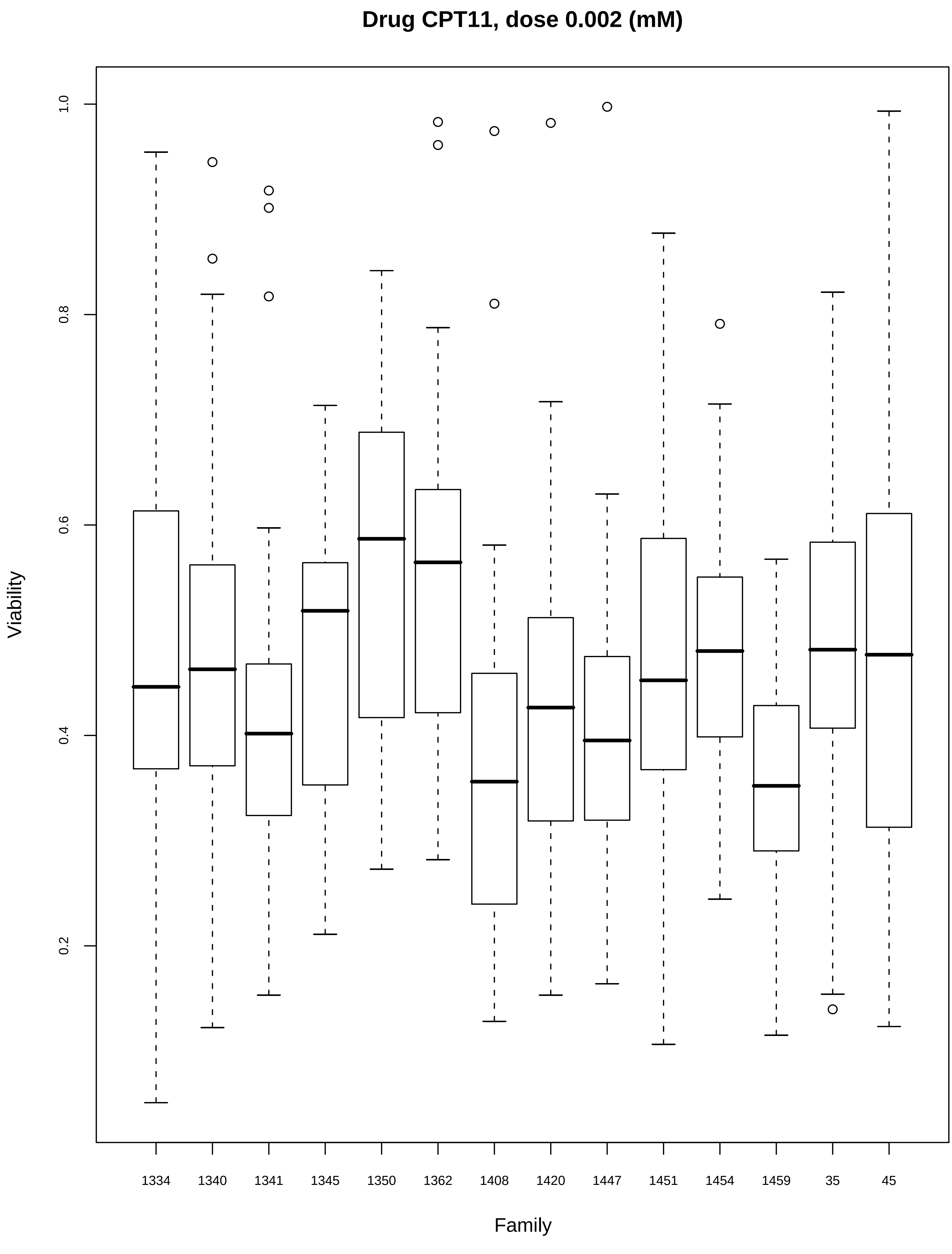

Drug CPT11, dose 0.002 (mM)
Viability
Family
1.0
0.8
0.6
0.4
0.2
1334
1340
1341
1345
1350
1362
1408
1420
1447
1451
1454
1459
35
45

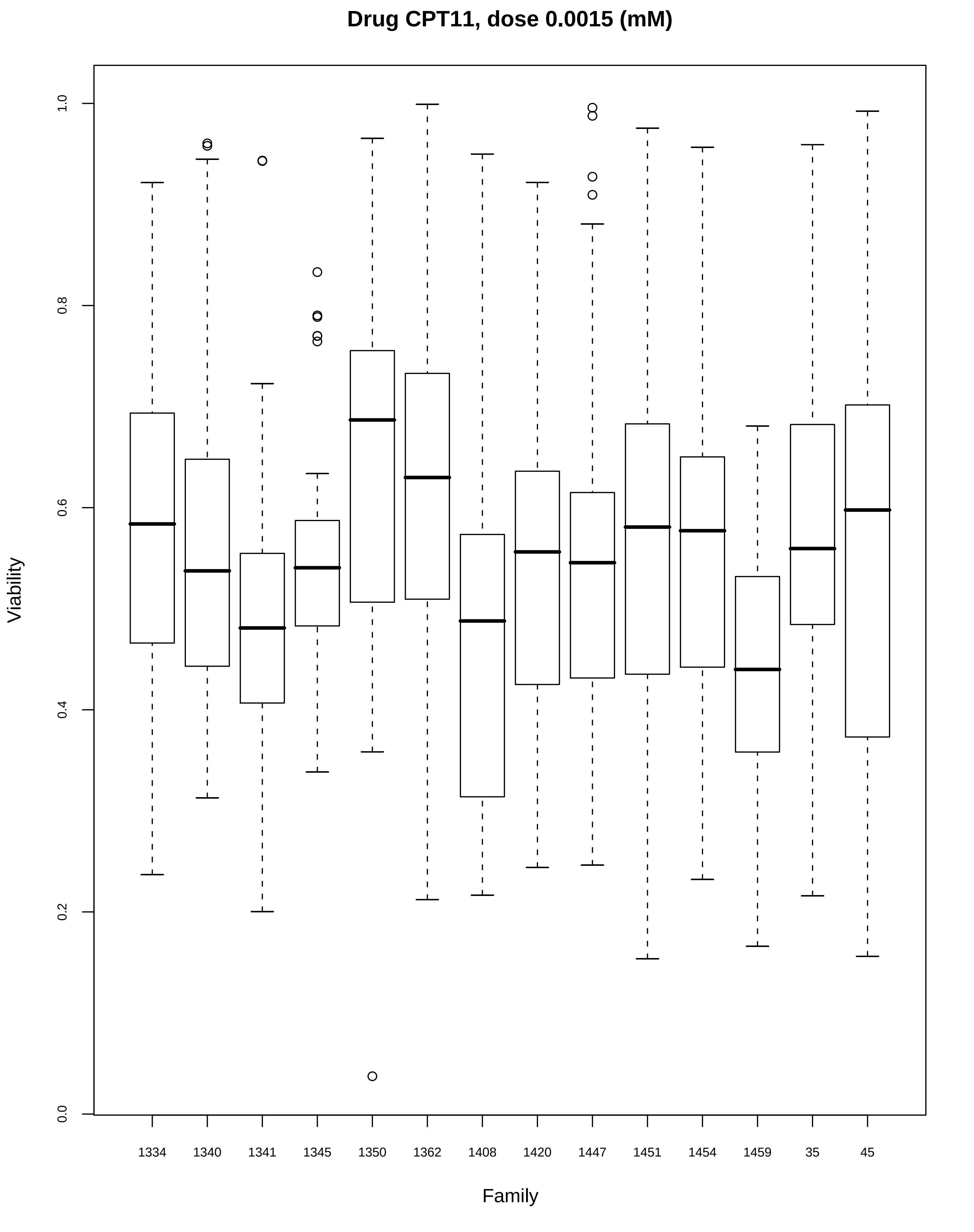

Drug CPT11, dose 0.0015 (mM)
Viability
0.0
0.2
0.4
0.6
0.8
1.0
1334
1340
1341
1345
1350
1362
1408
1420
1447
1451
1454
1459
35
45
Family

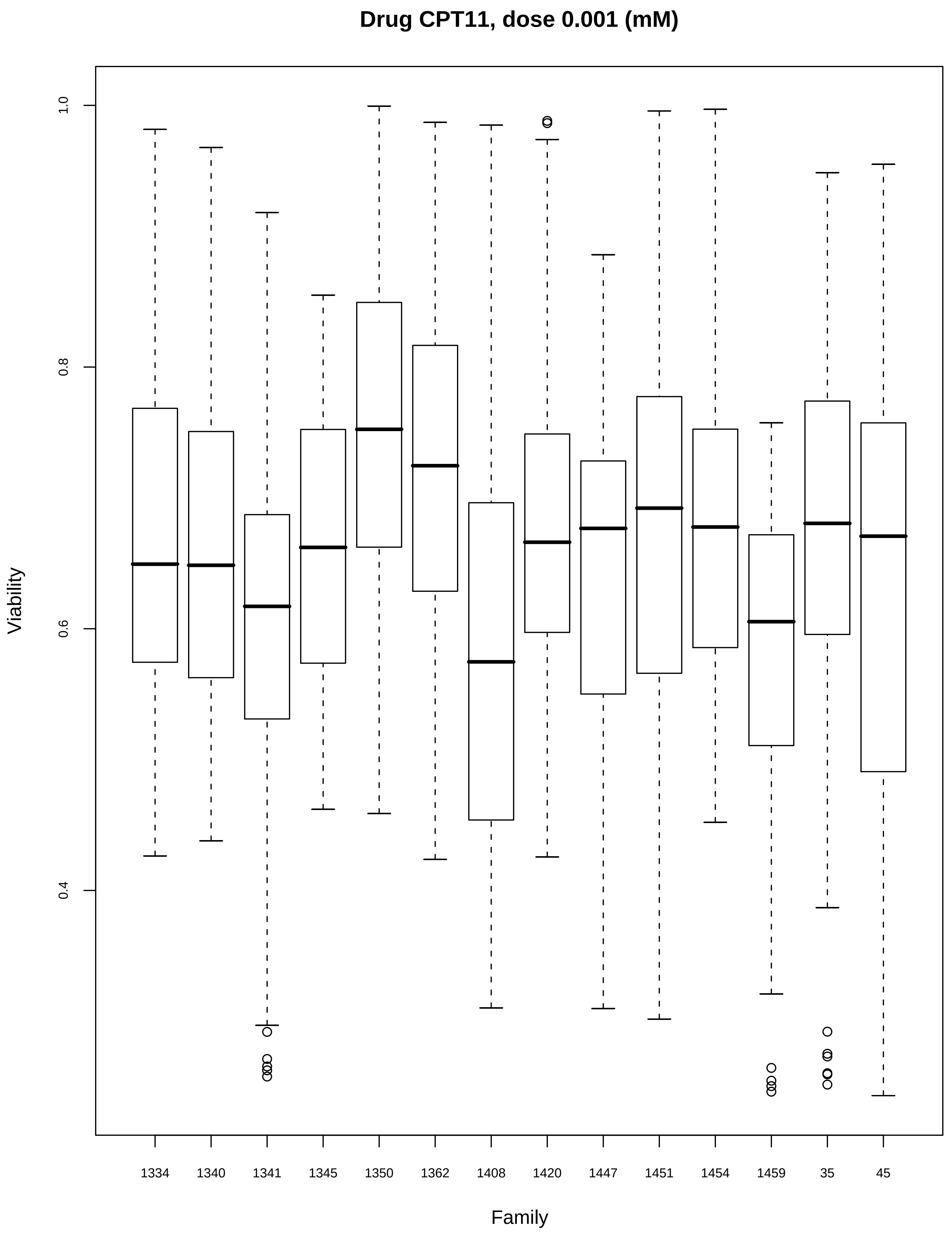
Drug CPT11, dose 0.001 (mM)
Viability
1.0
0.8
0.6
0.4
1334
1340
1341
1345
1350
1362
1408
1420
1447
1451
1454
1459
35
45
Family

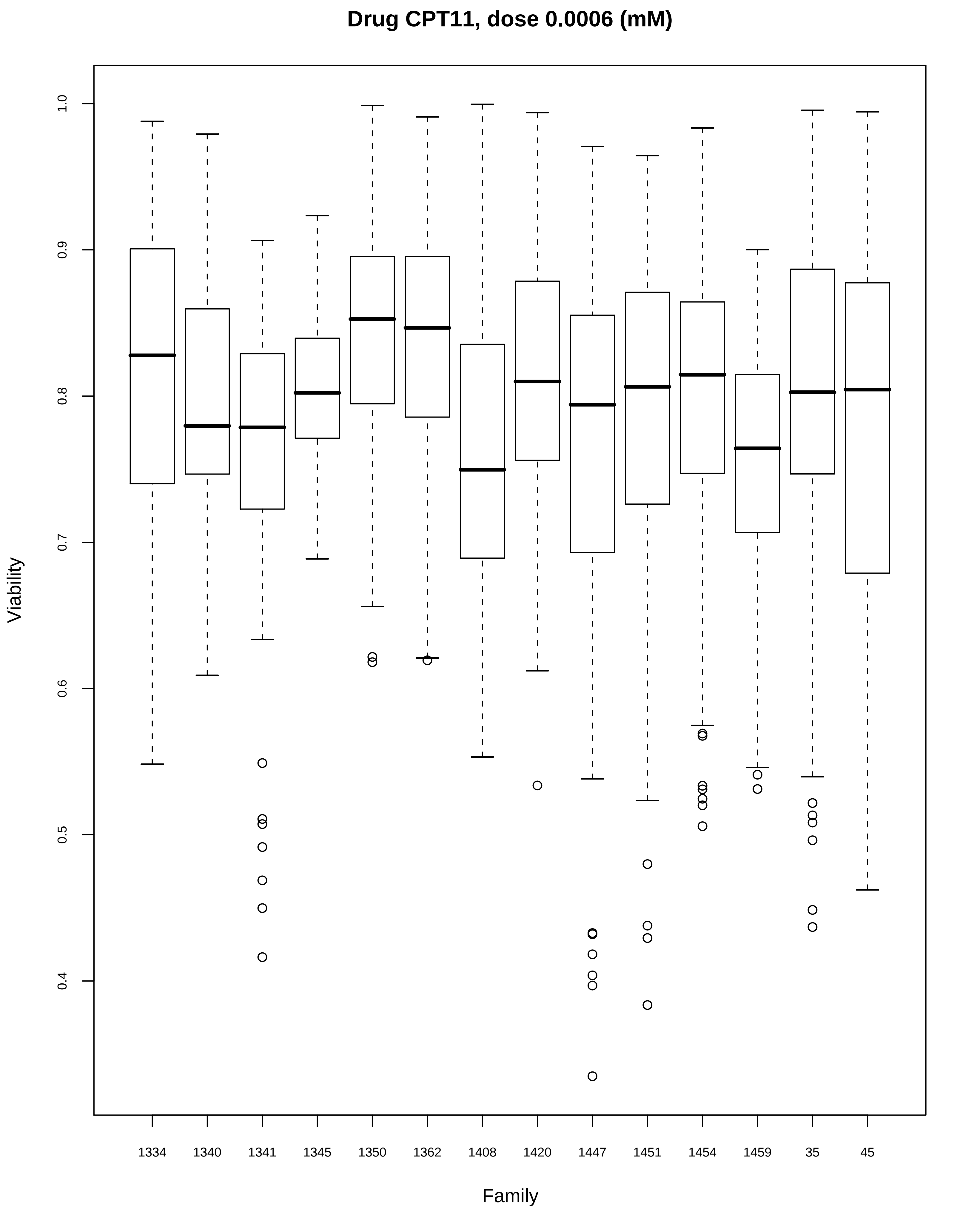
Drug CPT11, dose 0.0006 (mM)
Viability
0.4
0.5
0.6
0.7
0.8
0.9
1.0
1334
1340
1341
1345
1350
1362
1408
1420
1447
1451
1454
1459
35
45
Family

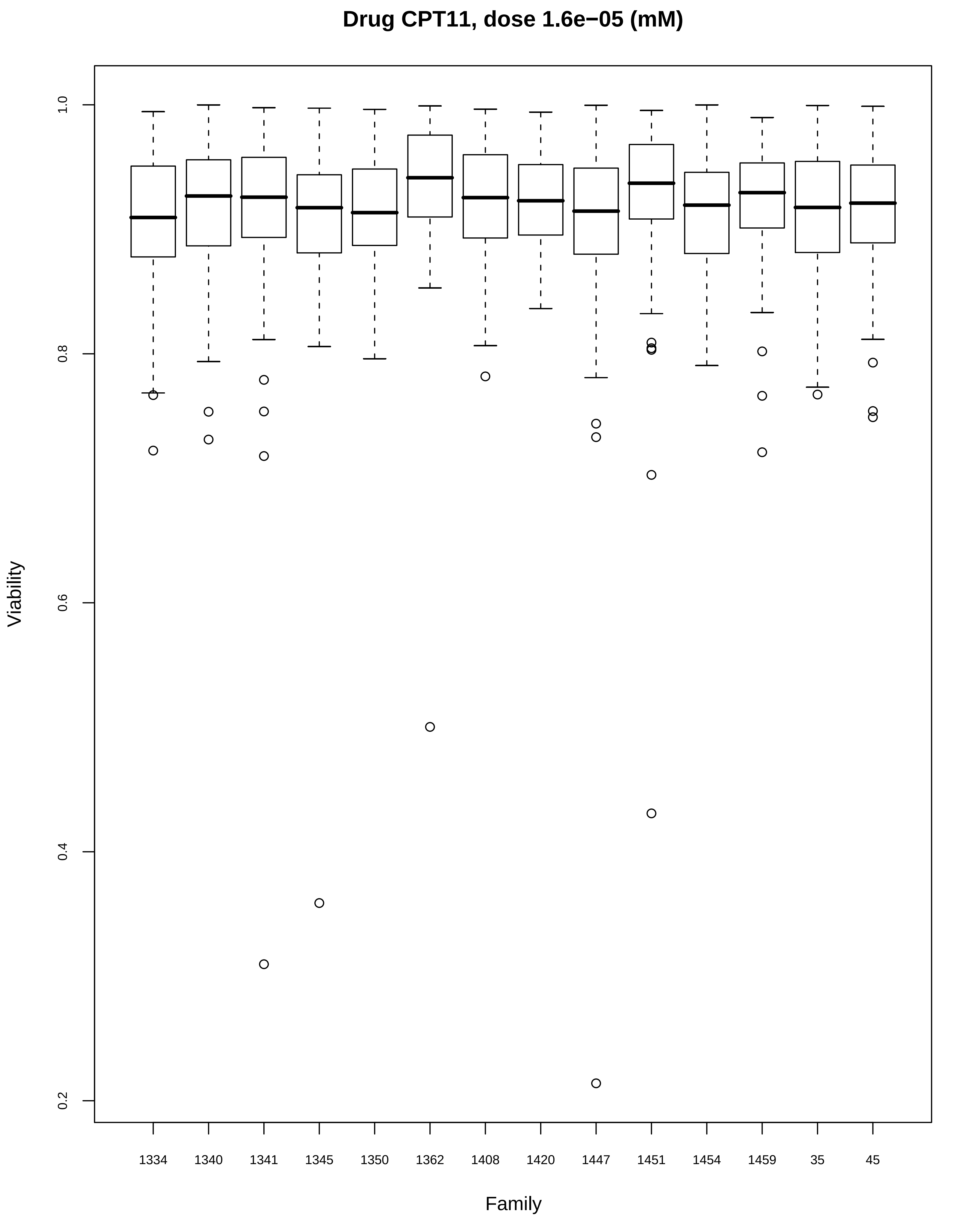

Drug CPT11, dose 1.6e−05 (mM)
Viability
1.0
0.8
0.6
0.4
0.2
1334
1340
1341
1345
1350
1362
1408
1420
1447
1451
1454
1459
35
45
Family

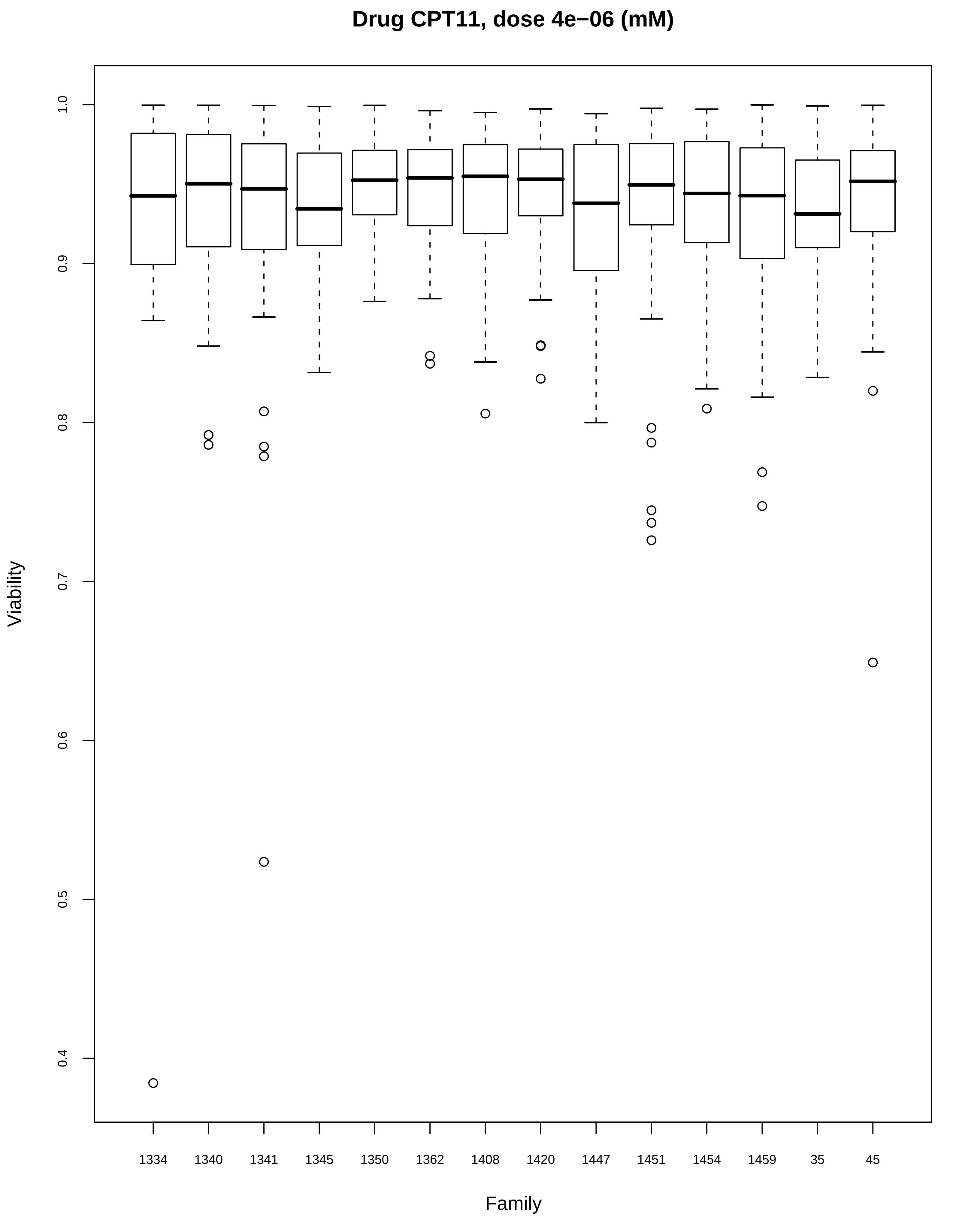
Drug CPT11, dose 4e−06 (mM)
Viability
1.0
0.9
0.8
0.7
0.6
0.5
0.4
1334
1340
1341
1345
1350
1362
1408
1420
1447
1451
1454
1459
35
45
Family

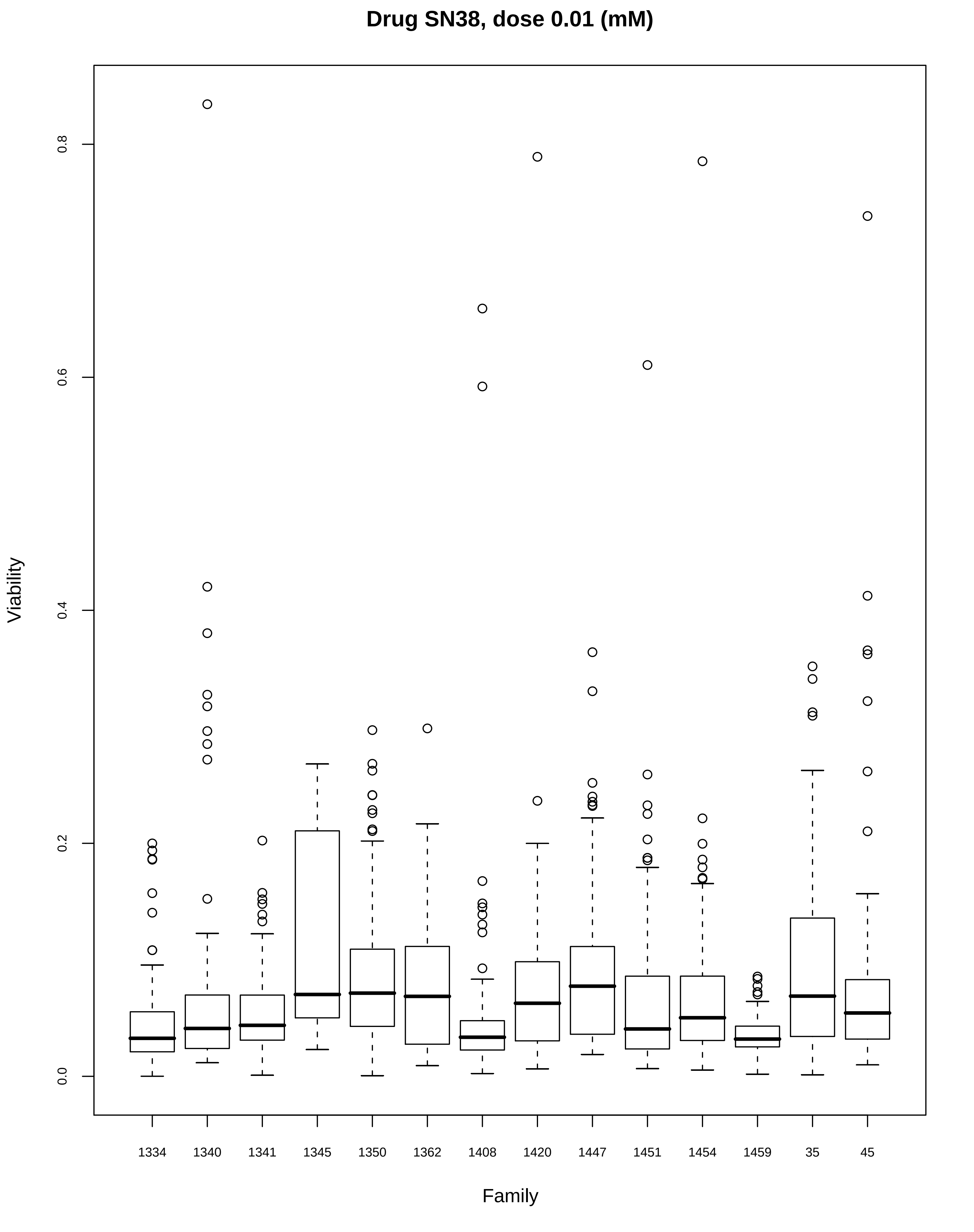

Drug SN38, dose 0.01 (mM)
Viability
0.0
0.2
0.4
0.6
0.8
1334
1340
1341
1345
1350
1362
1408
1420
1447
1451
1454
1459
35
45
Family

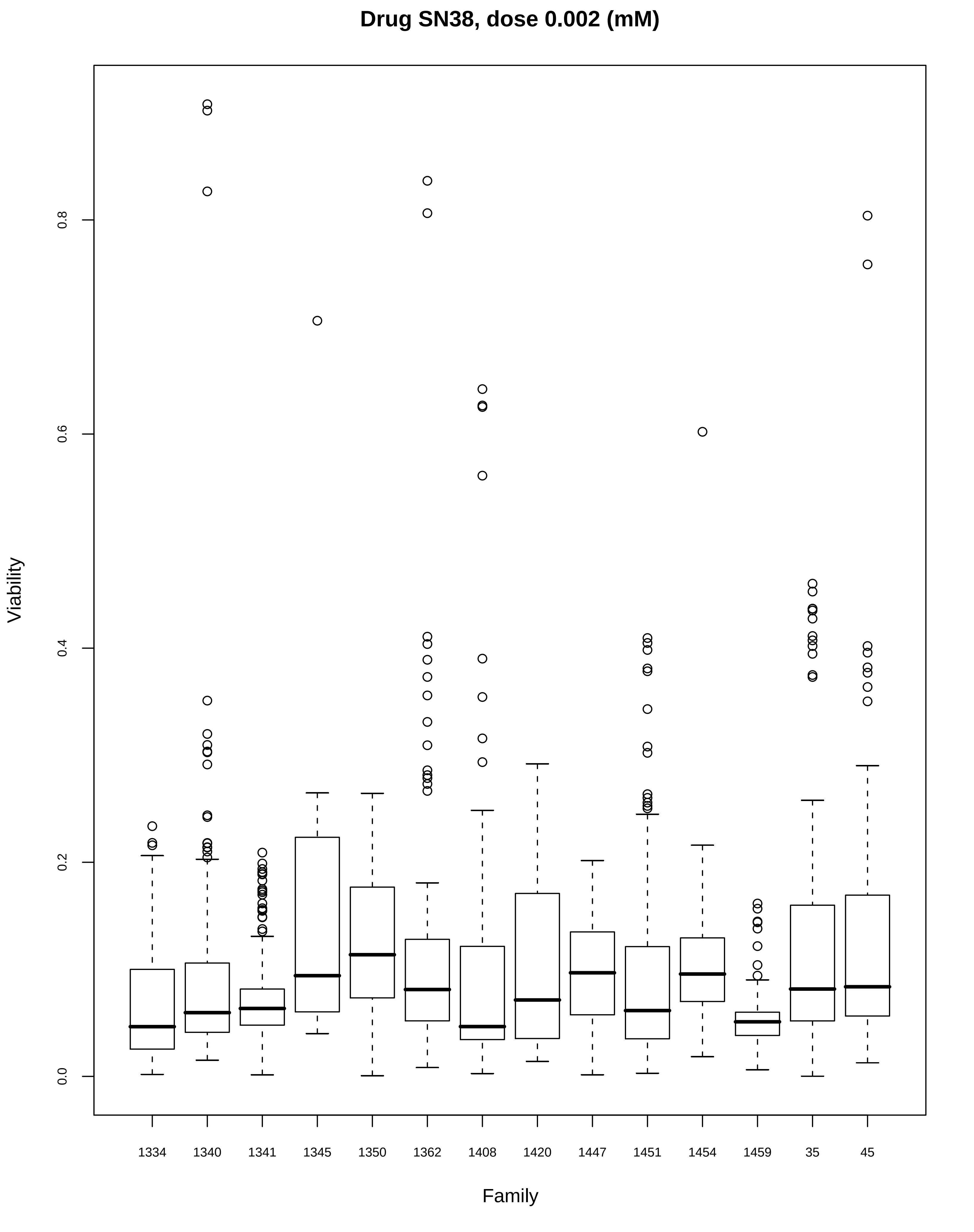

Drug SN38, dose 0.002 (mM)
Viability
0.0
0.2
0.4
0.6
0.8
Family
1334
1340
1341
1345
1350
1362
1408
1420
1447
1451
1454
1459
35
45

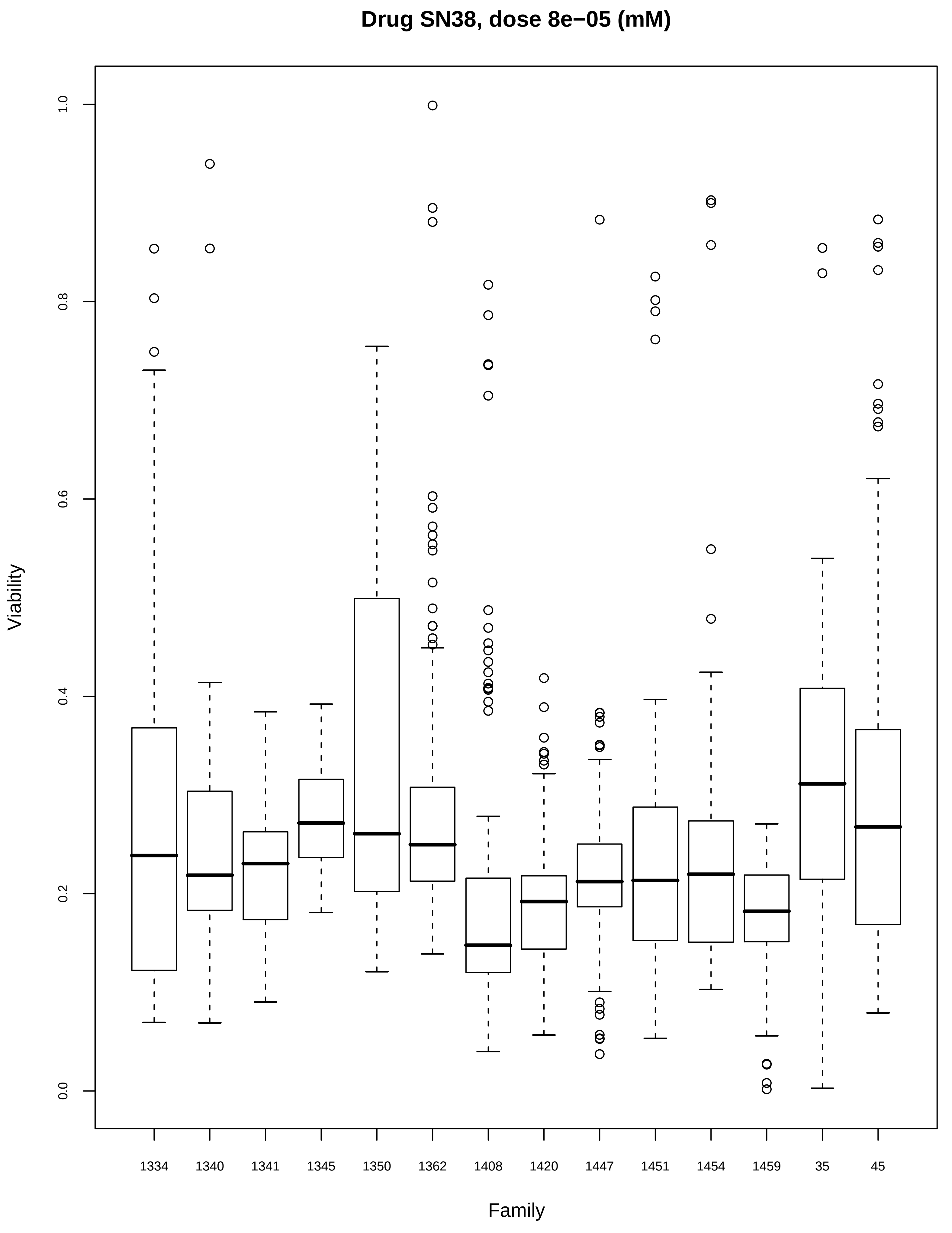
Drug SN38, dose 8e−05 (mM)
Viability
0.0
0.2
0.4
0.6
0.8
1.0
1334
1340
1341
1345
1350
1362
1408
1420
1447
1451
1454
1459
35
45
Family

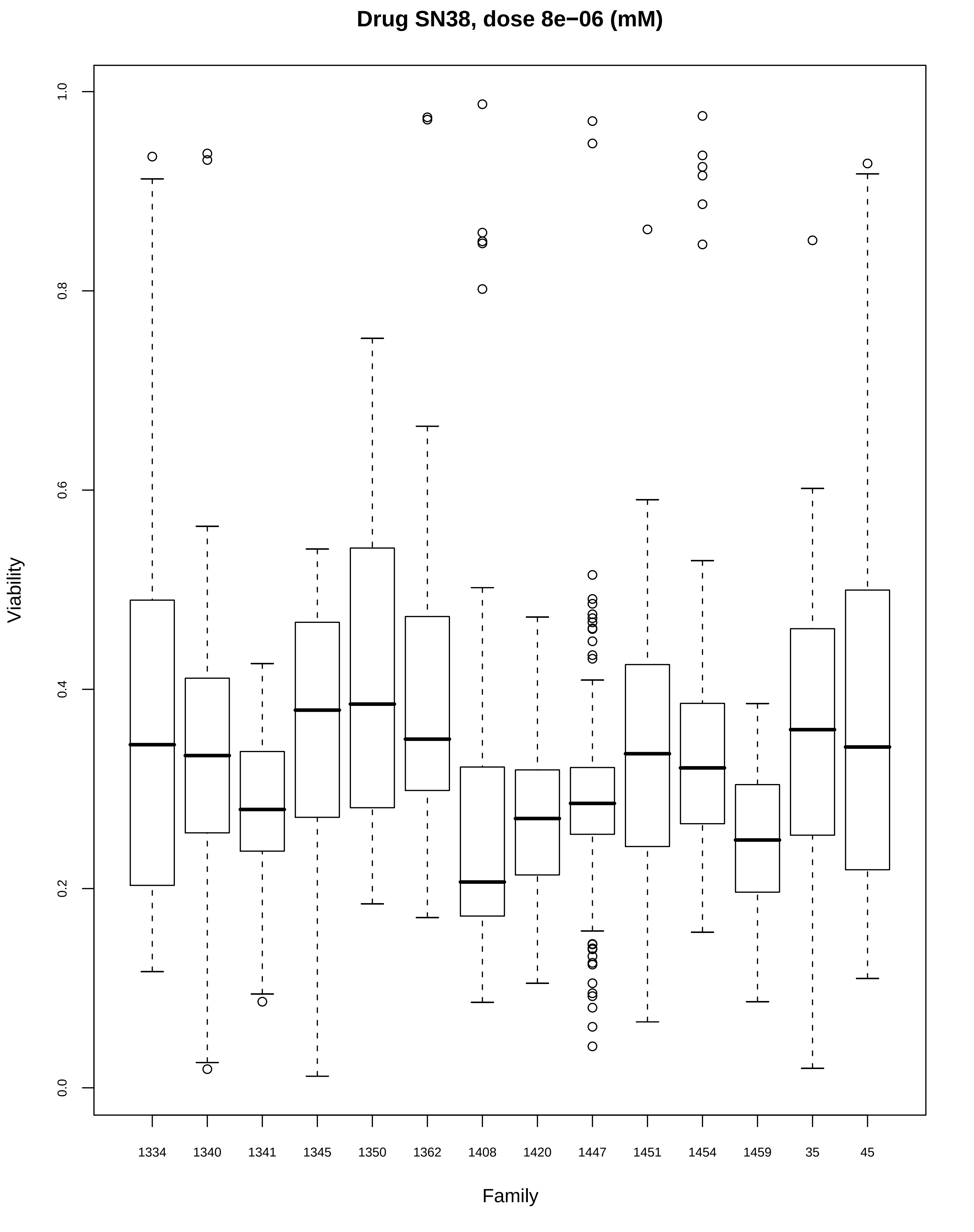
Drug SN38, dose 8e−06 (mM)
Viability
0.0
0.2
0.4
0.6
0.8
1.0
1334
1340
1341
1345
1350
1362
1408
1420
1447
1451
1454
1459
35
45
Family

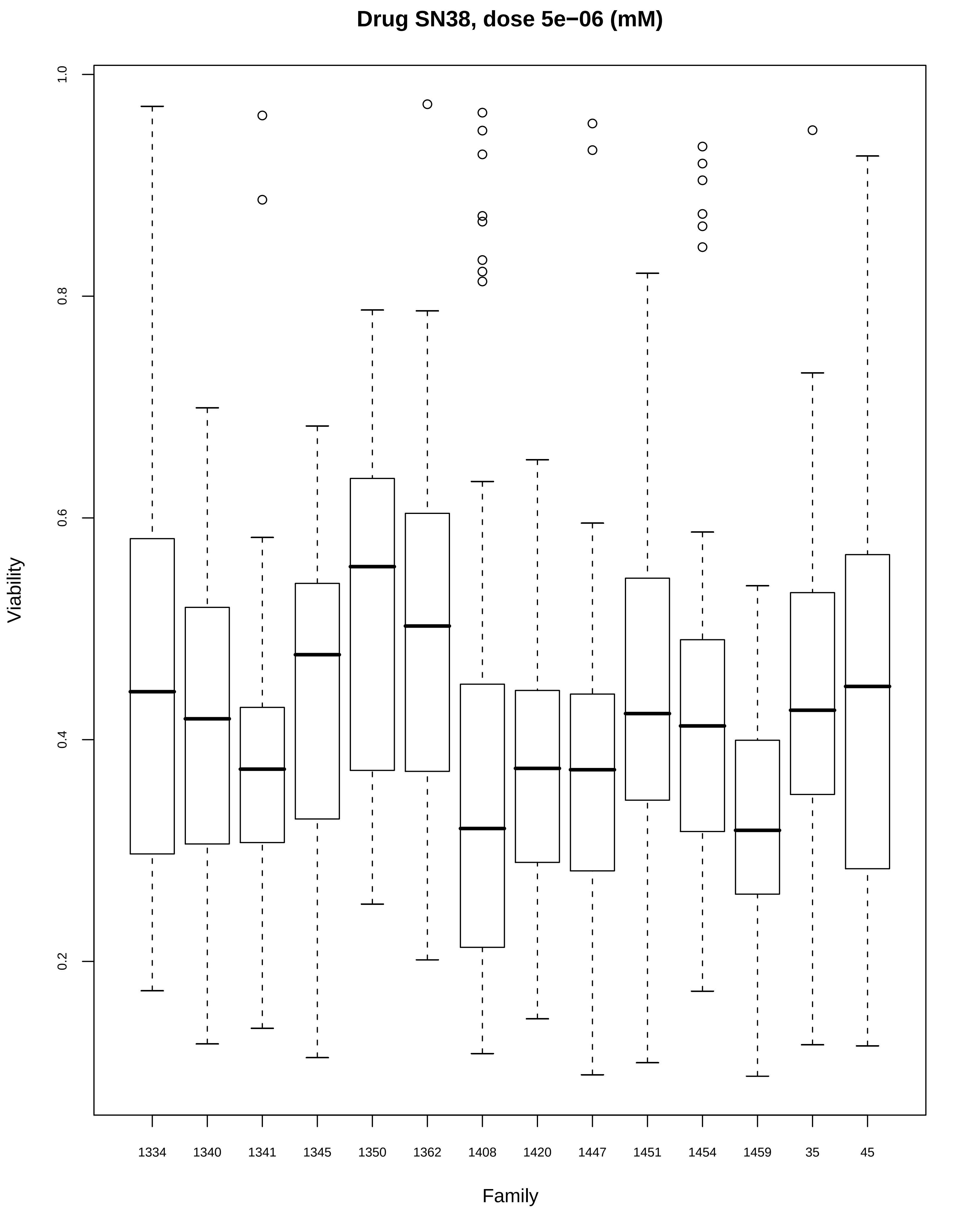
Drug SN38, dose 5e−06 (mM)
Viability
1.0
0.8
0.6
0.4
0.2
1334
1340
1341
1345
1350
1362
1408
1420
1447
1451
1454
1459
35
45
Family

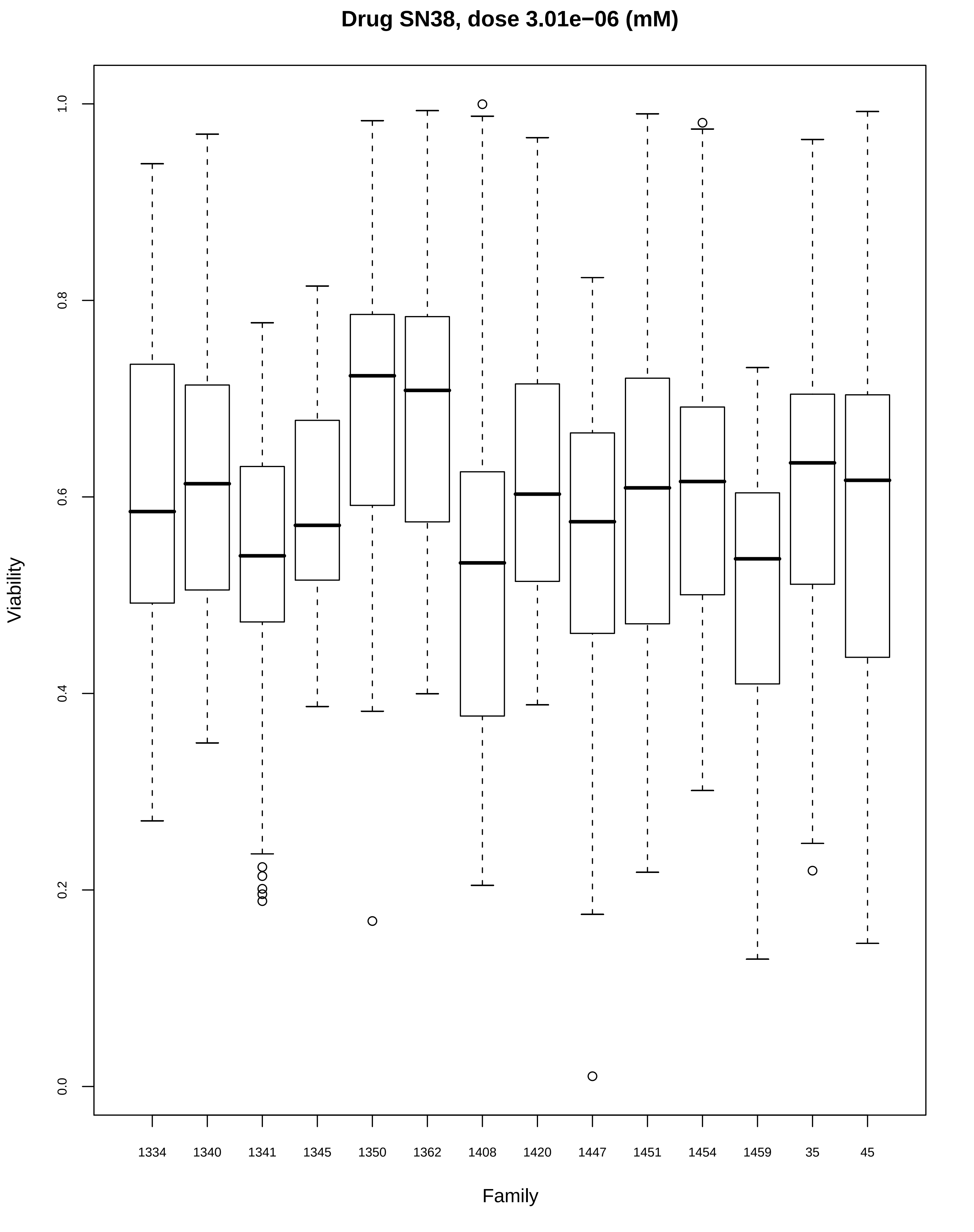

Drug SN38, dose 3.01e−06 (mM)
Viability
0.0
0.2
0.4
0.6
0.8
1.0
1334
1340
1341
1345
1350
1362
1408
1420
1447
1451
1454
1459
35
45
Family

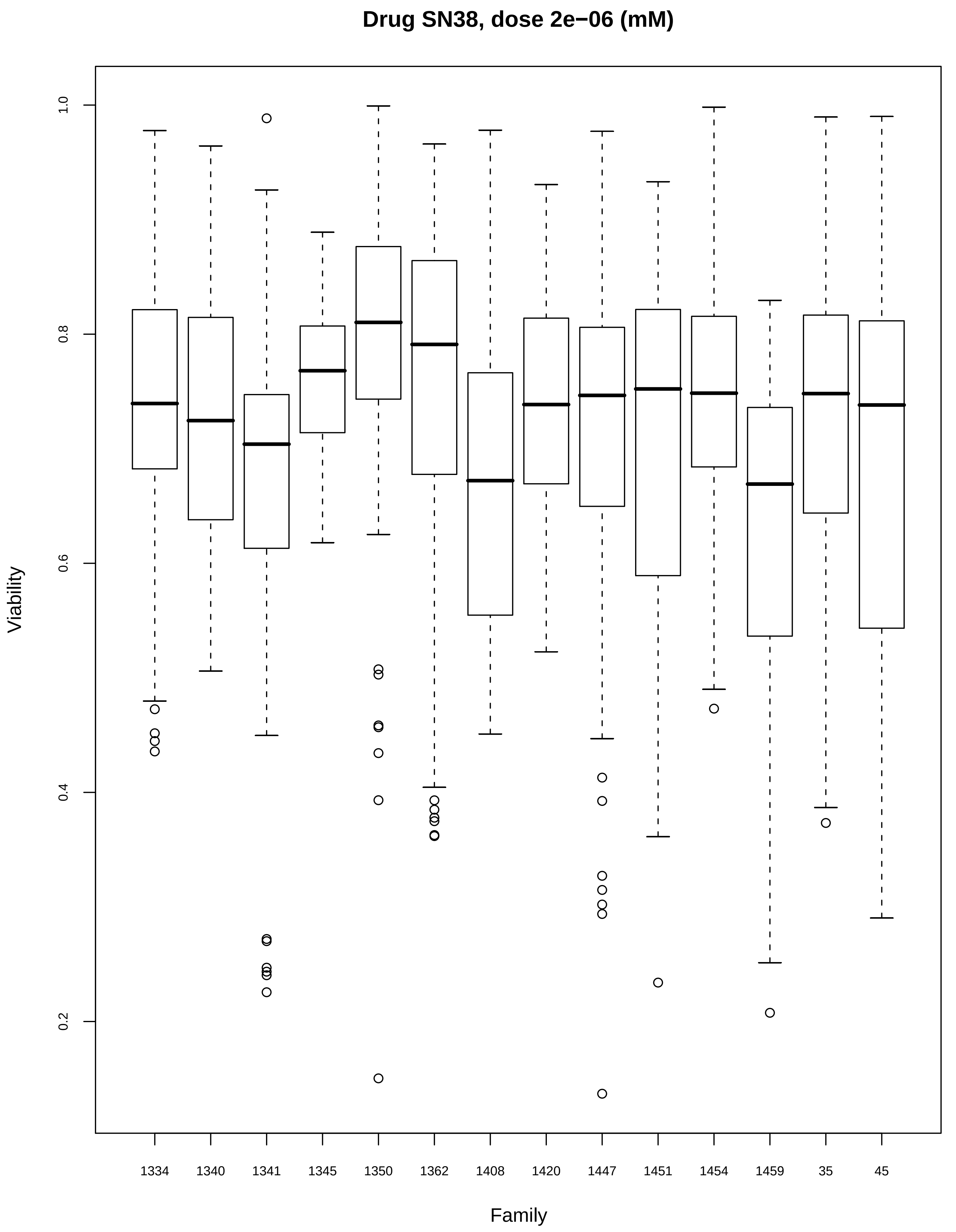
Drug SN38, dose 2e−06 (mM)
Viability
Family
1.0
0.8
0.6
0.4
0.2
1334
1340
1341
1345
1350
1362
1408
1420
1447
1451
1454
1459
35
45

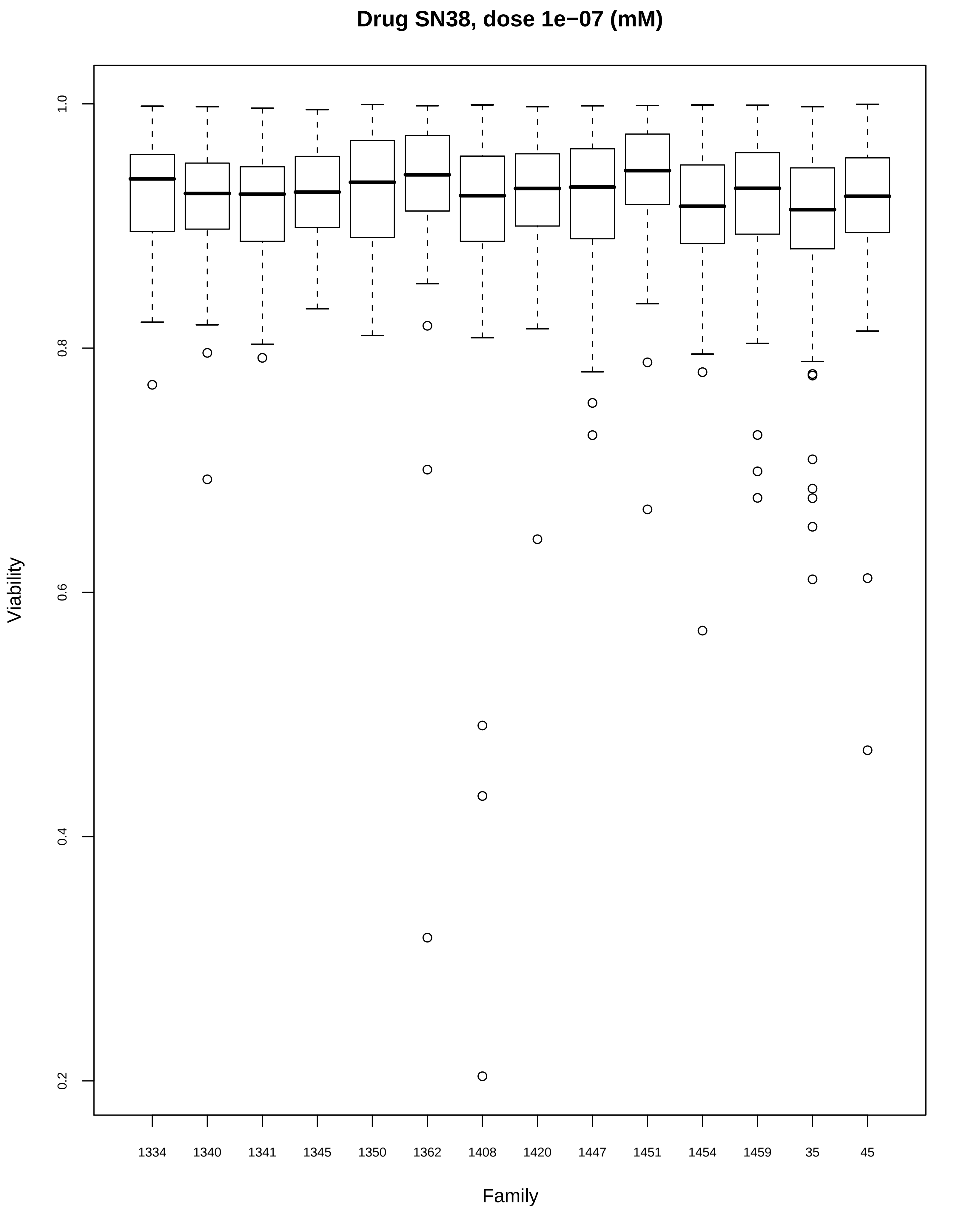
Drug SN38, dose 1e−07 (mM)
Viability
1.0
0.8
0.6
0.4
0.2
1334
1340
1341
1345
1350
1362
1408
1420
1447
1451
1454
1459
35
45
Family

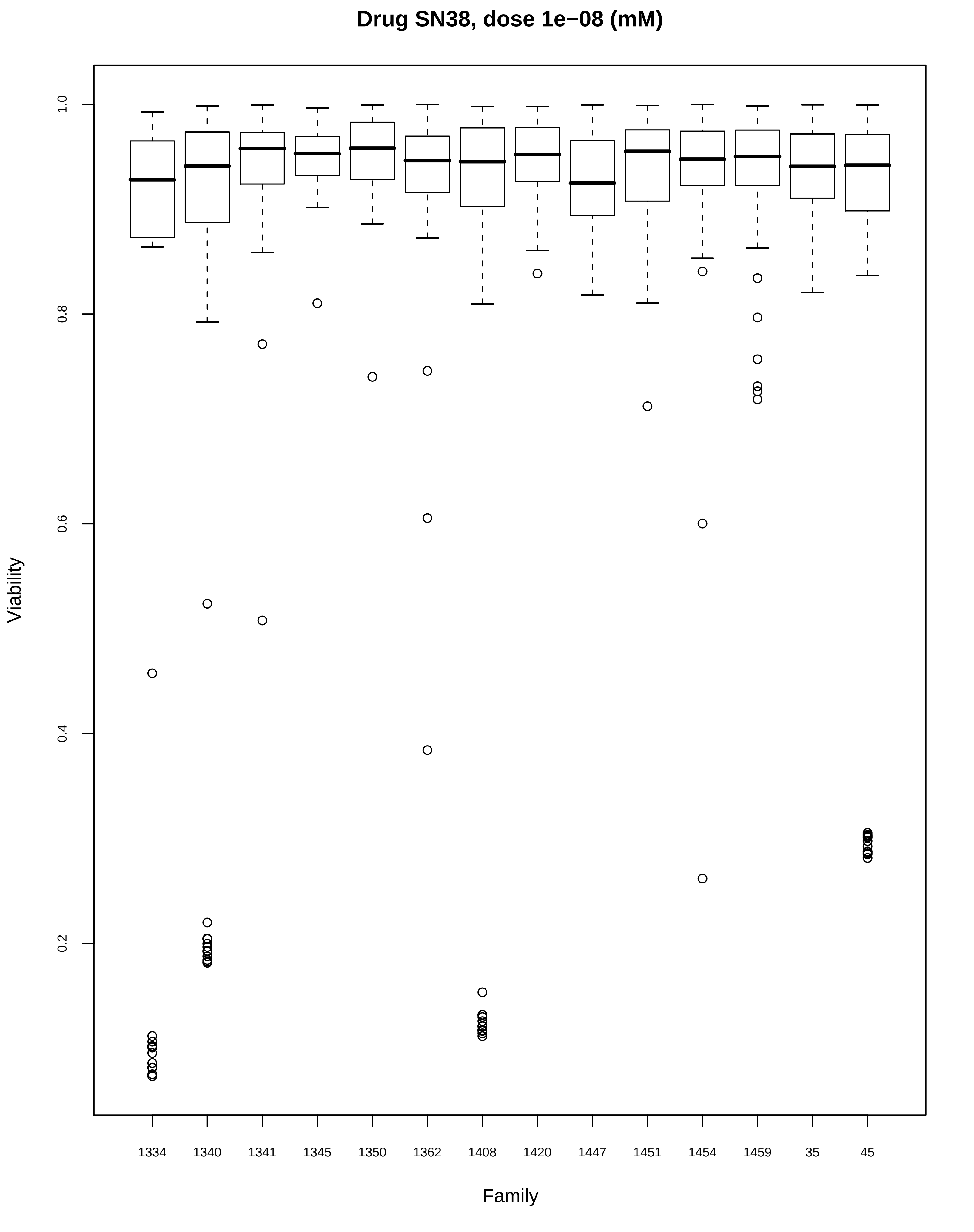
Drug SN38, dose 1e−08 (mM)
Viability
1.0
0.8
0.6
0.4
0.2
Family
1334
1340
1341
1345
1350
1362
1408
1420
1447
1451
1454
1459
35
45

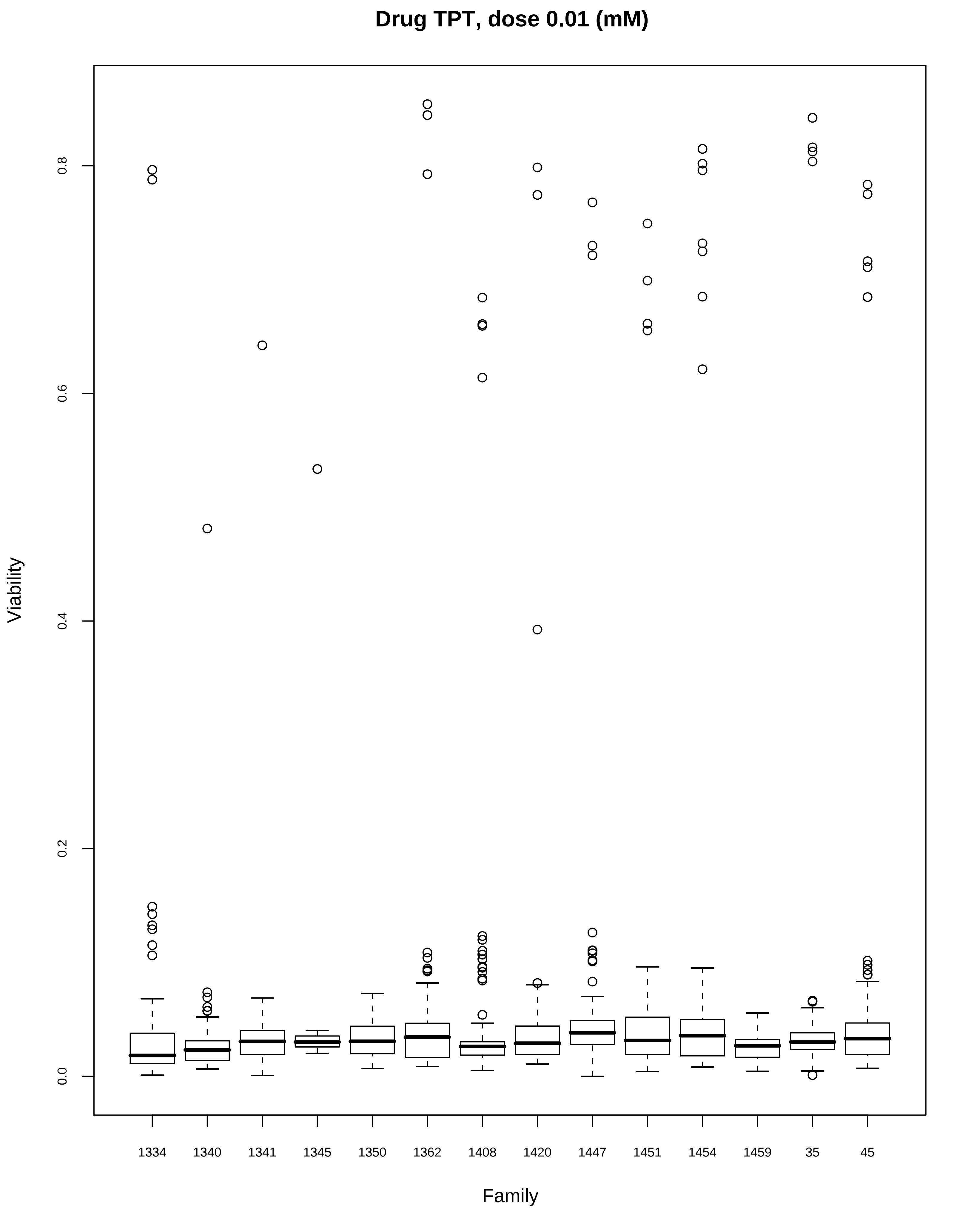
Drug TPT, dose 0.01 (mM)
Viability
Family
0.0
0.2
0.4
0.6
0.8
1334
1340
1341
1345
1350
1362
1408
1420
1447
1451
1454
1459
35
45

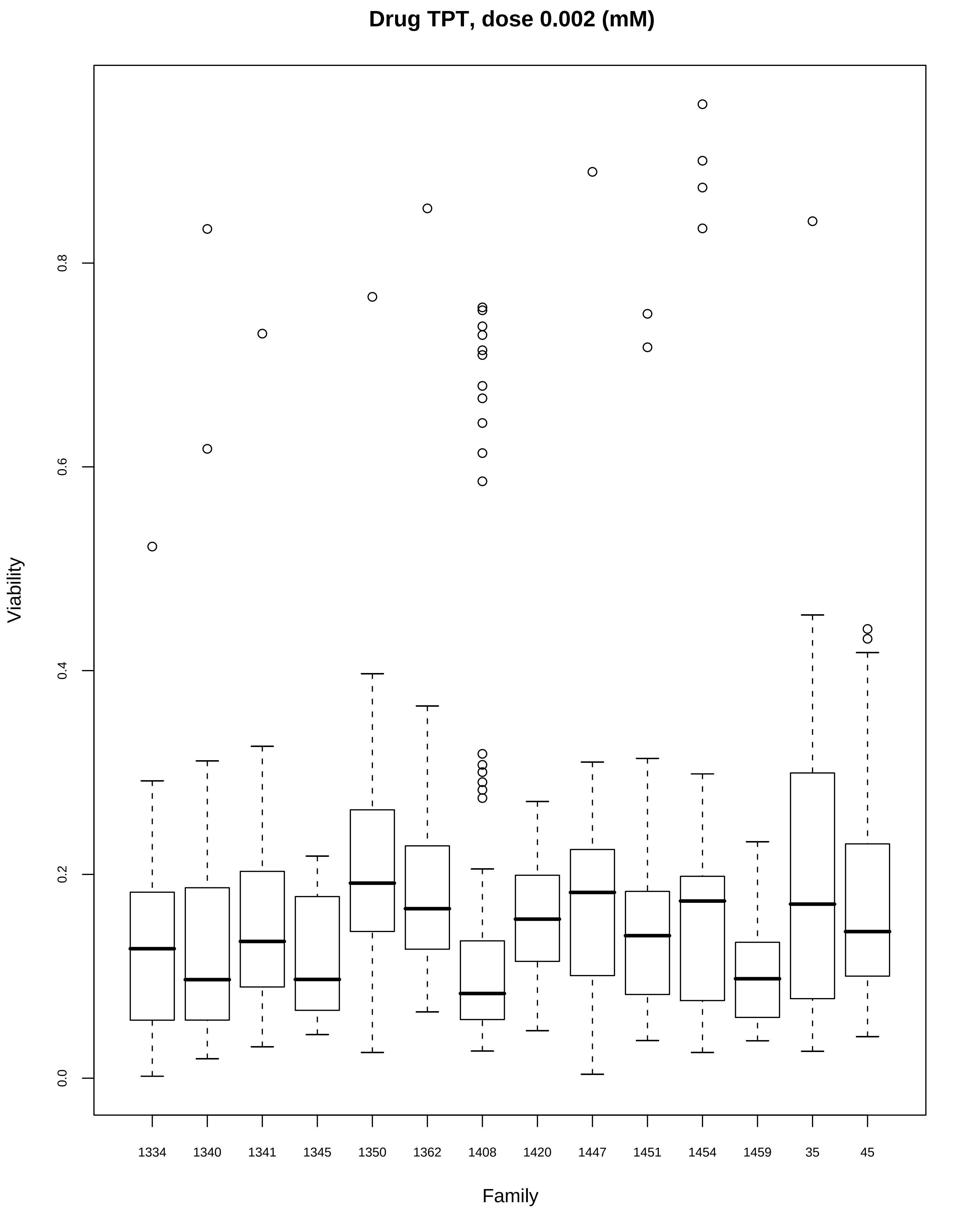
Drug TPT, dose 0.002 (mM)
Viability
0.0
0.2
0.4
0.6
0.8
Family
1334
1340
1341
1345
1350
1362
1408
1420
1447
1451
1454
1459
35
45

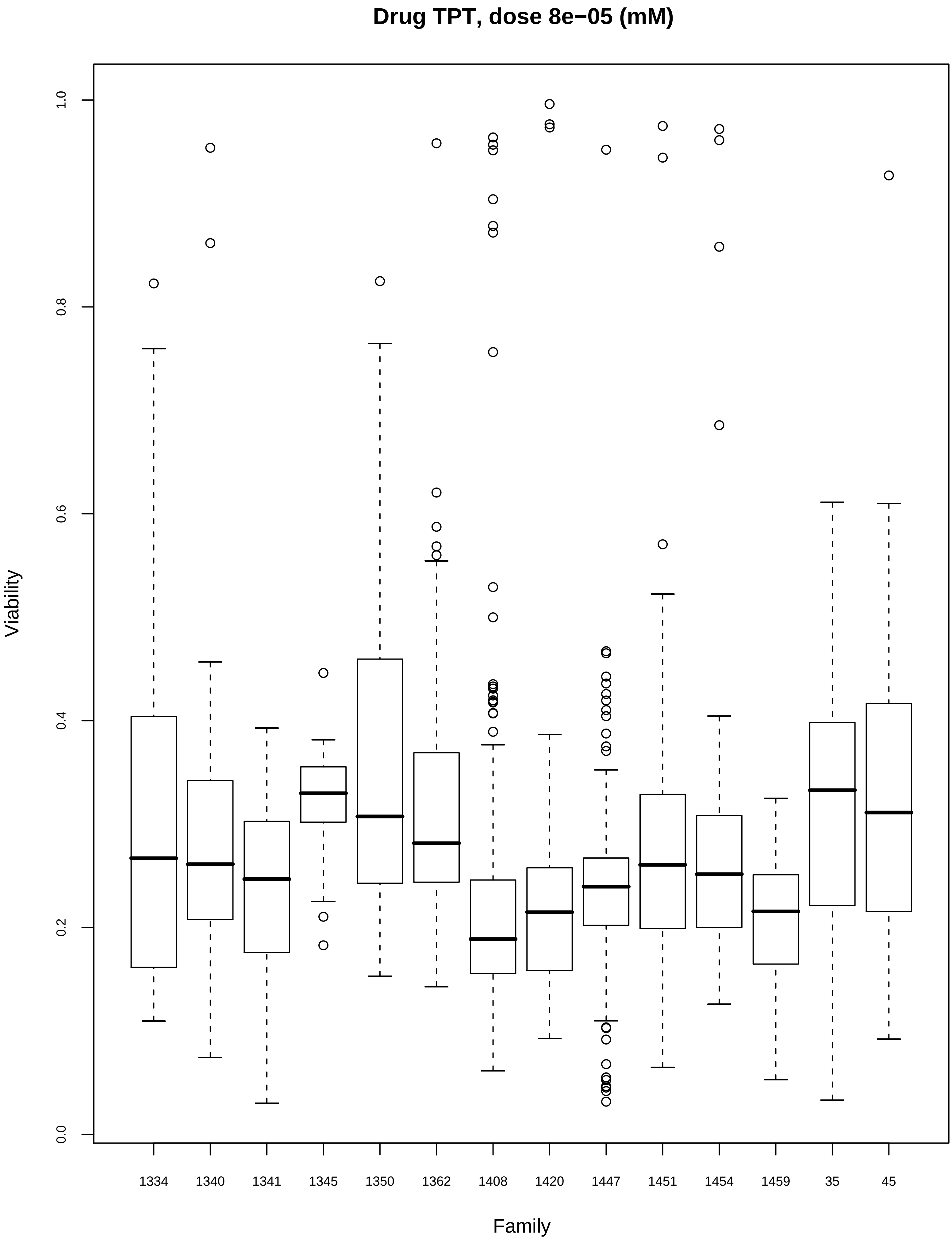
Drug TPT, dose 8e−05 (mM)
Viability
0.0
0.2
0.4
0.6
0.8
1.0
1334
1340
1341
1345
1350
1362
1408
1420
1447
1451
1454
1459
35
45
Family

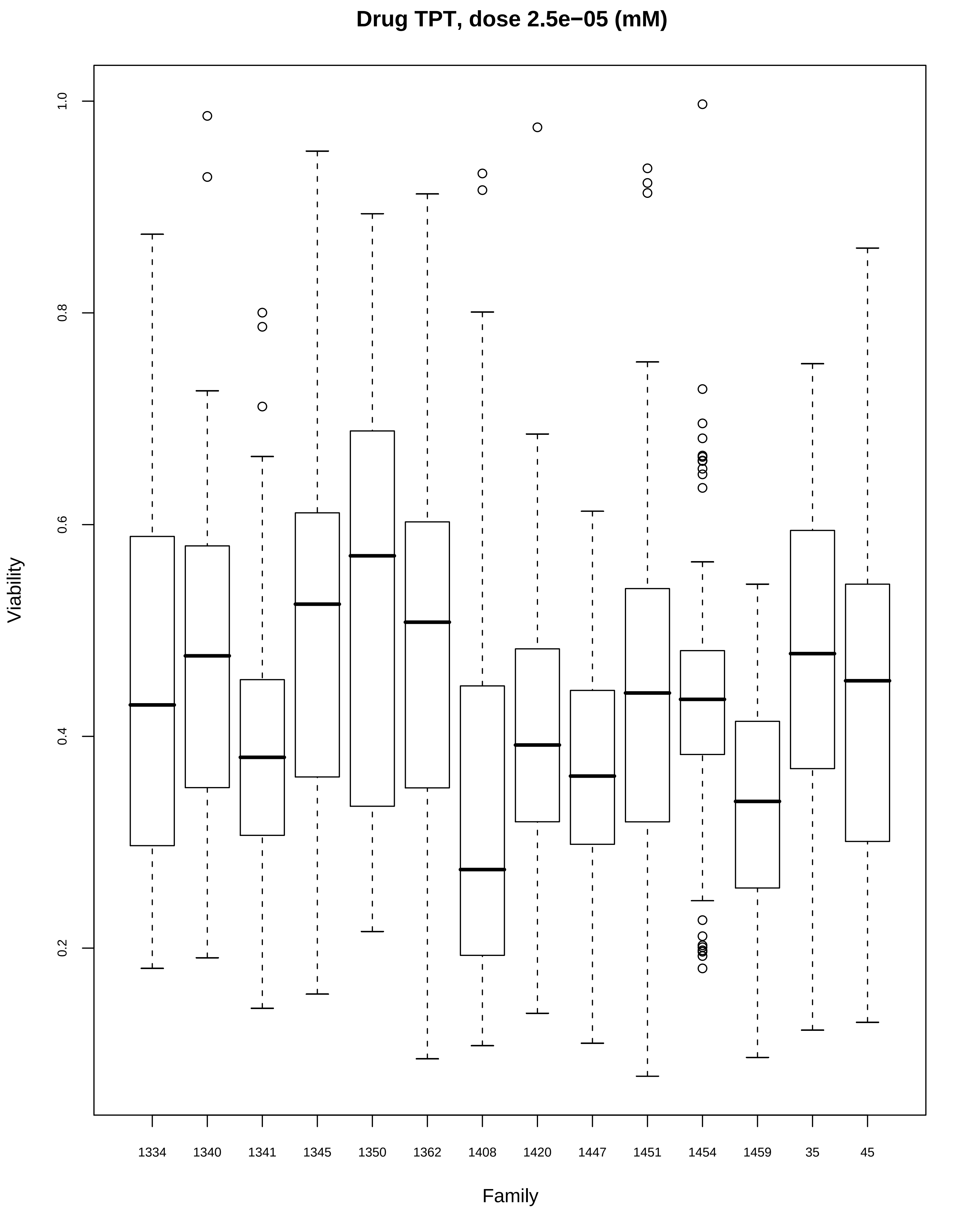

Drug TPT, dose 2.5e−05 (mM)
Viability
1.0
0.8
0.6
0.4
0.2
Family
1334
1340
1341
1345
1350
1362
1408
1420
1447
1451
1454
1459
35
45

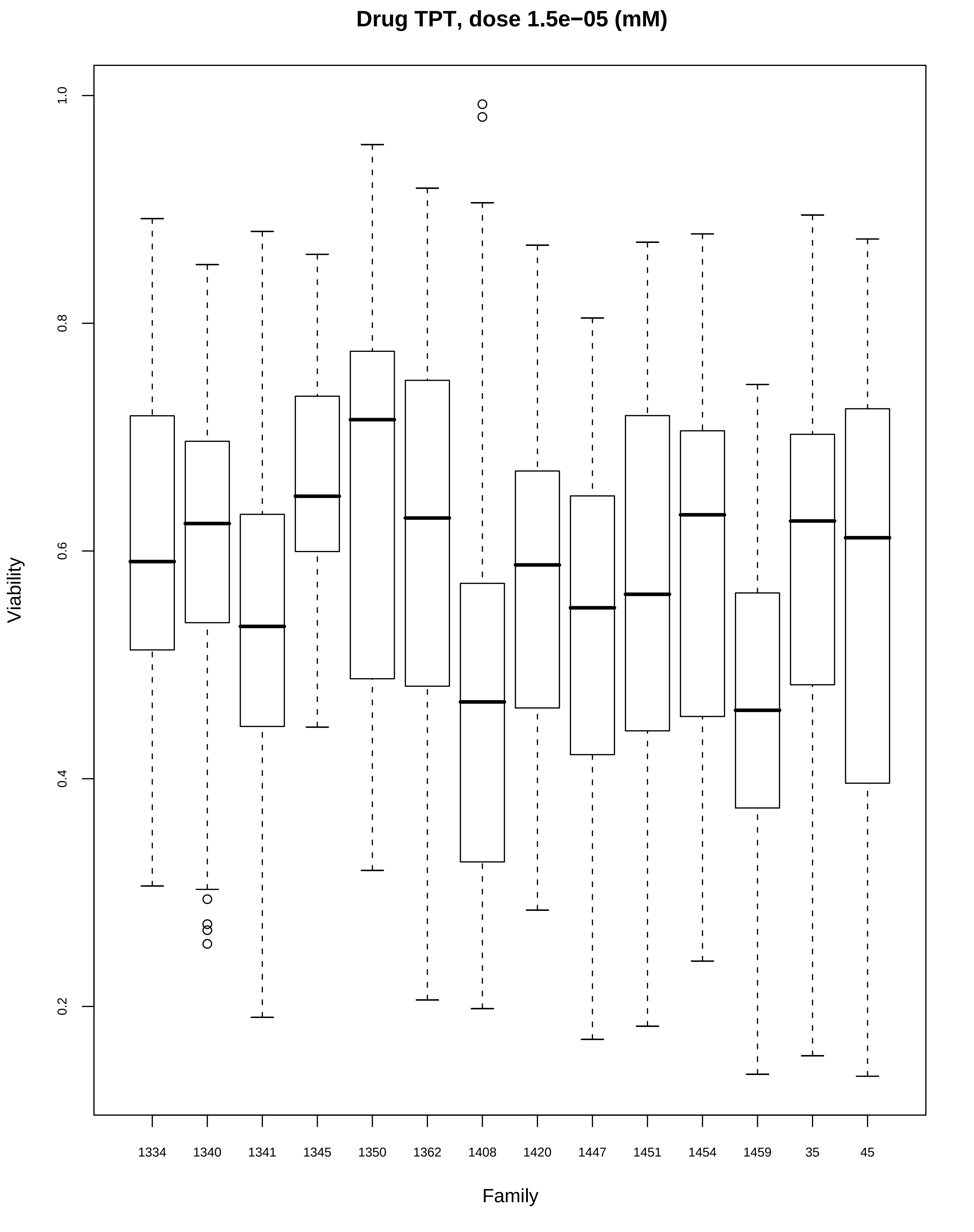
Drug TPT, dose 1.5e−05 (mM)
Viability
1.0
0.8
0.6
0.4
0.2
Family
1334
1340
1341
1345
1350
1362
1408
1420
1447
1451
1454
1459
35
45

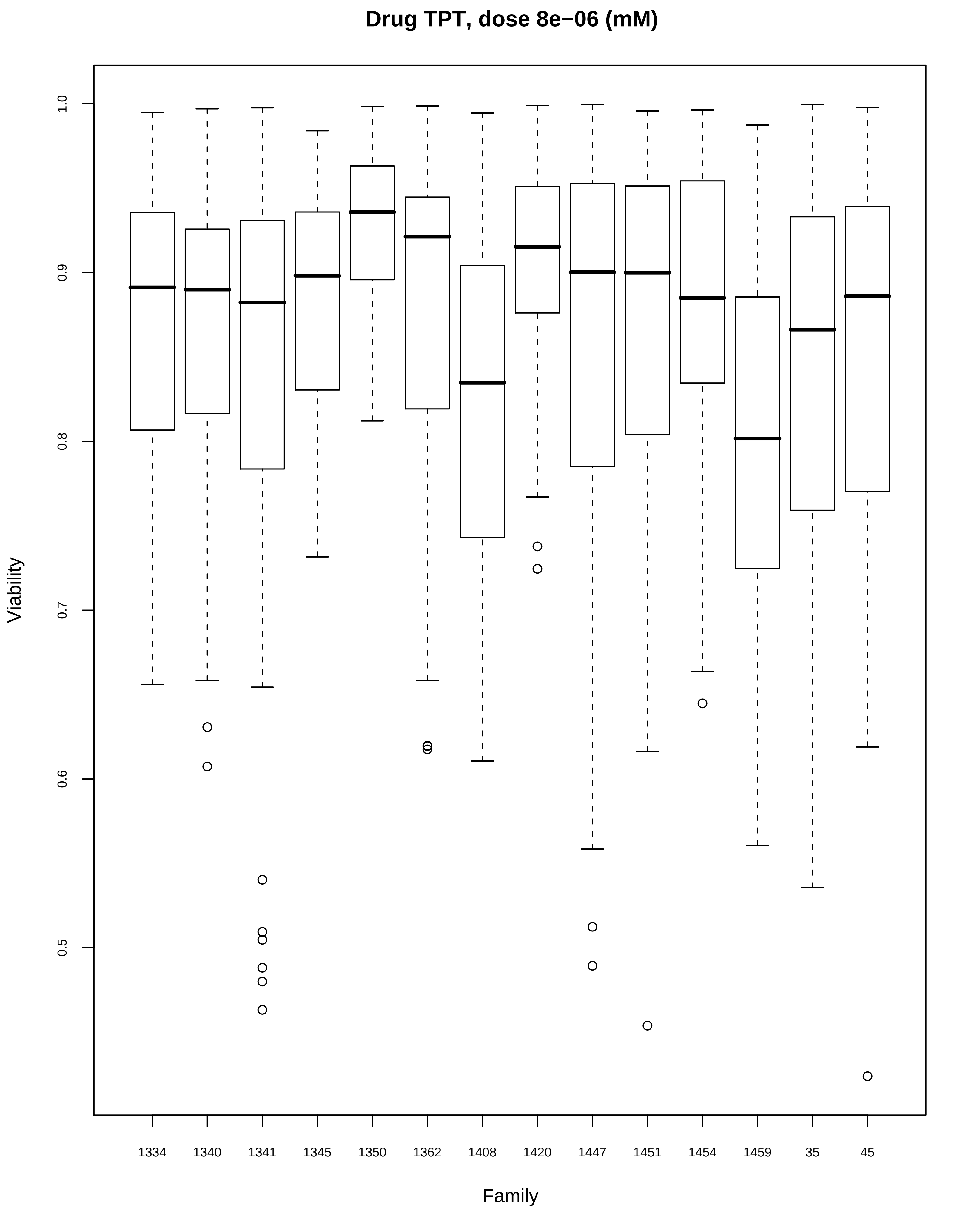

Drug TPT, dose 8e−06 (mM)
Viability
1.0
0.9
0.8
0.7
0.6
0.5
1334
1340
1341
1345
1350
1362
1408
1420
1447
1451
1454
1459
35
45
Family

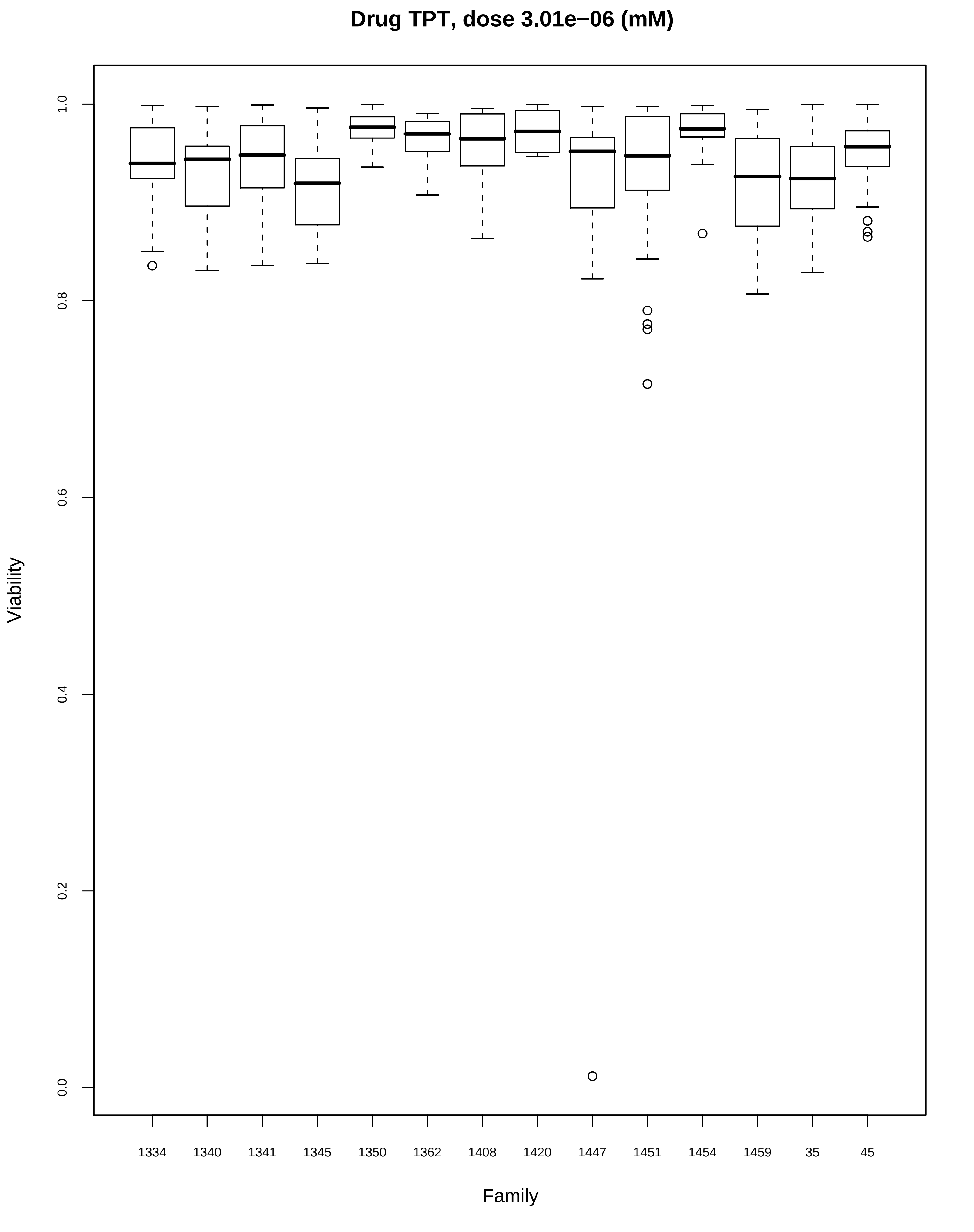

Drug TPT, dose 3.01e−06 (mM)
Viability
0.0
0.2
0.4
0.6
0.8
1.0
1334
1340
1341
1345
1350
1362
1408
1420
1447
1451
1454
1459
35
45
Family

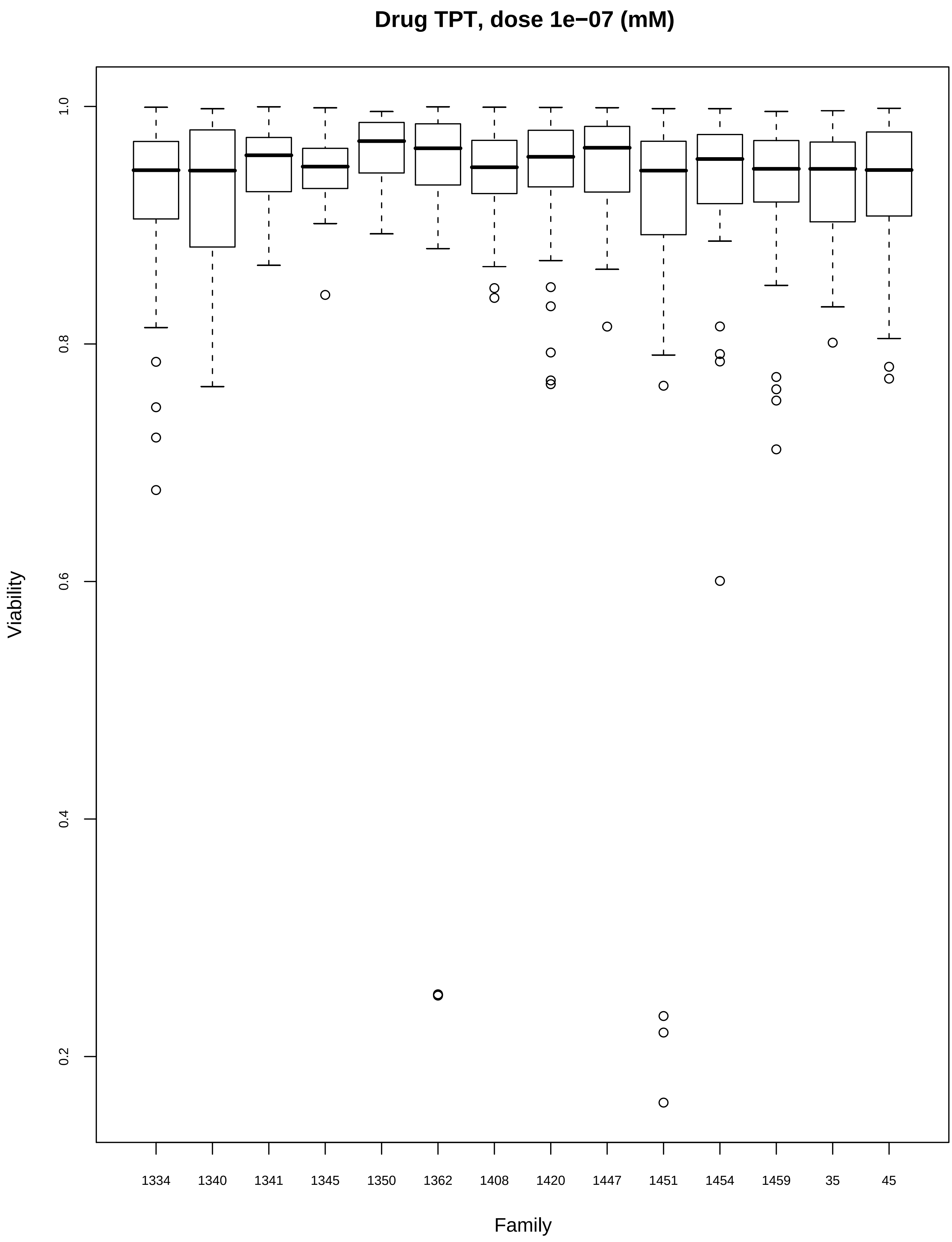

Drug TPT, dose 1e−07 (mM)
Viability
1.0
0.8
0.6
0.4
0.2
1334
1340
1341
1345
1350
1362
1408
1420
1447
1451
1454
1459
35
45
Family

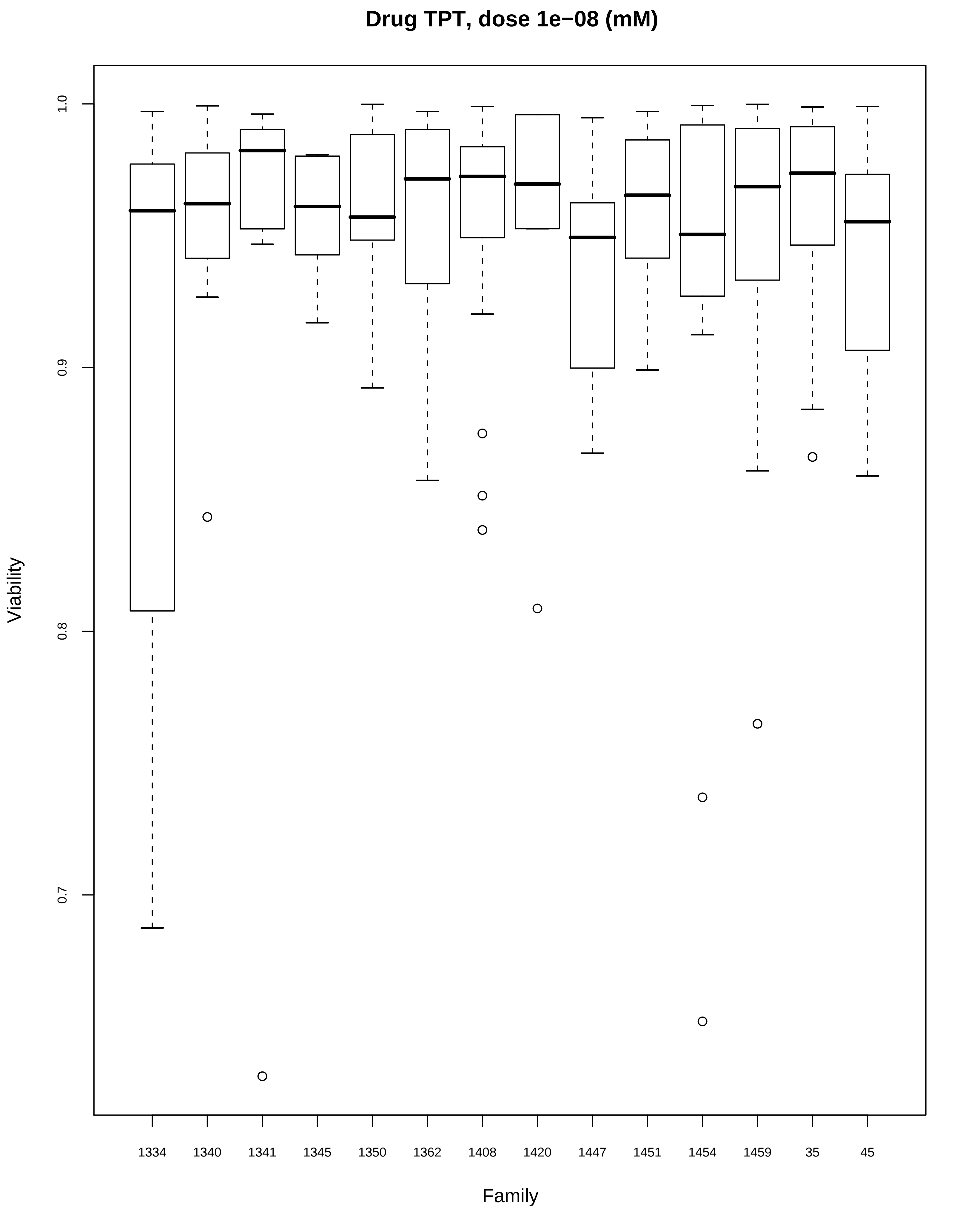
Drug TPT, dose 1e−08 (mM)
Viability
1.0
0.9
0.8
0.7
Family
1334
1340
1341
1345
1350
1362
1408
1420
1447
1451
1454
1459
35
45

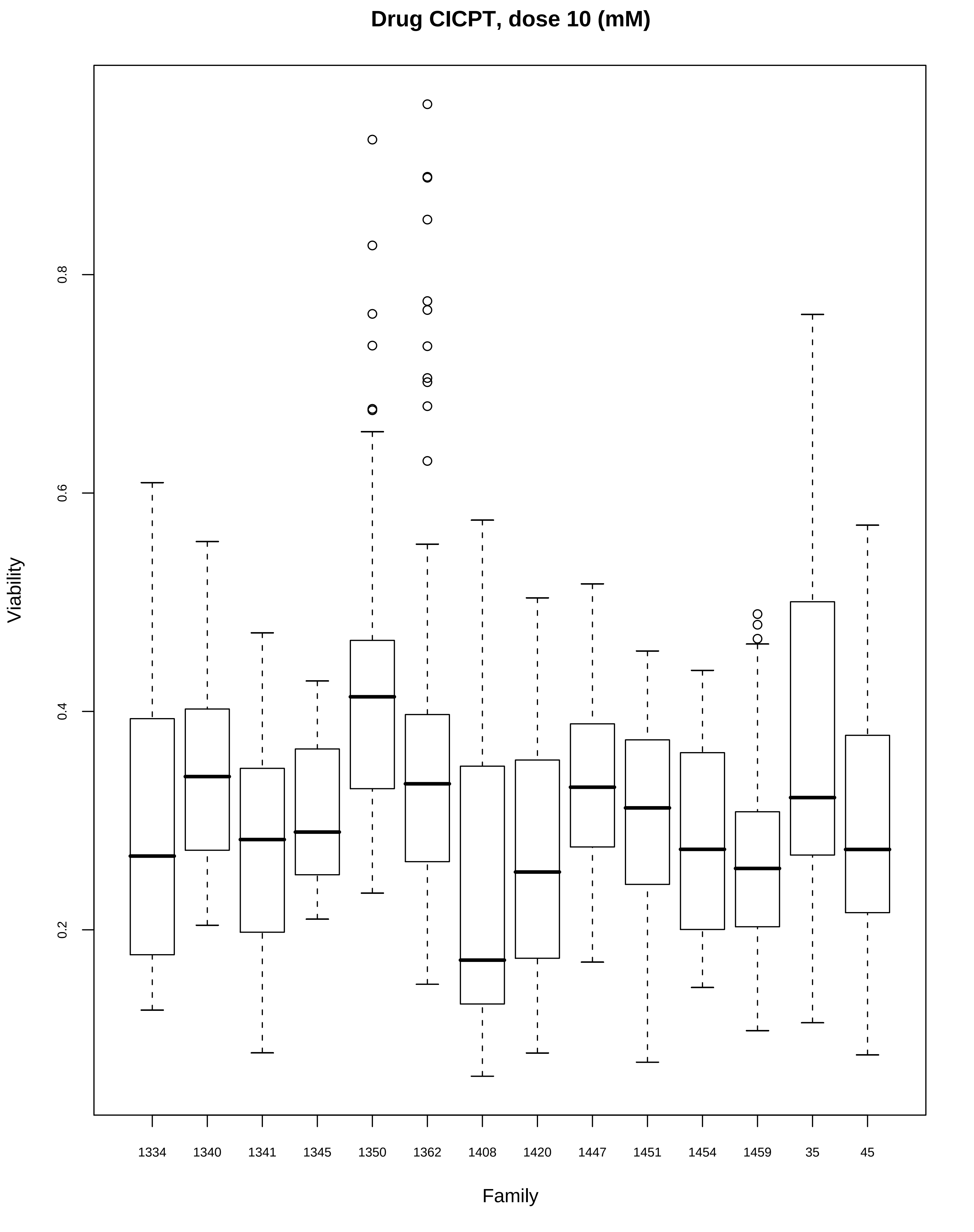

Drug ClCPT, dose 10 (mM)
Viability
0.2
0.4
0.6
0.8
Family
1334
1340
1341
1345
1350
1362
1408
1420
1447
1451
1454
1459
35
45

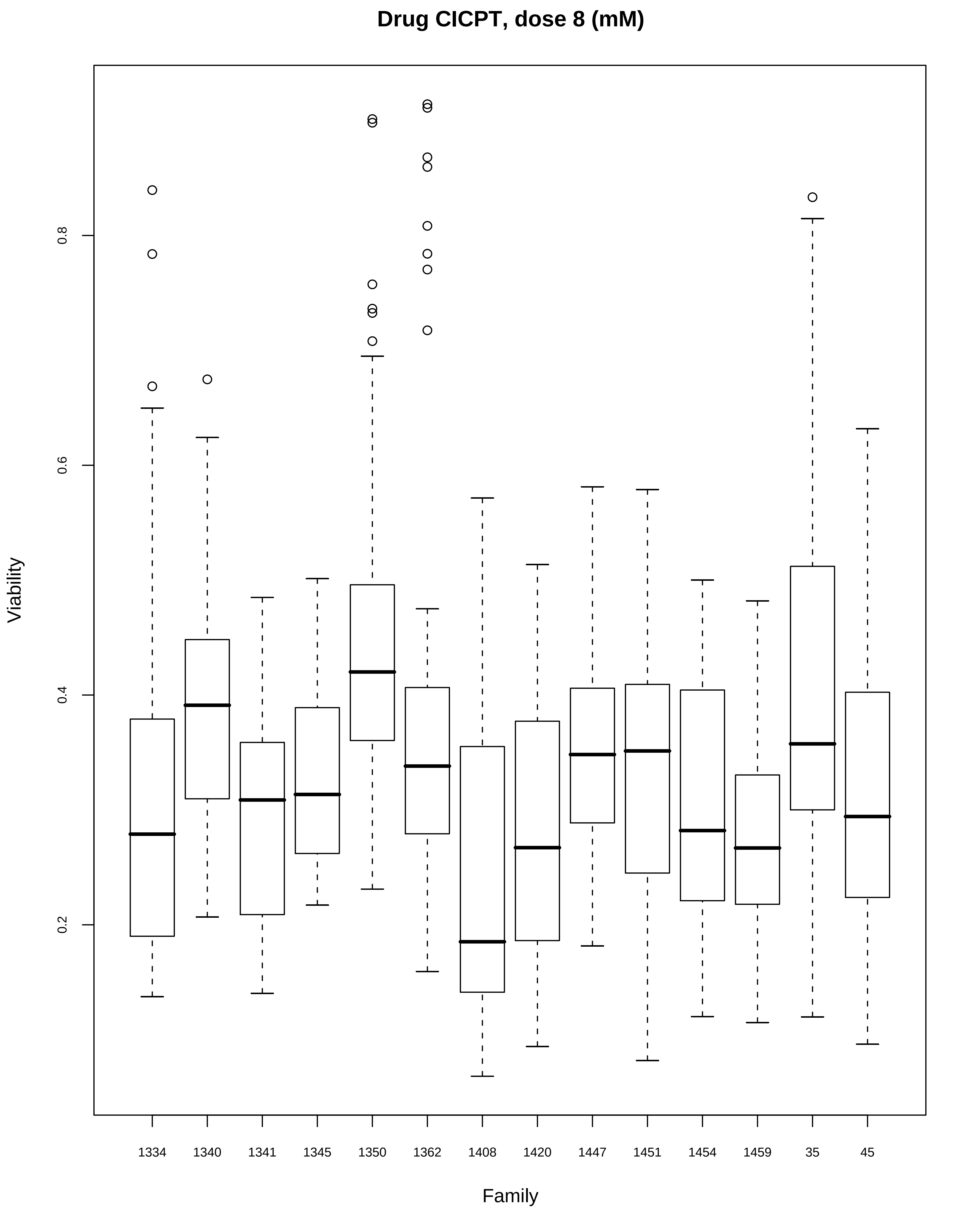
Drug CICPT, dose 8 (mM)
Viability
0.2
0.4
0.6
0.8
1334
1340
1341
1345
1350
1362
1408
1420
1447
1451
1454
1459
35
45
Family

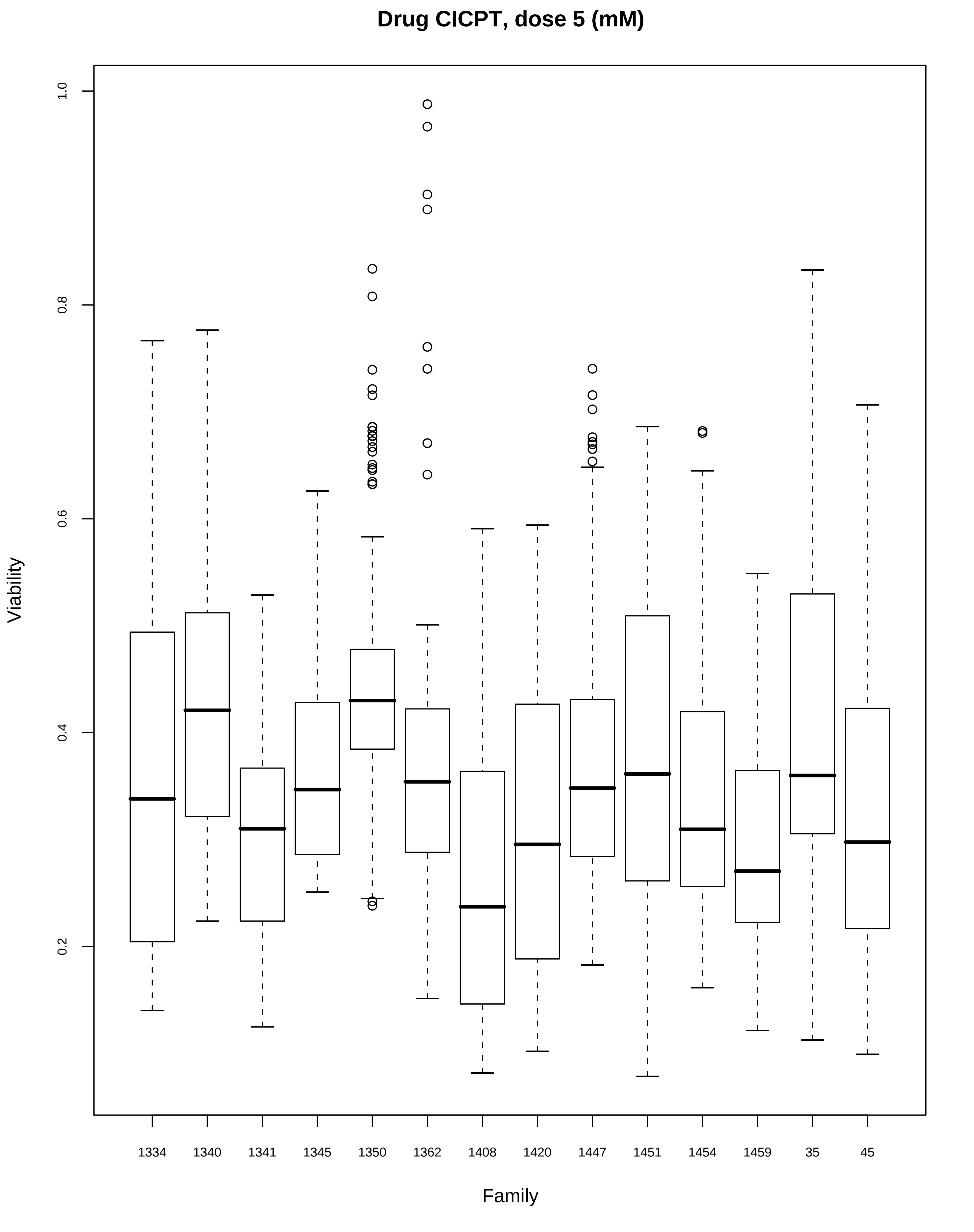
Drug CICPT, dose 5 (mM)
Viability
1.0
0.8
0.6
0.4
0.2
Family
1334
1340
1341
1345
1350
1362
1408
1420
1447
1451
1454
1459
35
45

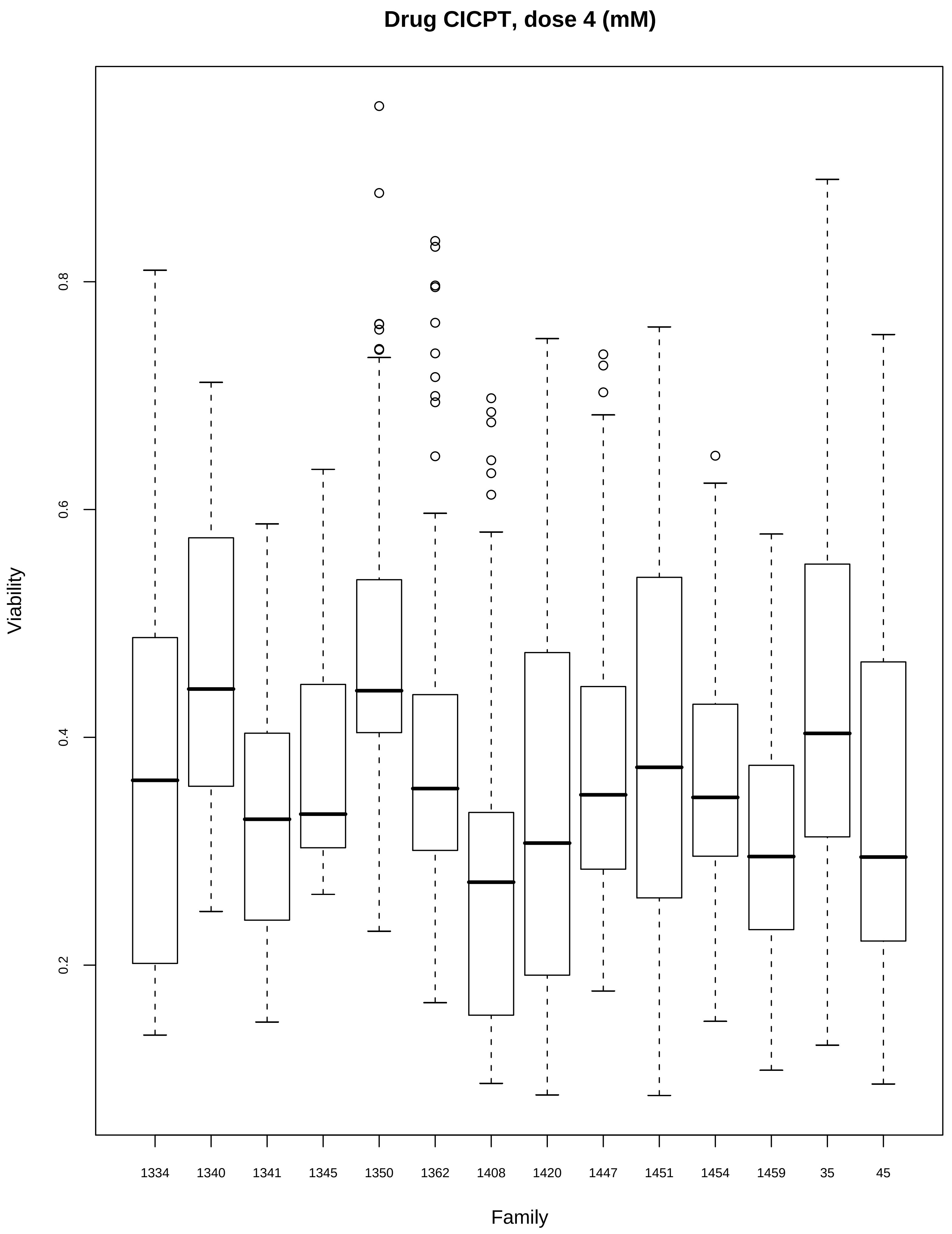
Drug CICPT, dose 4 (mM)
Viability
0.2
0.4
0.6
0.8
1334
1340
1341
1345
1350
1362
1408
1420
1447
1451
1454
1459
35
45
Family

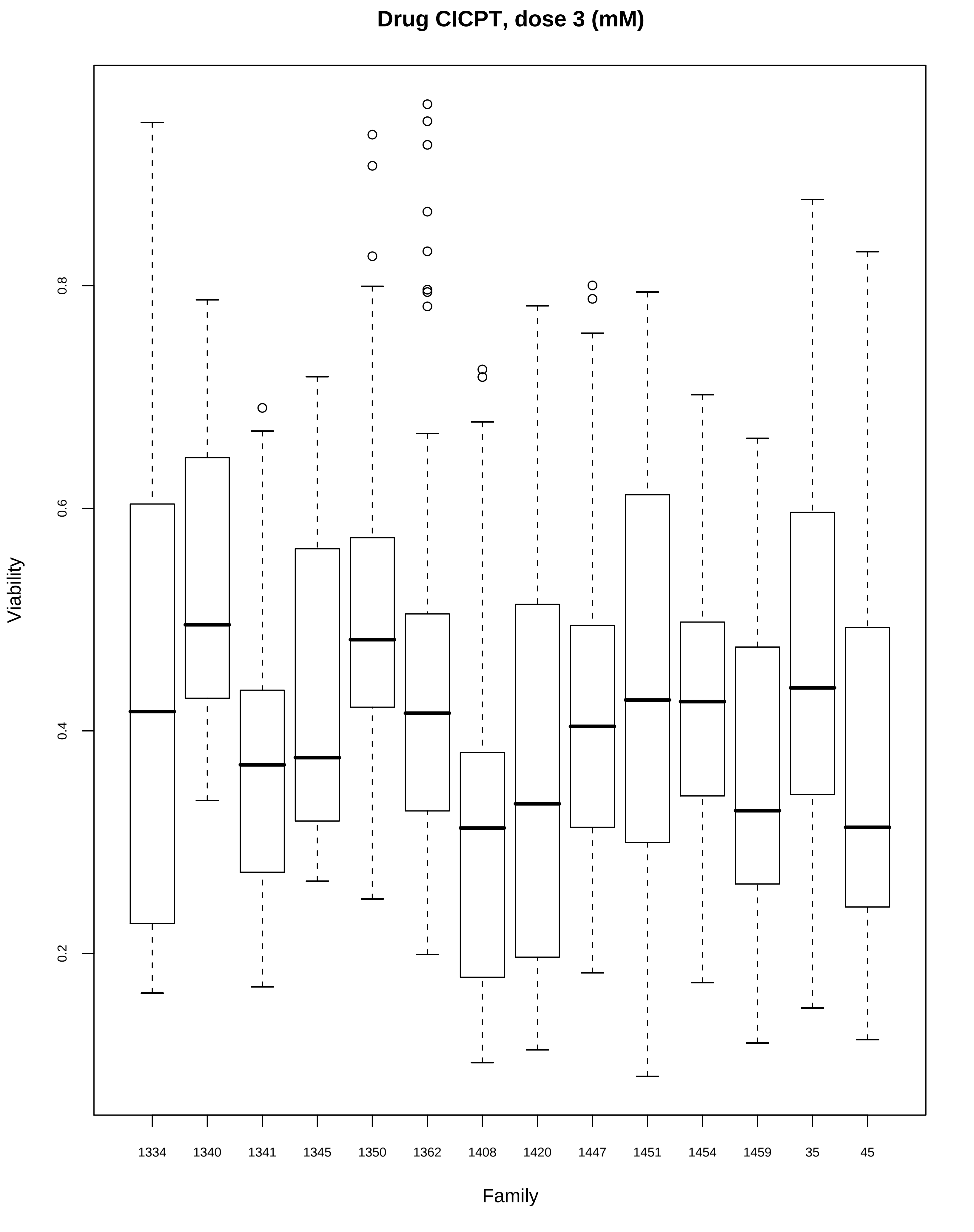
Drug ClCPT, dose 3 (mM)
Viability
0.2
0.4
0.6
0.8
1334
1340
1341
1345
1350
1362
1408
1420
1447
1451
1454
1459
35
45
Family

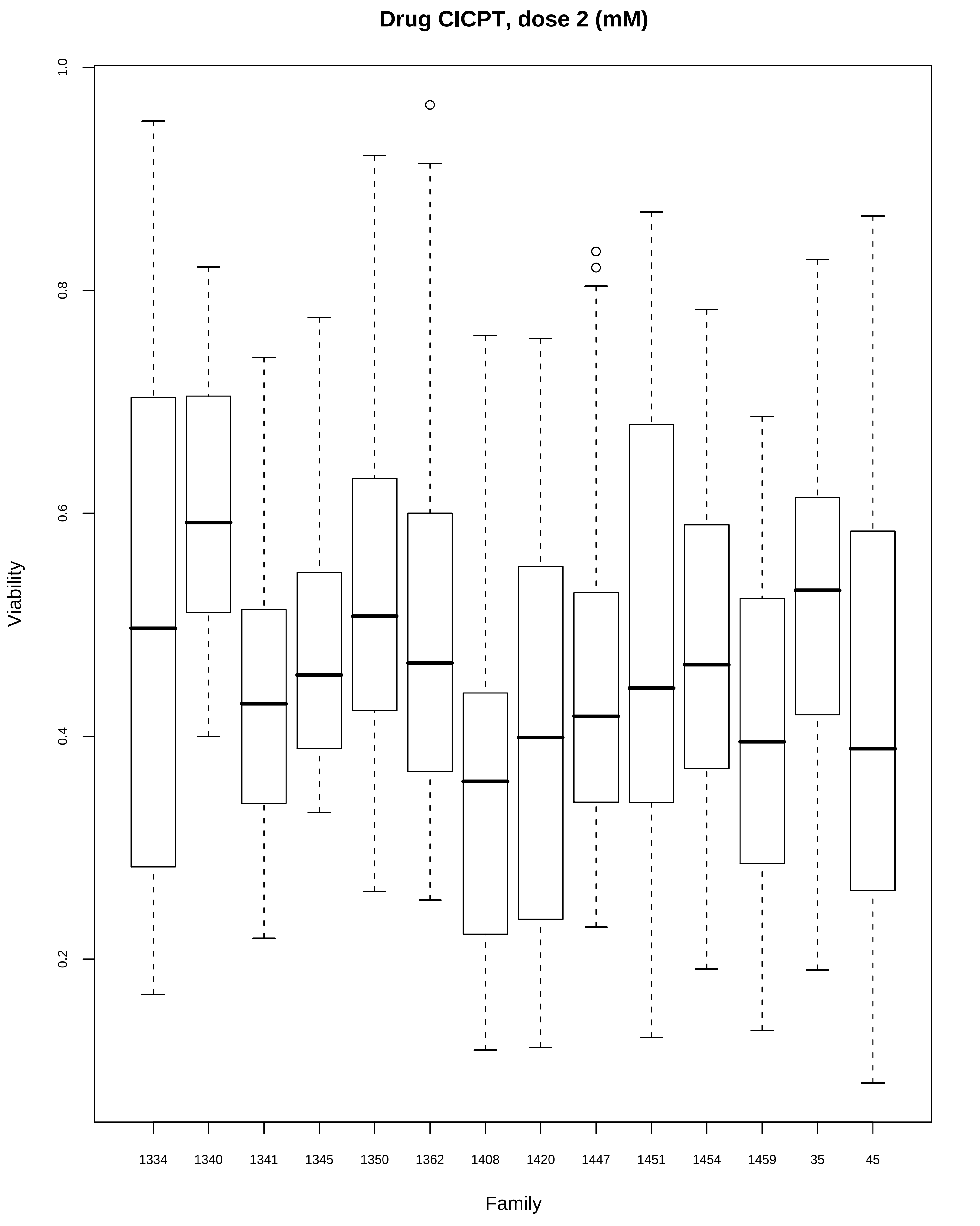

Drug CICPT, dose 2 (mM)
Viability
1.0
0.8
0.6
0.4
0.2
1334
1340
1341
1345
1350
1362
1408
1420
1447
1451
1454
1459
35
45
Family

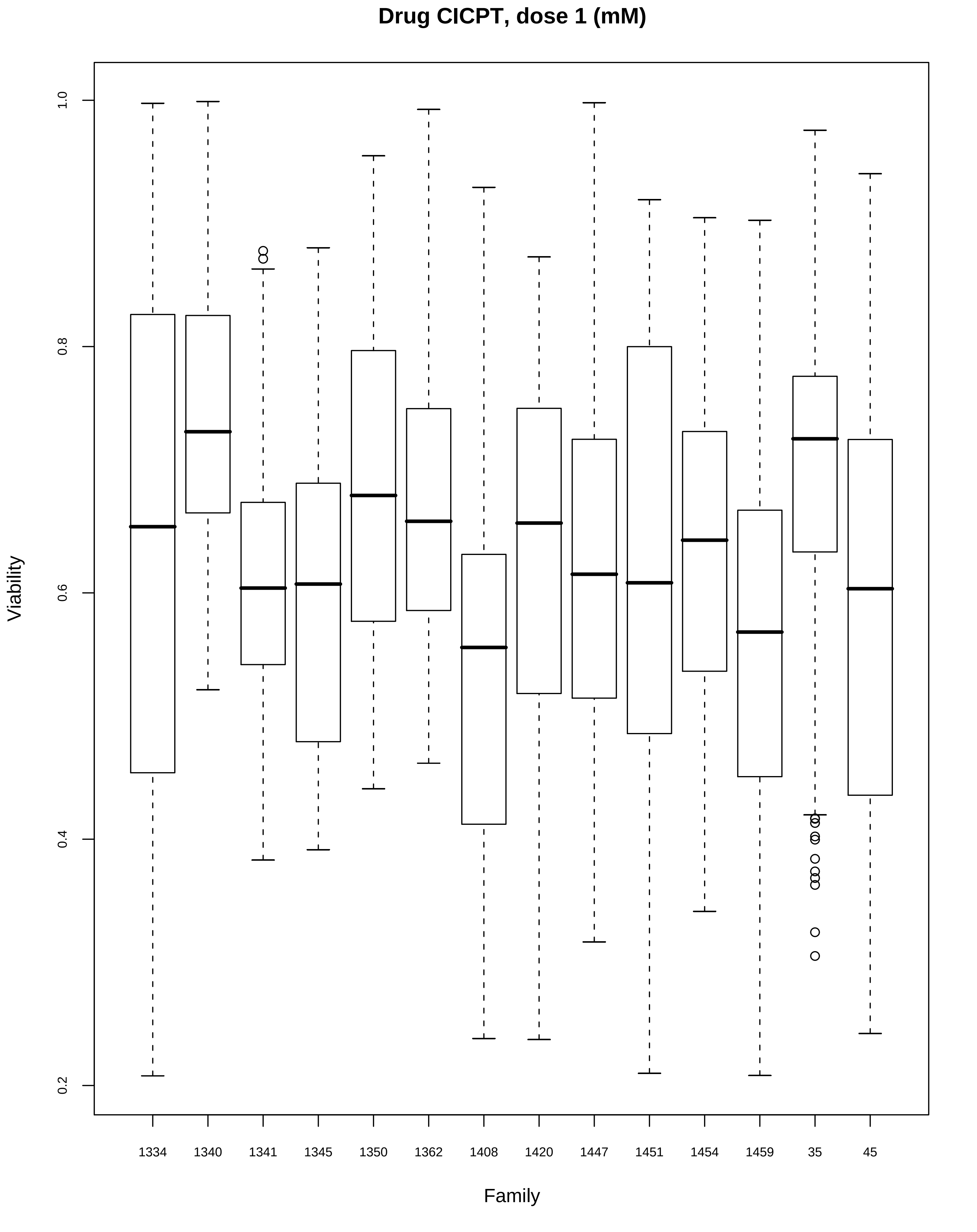
Drug ClCPT, dose 1 (mM)
Viability
0.2
0.4
0.6
0.8
1.0
Family
1334
1340
1341
1345
1350
1362
1408
1420
1447
1451
1454
1459
35
45

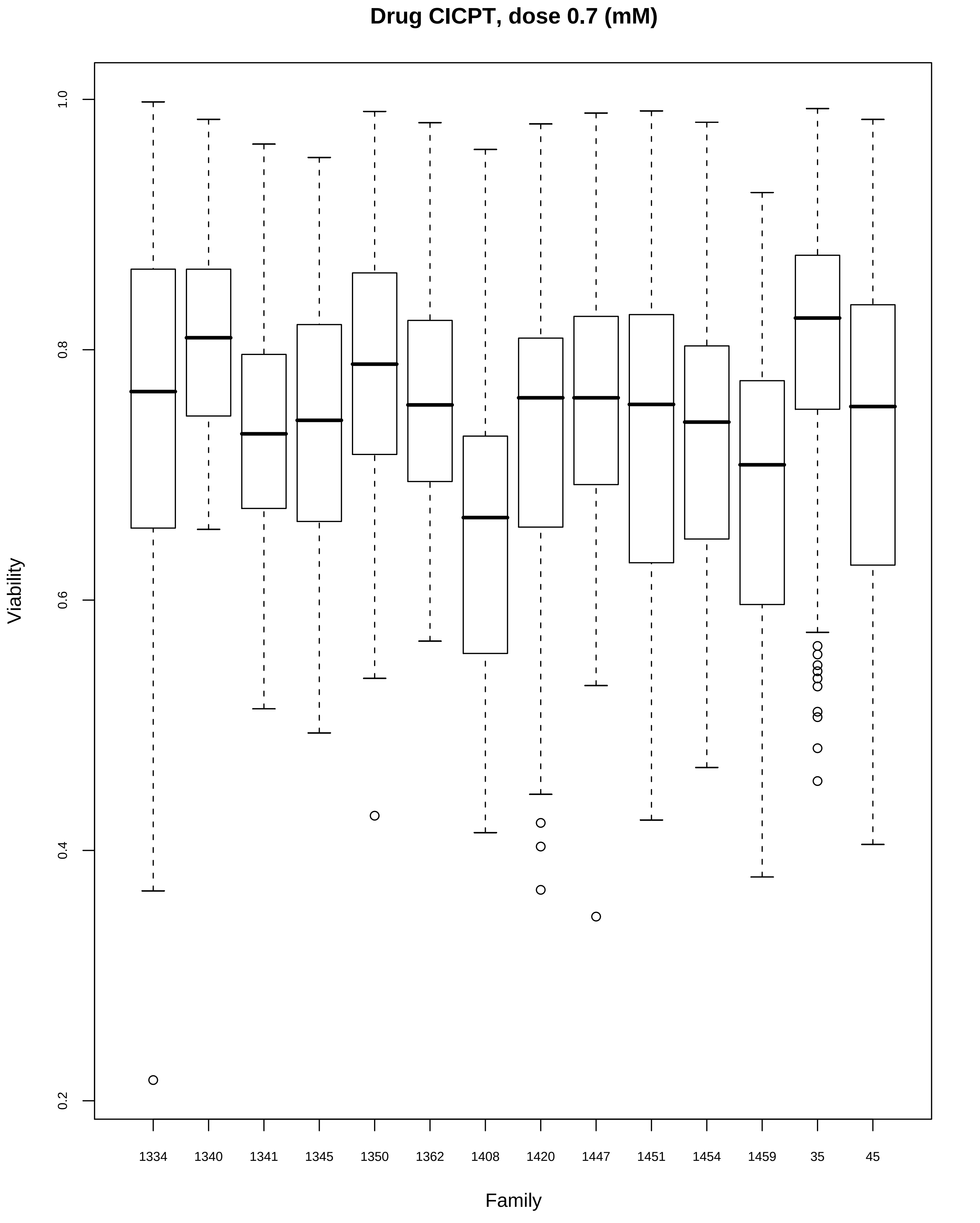
Drug CICPT, dose 0.7 (mM)
Viability
1.0
0.8
0.6
0.4
0.2
1334
1340
1341
1345
1350
1362
1408
1420
1447
1451
1454
1459
35
45
Family

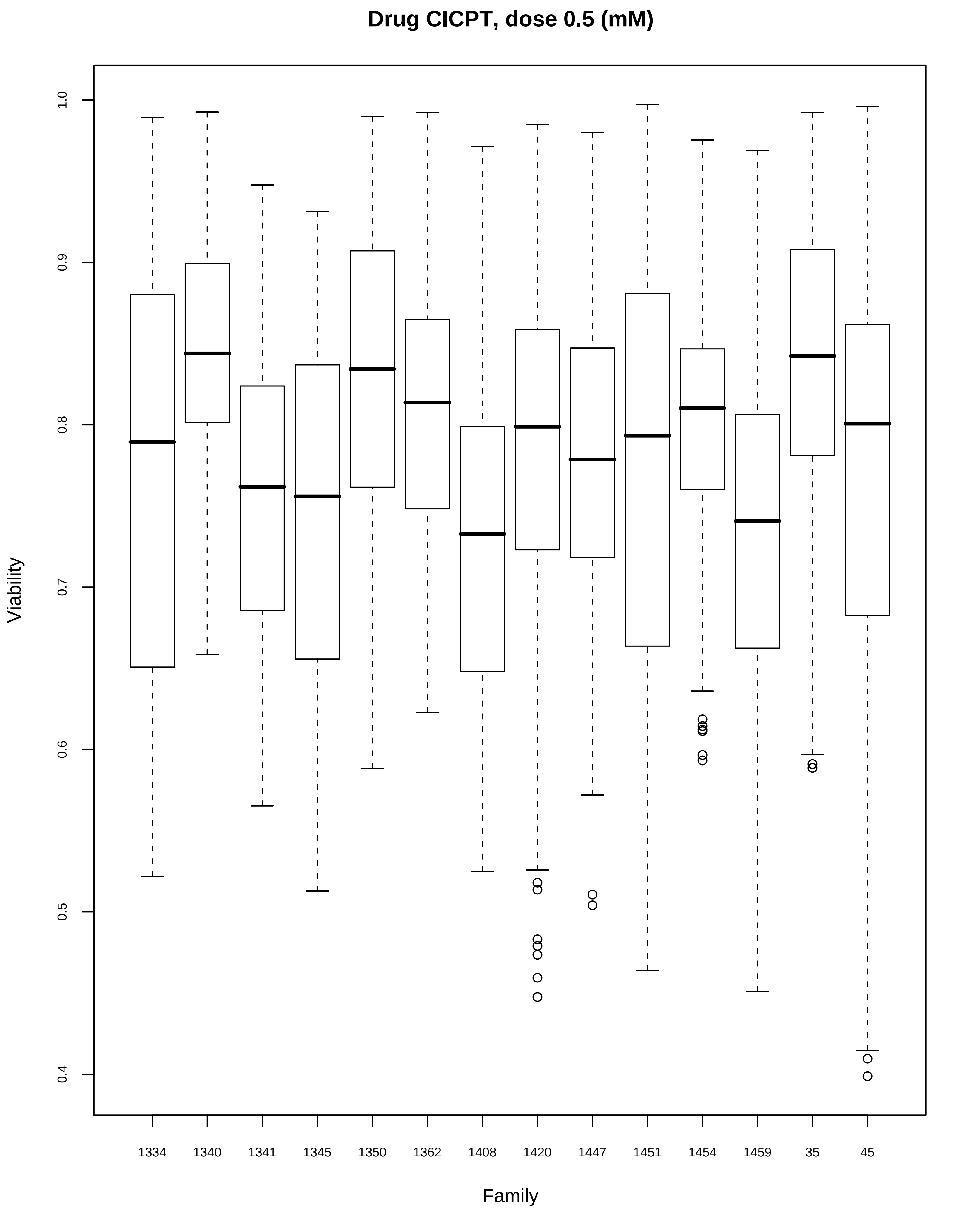
Drug ClCPT, dose 0.5 (mM)
Viability
1.0
0.9
0.8
0.7
0.6
0.5
0.4
1334
1340
1341
1345
1350
1362
1408
1420
1447
1451
1454
1459
35
45
Family

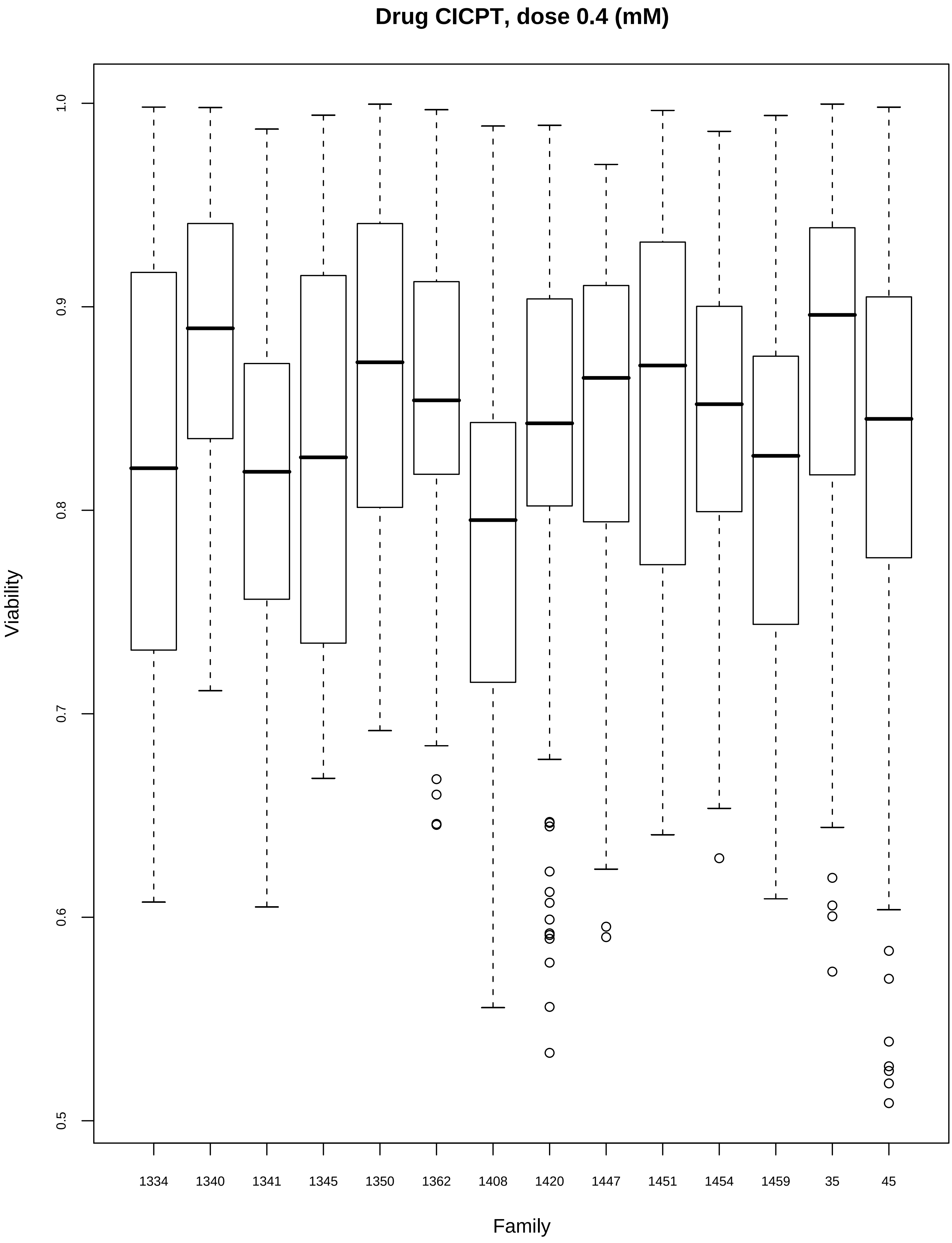
Drug CICPT, dose 0.4 (mM)
Viability
1.0
0.9
0.8
0.7
0.6
0.5
1334
1340
1341
1345
1350
1362
1408
1420
1447
1451
1454
1459
35
45
Family

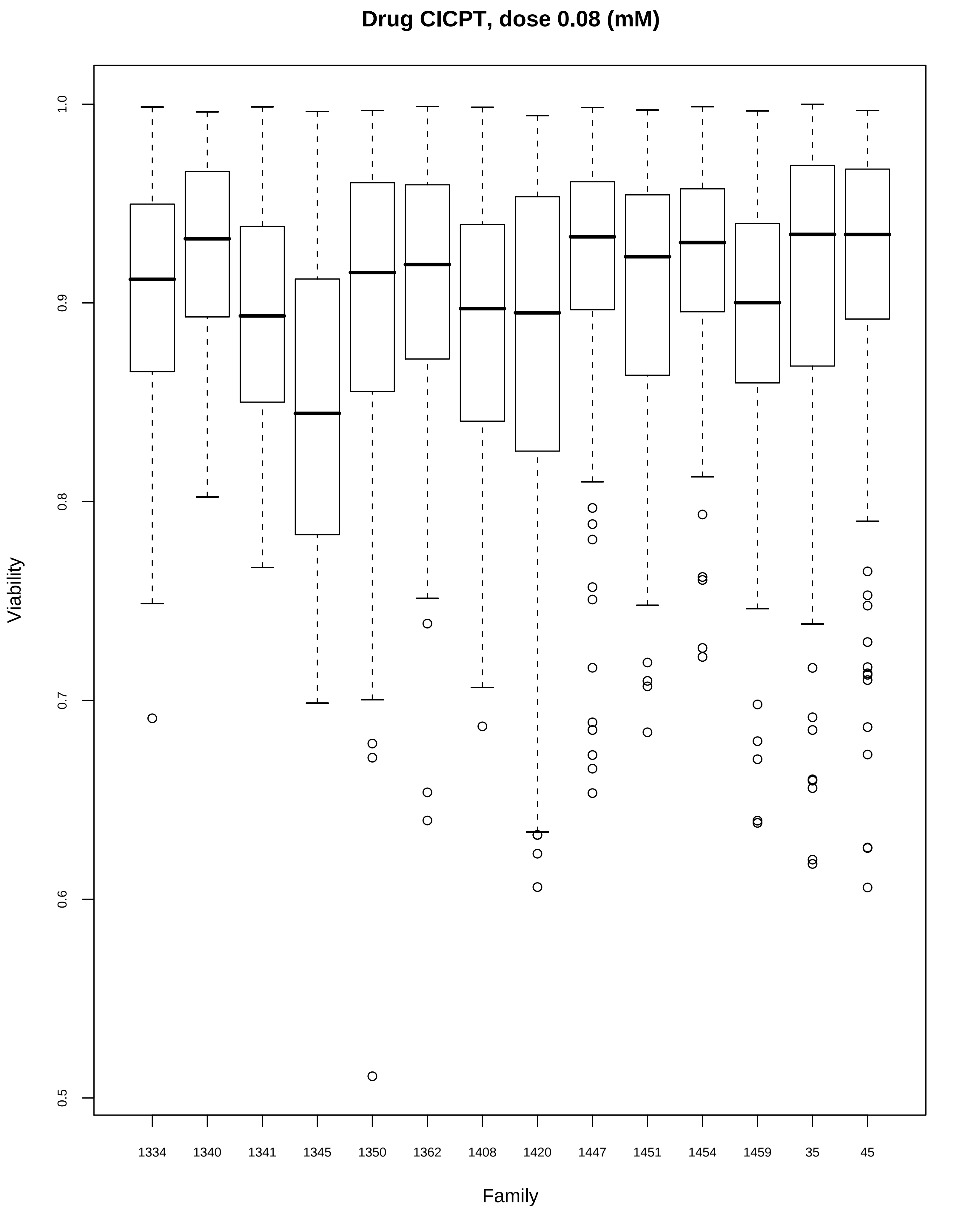
Drug CICPT, dose 0.08 (mM)
Viability
1.0
0.9
0.8
0.7
0.6
0.5
1334
1340
1341
1345
1350
1362
1408
1420
1447
1451
1454
1459
35
45
Family

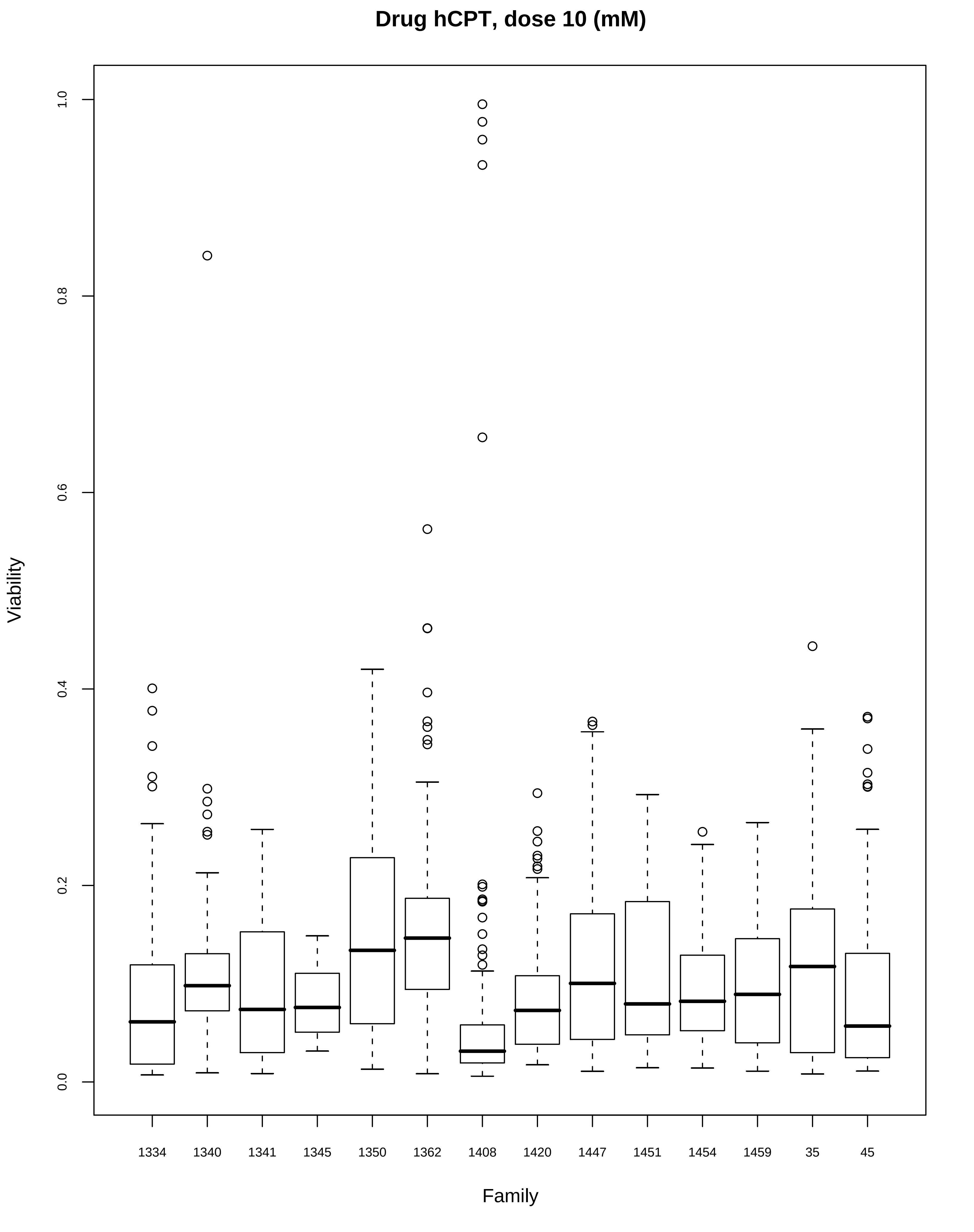

Drug hCPT, dose 10 (mM)
Viability
0.0
0.2
0.4
0.6
0.8
1.0
1334
1340
1341
1345
1350
1362
1408
1420
1447
1451
1454
1459
35
45
Family

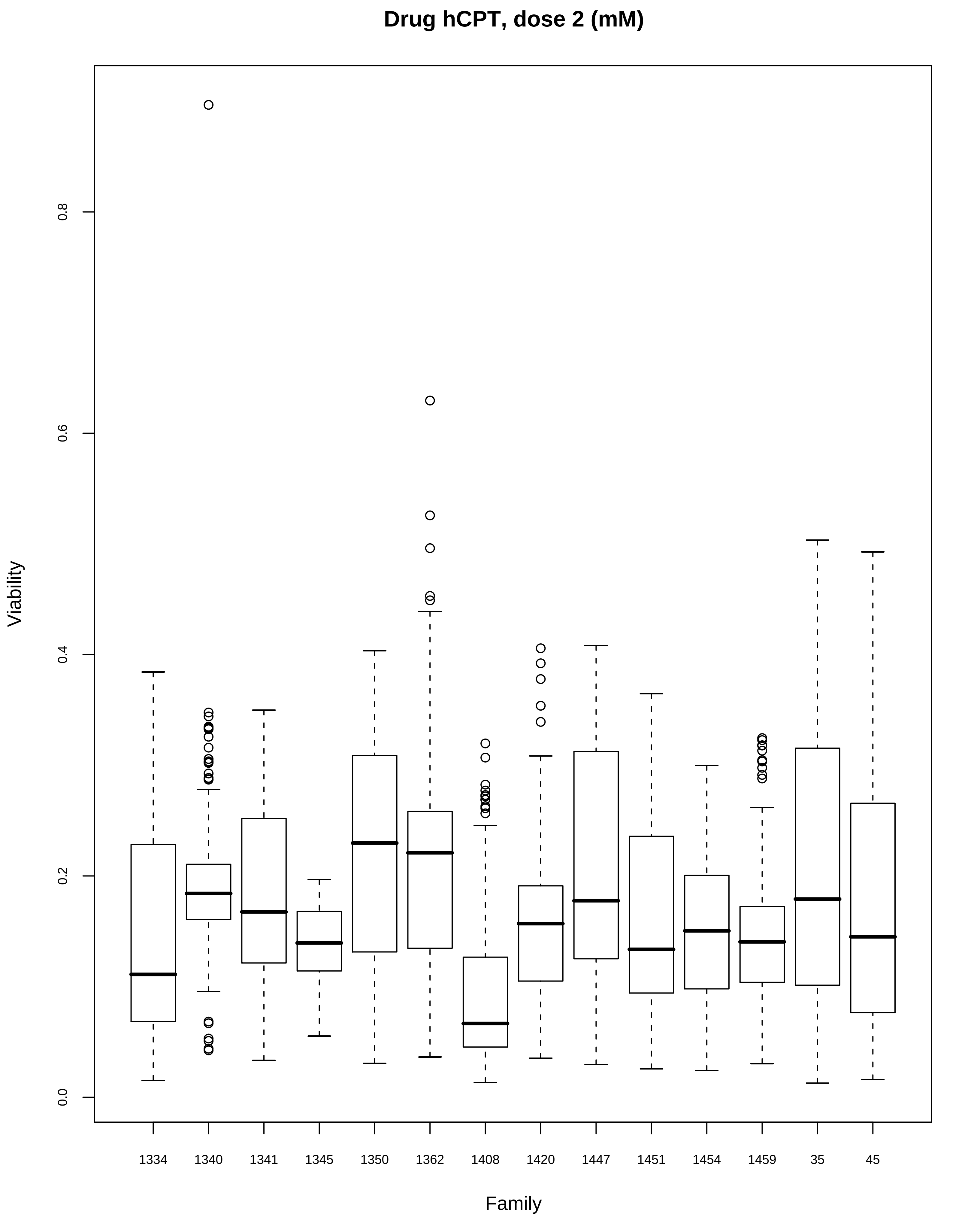
Drug hCPT, dose 2 (mM)
Viability
0.0
0.2
0.4
0.6
0.8
1334
1340
1341
1345
1350
1362
1408
1420
1447
1451
1454
1459
35
45
Family

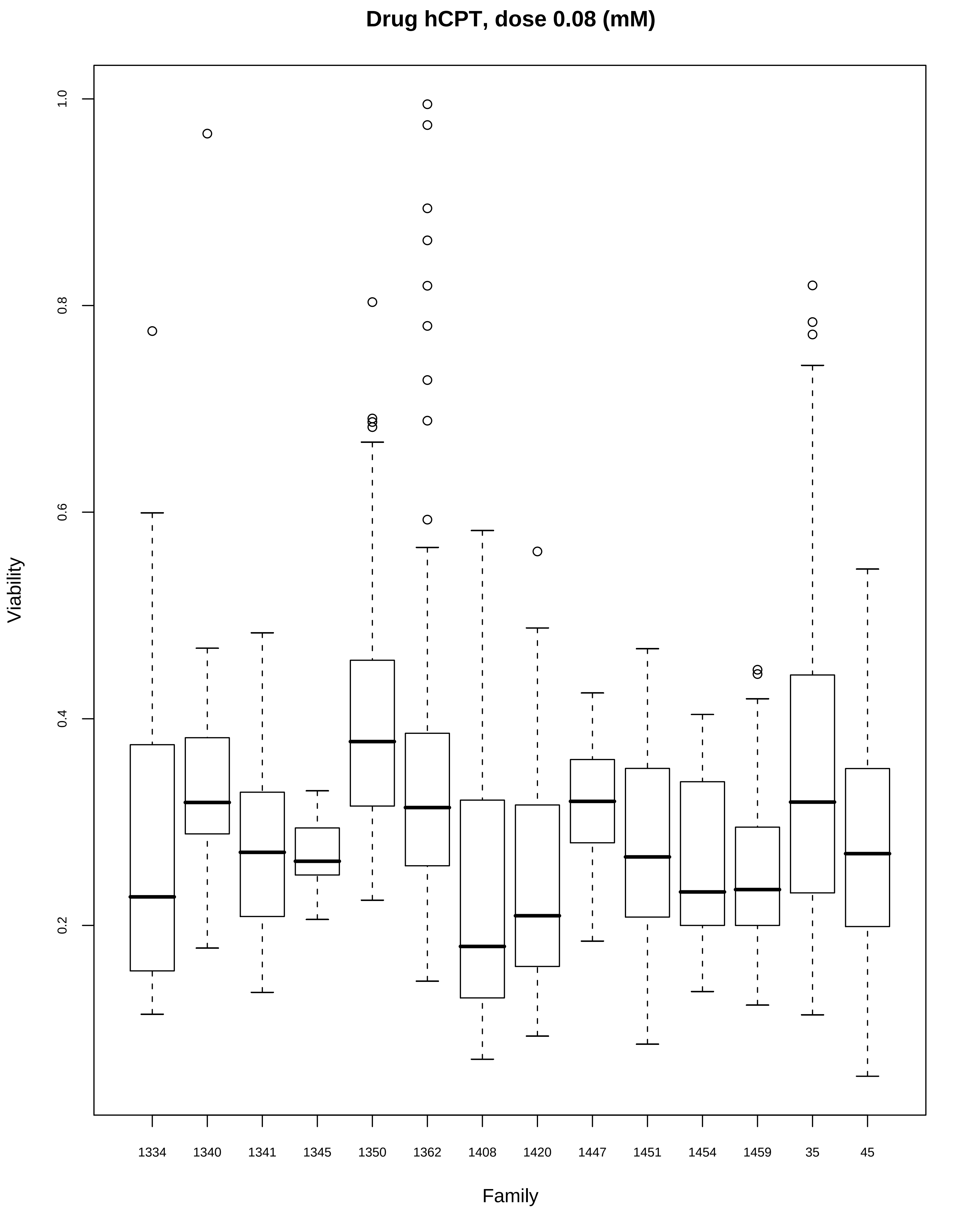

Drug hCPT, dose 0.08 (mM)
Viability
0.2
0.4
0.6
0.8
1.0
1334
1340
1341
1345
1350
1362
1408
1420
1447
1451
1454
1459
35
45
Family

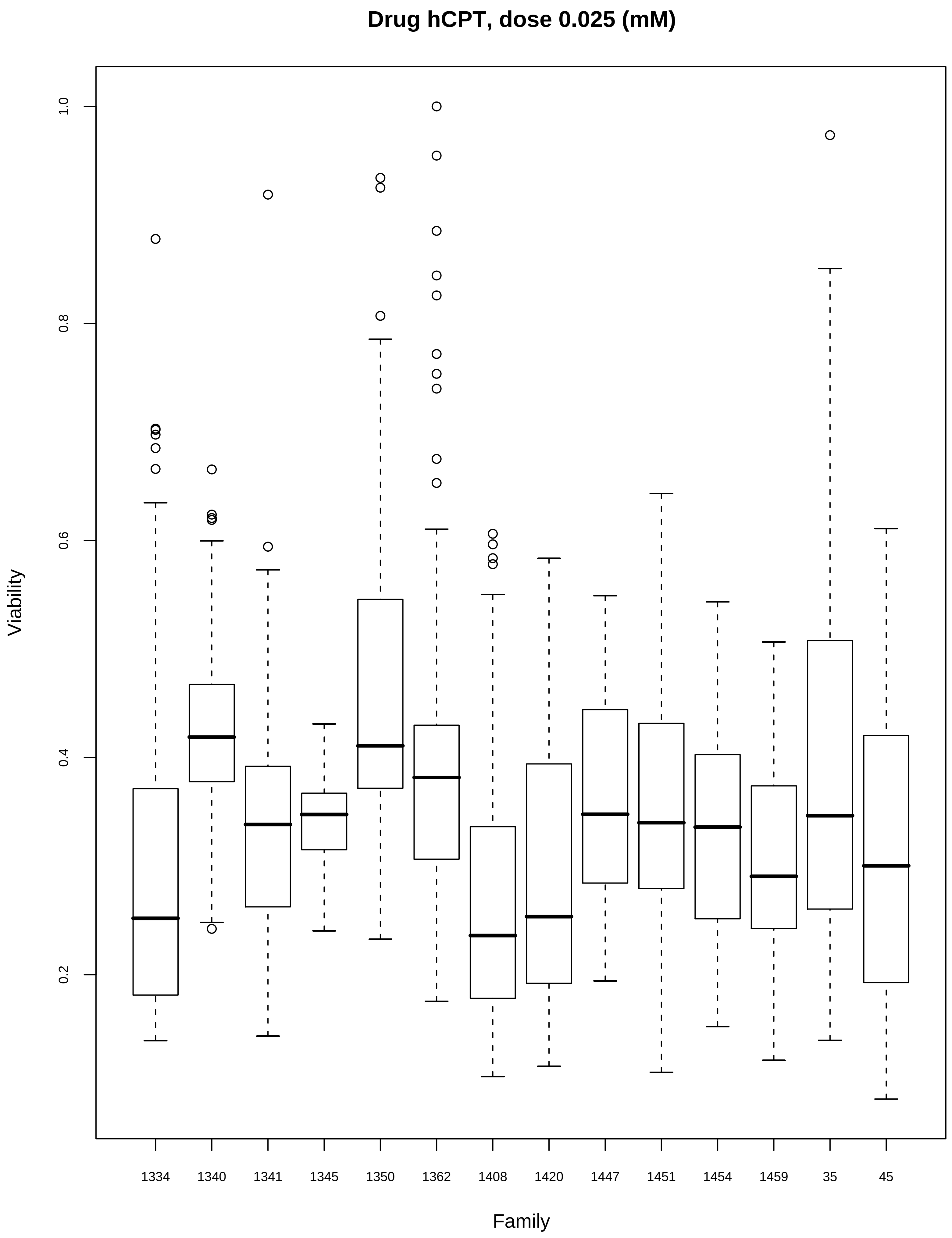

Drug hCPT, dose 0.025 (mM)
Viability
0.2
0.4
0.6
0.8
1.0
1334
1340
1341
1345
1350
1362
1408
1420
1447
1451
1454
1459
35
45
Family

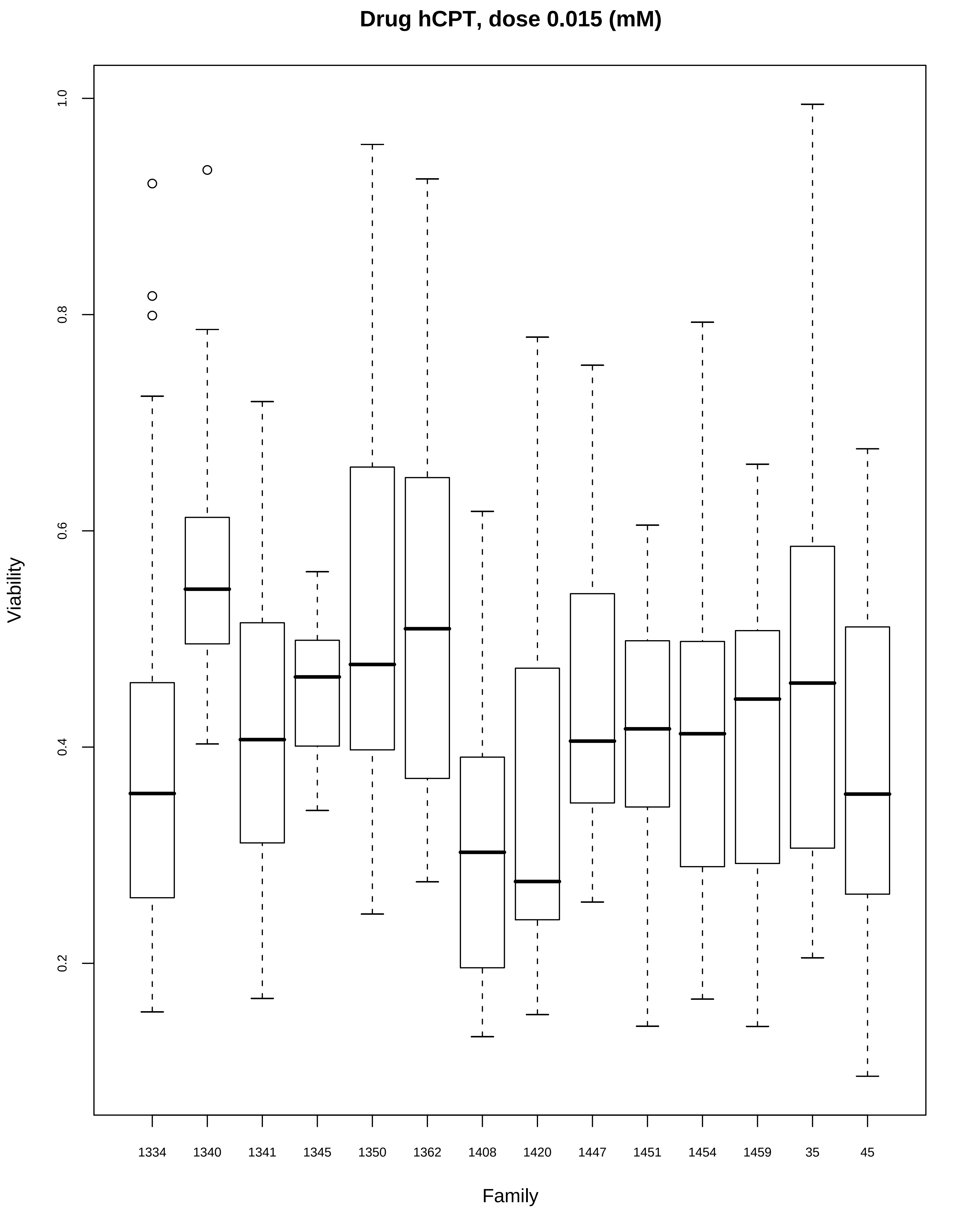

Drug hCPT, dose 0.015 (mM)
Viability
1.0
0.8
0.6
0.4
0.2
1334
1340
1341
1345
1350
1362
1408
1420
1447
1451
1454
1459
35
45
Family

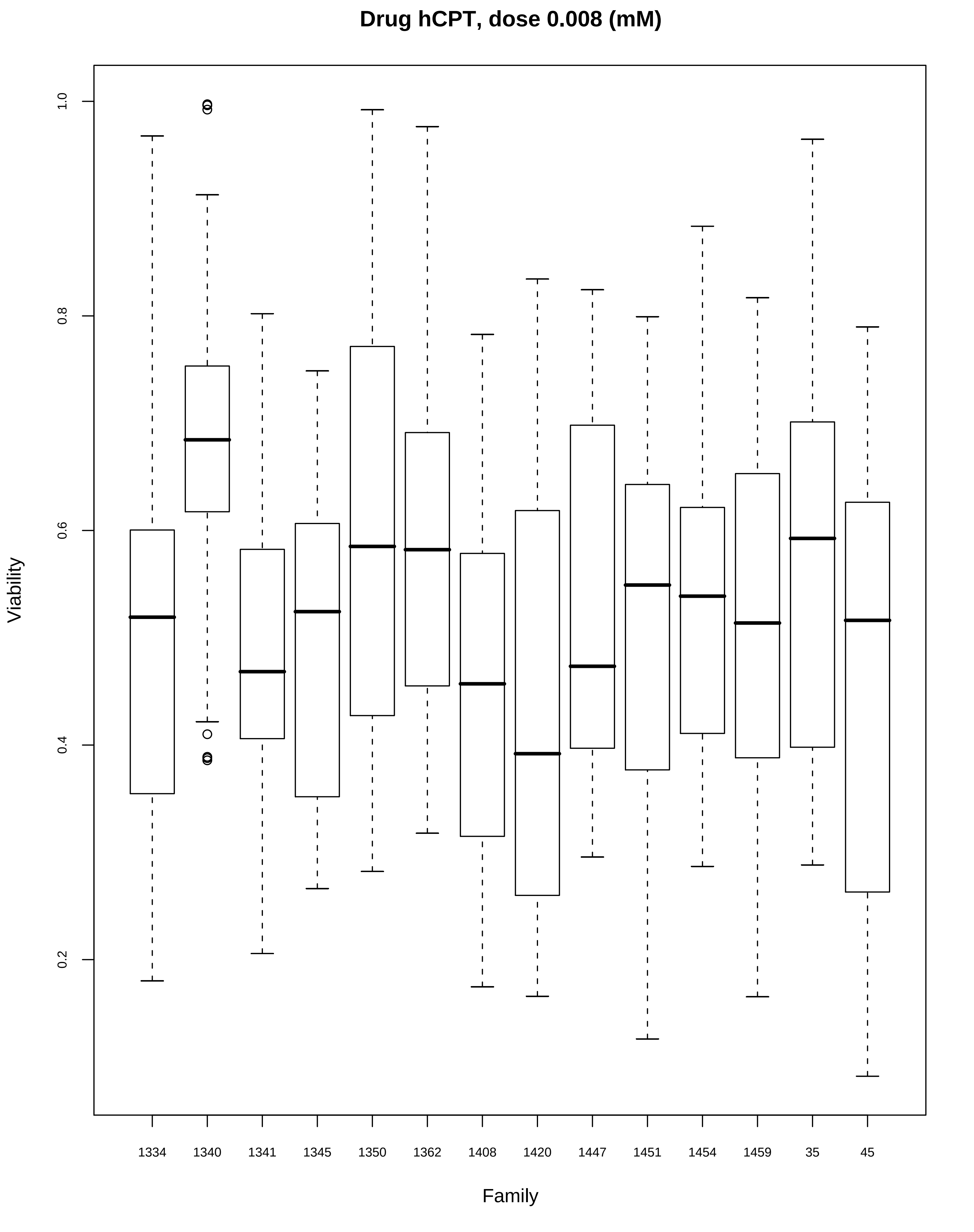

Drug hCPT, dose 0.008 (mM)
Viability
0.2
0.4
0.6
0.8
1.0
1334
1340
1341
1345
1350
1362
1408
1420
1447
1451
1454
1459
35
45
Family

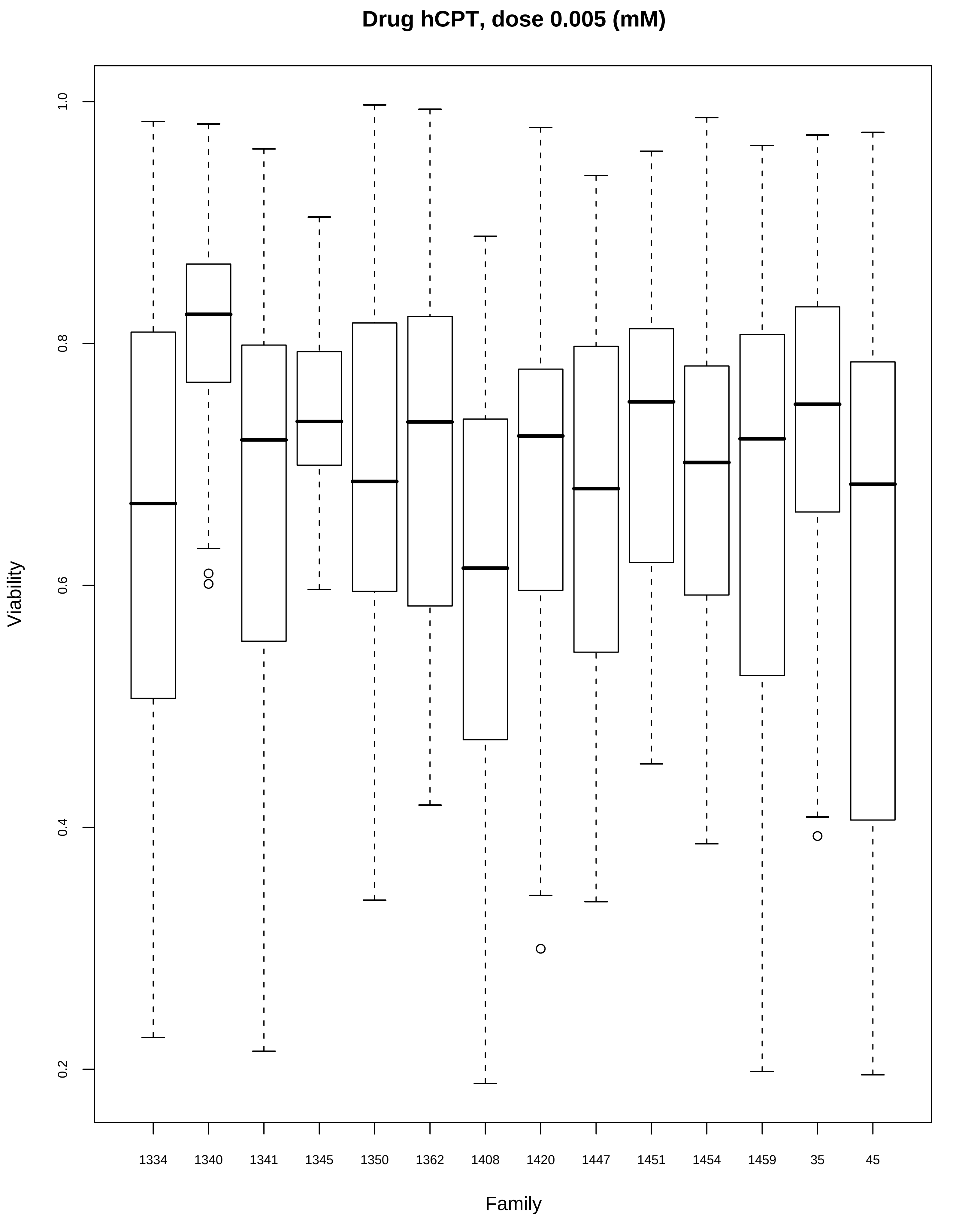

Drug hCPT, dose 0.005 (mM)
Viability
0.2
0.4
0.6
0.8
1.0
1334
1340
1341
1345
1350
1362
1408
1420
1447
1451
1454
1459
35
45
Family

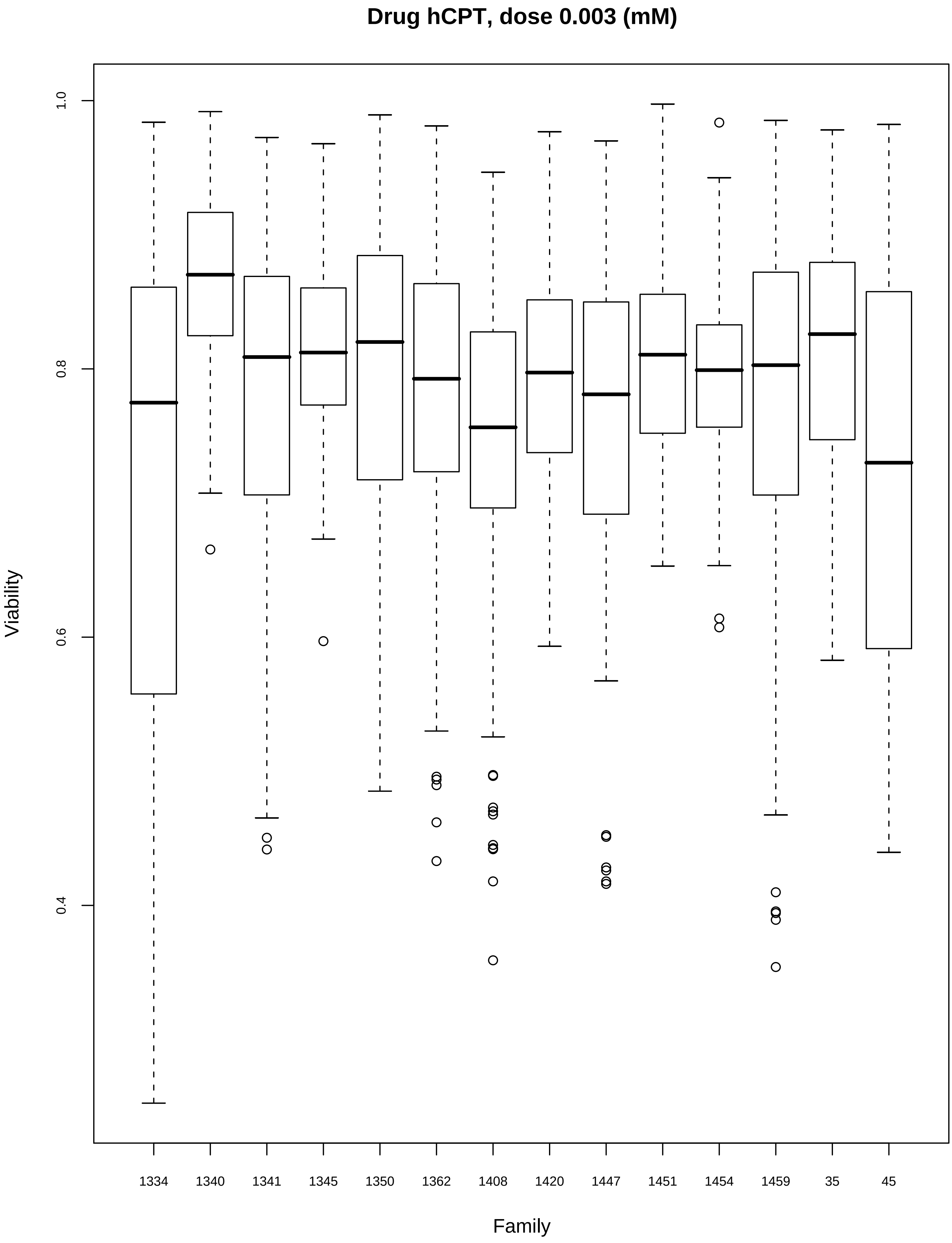
Drug hCPT, dose 0.003 (mM)
Viability
0.4
0.6
0.8
1.0
1334
1340
1341
1345
1350
1362
1408
1420
1447
1451
1454
1459
35
45
Family

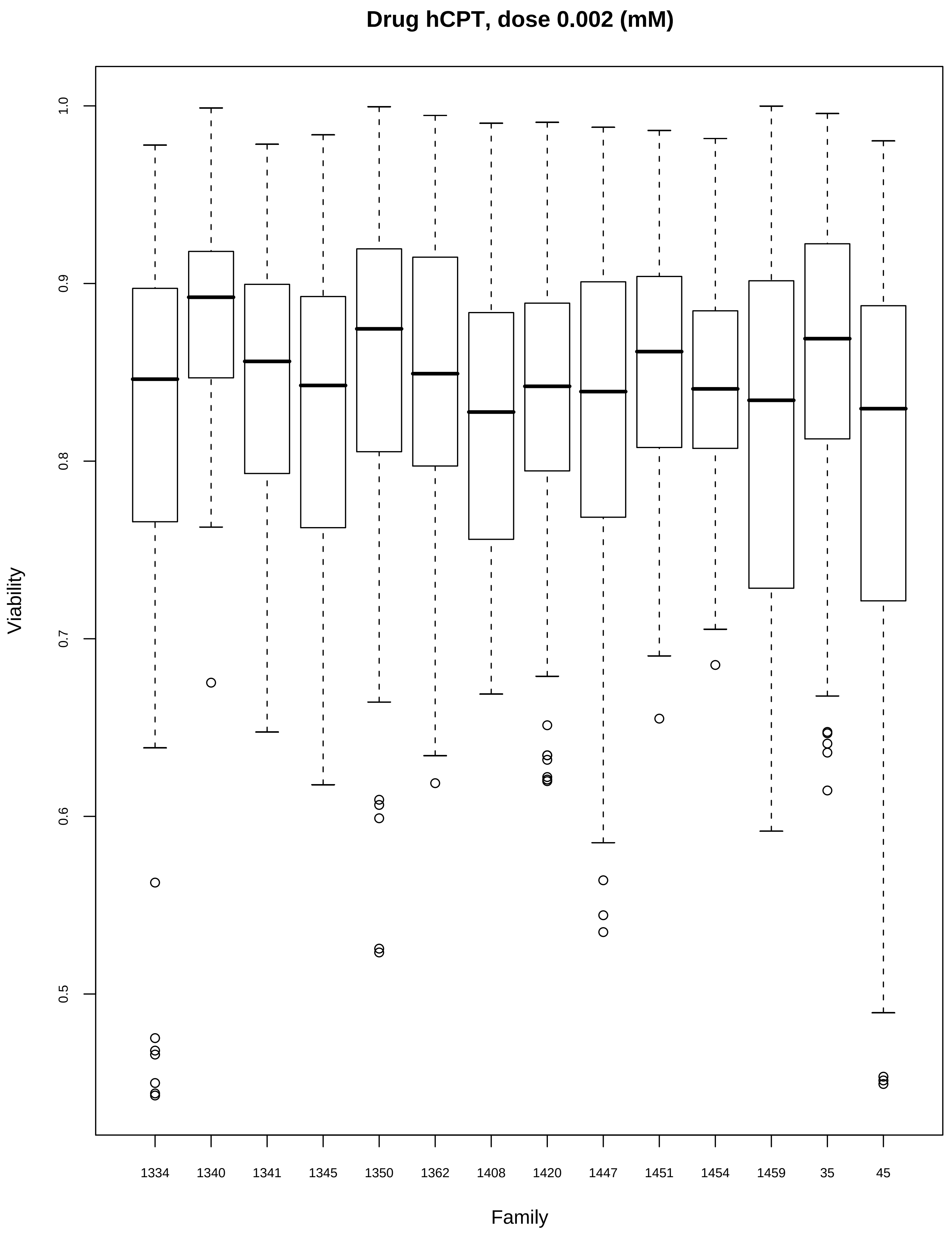

Drug hCPT, dose 0.002 (mM)
Viability
1.0
0.9
0.8
0.7
0.6
0.5
Family
1334
1340
1341
1345
1350
1362
1408
1420
1447
1451
1454
1459
35
45

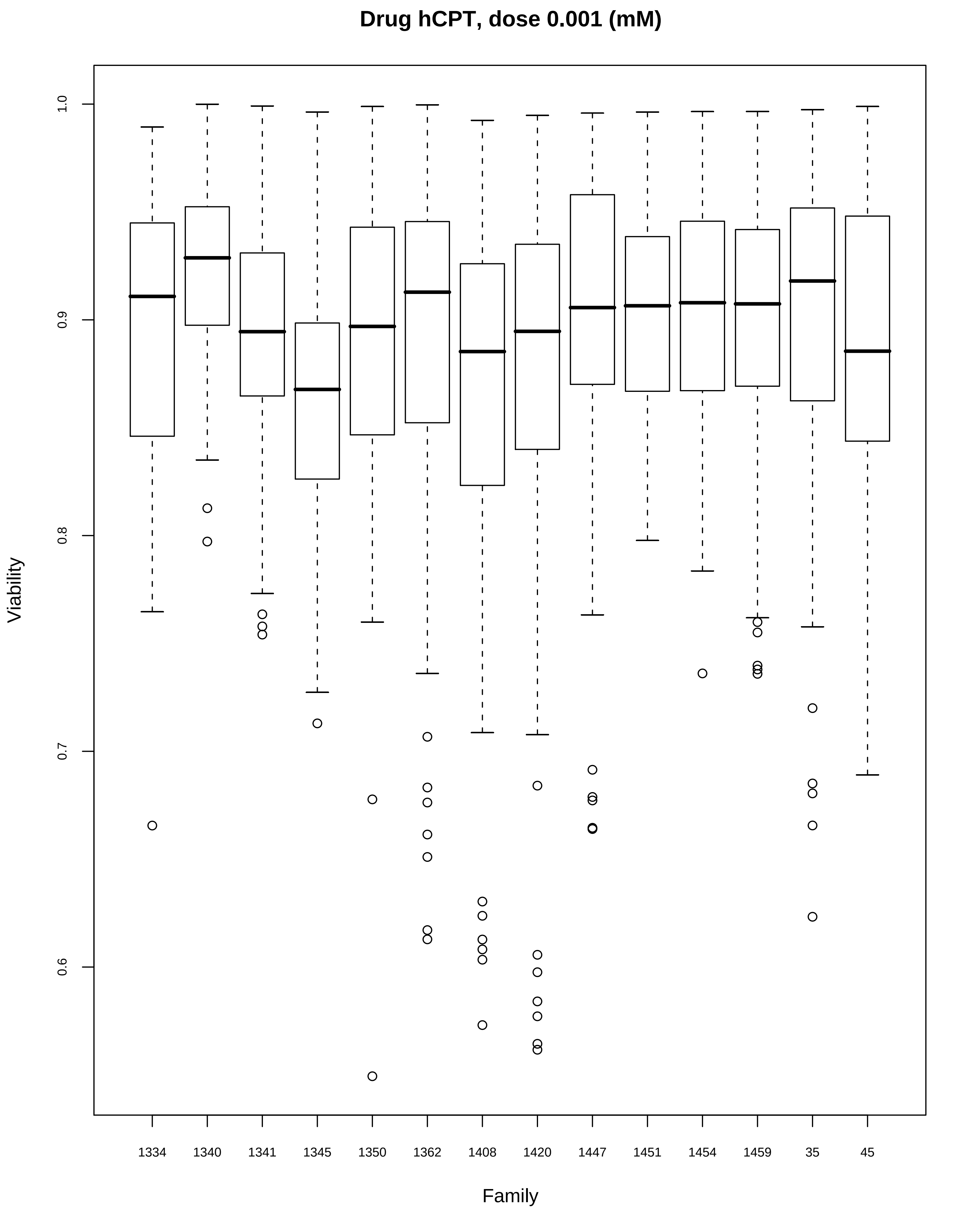

Drug hCPT, dose 0.001 (mM)
Viability
1.0
0.9
0.8
0.7
0.6
Family
1334
1340
1341
1345
1350
1362
1408
1420
1447
1451
1454
1459
35
45

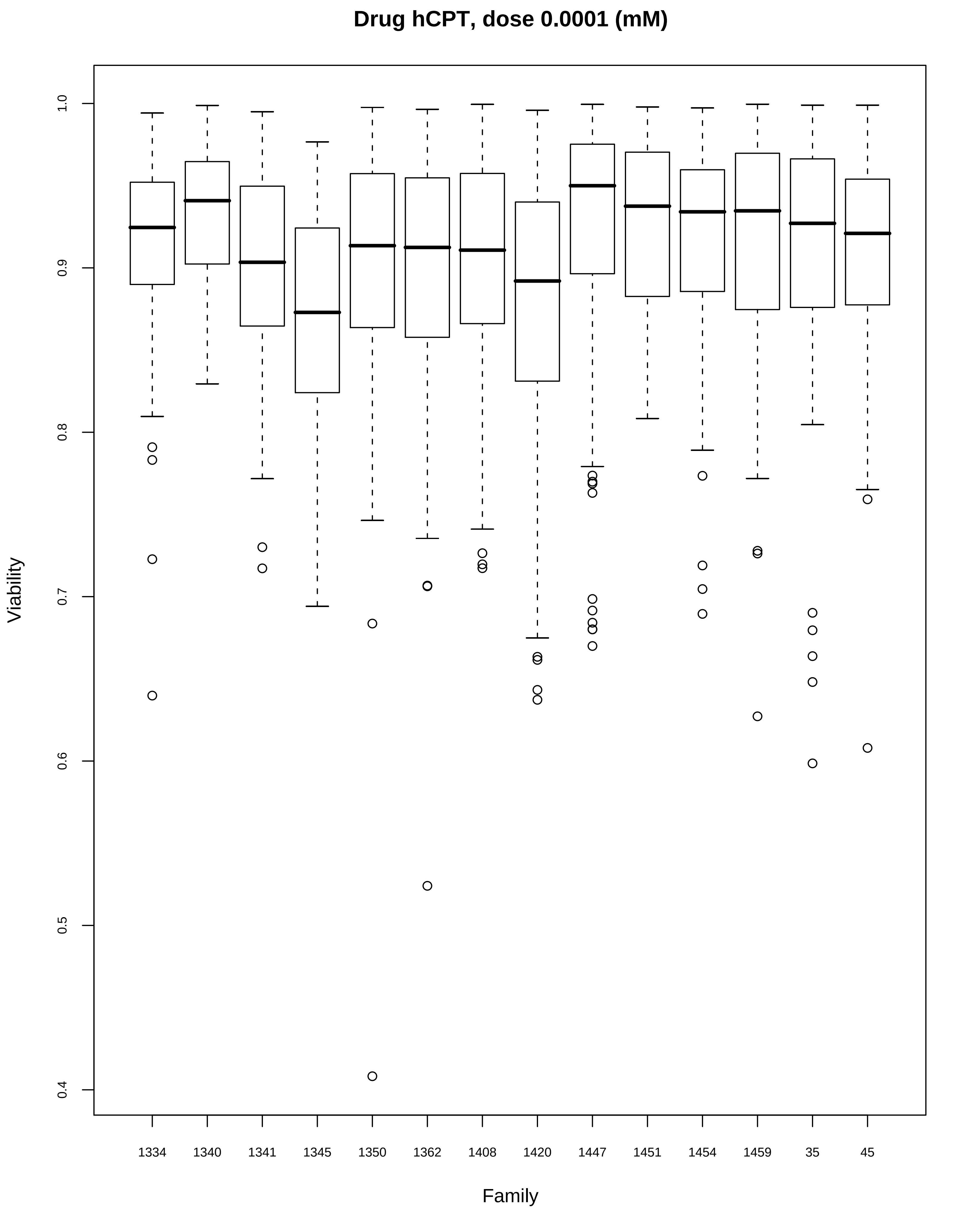
Drug hCPT, dose 0.0001 (mM)
Viability
Family
1.0
0.9
0.8
0.7
0.6
0.5
0.4
1334
1340
1341
1345
1350
1362
1408
1420
1447
1451
1454
1459
35
45

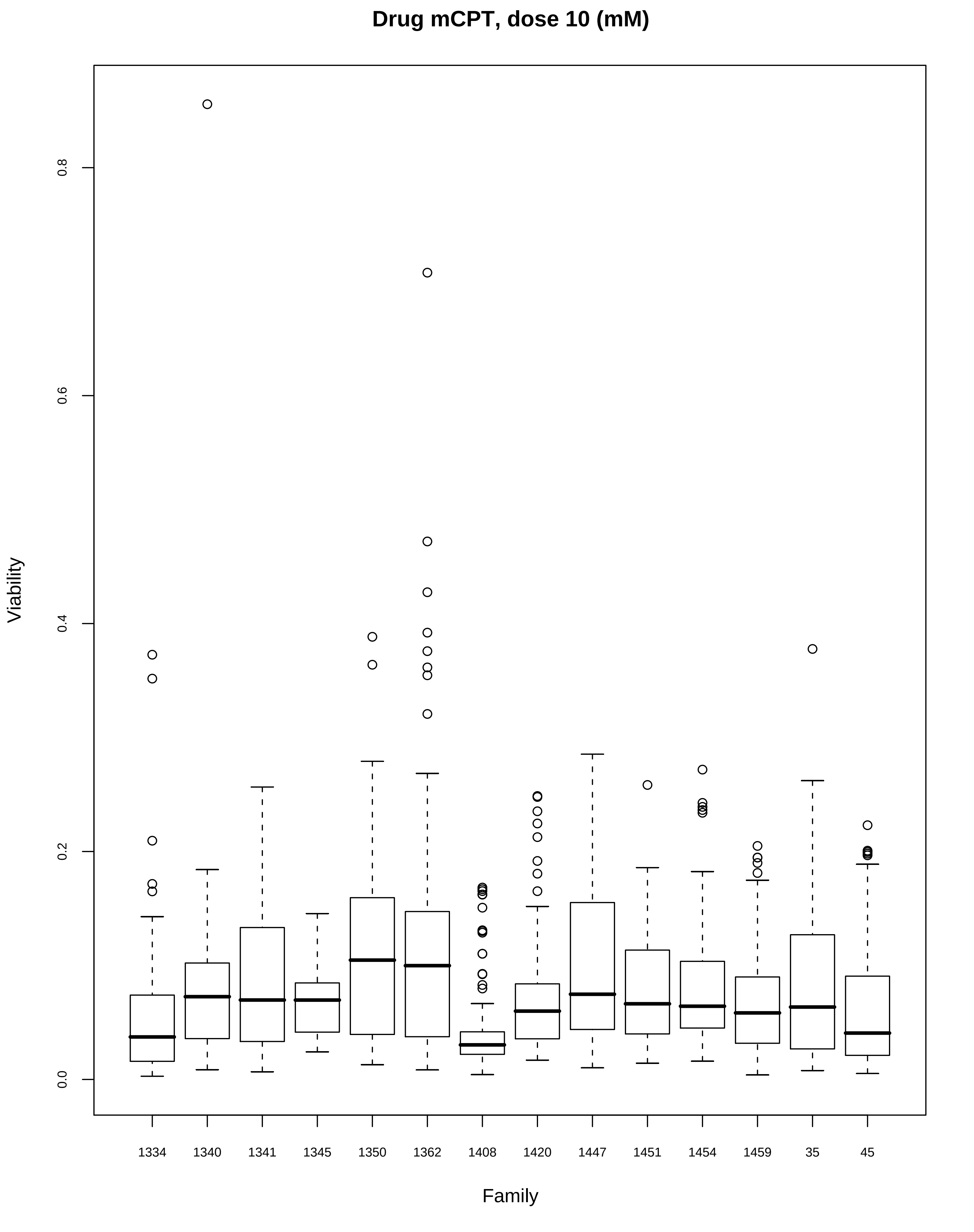
Drug mCPT, dose 10 (mM)
Viability
0.0
0.2
0.4
0.6
0.8
1334
1340
1341
1345
1350
1362
1408
1420
1447
1451
1454
1459
35
45
Family

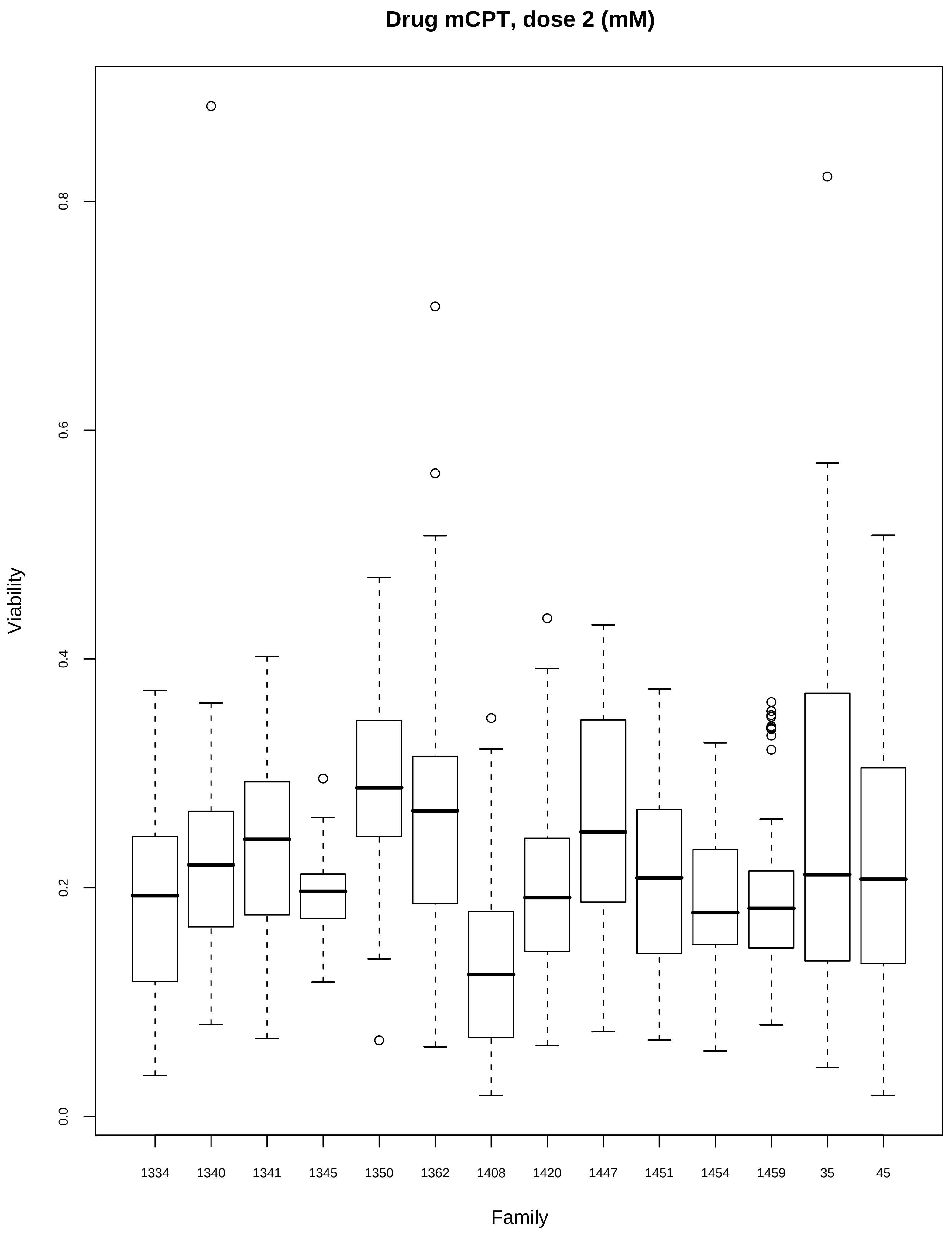
Drug mCPT, dose 2 (mM)
Viability
0.0
0.2
0.4
0.6
0.8
1334
1340
1341
1345
1350
1362
1408
1420
1447
1451
1454
1459
35
45
Family

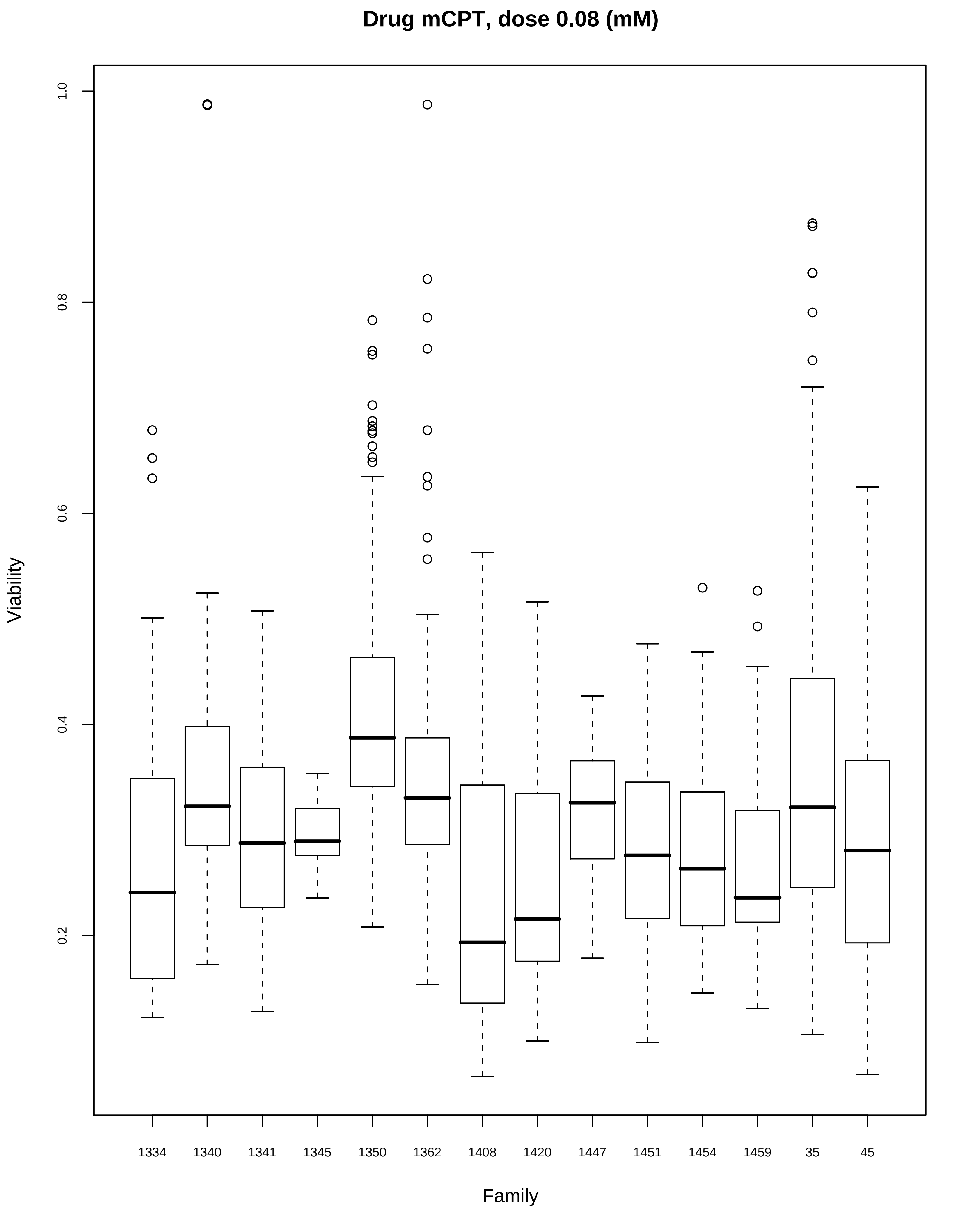
Drug mCPT, dose 0.08 (mM)
Viability
0.2
0.4
0.6
0.8
1.0
1334
1340
1341
1345
1350
1362
1408
1420
1447
1451
1454
1459
35
45
Family

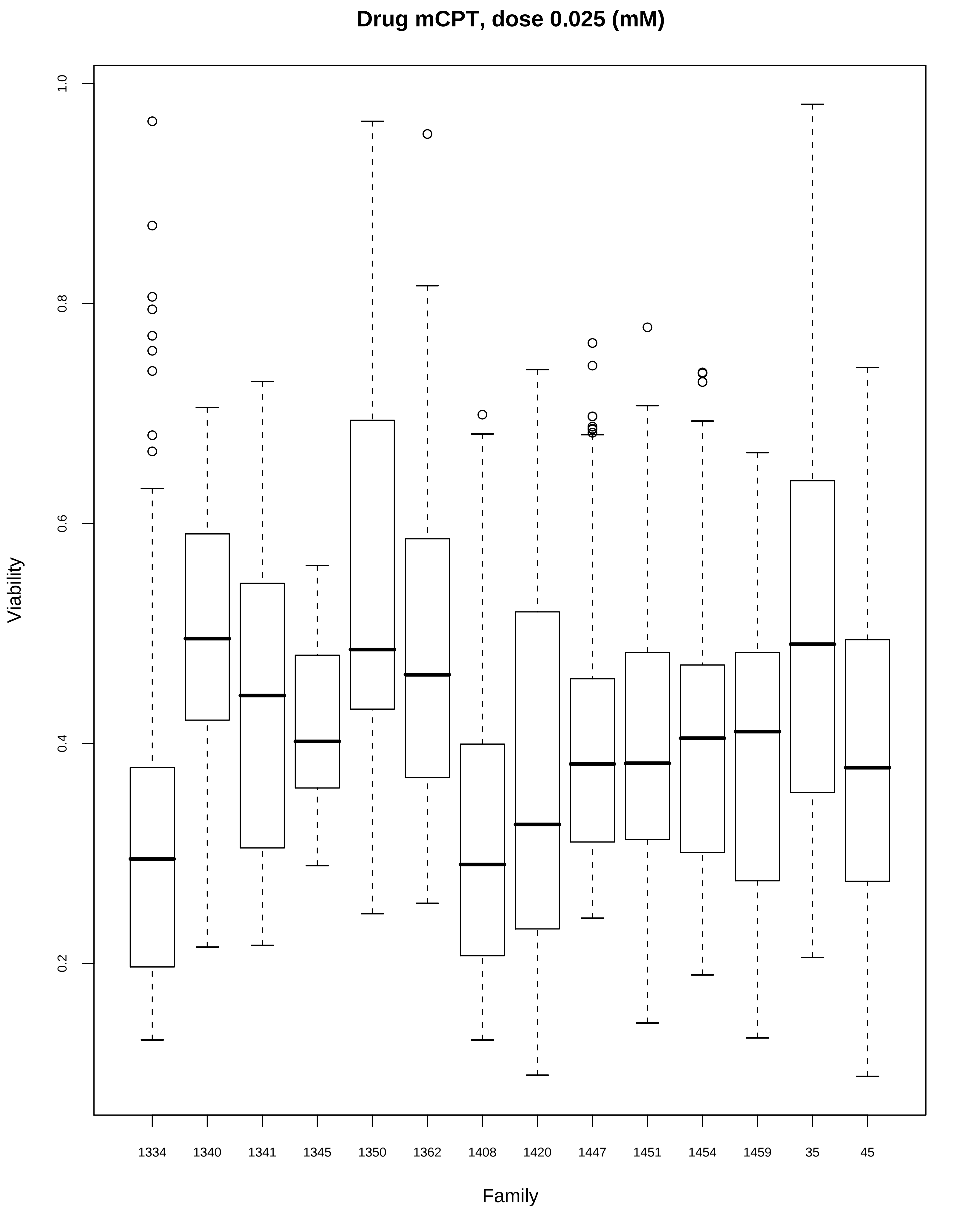
Drug mCPT, dose 0.025 (mM)
Viability
1.0
0.8
0.6
0.4
0.2
1334
1340
1341
1345
1350
1362
1408
1420
1447
1451
1454
1459
35
45
Family

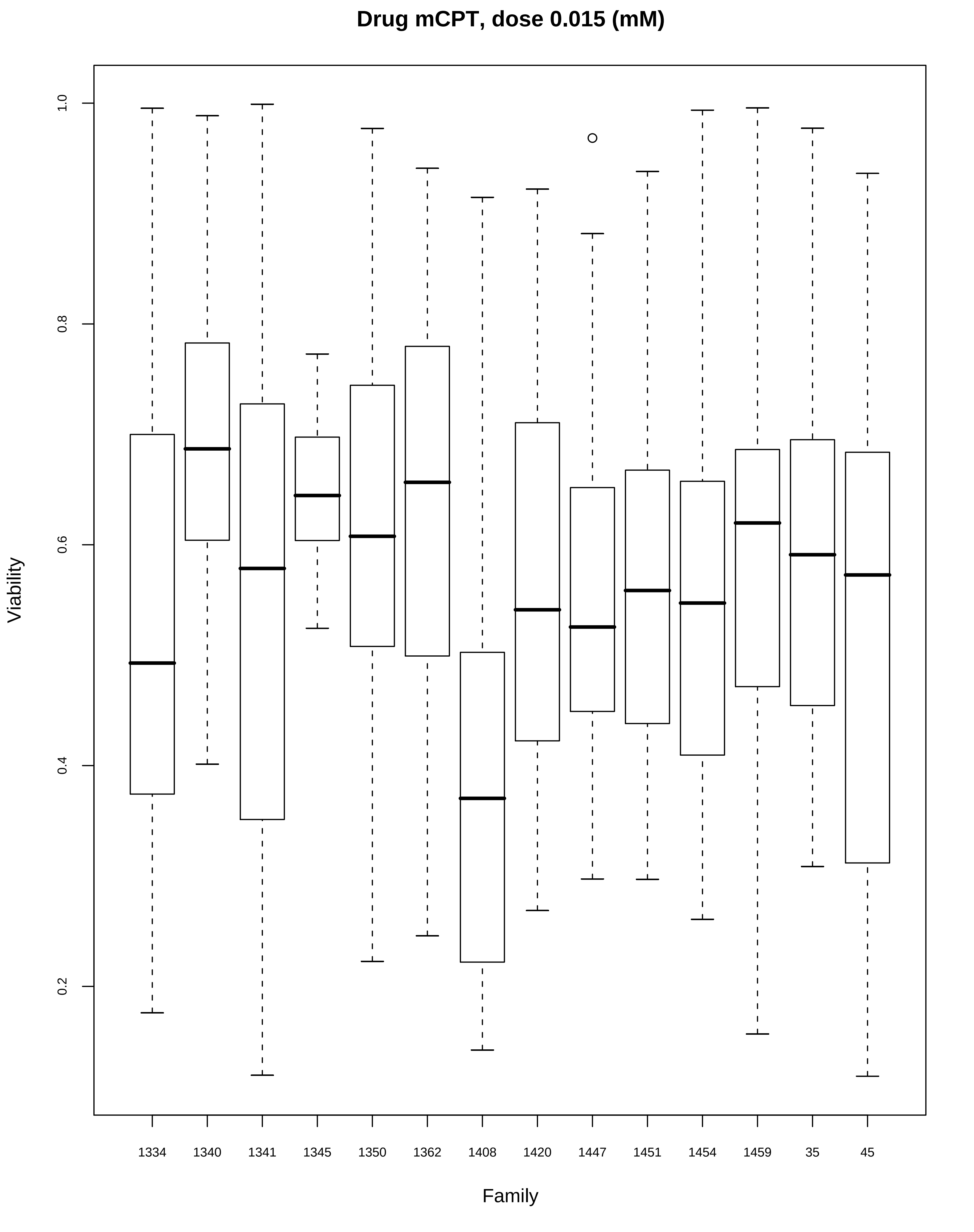
Drug mCPT, dose 0.015 (mM)
Viability
0.2
0.4
0.6
0.8
1.0
1334
1340
1341
1345
1350
1362
1408
1420
1447
1451
1454
1459
35
45
Family

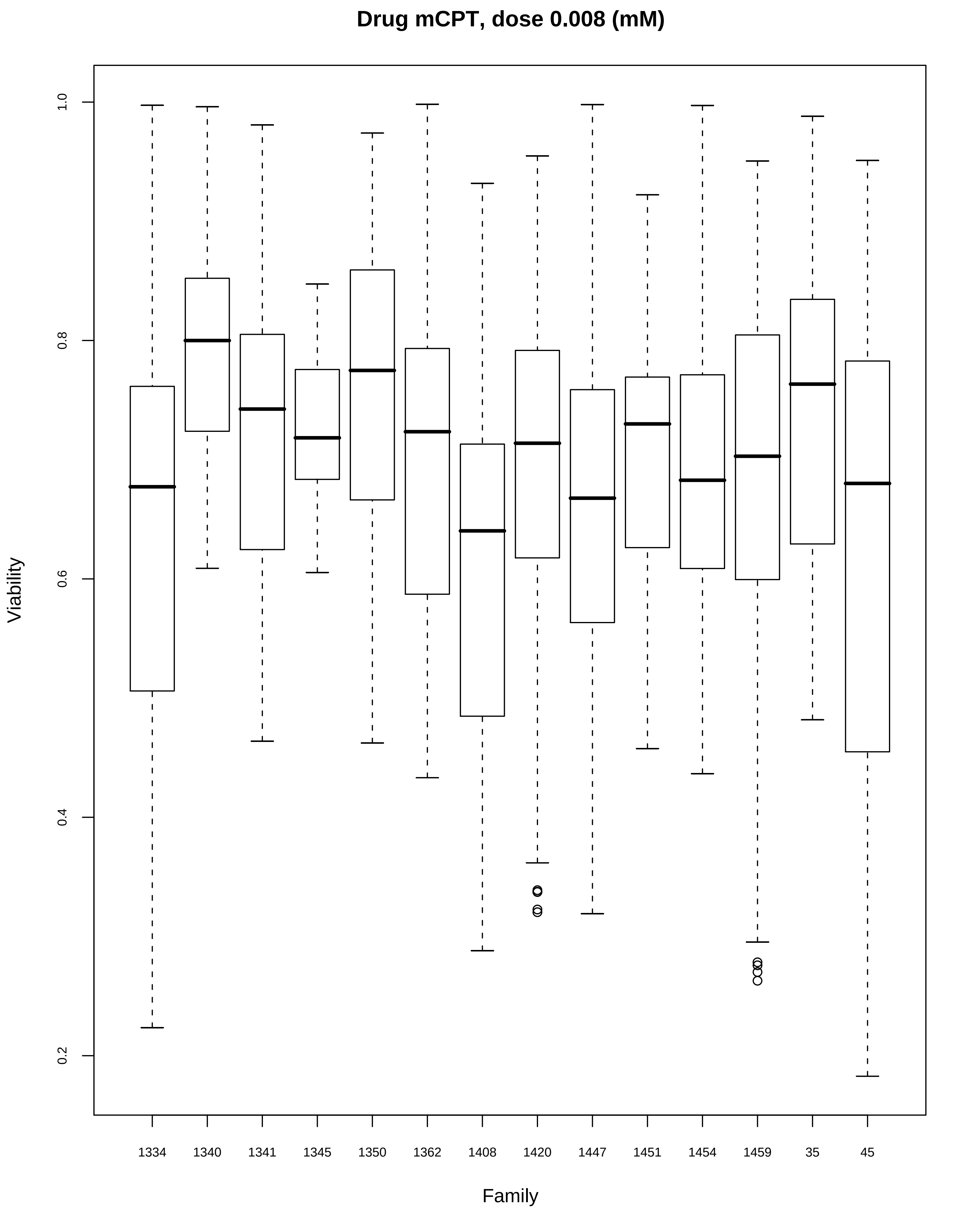

Drug mCPT, dose 0.008 (mM)
Viability
0.2
0.4
0.6
0.8
1.0
1334
1340
1341
1345
1350
1362
1408
1420
1447
1451
1454
1459
35
45
Family

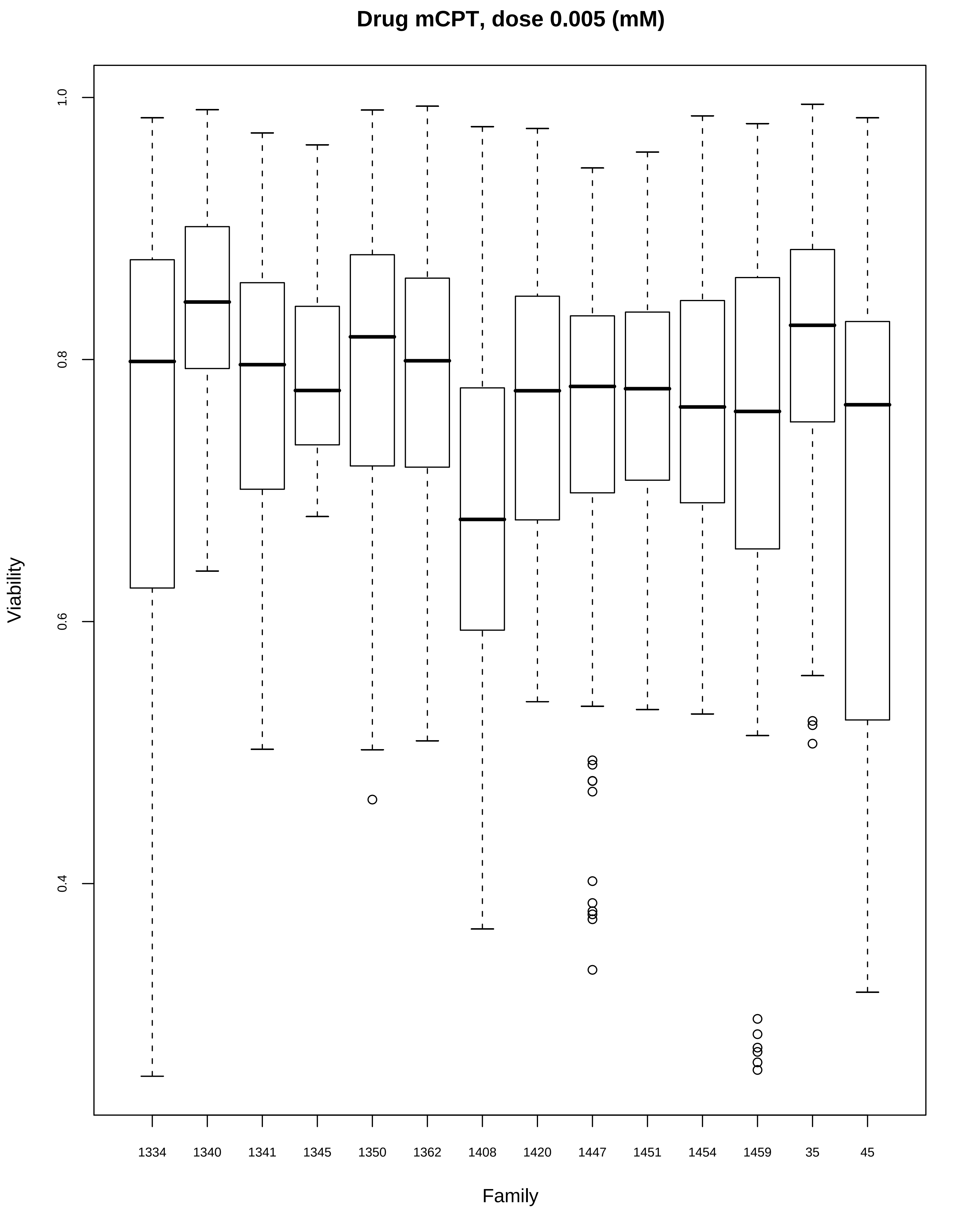
Drug mCPT, dose 0.005 (mM)
Viability
1.0
0.8
0.6
0.4
Family
1334
1340
1341
1345
1350
1362
1408
1420
1447
1451
1454
1459
35
45

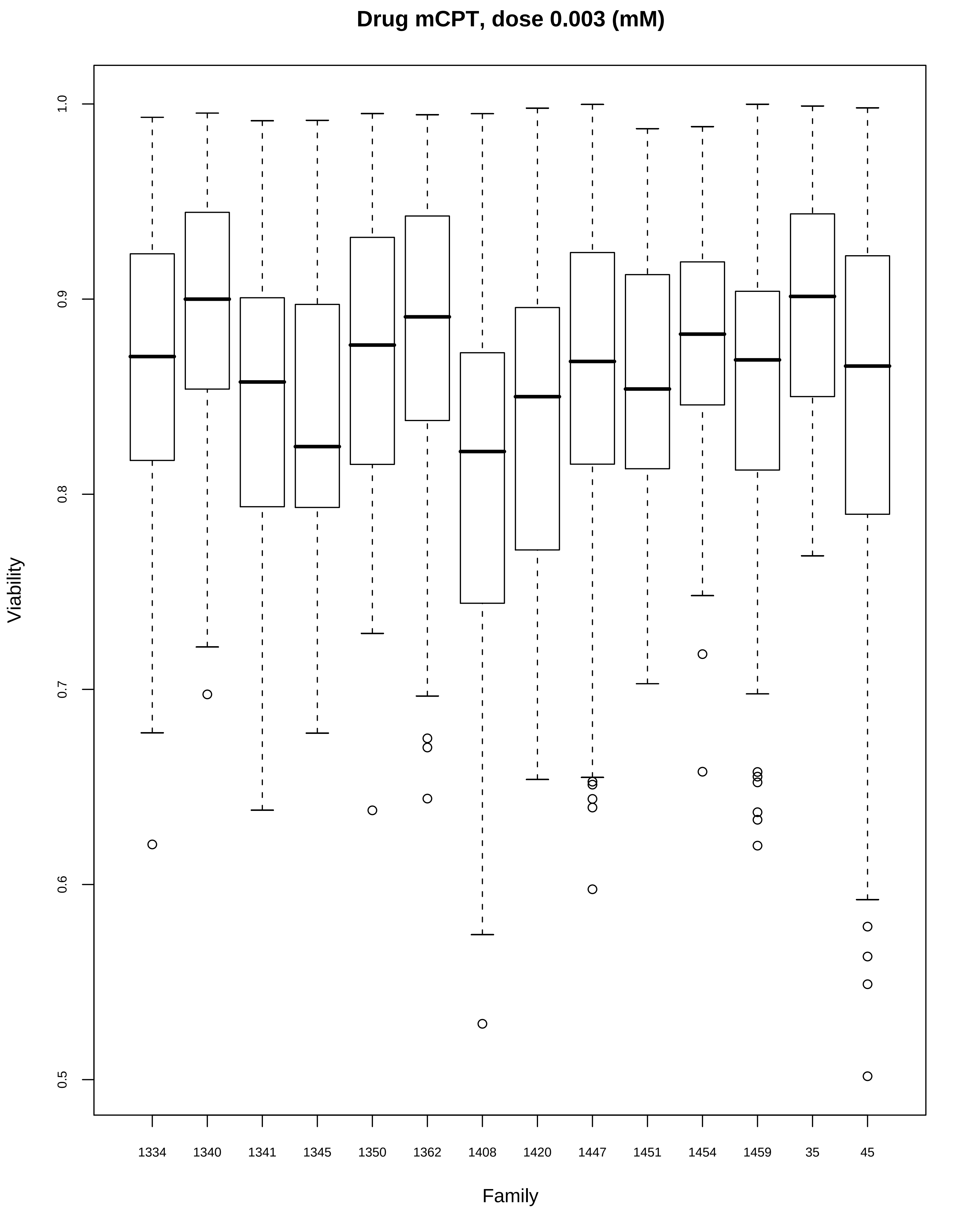

Drug mCPT, dose 0.003 (mM)
Viability
1.0
0.9
0.8
0.7
0.6
0.5
Family
1334
1340
1341
1345
1350
1362
1408
1420
1447
1451
1454
1459
35
45

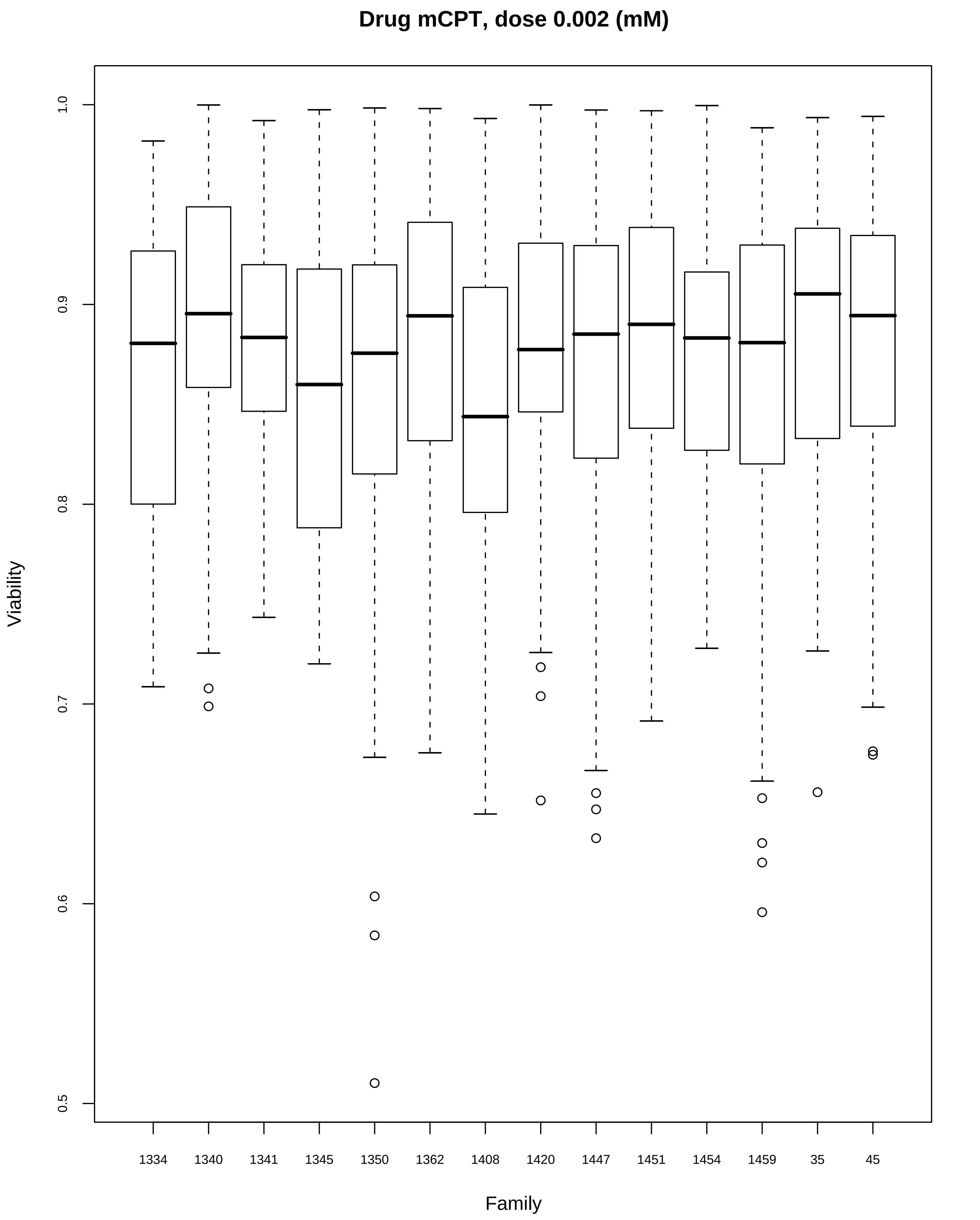
Drug mCPT, dose 0.002 (mM)
Viability
1.0
0.9
0.8
0.7
0.6
0.5
1334
1340
1341
1345
1350
1362
1408
1420
1447
1451
1454
1459
35
45
Family

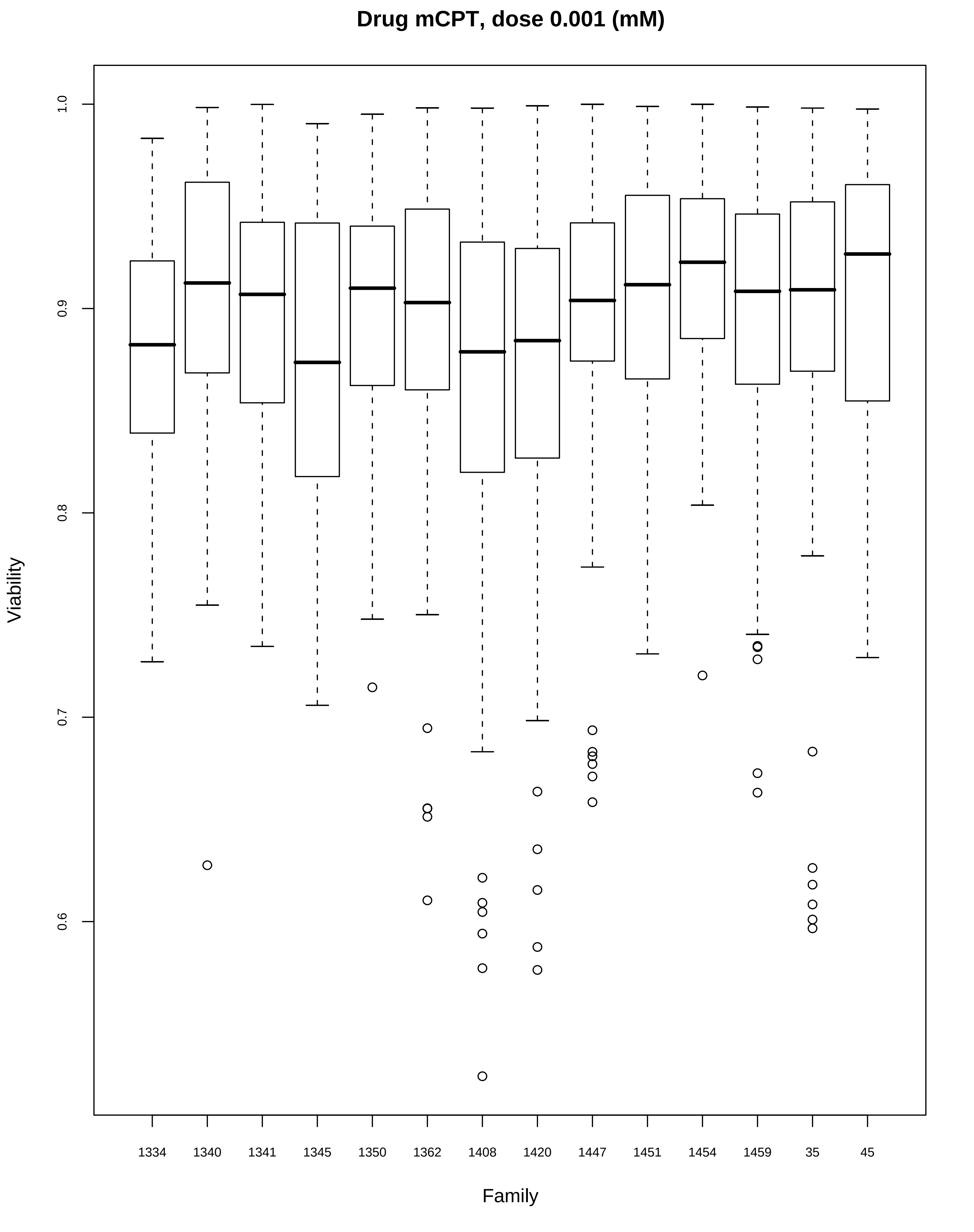
Drug mCPT, dose 0.001 (mM)
Viability
1.0
0.9
0.8
0.7
0.6
Family
1334
1340
1341
1345
1350
1362
1408
1420
1447
1451
1454
1459
35
45

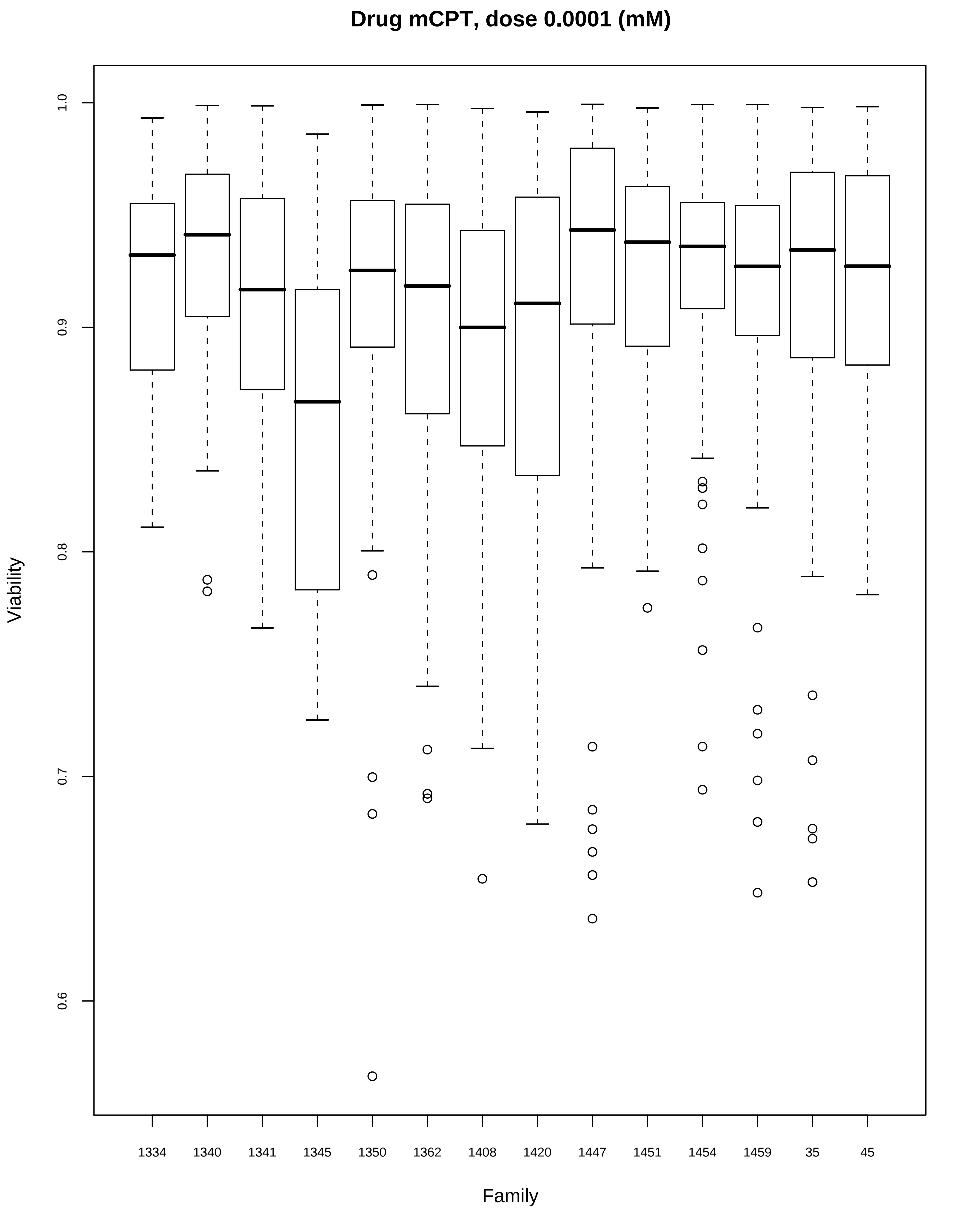
Drug mCPT, dose 0.0001 (mM)
Viability
1.0
0.9
0.8
0.7
0.6
Family
1334
1340
1341
1345
1350
1362
1408
1420
1447
1451
1454
1459
35
45
