# Supplementary material for: Identification and Replication of Loci Involved in Camptothecin-Induced Cytotoxicity Using CEPH Pedigrees
Source: PLoS One. 2011 May 5;6(5):e17561. doi: 10.1371/journal.pone.0017561 (PMC3088663; doi:10.1371/journal.pone.0017561)
Supplement: Dataset S5 — Genomewide QTL plots for each drug and dose. For each drug and dose, LOD scores are shown across each chromosome. (PDF) [file pone.0017561.s011.pdf]

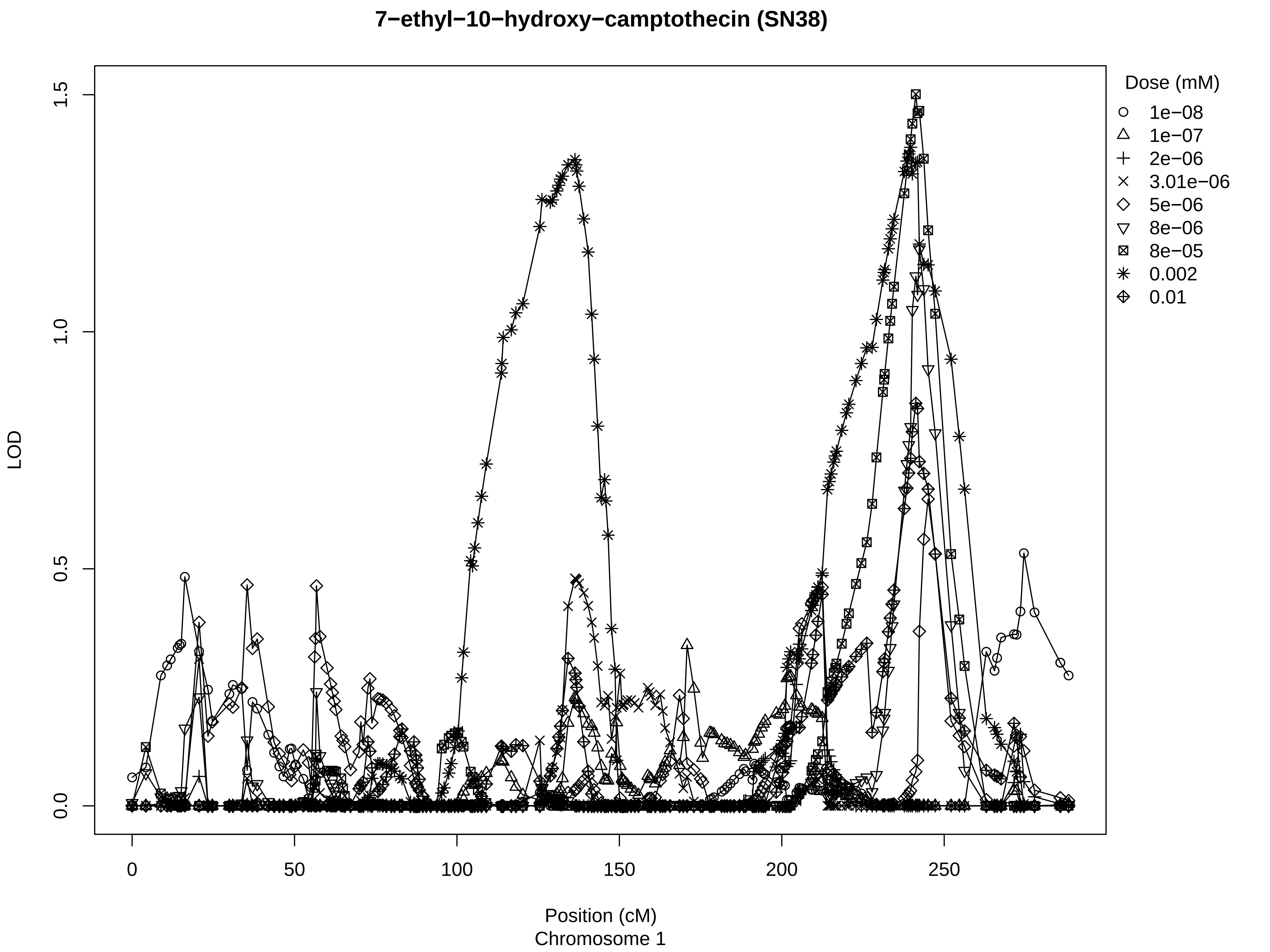

7−ethyl−10−hydroxy−camptothecin (SN38)
LOD
0.0
0.5
1.0
1.5
0
50
100
150
200
250
Position (cM)
Chromosome 1
Dose (mM)
1e−08
1e−07
2e−06
3.01e−06
5e−06
8e−06
8e−05
0.002
0.01

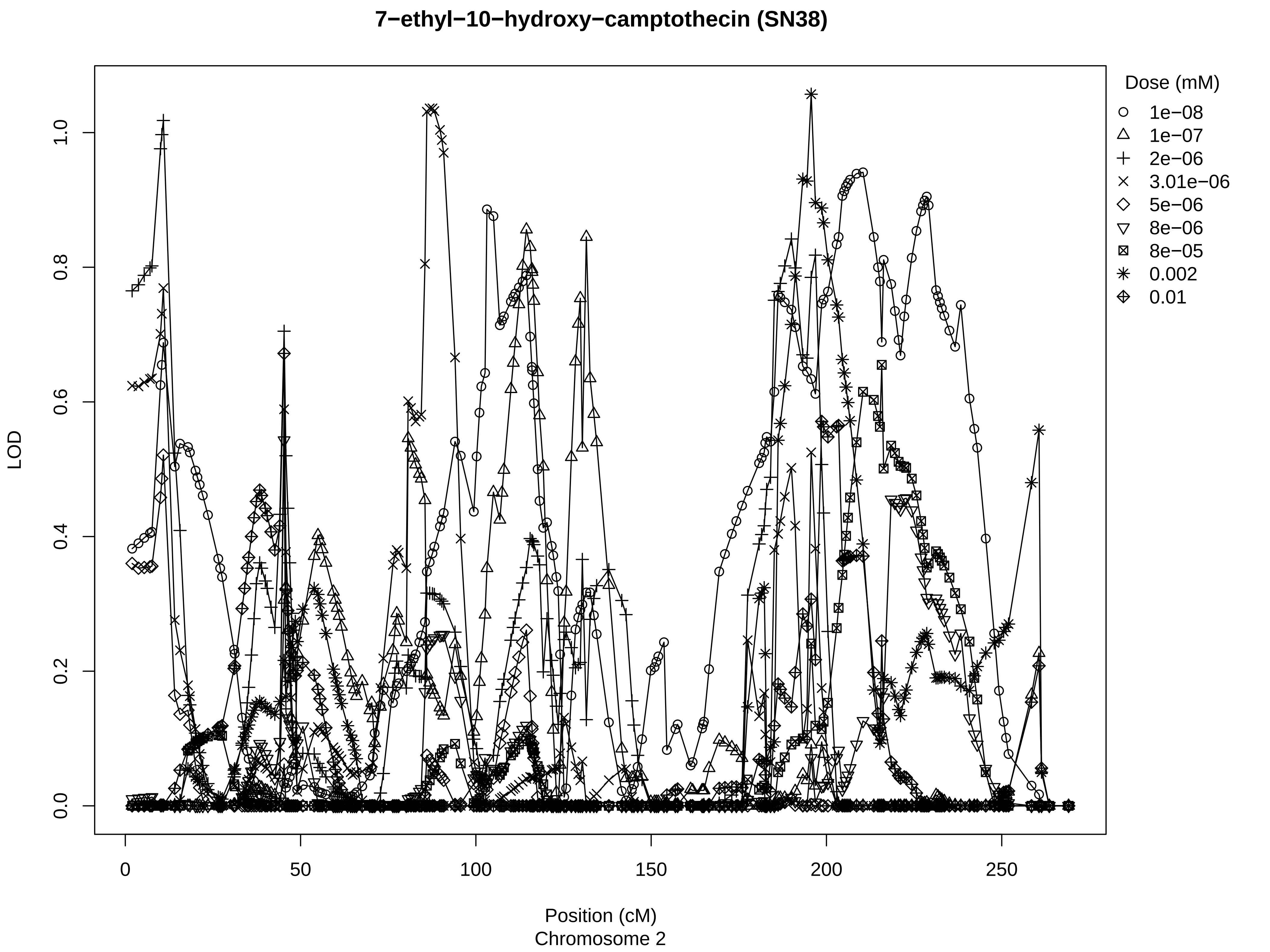
7−ethyl−10−hydroxy−camptothecin (SN38)
LOD
Position (cM)
Chromosome 2
Dose (mM)
1e−08
1e−07
2e−06
3.01e−06
5e−06
8e−06
8e−05
0.002
0.01
0.0
0.2
0.4
0.6
0.8
1.0
0
50
100
150
200
250

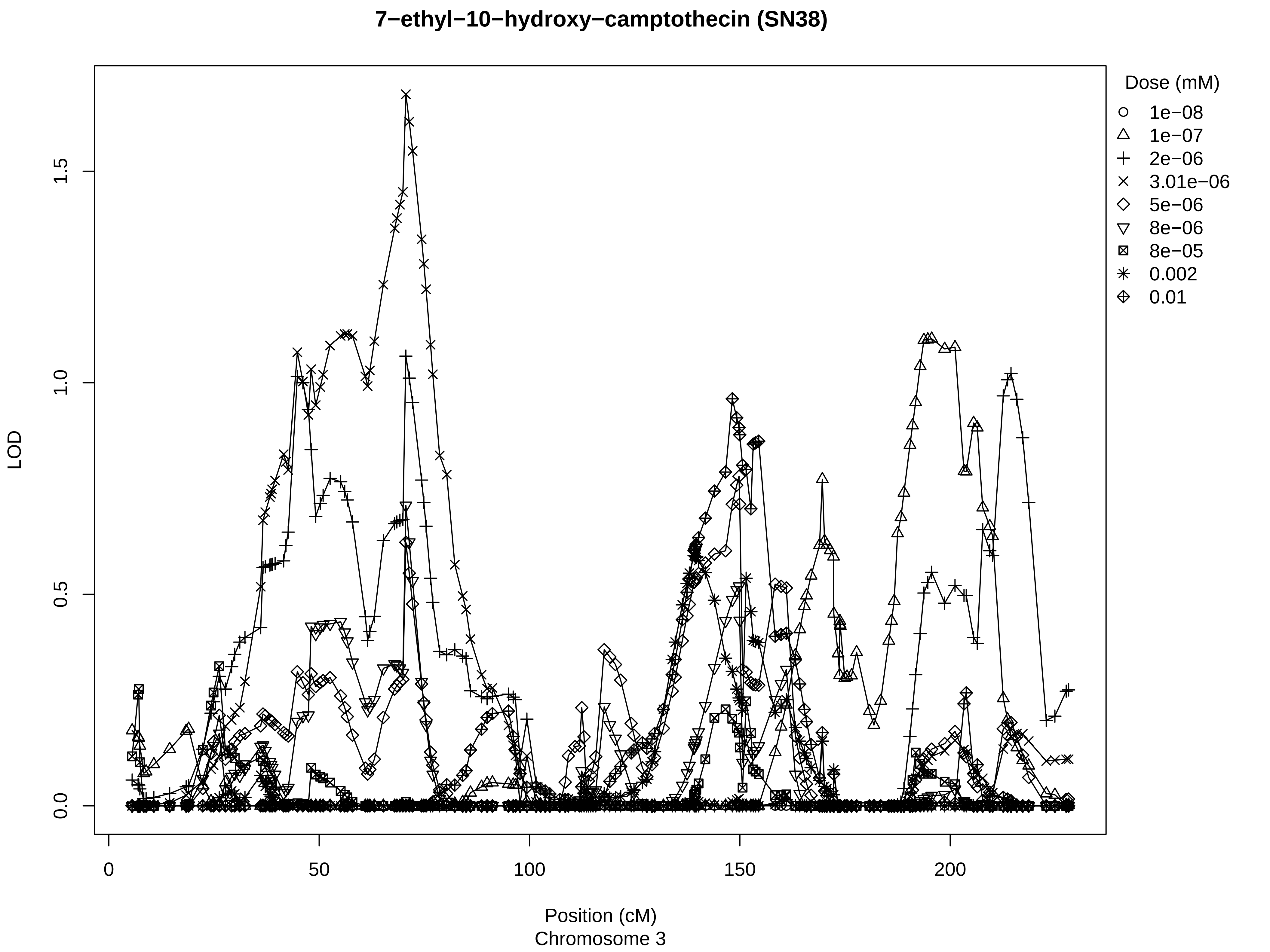

7−ethyl−10−hydroxy−camptothecin (SN38)
LOD
Position (cM)
Chromosome 3
Dose (mM)
1e−08
1e−07
2e−06
3.01e−06
5e−06
8e−06
8e−05
0.002
0.01
0.0
0.5
1.0
1.5
0
50
100
150
200

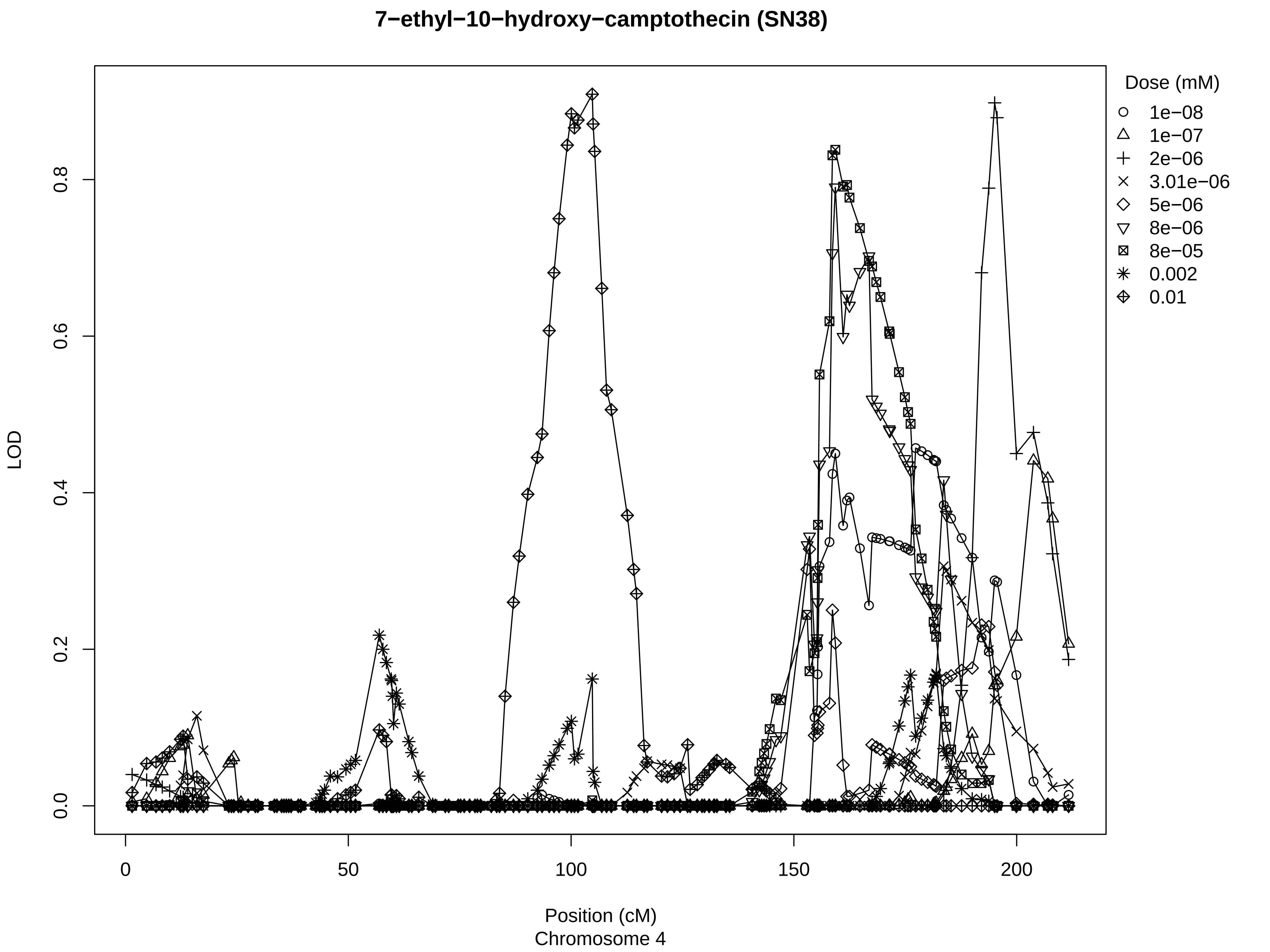
7−ethyl−10−hydroxy−camptothecin (SN38)
LOD
0.0
0.2
0.4
0.6
0.8
0
50
100
150
200
Position (cM)
Chromosome 4
Dose (mM)
1e−08
1e−07
2e−06
3.01e−06
5e−06
8e−06
8e−05
0.002
0.01

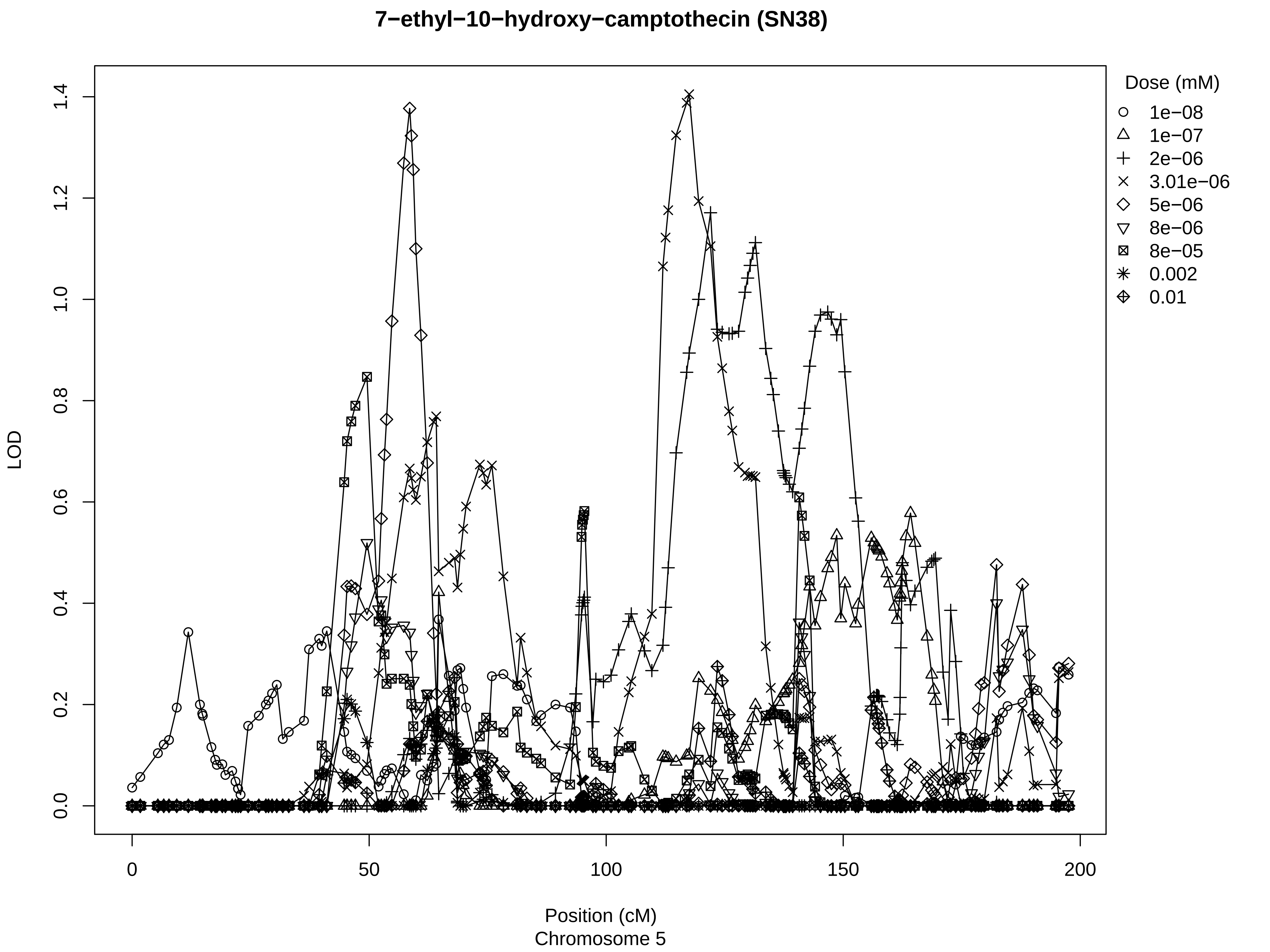

7−ethyl−10−hydroxy−camptothecin (SN38)
LOD
0.0
0.2
0.4
0.6
0.8
1.0
1.2
1.4
0
50
100
150
200
Position (cM)
Chromosome 5
Dose (mM)
1e−08
1e−07
2e−06
3.01e−06
5e−06
8e−06
8e−05
0.002
0.01

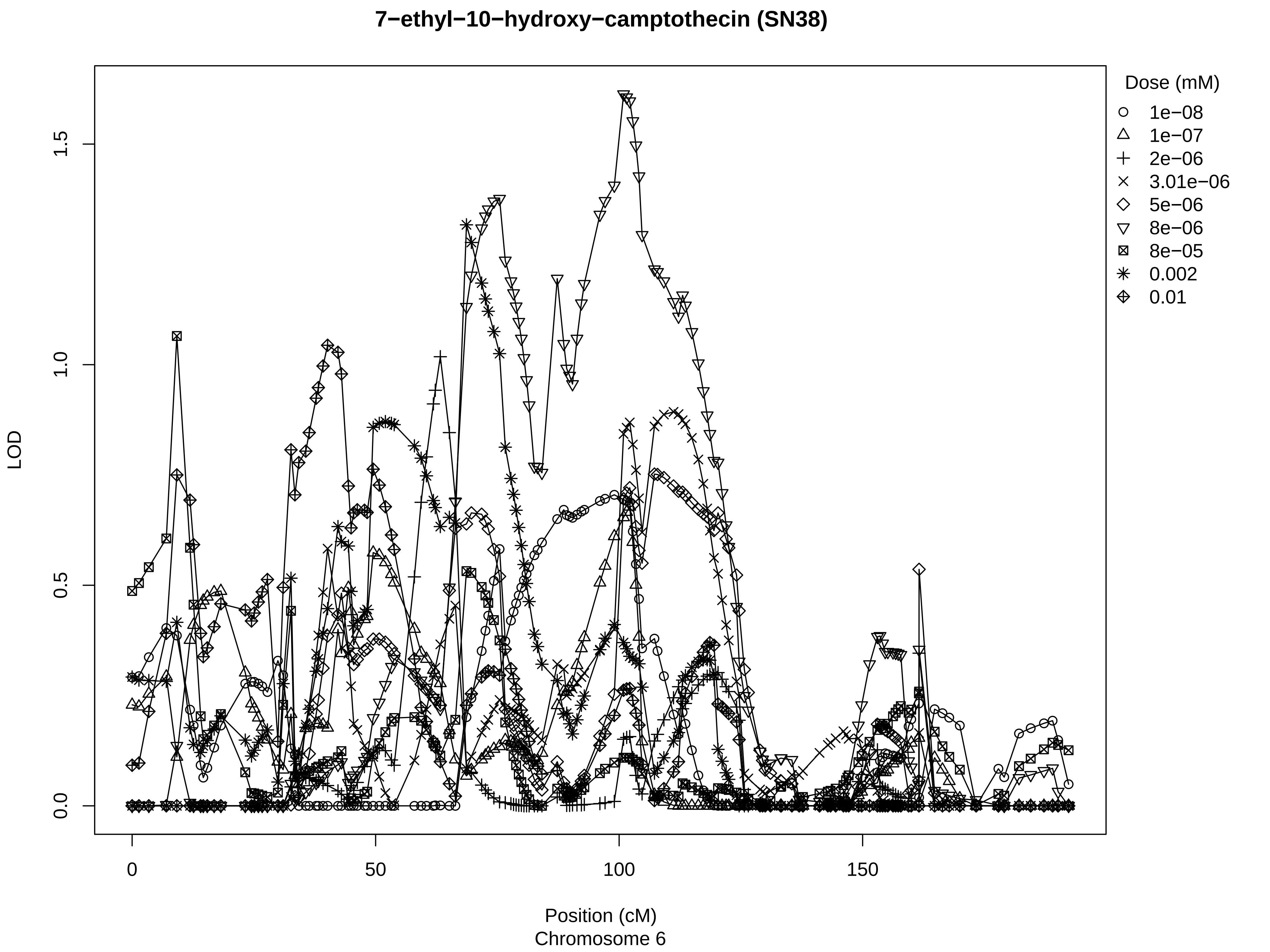
7−ethyl−10−hydroxy−camptothecin (SN38)
LOD
0.0
0.5
1.0
1.5
0
50
100
150
Position (cM)
Chromosome 6
Dose (mM)
1e−08
1e−07
2e−06
3.01e−06
5e−06
8e−06
8e−05
0.002
0.01

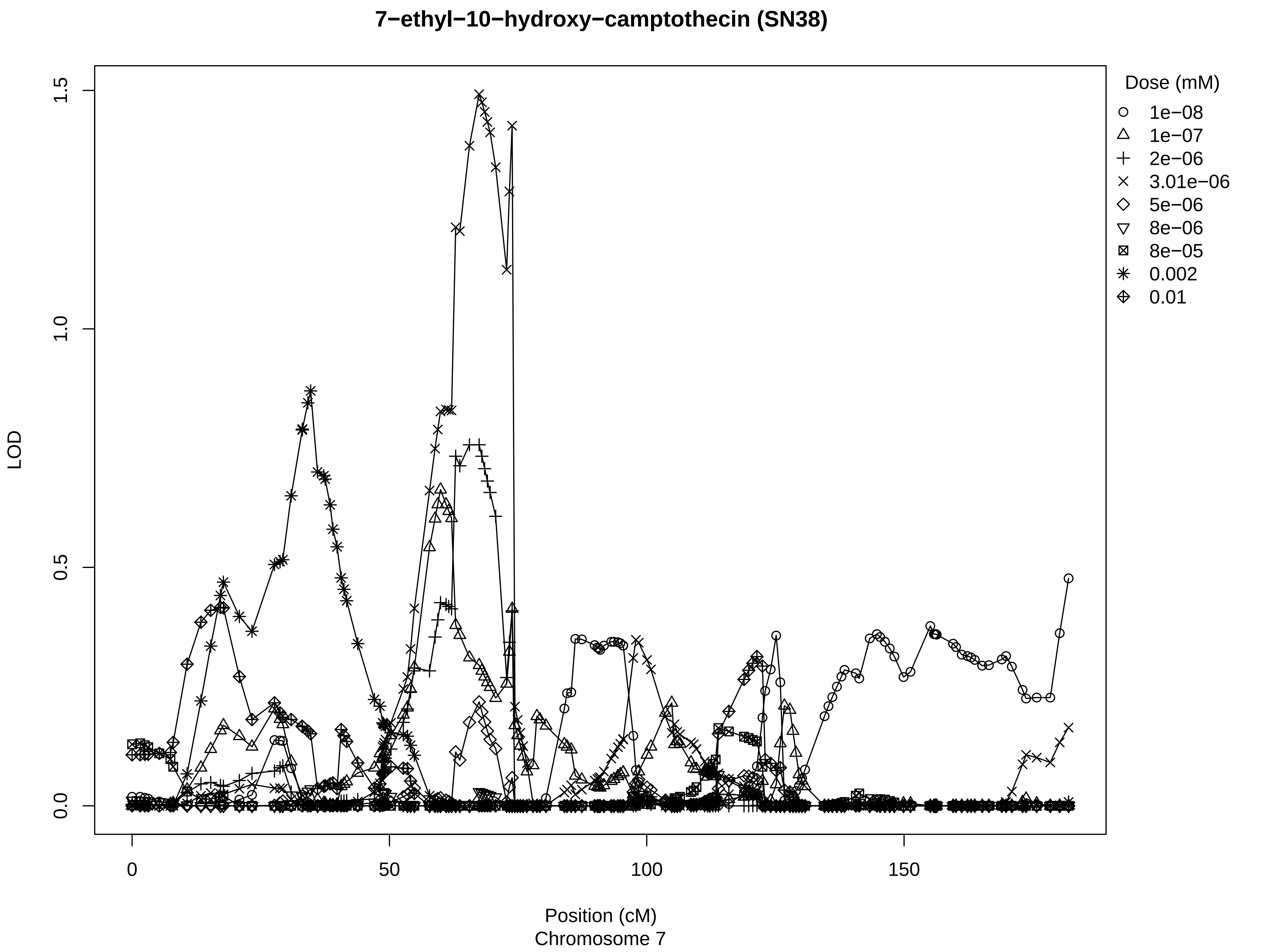

7−ethyl−10−hydroxy−camptothecin (SN38)
LOD
0.0
0.5
1.0
1.5
0
50
100
150
Position (cM)
Chromosome 7
Dose (mM)
1e−08
1e−07
2e−06
3.01e−06
5e−06
8e−06
8e−05
0.002
0.01

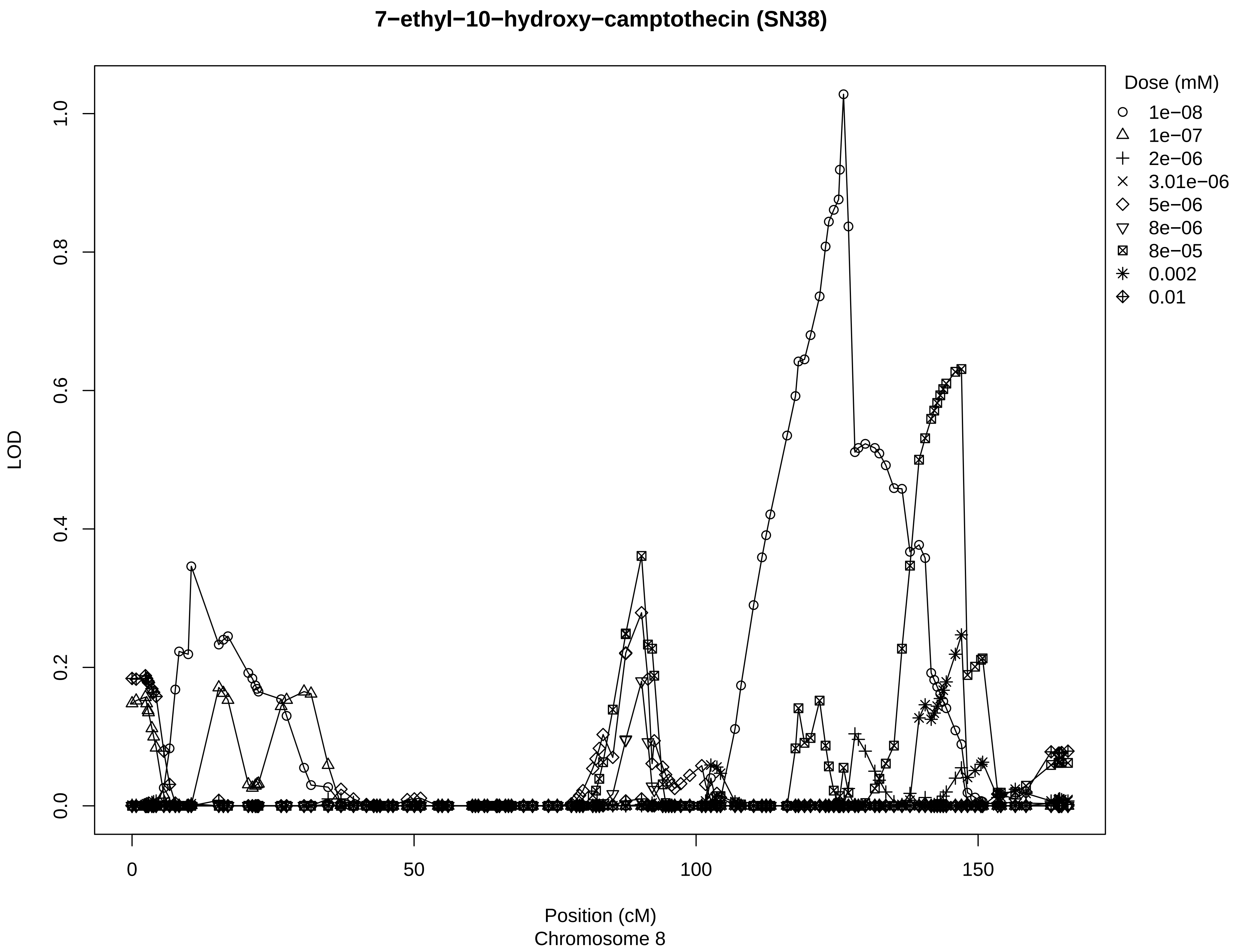

7−ethyl−10−hydroxy−camptothecin (SN38)
LOD
0.0
0.2
0.4
0.6
0.8
1.0
0
50
100
150
Position (cM)
Chromosome 8
Dose (mM)
1e−08
1e−07
2e−06
3.01e−06
5e−06
8e−06
8e−05
0.002
0.01

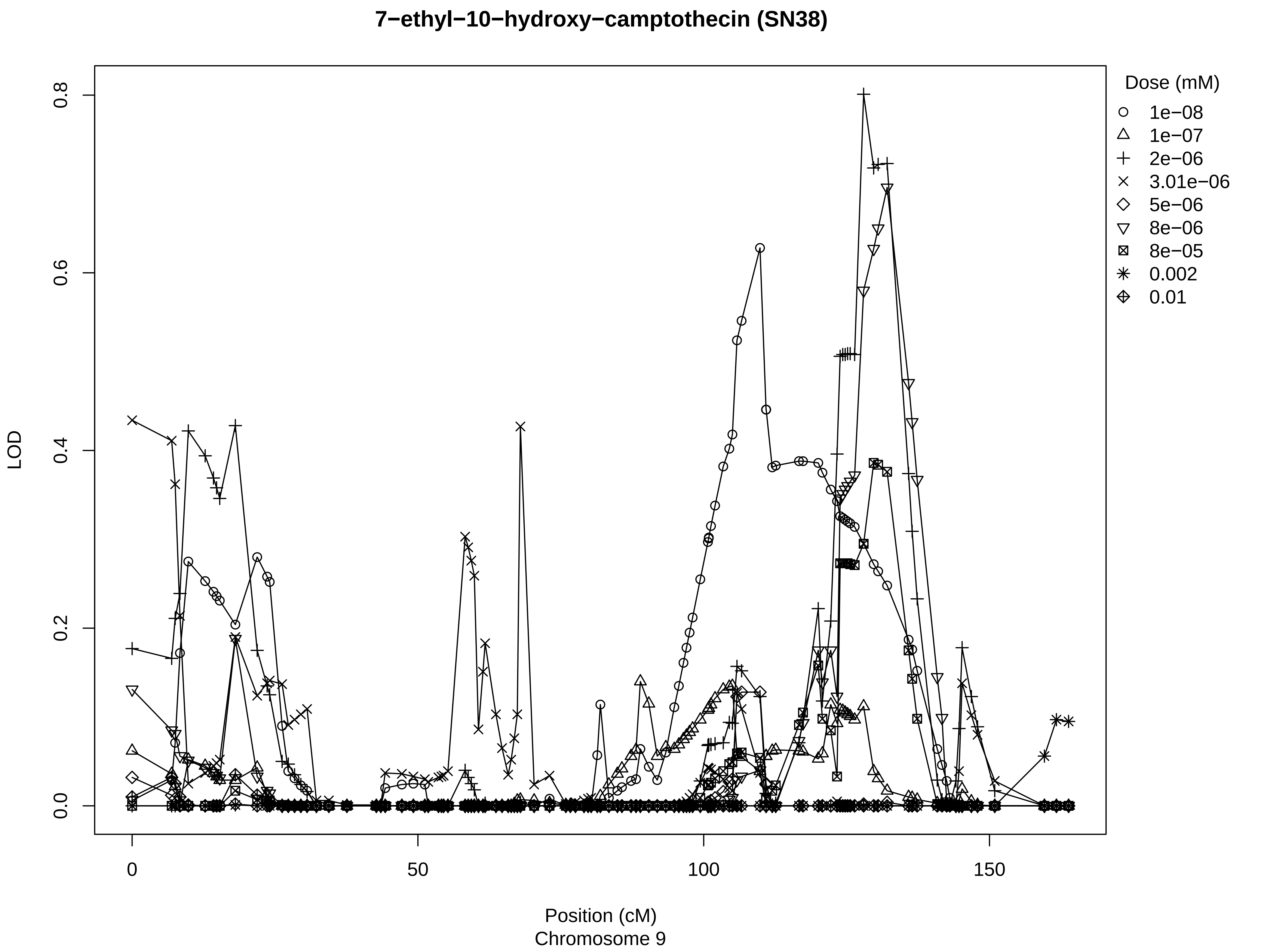
7−ethyl−10−hydroxy−camptothecin (SN38)
LOD
0.0
0.2
0.4
0.6
0.8
0
50
100
150
Position (cM)
Chromosome 9
Dose (mM)
1e−08
1e−07
2e−06
3.01e−06
5e−06
8e−06
8e−05
0.002
0.01

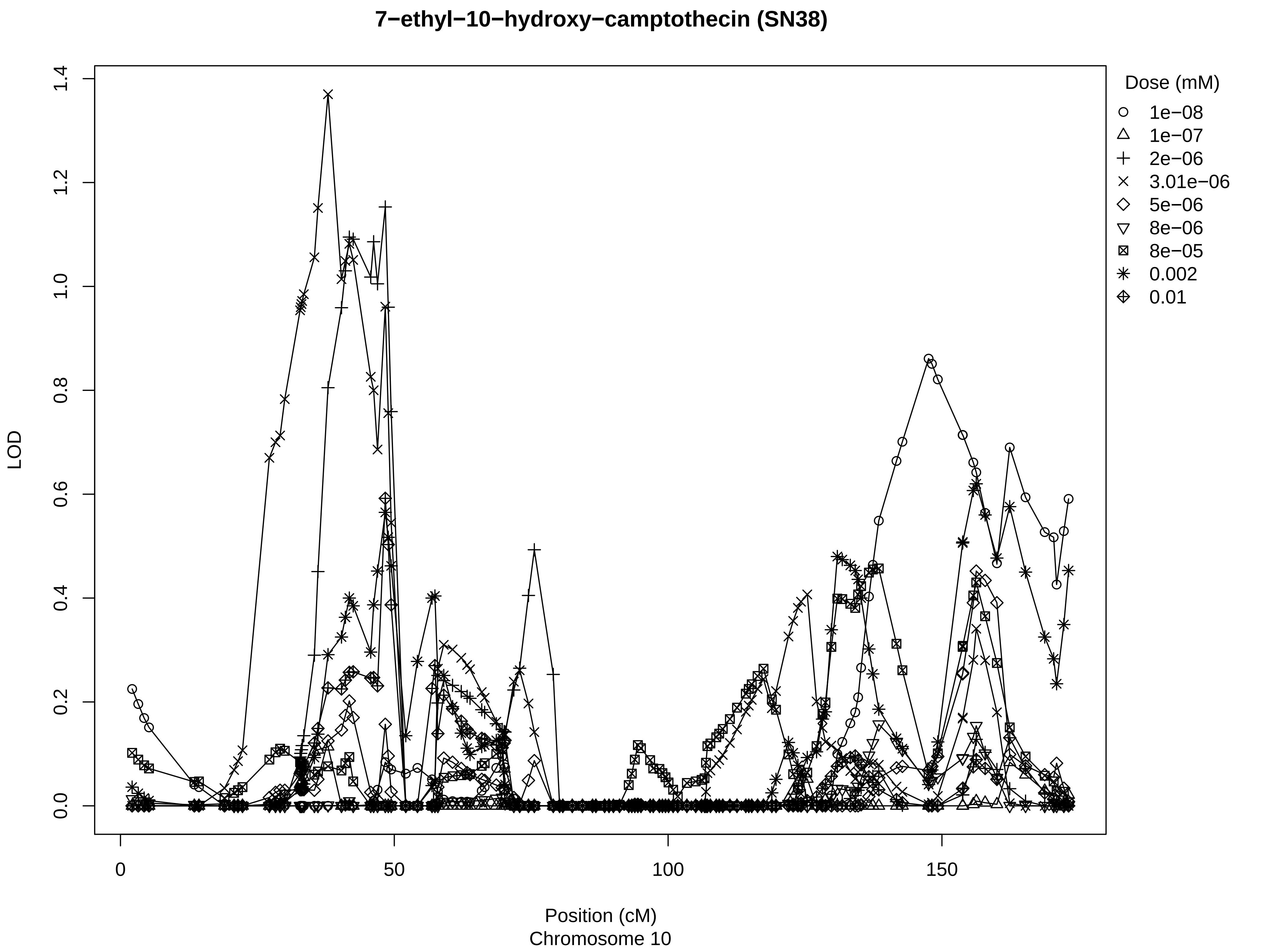

7−ethyl−10−hydroxy−camptothecin (SN38)
LOD
Position (cM)
Chromosome 10
Dose (mM)
1e−08
1e−07
2e−06
3.01e−06
5e−06
8e−06
8e−05
0.002
0.01
0.0
0.2
0.4
0.6
0.8
1.0
1.2
1.4
0
50
100
150

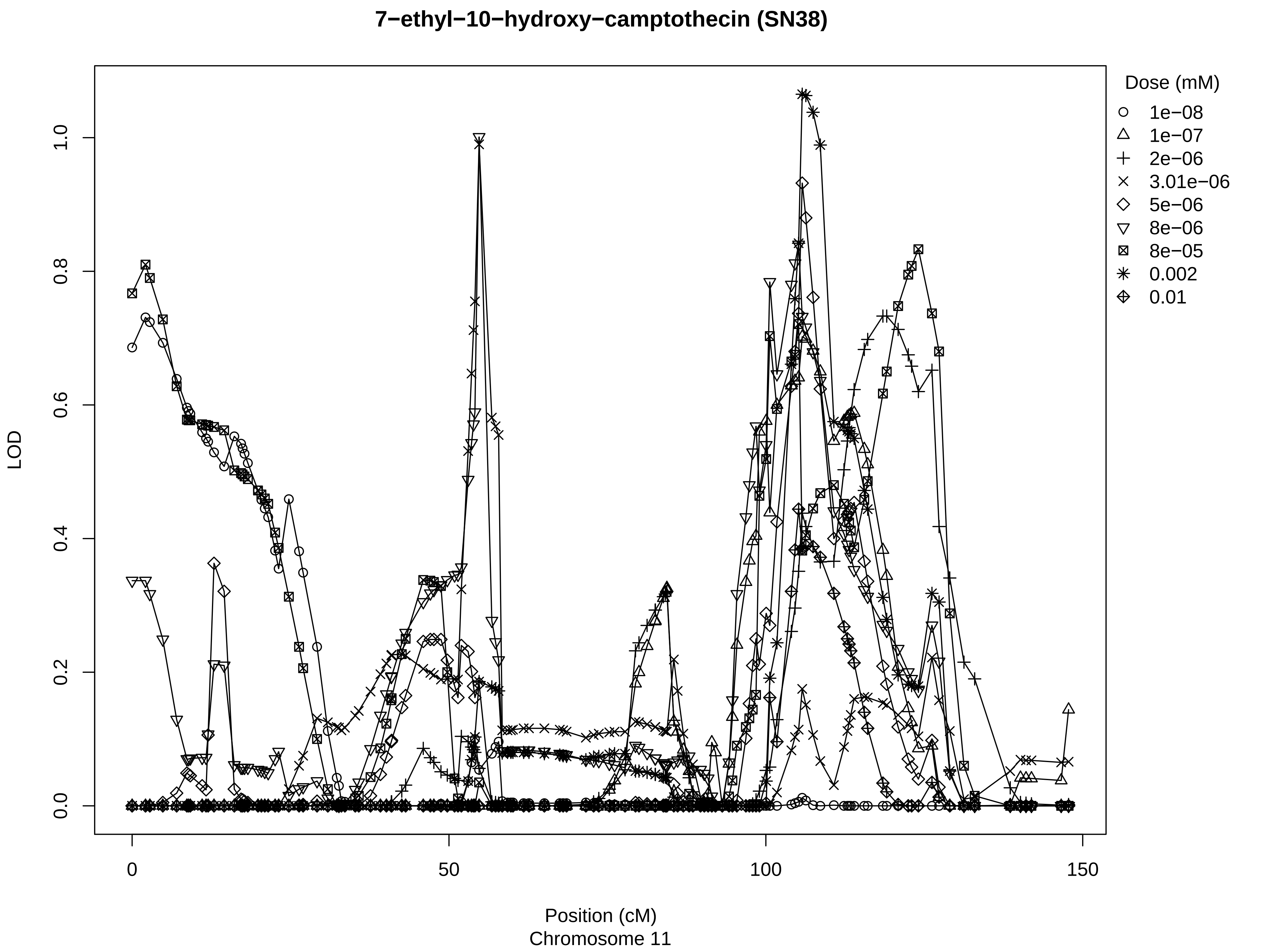
7−ethyl−10−hydroxy−camptothecin (SN38)
LOD
0.0
0.2
0.4
0.6
0.8
1.0
0
50
100
150
Position (cM)
Chromosome 11
Dose (mM)
1e−08
1e−07
2e−06
3.01e−06
5e−06
8e−06
8e−05
0.002
0.01

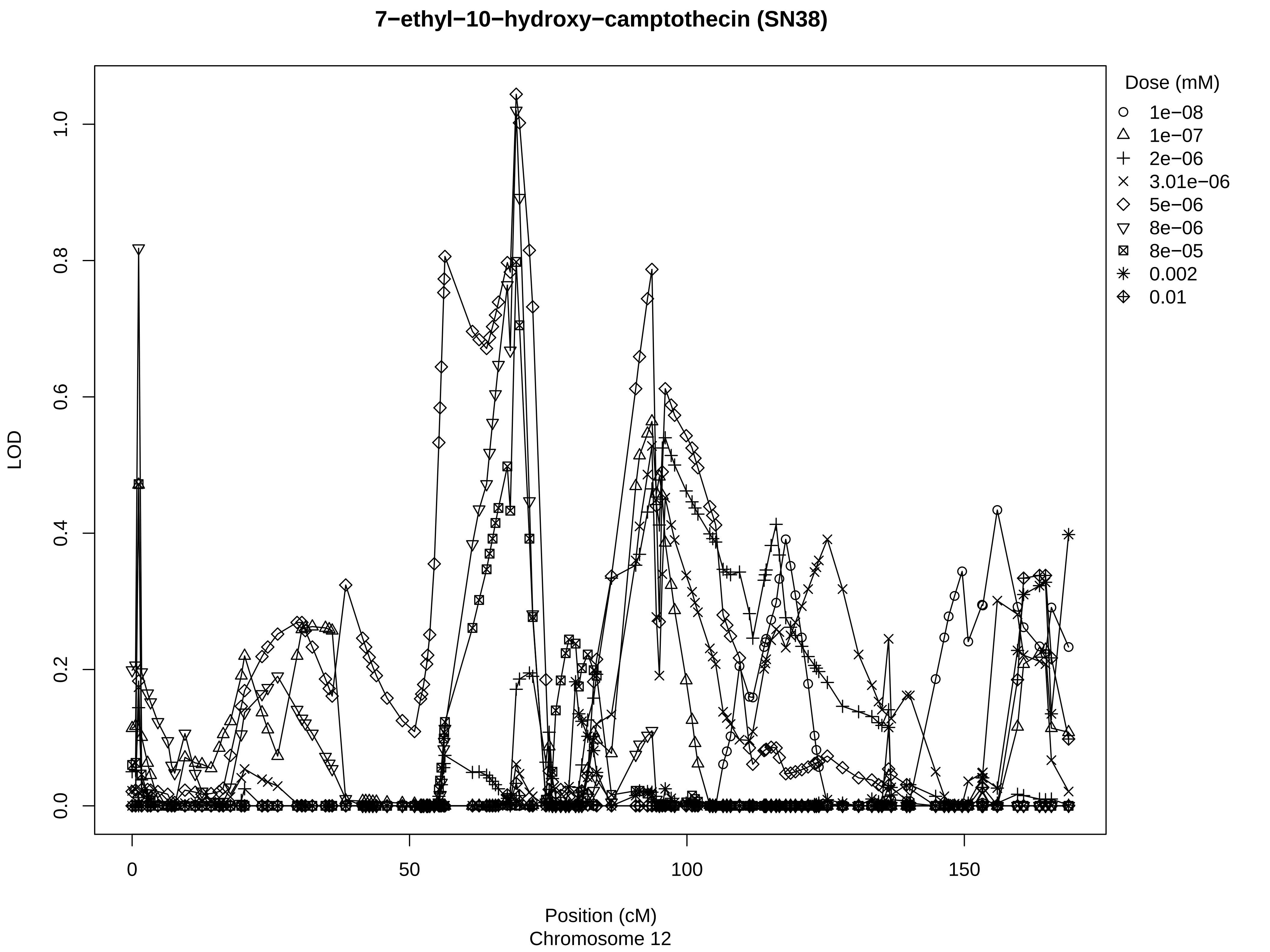

7−ethyl−10−hydroxy−camptothecin (SN38)
LOD
0.0
0.2
0.4
0.6
0.8
1.0
0
50
100
150
Position (cM)
Chromosome 12
Dose (mM)
1e−08
1e−07
2e−06
3.01e−06
5e−06
8e−06
8e−05
0.002
0.01

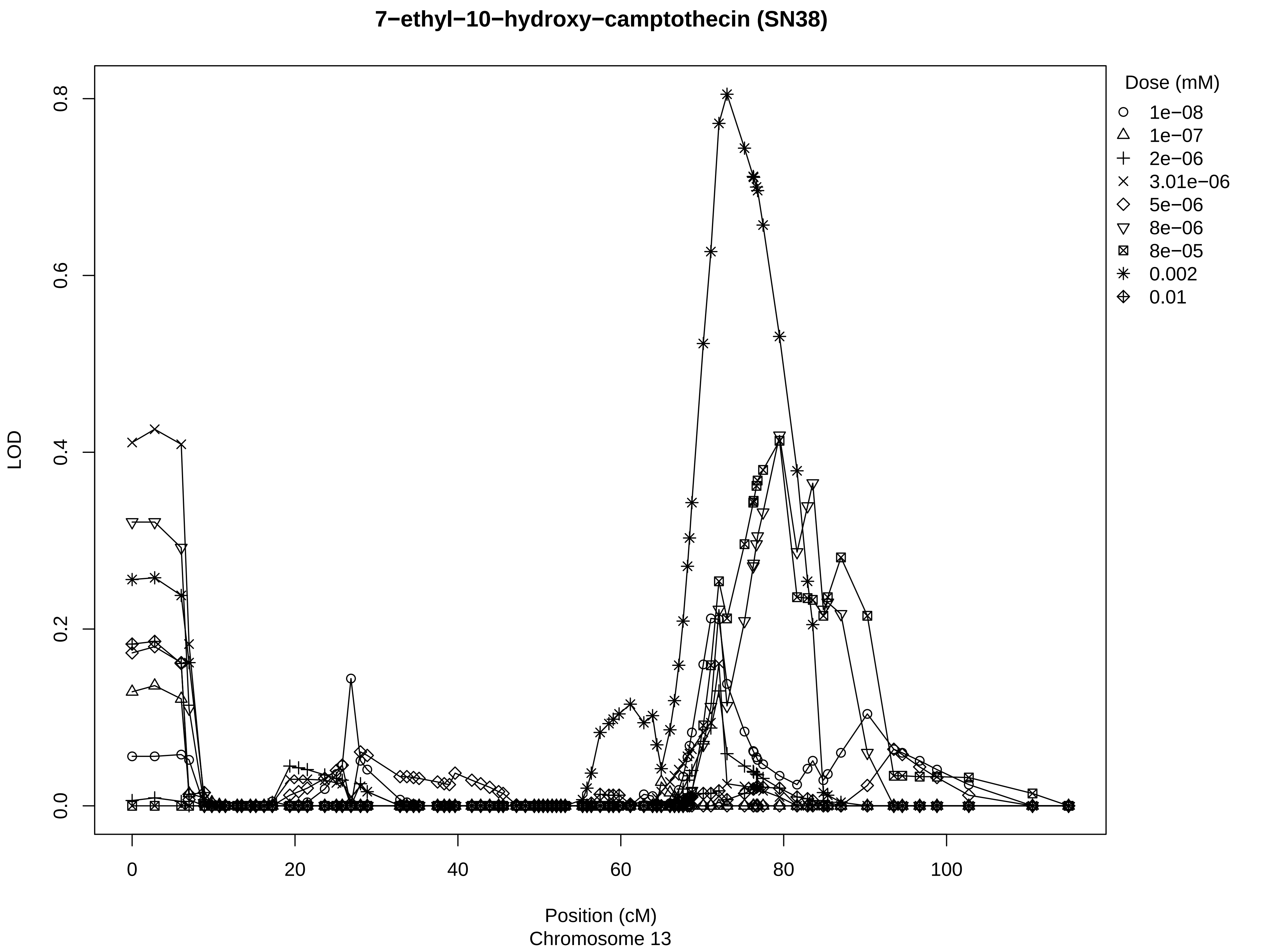

7−ethyl−10−hydroxy−camptothecin (SN38)
LOD
0.0
0.2
0.4
0.6
0.8
0
20
40
60
80
100
Position (cM)
Chromosome 13
Dose (mM)
1e−08
1e−07
2e−06
3.01e−06
5e−06
8e−06
8e−05
0.002
0.01

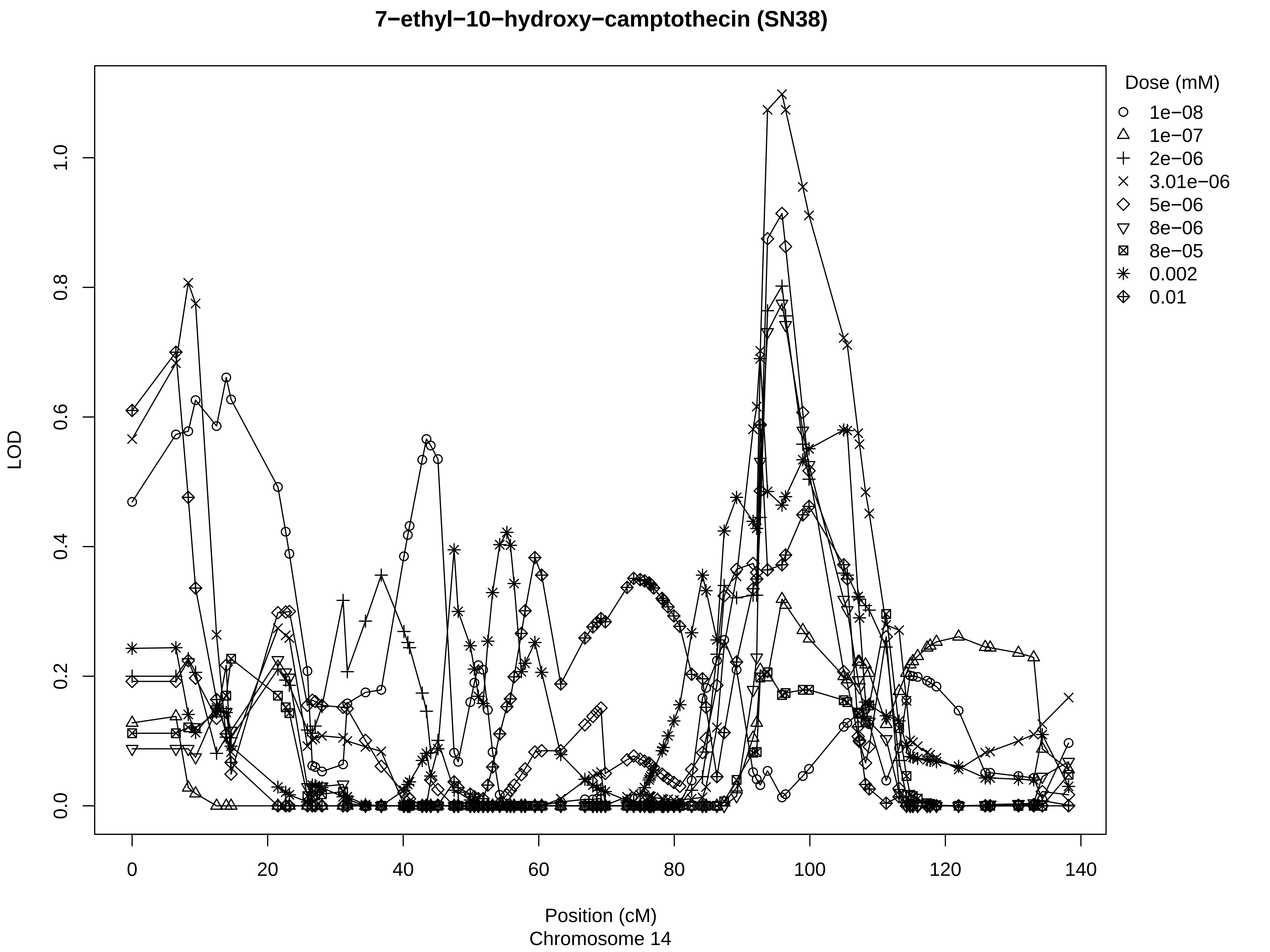

7−ethyl−10−hydroxy−camptothecin (SN38)
Dose (mM)
1e−08
1e−07
2e−06
3.01e−06
5e−06
8e−06
8e−05
0.002
0.01
LOD
0.0
0.2
0.4
0.6
0.8
1.0
0
20
40
60
80
100
120
140
Position (cM)
Chromosome 14

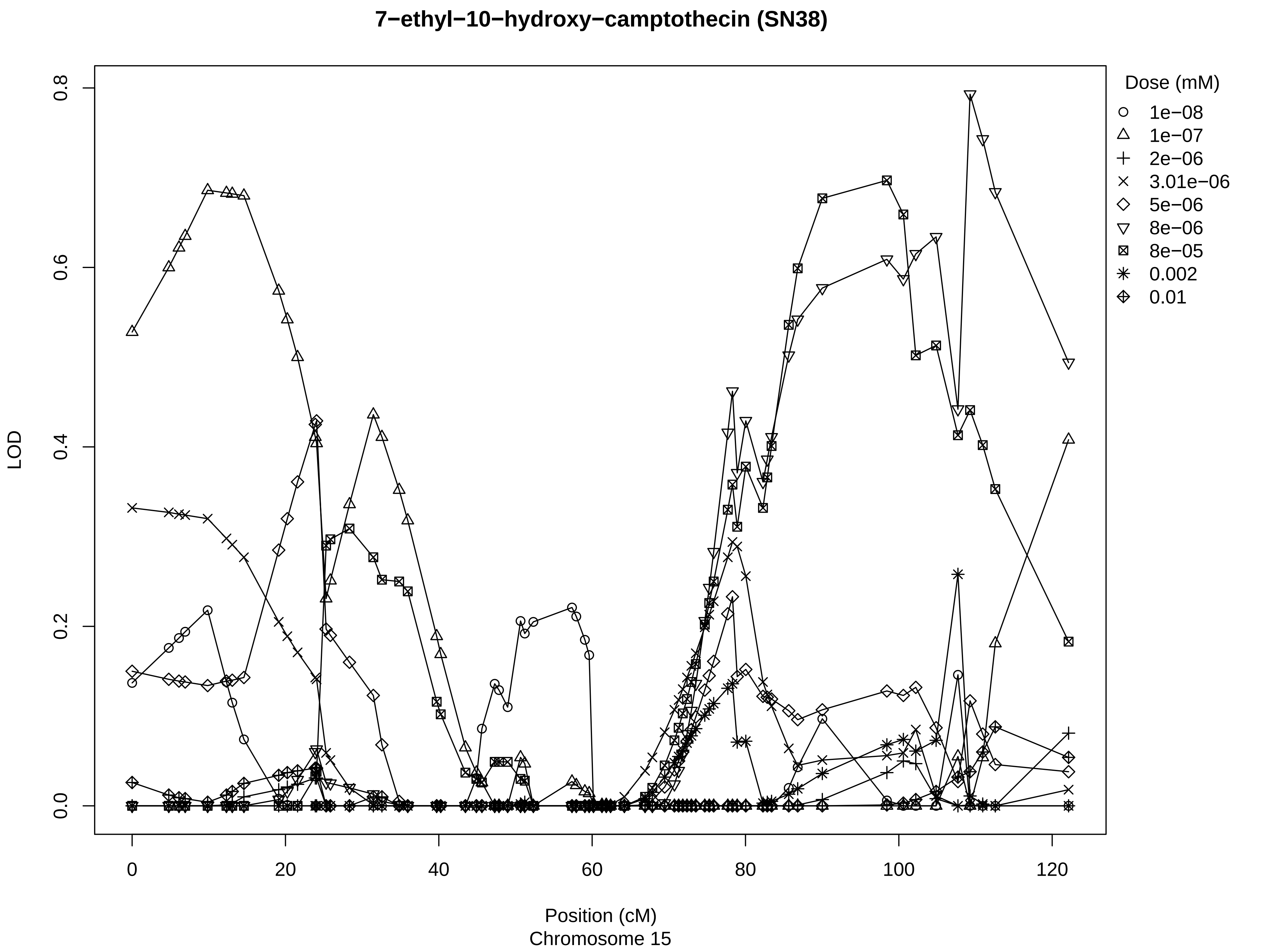

7−ethyl−10−hydroxy−camptothecin (SN38)
LOD
0.0
0.2
0.4
0.6
0.8
0
20
40
60
80
100
120
Position (cM)
Chromosome 15
Dose (mM)
1e−08
1e−07
2e−06
3.01e−06
5e−06
8e−06
8e−05
0.002
0.01

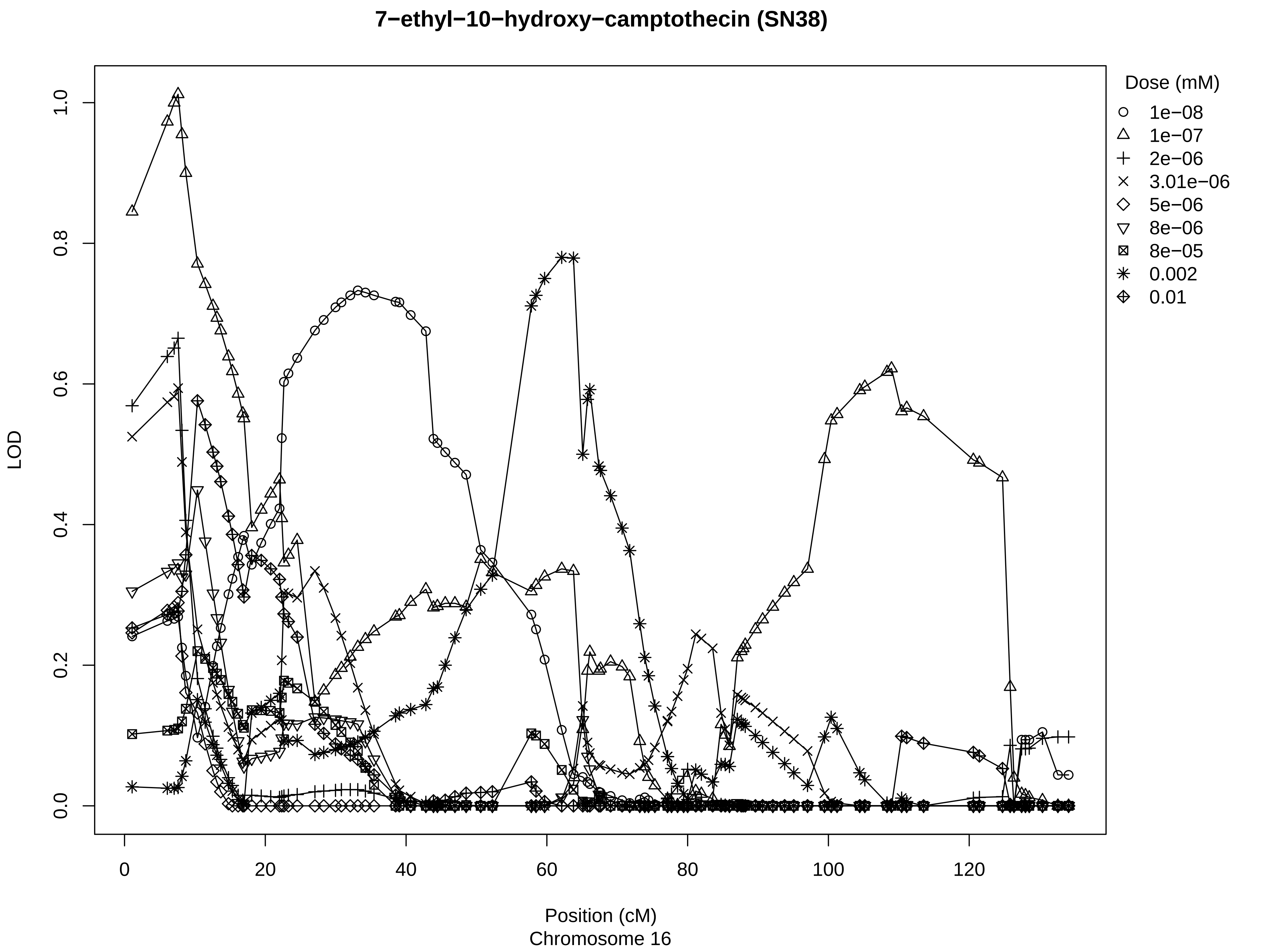
7−ethyl−10−hydroxy−camptothecin (SN38)
LOD
0.0
0.2
0.4
0.6
0.8
1.0
0
20
40
60
80
100
120
Position (cM)
Chromosome 16
Dose (mM)
1e−08
1e−07
2e−06
3.01e−06
5e−06
8e−06
8e−05
0.002
0.01

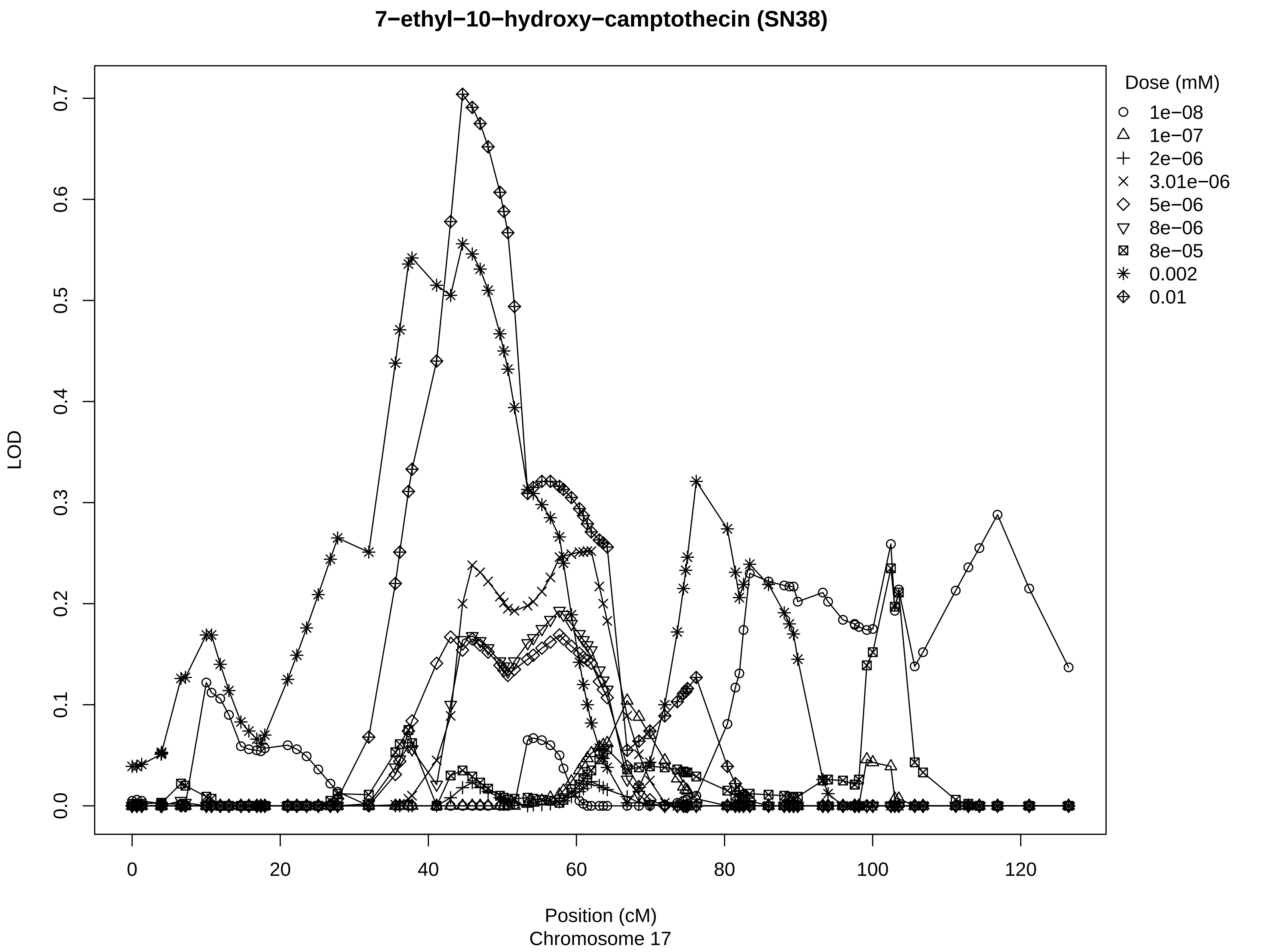

7−ethyl−10−hydroxy−camptothecin (SN38)
LOD
0.0
0.1
0.2
0.3
0.4
0.5
0.6
0.7
Position (cM)
Chromosome 17
0
20
40
60
80
100
120
Dose (mM)
1e−08
1e−07
2e−06
3.01e−06
5e−06
8e−06
8e−05
0.002
0.01

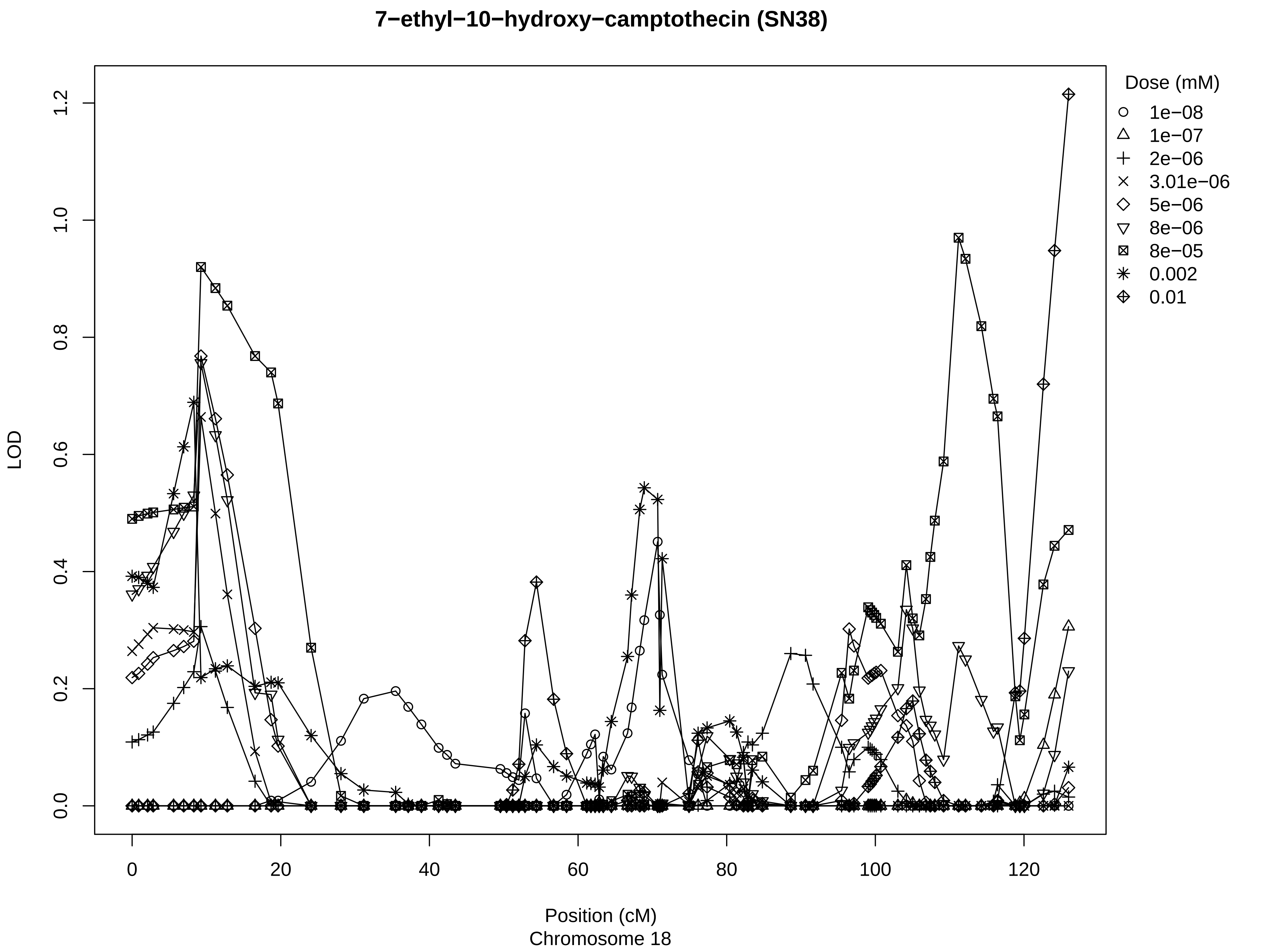

7−ethyl−10−hydroxy−camptothecin (SN38)
LOD
0.0
0.2
0.4
0.6
0.8
1.0
1.2
0
20
40
60
80
100
120
Position (cM)
Chromosome 18
Dose (mM)
1e−08
1e−07
2e−06
3.01e−06
5e−06
8e−06
8e−05
0.002
0.01

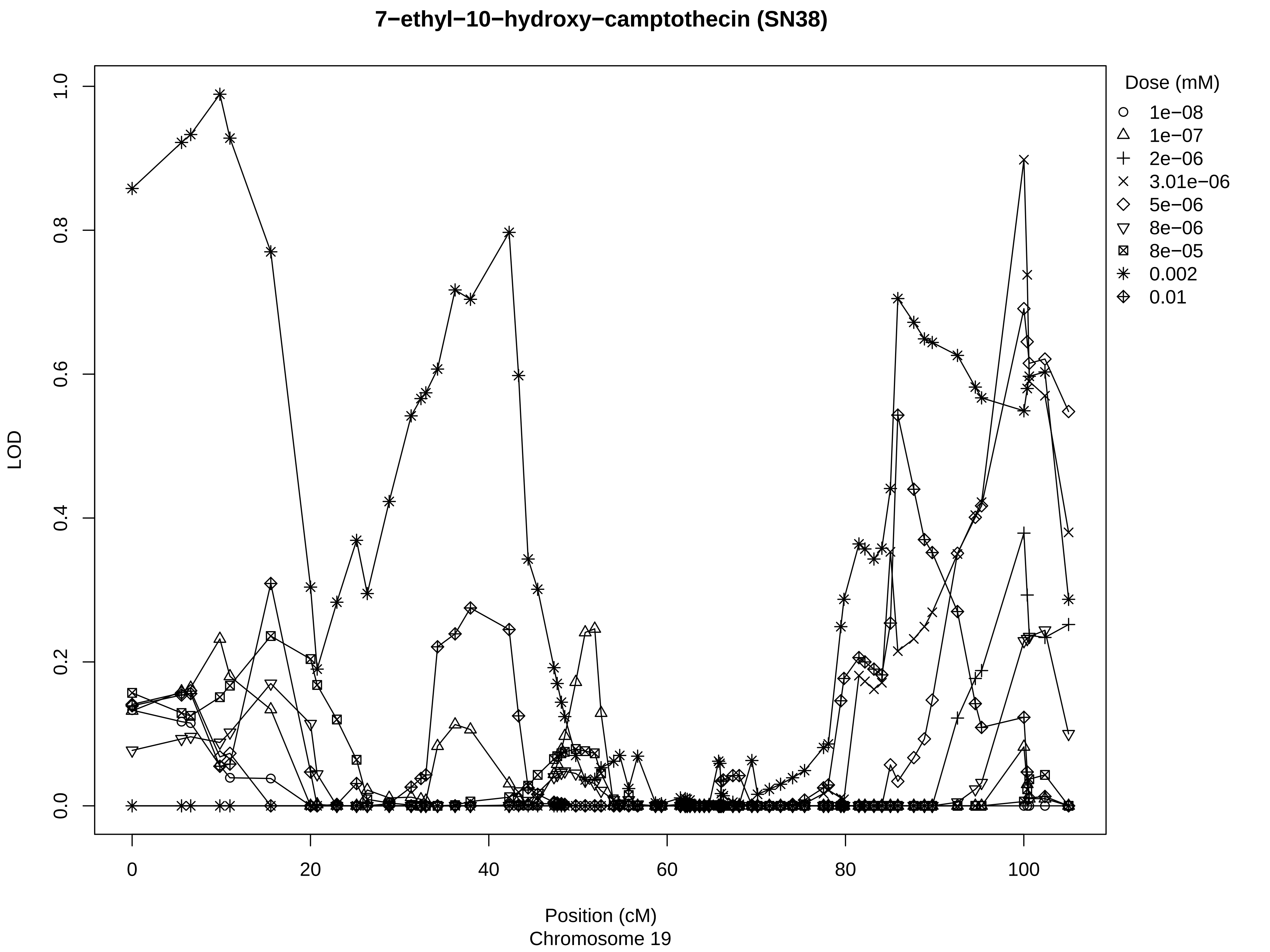
7−ethyl−10−hydroxy−camptothecin (SN38)
LOD
Position (cM)
Chromosome 19
Dose (mM)
1e−08
1e−07
2e−06
3.01e−06
5e−06
8e−06
8e−05
0.002
0.01
0.0
0.2
0.4
0.6
0.8
1.0
0
20
40
60
80
100

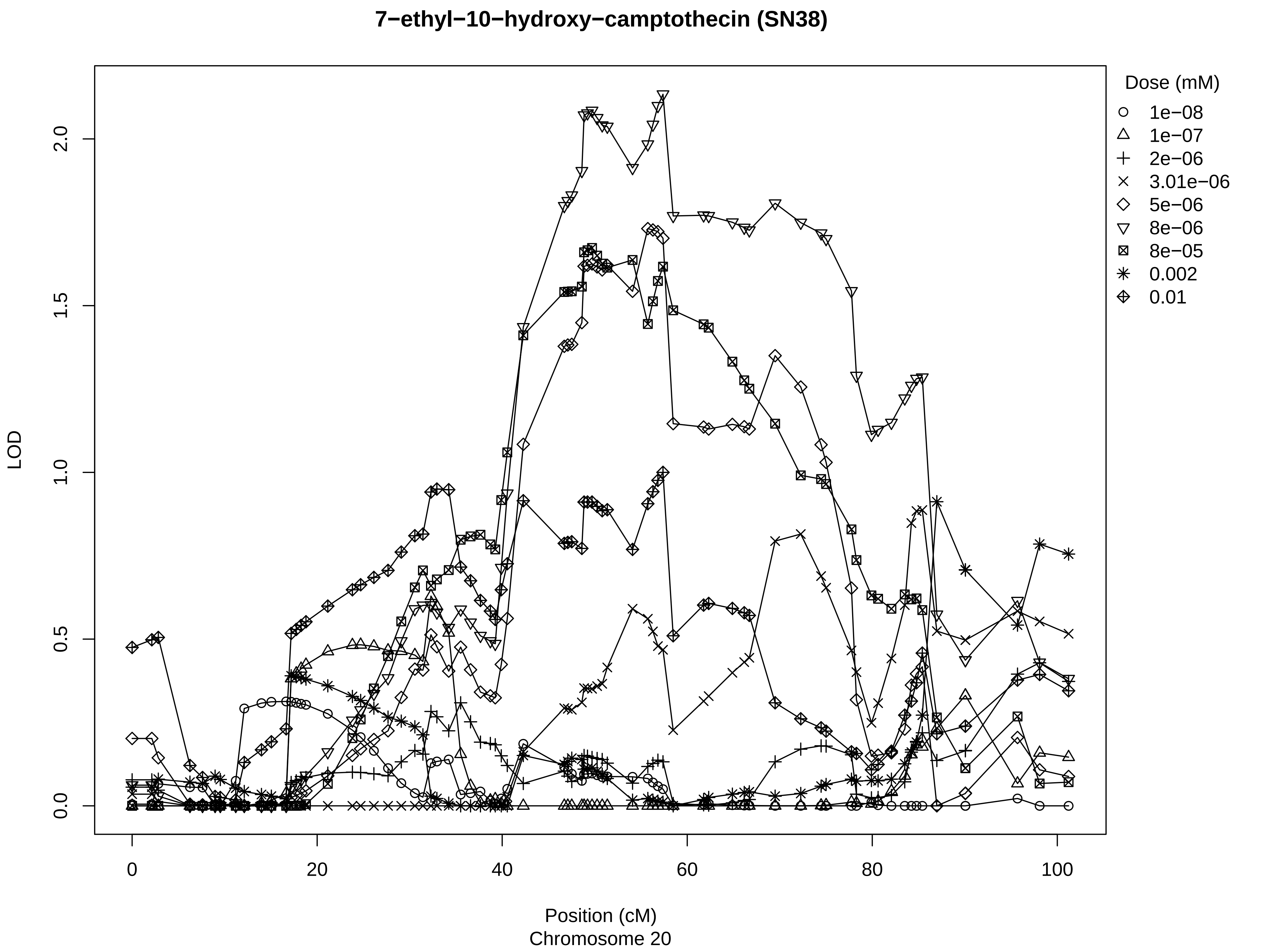
7−ethyl−10−hydroxy−camptothecin (SN38)
LOD
Position (cM)
Chromosome 20
Dose (mM)
1e−08
1e−07
2e−06
3.01e−06
5e−06
8e−06
8e−05
0.002
0.01
0.0
0.5
1.0
1.5
2.0
0
20
40
60
80
100

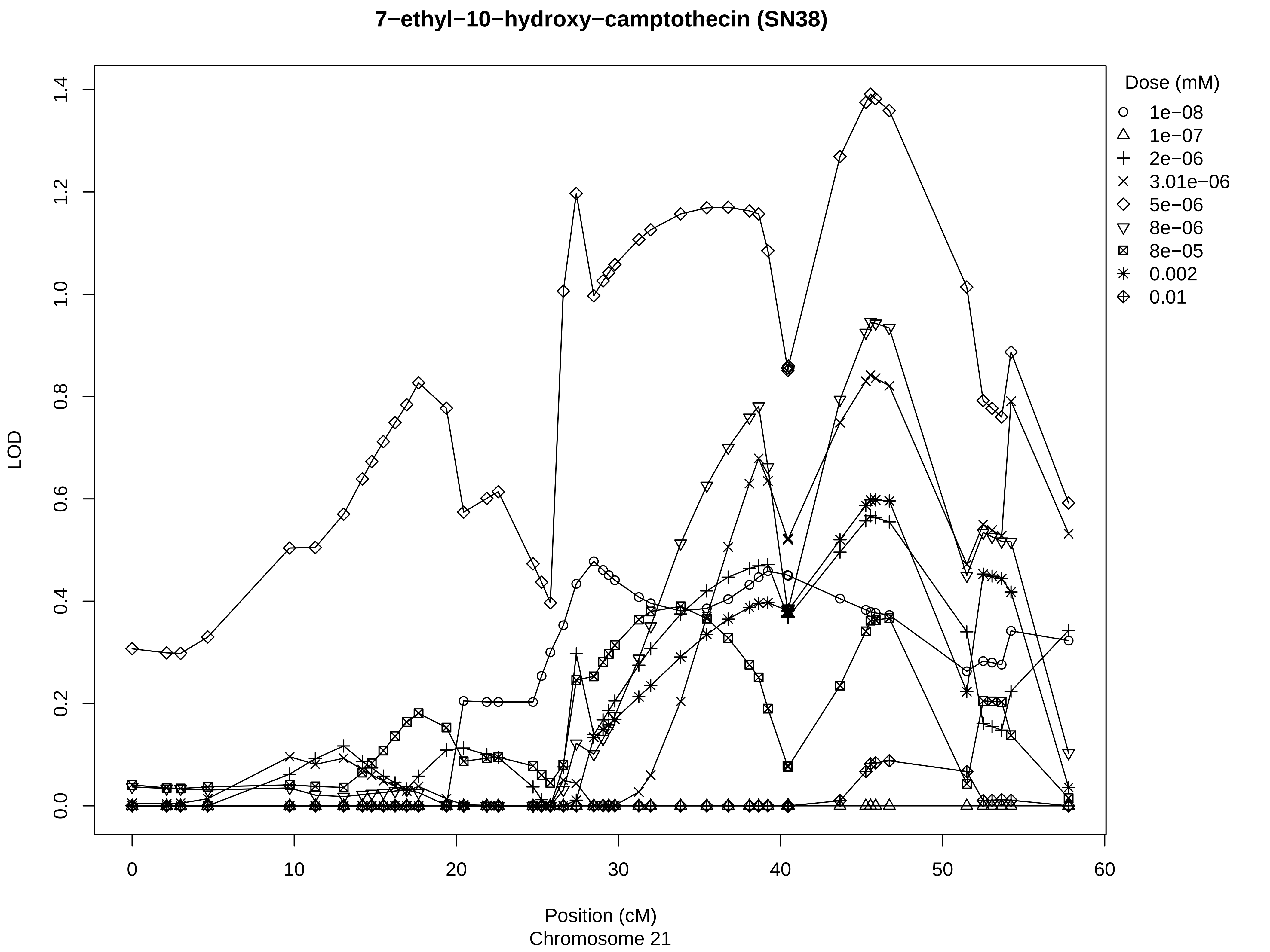
7−ethyl−10−hydroxy−camptothecin (SN38)
Dose (mM)
1e−08
1e−07
2e−06
3.01e−06
5e−06
8e−06
8e−05
0.002
0.01
LOD
Position (cM)
Chromosome 21

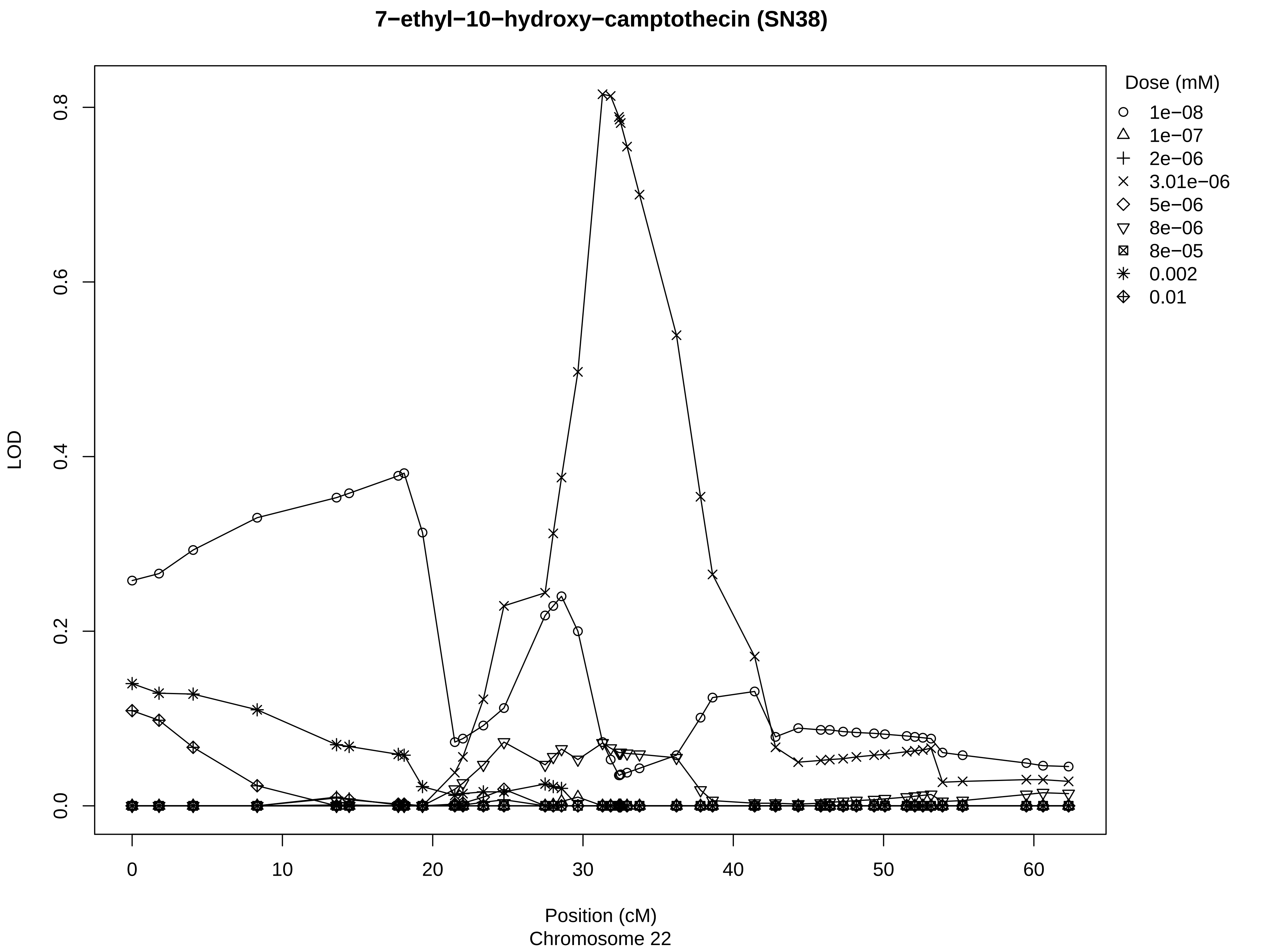

7−ethyl−10−hydroxy−camptothecin (SN38)
LOD
0.0
0.2
0.4
0.6
0.8
0
10
20
30
40
50
60
Position (cM)
Chromosome 22
Dose (mM)
1e−08
1e−07
2e−06
3.01e−06
5e−06
8e−06
8e−05
0.002
0.01

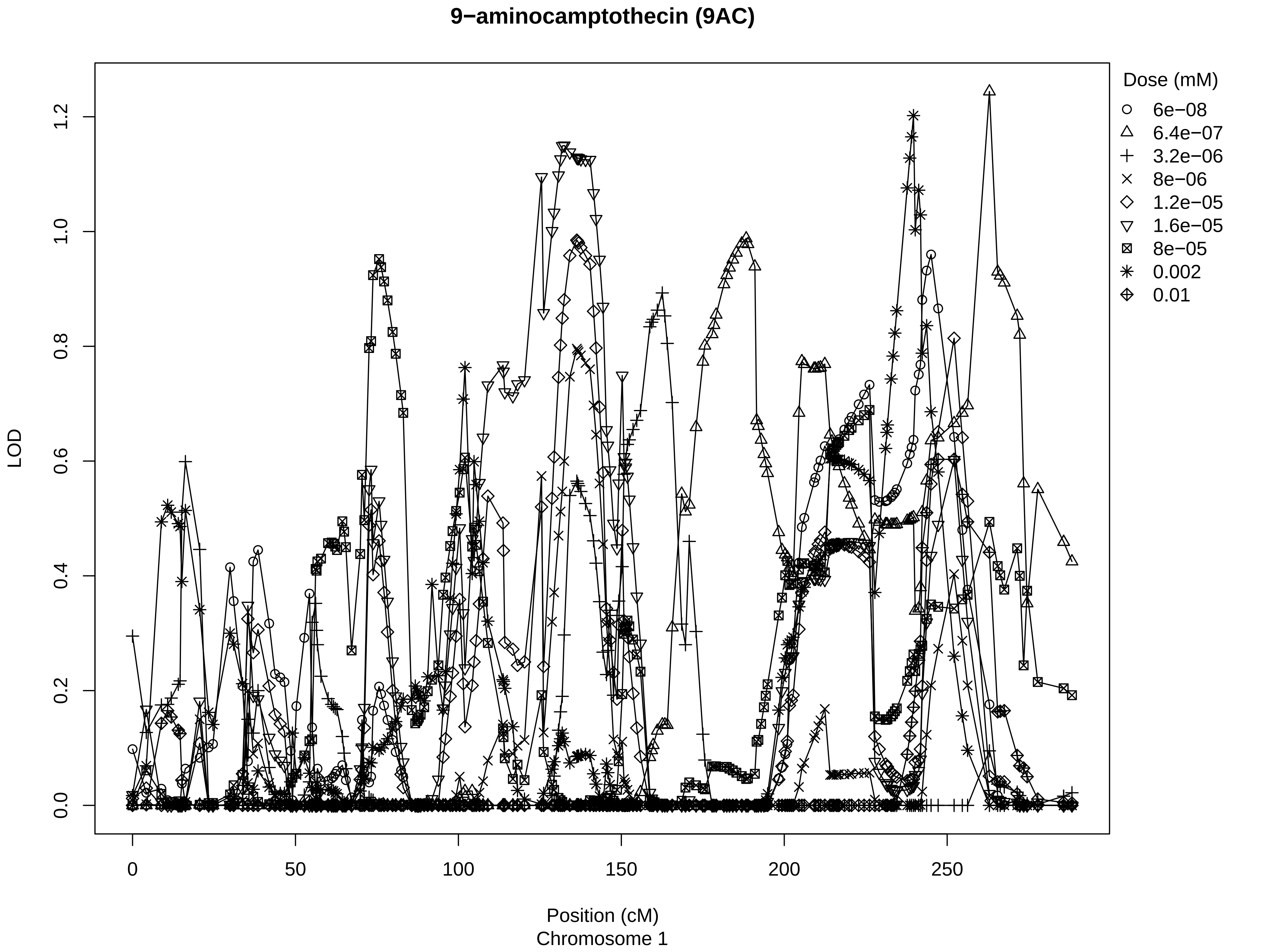
9−aminocamptothecin (9AC)
LOD
0.0
0.2
0.4
0.6
0.8
1.0
1.2
0
50
100
150
200
250
Position (cM)
Chromosome 1
Dose (mM)
6e−08
6.4e−07
3.2e−06
8e−06
1.2e−05
1.6e−05
8e−05
0.002
0.01

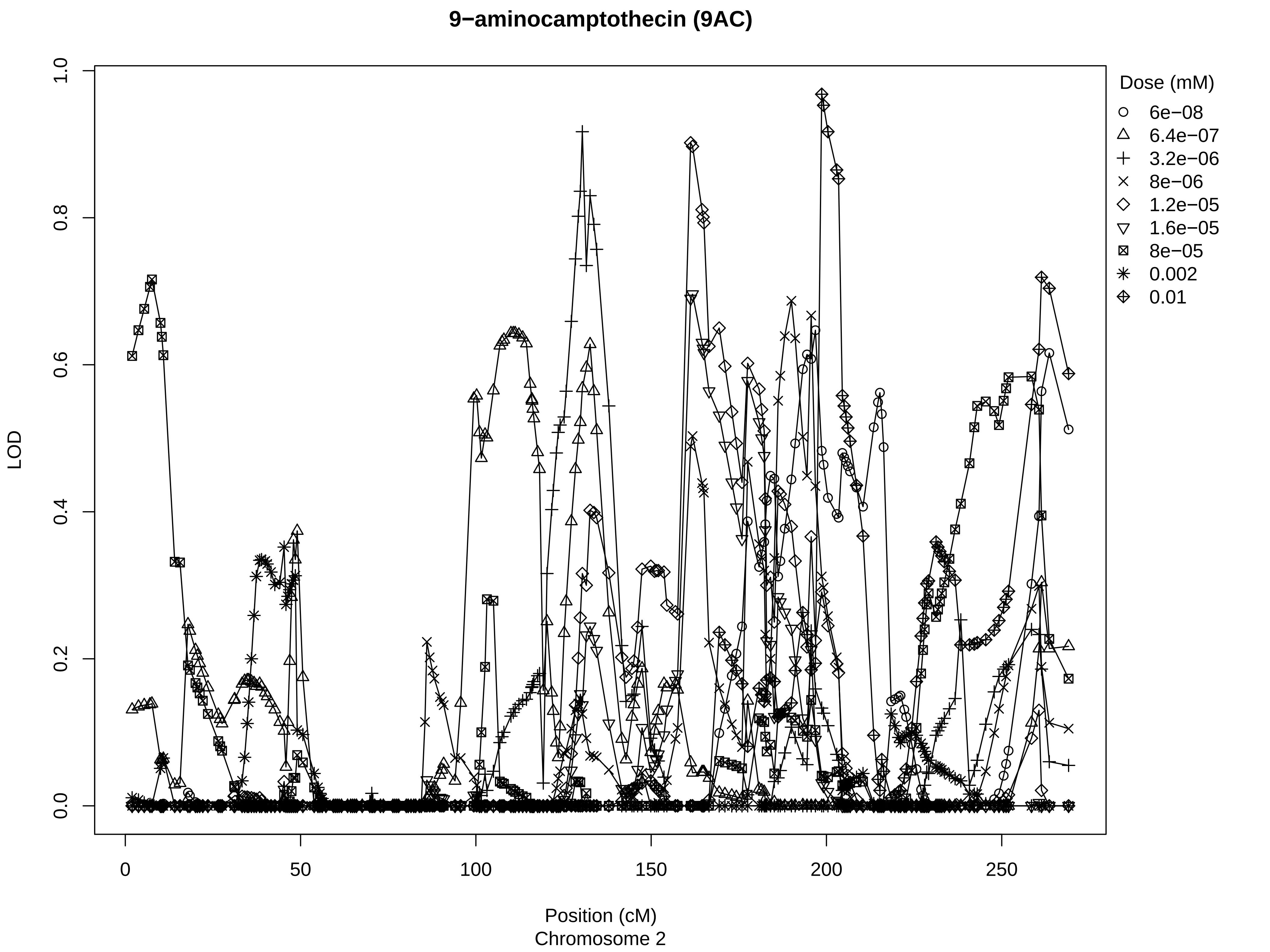
9−aminocamptothecin (9AC)
LOD
0.0
0.2
0.4
0.6
0.8
1.0
0
50
100
150
200
250
Position (cM)
Chromosome 2
Dose (mM)
6e−08
6.4e−07
3.2e−06
8e−06
1.2e−05
1.6e−05
8e−05
0.002
0.01

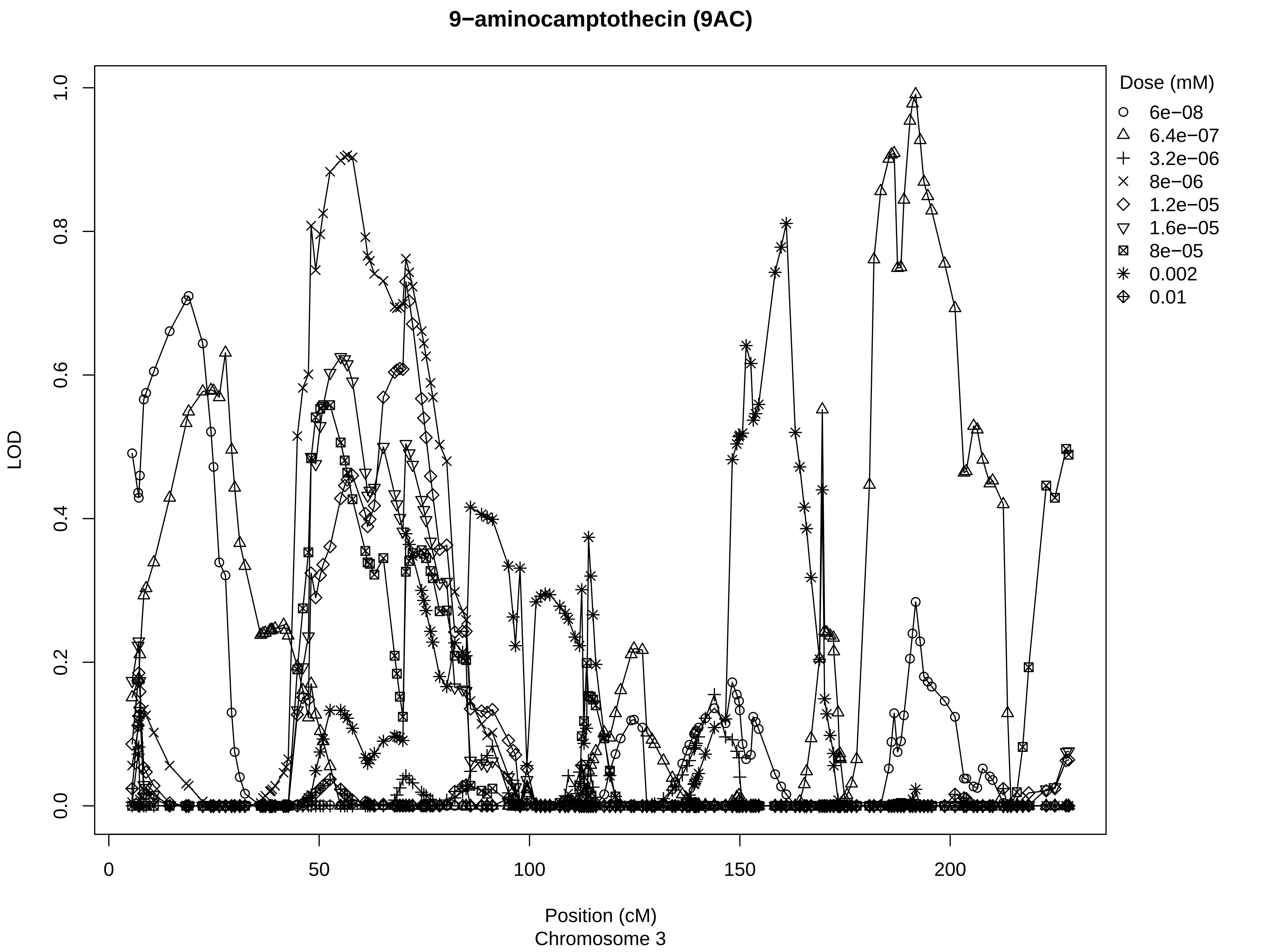

9−aminocamptothecin (9AC)
LOD
0.0
0.2
0.4
0.6
0.8
1.0
0
50
100
150
200
Position (cM)
Chromosome 3
Dose (mM)
6e−08
6.4e−07
3.2e−06
8e−06
1.2e−05
1.6e−05
8e−05
0.002
0.01

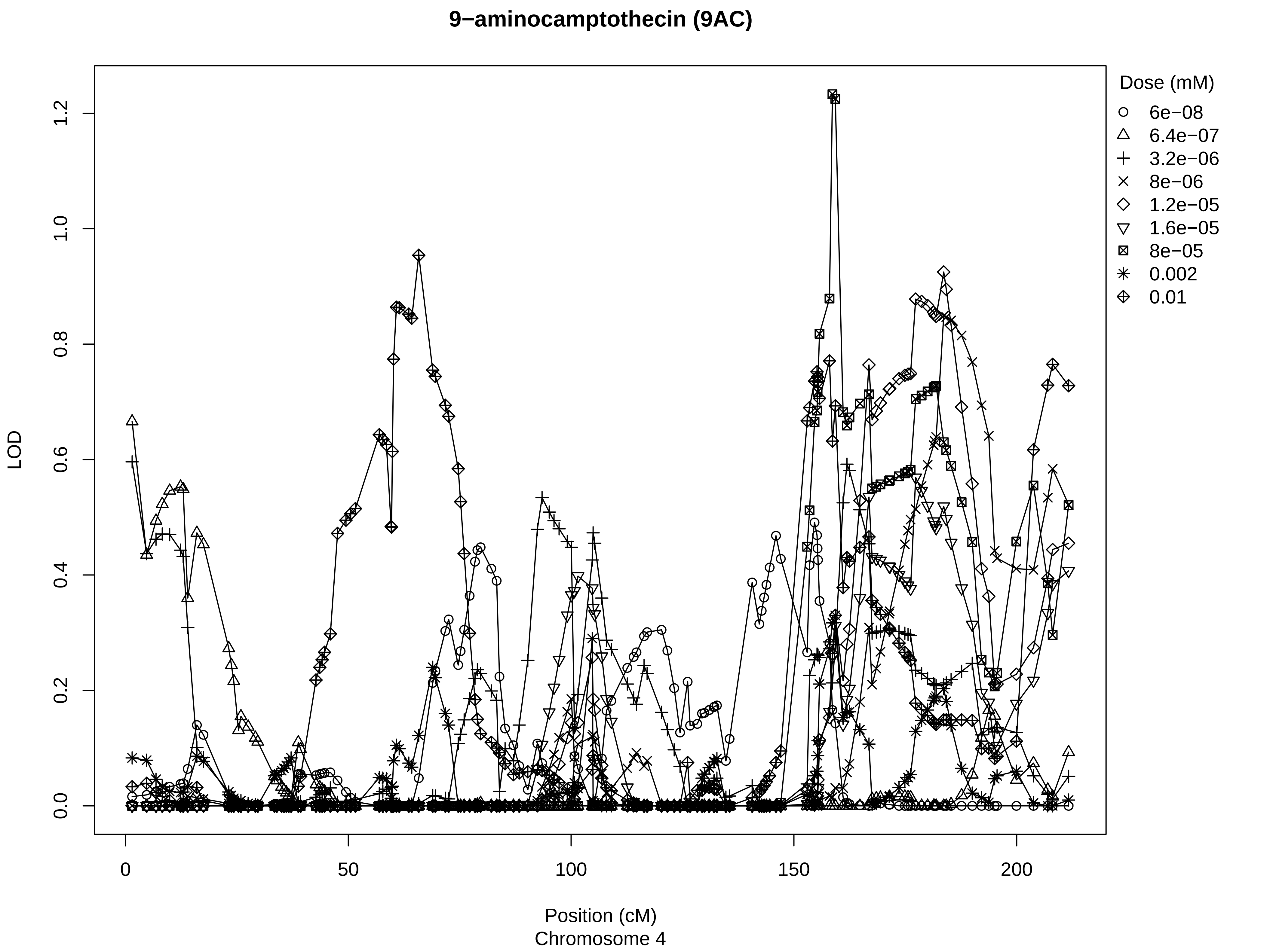

9−aminocamptothecin (9AC)
LOD
0.0
0.2
0.4
0.6
0.8
1.0
1.2
0
50
100
150
200
Position (cM)
Chromosome 4
Dose (mM)
6e−08
6.4e−07
3.2e−06
8e−06
1.2e−05
1.6e−05
8e−05
0.002
0.01

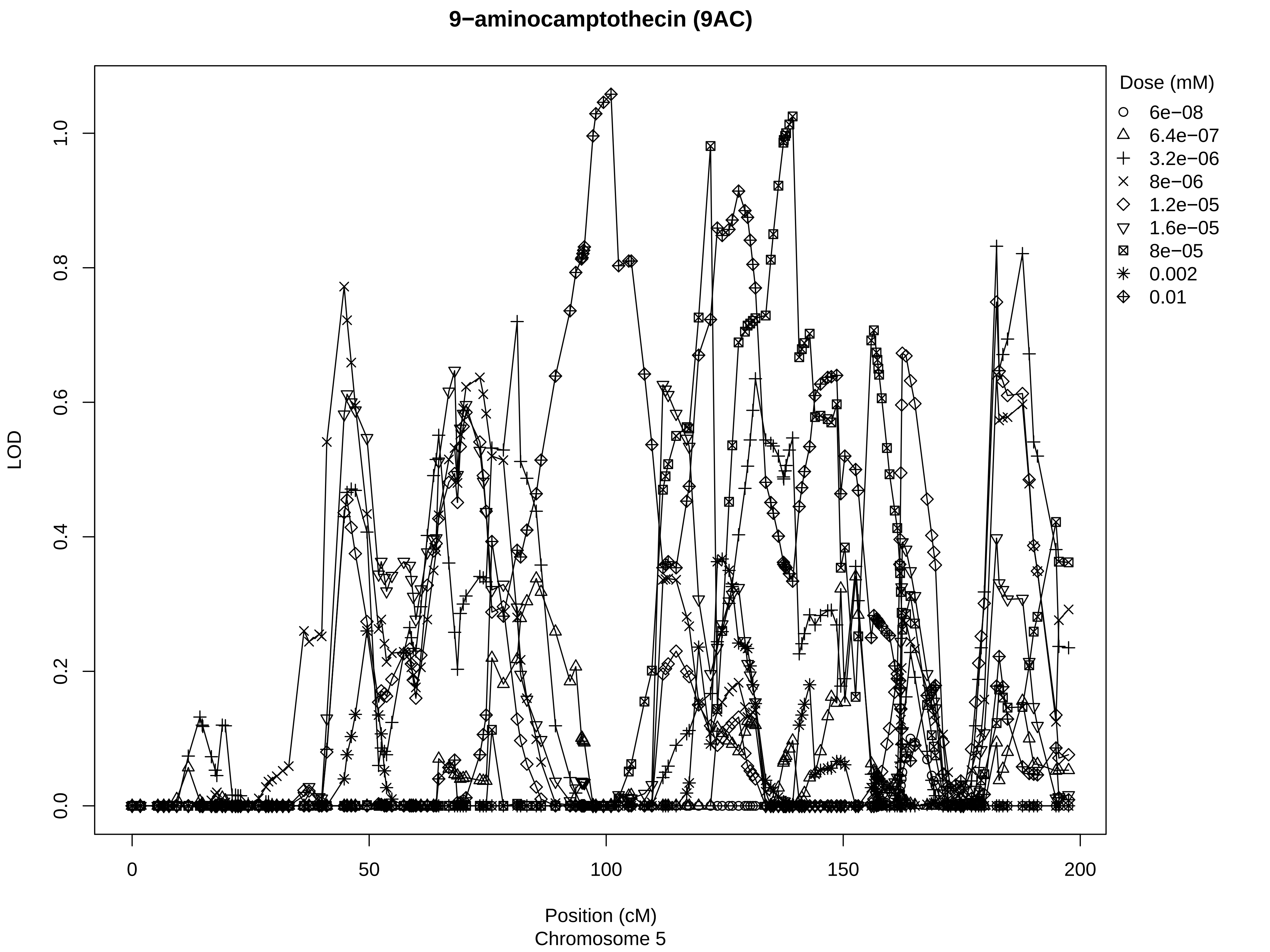

9−aminocamptothecin (9AC)
LOD
0.0
0.2
0.4
0.6
0.8
1.0
0
50
100
150
200
Position (cM)
Chromosome 5
Dose (mM)
6e−08
6.4e−07
3.2e−06
8e−06
1.2e−05
1.6e−05
8e−05
0.002
0.01

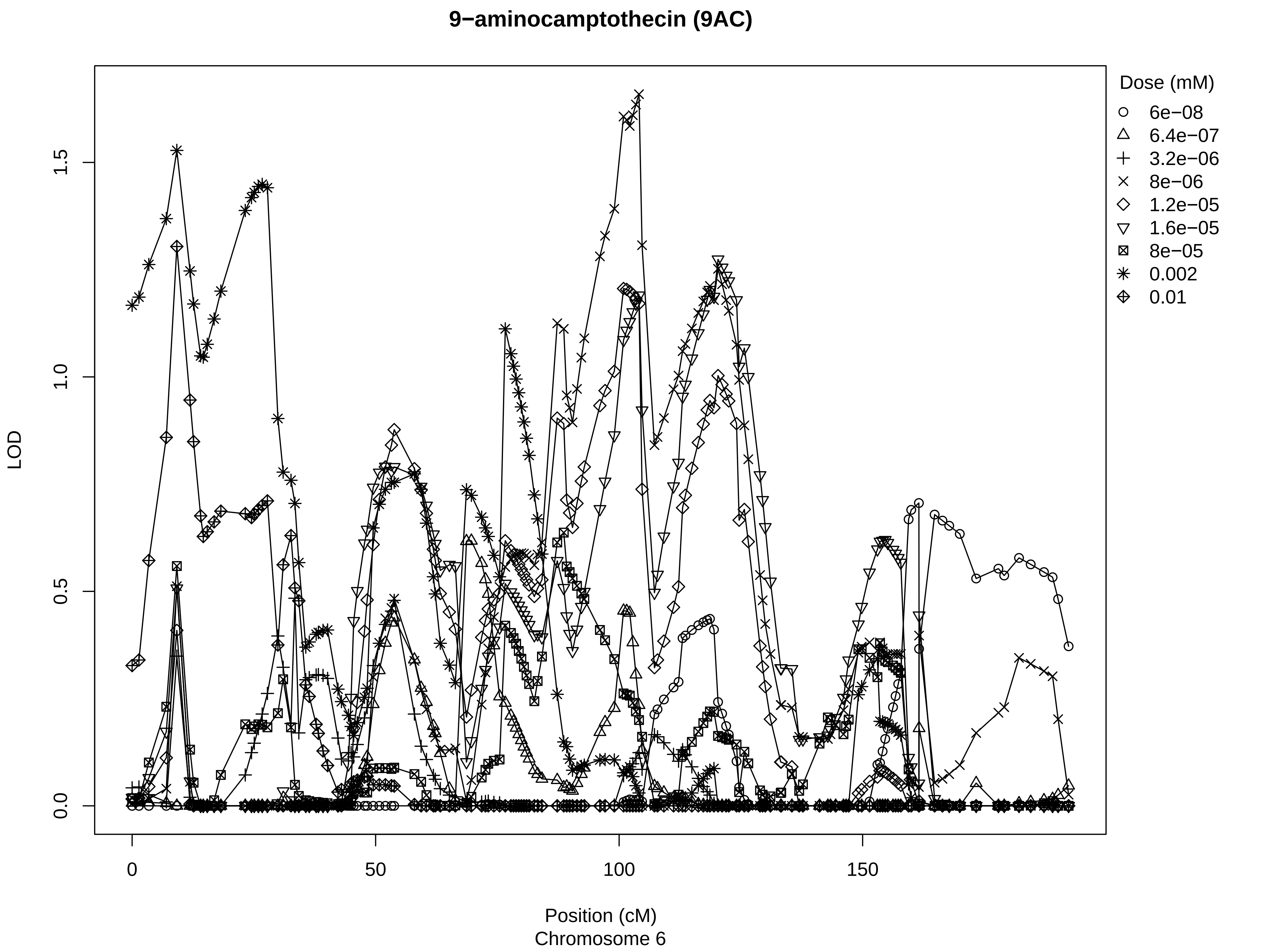
9−aminocamptothecin (9AC)
LOD
0.0
0.5
1.0
1.5
0
50
100
150
Position (cM)
Chromosome 6
Dose (mM)
6e−08
6.4e−07
3.2e−06
8e−06
1.2e−05
1.6e−05
8e−05
0.002
0.01

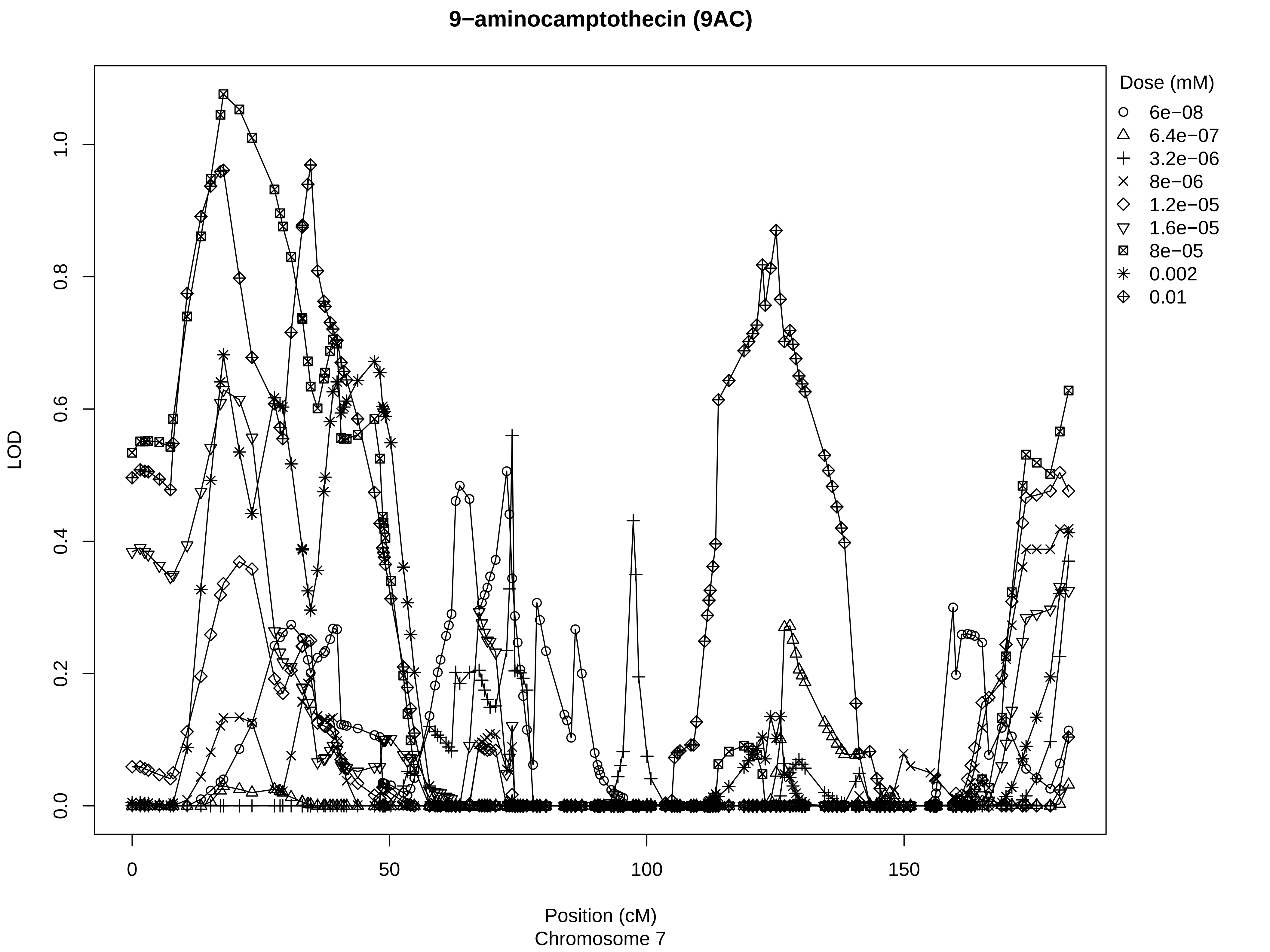

9−aminocamptothecin (9AC)
Dose (mM)
6e−08
6.4e−07
3.2e−06
8e−06
1.2e−05
1.6e−05
8e−05
0.002
0.01
LOD
0.0
0.2
0.4
0.6
0.8
1.0
0
50
100
150
Position (cM)
Chromosome 7

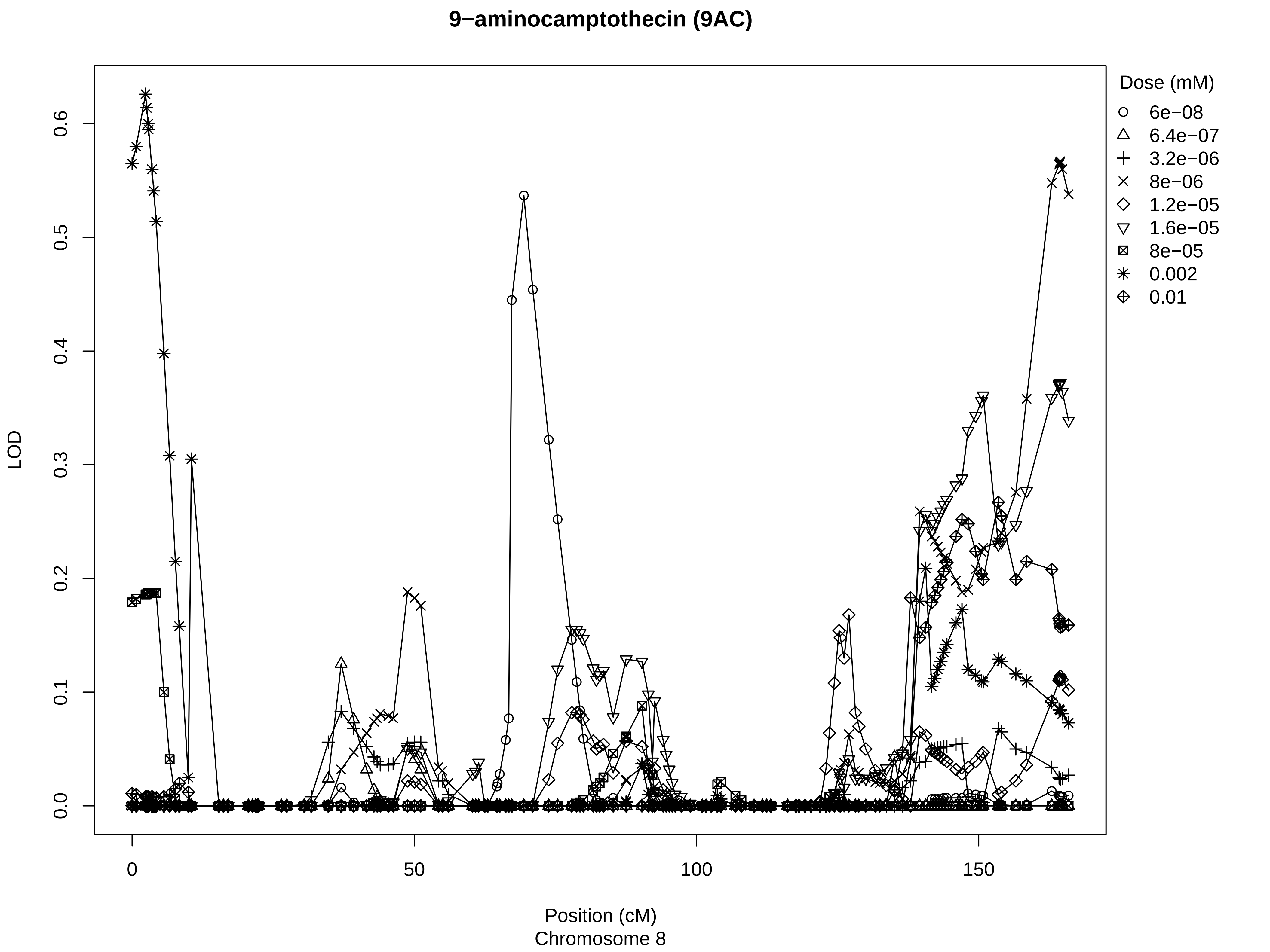
9−aminocamptothecin (9AC)
LOD
0.0
0.1
0.2
0.3
0.4
0.5
0.6
0
50
100
150
Position (cM)
Chromosome 8
Dose (mM)
6e−08
6.4e−07
3.2e−06
8e−06
1.2e−05
1.6e−05
8e−05
0.002
0.01

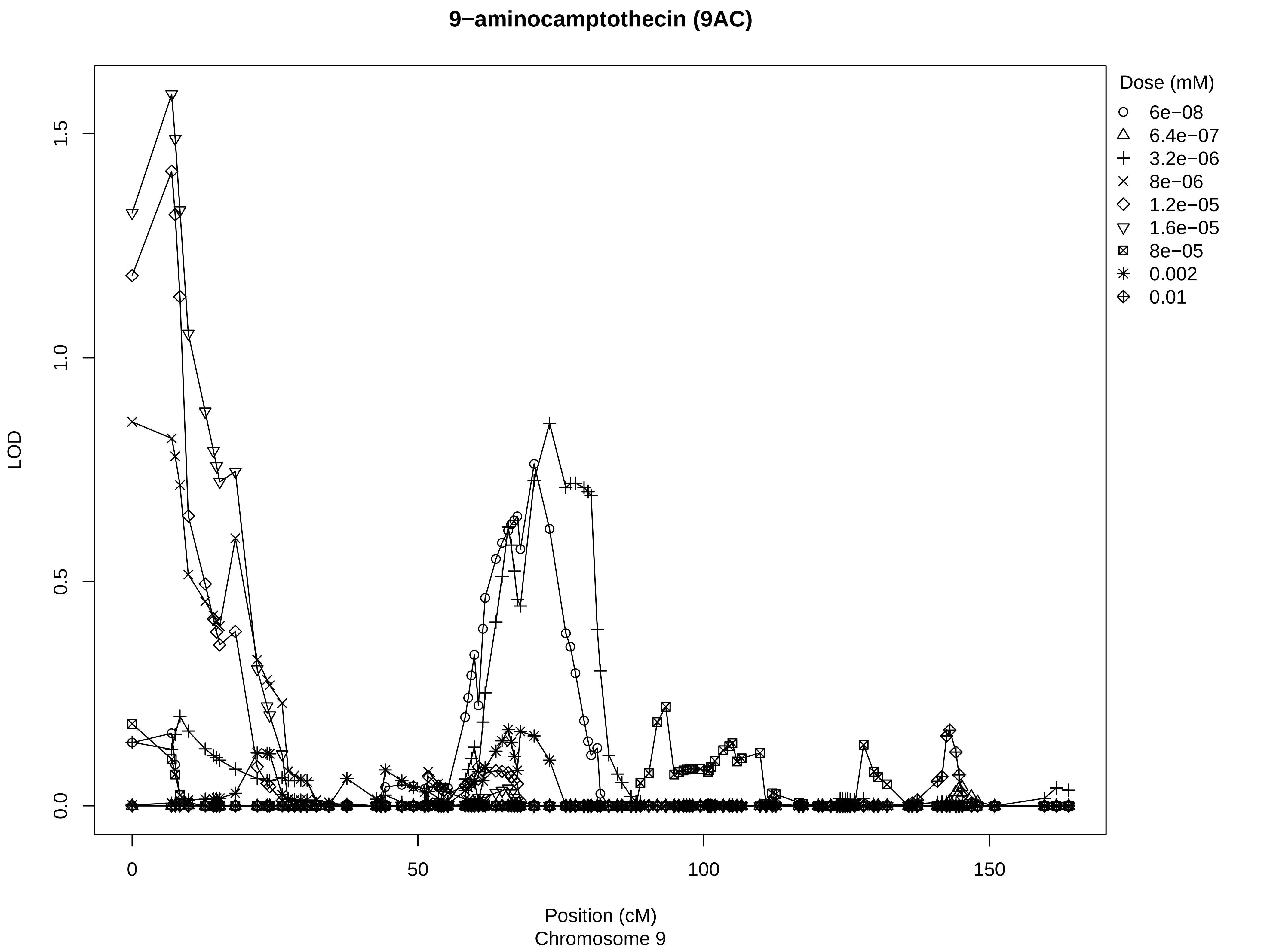
9−aminocamptothecin (9AC)
LOD
0.0
0.5
1.0
1.5
0
50
100
150
Position (cM)
Chromosome 9
Dose (mM)
6e−08
6.4e−07
3.2e−06
8e−06
1.2e−05
1.6e−05
8e−05
0.002
0.01

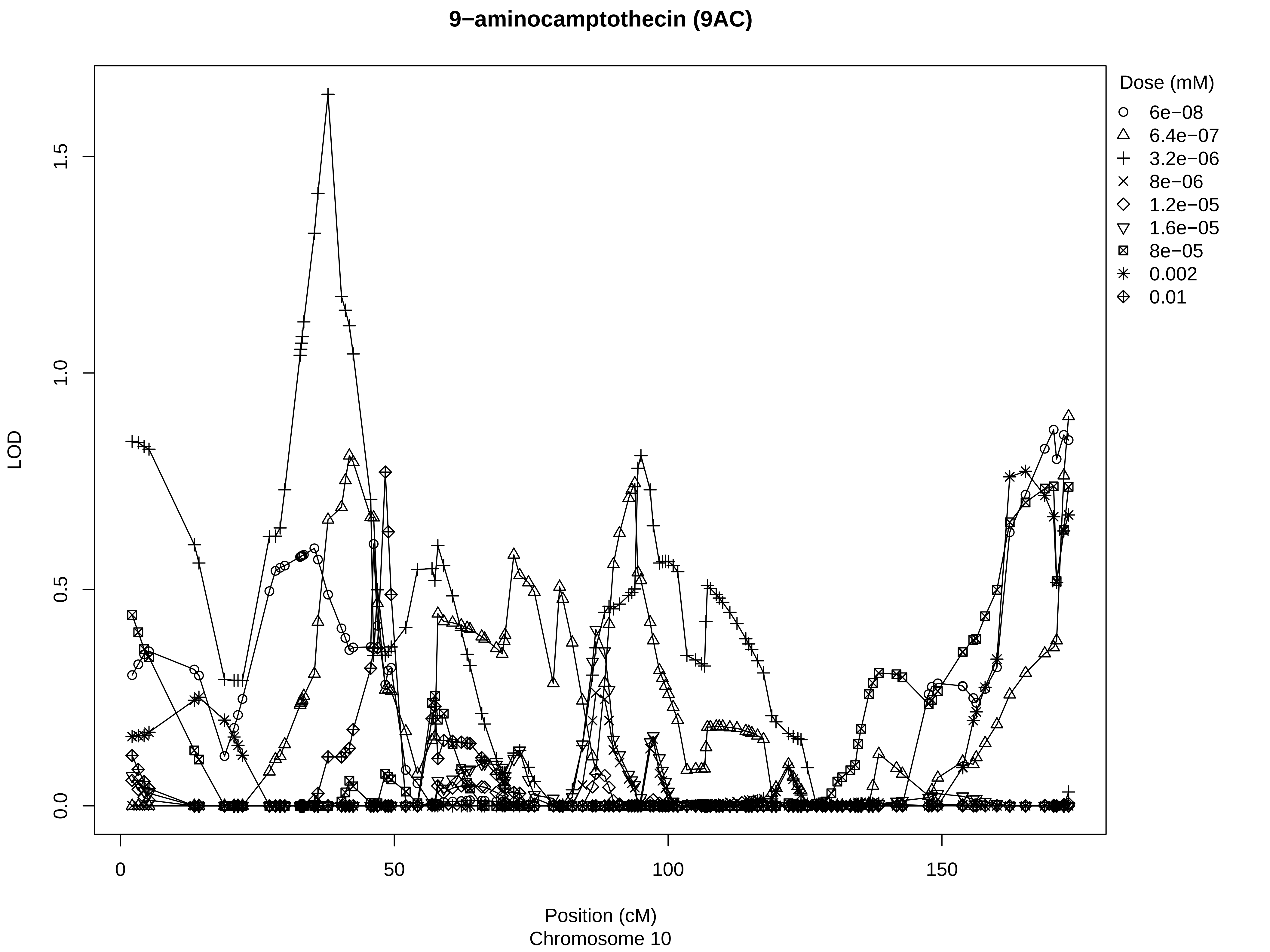

9−aminocamptothecin (9AC)
Dose (mM)
6e−08
6.4e−07
3.2e−06
8e−06
1.2e−05
1.6e−05
8e−05
0.002
0.01
LOD
0.0
0.5
1.0
1.5
0
50
100
150
Position (cM)
Chromosome 10

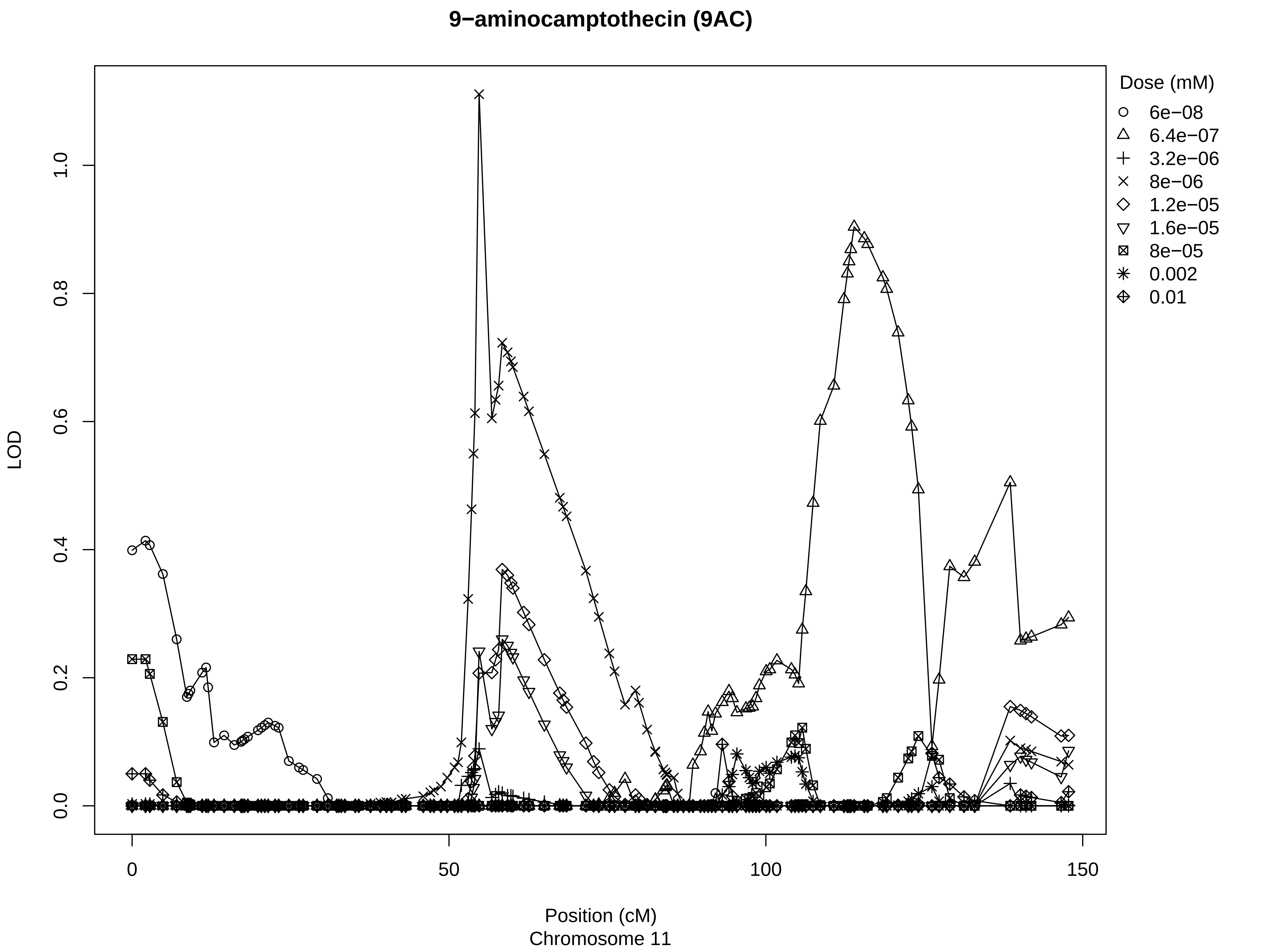

9−aminocamptothecin (9AC)
LOD
0.0
0.2
0.4
0.6
0.8
1.0
0
50
100
150
Position (cM)
Chromosome 11
Dose (mM)
6e−08
6.4e−07
3.2e−06
8e−06
1.2e−05
1.6e−05
8e−05
0.002
0.01

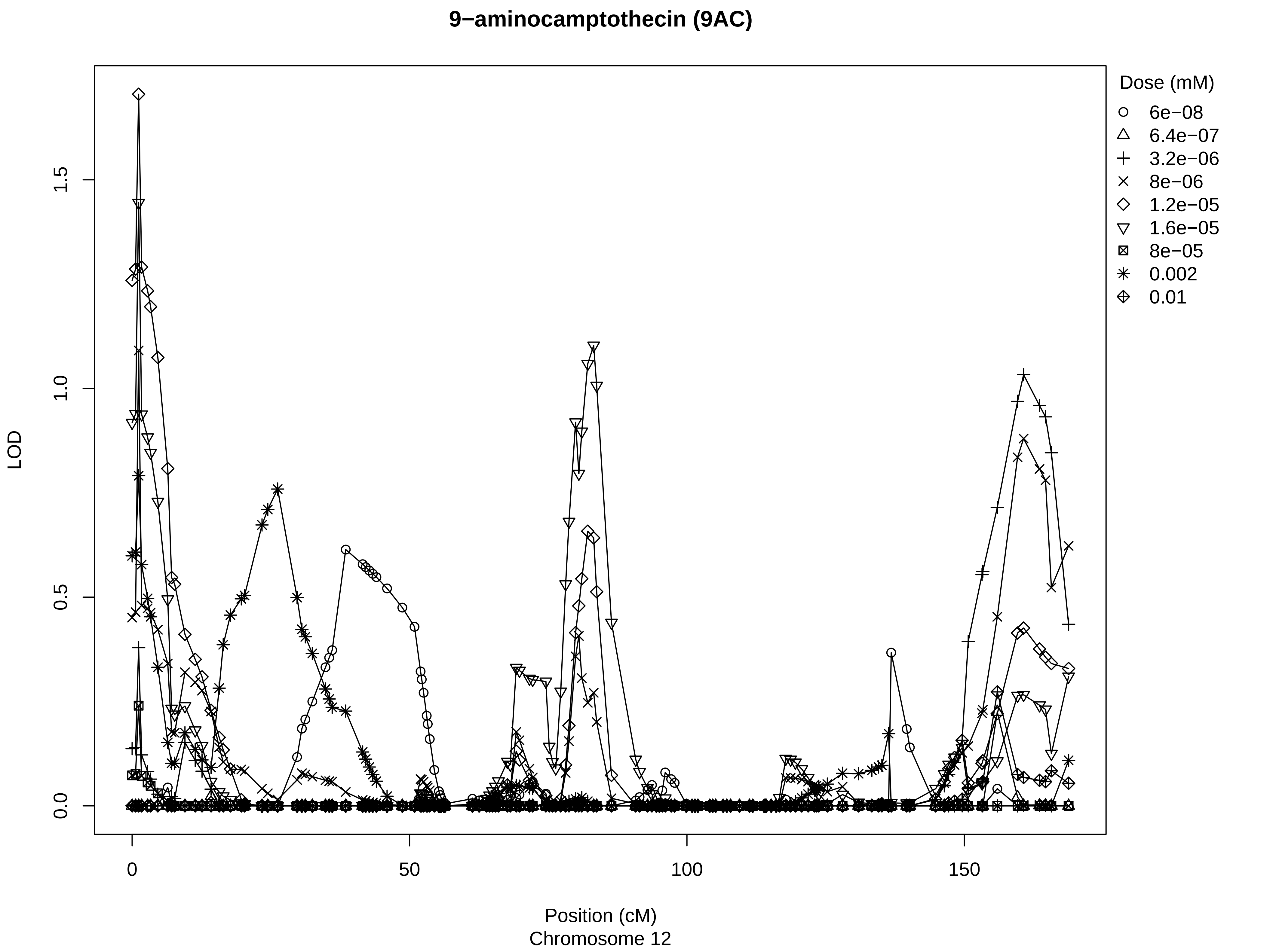
9−aminocamptothecin (9AC)
LOD
0.0
0.5
1.0
1.5
0
50
100
150
Position (cM)
Chromosome 12
Dose (mM)
6e−08
6.4e−07
3.2e−06
8e−06
1.2e−05
1.6e−05
8e−05
0.002
0.01

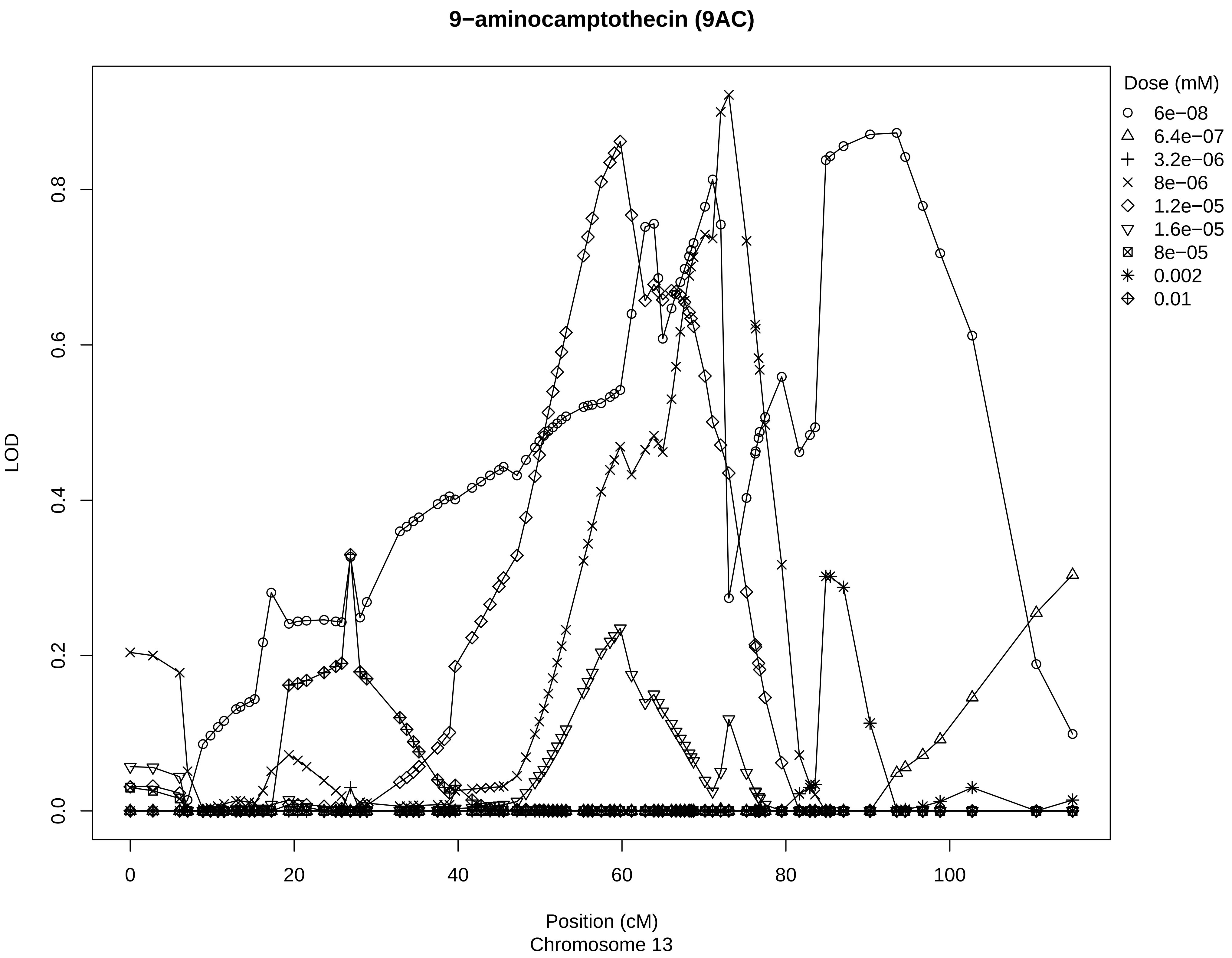
9−aminocamptothecin (9AC)
LOD
0.0
0.2
0.4
0.6
0.8
0
20
40
60
80
100
Position (cM)
Chromosome 13
Dose (mM)
6e−08
6.4e−07
3.2e−06
8e−06
1.2e−05
1.6e−05
8e−05
0.002
0.01

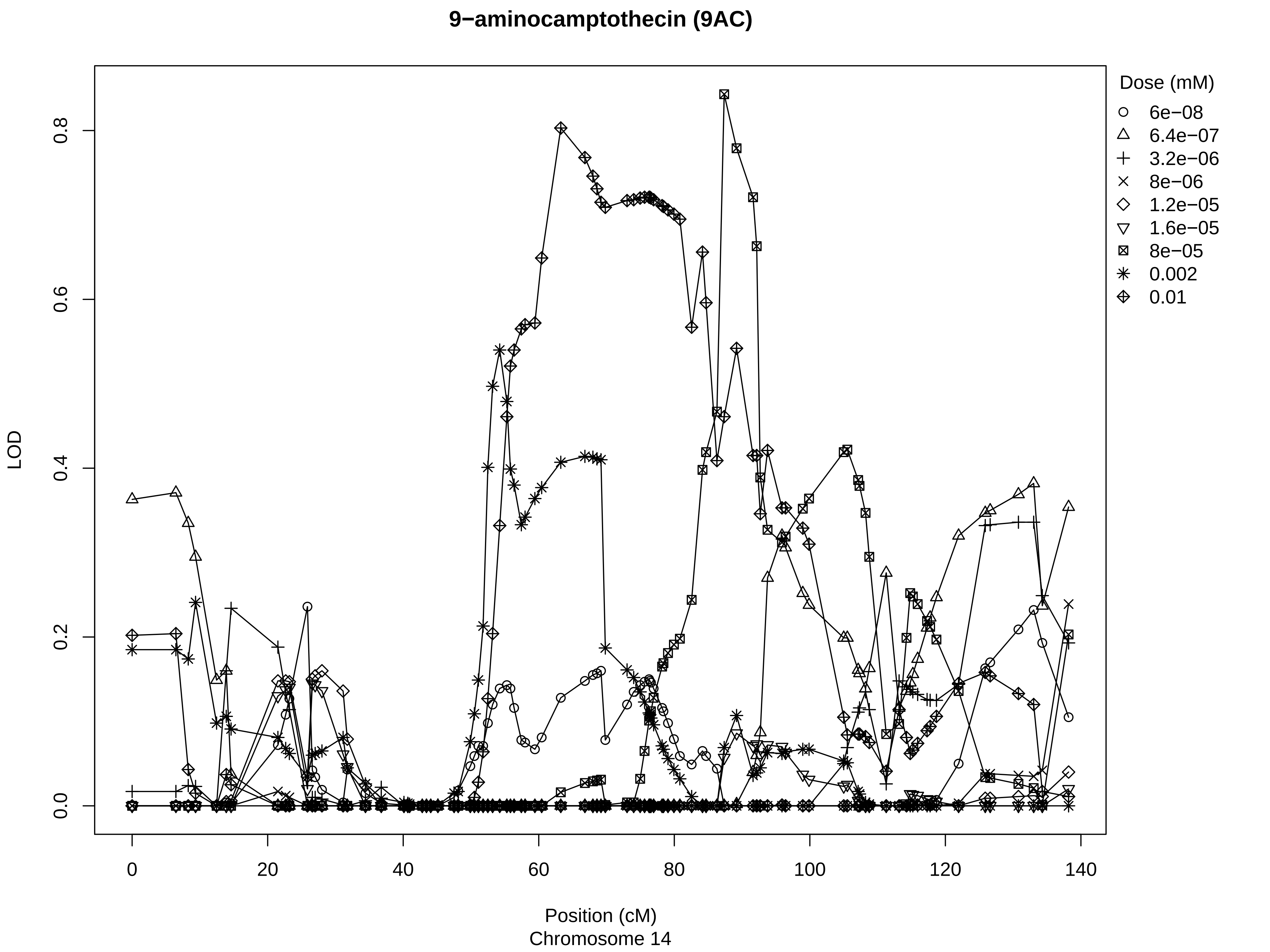

9−aminocamptothecin (9AC)
LOD
0.0
0.2
0.4
0.6
0.8
0
20
40
60
80
100
120
140
Position (cM)
Chromosome 14
Dose (mM)
6e−08
6.4e−07
3.2e−06
8e−06
1.2e−05
1.6e−05
8e−05
0.002
0.01

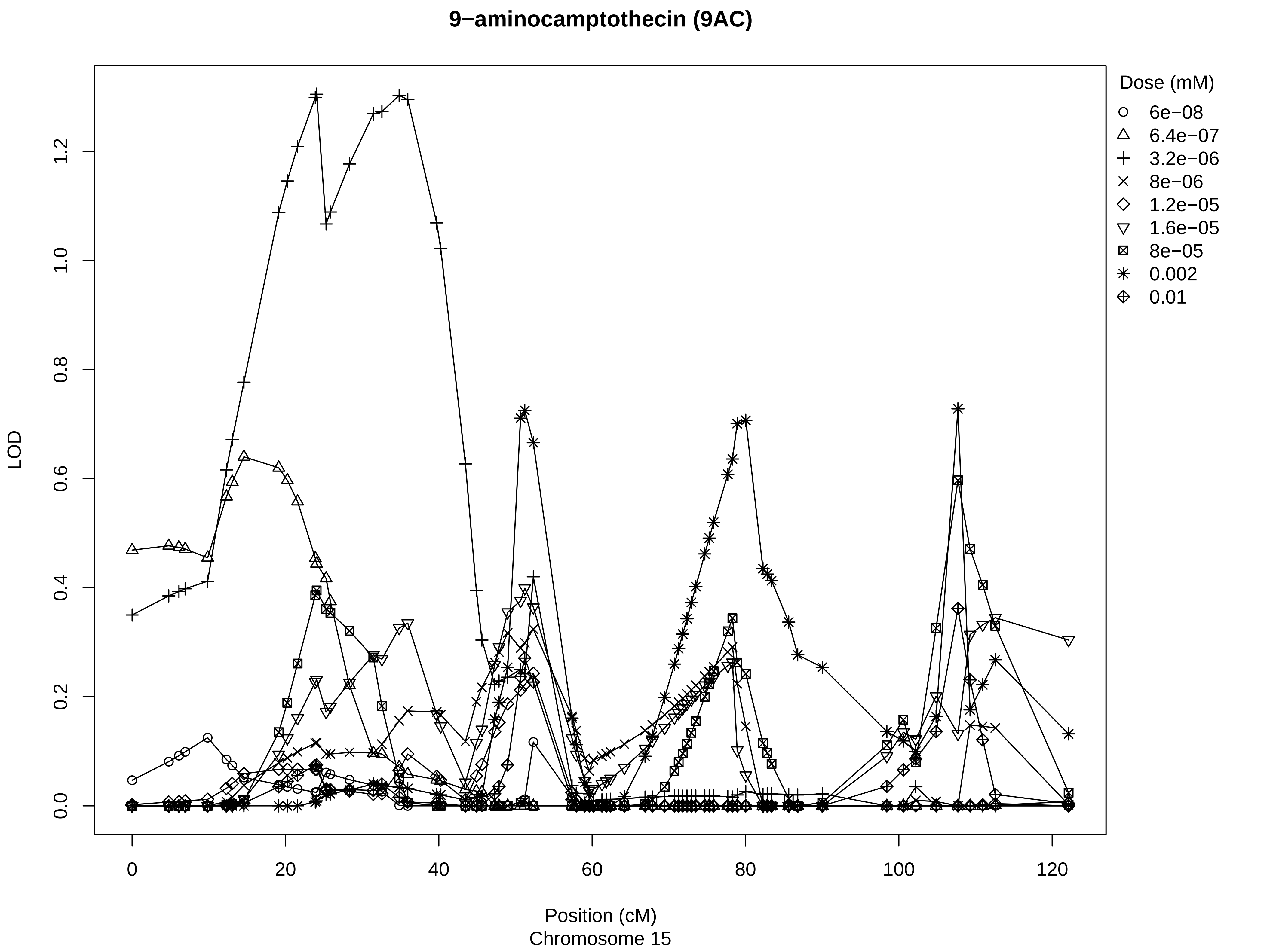

9−aminocamptothecin (9AC)
Dose (mM)
6e−08
6.4e−07
3.2e−06
8e−06
1.2e−05
1.6e−05
8e−05
0.002
0.01
LOD
0.0
0.2
0.4
0.6
0.8
1.0
1.2
0
20
40
60
80
100
120
Position (cM)
Chromosome 15

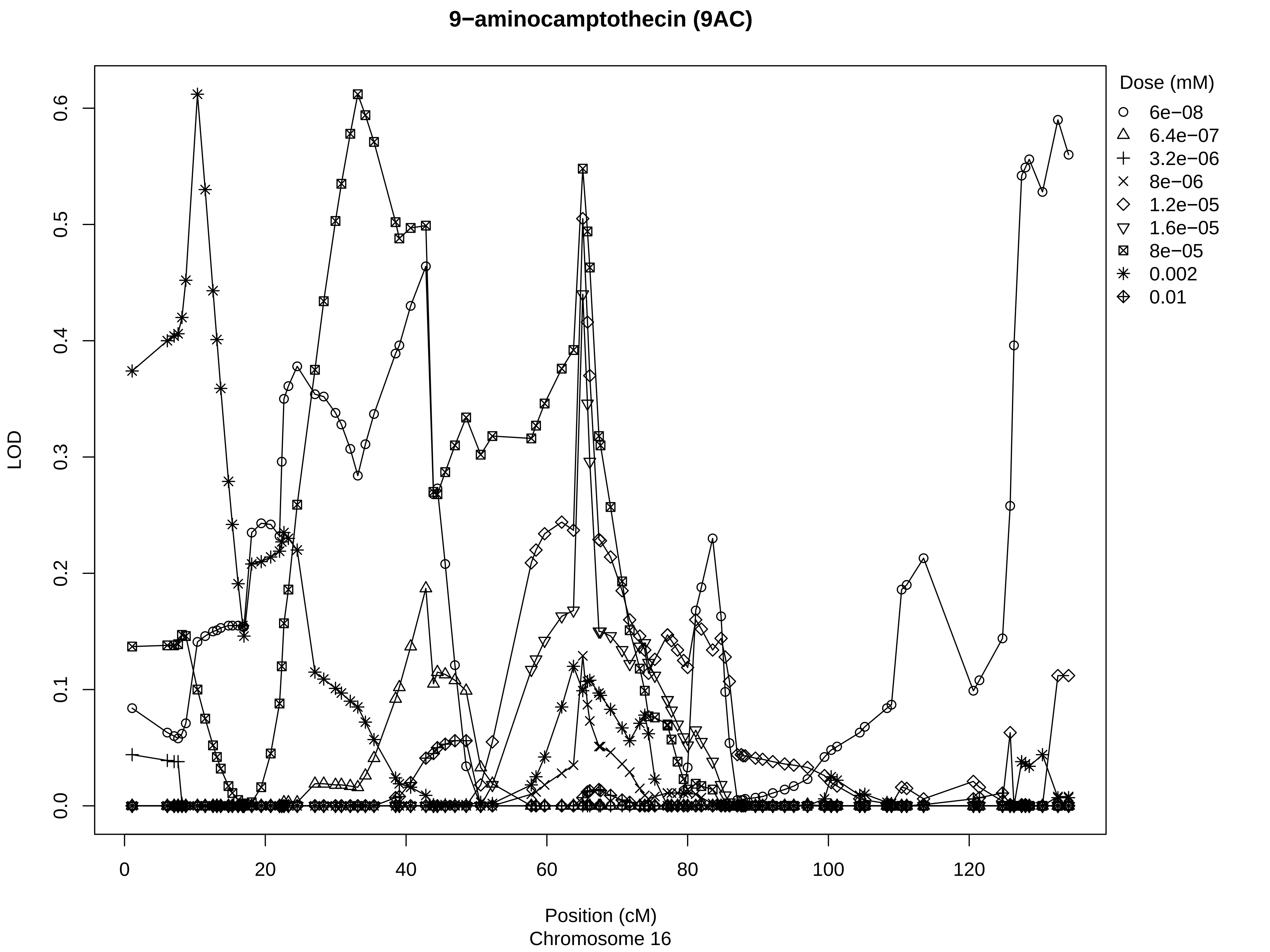

9−aminocamptothecin (9AC)
Dose (mM)
6e−08
6.4e−07
3.2e−06
8e−06
1.2e−05
1.6e−05
8e−05
0.002
0.01
LOD
0.0
0.1
0.2
0.3
0.4
0.5
0.6
0
20
40
60
80
100
120
Position (cM)
Chromosome 16

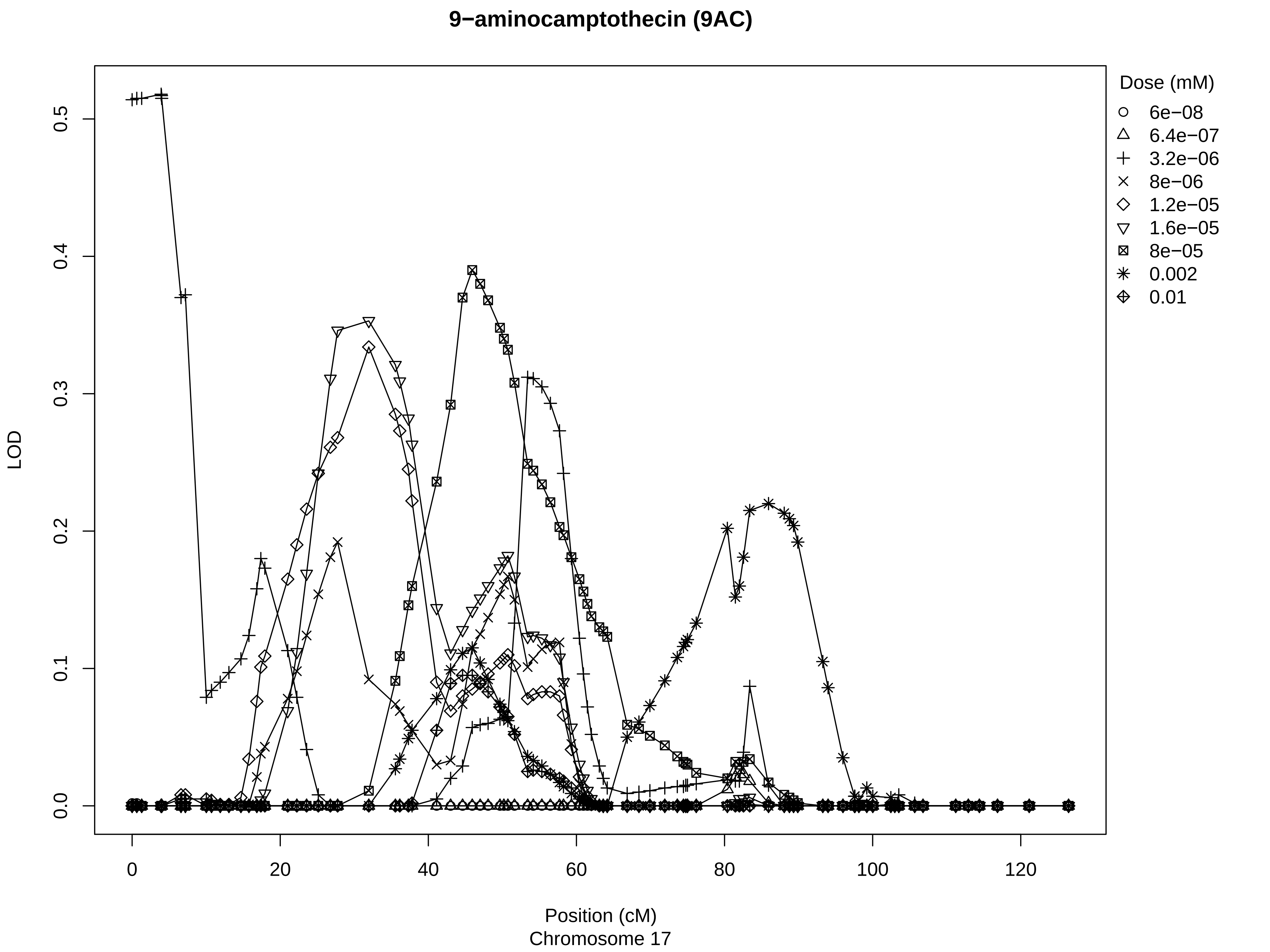
9−aminocamptothecin (9AC)
LOD
0.0
0.1
0.2
0.3
0.4
0.5
0
20
40
60
80
100
120
Position (cM)
Chromosome 17
Dose (mM)
6e−08
6.4e−07
3.2e−06
8e−06
1.2e−05
1.6e−05
8e−05
0.002
0.01

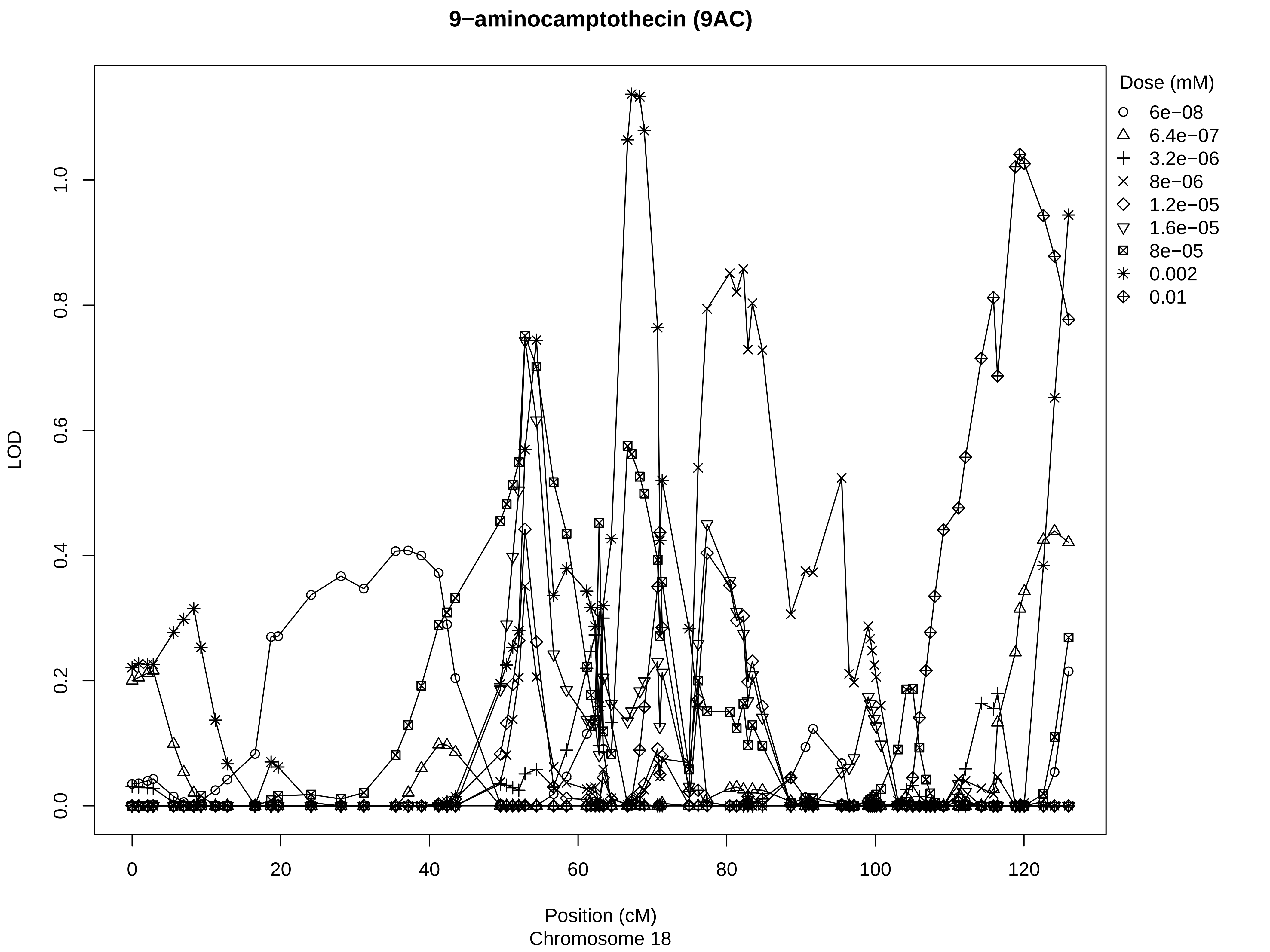
9−aminocamptothecin (9AC)
Dose (mM)
6e−08
6.4e−07
3.2e−06
8e−06
1.2e−05
1.6e−05
8e−05
0.002
0.01
LOD
0.0
0.2
0.4
0.6
0.8
1.0
0
20
40
60
80
100
120
Position (cM)
Chromosome 18

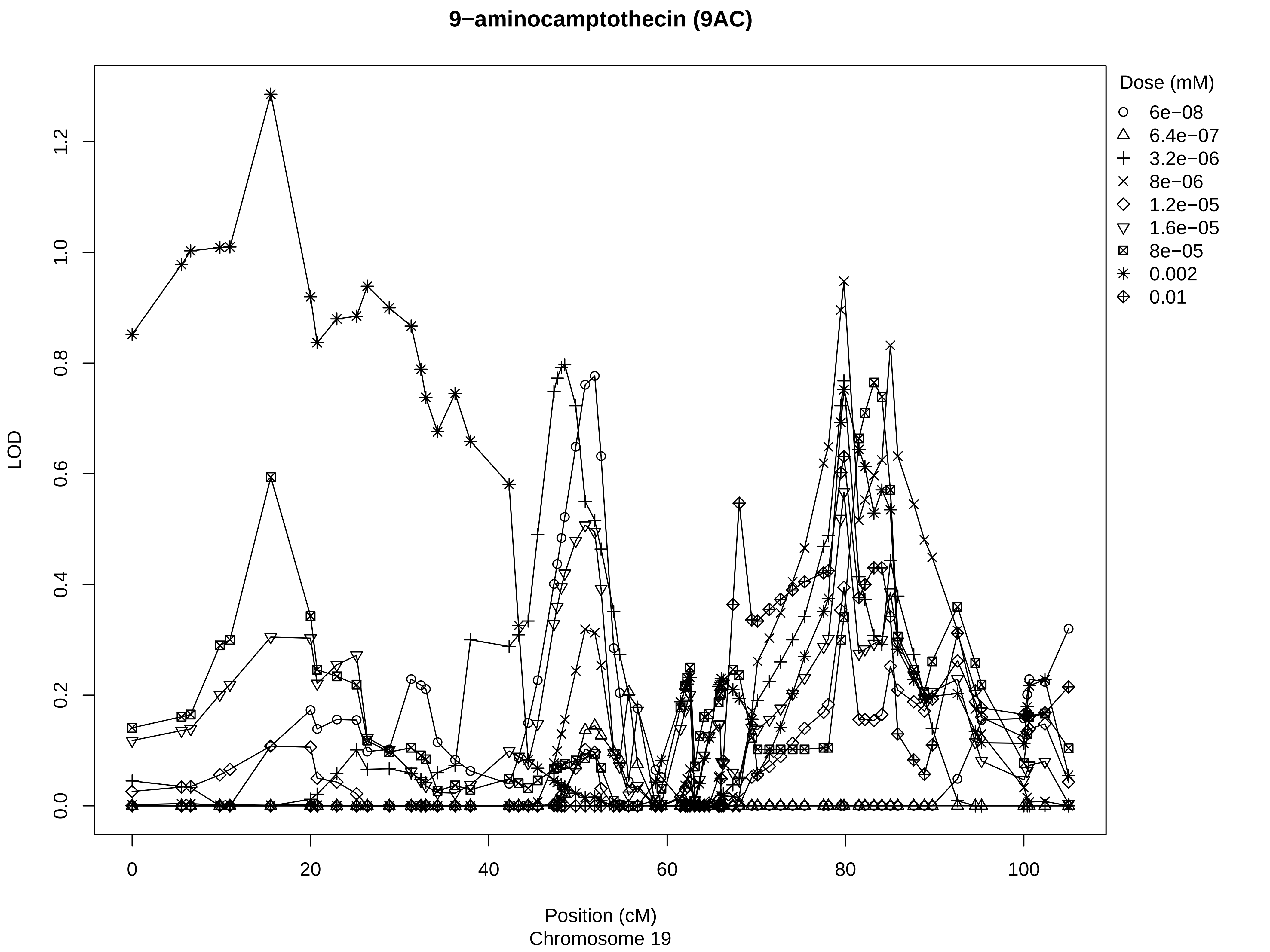
9−aminocamptothecin (9AC)
LOD
Position (cM)
Chromosome 19
Dose (mM)
6e−08
6.4e−07
3.2e−06
8e−06
1.2e−05
1.6e−05
8e−05
0.002
0.01
0.0
0.2
0.4
0.6
0.8
1.0
1.2
0
20
40
60
80
100

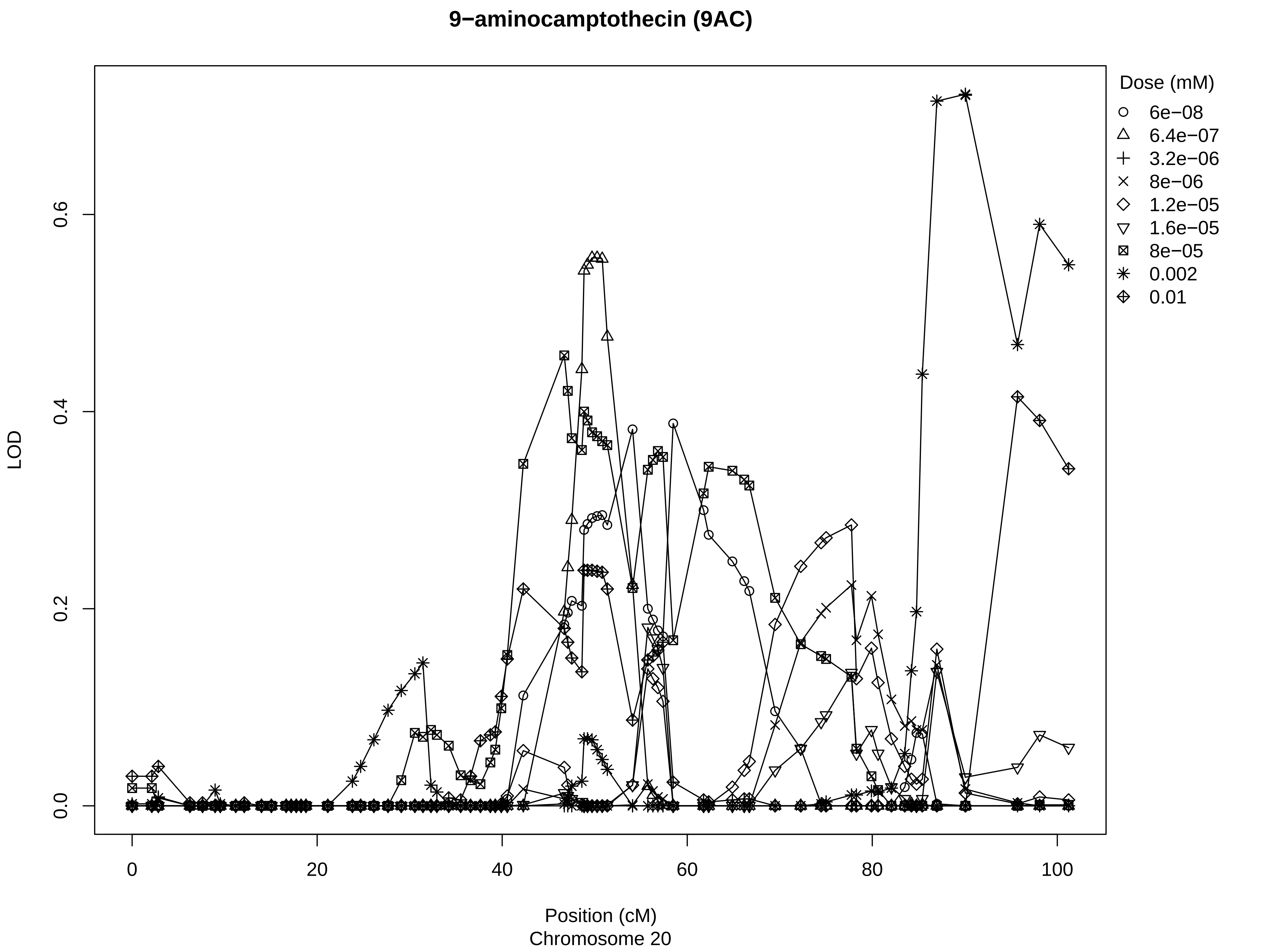
9−aminocamptothecin (9AC)
Dose (mM)
6e−08
6.4e−07
3.2e−06
8e−06
1.2e−05
1.6e−05
8e−05
0.002
0.01
LOD
0.0
0.2
0.4
0.6
0
20
40
60
80
100
Position (cM)
Chromosome 20

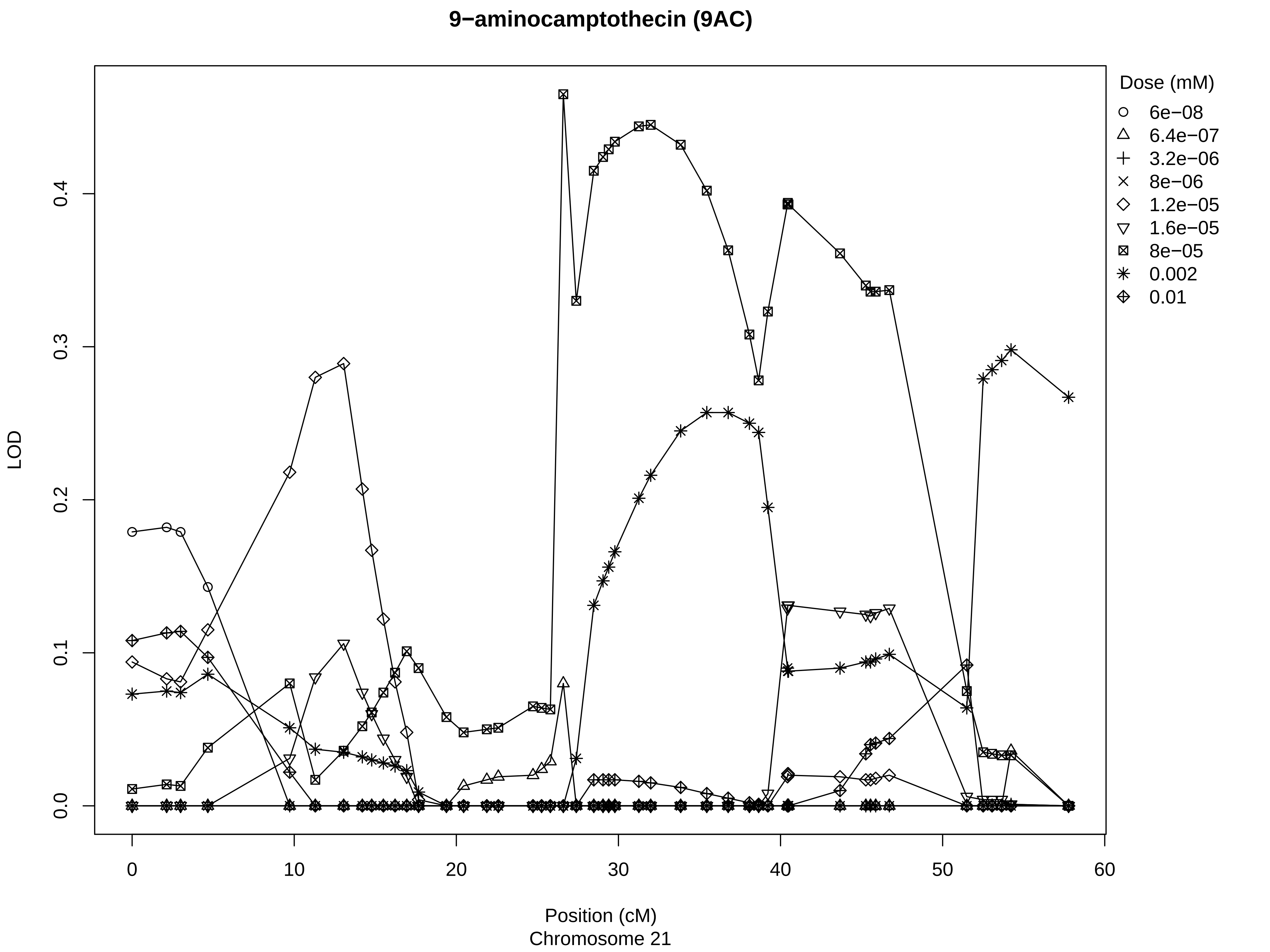

9−aminocamptothecin (9AC)
Dose (mM)
6e−08
6.4e−07
3.2e−06
8e−06
1.2e−05
1.6e−05
8e−05
0.002
0.01
LOD
0.0
0.1
0.2
0.3
0.4
0
10
20
30
40
50
60
Position (cM)
Chromosome 21

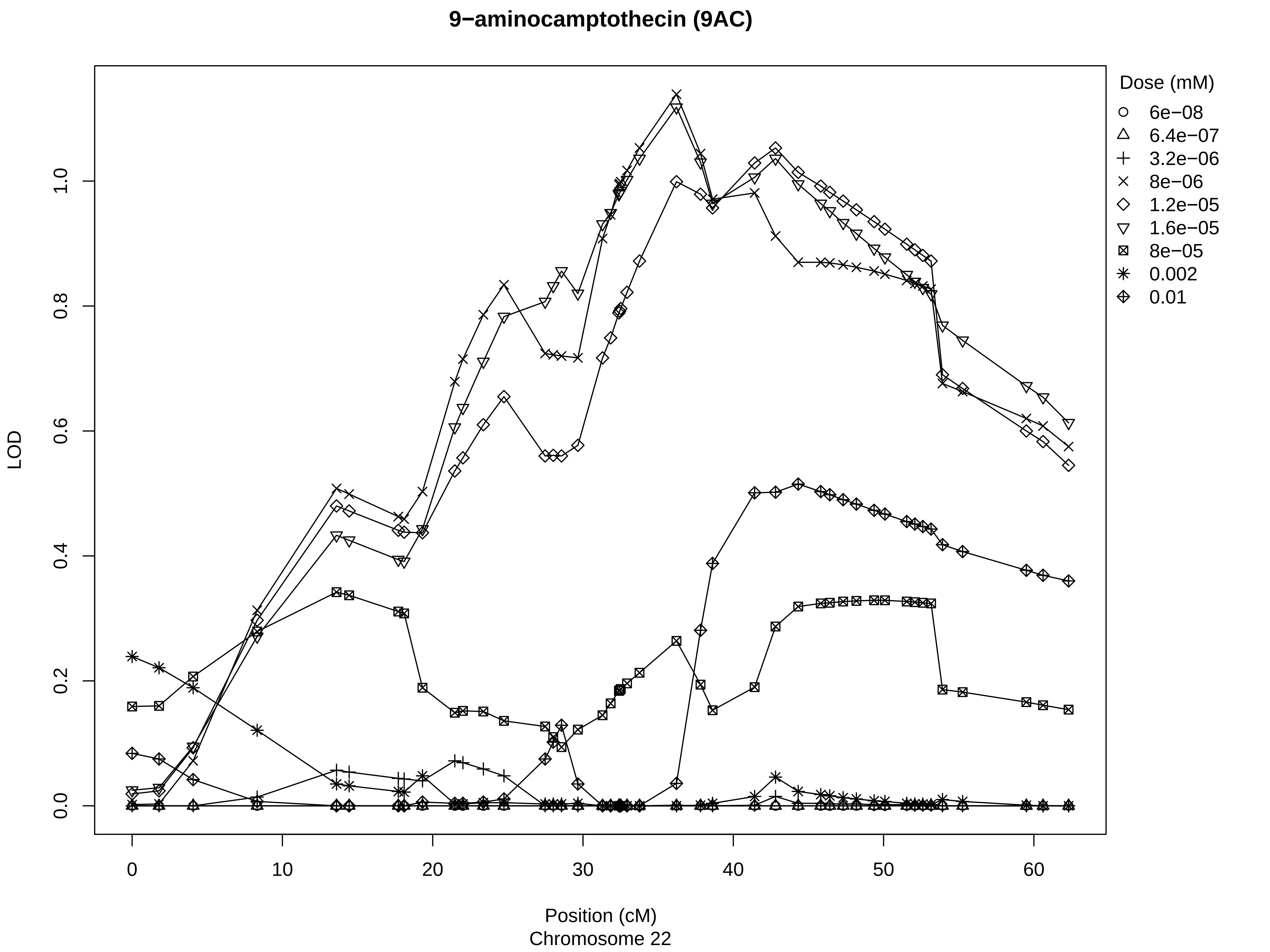

9−aminocamptothecin (9AC)
LOD
0.0
0.2
0.4
0.6
0.8
1.0
0
10
20
30
40
50
60
Position (cM)
Chromosome 22
Dose (mM)
6e−08
6.4e−07
3.2e−06
8e−06
1.2e−05
1.6e−05
8e−05
0.002
0.01

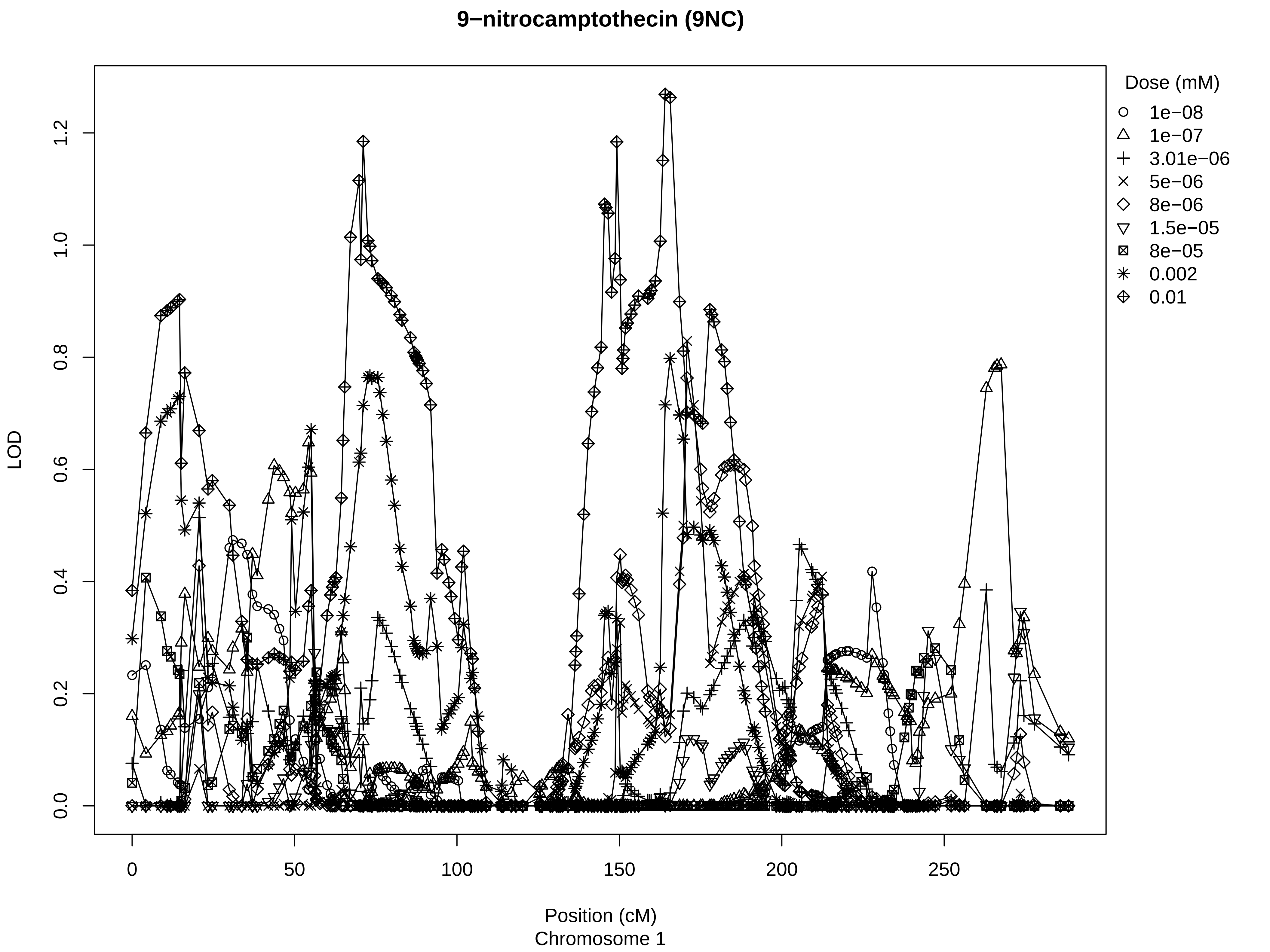
9−nitrocamptothecin (9NC)
LOD
Position (cM)
Chromosome 1
Dose (mM)
1e−08
1e−07
3.01e−06
5e−06
8e−06
1.5e−05
8e−05
0.002
0.01
0.0
0.2
0.4
0.6
0.8
1.0
1.2
0
50
100
150
200
250

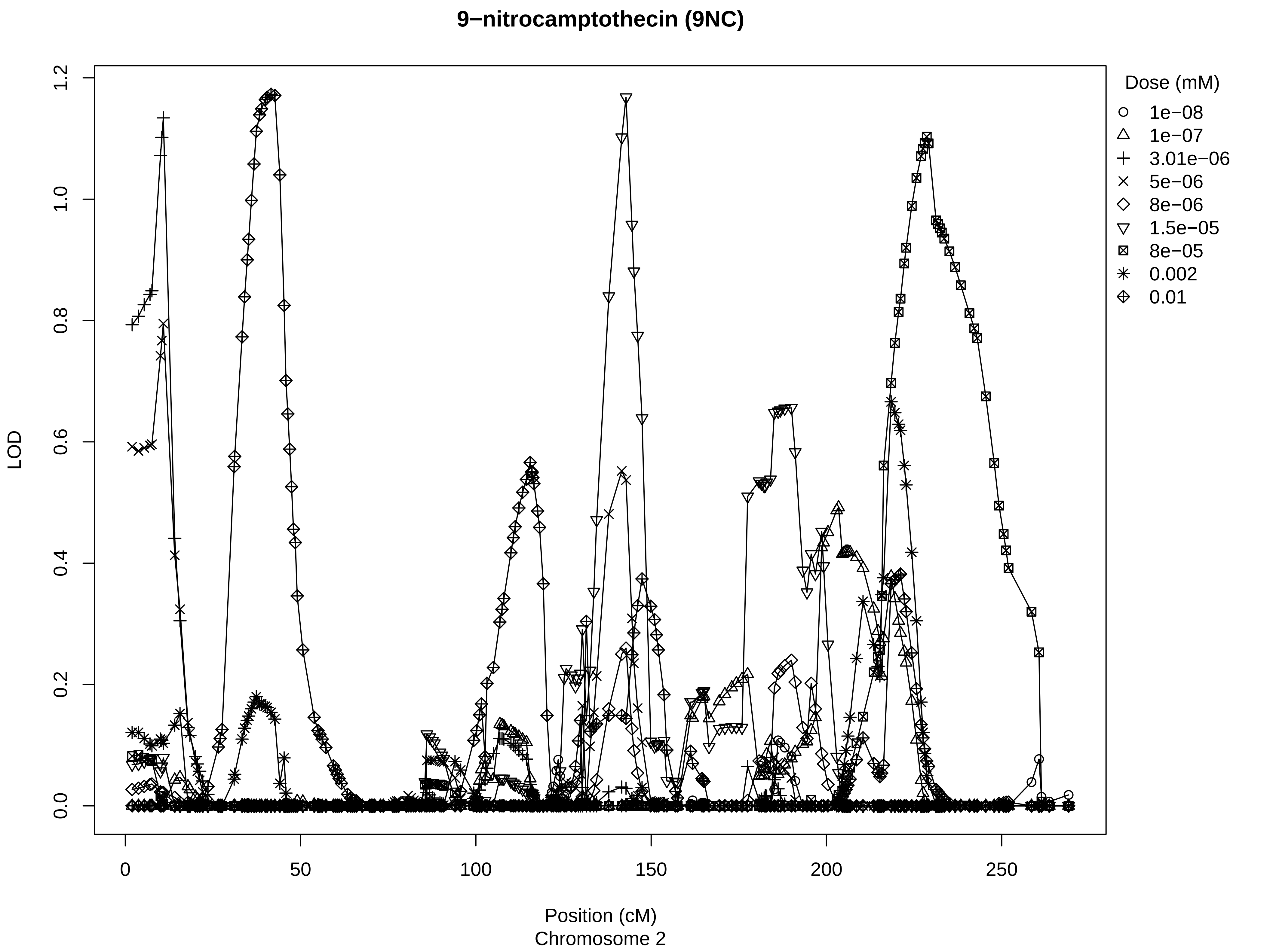
9−nitrocamptothecin (9NC)
LOD
Position (cM)
Chromosome 2
Dose (mM)
1e−08
1e−07
3.01e−06
5e−06
8e−06
1.5e−05
8e−05
0.002
0.01
0.0
0.2
0.4
0.6
0.8
1.0
1.2
0
50
100
150
200
250

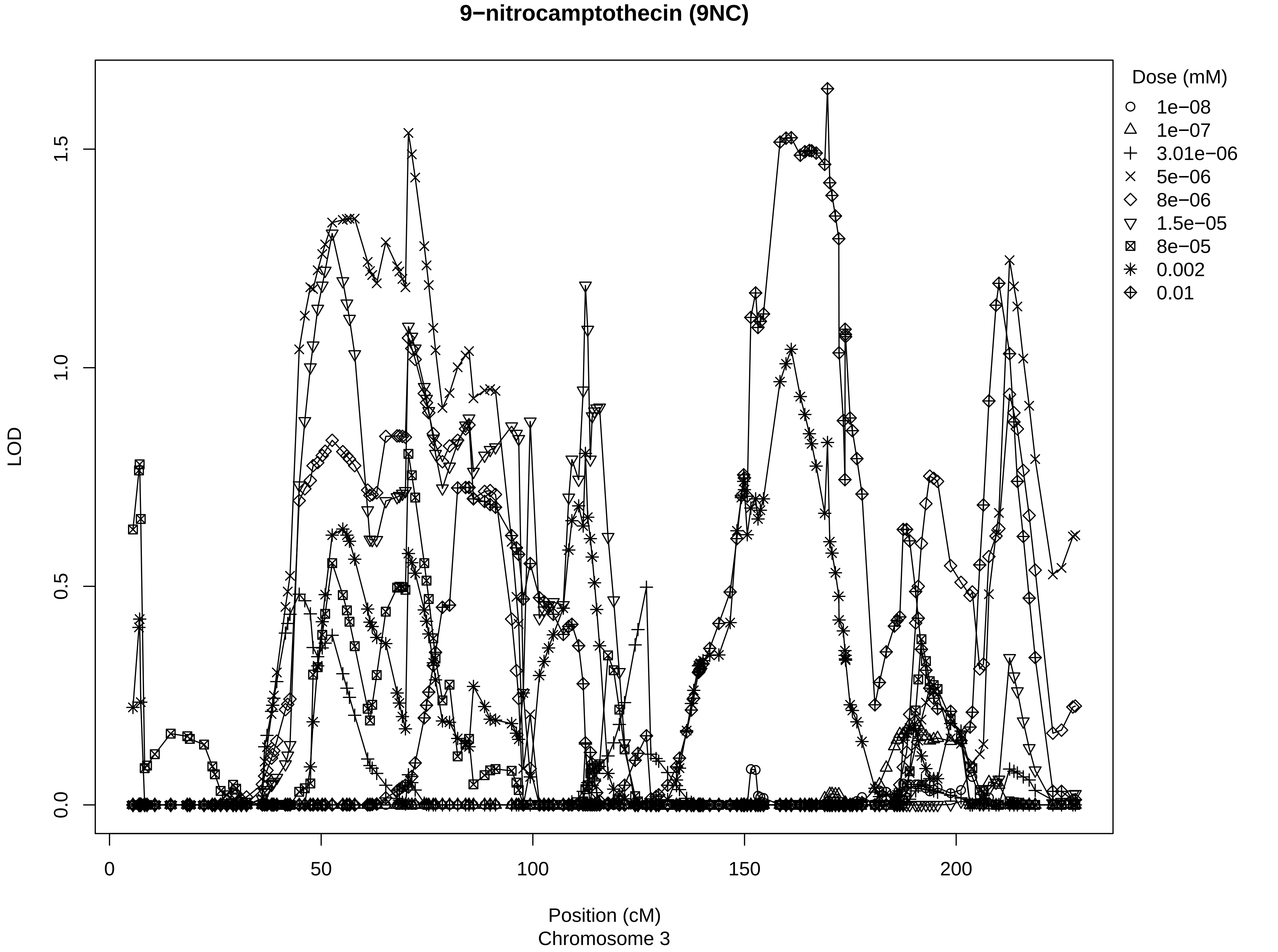

9−nitrocamptothecin (9NC)
LOD
0.0
0.5
1.0
1.5
0
50
100
150
200
Position (cM)
Chromosome 3
Dose (mM)
1e−08
1e−07
3.01e−06
5e−06
8e−06
1.5e−05
8e−05
0.002
0.01

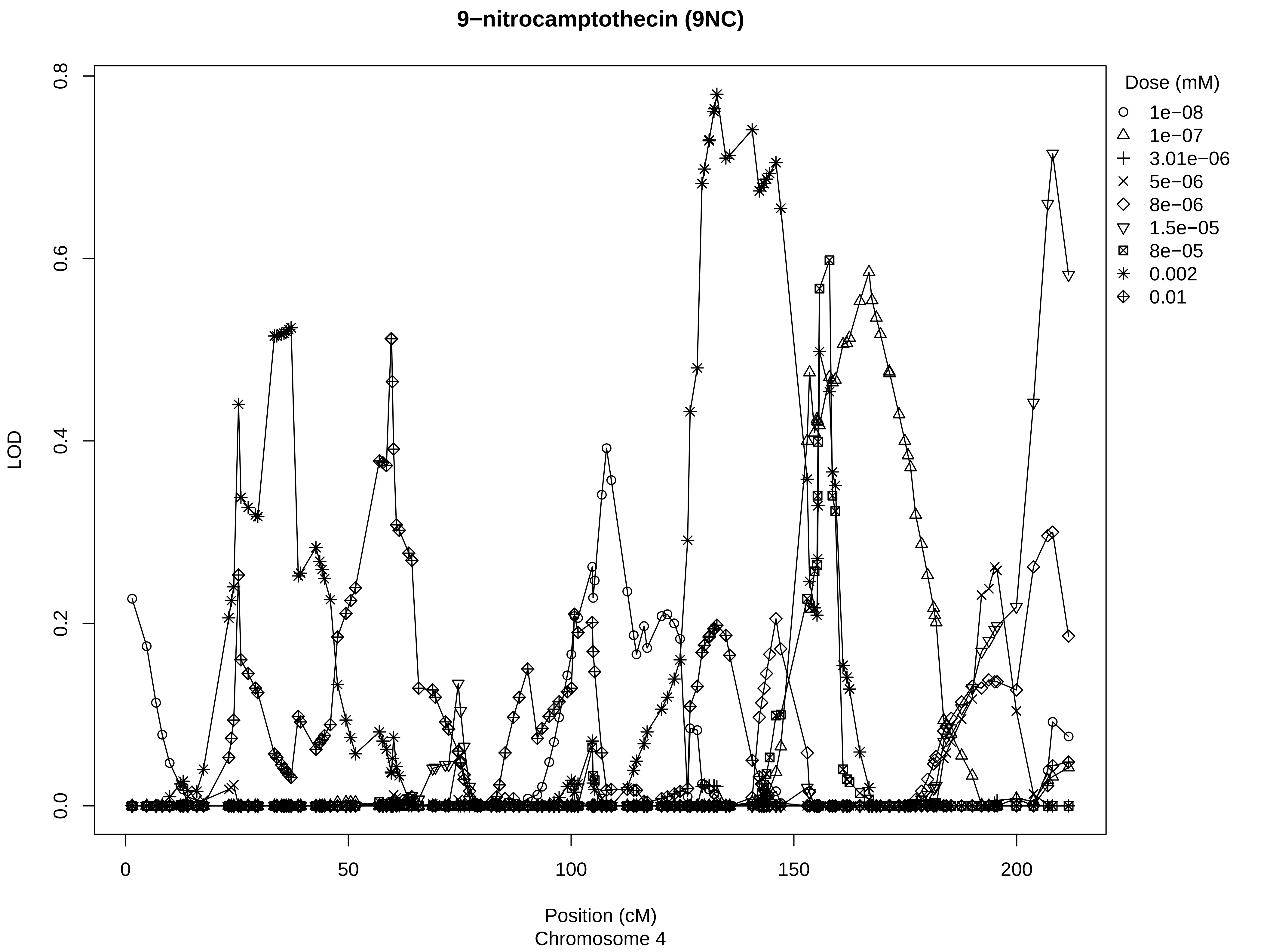
9−nitrocamptothecin (9NC)
Dose (mM)
1e−08
1e−07
3.01e−06
5e−06
8e−06
1.5e−05
8e−05
0.002
0.01
LOD
0.0
0.2
0.4
0.6
0.8
0
50
100
150
200
Position (cM)
Chromosome 4

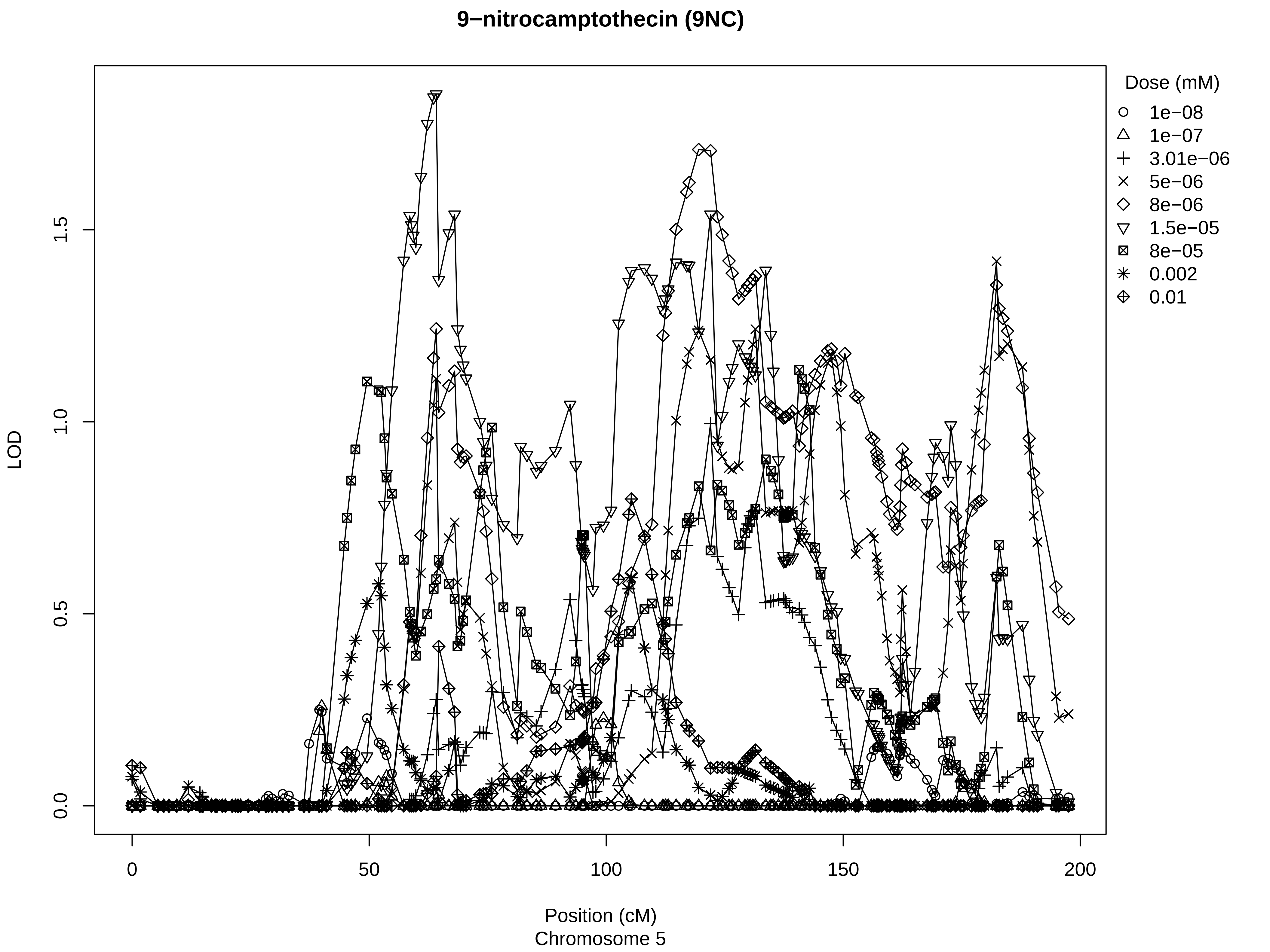

9−nitrocamptothecin (9NC)
LOD
0.0
0.5
1.0
1.5
0
50
100
150
200
Position (cM)
Chromosome 5
Dose (mM)
1e−08
1e−07
3.01e−06
5e−06
8e−06
1.5e−05
8e−05
0.002
0.01

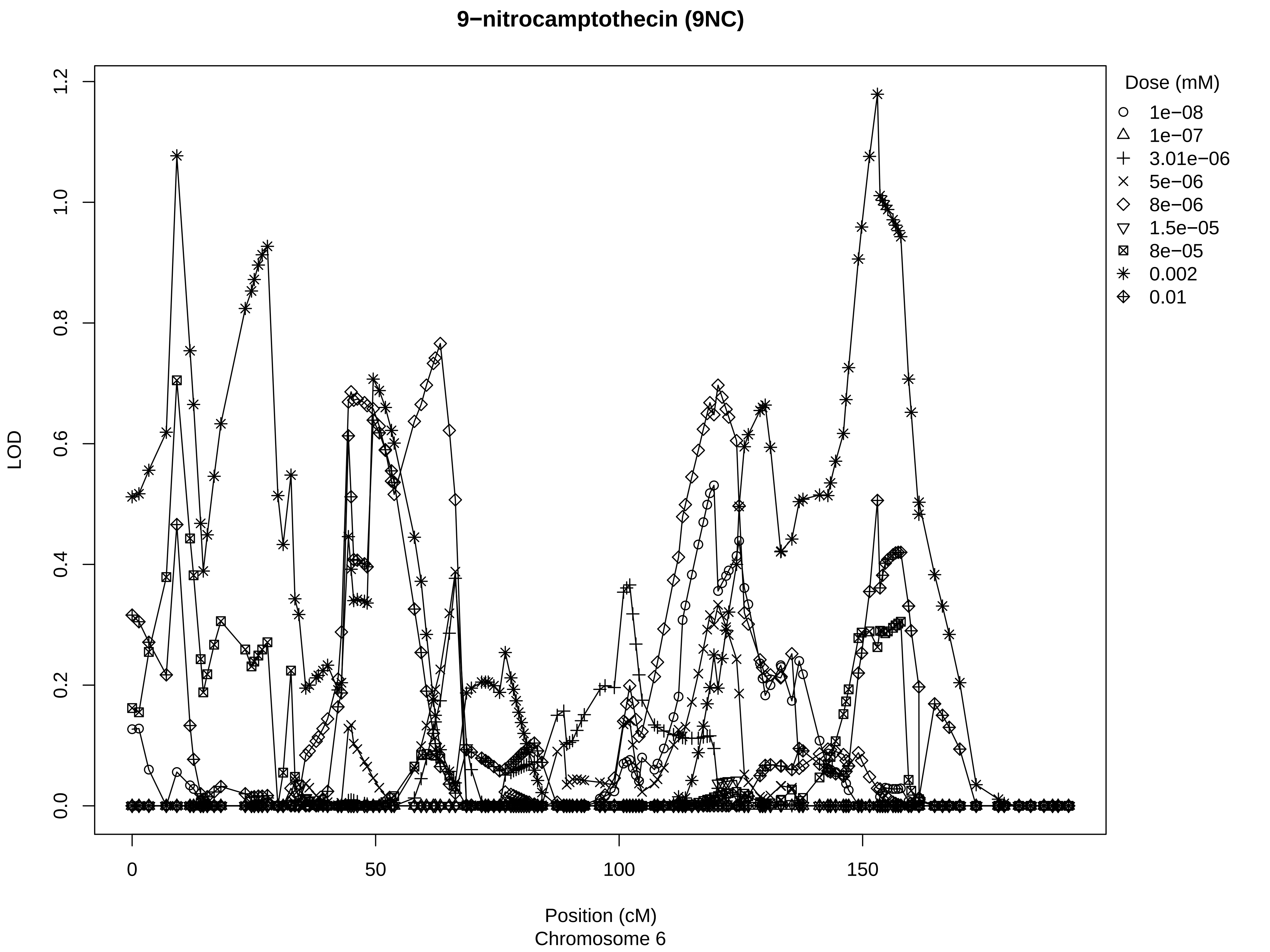
9−nitrocamptothecin (9NC)
LOD
Position (cM)
Chromosome 6
Dose (mM)
1e−08
1e−07
3.01e−06
5e−06
8e−06
1.5e−05
8e−05
0.002
0.01
0.0
0.2
0.4
0.6
0.8
1.0
1.2
0
50
100
150

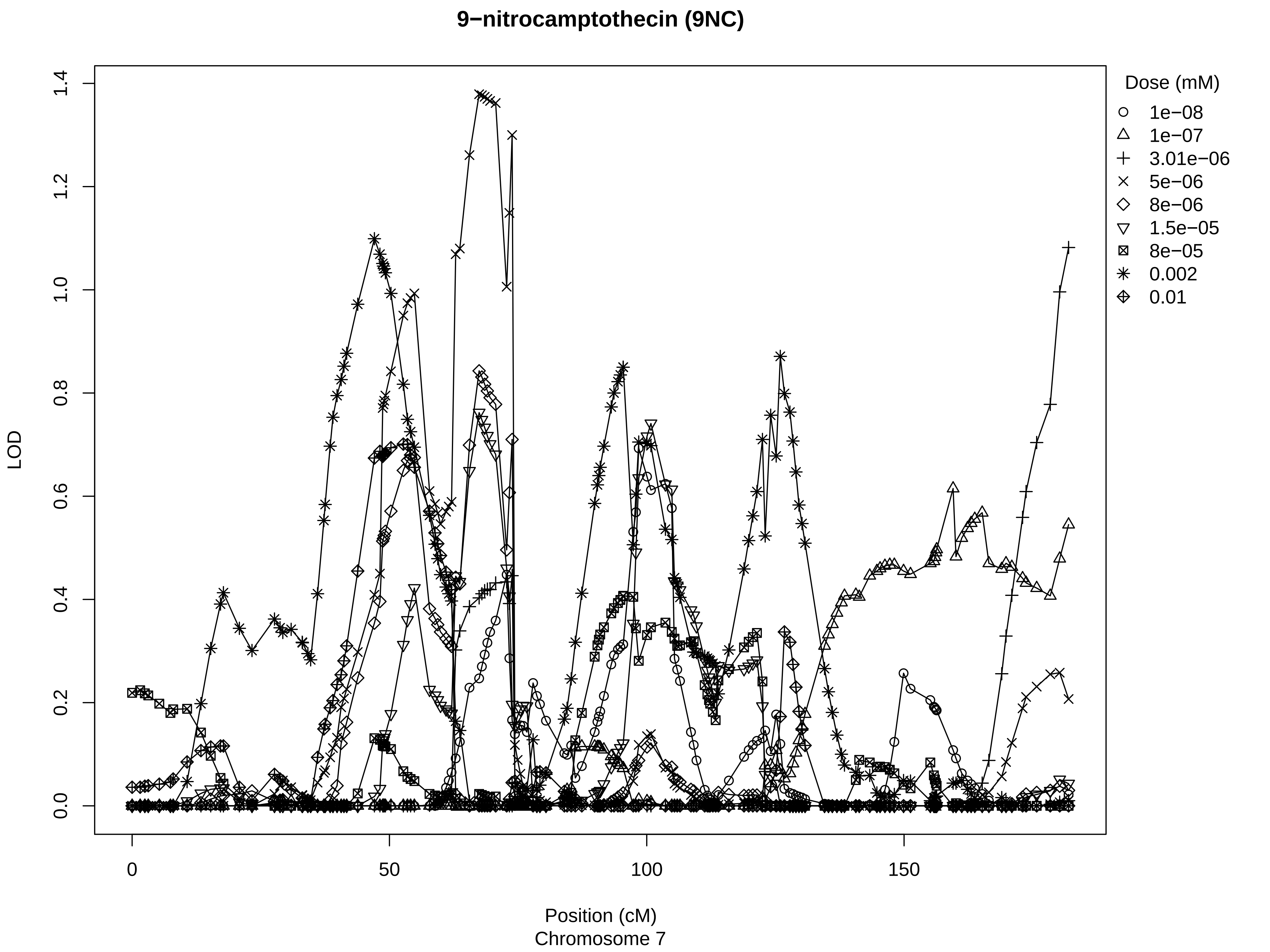
9−nitrocamptothecin (9NC)
LOD
0.0
0.2
0.4
0.6
0.8
1.0
1.2
1.4
0
50
100
150
Position (cM)
Chromosome 7
Dose (mM)
1e−08
1e−07
3.01e−06
5e−06
8e−06
1.5e−05
8e−05
0.002
0.01

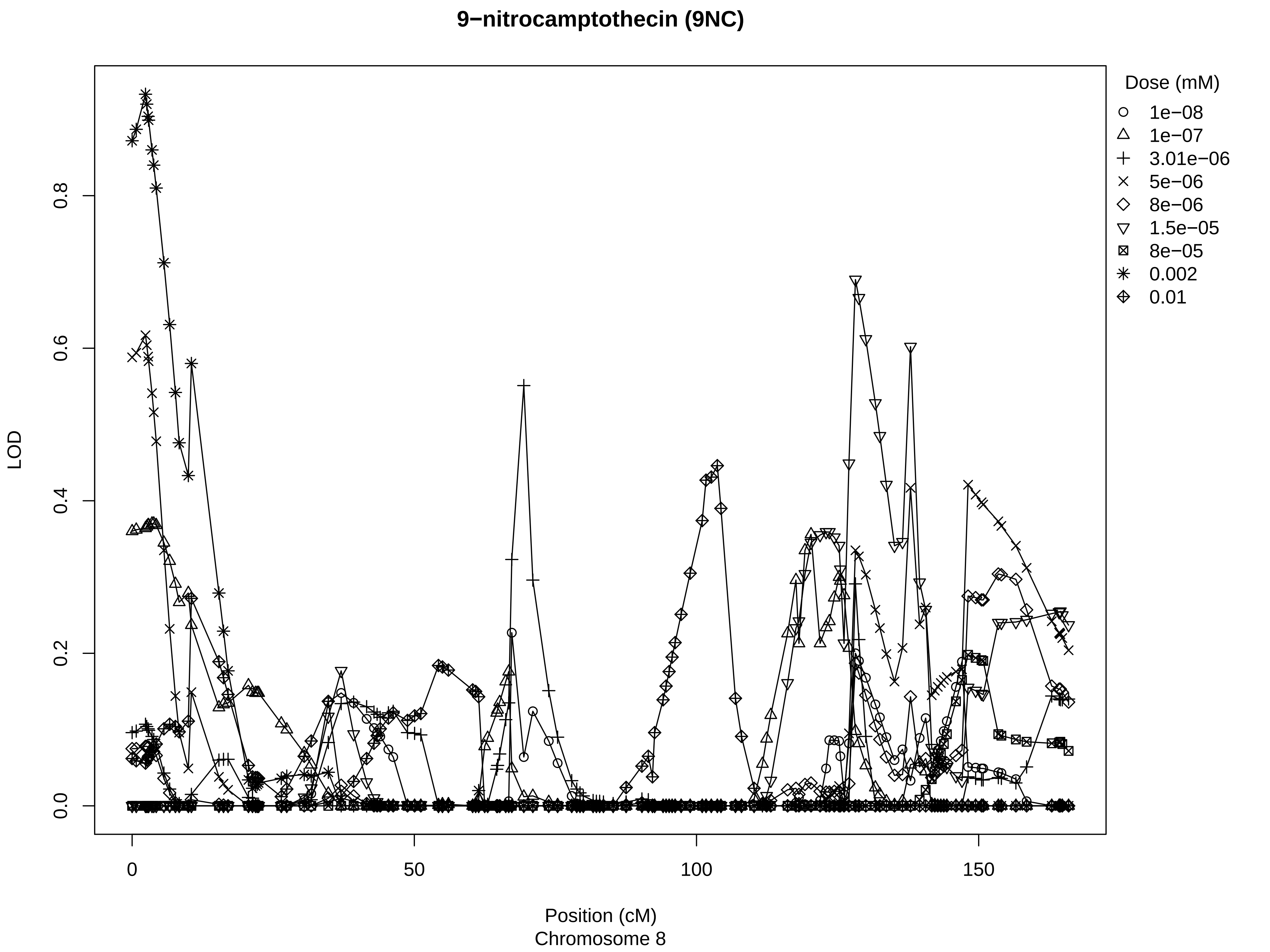
9−nitrocamptothecin (9NC)
LOD
0.0
0.2
0.4
0.6
0.8
0
50
100
150
Position (cM)
Chromosome 8
Dose (mM)
1e−08
1e−07
3.01e−06
5e−06
8e−06
1.5e−05
8e−05
0.002
0.01

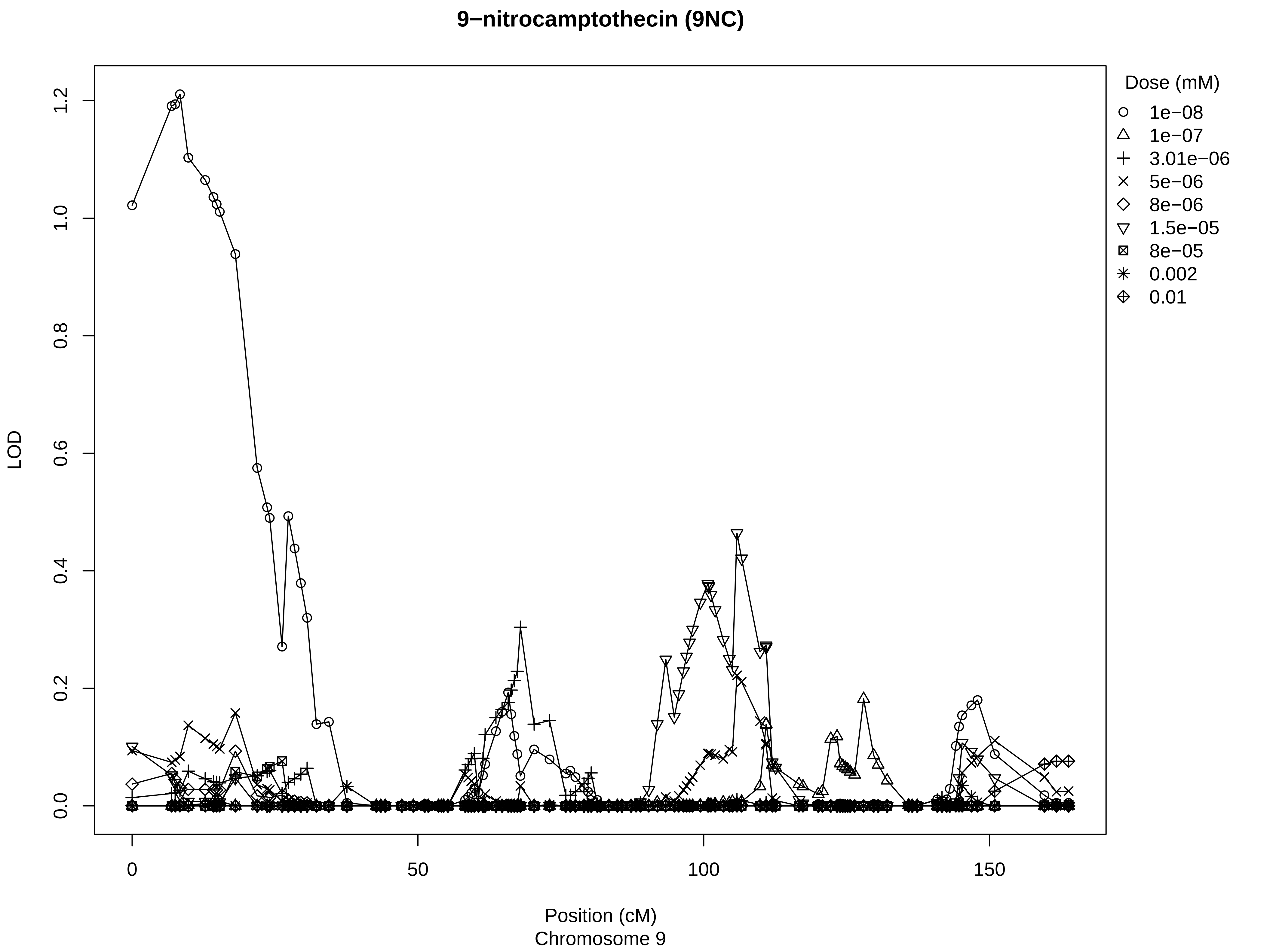
9−nitrocamptothecin (9NC)
Dose (mM)
1e−08
1e−07
3.01e−06
5e−06
8e−06
1.5e−05
8e−05
0.002
0.01
LOD
0.0
0.2
0.4
0.6
0.8
1.0
1.2
0
50
100
150
Position (cM)
Chromosome 9

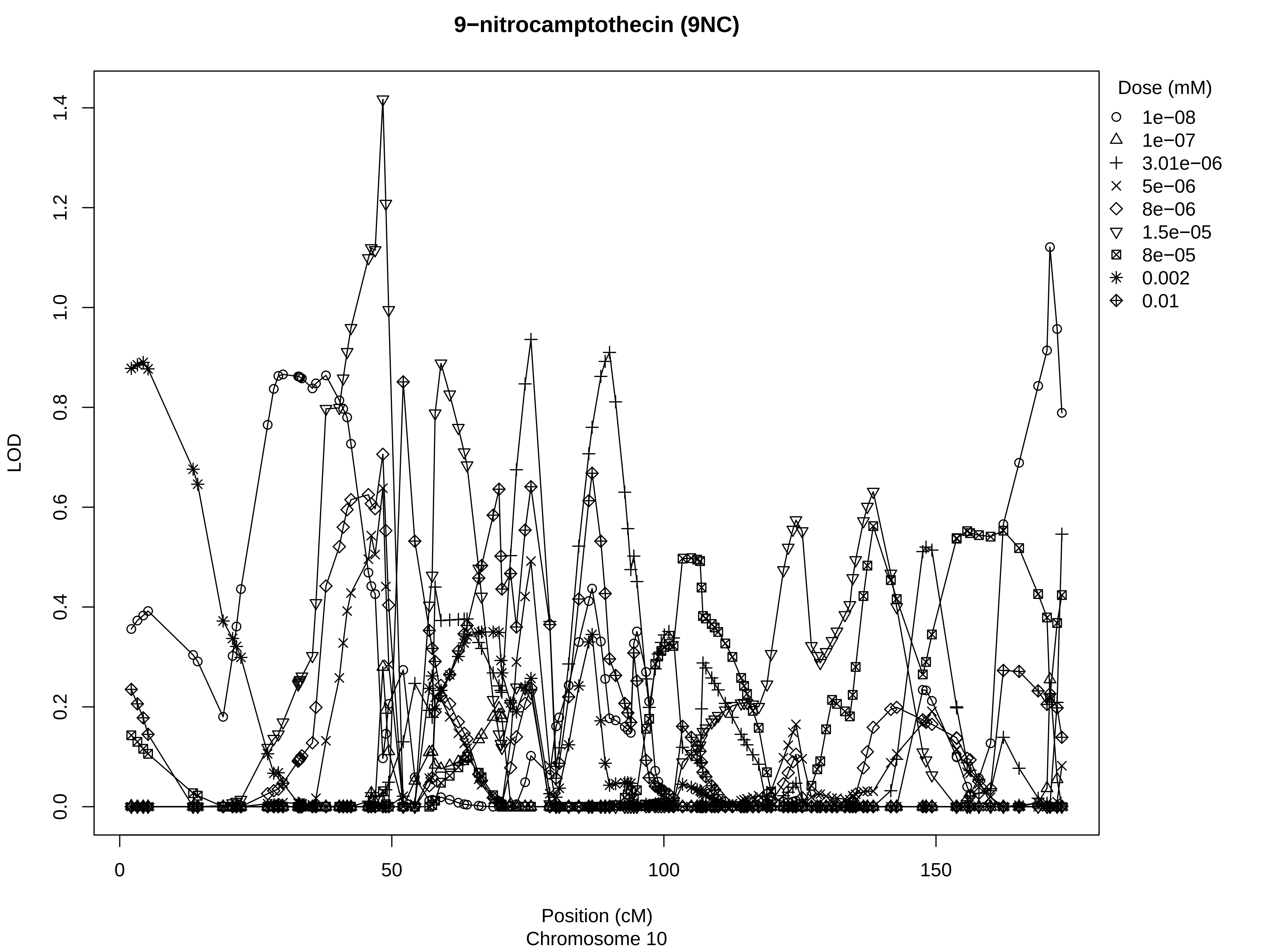
9−nitrocamptothecin (9NC)
LOD
Position (cM)
Chromosome 10
Dose (mM)
1e−08
1e−07
3.01e−06
5e−06
8e−06
1.5e−05
8e−05
0.002
0.01
0.0
0.2
0.4
0.6
0.8
1.0
1.2
1.4
0
50
100
150

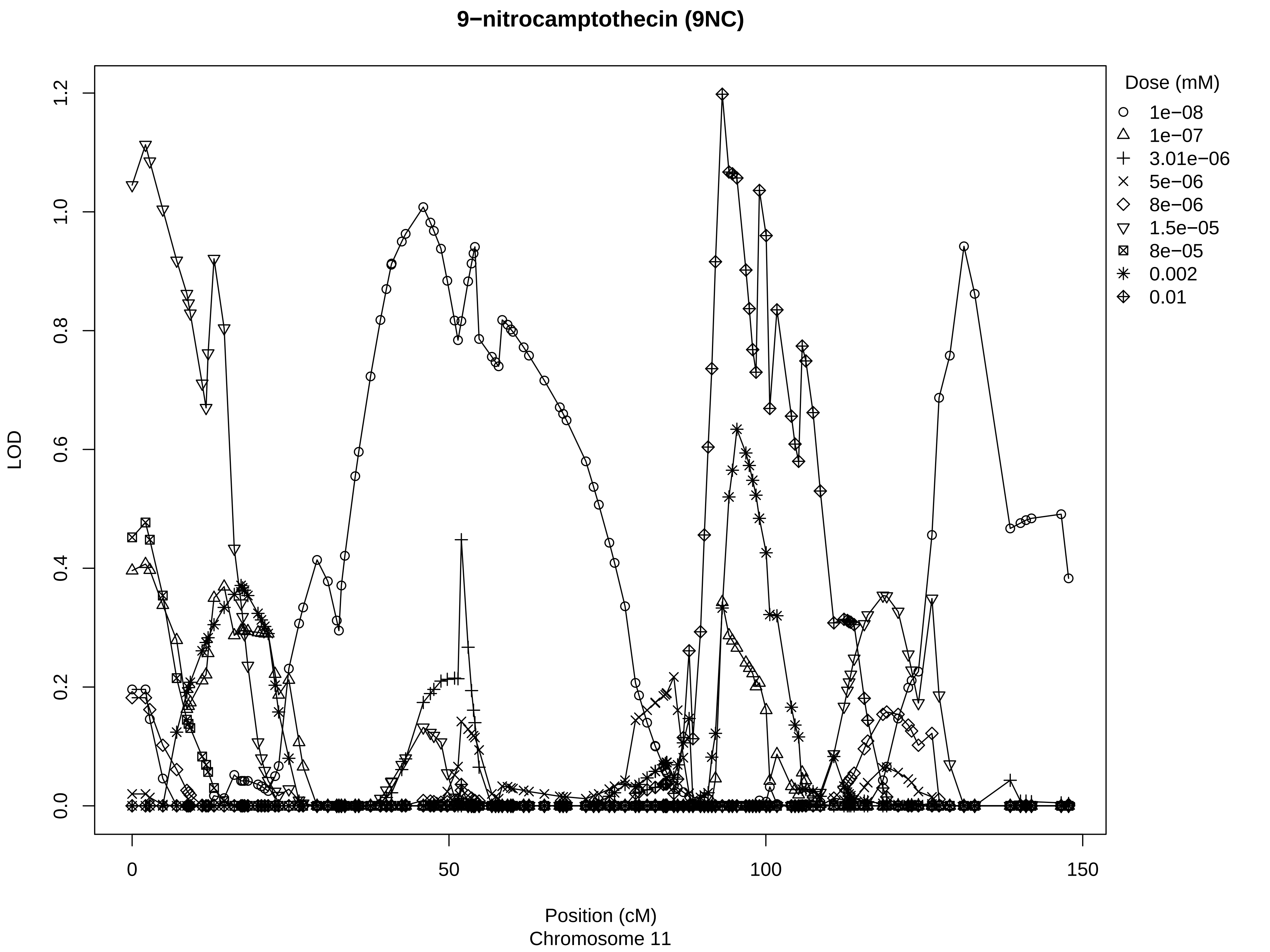

9−nitrocamptothecin (9NC)
LOD
Position (cM)
Chromosome 11
Dose (mM)
1e−08
1e−07
3.01e−06
5e−06
8e−06
1.5e−05
8e−05
0.002
0.01
0.0
0.2
0.4
0.6
0.8
1.0
1.2
0
50
100
150

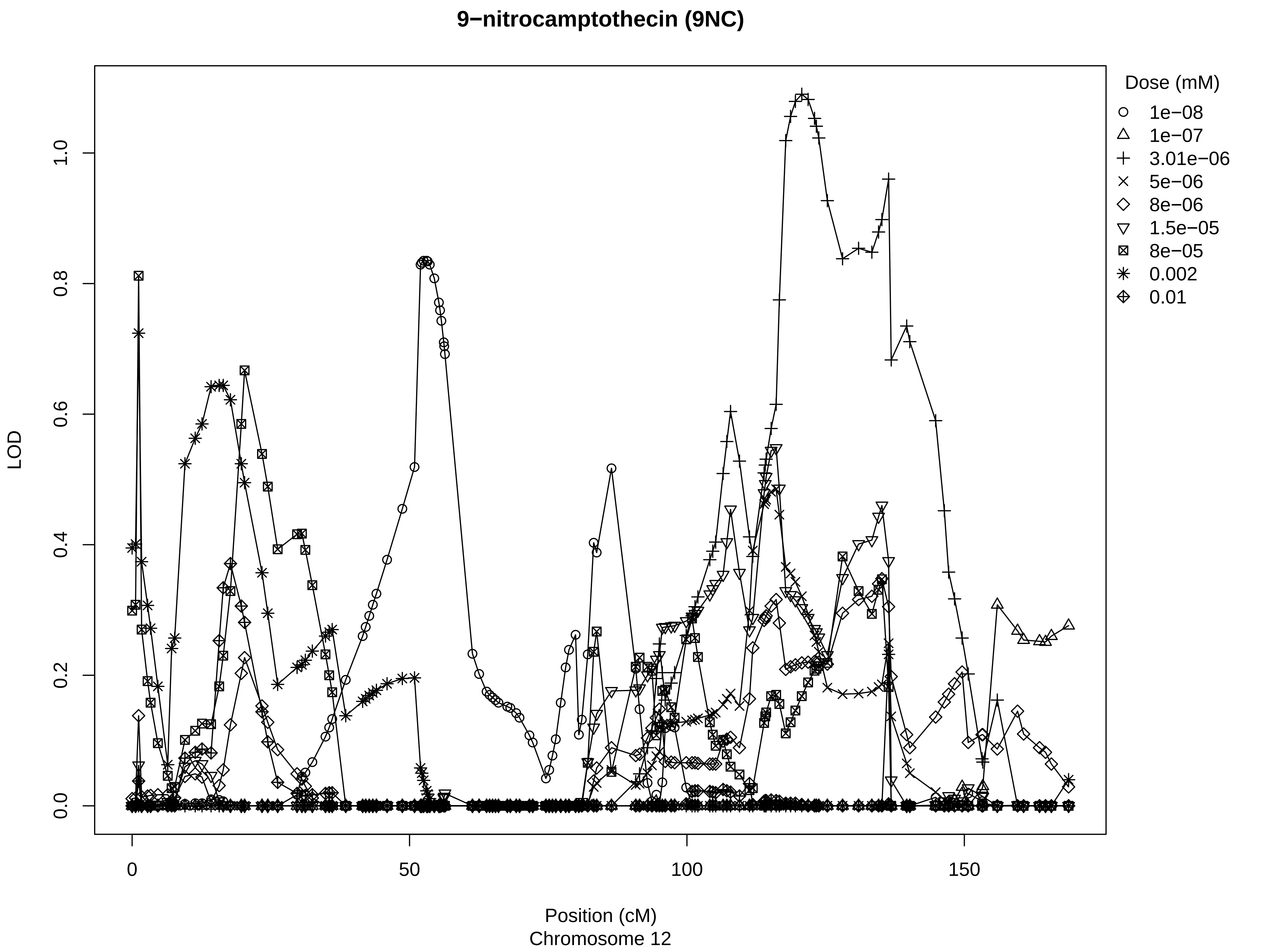

9−nitrocamptothecin (9NC)
Dose (mM)
1e−08
1e−07
3.01e−06
5e−06
8e−06
1.5e−05
8e−05
0.002
0.01
LOD
0.0
0.2
0.4
0.6
0.8
1.0
0
50
100
150
Position (cM)
Chromosome 12

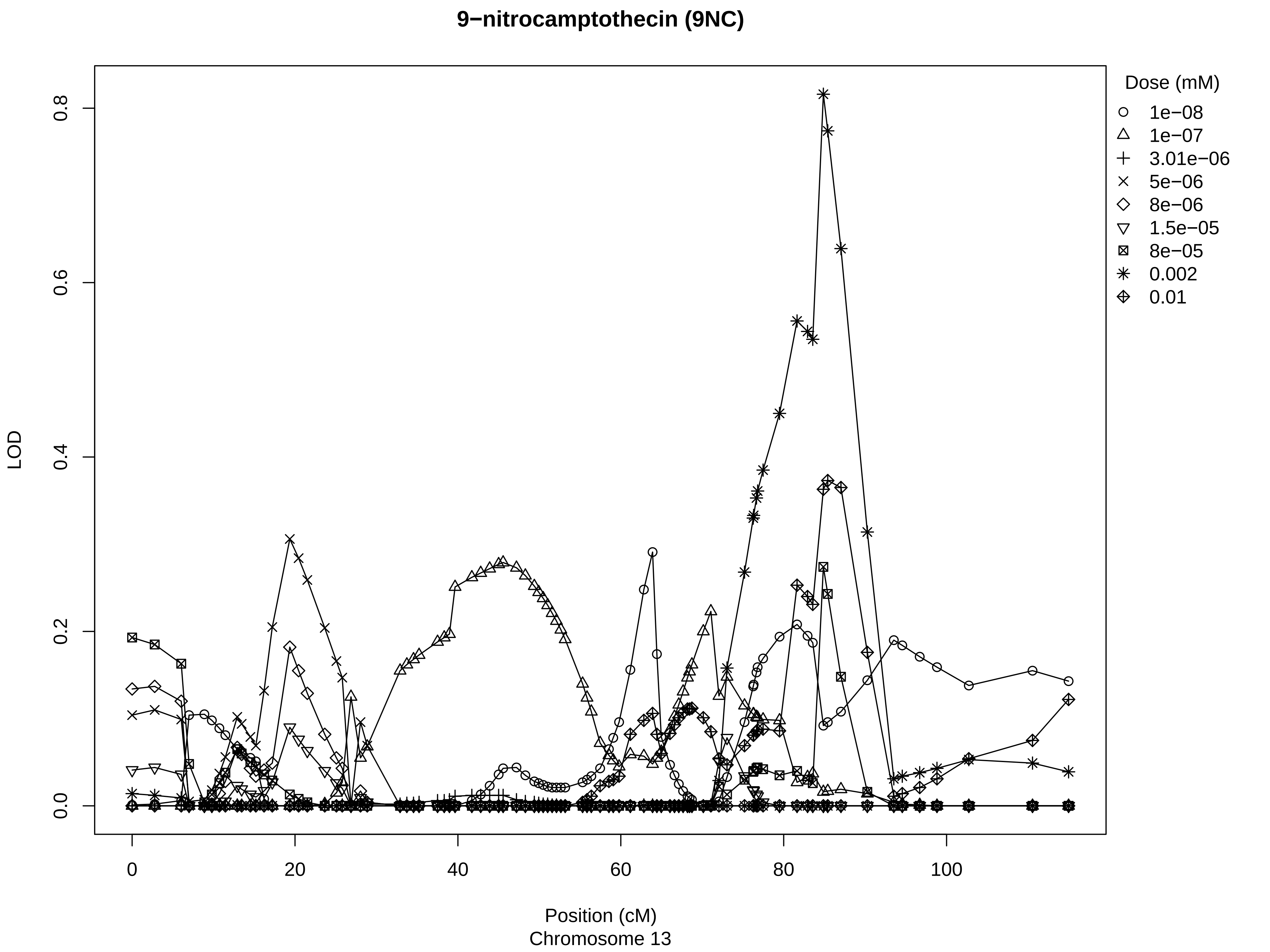
9−nitrocamptothecin (9NC)
LOD
0.0
0.2
0.4
0.6
0.8
0
20
40
60
80
100
Position (cM)
Chromosome 13
Dose (mM)
1e−08
1e−07
3.01e−06
5e−06
8e−06
1.5e−05
8e−05
0.002
0.01

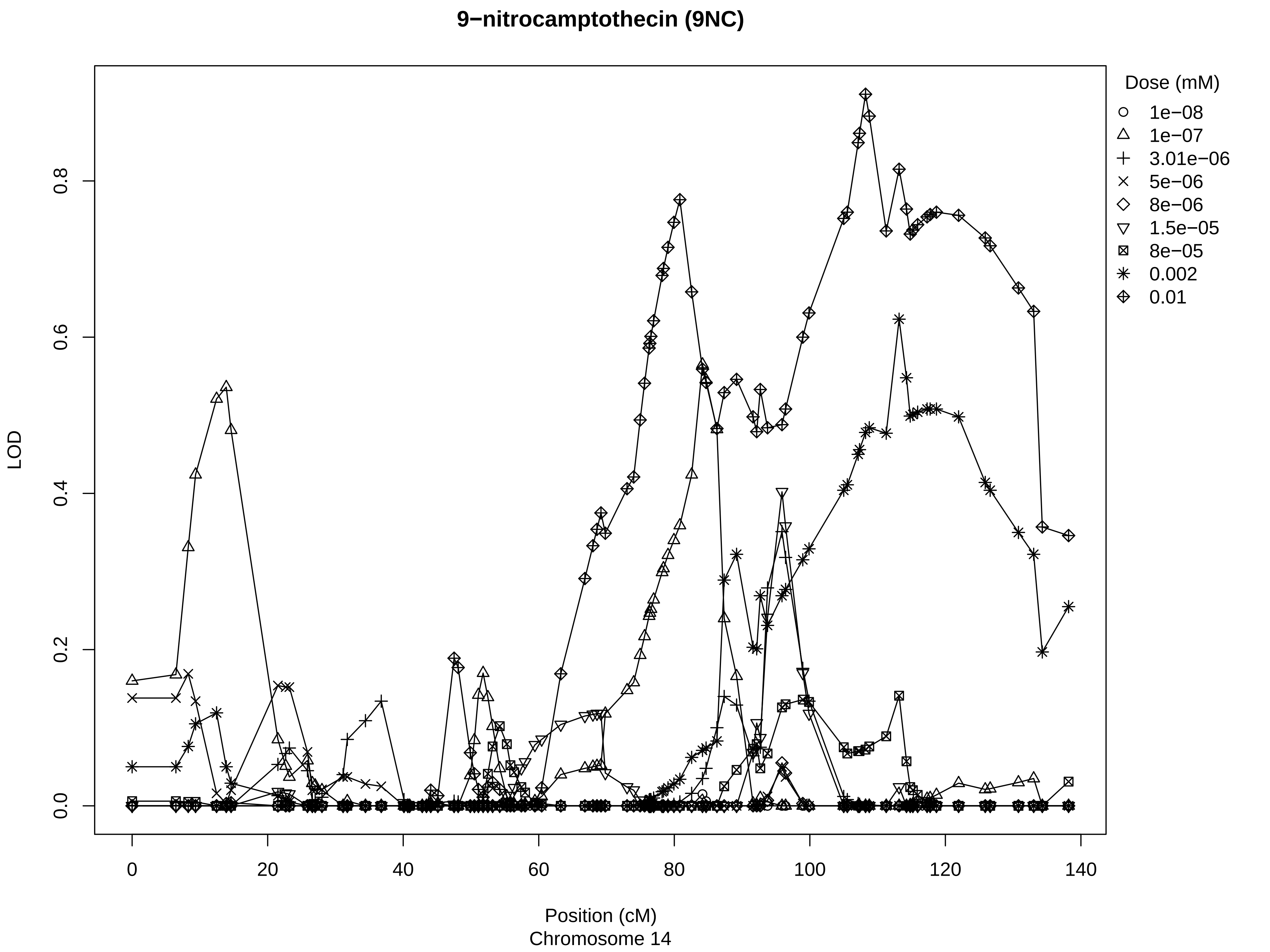
9−nitrocamptothecin (9NC)
LOD
0.0
0.2
0.4
0.6
0.8
0
20
40
60
80
100
120
140
Position (cM)
Chromosome 14
Dose (mM)
1e−08
1e−07
3.01e−06
5e−06
8e−06
1.5e−05
8e−05
0.002
0.01

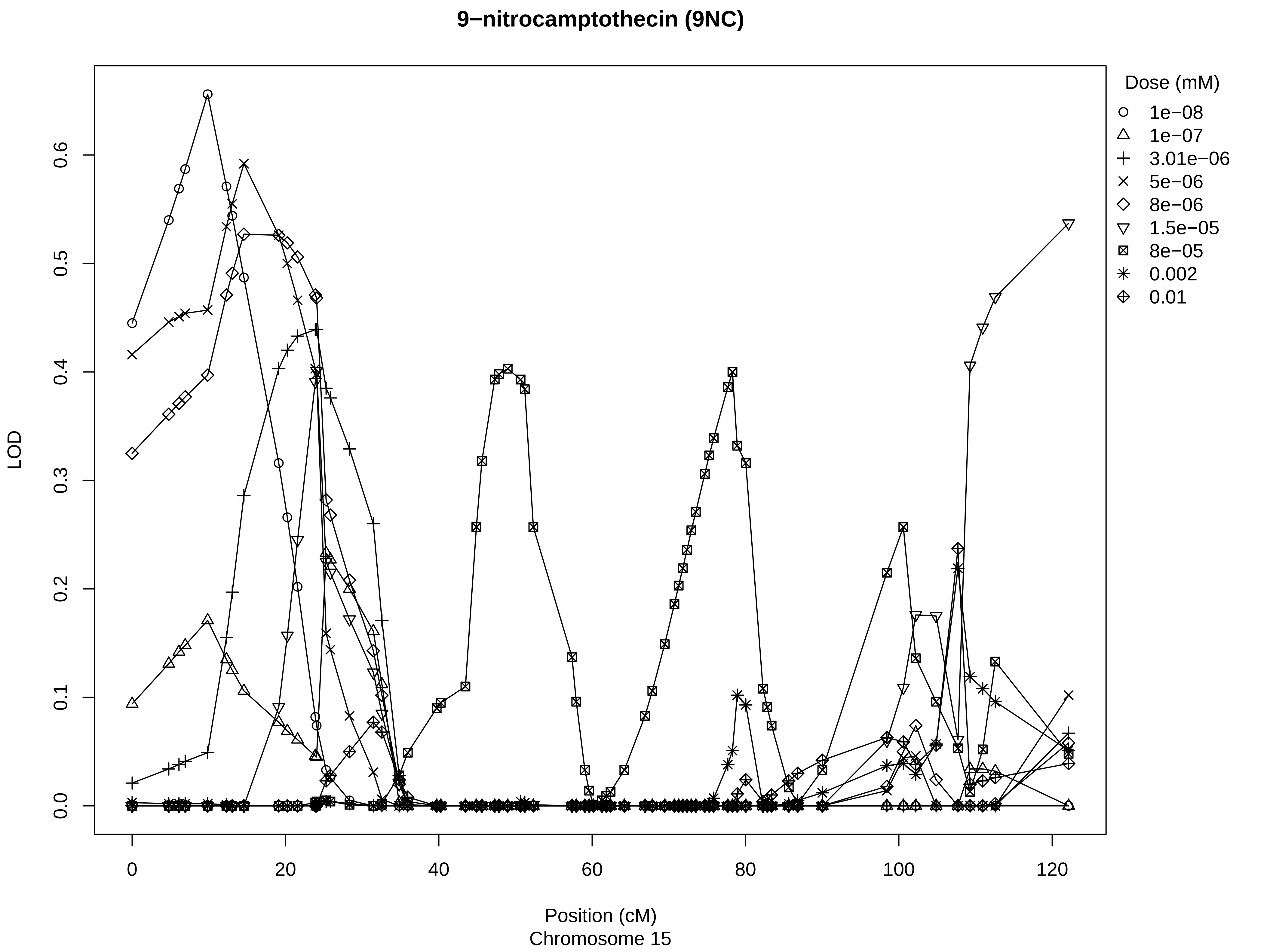
9−nitrocamptothecin (9NC)
LOD
0.0
0.1
0.2
0.3
0.4
0.5
0.6
0
20
40
60
80
100
120
Position (cM)
Chromosome 15
Dose (mM)
1e−08
1e−07
3.01e−06
5e−06
8e−06
1.5e−05
8e−05
0.002
0.01

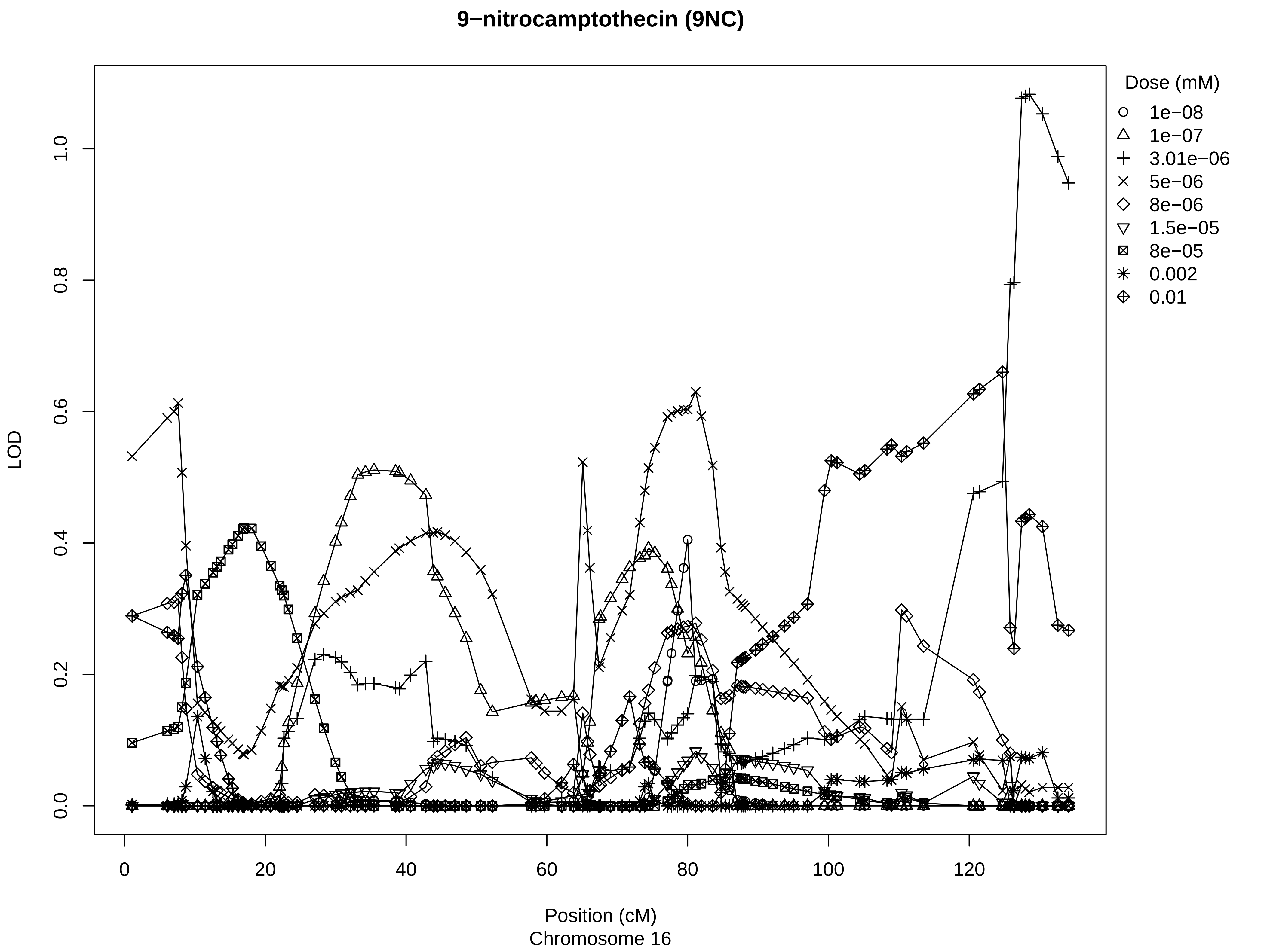
9−nitrocamptothecin (9NC)
LOD
0.0
0.2
0.4
0.6
0.8
1.0
0
20
40
60
80
100
120
Position (cM)
Chromosome 16
Dose (mM)
1e−08
1e−07
3.01e−06
5e−06
8e−06
1.5e−05
8e−05
0.002
0.01

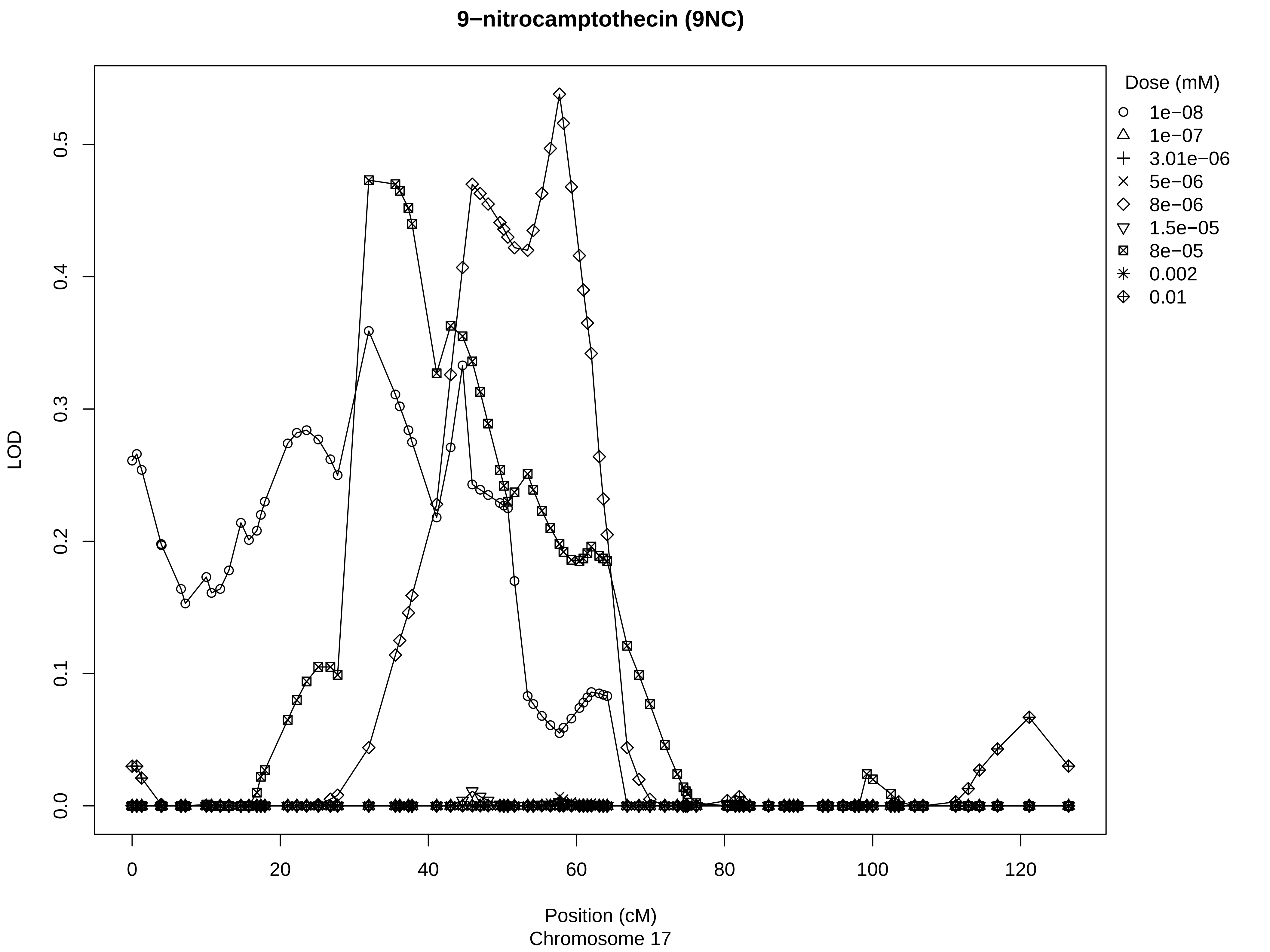
9−nitrocamptothecin (9NC)
LOD
Position (cM)
Chromosome 17
Dose (mM)
1e−08
1e−07
3.01e−06
5e−06
8e−06
1.5e−05
8e−05
0.002
0.01
0.0
0.1
0.2
0.3
0.4
0.5
0
20
40
60
80
100
120

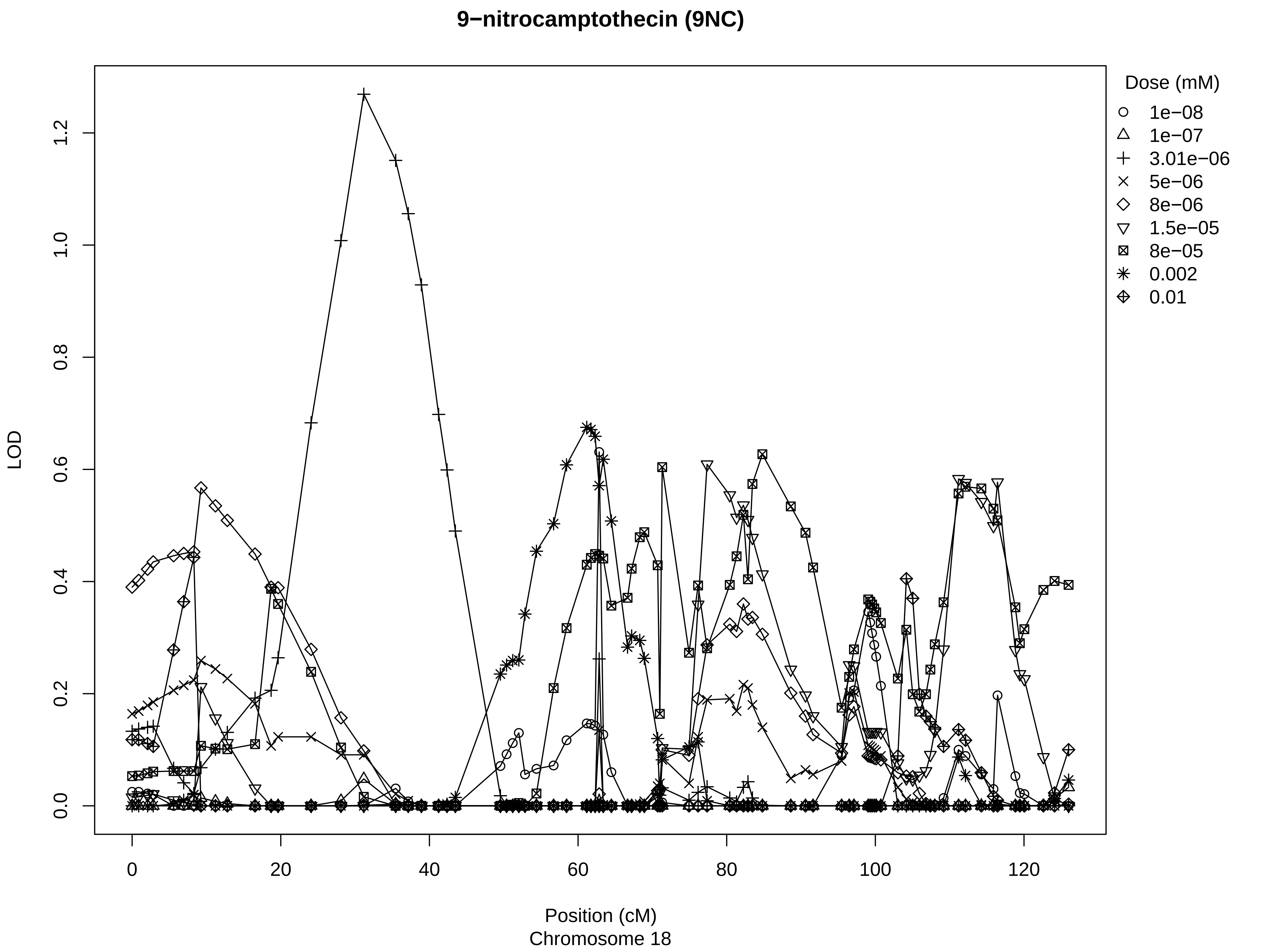

9−nitrocamptothecin (9NC)
LOD
0.0
0.2
0.4
0.6
0.8
1.0
1.2
0
20
40
60
80
100
120
Position (cM)
Chromosome 18
Dose (mM)
1e−08
1e−07
3.01e−06
5e−06
8e−06
1.5e−05
8e−05
0.002
0.01

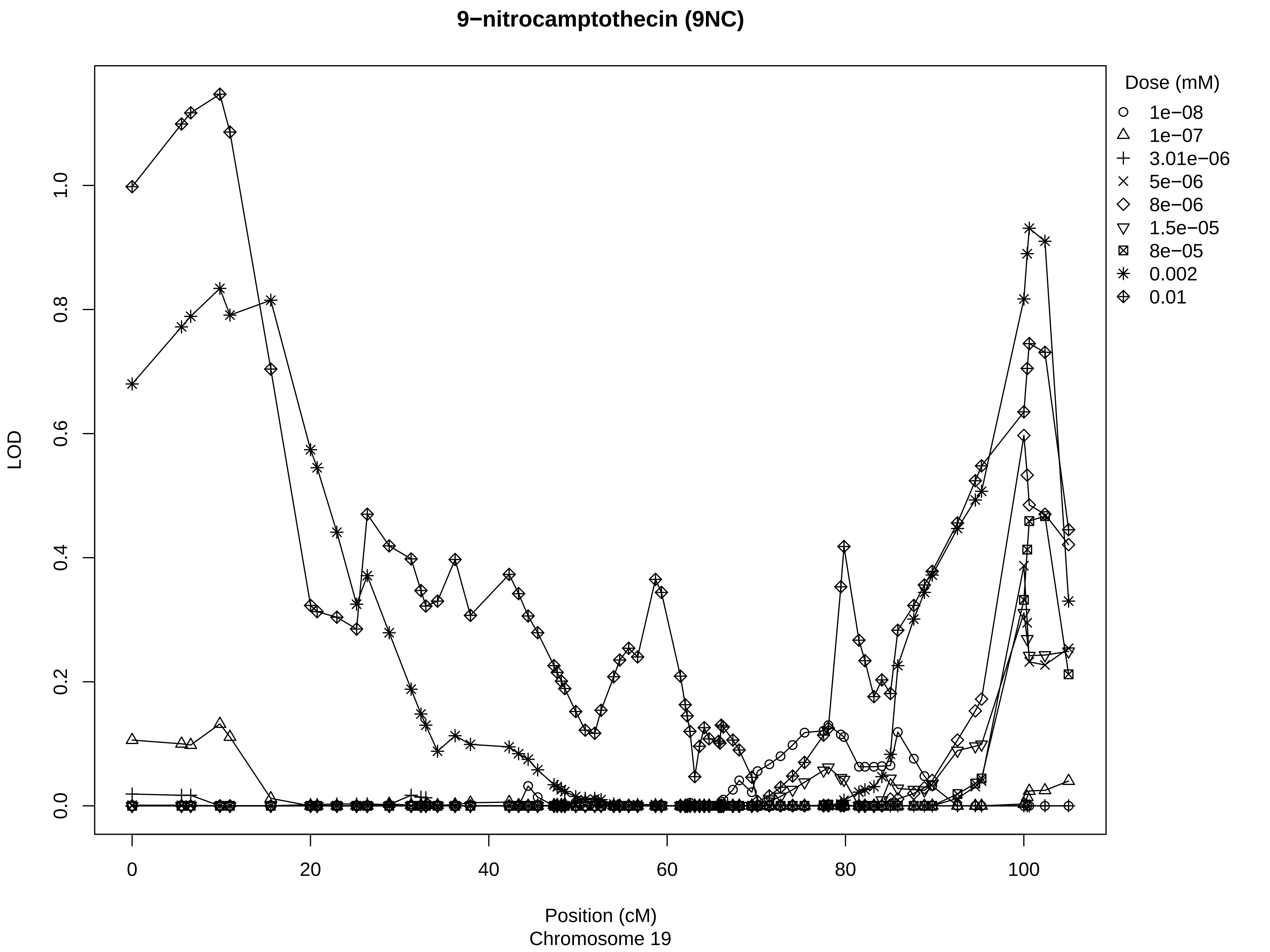

9−nitrocamptothecin (9NC)
Dose (mM)
1e−08
1e−07
3.01e−06
5e−06
8e−06
1.5e−05
8e−05
0.002
0.01
LOD
0.0
0.2
0.4
0.6
0.8
1.0
0
20
40
60
80
100
Position (cM)
Chromosome 19

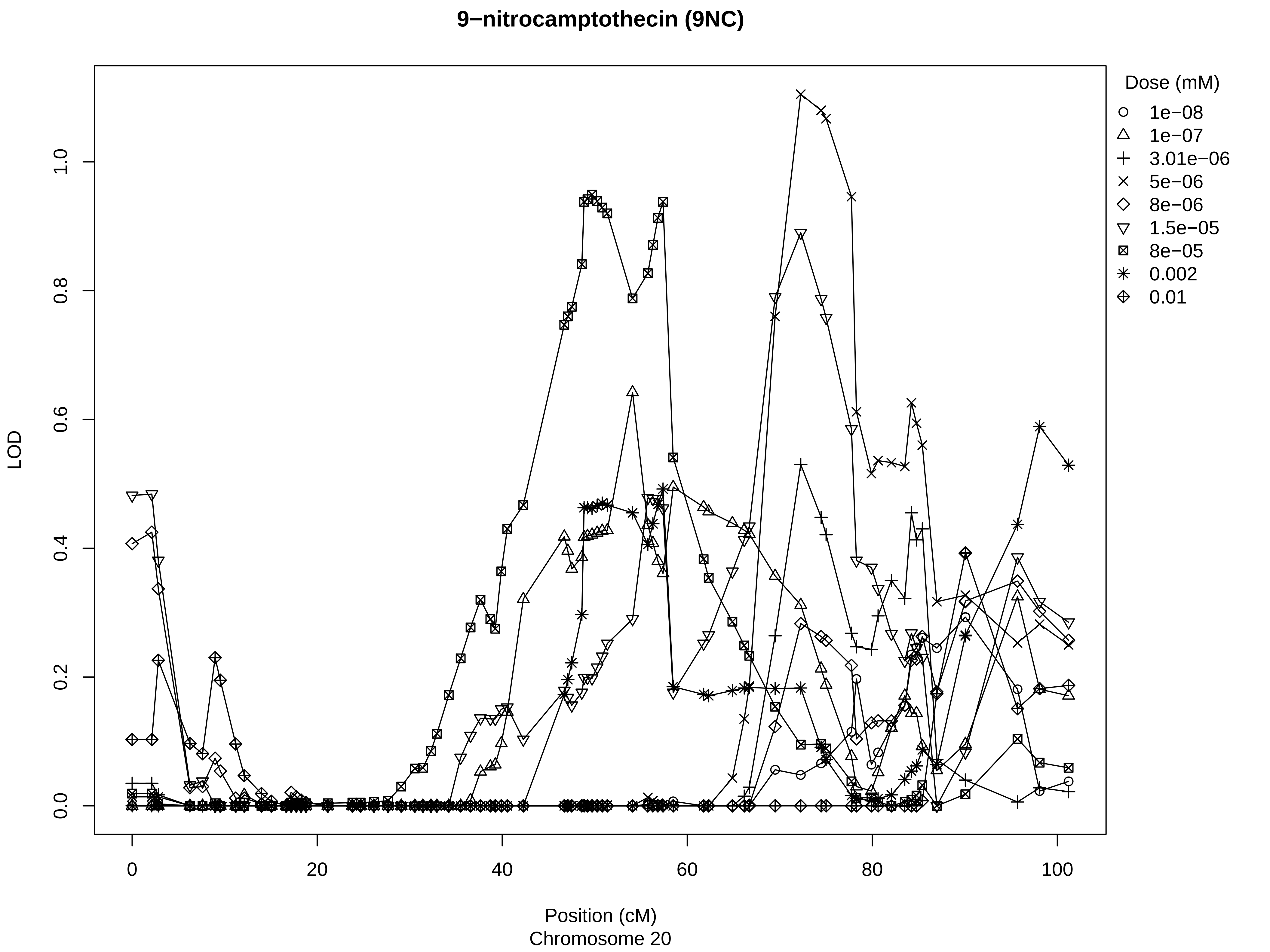

9−nitrocamptothecin (9NC)
Dose (mM)
1e−08
1e−07
3.01e−06
5e−06
8e−06
1.5e−05
8e−05
0.002
0.01
LOD
0.0
0.2
0.4
0.6
0.8
1.0
0
20
40
60
80
100
Position (cM)
Chromosome 20

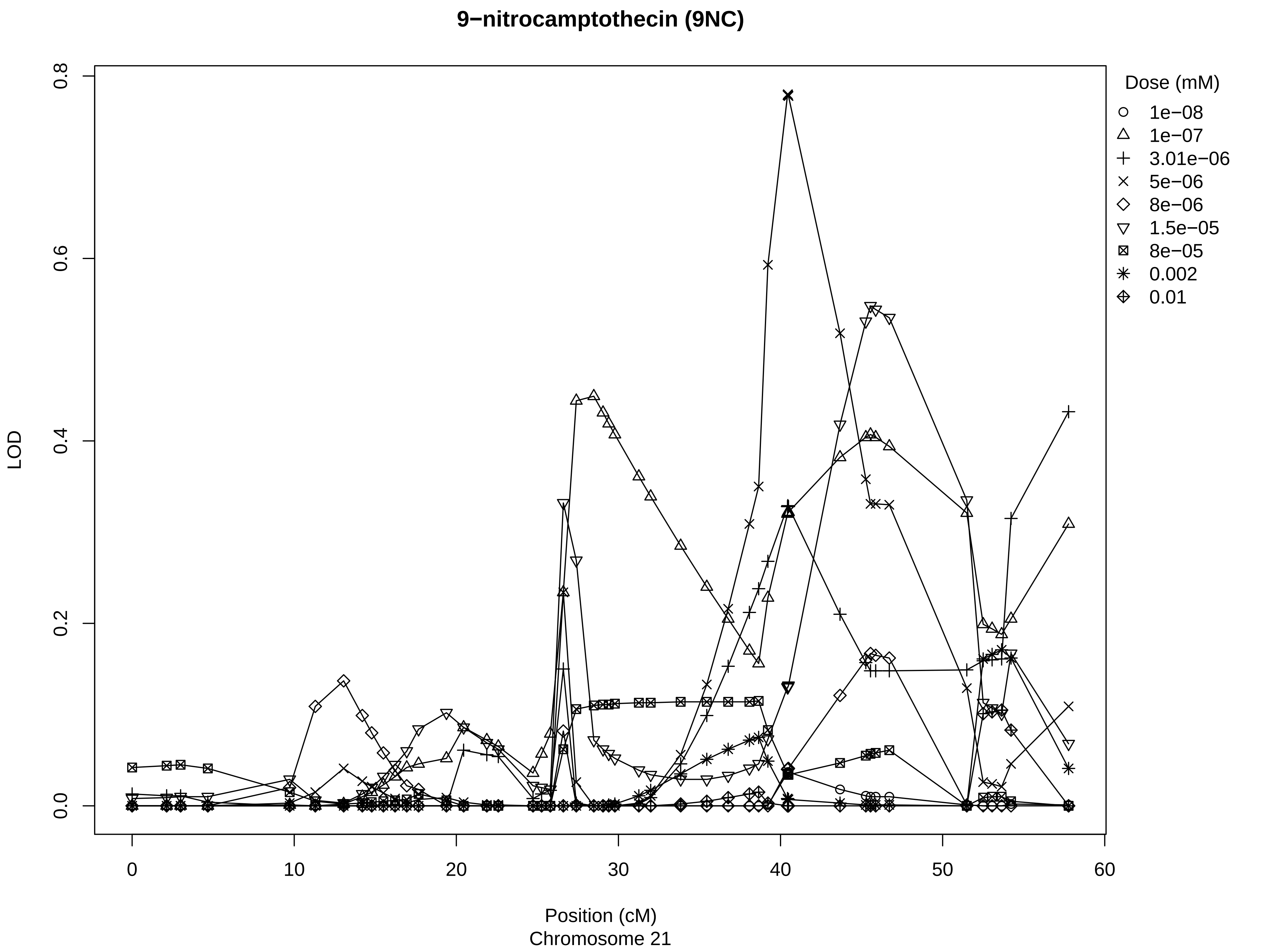

9−nitrocamptothecin (9NC)
LOD
0.0
0.2
0.4
0.6
0.8
0
10
20
30
40
50
60
Position (cM)
Chromosome 21
Dose (mM)
1e−08
1e−07
3.01e−06
5e−06
8e−06
1.5e−05
8e−05
0.002
0.01

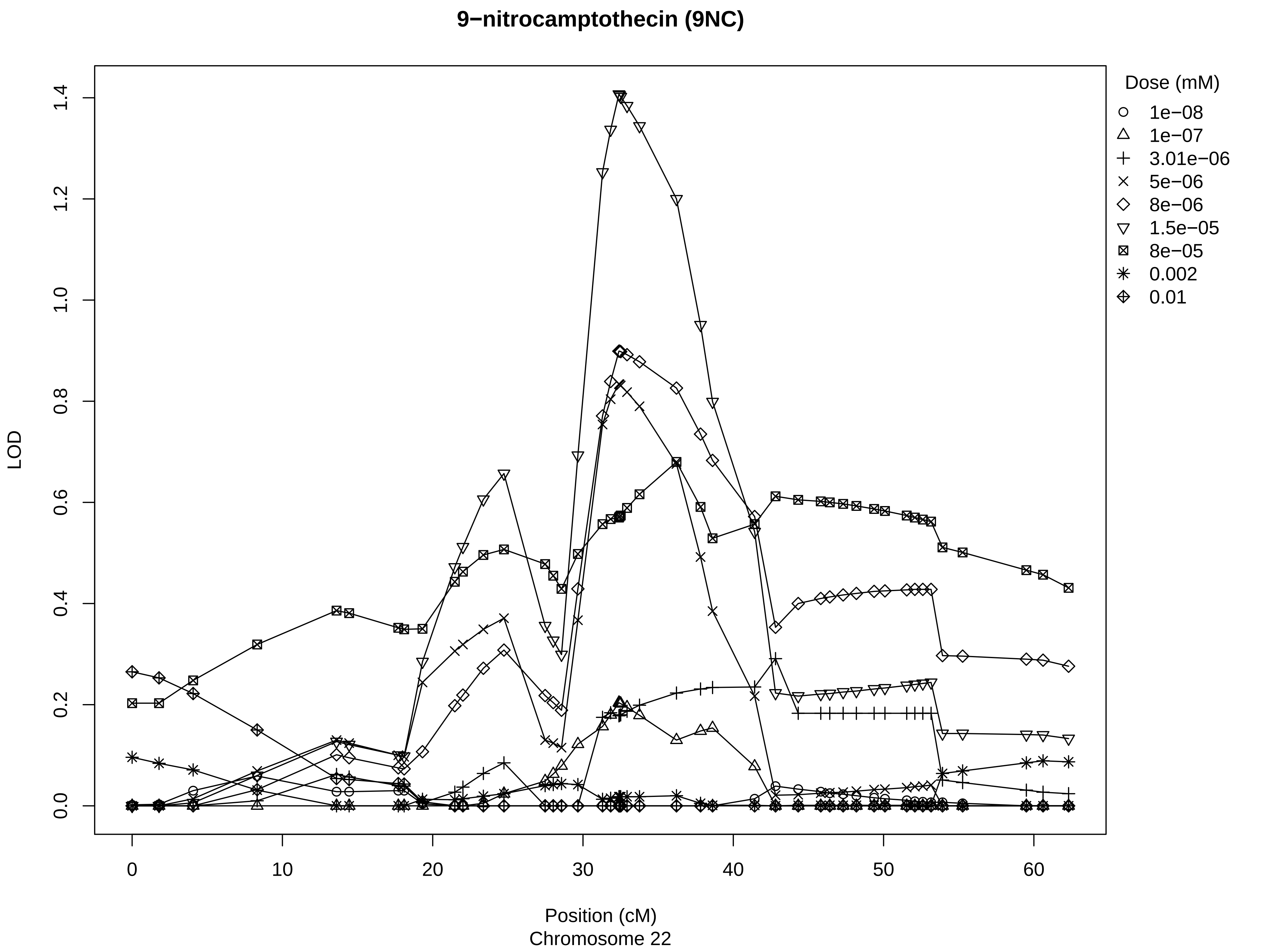
9−nitrocamptothecin (9NC)
Dose (mM)
1e−08
1e−07
3.01e−06
5e−06
8e−06
1.5e−05
8e−05
0.002
0.01
LOD
0.0
0.2
0.4
0.6
0.8
1.0
1.2
1.4
0
10
20
30
40
50
60
Position (cM)
Chromosome 22

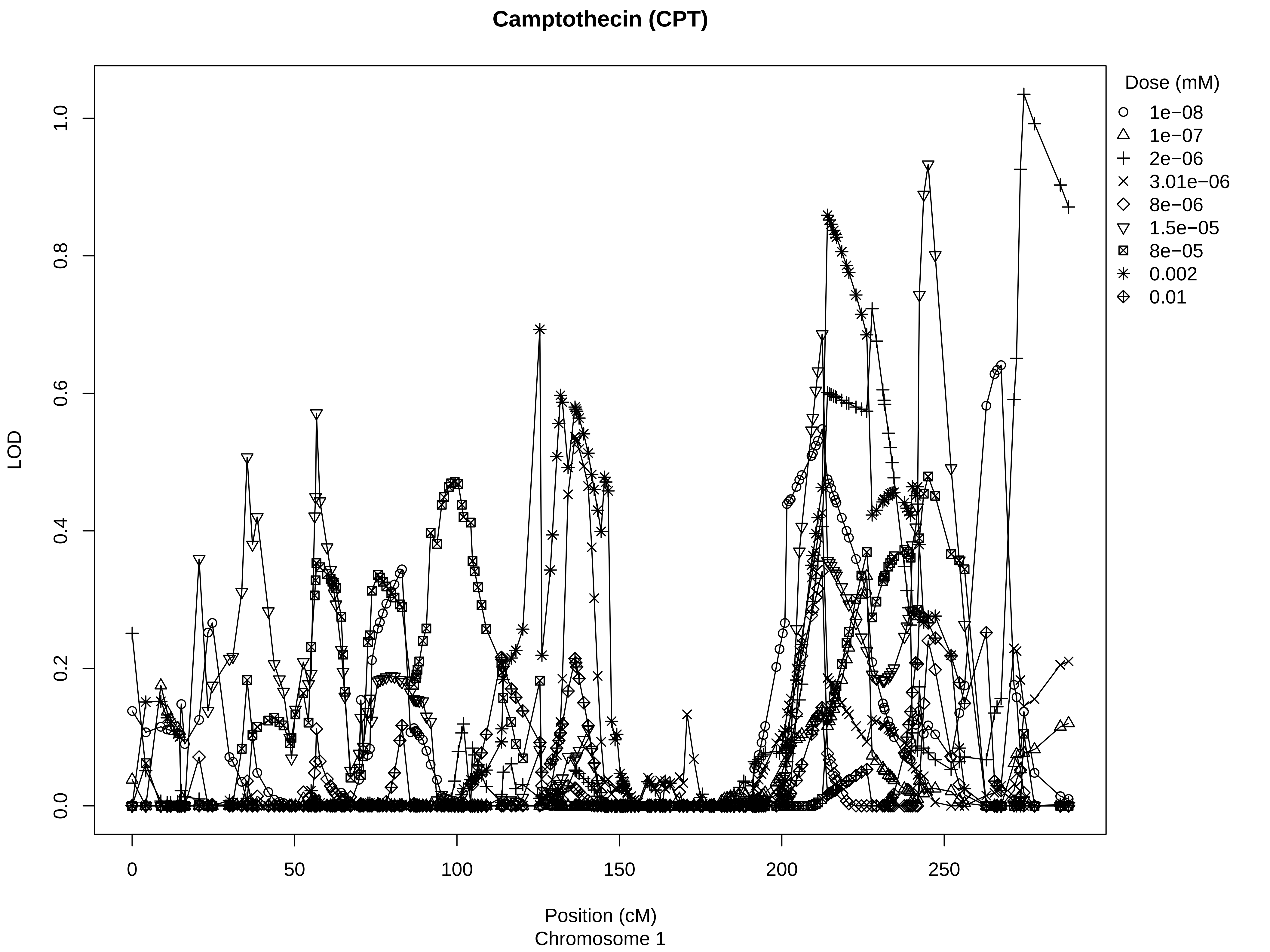

Camptothecin (CPT)
Dose (mM)
1e−08
1e−07
2e−06
3.01e−06
8e−06
1.5e−05
8e−05
0.002
0.01
LOD
0.0
0.2
0.4
0.6
0.8
1.0
0
50
100
150
200
250
Position (cM)
Chromosome 1

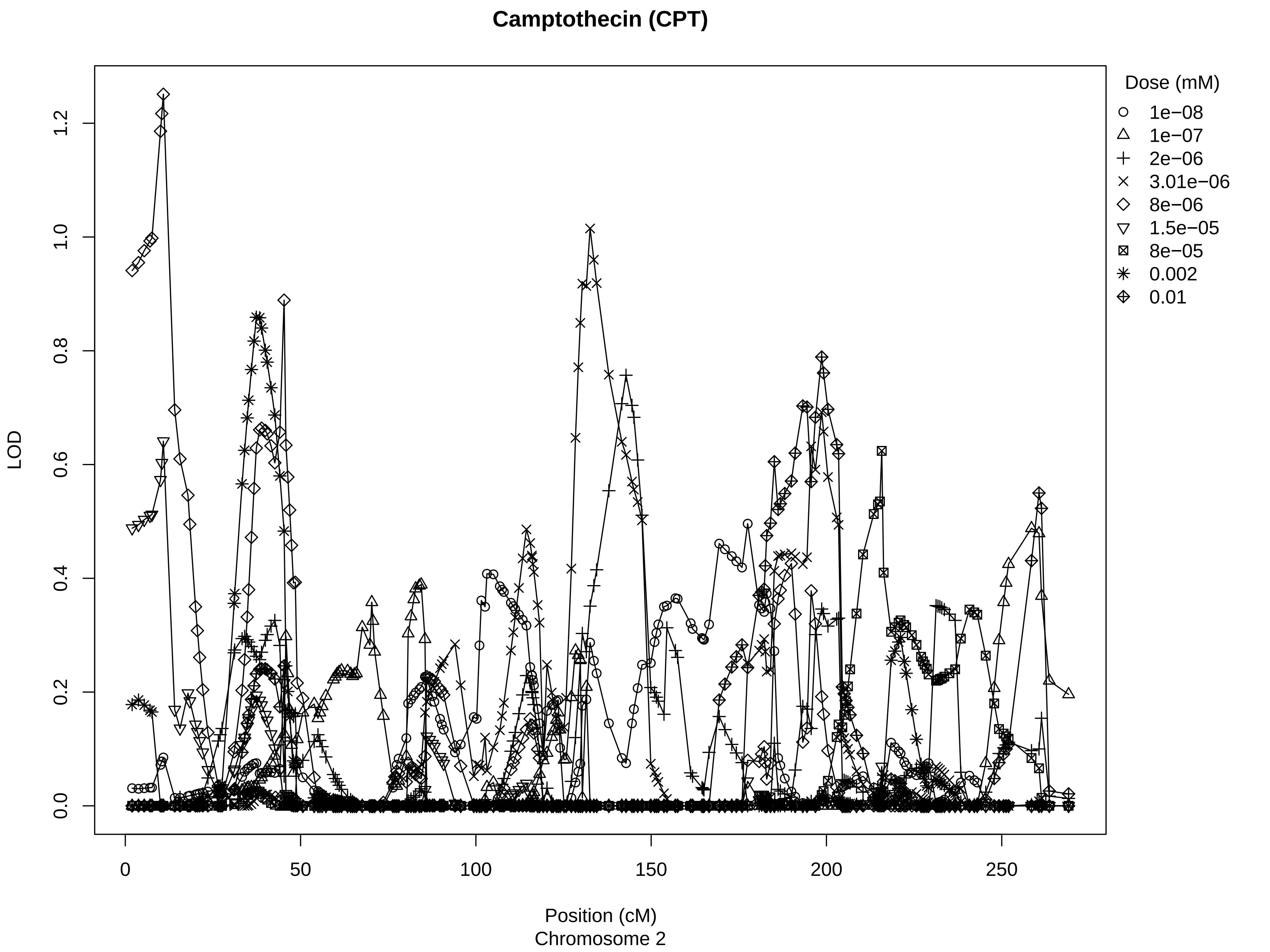
Camptothecin (CPT)
LOD
Position (cM)
Chromosome 2
Dose (mM)
1e−08
1e−07
2e−06
3.01e−06
8e−06
1.5e−05
8e−05
0.002
0.01
0.0
0.2
0.4
0.6
0.8
1.0
1.2
0
50
100
150
200
250

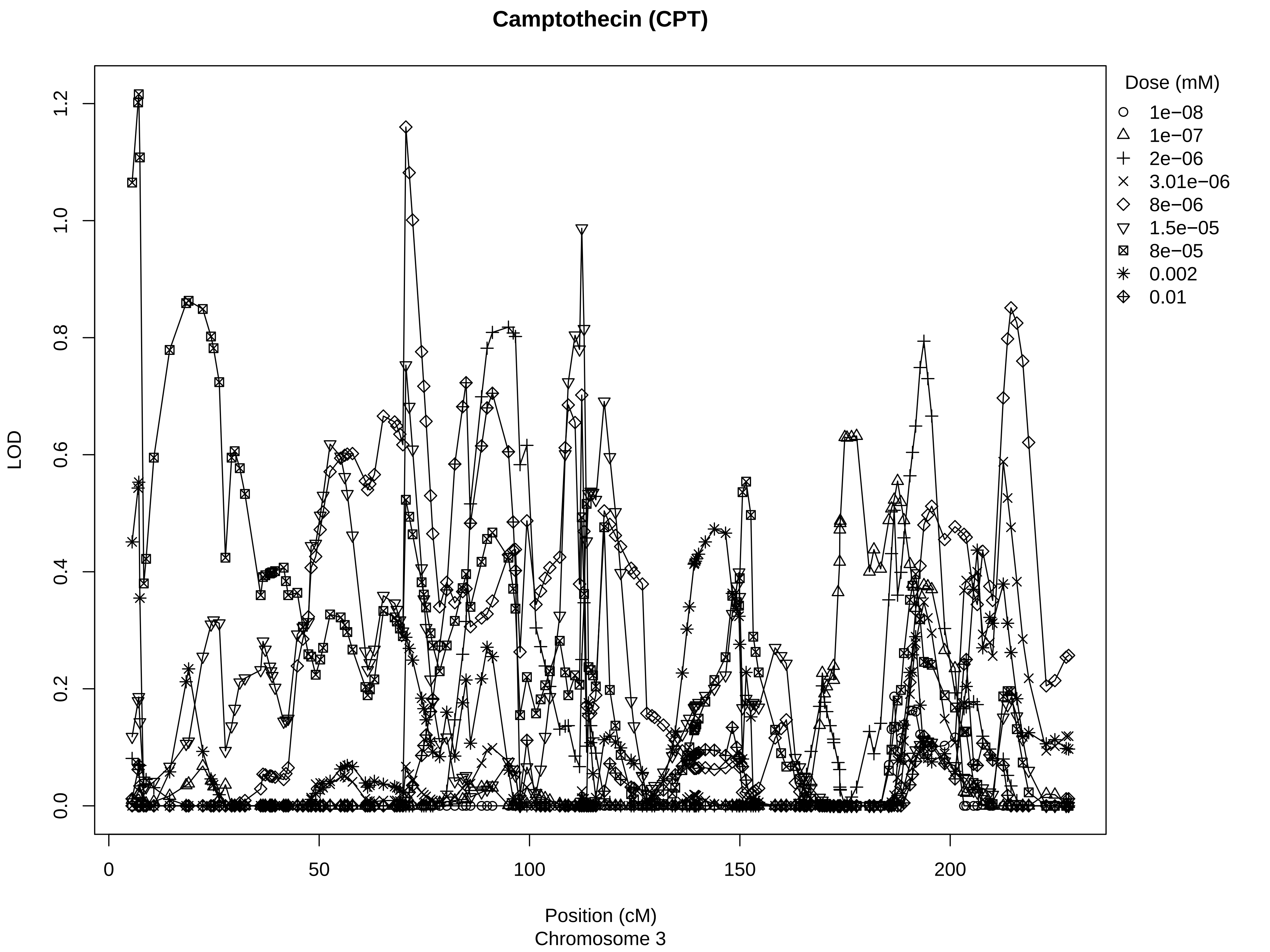

Camptothecin (CPT)
Dose (mM)
1e−08
1e−07
2e−06
3.01e−06
8e−06
1.5e−05
8e−05
0.002
0.01
LOD
0.0
0.2
0.4
0.6
0.8
1.0
1.2
0
50
100
150
200
Position (cM)
Chromosome 3

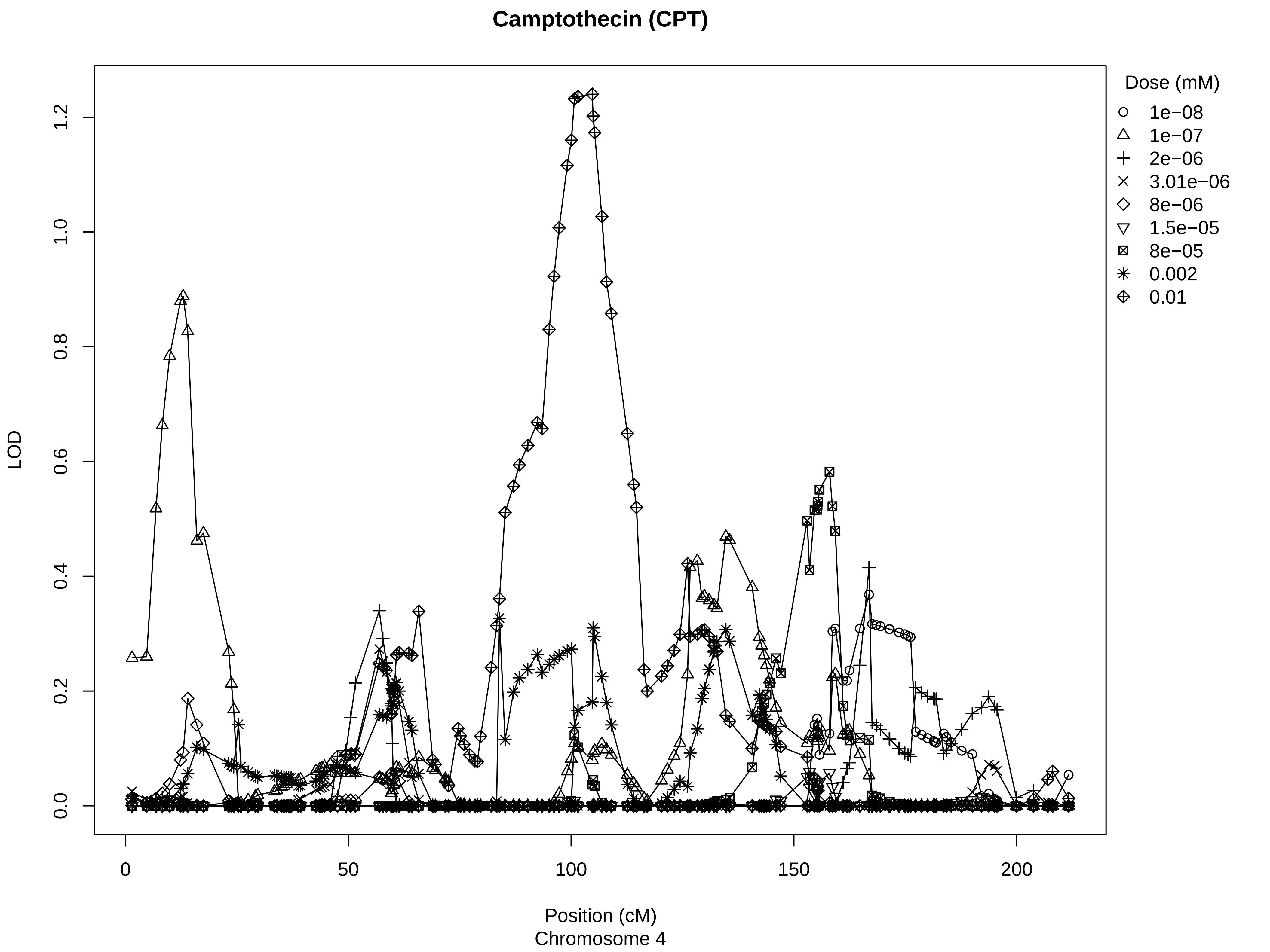
Camptothecin (CPT)
LOD
Position (cM)
Chromosome 4
Dose (mM)
1e−08
1e−07
2e−06
3.01e−06
8e−06
1.5e−05
8e−05
0.002
0.01
0.0
0.2
0.4
0.6
0.8
1.0
1.2
0
50
100
150
200

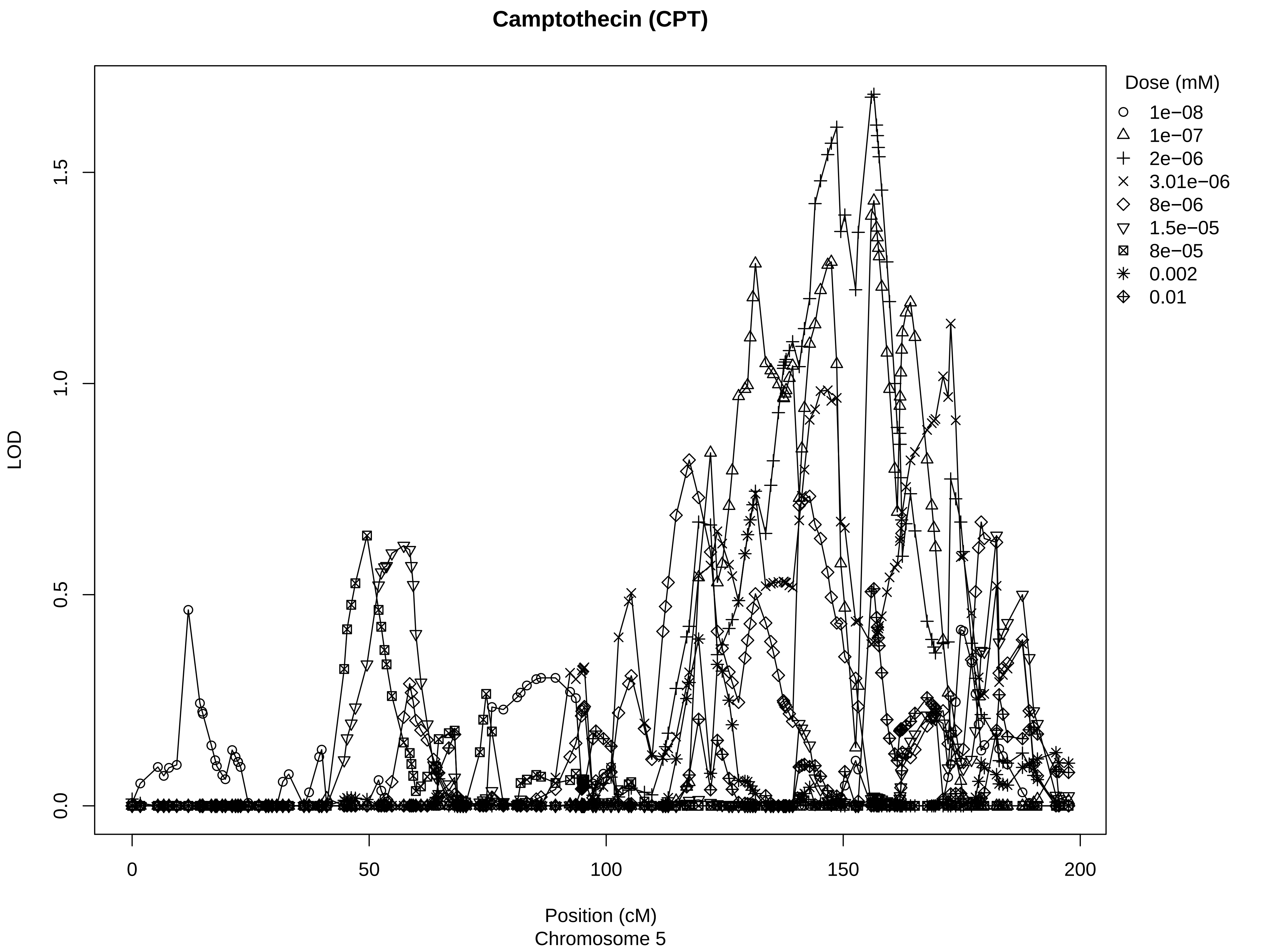

Camptothecin (CPT)
Dose (mM)
1e−08
1e−07
2e−06
3.01e−06
8e−06
1.5e−05
8e−05
0.002
0.01
LOD
0.0
0.5
1.0
1.5
0
50
100
150
200
Position (cM)
Chromosome 5

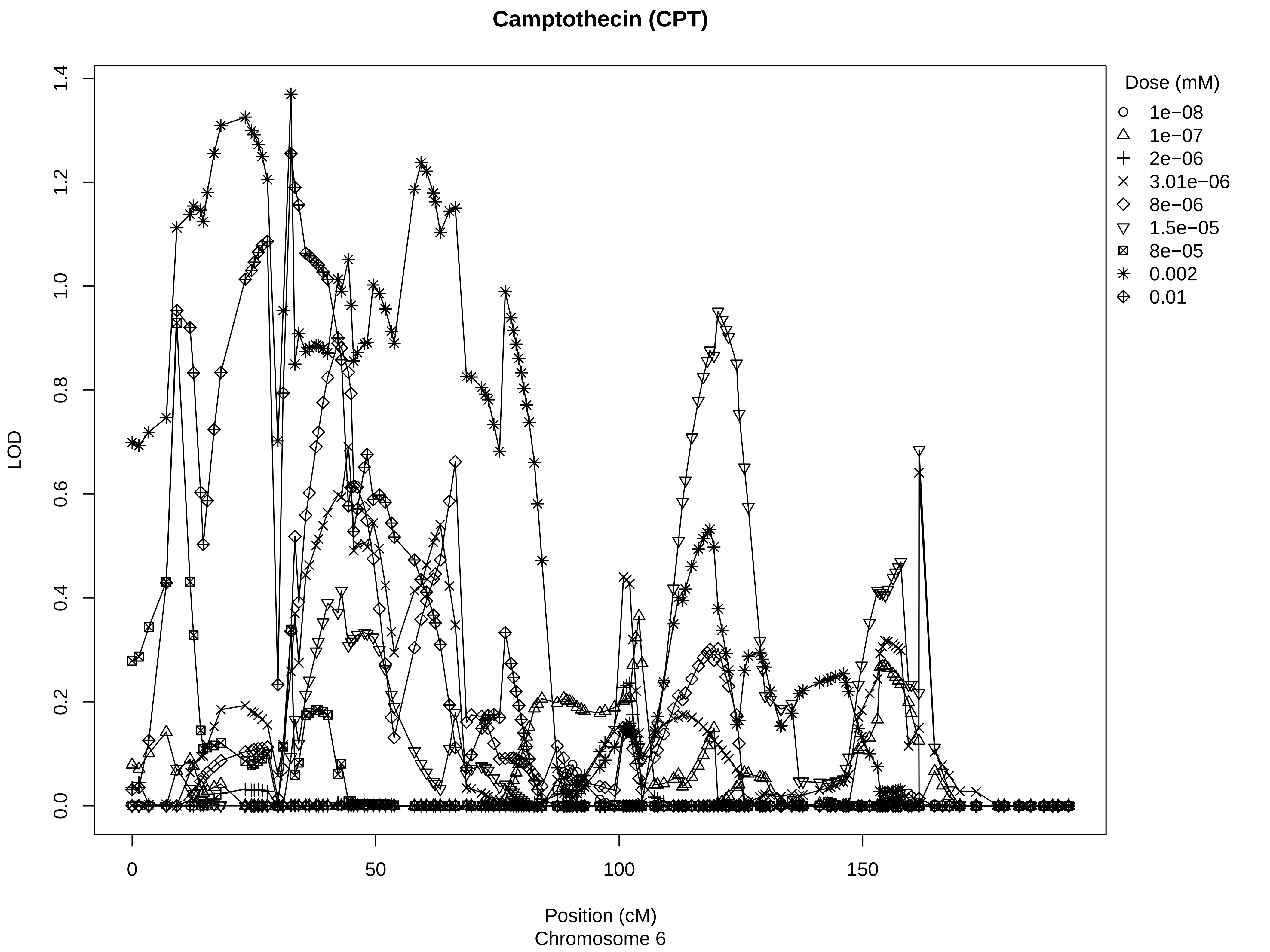

Camptothecin (CPT)
Dose (mM)
1e−08
1e−07
2e−06
3.01e−06
8e−06
1.5e−05
8e−05
0.002
0.01
LOD
0.0
0.2
0.4
0.6
0.8
1.0
1.2
1.4
0
50
100
150
Position (cM)
Chromosome 6

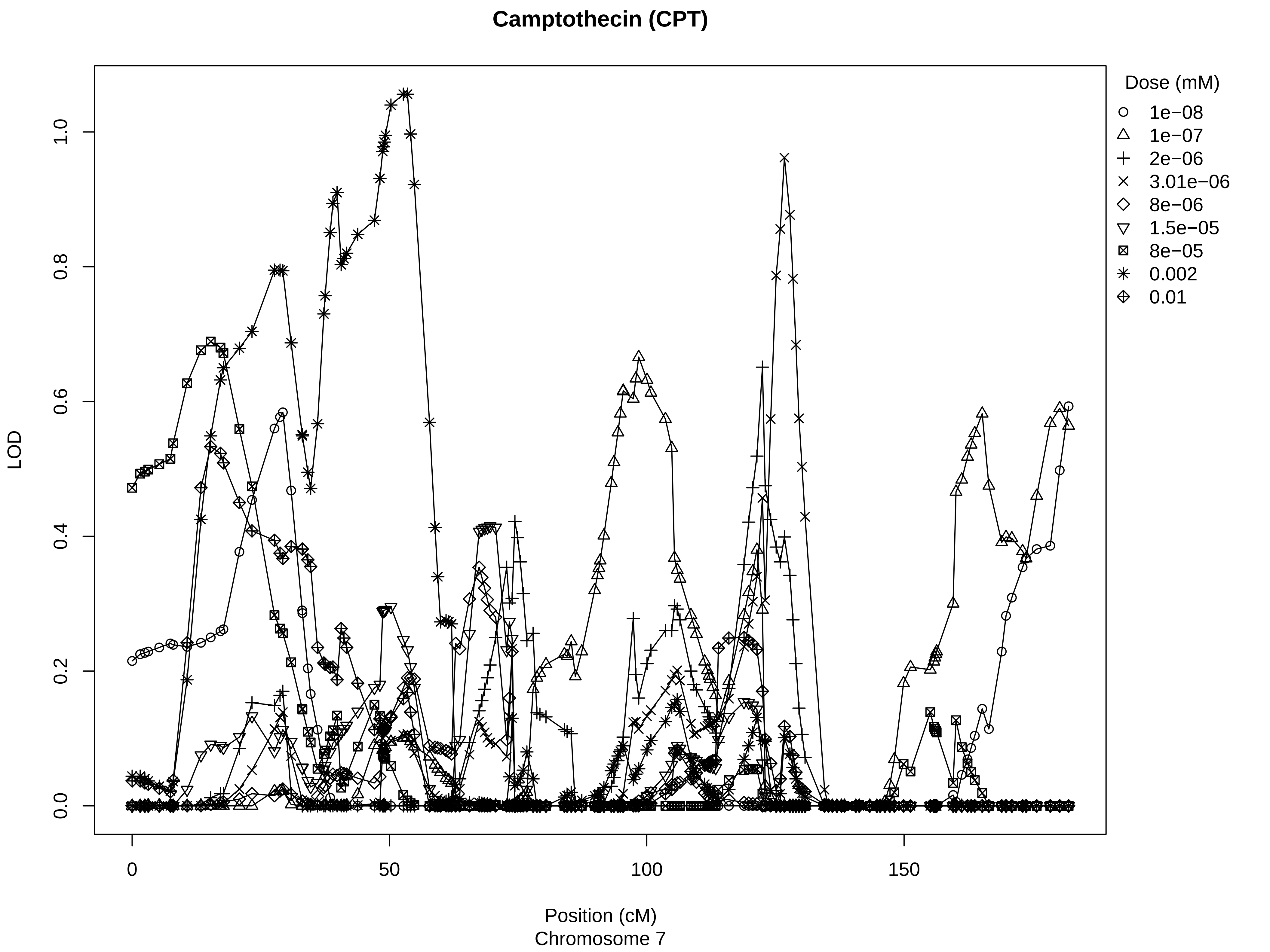

Camptothecin (CPT)
LOD
0.0
0.2
0.4
0.6
0.8
1.0
0
50
100
150
Position (cM)
Chromosome 7
Dose (mM)
1e−08
1e−07
2e−06
3.01e−06
8e−06
1.5e−05
8e−05
0.002
0.01

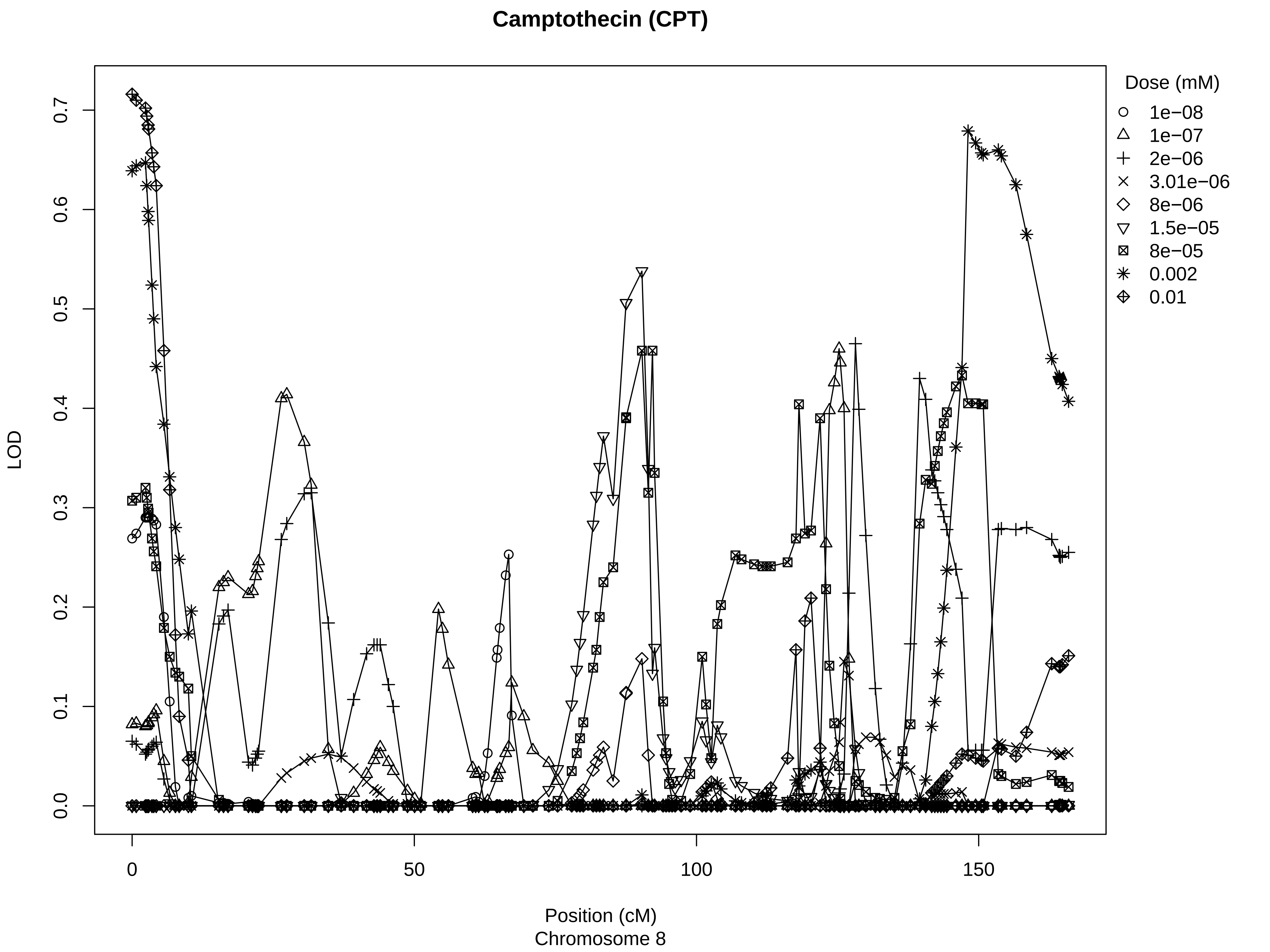

Camptothecin (CPT)
Dose (mM)
1e−08
1e−07
2e−06
3.01e−06
8e−06
1.5e−05
8e−05
0.002
0.01
LOD
0.0
0.1
0.2
0.3
0.4
0.5
0.6
0.7
0
50
100
150
Position (cM)
Chromosome 8

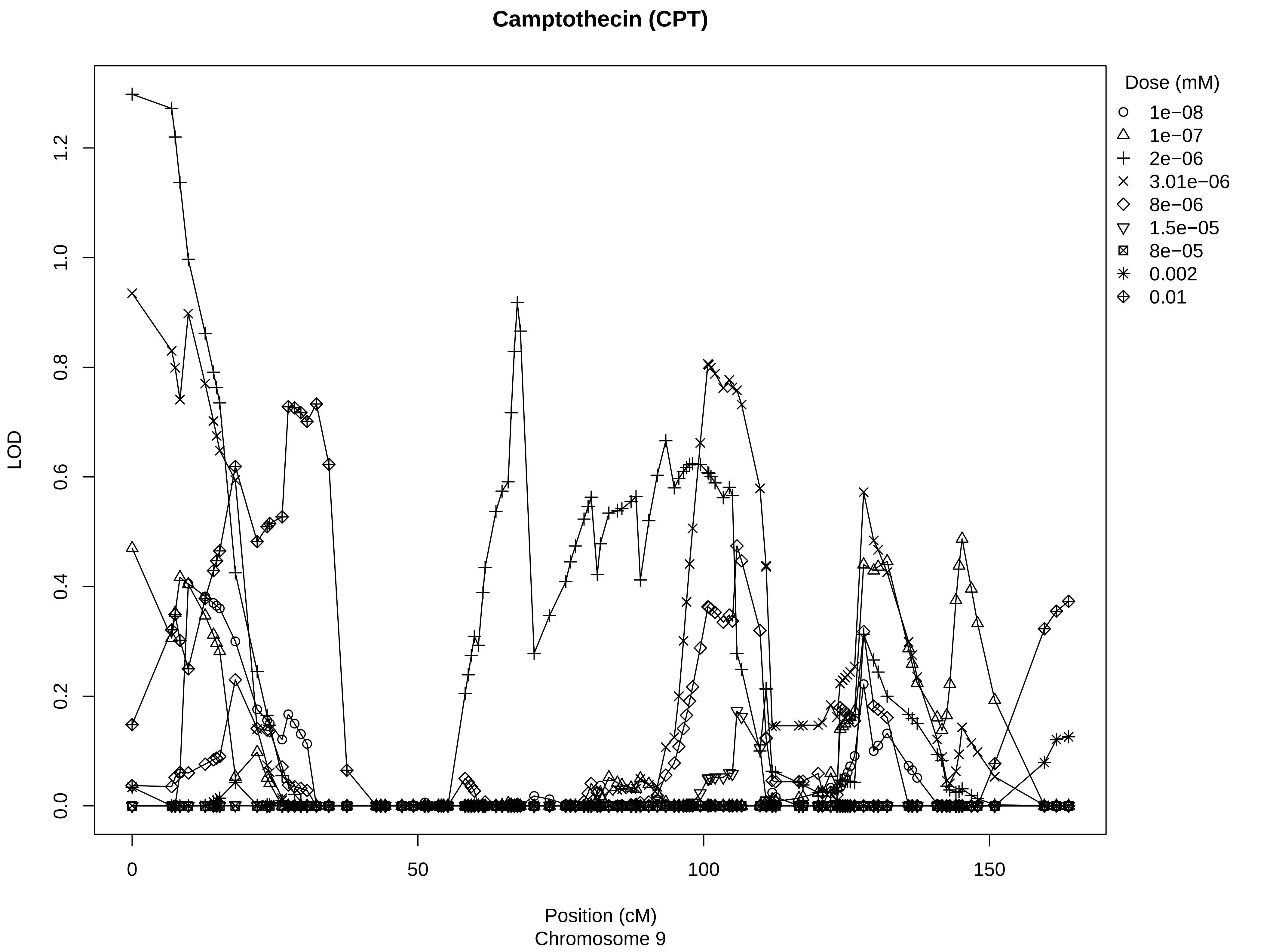
Camptothecin (CPT)
LOD
0.0
0.2
0.4
0.6
0.8
1.0
1.2
0
50
100
150
Position (cM)
Chromosome 9
Dose (mM)
1e−08
1e−07
2e−06
3.01e−06
8e−06
1.5e−05
8e−05
0.002
0.01

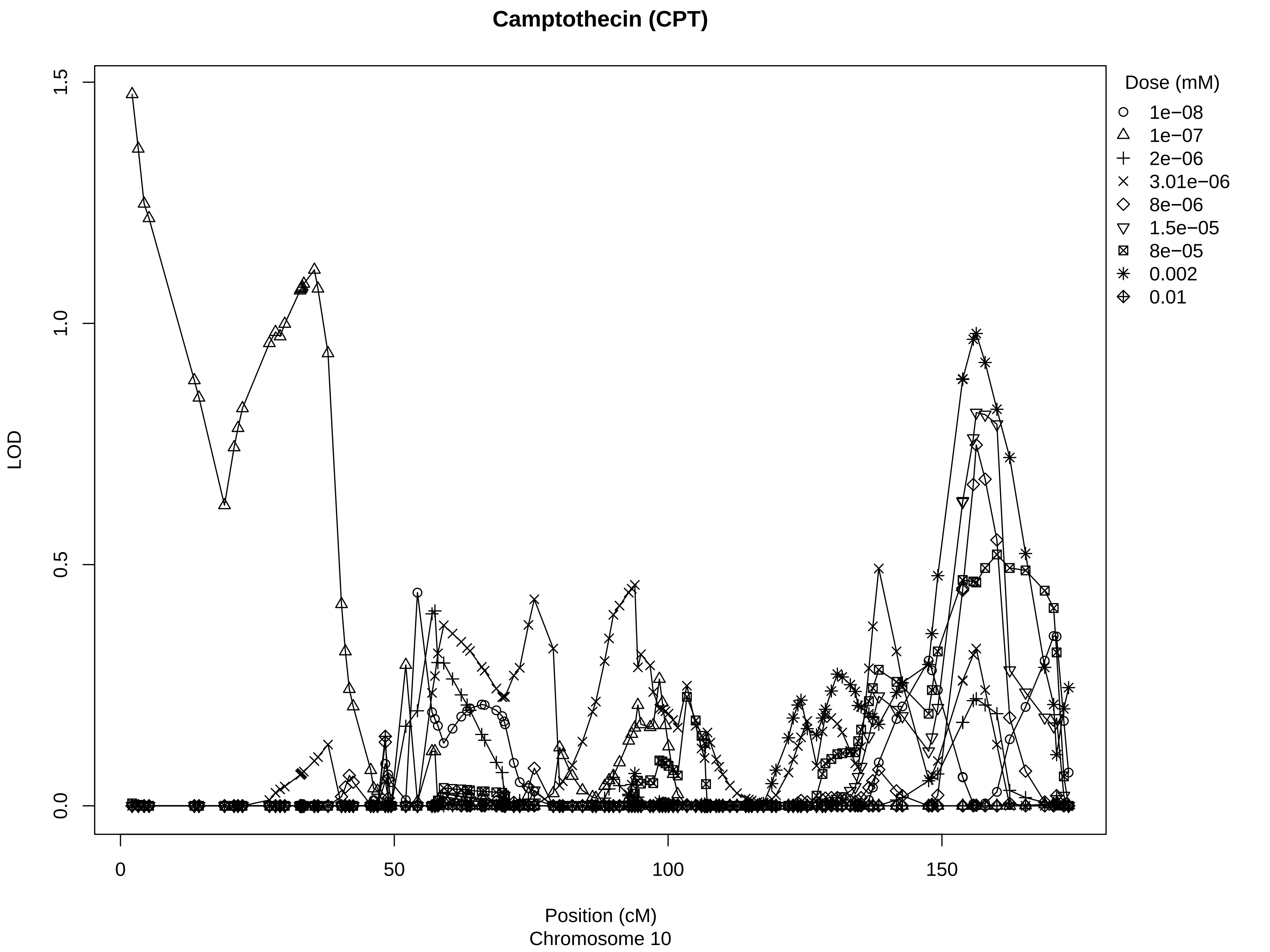
Camptothecin (CPT)
LOD
1.5
1.0
0.5
0.0
0
50
100
150
Position (cM)
Chromosome 10
Dose (mM)
1e−08
1e−07
2e−06
3.01e−06
8e−06
1.5e−05
8e−05
0.002
0.01

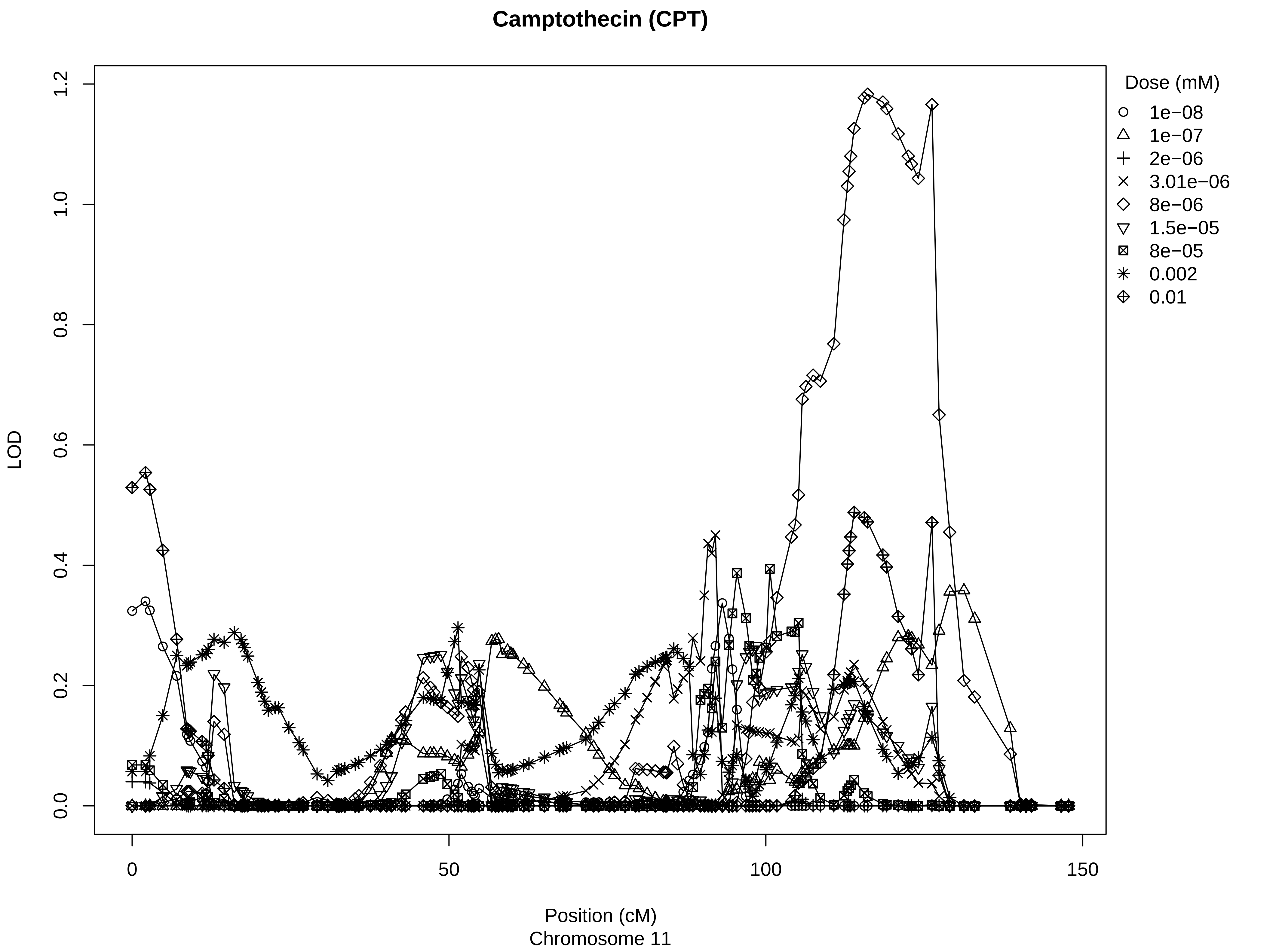

Camptothecin (CPT)
LOD
0.0
0.2
0.4
0.6
0.8
1.0
1.2
0
50
100
150
Position (cM)
Chromosome 11
Dose (mM)
1e−08
1e−07
2e−06
3.01e−06
8e−06
1.5e−05
8e−05
0.002
0.01

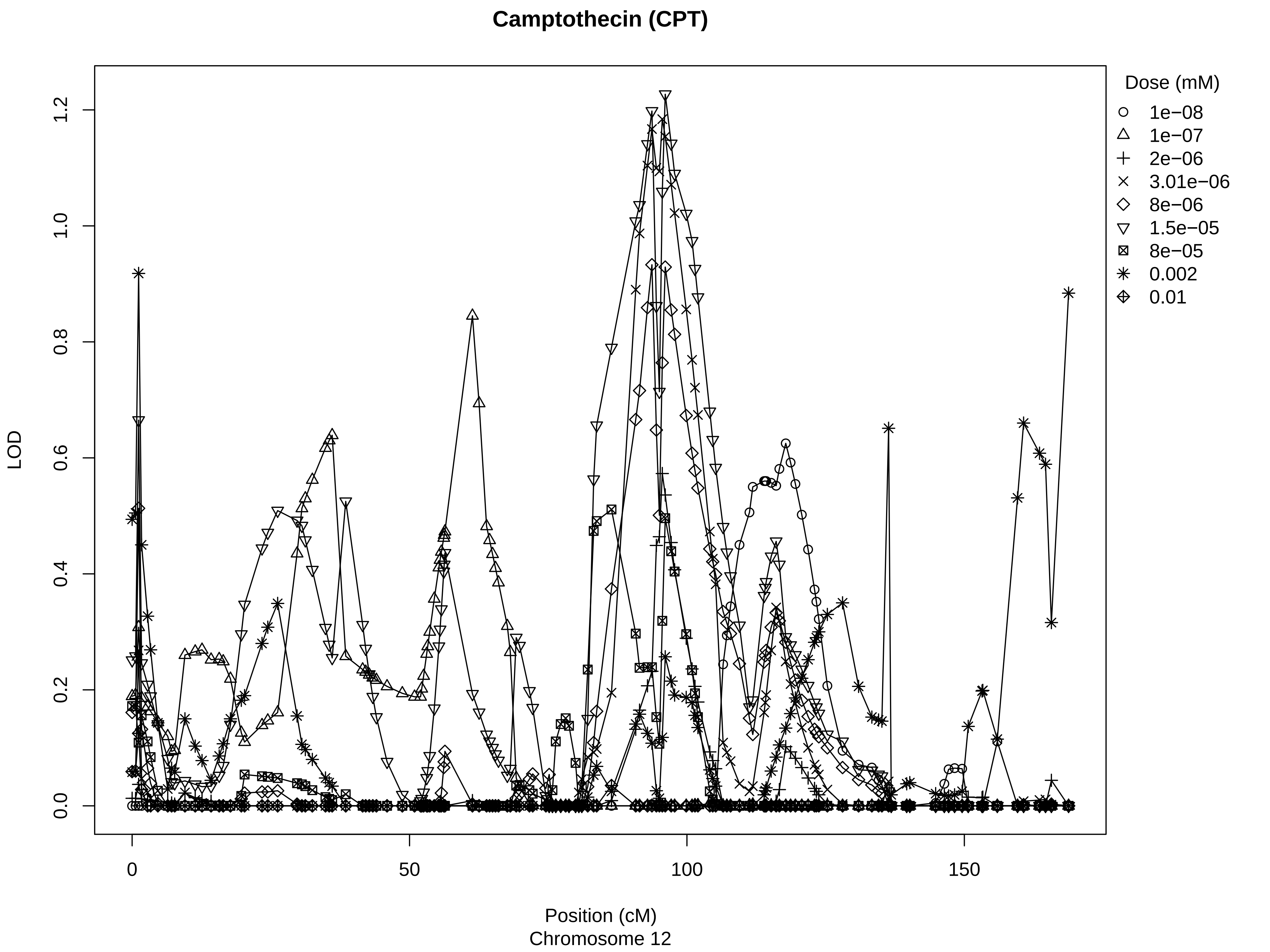

Camptothecin (CPT)
LOD
0.0
0.2
0.4
0.6
0.8
1.0
1.2
0
50
100
150
Position (cM)
Chromosome 12
Dose (mM)
1e−08
1e−07
2e−06
3.01e−06
8e−06
1.5e−05
8e−05
0.002
0.01

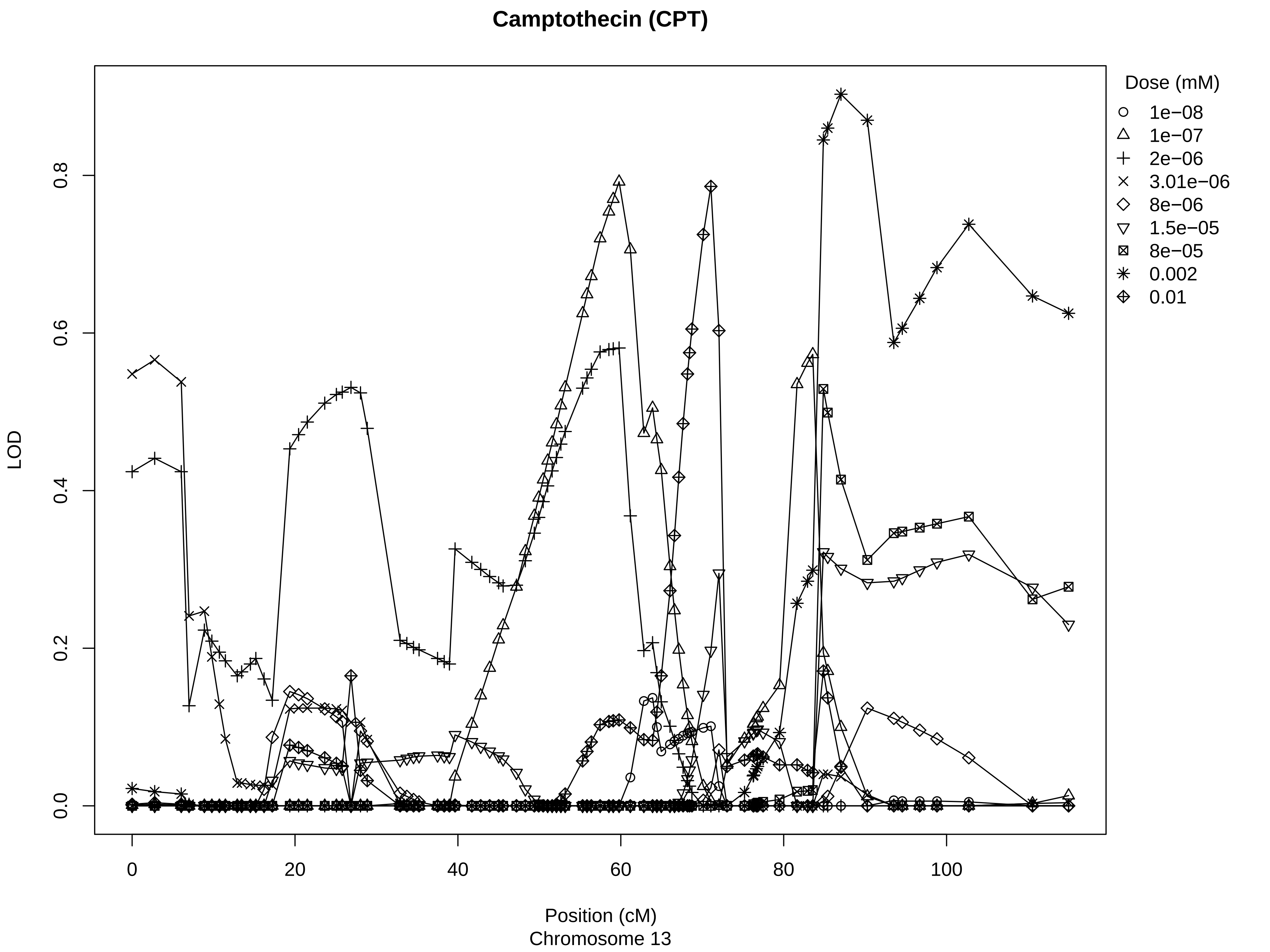

Camptothecin (CPT)
Dose (mM)
1e−08
1e−07
2e−06
3.01e−06
8e−06
1.5e−05
8e−05
0.002
0.01
LOD
0.0
0.2
0.4
0.6
0.8
0
20
40
60
80
100
Position (cM)
Chromosome 13

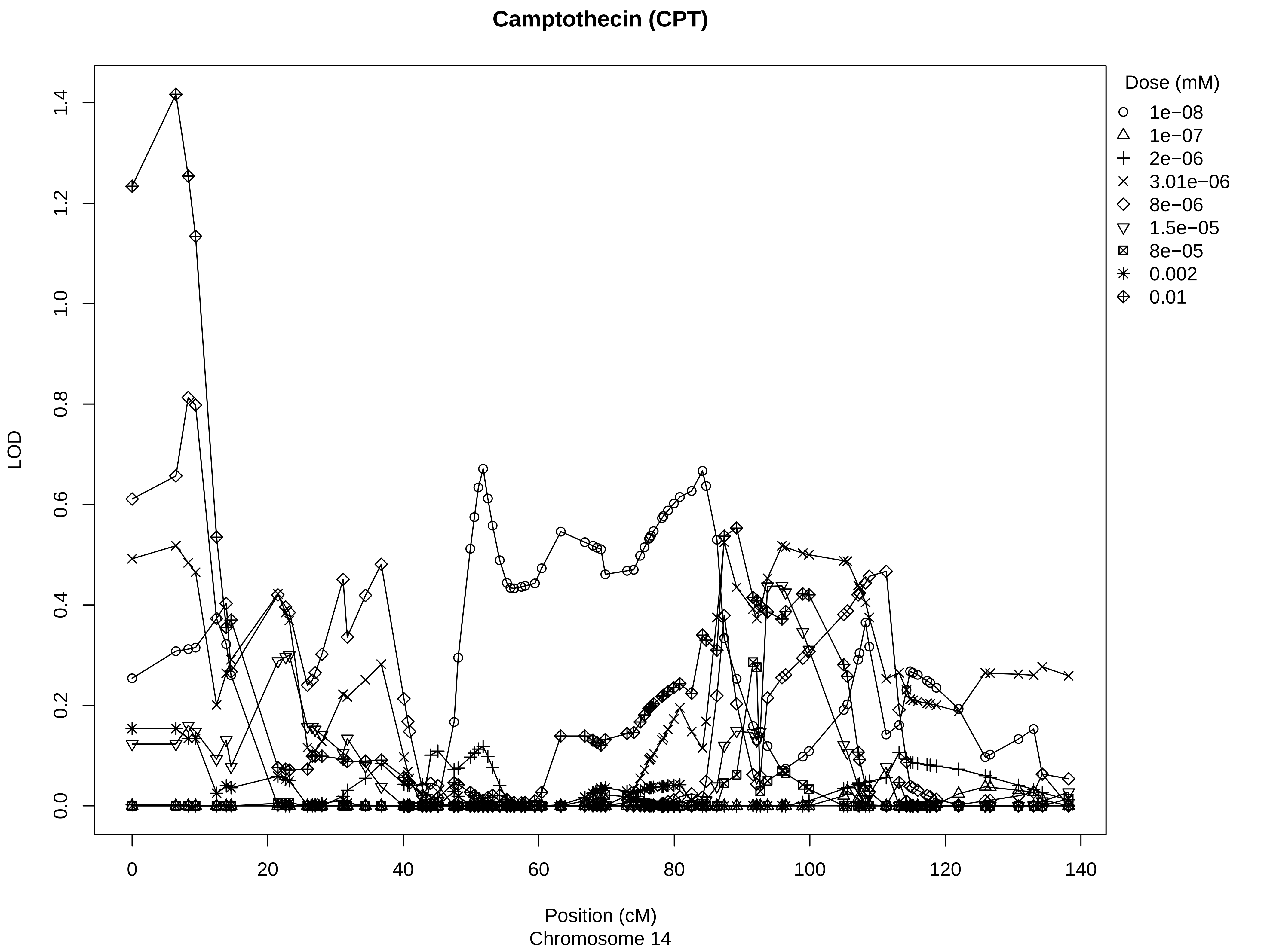
Camptothecin (CPT)
LOD
0.0
0.2
0.4
0.6
0.8
1.0
1.2
1.4
0
20
40
60
80
100
120
140
Position (cM)
Chromosome 14
Dose (mM)
1e−08
1e−07
2e−06
3.01e−06
8e−06
1.5e−05
8e−05
0.002
0.01

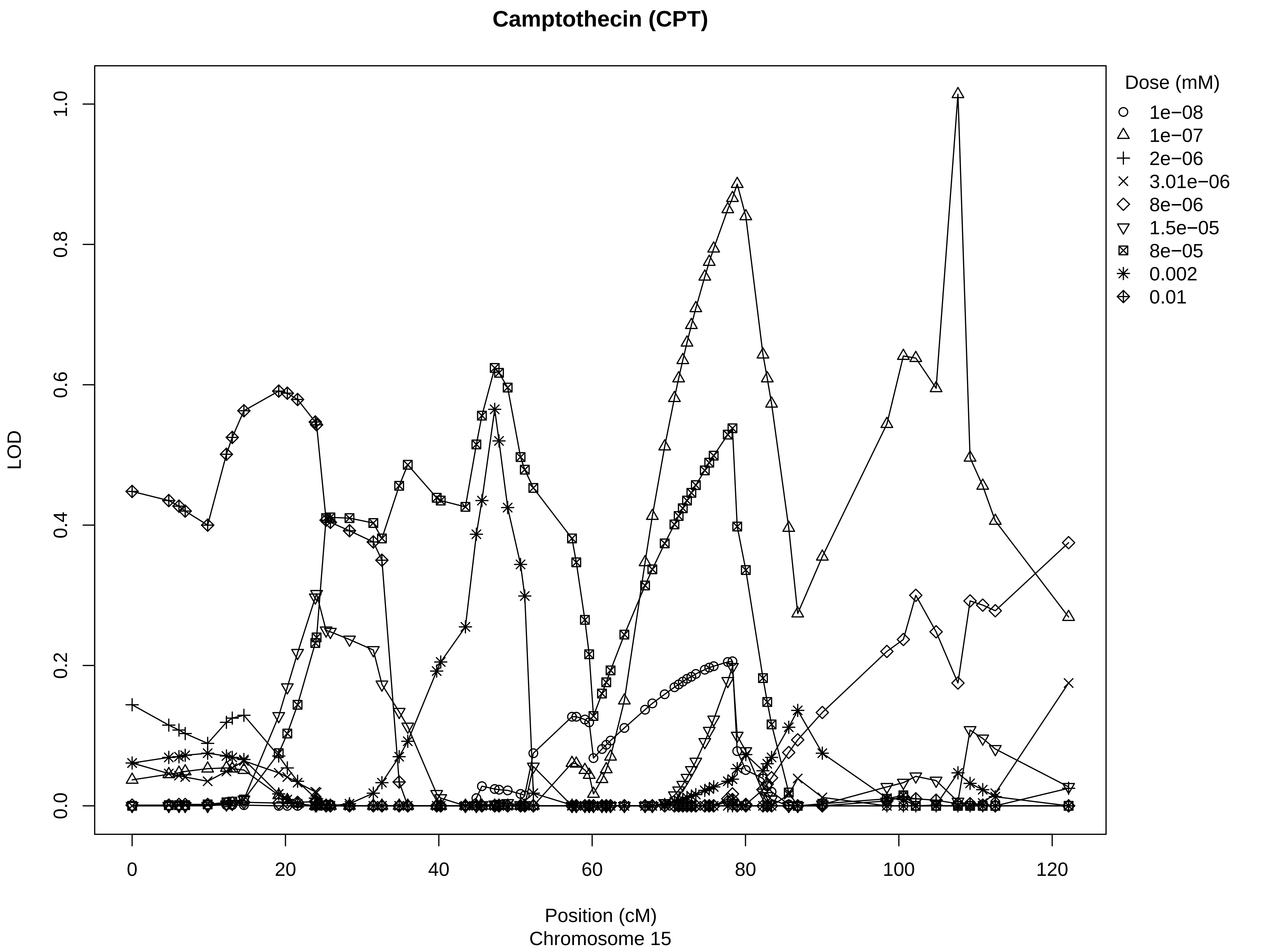
Camptothecin (CPT)
LOD
0.0
0.2
0.4
0.6
0.8
1.0
0
20
40
60
80
100
120
Position (cM)
Chromosome 15
Dose (mM)
1e−08
1e−07
2e−06
3.01e−06
8e−06
1.5e−05
8e−05
0.002
0.01

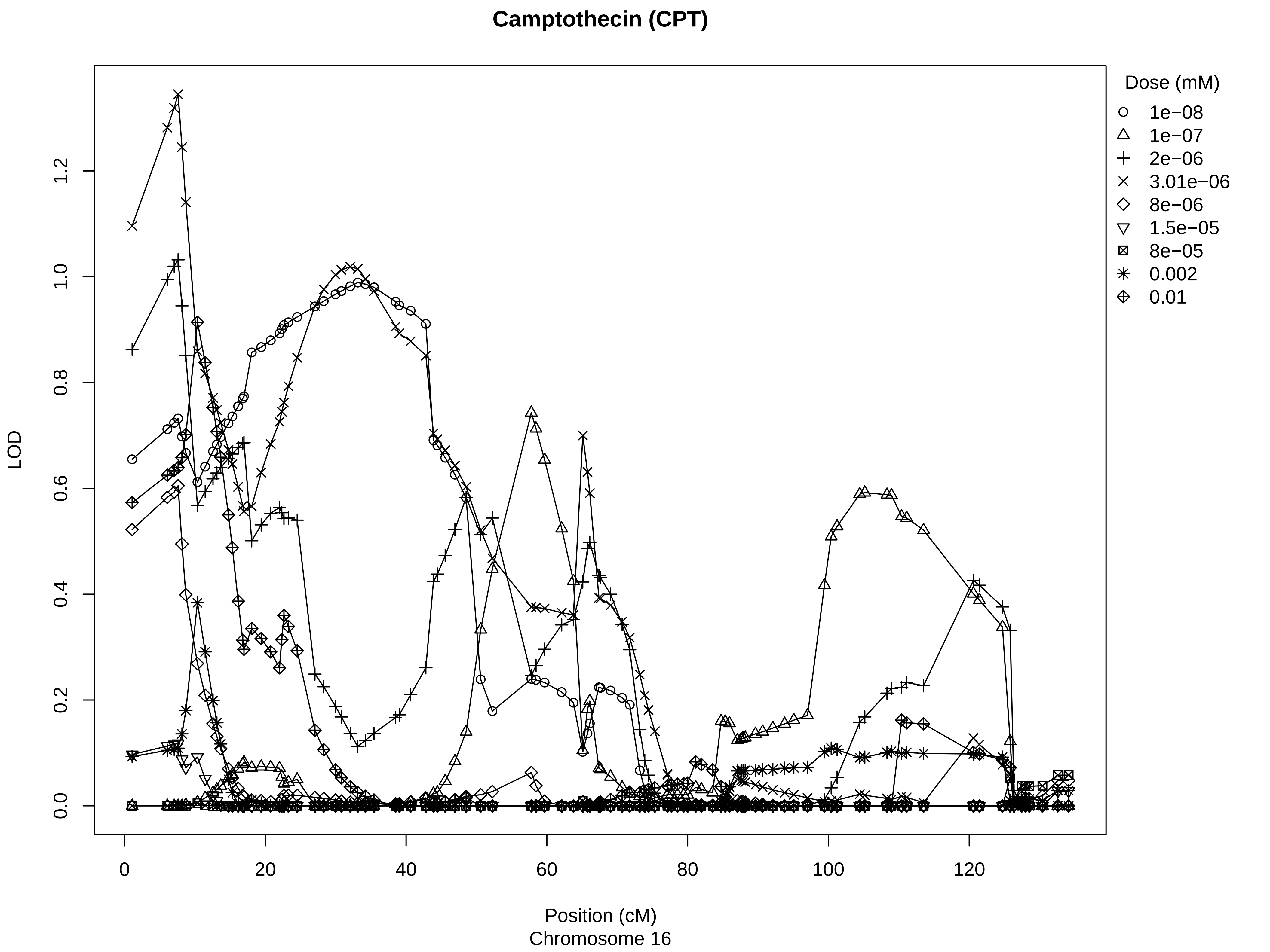
Camptothecin (CPT)
Dose (mM)
1e−08
1e−07
2e−06
3.01e−06
8e−06
1.5e−05
8e−05
0.002
0.01
LOD
0.0
0.2
0.4
0.6
0.8
1.0
1.2
0
20
40
60
80
100
120
Position (cM)
Chromosome 16

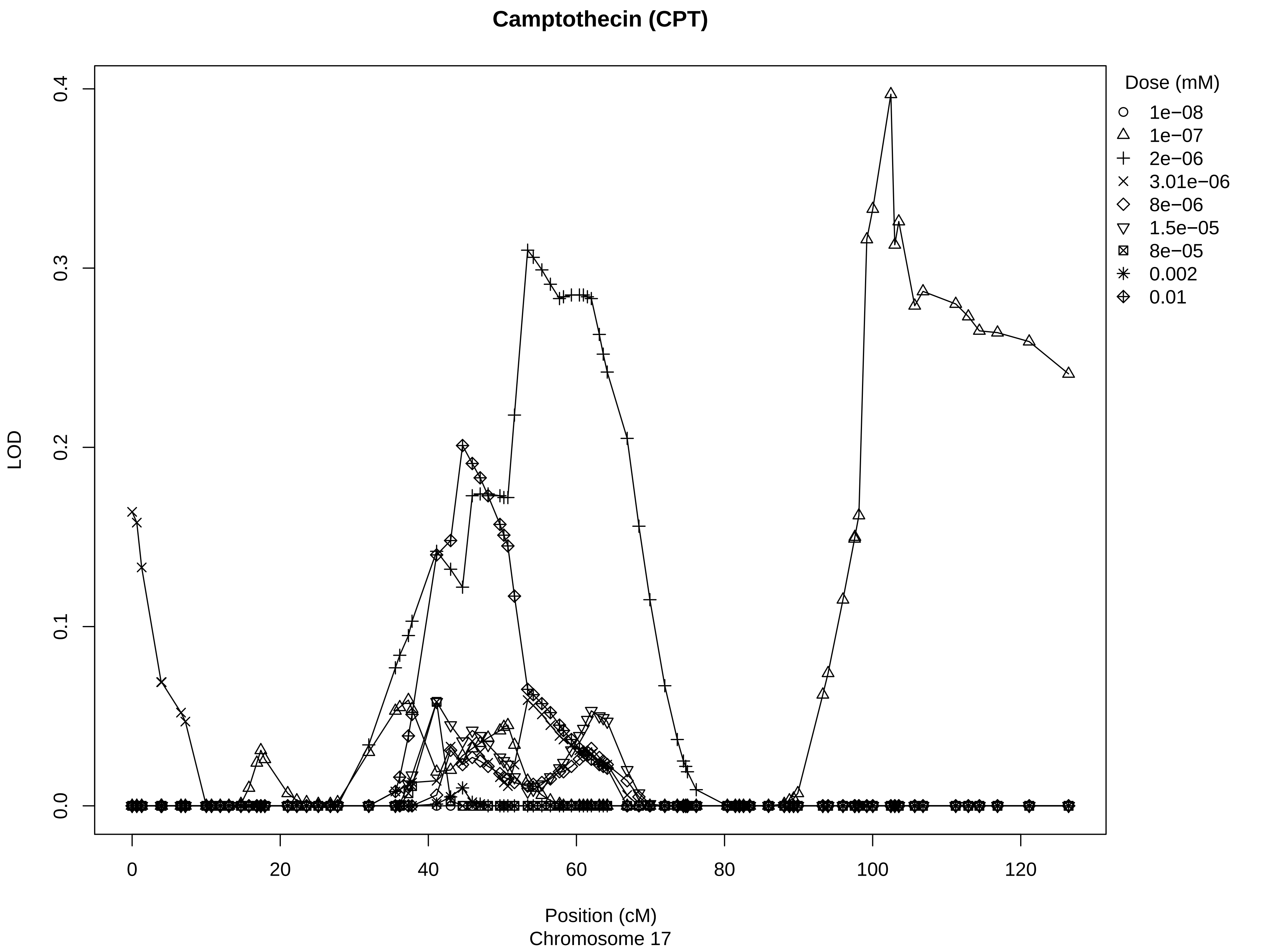
Camptothecin (CPT)
Dose (mM)
1e−08
1e−07
2e−06
3.01e−06
8e−06
1.5e−05
8e−05
0.002
0.01
LOD
0.0
0.1
0.2
0.3
0.4
0
20
40
60
80
100
120
Position (cM)
Chromosome 17

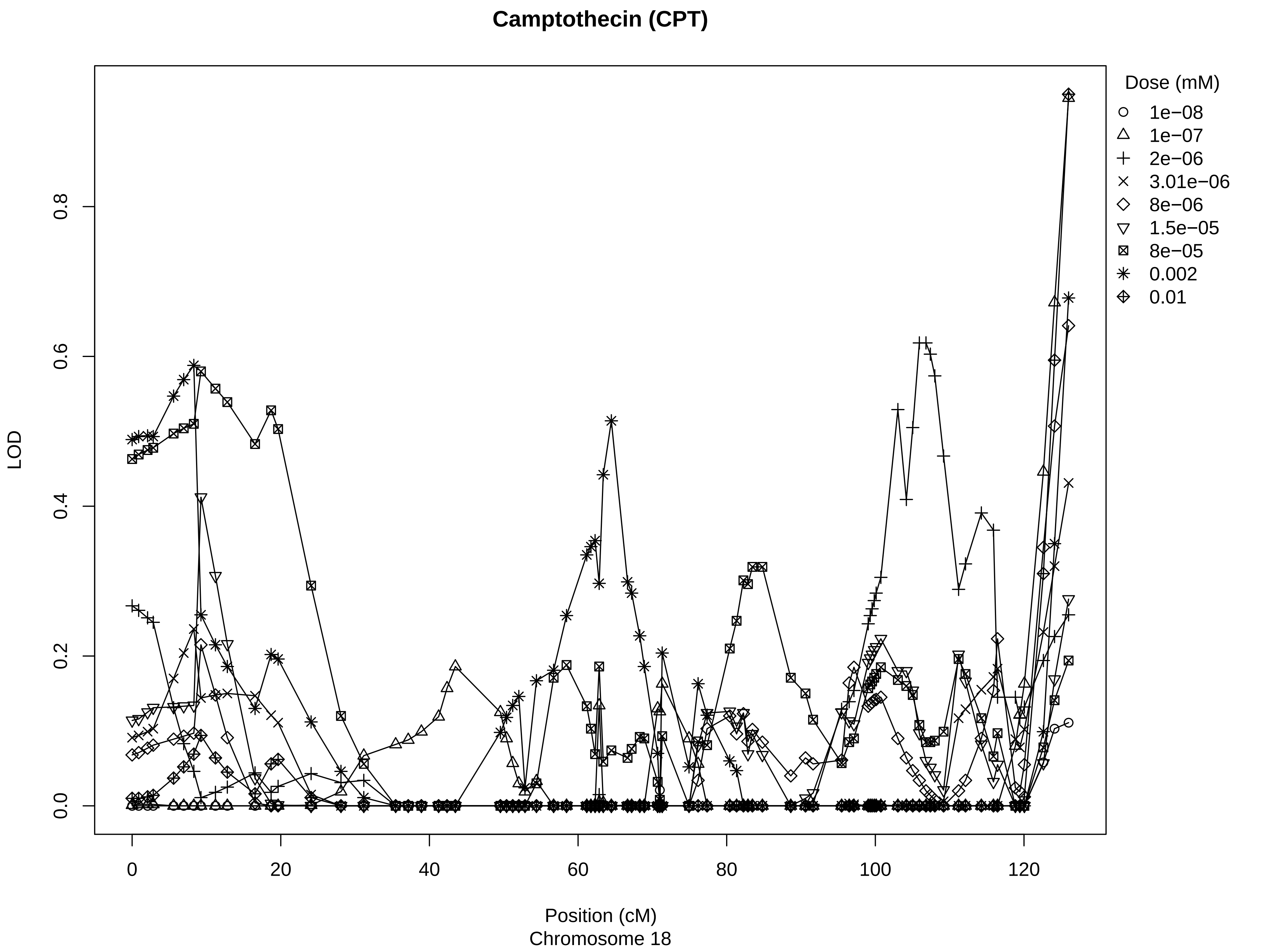
Camptothecin (CPT)
Dose (mM)
1e−08
1e−07
2e−06
3.01e−06
8e−06
1.5e−05
8e−05
0.002
0.01
LOD
0.0
0.2
0.4
0.6
0.8
0
20
40
60
80
100
120
Position (cM)
Chromosome 18

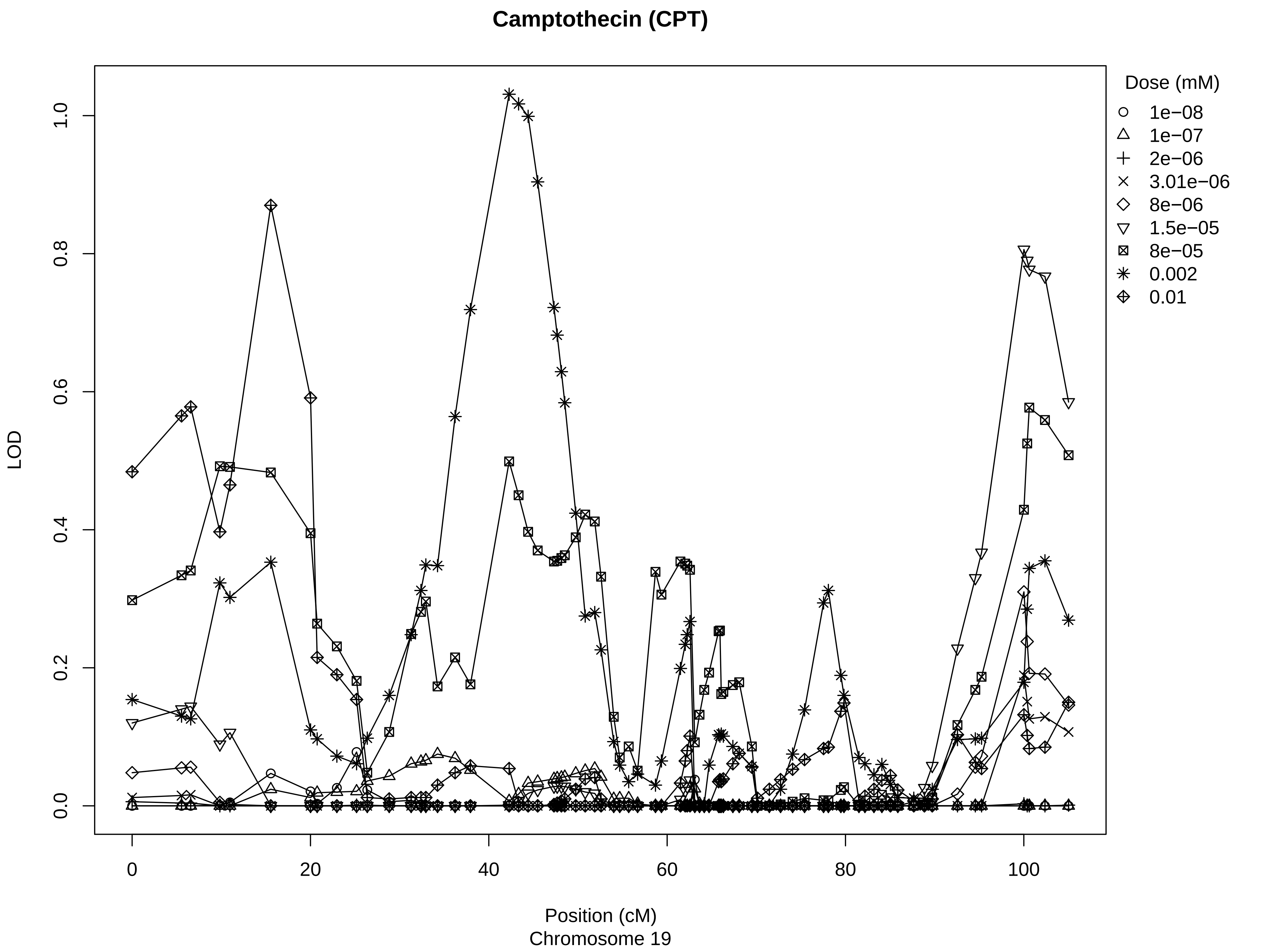

Camptothecin (CPT)
LOD
Position (cM)
Chromosome 19
Dose (mM)
1e−08
1e−07
2e−06
3.01e−06
8e−06
1.5e−05
8e−05
0.002
0.01
0.0
0.2
0.4
0.6
0.8
1.0
0
20
40
60
80
100

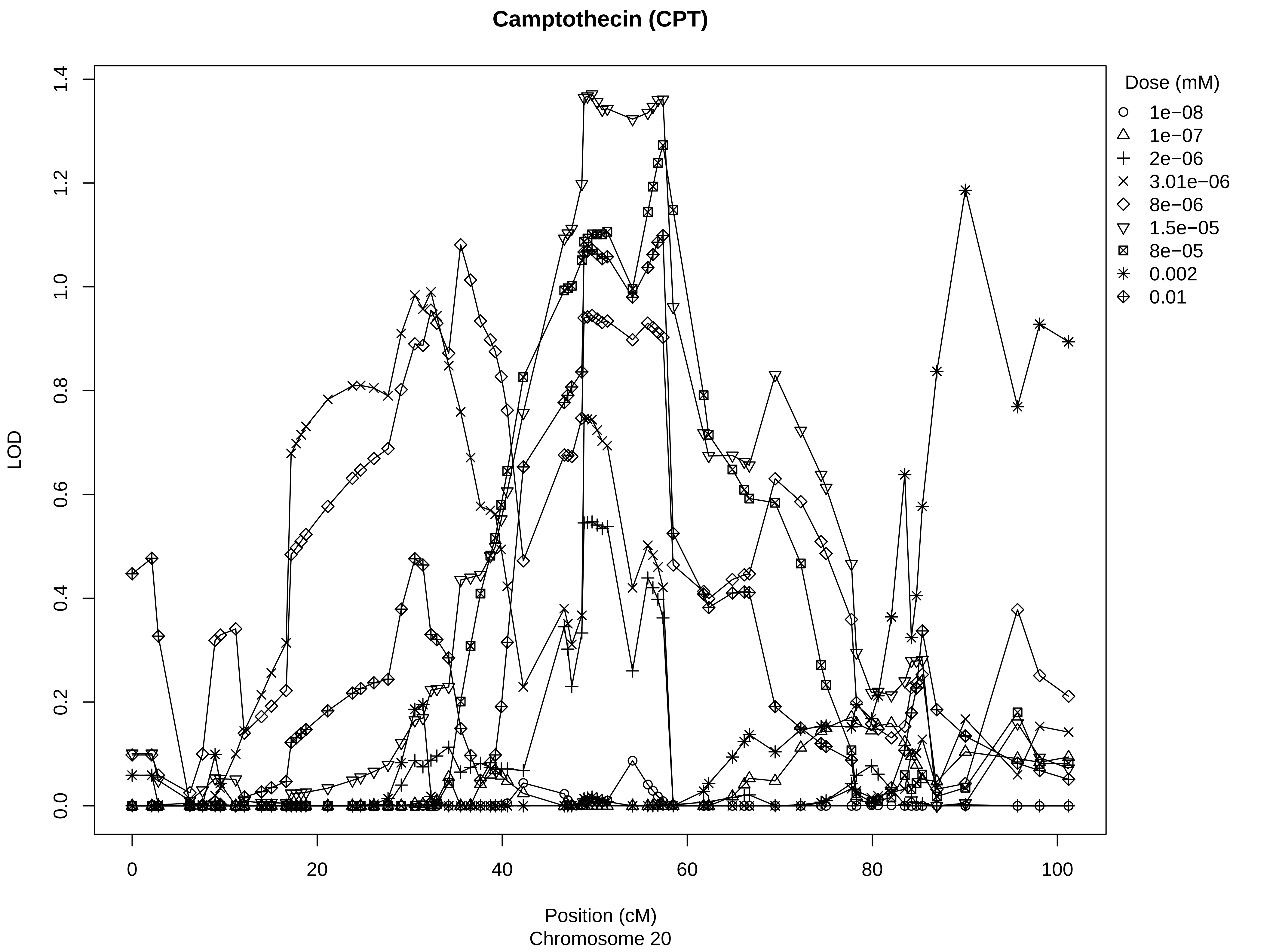
Camptothecin (CPT)
LOD
0.0
0.2
0.4
0.6
0.8
1.0
1.2
1.4
0
20
40
60
80
100
Position (cM)
Chromosome 20
Dose (mM)
1e−08
1e−07
2e−06
3.01e−06
8e−06
1.5e−05
8e−05
0.002
0.01

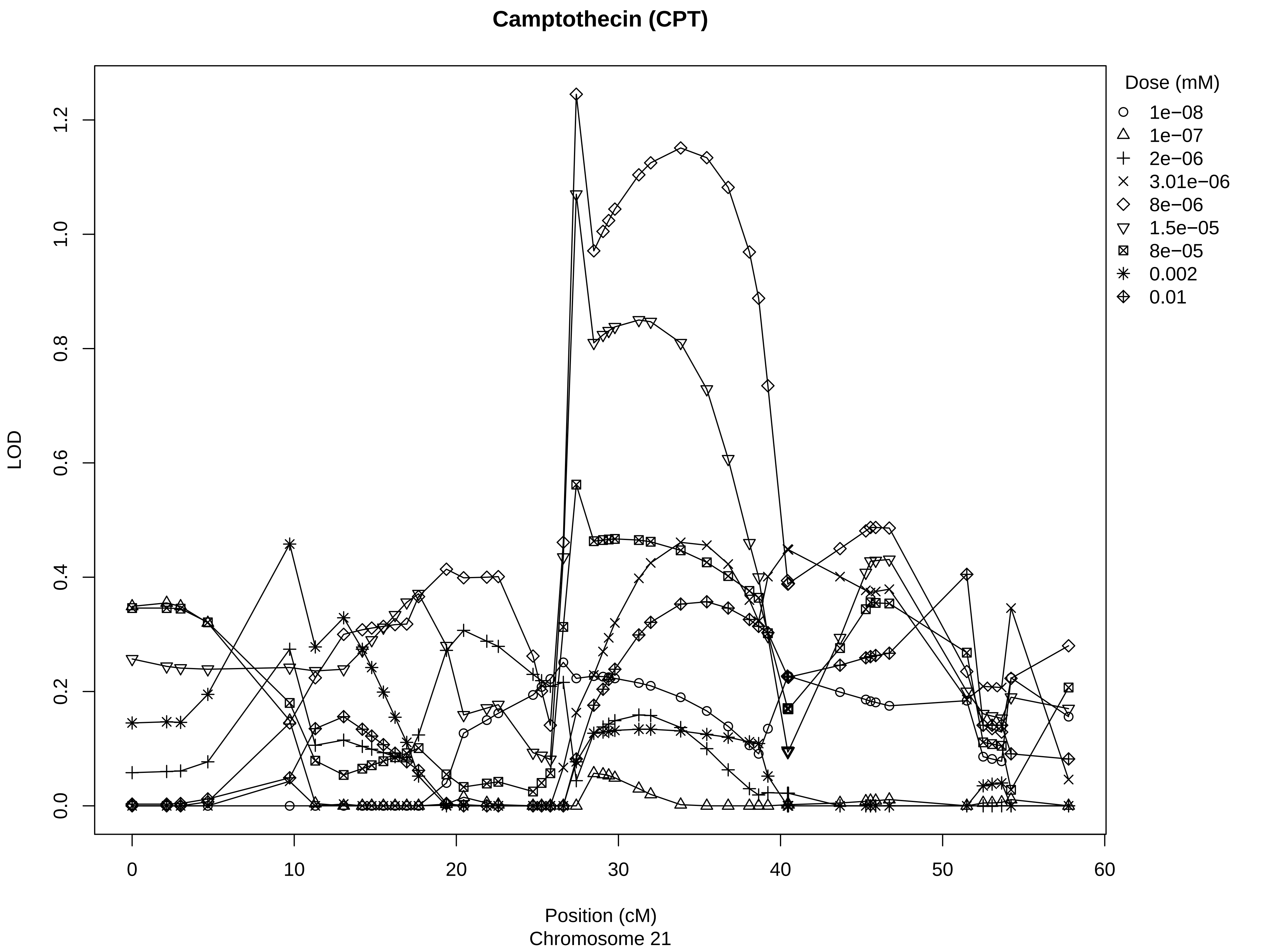

Camptothecin (CPT)
Dose (mM)
1e−08
1e−07
2e−06
3.01e−06
8e−06
1.5e−05
8e−05
0.002
0.01
LOD
0.0
0.2
0.4
0.6
0.8
1.0
1.2
0
10
20
30
40
50
60
Position (cM)
Chromosome 21

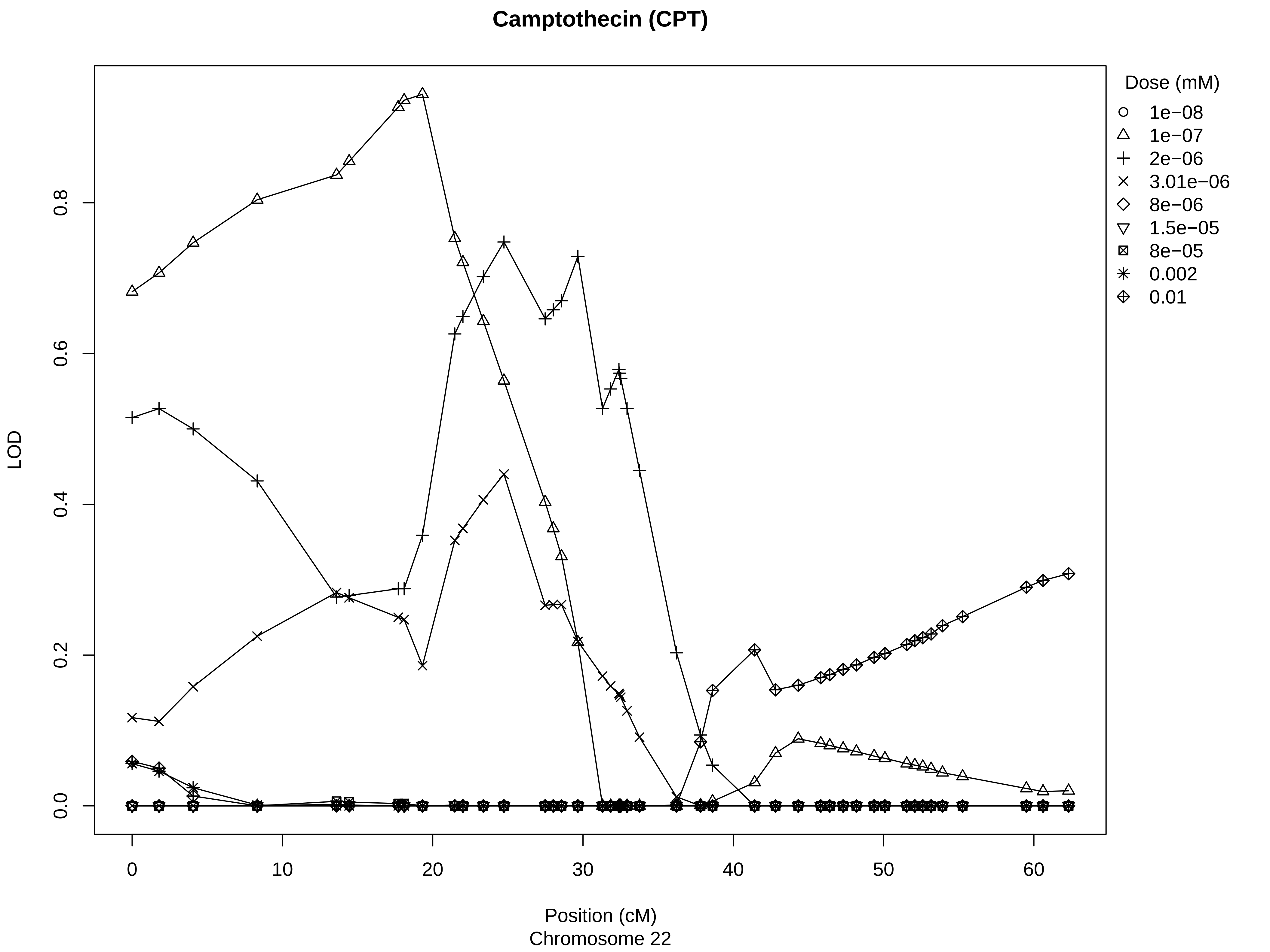
Camptothecin (CPT)
LOD
0.0
0.2
0.4
0.6
0.8
0
10
20
30
40
50
60
Position (cM)
Chromosome 22
Dose (mM)
1e−08
1e−07
2e−06
3.01e−06
8e−06
1.5e−05
8e−05
0.002
0.01

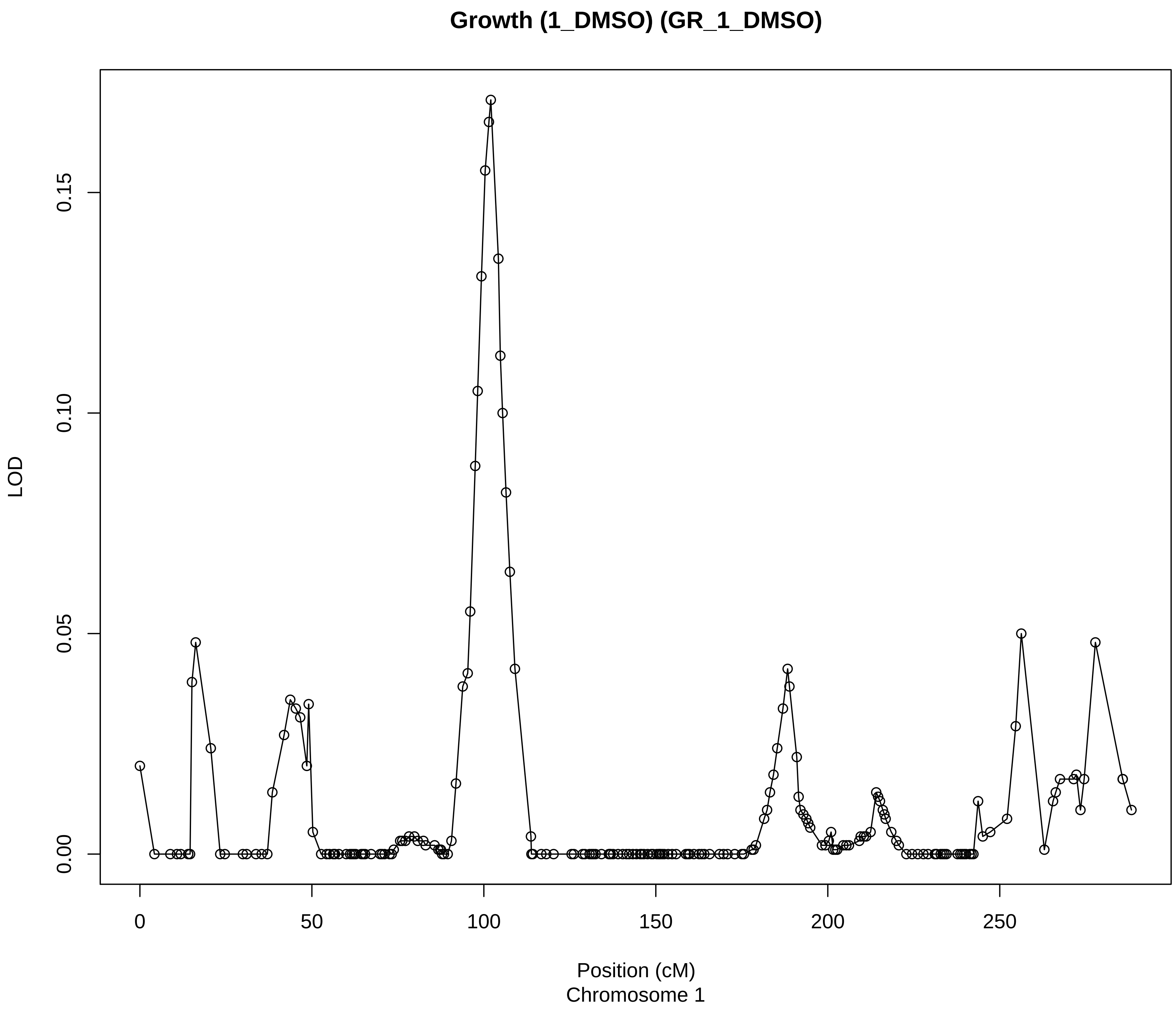

Growth (1_DMSO) (GR_1_DMSO)
LOD
0.00
0.05
0.10
0.15
0
50
100
150
200
250
Position (cM)
Chromosome 1

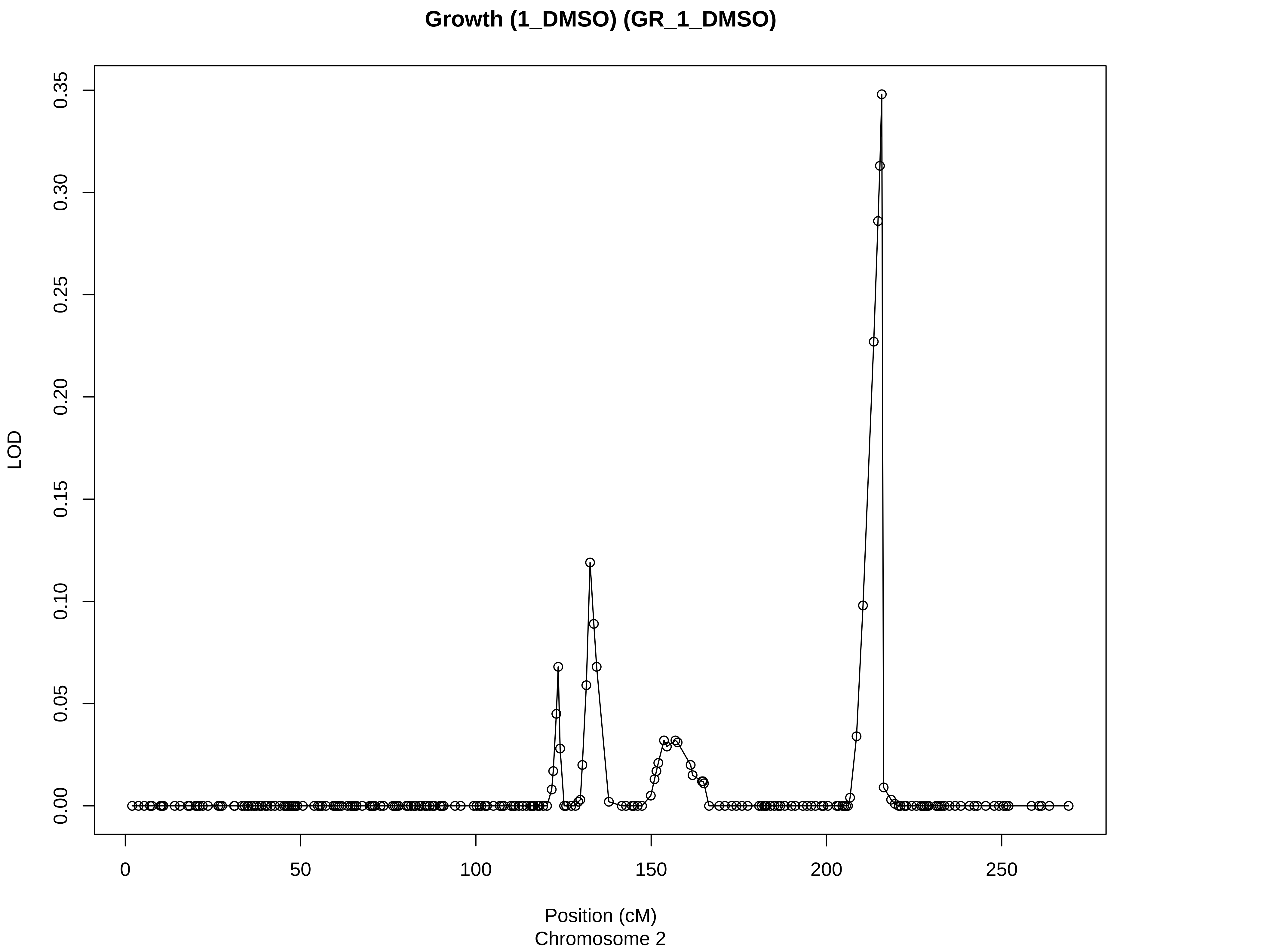
Growth (1_DMSO) (GR_1_DMSO)
LOD
0.00
0.05
0.10
0.15
0.20
0.25
0.30
0.35
0
50
100
150
200
250
Position (cM)
Chromosome 2

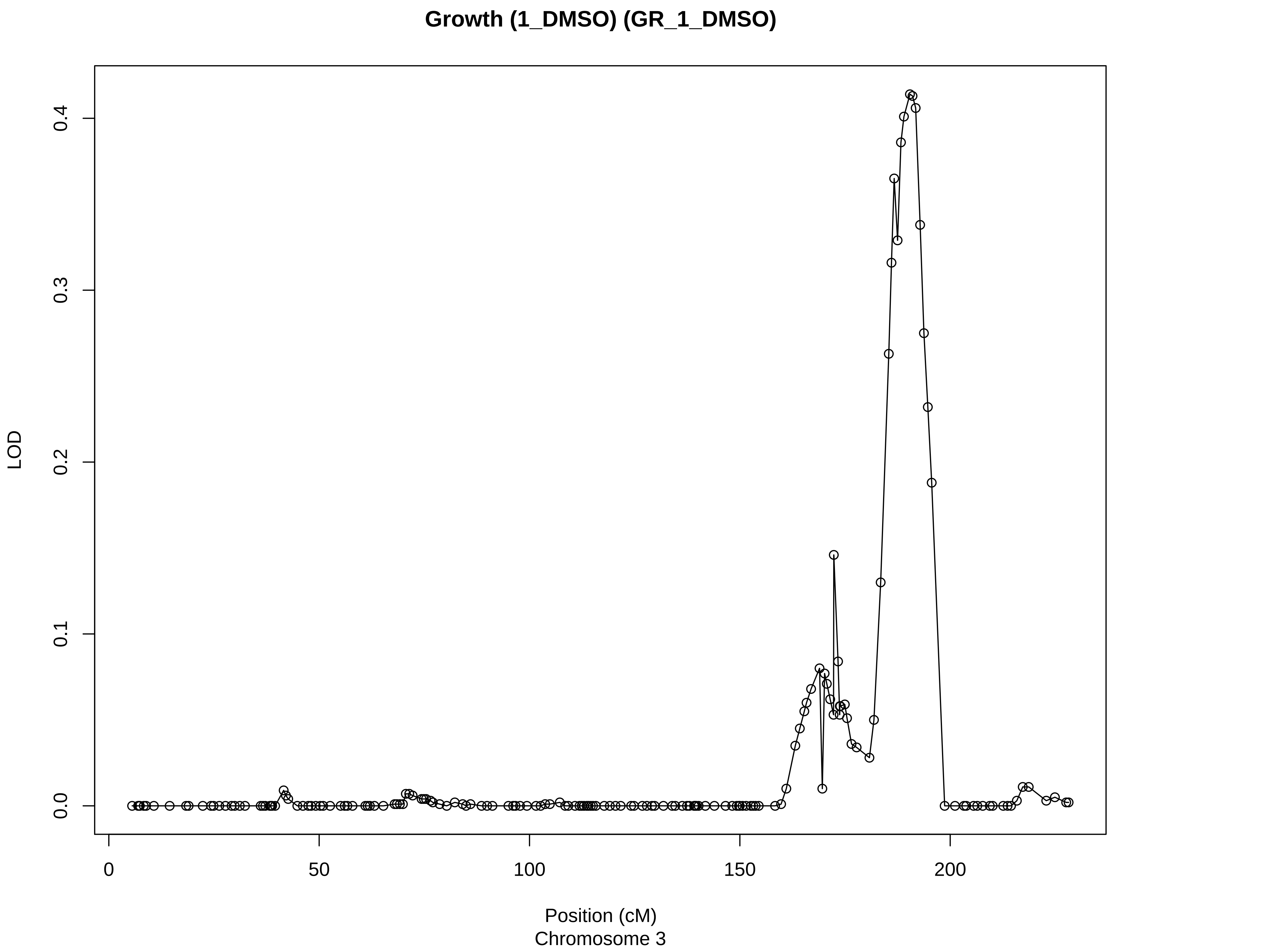

Growth (1_DMSO) (GR_1_DMSO)
LOD
0.0
0.1
0.2
0.3
0.4
0
50
100
150
200
Position (cM)
Chromosome 3

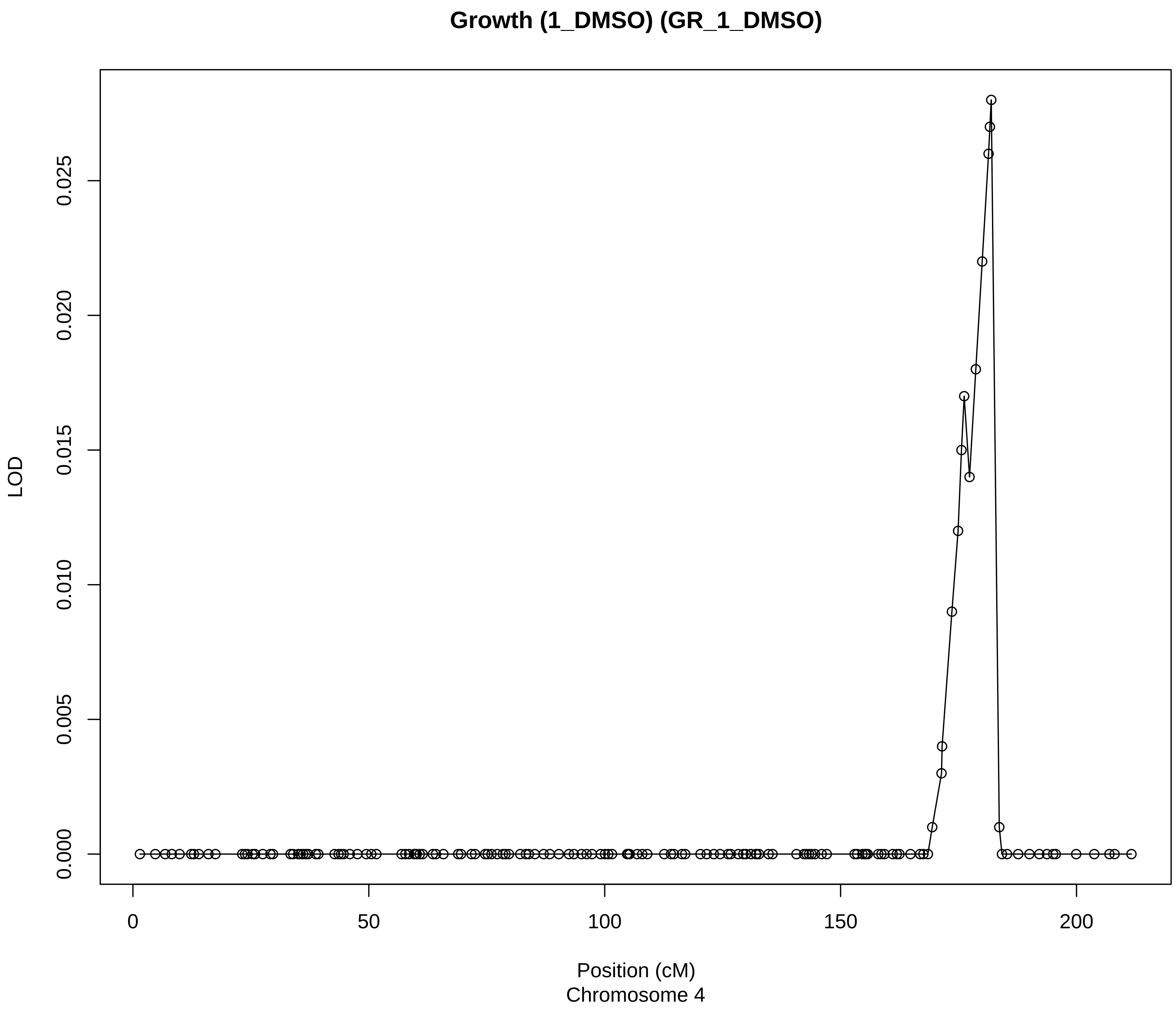

Growth (1_DMSO) (GR_1_DMSO)
LOD
0.000
0.005
0.010
0.015
0.020
0.025
0
50
100
150
200
Position (cM)
Chromosome 4

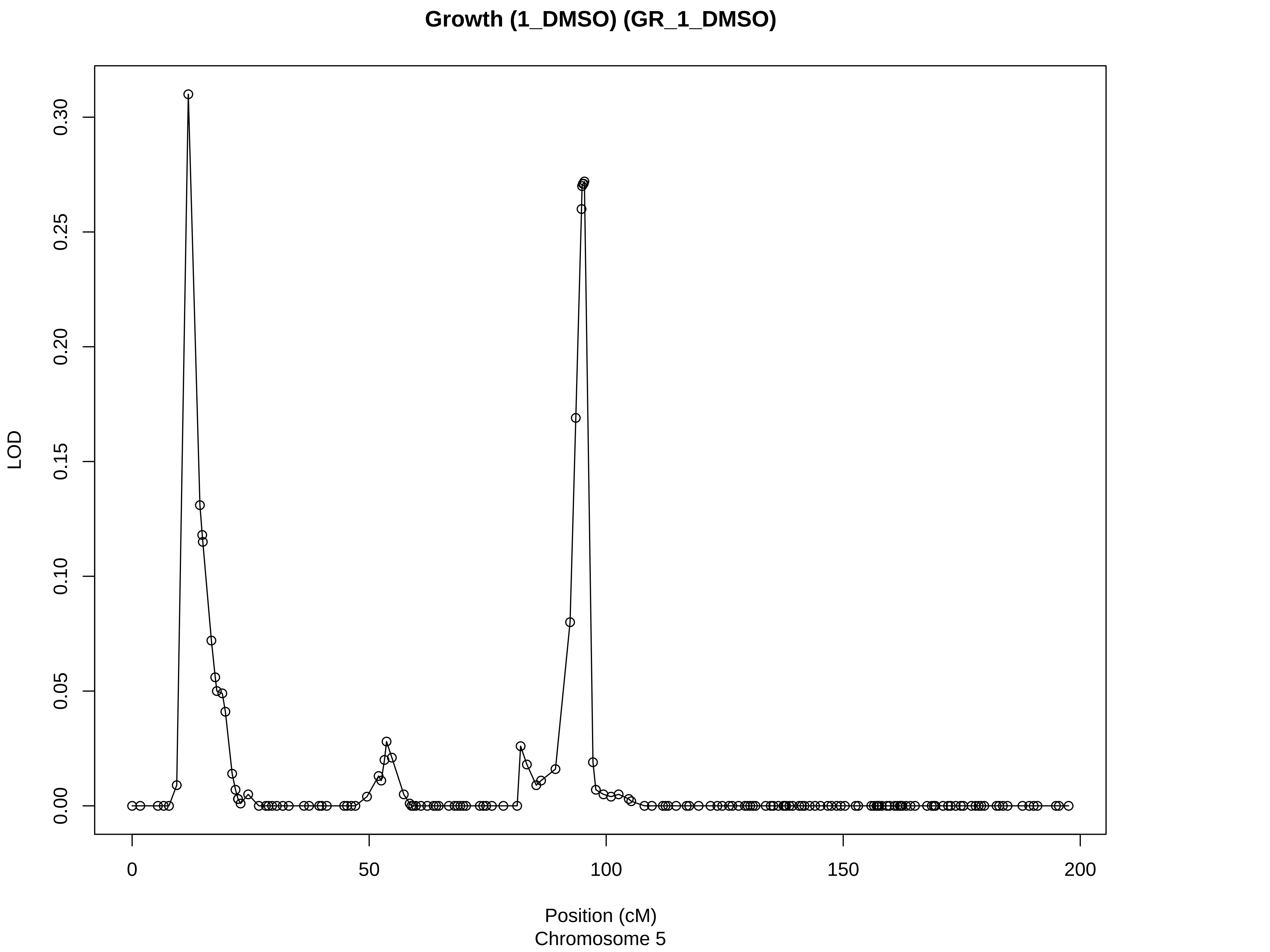
Growth (1_DMSO) (GR_1_DMSO)
LOD
0.00
0.05
0.10
0.15
0.20
0.25
0.30
0
50
100
150
200
Position (cM)
Chromosome 5

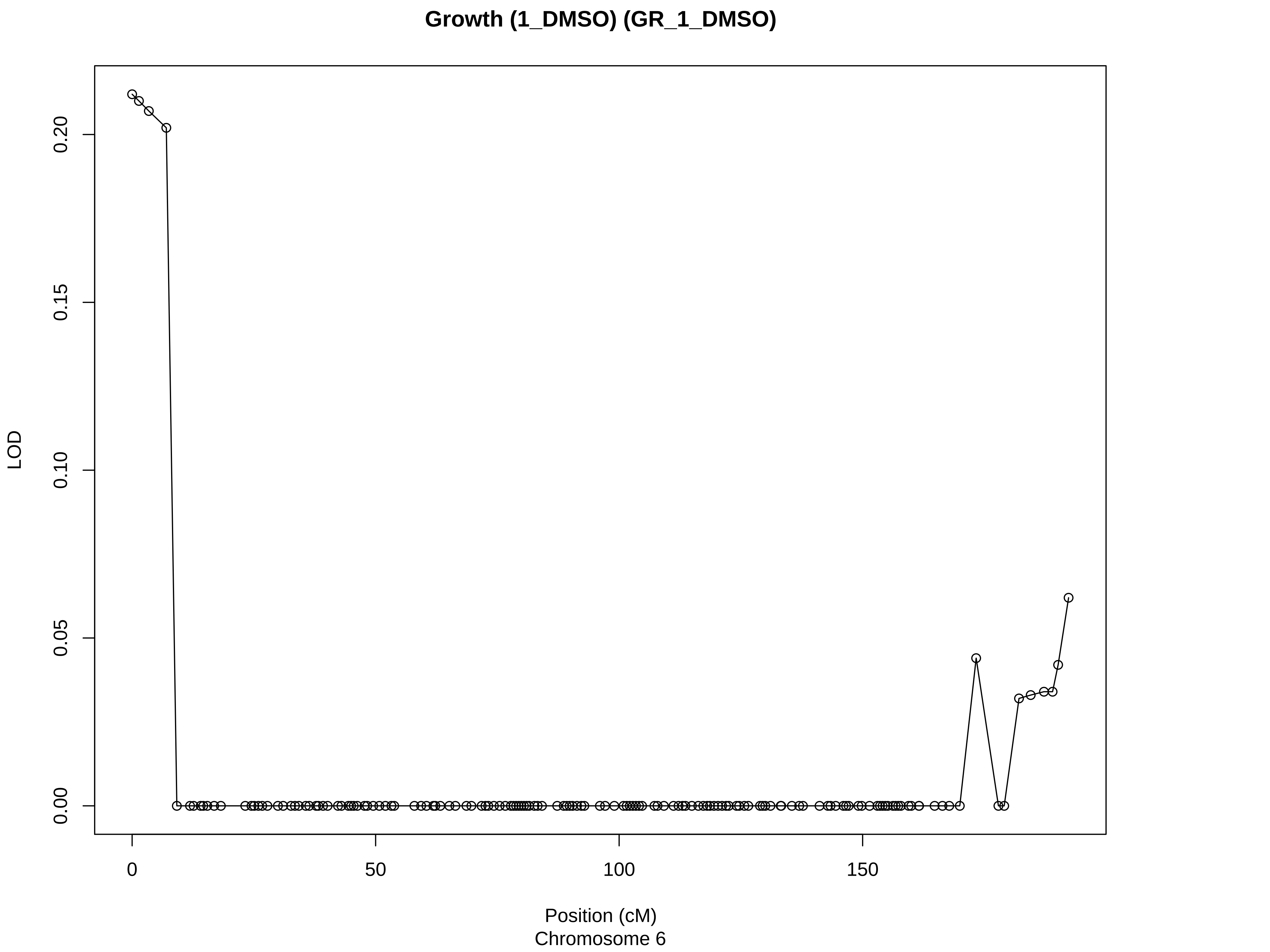
Growth (1_DMSO) (GR_1_DMSO)
LOD
0.00
0.05
0.10
0.15
0.20
0
50
100
150
Position (cM)
Chromosome 6

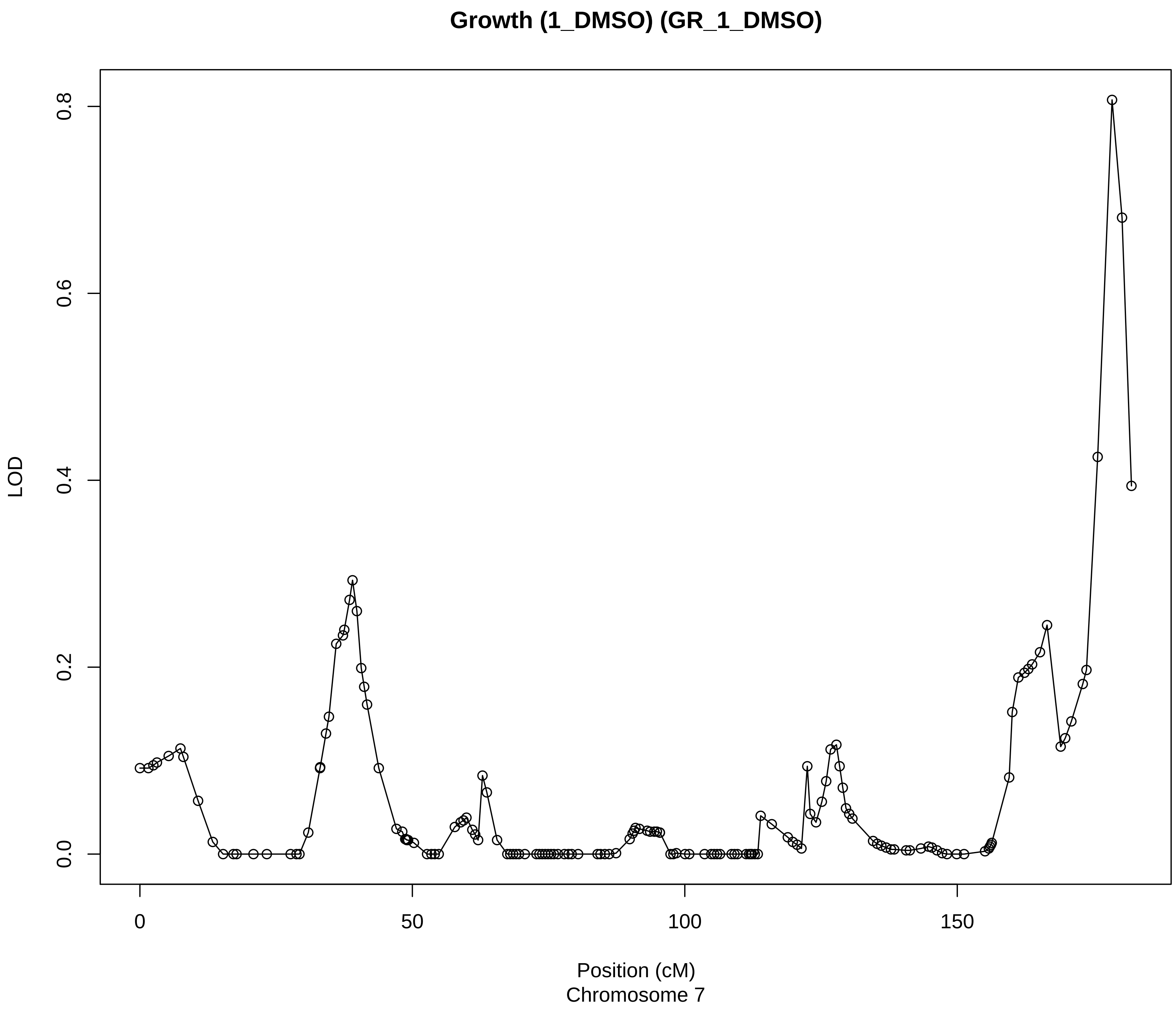
Growth (1_DMSO) (GR_1_DMSO)
LOD
0.0
0.2
0.4
0.6
0.8
0
50
100
150
Position (cM)
Chromosome 7

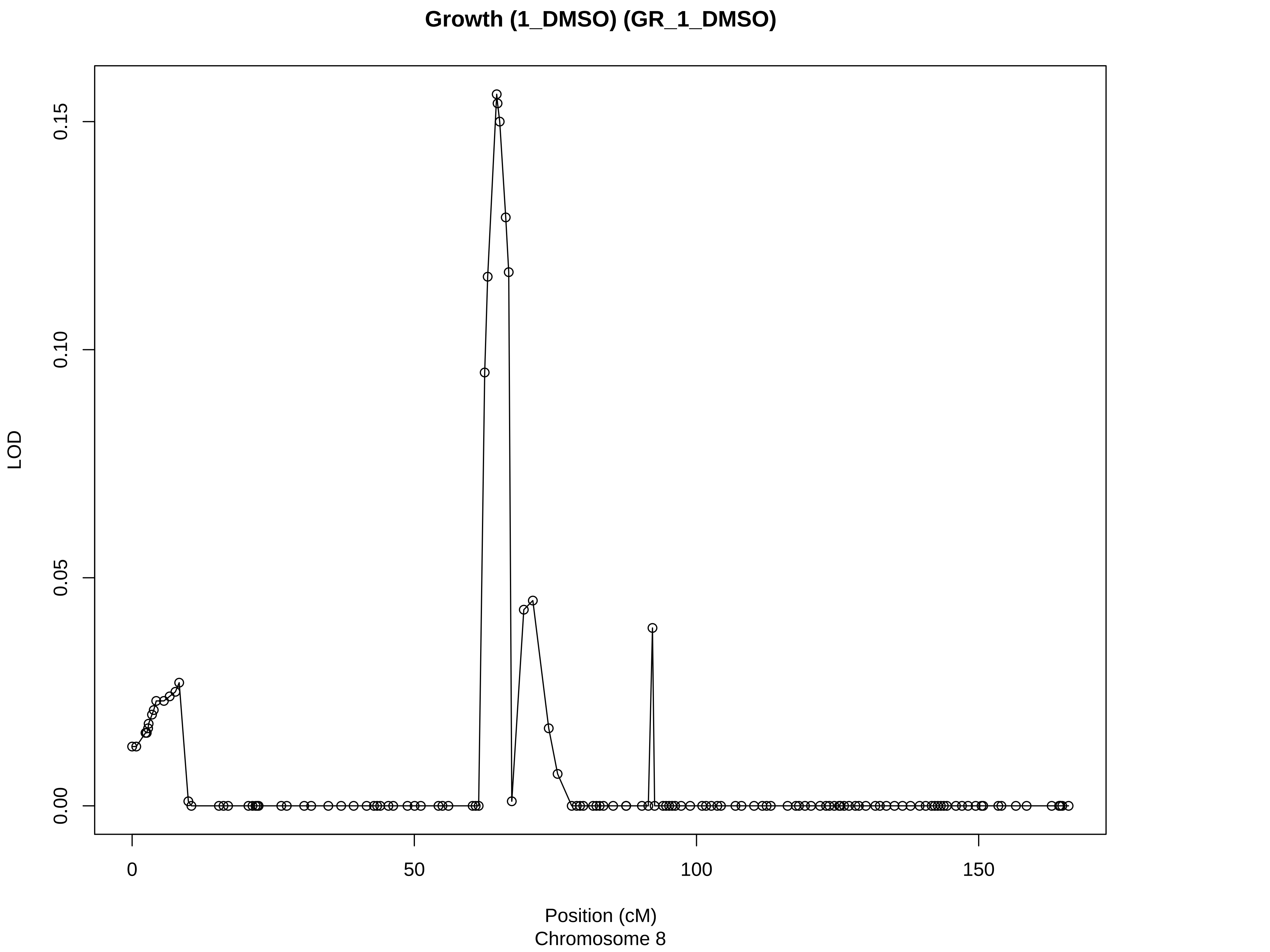

Growth (1_DMSO) (GR_1_DMSO)
LOD
0.00
0.05
0.10
0.15
0
50
100
150
Position (cM)
Chromosome 8

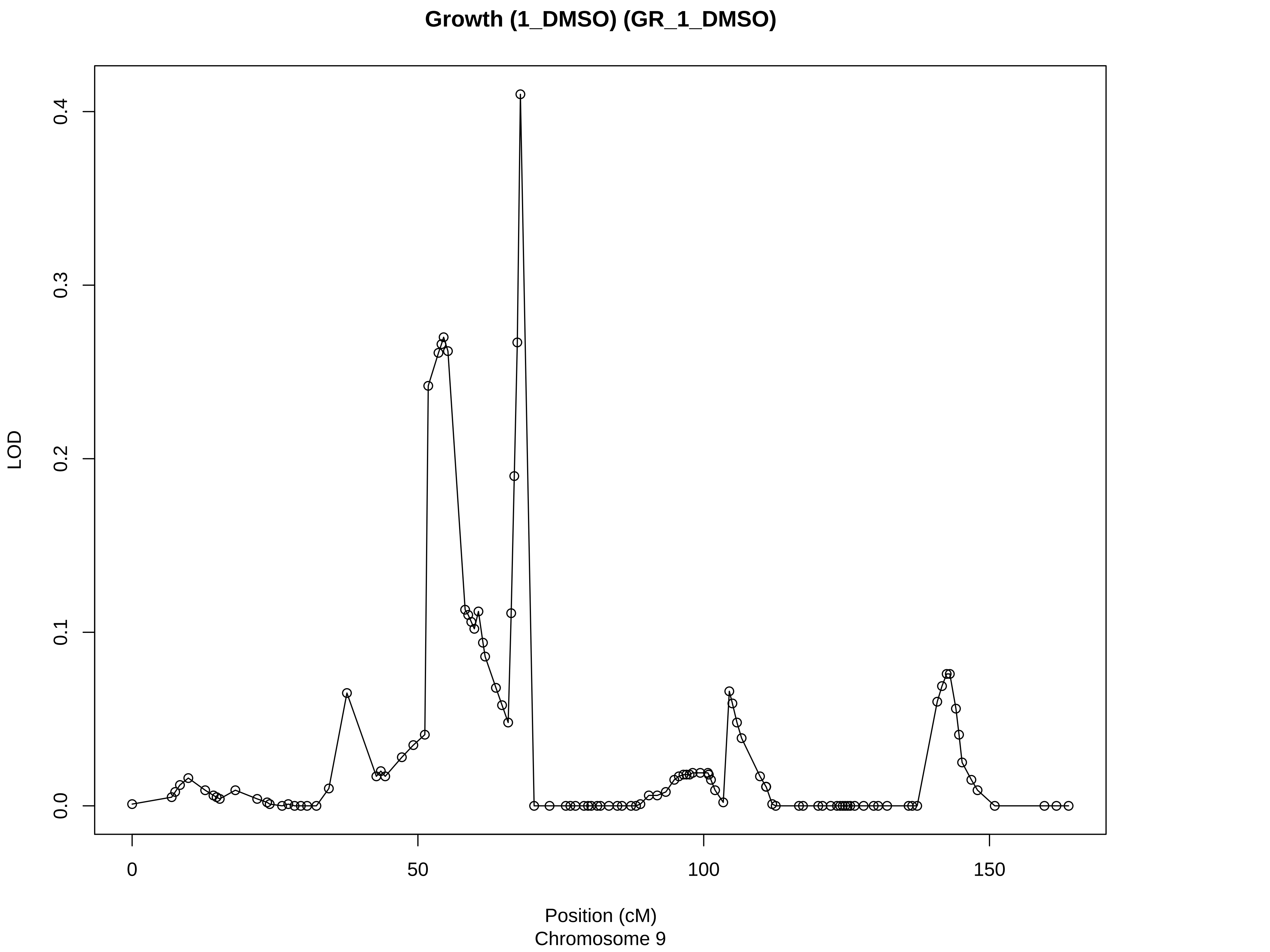
Growth (1_DMSO) (GR_1_DMSO)
LOD
0.0
0.1
0.2
0.3
0.4
0
50
100
150
Position (cM)
Chromosome 9

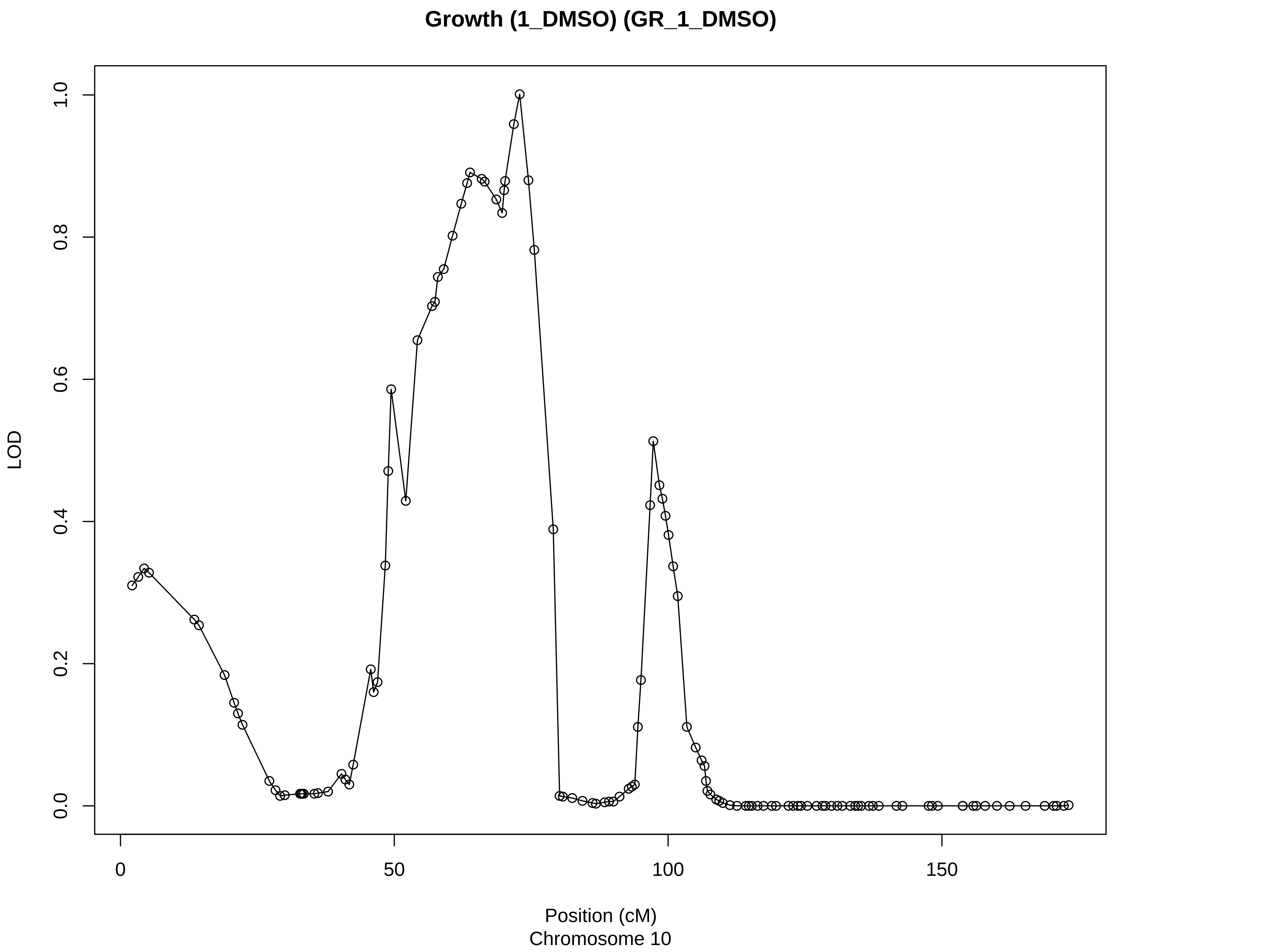
Growth (1_DMSO) (GR_1_DMSO)
LOD
0.0
0.2
0.4
0.6
0.8
1.0
0
50
100
150
Position (cM)
Chromosome 10

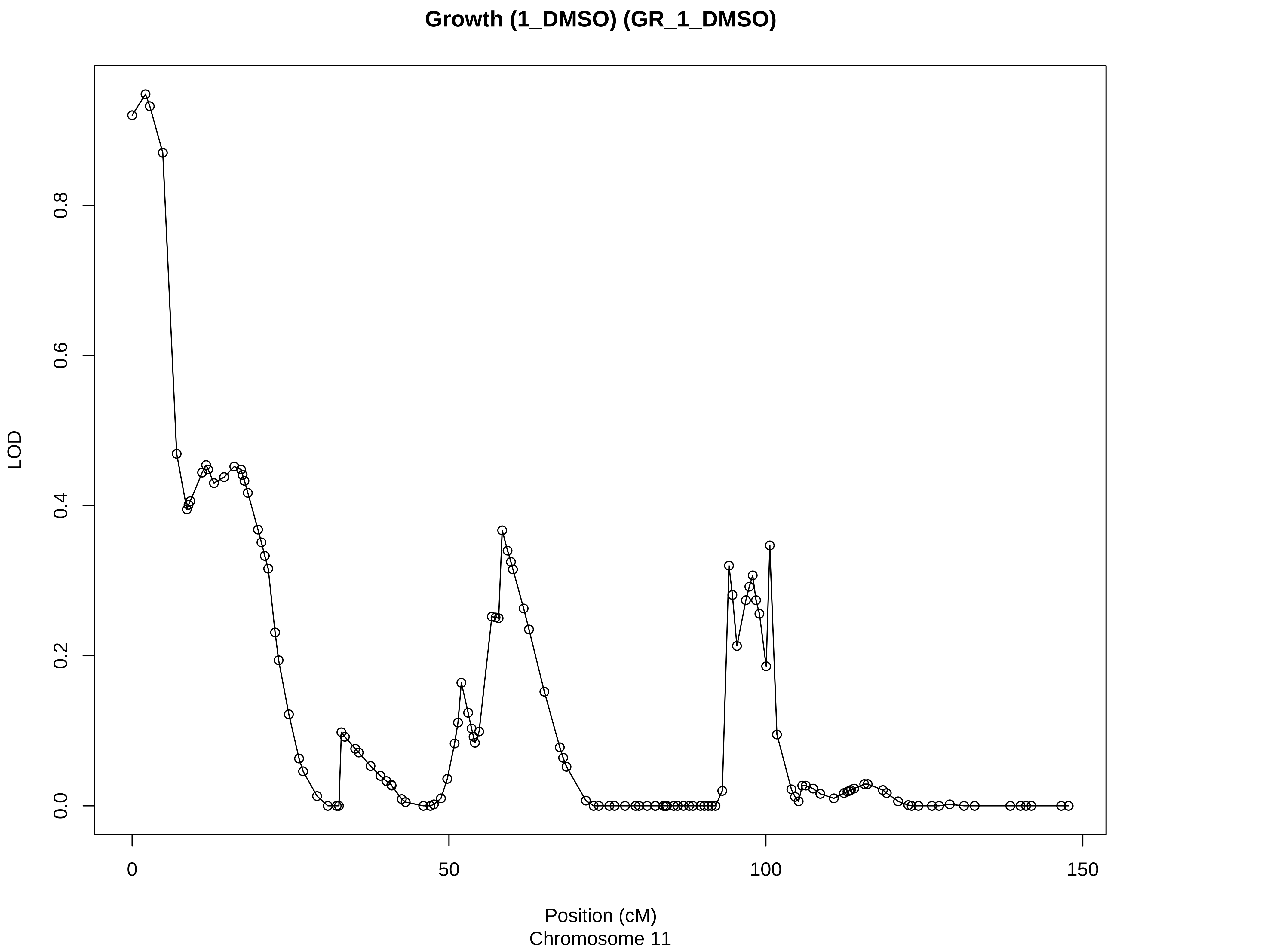

Growth (1_DMSO) (GR_1_DMSO)
LOD
0.0
0.2
0.4
0.6
0.8
0
50
100
150
Position (cM)
Chromosome 11

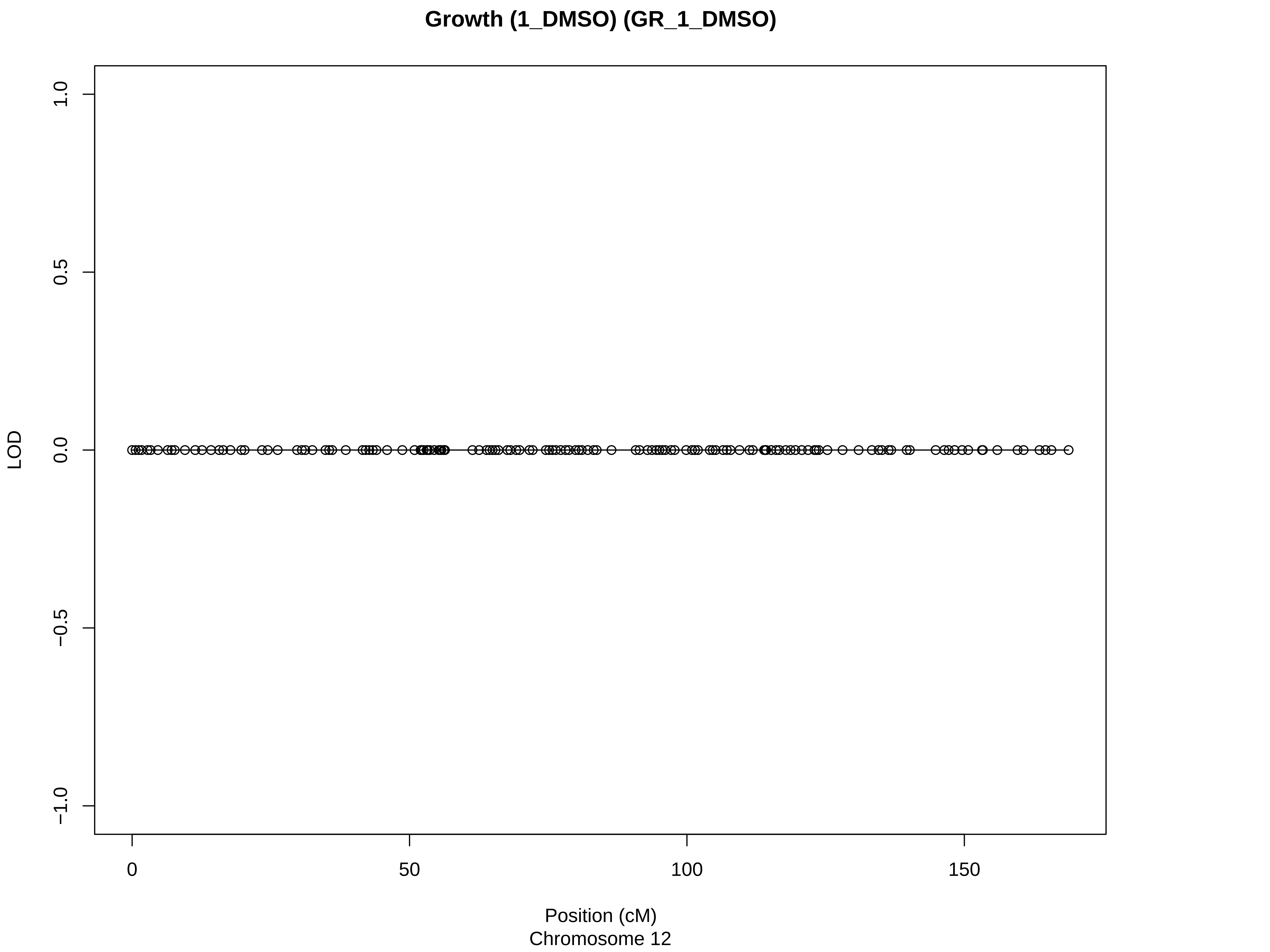

Growth (1_DMSO) (GR_1_DMSO)
LOD
1.0
0.5
0.0
−0.5
−1.0
0
50
100
150
Position (cM)
Chromosome 12

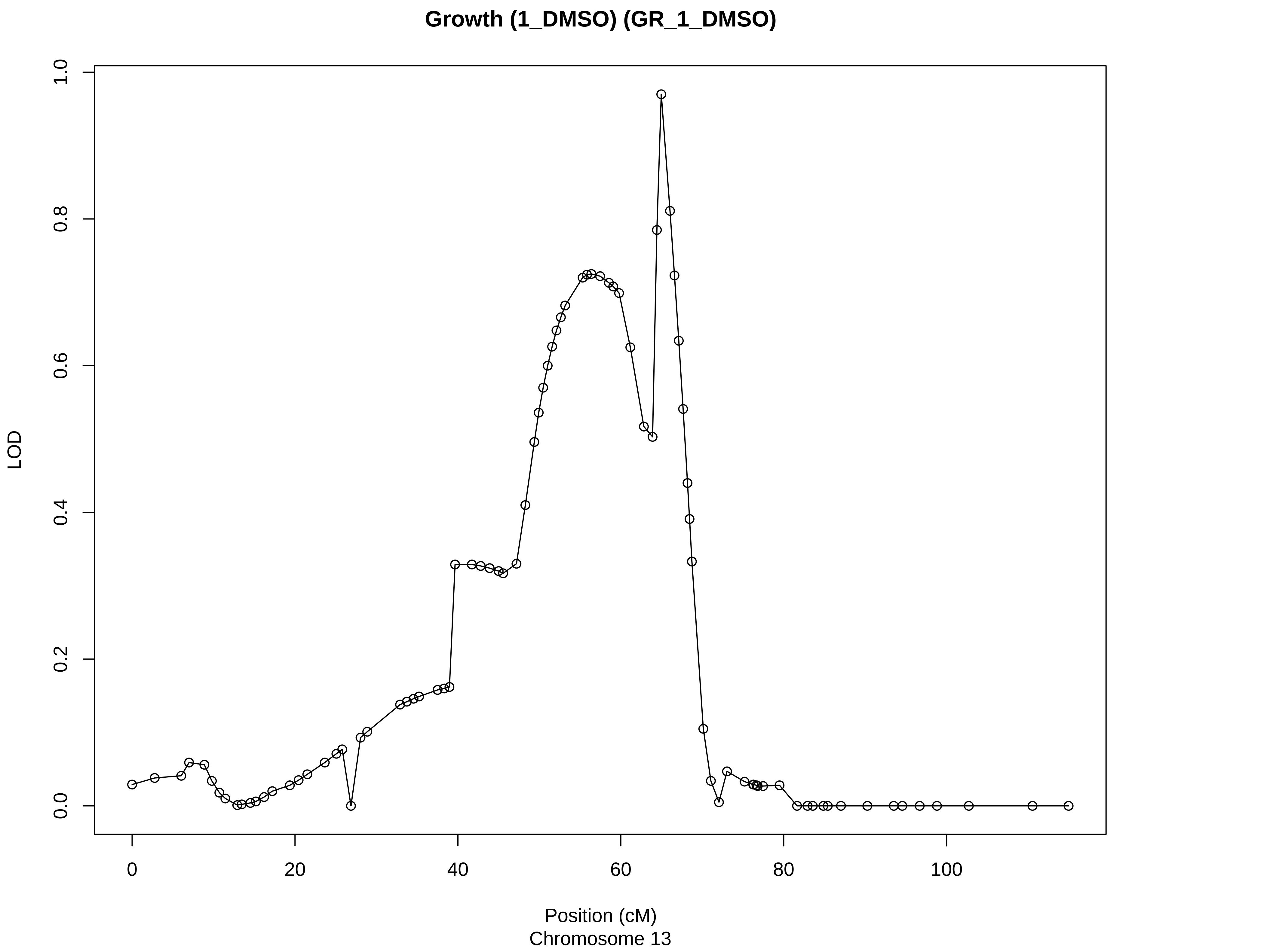
Growth (1_DMSO) (GR_1_DMSO)
LOD
0.0
0.2
0.4
0.6
0.8
1.0
0
20
40
60
80
100
Position (cM)
Chromosome 13

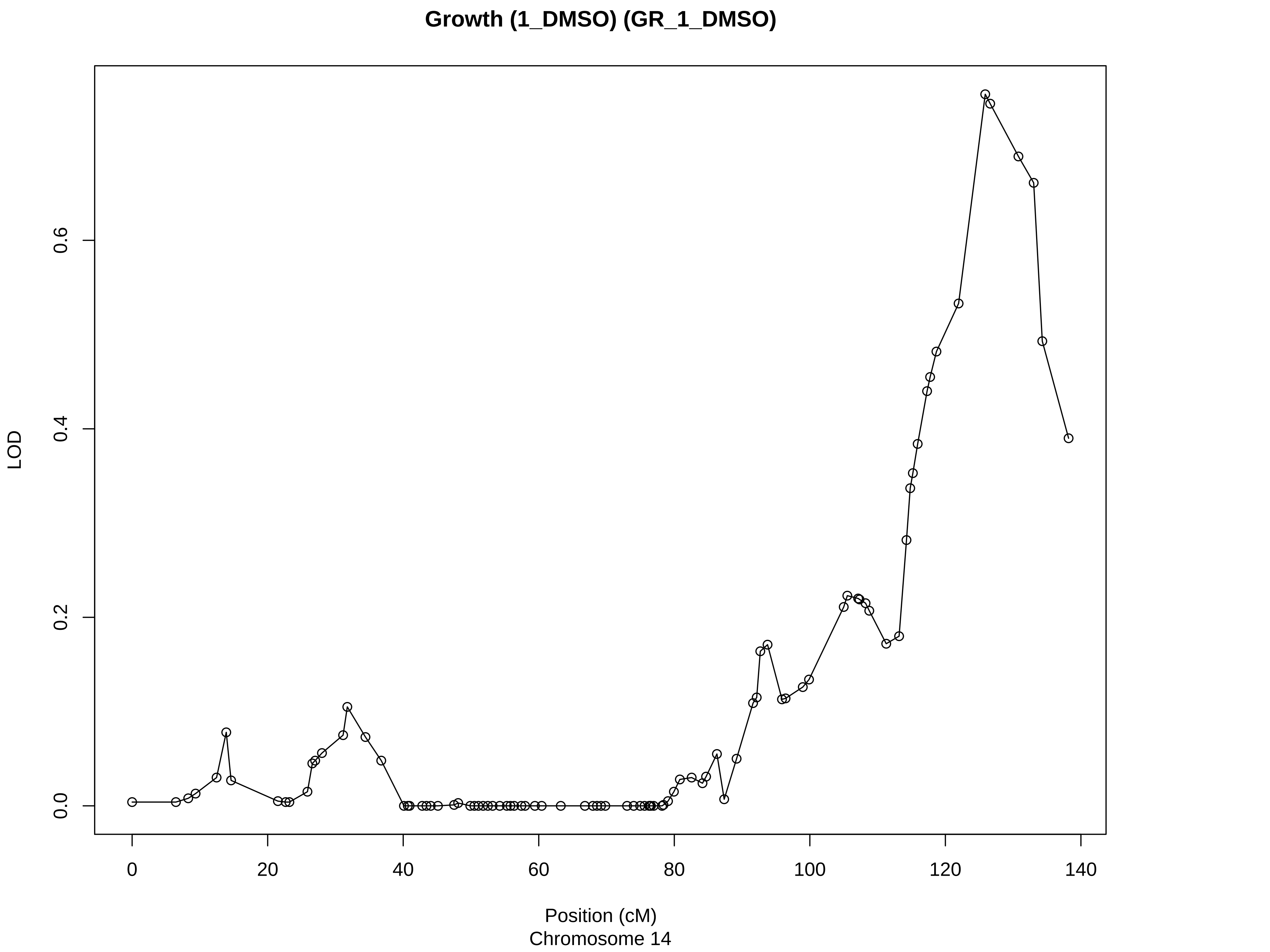

Growth (1_DMSO) (GR_1_DMSO)
LOD
0.0
0.2
0.4
0.6
0
20
40
60
80
100
120
140
Position (cM)
Chromosome 14

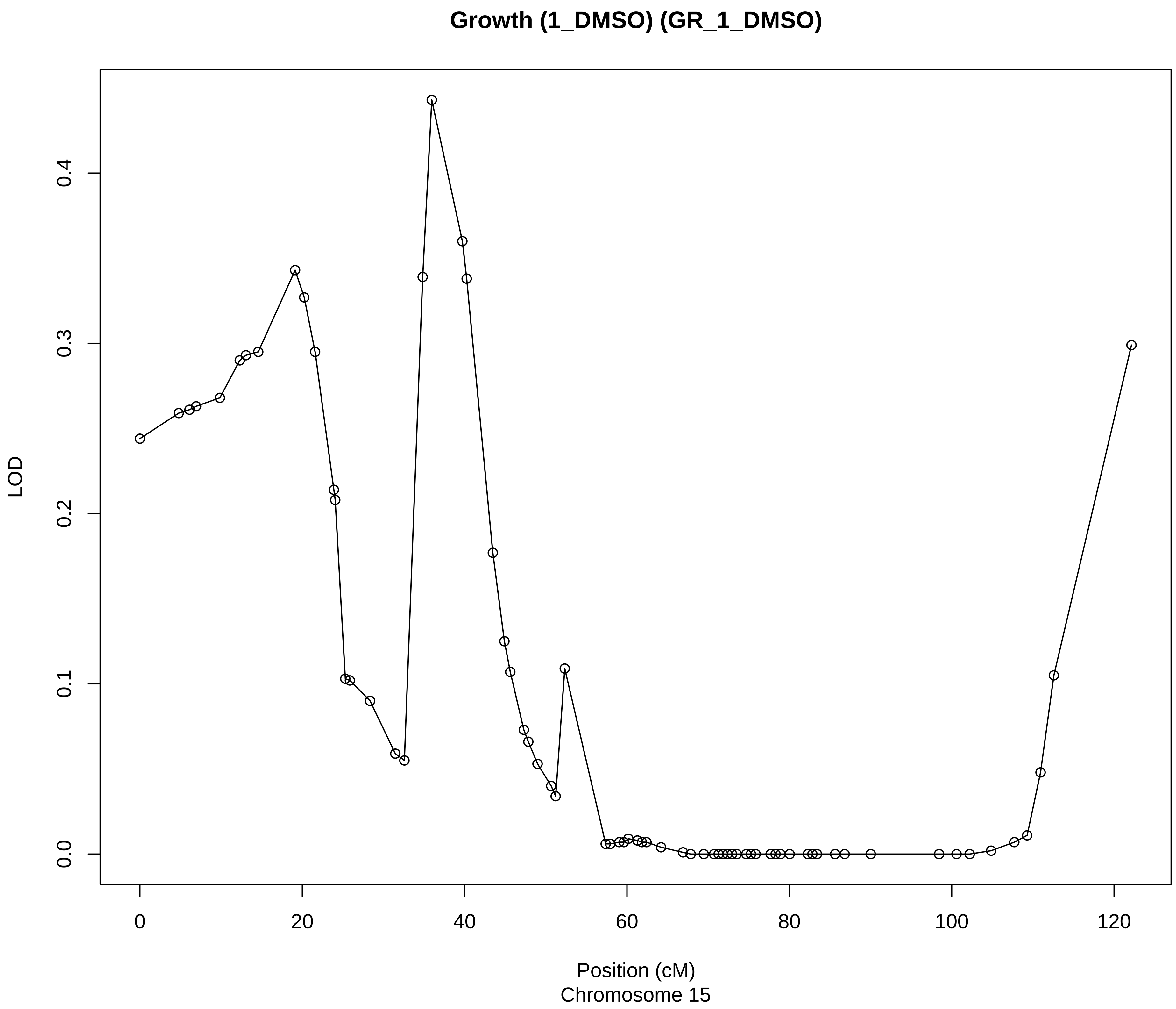
Growth (1_DMSO) (GR_1_DMSO)
LOD
0.0
0.1
0.2
0.3
0.4
0
20
40
60
80
100
120
Position (cM)
Chromosome 15

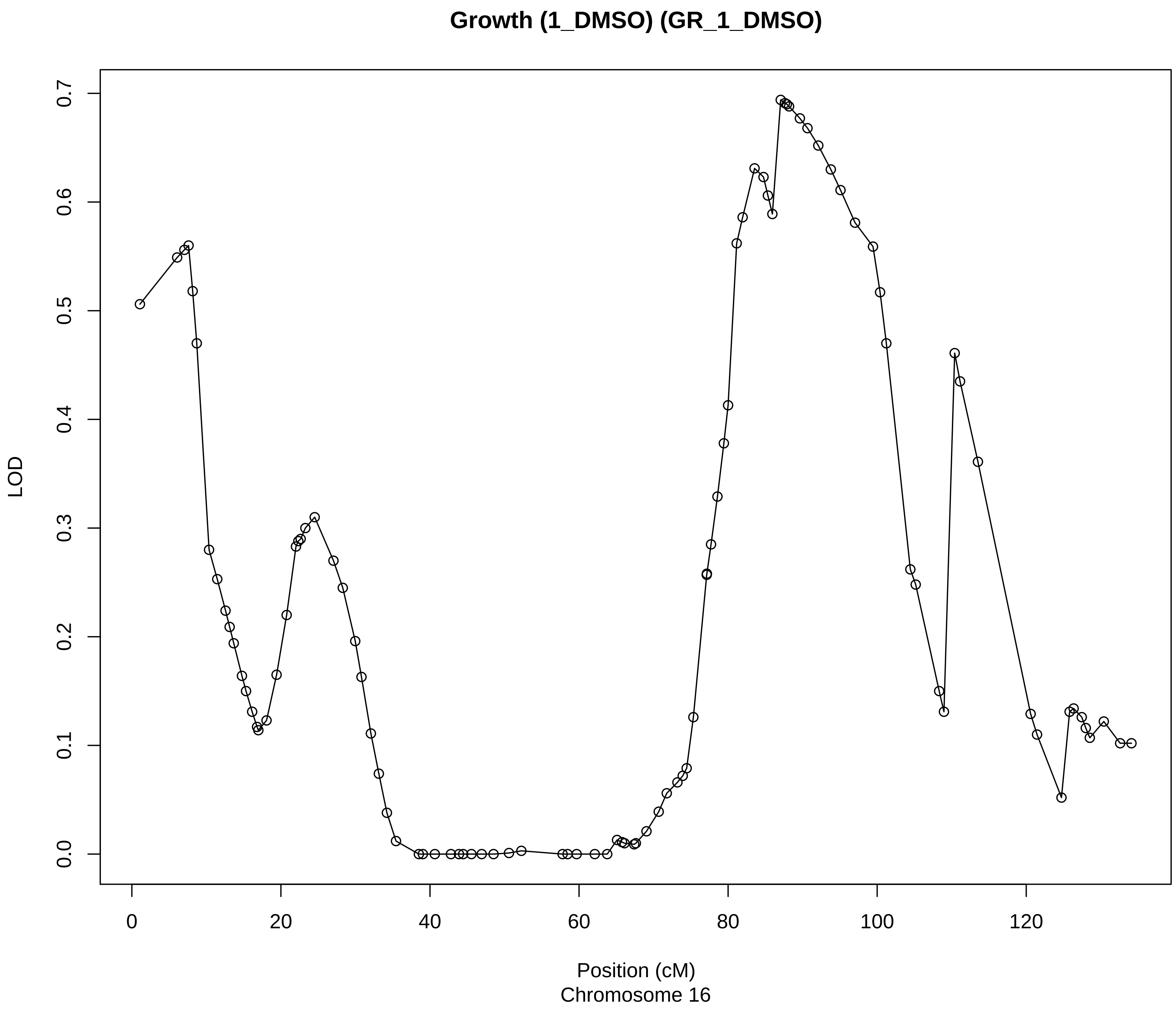
Growth (1_DMSO) (GR_1_DMSO)
LOD
0.0
0.1
0.2
0.3
0.4
0.5
0.6
0.7
0
20
40
60
80
100
120
Position (cM)
Chromosome 16

Growth (1_DMSO) (GR_1_DMSO)

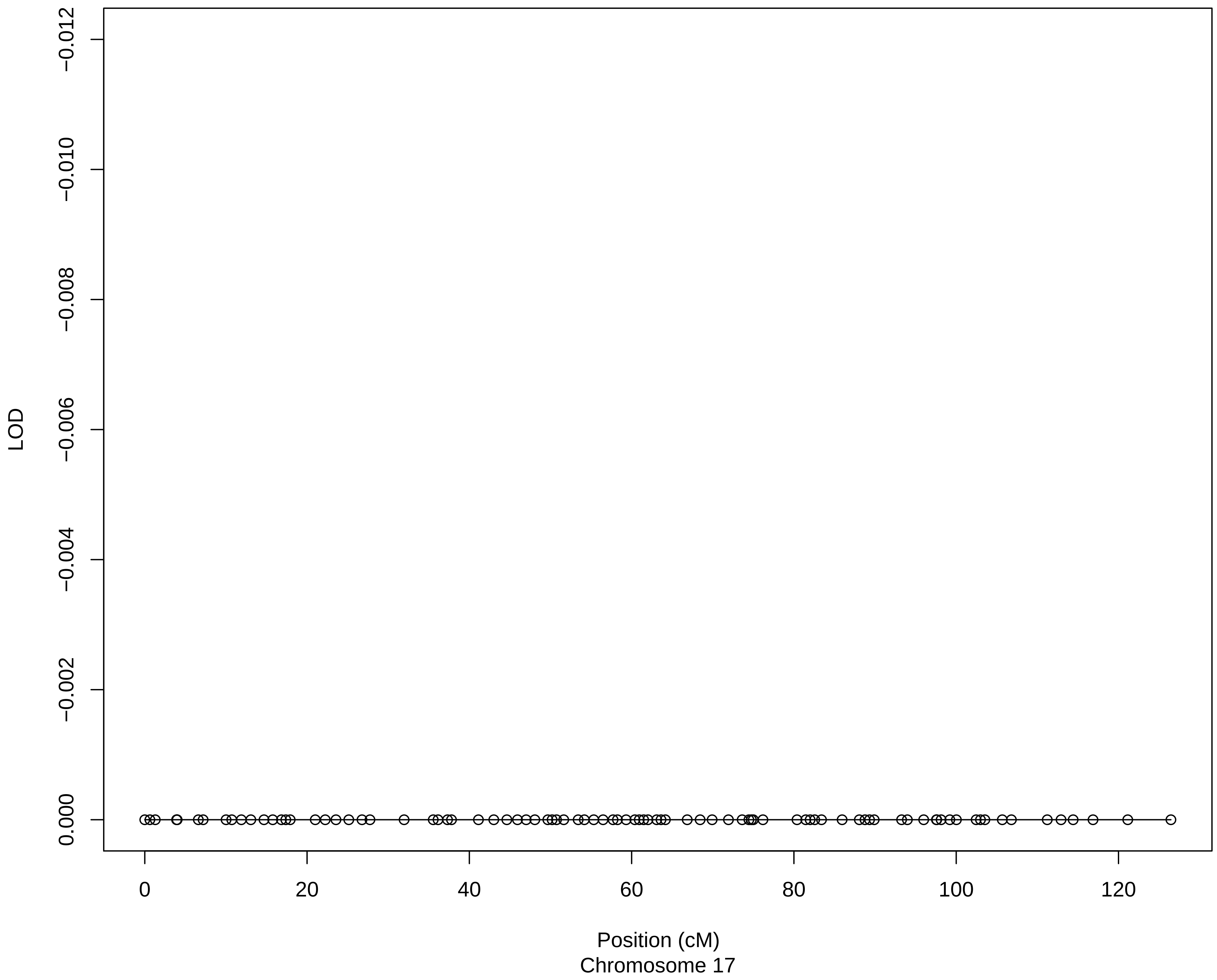

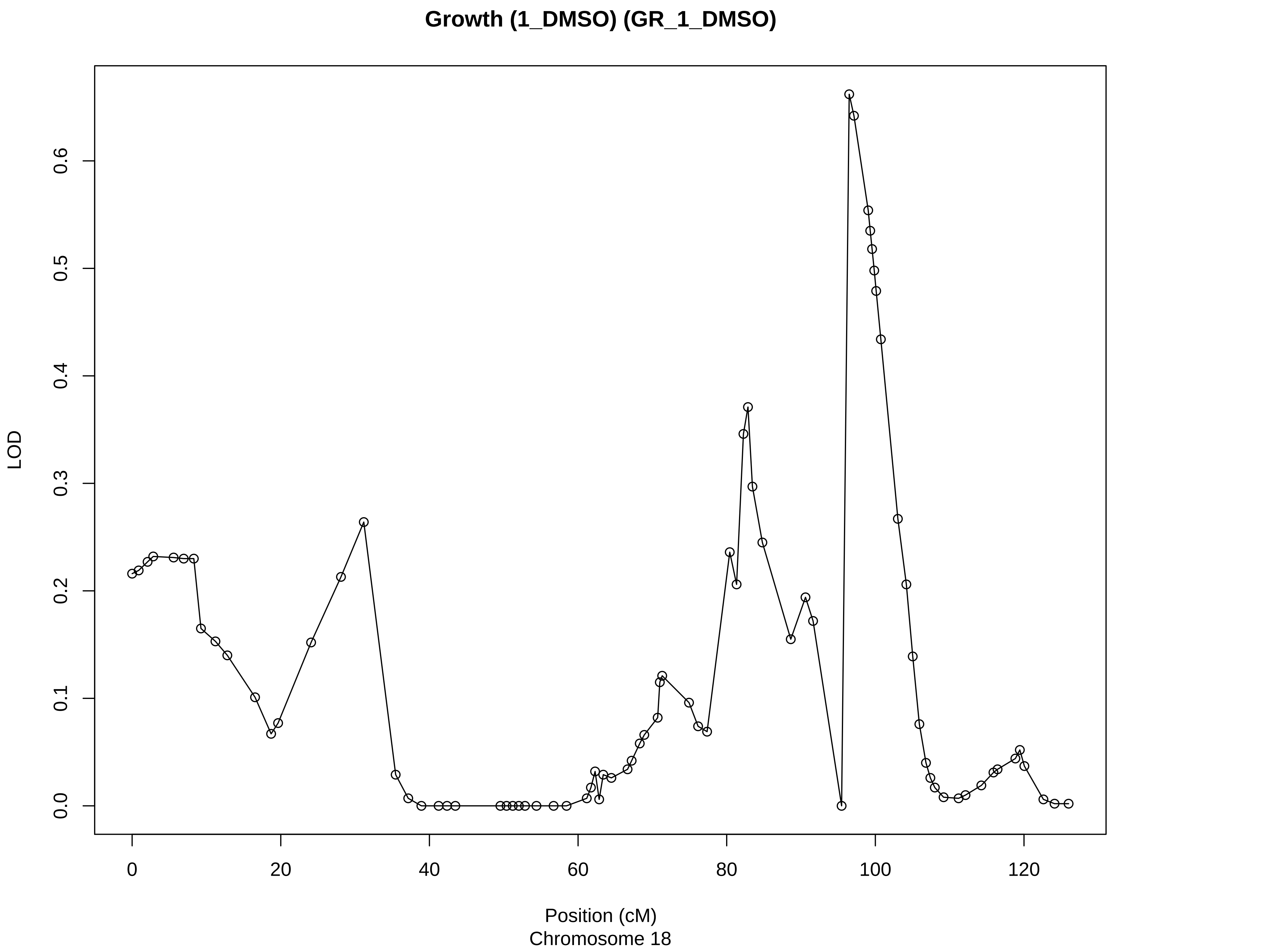
Growth (1_DMSO) (GR_1_DMSO)
LOD
0.0
0.1
0.2
0.3
0.4
0.5
0.6
0
20
40
60
80
100
120
Position (cM)
Chromosome 18

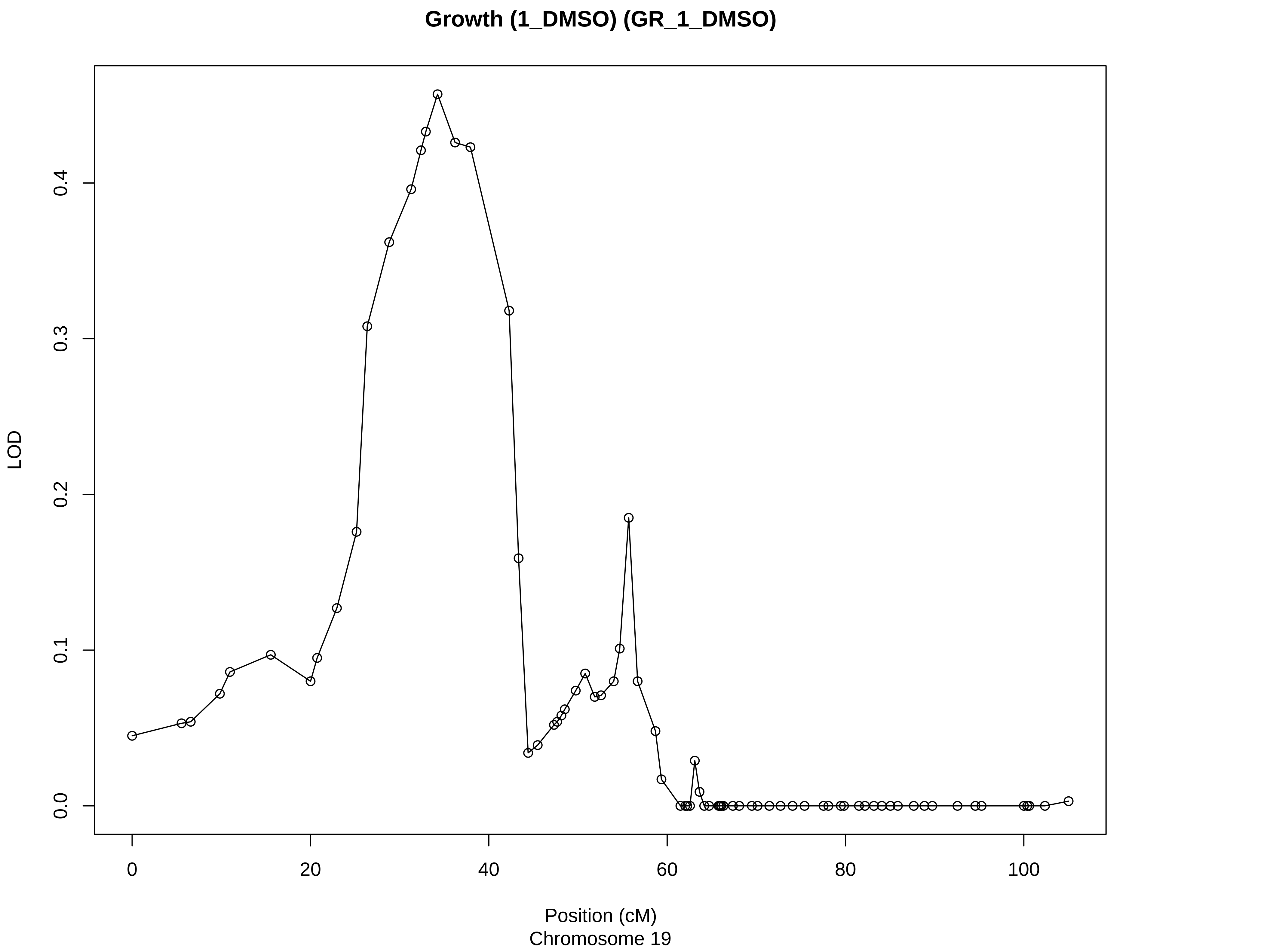
Growth (1_DMSO) (GR_1_DMSO)
LOD
0.0
0.1
0.2
0.3
0.4
0
20
40
60
80
100
Position (cM)
Chromosome 19

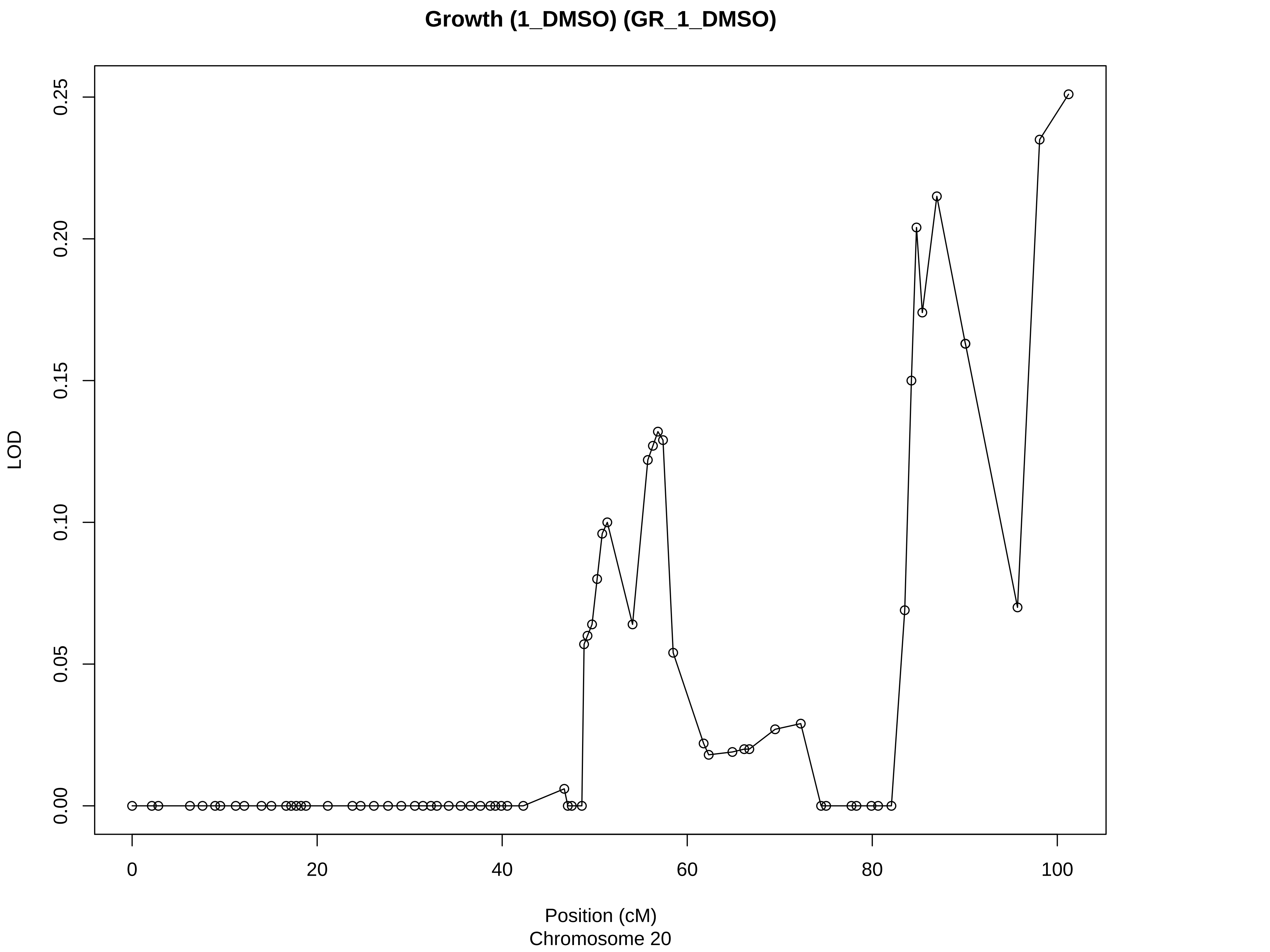

Growth (1_DMSO) (GR_1_DMSO)
LOD
0.00
0.05
0.10
0.15
0.20
0.25
0
20
40
60
80
100
Position (cM)
Chromosome 20

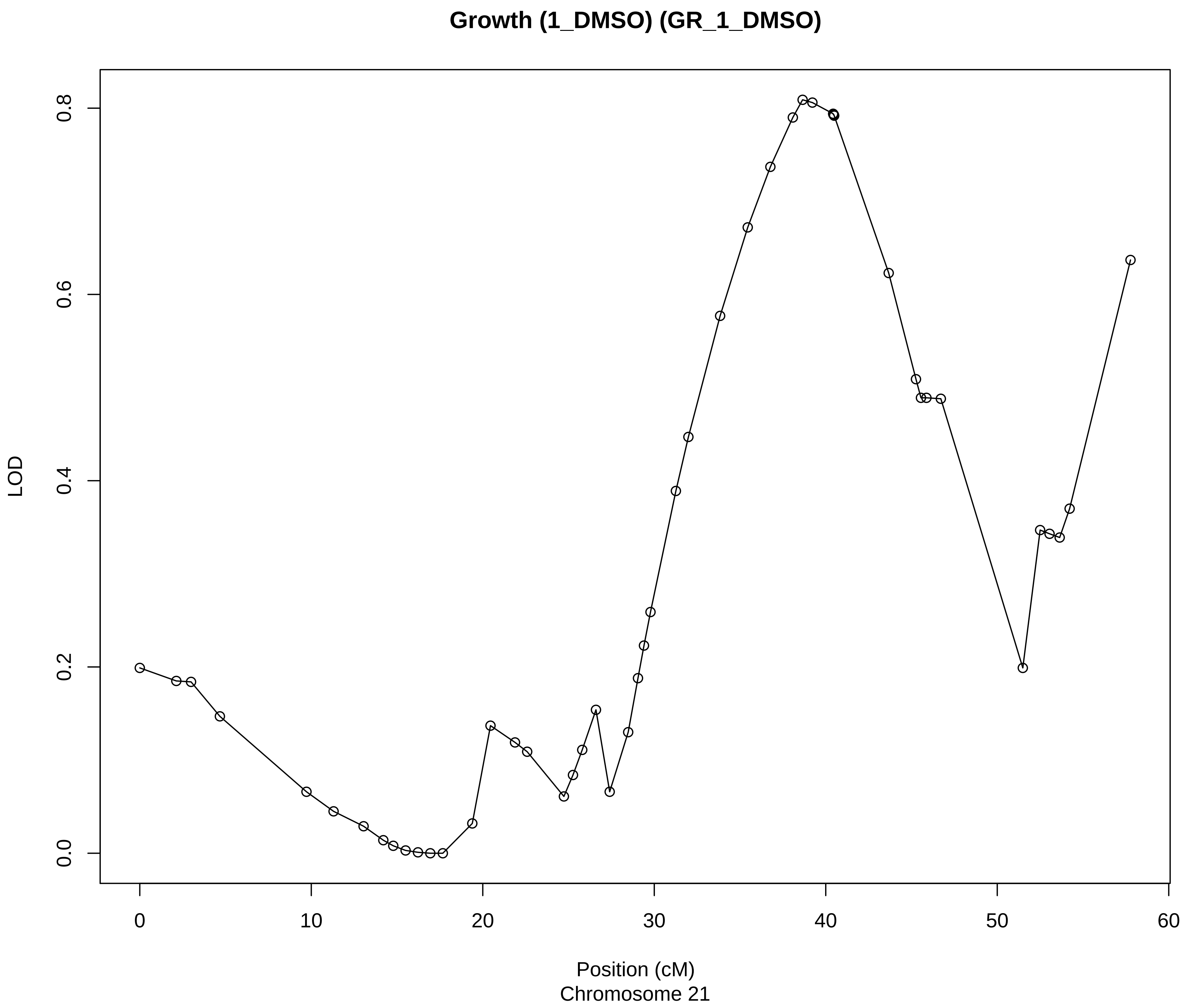

Growth (1_DMSO) (GR_1_DMSO)
LOD
0.0
0.2
0.4
0.6
0.8
0
10
20
30
40
50
60
Position (cM)
Chromosome 21

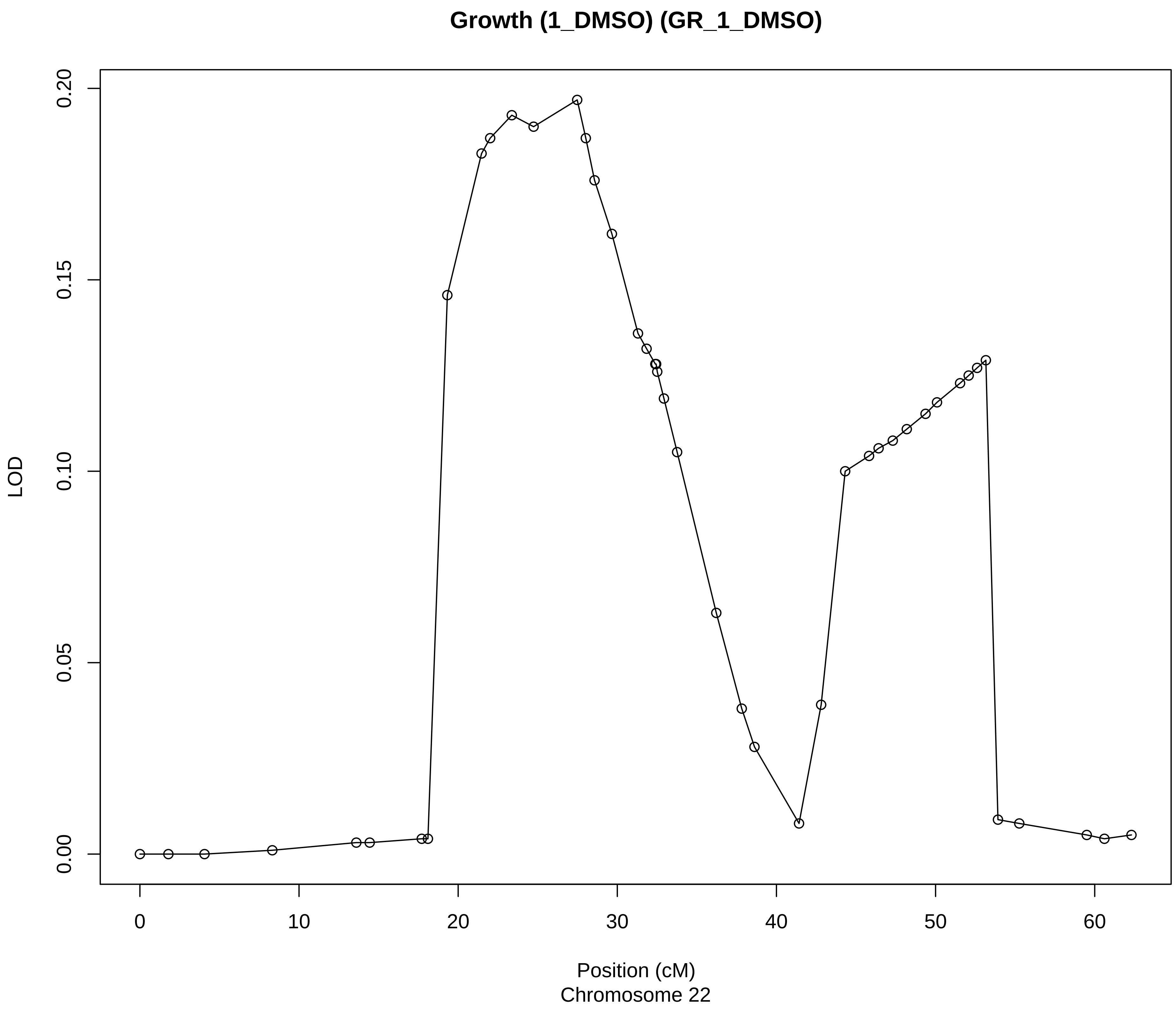
Growth (1_DMSO) (GR_1_DMSO)
LOD
0.00
0.05
0.10
0.15
0.20
0
10
20
30
40
50
60
Position (cM)
Chromosome 22

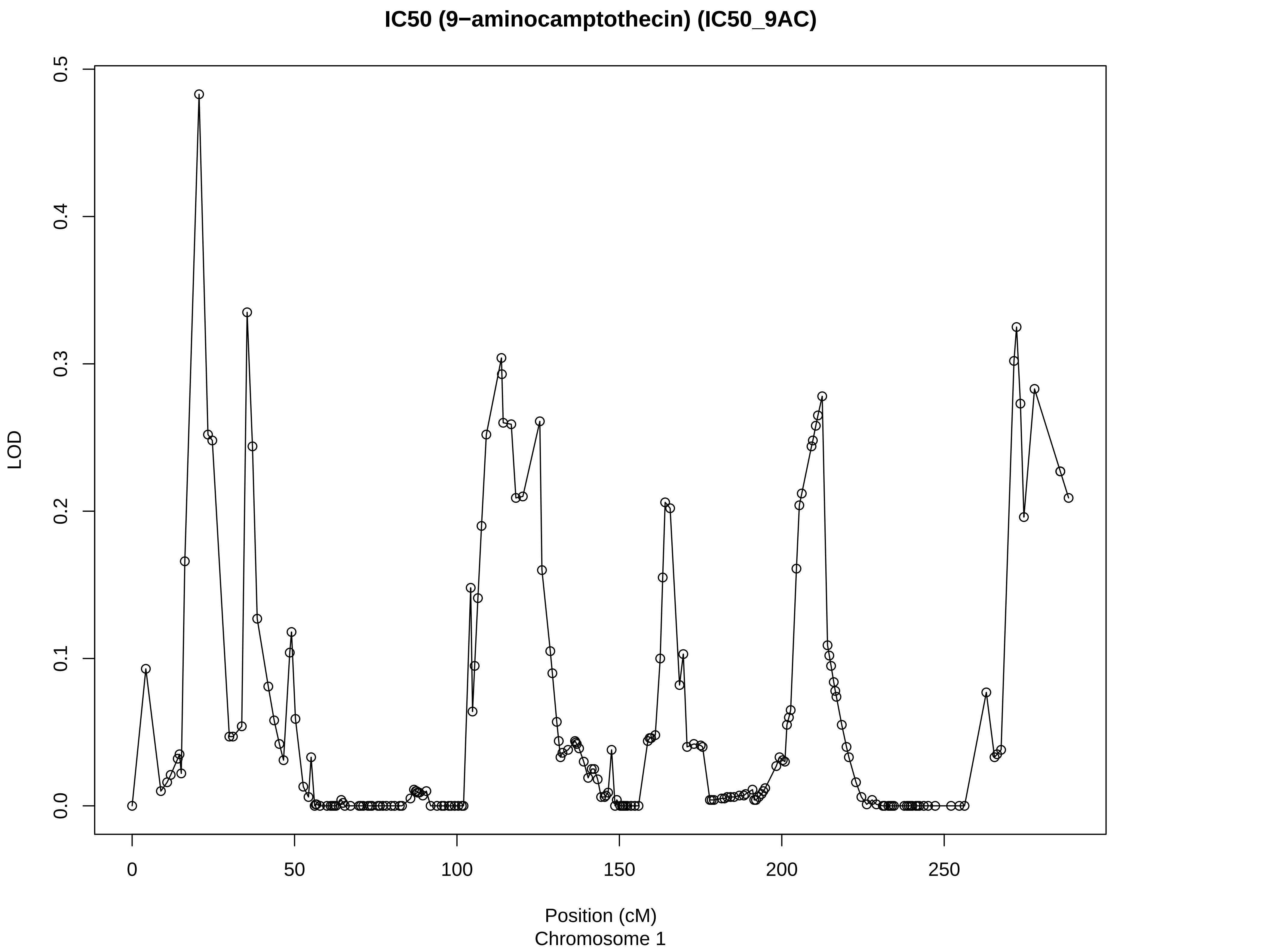
IC50 (9−aminocamptothecin) (IC50_9AC)
LOD
0.0
0.1
0.2
0.3
0.4
0.5
0
50
100
150
200
250
Position (cM)
Chromosome 1

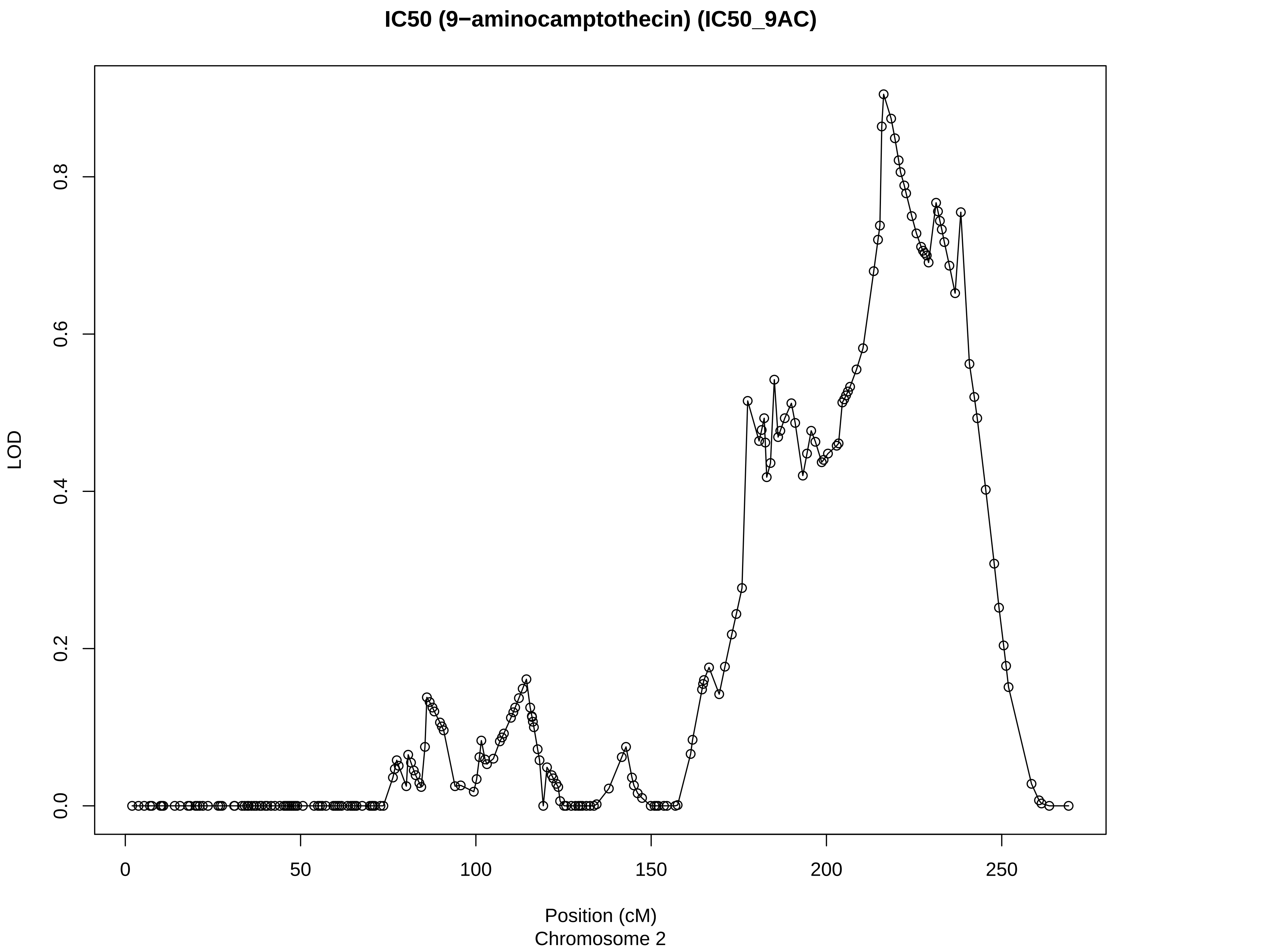

IC50 (9−aminocamptothecin) (IC50_9AC)
LOD
0.0
0.2
0.4
0.6
0.8
0
50
100
150
200
250
Position (cM)
Chromosome 2

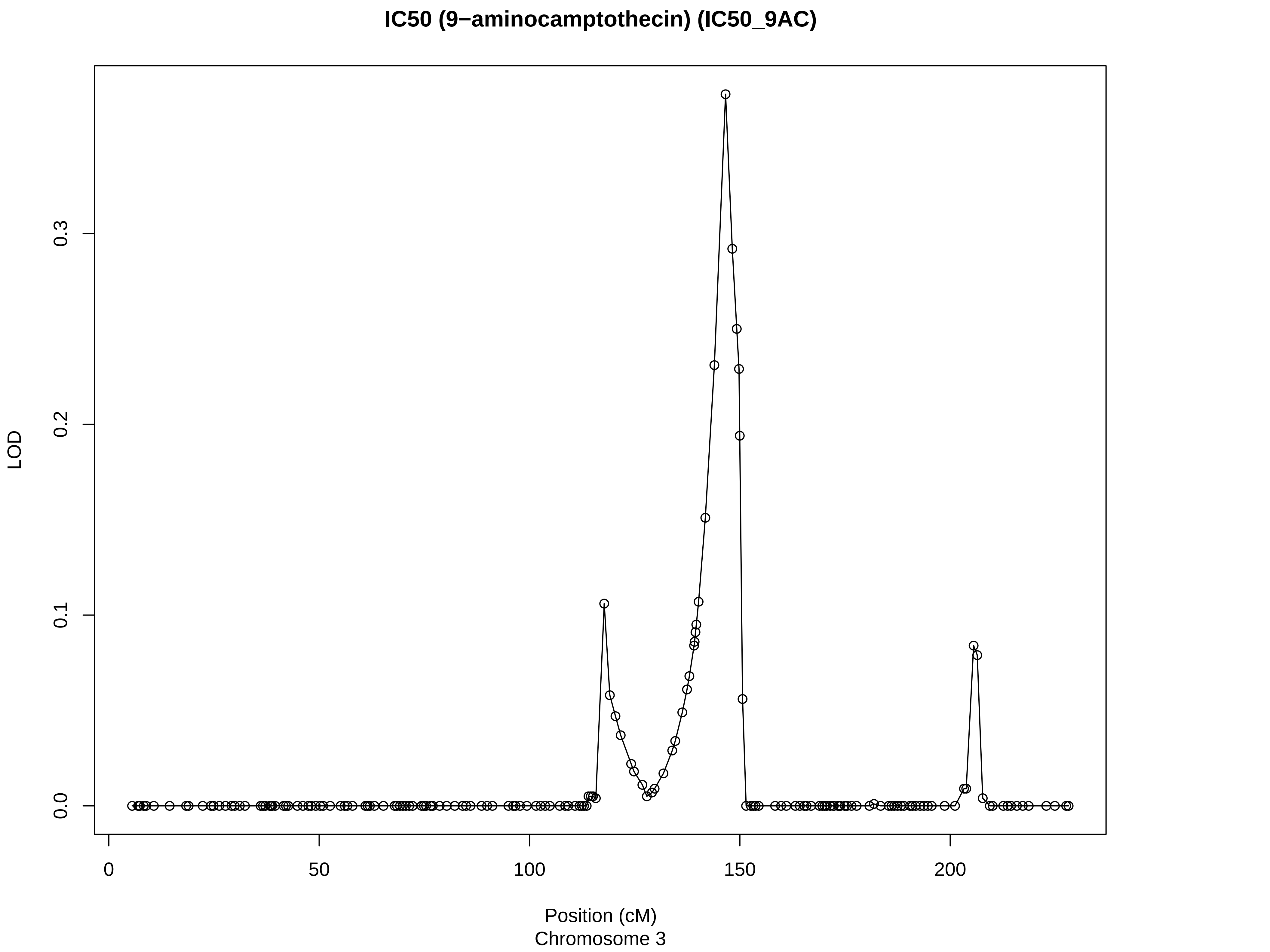
IC50 (9−aminocamptothecin) (IC50_9AC)
LOD
0.0
0.1
0.2
0.3
0
50
100
150
200
Position (cM)
Chromosome 3

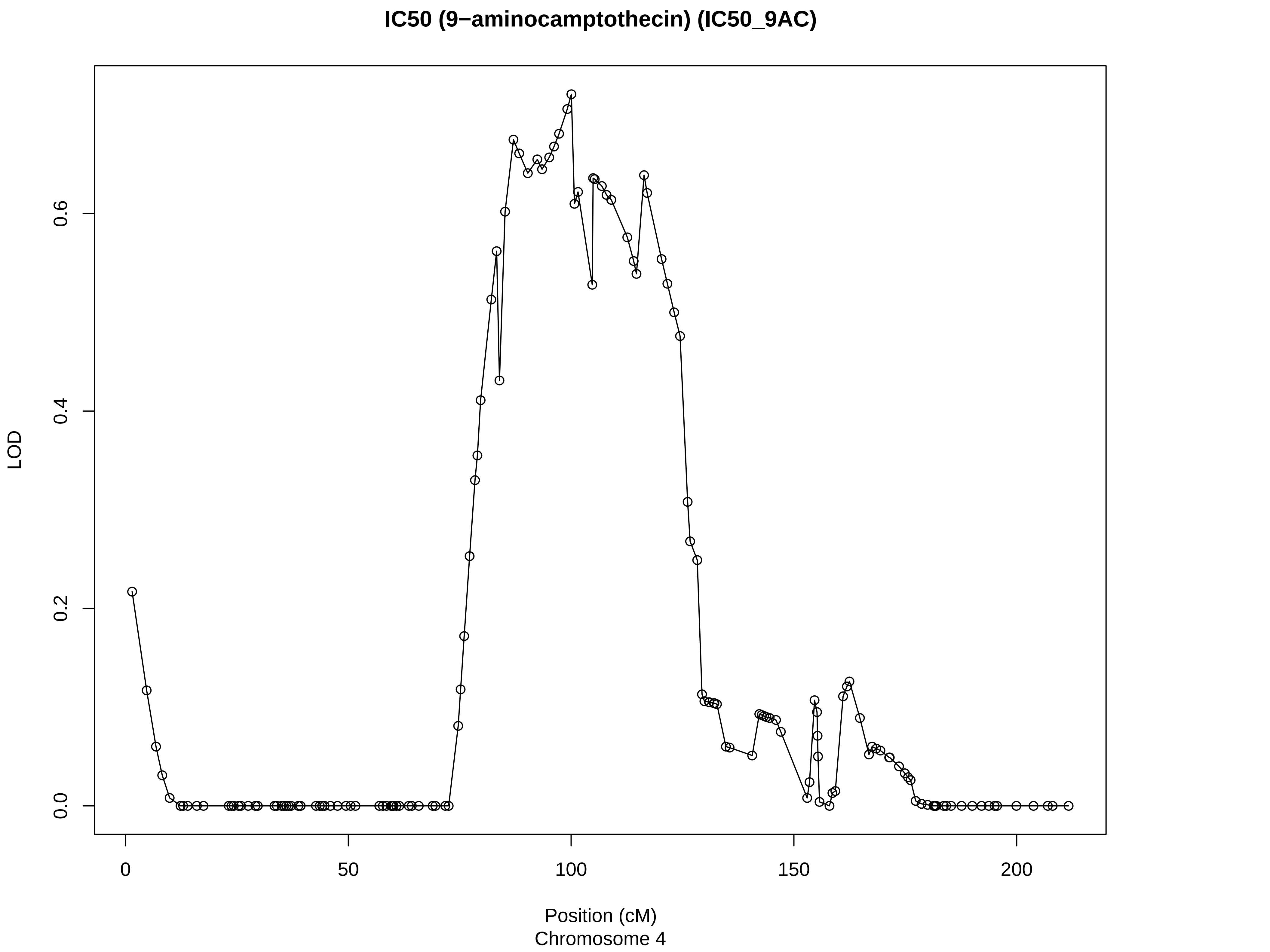
IC50 (9−aminocamptothecin) (IC50_9AC)
LOD
0.0
0.2
0.4
0.6
0
50
100
150
200
Position (cM)
Chromosome 4

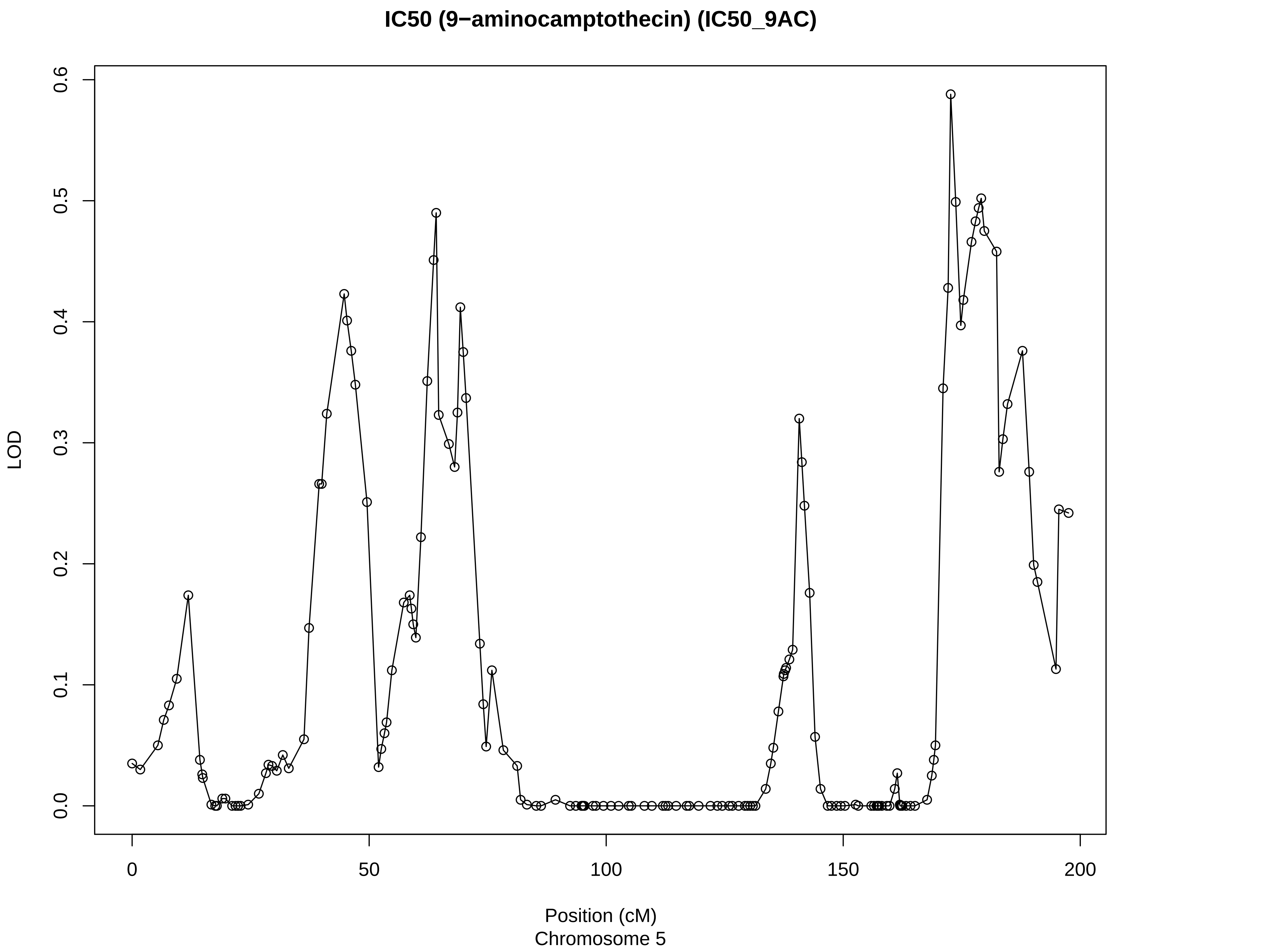
IC50 (9−aminocamptothecin) (IC50_9AC)
LOD
0.0
0.1
0.2
0.3
0.4
0.5
0.6
0
50
100
150
200
Position (cM)
Chromosome 5

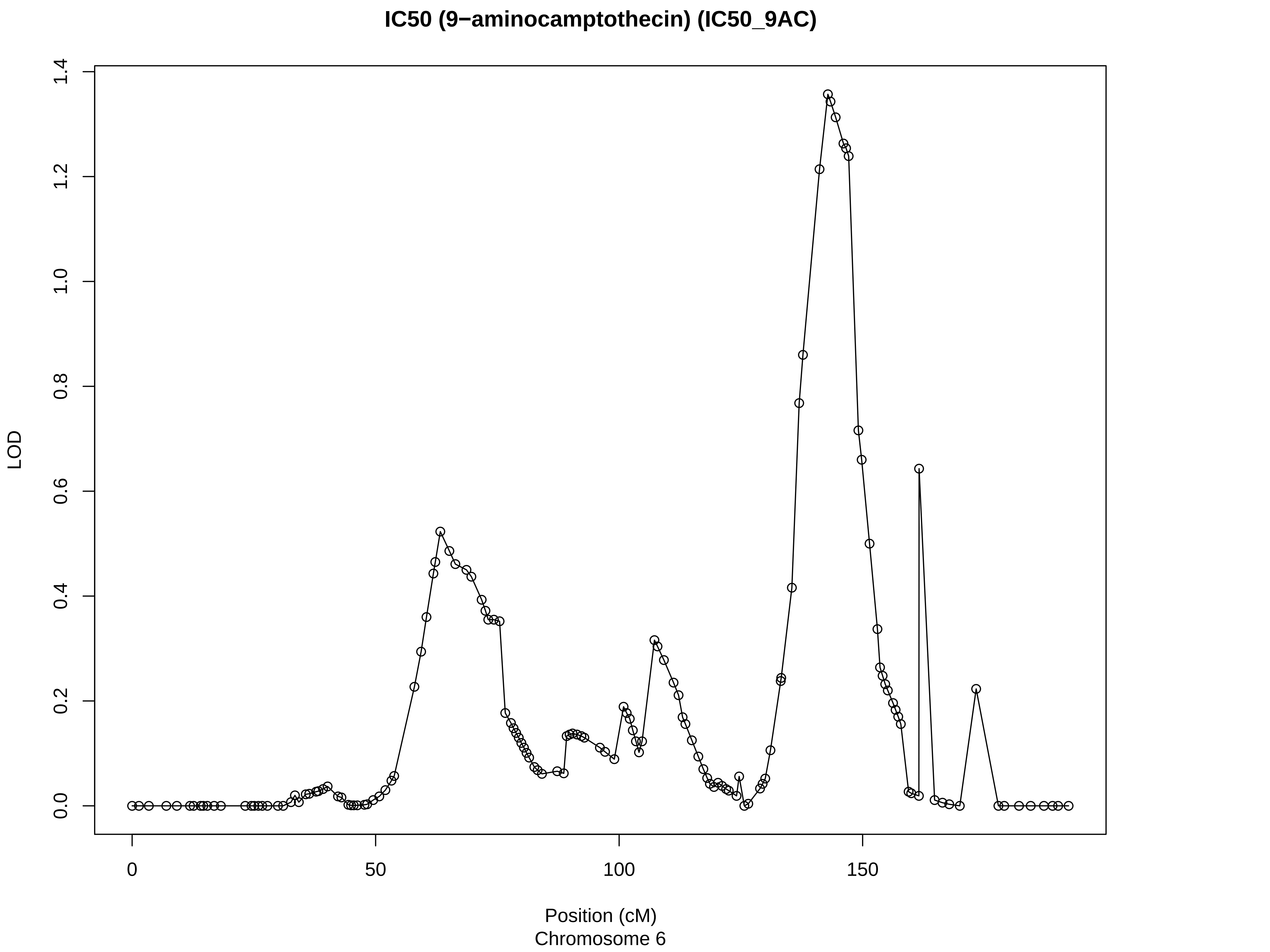

IC50 (9−aminocamptothecin) (IC50_9AC)
LOD
0.0
0.2
0.4
0.6
0.8
1.0
1.2
1.4
0
50
100
150
Position (cM)
Chromosome 6

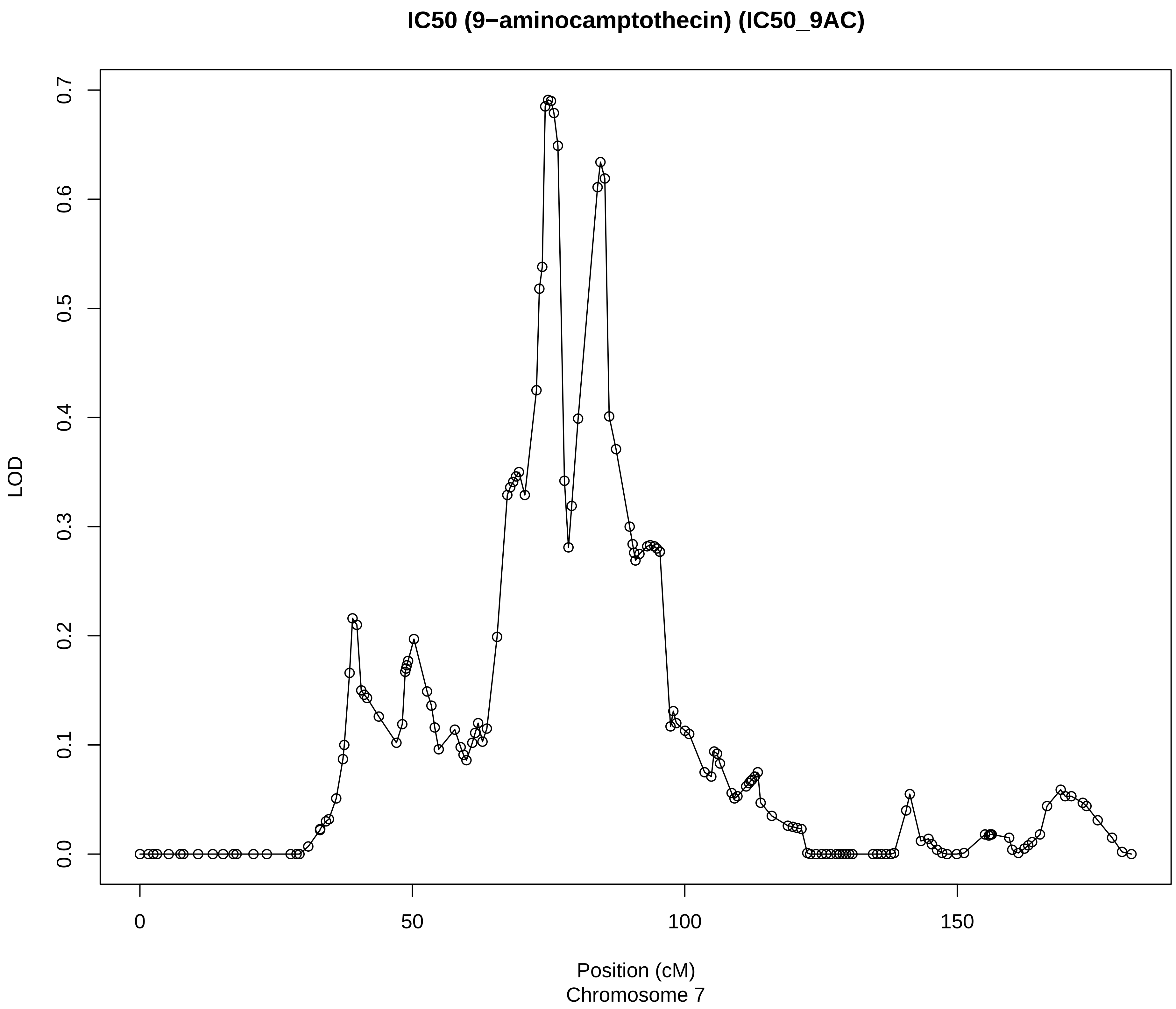

IC50 (9−aminocamptothecin) (IC50_9AC)
LOD
0.0
0.1
0.2
0.3
0.4
0.5
0.6
0.7
0
50
100
150
Position (cM)
Chromosome 7

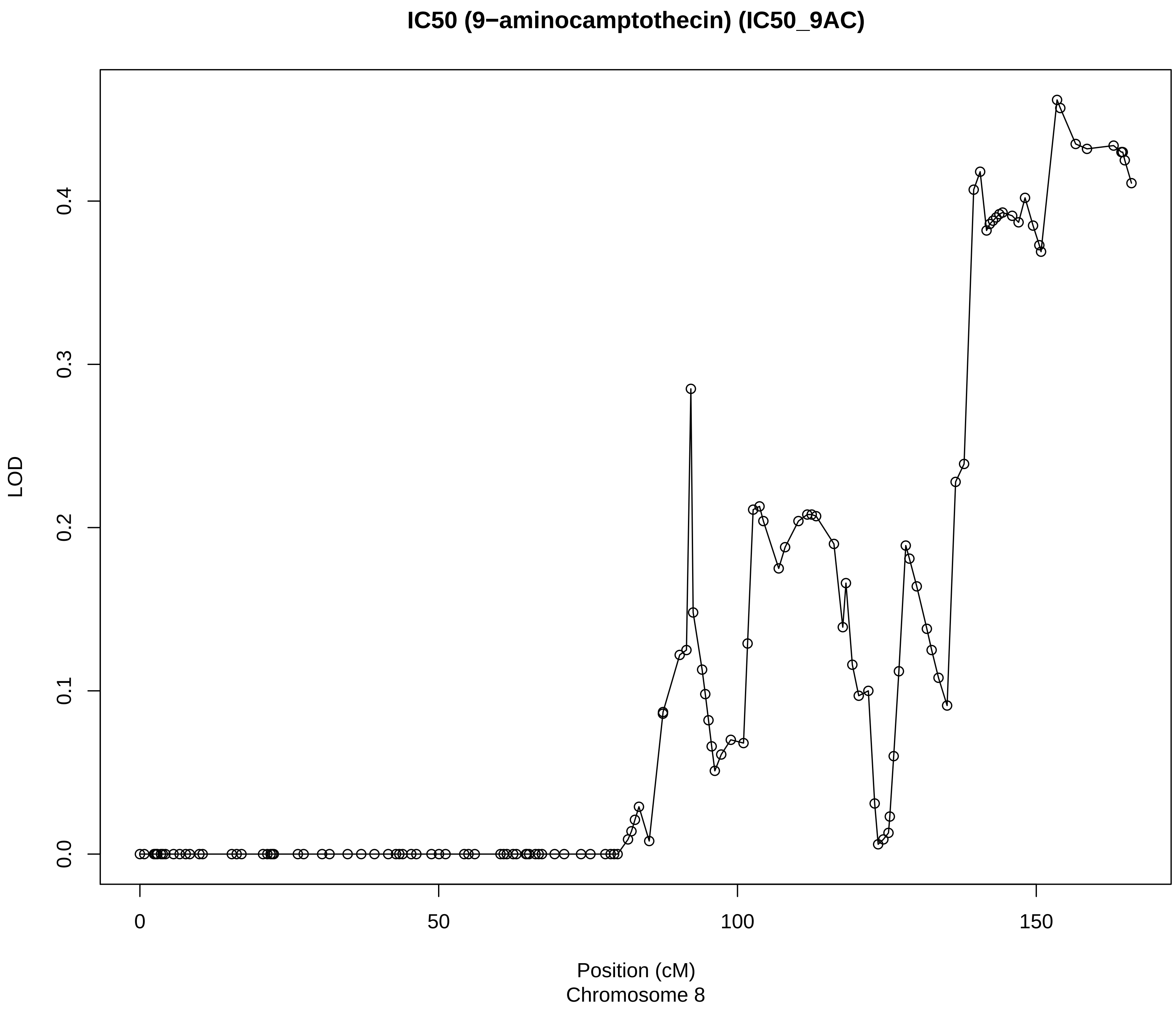
IC50 (9−aminocamptothecin) (IC50_9AC)
LOD
0.0
0.1
0.2
0.3
0.4
0
50
100
150
Position (cM)
Chromosome 8

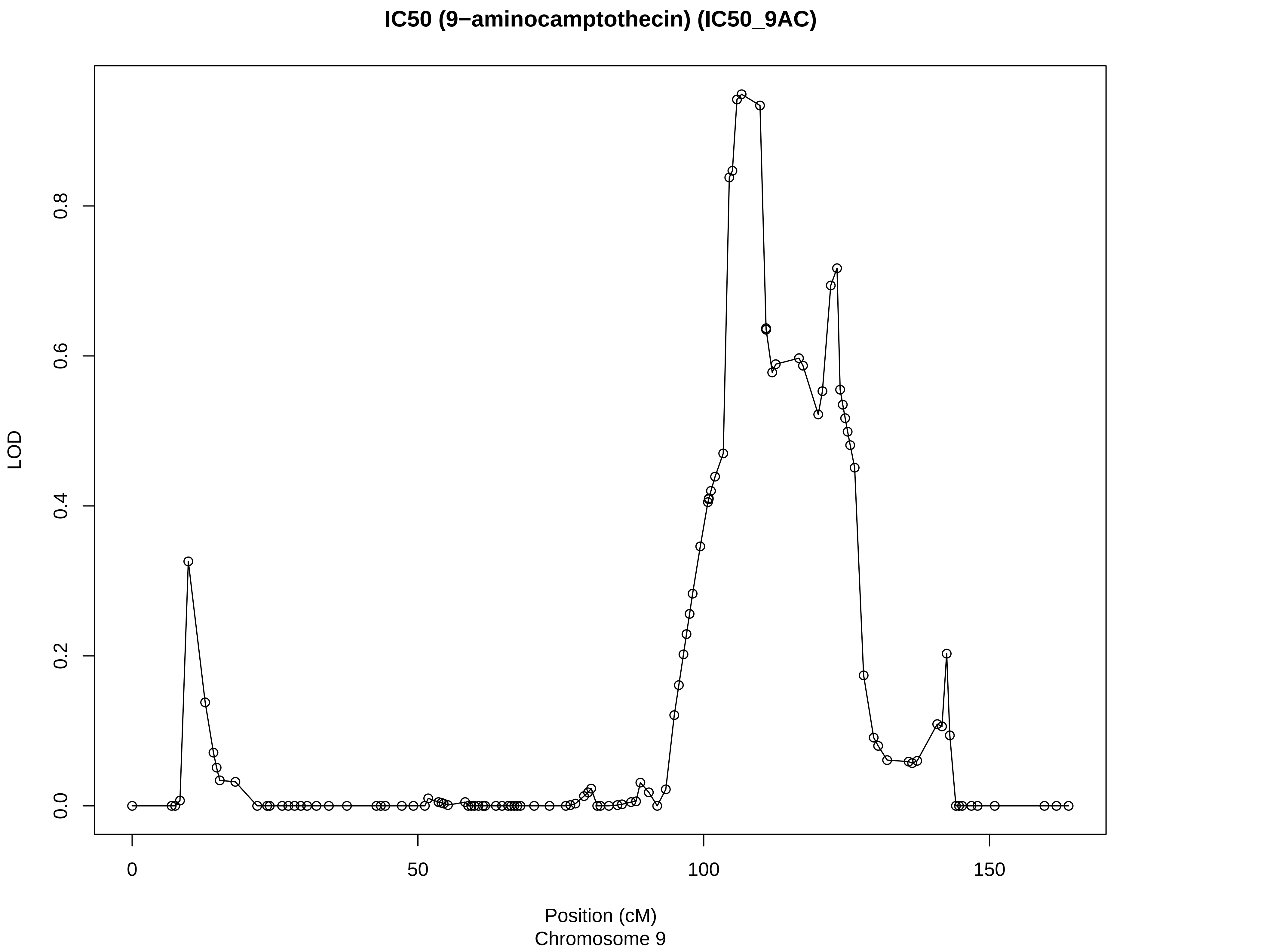
IC50 (9−aminocamptothecin) (IC50_9AC)
LOD
0.0
0.2
0.4
0.6
0.8
0
50
100
150
Position (cM)
Chromosome 9

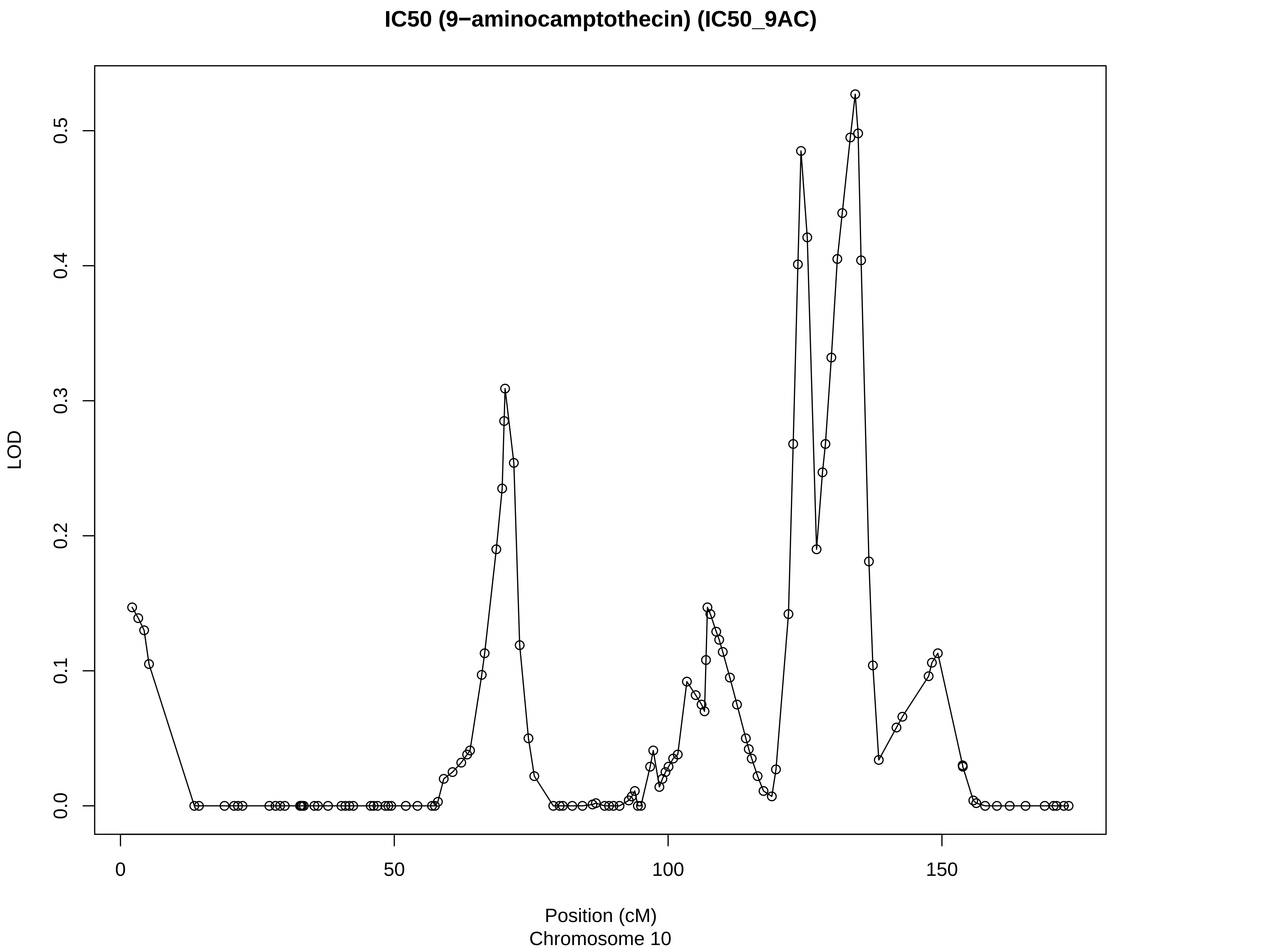
IC50 (9−aminocamptothecin) (IC50_9AC)
LOD
0.0
0.1
0.2
0.3
0.4
0.5
0
50
100
150
Position (cM)
Chromosome 10

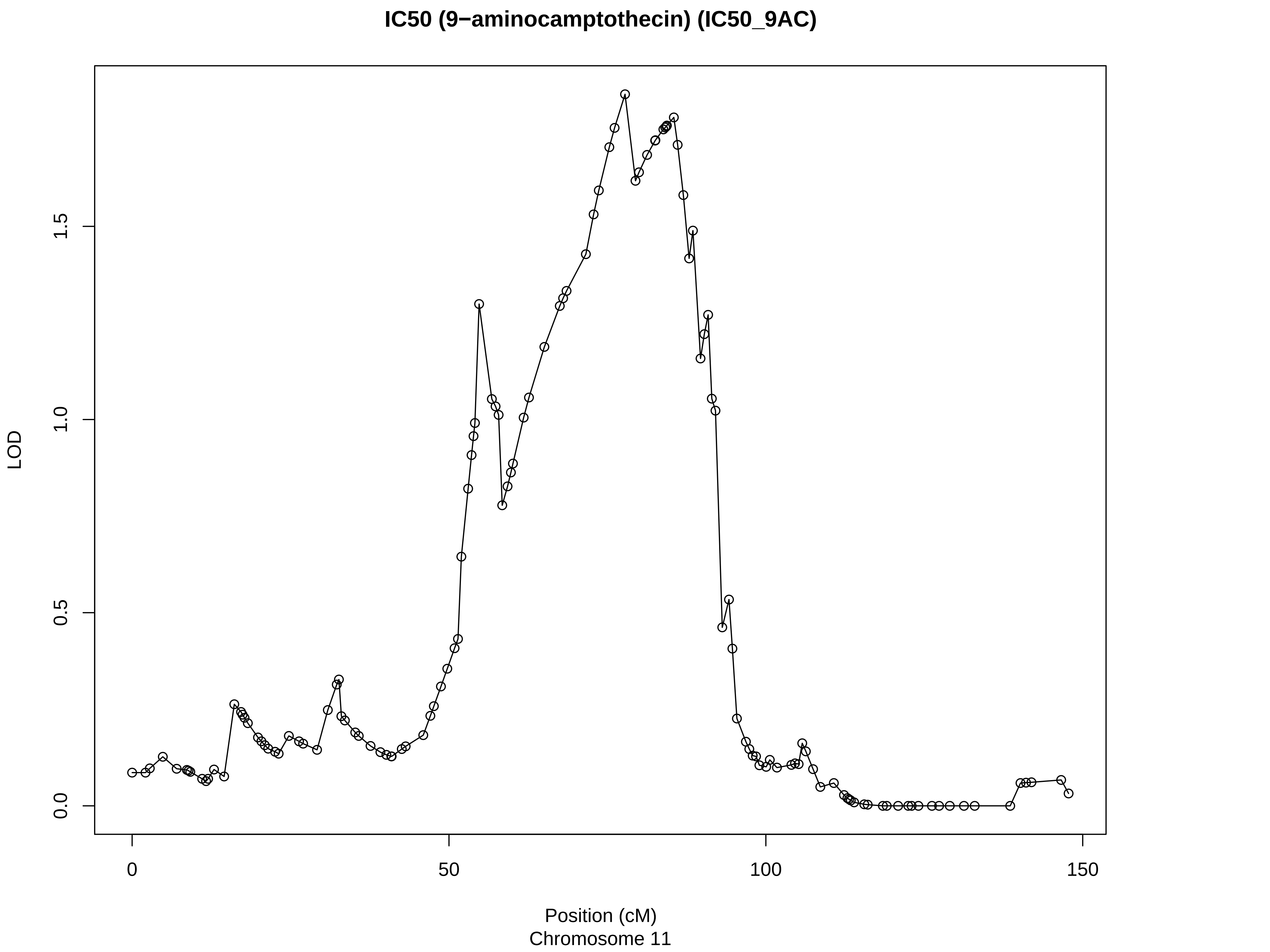
IC50 (9−aminocamptothecin) (IC50_9AC)
LOD
0.0
0.5
1.0
1.5
0
50
100
150
Position (cM)
Chromosome 11

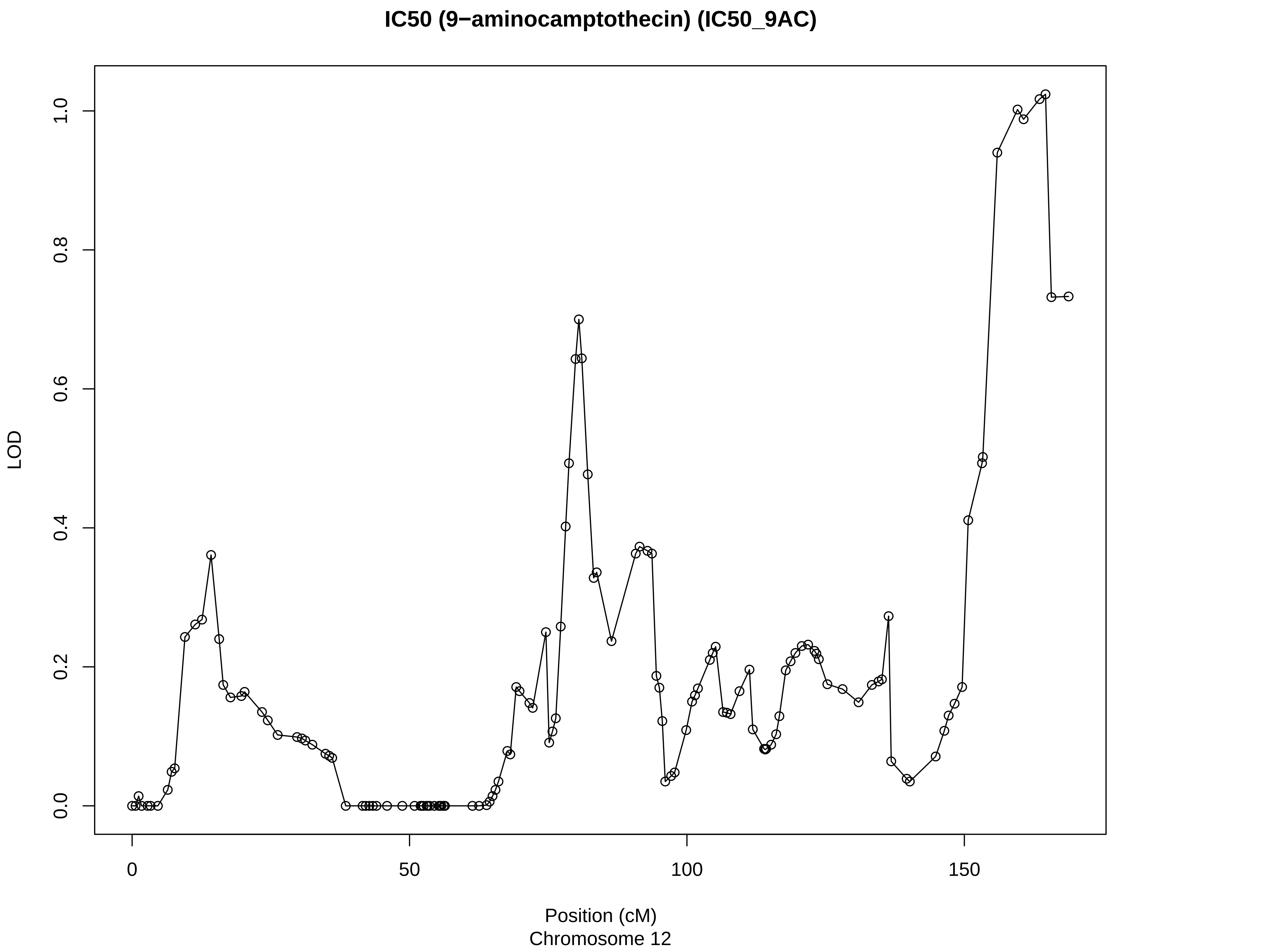
IC50 (9−aminocamptothecin) (IC50_9AC)
LOD
0.0
0.2
0.4
0.6
0.8
1.0
0
50
100
150
Position (cM)
Chromosome 12

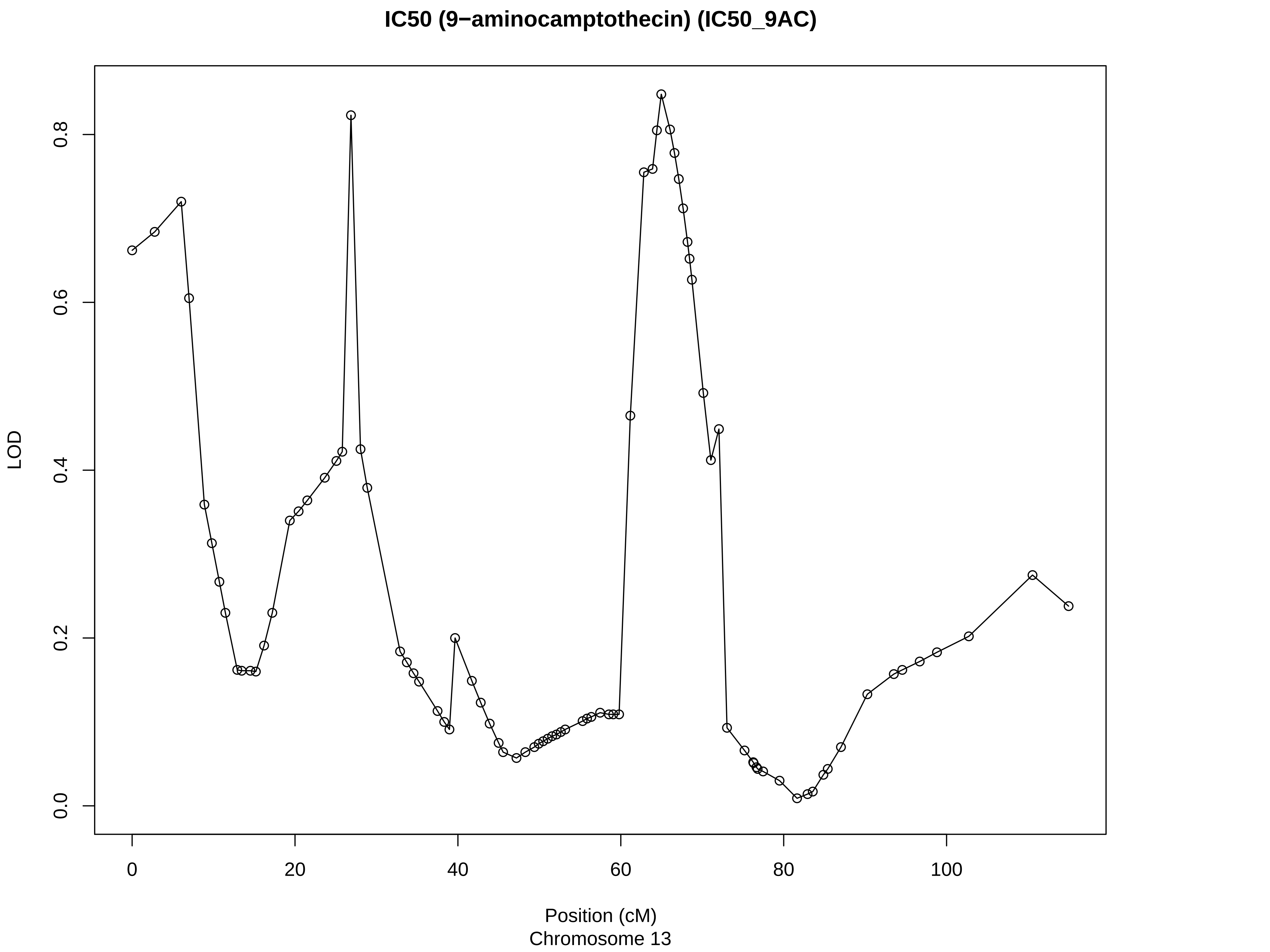

IC50 (9−aminocamptothecin) (IC50_9AC)
LOD
0.0
0.2
0.4
0.6
0.8
0
20
40
60
80
100
Position (cM)
Chromosome 13

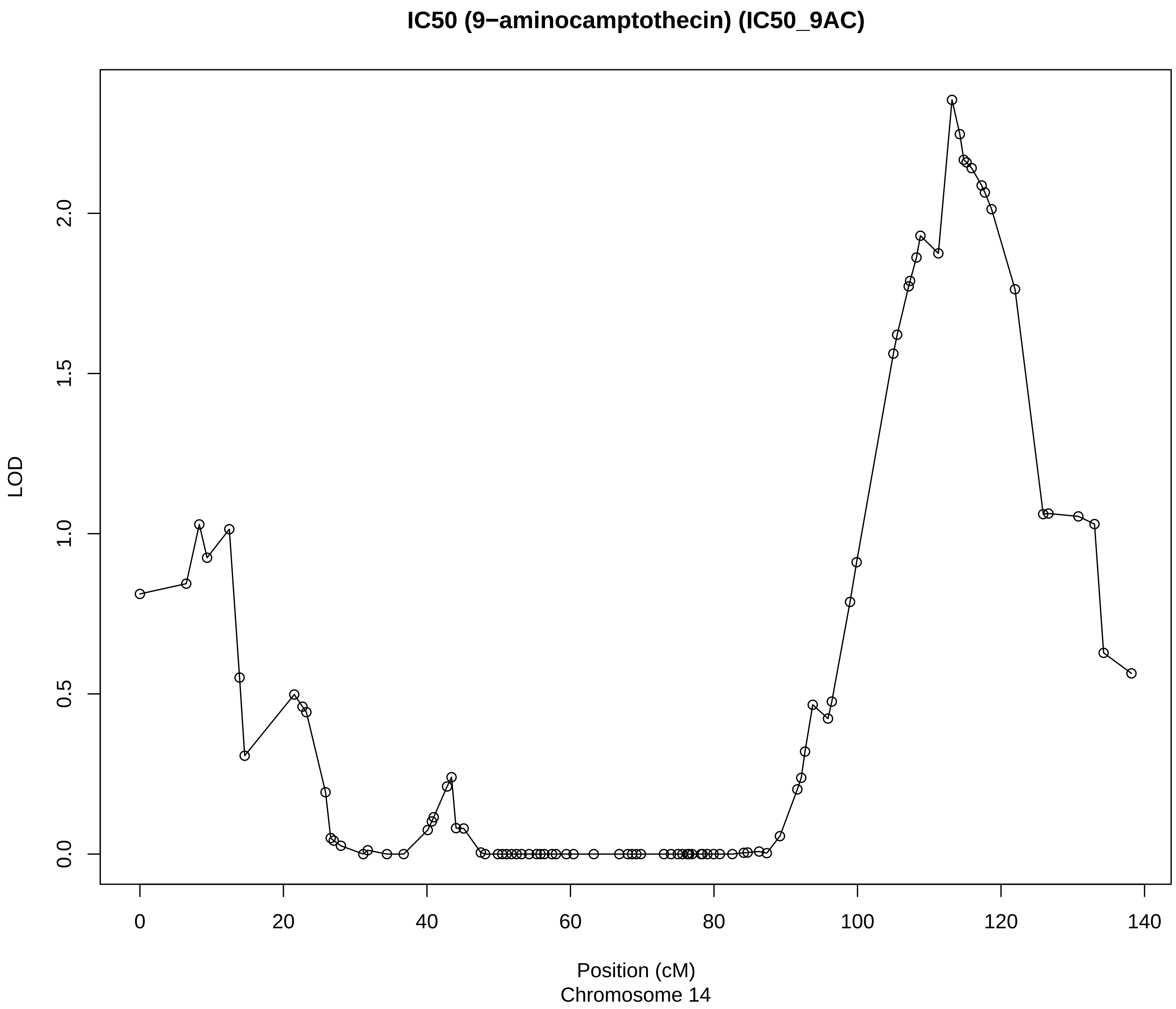
IC50 (9−aminocamptothecin) (IC50_9AC)
LOD
0.0
0.5
1.0
1.5
2.0
0
20
40
60
80
100
120
140
Position (cM)
Chromosome 14

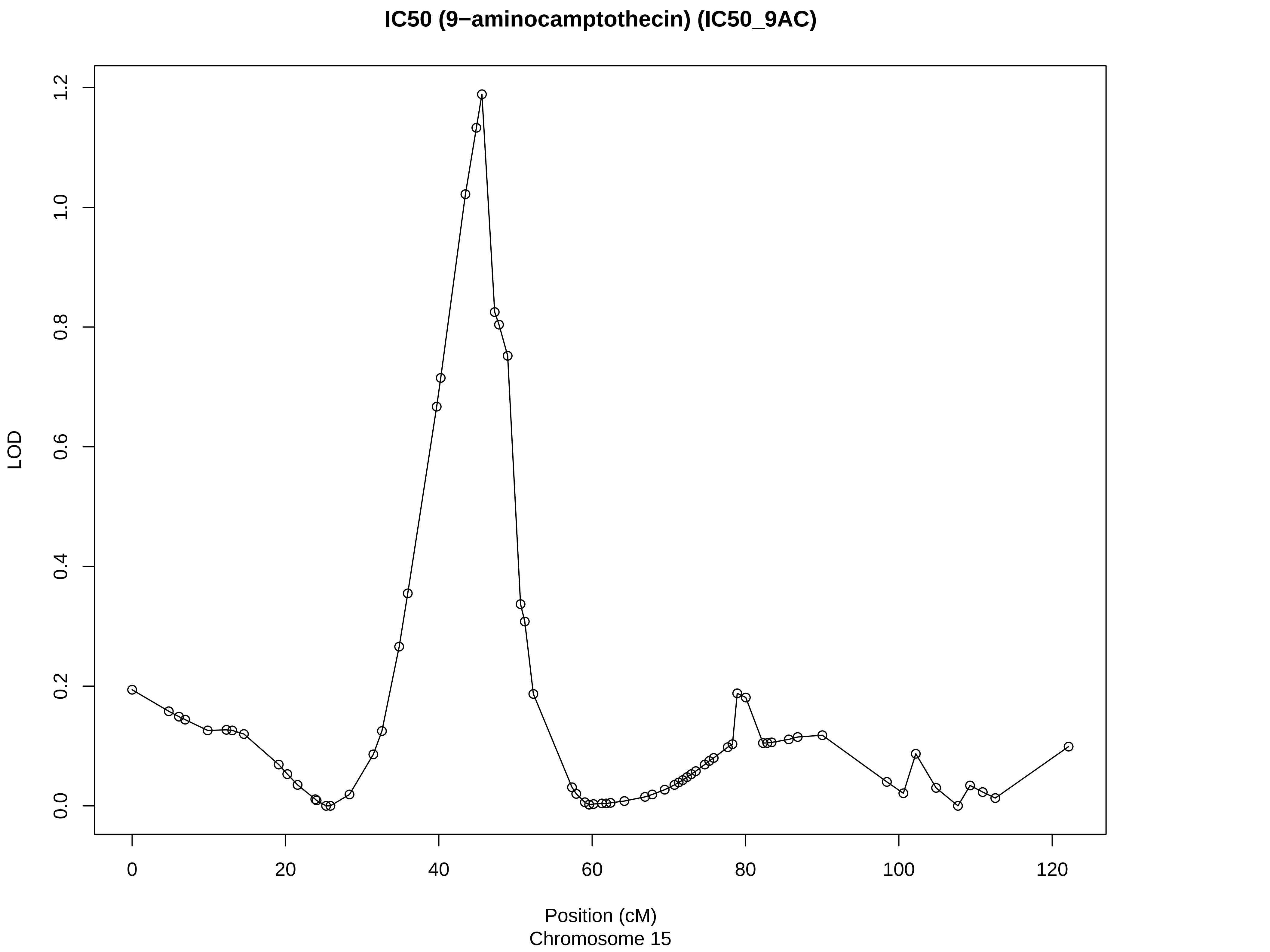
IC50 (9−aminocamptothecin) (IC50_9AC)
LOD
0.0
0.2
0.4
0.6
0.8
1.0
1.2
0
20
40
60
80
100
120
Position (cM)
Chromosome 15

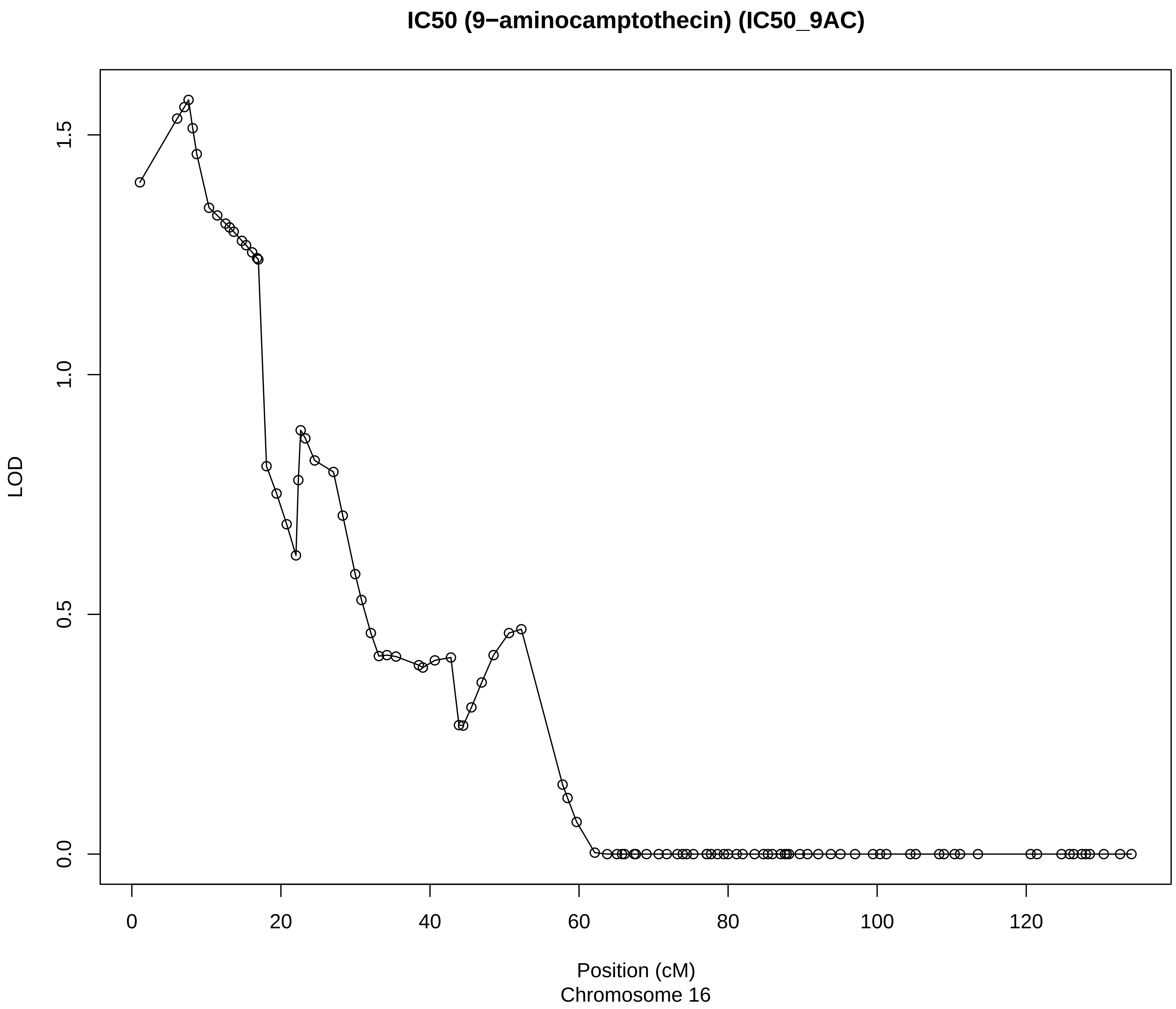
IC50 (9−aminocamptothecin) (IC50_9AC)
LOD
0.0
0.5
1.0
1.5
0
20
40
60
80
100
120
Position (cM)
Chromosome 16

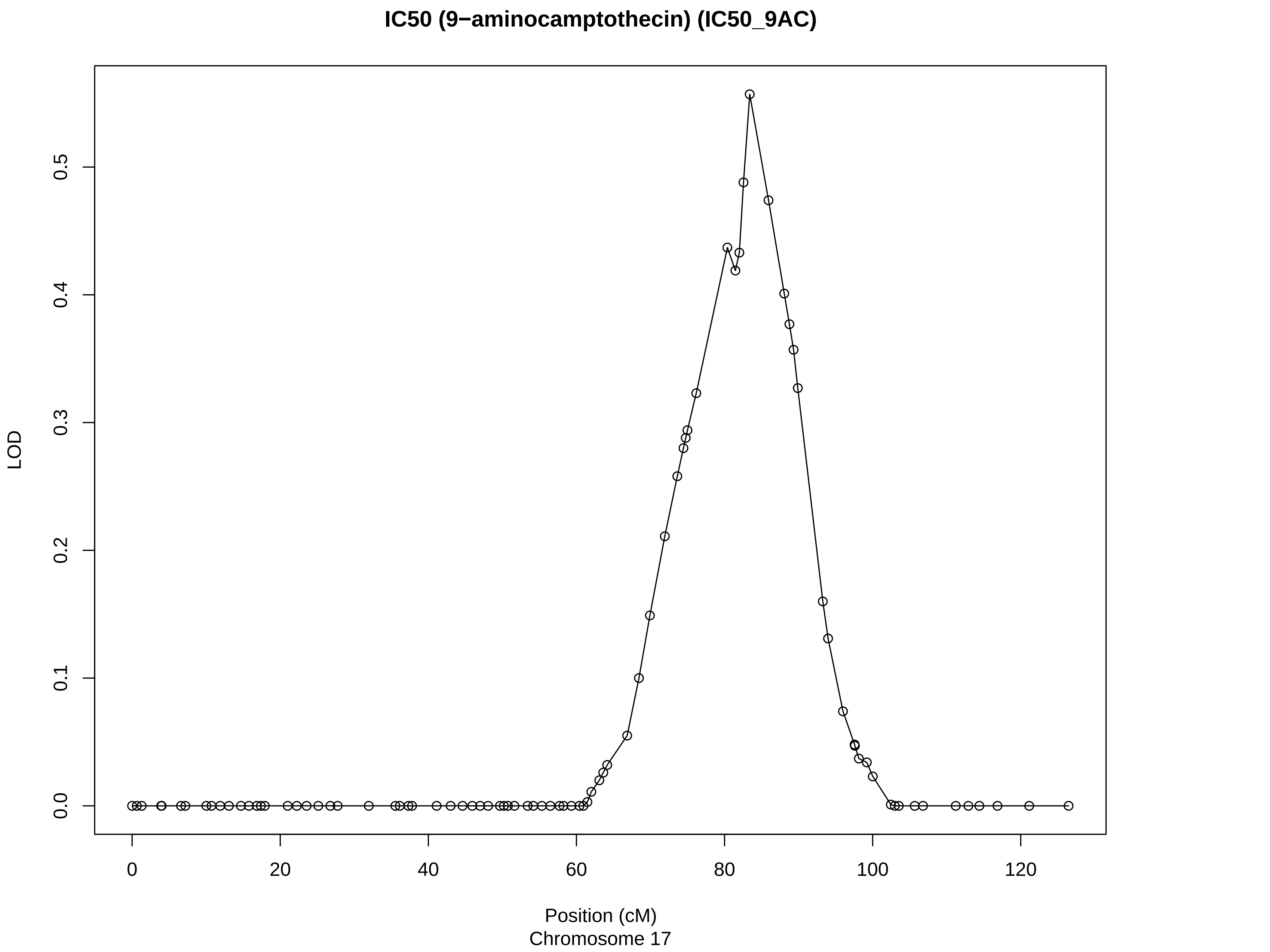
IC50 (9−aminocamptothecin) (IC50_9AC)
LOD
0.0
0.1
0.2
0.3
0.4
0.5
0
20
40
60
80
100
120
Position (cM)
Chromosome 17

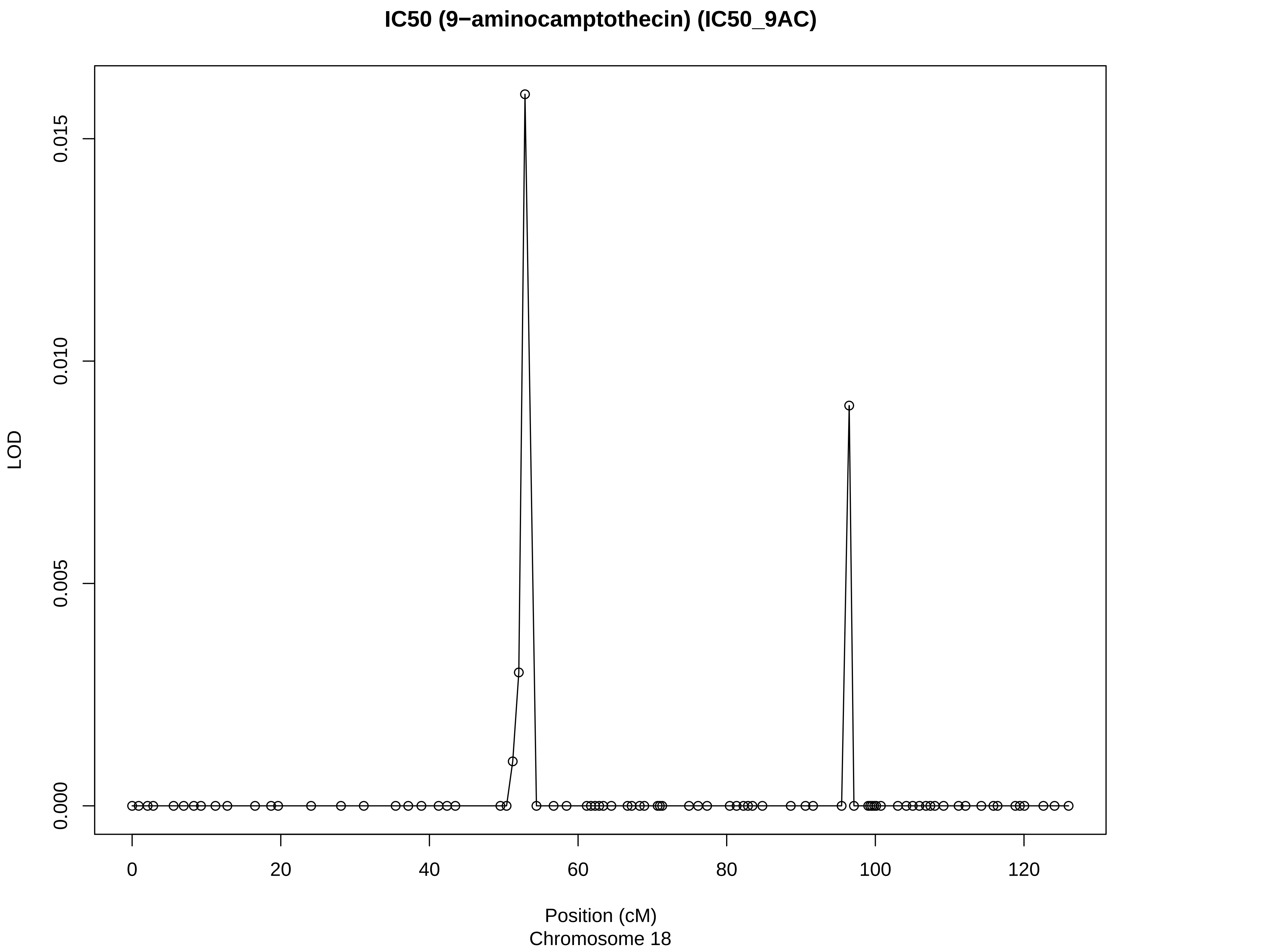
IC50 (9−aminocamptothecin) (IC50_9AC)
LOD
0.000
0.005
0.010
0.015
0
20
40
60
80
100
120
Position (cM)
Chromosome 18

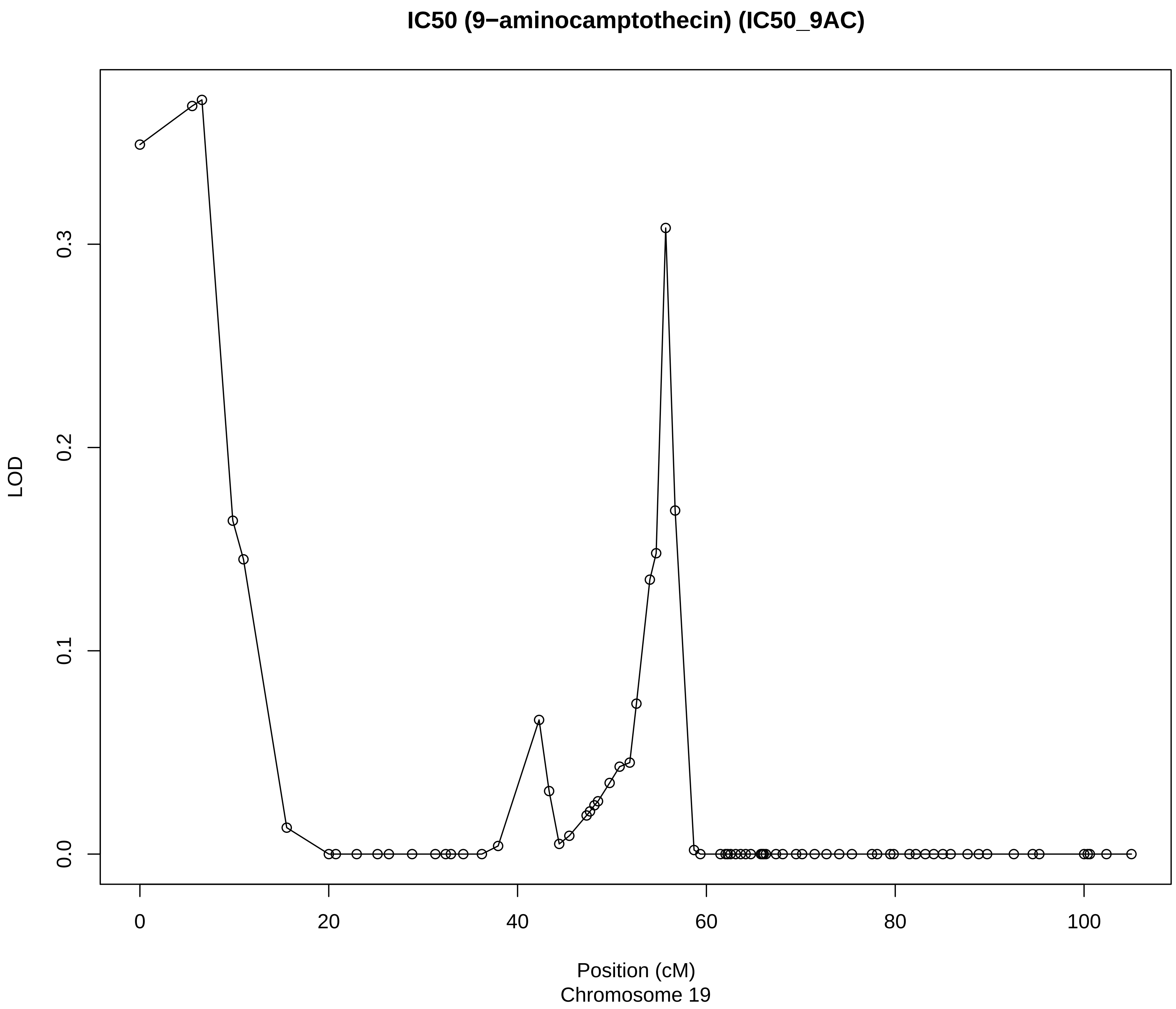
IC50 (9−aminocamptothecin) (IC50_9AC)
LOD
0.0
0.1
0.2
0.3
0
20
40
60
80
100
Position (cM)
Chromosome 19

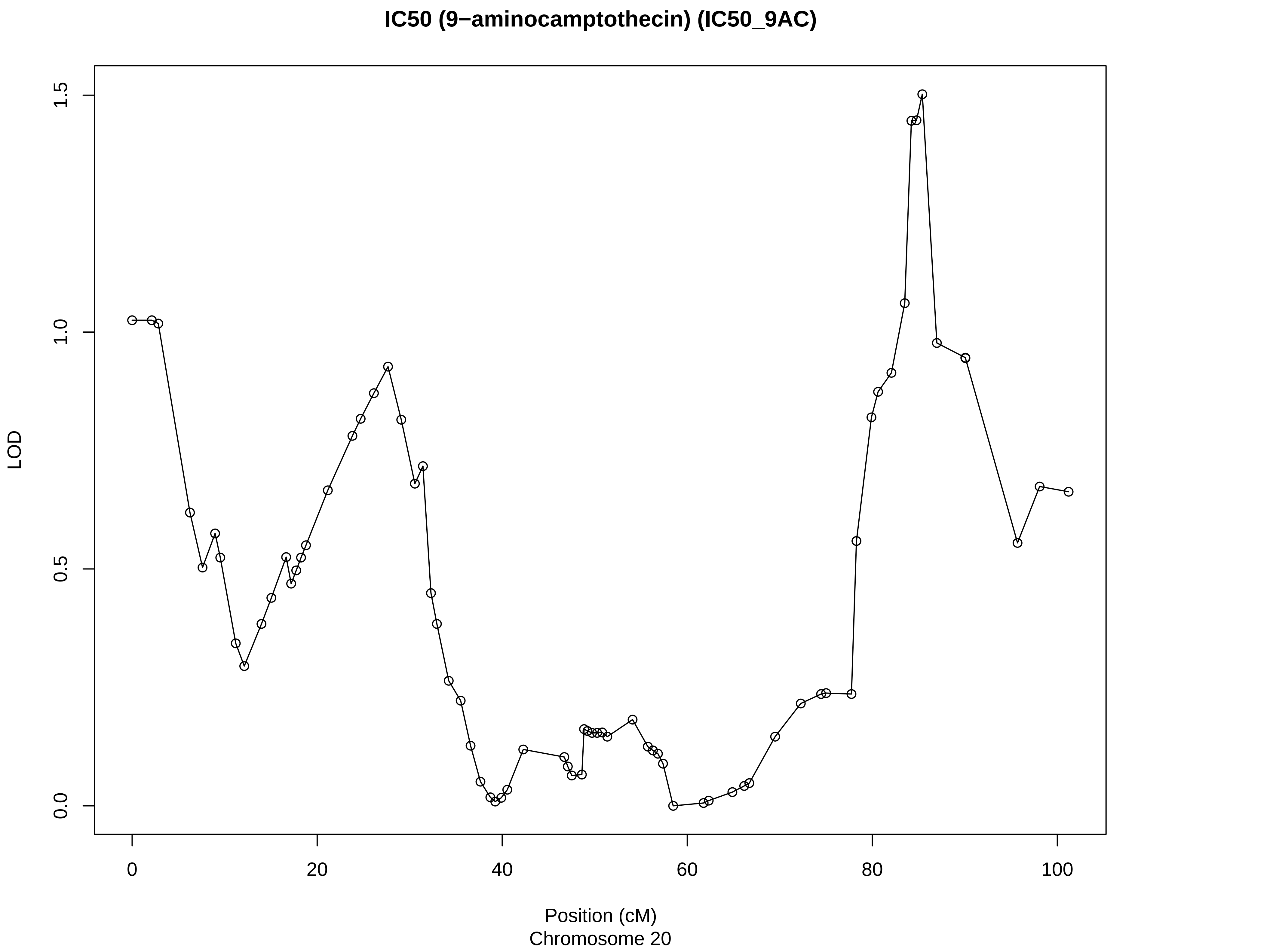
IC50 (9−aminocamptothecin) (IC50_9AC)
LOD
1.5
1.0
0.5
0.0
0
20
40
60
80
100
Position (cM)
Chromosome 20

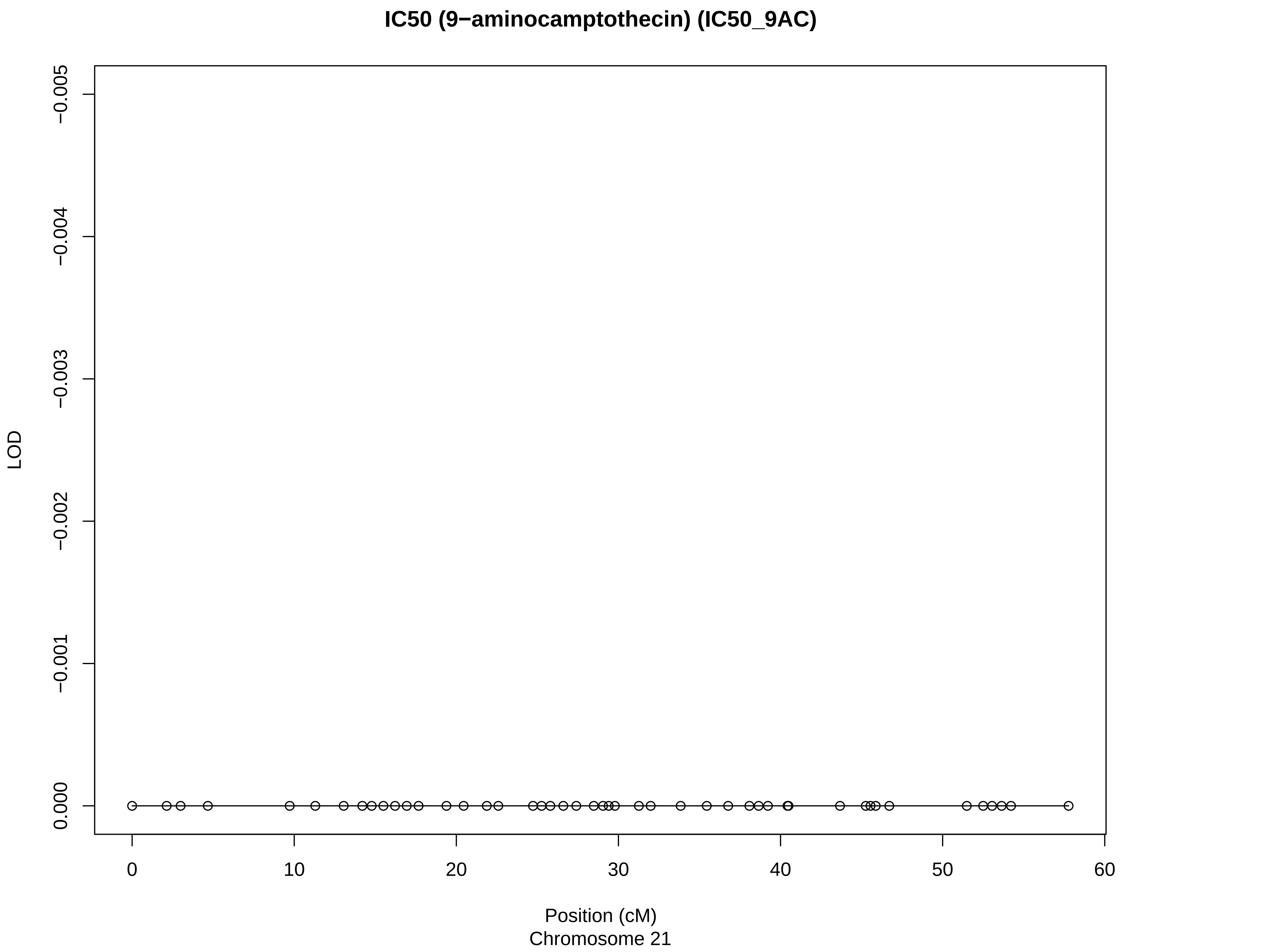
IC50 (9−aminocamptothecin) (IC50_9AC)
LOD
−0.005
−0.004
−0.003
−0.002
−0.001
0.000
0
10
20
30
40
50
60
Position (cM)
Chromosome 21

IC50 (9−aminocamptothecin) (IC50_9AC)

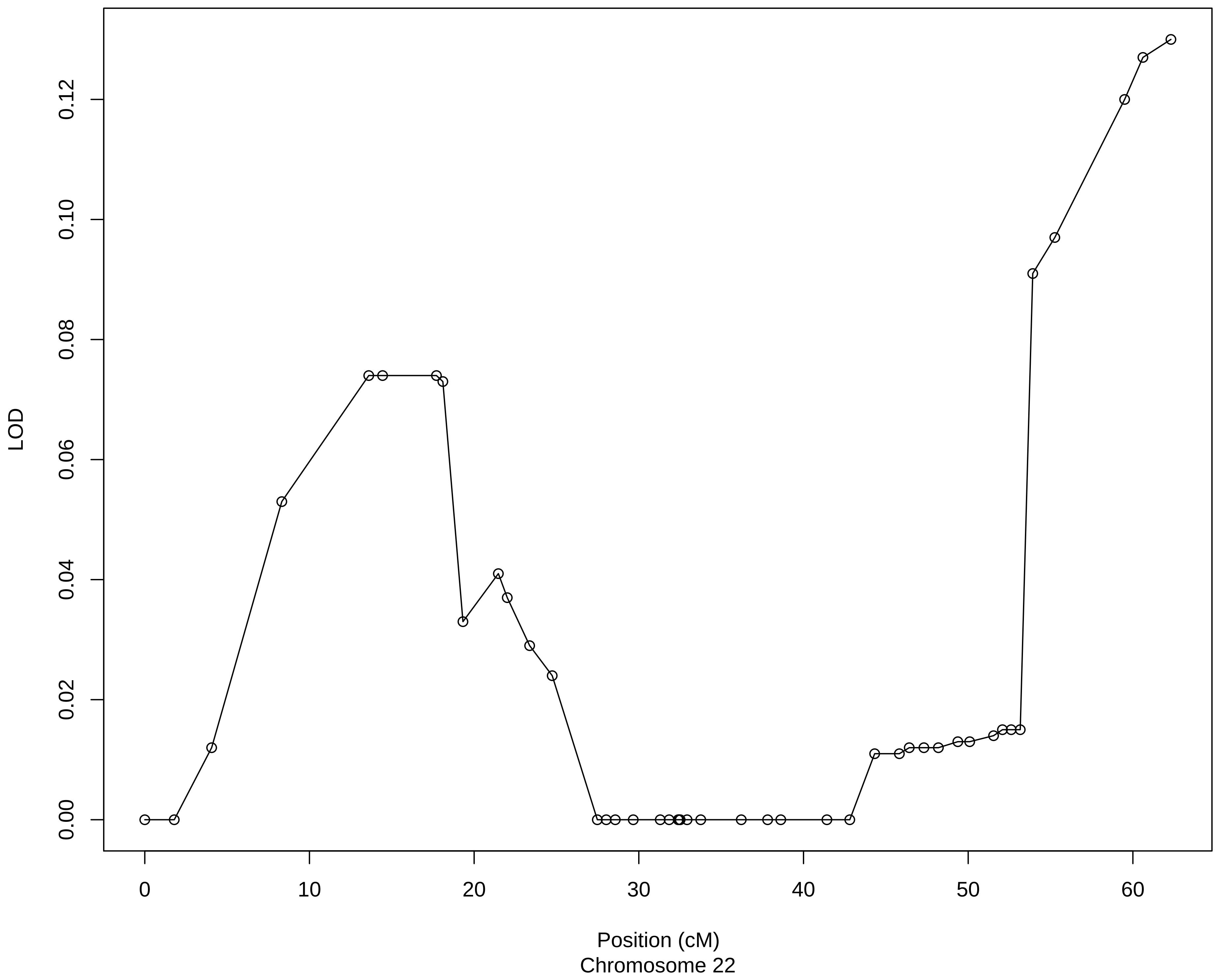

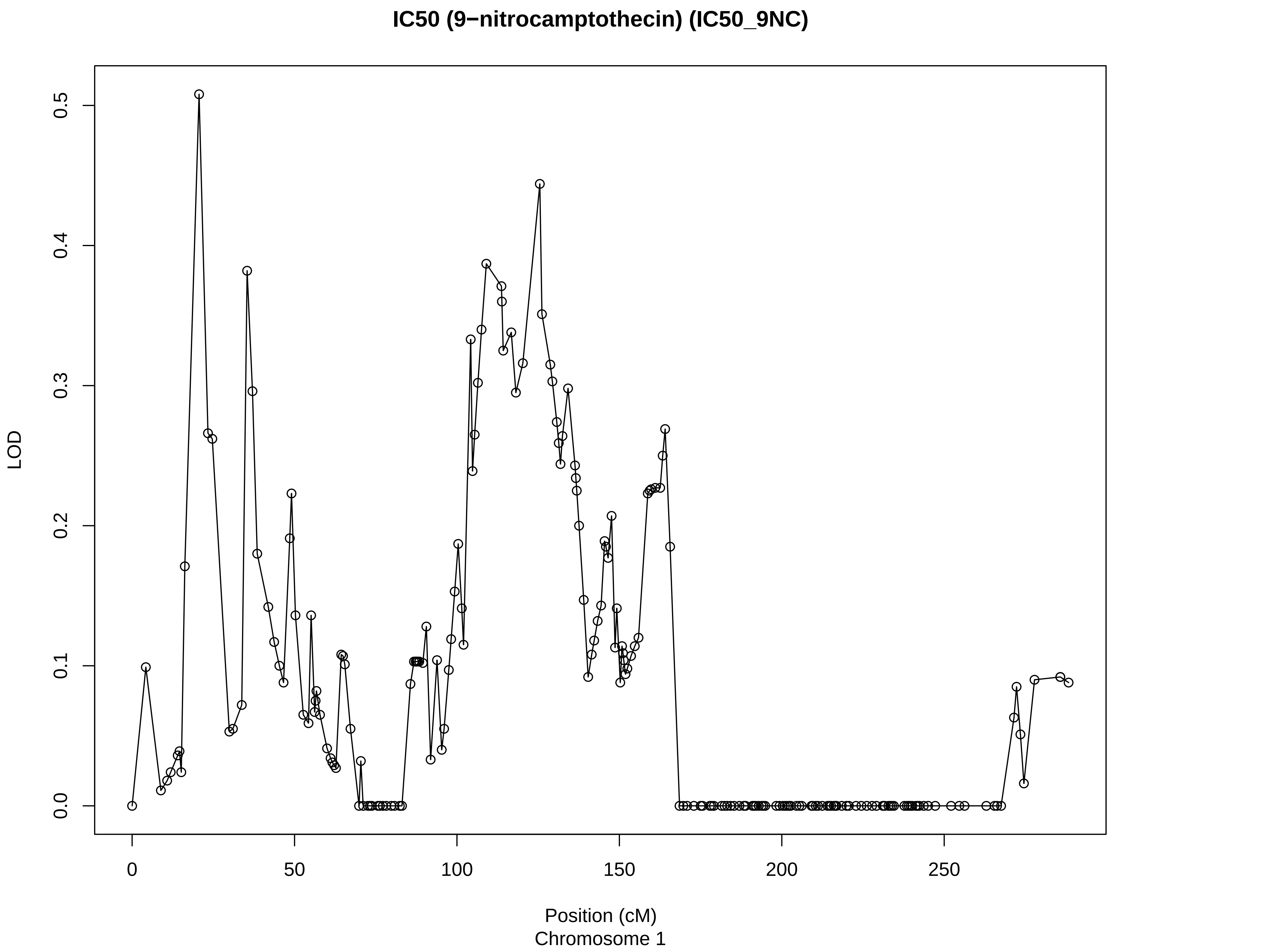

IC50 (9−nitrocamptothecin) (IC50_9NC)
LOD
0.0
0.1
0.2
0.3
0.4
0.5
0
50
100
150
200
250
Position (cM)
Chromosome 1

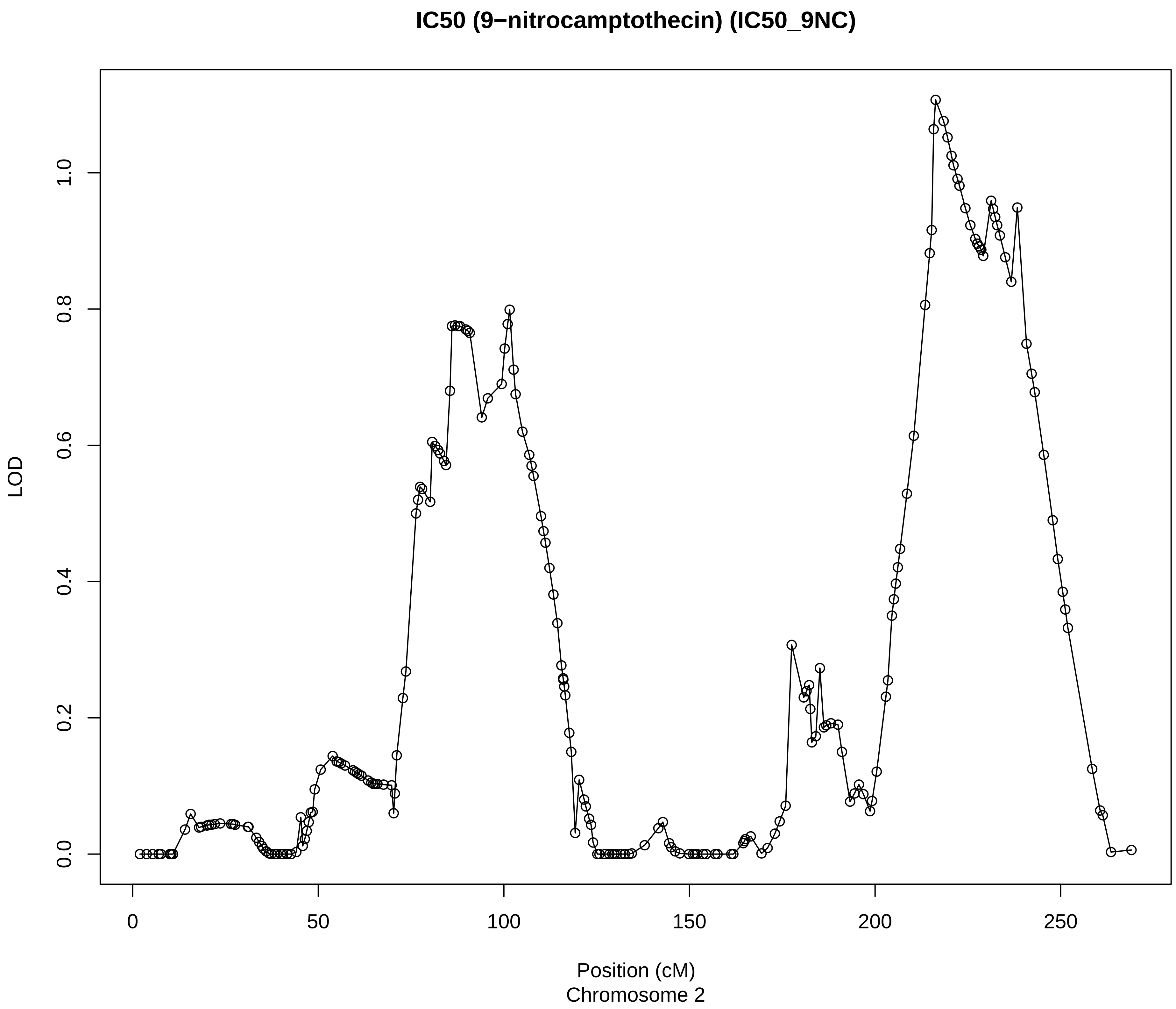

IC50 (9−nitrocamptothecin) (IC50_9NC)
LOD
0.0
0.2
0.4
0.6
0.8
1.0
0
50
100
150
200
250
Position (cM)
Chromosome 2

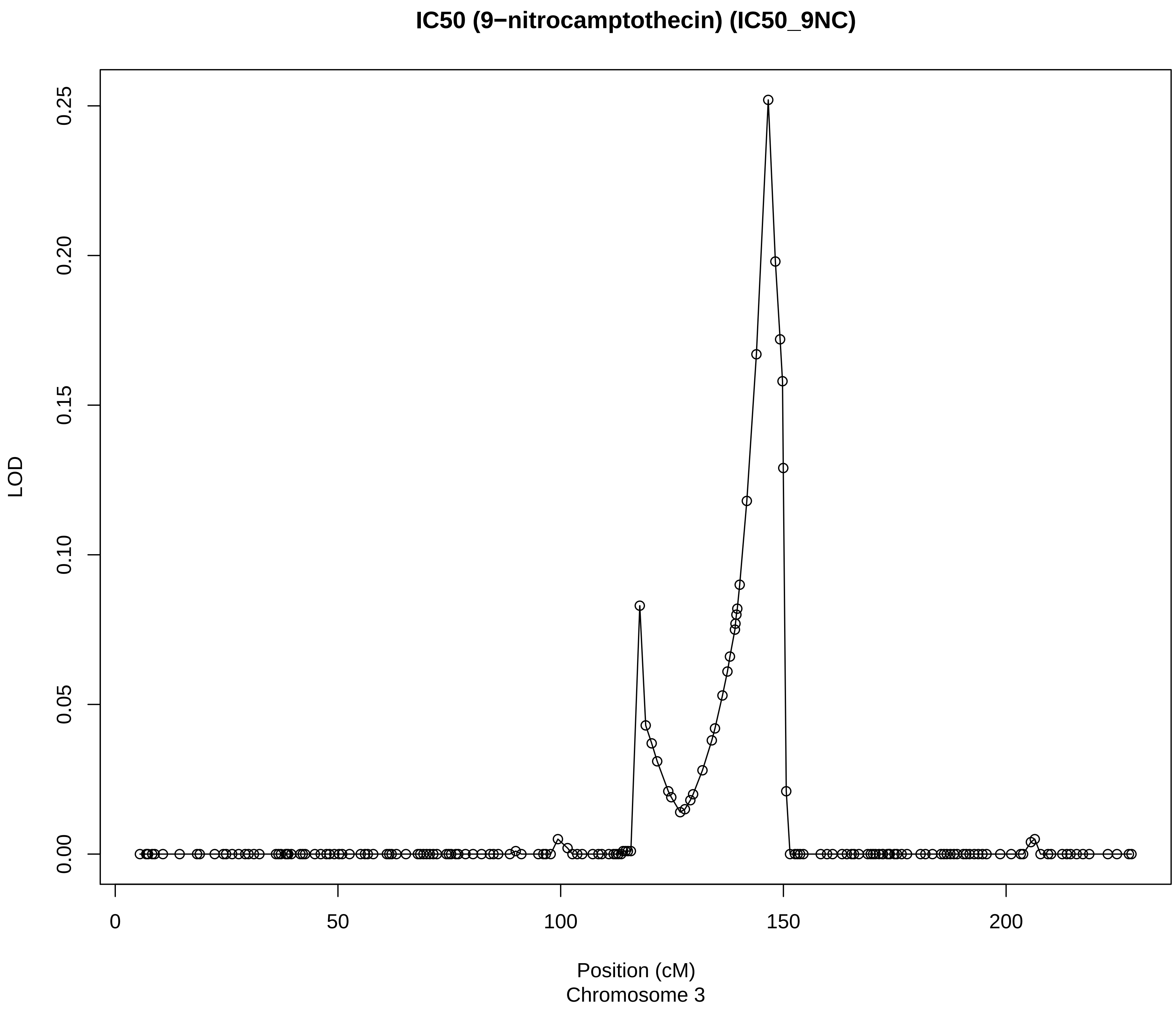
IC50 (9−nitrocamptothecin) (IC50_9NC)
LOD
0.00
0.05
0.10
0.15
0.20
0.25
0
50
100
150
200
Position (cM)
Chromosome 3

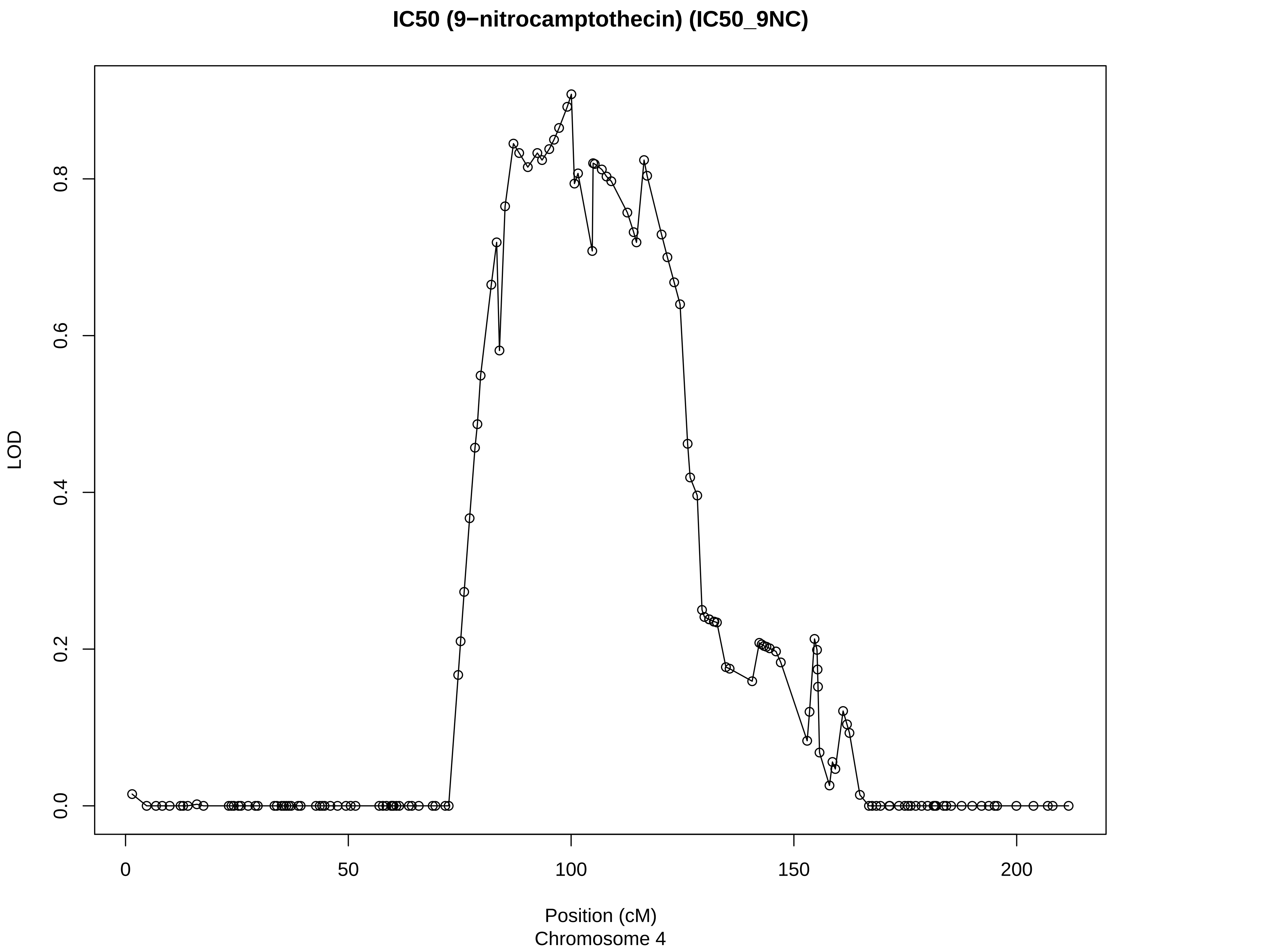
IC50 (9−nitrocamptothecin) (IC50_9NC)
LOD
0.0
0.2
0.4
0.6
0.8
0
50
100
150
200
Position (cM)
Chromosome 4

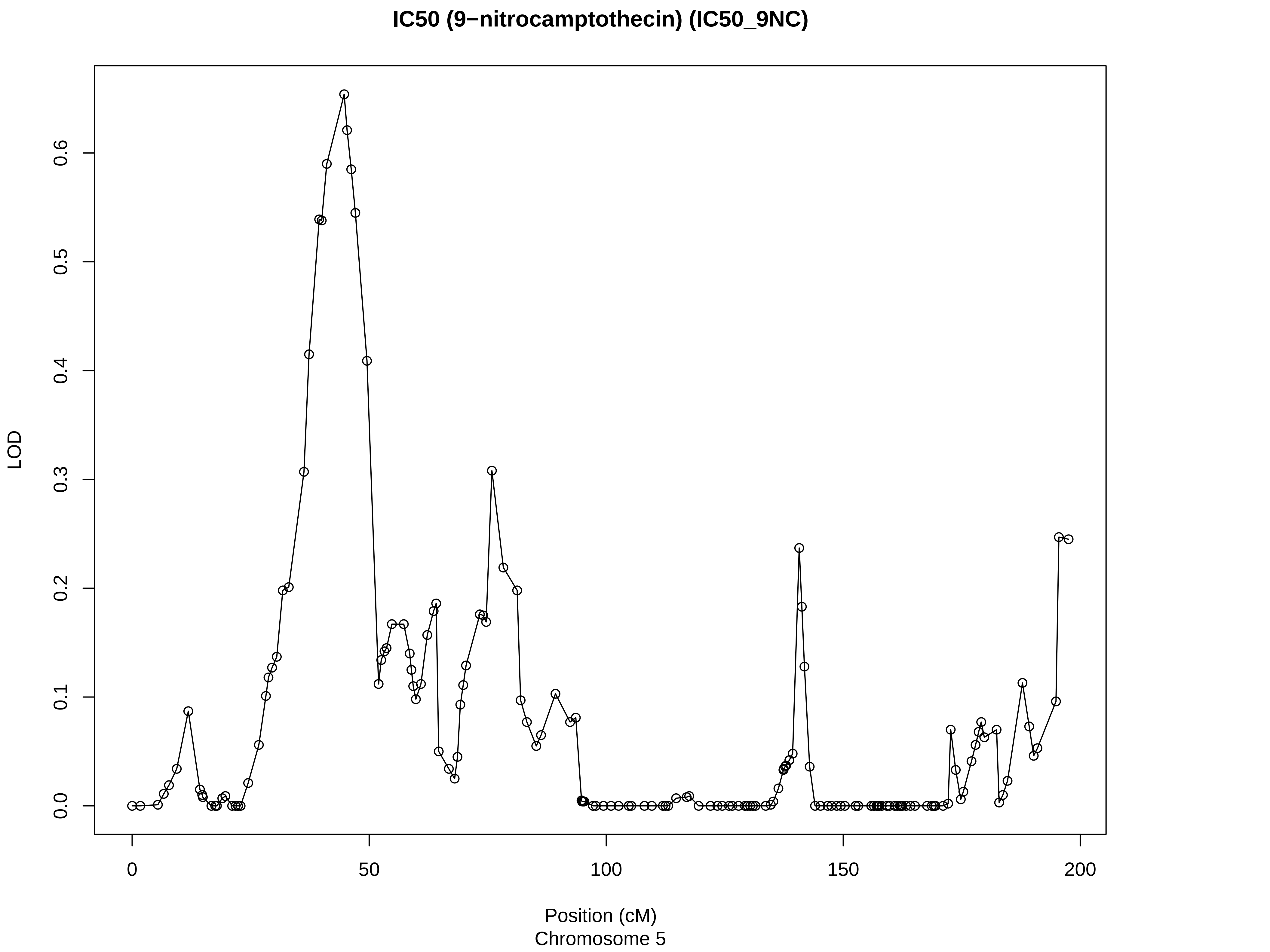
IC50 (9−nitrocamptothecin) (IC50_9NC)
LOD
0.0
0.1
0.2
0.3
0.4
0.5
0.6
0
50
100
150
200
Position (cM)
Chromosome 5

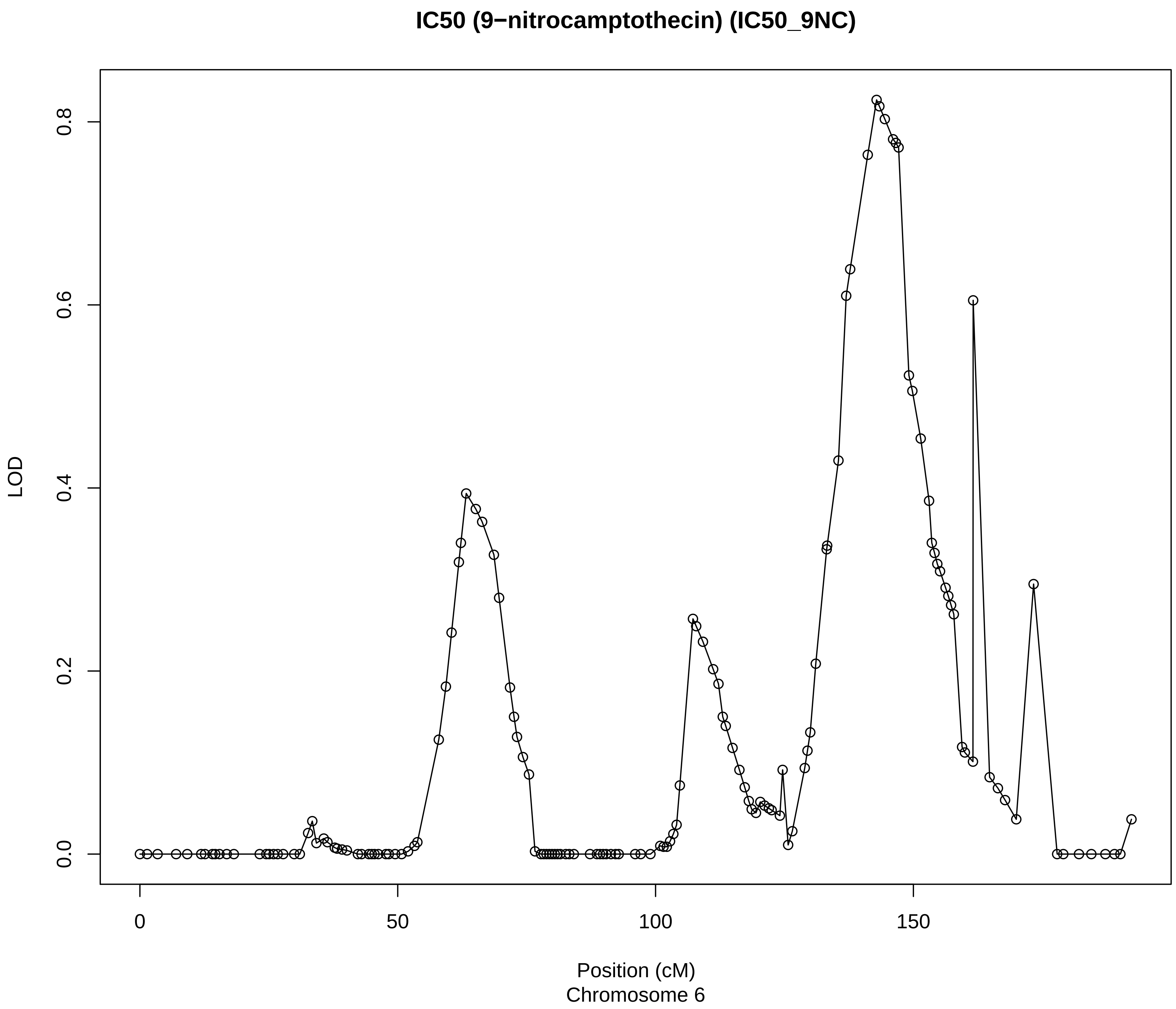

IC50 (9−nitrocamptothecin) (IC50_9NC)
LOD
0.0
0.2
0.4
0.6
0.8
0
50
100
150
Position (cM)
Chromosome 6

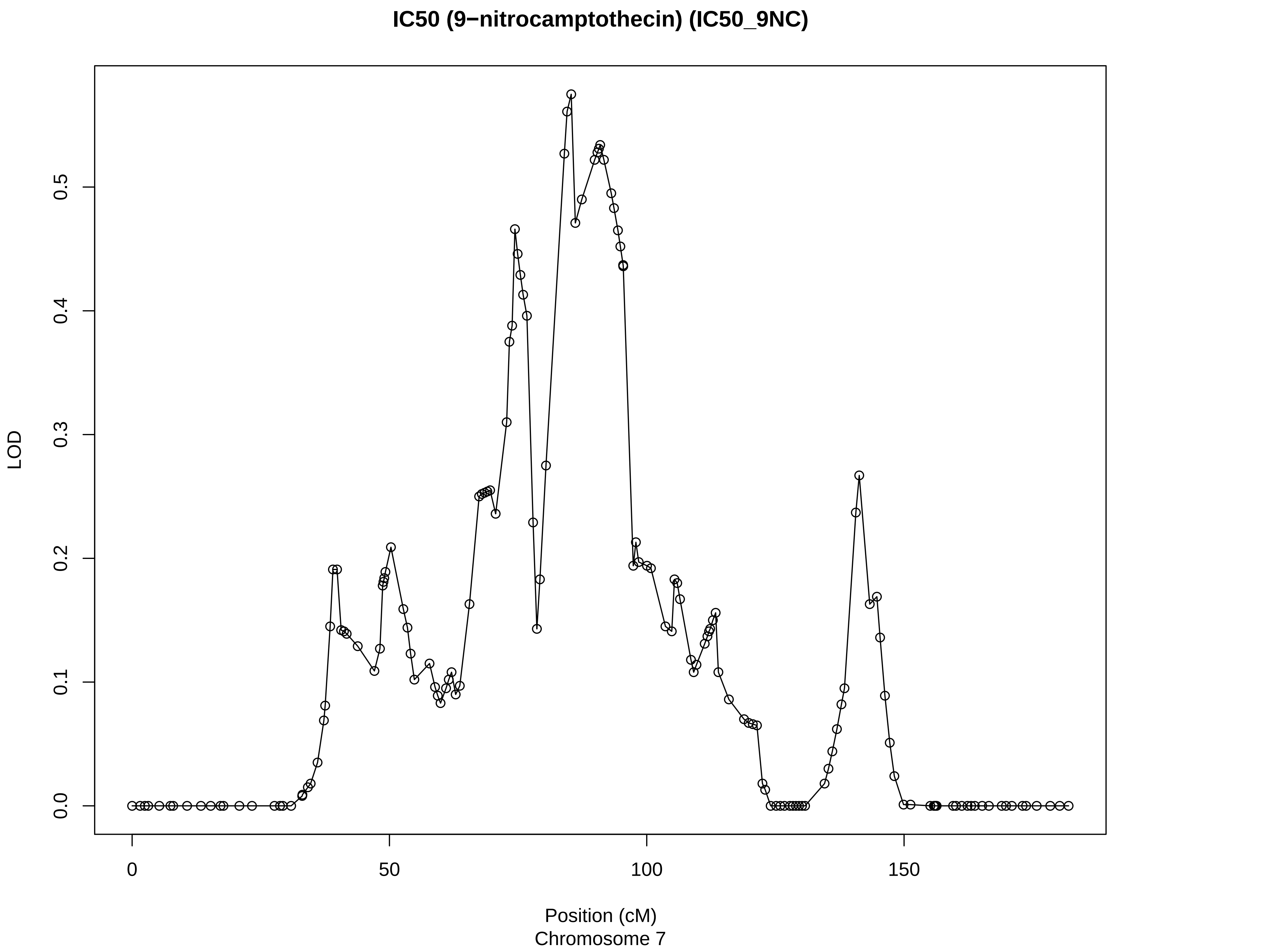
IC50 (9−nitrocamptothecin) (IC50_9NC)
LOD
0.0
0.1
0.2
0.3
0.4
0.5
0
50
100
150
Position (cM)
Chromosome 7

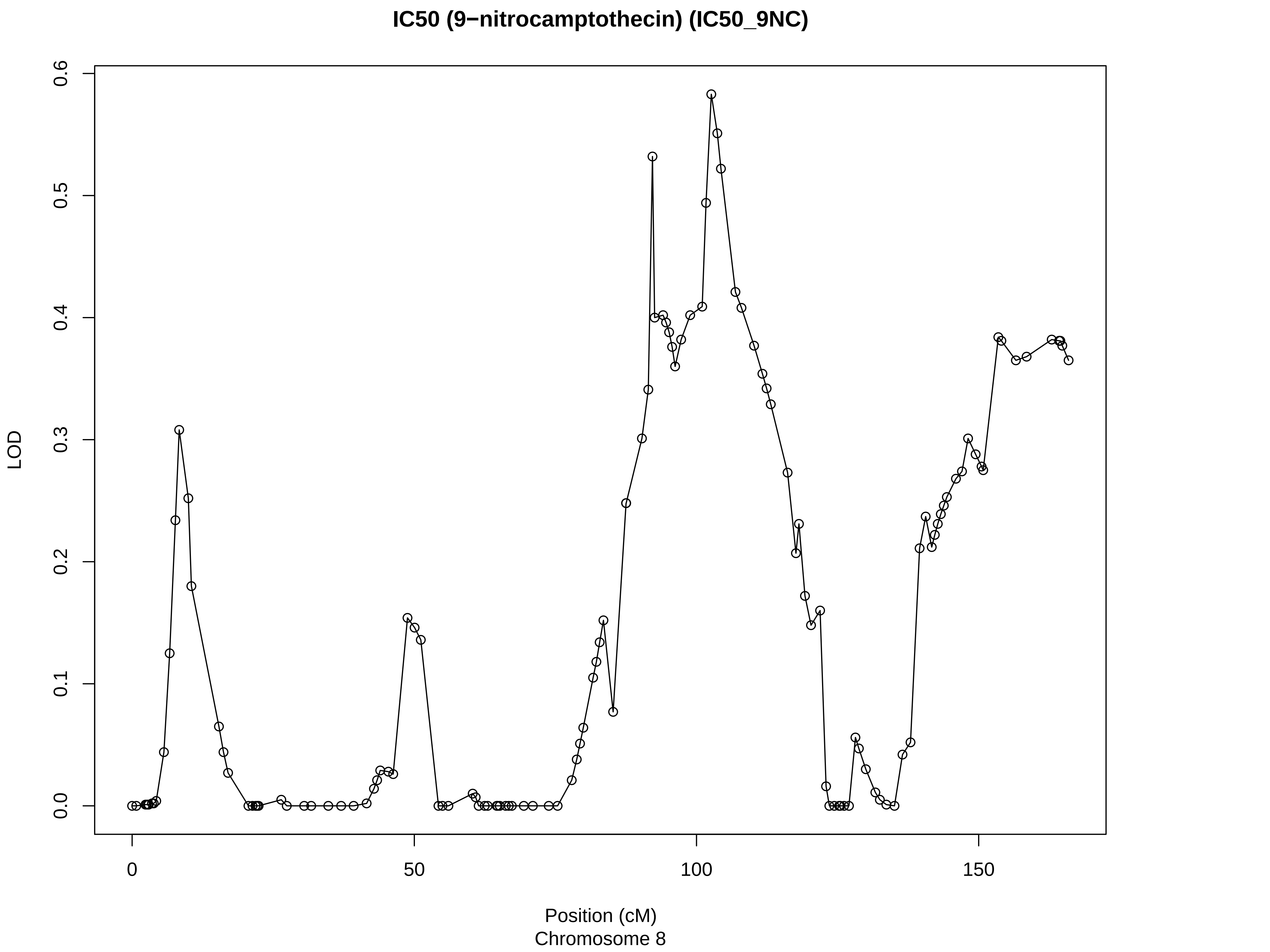
IC50 (9−nitrocamptothecin) (IC50_9NC)
LOD
0.0
0.1
0.2
0.3
0.4
0.5
0.6
0
50
100
150
Position (cM)
Chromosome 8

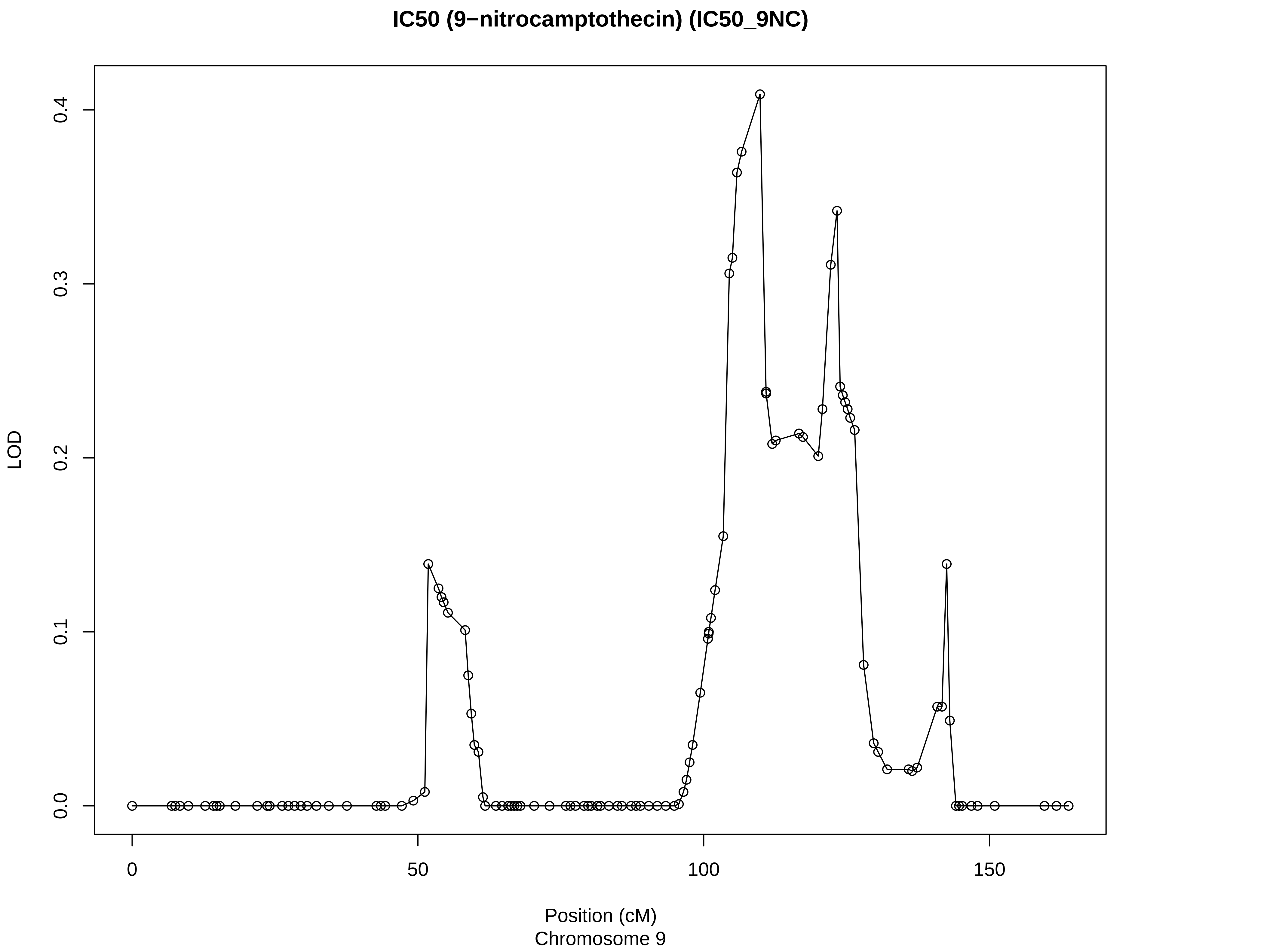

IC50 (9−nitrocamptothecin) (IC50_9NC)
LOD
0.0
0.1
0.2
0.3
0.4
0
50
100
150
Position (cM)
Chromosome 9

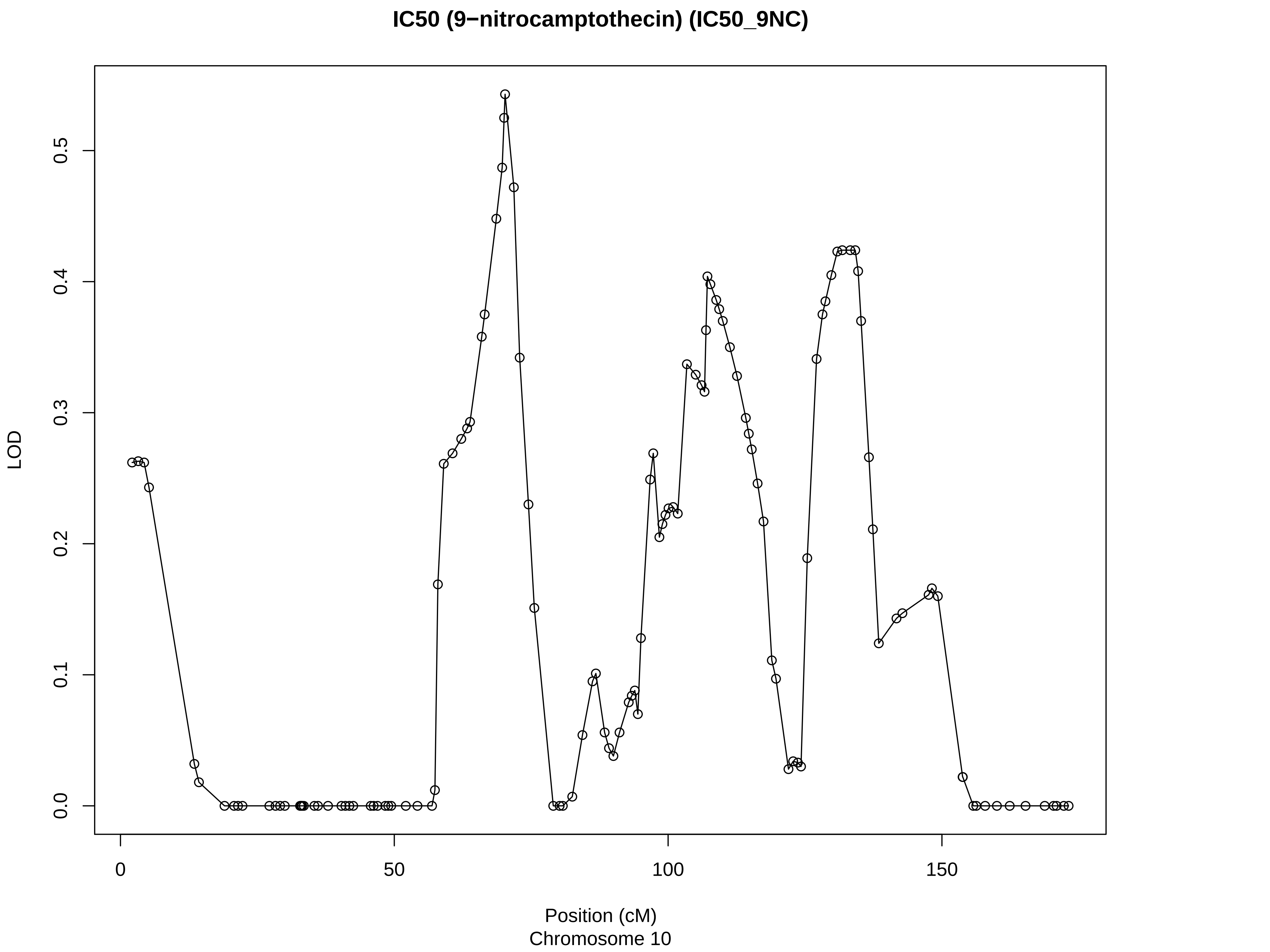

IC50 (9−nitrocamptothecin) (IC50_9NC)
LOD
0.0
0.1
0.2
0.3
0.4
0.5
0
50
100
150
Position (cM)
Chromosome 10

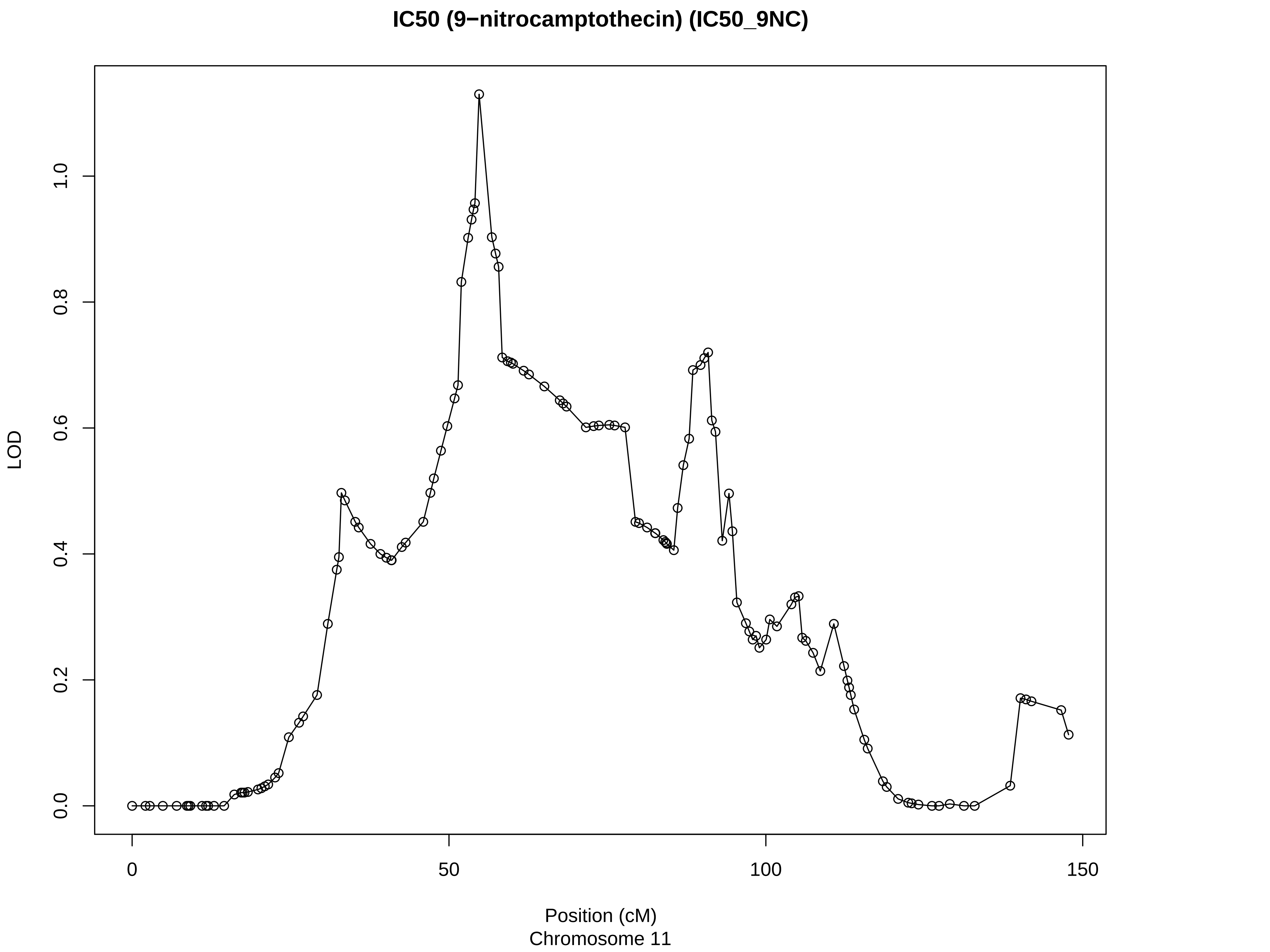
IC50 (9−nitrocamptothecin) (IC50_9NC)
LOD
0.0
0.2
0.4
0.6
0.8
1.0
0
50
100
150
Position (cM)
Chromosome 11

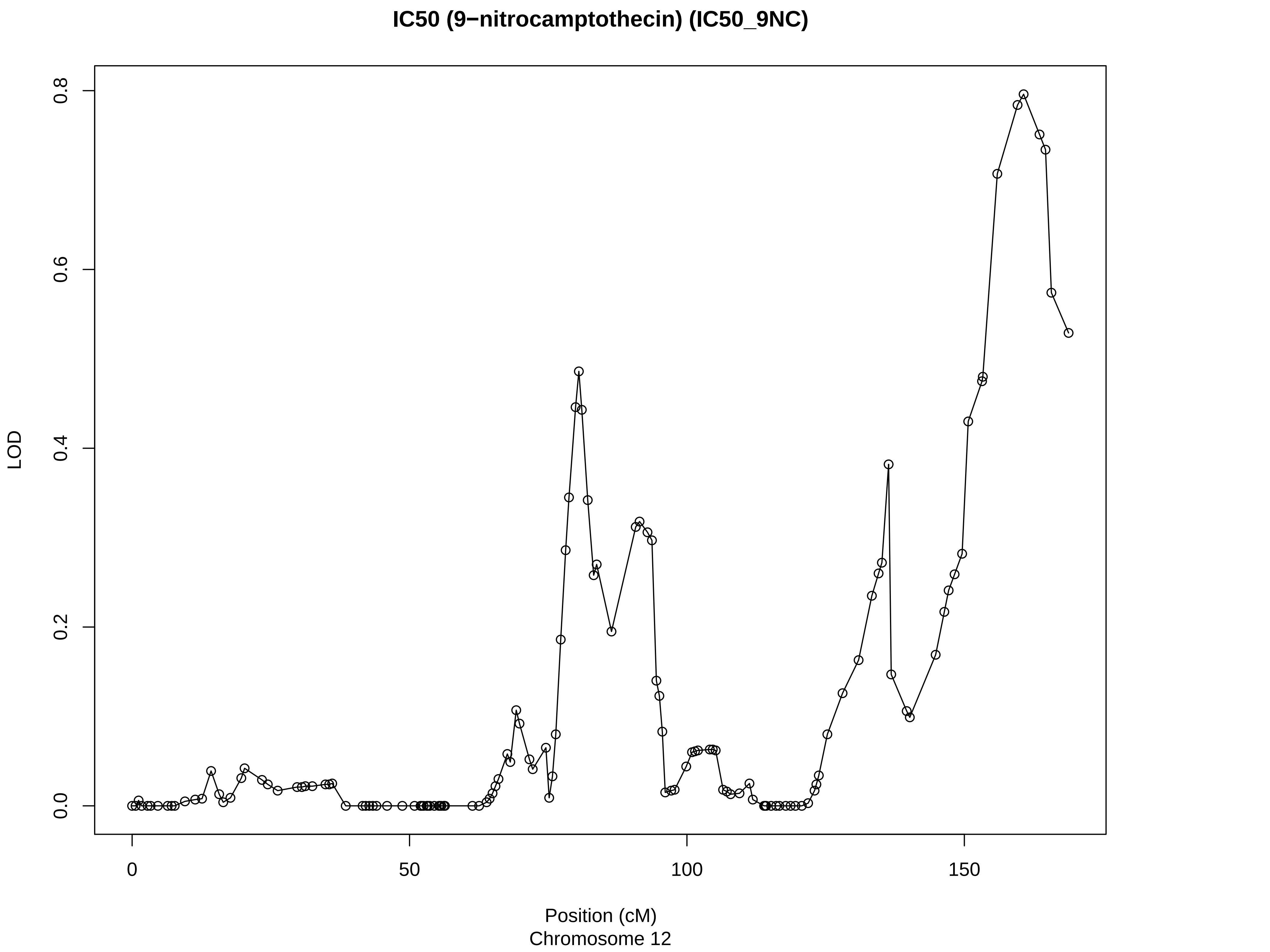
IC50 (9−nitrocamptothecin) (IC50_9NC)
LOD
0.0
0.2
0.4
0.6
0.8
0
50
100
150
Position (cM)
Chromosome 12

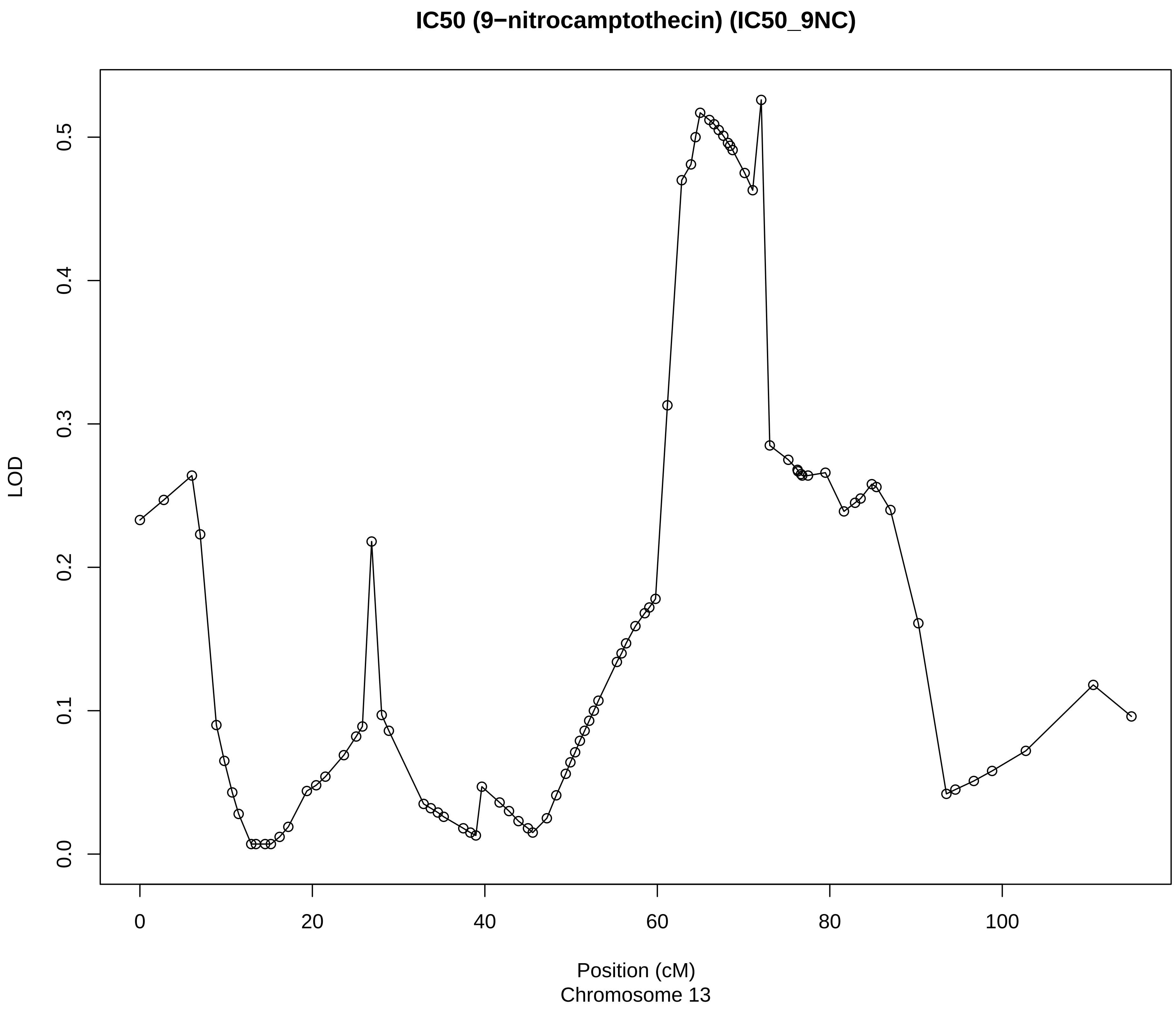
IC50 (9−nitrocamptothecin) (IC50_9NC)
LOD
0.0
0.1
0.2
0.3
0.4
0.5
0
20
40
60
80
100
Position (cM)
Chromosome 13

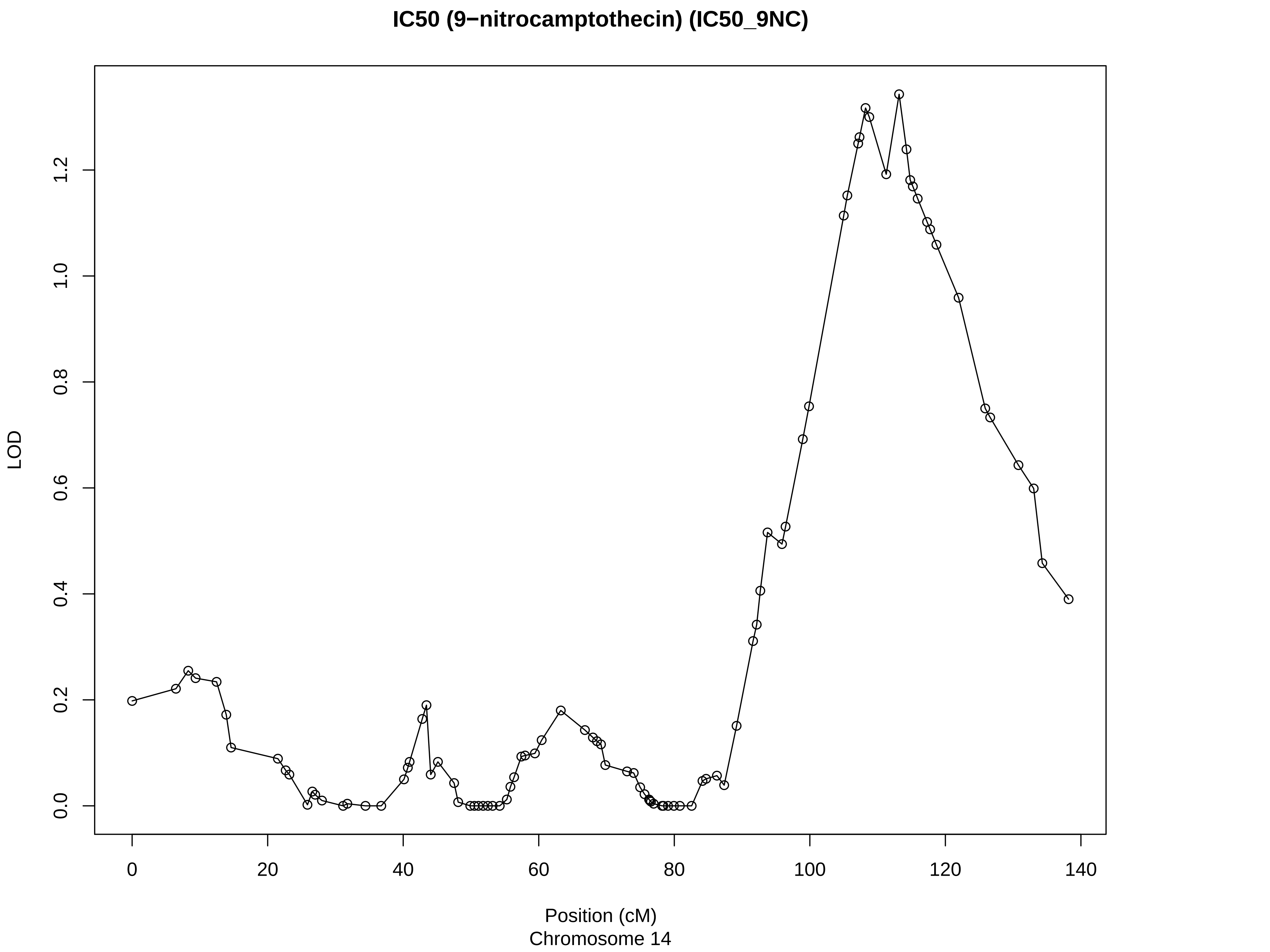
IC50 (9−nitrocamptothecin) (IC50_9NC)
LOD
0.0
0.2
0.4
0.6
0.8
1.0
1.2
0
20
40
60
80
100
120
140
Position (cM)
Chromosome 14

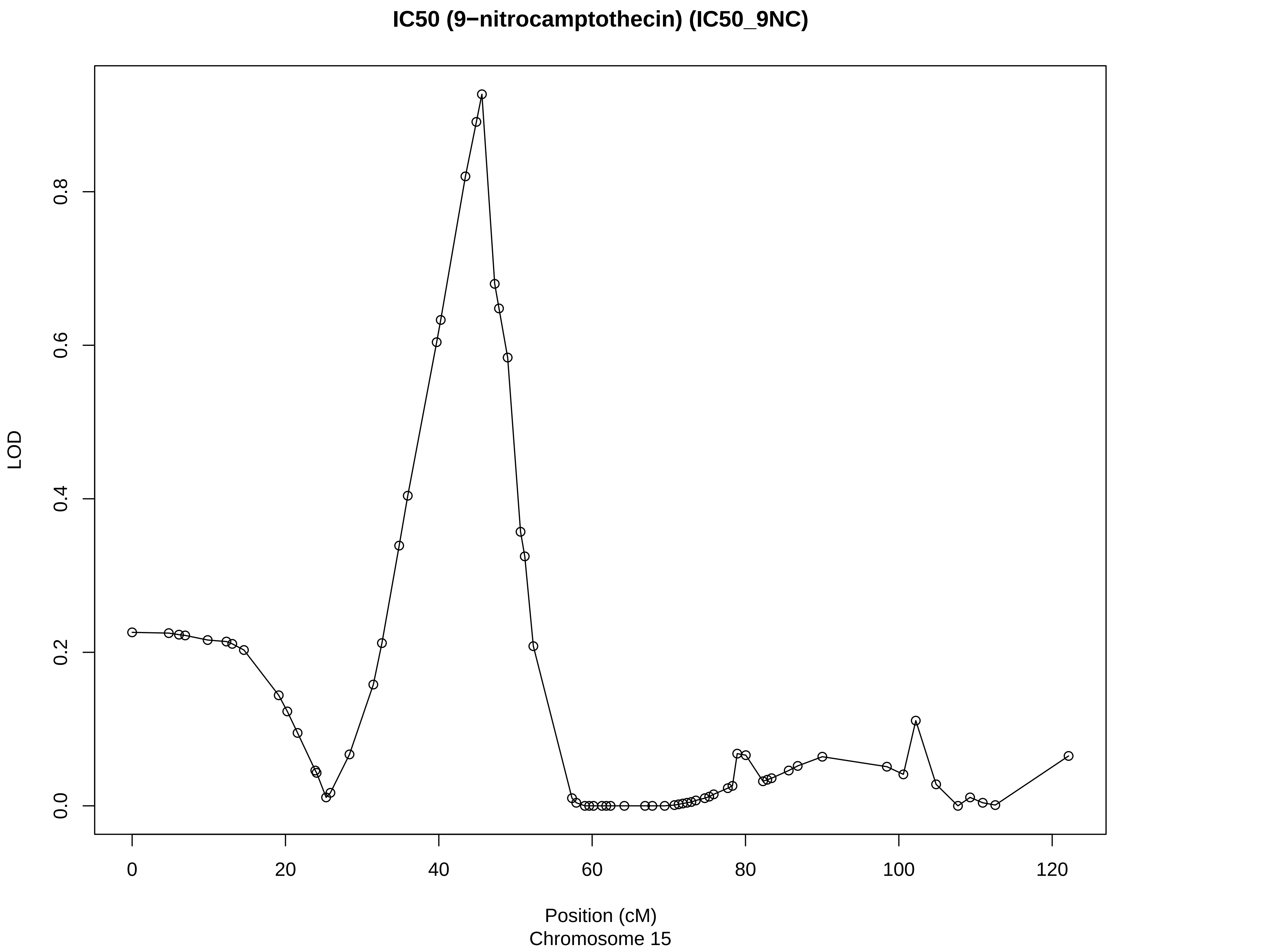
IC50 (9−nitrocamptothecin) (IC50_9NC)
LOD
0.0
0.2
0.4
0.6
0.8
0
20
40
60
80
100
120
Position (cM)
Chromosome 15

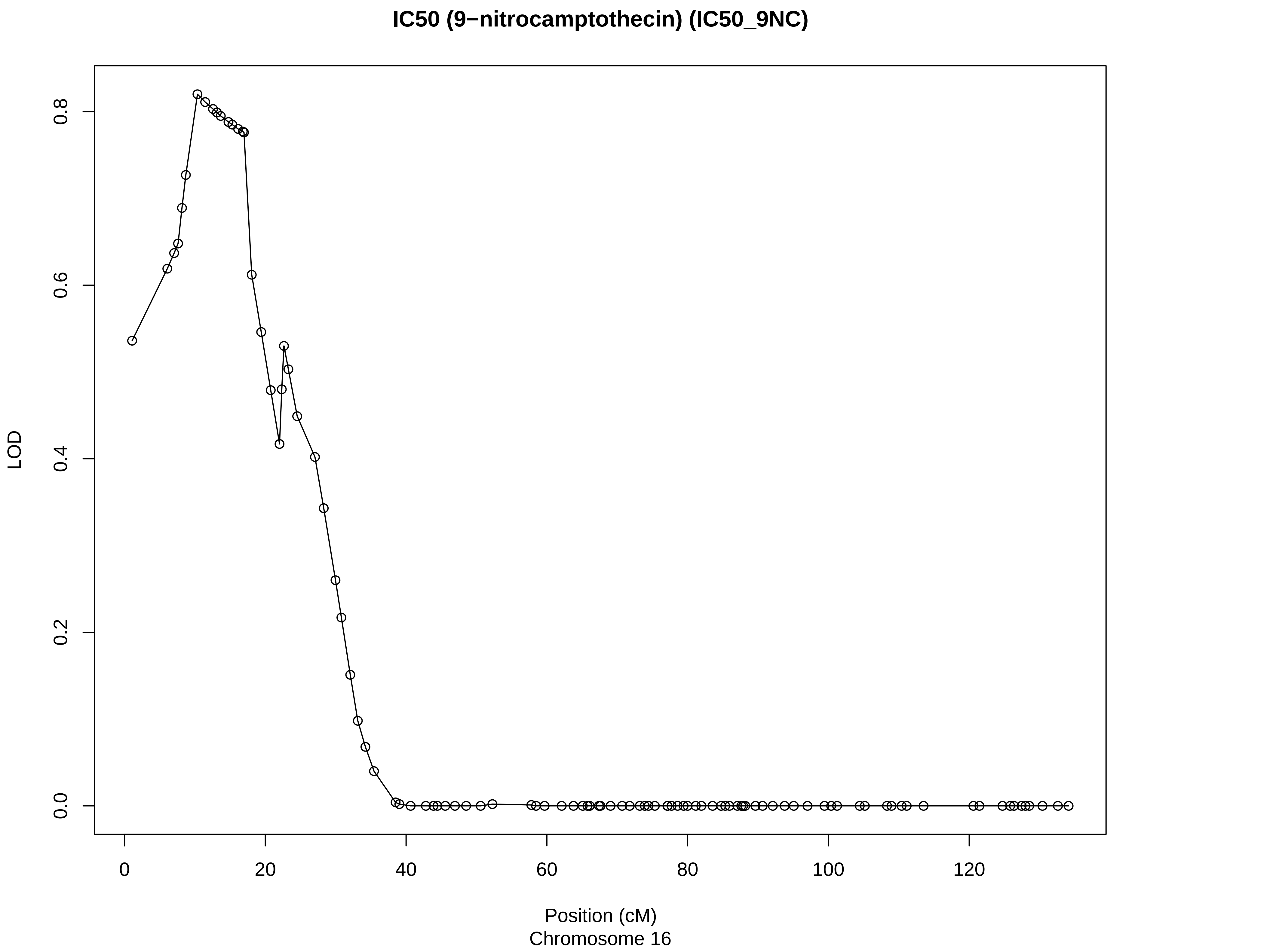
IC50 (9−nitrocamptothecin) (IC50_9NC)
LOD
0.0
0.2
0.4
0.6
0.8
0
20
40
60
80
100
120
Position (cM)
Chromosome 16

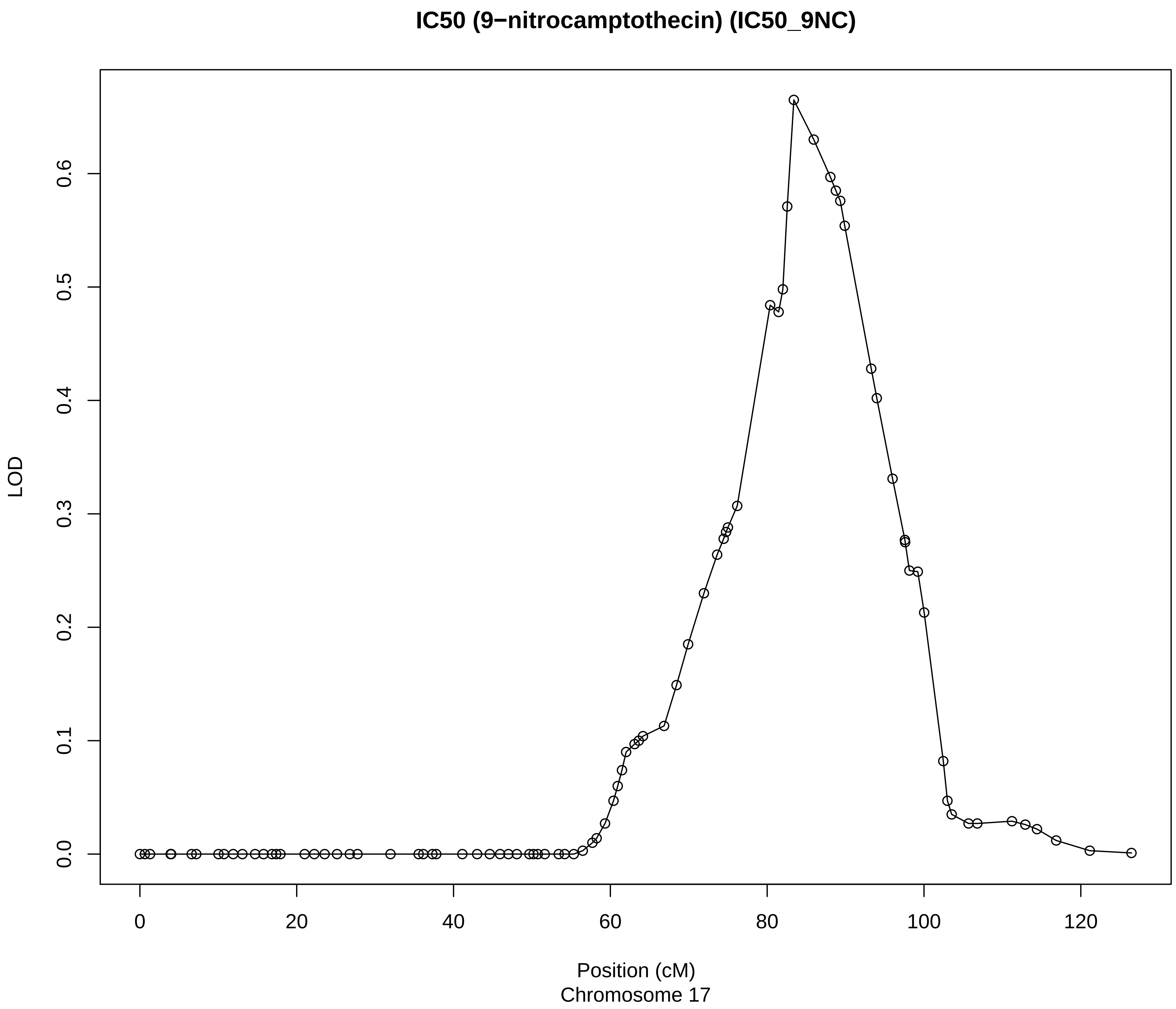
IC50 (9−nitrocamptothecin) (IC50_9NC)
LOD
0.0
0.1
0.2
0.3
0.4
0.5
0.6
0
20
40
60
80
100
120
Position (cM)
Chromosome 17

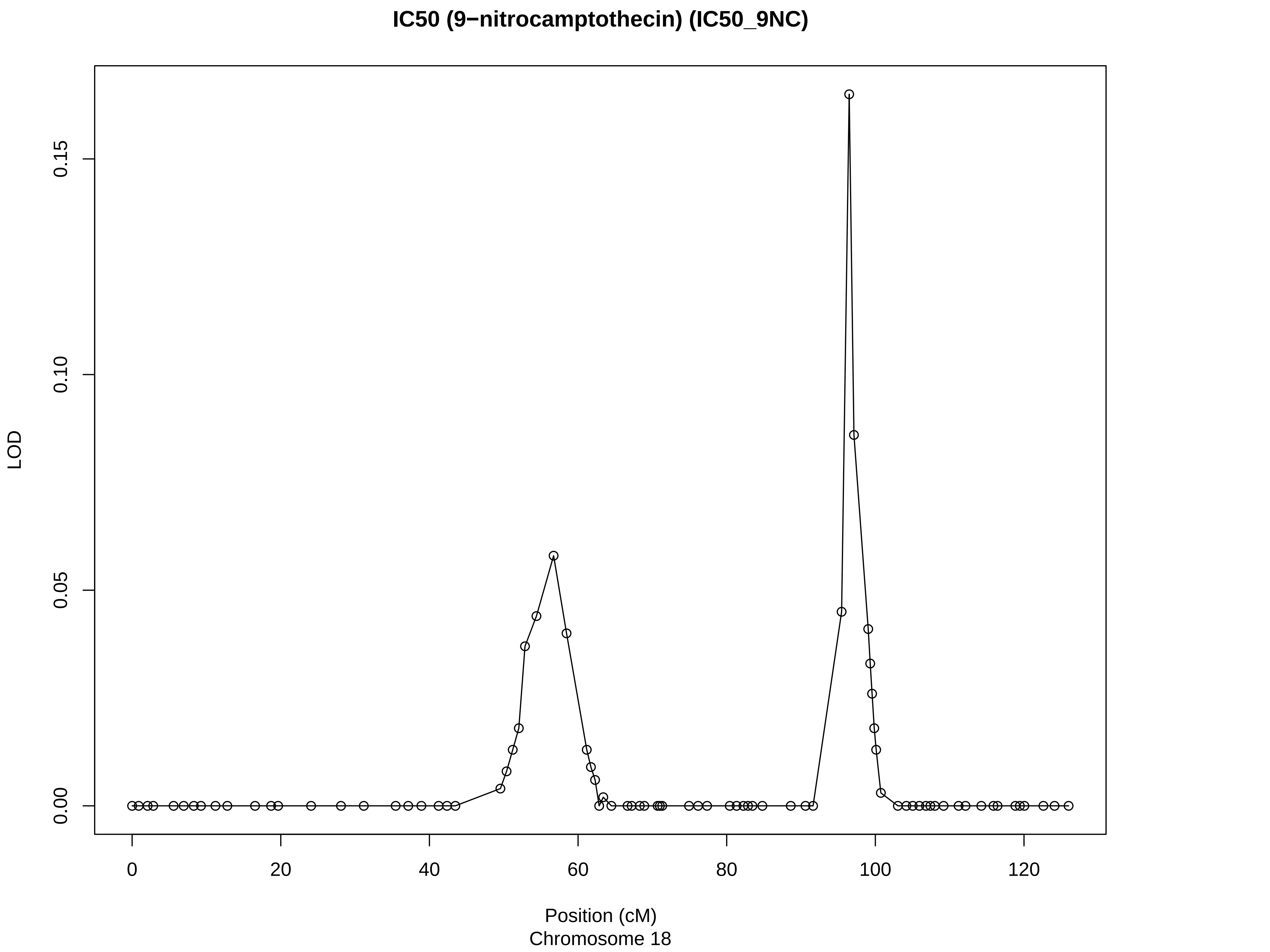
IC50 (9−nitrocamptothecin) (IC50_9NC)
LOD
0.00
0.05
0.10
0.15
0
20
40
60
80
100
120
Position (cM)
Chromosome 18

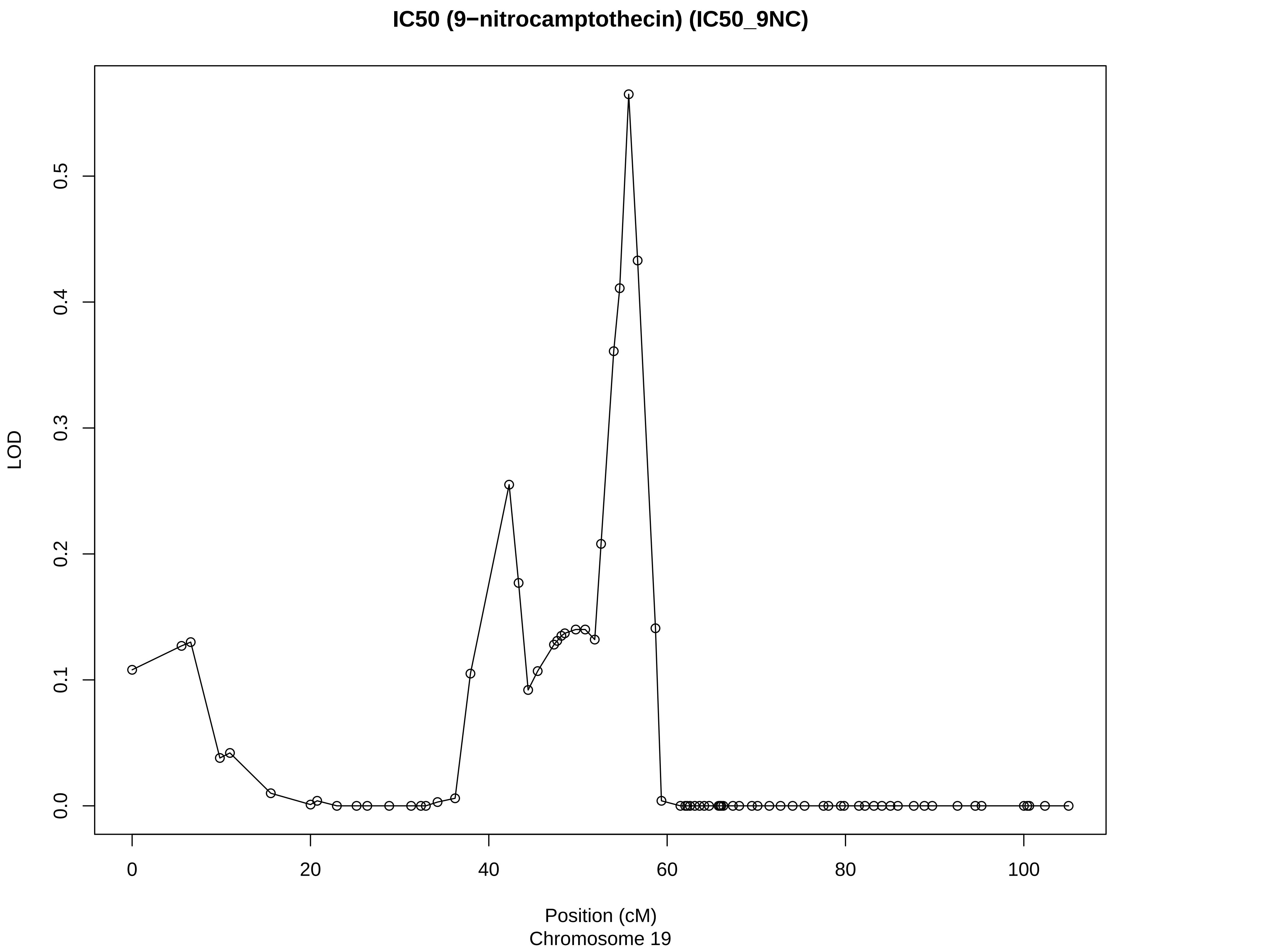

IC50 (9−nitrocamptothecin) (IC50_9NC)
LOD
0.0
0.1
0.2
0.3
0.4
0.5
0
20
40
60
80
100
Position (cM)
Chromosome 19

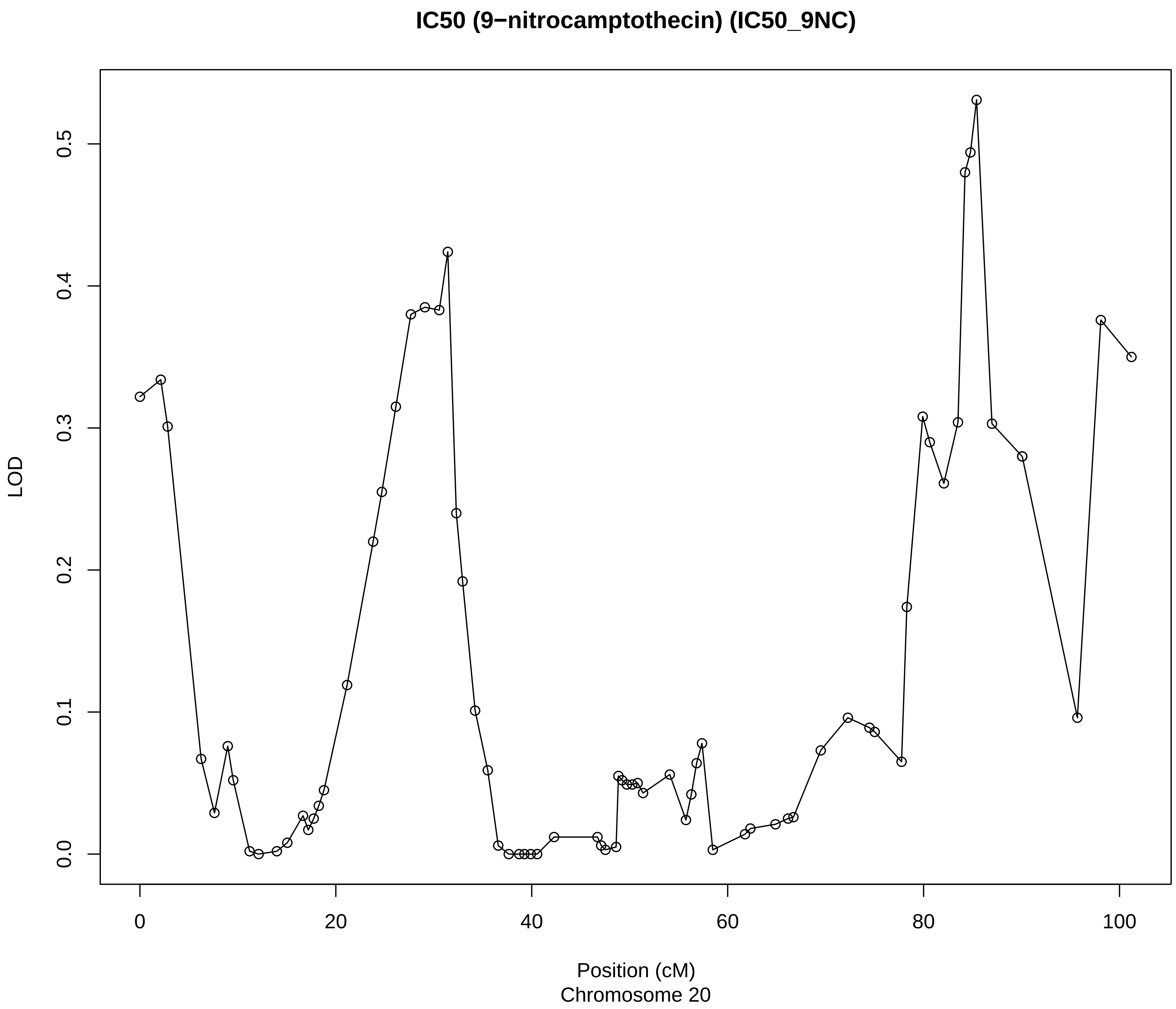
IC50 (9−nitrocamptothecin) (IC50_9NC)
LOD
0.0
0.1
0.2
0.3
0.4
0.5
0
20
40
60
80
100
Position (cM)
Chromosome 20

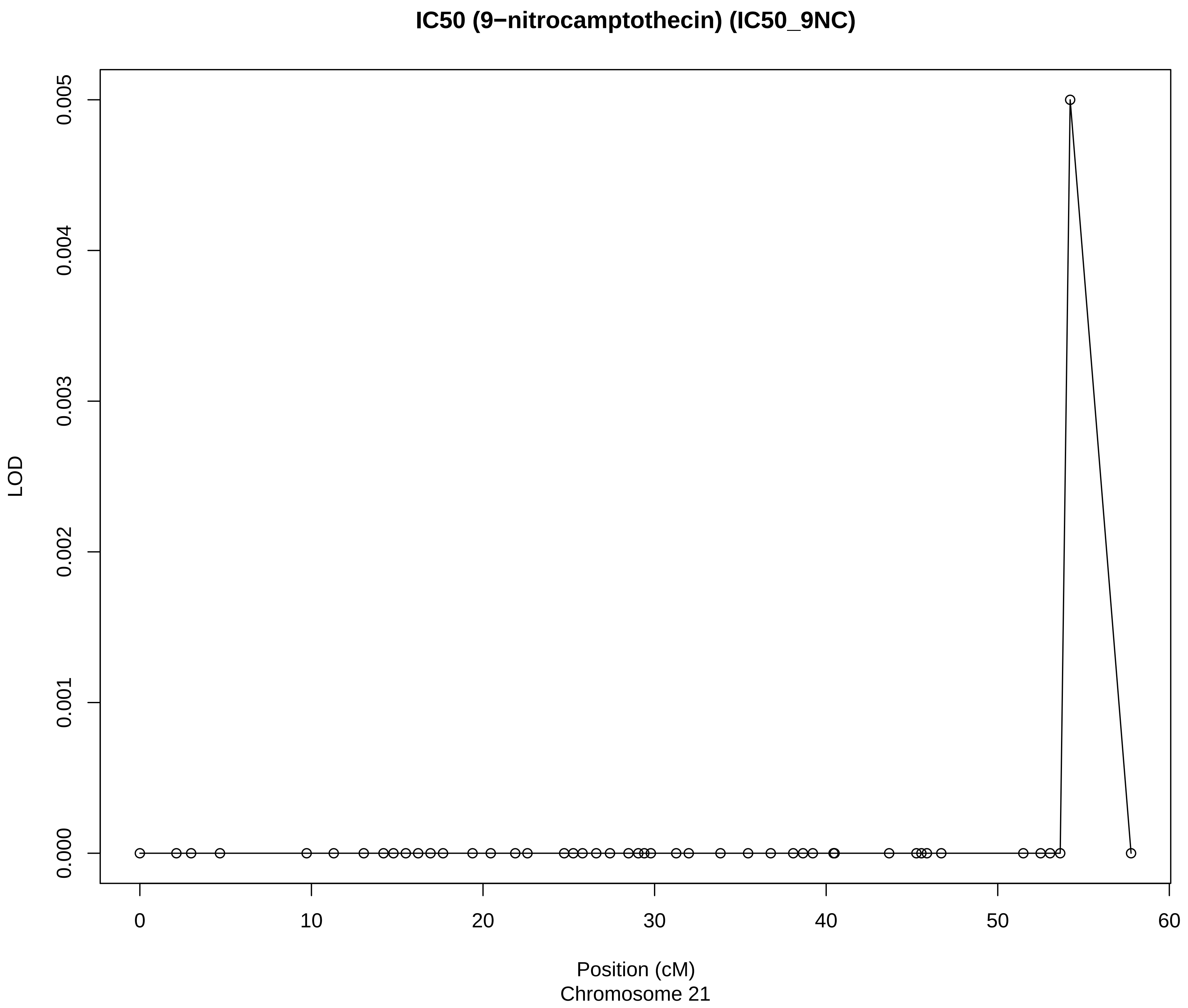
IC50 (9−nitrocamptothecin) (IC50_9NC)
LOD
0.000
0.001
0.002
0.003
0.004
0.005
0
10
20
30
40
50
60
Position (cM)
Chromosome 21

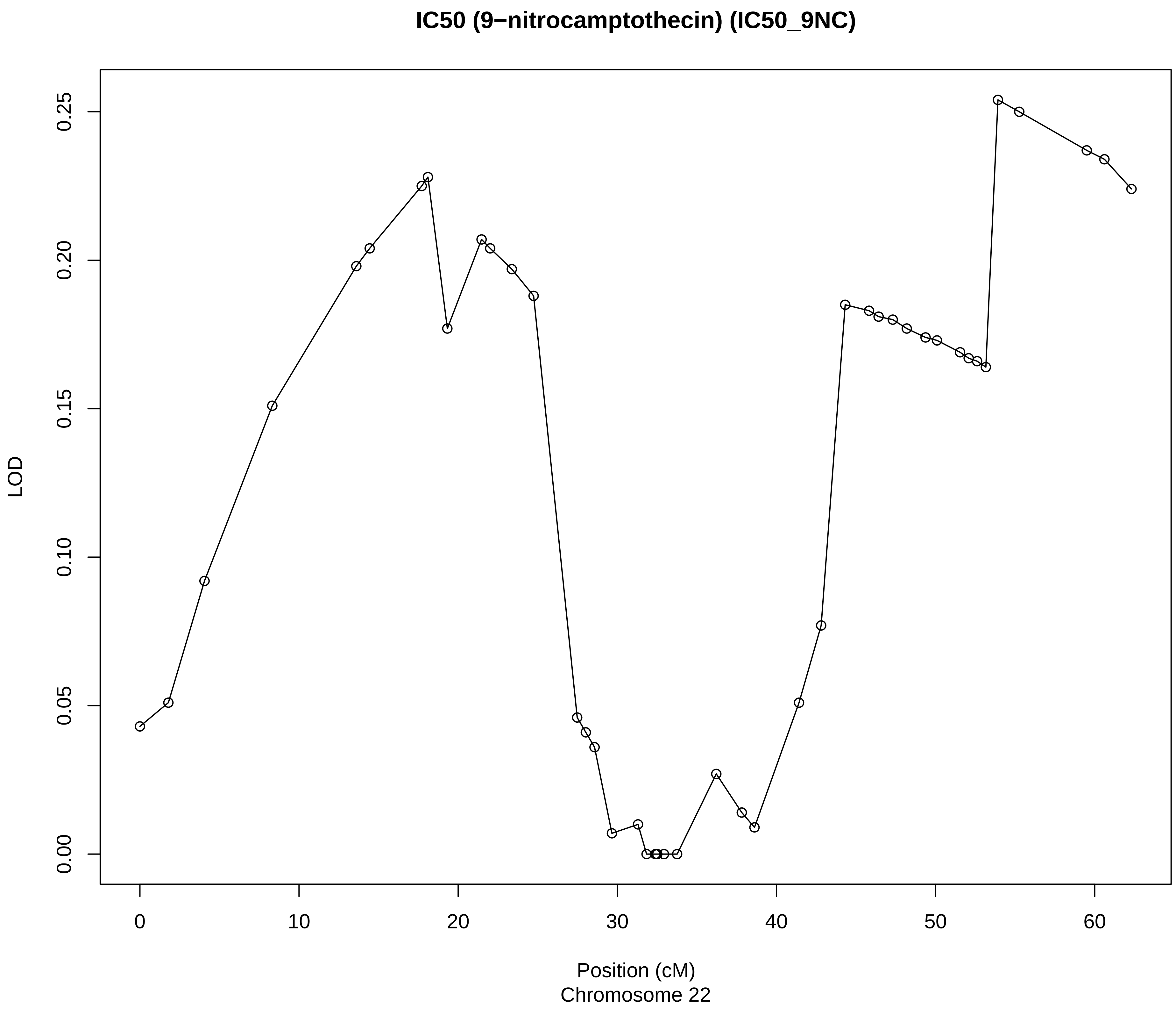

IC50 (9−nitrocamptothecin) (IC50_9NC)
LOD
0.00
0.05
0.10
0.15
0.20
0.25
0
10
20
30
40
50
60
Position (cM)
Chromosome 22

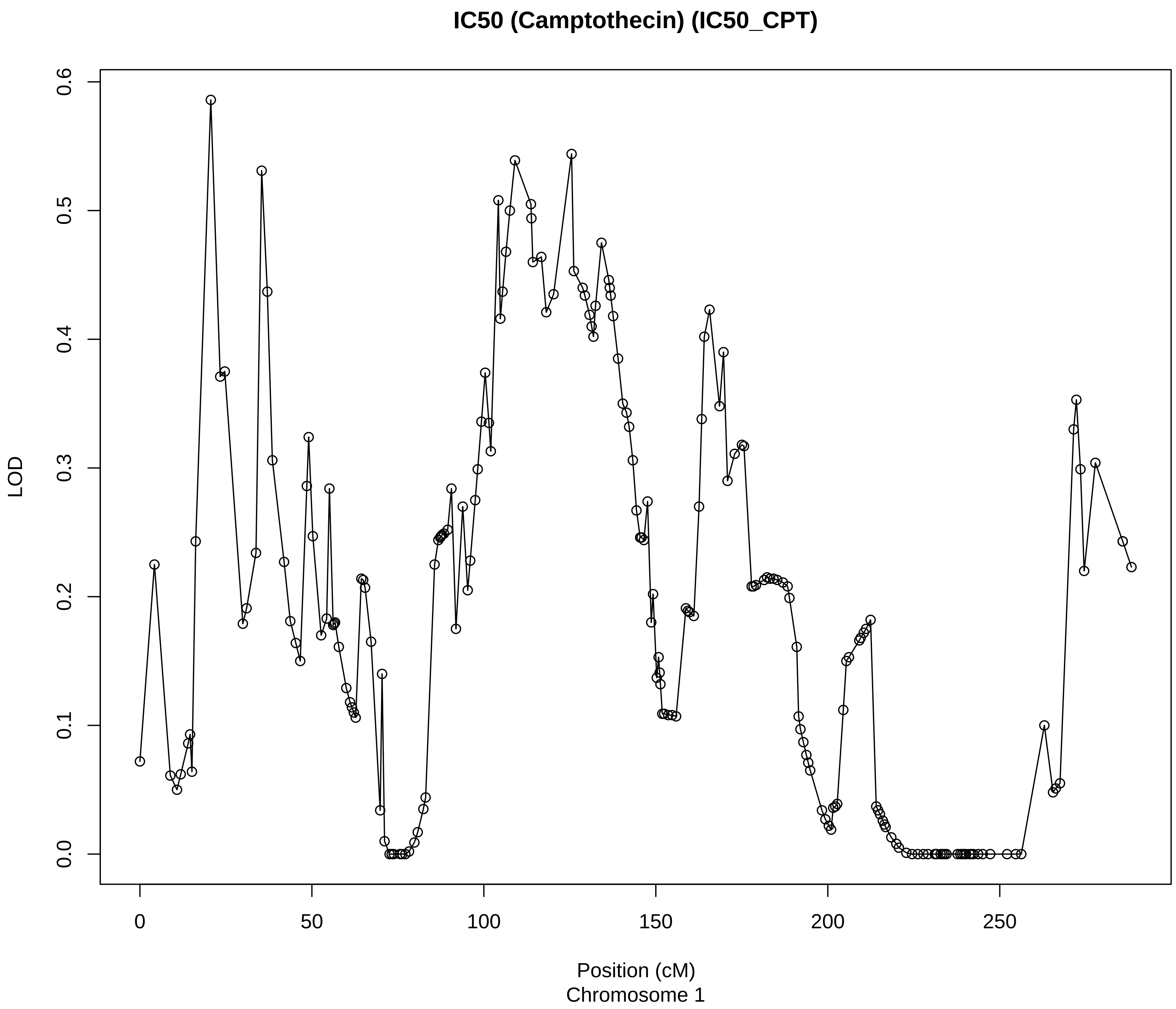

IC50 (Camptothecin) (IC50_CPT)
LOD
0.0
0.1
0.2
0.3
0.4
0.5
0.6
0
50
100
150
200
250
Position (cM)
Chromosome 1

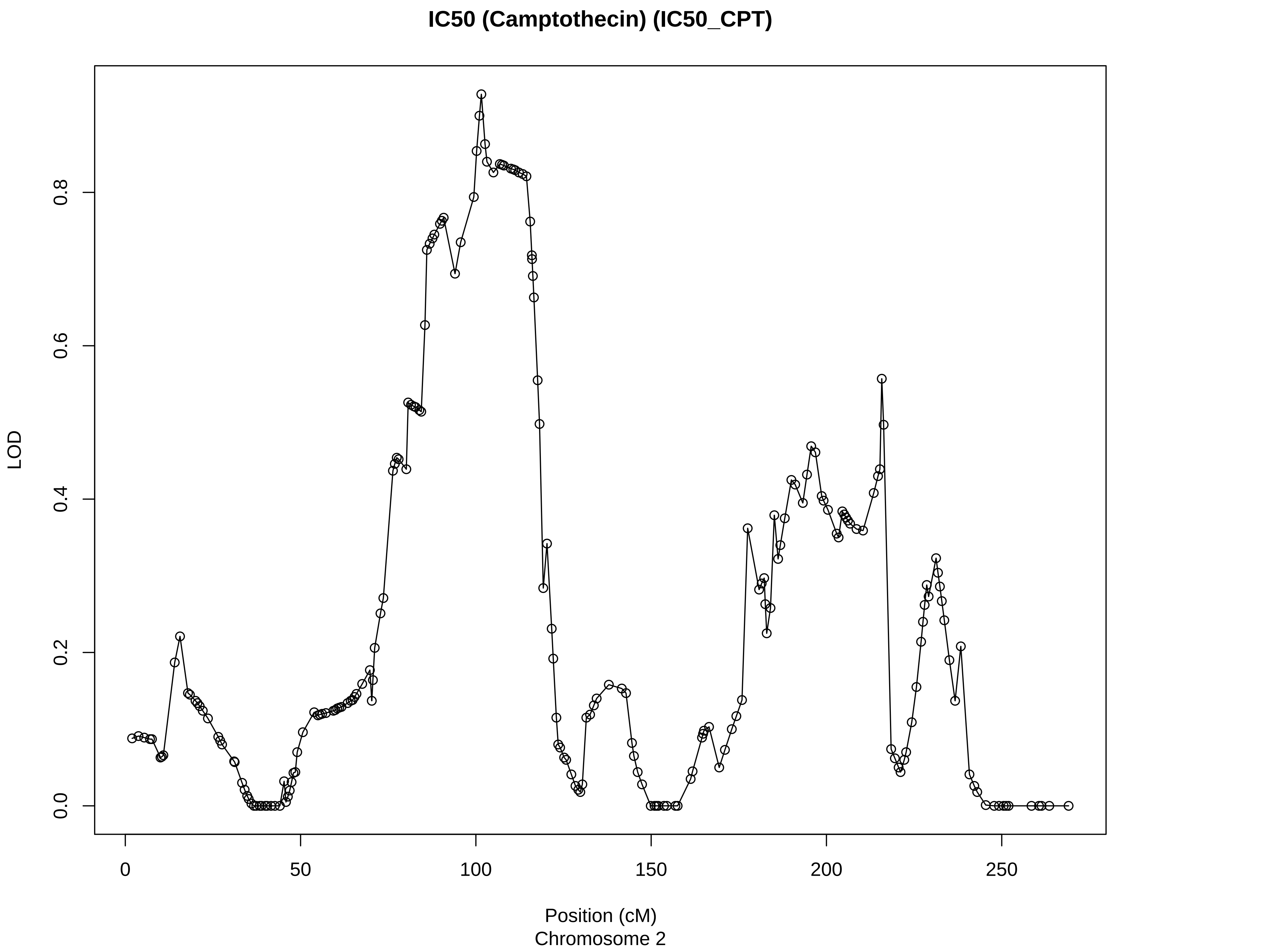
IC50 (Camptothecin) (IC50_CPT)
LOD
0.0
0.2
0.4
0.6
0.8
0
50
100
150
200
250
Position (cM)
Chromosome 2

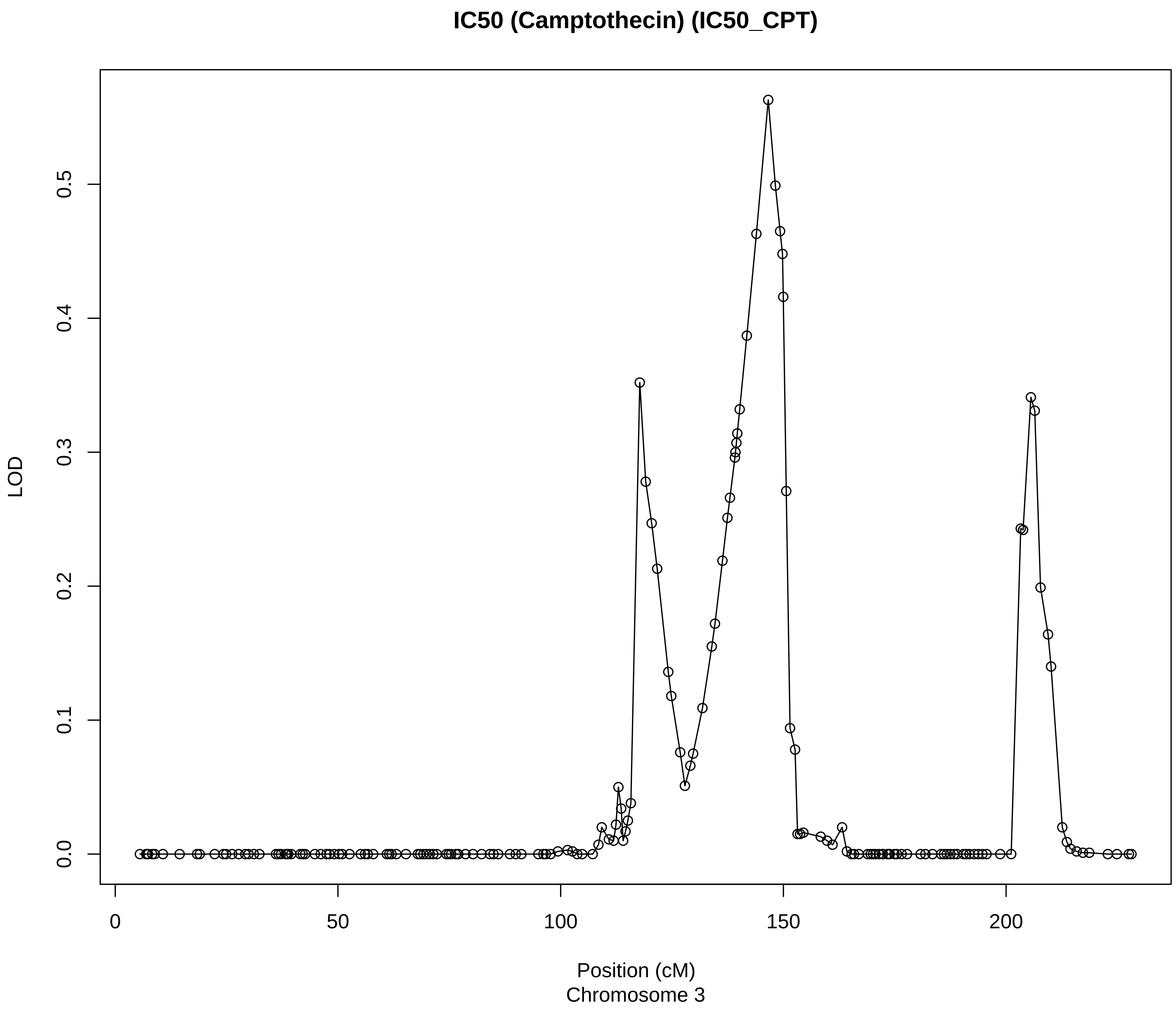

IC50 (Camptothecin) (IC50_CPT)
LOD
0.0
0.1
0.2
0.3
0.4
0.5
0
50
100
150
200
Position (cM)
Chromosome 3

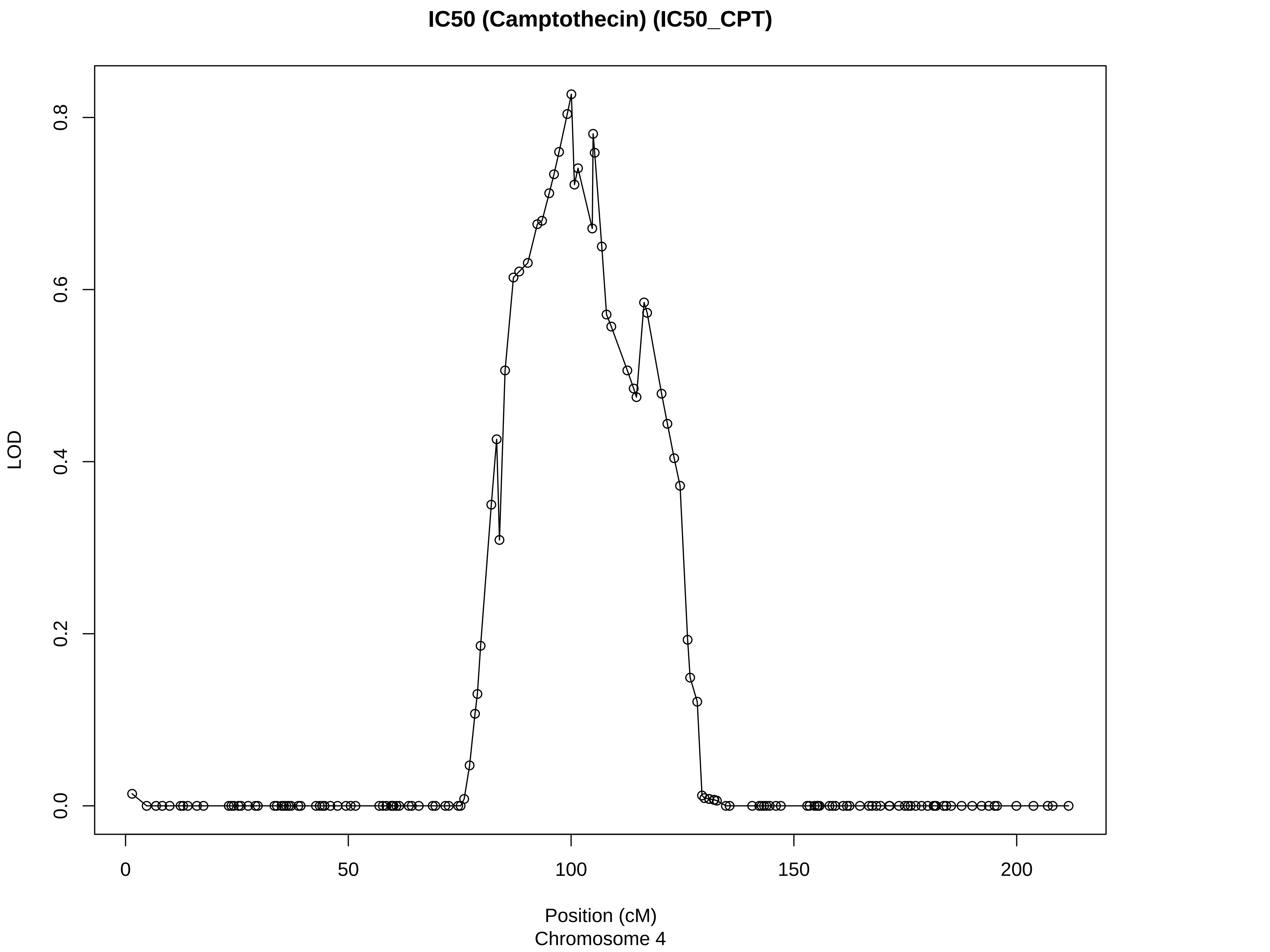
IC50 (Camptothecin) (IC50_CPT)
LOD
0.0
0.2
0.4
0.6
0.8
0
50
100
150
200
Position (cM)
Chromosome 4

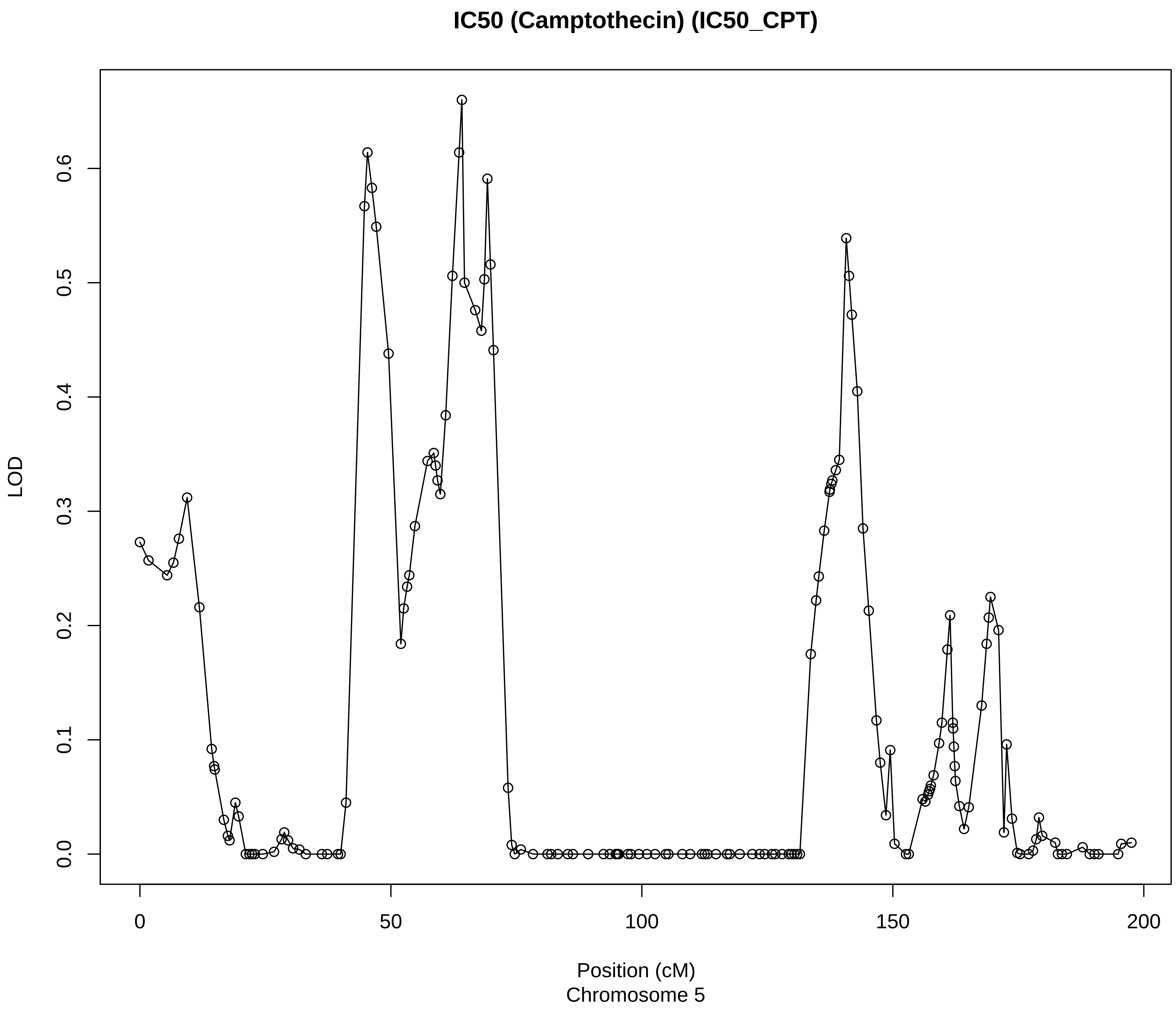
IC50 (Camptothecin) (IC50_CPT)
LOD
0.0
0.1
0.2
0.3
0.4
0.5
0.6
0
50
100
150
200
Position (cM)
Chromosome 5

IC50 (Camptothecin) (IC50_CPT)

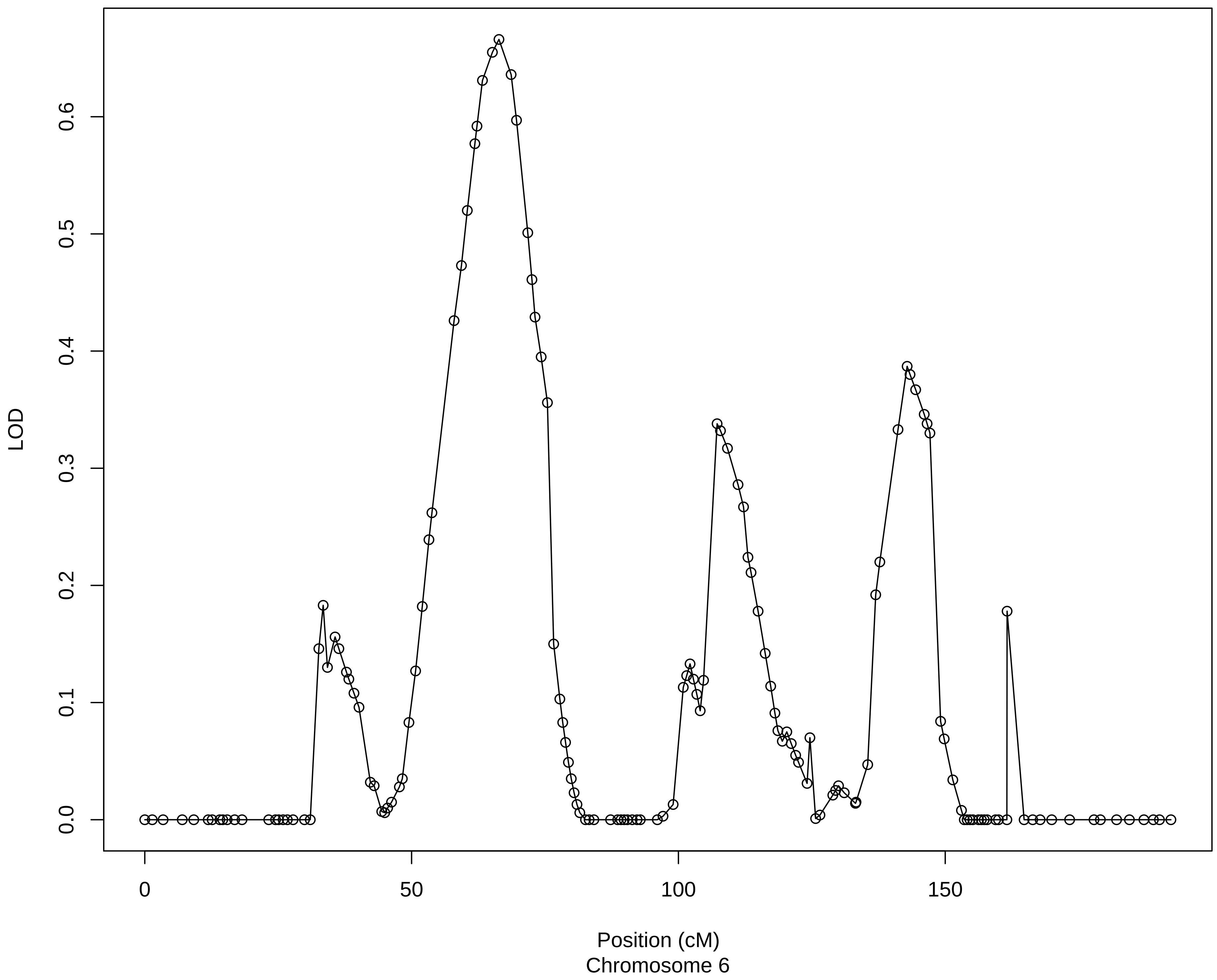

IC50 (Camptothecin) (IC50_CPT)

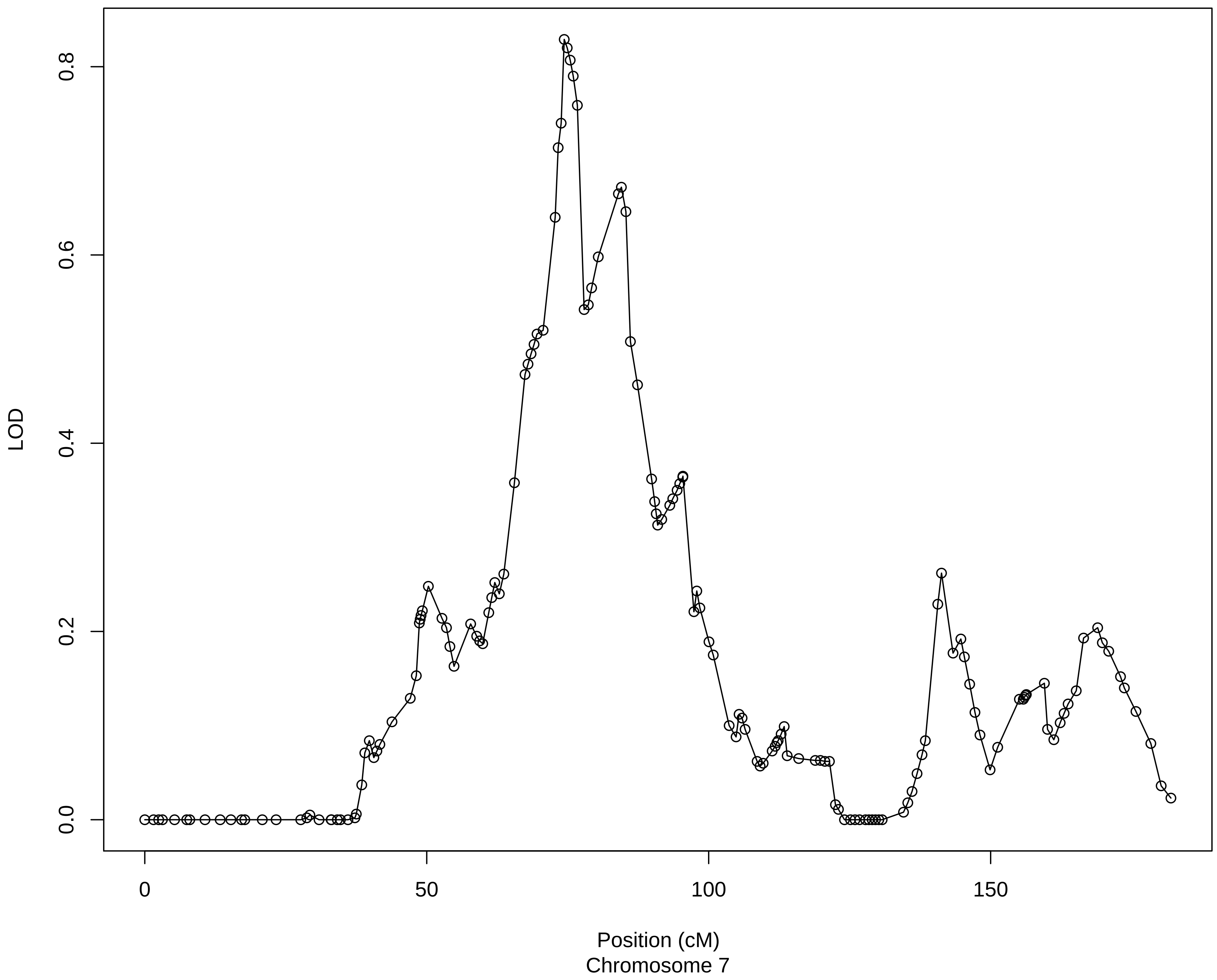

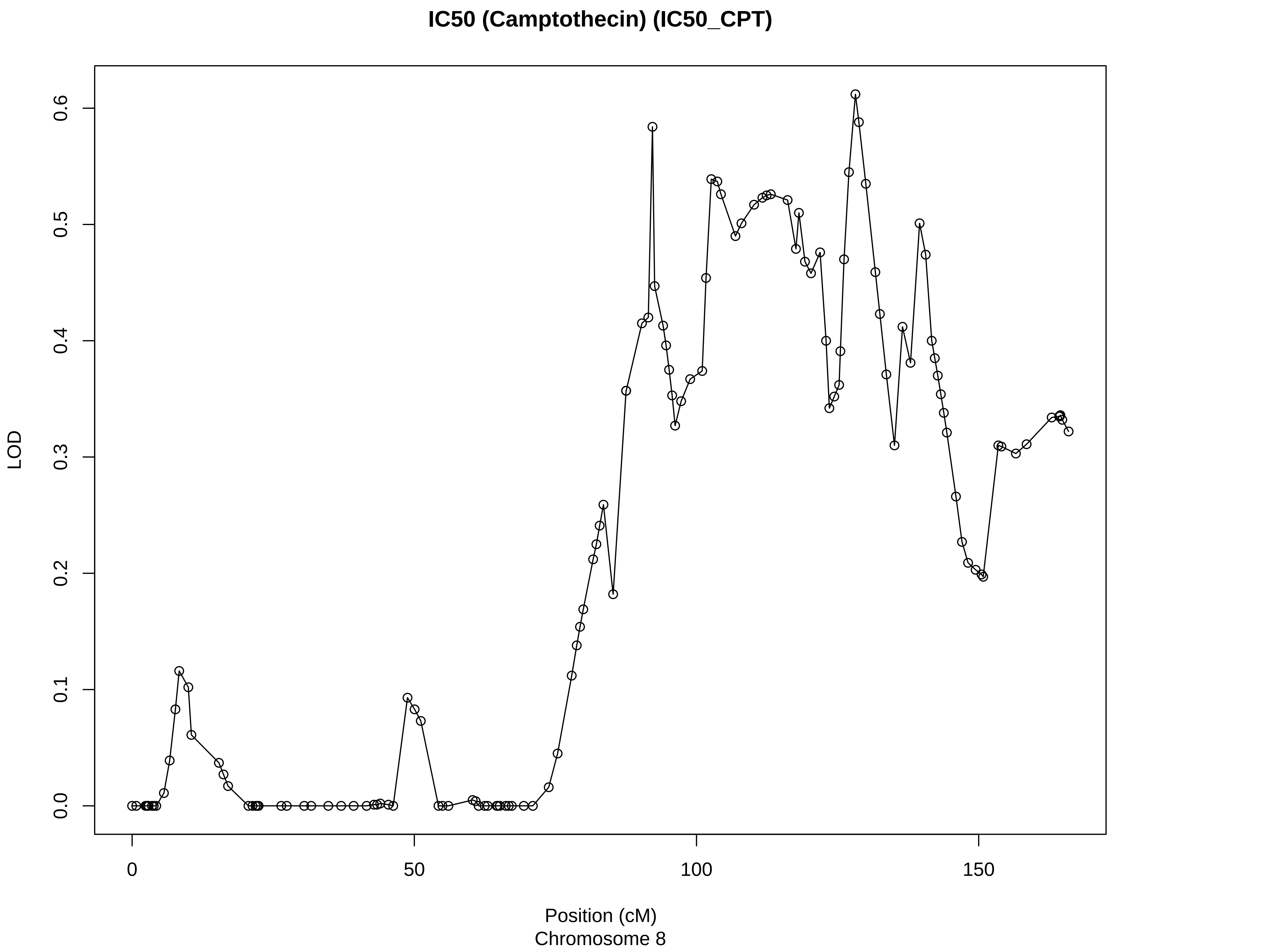

IC50 (Camptothecin) (IC50_CPT)
LOD
0.0
0.1
0.2
0.3
0.4
0.5
0.6
0
50
100
150
Position (cM)
Chromosome 8

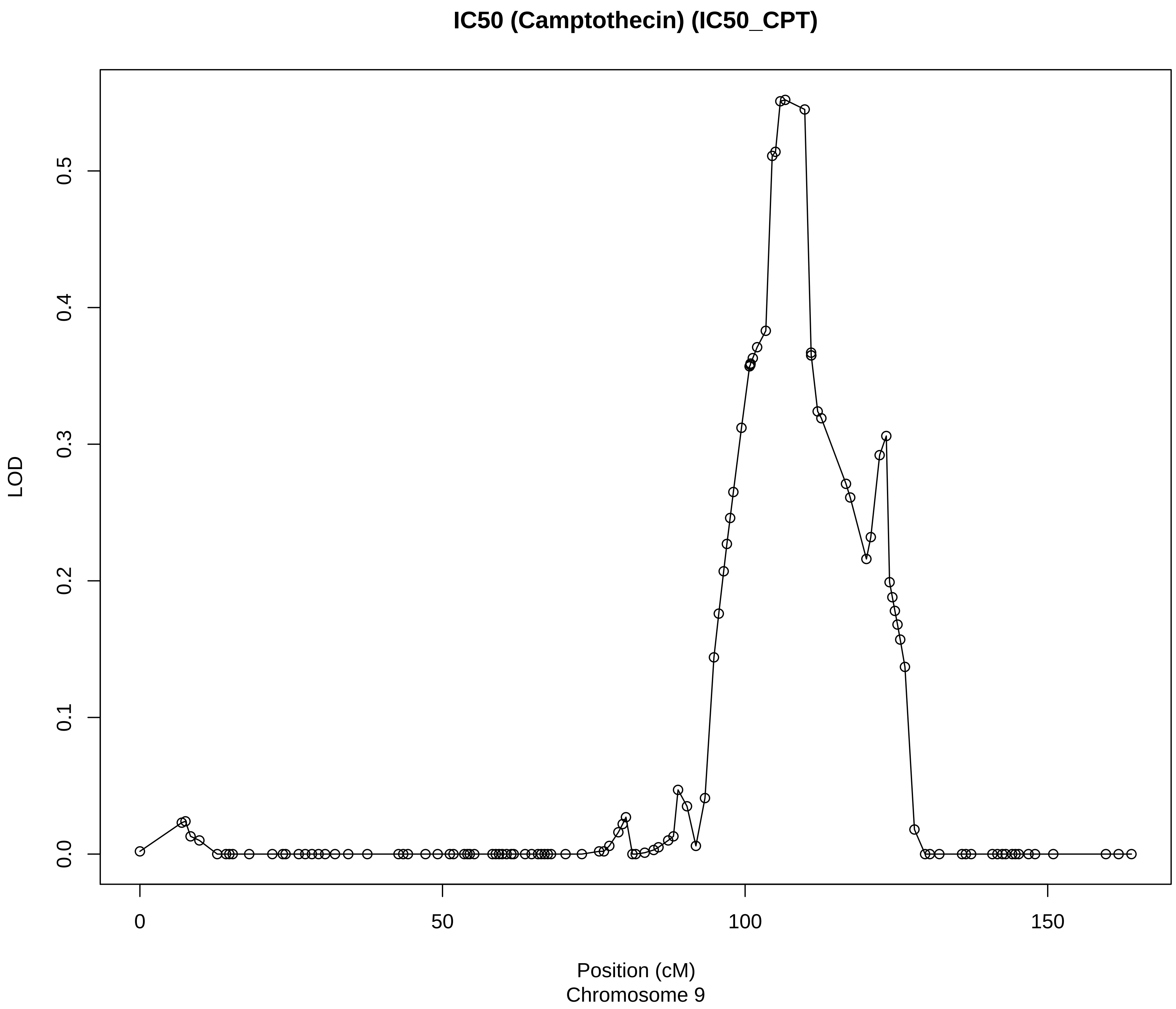
IC50 (Camptothecin) (IC50_CPT)
LOD
0.0
0.1
0.2
0.3
0.4
0.5
0
50
100
150
Position (cM)
Chromosome 9

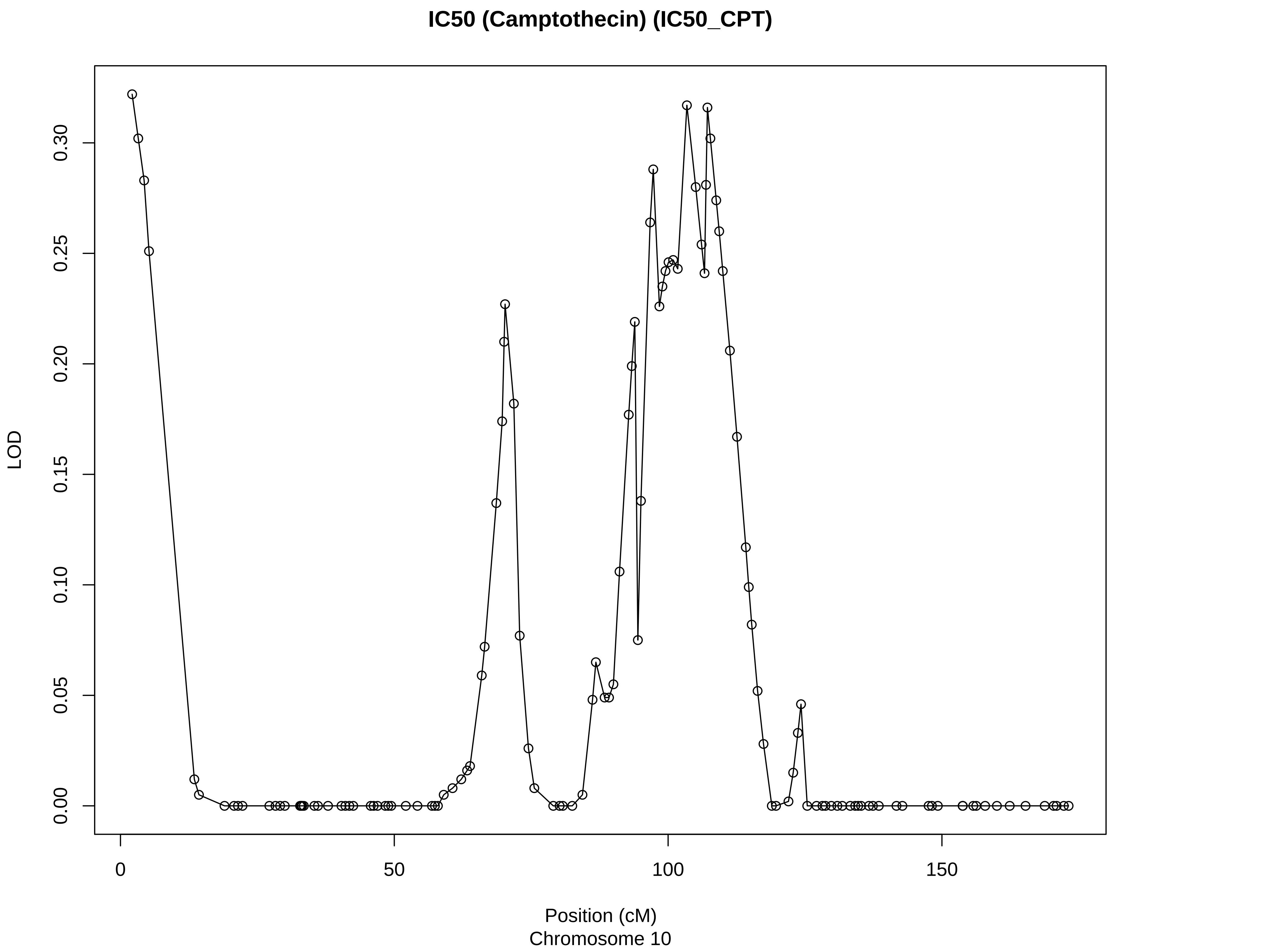

IC50 (Camptothecin) (IC50_CPT)
LOD
0.00
0.05
0.10
0.15
0.20
0.25
0.30
0
50
100
150
Position (cM)
Chromosome 10

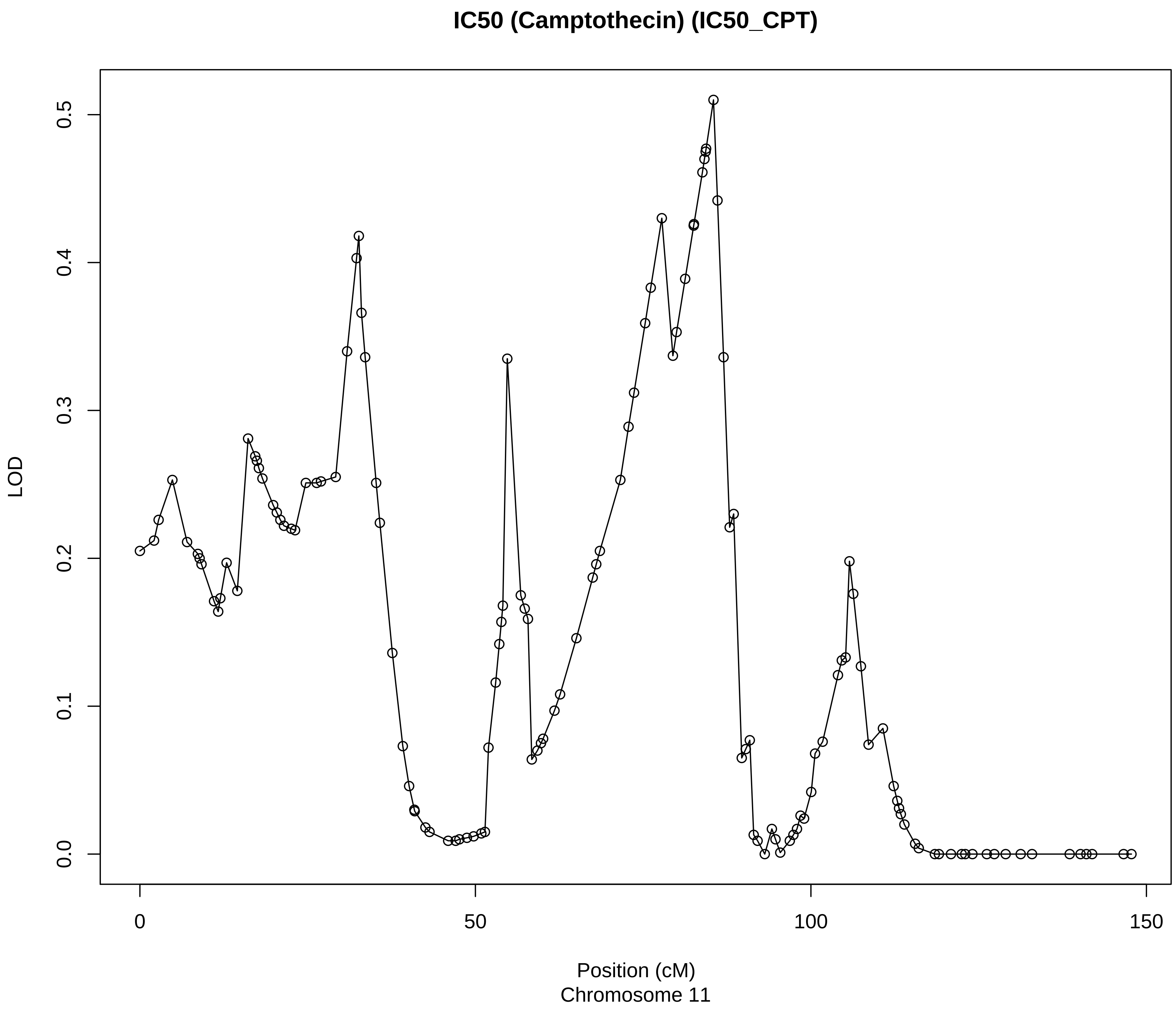
IC50 (Camptothecin) (IC50_CPT)
LOD
0.0
0.1
0.2
0.3
0.4
0.5
0
50
100
150
Position (cM)
Chromosome 11

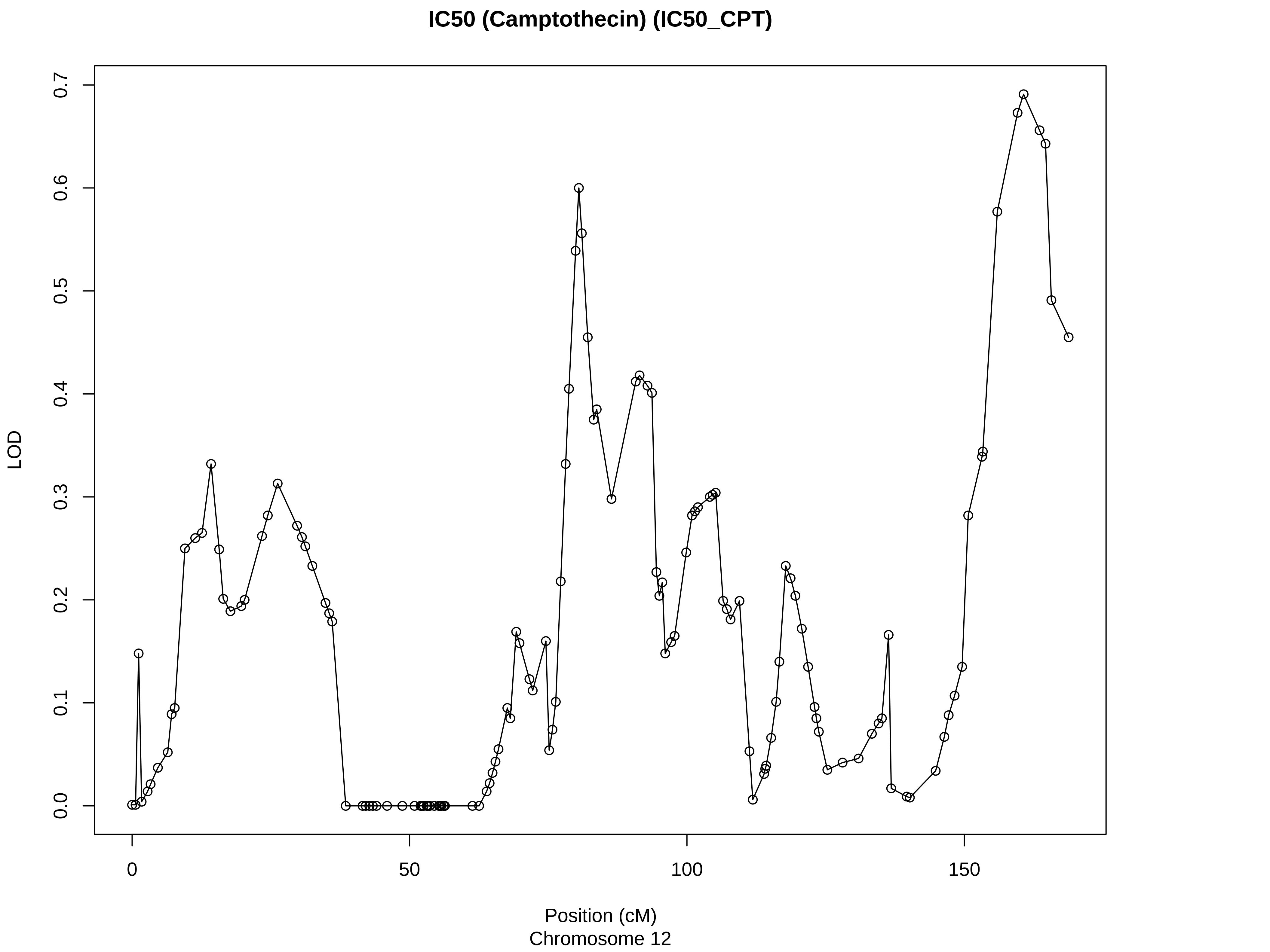

IC50 (Camptothecin) (IC50_CPT)
LOD
0.0
0.1
0.2
0.3
0.4
0.5
0.6
0.7
0
50
100
150
Position (cM)
Chromosome 12

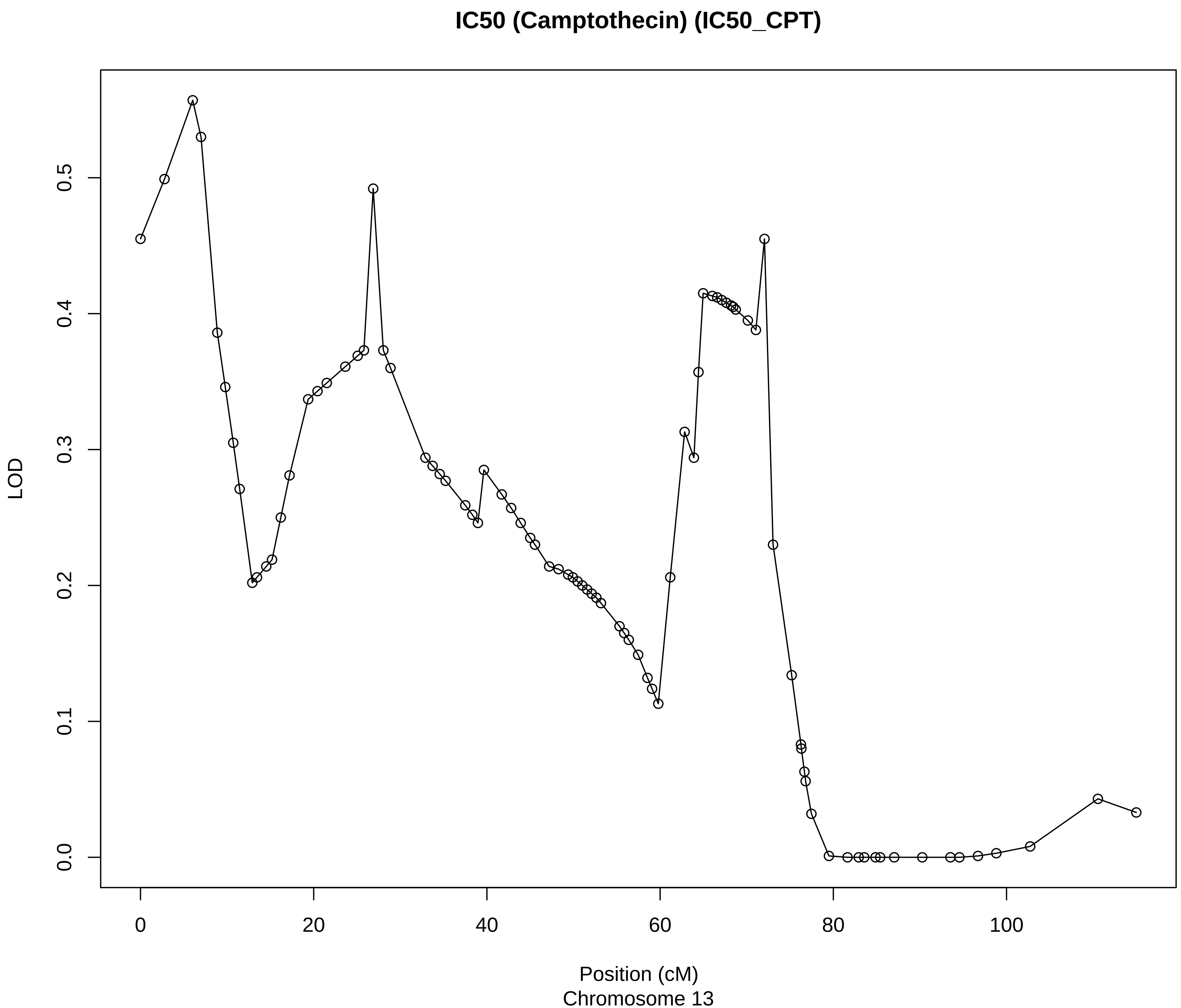
IC50 (Camptothecin) (IC50_CPT)
LOD
0.0
0.1
0.2
0.3
0.4
0.5
0
20
40
60
80
100
Position (cM)
Chromosome 13

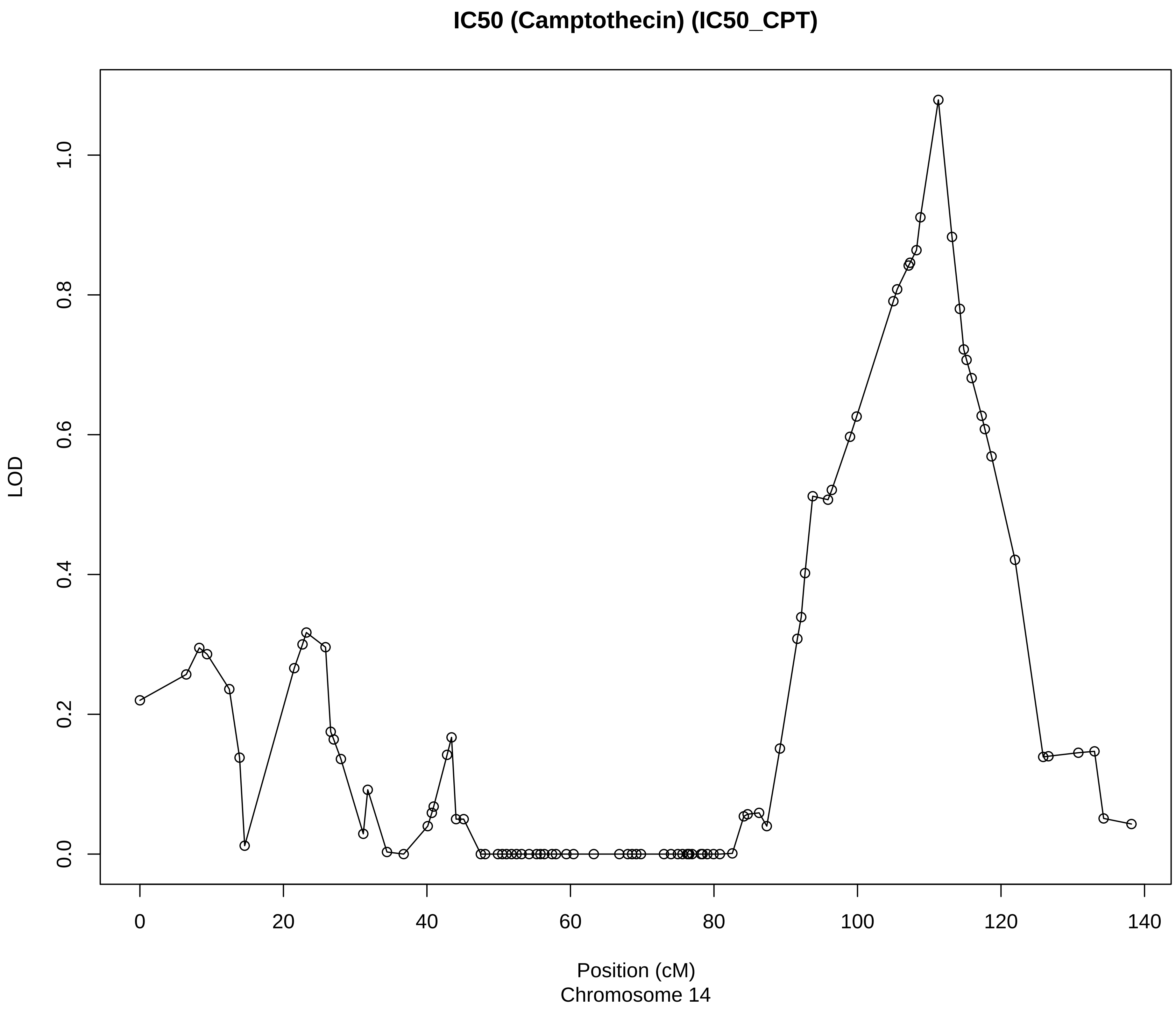
IC50 (Camptothecin) (IC50_CPT)
LOD
0.0
0.2
0.4
0.6
0.8
1.0
0
20
40
60
80
100
120
140
Position (cM)
Chromosome 14

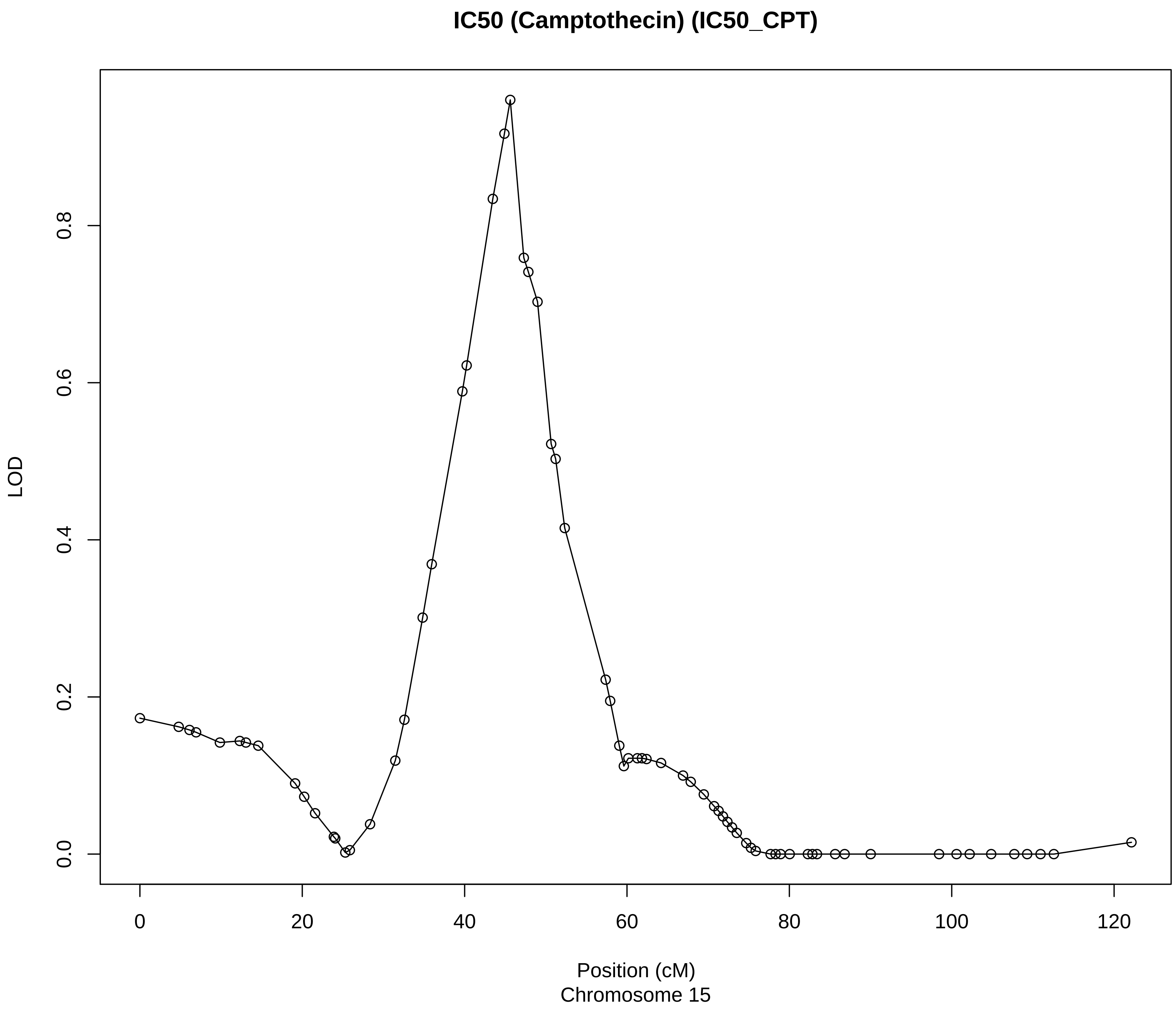

IC50 (Camptothecin) (IC50_CPT)
LOD
0.0
0.2
0.4
0.6
0.8
0
20
40
60
80
100
120
Position (cM)
Chromosome 15

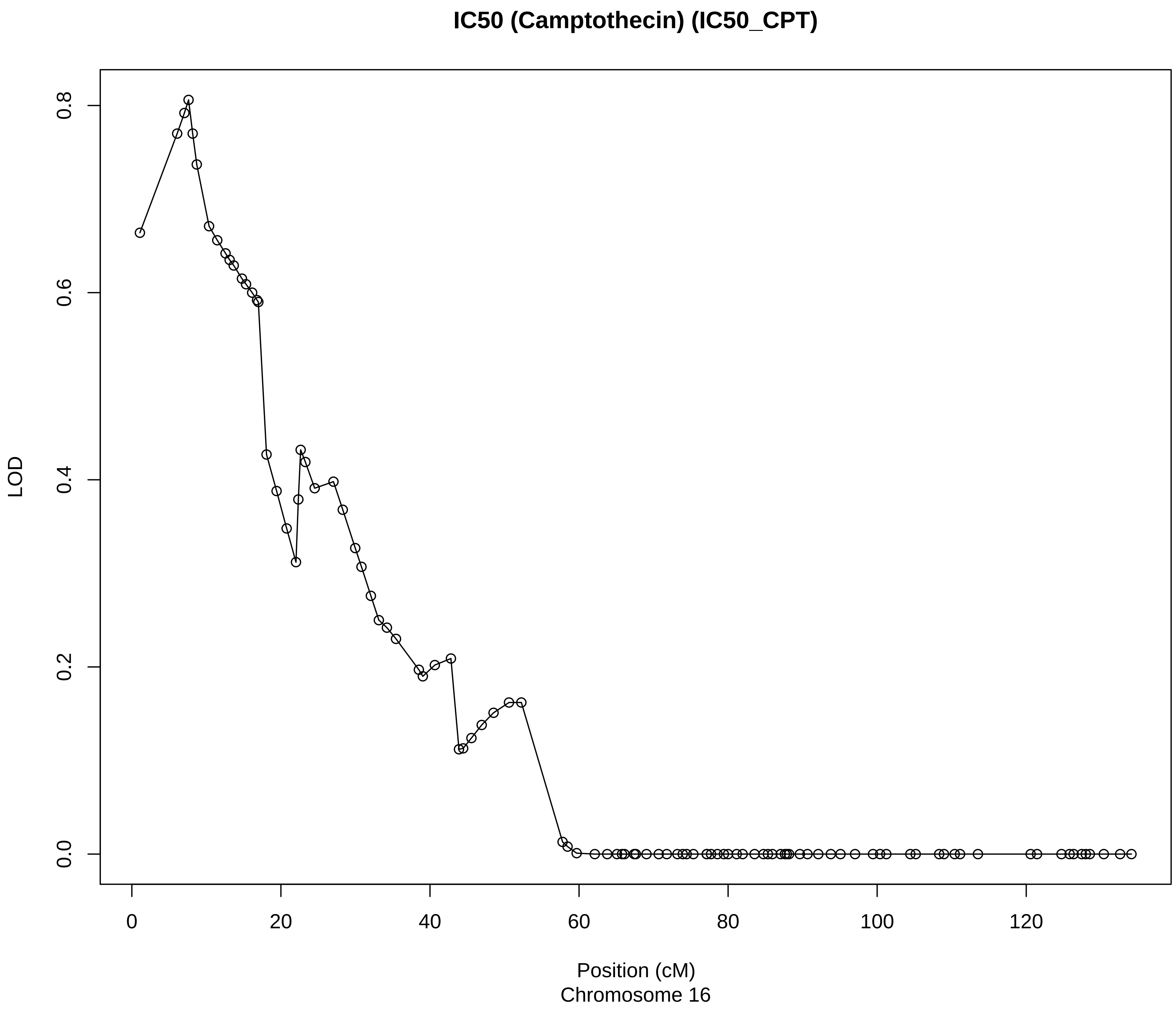

IC50 (Camptothecin) (IC50_CPT)
LOD
0.0
0.2
0.4
0.6
0.8
0
20
40
60
80
100
120
Position (cM)
Chromosome 16

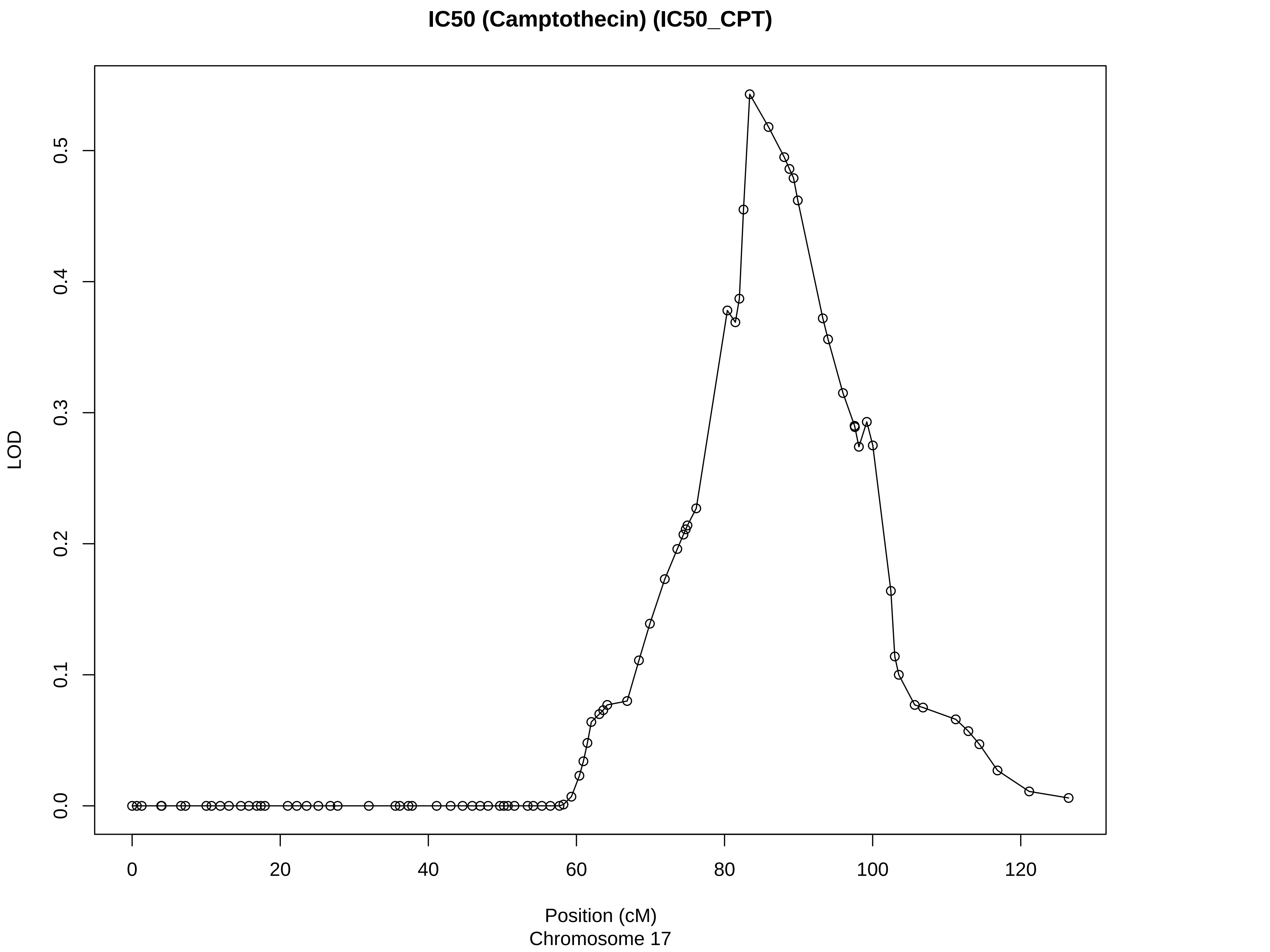
IC50 (Camptothecin) (IC50_CPT)
LOD
0.0
0.1
0.2
0.3
0.4
0.5
0
20
40
60
80
100
120
Position (cM)
Chromosome 17

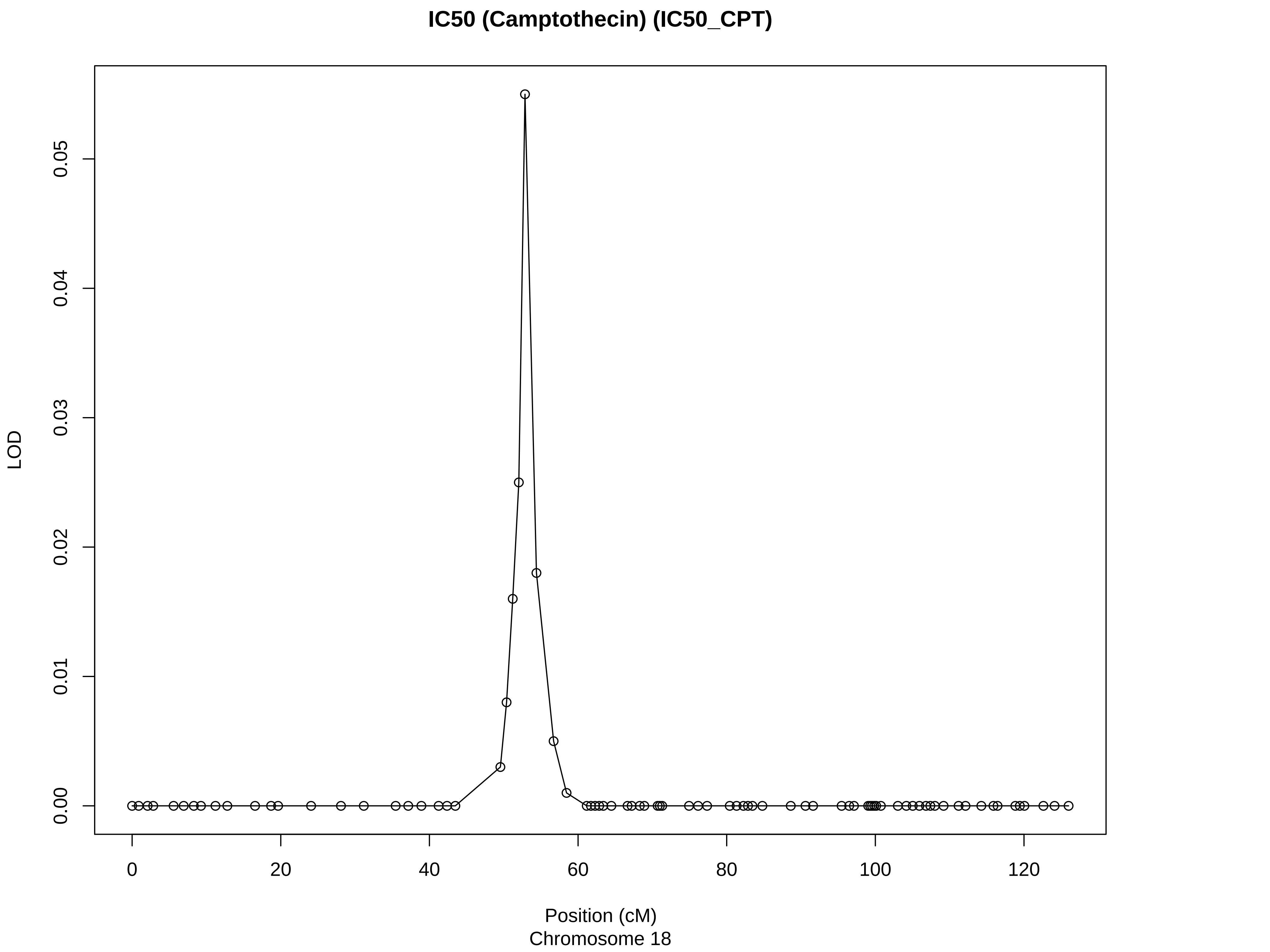
IC50 (Camptothecin) (IC50_CPT)
LOD
0.00
0.01
0.02
0.03
0.04
0.05
0
20
40
60
80
100
120
Position (cM)
Chromosome 18

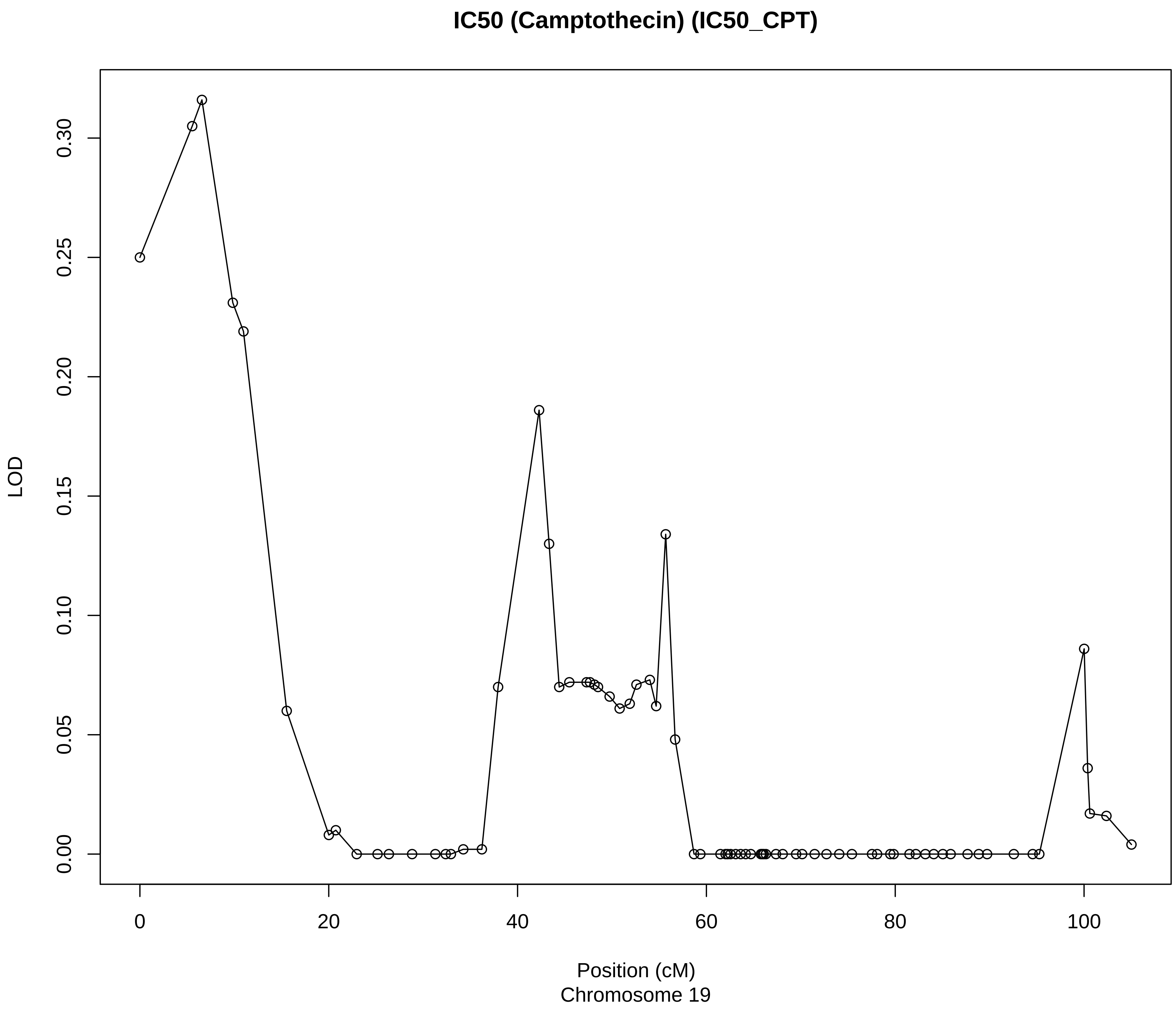

IC50 (Camptothecin) (IC50_CPT)
LOD
0.00
0.05
0.10
0.15
0.20
0.25
0.30
0
20
40
60
80
100
Position (cM)
Chromosome 19

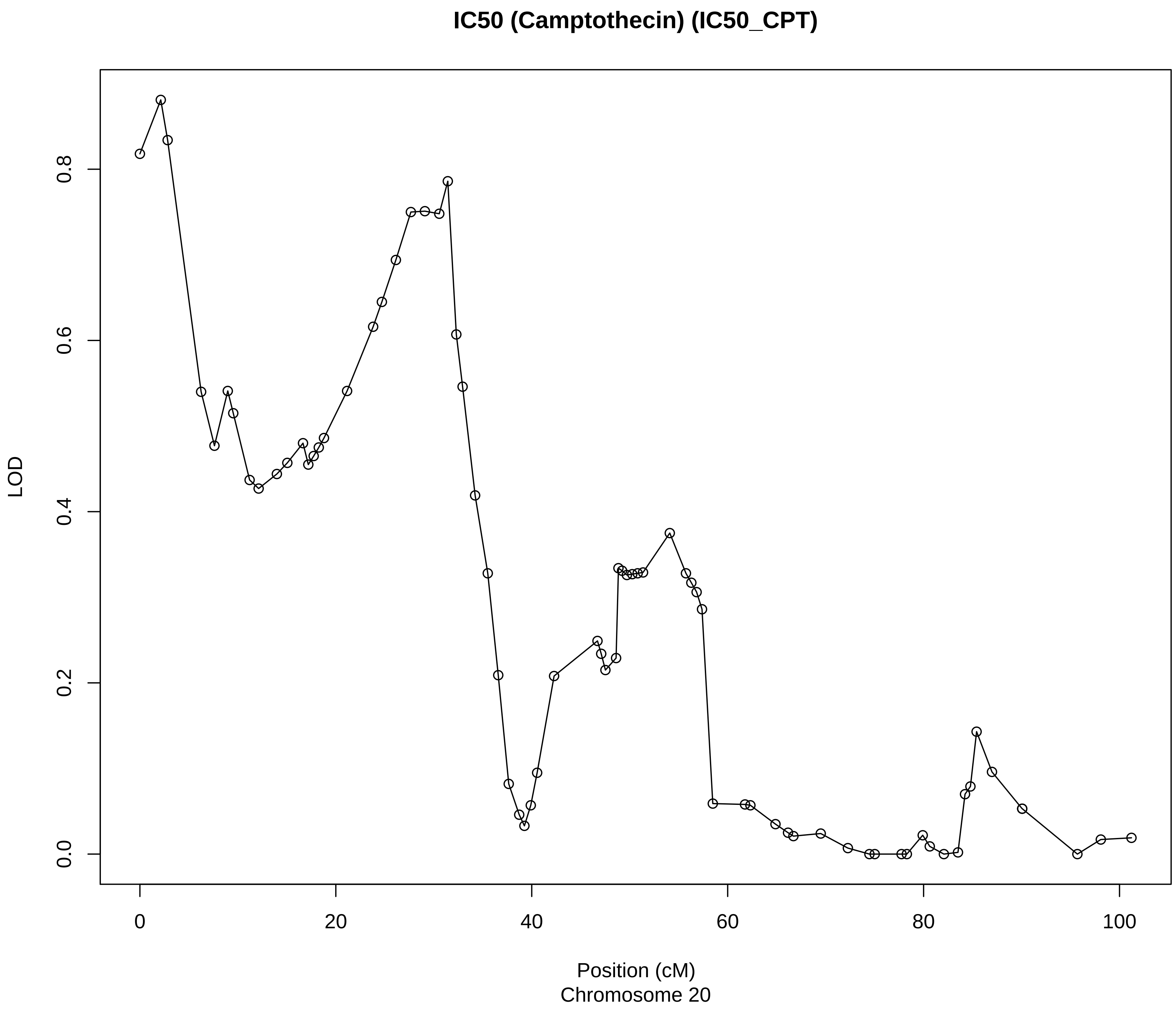
IC50 (Camptothecin) (IC50_CPT)
LOD
0.0
0.2
0.4
0.6
0.8
0
20
40
60
80
100
Position (cM)
Chromosome 20

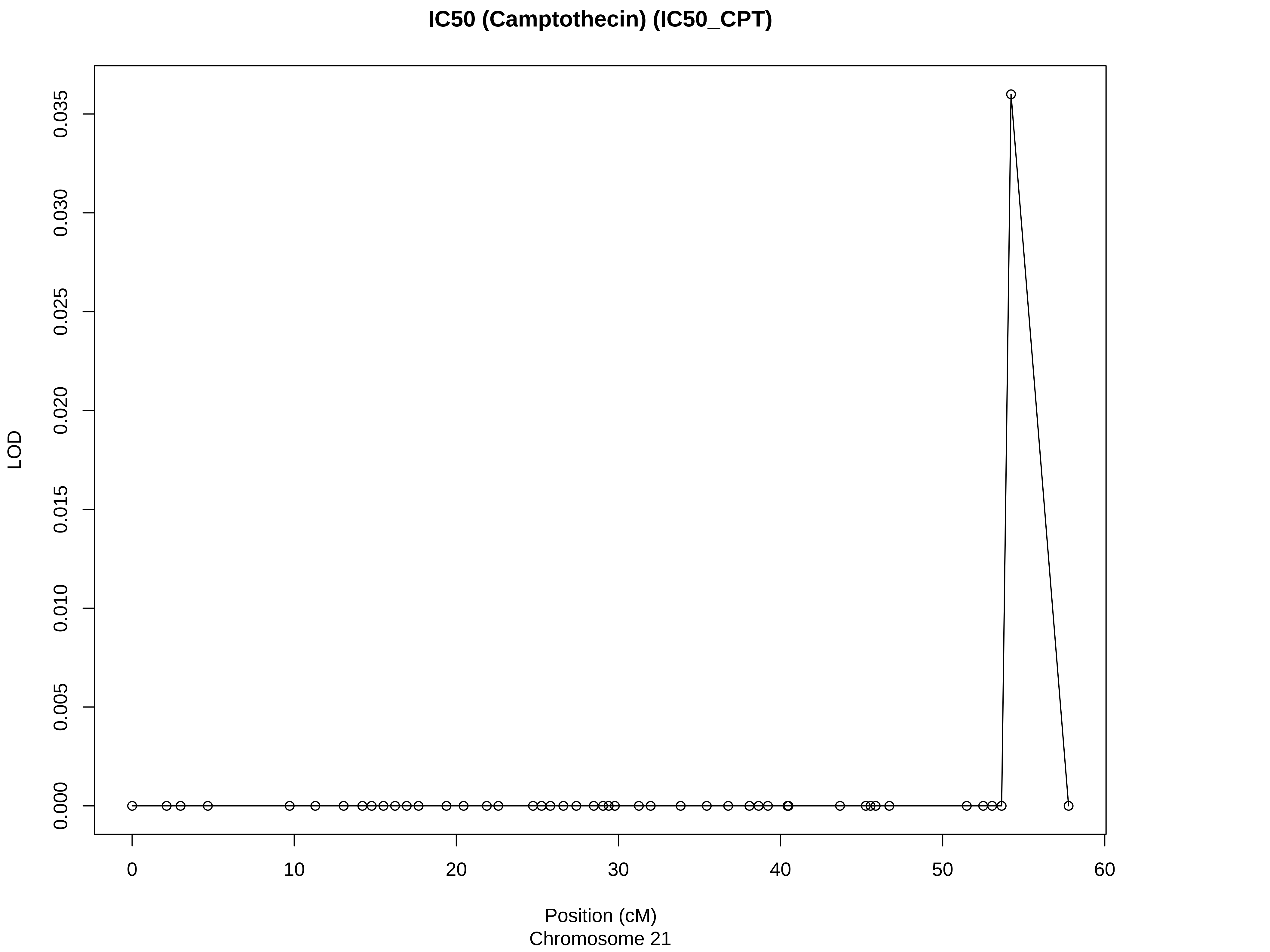
IC50 (Camptothecin) (IC50_CPT)
LOD
0.000
0.005
0.010
0.015
0.020
0.025
0.030
0.035
0
10
20
30
40
50
60
Position (cM)
Chromosome 21

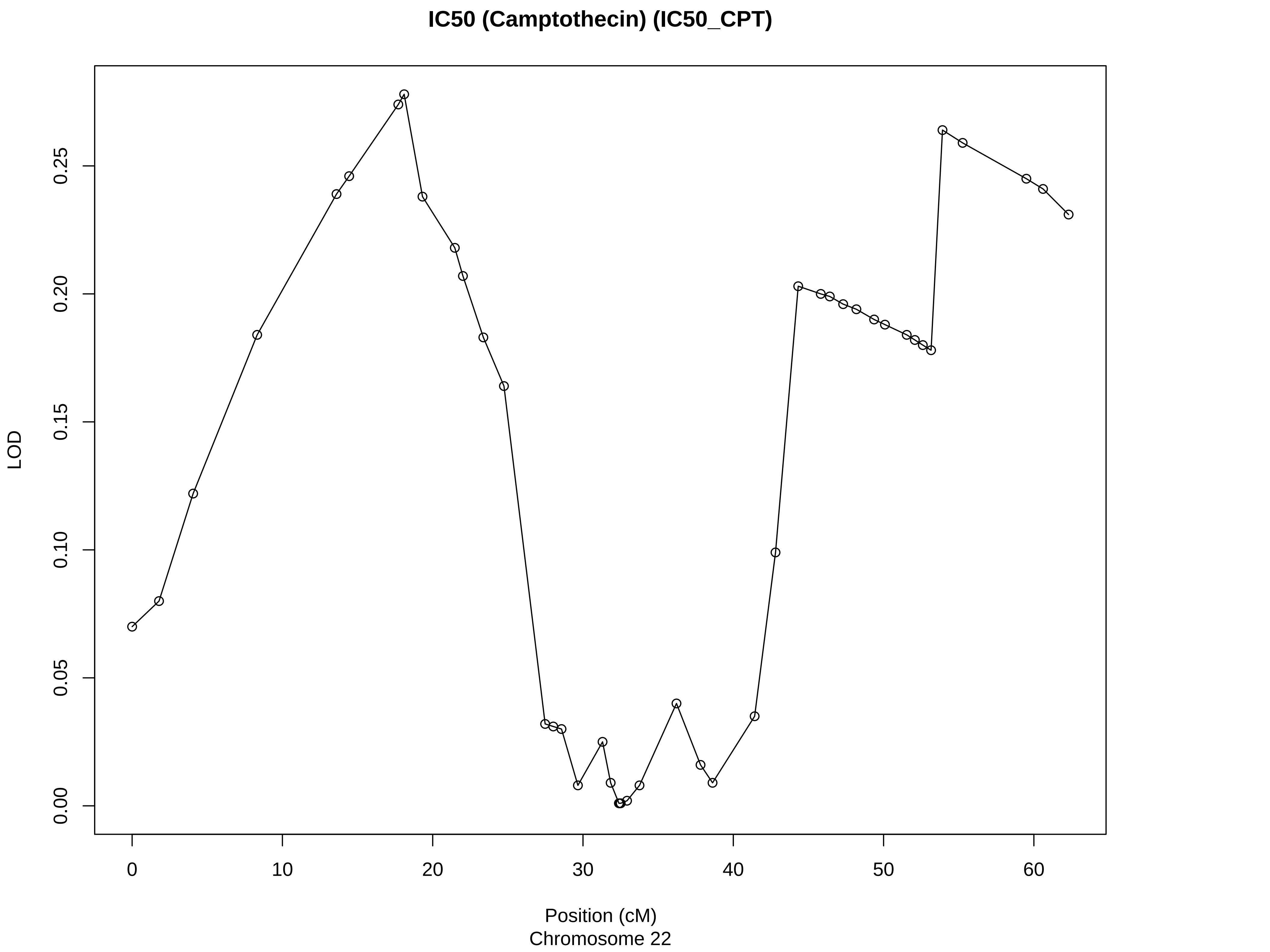
IC50 (Camptothecin) (IC50_CPT)
LOD
0.00
0.05
0.10
0.15
0.20
0.25
0
10
20
30
40
50
60
Position (cM)
Chromosome 22

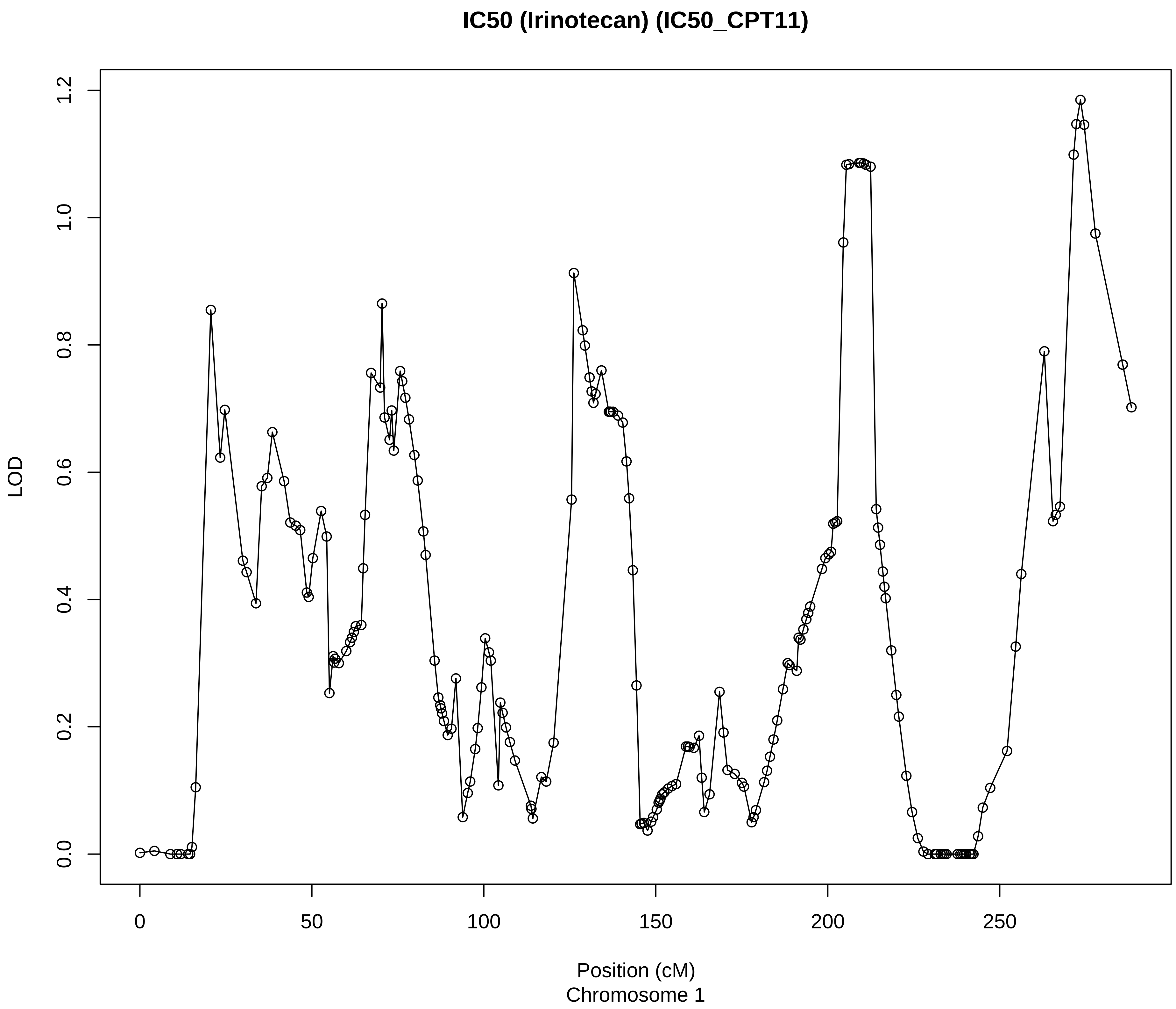
IC50 (Irinotecan) (IC50_CPT11)
LOD
0.0
0.2
0.4
0.6
0.8
1.0
1.2
0
50
100
150
200
250
Position (cM)
Chromosome 1

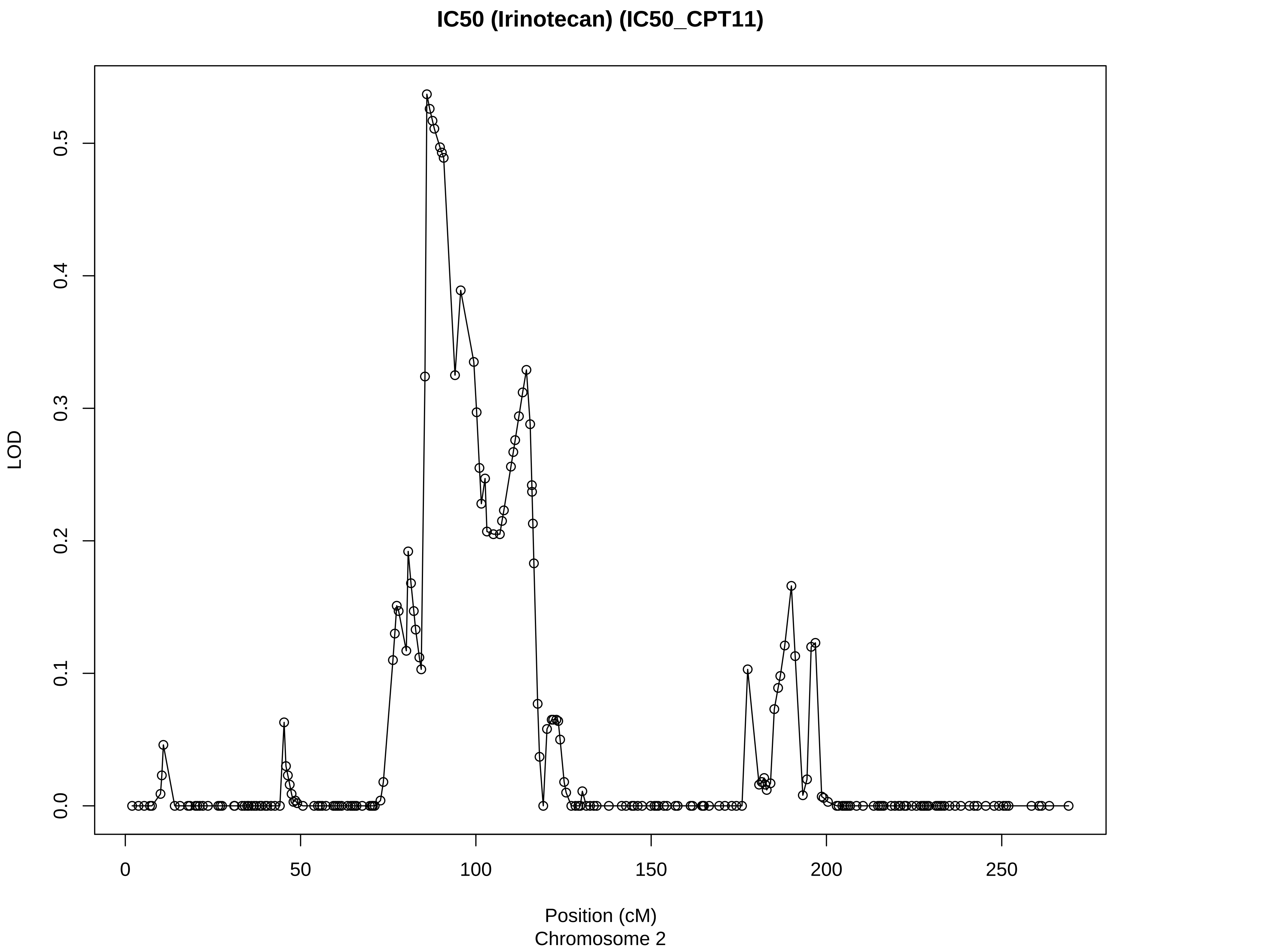

IC50 (Irinotecan) (IC50_CPT11)
LOD
0.0
0.1
0.2
0.3
0.4
0.5
0
50
100
150
200
250
Position (cM)
Chromosome 2

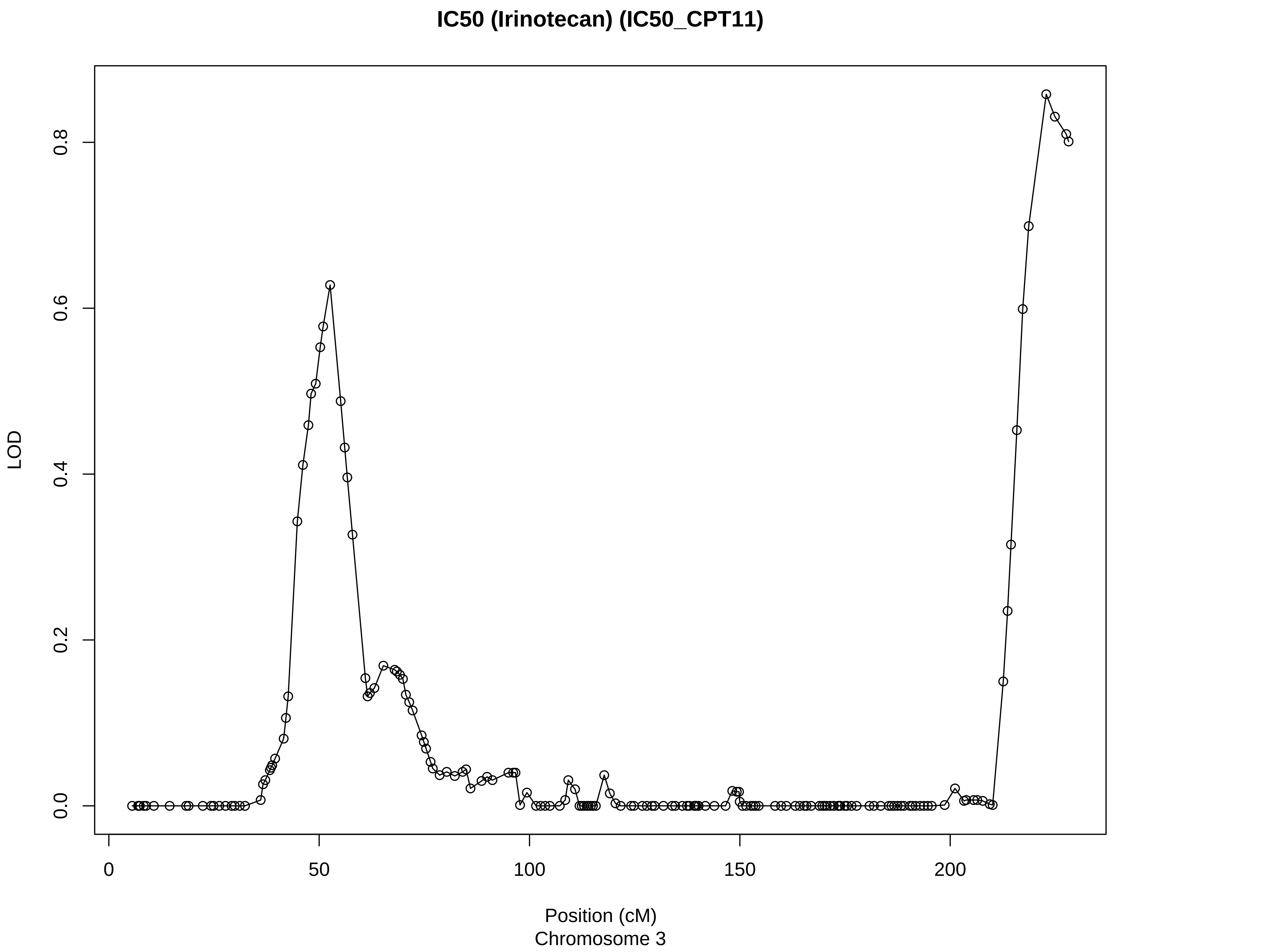
IC50 (Irinotecan) (IC50_CPT11)
LOD
0.0
0.2
0.4
0.6
0.8
0
50
100
150
200
Position (cM)
Chromosome 3

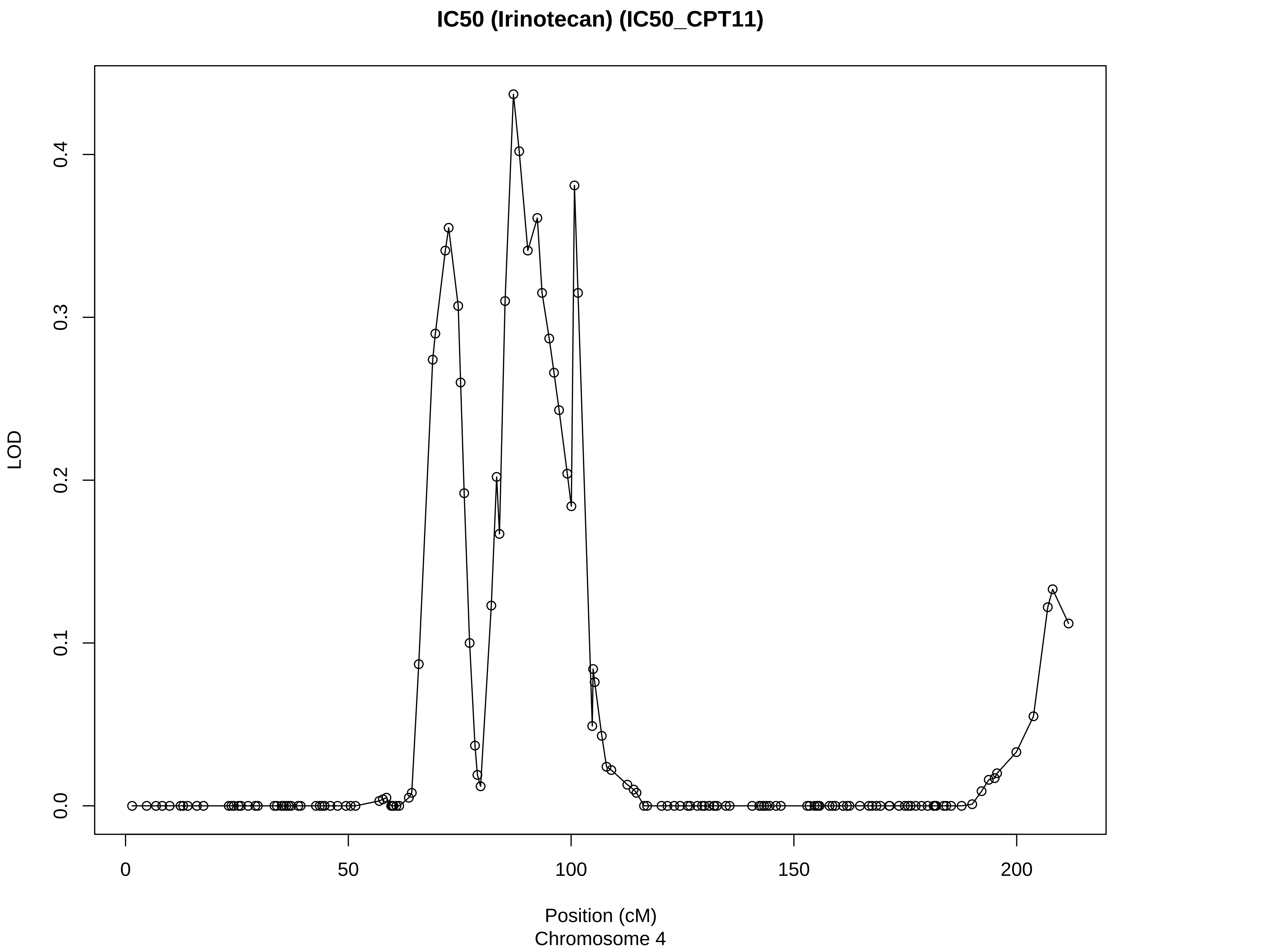
IC50 (Irinotecan) (IC50_CPT11)
LOD
0.0
0.1
0.2
0.3
0.4
0
50
100
150
200
Position (cM)
Chromosome 4

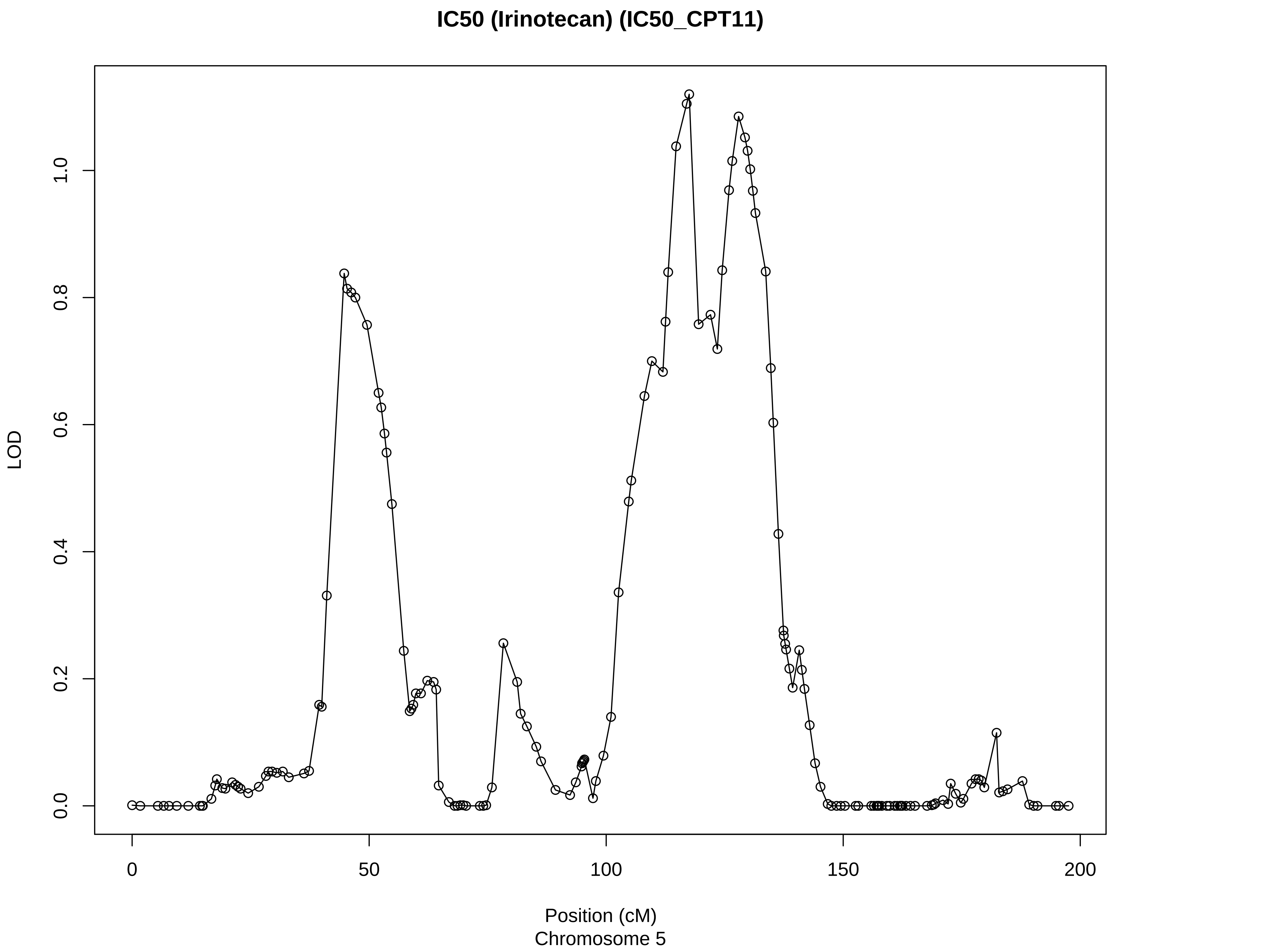
IC50 (Irinotecan) (IC50_CPT11)
LOD
0.0
0.2
0.4
0.6
0.8
1.0
0
50
100
150
200
Position (cM)
Chromosome 5

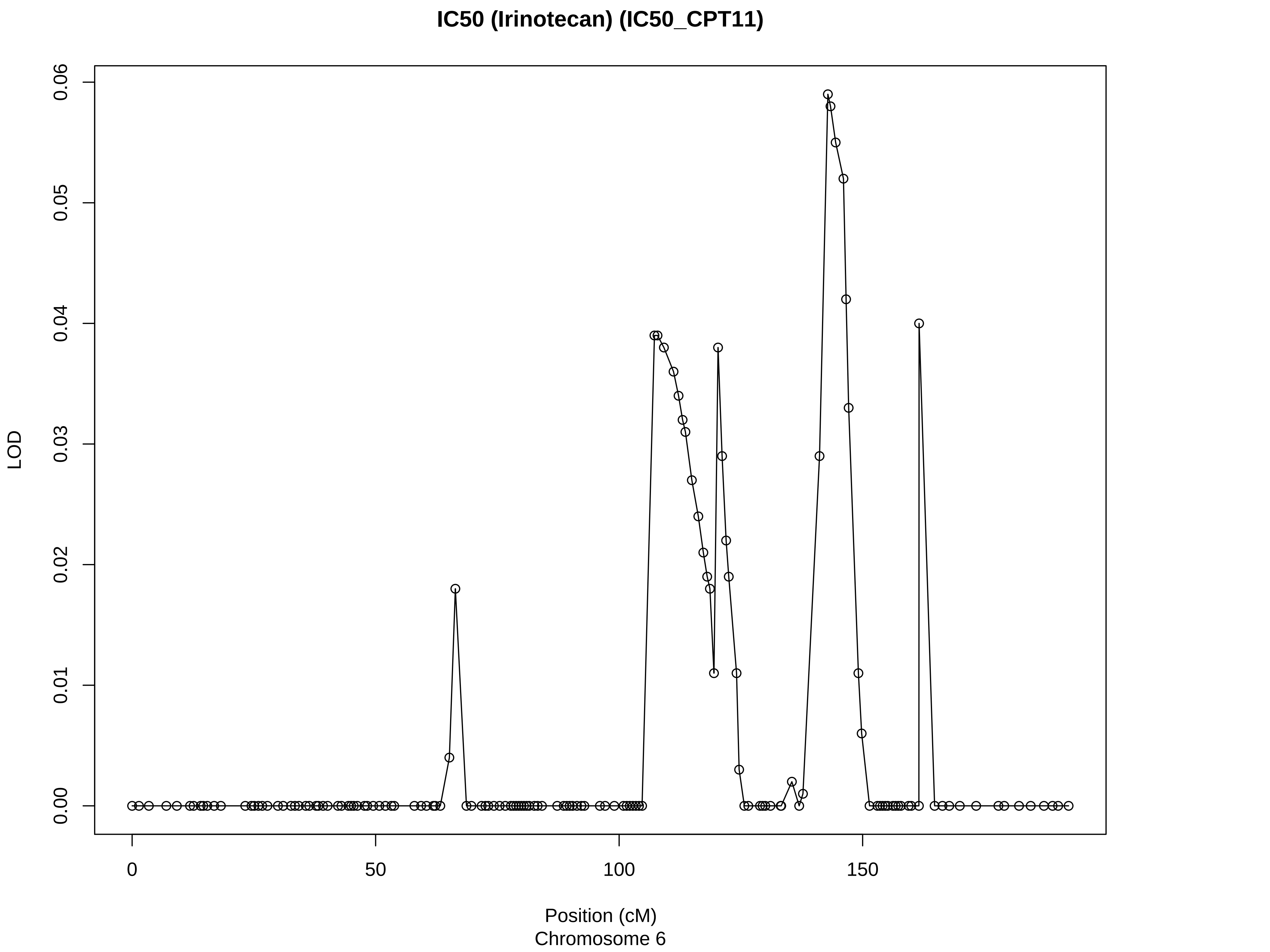
IC50 (Irinotecan) (IC50_CPT11)
LOD
0.00
0.01
0.02
0.03
0.04
0.05
0.06
0
50
100
150
Position (cM)
Chromosome 6

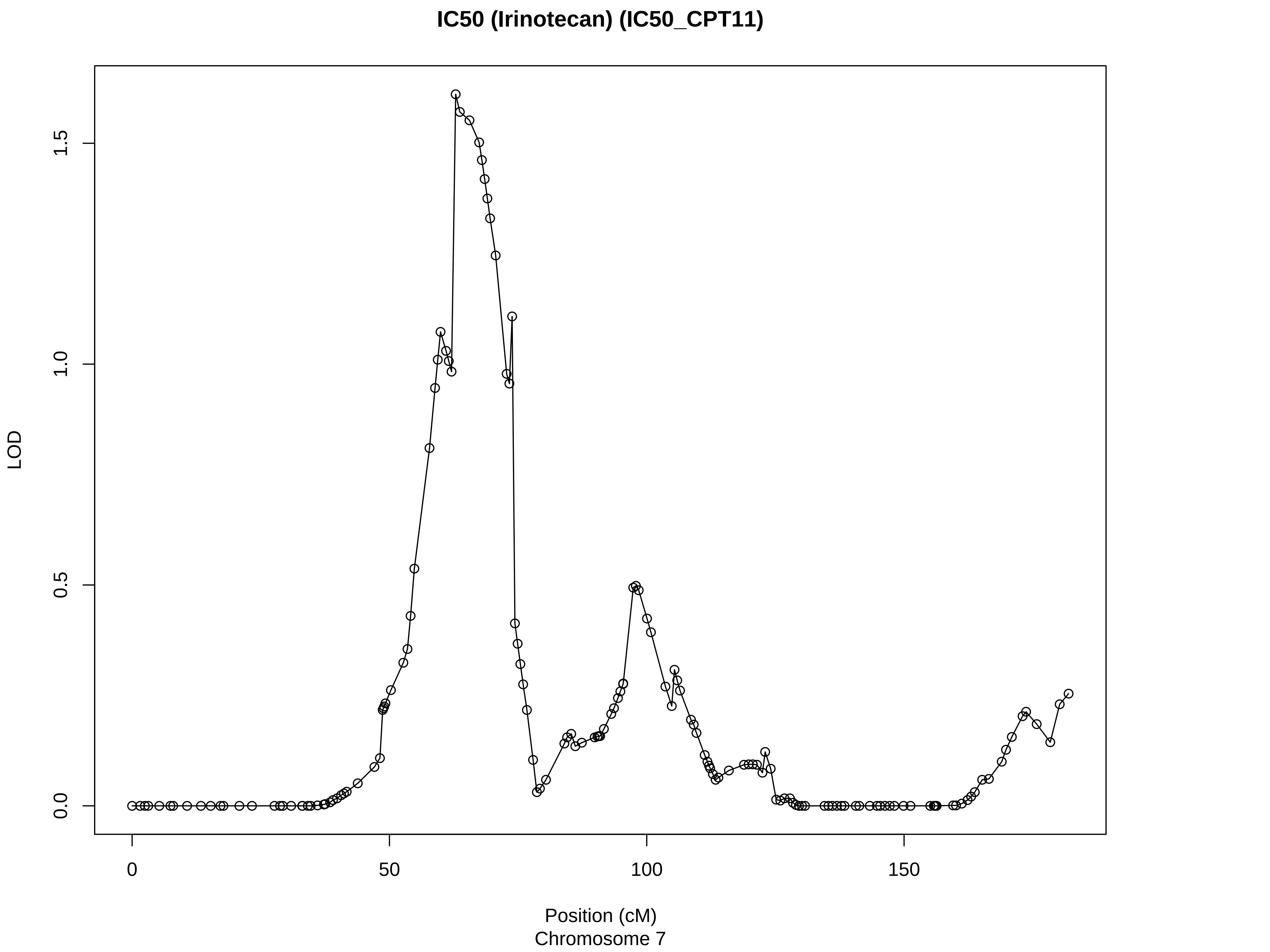
IC50 (Irinotecan) (IC50_CPT11)
LOD
0.0
0.5
1.0
1.5
0
50
100
150
Position (cM)
Chromosome 7

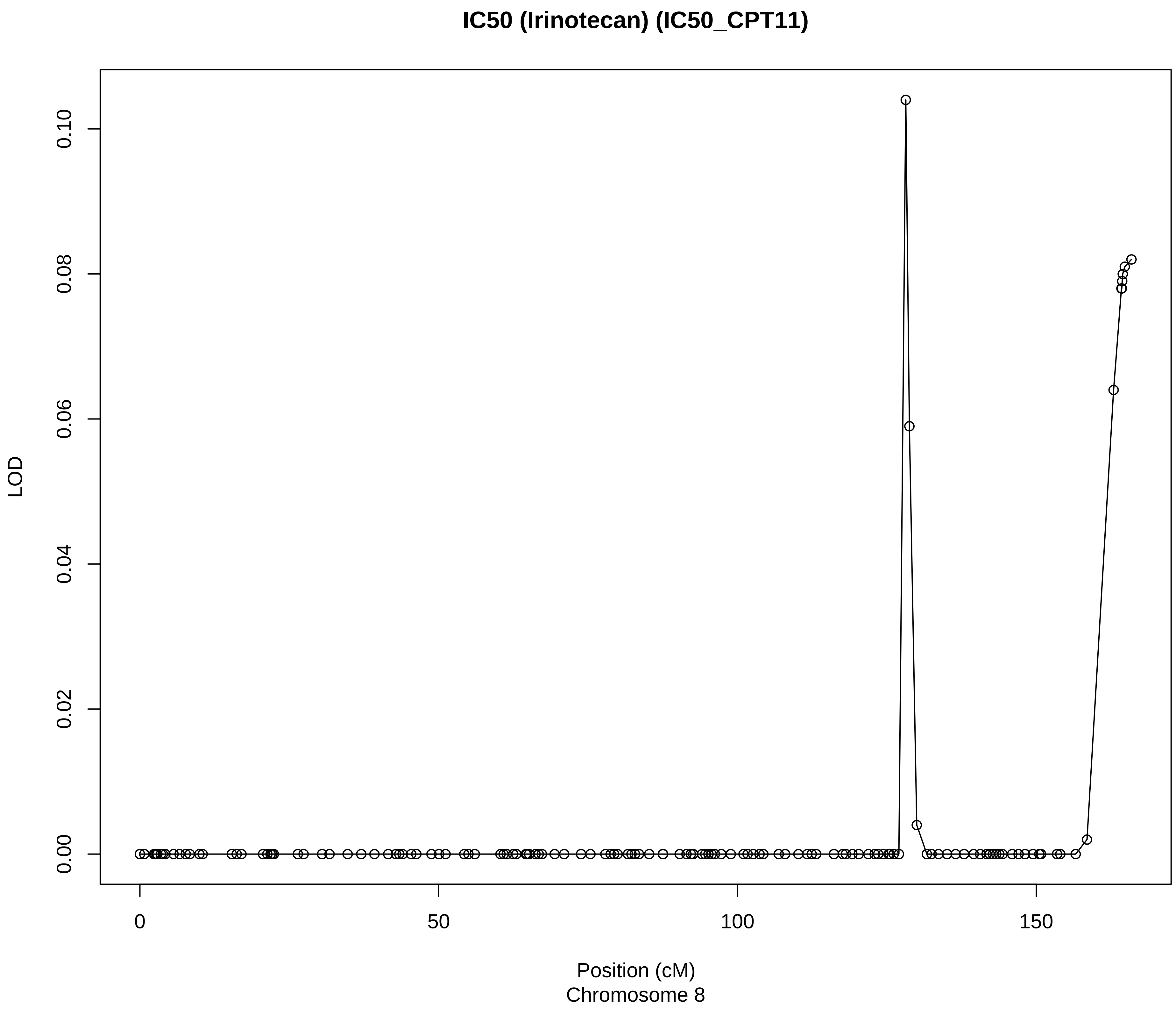
IC50 (Irinotecan) (IC50_CPT11)
LOD
0.00
0.02
0.04
0.06
0.08
0.10
0
50
100
150
Position (cM)
Chromosome 8

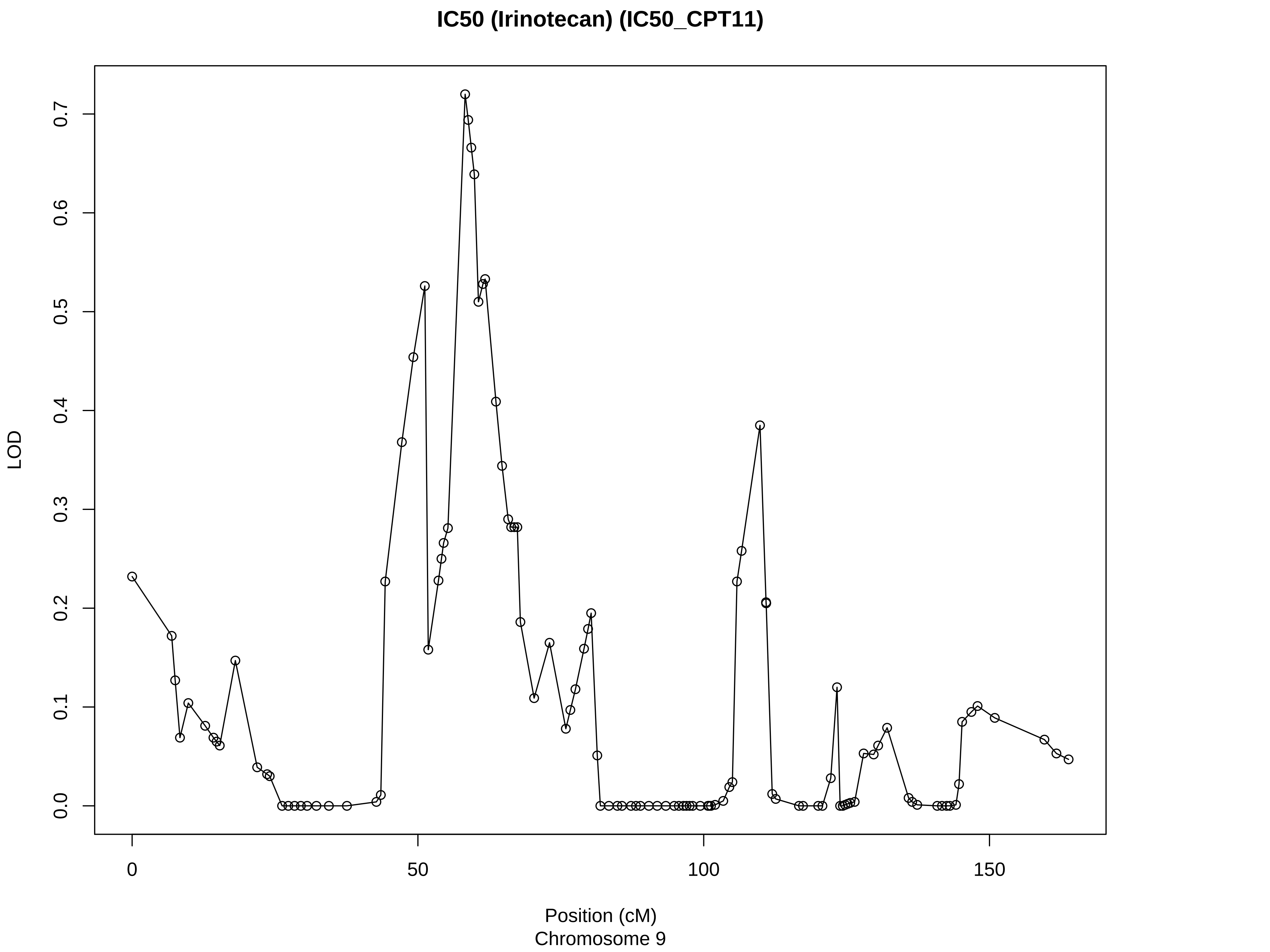
IC50 (Irinotecan) (IC50_CPT11)
LOD
0.0
0.1
0.2
0.3
0.4
0.5
0.6
0.7
0
50
100
150
Position (cM)
Chromosome 9

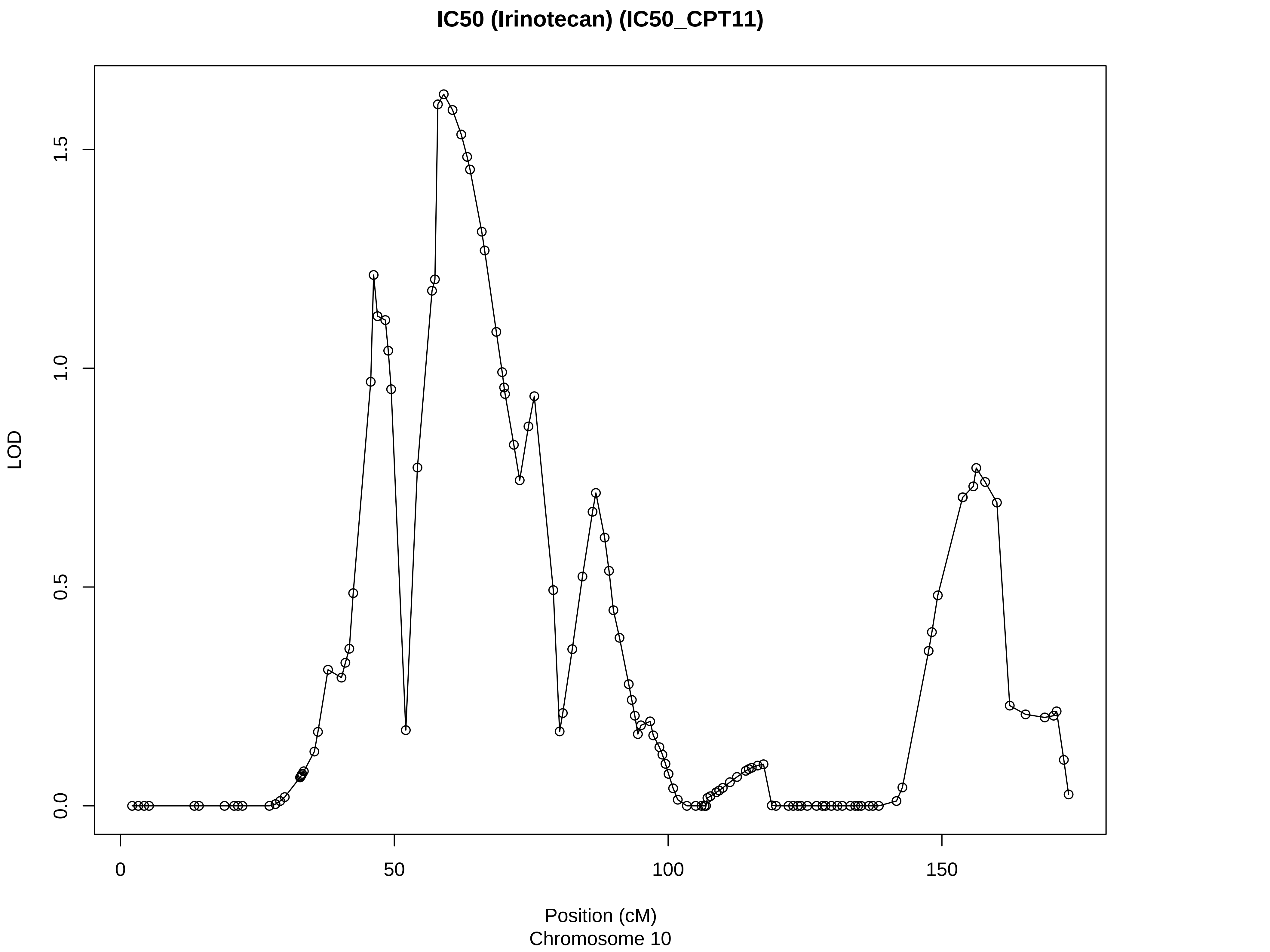

IC50 (Irinotecan) (IC50_CPT11)
LOD
0.0
0.5
1.0
1.5
0
50
100
150
Position (cM)
Chromosome 10

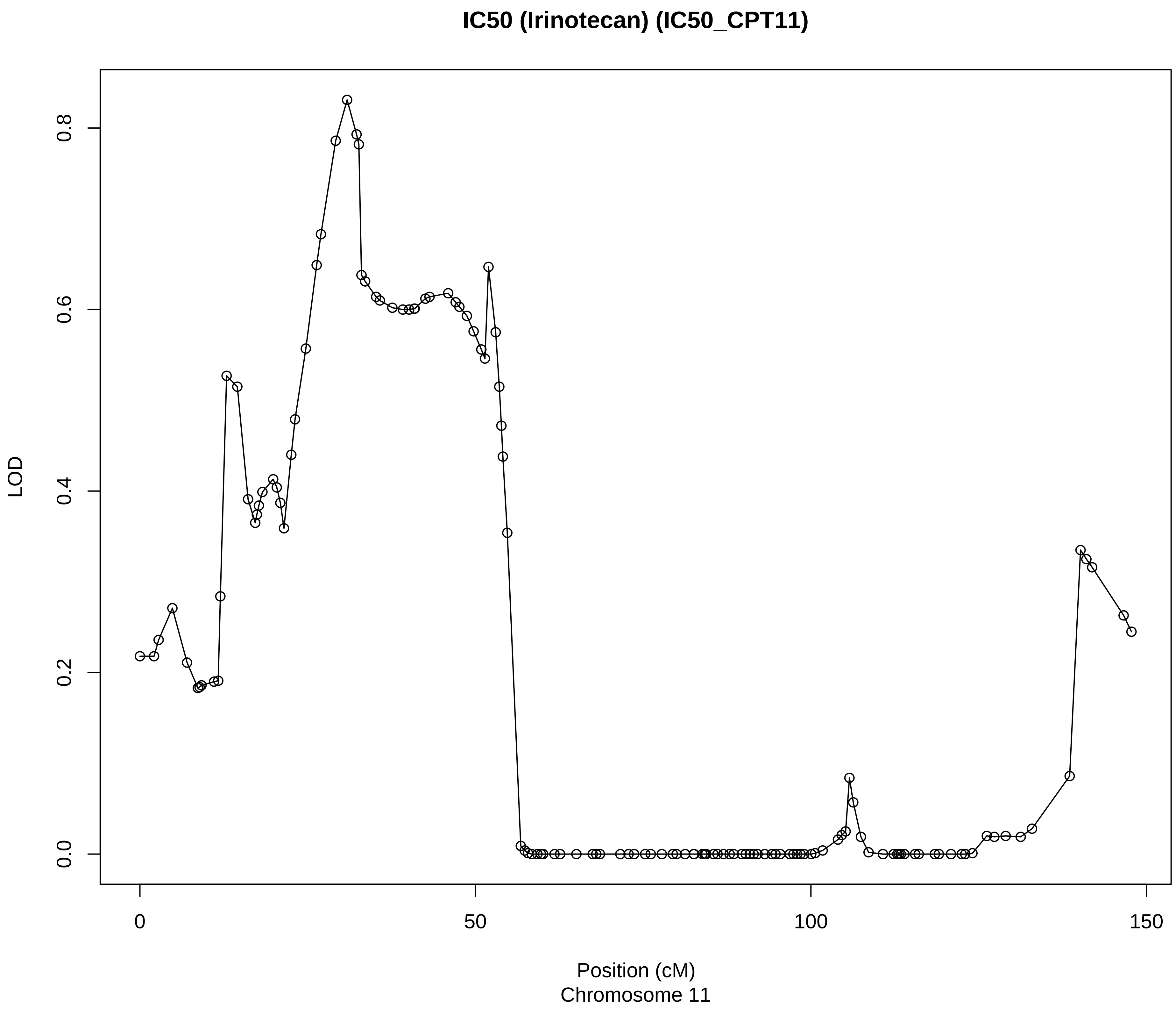

IC50 (Irinotecan) (IC50_CPT11)
LOD
0.0
0.2
0.4
0.6
0.8
0
50
100
150
Position (cM)
Chromosome 11

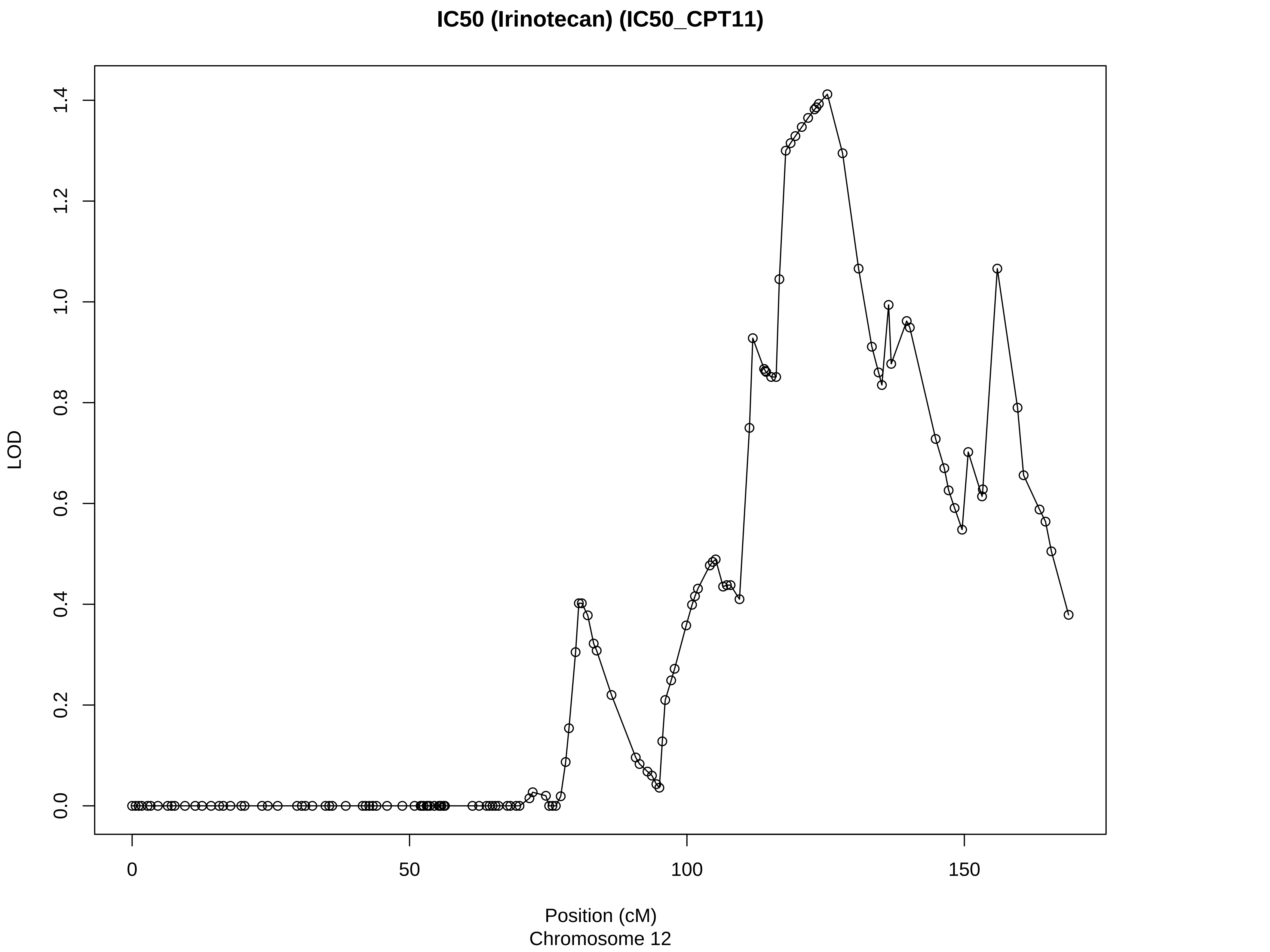

IC50 (Irinotecan) (IC50_CPT11)
LOD
0.0
0.2
0.4
0.6
0.8
1.0
1.2
1.4
0
50
100
150
Position (cM)
Chromosome 12

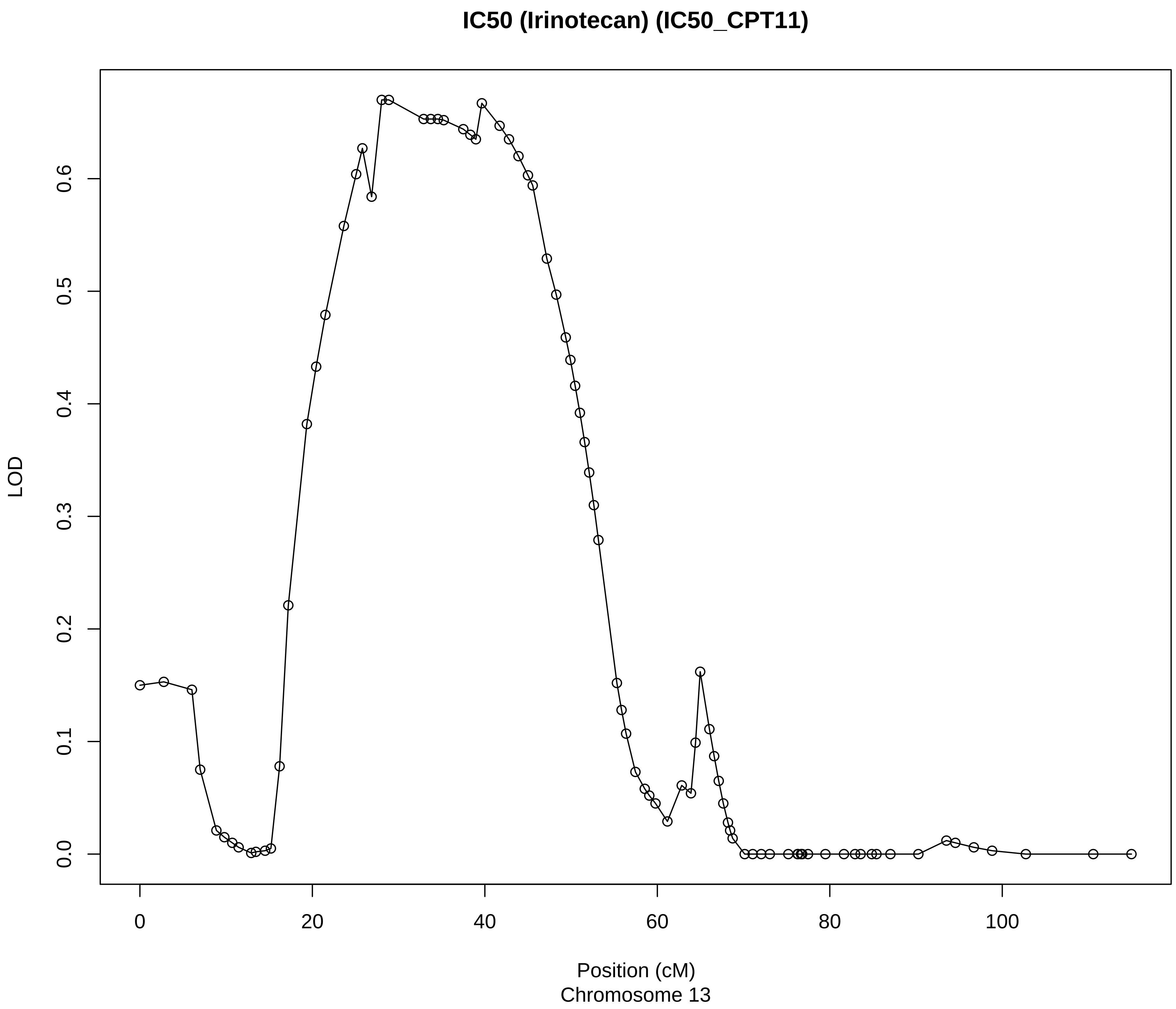

IC50 (Irinotecan) (IC50_CPT11)
LOD
0.0
0.1
0.2
0.3
0.4
0.5
0.6
0
20
40
60
80
100
Position (cM)
Chromosome 13

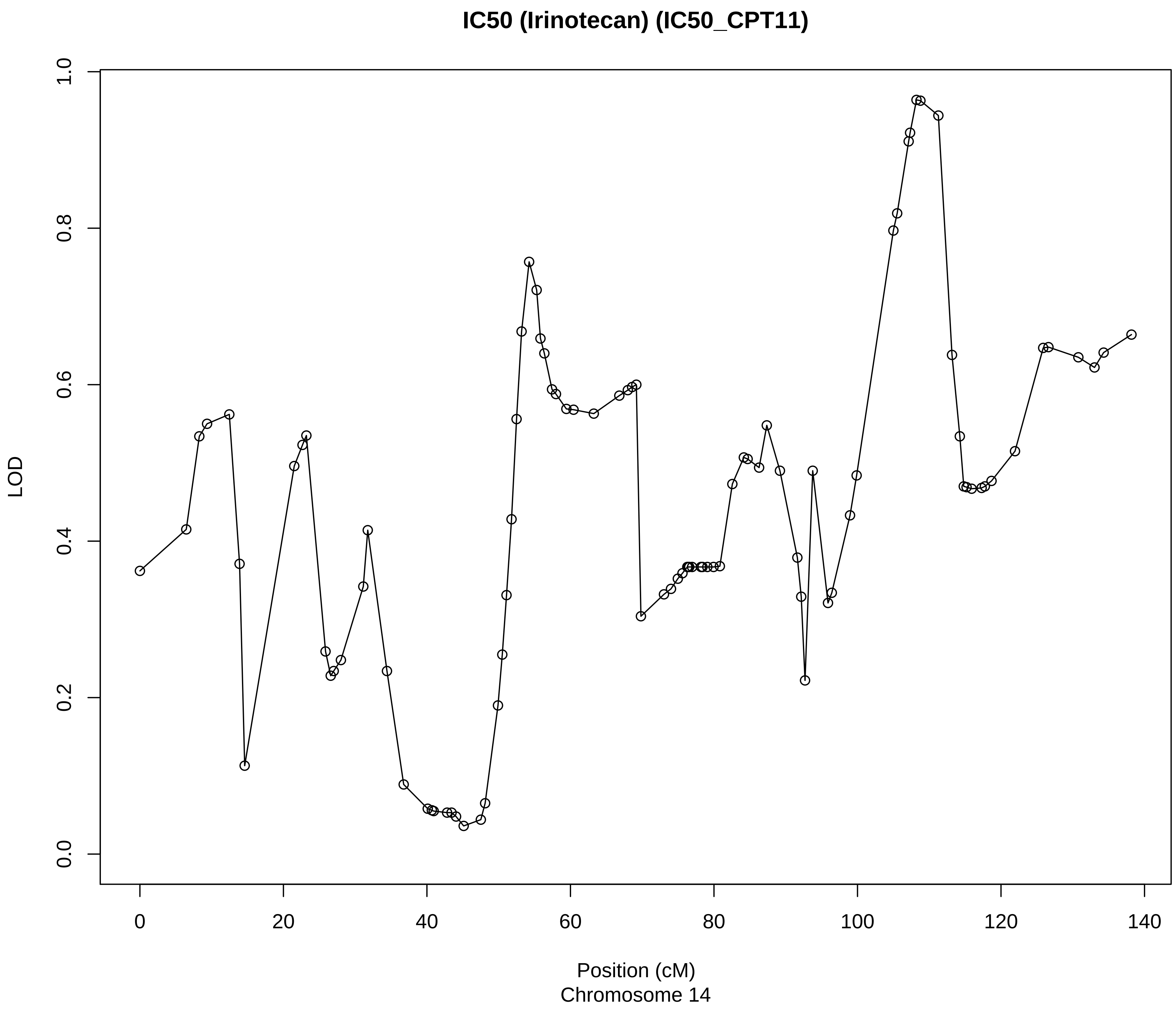
IC50 (Irinotecan) (IC50_CPT11)
LOD
0.0
0.2
0.4
0.6
0.8
1.0
0
20
40
60
80
100
120
140
Position (cM)
Chromosome 14

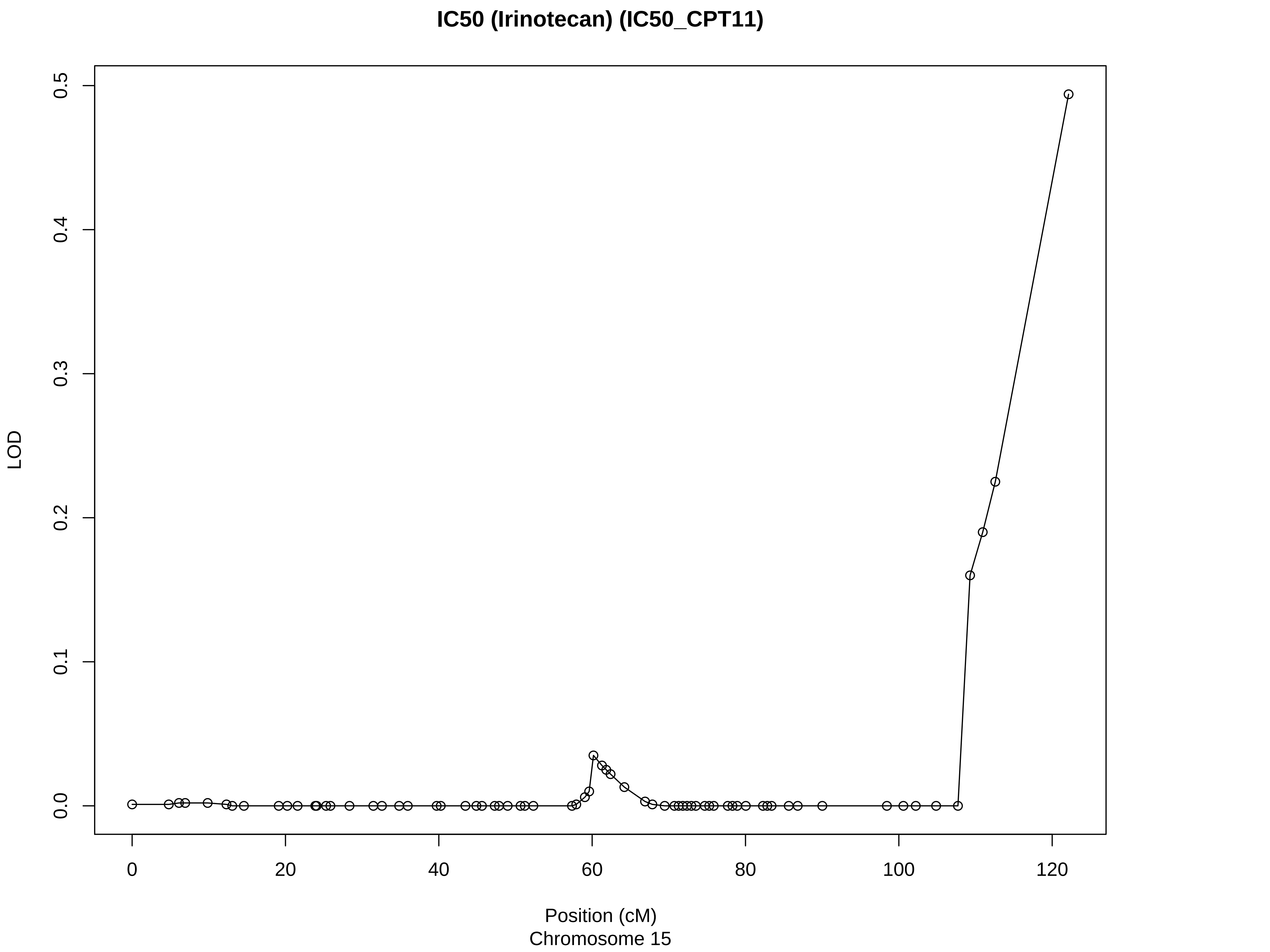
IC50 (Irinotecan) (IC50_CPT11)
LOD
0.0
0.1
0.2
0.3
0.4
0.5
0
20
40
60
80
100
120
Position (cM)
Chromosome 15

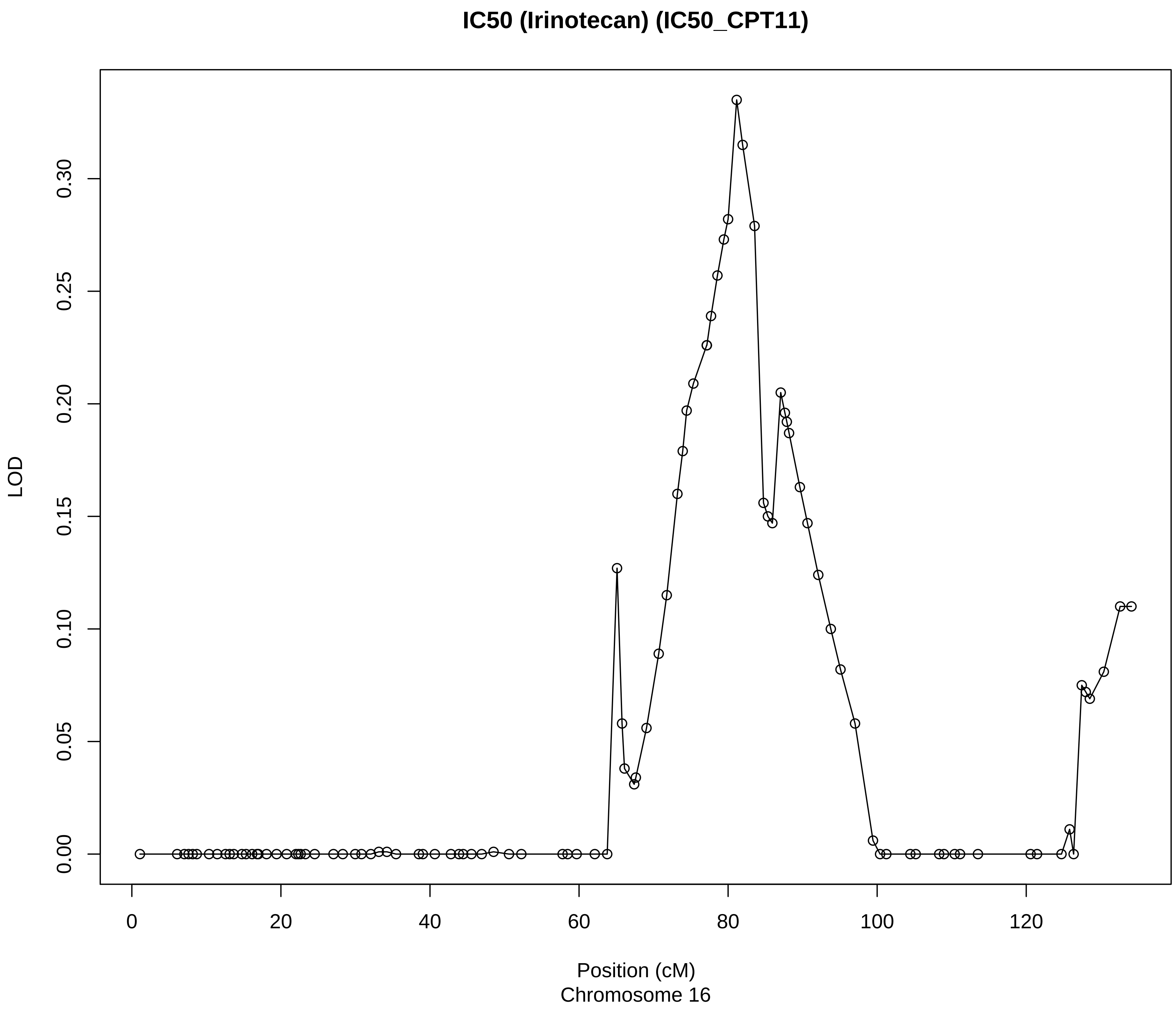

IC50 (Irinotecan) (IC50_CPT11)
LOD
0.00
0.05
0.10
0.15
0.20
0.25
0.30
0
20
40
60
80
100
120
Position (cM)
Chromosome 16

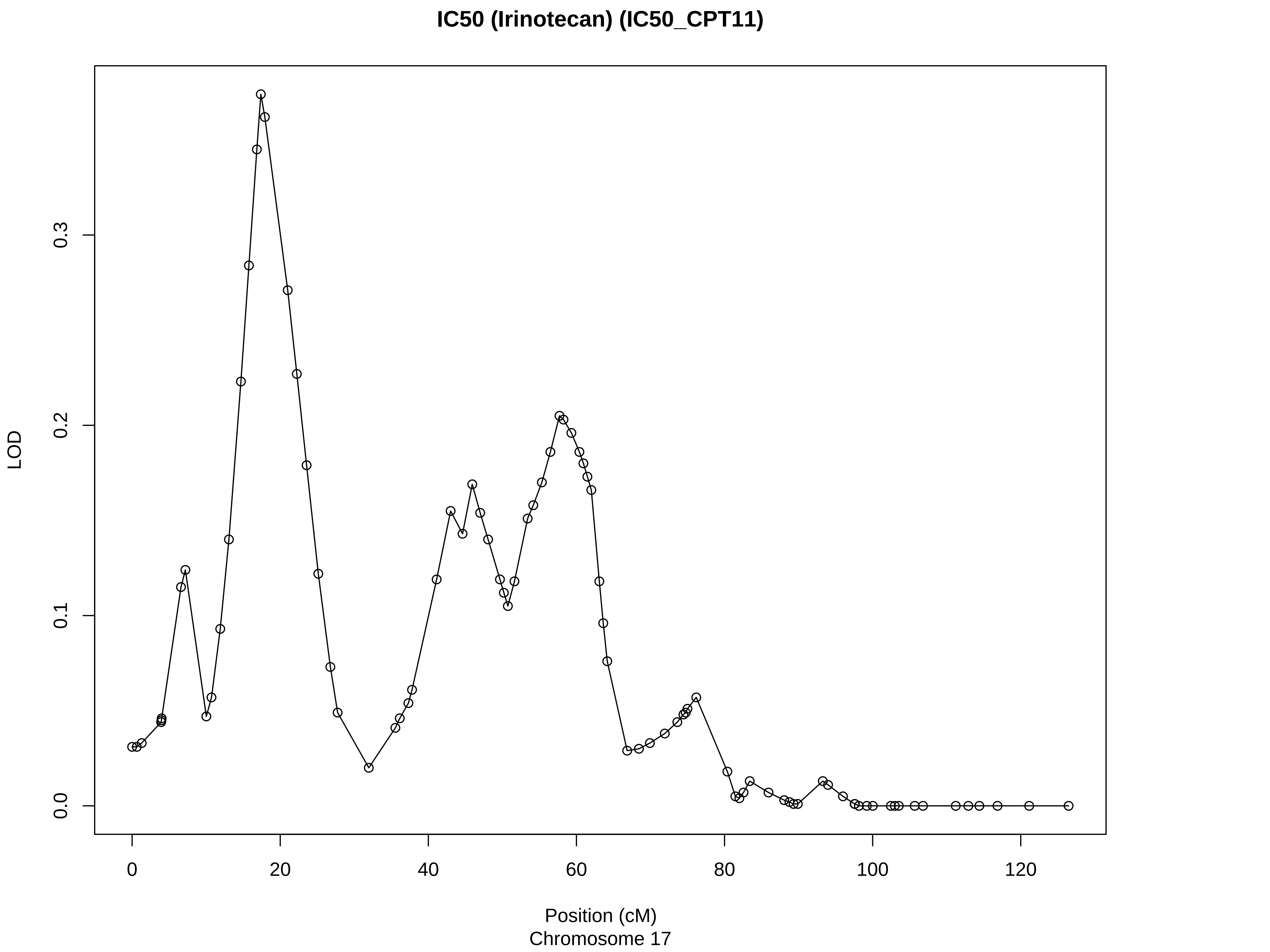
IC50 (Irinotecan) (IC50_CPT11)
LOD
0.0
0.1
0.2
0.3
0
20
40
60
80
100
120
Position (cM)
Chromosome 17

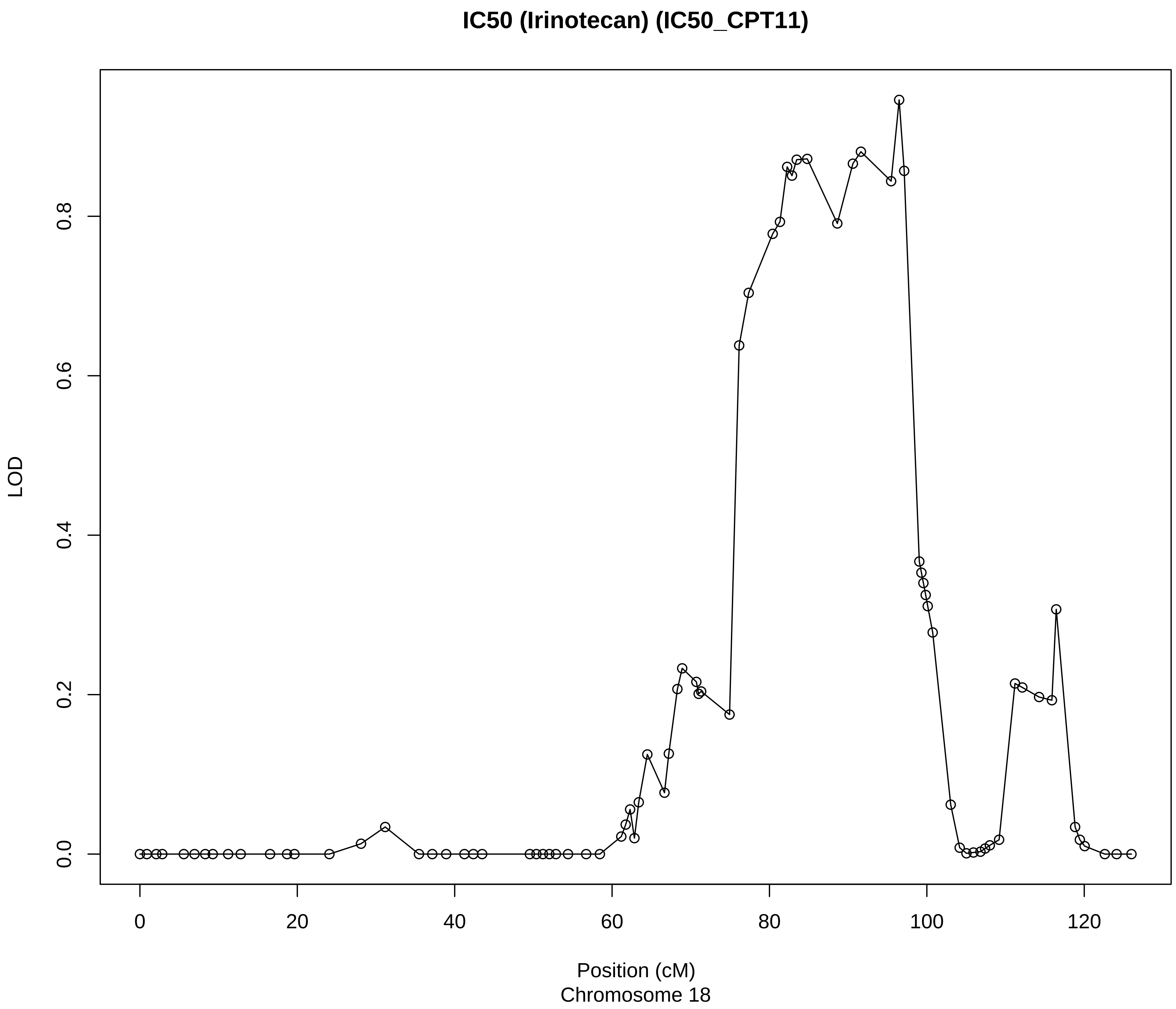

IC50 (Irinotecan) (IC50_CPT11)
LOD
0.0
0.2
0.4
0.6
0.8
0
20
40
60
80
100
120
Position (cM)
Chromosome 18

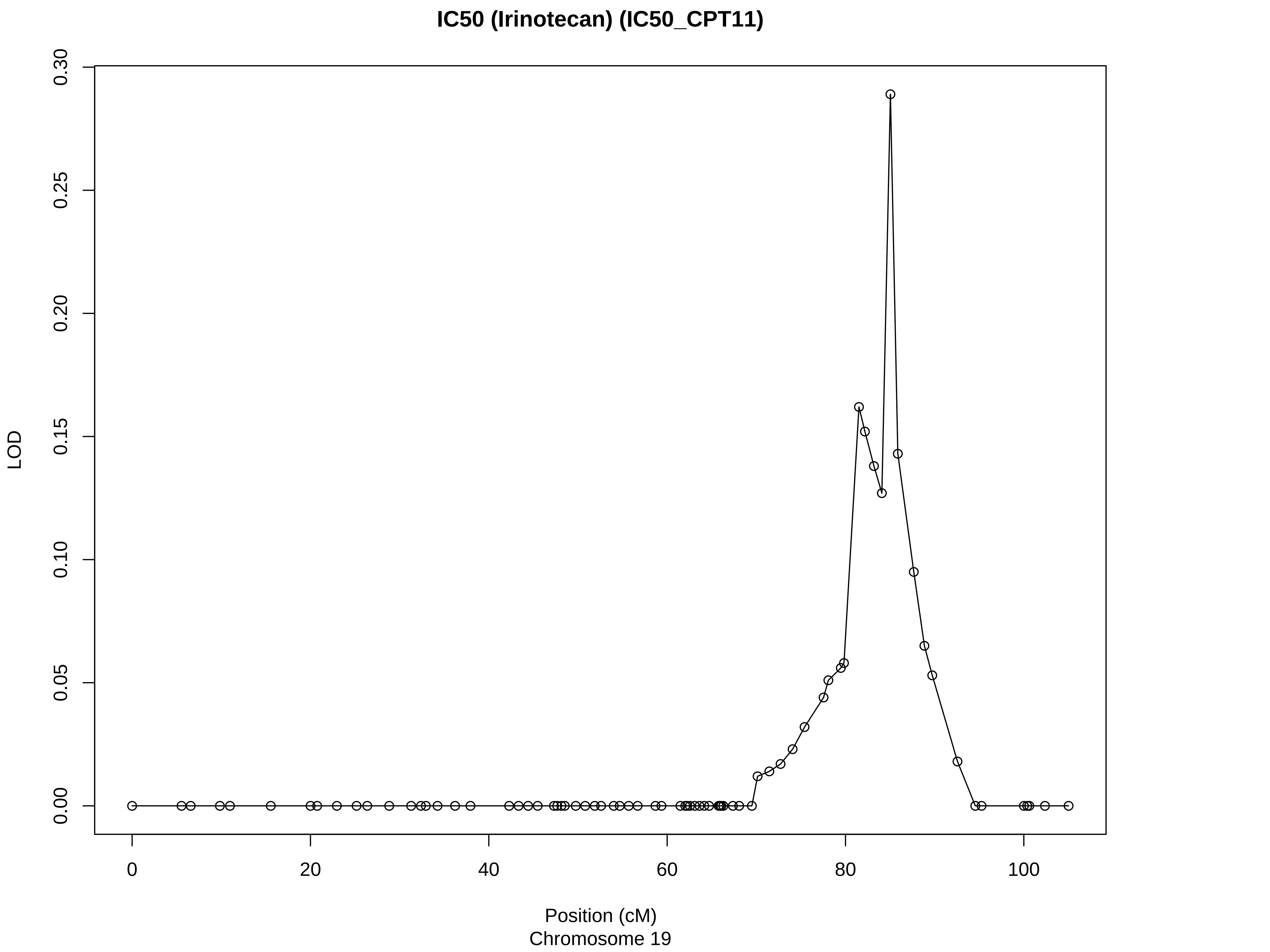

IC50 (Irinotecan) (IC50_CPT11)
LOD
0.00
0.05
0.10
0.15
0.20
0.25
0.30
0
20
40
60
80
100
Position (cM)
Chromosome 19

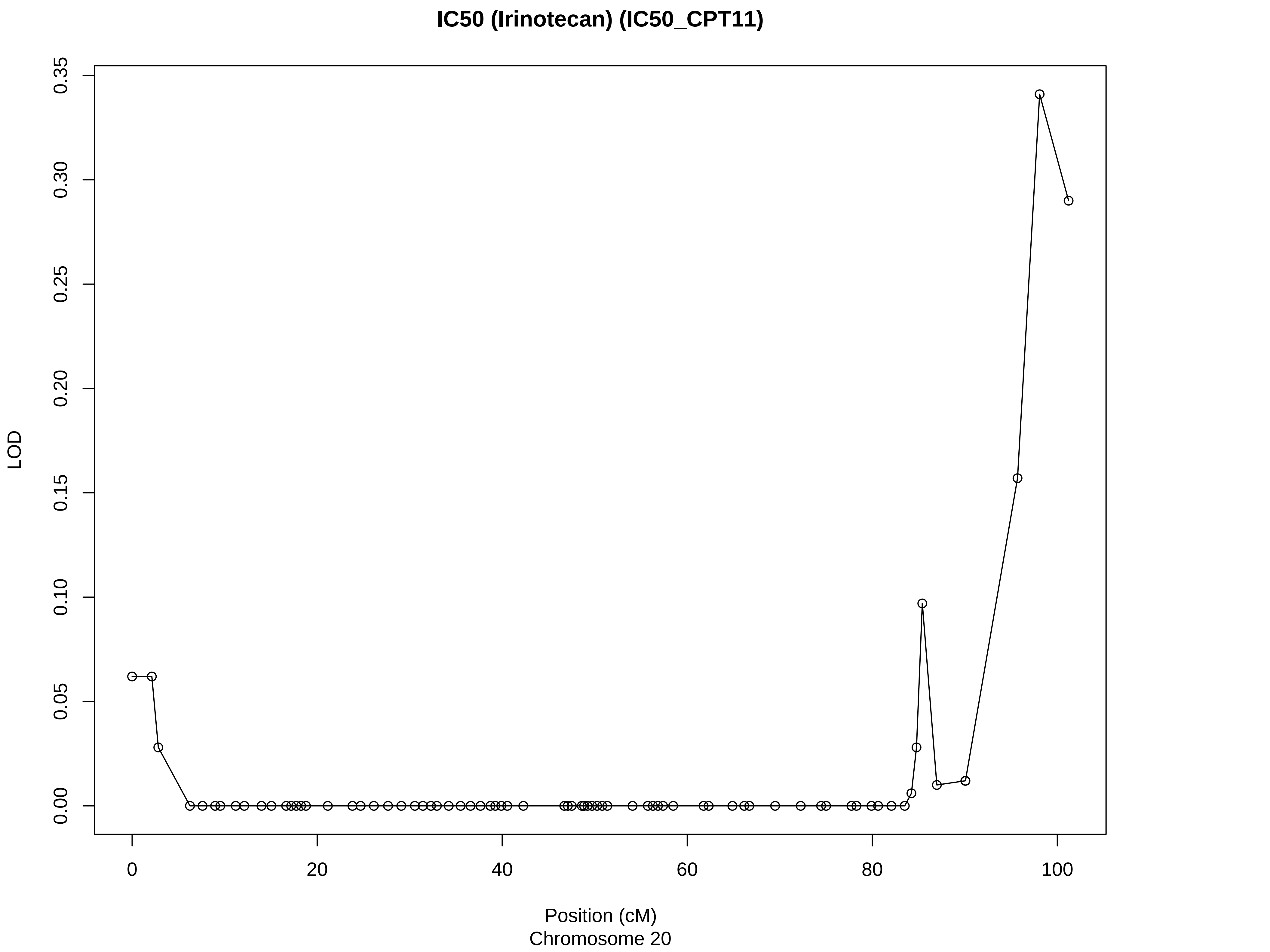
IC50 (Irinotecan) (IC50_CPT11)
LOD
0.00
0.05
0.10
0.15
0.20
0.25
0.30
0.35
0
20
40
60
80
100
Position (cM)
Chromosome 20

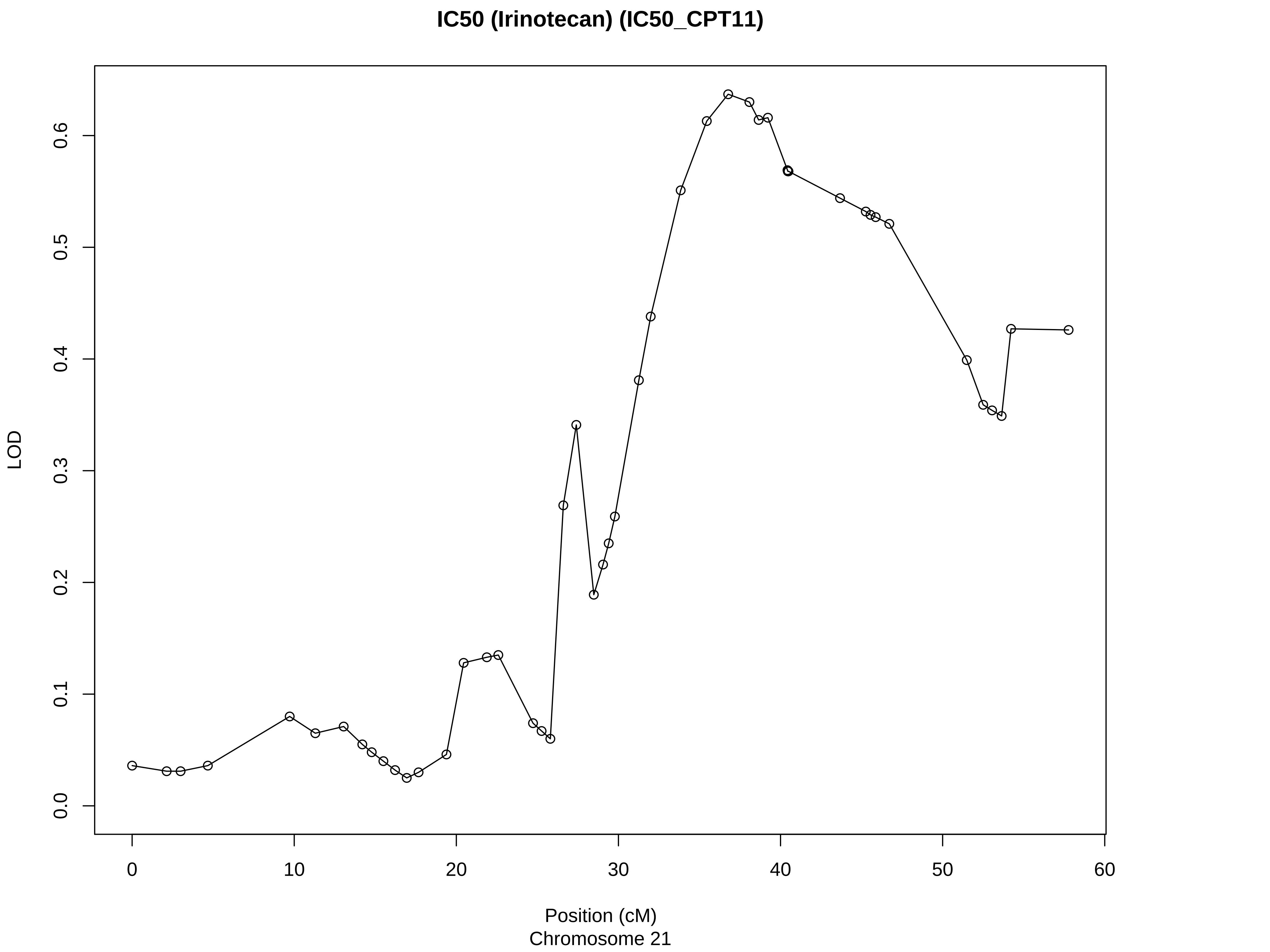

IC50 (Irinotecan) (IC50_CPT11)
LOD
0.0
0.1
0.2
0.3
0.4
0.5
0.6
0
10
20
30
40
50
60
Position (cM)
Chromosome 21

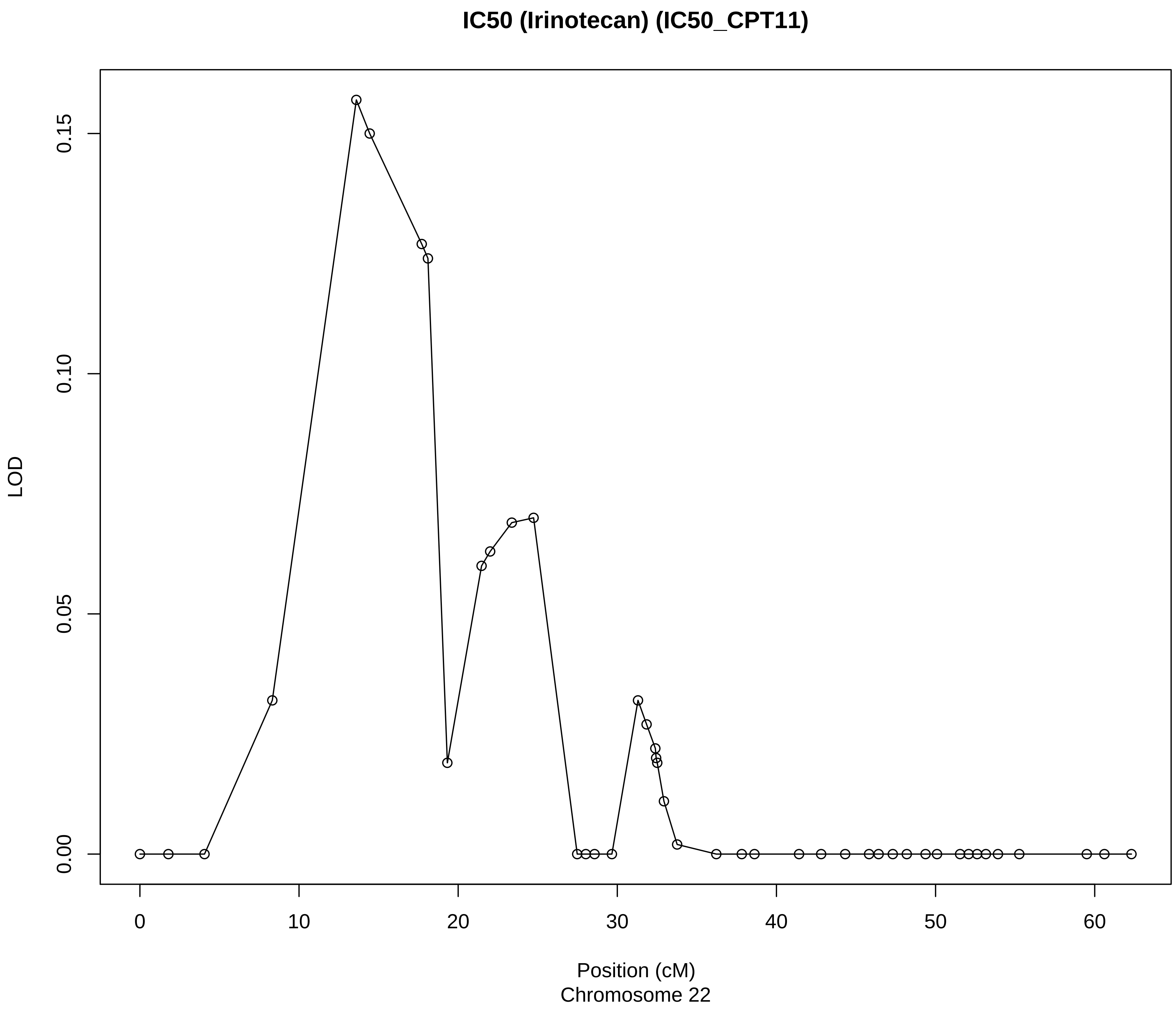
IC50 (Irinotecan) (IC50_CPT11)
LOD
0.00
0.05
0.10
0.15
0
10
20
30
40
50
60
Position (cM)
Chromosome 22

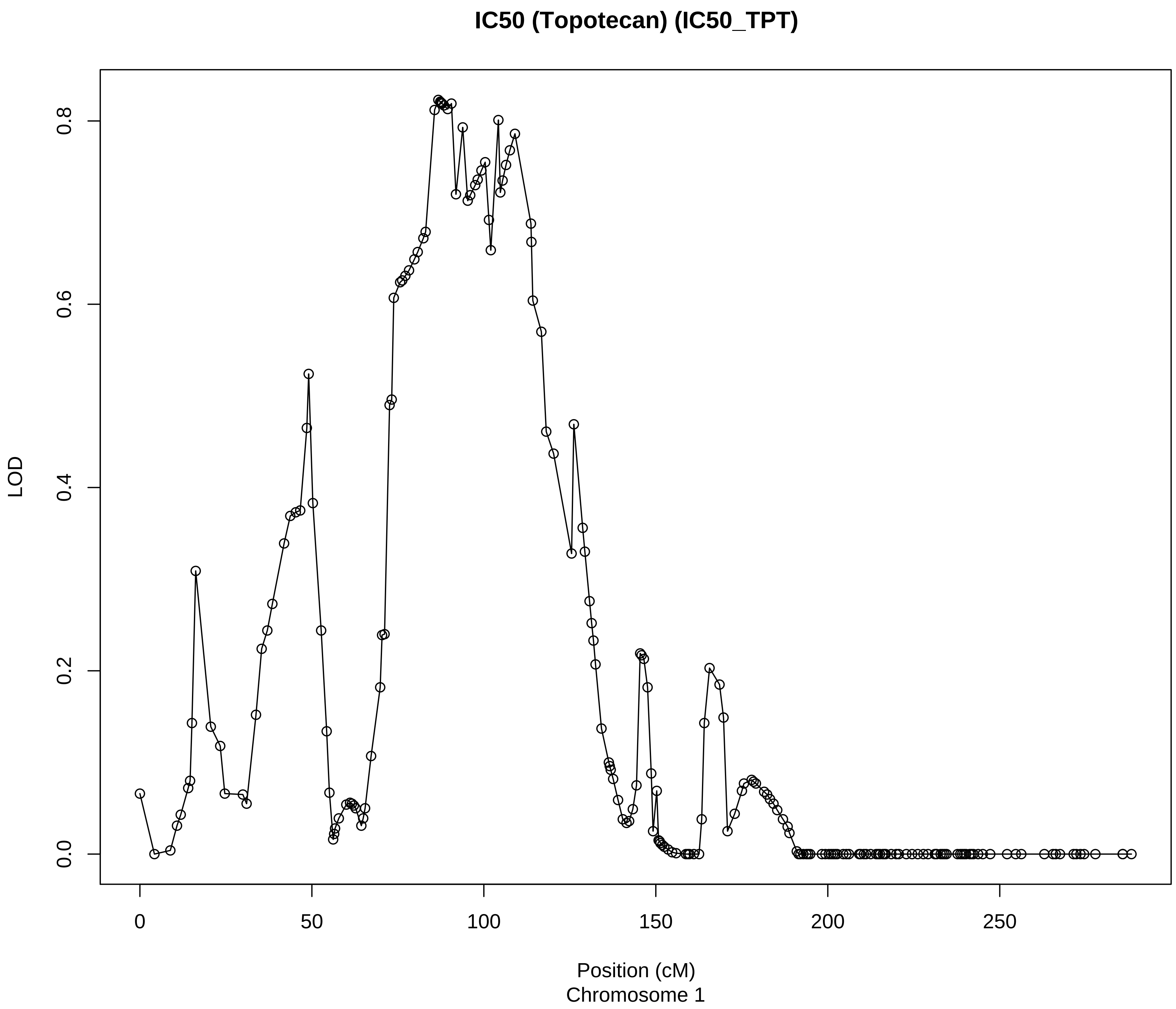

IC50 (Topotecan) (IC50_TPT)
LOD
0.0
0.2
0.4
0.6
0.8
0
50
100
150
200
250
Position (cM)
Chromosome 1

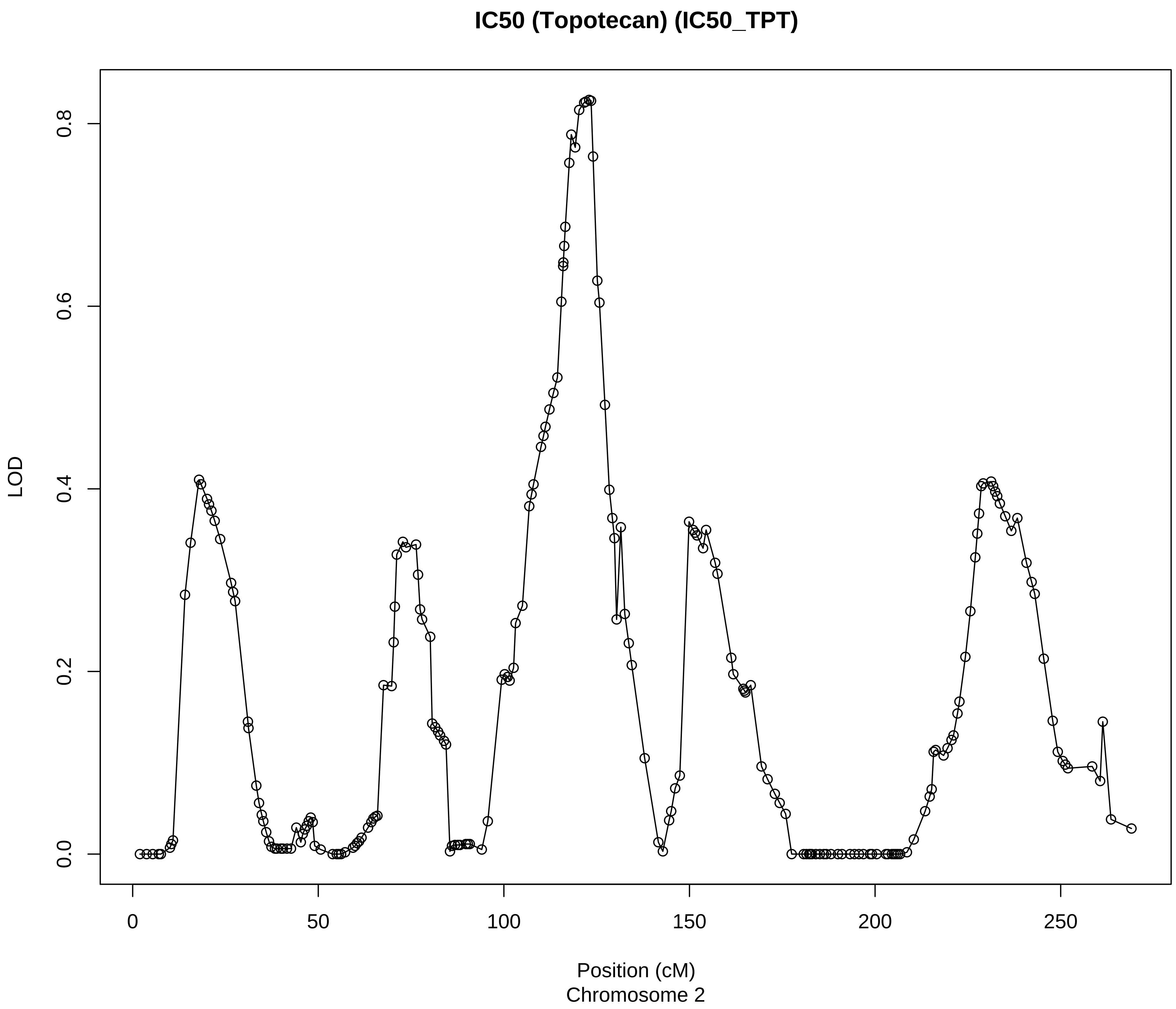
IC50 (Topotecan) (IC50_TPT)
LOD
0.0
0.2
0.4
0.6
0.8
0
50
100
150
200
250
Position (cM)
Chromosome 2

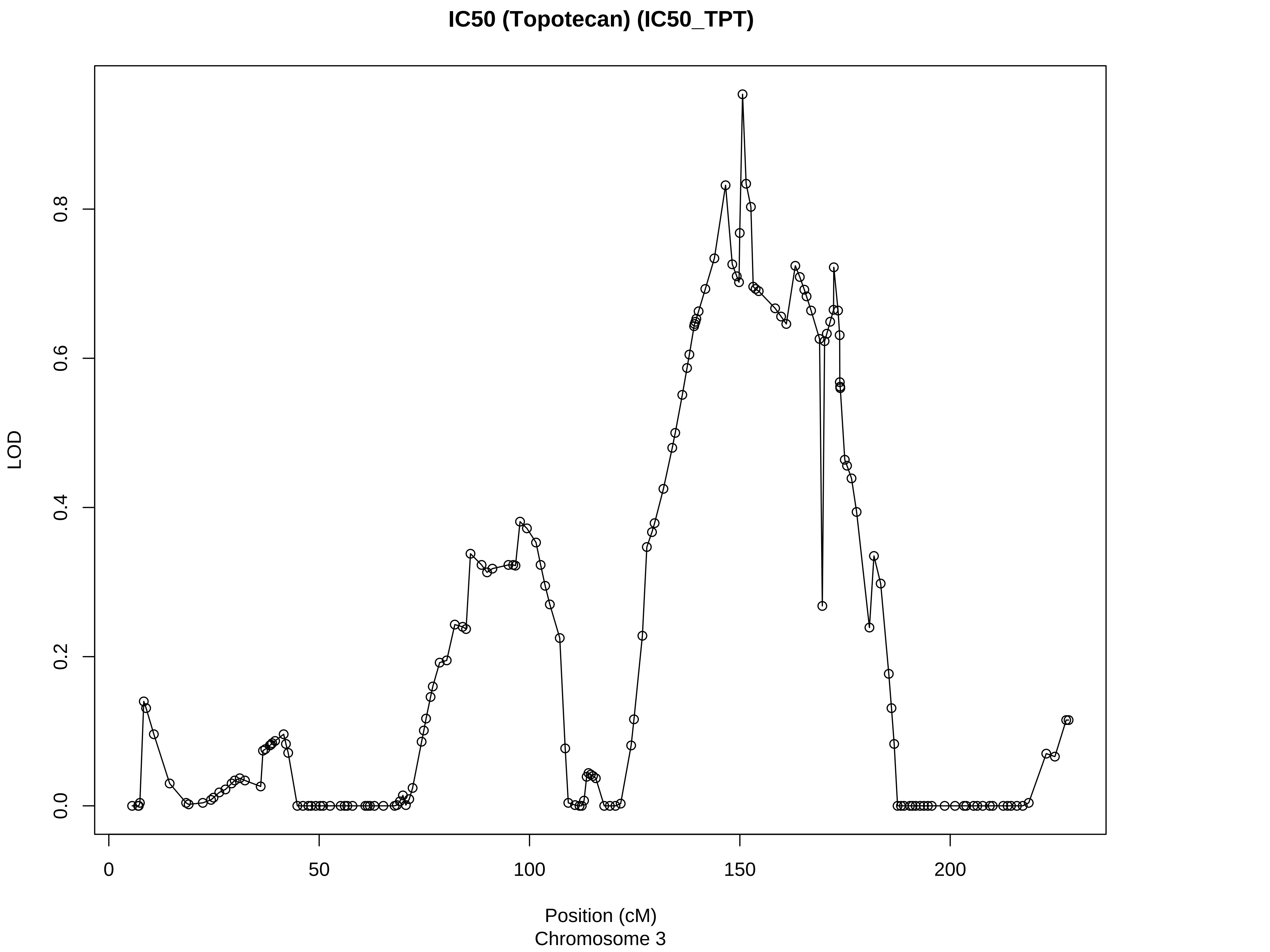
IC50 (Topotecan) (IC50_TPT)
LOD
0.0
0.2
0.4
0.6
0.8
0
50
100
150
200
Position (cM)
Chromosome 3

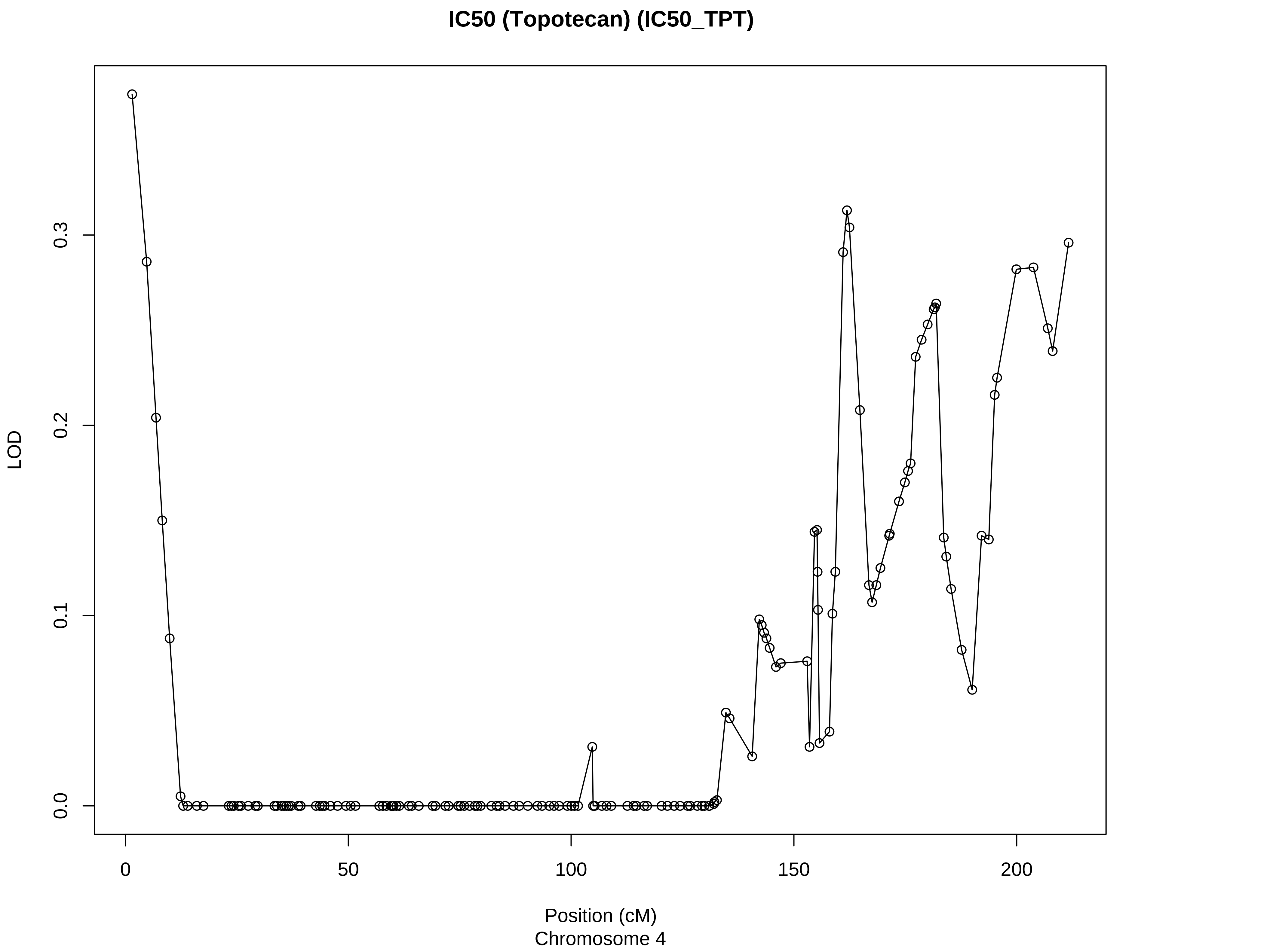

IC50 (Topotecan) (IC50_TPT)
LOD
0.0
0.1
0.2
0.3
0
50
100
150
200
Position (cM)
Chromosome 4

IC50 (Topotecan) (IC50_TPT)

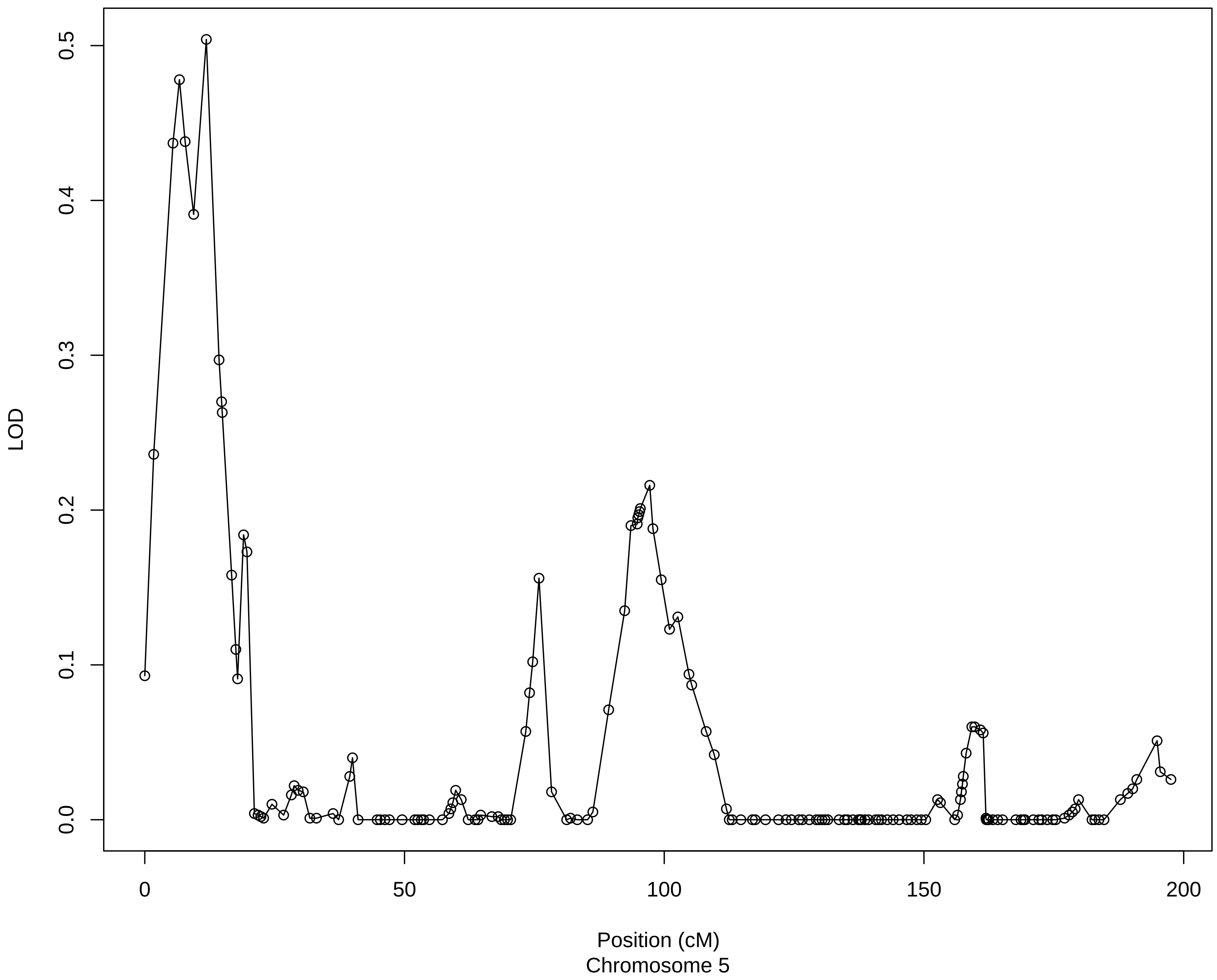

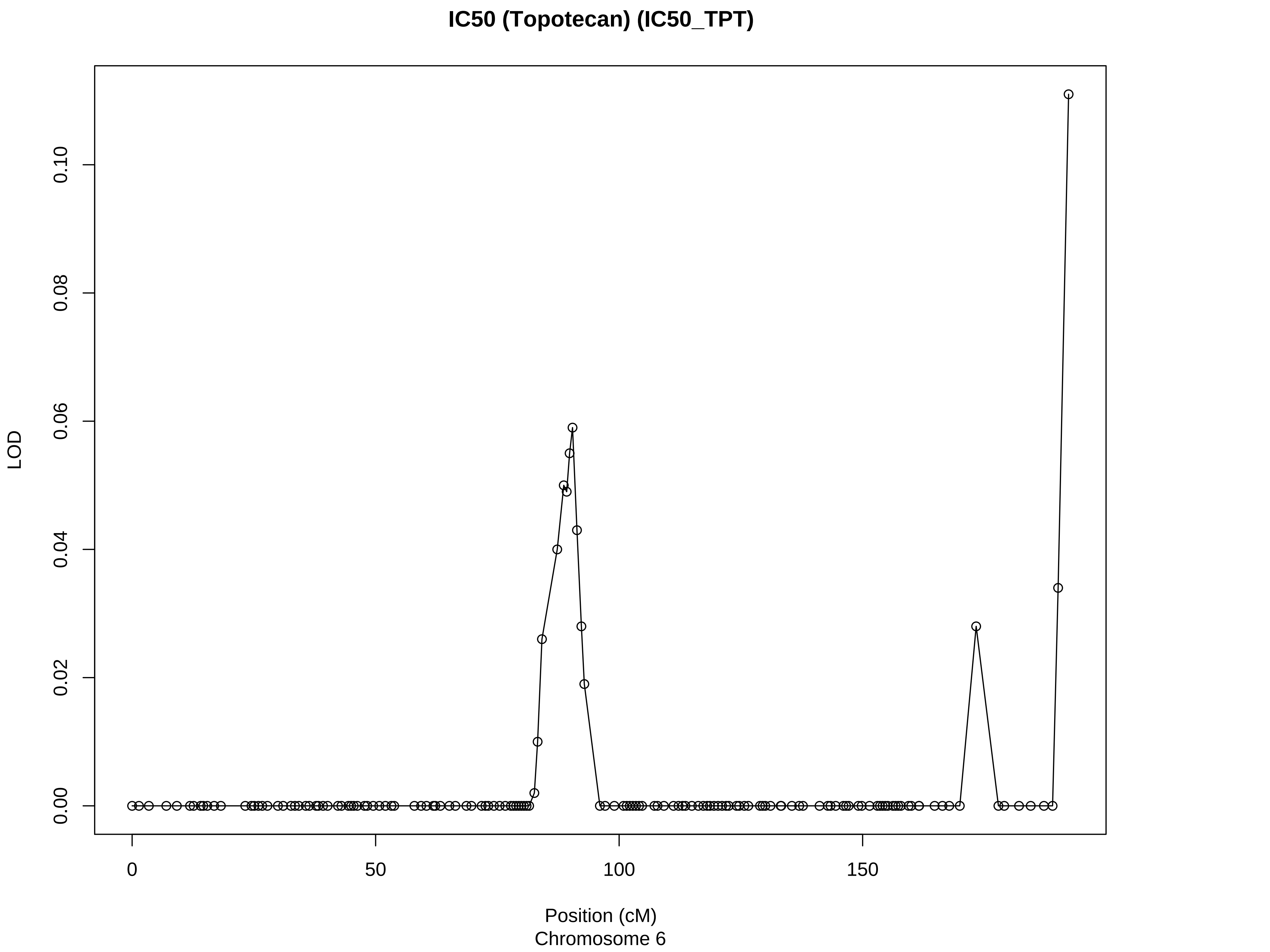
IC50 (Topotecan) (IC50_TPT)
LOD
0.00
0.02
0.04
0.06
0.08
0.10
0
50
100
150
Position (cM)
Chromosome 6

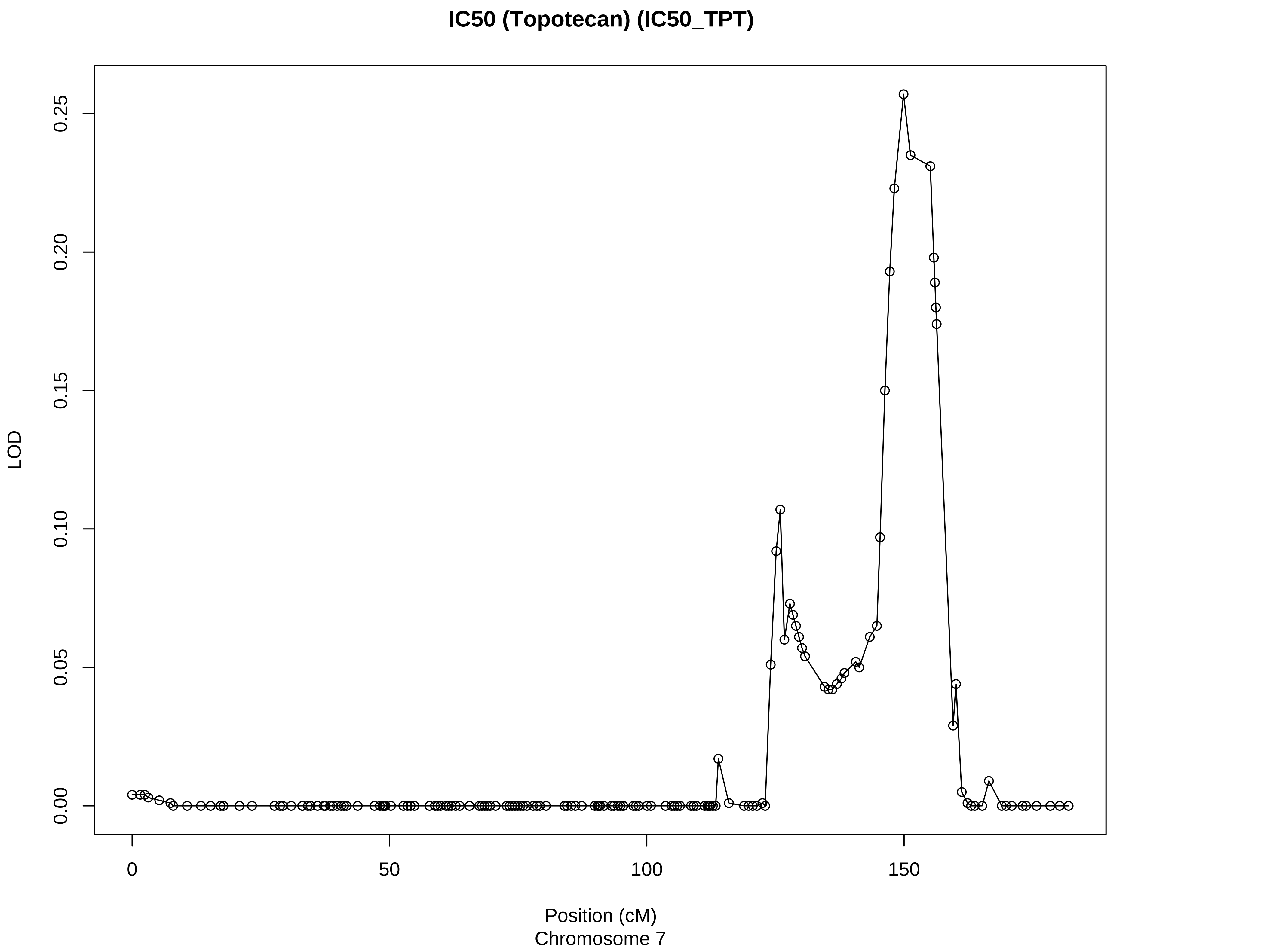
IC50 (Topotecan) (IC50_TPT)
LOD
0.00
0.05
0.10
0.15
0.20
0.25
0
50
100
150
Position (cM)
Chromosome 7

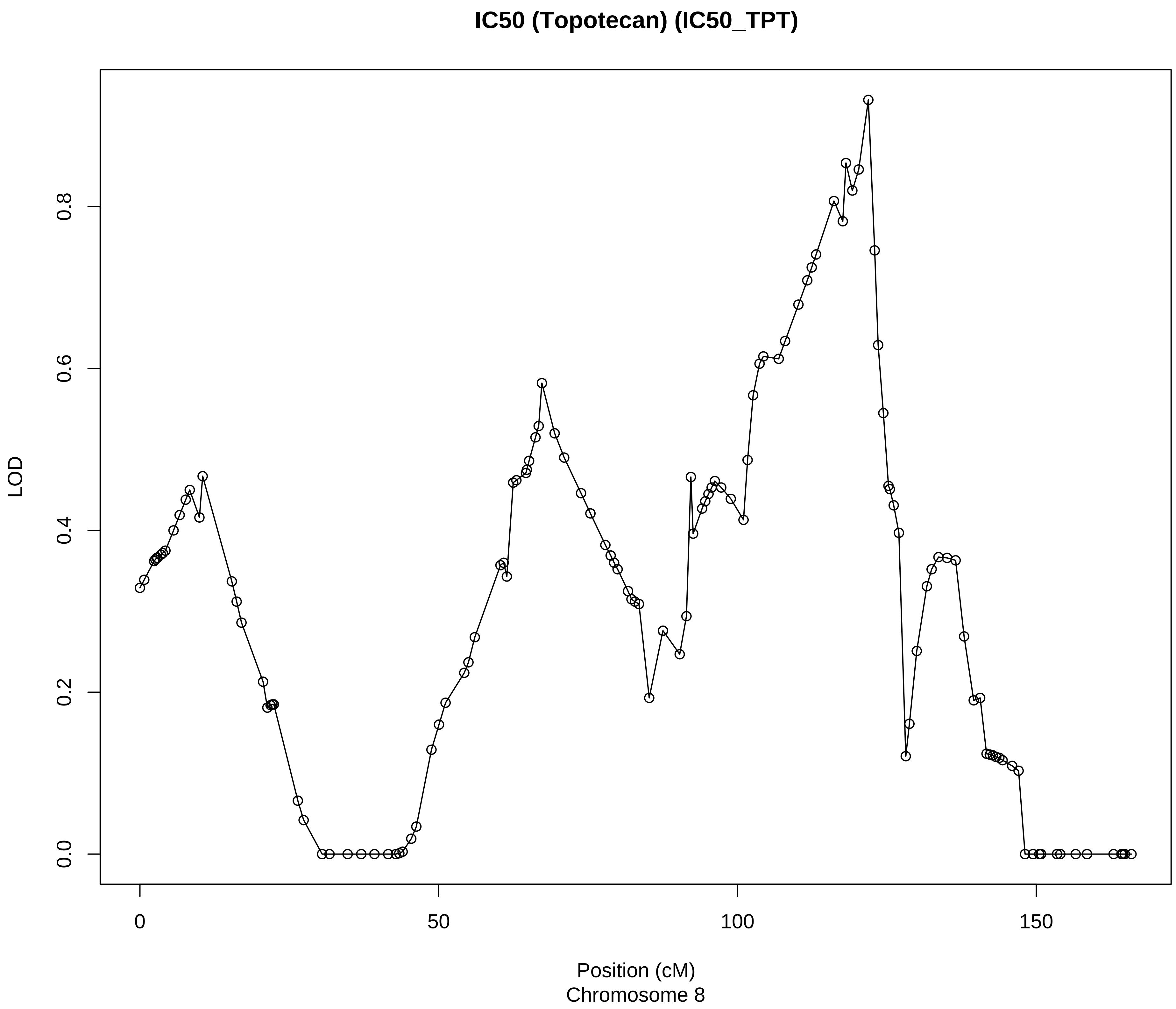
IC50 (Topotecan) (IC50_TPT)
LOD
0.0
0.2
0.4
0.6
0.8
0
50
100
150
Position (cM)
Chromosome 8

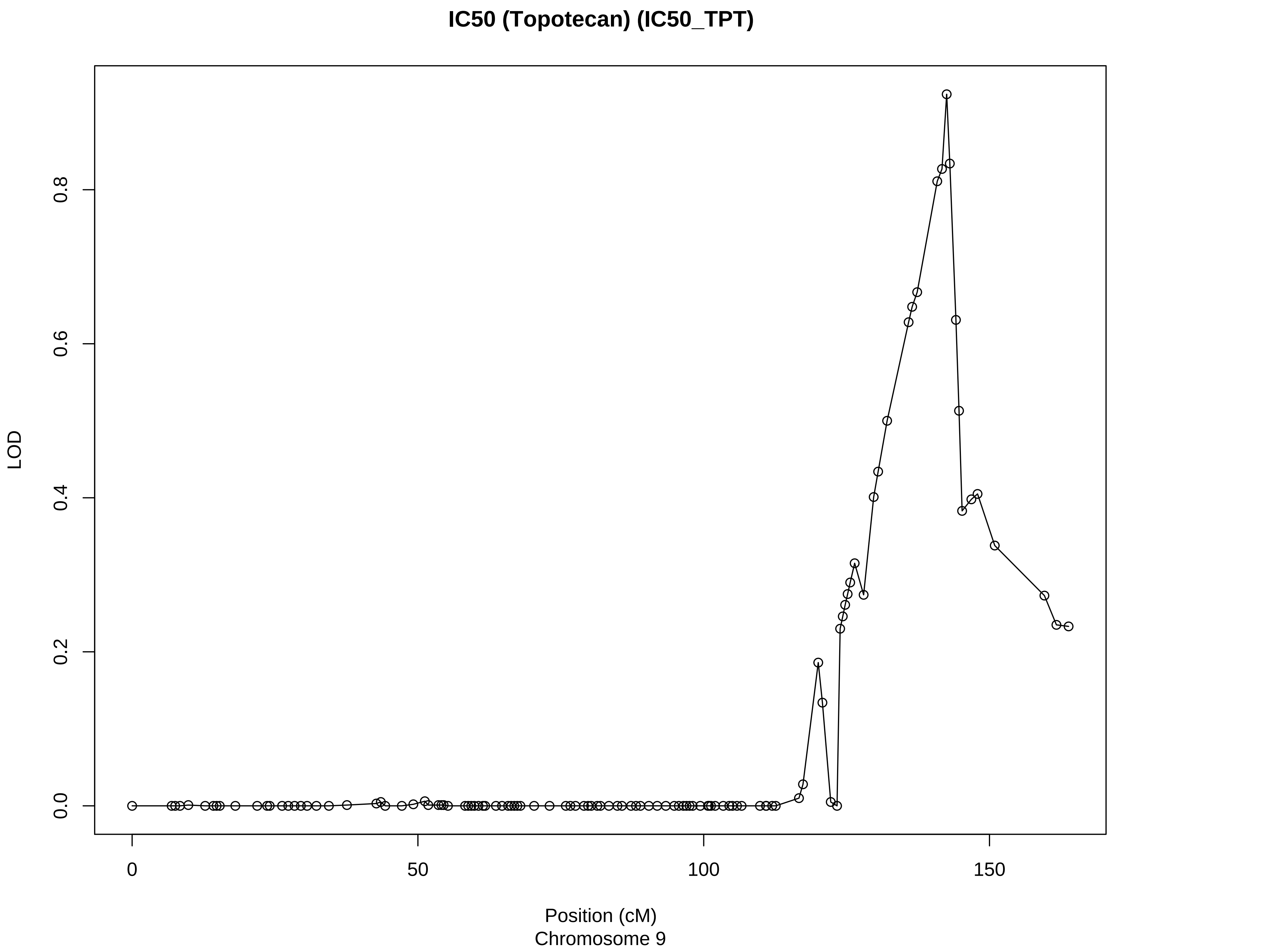
IC50 (Topotecan) (IC50_TPT)
LOD
0.0
0.2
0.4
0.6
0.8
0
50
100
150
Position (cM)
Chromosome 9

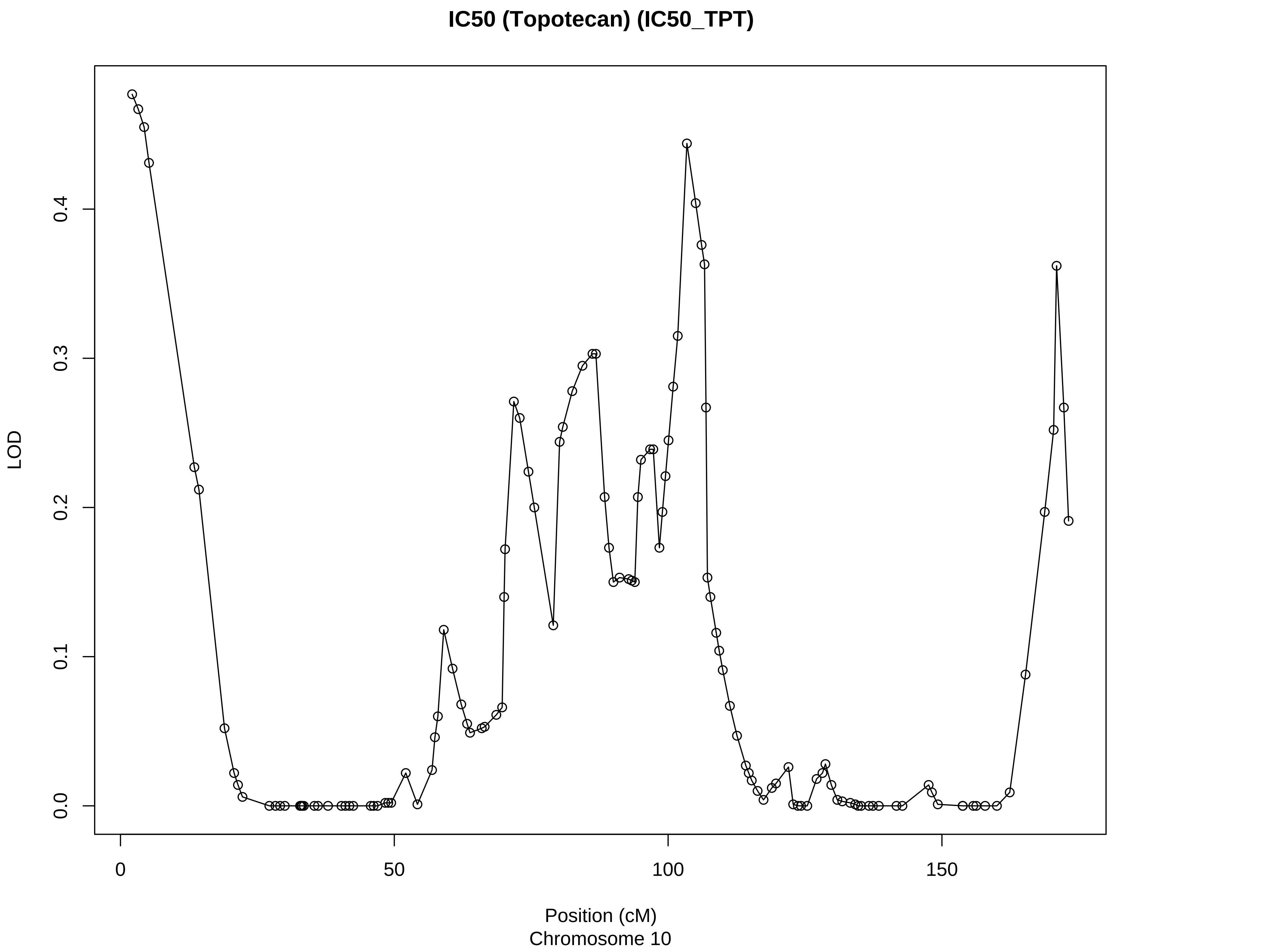

IC50 (Topotecan) (IC50_TPT)
LOD
0.0
0.1
0.2
0.3
0.4
0
50
100
150
Position (cM)
Chromosome 10

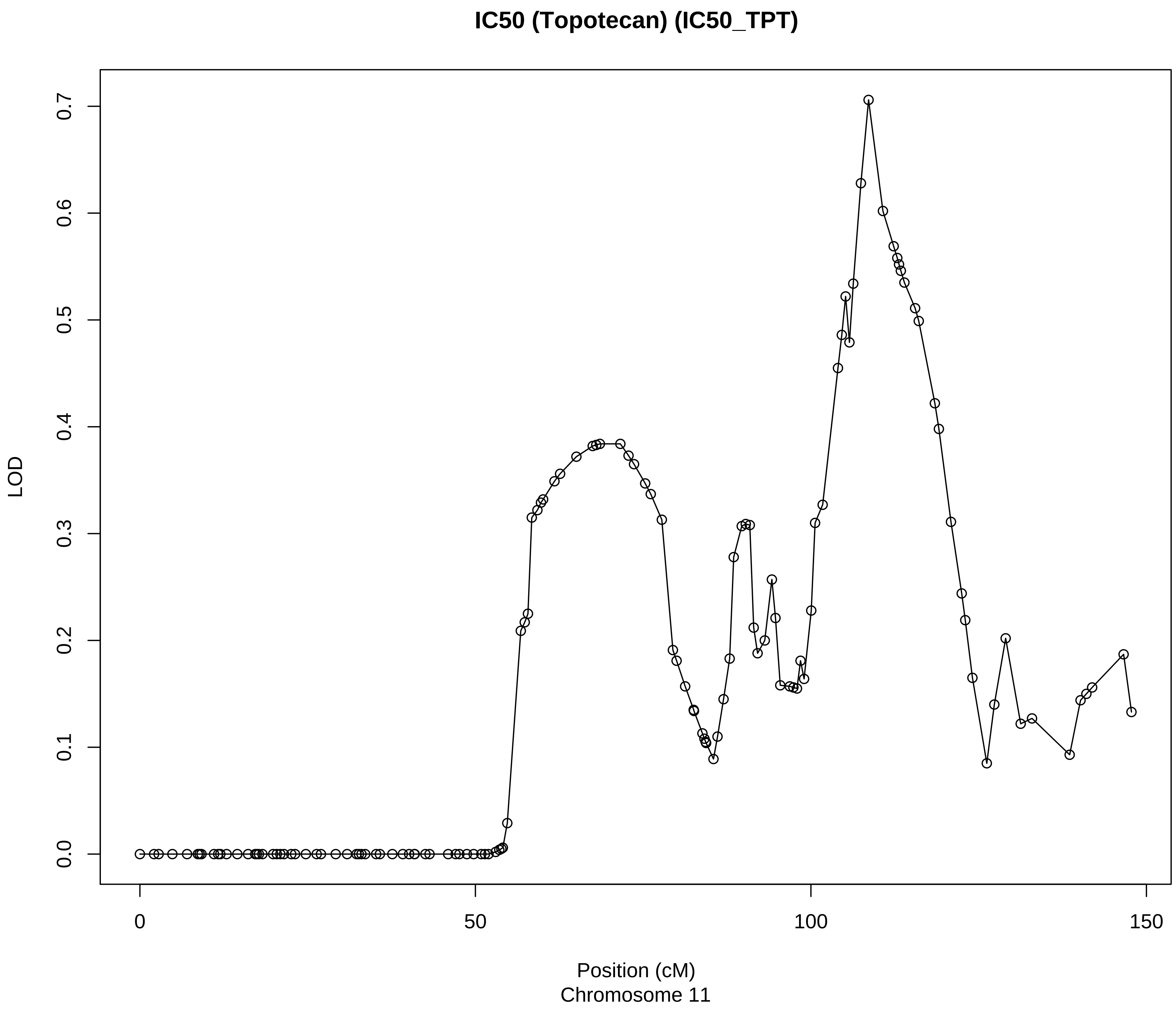

IC50 (Topotecan) (IC50_TPT)
LOD
0.0
0.1
0.2
0.3
0.4
0.5
0.6
0.7
0
50
100
150
Position (cM)
Chromosome 11

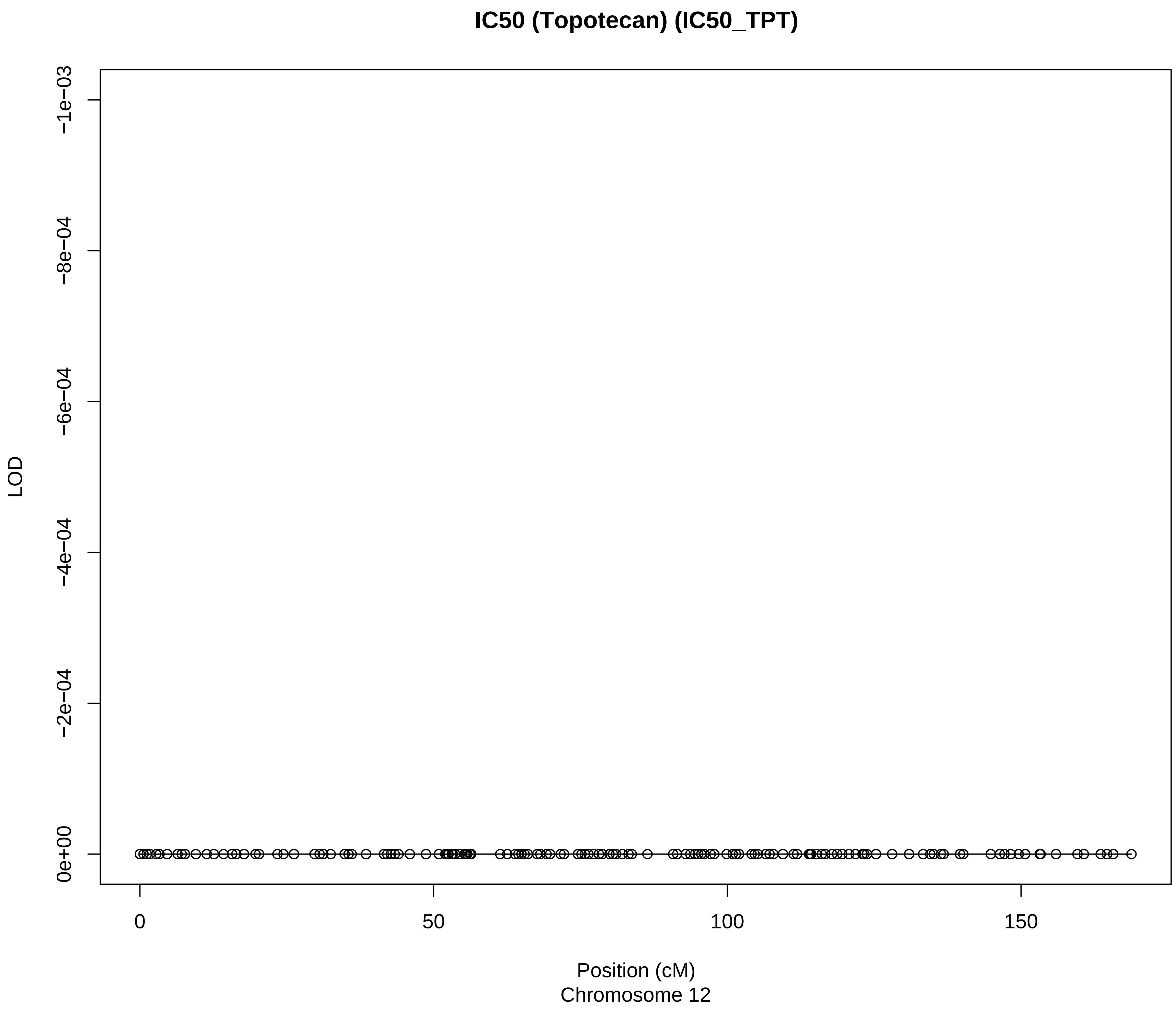

IC50 (Topotecan) (IC50_TPT)
LOD
−1e−03
−8e−04
−6e−04
−4e−04
−2e−04
0e+00
0
50
100
150
Position (cM)
Chromosome 12

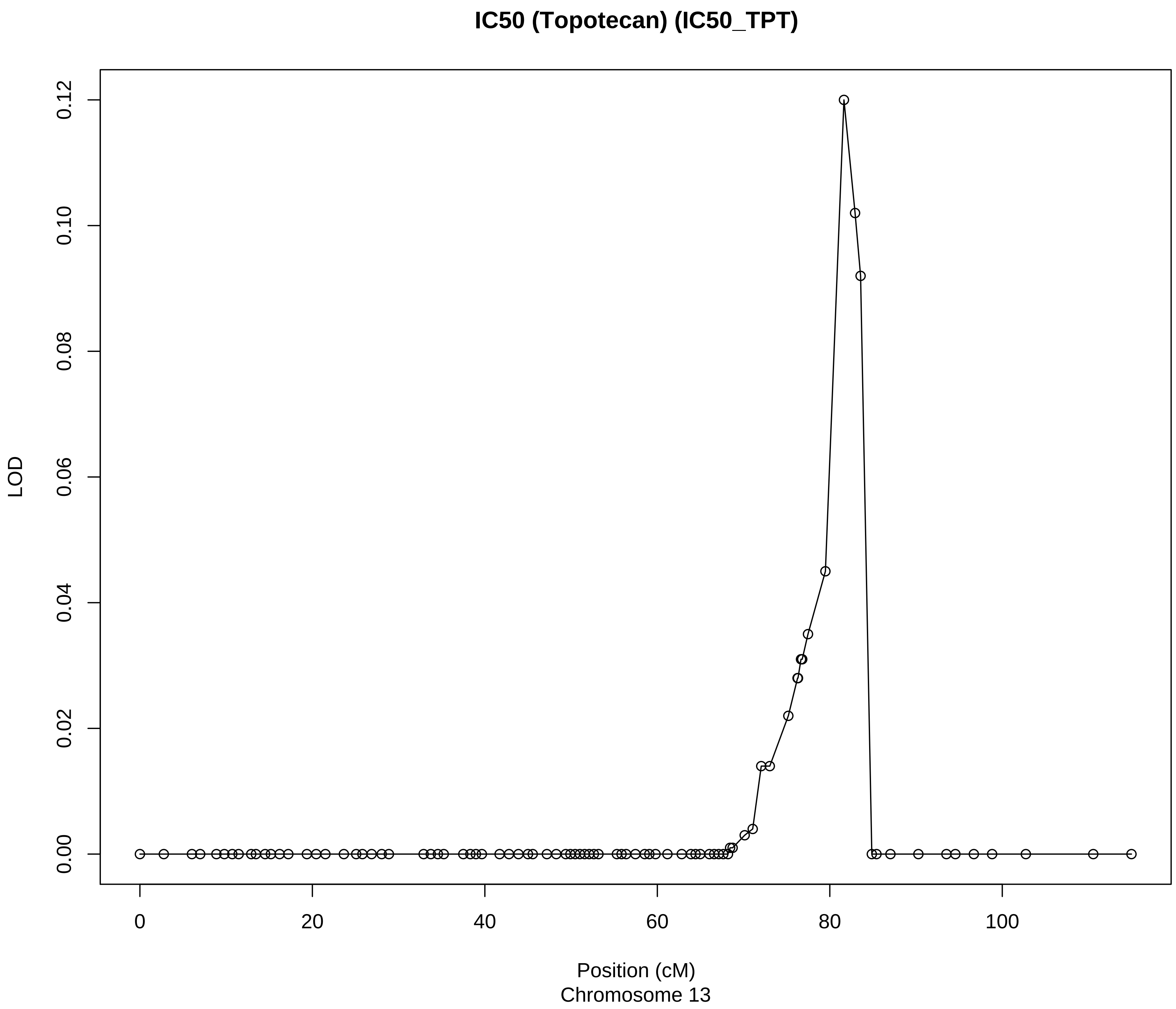
IC50 (Topotecan) (IC50_TPT)
LOD
0.00
0.02
0.04
0.06
0.08
0.10
0.12
0
20
40
60
80
100
Position (cM)
Chromosome 13

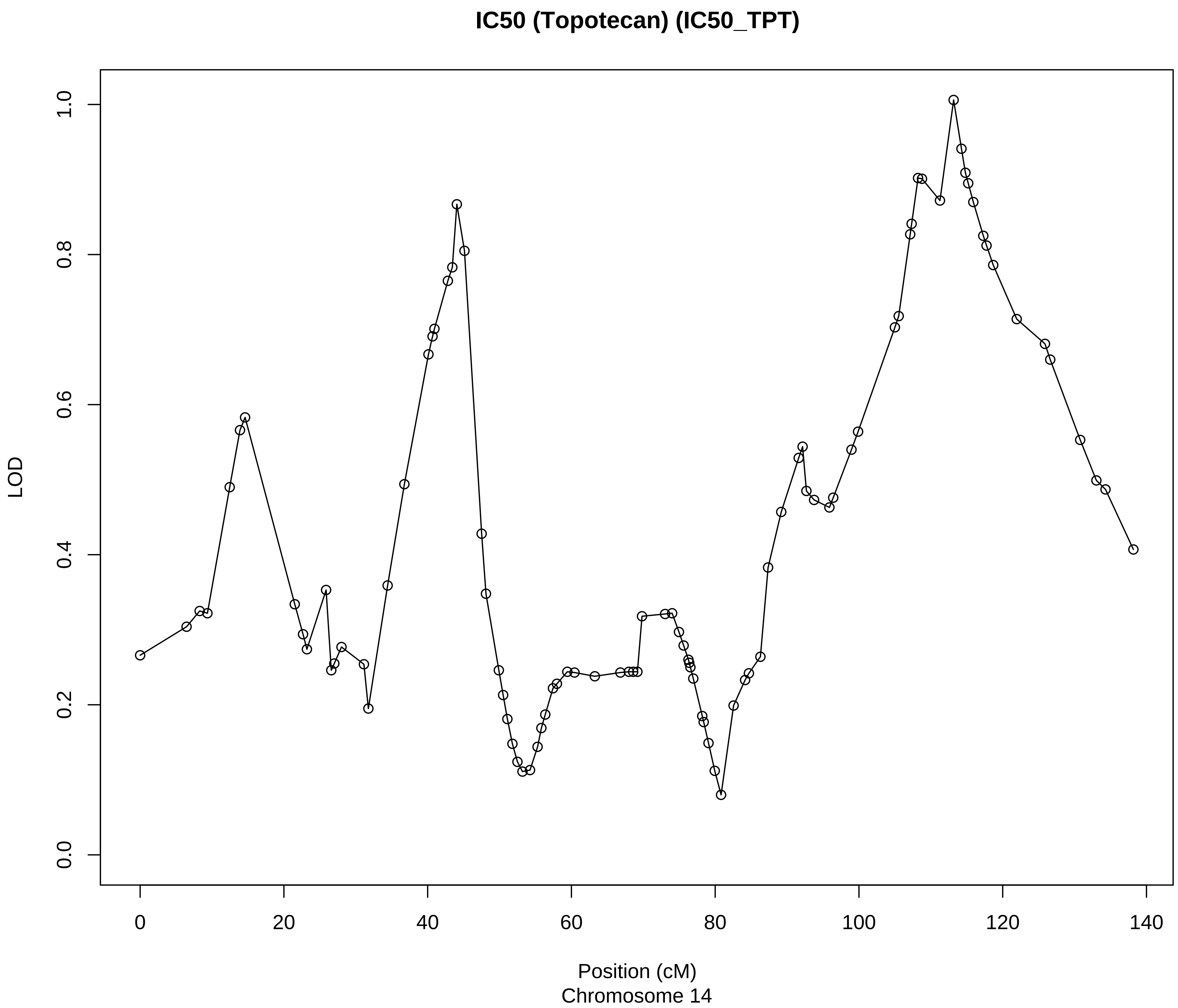

IC50 (Topotecan) (IC50_TPT)
LOD
0.0
0.2
0.4
0.6
0.8
1.0
0
20
40
60
80
100
120
140
Position (cM)
Chromosome 14

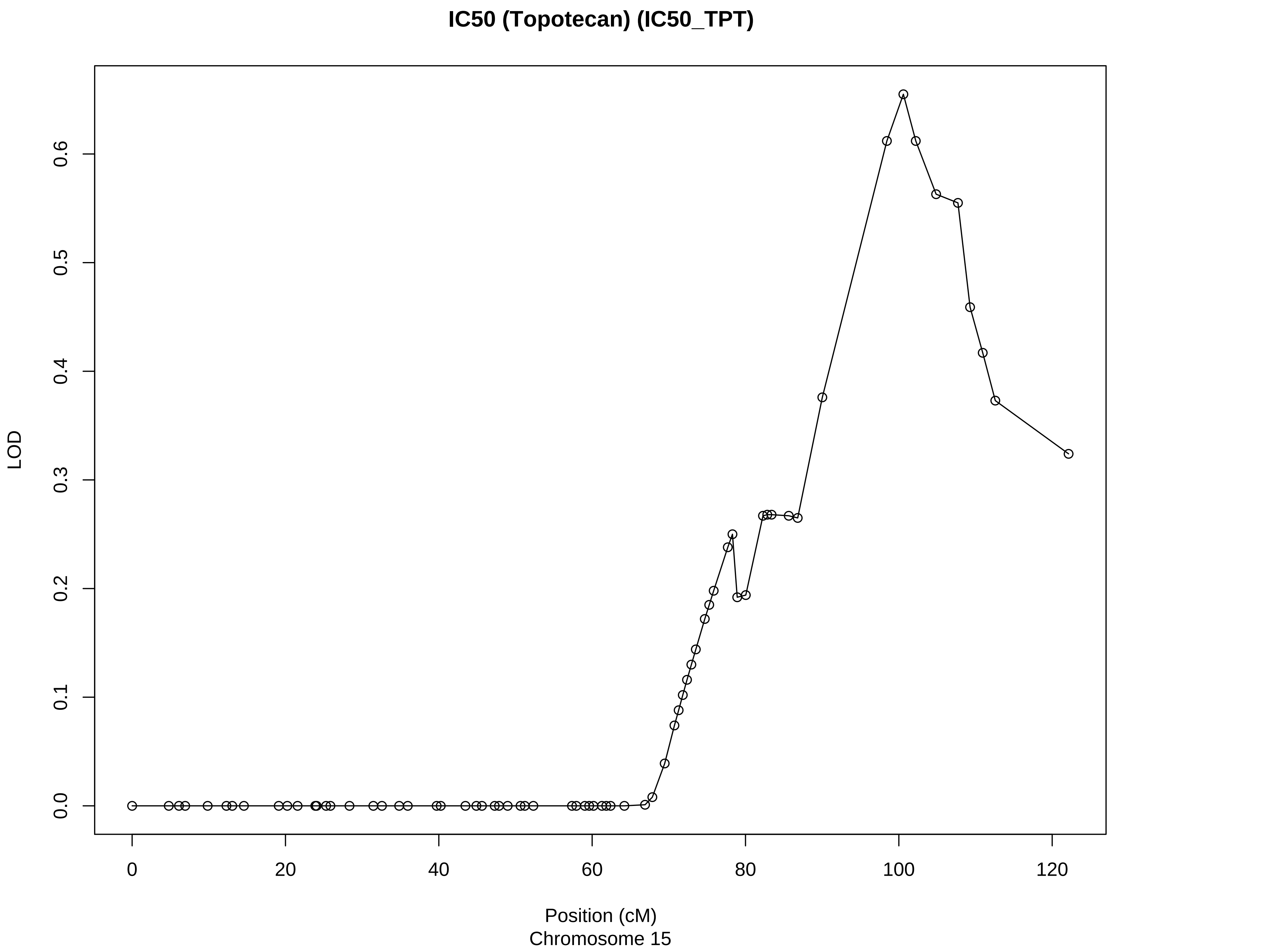
IC50 (Topotecan) (IC50_TPT)
LOD
0.0
0.1
0.2
0.3
0.4
0.5
0.6
0
20
40
60
80
100
120
Position (cM)
Chromosome 15

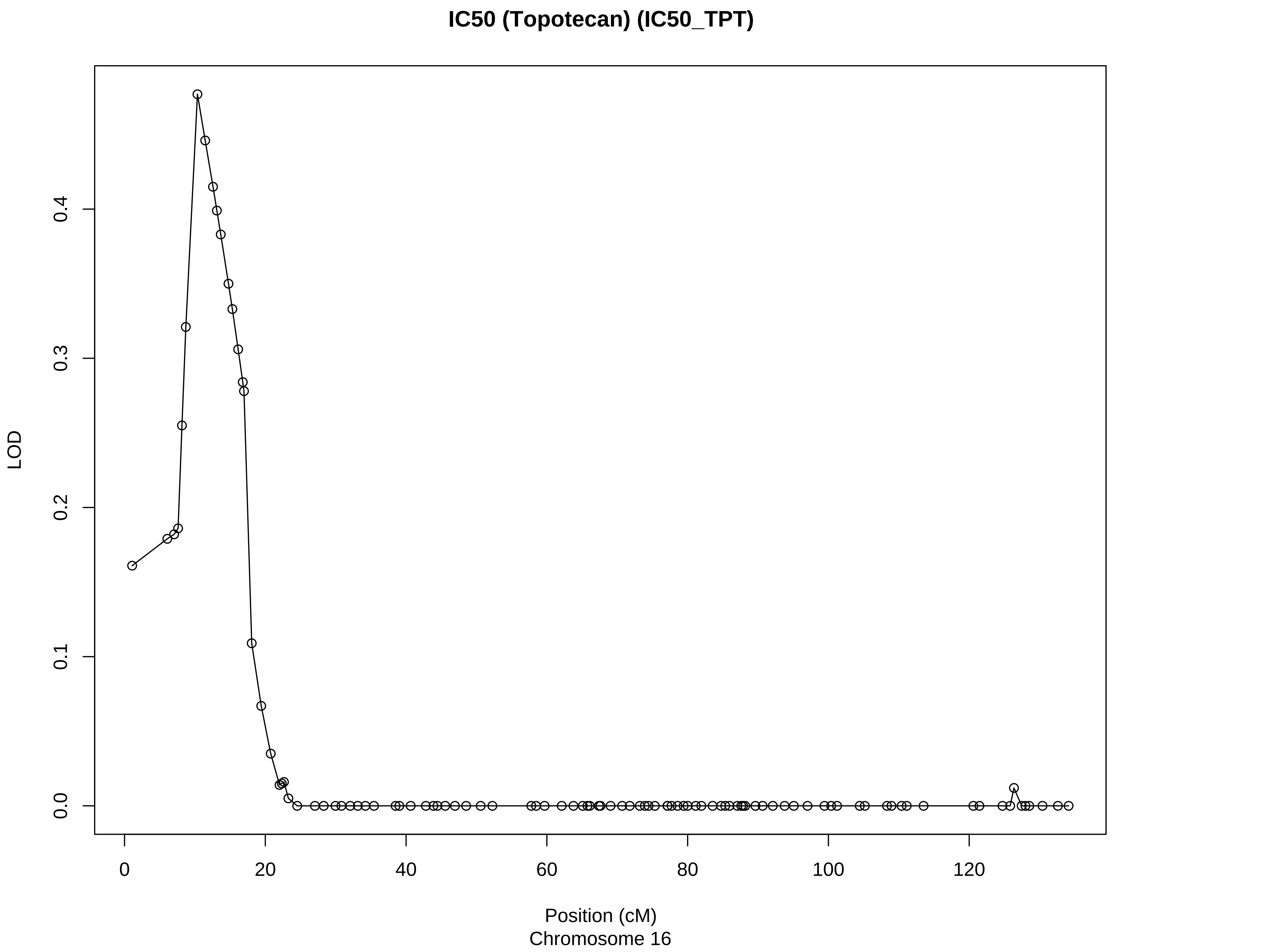
IC50 (Topotecan) (IC50_TPT)
LOD
0.0
0.1
0.2
0.3
0.4
0
20
40
60
80
100
120
Position (cM)
Chromosome 16

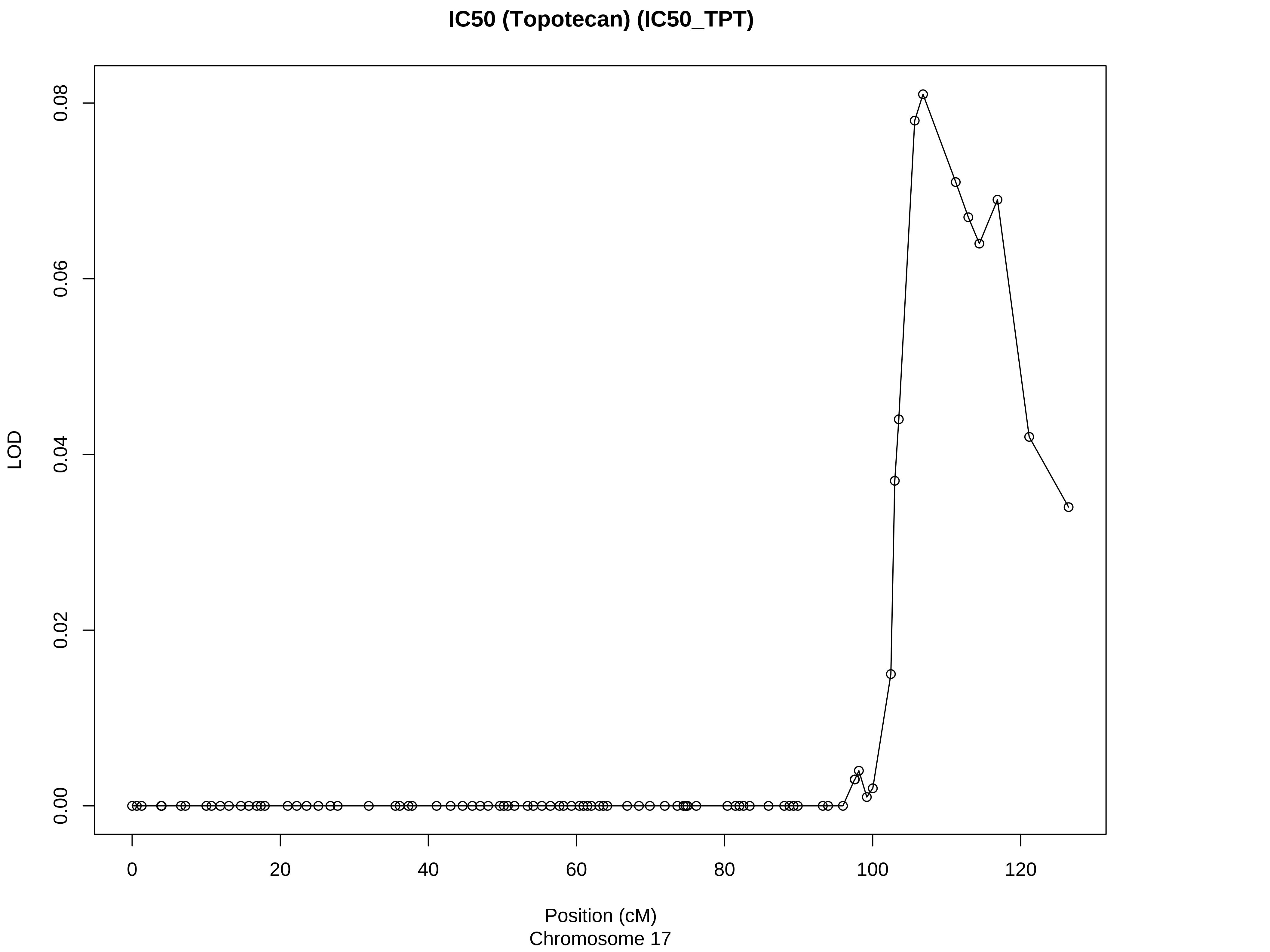
IC50 (Topotecan) (IC50_TPT)
LOD
0.00
0.02
0.04
0.06
0.08
0
20
40
60
80
100
120
Position (cM)
Chromosome 17

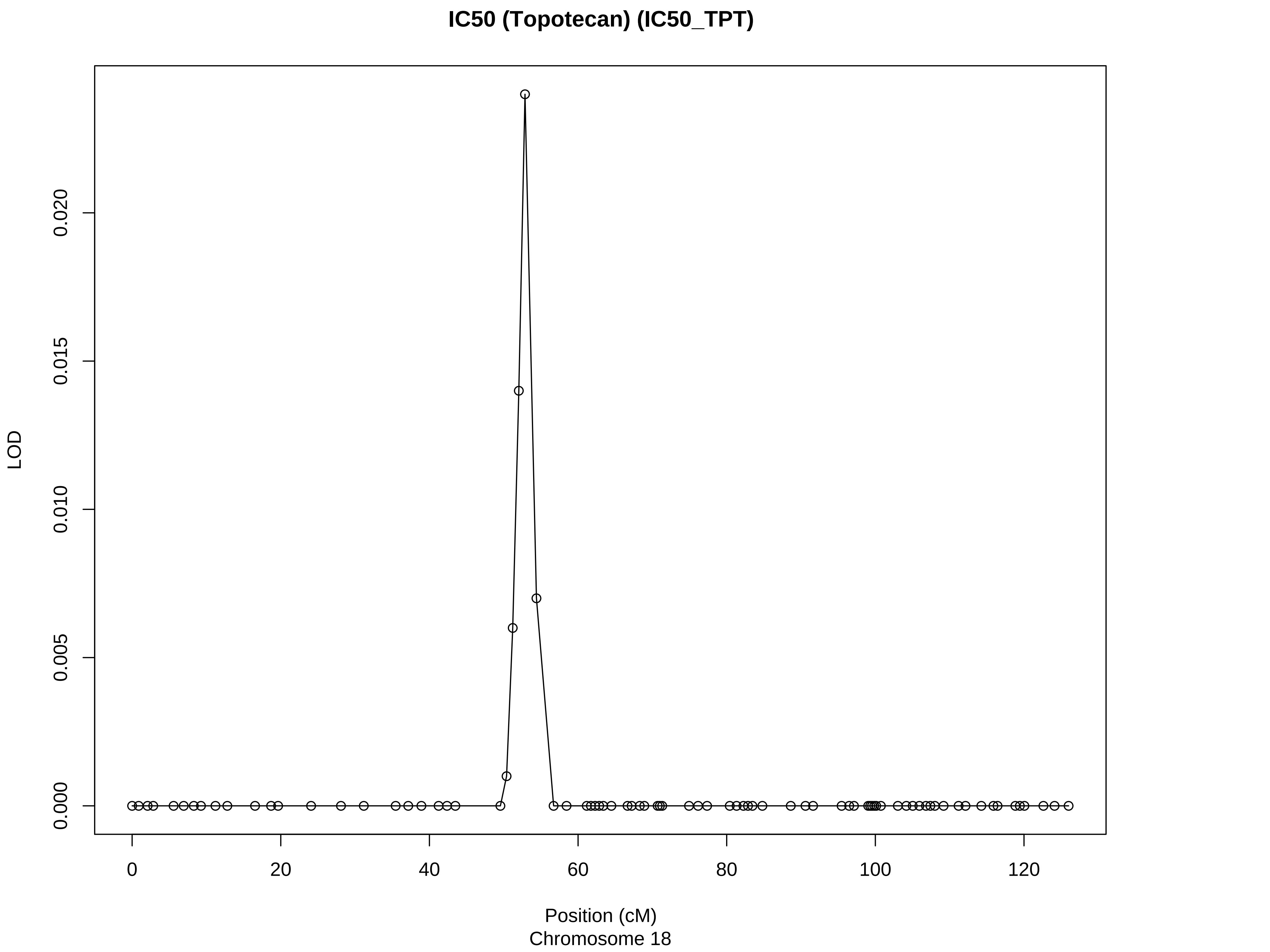

IC50 (Topotecan) (IC50_TPT)
LOD
0.000
0.005
0.010
0.015
0.020
0
20
40
60
80
100
120
Position (cM)
Chromosome 18

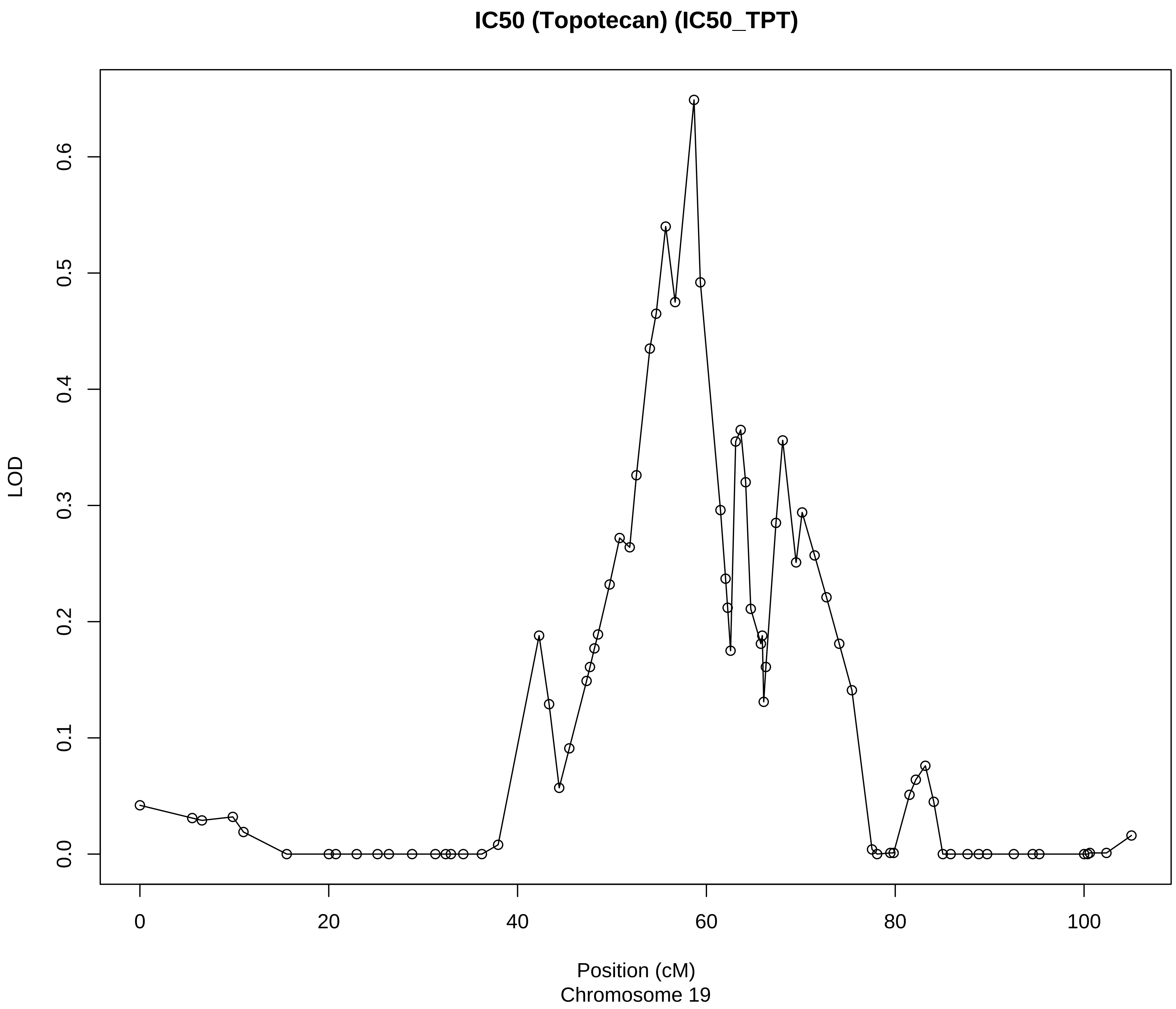
IC50 (Topotecan) (IC50_TPT)
LOD
0.0
0.1
0.2
0.3
0.4
0.5
0.6
0
20
40
60
80
100
Position (cM)
Chromosome 19

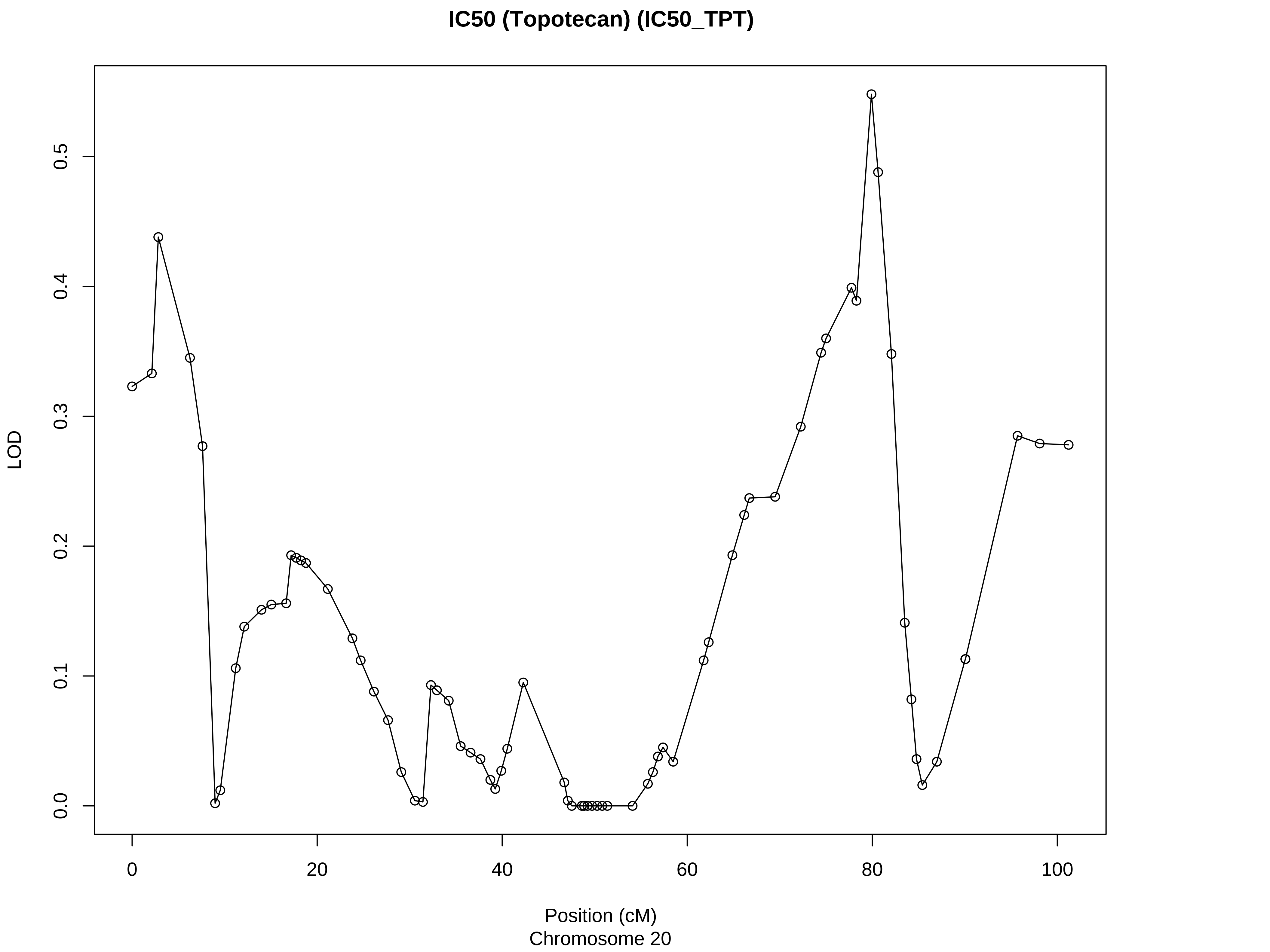

IC50 (Topotecan) (IC50_TPT)
LOD
0.0
0.1
0.2
0.3
0.4
0.5
0
20
40
60
80
100
Position (cM)
Chromosome 20

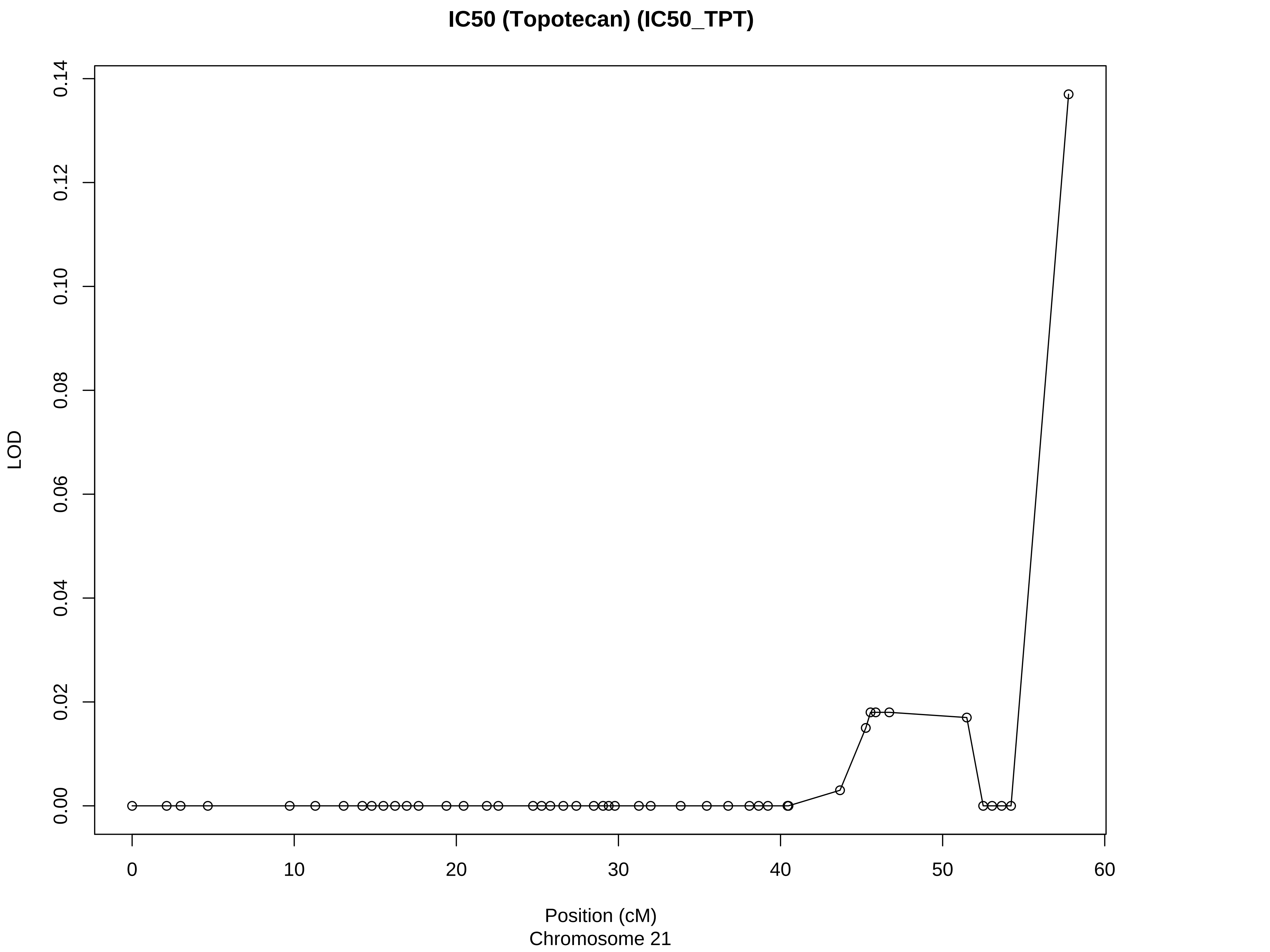
IC50 (Topotecan) (IC50_TPT)
LOD
0.00
0.02
0.04
0.06
0.08
0.10
0.12
0.14
0
10
20
30
40
50
60
Position (cM)
Chromosome 21

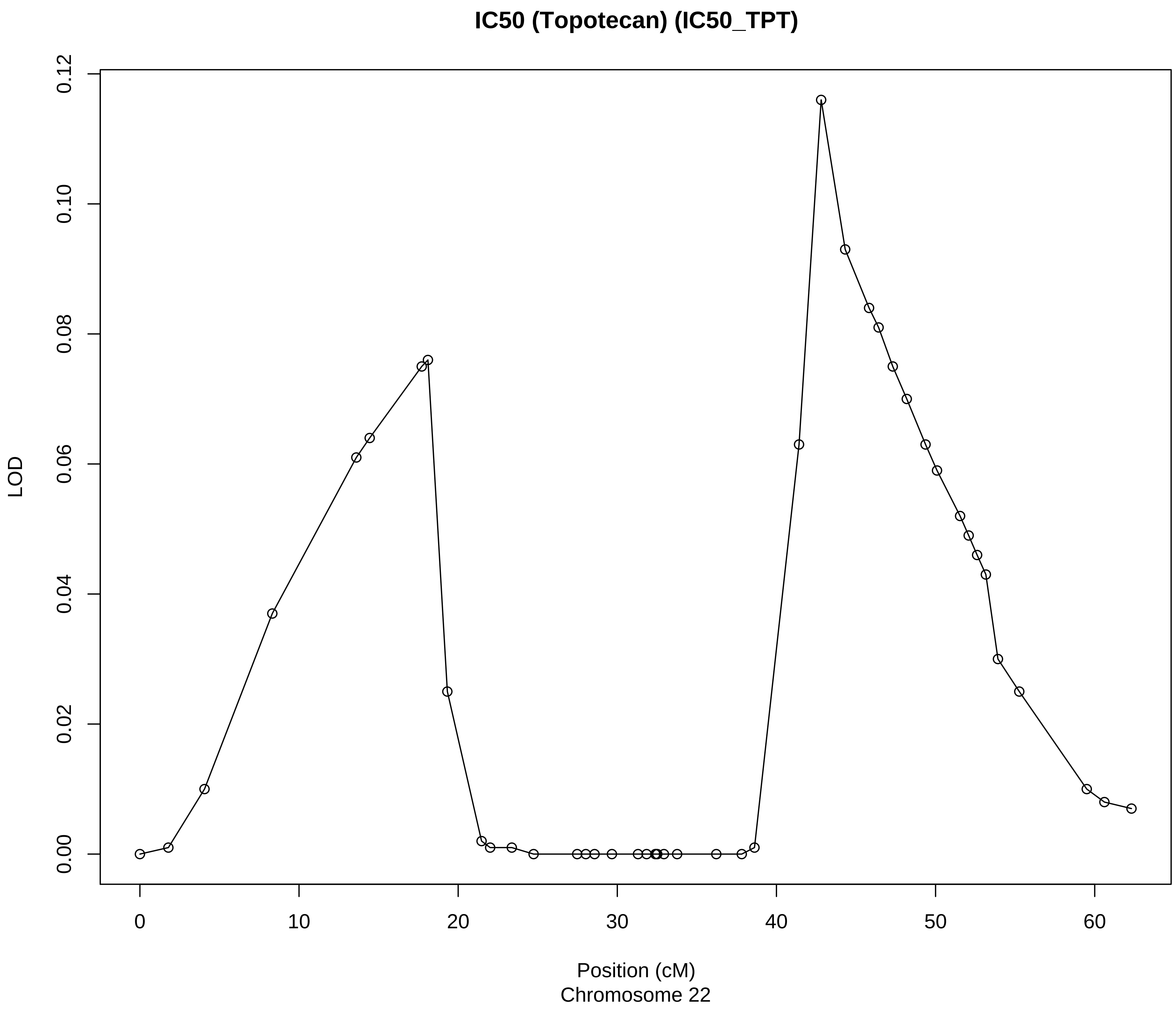
IC50 (Topotecan) (IC50_TPT)
LOD
0.00
0.02
0.04
0.06
0.08
0.10
0.12
0
10
20
30
40
50
60
Position (cM)
Chromosome 22

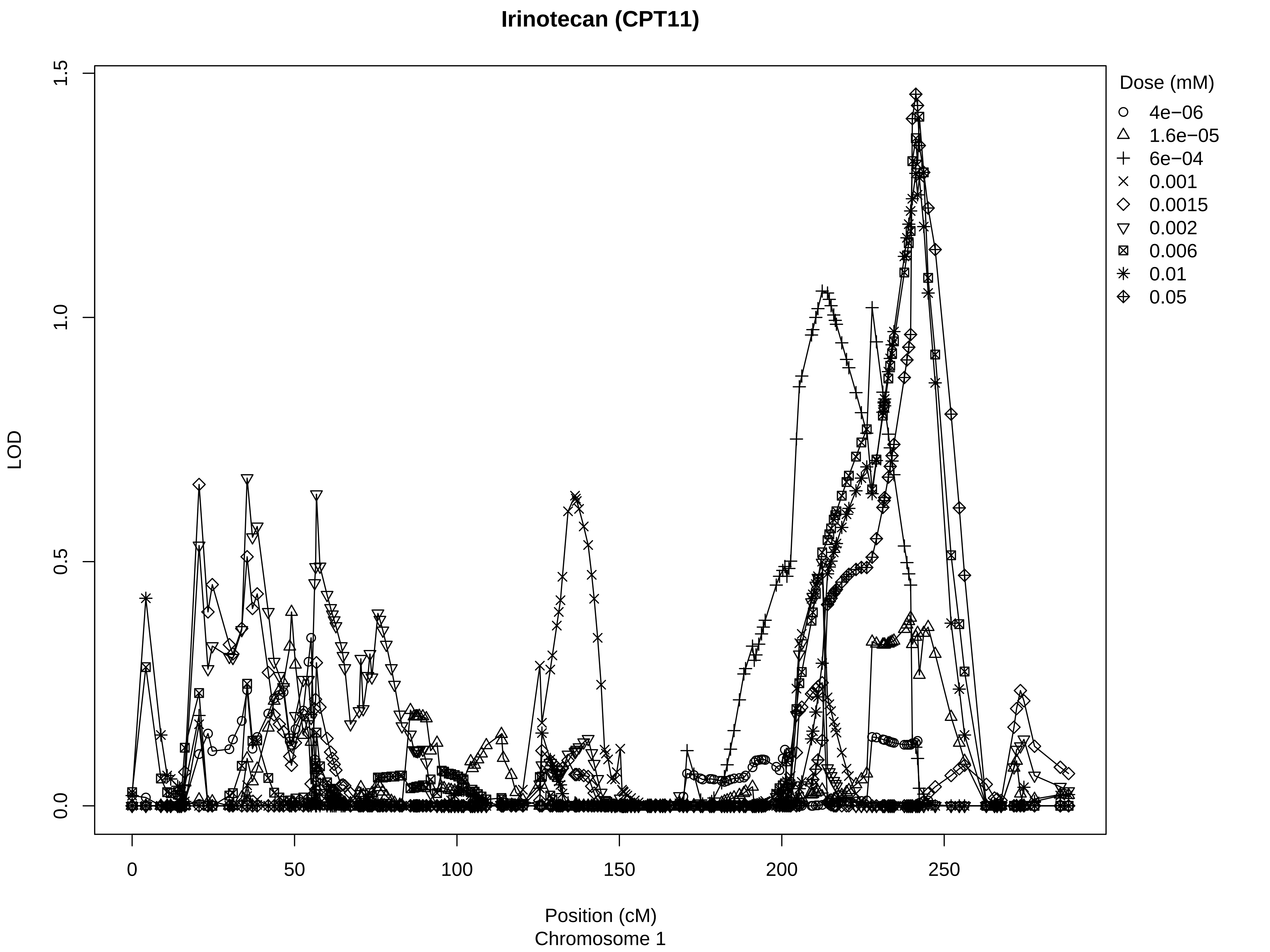

Irinotecan (CPT11)
Dose (mM)
4e−06
1.6e−05
6e−04
0.001
0.0015
0.002
0.006
0.01
0.05
LOD
0.0
0.5
1.0
1.5
0
50
100
150
200
250
Position (cM)
Chromosome 1

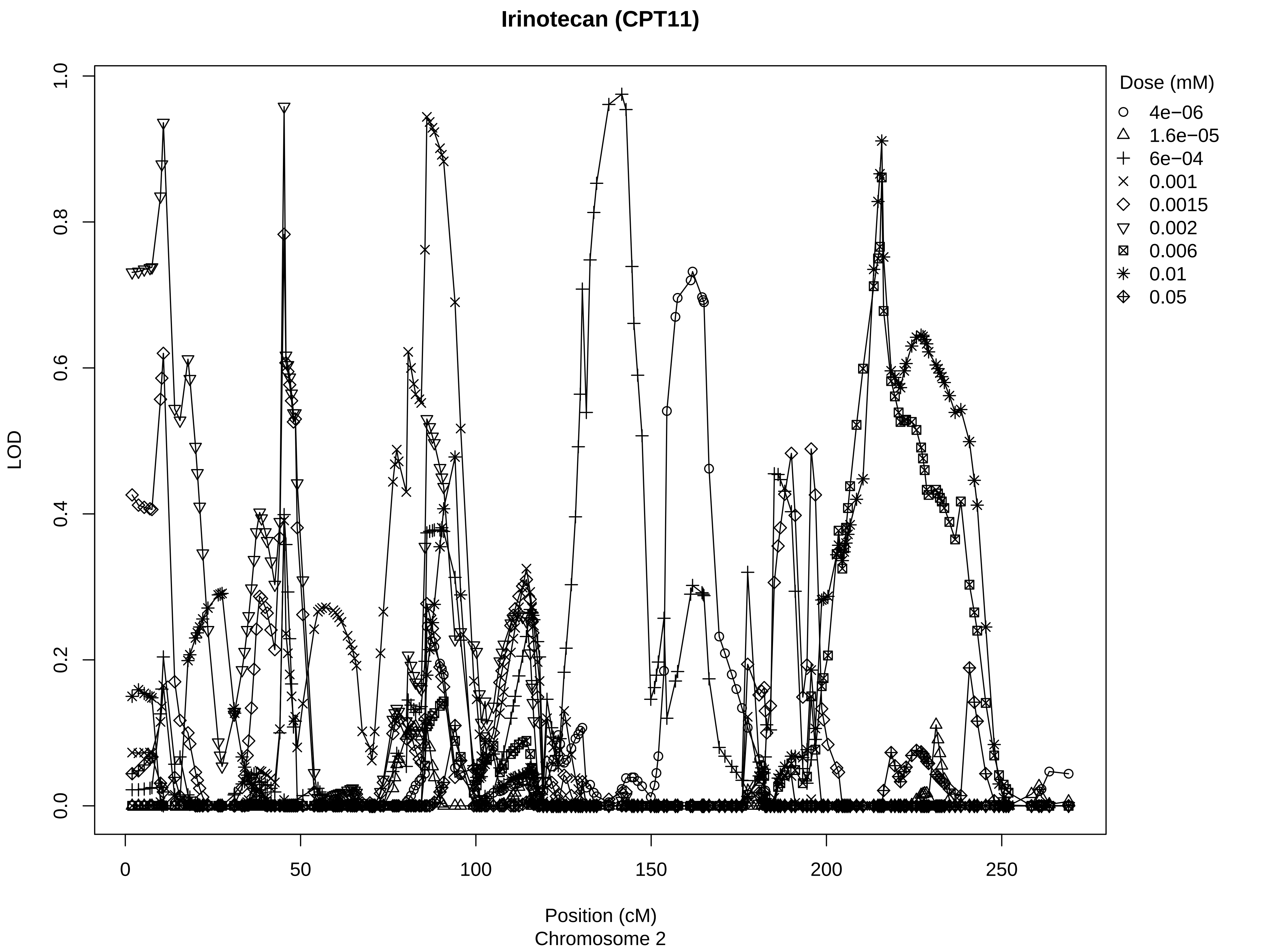

Irinotecan (CPT11)
LOD
1.0
0.8
0.6
0.4
0.2
0.0
0
50
100
150
200
250
Position (cM)
Chromosome 2
Dose (mM)
4e−06
1.6e−05
6e−04
0.001
0.0015
0.002
0.006
0.01
0.05

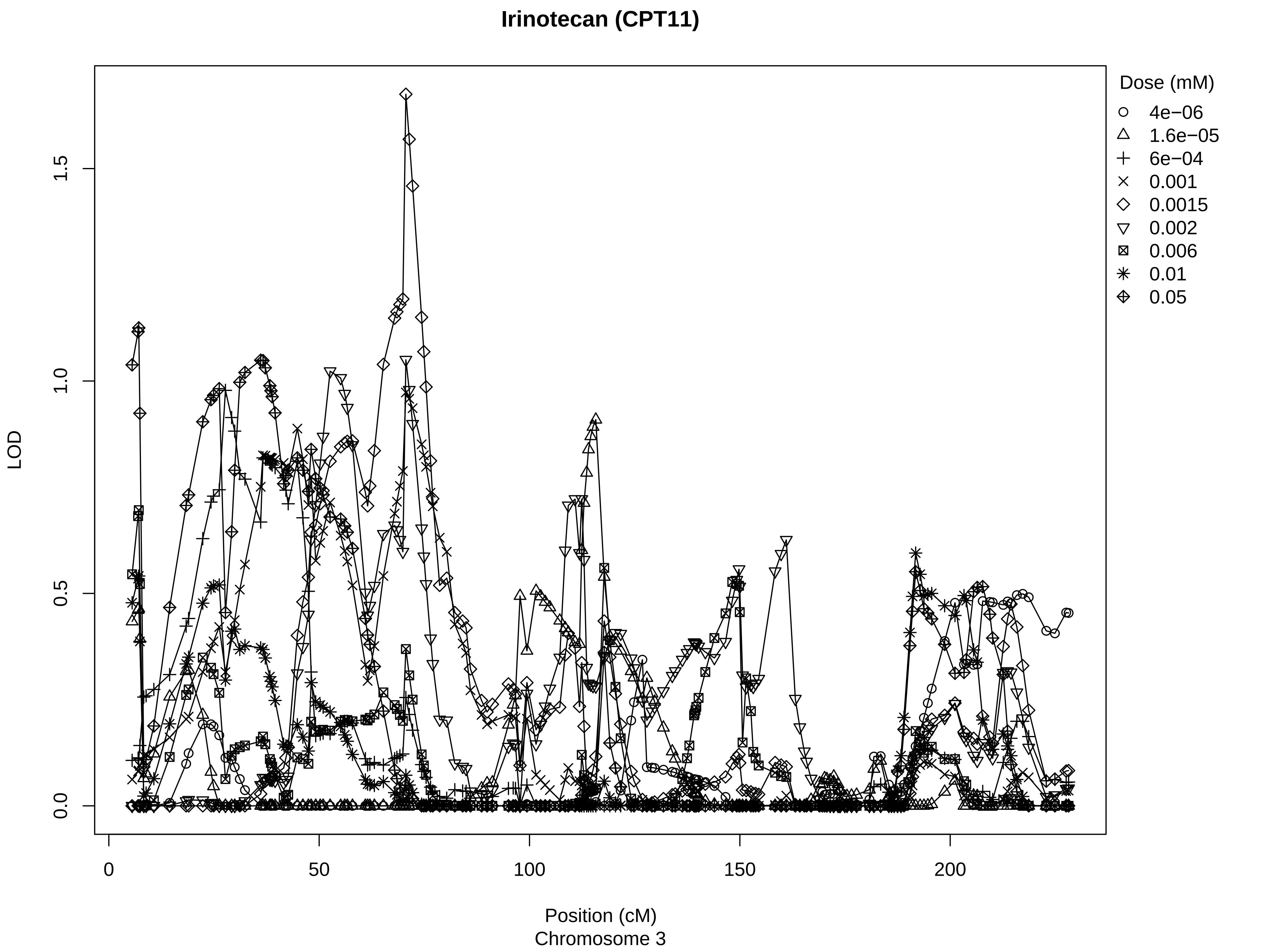
Irinotecan (CPT11)
Dose (mM)
4e−06
1.6e−05
6e−04
0.001
0.0015
0.002
0.006
0.01
0.05
LOD
0.0
0.5
1.0
1.5
0
50
100
150
200
Position (cM)
Chromosome 3

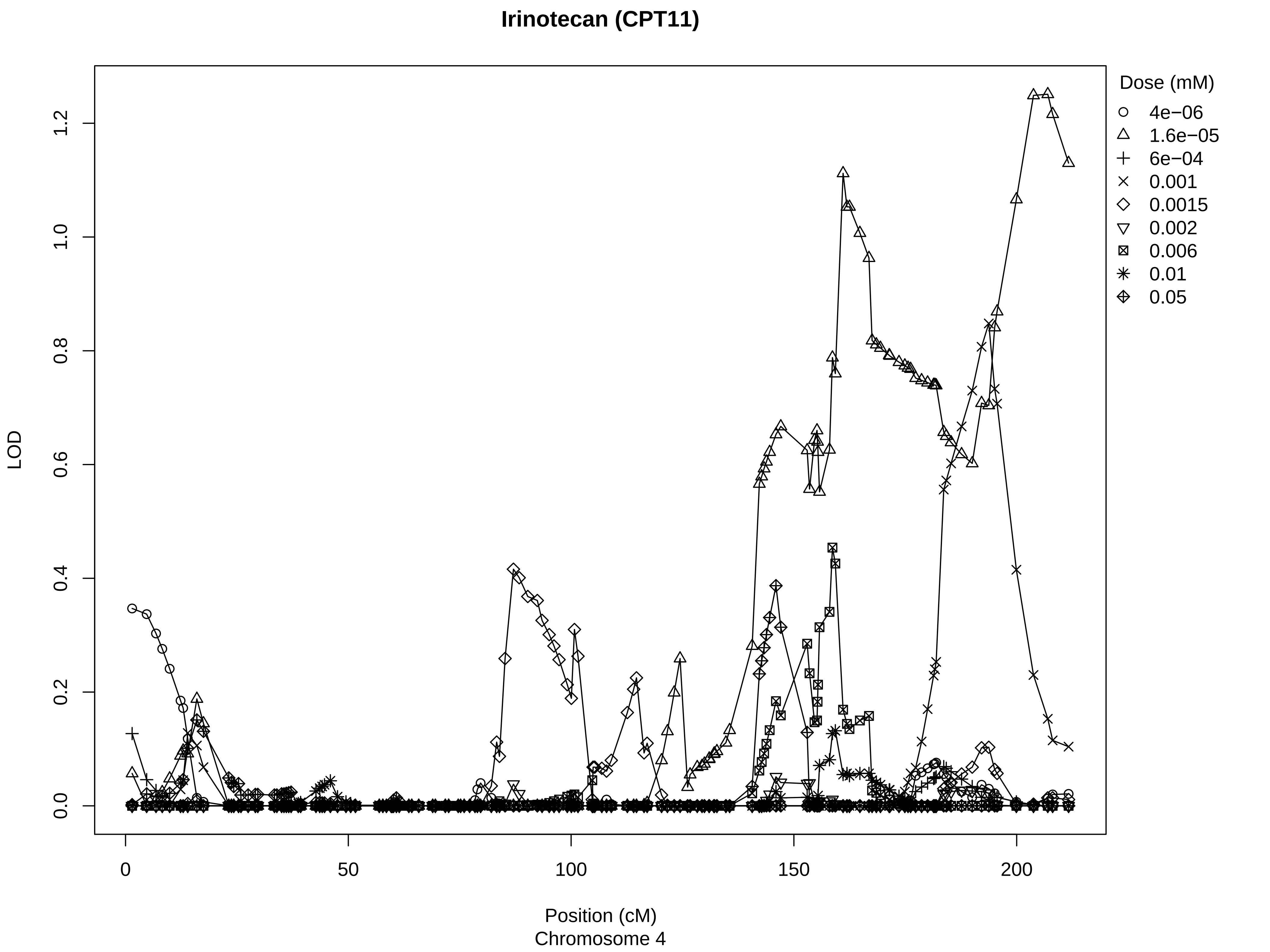

Irinotecan (CPT11)
Dose (mM)
4e−06
1.6e−05
6e−04
0.001
0.0015
0.002
0.006
0.01
0.05
LOD
0.0
0.2
0.4
0.6
0.8
1.0
1.2
0
50
100
150
200
Position (cM)
Chromosome 4

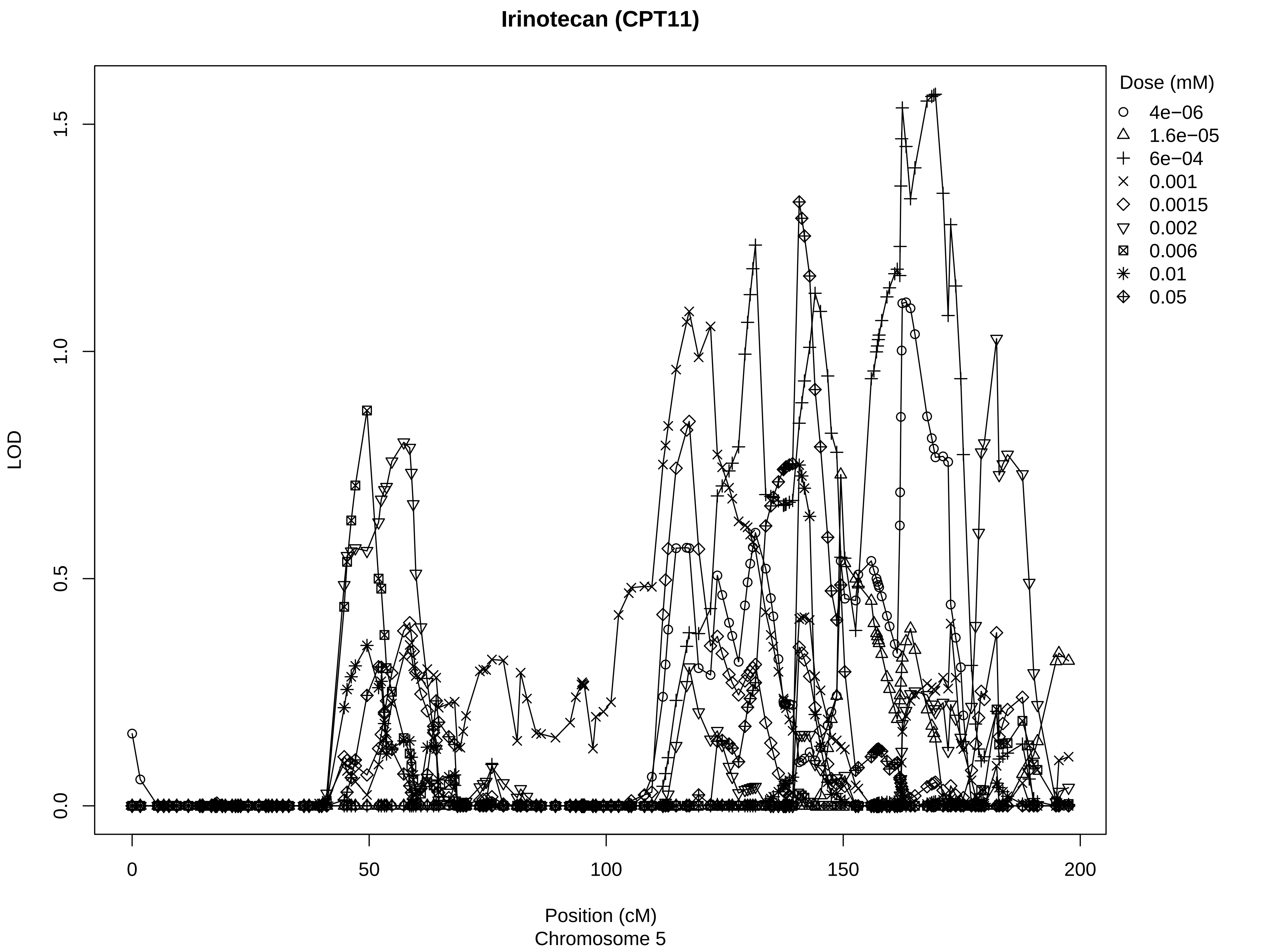

Irinotecan (CPT11)
LOD
0.0
0.5
1.0
1.5
0
50
100
150
200
Position (cM)
Chromosome 5
Dose (mM)
4e−06
1.6e−05
6e−04
0.001
0.0015
0.002
0.006
0.01
0.05

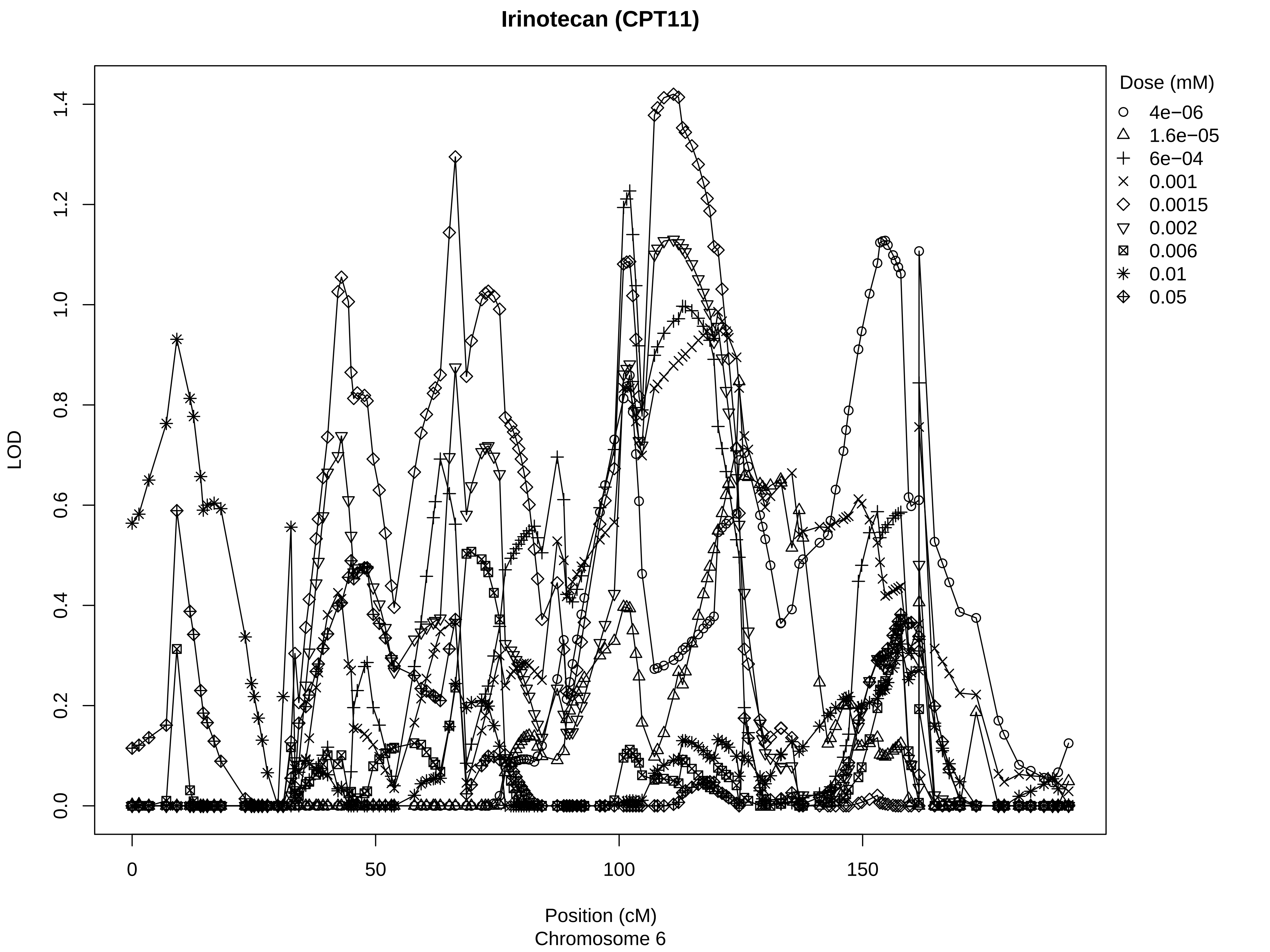
Irinotecan (CPT11)
Dose (mM)
4e−06
1.6e−05
6e−04
0.001
0.0015
0.002
0.006
0.01
0.05
LOD
0.0
0.2
0.4
0.6
0.8
1.0
1.2
1.4
0
50
100
150
Position (cM)
Chromosome 6

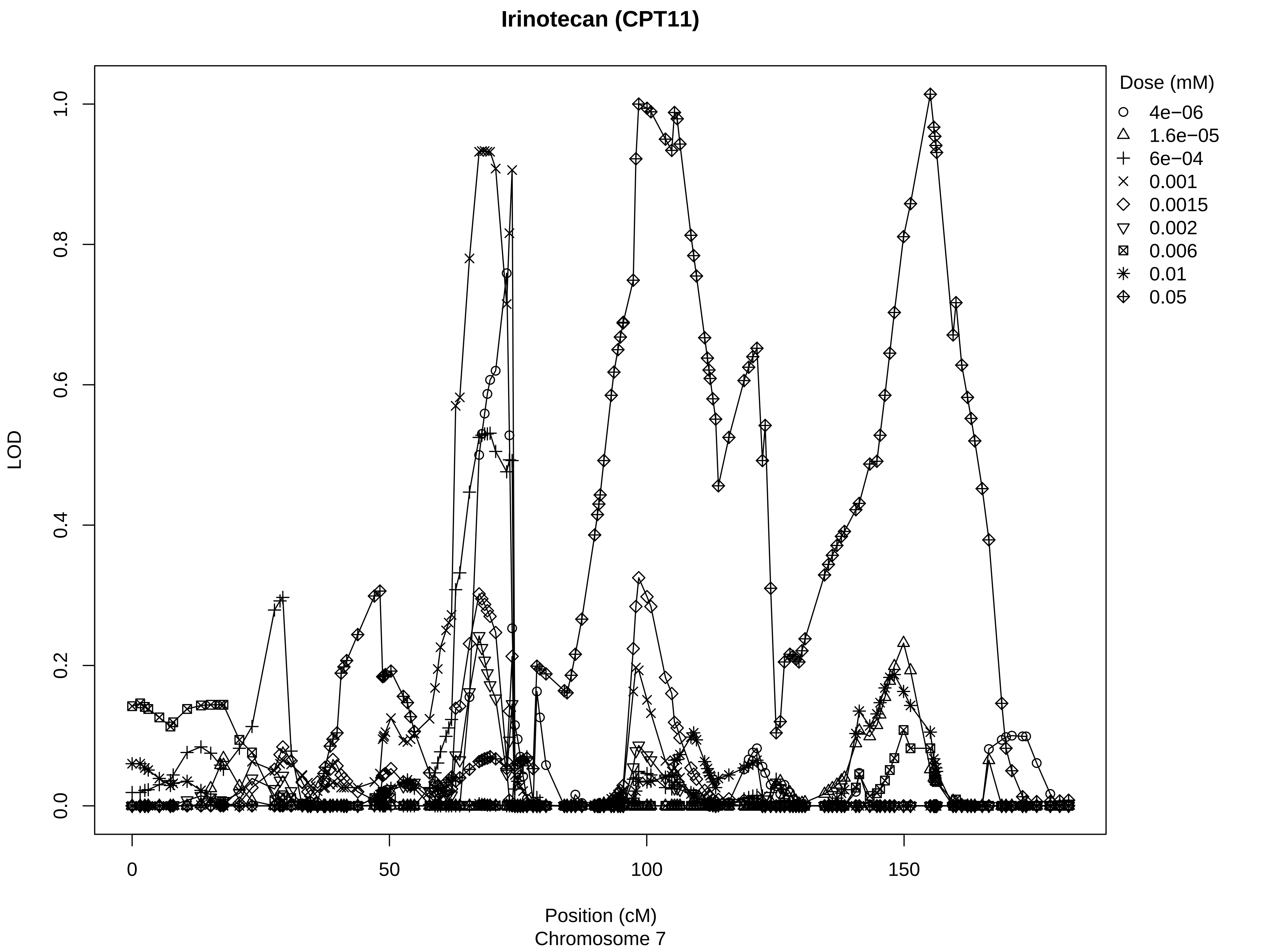

Irinotecan (CPT11)
LOD
0.0
0.2
0.4
0.6
0.8
1.0
0
50
100
150
Position (cM)
Chromosome 7
Dose (mM)
4e−06
1.6e−05
6e−04
0.001
0.0015
0.002
0.006
0.01
0.05

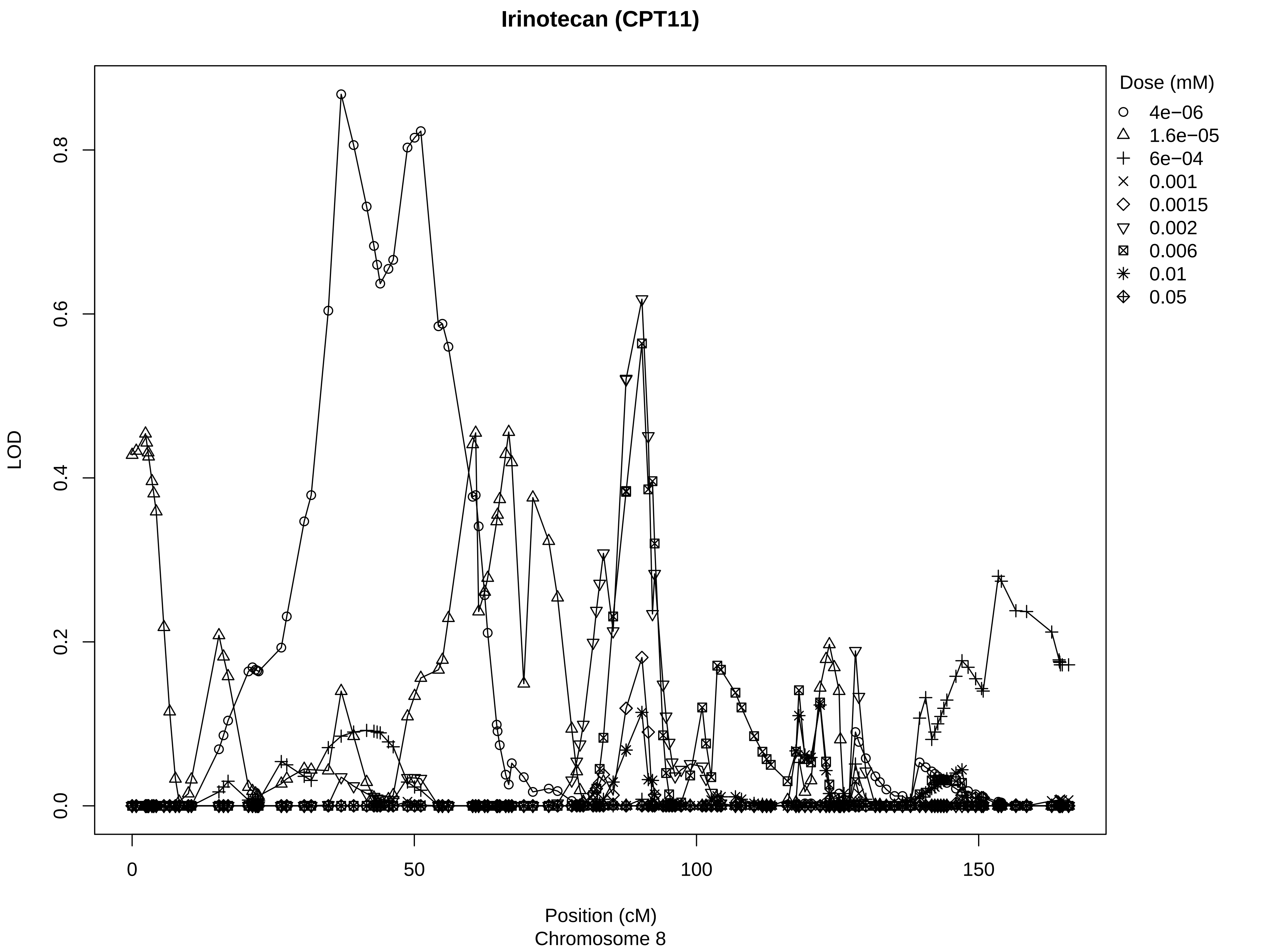

Irinotecan (CPT11)
LOD
0.0
0.2
0.4
0.6
0.8
0
50
100
150
Position (cM)
Chromosome 8
Dose (mM)
4e−06
1.6e−05
6e−04
0.001
0.0015
0.002
0.006
0.01
0.05

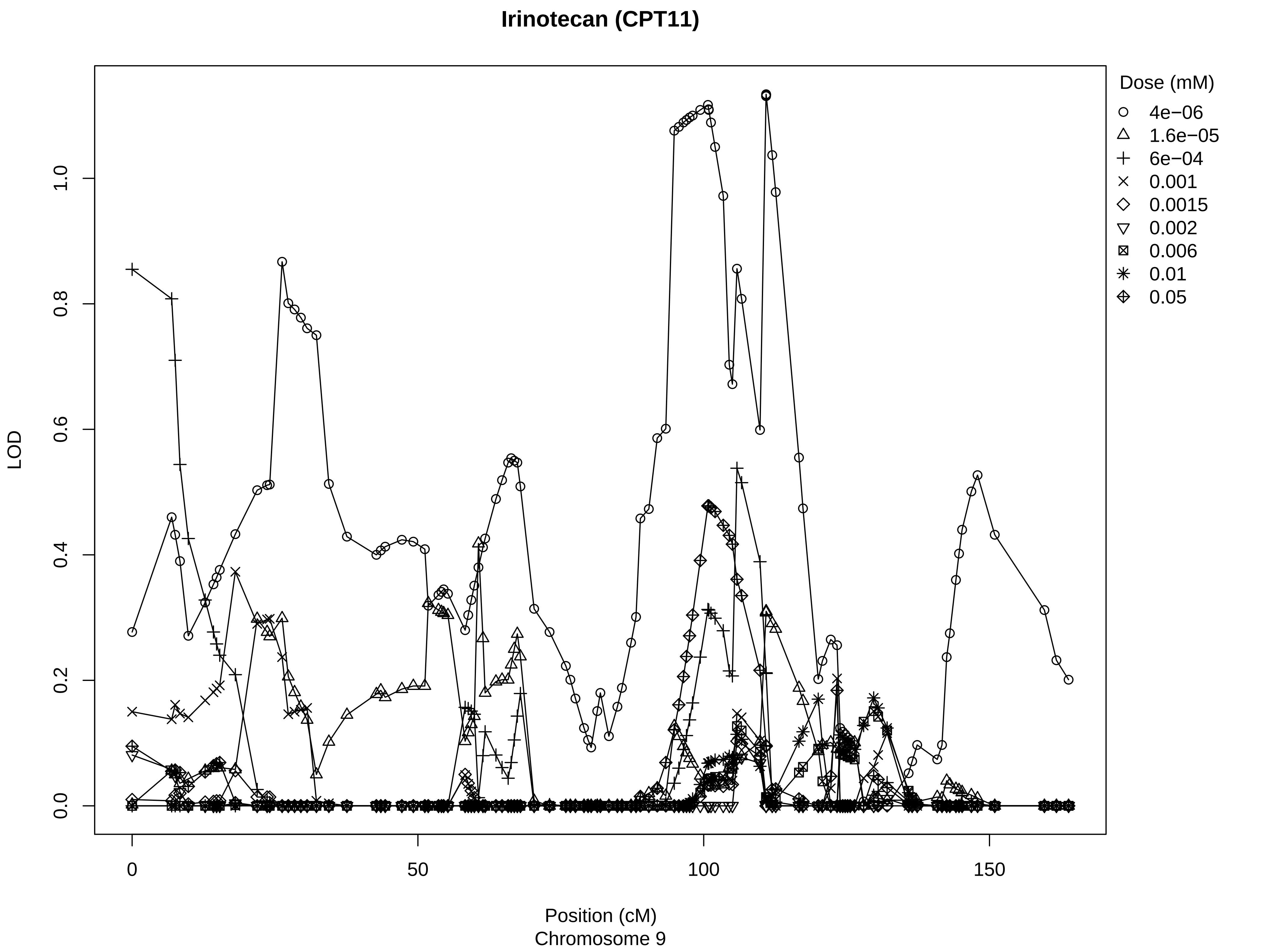

Irinotecan (CPT11)
LOD
0.0
0.2
0.4
0.6
0.8
1.0
0
50
100
150
Position (cM)
Chromosome 9
Dose (mM)
4e−06
1.6e−05
6e−04
0.001
0.0015
0.002
0.006
0.01
0.05

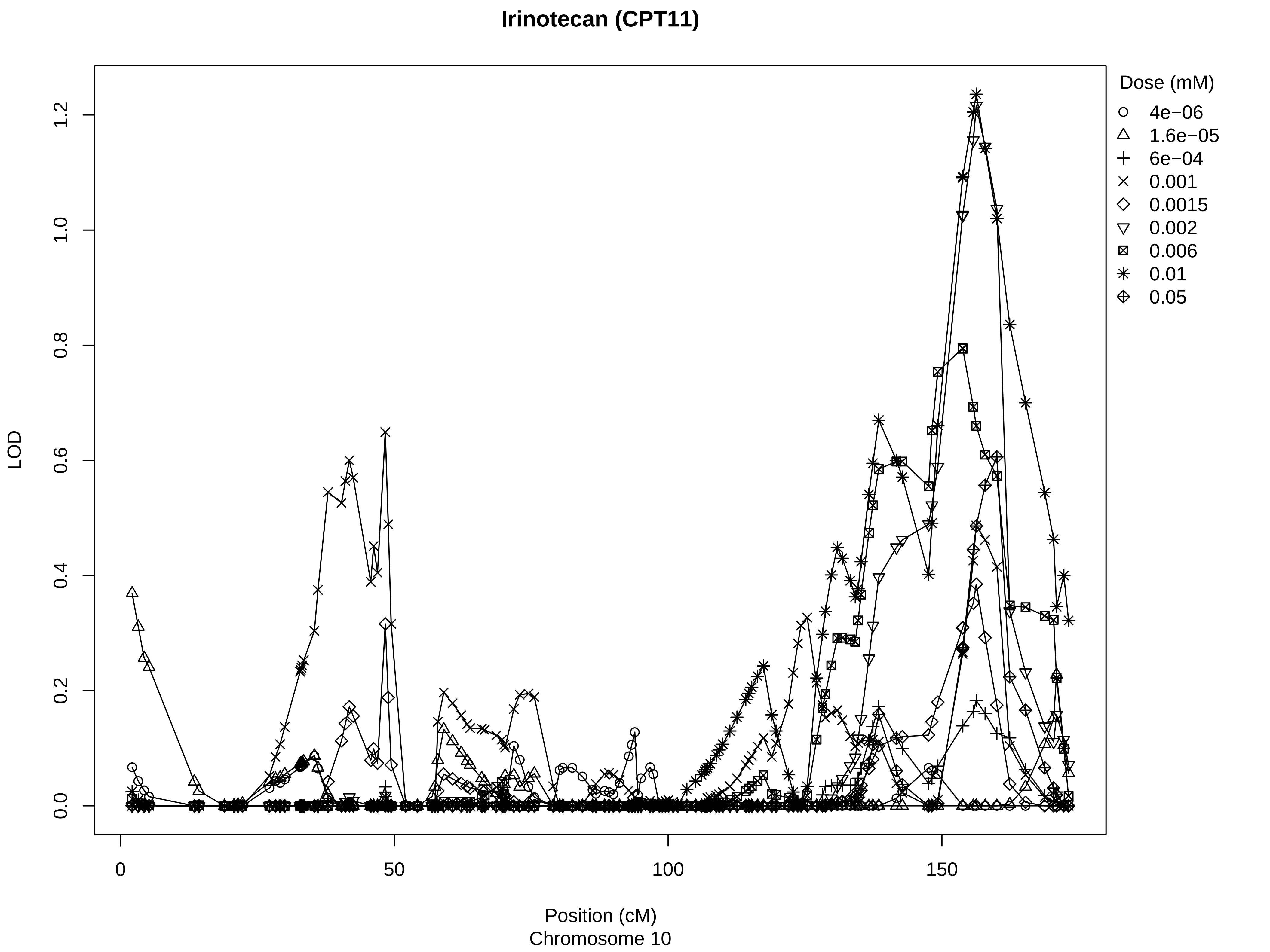
Irinotecan (CPT11)
LOD
Position (cM)
Chromosome 10
Dose (mM)
4e−06
1.6e−05
6e−04
0.001
0.0015
0.002
0.006
0.01
0.05
0.0
0.2
0.4
0.6
0.8
1.0
1.2
0
50
100
150

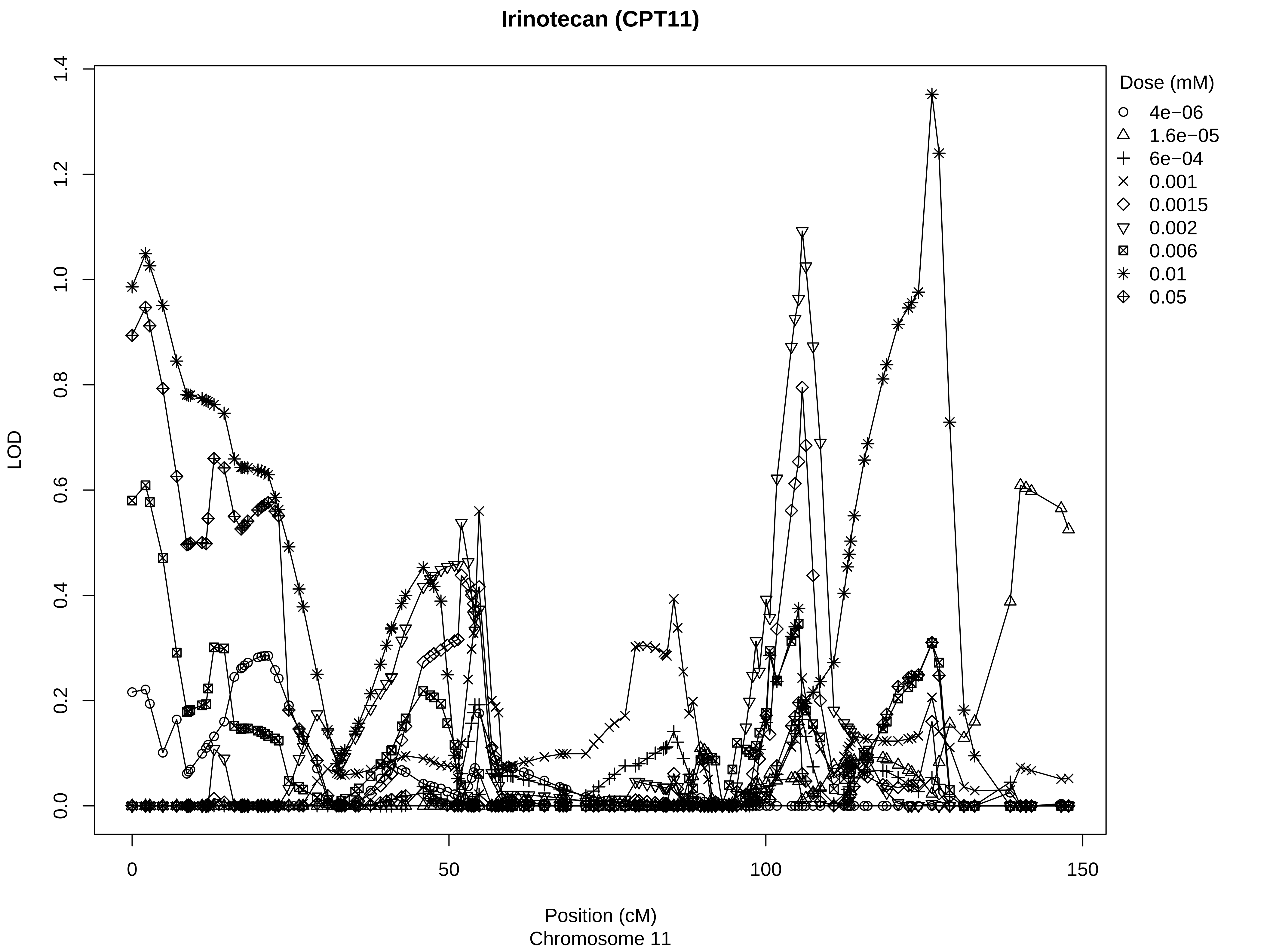

Irinotecan (CPT11)
LOD
0.0
0.2
0.4
0.6
0.8
1.0
1.2
1.4
0
50
100
150
Position (cM)
Chromosome 11
Dose (mM)
4e−06
1.6e−05
6e−04
0.001
0.0015
0.002
0.006
0.01
0.05

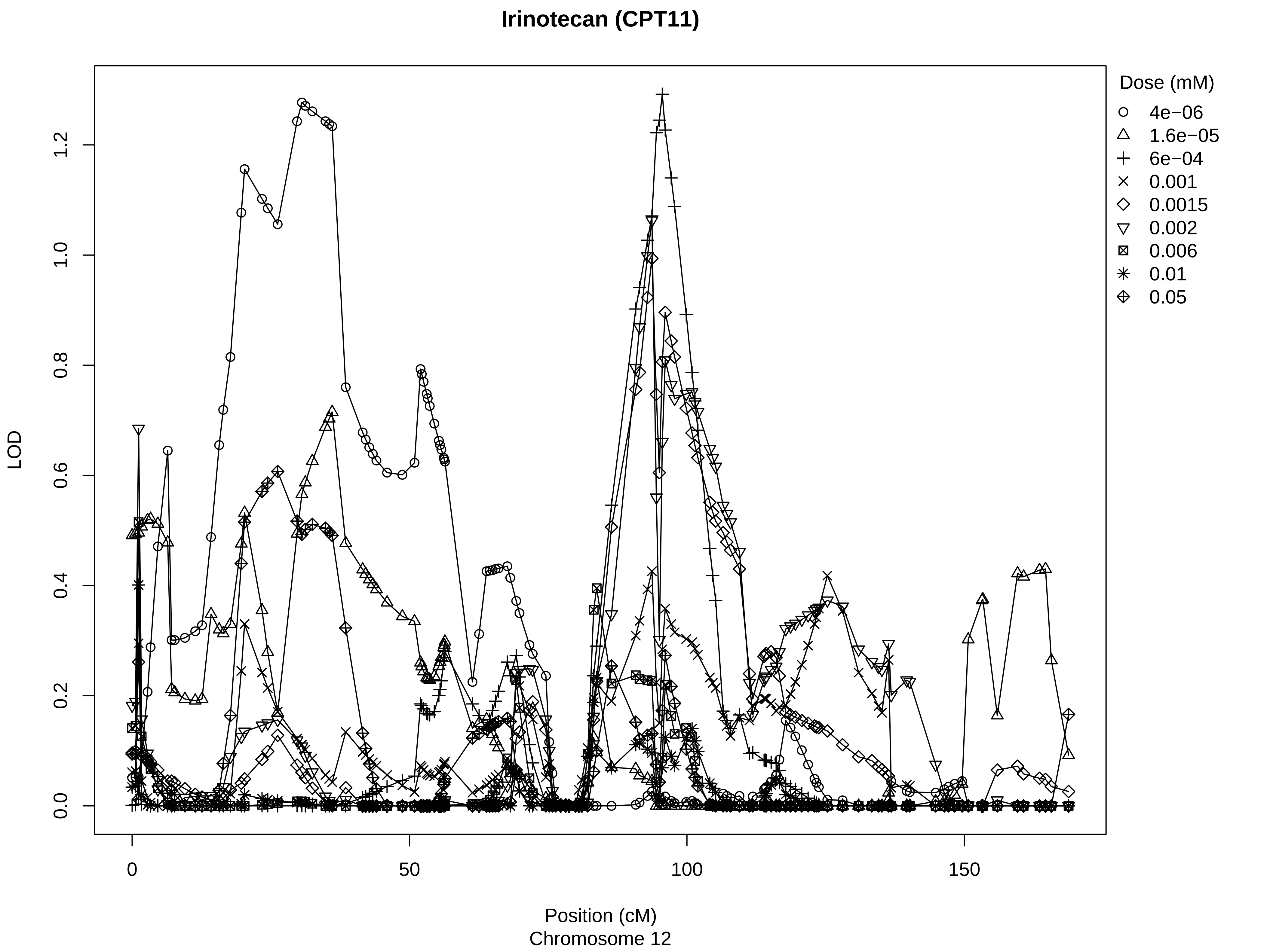

Irinotecan (CPT11)
LOD
0.0
0.2
0.4
0.6
0.8
1.0
1.2
0
50
100
150
Position (cM)
Chromosome 12
Dose (mM)
4e−06
1.6e−05
6e−04
0.001
0.0015
0.002
0.006
0.01
0.05

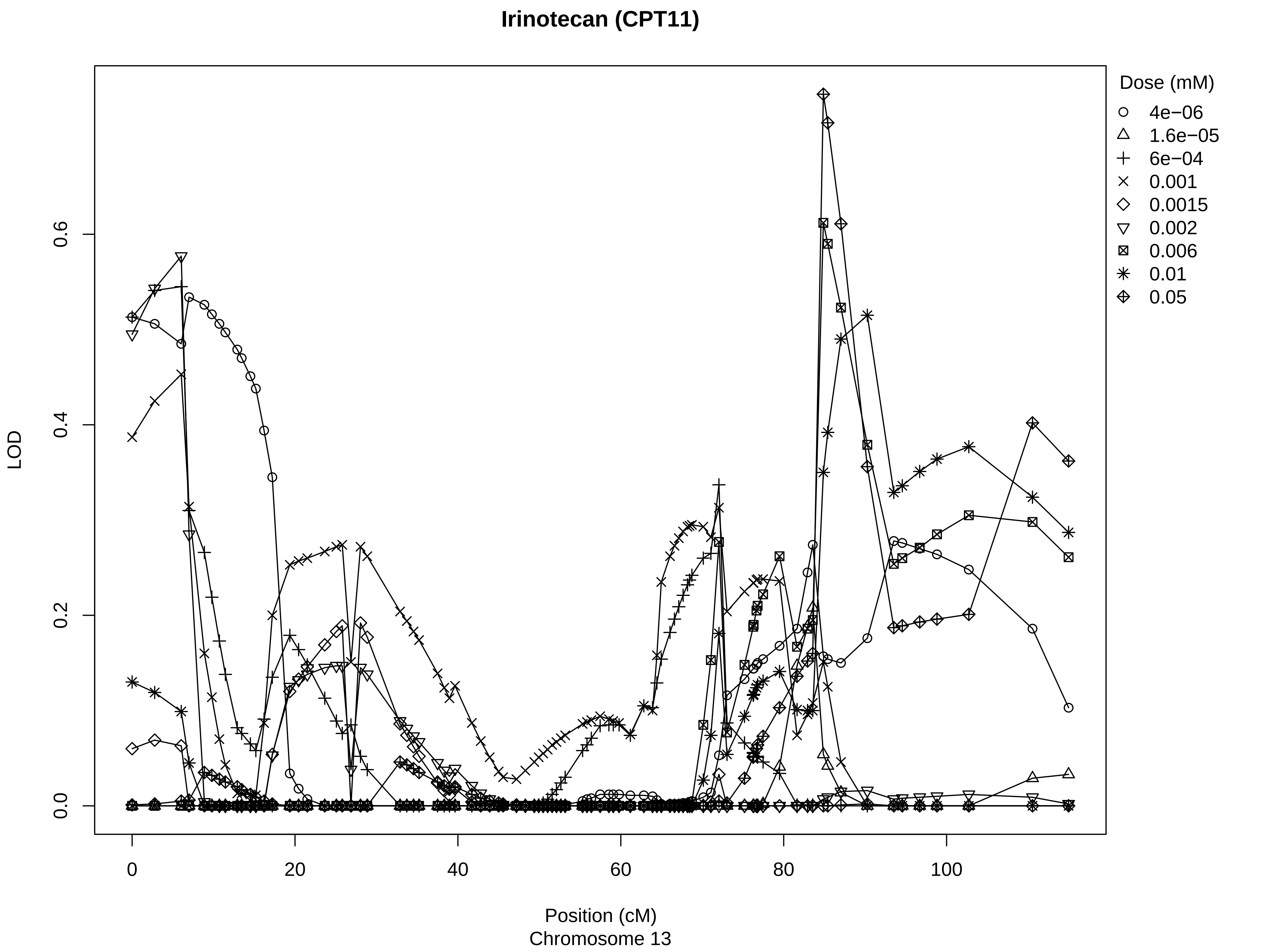

Irinotecan (CPT11)
Dose (mM)
4e−06
1.6e−05
6e−04
0.001
0.0015
0.002
0.006
0.01
0.05
LOD
0.0
0.2
0.4
0.6
0
20
40
60
80
100
Position (cM)
Chromosome 13

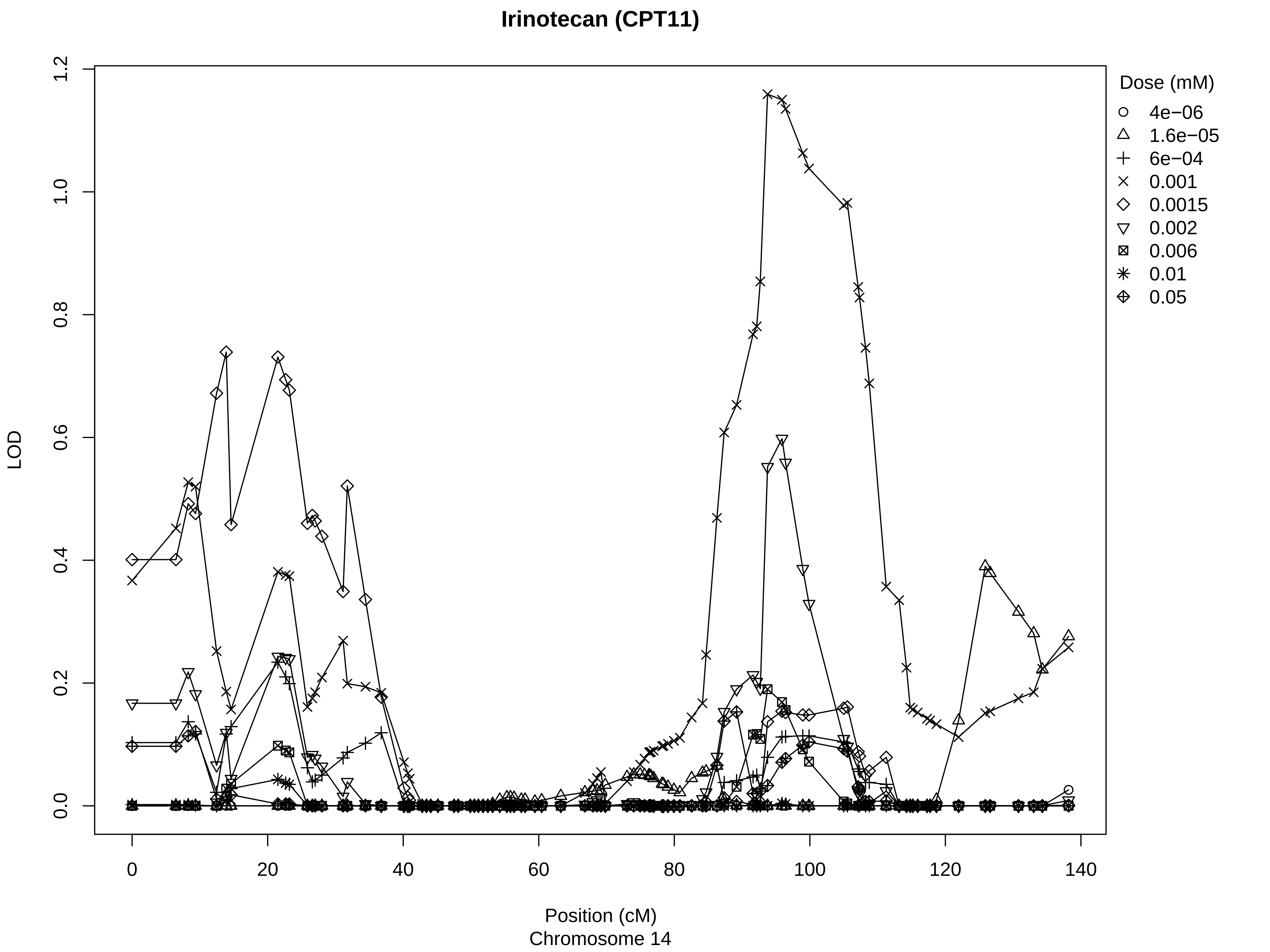

Irinotecan (CPT11)
LOD
0.0
0.2
0.4
0.6
0.8
1.0
1.2
0
20
40
60
80
100
120
140
Position (cM)
Chromosome 14
Dose (mM)
4e−06
1.6e−05
6e−04
0.001
0.0015
0.002
0.006
0.01
0.05

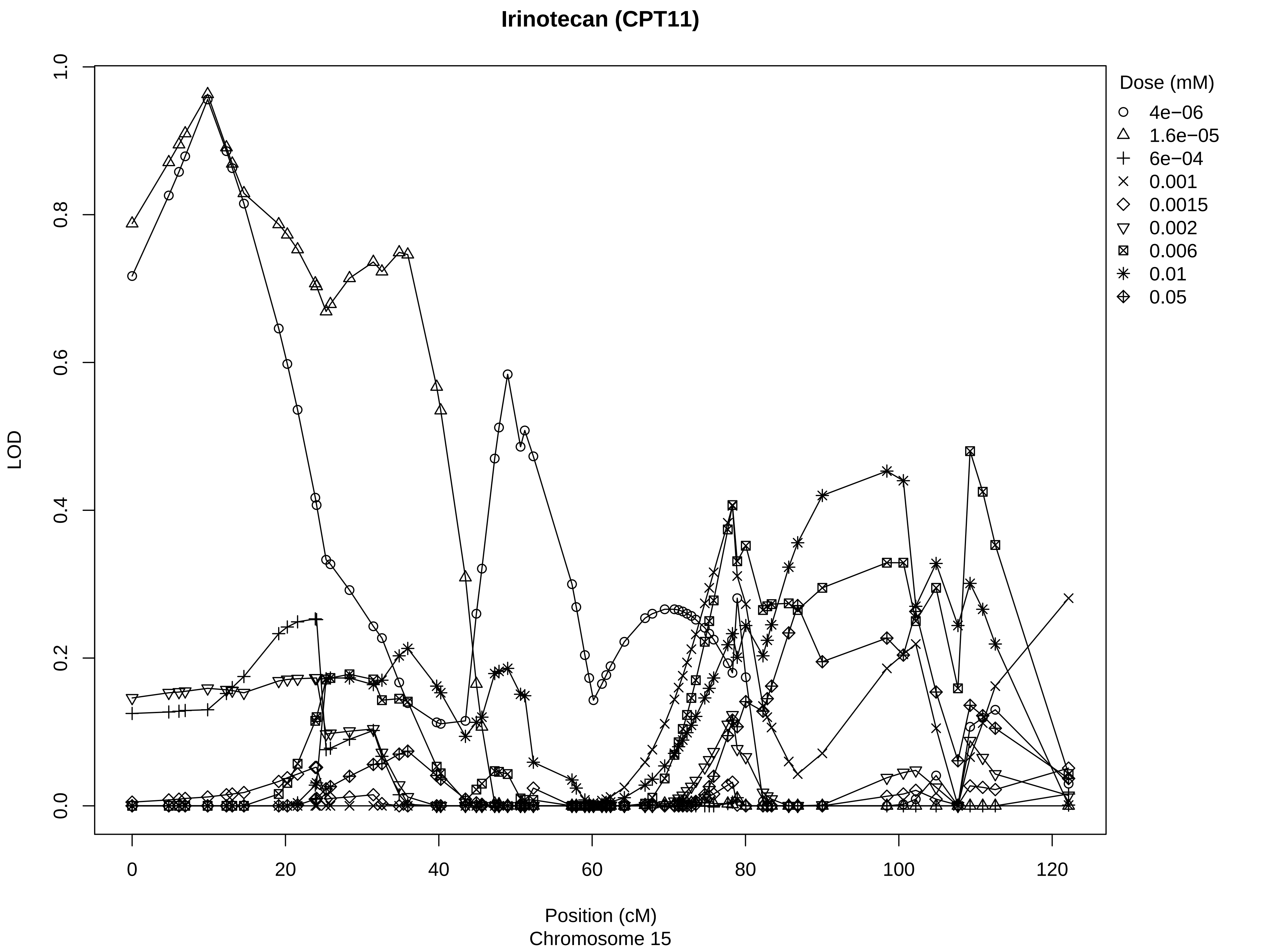

Irinotecan (CPT11)
LOD
0.0
0.2
0.4
0.6
0.8
1.0
0
20
40
60
80
100
120
Position (cM)
Chromosome 15
Dose (mM)
4e−06
1.6e−05
6e−04
0.001
0.0015
0.002
0.006
0.01
0.05

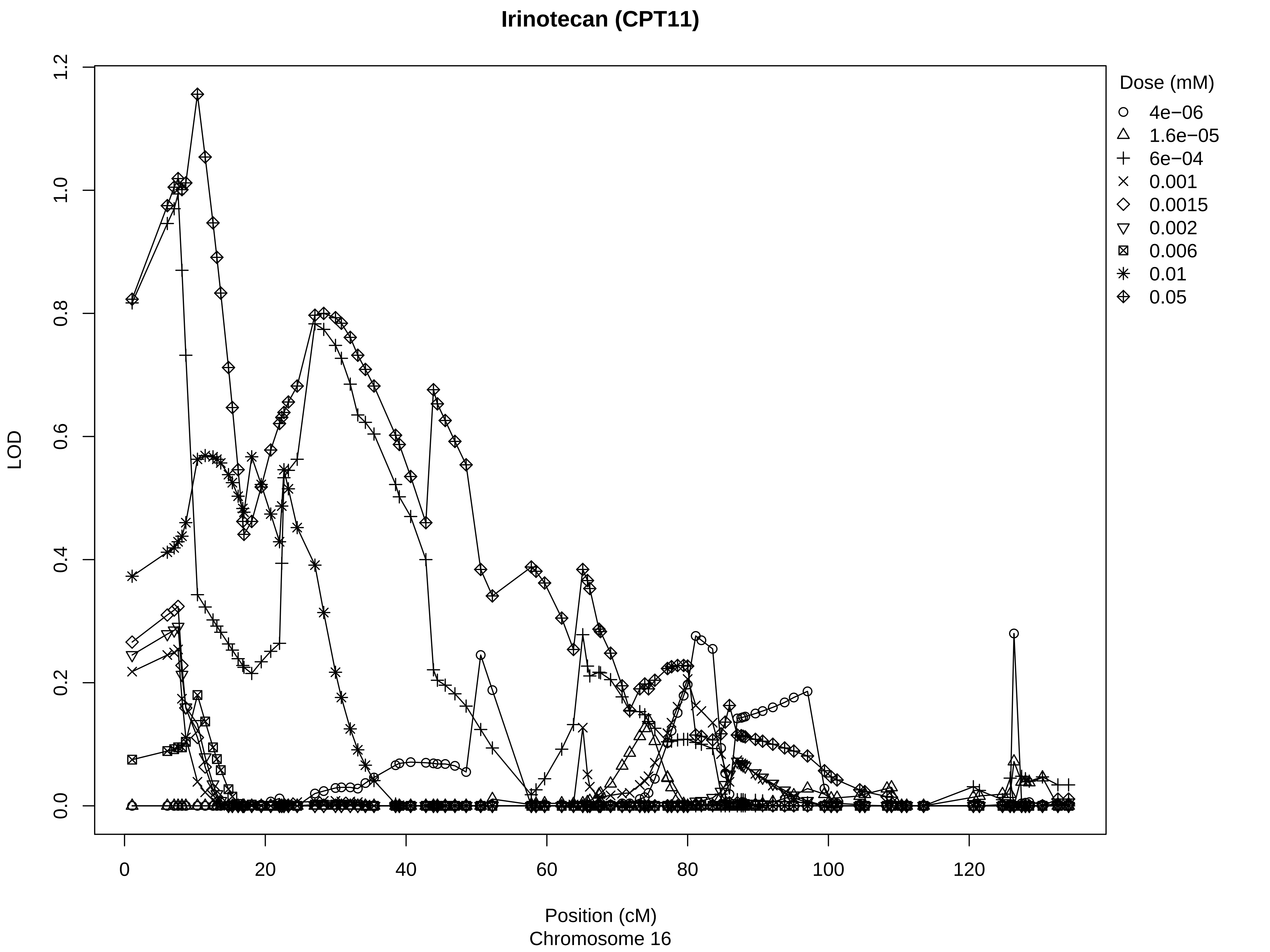

Irinotecan (CPT11)
LOD
0.0
0.2
0.4
0.6
0.8
1.0
1.2
0
20
40
60
80
100
120
Position (cM)
Chromosome 16
Dose (mM)
4e−06
1.6e−05
6e−04
0.001
0.0015
0.002
0.006
0.01
0.05

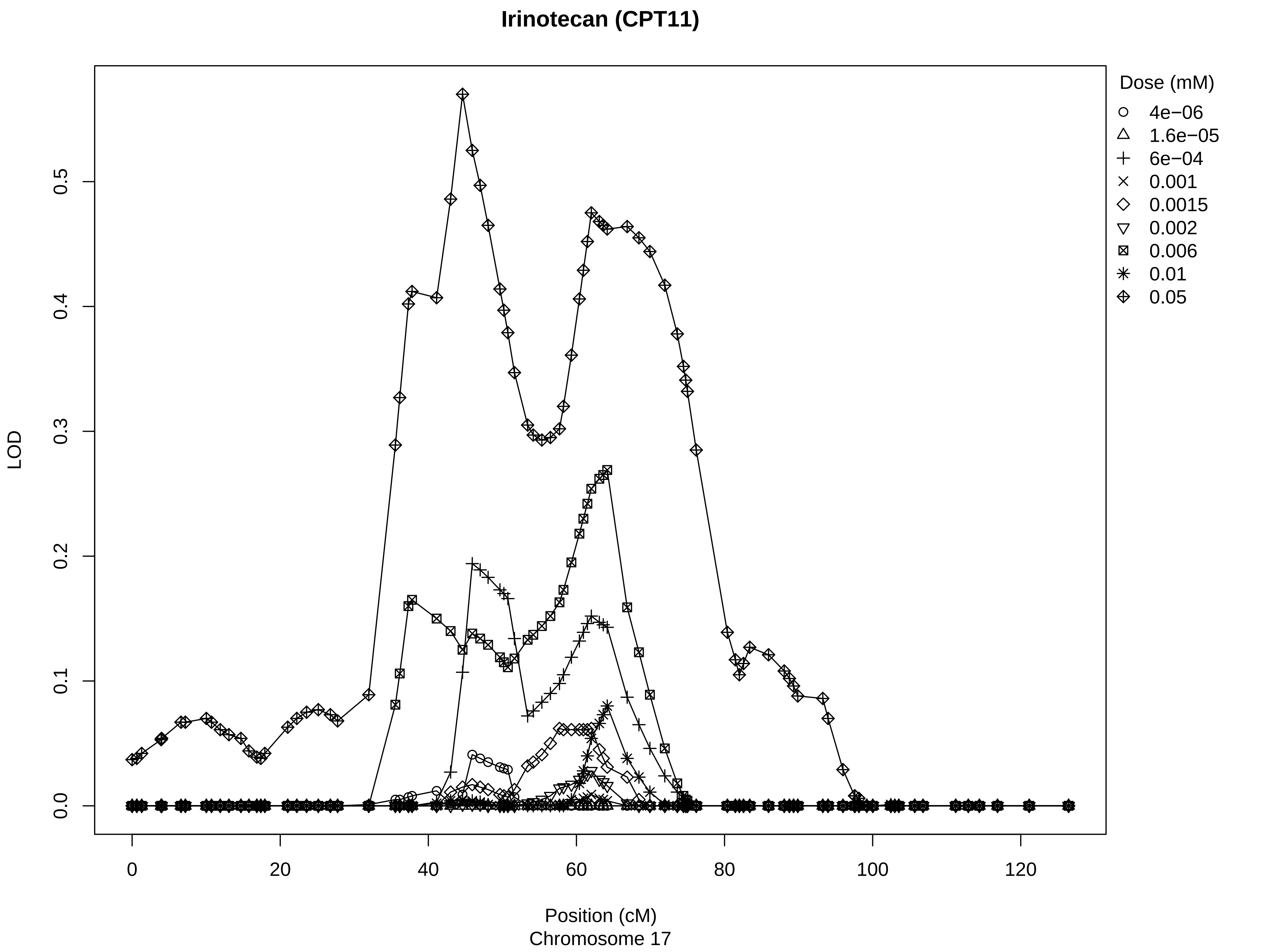
Irinotecan (CPT11)
LOD
0.0
0.1
0.2
0.3
0.4
0.5
0
20
40
60
80
100
120
Position (cM)
Chromosome 17
Dose (mM)
4e−06
1.6e−05
6e−04
0.001
0.0015
0.002
0.006
0.01
0.05

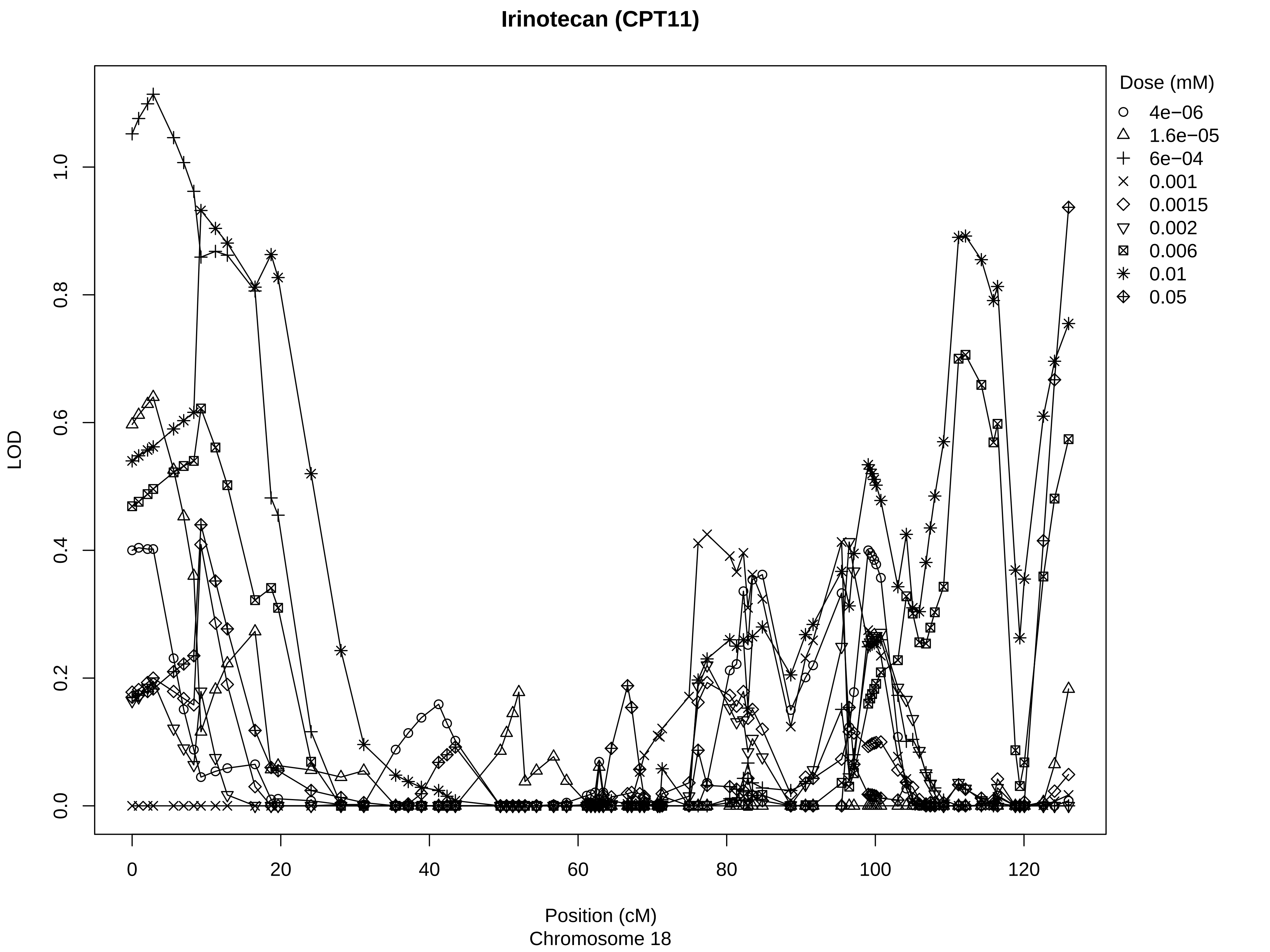

Irinotecan (CPT11)
LOD
Position (cM)
Chromosome 18
Dose (mM)
4e−06
1.6e−05
6e−04
0.001
0.0015
0.002
0.006
0.01
0.05
0.0
0.2
0.4
0.6
0.8
1.0
0
20
40
60
80
100
120

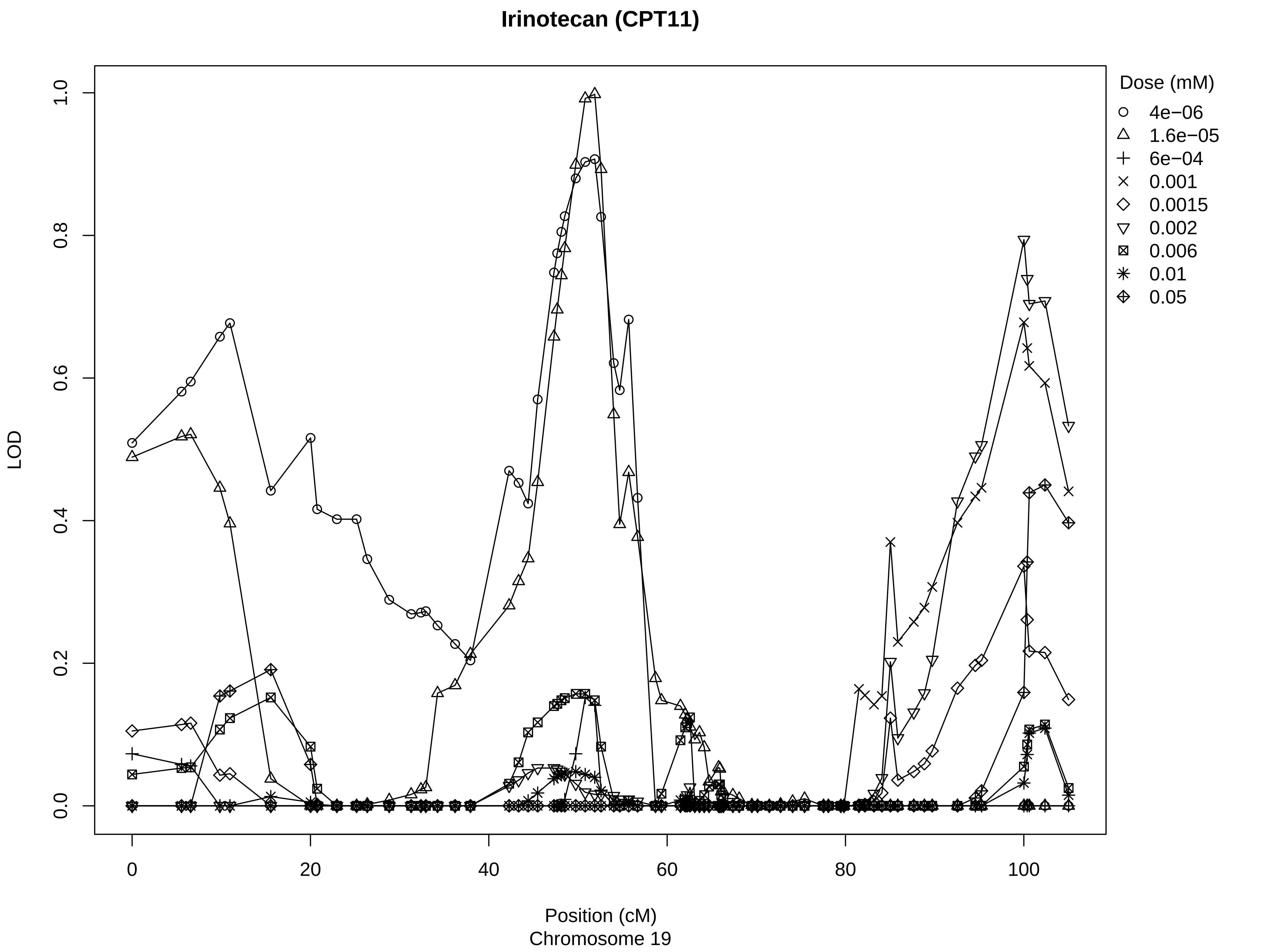

Irinotecan (CPT11)
Dose (mM)
4e−06
1.6e−05
6e−04
0.001
0.0015
0.002
0.006
0.01
0.05
LOD
0.0
0.2
0.4
0.6
0.8
1.0
0
20
40
60
80
100
Position (cM)
Chromosome 19

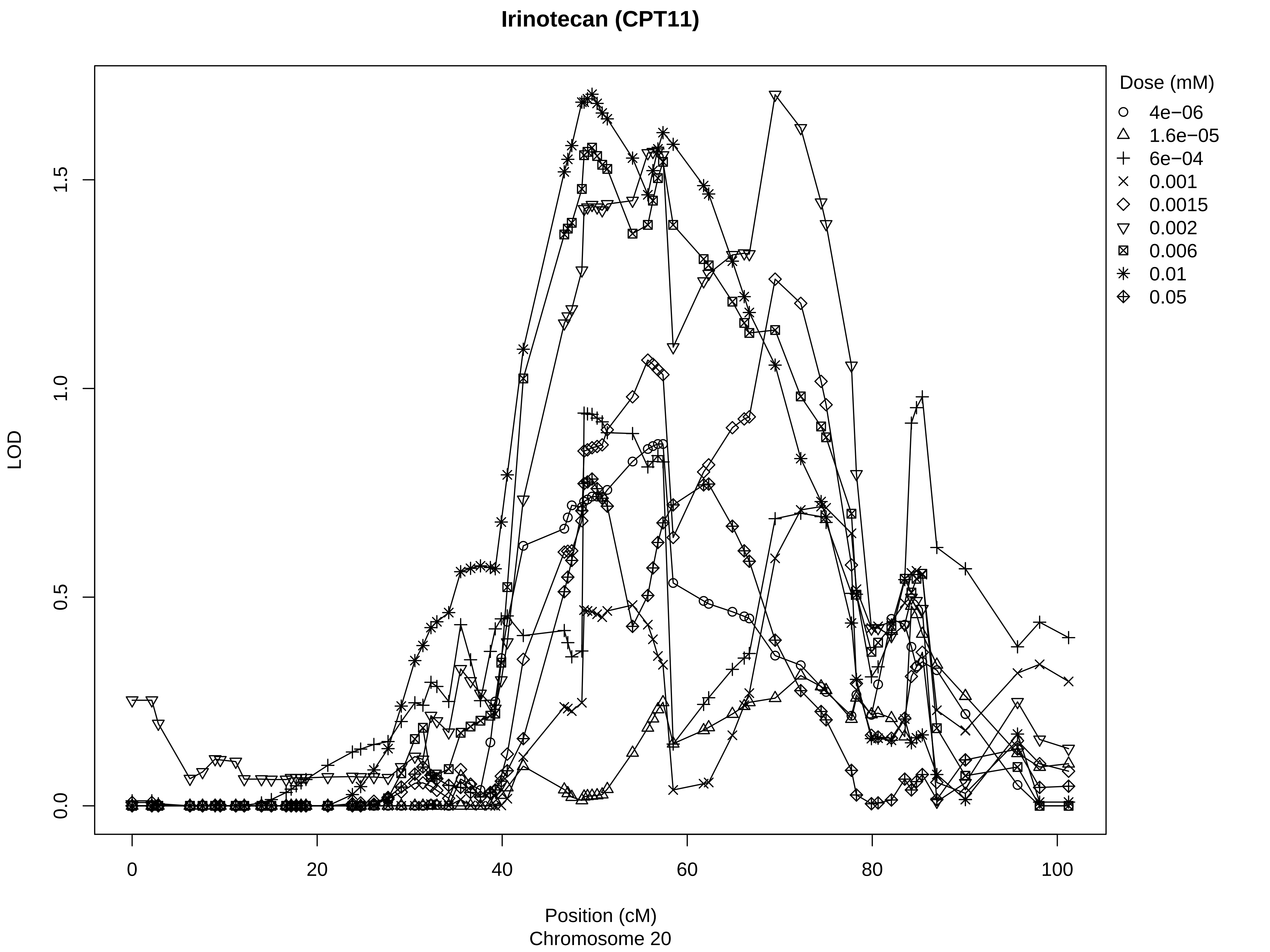

Irinotecan (CPT11)
Dose (mM)
4e−06
1.6e−05
6e−04
0.001
0.0015
0.002
0.006
0.01
0.05
LOD
0.0
0.5
1.0
1.5
0
20
40
60
80
100
Position (cM)
Chromosome 20

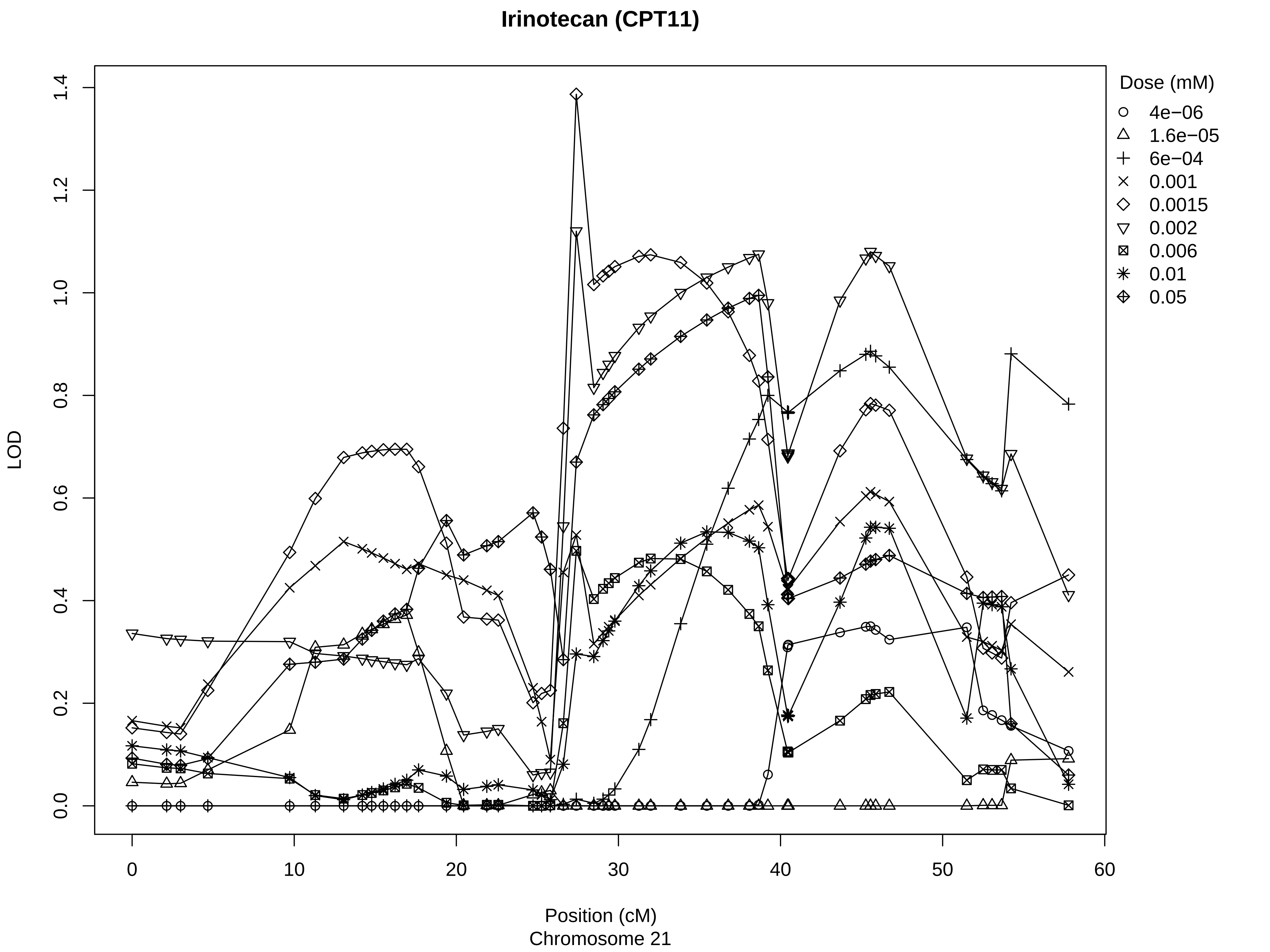

Irinotecan (CPT11)
Dose (mM)
4e−06
1.6e−05
6e−04
0.001
0.0015
0.002
0.006
0.01
0.05
LOD
0.0
0.2
0.4
0.6
0.8
1.0
1.2
1.4
0
10
20
30
40
50
60
Position (cM)
Chromosome 21

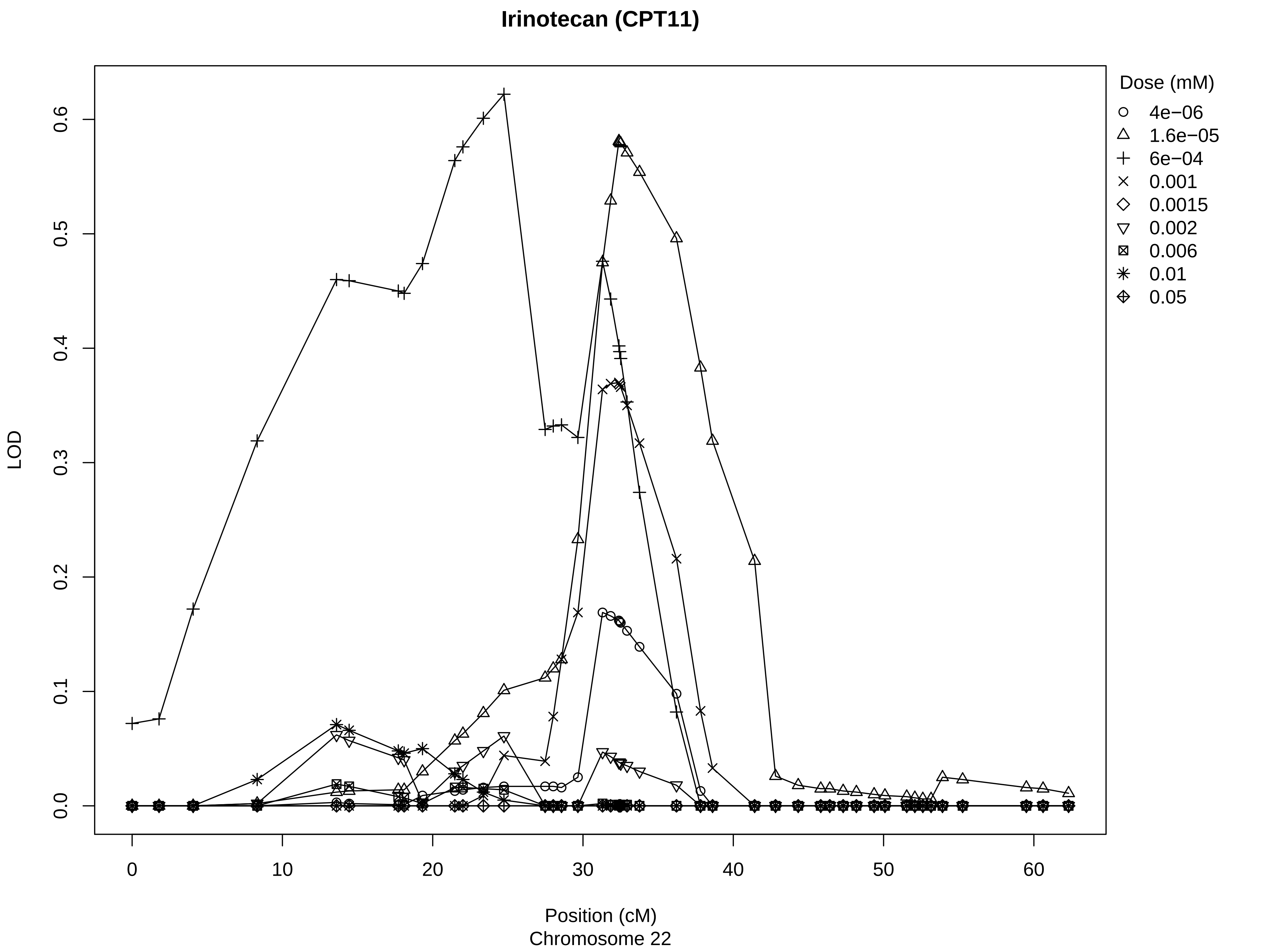
Irinotecan (CPT11)
Dose (mM)
4e−06
1.6e−05
6e−04
0.001
0.0015
0.002
0.006
0.01
0.05
LOD
0.0
0.1
0.2
0.3
0.4
0.5
0.6
0
10
20
30
40
50
60
Position (cM)
Chromosome 22

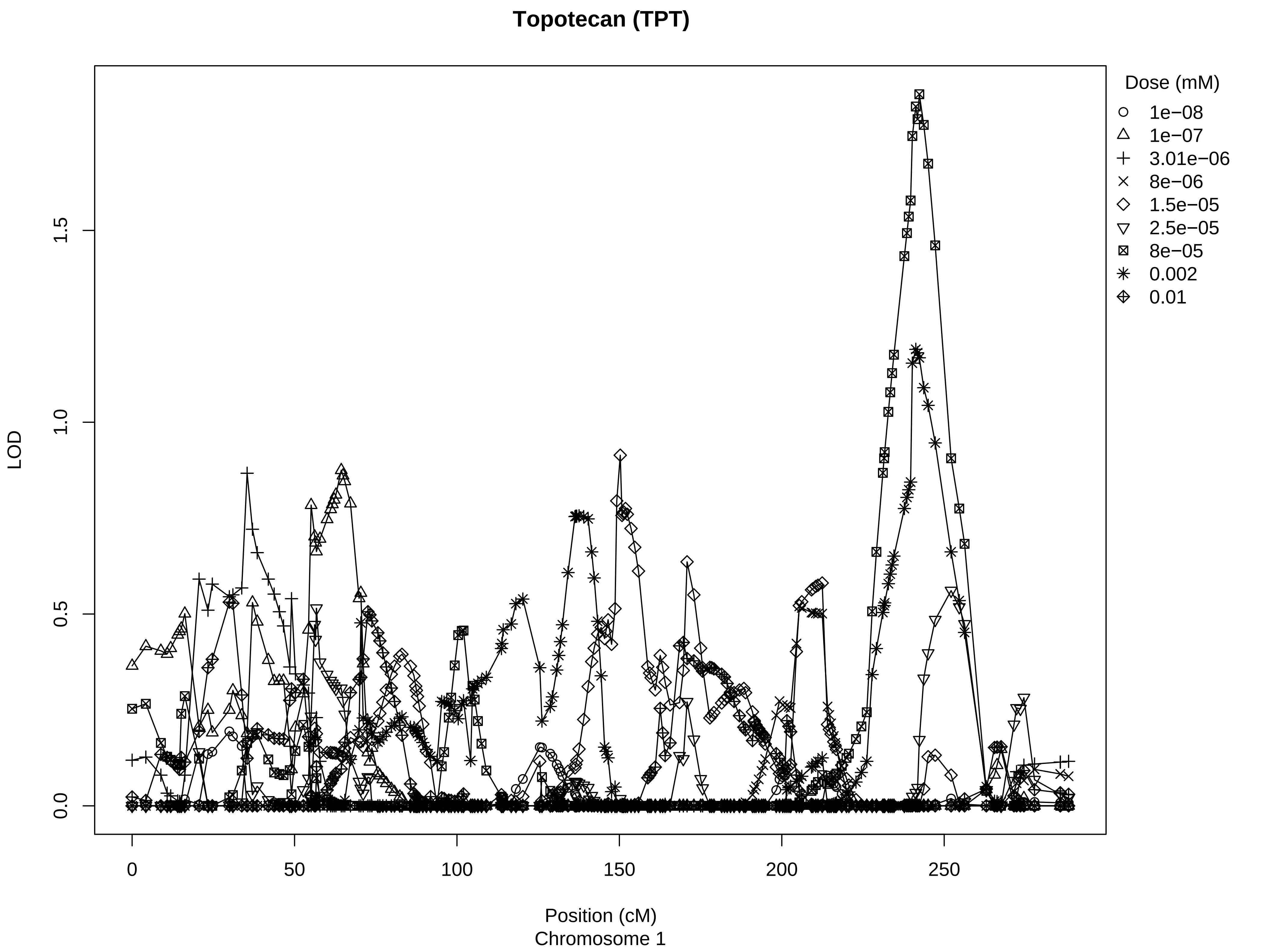

Topotecan (TPT)
Dose (mM)
1e−08
1e−07
3.01e−06
8e−06
1.5e−05
2.5e−05
8e−05
0.002
0.01
LOD
0.0
0.5
1.0
1.5
0
50
100
150
200
250
Position (cM)
Chromosome 1

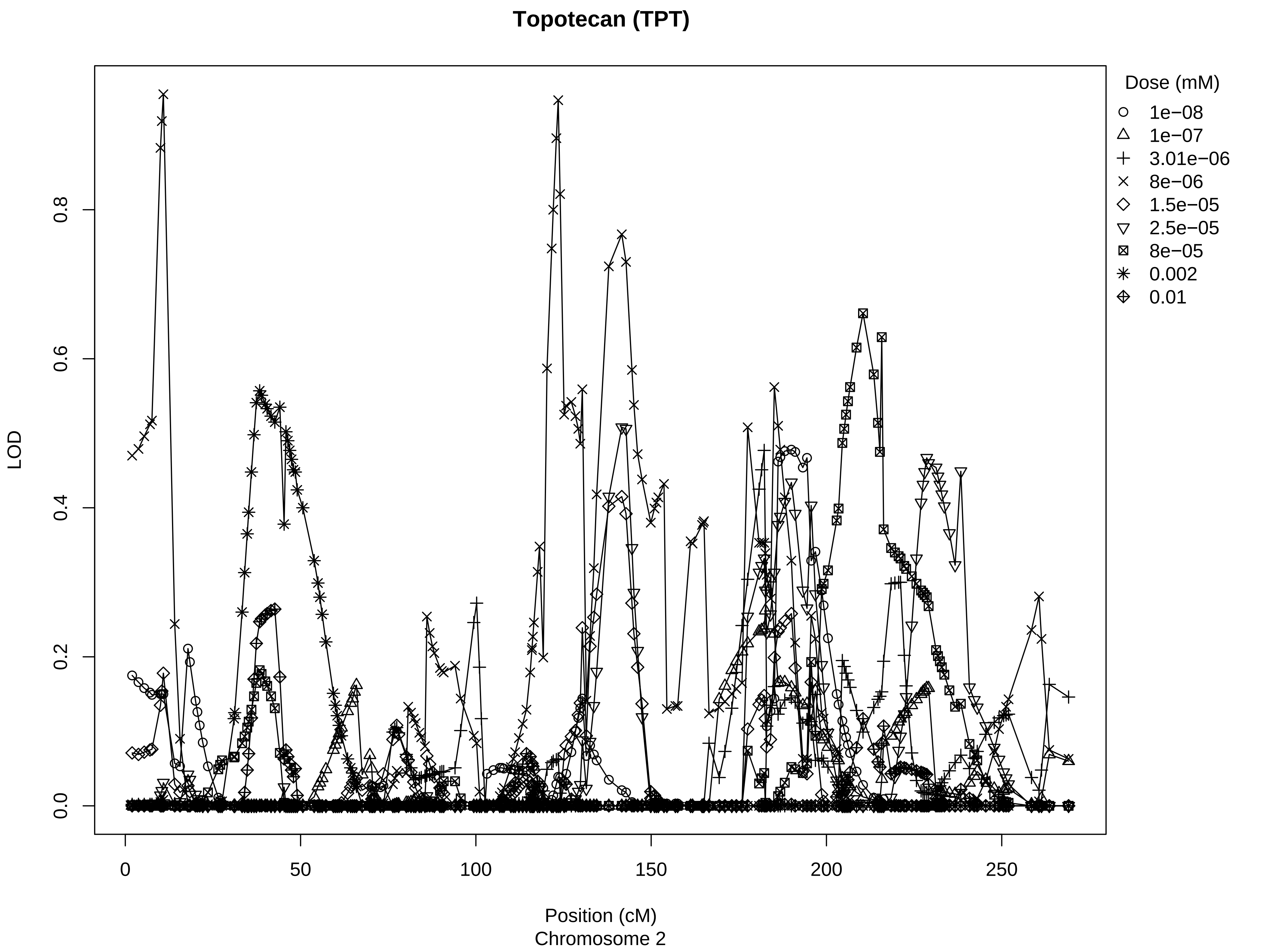
Topotecan (TPT)
LOD
0.0
0.2
0.4
0.6
0.8
0
50
100
150
200
250
Position (cM)
Chromosome 2
Dose (mM)
1e−08
1e−07
3.01e−06
8e−06
1.5e−05
2.5e−05
8e−05
0.002
0.01

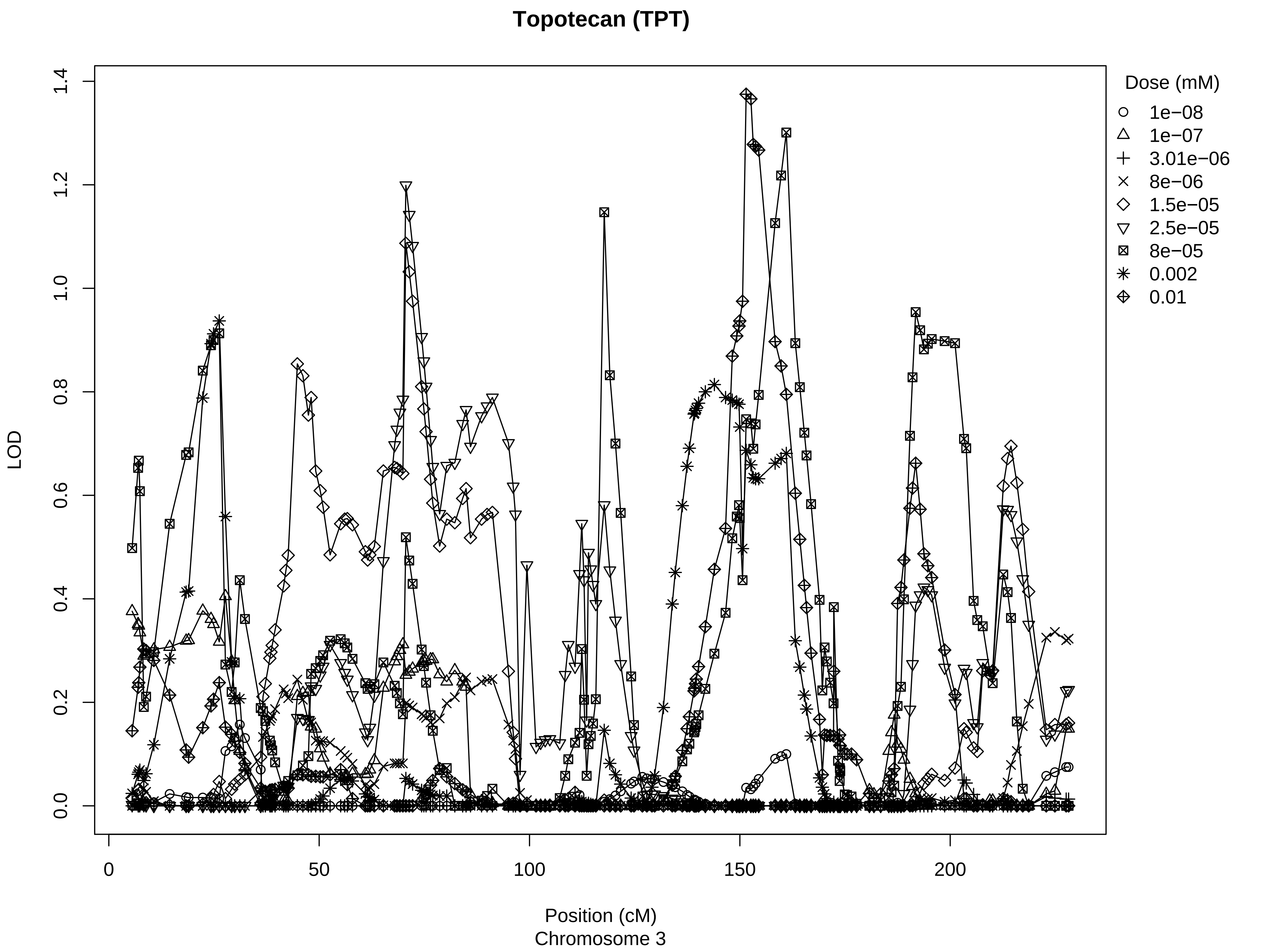

Topotecan (TPT)
LOD
0.0
0.2
0.4
0.6
0.8
1.0
1.2
1.4
0
50
100
150
200
Position (cM)
Chromosome 3
Dose (mM)
1e−08
1e−07
3.01e−06
8e−06
1.5e−05
2.5e−05
8e−05
0.002
0.01

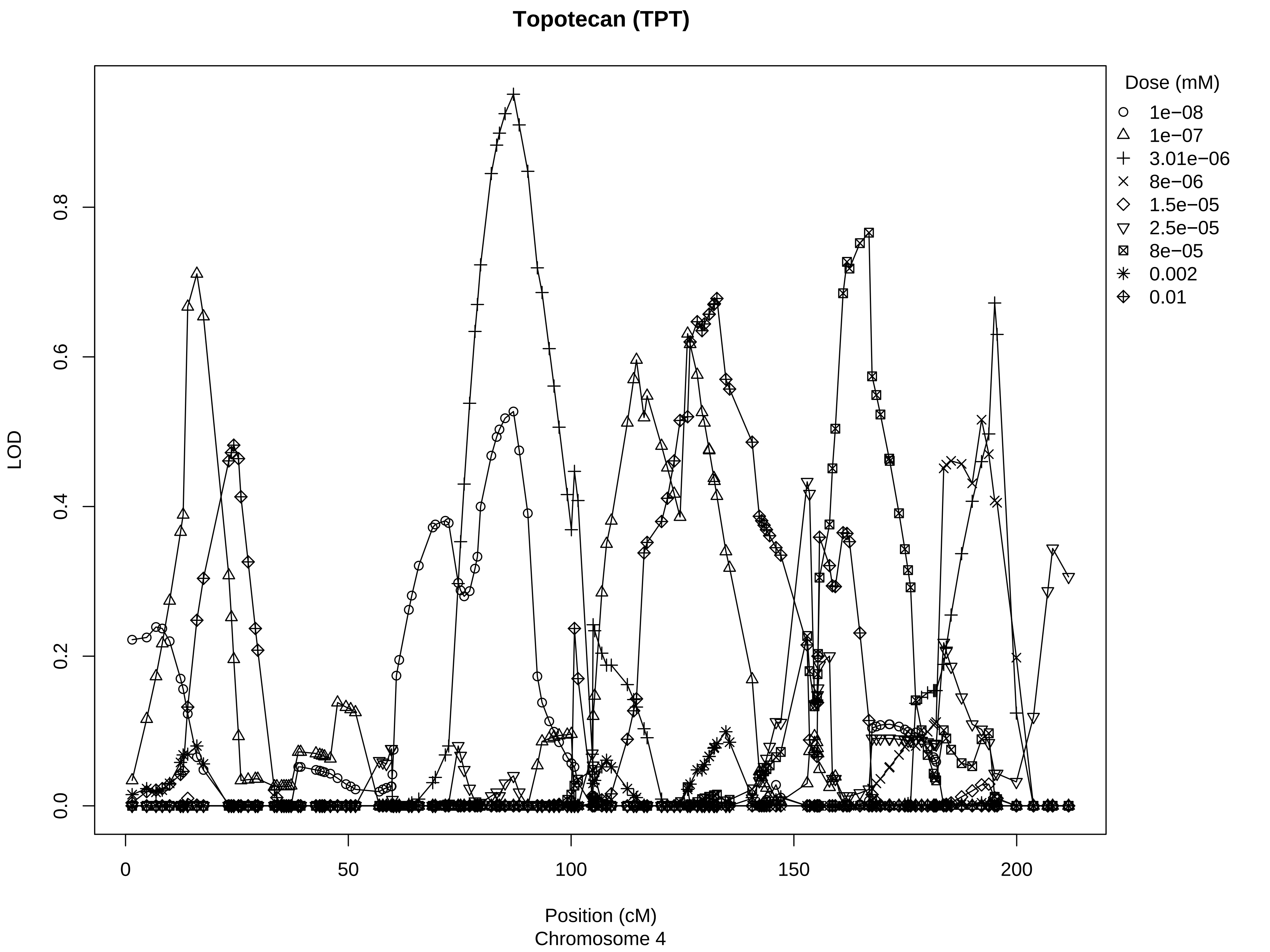

Topotecan (TPT)
Dose (mM)
1e−08
1e−07
3.01e−06
8e−06
1.5e−05
2.5e−05
8e−05
0.002
0.01
LOD
0.0
0.2
0.4
0.6
0.8
0
50
100
150
200
Position (cM)
Chromosome 4

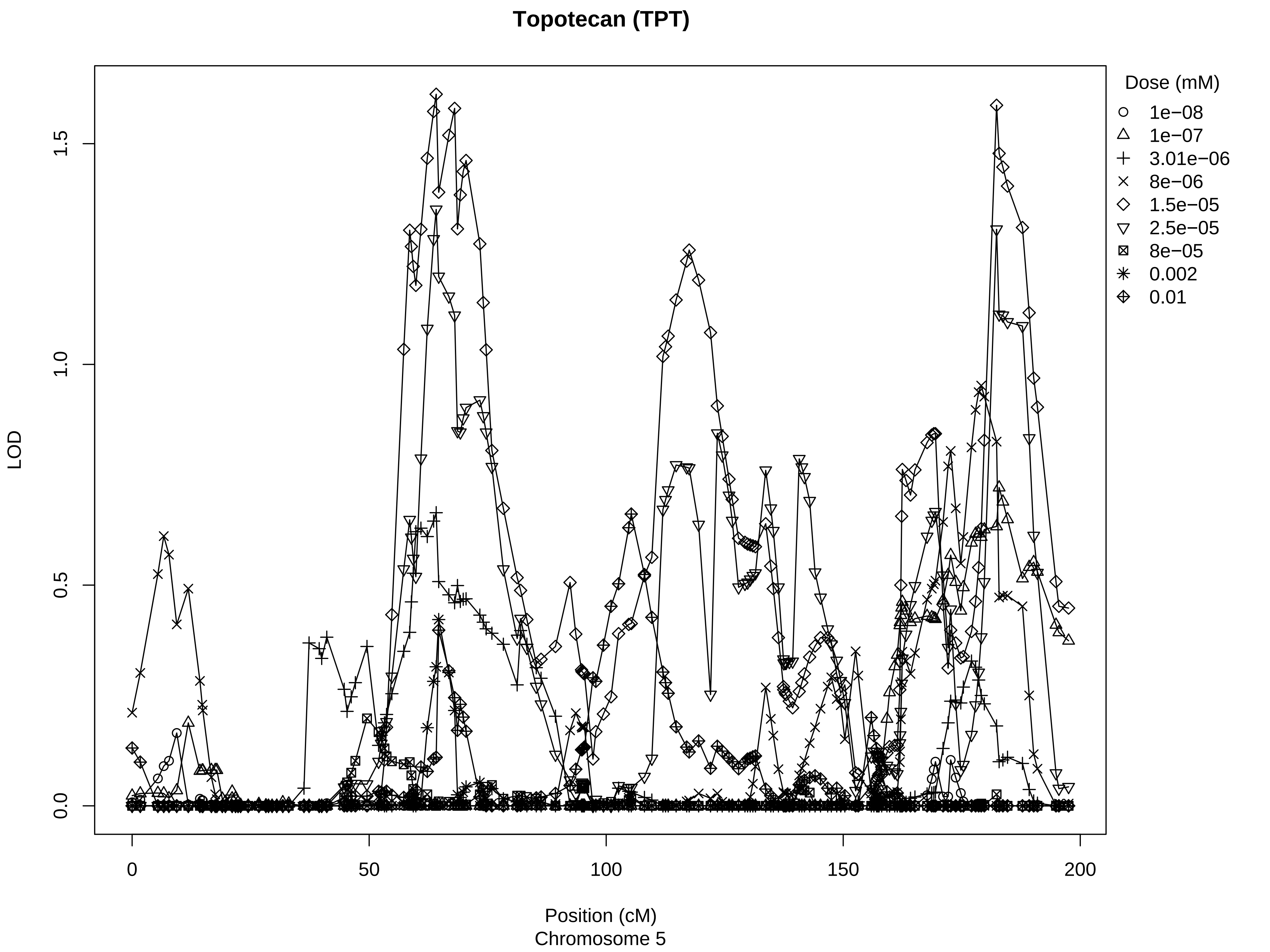

Topotecan (TPT)
LOD
0.0
0.5
1.0
1.5
0
50
100
150
200
Position (cM)
Chromosome 5
Dose (mM)
1e−08
1e−07
3.01e−06
8e−06
1.5e−05
2.5e−05
8e−05
0.002
0.01

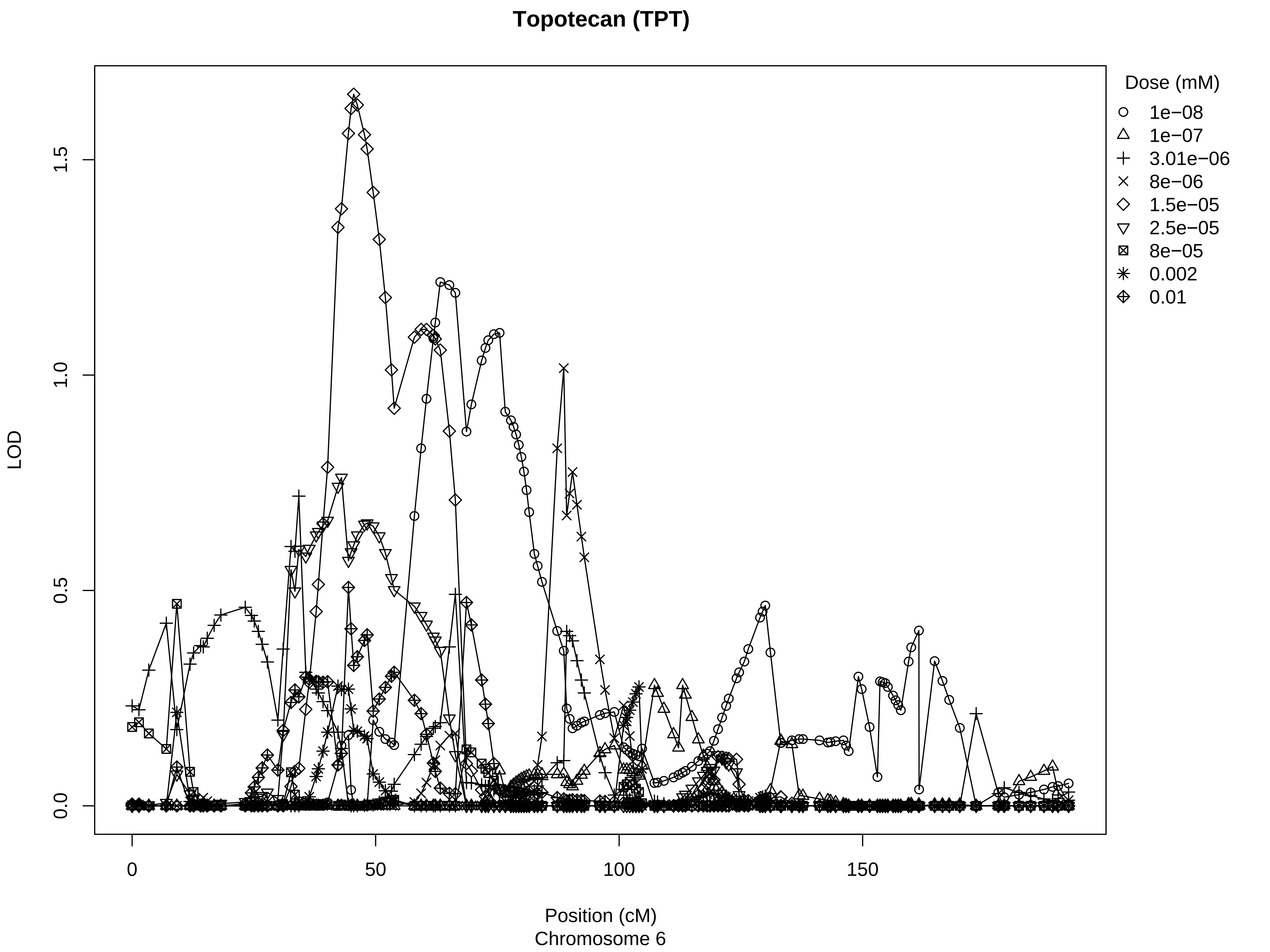
Topotecan (TPT)
LOD
1.5
1.0
0.5
0.0
0
50
100
150
Position (cM)
Chromosome 6
Dose (mM)
1e−08
1e−07
3.01e−06
8e−06
1.5e−05
2.5e−05
8e−05
0.002
0.01

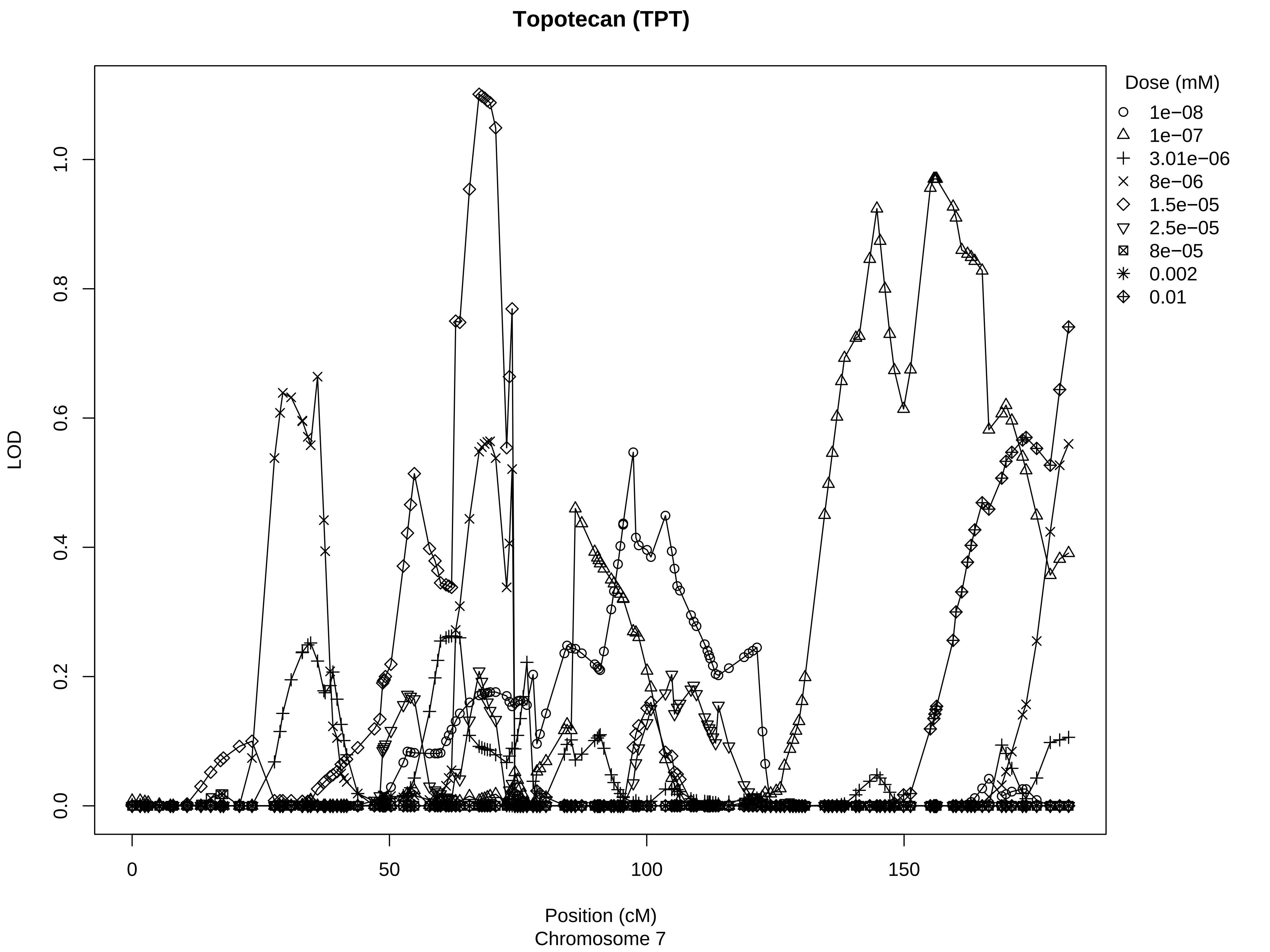

Topotecan (TPT)
LOD
0.0
0.2
0.4
0.6
0.8
1.0
0
50
100
150
Position (cM)
Chromosome 7
Dose (mM)
1e−08
1e−07
3.01e−06
8e−06
1.5e−05
2.5e−05
8e−05
0.002
0.01

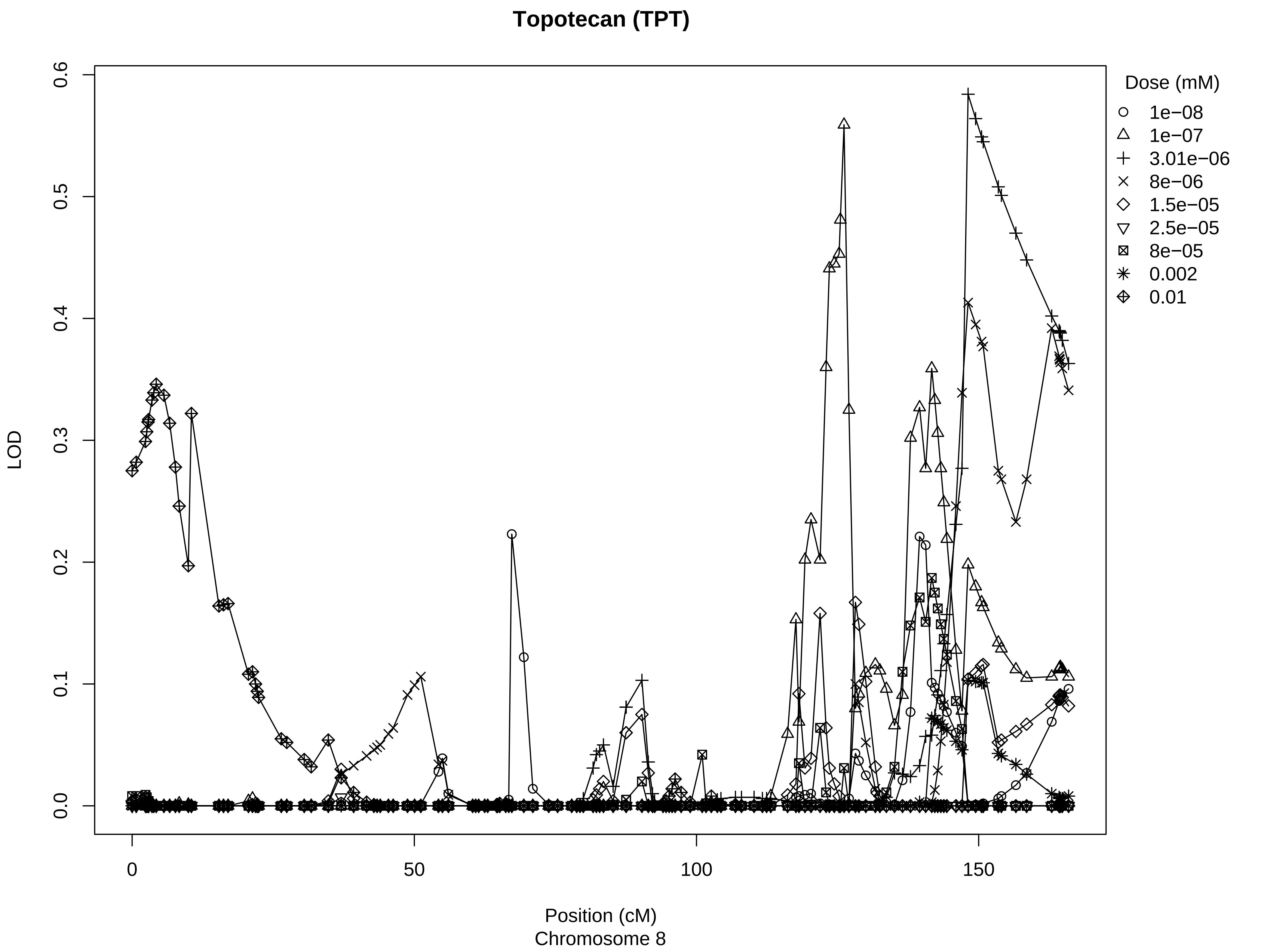

Topotecan (TPT)
LOD
0.0
0.1
0.2
0.3
0.4
0.5
0.6
0
50
100
150
Position (cM)
Chromosome 8
Dose (mM)
1e−08
1e−07
3.01e−06
8e−06
1.5e−05
2.5e−05
8e−05
0.002
0.01

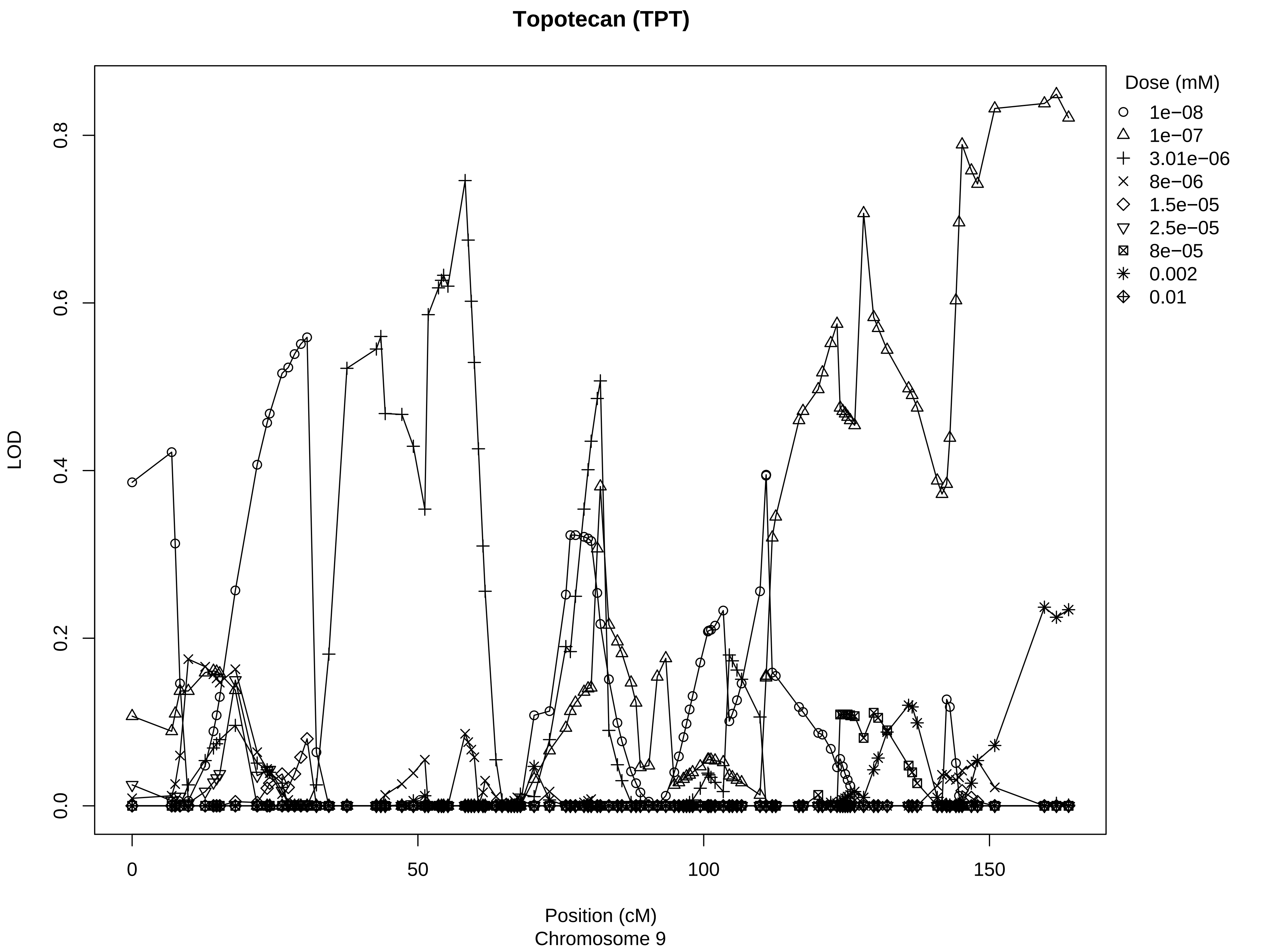

Topotecan (TPT)
LOD
0.0
0.2
0.4
0.6
0.8
0
50
100
150
Position (cM)
Chromosome 9
Dose (mM)
1e−08
1e−07
3.01e−06
8e−06
1.5e−05
2.5e−05
8e−05
0.002
0.01

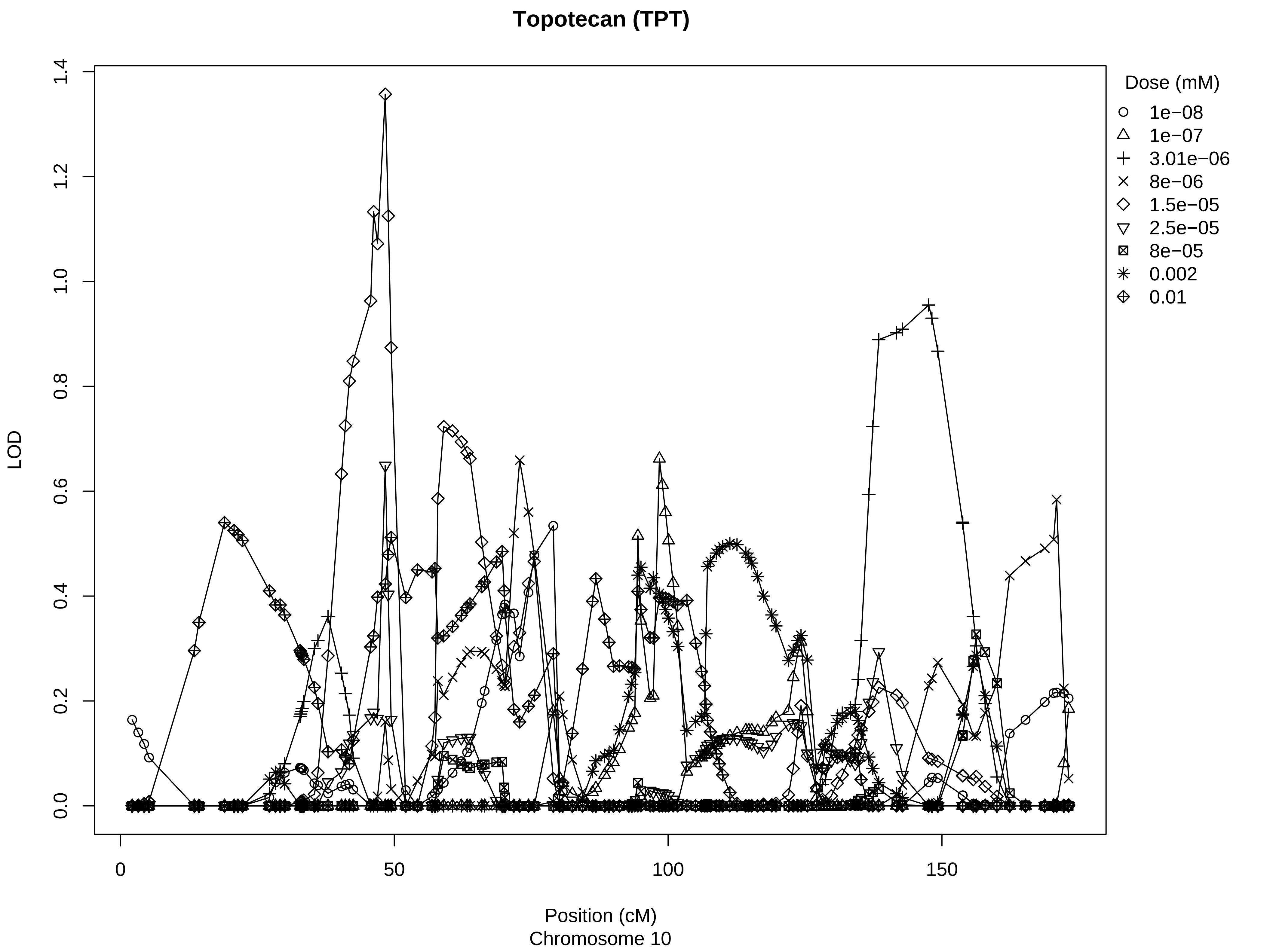
Topotecan (TPT)
LOD
0.0
0.2
0.4
0.6
0.8
1.0
1.2
1.4
0
50
100
150
Position (cM)
Chromosome 10
Dose (mM)
1e−08
1e−07
3.01e−06
8e−06
1.5e−05
2.5e−05
8e−05
0.002
0.01

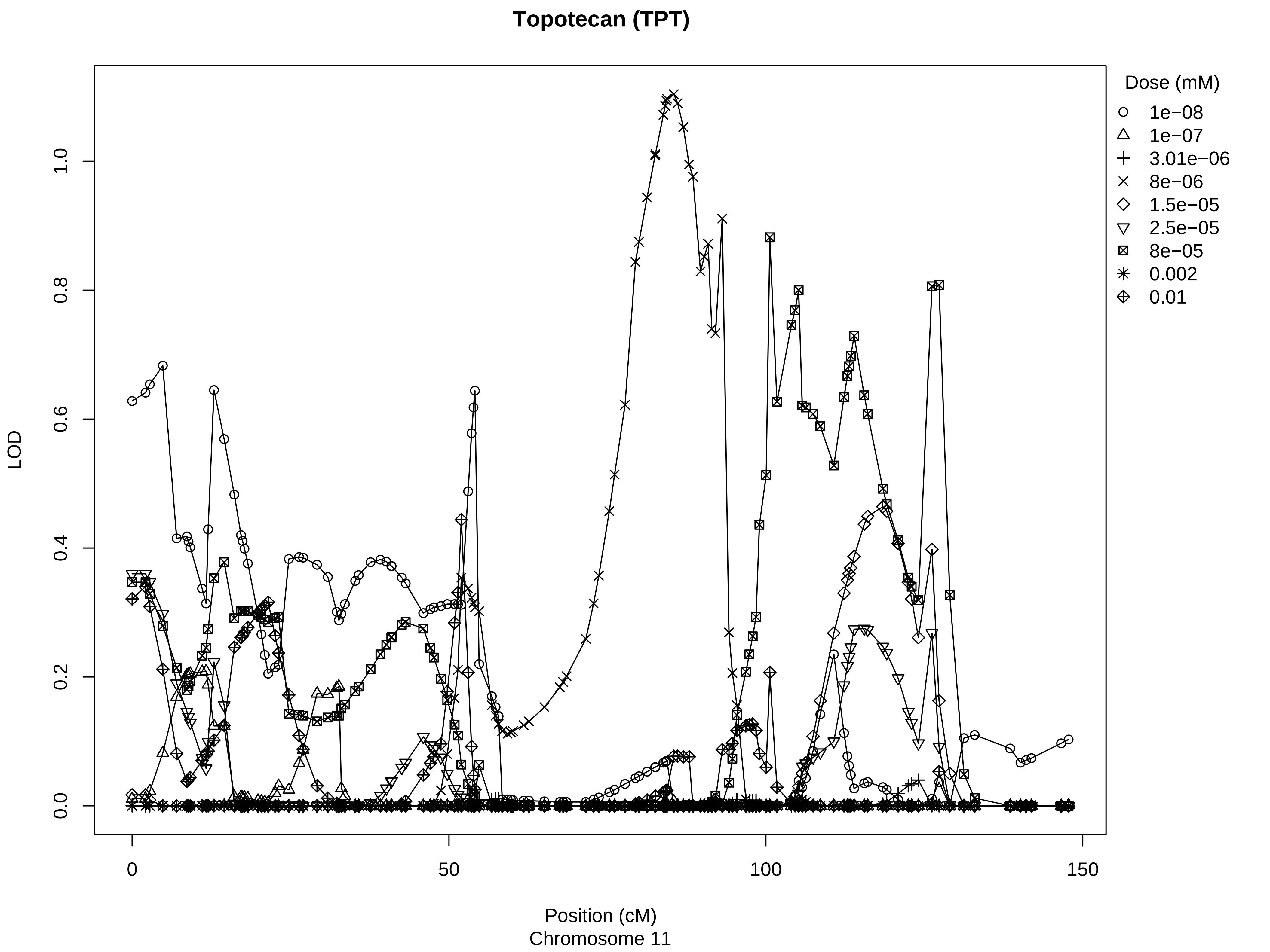
Topotecan (TPT)
Dose (mM)
1e−08
1e−07
3.01e−06
8e−06
1.5e−05
2.5e−05
8e−05
0.002
0.01
LOD
0.0
0.2
0.4
0.6
0.8
1.0
0
50
100
150
Position (cM)
Chromosome 11

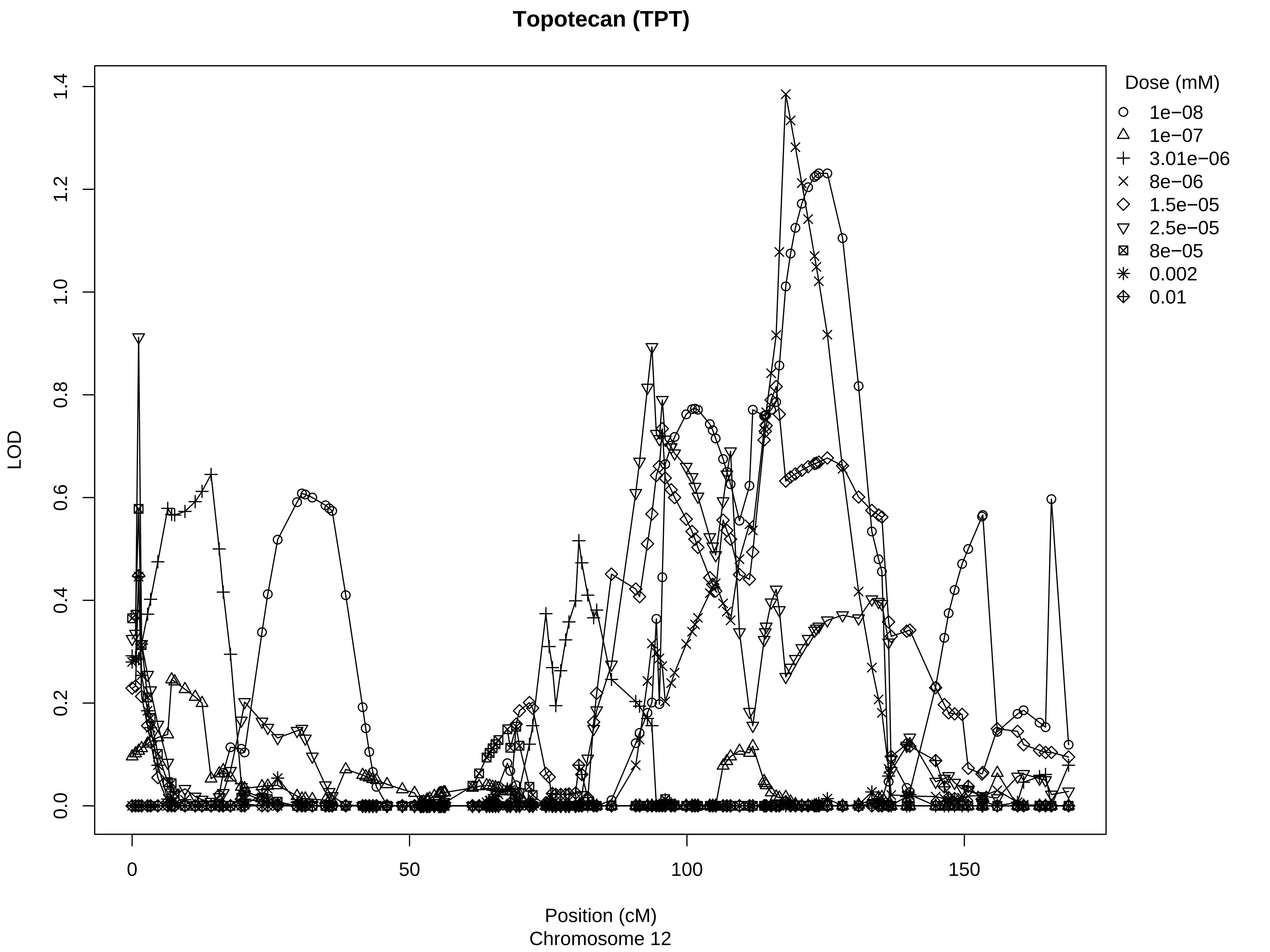
Topotecan (TPT)
LOD
0.0
0.2
0.4
0.6
0.8
1.0
1.2
1.4
0
50
100
150
Position (cM)
Chromosome 12
Dose (mM)
1e−08
1e−07
3.01e−06
8e−06
1.5e−05
2.5e−05
8e−05
0.002
0.01

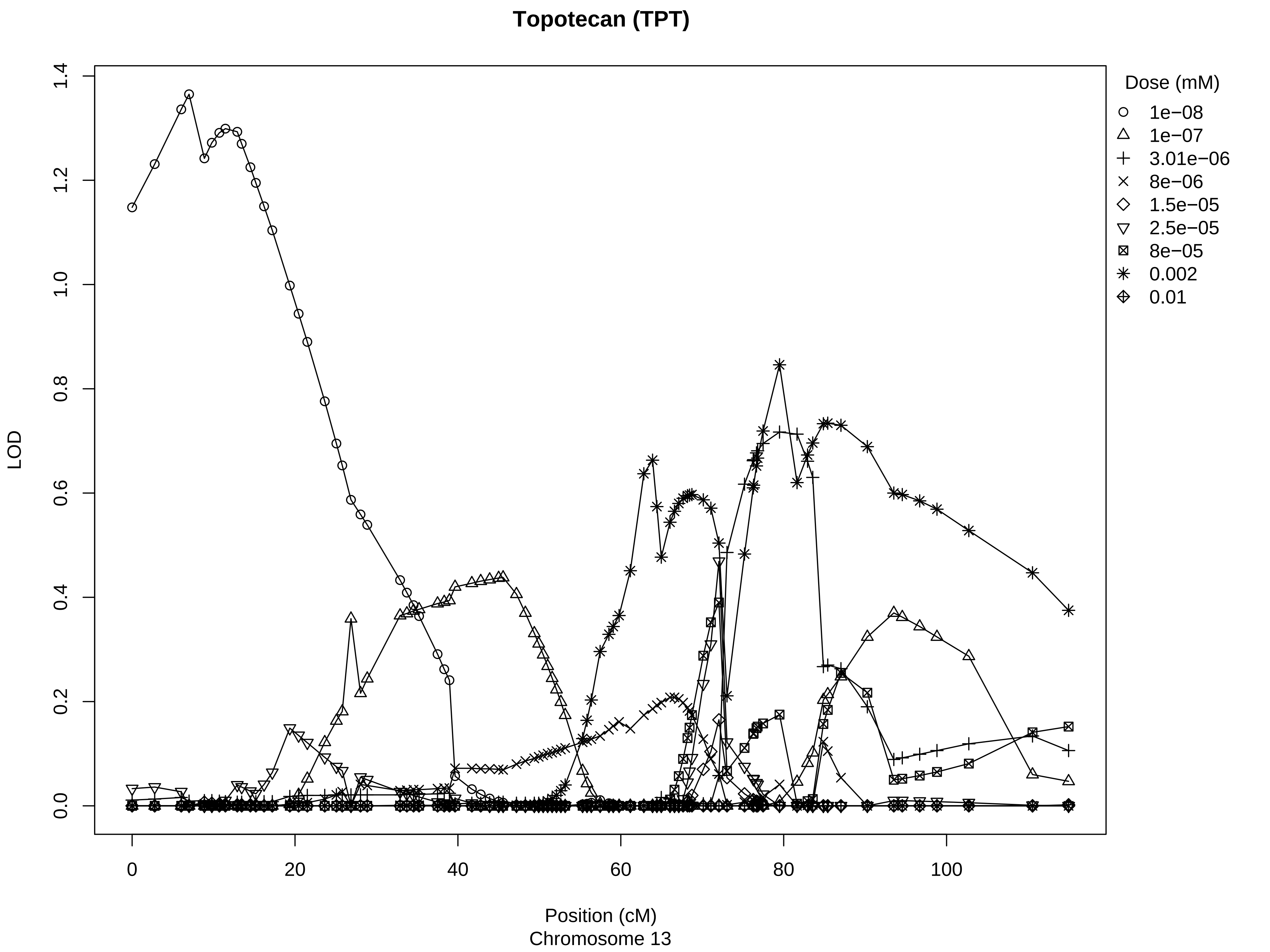

Topotecan (TPT)
LOD
0.0
0.2
0.4
0.6
0.8
1.0
1.2
1.4
0
20
40
60
80
100
Position (cM)
Chromosome 13
Dose (mM)
1e−08
1e−07
3.01e−06
8e−06
1.5e−05
2.5e−05
8e−05
0.002
0.01

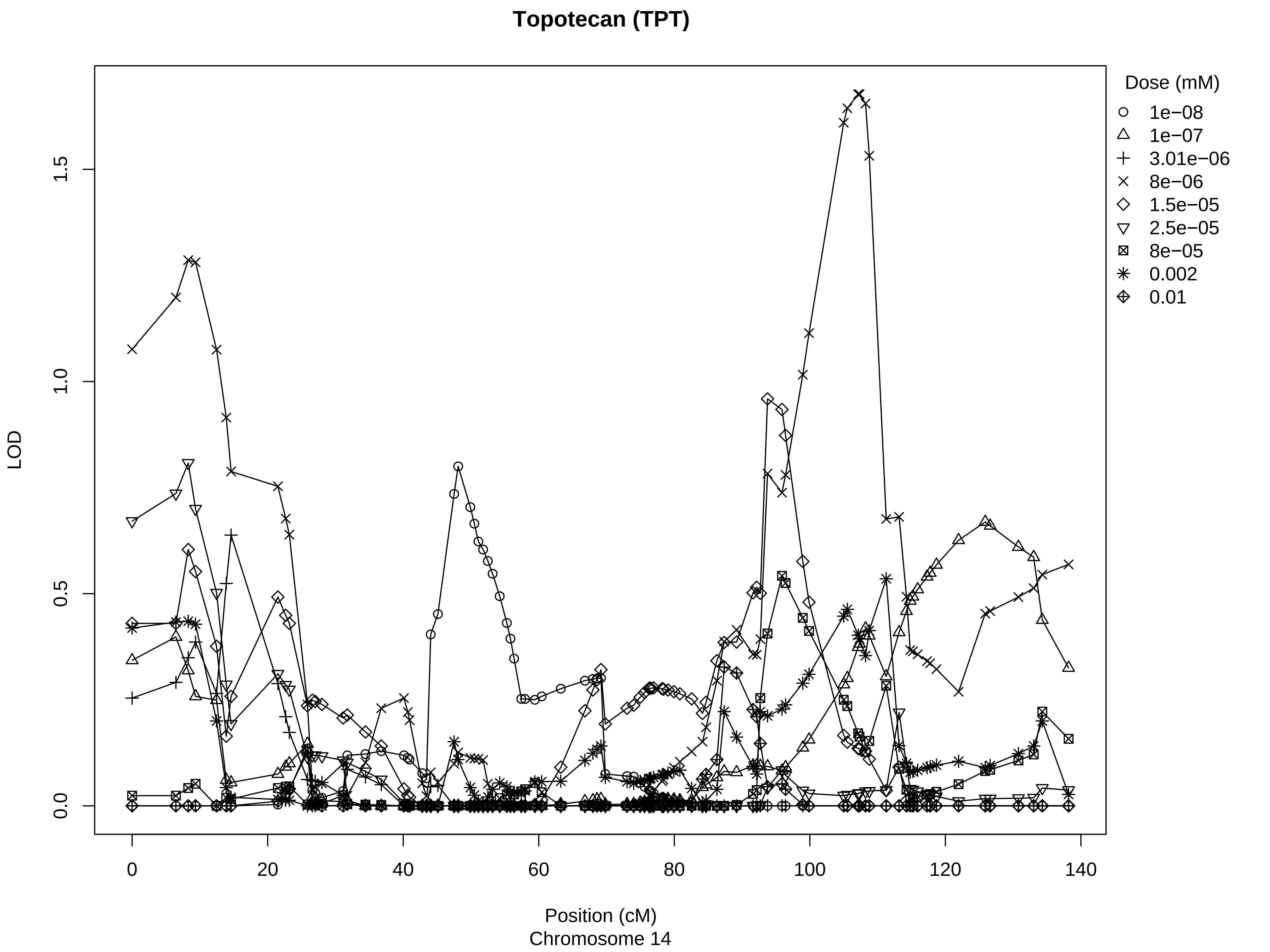

Topotecan (TPT)
LOD
0.0
0.5
1.0
1.5
0
20
40
60
80
100
120
140
Position (cM)
Chromosome 14
Dose (mM)
1e−08
1e−07
3.01e−06
8e−06
1.5e−05
2.5e−05
8e−05
0.002
0.01

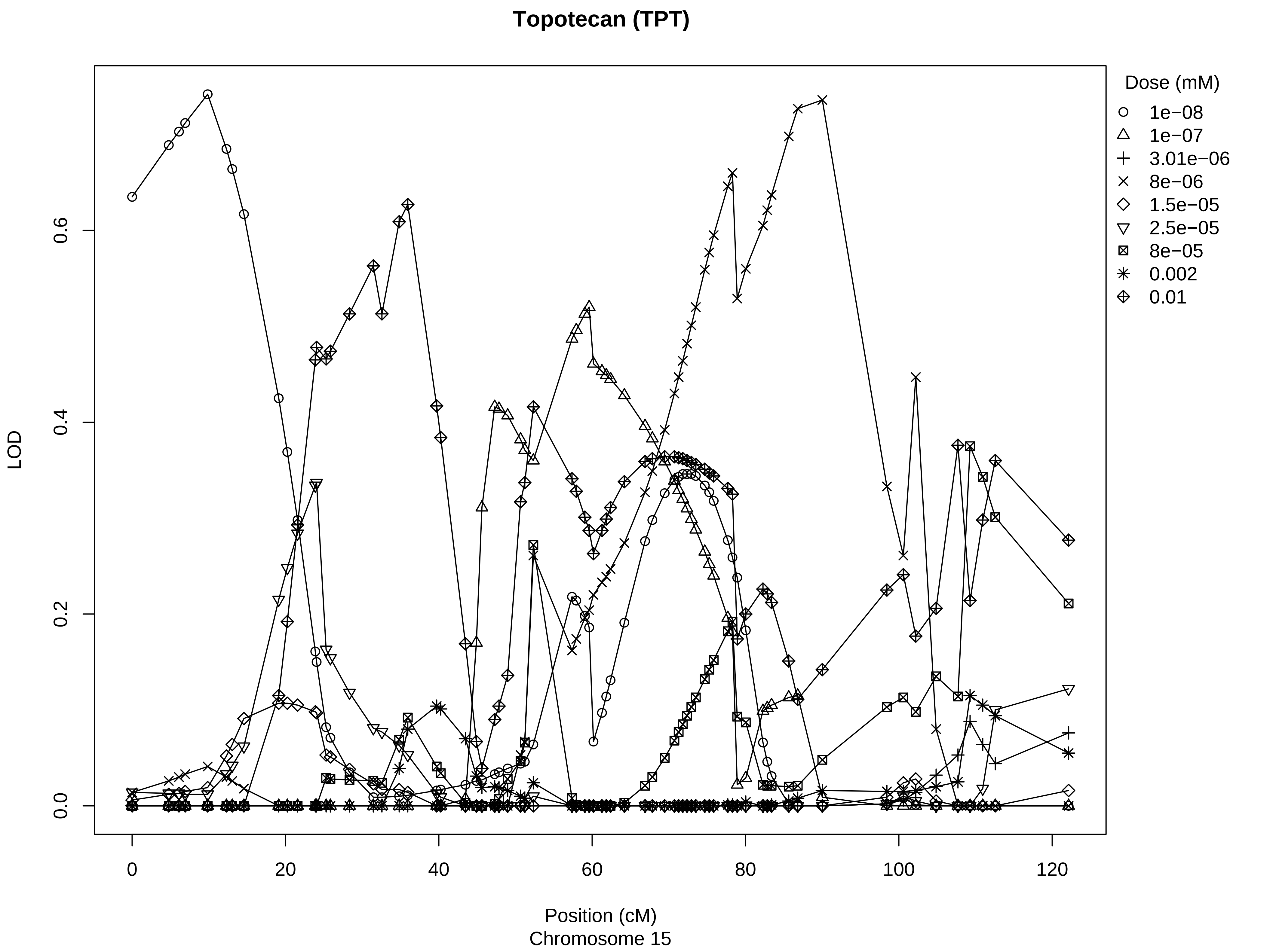
Topotecan (TPT)
Dose (mM)
1e−08
1e−07
3.01e−06
8e−06
1.5e−05
2.5e−05
8e−05
0.002
0.01
LOD
0.0
0.2
0.4
0.6
0
20
40
60
80
100
120
Position (cM)
Chromosome 15

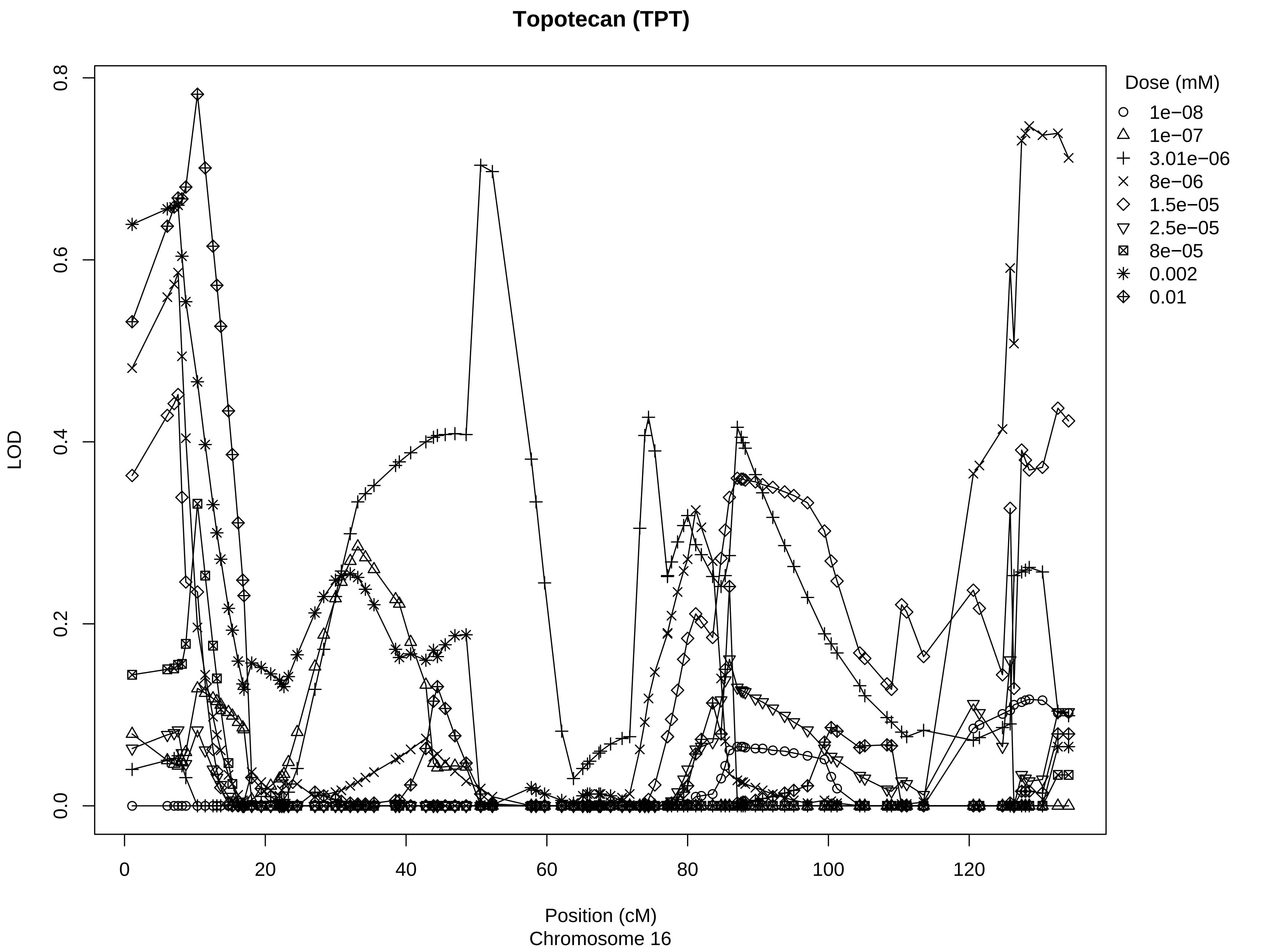
Topotecan (TPT)
Dose (mM)
1e−08
1e−07
3.01e−06
8e−06
1.5e−05
2.5e−05
8e−05
0.002
0.01
LOD
0.0
0.2
0.4
0.6
0.8
0
20
40
60
80
100
120
Position (cM)
Chromosome 16

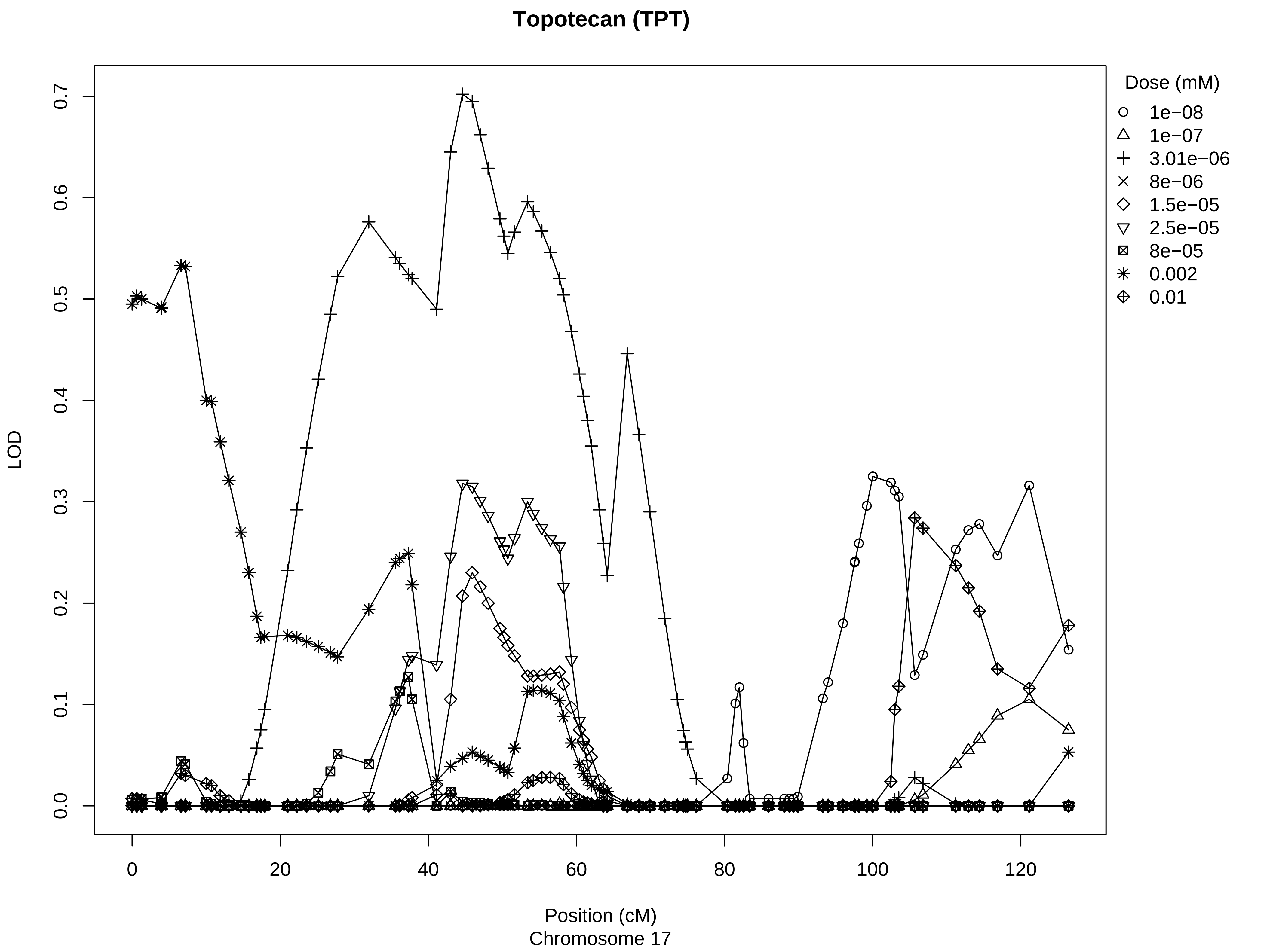

Topotecan (TPT)
Dose (mM)
1e−08
1e−07
3.01e−06
8e−06
1.5e−05
2.5e−05
8e−05
0.002
0.01
LOD
0.0
0.1
0.2
0.3
0.4
0.5
0.6
0.7
0
20
40
60
80
100
120
Position (cM)
Chromosome 17

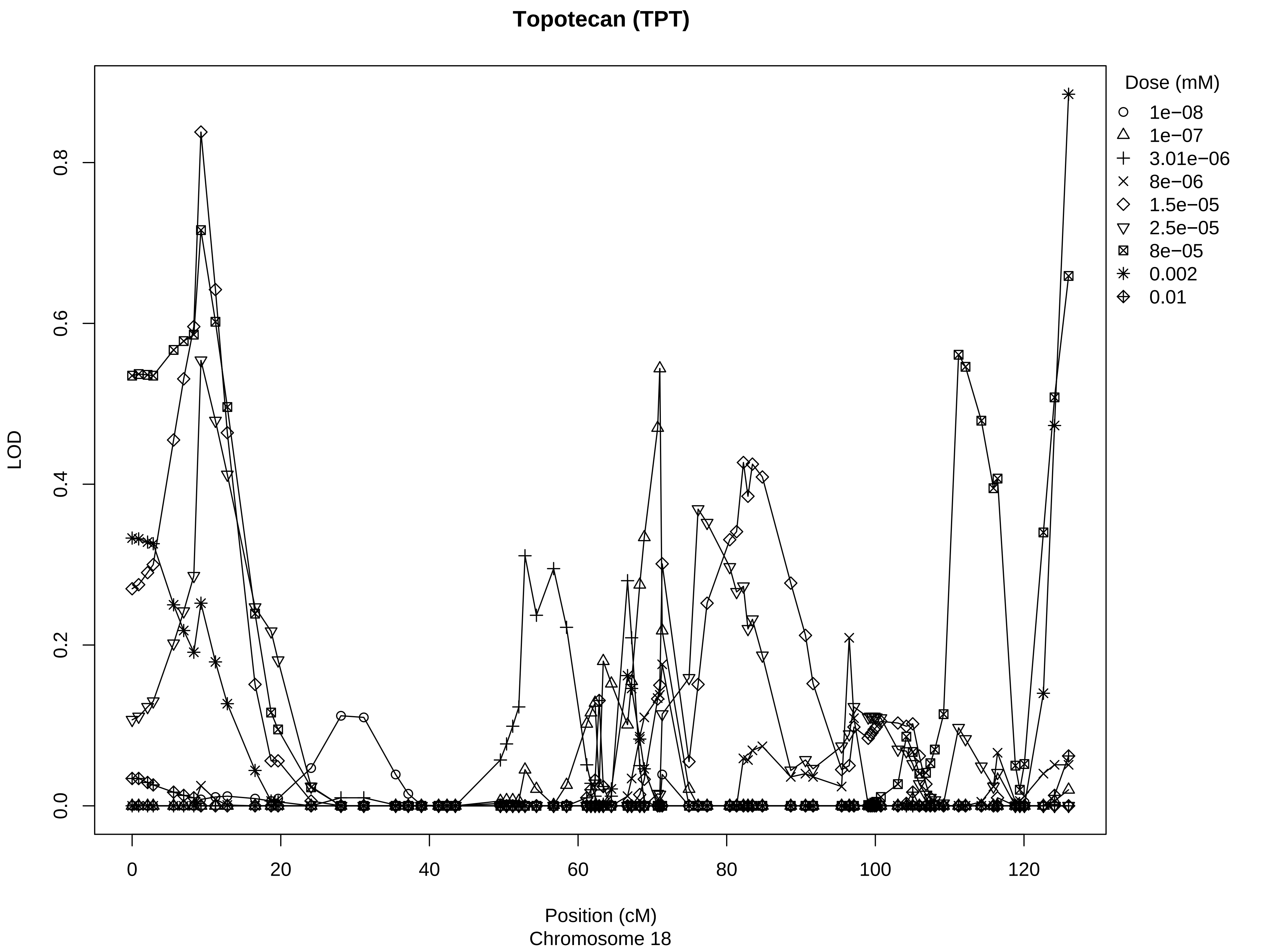

Topotecan (TPT)
Dose (mM)
1e−08
1e−07
3.01e−06
8e−06
1.5e−05
2.5e−05
8e−05
0.002
0.01
LOD
0.0
0.2
0.4
0.6
0.8
0
20
40
60
80
100
120
Position (cM)
Chromosome 18

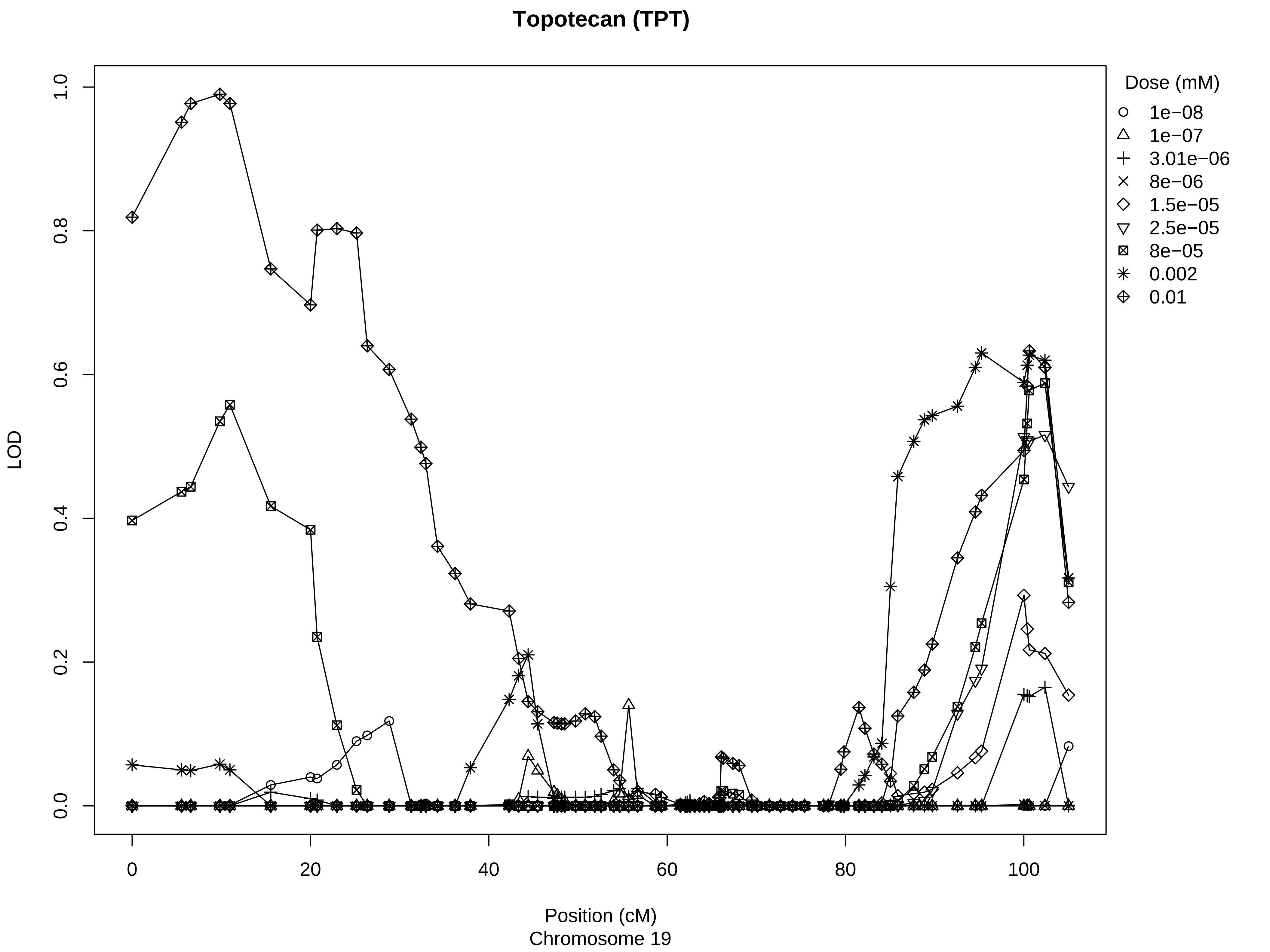
Topotecan (TPT)
Dose (mM)
1e−08
1e−07
3.01e−06
8e−06
1.5e−05
2.5e−05
8e−05
0.002
0.01
LOD
0.0
0.2
0.4
0.6
0.8
1.0
0
20
40
60
80
100
Position (cM)
Chromosome 19

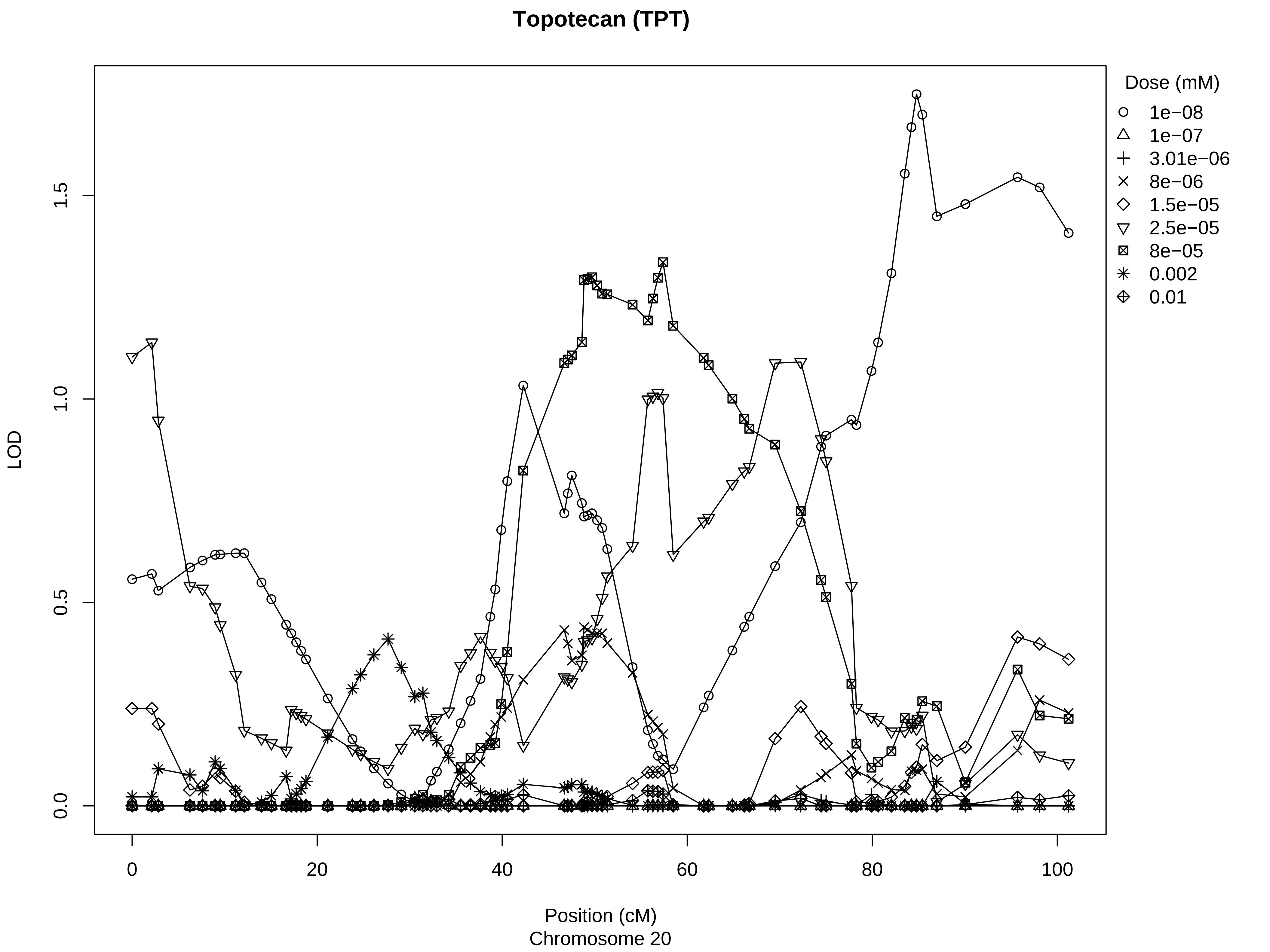

Topotecan (TPT)
Dose (mM)
1e−08
1e−07
3.01e−06
8e−06
1.5e−05
2.5e−05
8e−05
0.002
0.01
LOD
0.0
0.5
1.0
1.5
0
20
40
60
80
100
Position (cM)
Chromosome 20

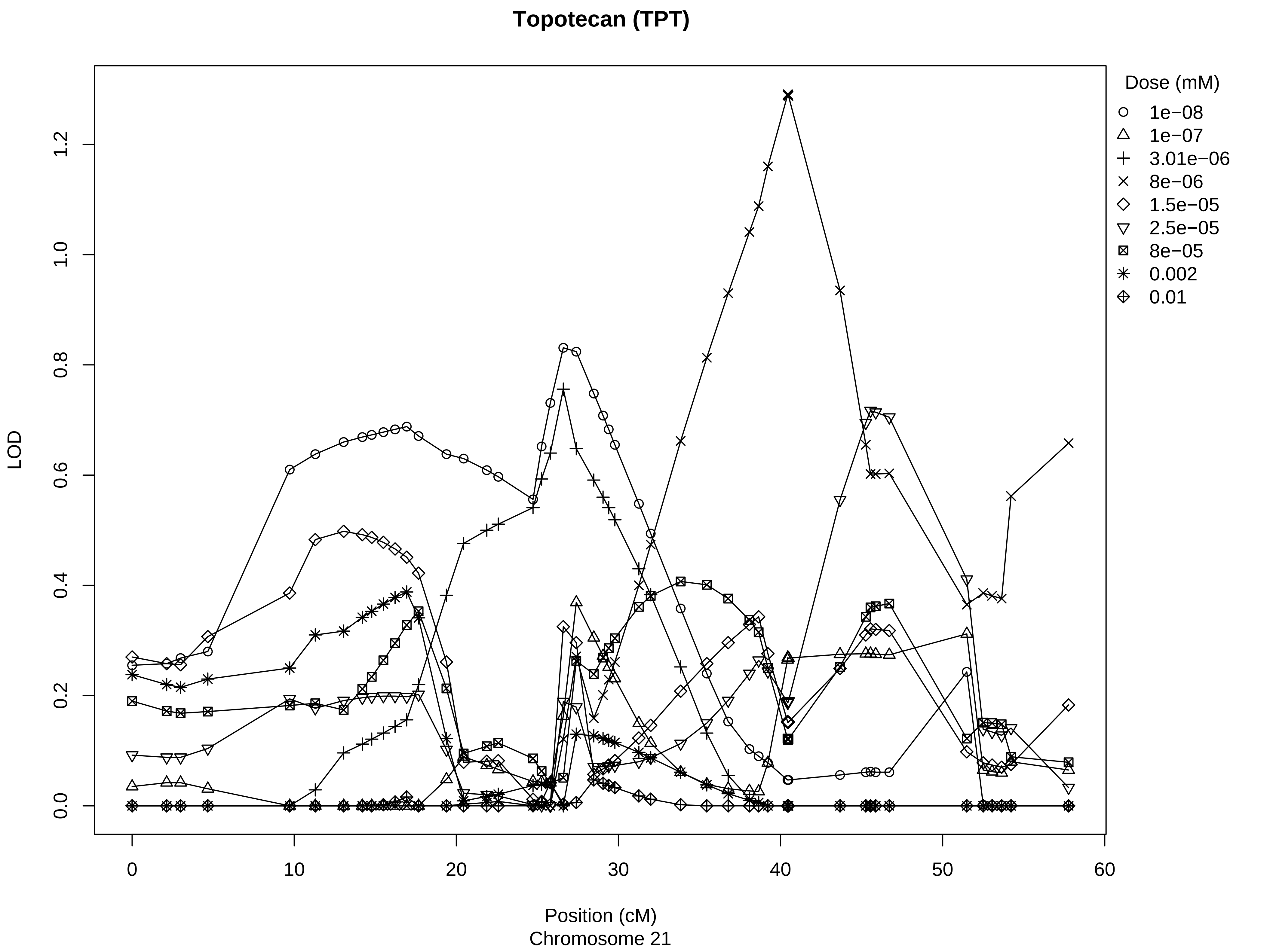

Topotecan (TPT)
Dose (mM)
1e−08
1e−07
3.01e−06
8e−06
1.5e−05
2.5e−05
8e−05
0.002
0.01
LOD
0.0
0.2
0.4
0.6
0.8
1.0
1.2
0
10
20
30
40
50
60
Position (cM)
Chromosome 21

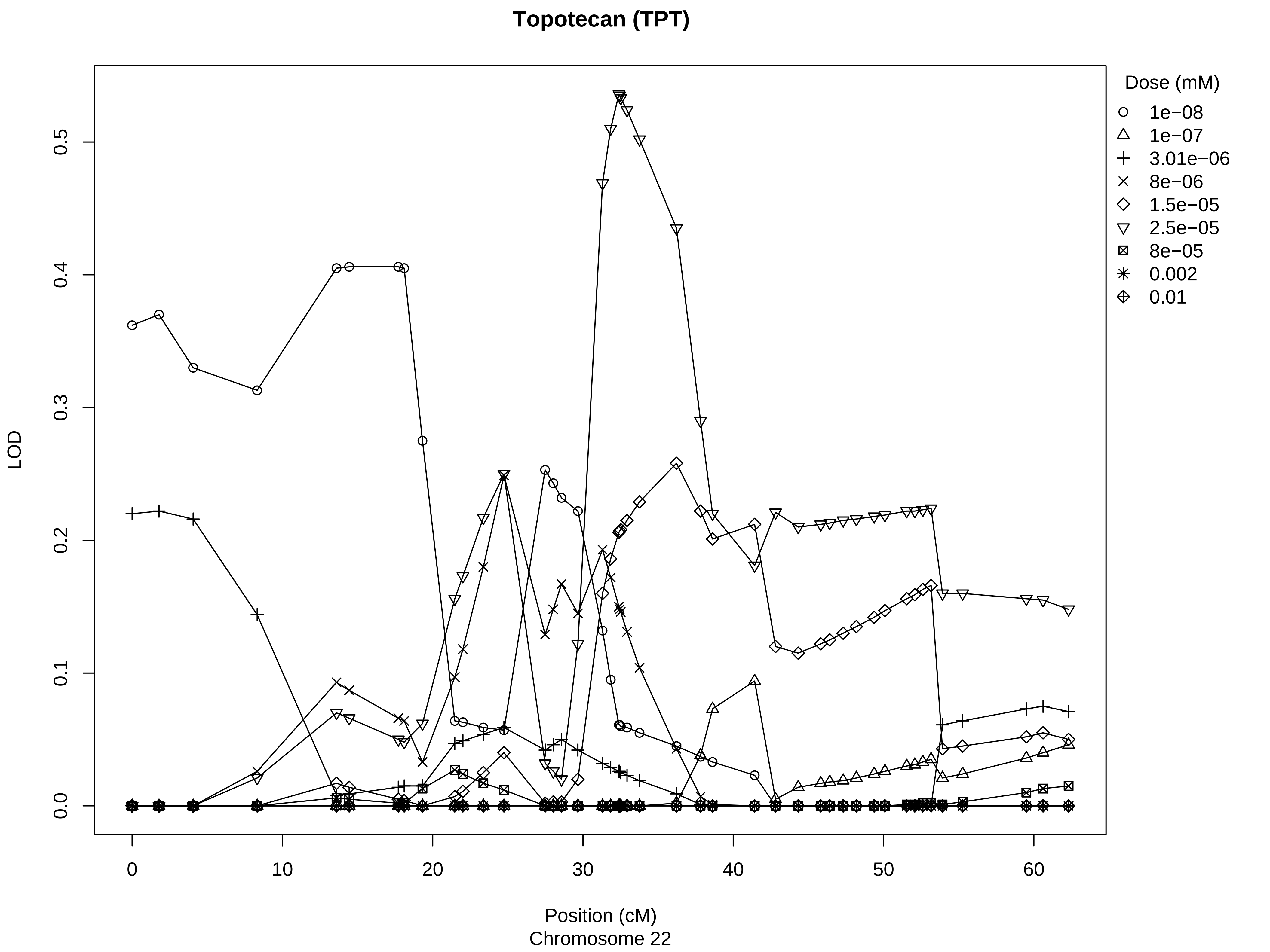
Topotecan (TPT)
Dose (mM)
1e−08
1e−07
3.01e−06
8e−06
1.5e−05
2.5e−05
8e−05
0.002
0.01
LOD
0.0
0.1
0.2
0.3
0.4
0.5
0
10
20
30
40
50
60
Position (cM)
Chromosome 22

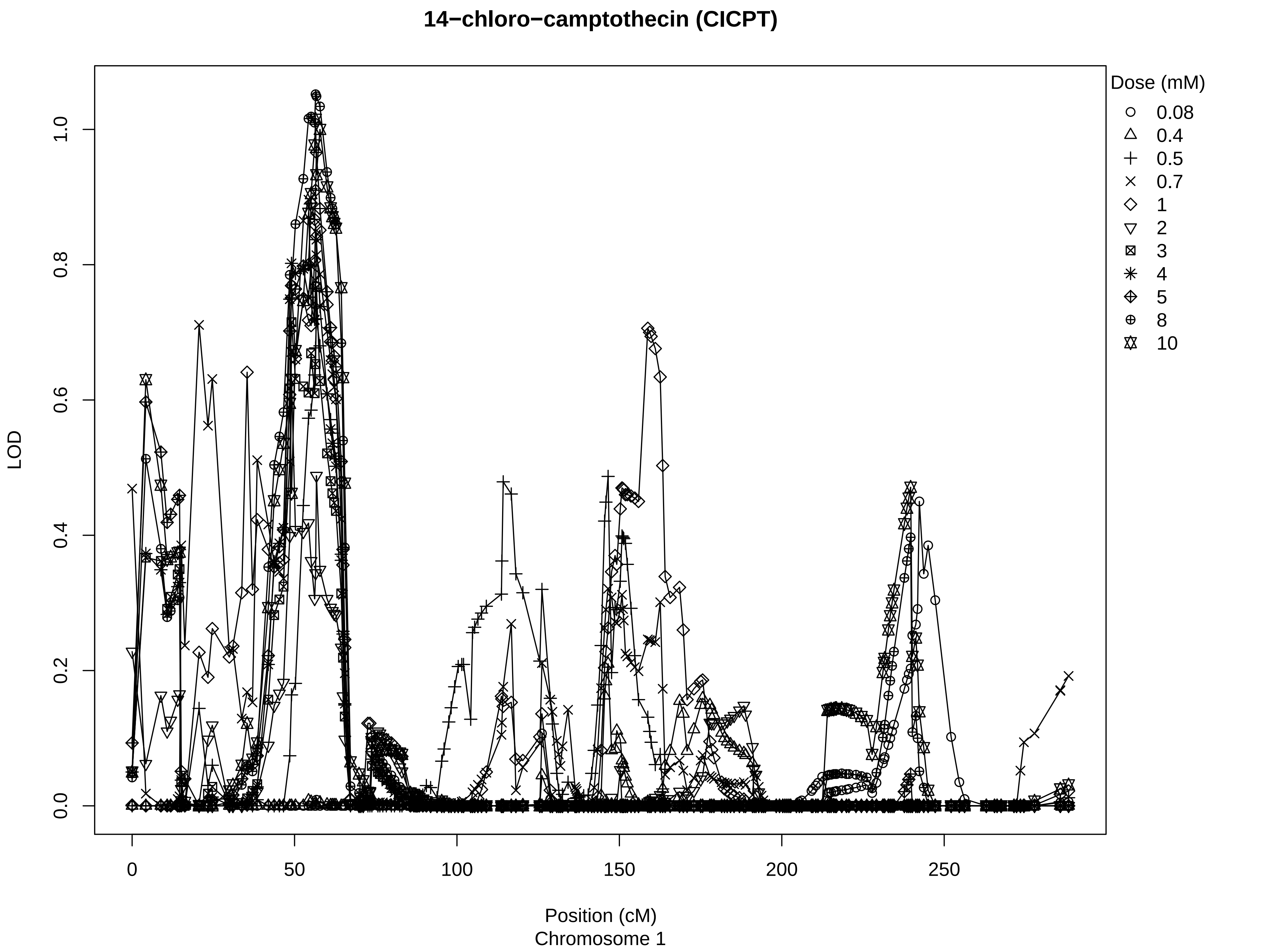

14−chloro−camptothecin (ClCPT)
LOD
0.0
0.2
0.4
0.6
0.8
1.0
0
50
100
150
200
250
Position (cM)
Chromosome 1
Dose (mM)
0.08
0.4
0.5
0.7
1
2
3
4
5
8
10

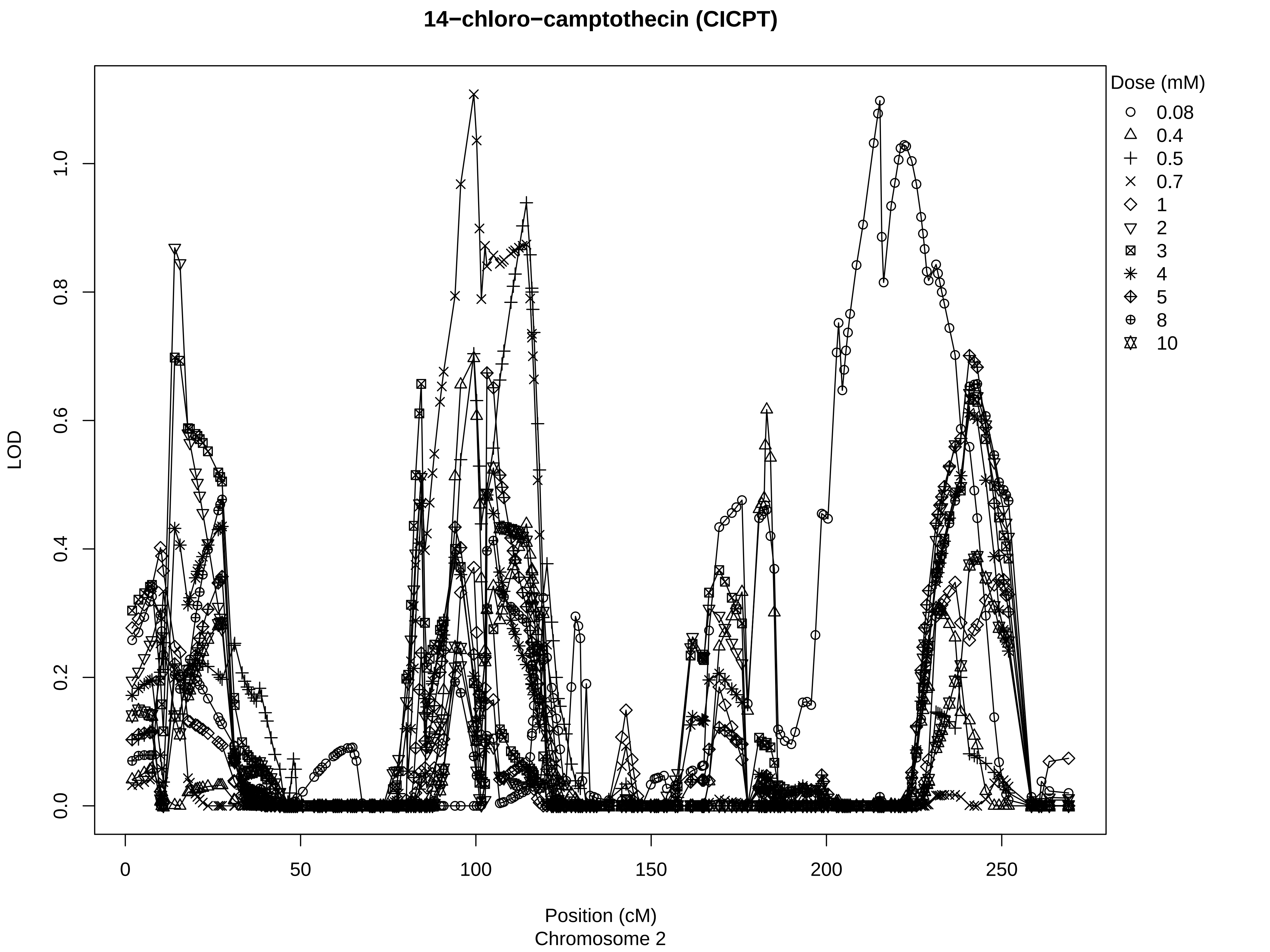

14−chloro−camptothecin (ClCPT)
LOD
0.0
0.2
0.4
0.6
0.8
1.0
0
50
100
150
200
250
Position (cM)
Chromosome 2
Dose (mM)
0.08
0.4
0.5
0.7
1
2
3
4
5
8
10

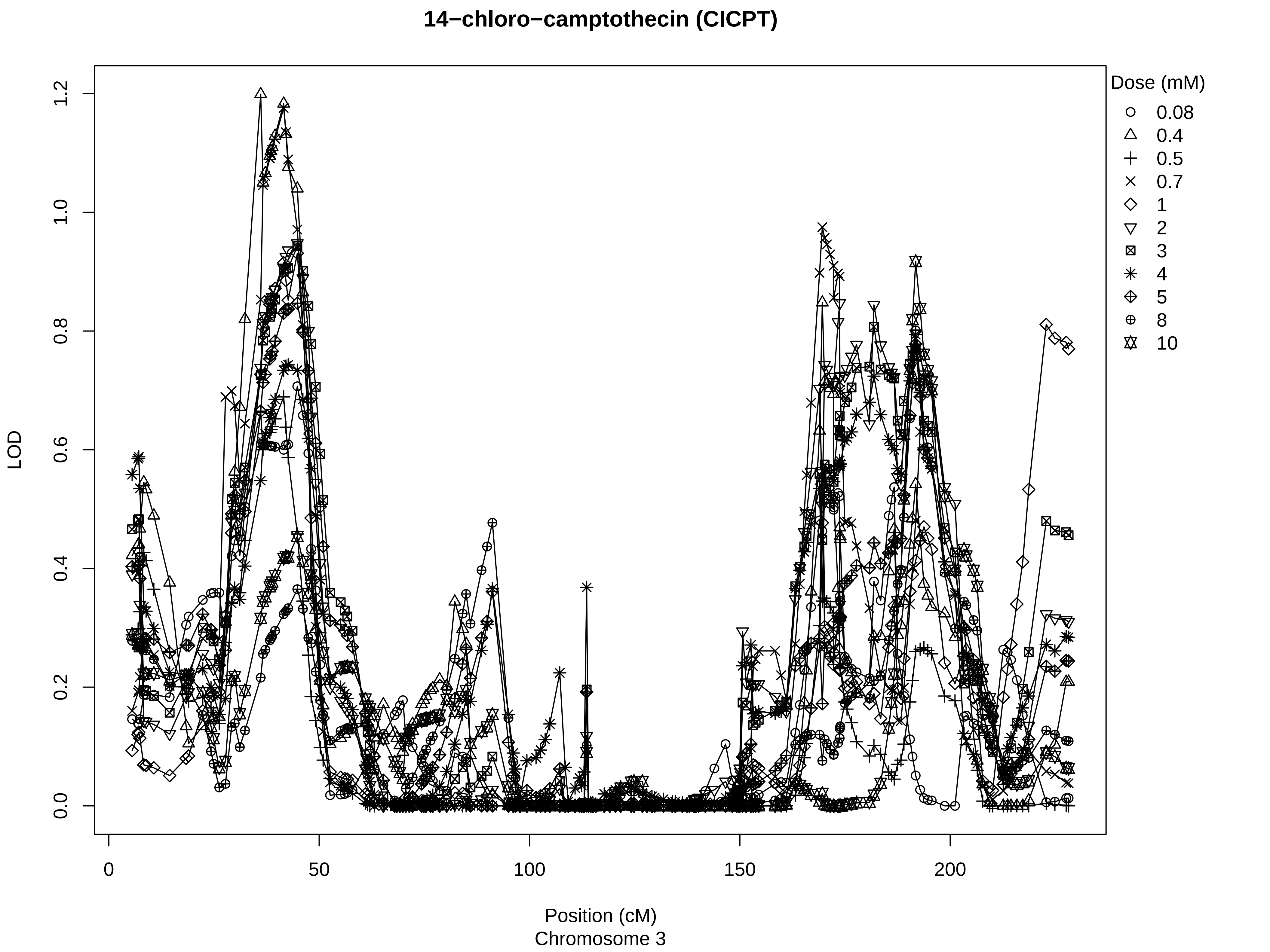

14−chloro−camptothecin (ClCPT)
Dose (mM)
0.08
0.4
0.5
0.7
1
2
3
4
5
8
10
LOD
0.0
0.2
0.4
0.6
0.8
1.0
1.2
0
50
100
150
200
Position (cM)
Chromosome 3

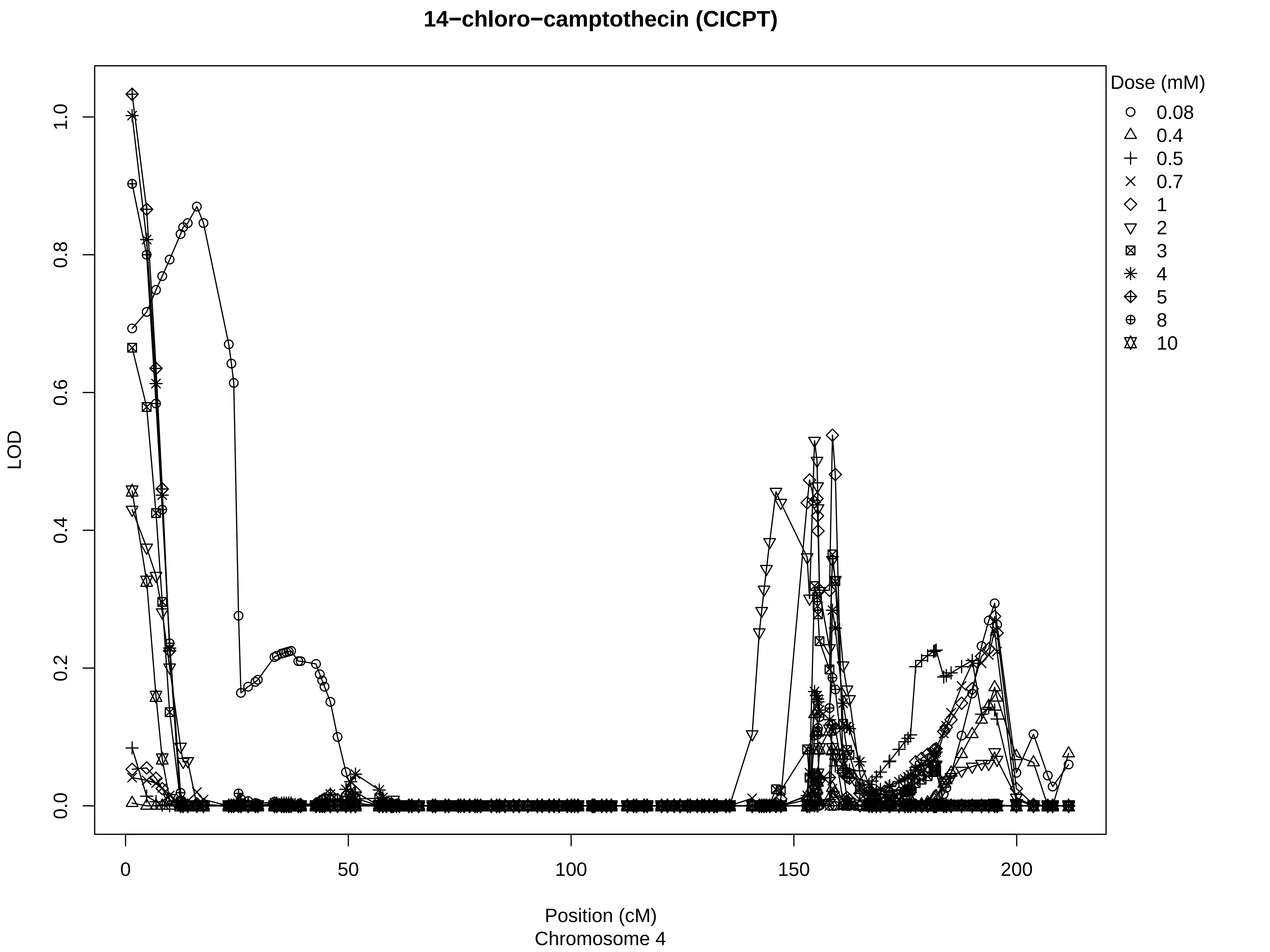
14−chloro−camptothecin (ClCPT)
LOD
0.0
0.2
0.4
0.6
0.8
1.0
0
50
100
150
200
Position (cM)
Chromosome 4
Dose (mM)
0.08
0.4
0.5
0.7
1
2
3
4
5
8
10

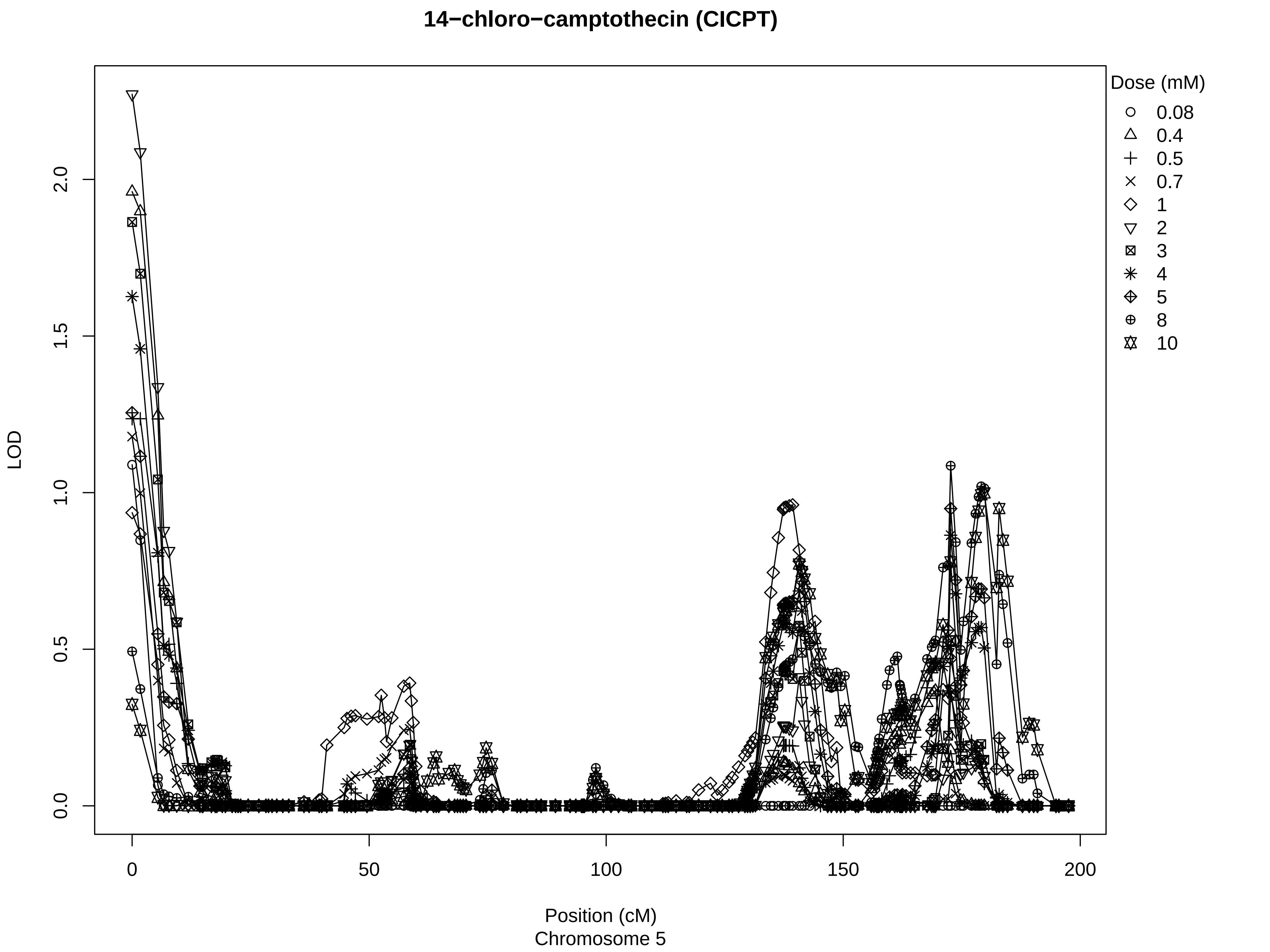

14−chloro−camptothecin (ClCPT)
LOD
0.0
0.5
1.0
1.5
2.0
0
50
100
150
200
Position (cM)
Chromosome 5
Dose (mM)
0.08
0.4
0.5
0.7
1
2
3
4
5
8
10

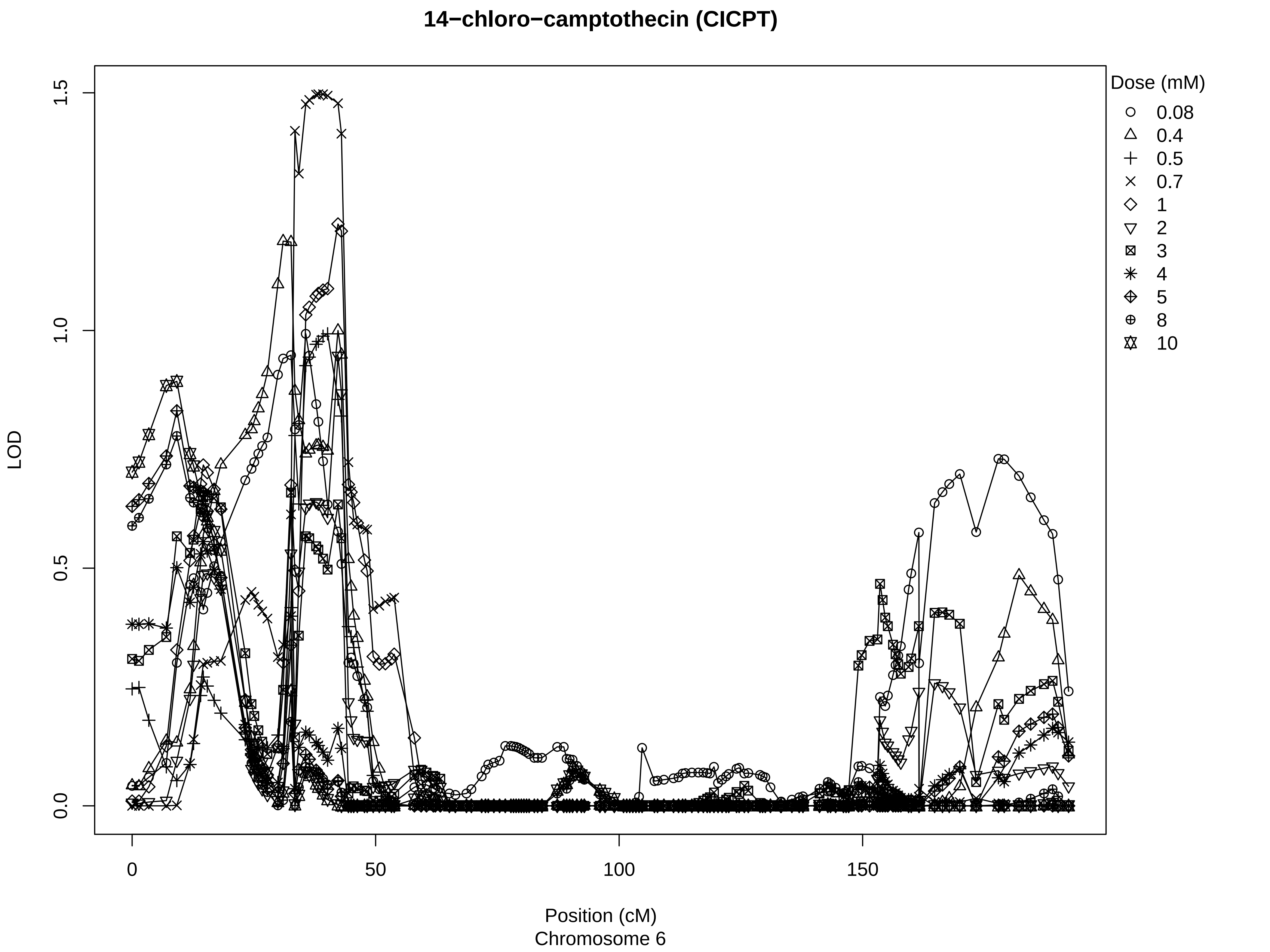

14−chloro−camptothecin (ClCPT)
LOD
0.0
0.5
1.0
1.5
0
50
100
150
Position (cM)
Chromosome 6
Dose (mM)
0.08
0.4
0.5
0.7
1
2
3
4
5
8
10

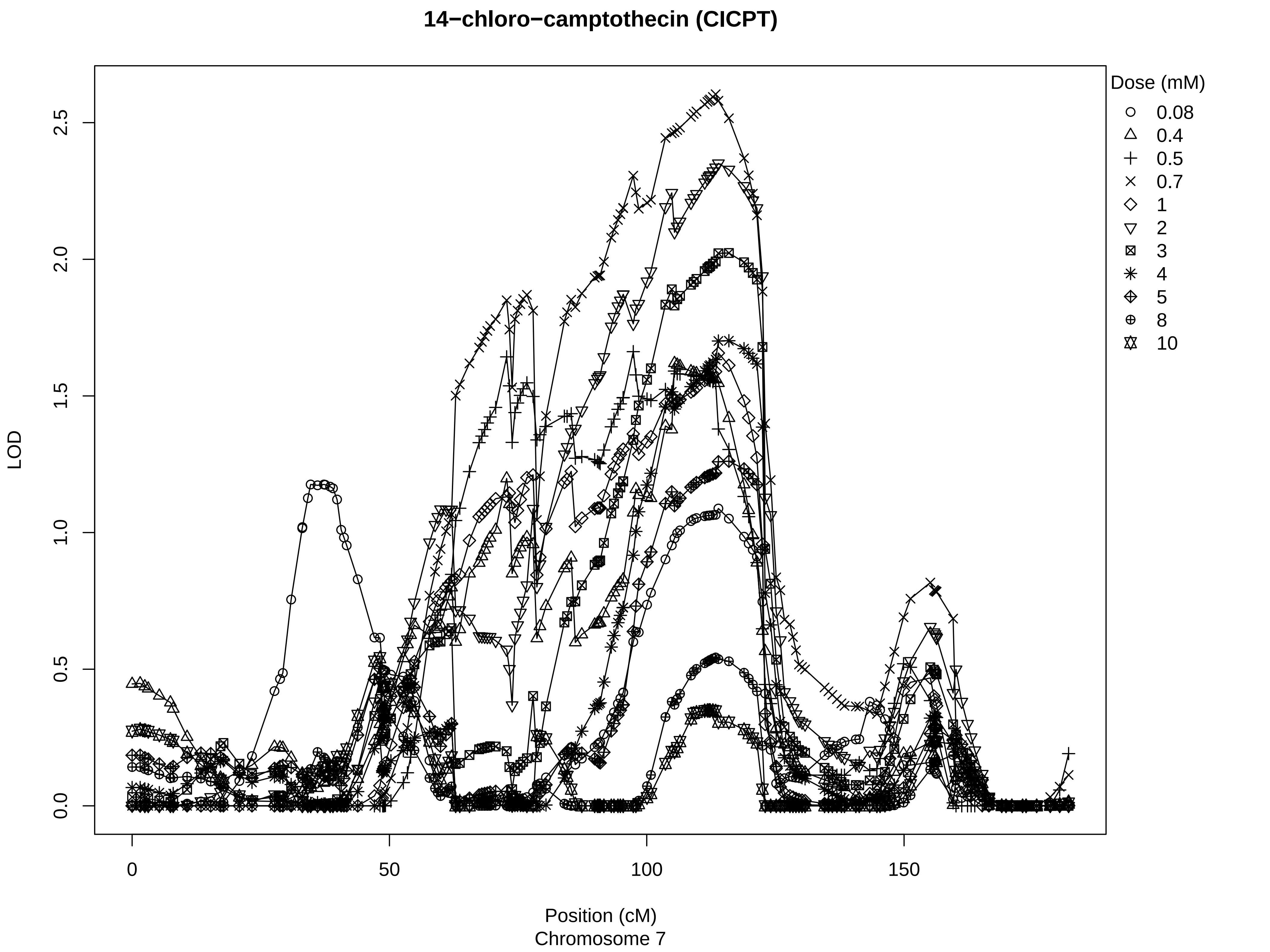
14−chloro−camptothecin (ClCPT)
LOD
Position (cM)
Chromosome 7
Dose (mM)
0.08
0.4
0.5
0.7
1
2
3
4
5
8
10
0.0
0.5
1.0
1.5
2.0
2.5
0
50
100
150

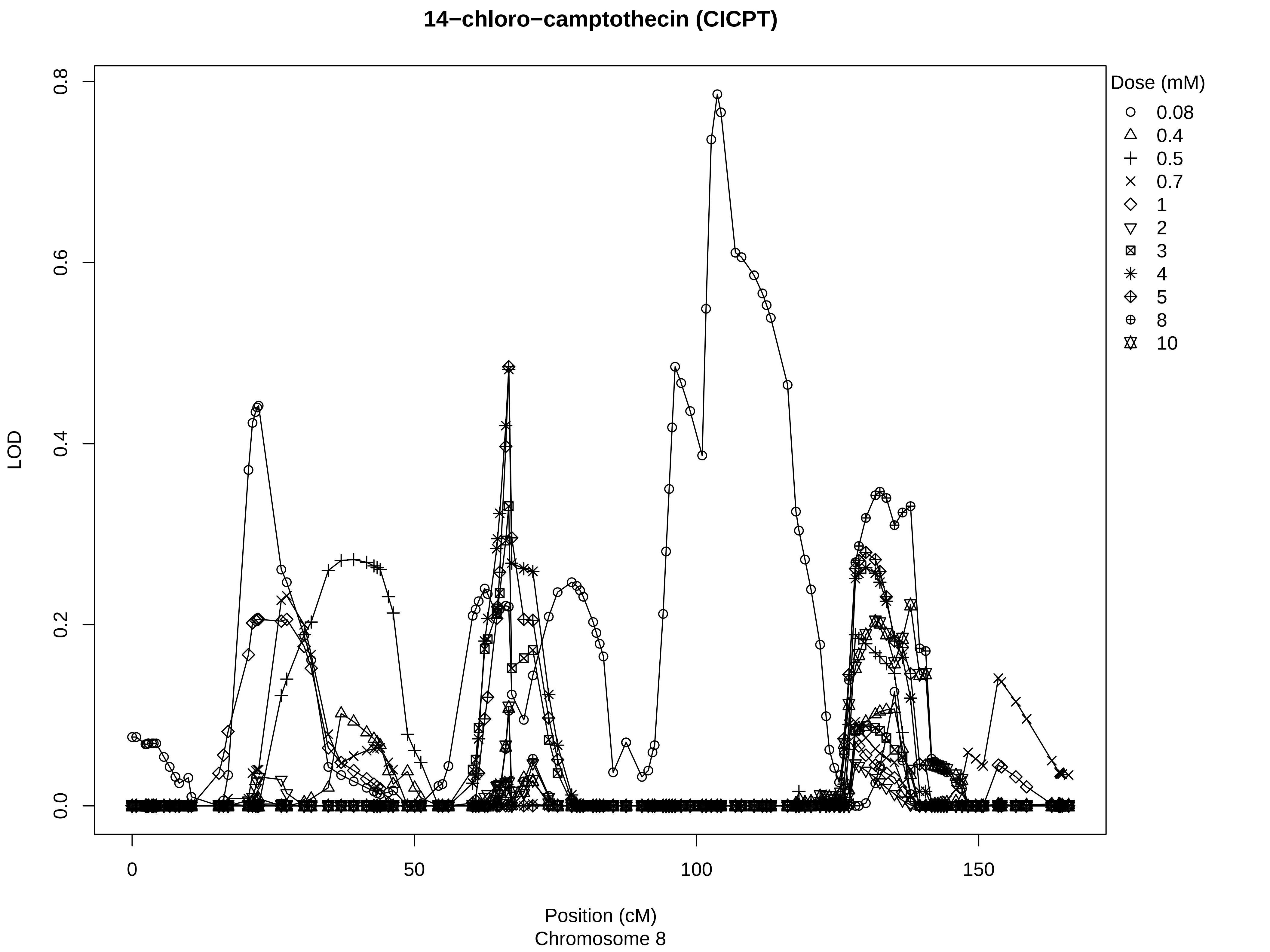

14−chloro−camptothecin (ClCPT)
LOD
0.0
0.2
0.4
0.6
0.8
0
50
100
150
Position (cM)
Chromosome 8
Dose (mM)
0.08
0.4
0.5
0.7
1
2
3
4
5
8
10

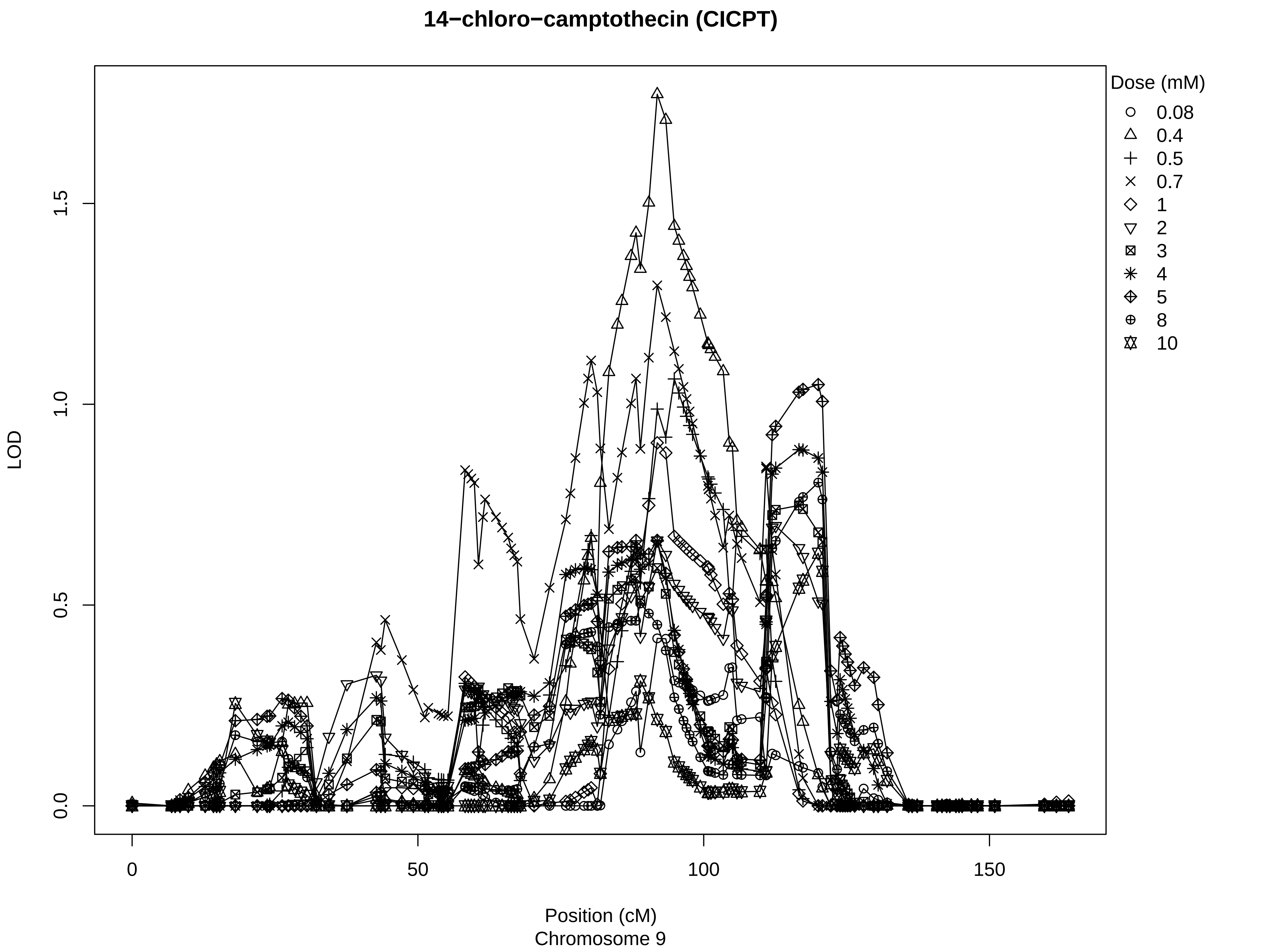
14−chloro−camptothecin (ClCPT)
LOD
0.0
0.5
1.0
1.5
0
50
100
150
Position (cM)
Chromosome 9
Dose (mM)
0.08
0.4
0.5
0.7
1
2
3
4
5
8
10

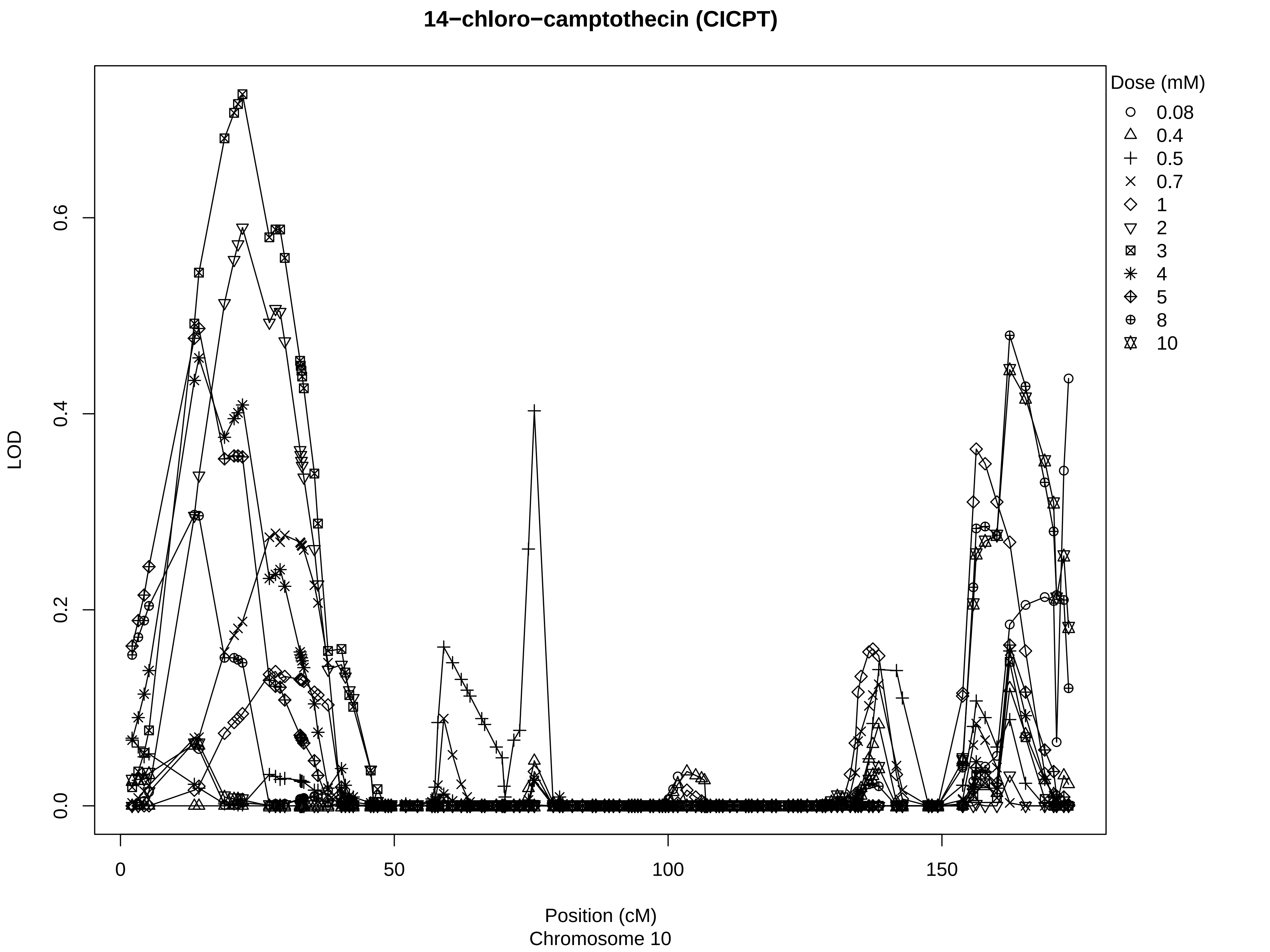
14−chloro−camptothecin (CICPT)
Dose (mM)
0.08
0.4
0.5
0.7
1
2
3
4
5
8
10
LOD
0.0
0.2
0.4
0.6
0
50
100
150
Position (cM)
Chromosome 10

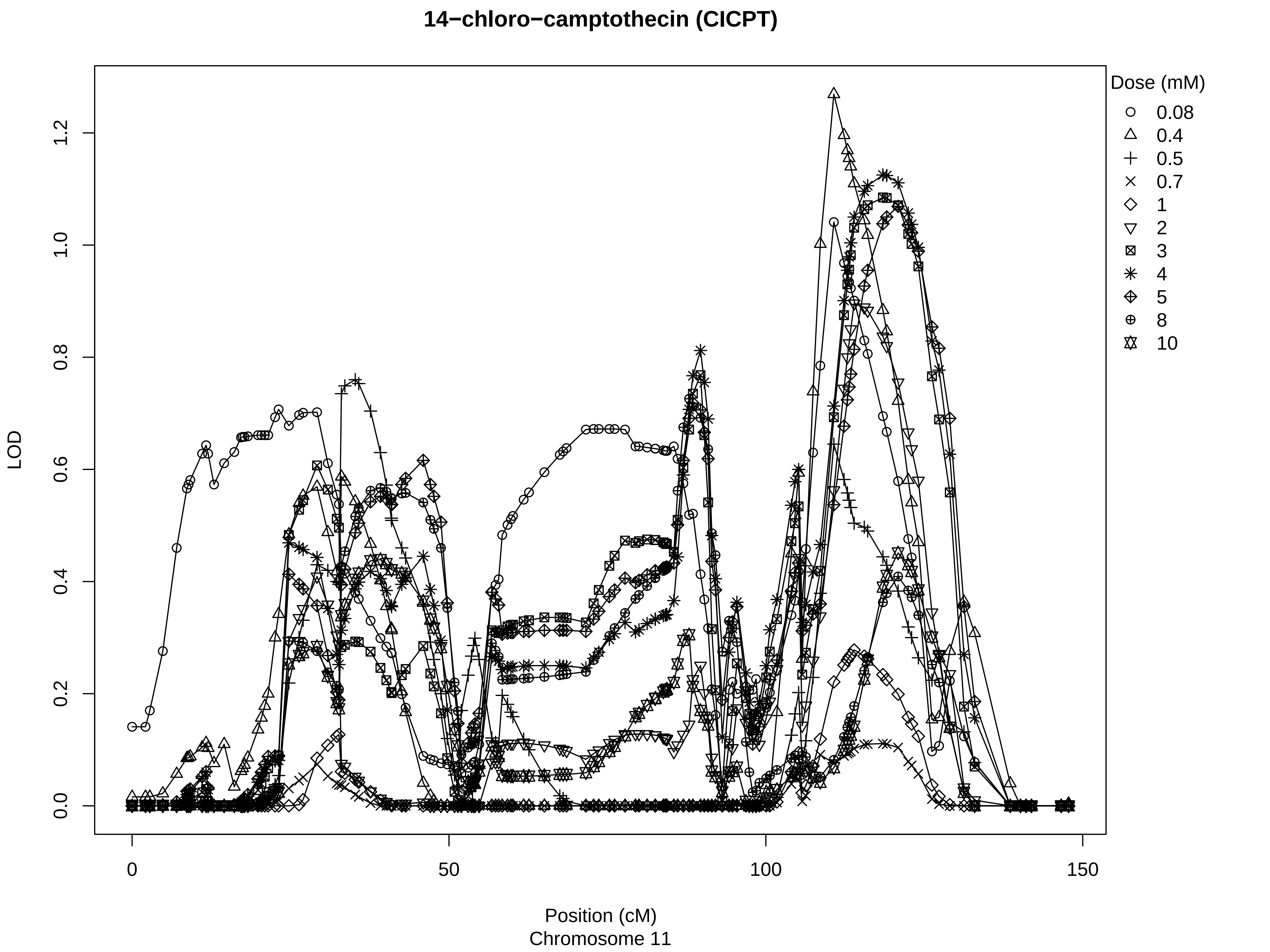
14−chloro−camptothecin (ClCPT)
LOD
0.0
0.2
0.4
0.6
0.8
1.0
1.2
0
50
100
150
Position (cM)
Chromosome 11
Dose (mM)
0.08
0.4
0.5
0.7
1
2
3
4
5
8
10

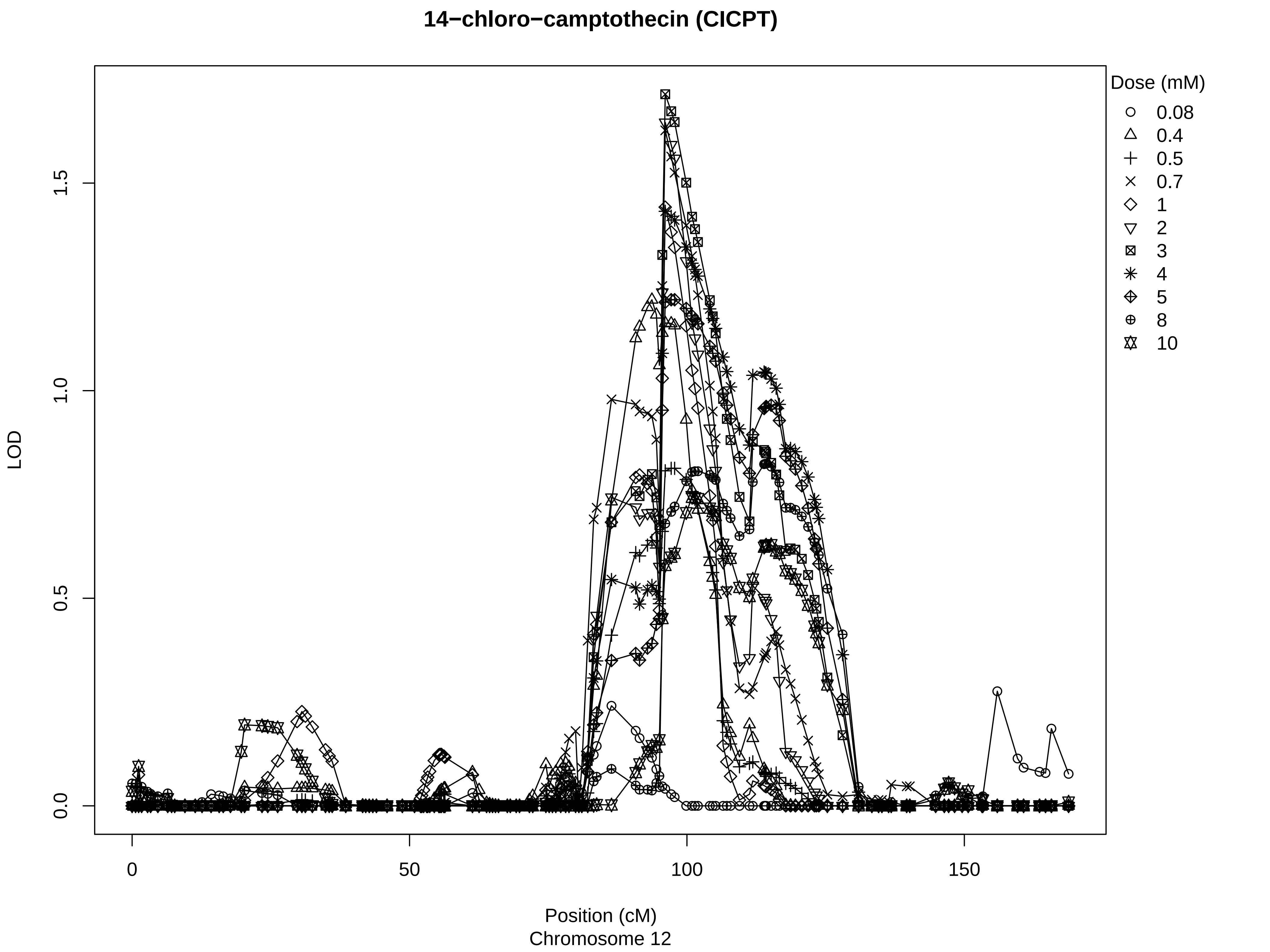

14−chloro−camptothecin (ClCPT)
Dose (mM)
0.08
0.4
0.5
0.7
1
2
3
4
5
8
10
LOD
0.0
0.5
1.0
1.5
0
50
100
150
Position (cM)
Chromosome 12

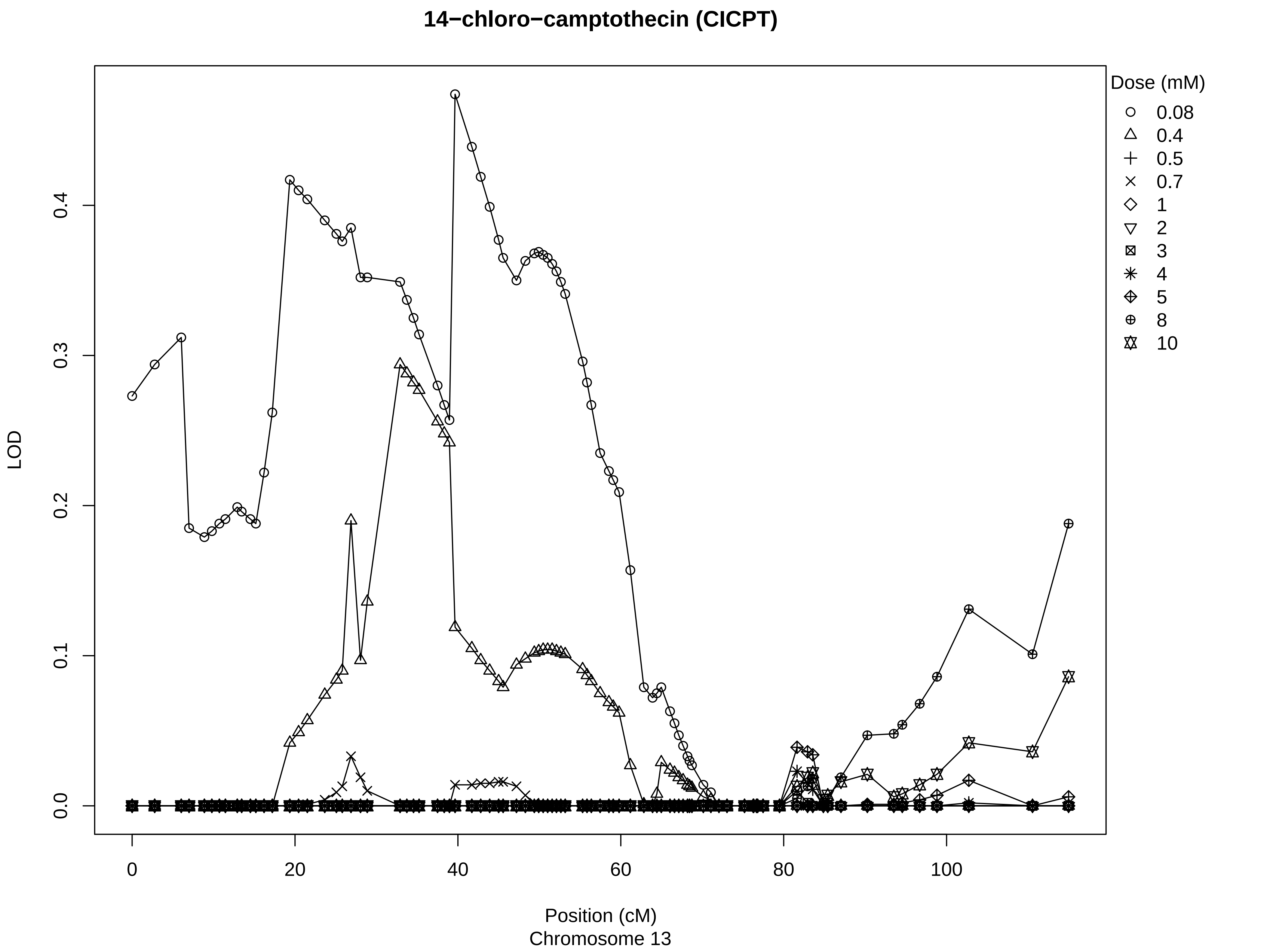
14−chloro−camptothecin (ClCPT)
LOD
Position (cM)
Chromosome 13
Dose (mM)
0.08
0.4
0.5
0.7
1
2
3
4
5
8
10
0.0
0.1
0.2
0.3
0.4
0
20
40
60
80
100

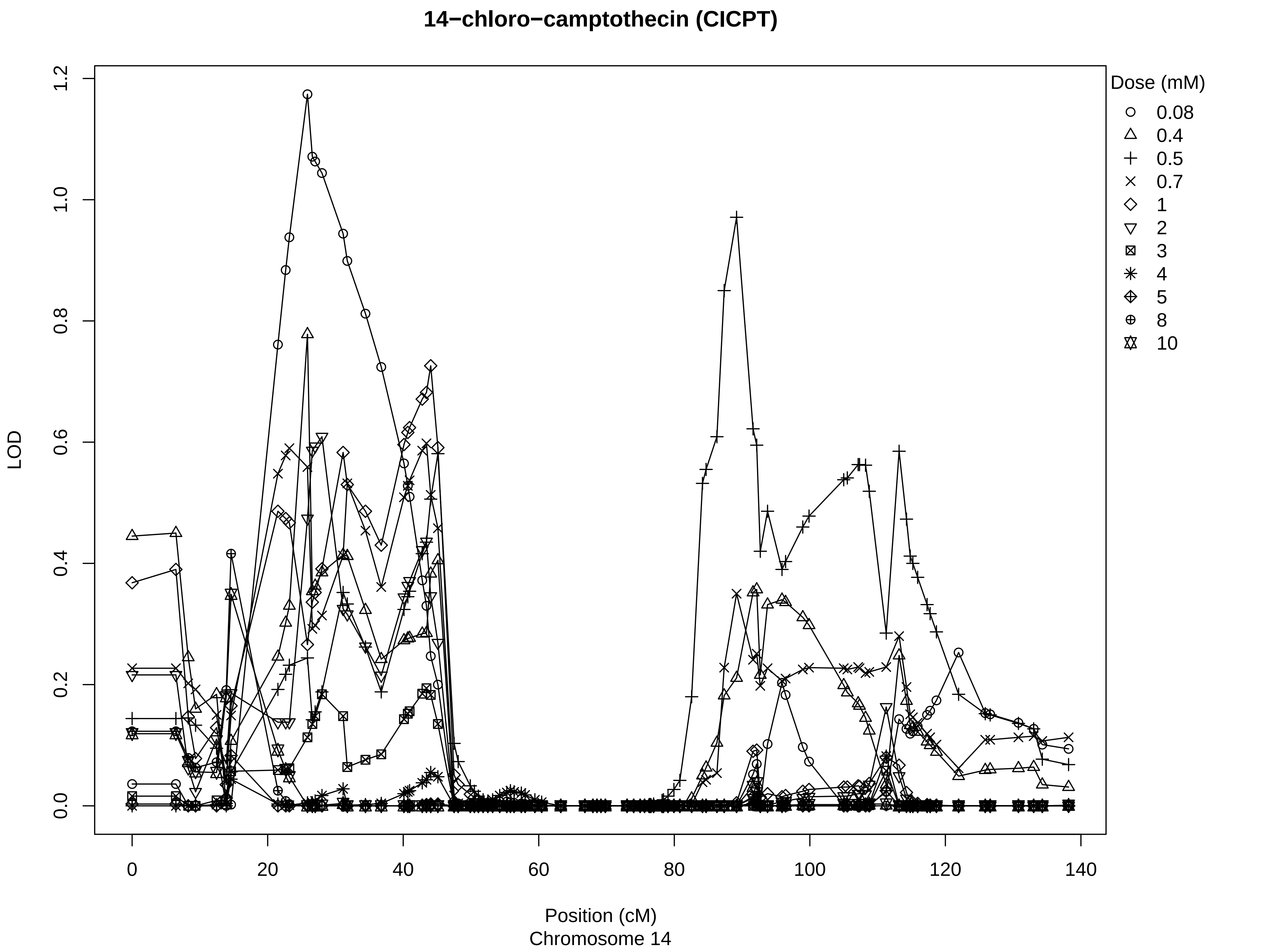
14−chloro−camptothecin (ClCPT)
LOD
Position (cM)
Chromosome 14
Dose (mM)
0.08
0.4
0.5
0.7
1
2
3
4
5
8
10
0.0
0.2
0.4
0.6
0.8
1.0
1.2
0
20
40
60
80
100
120
140

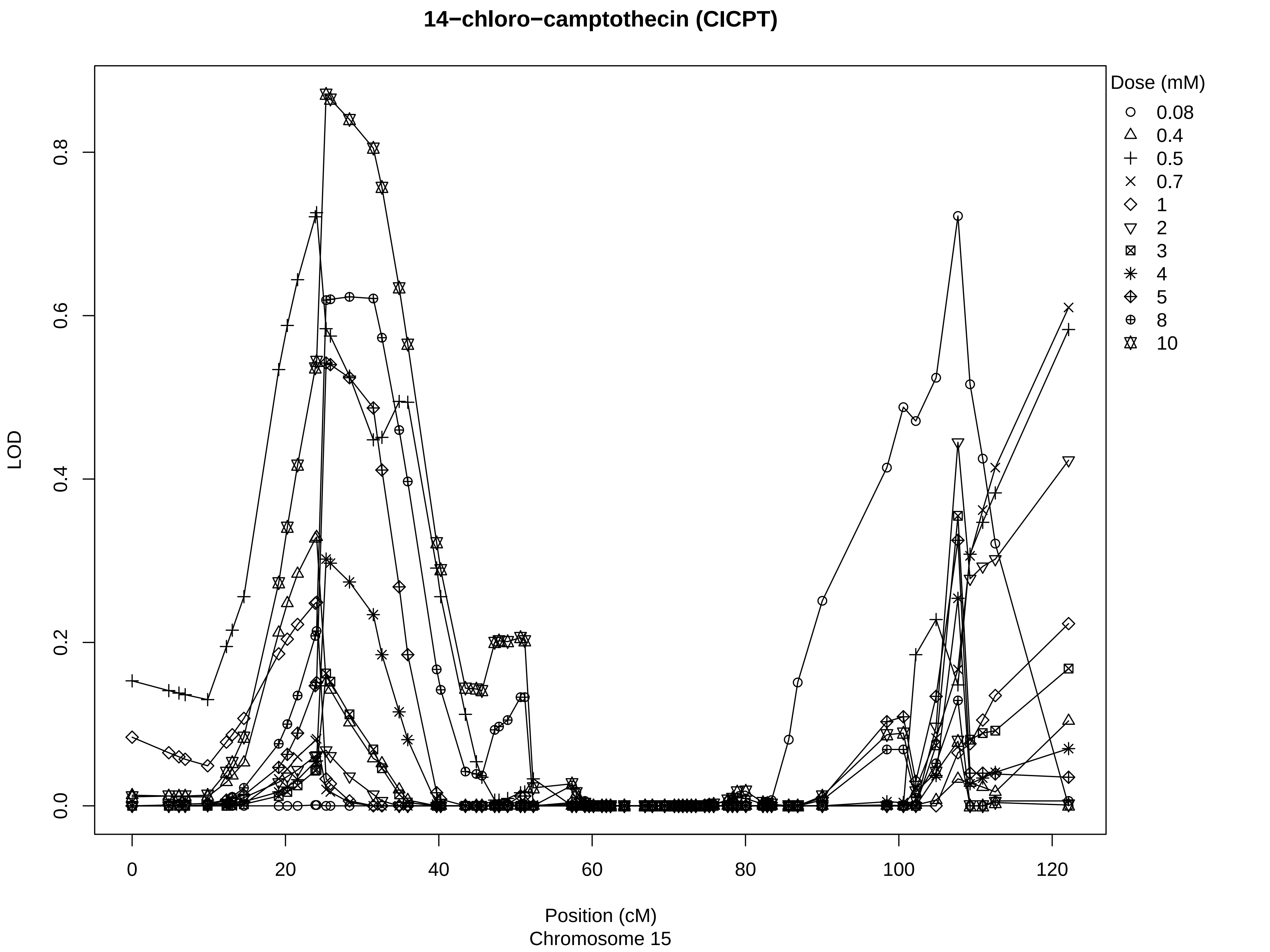

14−chloro−camptothecin (ClCPT)
LOD
0.0
0.2
0.4
0.6
0.8
0
20
40
60
80
100
120
Position (cM)
Chromosome 15
Dose (mM)
0.08
0.4
0.5
0.7
1
2
3
4
5
8
10

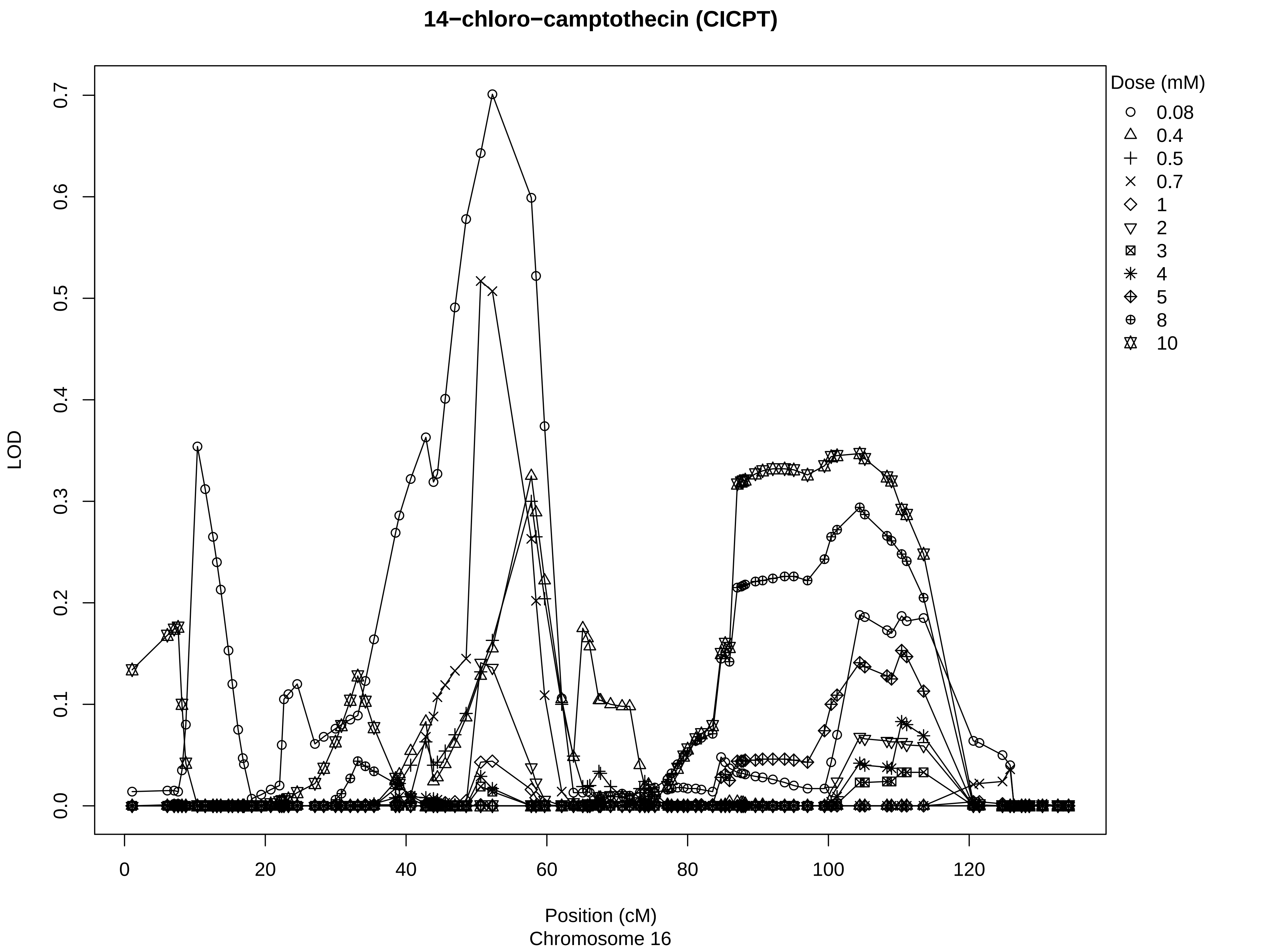
14−chloro−camptothecin (ClCPT)
LOD
0.0
0.1
0.2
0.3
0.4
0.5
0.6
0.7
Position (cM)
Chromosome 16
0
20
40
60
80
100
120
Dose (mM)
0.08
0.4
0.5
0.7
1
2
3
4
5
8
10

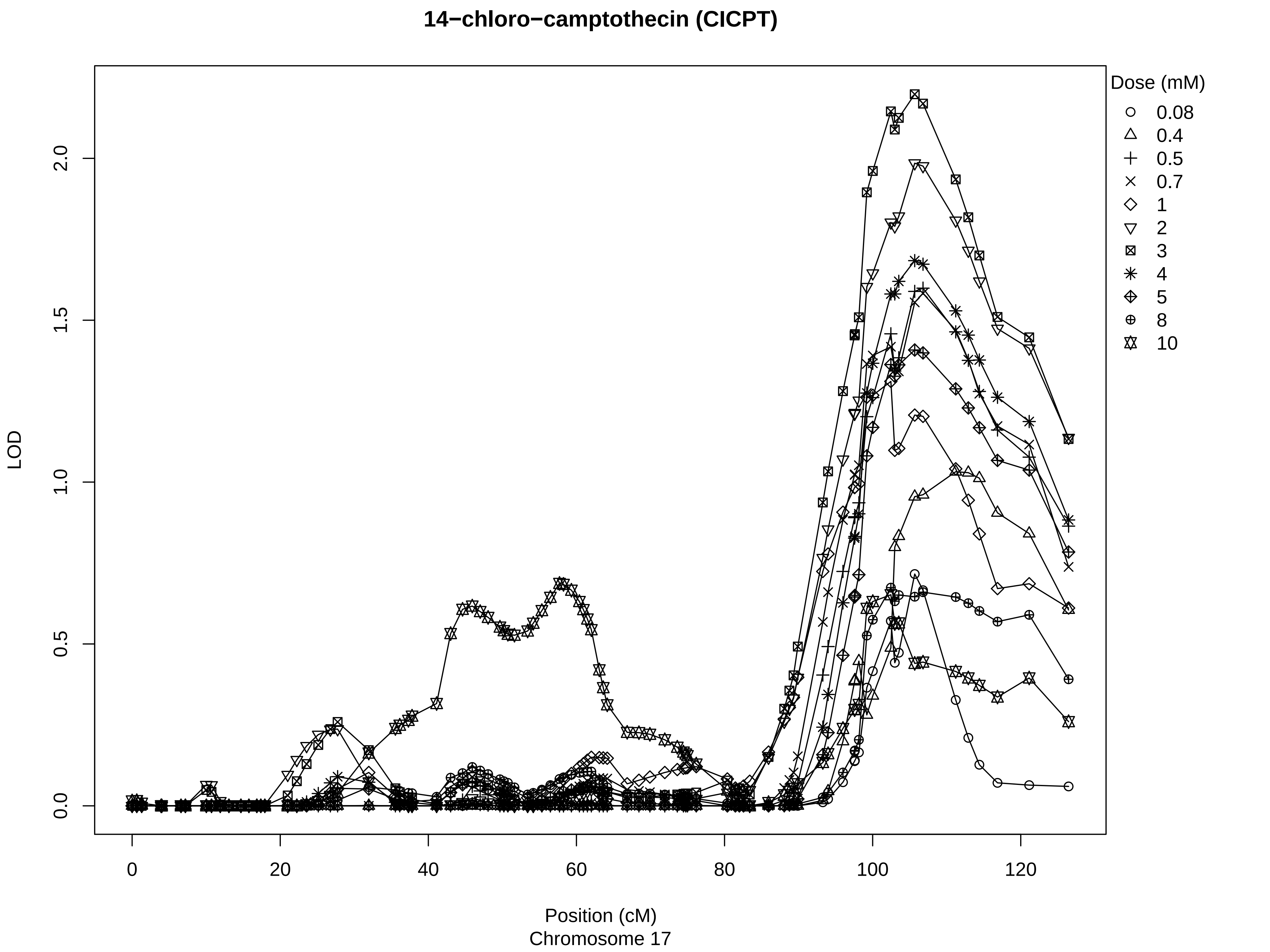
14−chloro−camptothecin (ClCPT)
Dose (mM)
0.08
0.4
0.5
0.7
1
2
3
4
5
8
10
LOD
0.0
0.5
1.0
1.5
2.0
0
20
40
60
80
100
120
Position (cM)
Chromosome 17

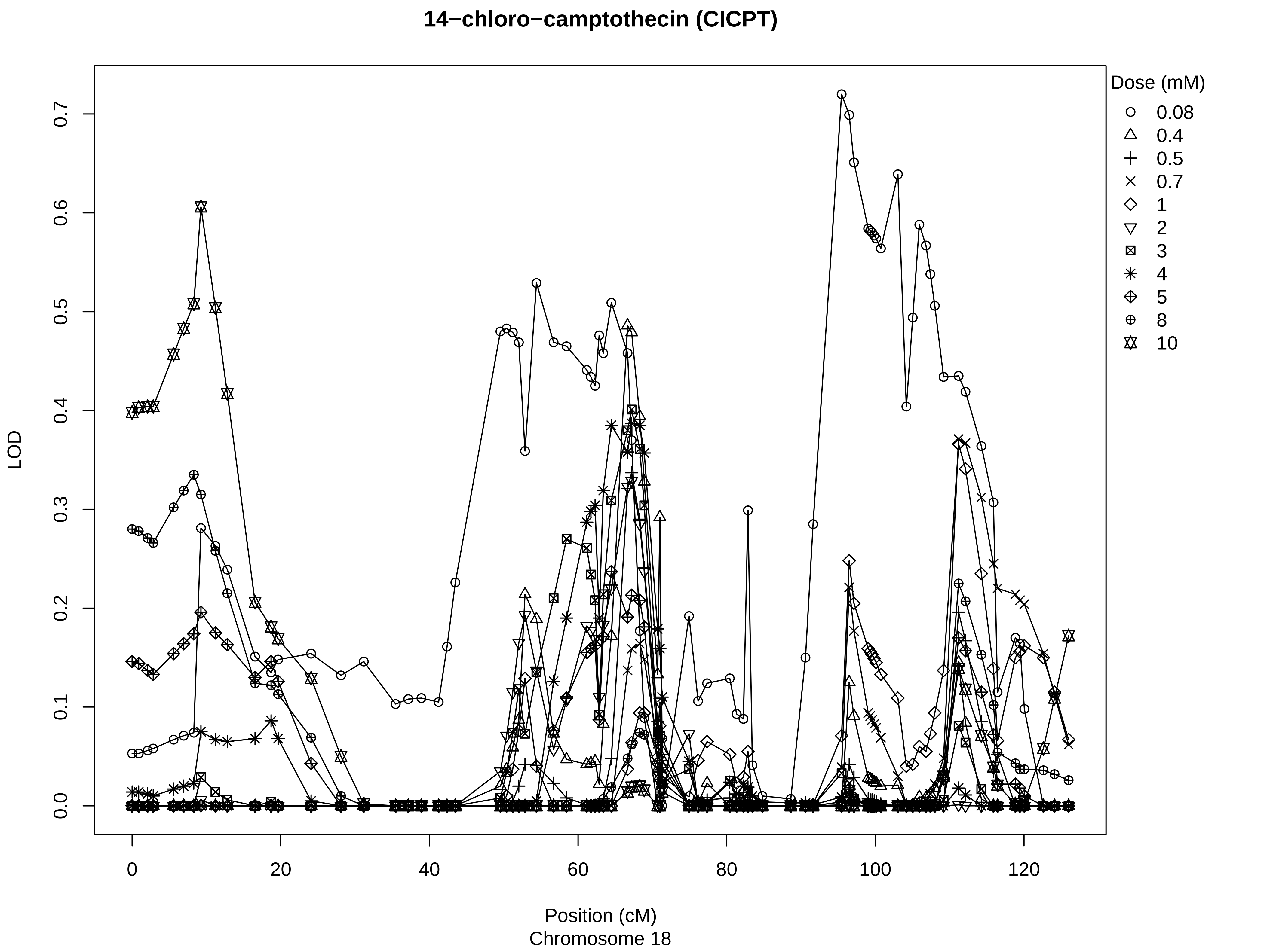
14−chloro−camptothecin (ClCPT)
LOD
0.0
0.1
0.2
0.3
0.4
0.5
0.6
0.7
0
20
40
60
80
100
120
Position (cM)
Chromosome 18
Dose (mM)
0.08
0.4
0.5
0.7
1
2
3
4
5
8
10

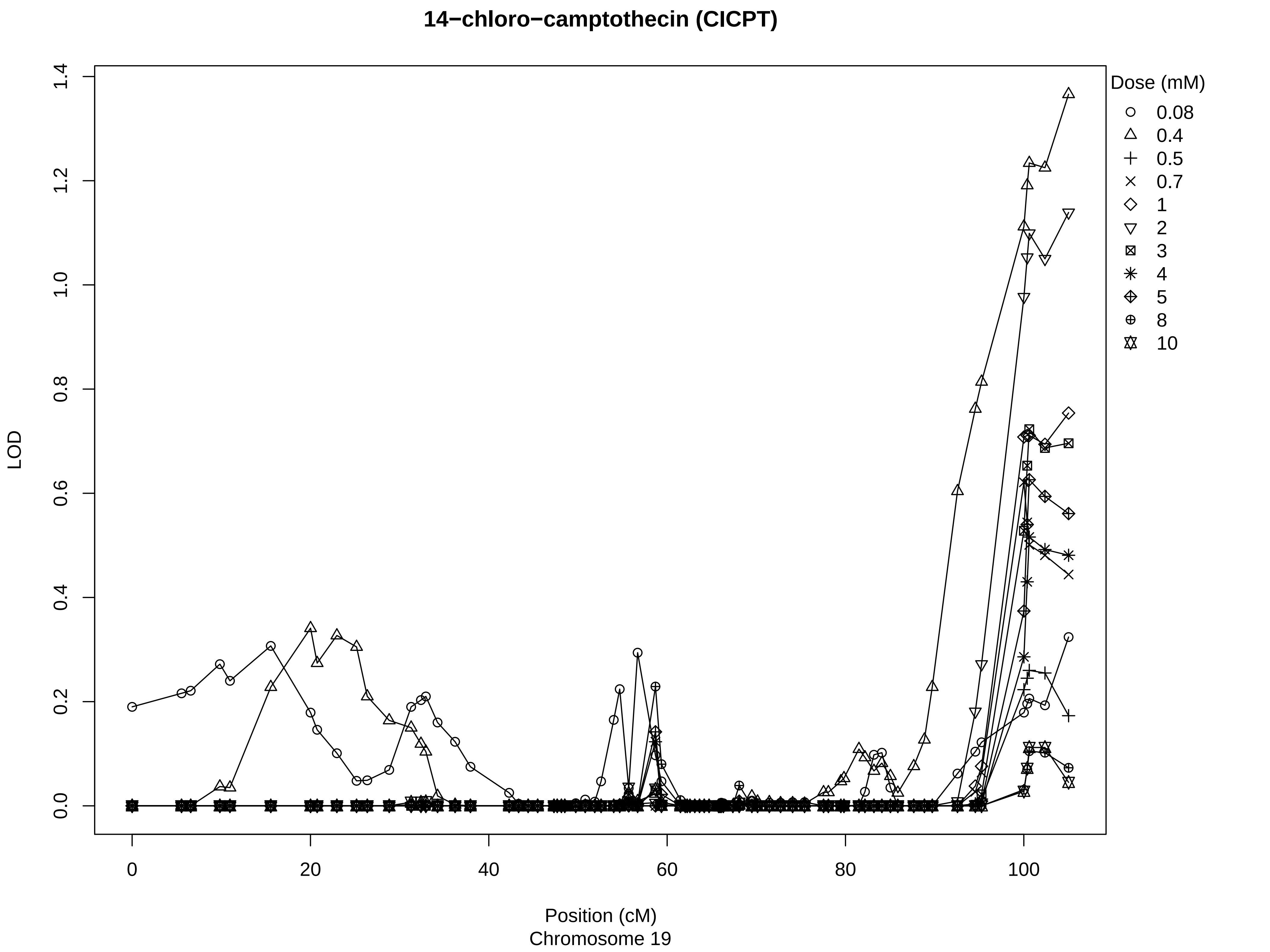

14−chloro−camptothecin (ClCPT)
LOD
0.0
0.2
0.4
0.6
0.8
1.0
1.2
1.4
0
20
40
60
80
100
Position (cM)
Chromosome 19
Dose (mM)
0.08
0.4
0.5
0.7
1
2
3
4
5
8
10

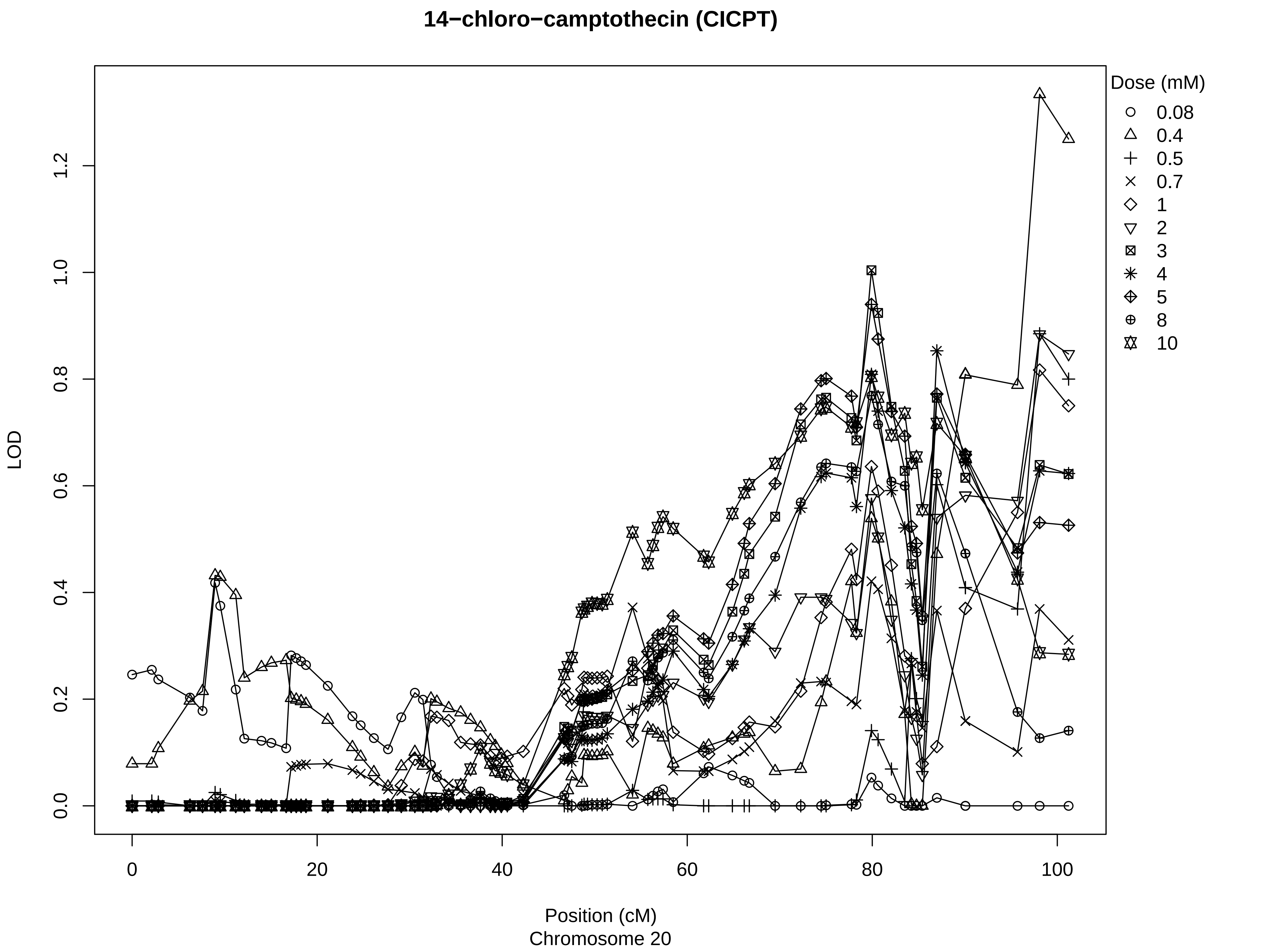

14−chloro−camptothecin (CICPT)
Dose (mM)
0.08
0.4
0.5
0.7
1
2
3
4
5
8
10
LOD
0.0
0.2
0.4
0.6
0.8
1.0
1.2
0
20
40
60
80
100
Position (cM)
Chromosome 20

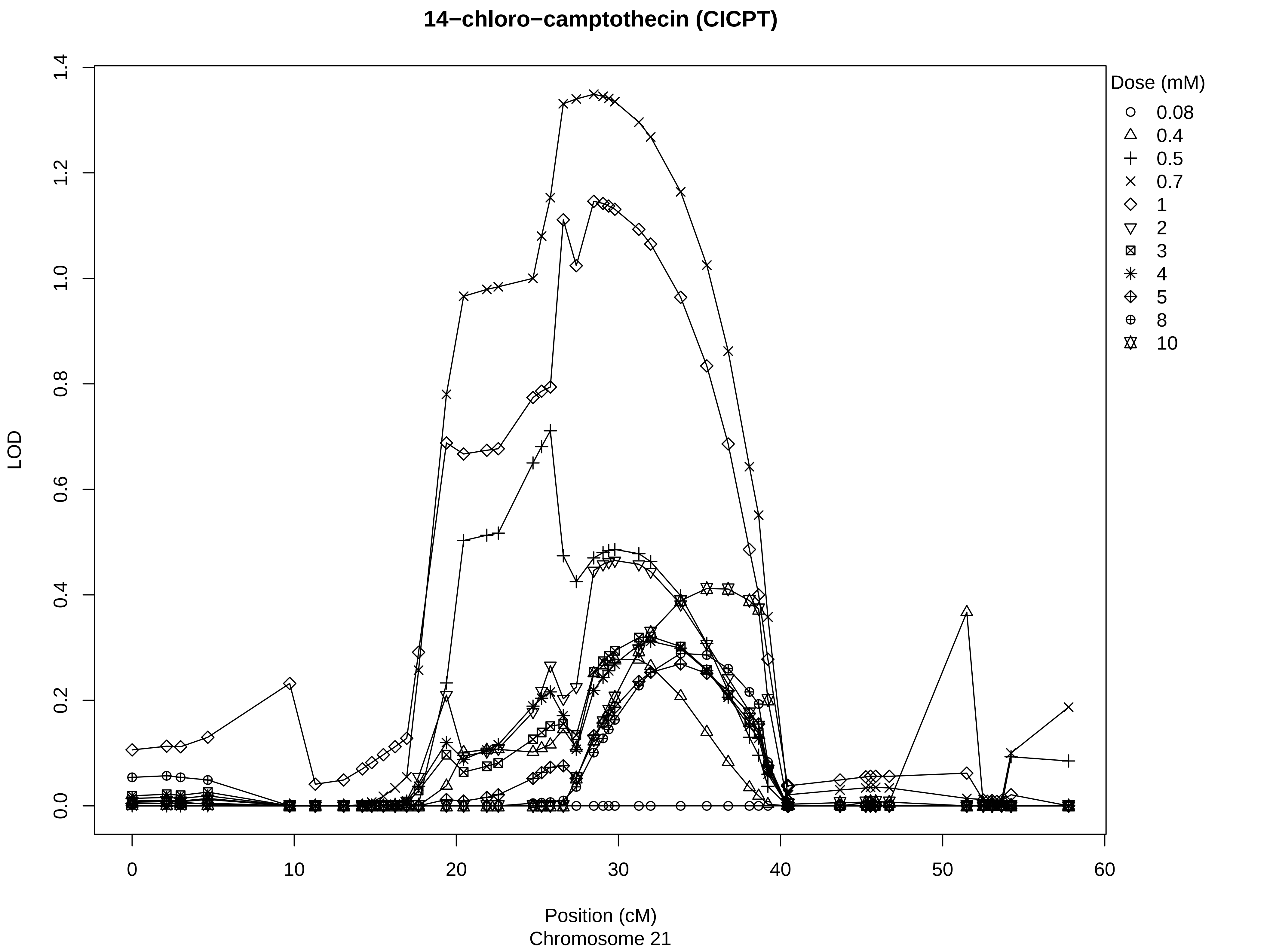
14−chloro−camptothecin (ClCPT)
LOD
Position (cM)
Chromosome 21
Dose (mM)
0.08
0.4
0.5
0.7
1
2
3
4
5
8
10
0.0
0.2
0.4
0.6
0.8
1.0
1.2
1.4
0
10
20
30
40
50
60

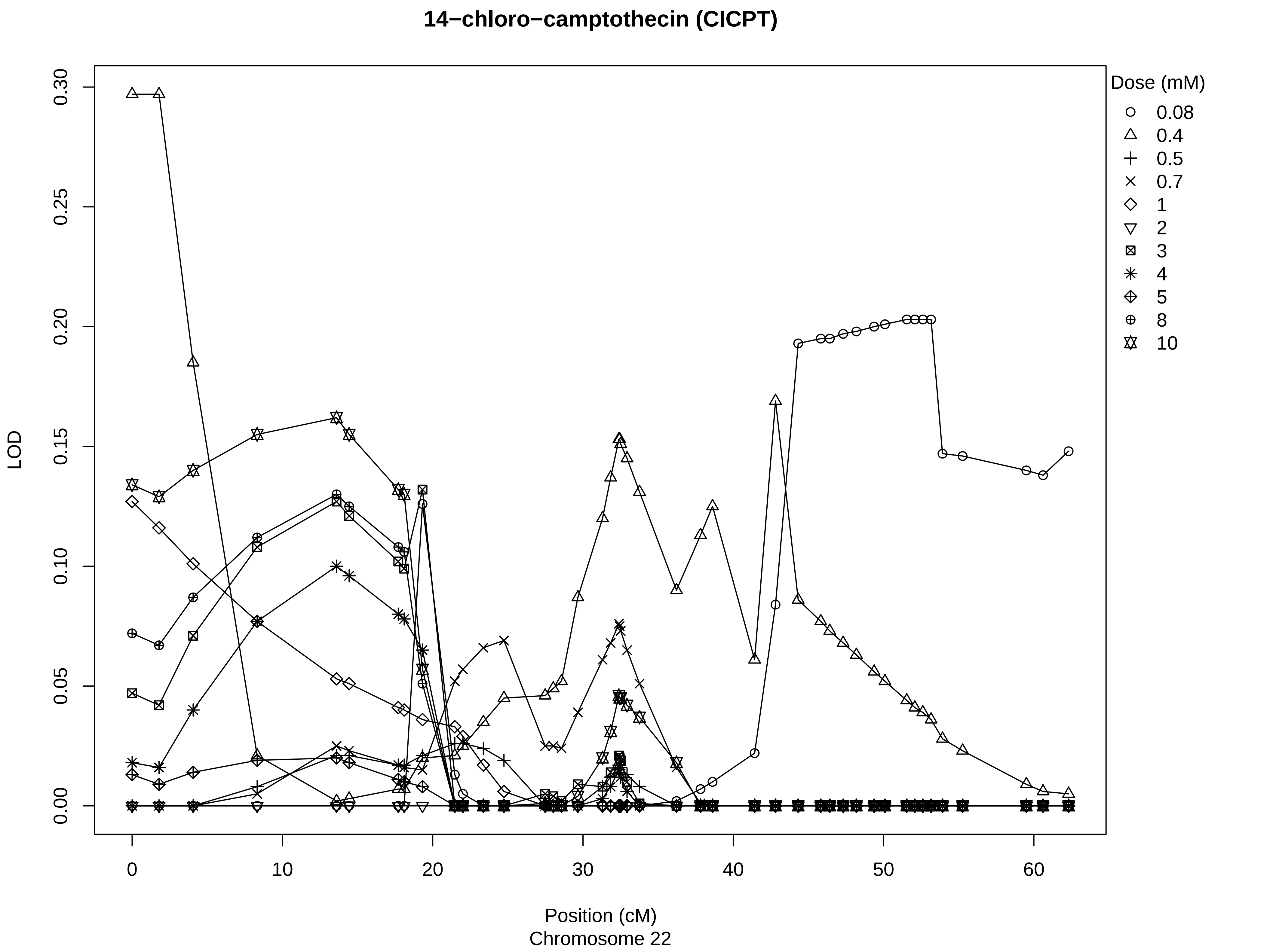
14−chloro−camptothecin (ClCPT)
LOD
0.00
0.05
0.10
0.15
0.20
0.25
0.30
0
10
20
30
40
50
60
Position (cM)
Chromosome 22
Dose (mM)
0.08
0.4
0.5
0.7
1
2
3
4
5
8
10

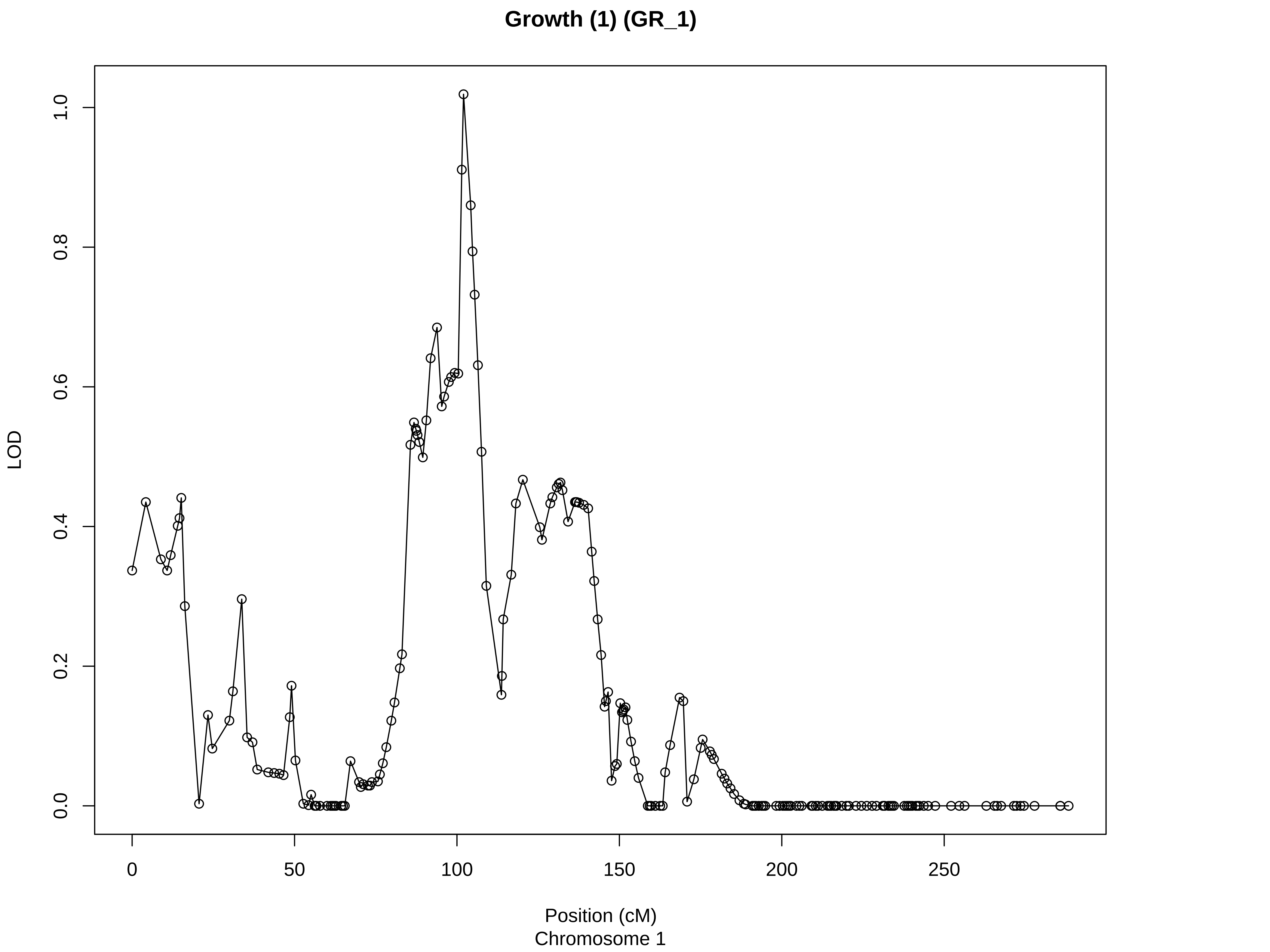

Growth (1) (GR_1)
LOD
0.0
0.2
0.4
0.6
0.8
1.0
0
50
100
150
200
250
Position (cM)
Chromosome 1

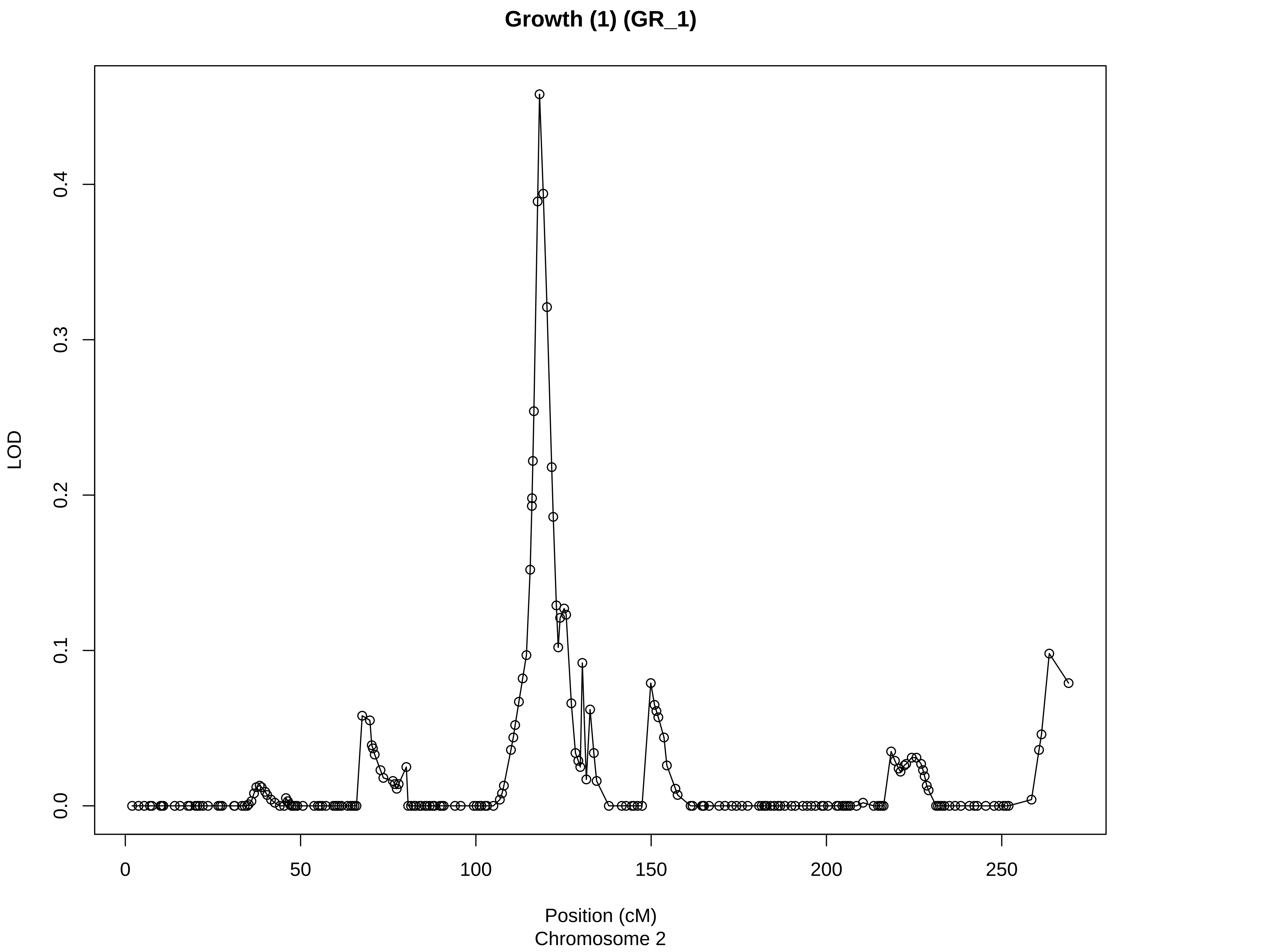
Growth (1) (GR_1)
LOD
0.0
0.1
0.2
0.3
0.4
0
50
100
150
200
250
Position (cM)
Chromosome 2

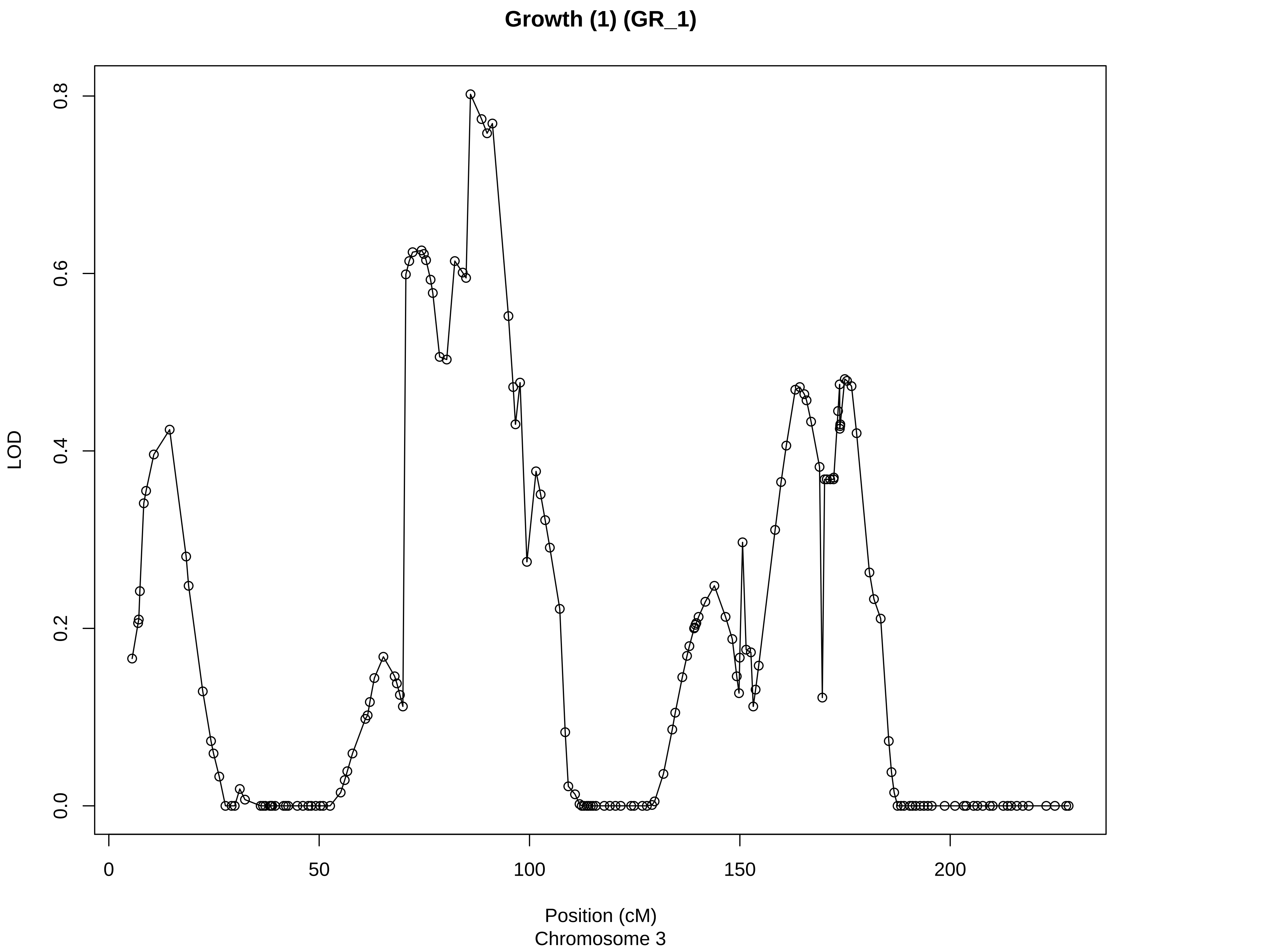
Growth (1) (GR_1)
LOD
0.0
0.2
0.4
0.6
0.8
0
50
100
150
200
Position (cM)
Chromosome 3

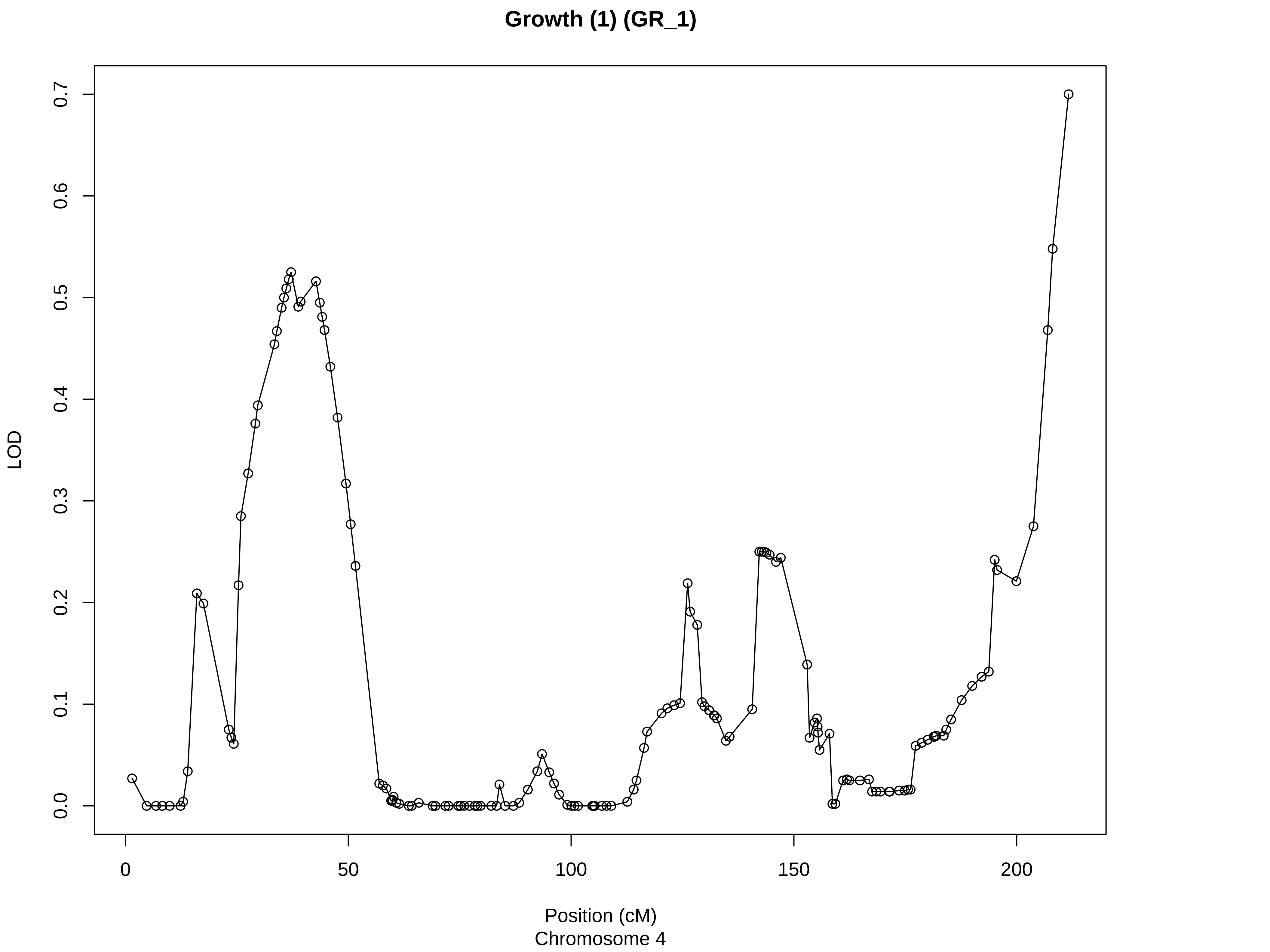
Growth (1) (GR_1)
LOD
0.0
0.1
0.2
0.3
0.4
0.5
0.6
0.7
0
50
100
150
200
Position (cM)
Chromosome 4

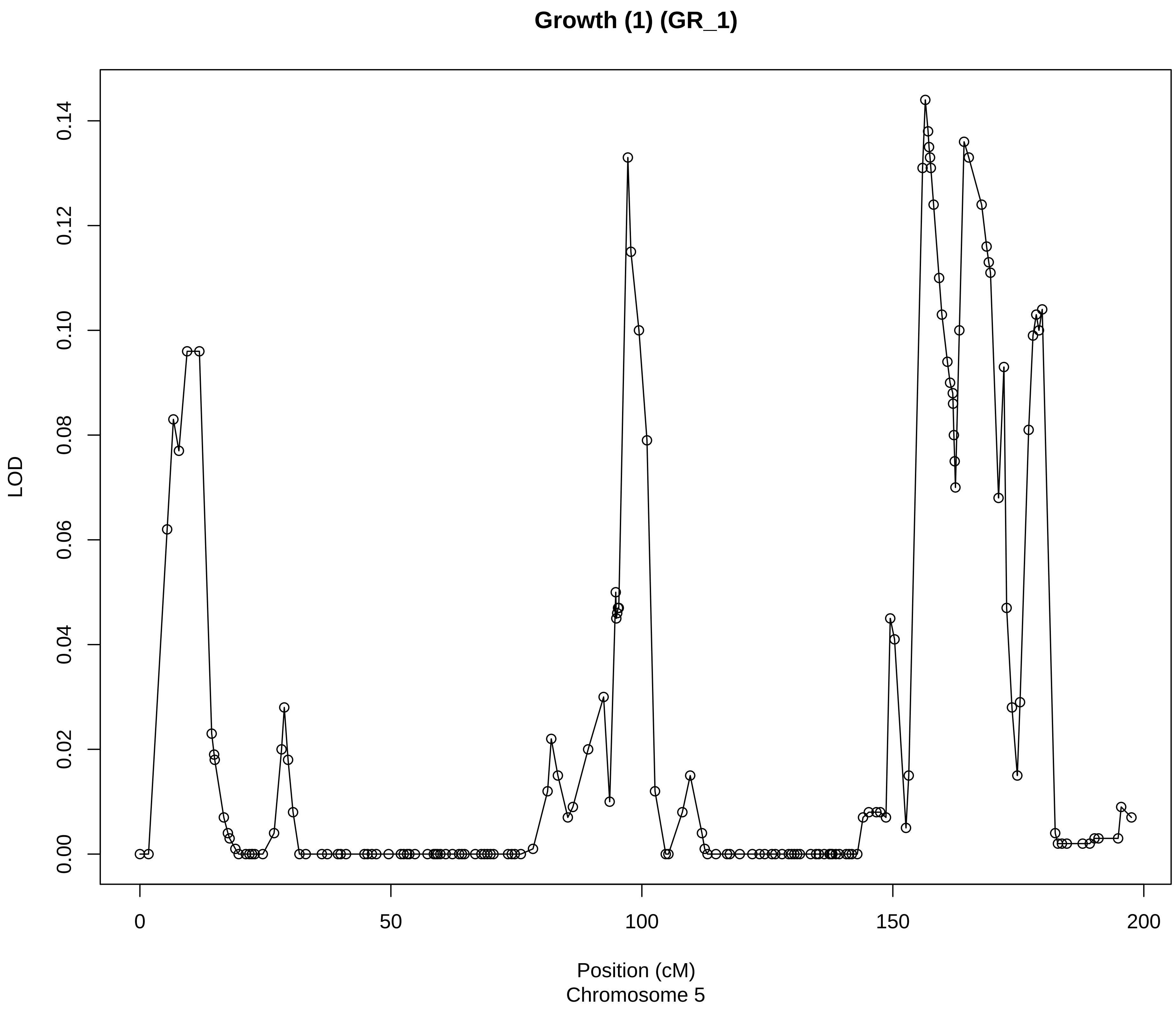
Growth (1) (GR_1)
LOD
0.00
0.02
0.04
0.06
0.08
0.10
0.12
0.14
0
50
100
150
200
Position (cM)
Chromosome 5

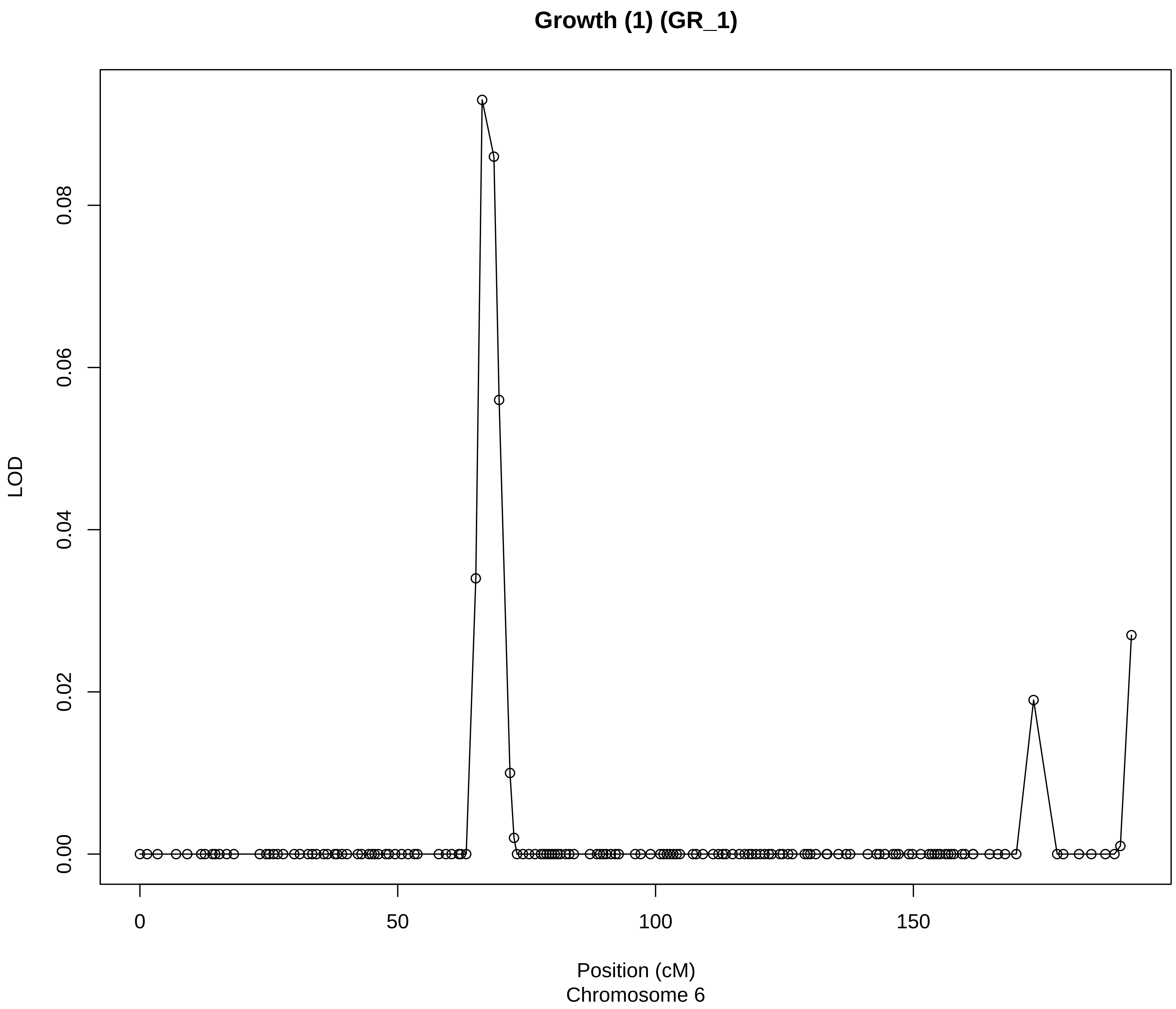

Growth (1) (GR_1)
LOD
0.00
0.02
0.04
0.06
0.08
0
50
100
150
Position (cM)
Chromosome 6

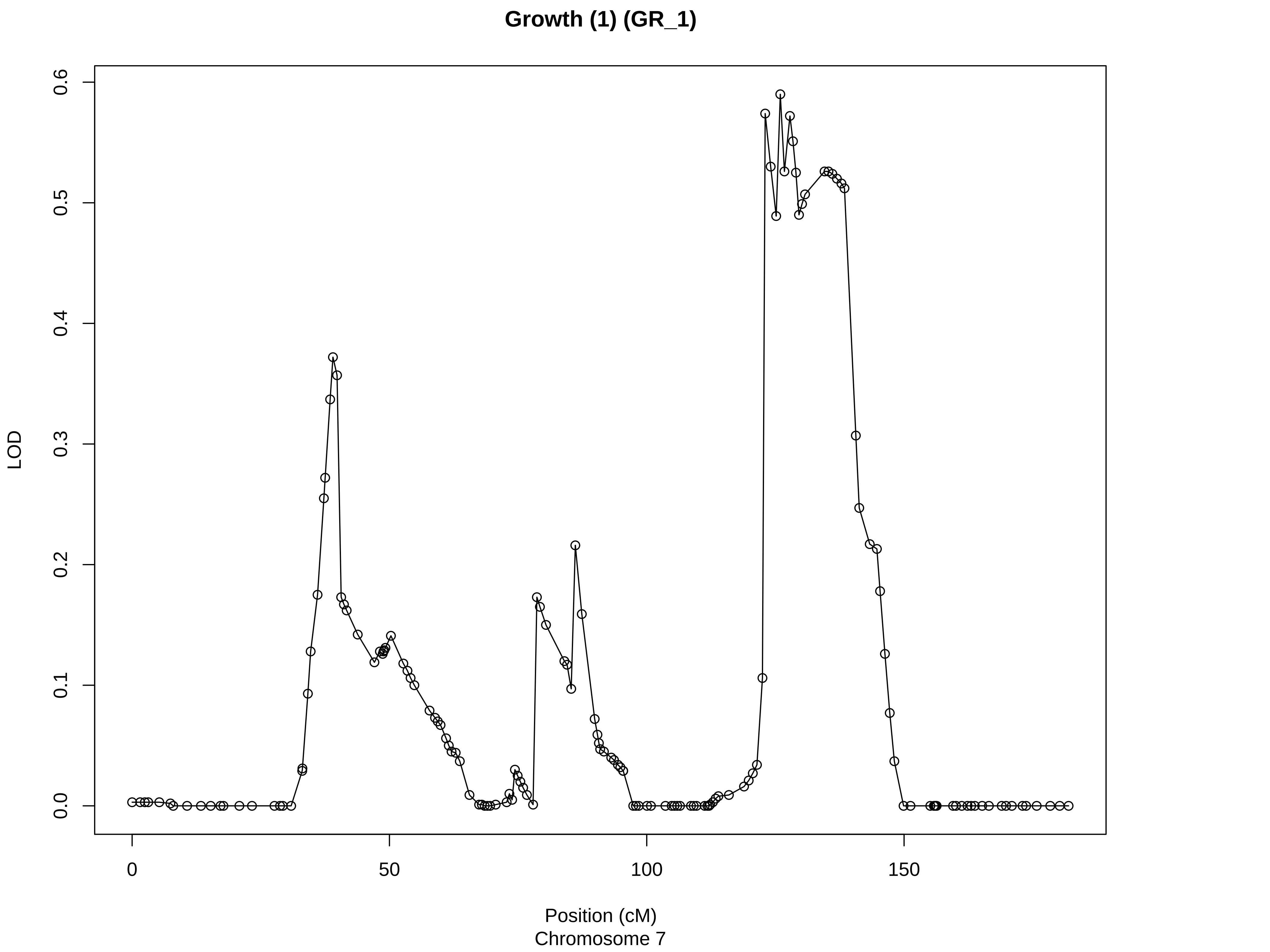

Growth (1) (GR_1)
LOD
0.0
0.1
0.2
0.3
0.4
0.5
0.6
0
50
100
150
Position (cM)
Chromosome 7

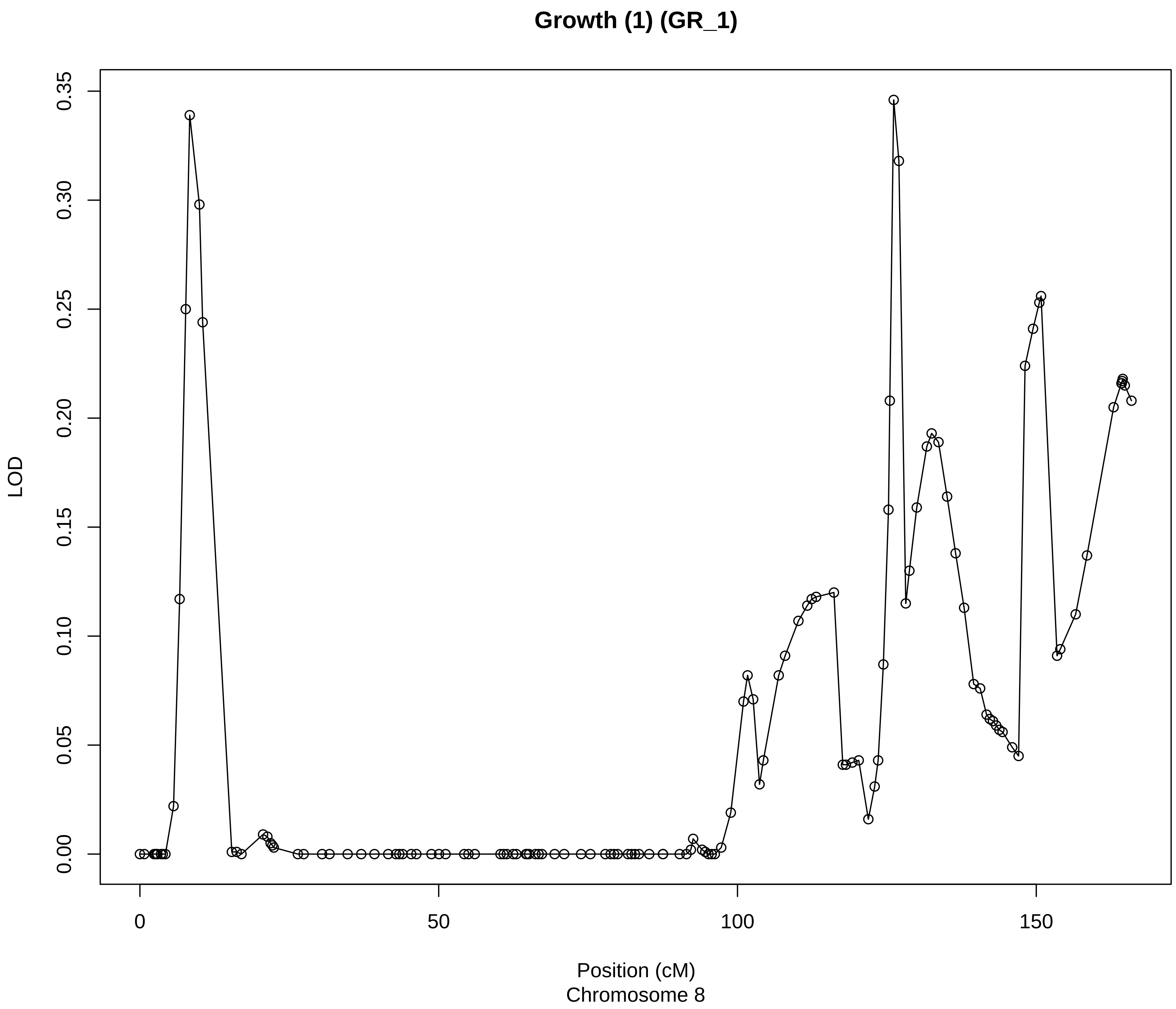
Growth (1) (GR_1)
LOD
0.00
0.05
0.10
0.15
0.20
0.25
0.30
0.35
0
50
100
150
Position (cM)
Chromosome 8

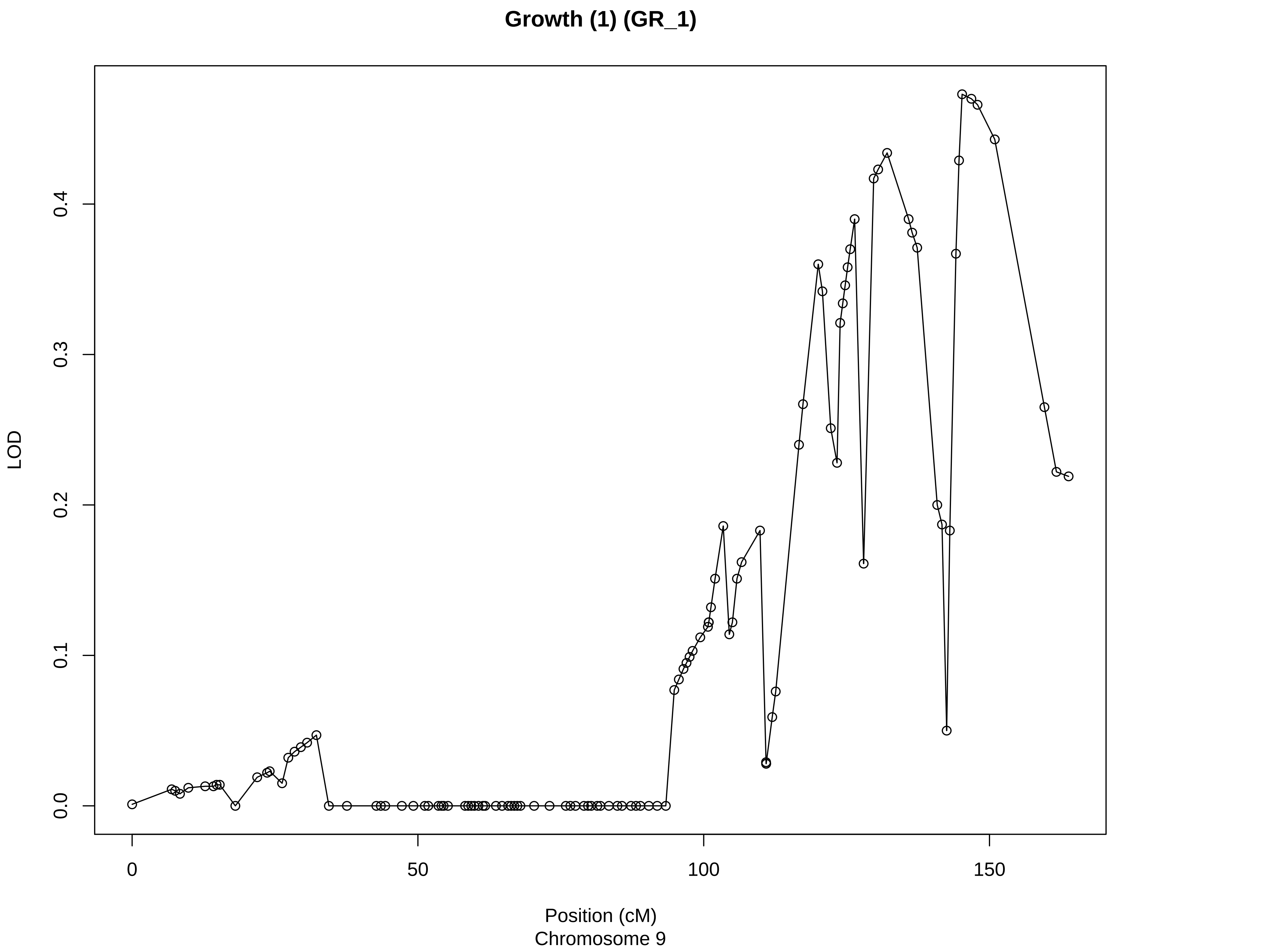

Growth (1) (GR_1)
LOD
0.0
0.1
0.2
0.3
0.4
0
50
100
150
Position (cM)
Chromosome 9

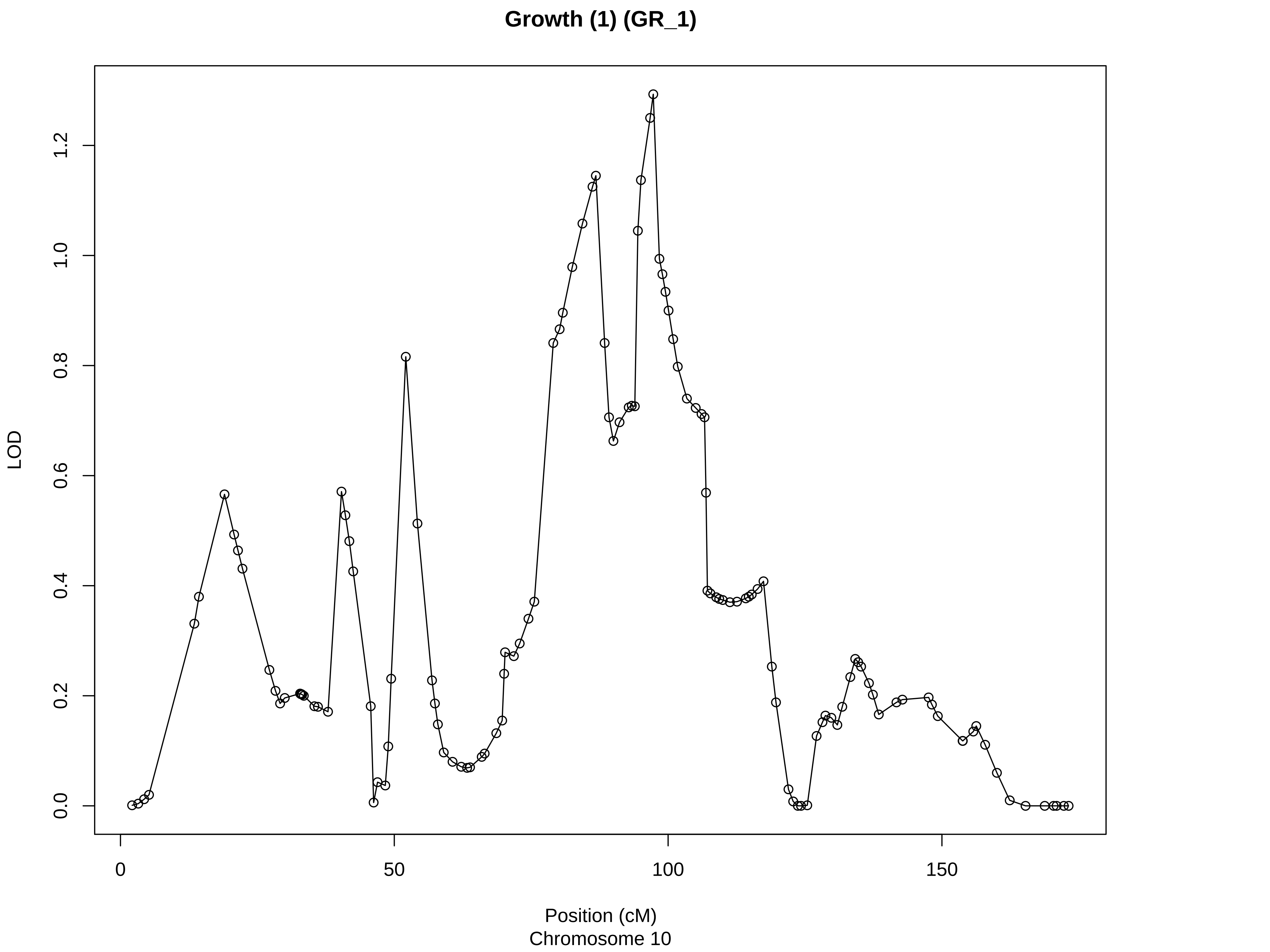
Growth (1) (GR_1)
LOD
0.0
0.2
0.4
0.6
0.8
1.0
1.2
0
50
100
150
Position (cM)
Chromosome 10

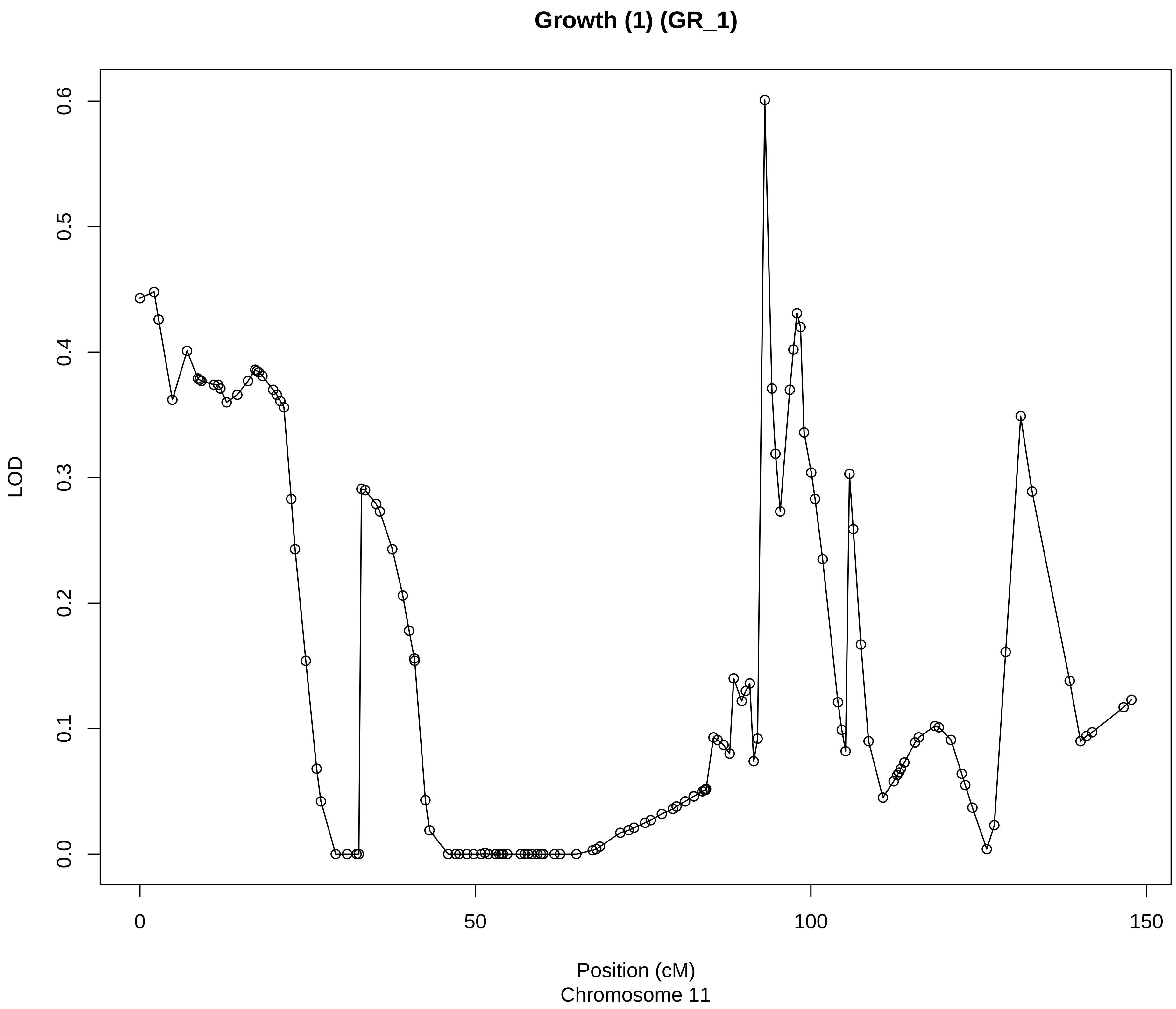

Growth (1) (GR_1)
LOD
0.0
0.1
0.2
0.3
0.4
0.5
0.6
0
50
100
150
Position (cM)
Chromosome 11

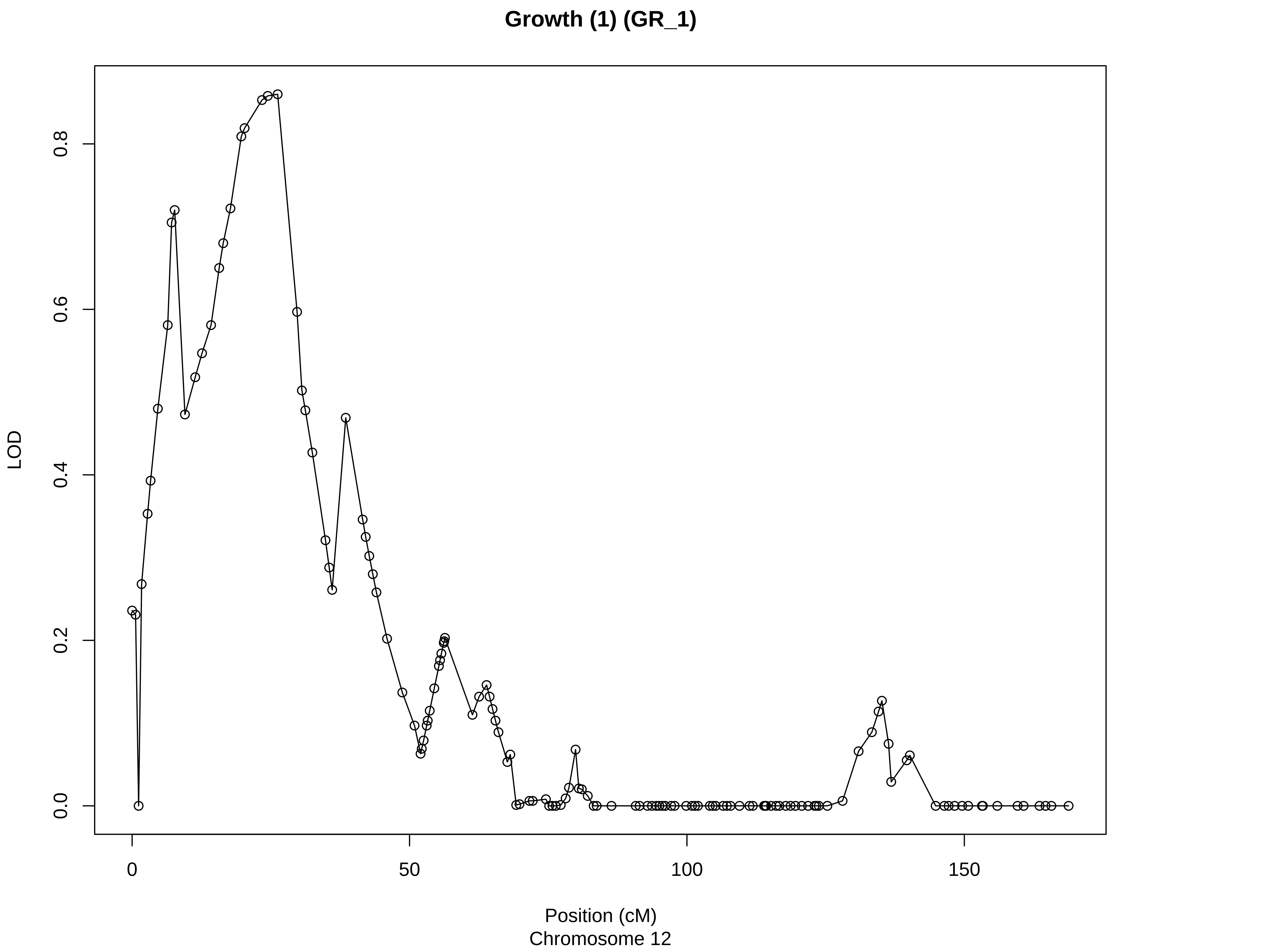
Growth (1) (GR_1)
LOD
0.0
0.2
0.4
0.6
0.8
0
50
100
150
Position (cM)
Chromosome 12

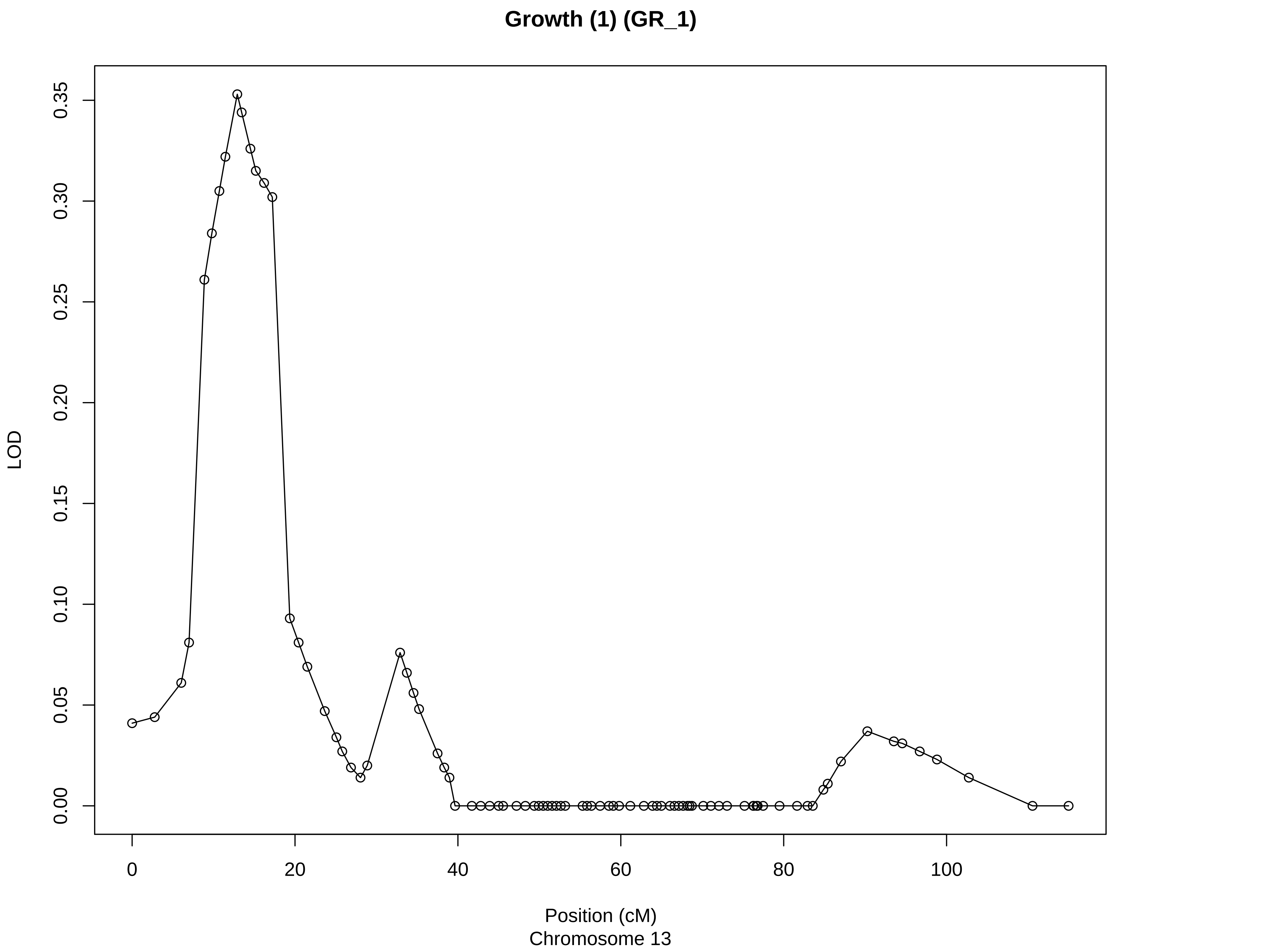

Growth (1) (GR_1)
LOD
0.00
0.05
0.10
0.15
0.20
0.25
0.30
0.35
0
20
40
60
80
100
Position (cM)
Chromosome 13

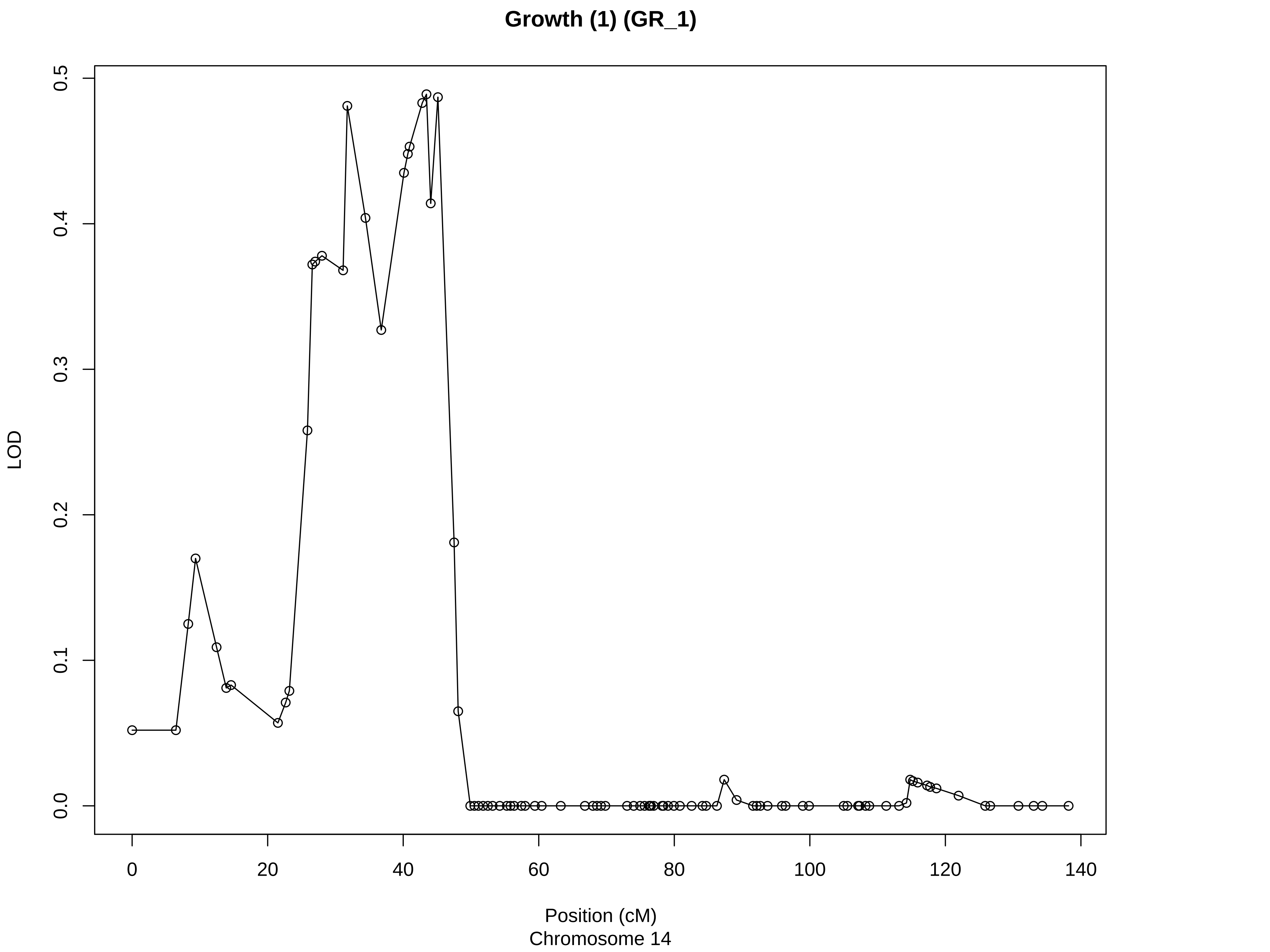
Growth (1) (GR_1)
LOD
0.0
0.1
0.2
0.3
0.4
0.5
0
20
40
60
80
100
120
140
Position (cM)
Chromosome 14

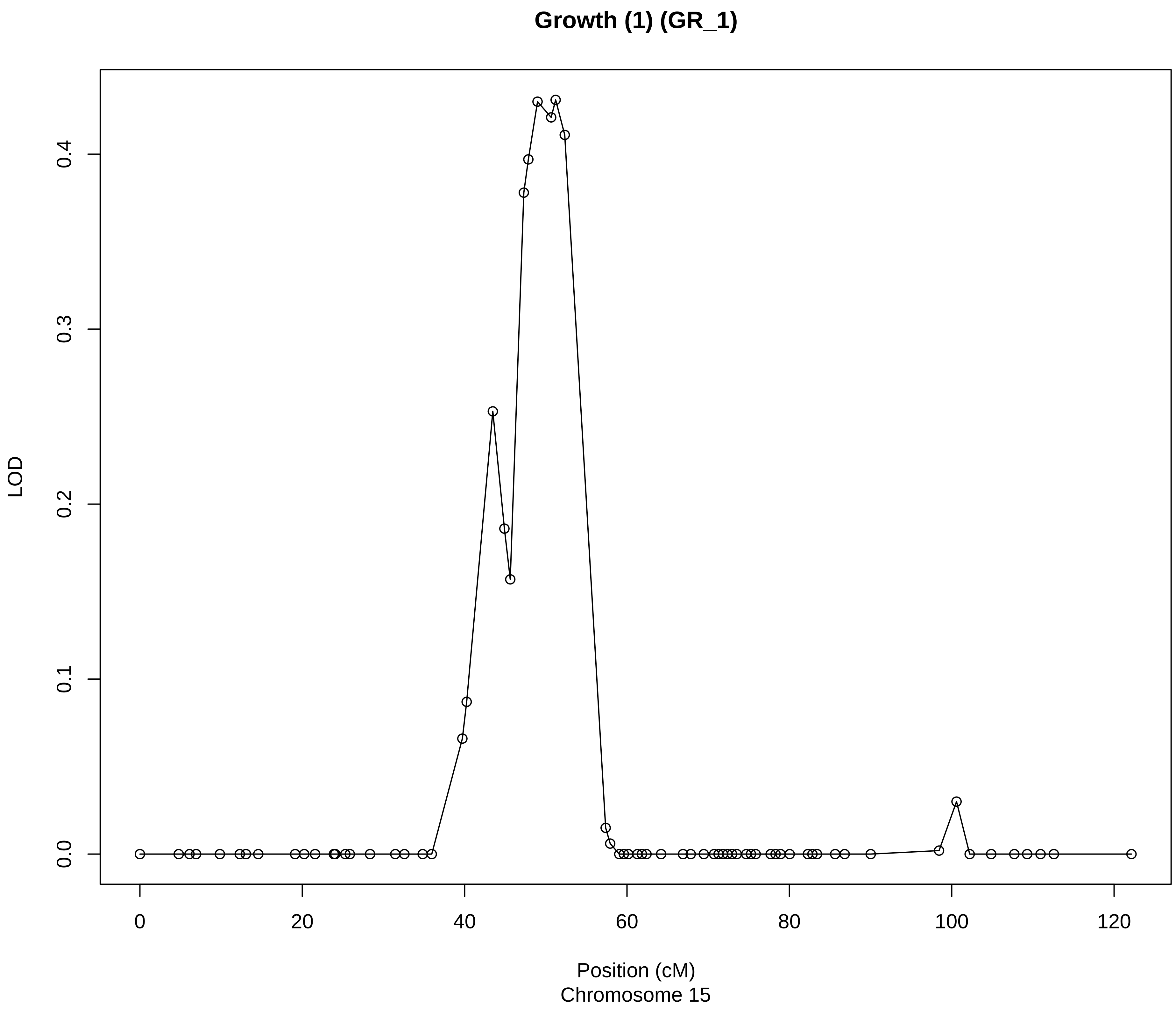

Growth (1) (GR_1)
LOD
0.0
0.1
0.2
0.3
0.4
0
20
40
60
80
100
120
Position (cM)
Chromosome 15

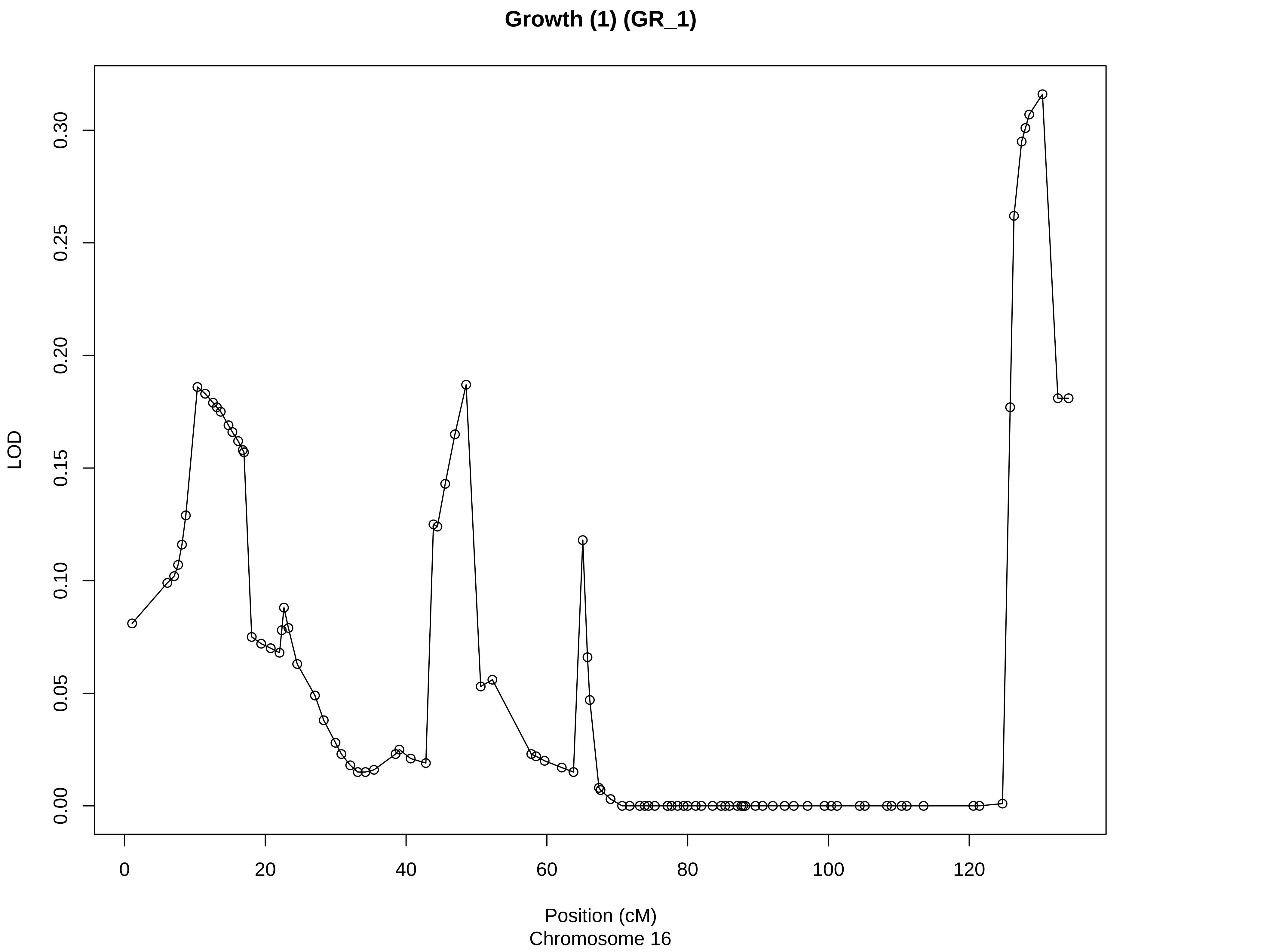
Growth (1) (GR_1)
LOD
0.00
0.05
0.10
0.15
0.20
0.25
0.30
0
20
40
60
80
100
120
Position (cM)
Chromosome 16

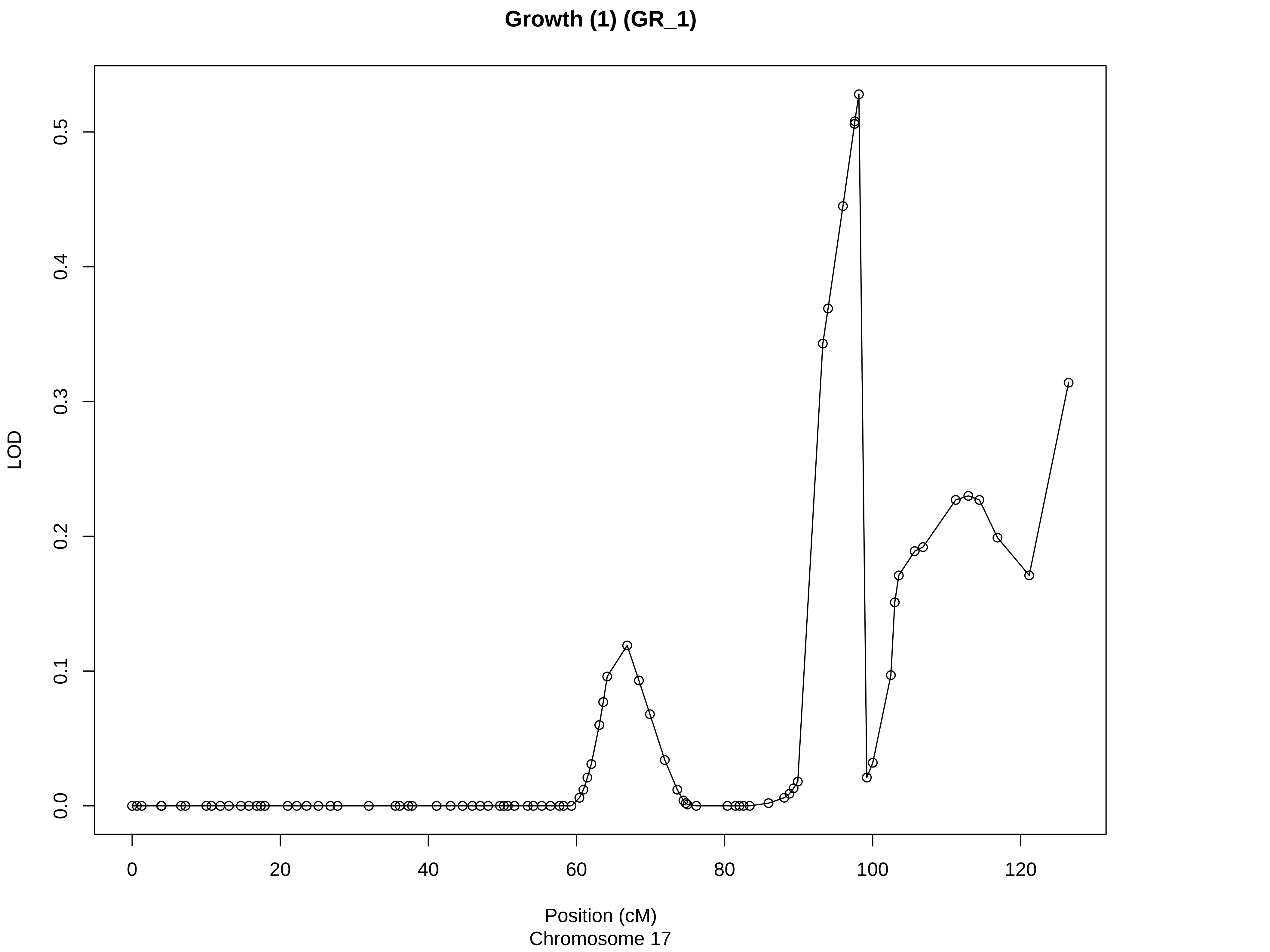
Growth (1) (GR_1)
LOD
0.0
0.1
0.2
0.3
0.4
0.5
0
20
40
60
80
100
120
Position (cM)
Chromosome 17

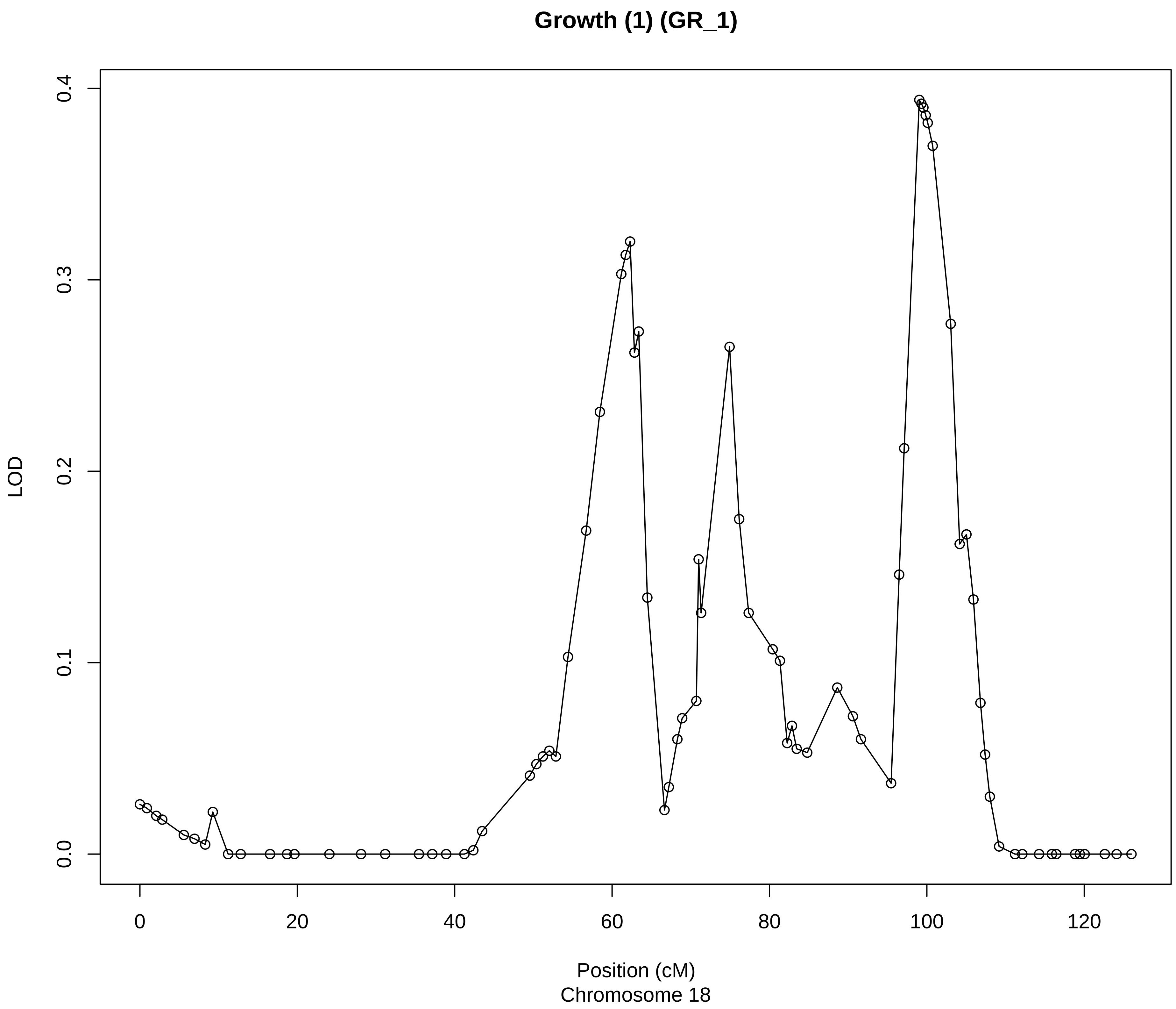

Growth (1) (GR_1)
LOD
0.0
0.1
0.2
0.3
0.4
0
20
40
60
80
100
120
Position (cM)
Chromosome 18

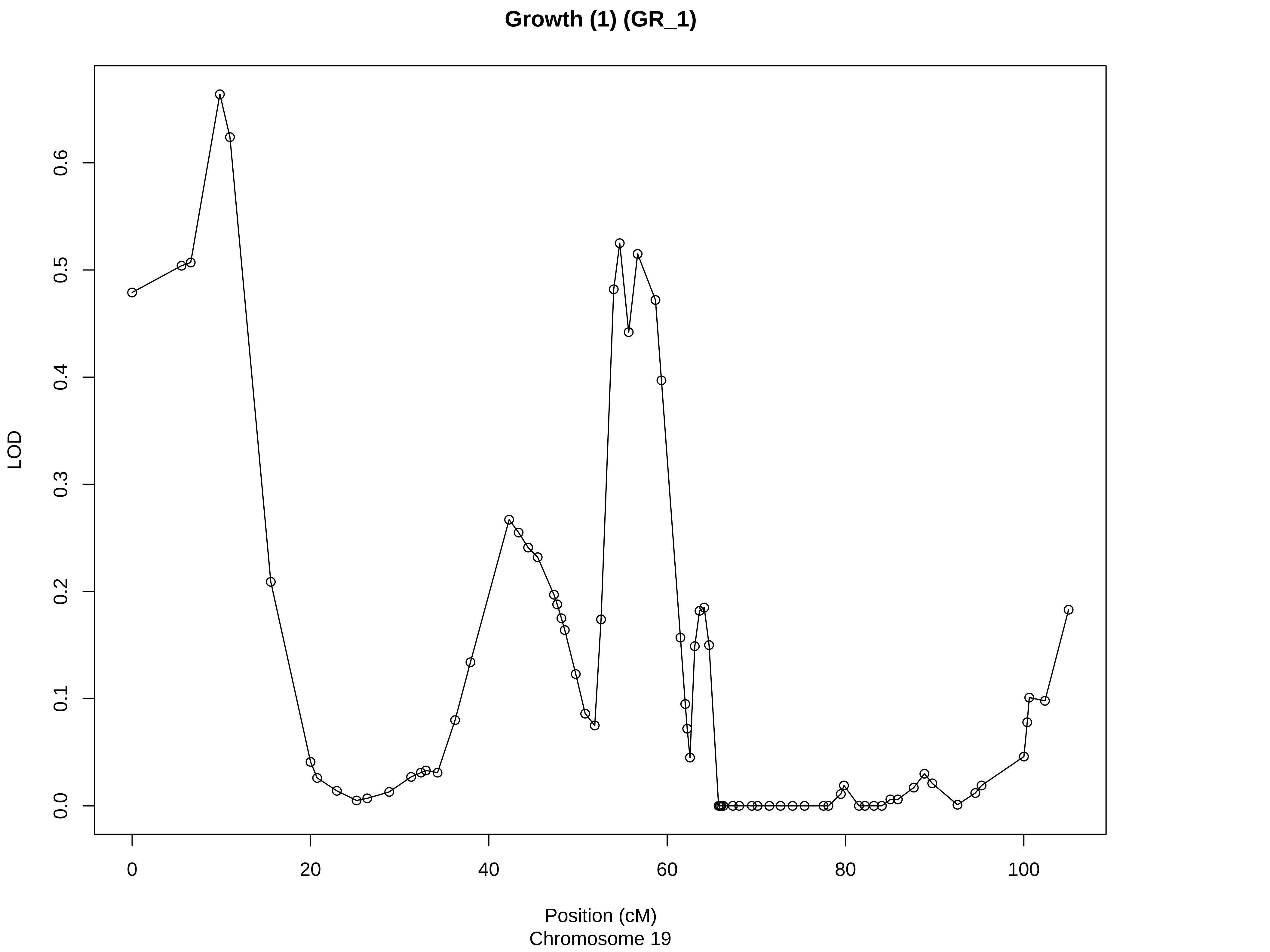

Growth (1) (GR_1)
LOD
0.0
0.1
0.2
0.3
0.4
0.5
0.6
0
20
40
60
80
100
Position (cM)
Chromosome 19

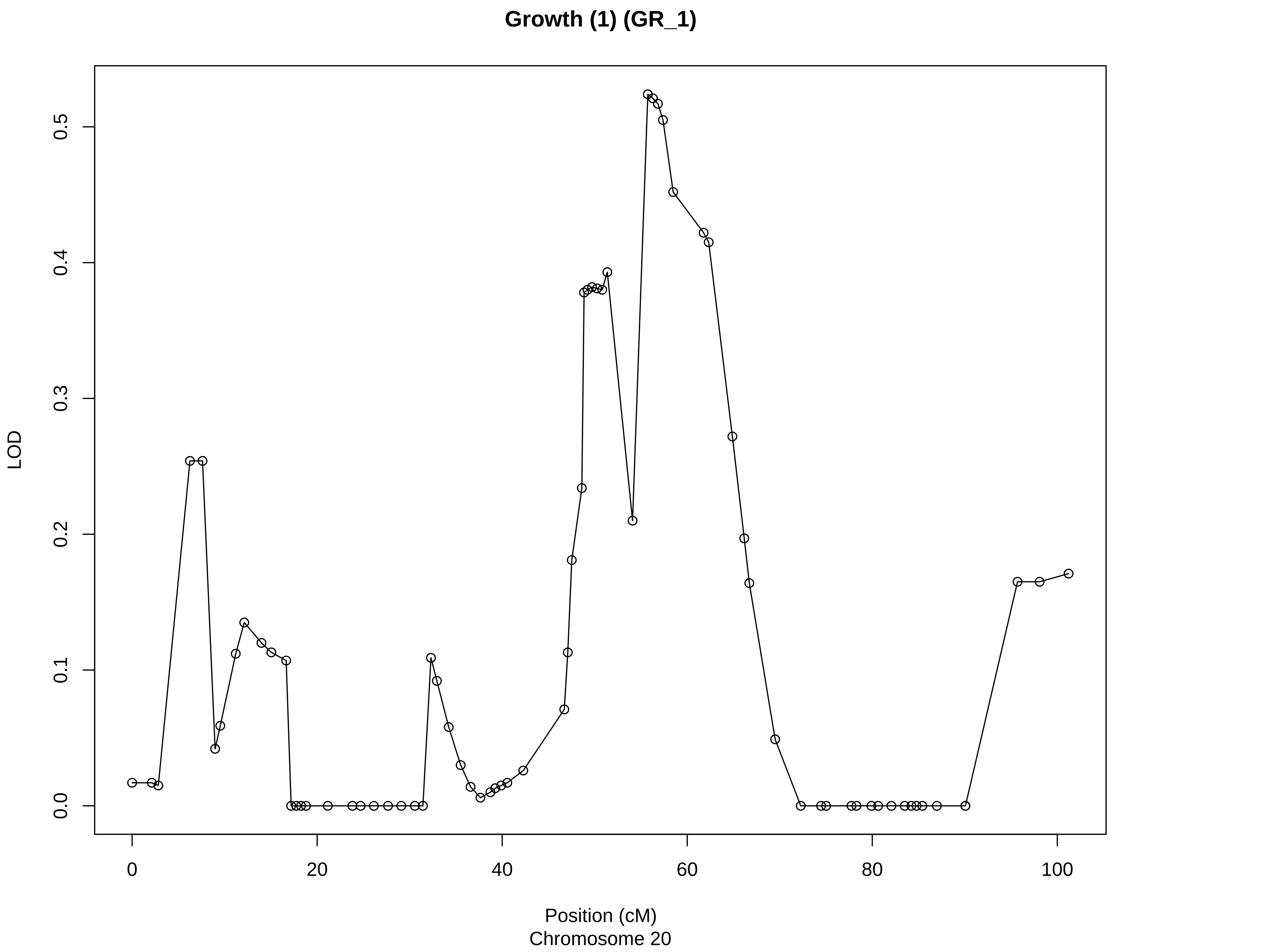

Growth (1) (GR_1)
LOD
0.0
0.1
0.2
0.3
0.4
0.5
0
20
40
60
80
100
Position (cM)
Chromosome 20

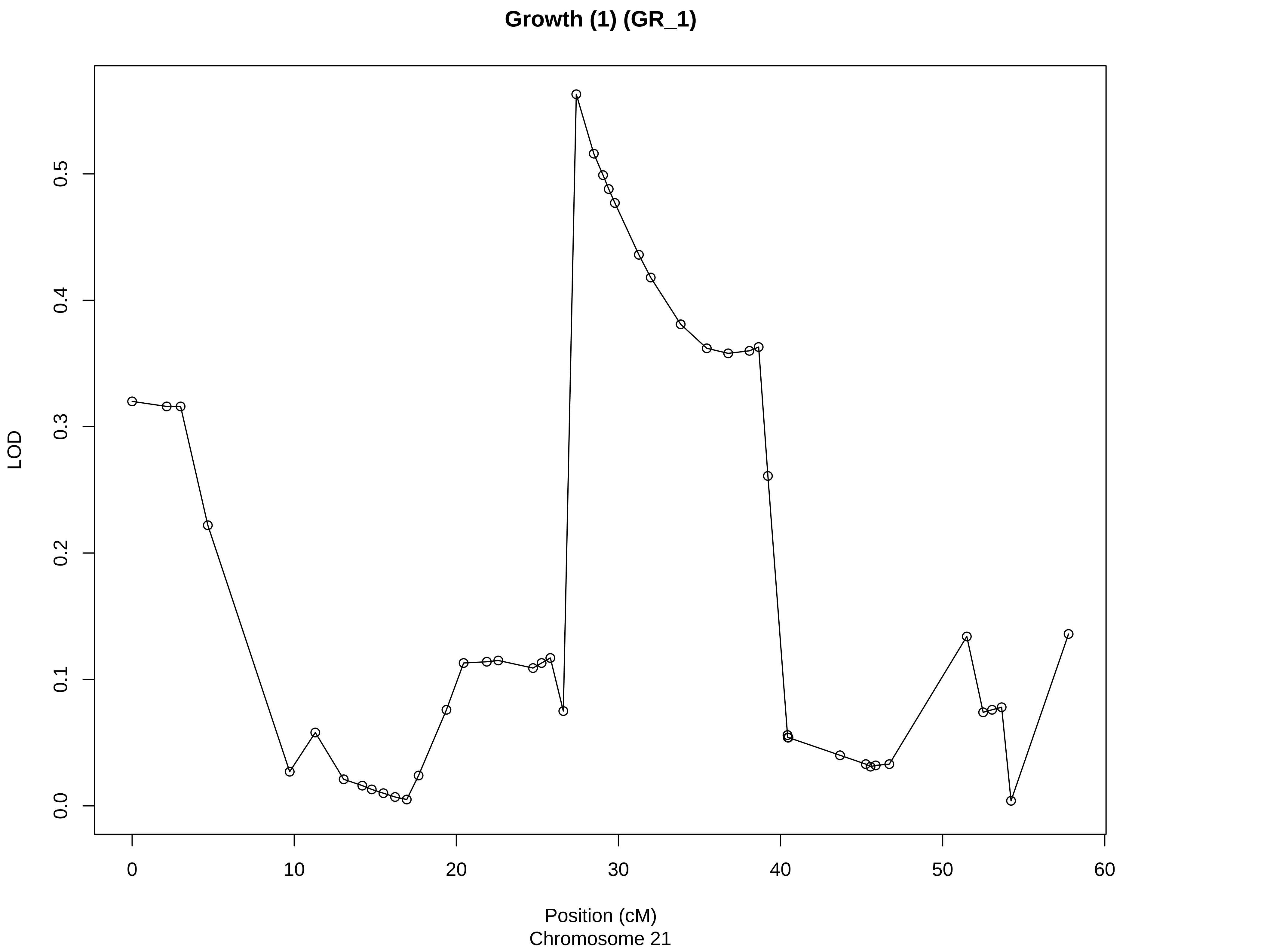

Growth (1) (GR_1)
LOD
0.0
0.1
0.2
0.3
0.4
0.5
0
10
20
30
40
50
60
Position (cM)
Chromosome 21

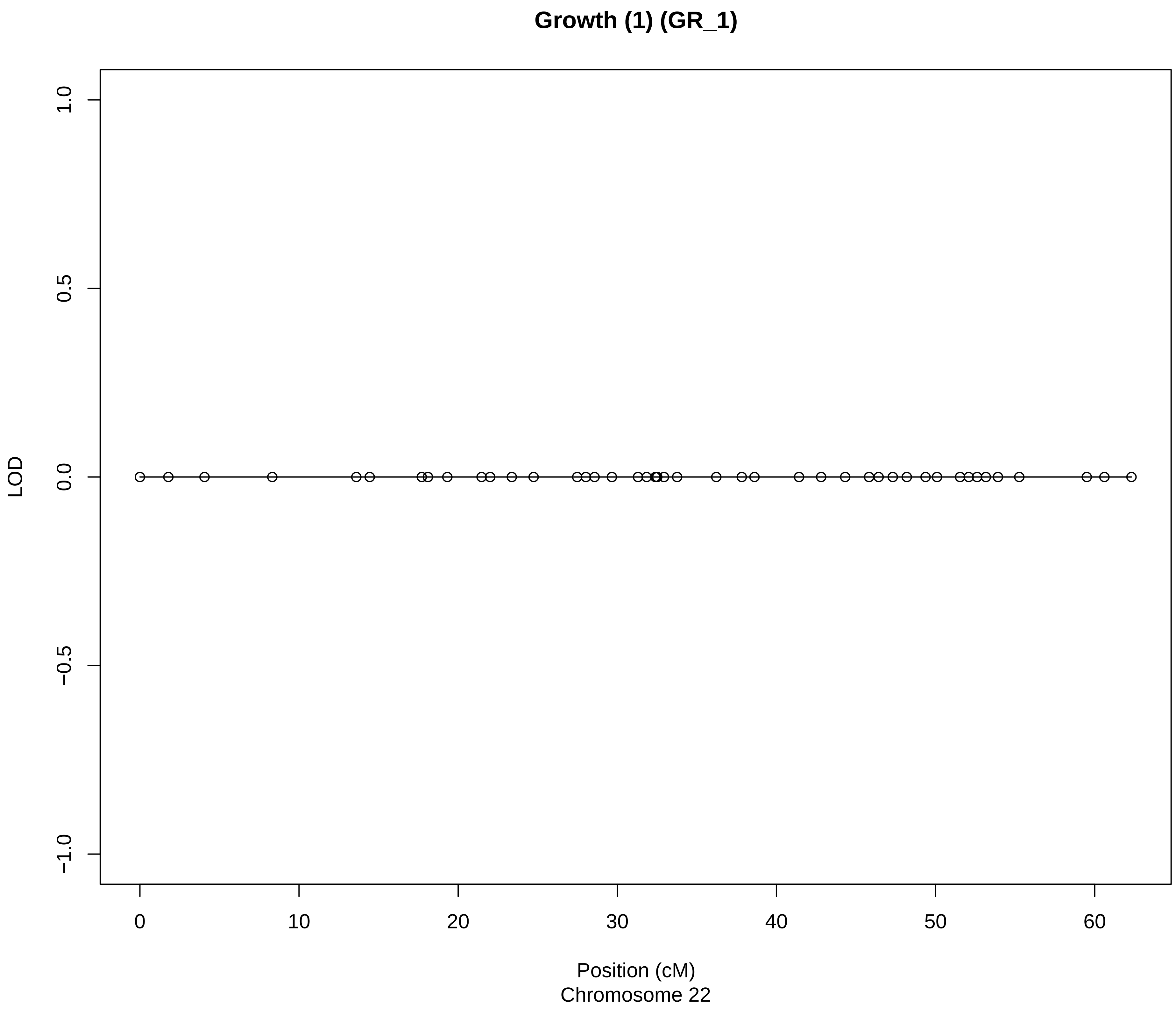
Growth (1) (GR_1)
LOD
1.0
0.5
0.0
−0.5
−1.0
0
10
20
30
40
50
60
Position (cM)
Chromosome 22

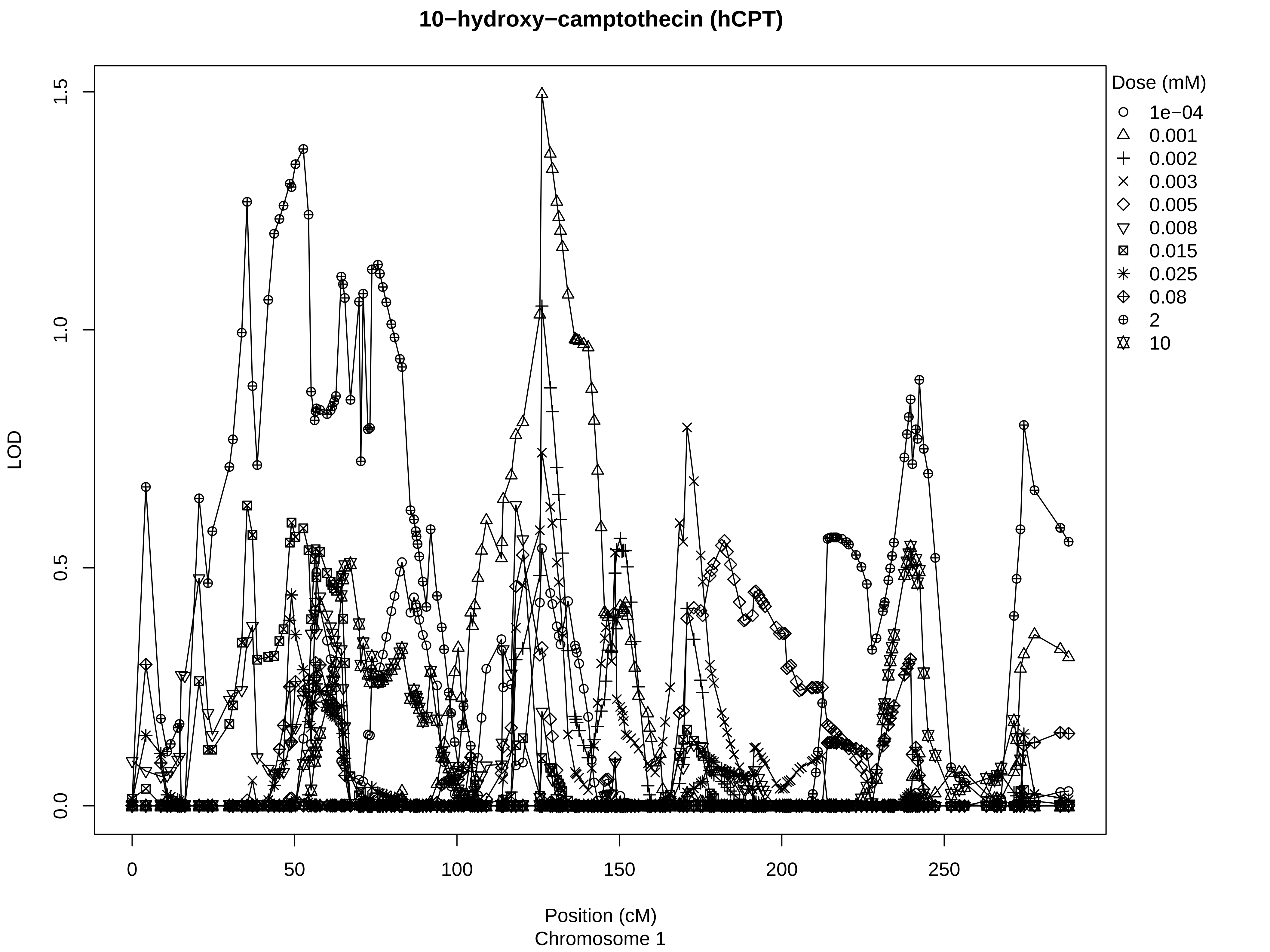
10−hydroxy−camptothecin (hCPT)
LOD
0.0
0.5
1.0
1.5
0
50
100
150
200
250
Position (cM)
Chromosome 1
Dose (mM)
1e−04
0.001
0.002
0.003
0.005
0.008
0.015
0.025
0.08
2
10

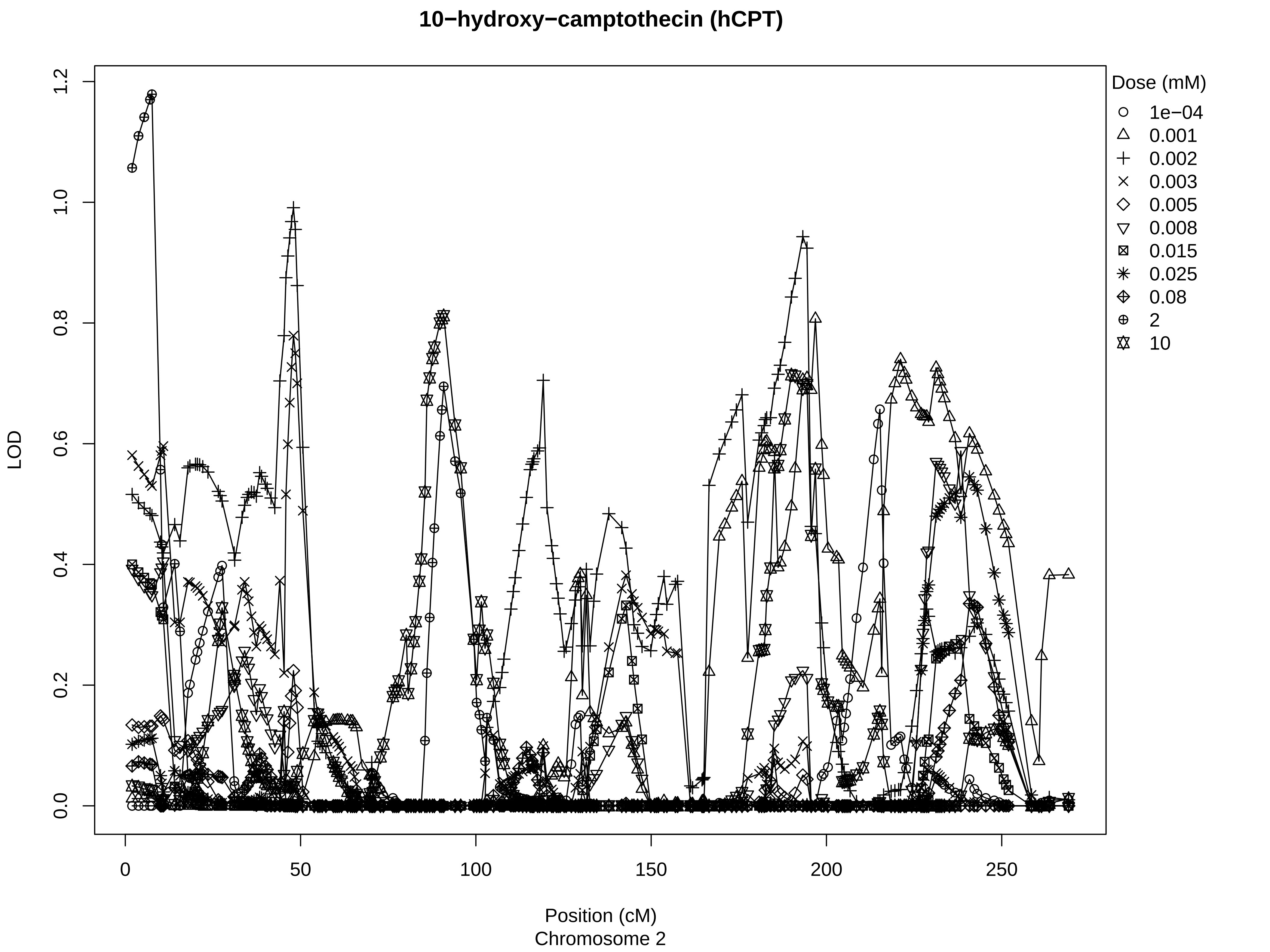
10−hydroxy−camptothecin (hCPT)
LOD
0.0
0.2
0.4
0.6
0.8
1.0
1.2
0
50
100
150
200
250
Position (cM)
Chromosome 2
Dose (mM)
1e−04
0.001
0.002
0.003
0.005
0.008
0.015
0.025
0.08
2
10

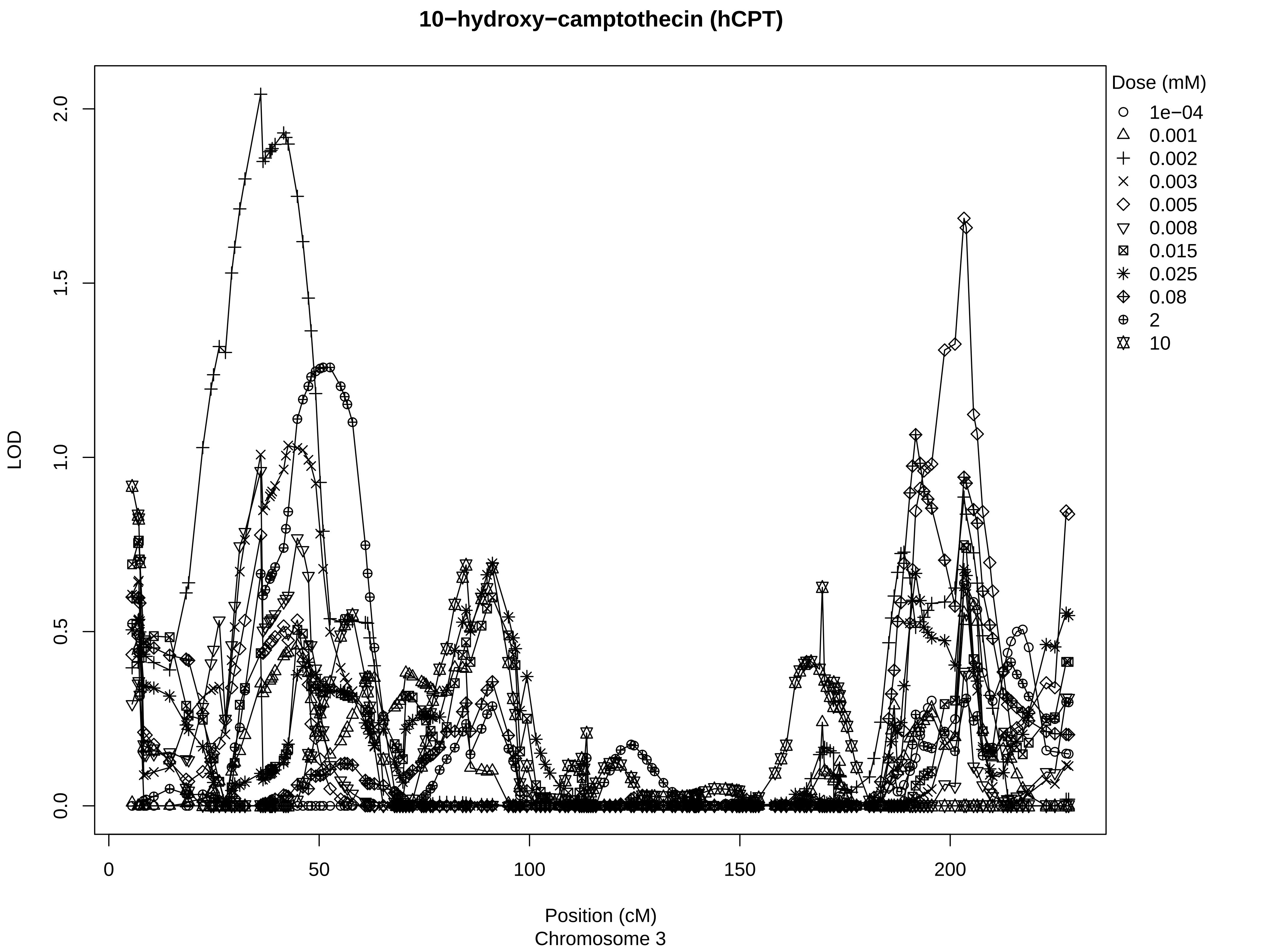

10−hydroxy−camptothecin (hCPT)
Dose (mM)
1e−04
0.001
0.002
0.003
0.005
0.008
0.015
0.025
0.08
2
10
LOD
0.0
0.5
1.0
1.5
2.0
0
50
100
150
200
Position (cM)
Chromosome 3

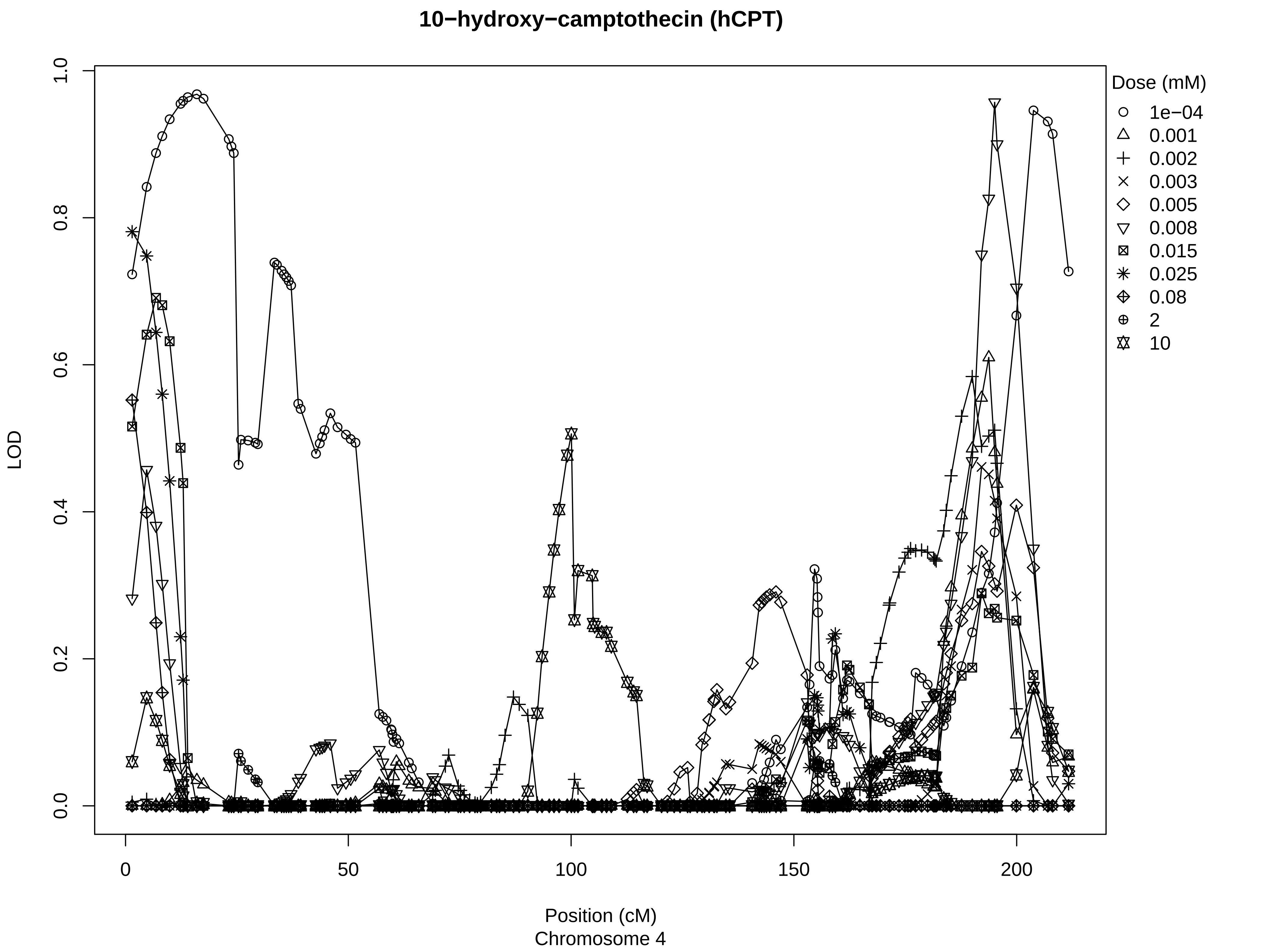
10−hydroxy−camptothecin (hCPT)
LOD
0.0
0.2
0.4
0.6
0.8
1.0
0
50
100
150
200
Position (cM)
Chromosome 4
Dose (mM)
1e−04
0.001
0.002
0.003
0.005
0.008
0.015
0.025
0.08
2
10

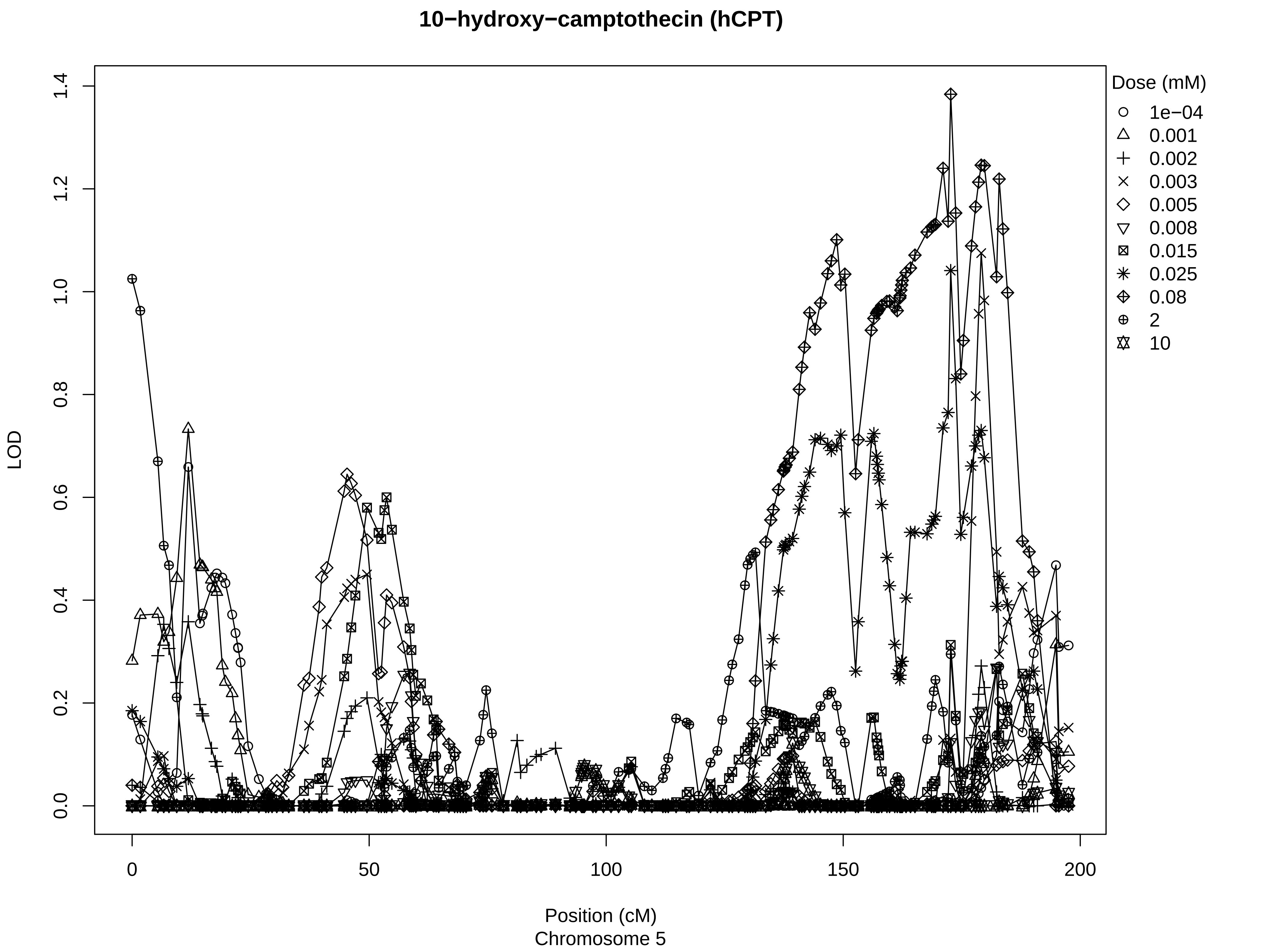
10−hydroxy−camptothecin (hCPT)
LOD
0.0
0.2
0.4
0.6
0.8
1.0
1.2
1.4
0
50
100
150
200
Position (cM)
Chromosome 5
Dose (mM)
1e−04
0.001
0.002
0.003
0.005
0.008
0.015
0.025
0.08
2
10

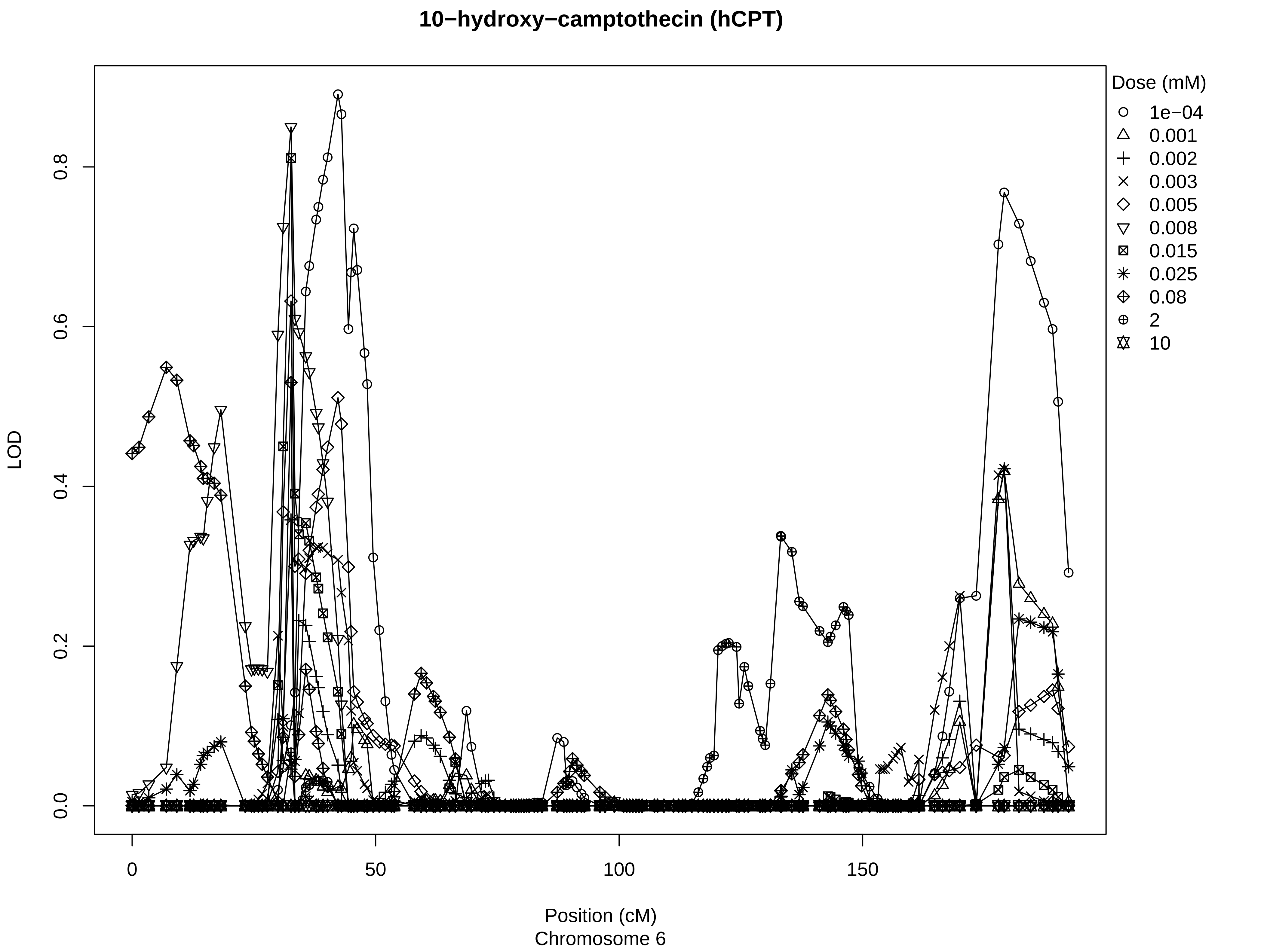
10−hydroxy−camptothecin (hCPT)
LOD
0.0
0.2
0.4
0.6
0.8
0
50
100
150
Position (cM)
Chromosome 6
Dose (mM)
1e−04
0.001
0.002
0.003
0.005
0.008
0.015
0.025
0.08
2
10

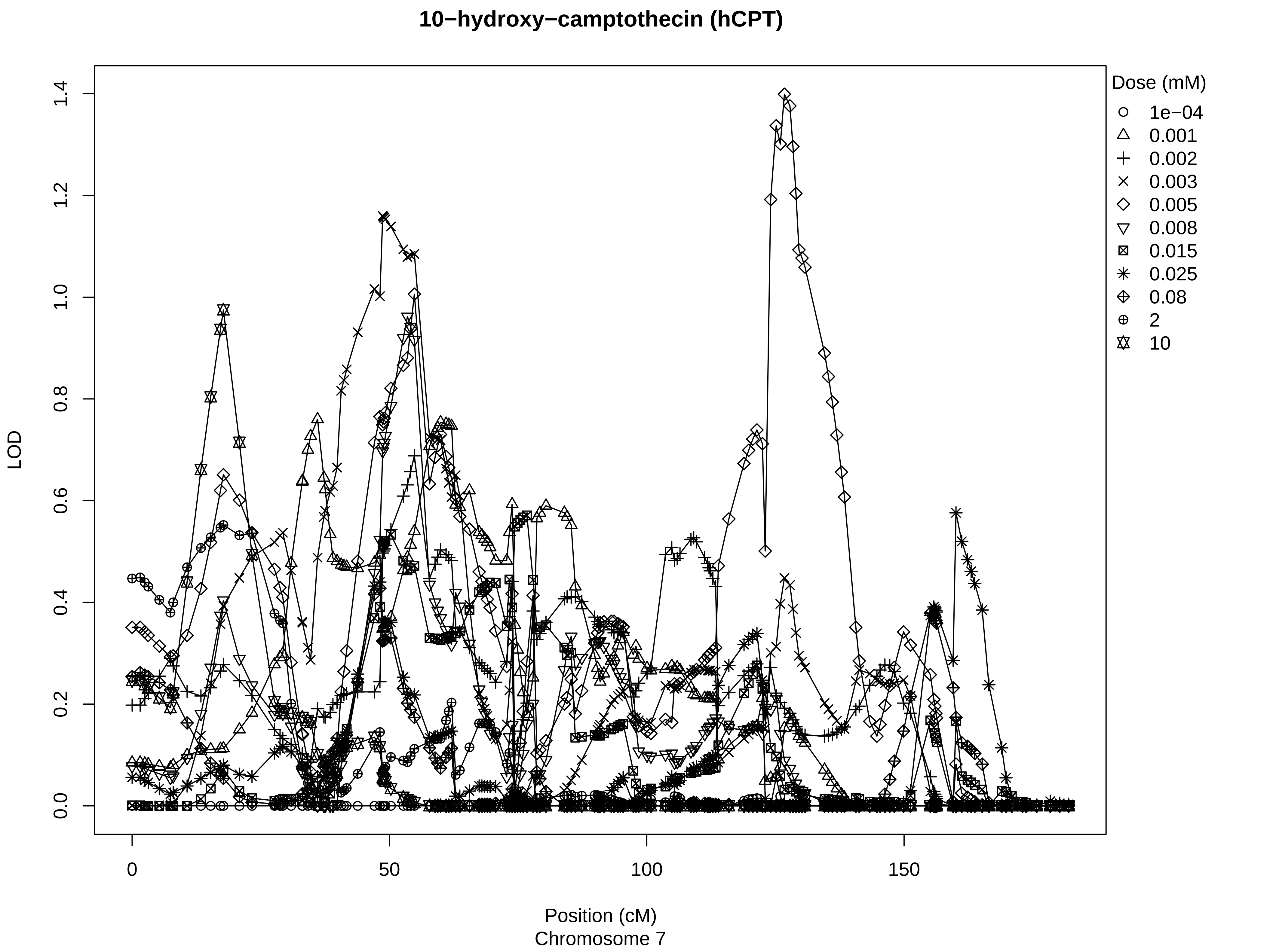

10−hydroxy−camptothecin (hCPT)
LOD
0.0
0.2
0.4
0.6
0.8
1.0
1.2
1.4
0
50
100
150
Position (cM)
Chromosome 7
Dose (mM)
1e−04
0.001
0.002
0.003
0.005
0.008
0.015
0.025
0.08
2
10

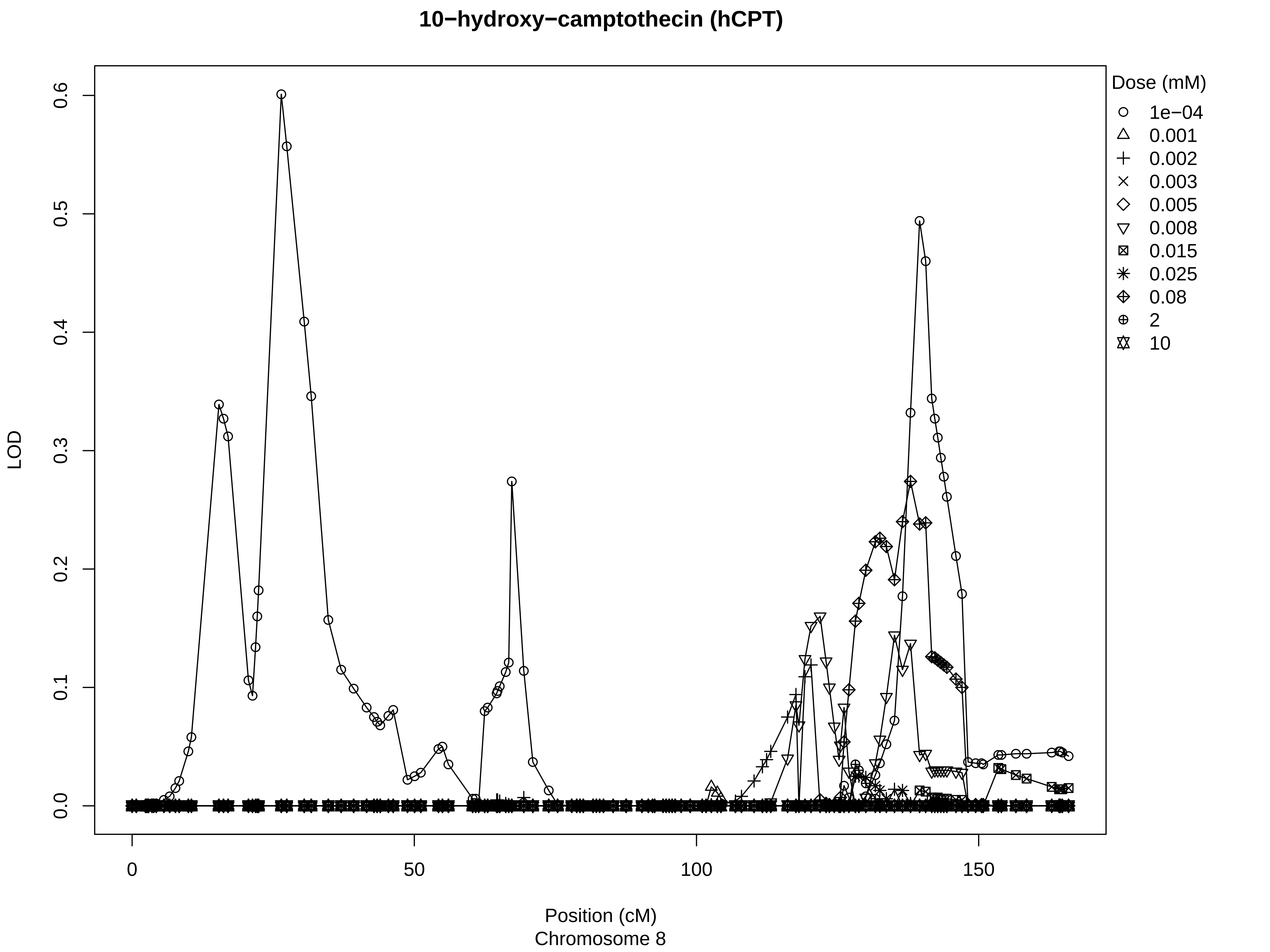

10−hydroxy−camptothecin (hCPT)
LOD
0.0
0.1
0.2
0.3
0.4
0.5
0.6
0
50
100
150
Position (cM)
Chromosome 8
Dose (mM)
1e−04
0.001
0.002
0.003
0.005
0.008
0.015
0.025
0.08
2
10

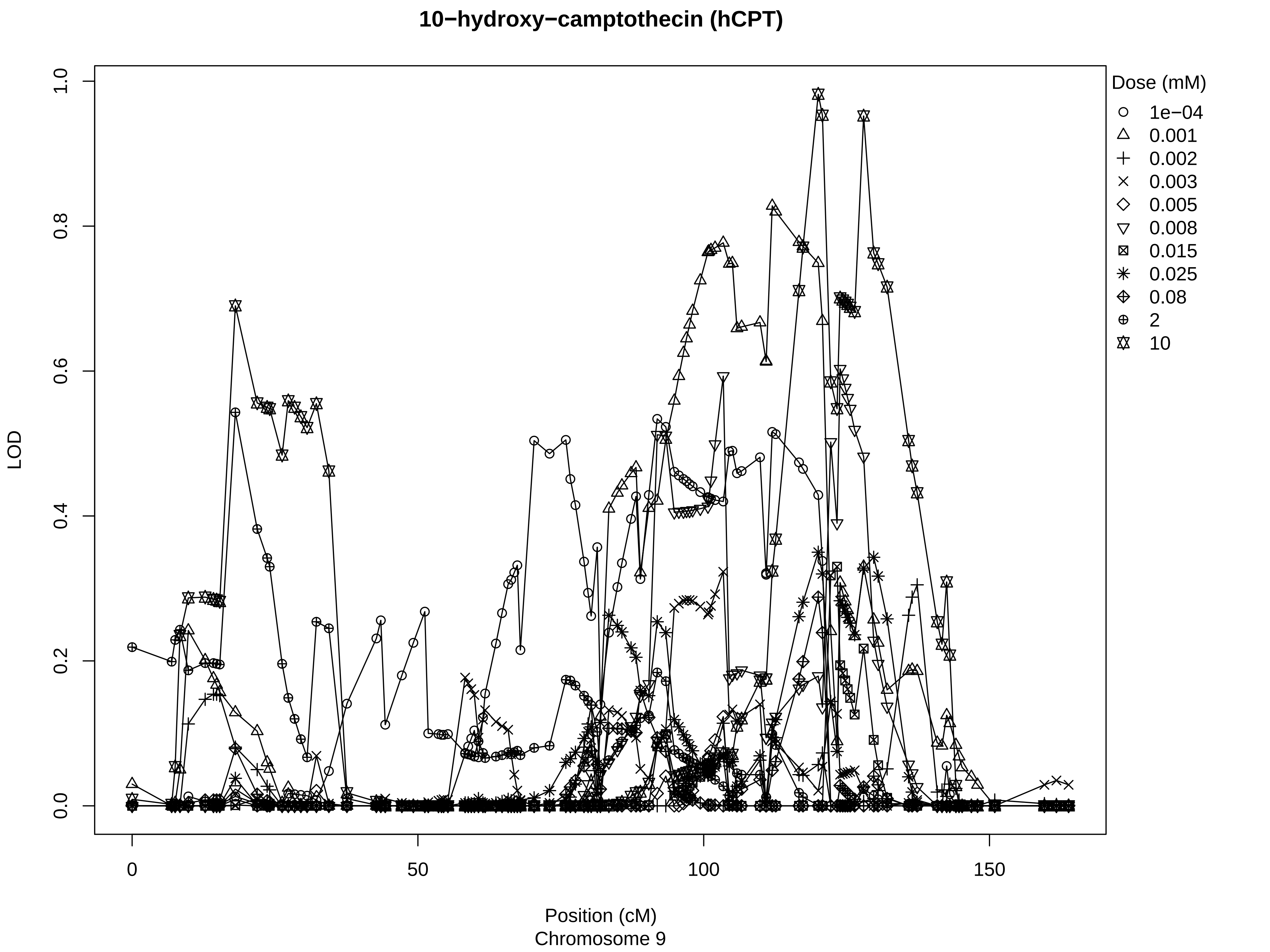
10−hydroxy−camptothecin (hCPT)
LOD
0.0
0.2
0.4
0.6
0.8
1.0
0
50
100
150
Position (cM)
Chromosome 9
Dose (mM)
1e−04
0.001
0.002
0.003
0.005
0.008
0.015
0.025
0.08
2
10

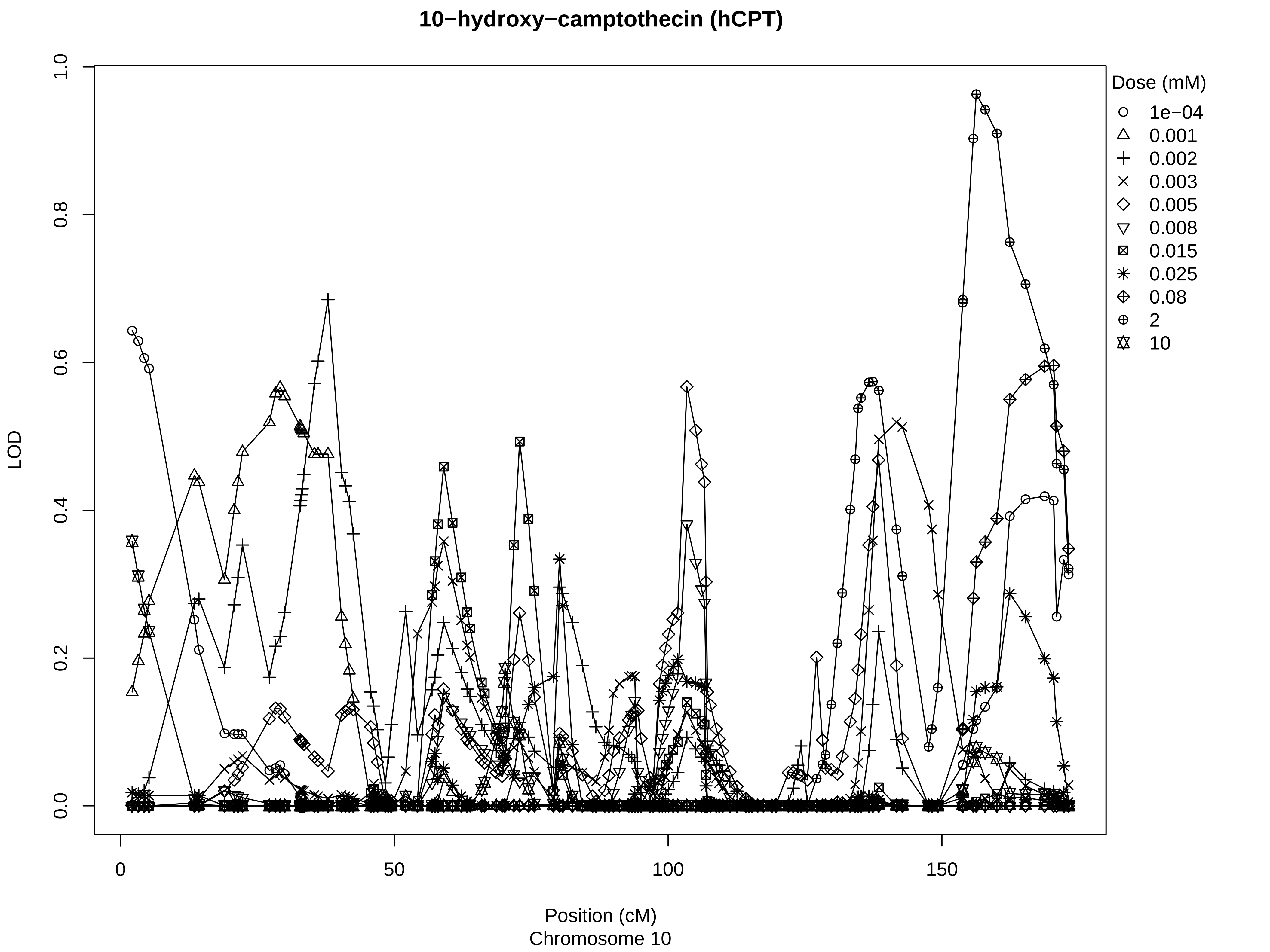

10−hydroxy−camptothecin (hCPT)
LOD
0.0
0.2
0.4
0.6
0.8
1.0
0
50
100
150
Position (cM)
Chromosome 10
Dose (mM)
1e−04
0.001
0.002
0.003
0.005
0.008
0.015
0.025
0.08
2
10

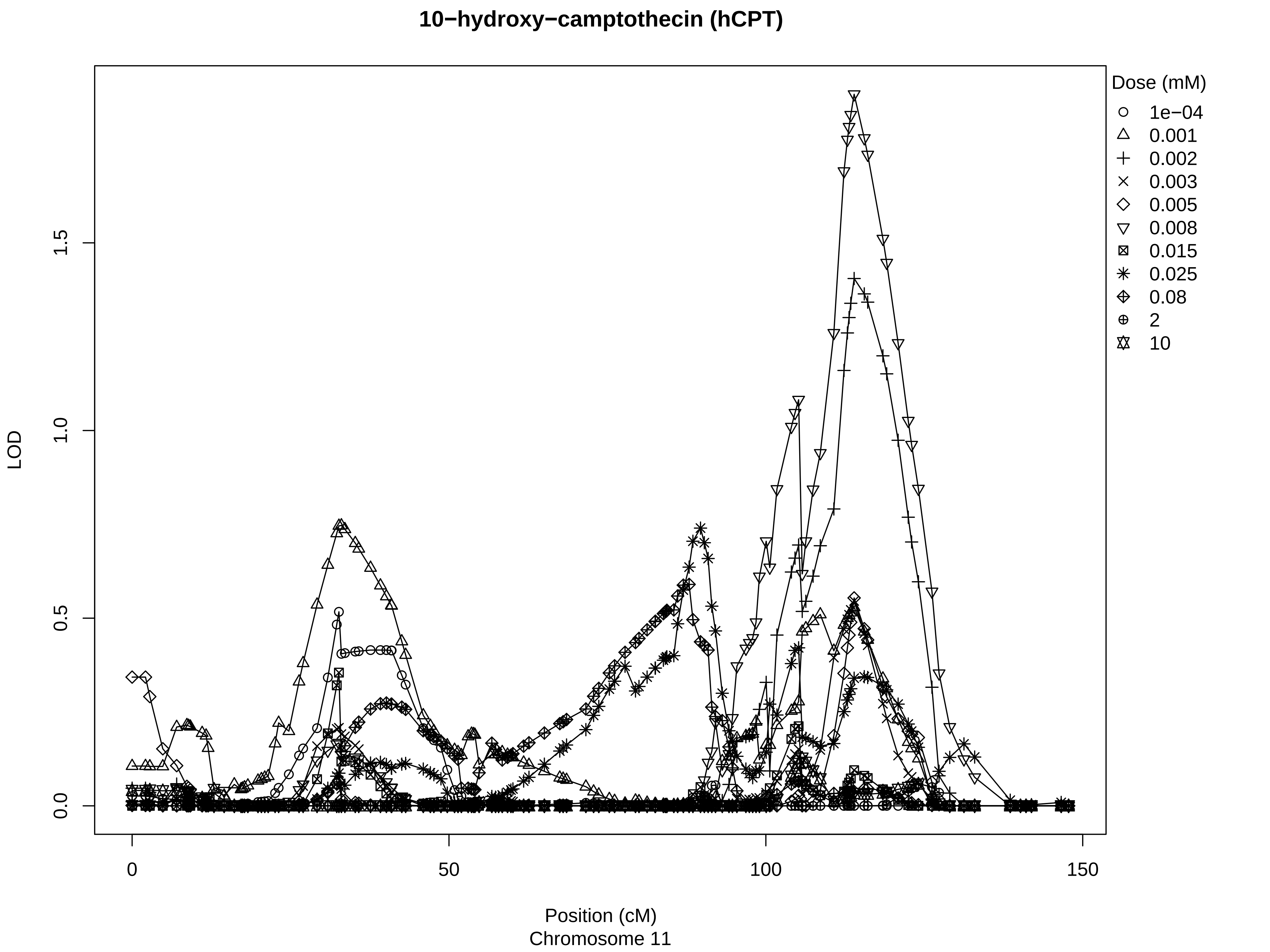

10−hydroxy−camptothecin (hCPT)
Dose (mM)
1e−04
0.001
0.002
0.003
0.005
0.008
0.015
0.025
0.08
2
10
LOD
0.0
0.5
1.0
1.5
0
50
100
150
Position (cM)
Chromosome 11

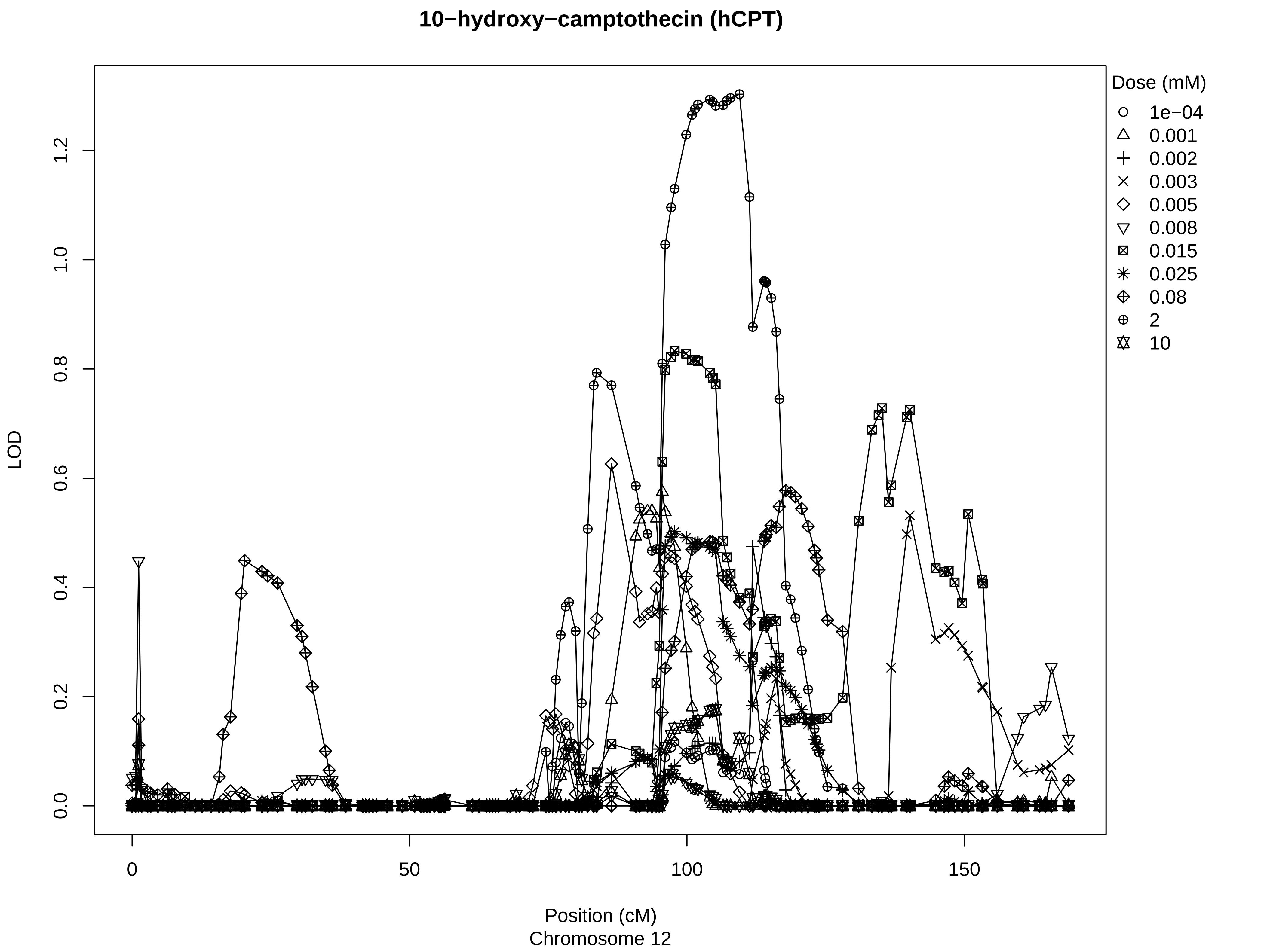

10−hydroxy−camptothecin (hCPT)
LOD
0.0
0.2
0.4
0.6
0.8
1.0
1.2
0
50
100
150
Position (cM)
Chromosome 12
Dose (mM)
1e−04
0.001
0.002
0.003
0.005
0.008
0.015
0.025
0.08
2
10

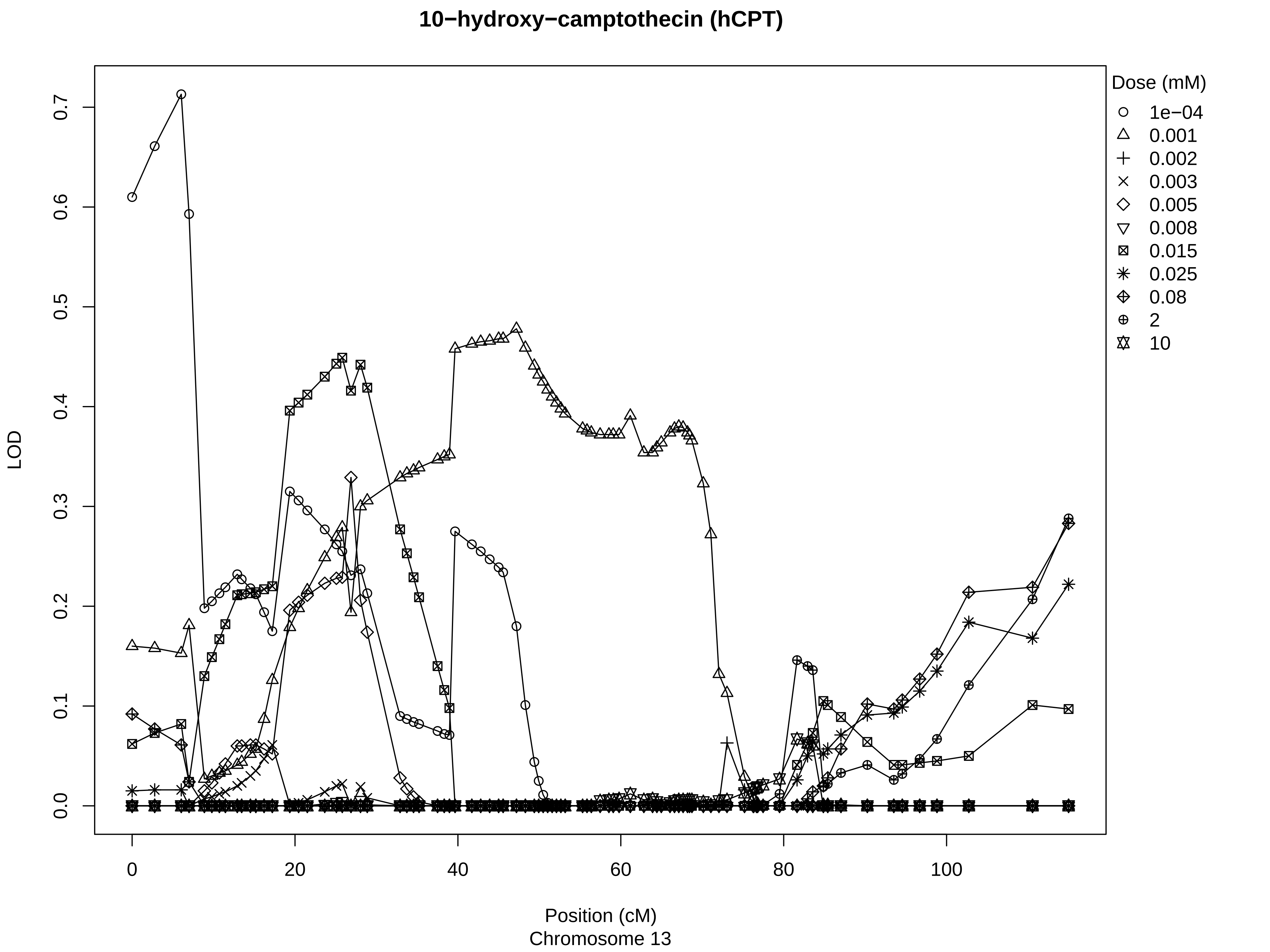
10−hydroxy−camptothecin (hCPT)
LOD
0.0
0.1
0.2
0.3
0.4
0.5
0.6
0.7
0
20
40
60
80
100
Position (cM)
Chromosome 13
Dose (mM)
1e−04
0.001
0.002
0.003
0.005
0.008
0.015
0.025
0.08
2
10

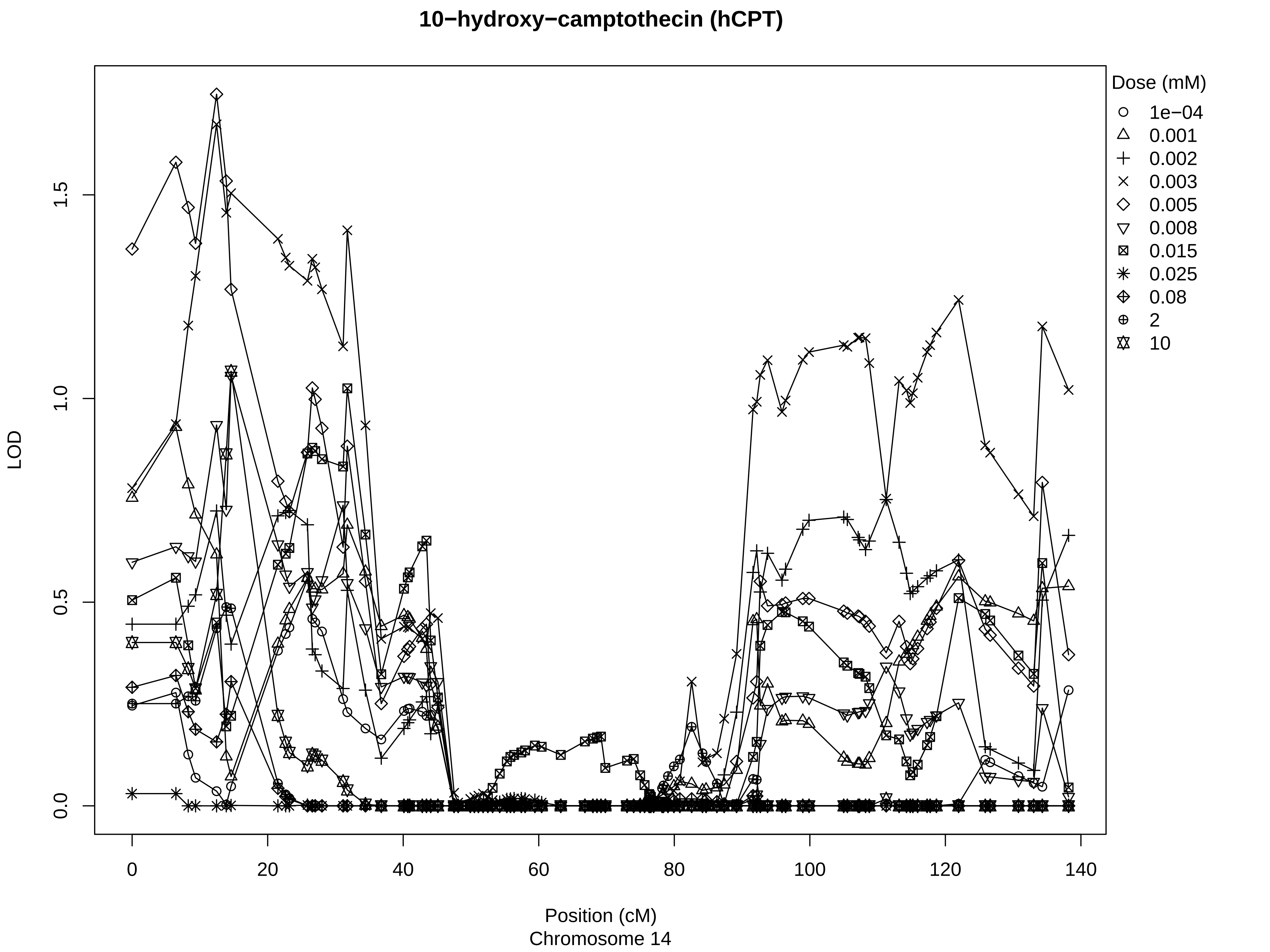
10−hydroxy−camptothecin (hCPT)
LOD
Position (cM)
Chromosome 14
Dose (mM)
1e−04
0.001
0.002
0.003
0.005
0.008
0.015
0.025
0.08
2
10
0.0
0.5
1.0
1.5
0
20
40
60
80
100
120
140

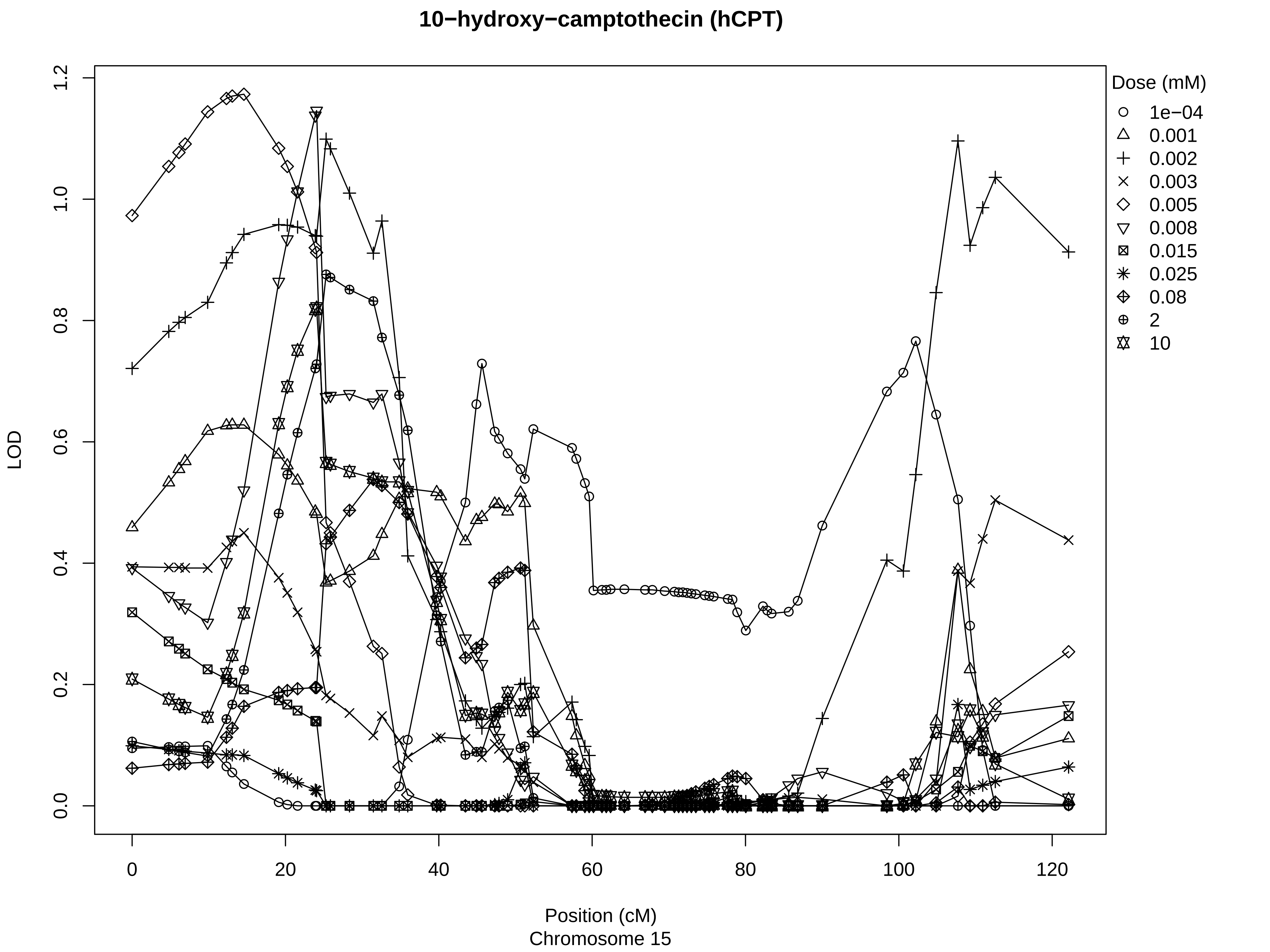

10−hydroxy−camptothecin (hCPT)
LOD
0.0
0.2
0.4
0.6
0.8
1.0
1.2
0
20
40
60
80
100
120
Position (cM)
Chromosome 15
Dose (mM)
1e−04
0.001
0.002
0.003
0.005
0.008
0.015
0.025
0.08
2
10

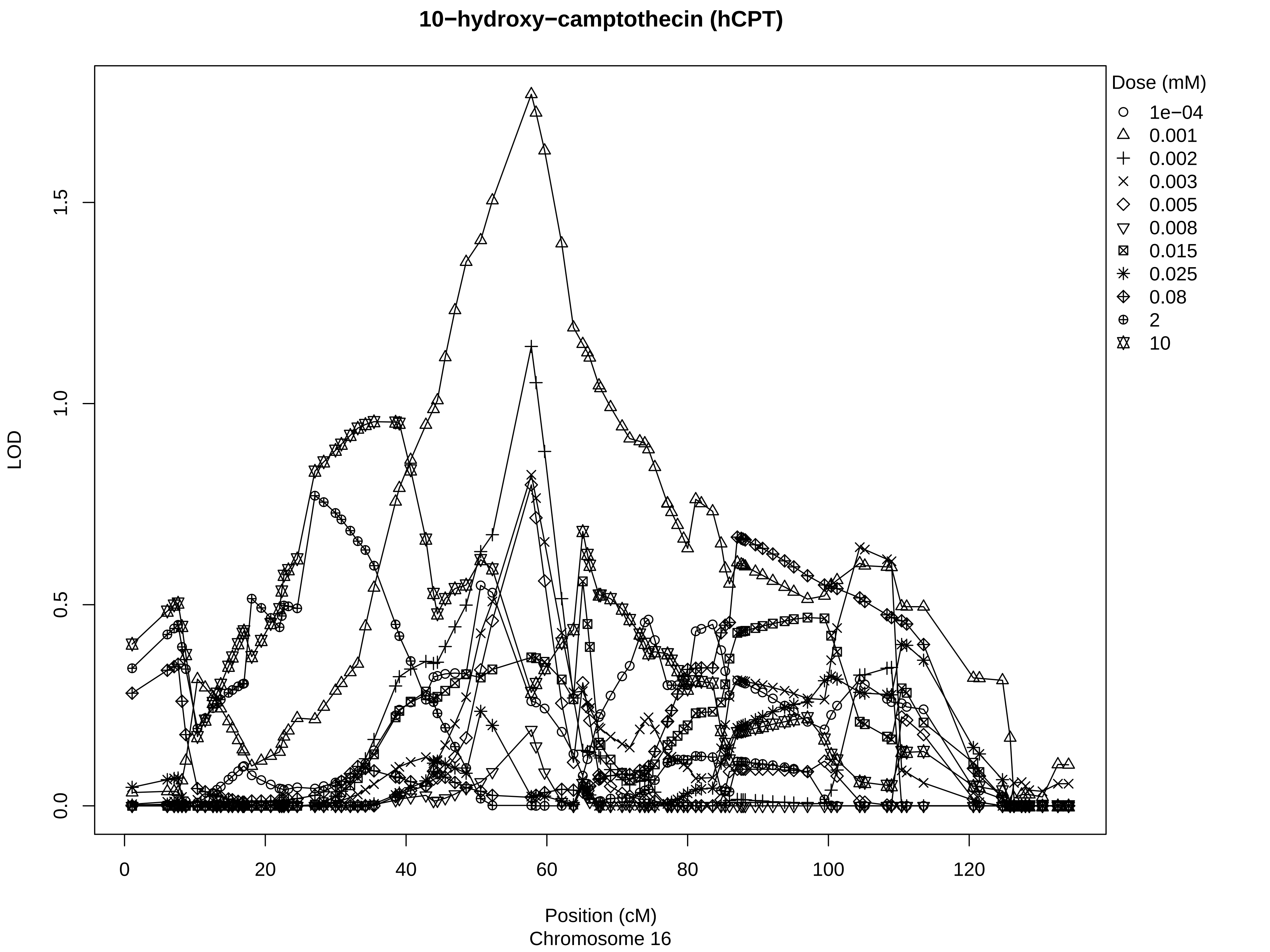

10−hydroxy−camptothecin (hCPT)
Dose (mM)
1e−04
0.001
0.002
0.003
0.005
0.008
0.015
0.025
0.08
2
10
LOD
0.0
0.5
1.0
1.5
0
20
40
60
80
100
120
Position (cM)
Chromosome 16

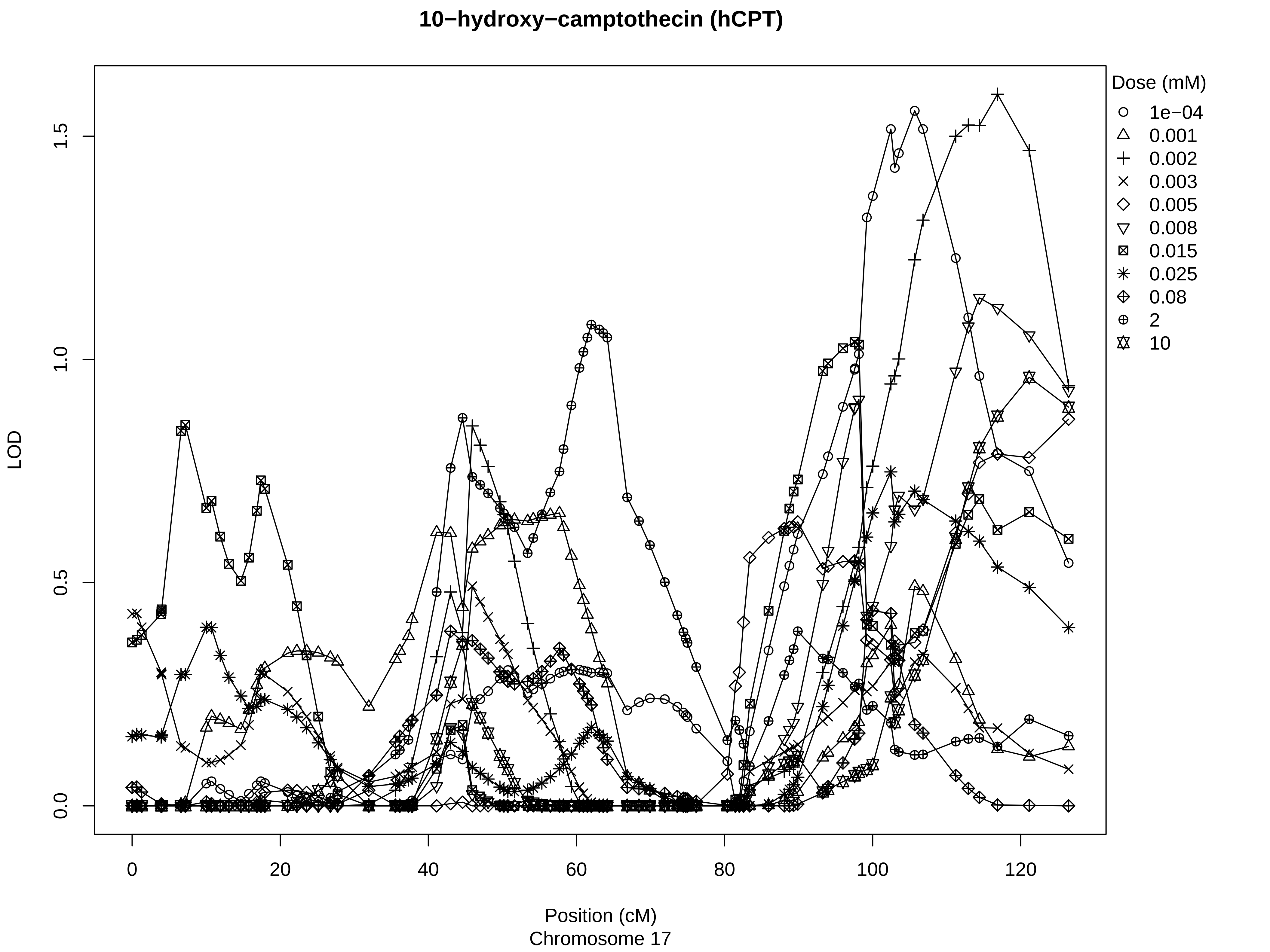

10−hydroxy−camptothecin (hCPT)
Dose (mM)
1e−04
0.001
0.002
0.003
0.005
0.008
0.015
0.025
0.08
2
10
LOD
0.0
0.5
1.0
1.5
0
20
40
60
80
100
120
Position (cM)
Chromosome 17

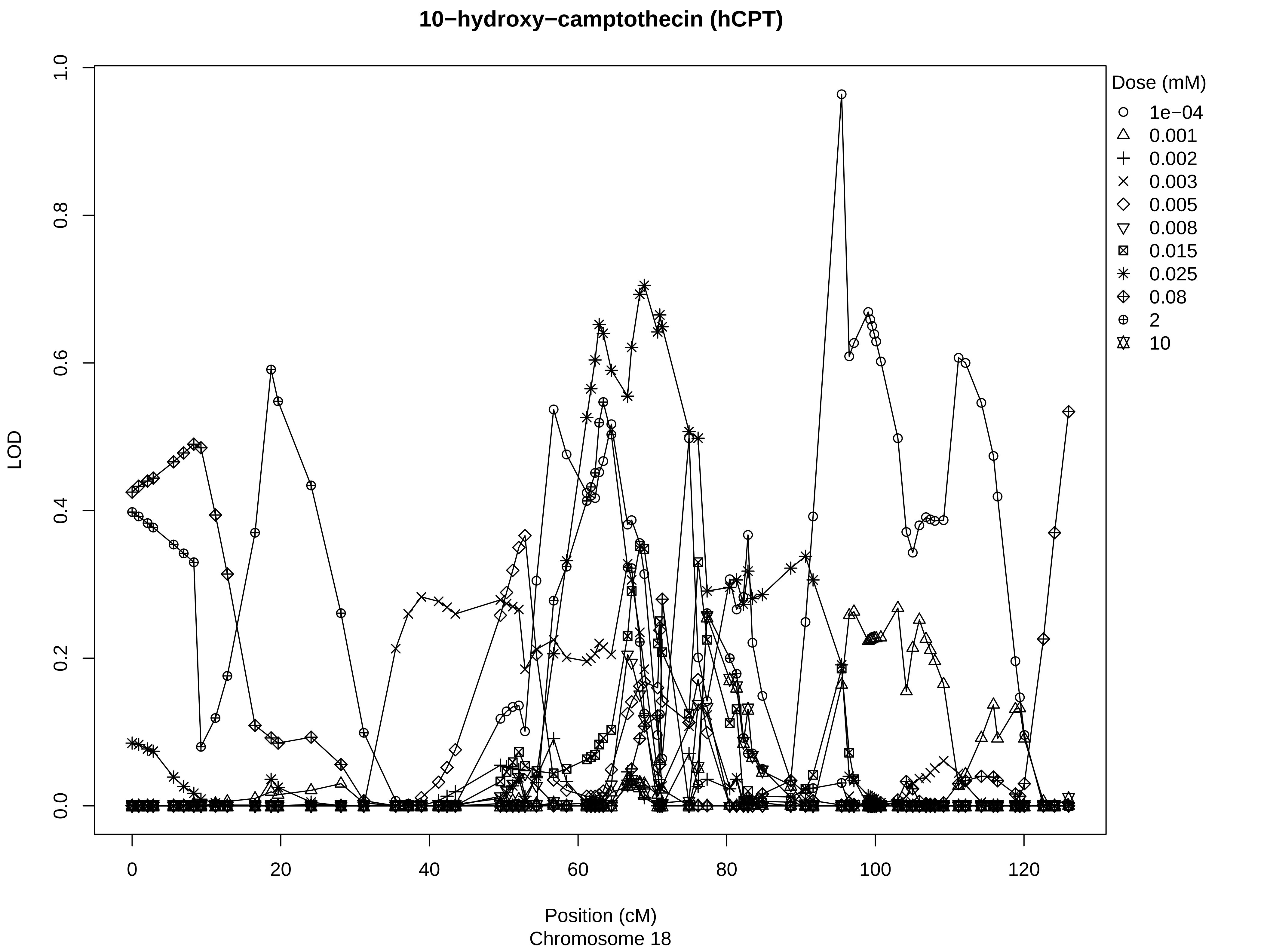

10−hydroxy−camptothecin (hCPT)
LOD
0.0
0.2
0.4
0.6
0.8
1.0
0
20
40
60
80
100
120
Position (cM)
Chromosome 18
Dose (mM)
1e−04
0.001
0.002
0.003
0.005
0.008
0.015
0.025
0.08
2
10

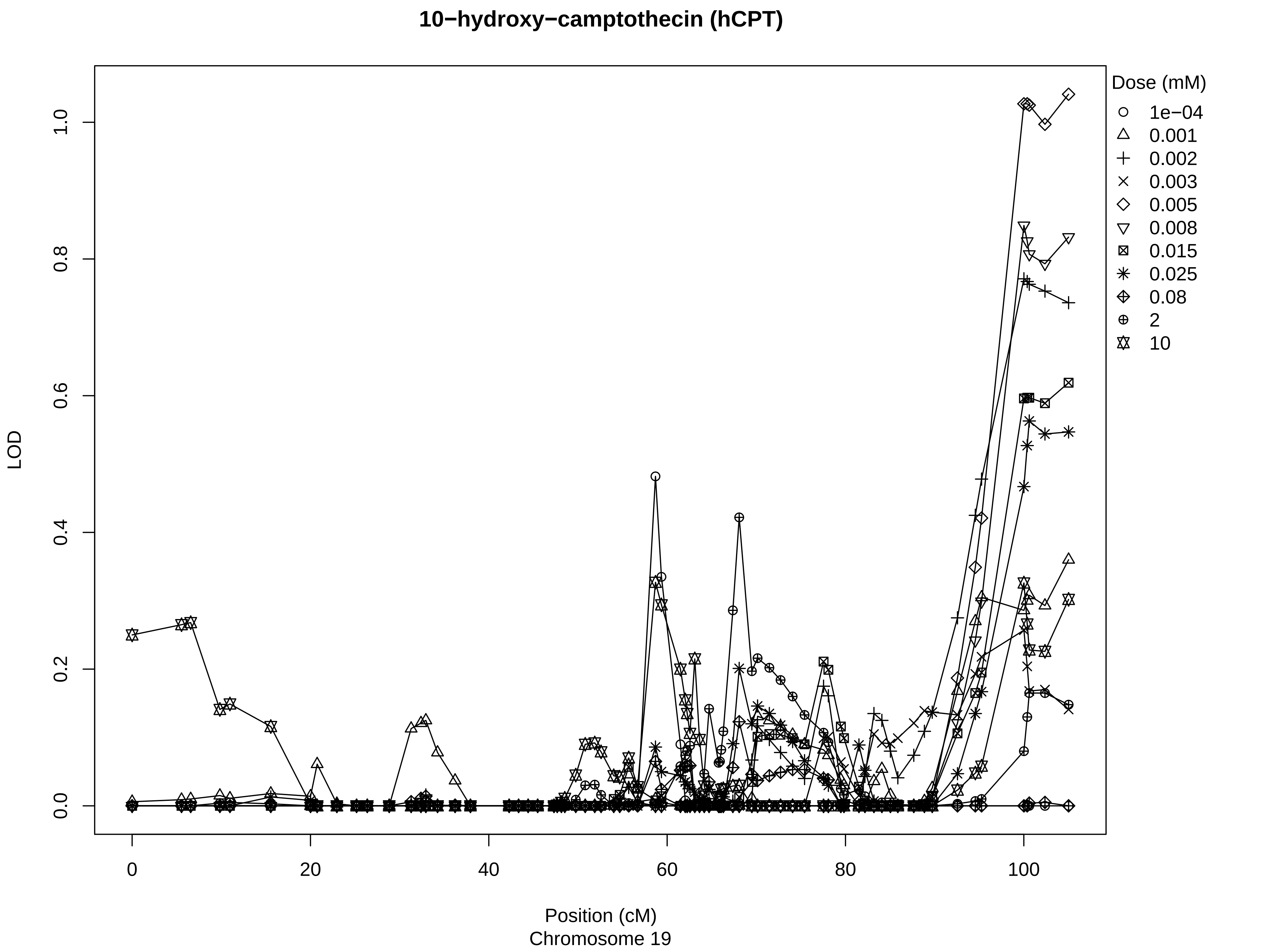

10−hydroxy−camptothecin (hCPT)
LOD
0.0
0.2
0.4
0.6
0.8
1.0
0
20
40
60
80
100
Position (cM)
Chromosome 19
Dose (mM)
1e−04
0.001
0.002
0.003
0.005
0.008
0.015
0.025
0.08
2
10

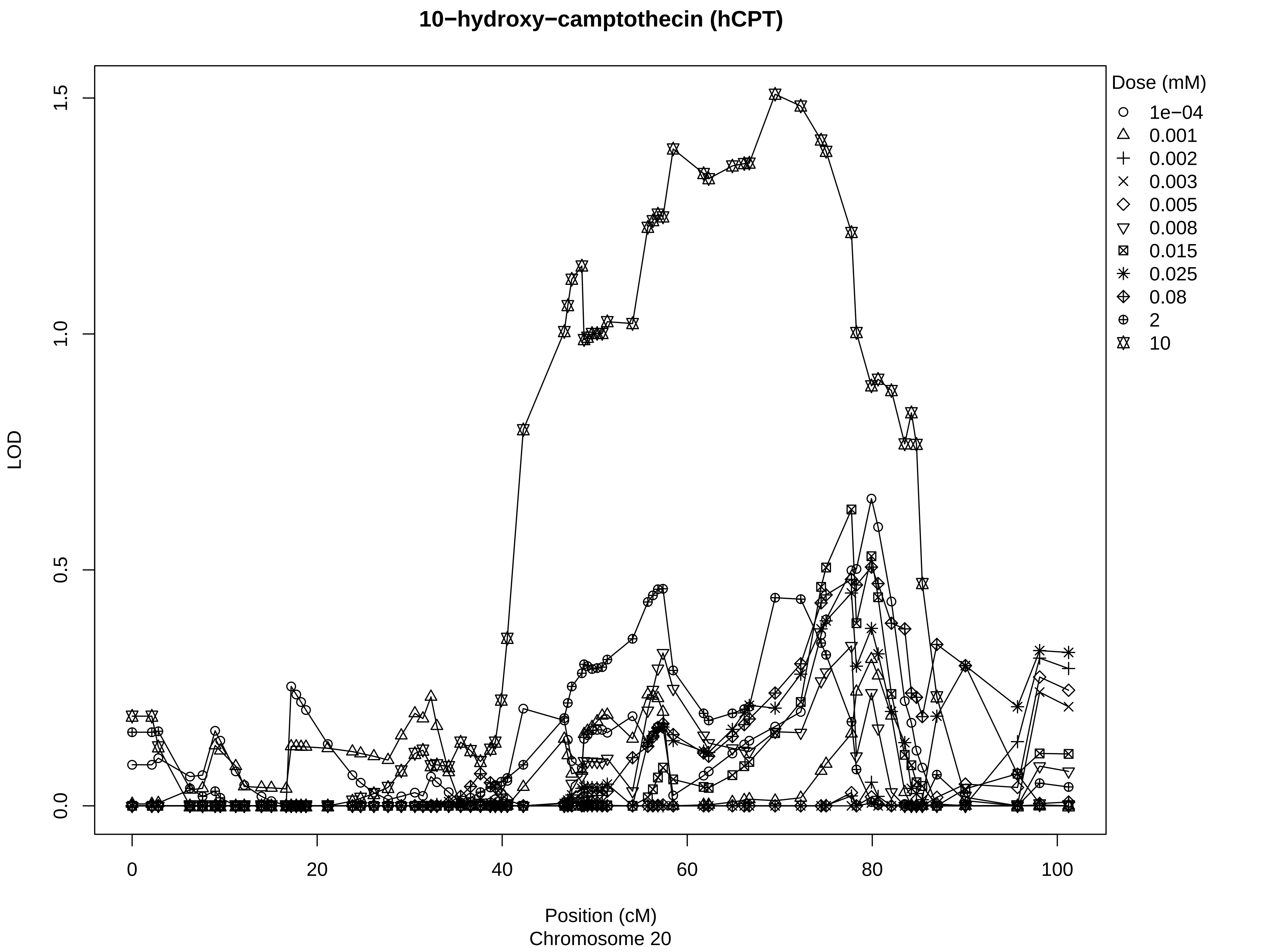

10−hydroxy−camptothecin (hCPT)
LOD
0.0
0.5
1.0
1.5
0
20
40
60
80
100
Position (cM)
Chromosome 20
Dose (mM)
1e−04
0.001
0.002
0.003
0.005
0.008
0.015
0.025
0.08
2
10

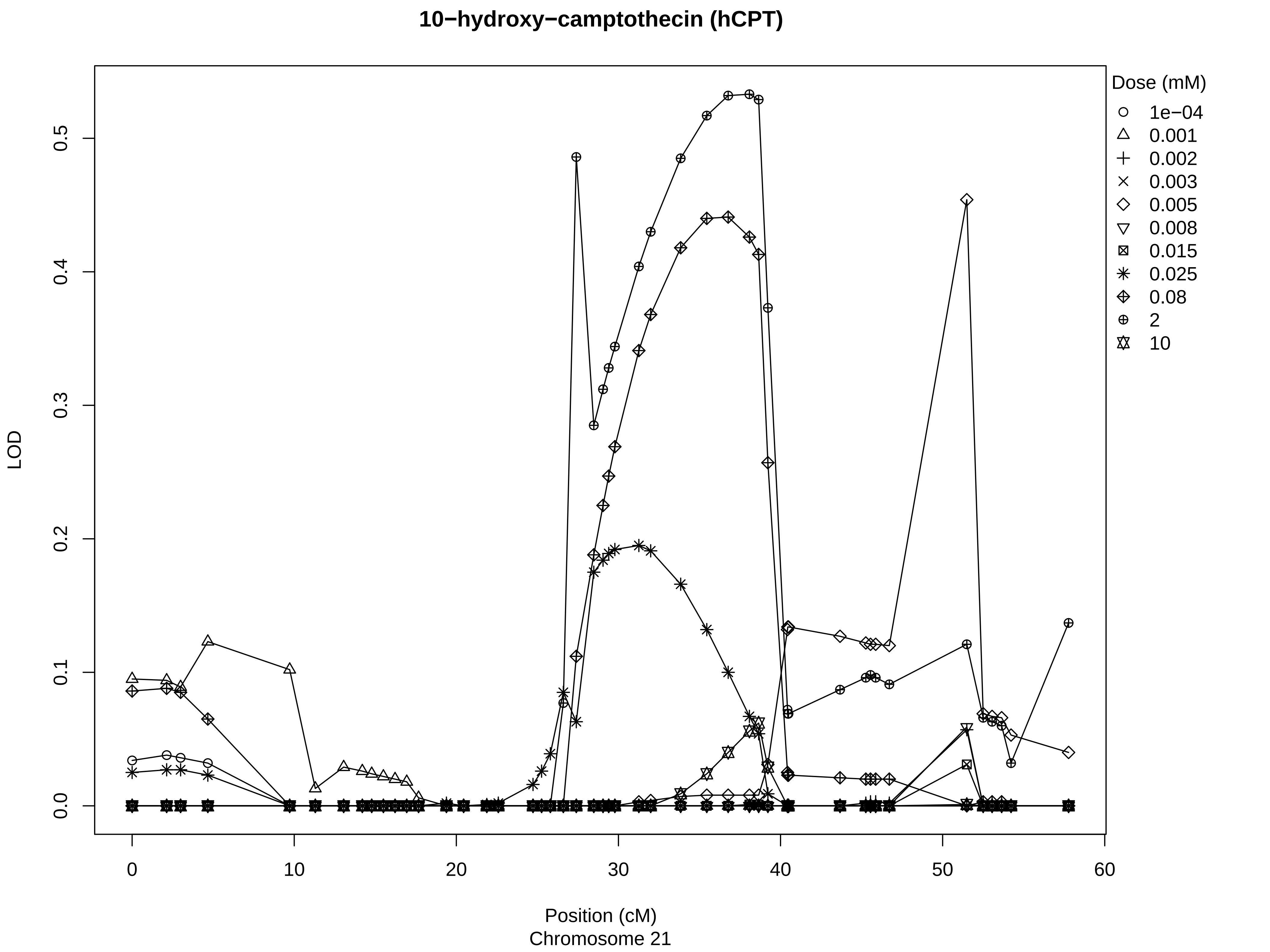

10−hydroxy−camptothecin (hCPT)
Dose (mM)
1e−04
0.001
0.002
0.003
0.005
0.008
0.015
0.025
0.08
2
10
LOD
0.0
0.1
0.2
0.3
0.4
0.5
0
10
20
30
40
50
60
Position (cM)
Chromosome 21

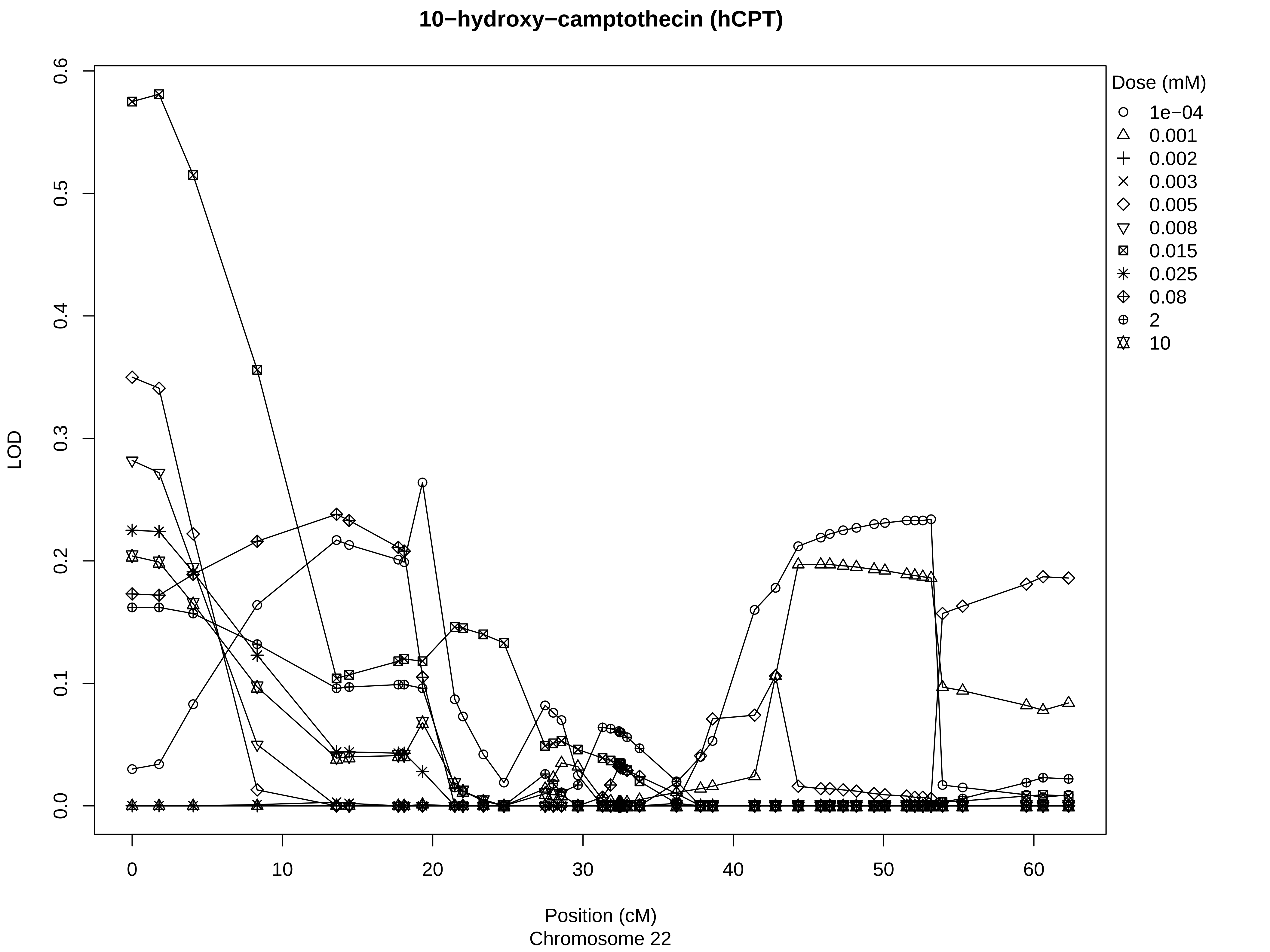
10−hydroxy−camptothecin (hCPT)
LOD
0.0
0.1
0.2
0.3
0.4
0.5
0.6
0
10
20
30
40
50
60
Position (cM)
Chromosome 22
Dose (mM)
1e−04
0.001
0.002
0.003
0.005
0.008
0.015
0.025
0.08
2
10

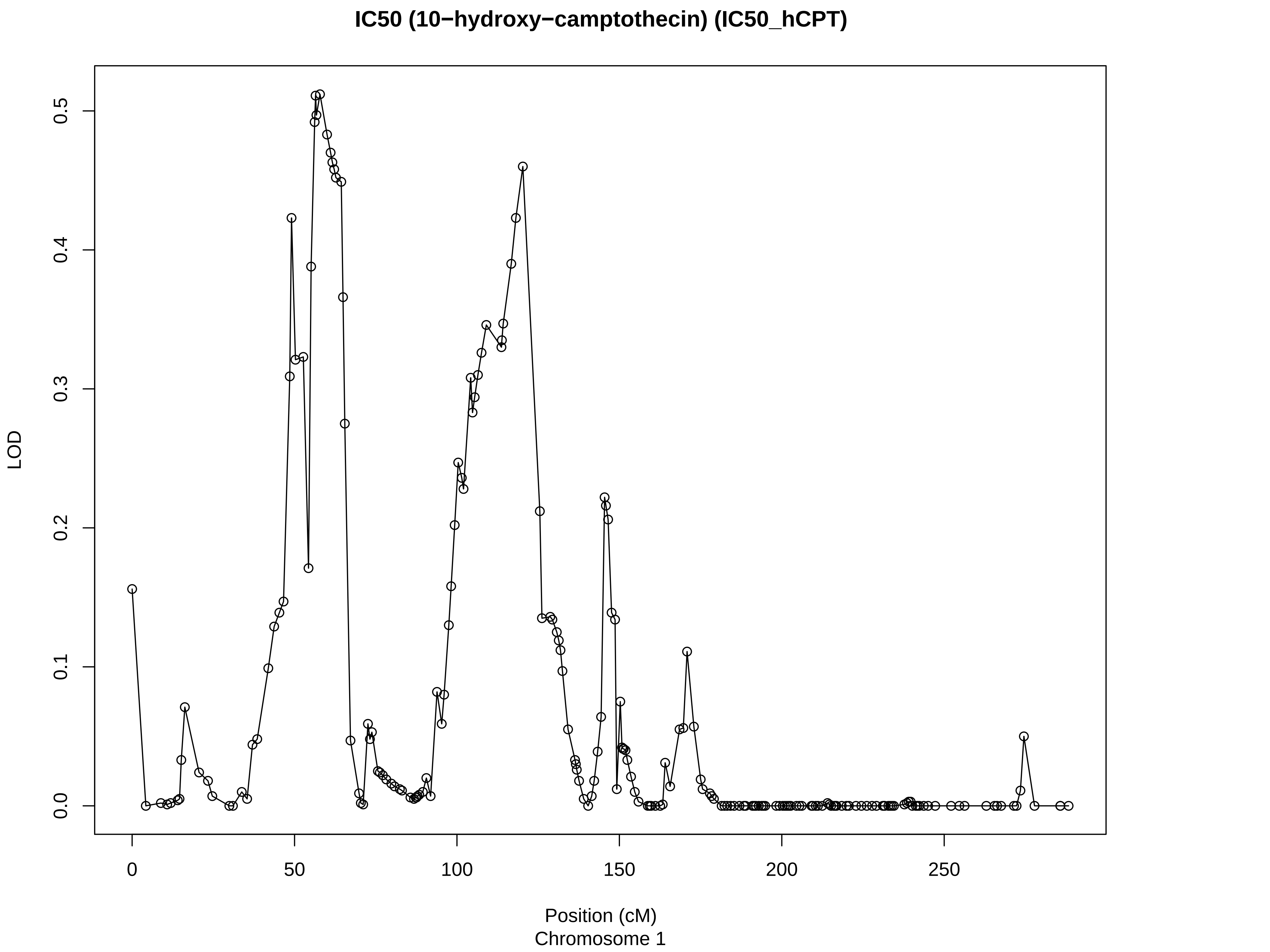
IC50 (10−hydroxy−camptothecin) (IC50_hCPT)
LOD
0.0
0.1
0.2
0.3
0.4
0.5
0
50
100
150
200
250
Position (cM)
Chromosome 1

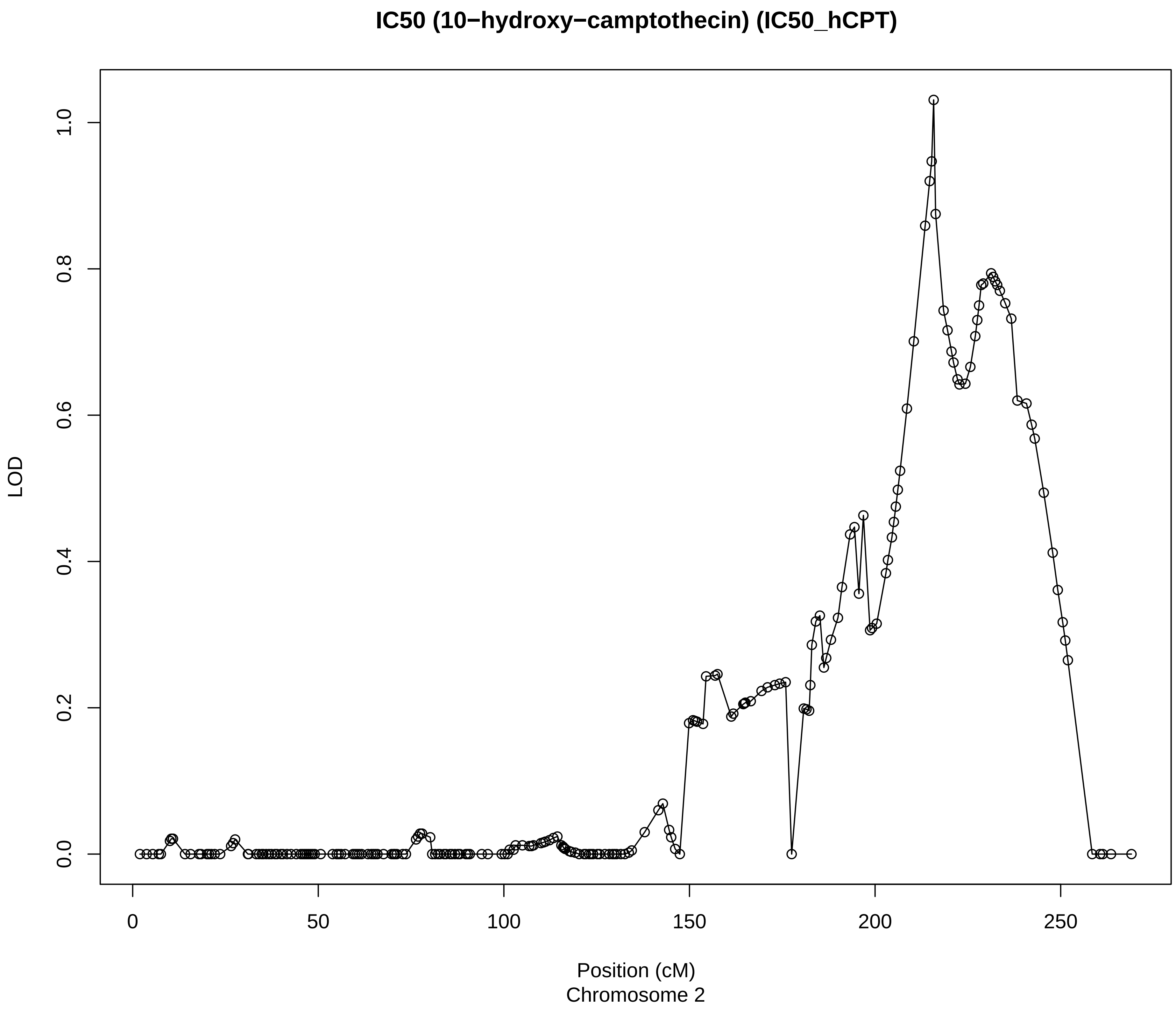

IC50 (10−hydroxy−camptothecin) (IC50_hCPT)
LOD
0.0
0.2
0.4
0.6
0.8
1.0
0
50
100
150
200
250
Position (cM)
Chromosome 2

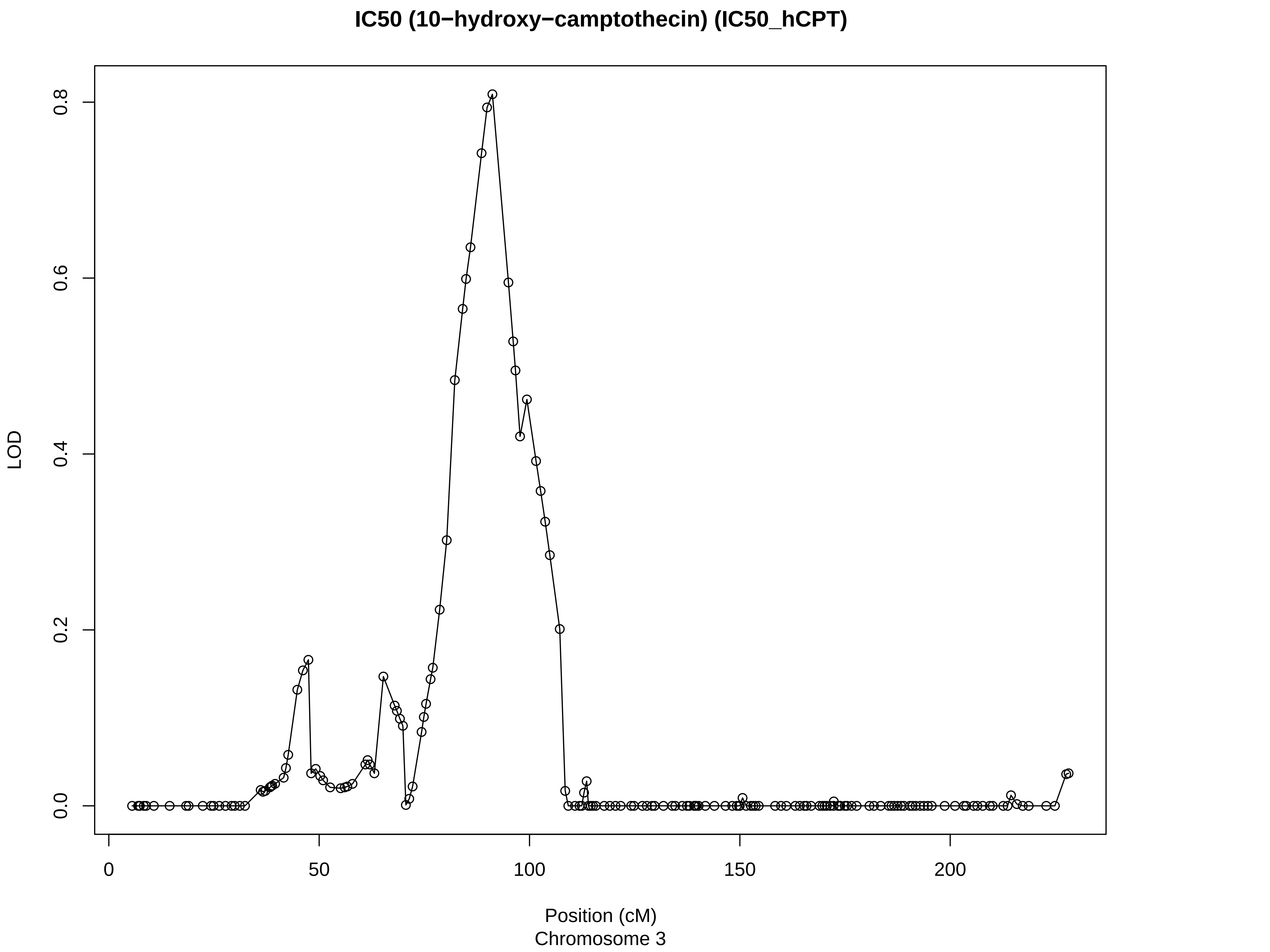
IC50 (10−hydroxy−camptothecin) (IC50_hCPT)
LOD
0.0
0.2
0.4
0.6
0.8
0
50
100
150
200
Position (cM)
Chromosome 3

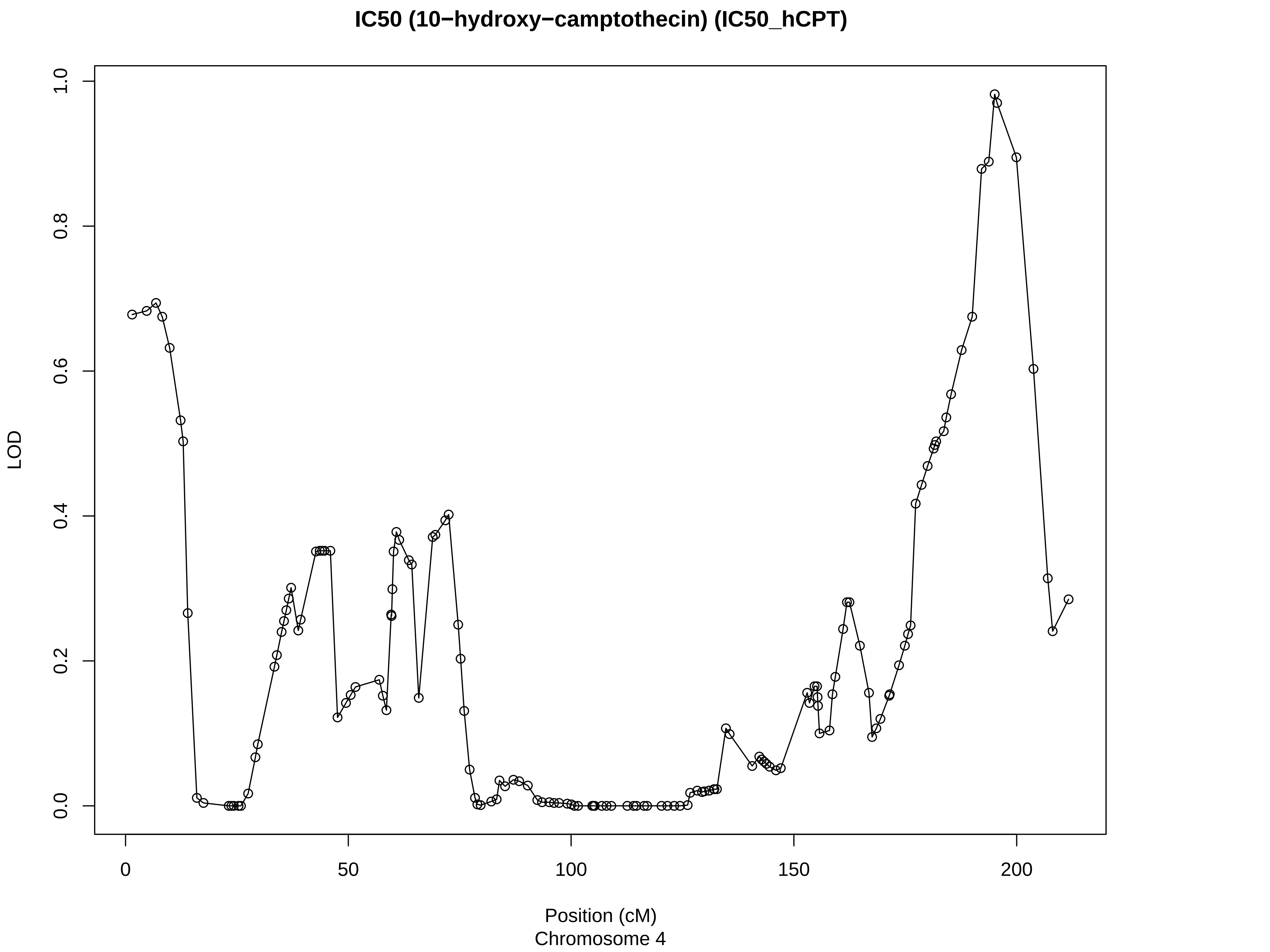
IC50 (10−hydroxy−camptothecin) (IC50_hCPT)
LOD
0.0
0.2
0.4
0.6
0.8
1.0
0
50
100
150
200
Position (cM)
Chromosome 4

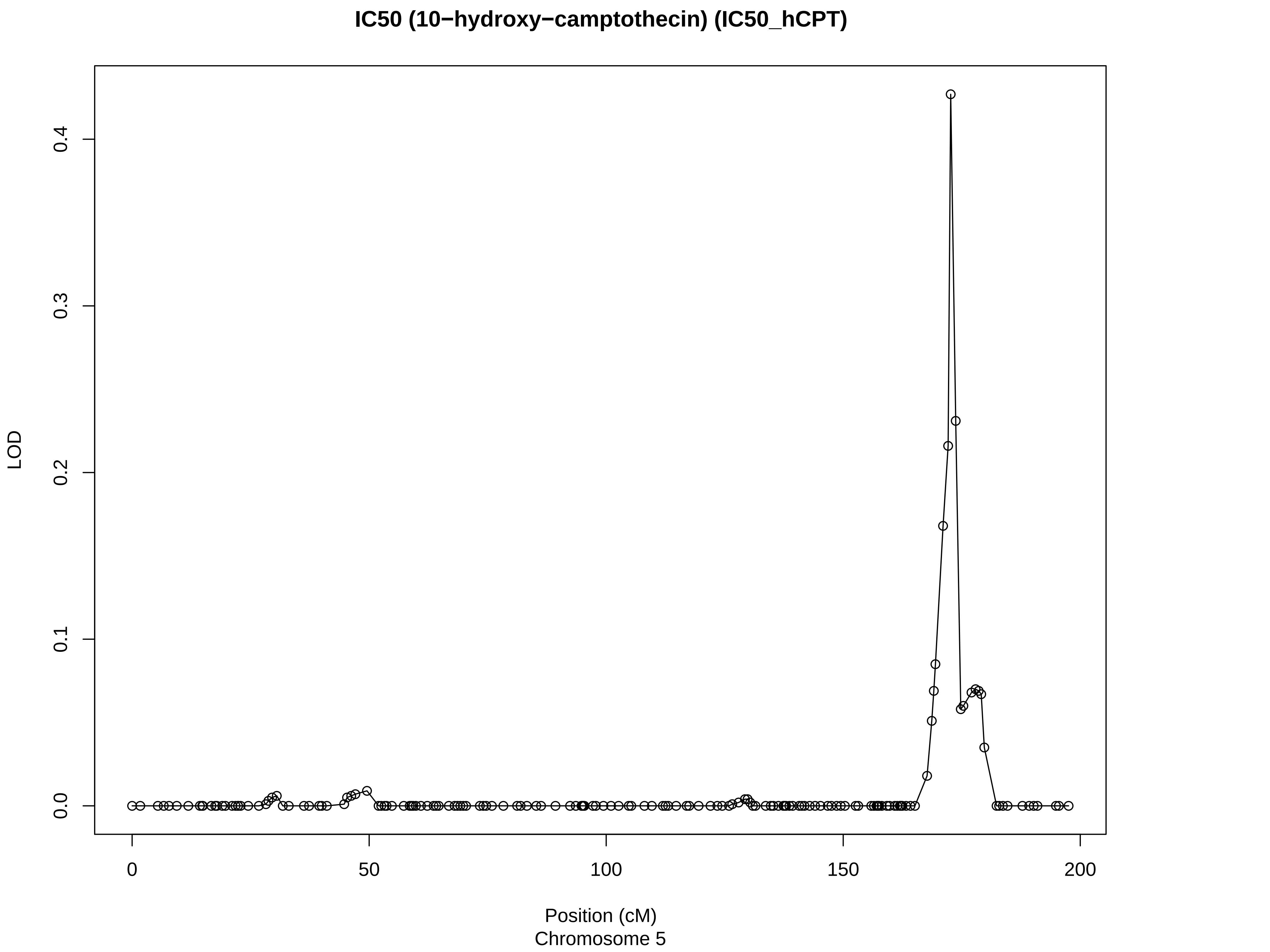
IC50 (10−hydroxy−camptothecin) (IC50_hCPT)
LOD
0.0
0.1
0.2
0.3
0.4
0
50
100
150
200
Position (cM)
Chromosome 5

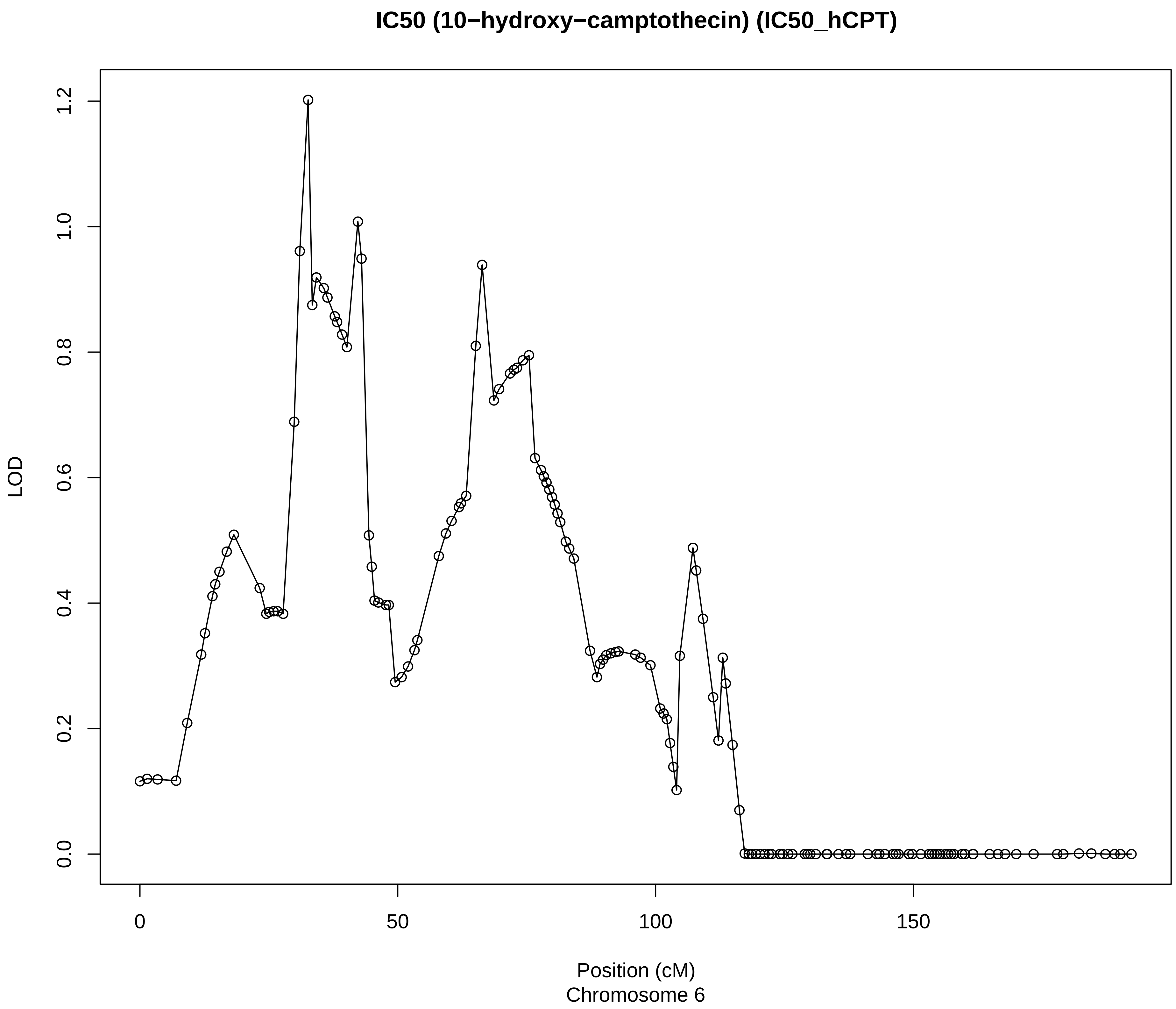
IC50 (10−hydroxy−camptothecin) (IC50_hCPT)
LOD
0.0
0.2
0.4
0.6
0.8
1.0
1.2
0
50
100
150
Position (cM)
Chromosome 6

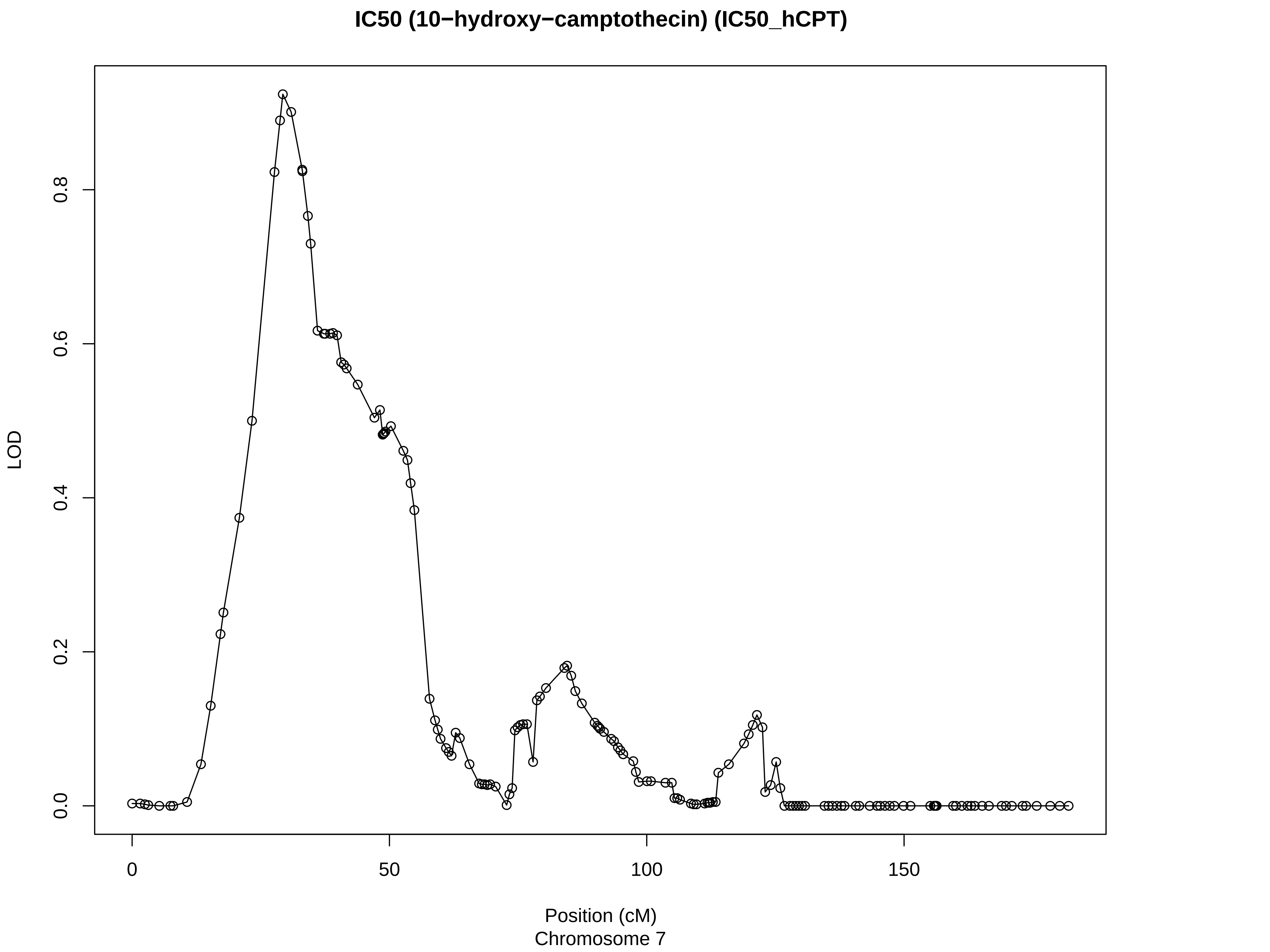
IC50 (10−hydroxy−camptothecin) (IC50_hCPT)
LOD
0.0
0.2
0.4
0.6
0.8
0
50
100
150
Position (cM)
Chromosome 7

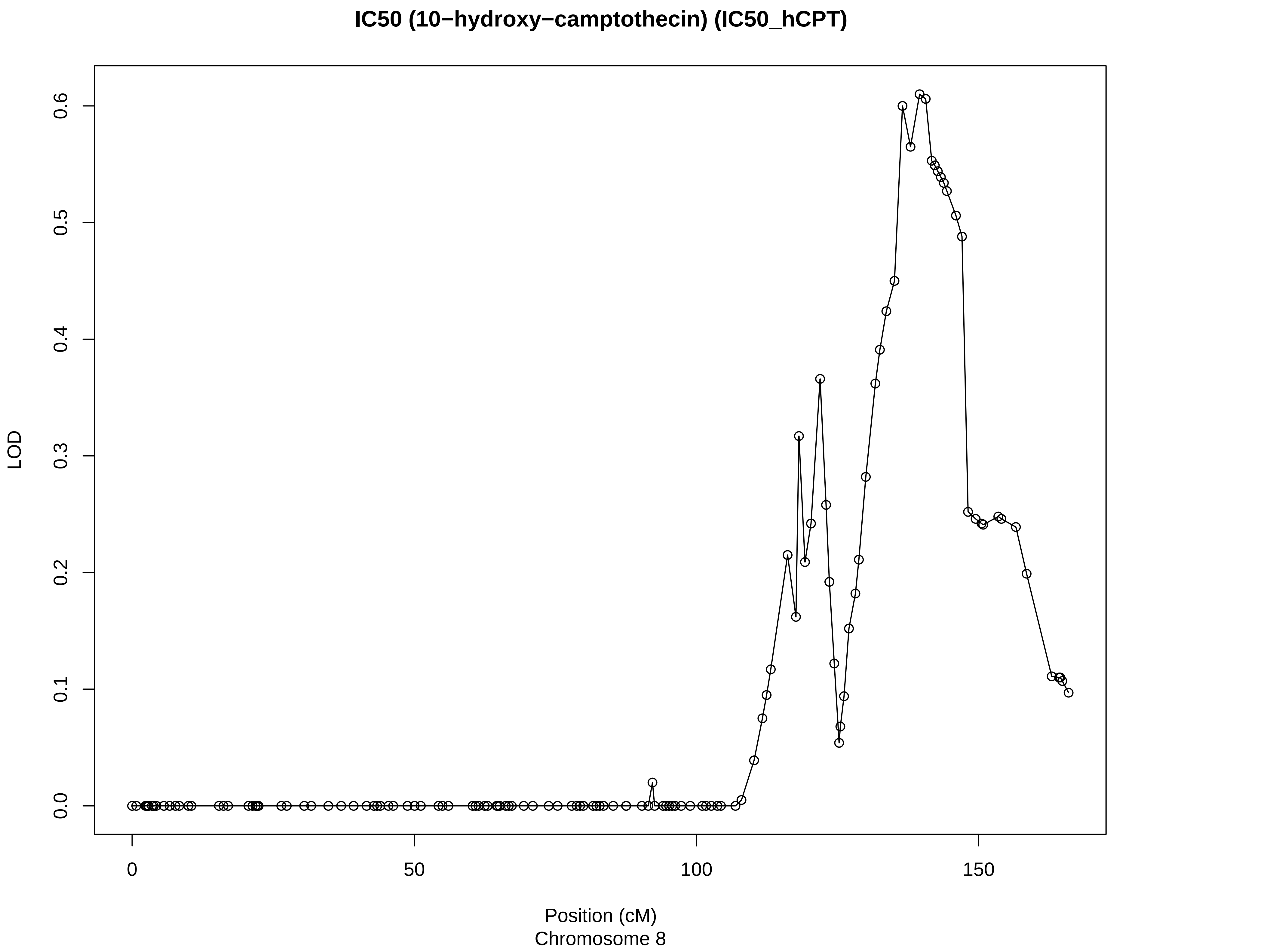
IC50 (10−hydroxy−camptothecin) (IC50_hCPT)
LOD
0.0
0.1
0.2
0.3
0.4
0.5
0.6
0
50
100
150
Position (cM)
Chromosome 8

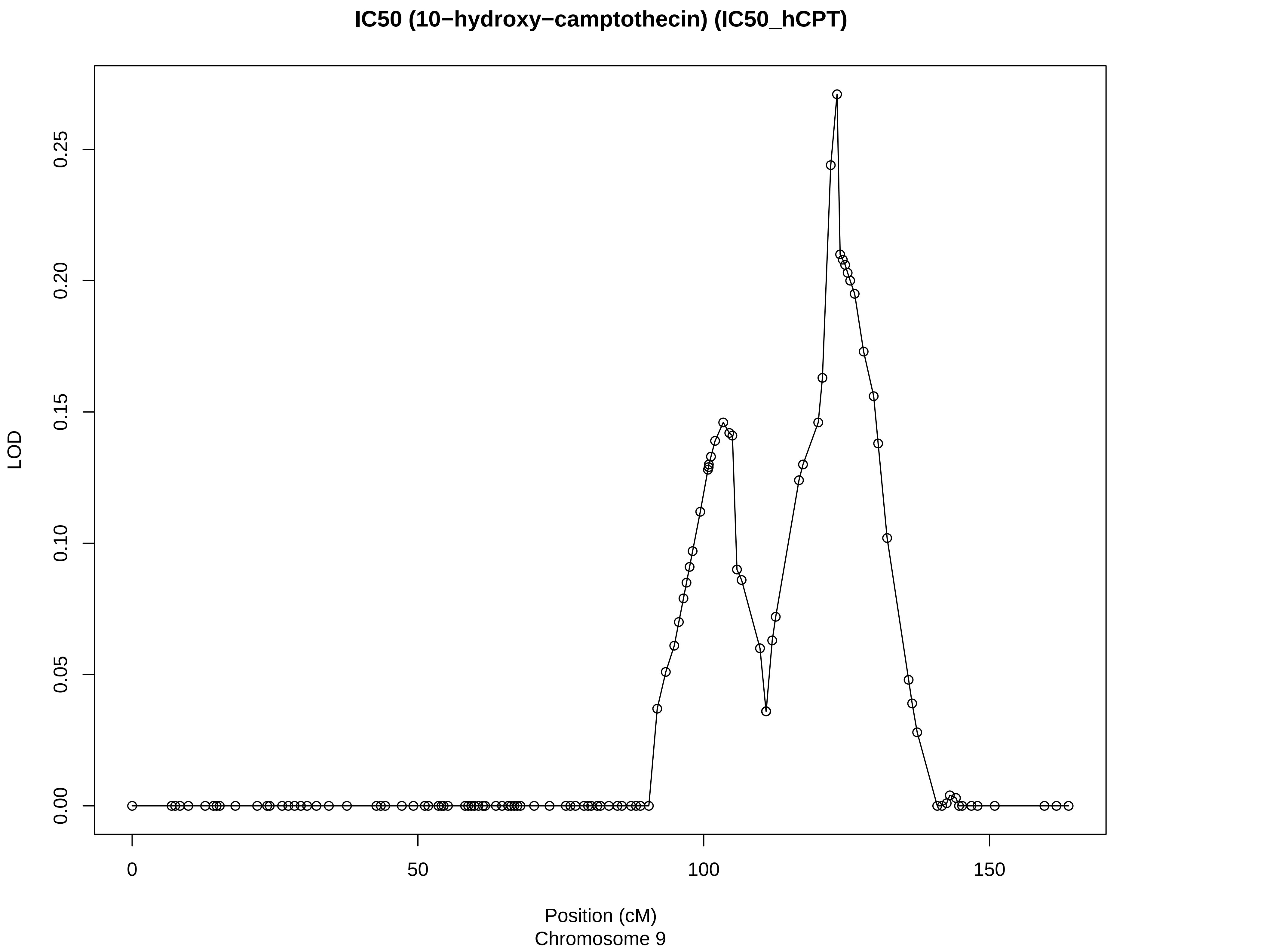
IC50 (10−hydroxy−camptothecin) (IC50_hCPT)
LOD
0.00
0.05
0.10
0.15
0.20
0.25
0
50
100
150
Position (cM)
Chromosome 9

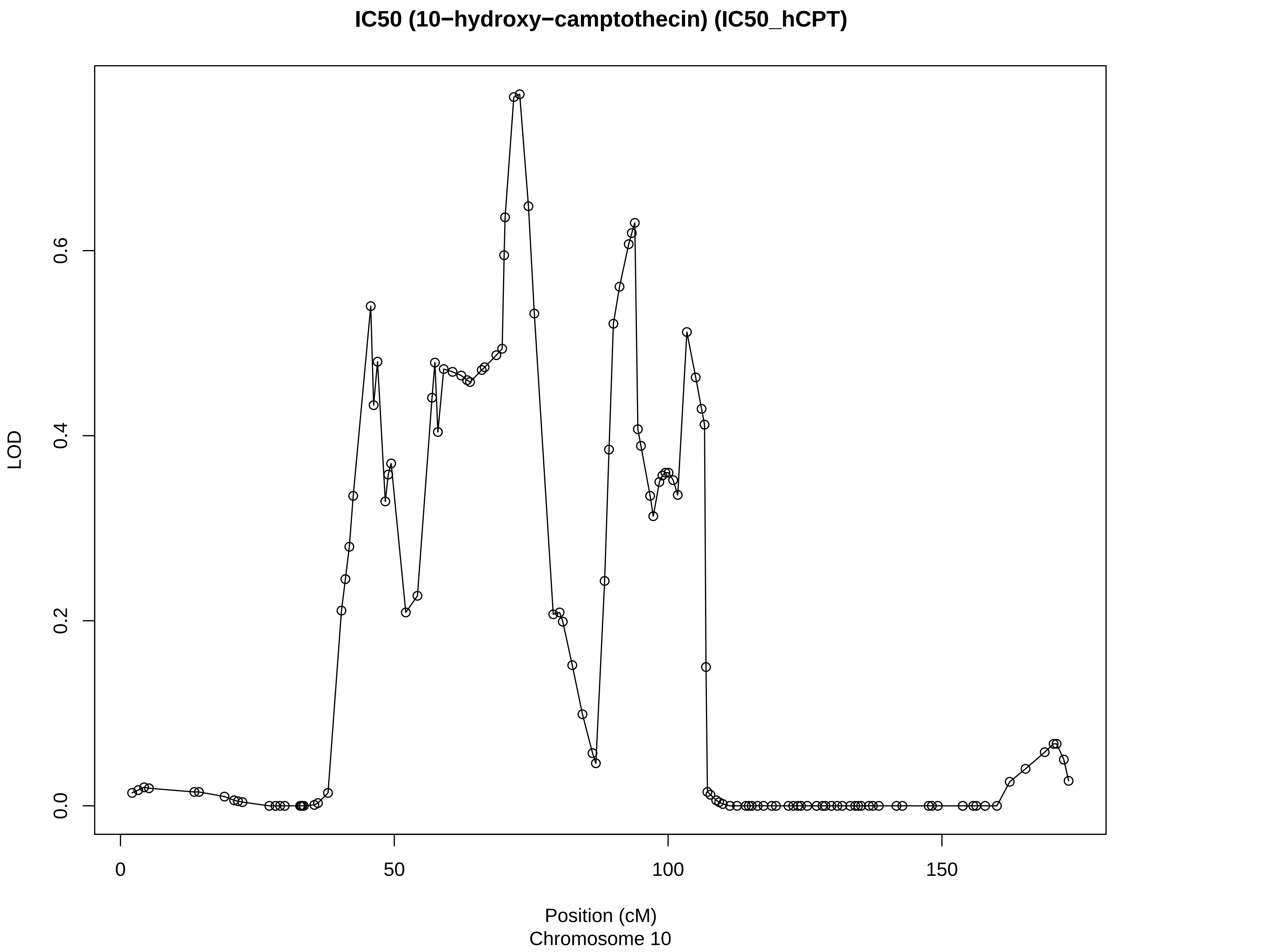
IC50 (10−hydroxy−camptothecin) (IC50_hCPT)
LOD
0.0
0.2
0.4
0.6
0
50
100
150
Position (cM)
Chromosome 10

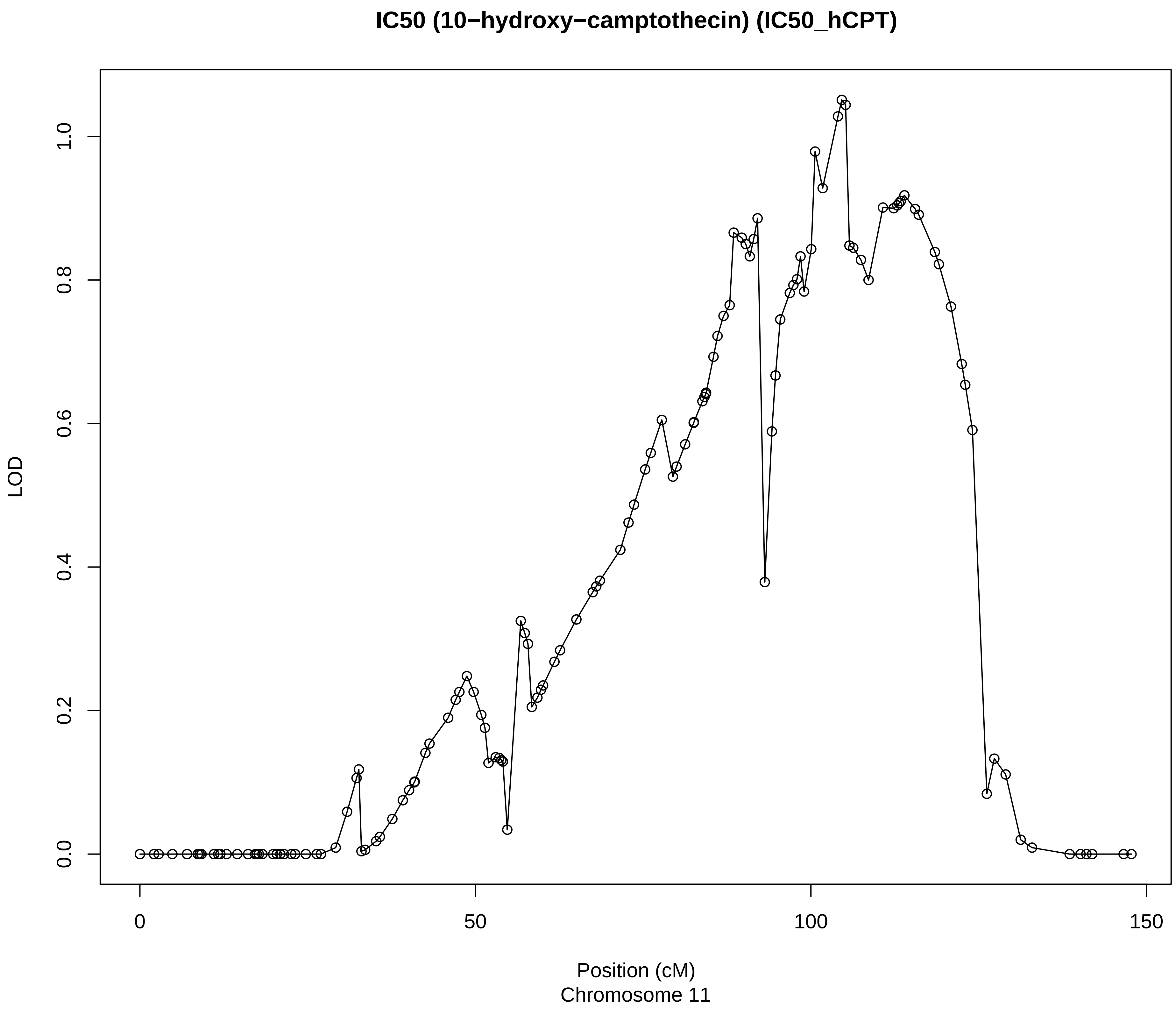
IC50 (10−hydroxy−camptothecin) (IC50_hCPT)
LOD
0.0
0.2
0.4
0.6
0.8
1.0
0
50
100
150
Position (cM)
Chromosome 11

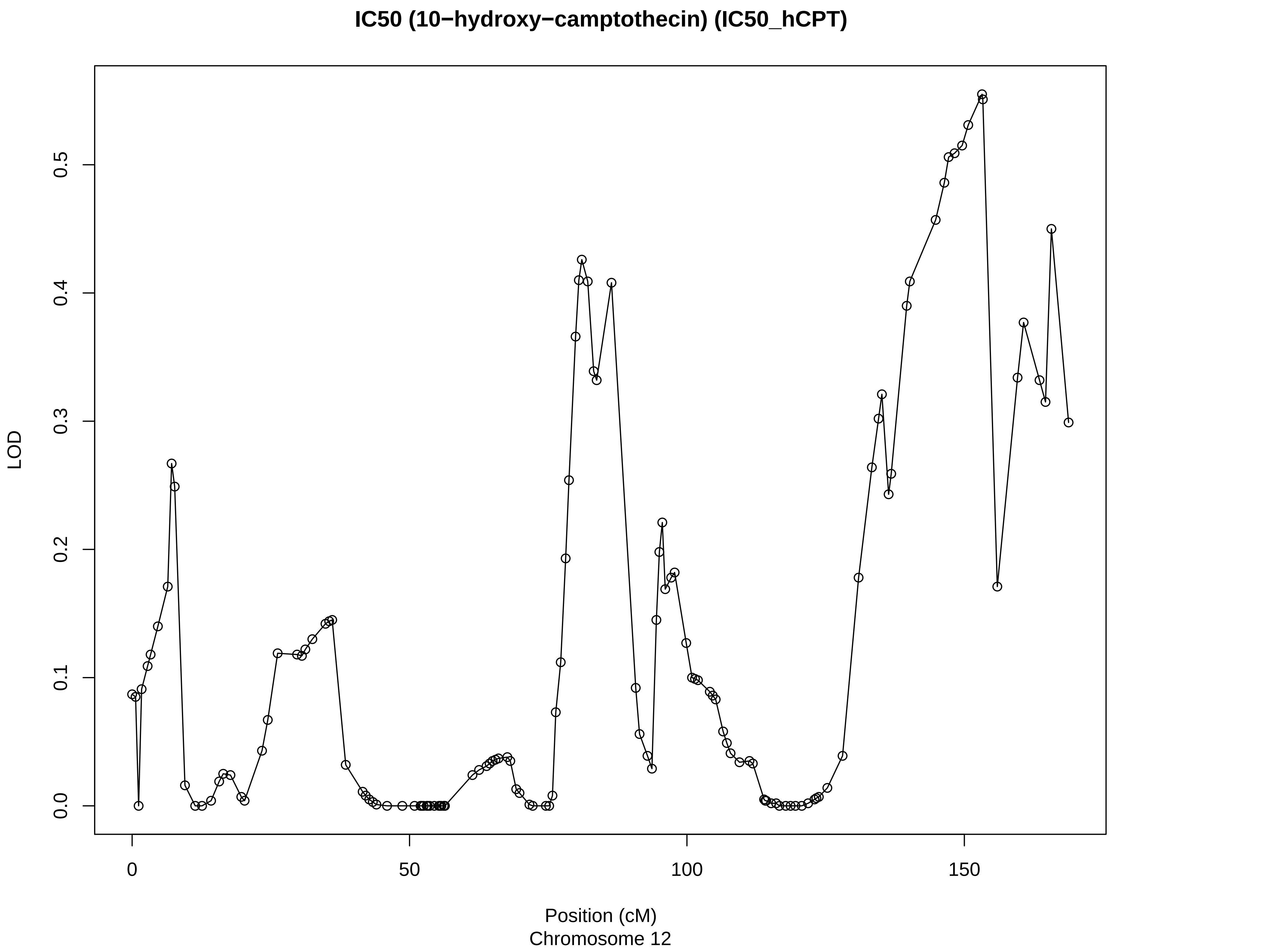

IC50 (10−hydroxy−camptothecin) (IC50_hCPT)
LOD
0.0
0.1
0.2
0.3
0.4
0.5
0
50
100
150
Position (cM)
Chromosome 12

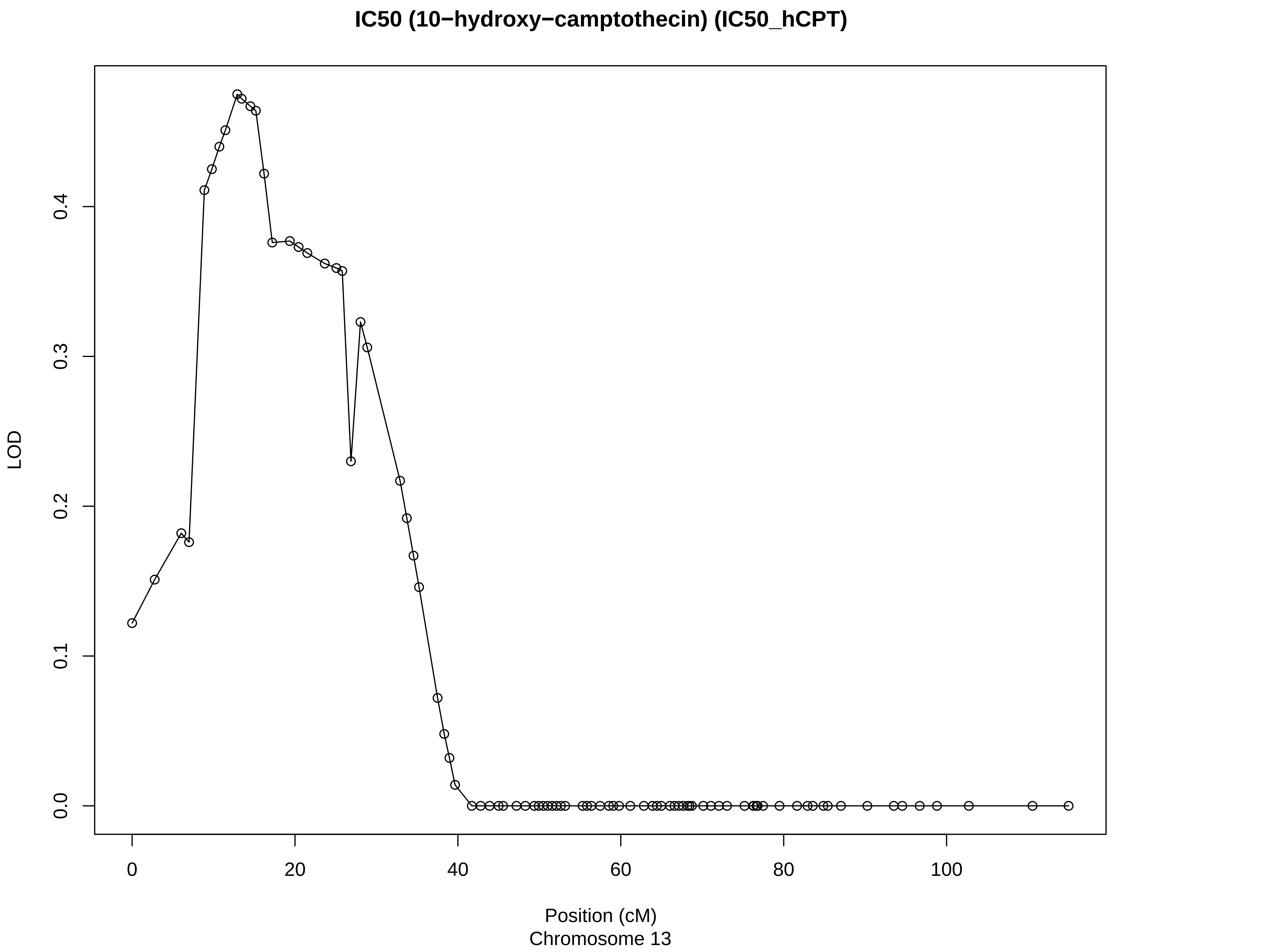

IC50 (10−hydroxy−camptothecin) (IC50_hCPT)
LOD
0.0
0.1
0.2
0.3
0.4
0
20
40
60
80
100
Position (cM)
Chromosome 13

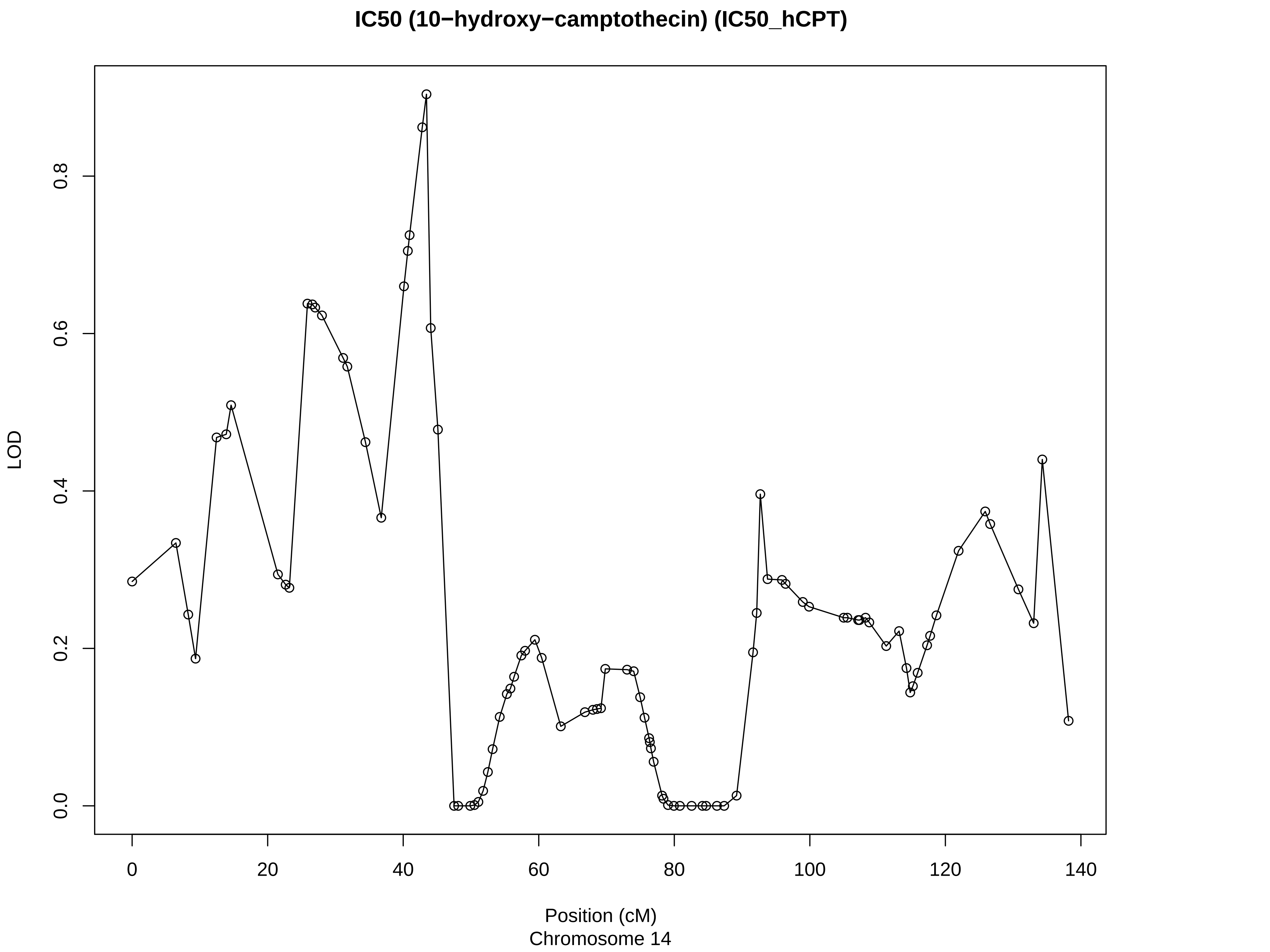
IC50 (10−hydroxy−camptothecin) (IC50_hCPT)
LOD
0.0
0.2
0.4
0.6
0.8
0
20
40
60
80
100
120
140
Position (cM)
Chromosome 14

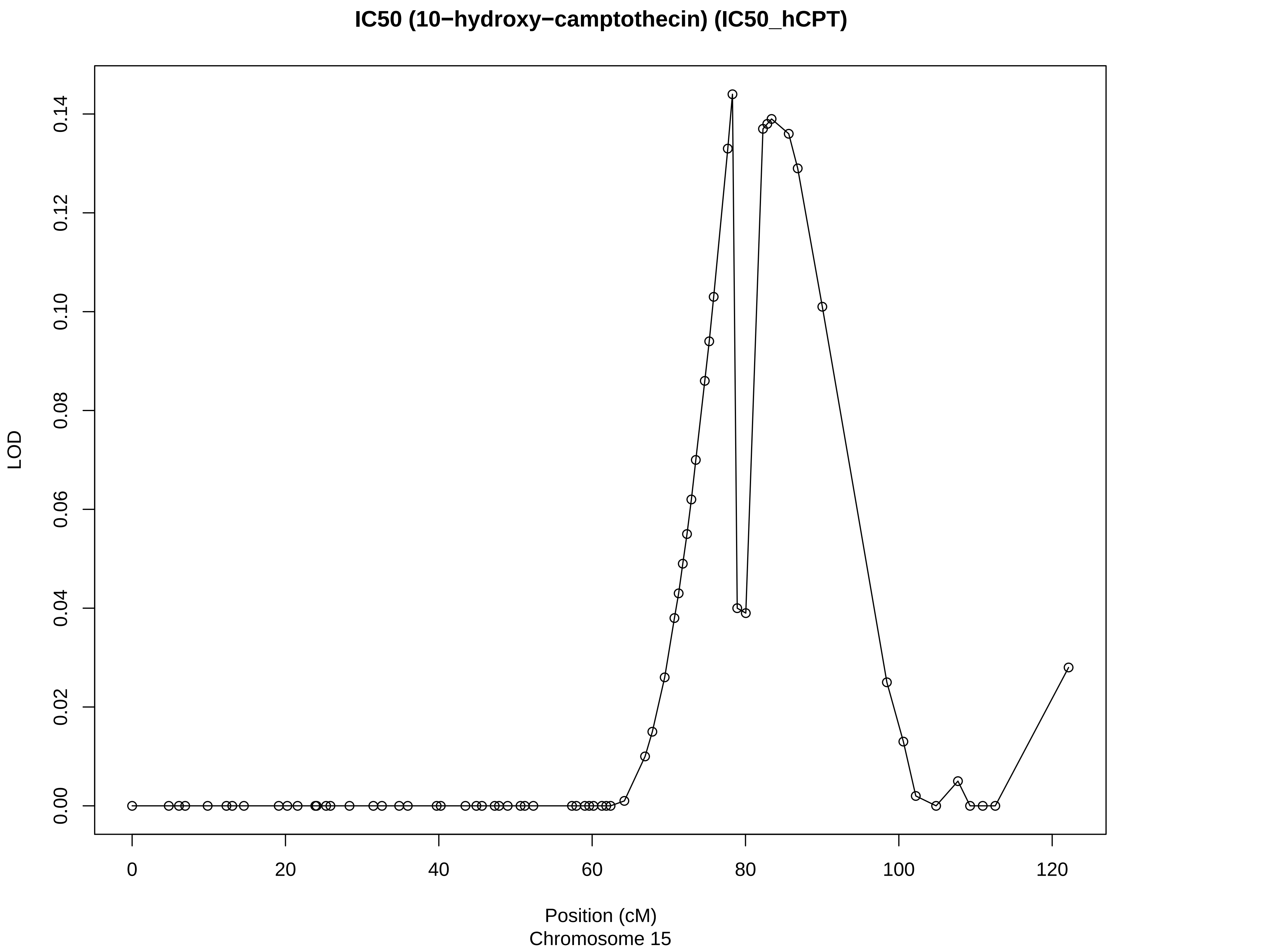

IC50 (10−hydroxy−camptothecin) (IC50_hCPT)
LOD
0.00
0.02
0.04
0.06
0.08
0.10
0.12
0.14
0
20
40
60
80
100
120
Position (cM)
Chromosome 15

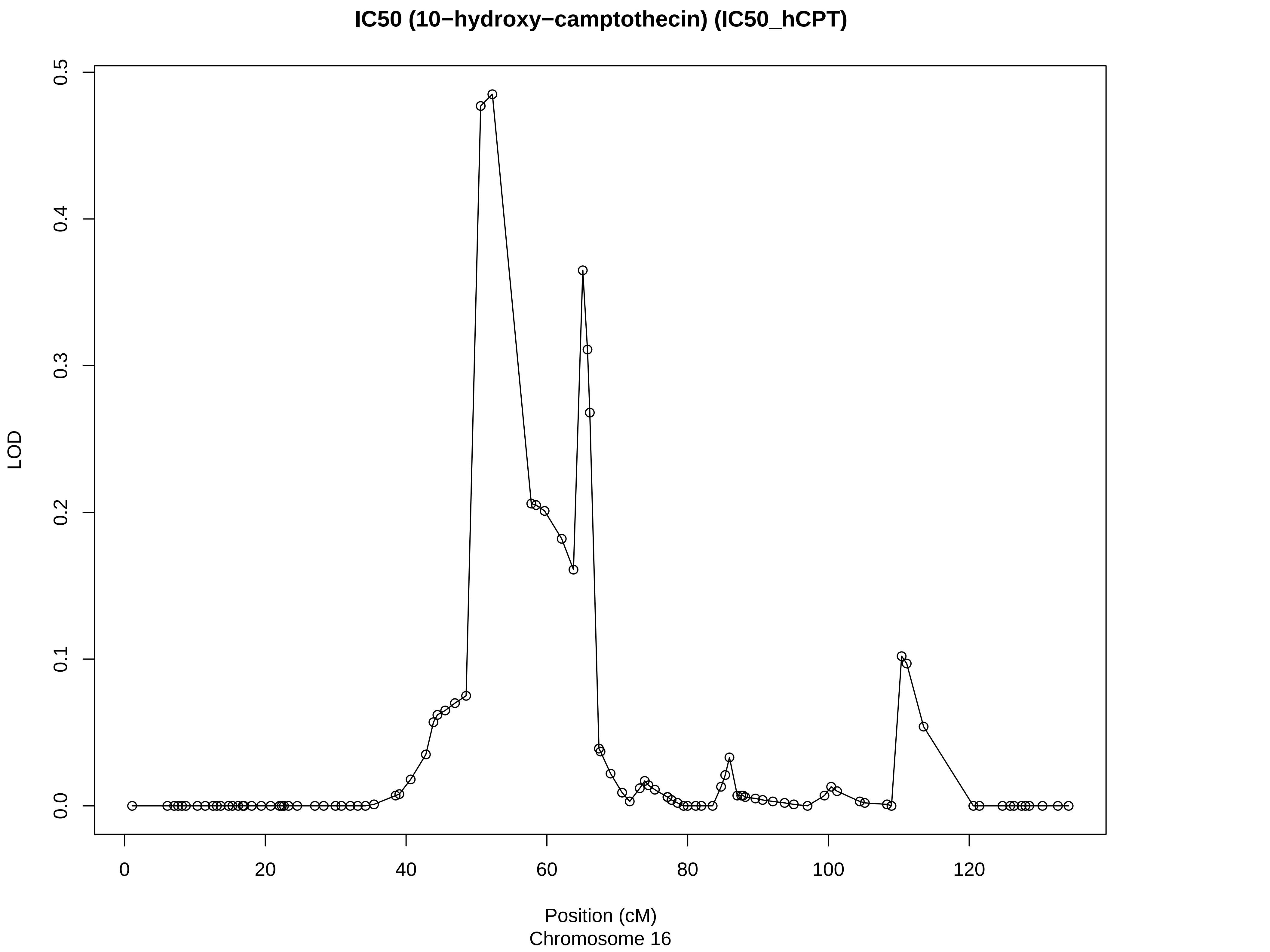
IC50 (10−hydroxy−camptothecin) (IC50_hCPT)
LOD
0.0
0.1
0.2
0.3
0.4
0.5
0
20
40
60
80
100
120
Position (cM)
Chromosome 16

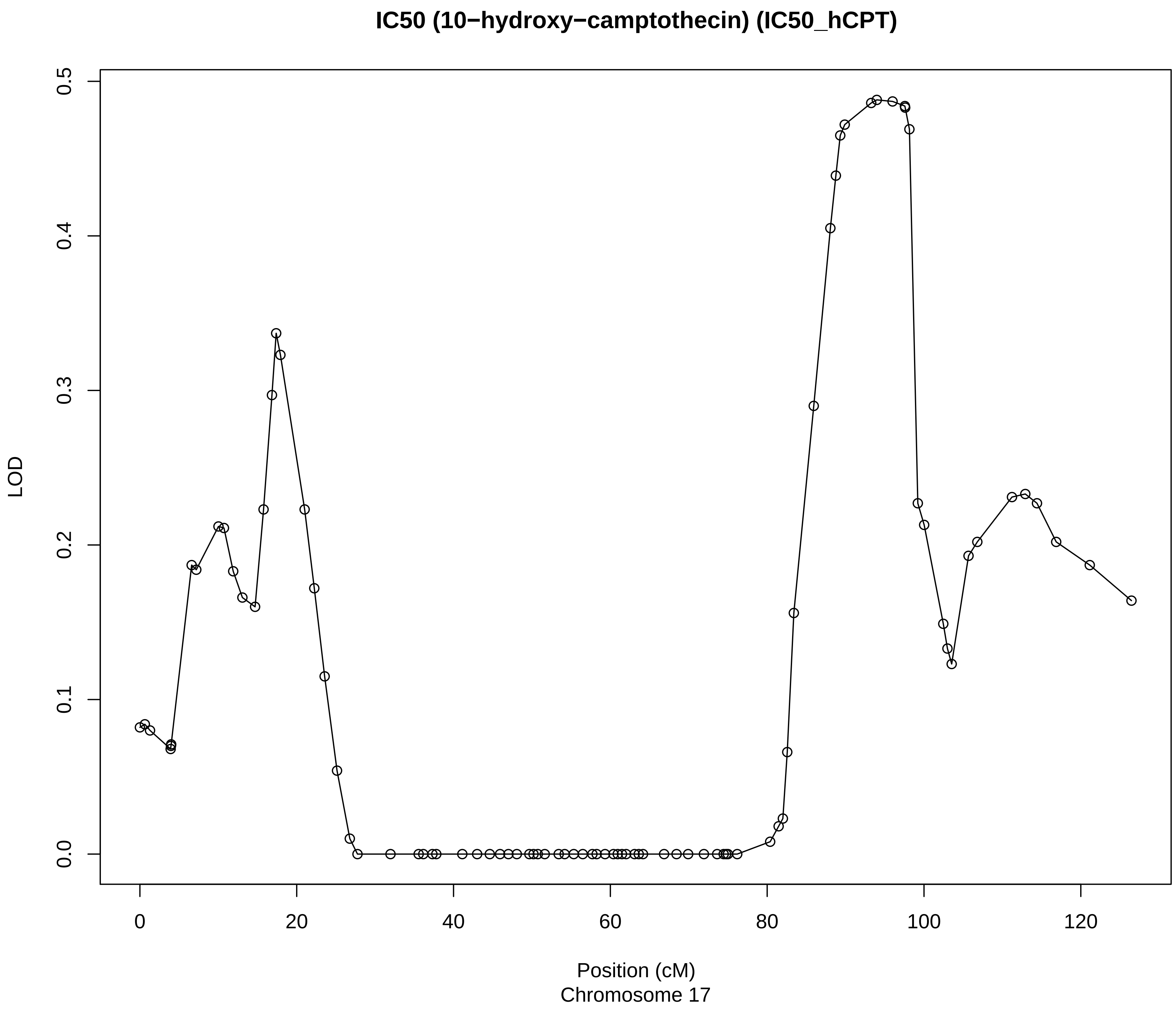

IC50 (10−hydroxy−camptothecin) (IC50_hCPT)
LOD
0.0
0.1
0.2
0.3
0.4
0.5
0
20
40
60
80
100
120
Position (cM)
Chromosome 17

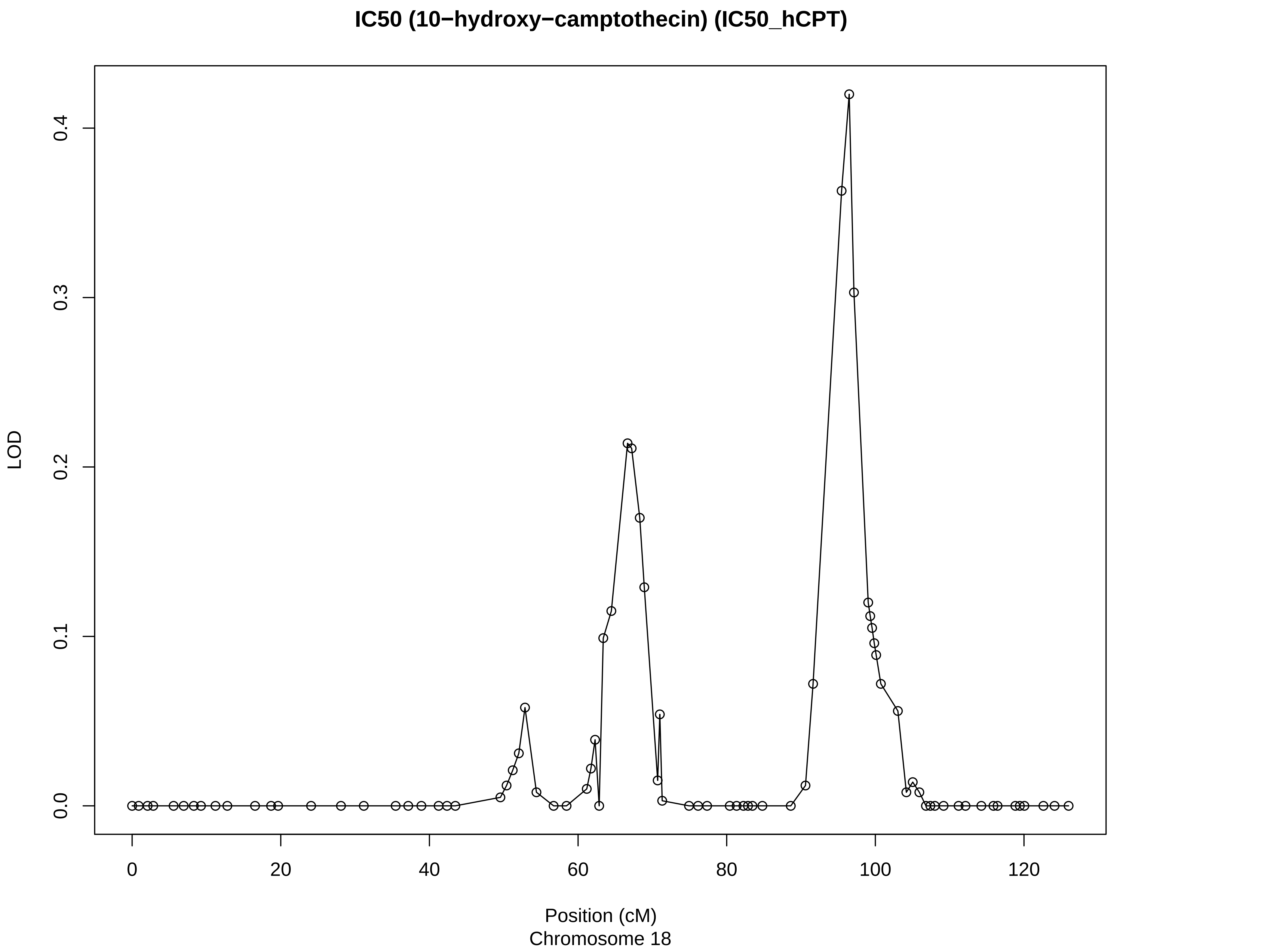
IC50 (10−hydroxy−camptothecin) (IC50_hCPT)
LOD
0.0
0.1
0.2
0.3
0.4
0
20
40
60
80
100
120
Position (cM)
Chromosome 18

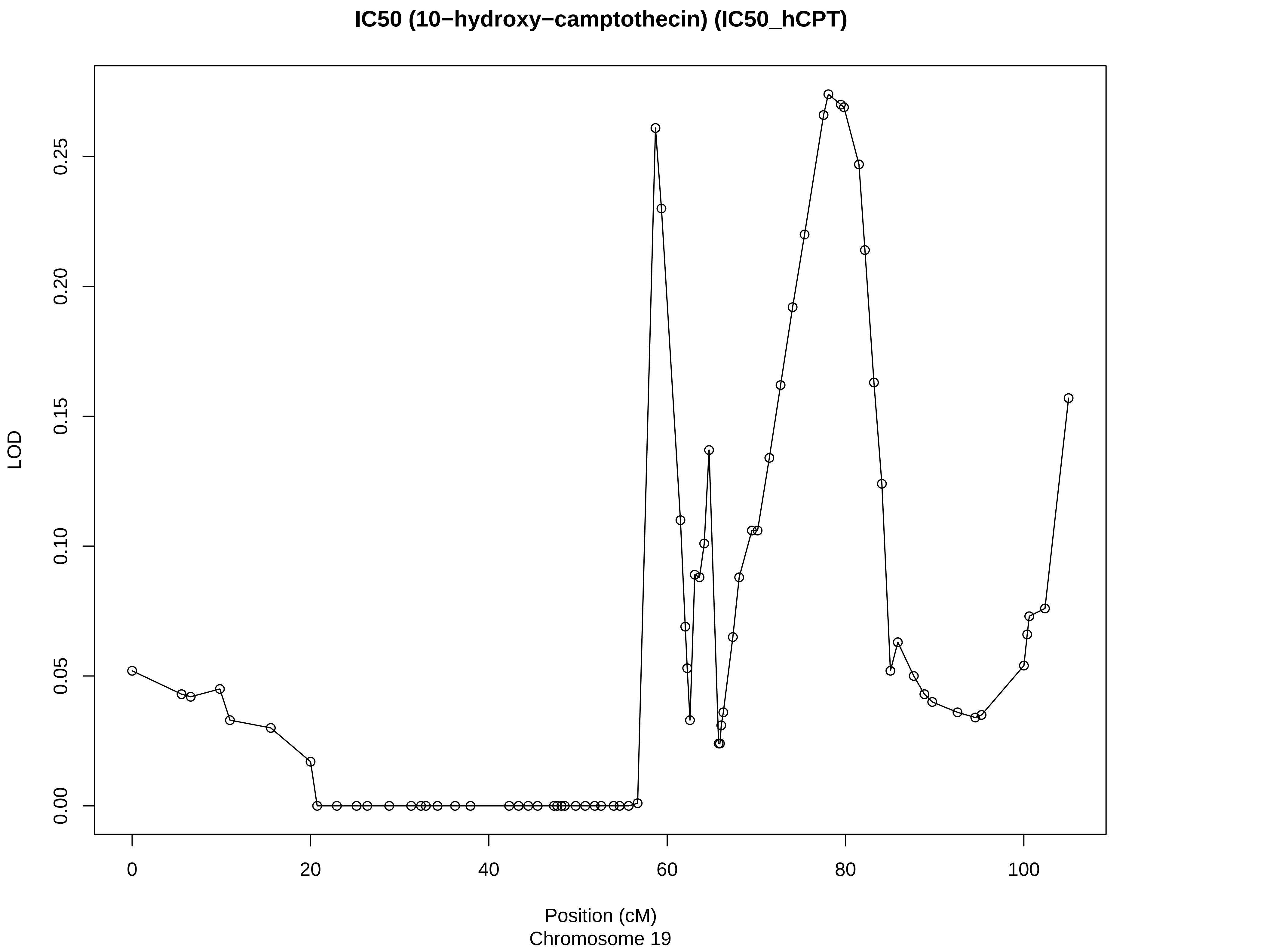
IC50 (10−hydroxy−camptothecin) (IC50_hCPT)
LOD
0.00
0.05
0.10
0.15
0.20
0.25
0
20
40
60
80
100
Position (cM)
Chromosome 19

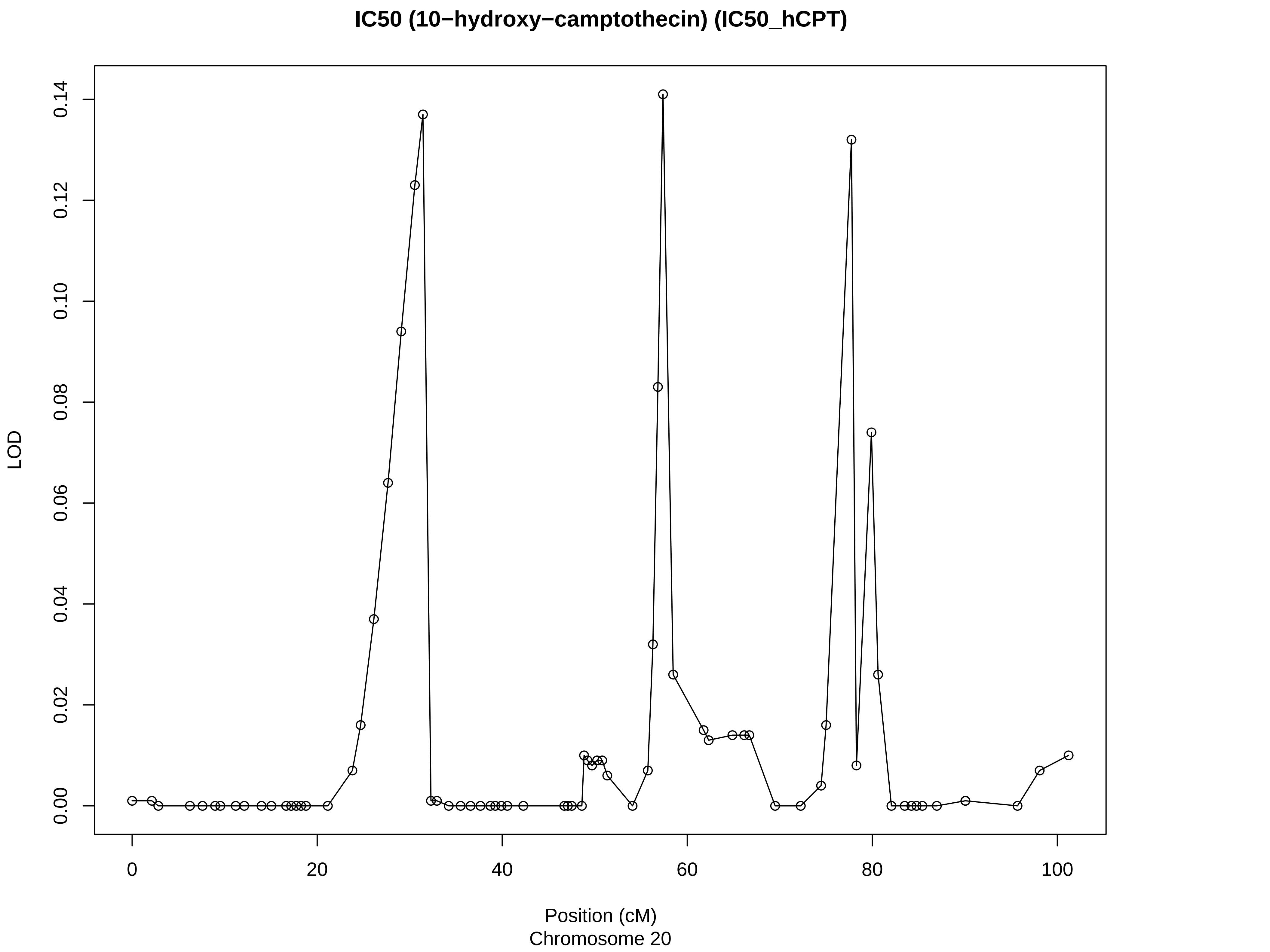
IC50 (10−hydroxy−camptothecin) (IC50_hCPT)
LOD
0.00
0.02
0.04
0.06
0.08
0.10
0.12
0.14
0
20
40
60
80
100
Position (cM)
Chromosome 20

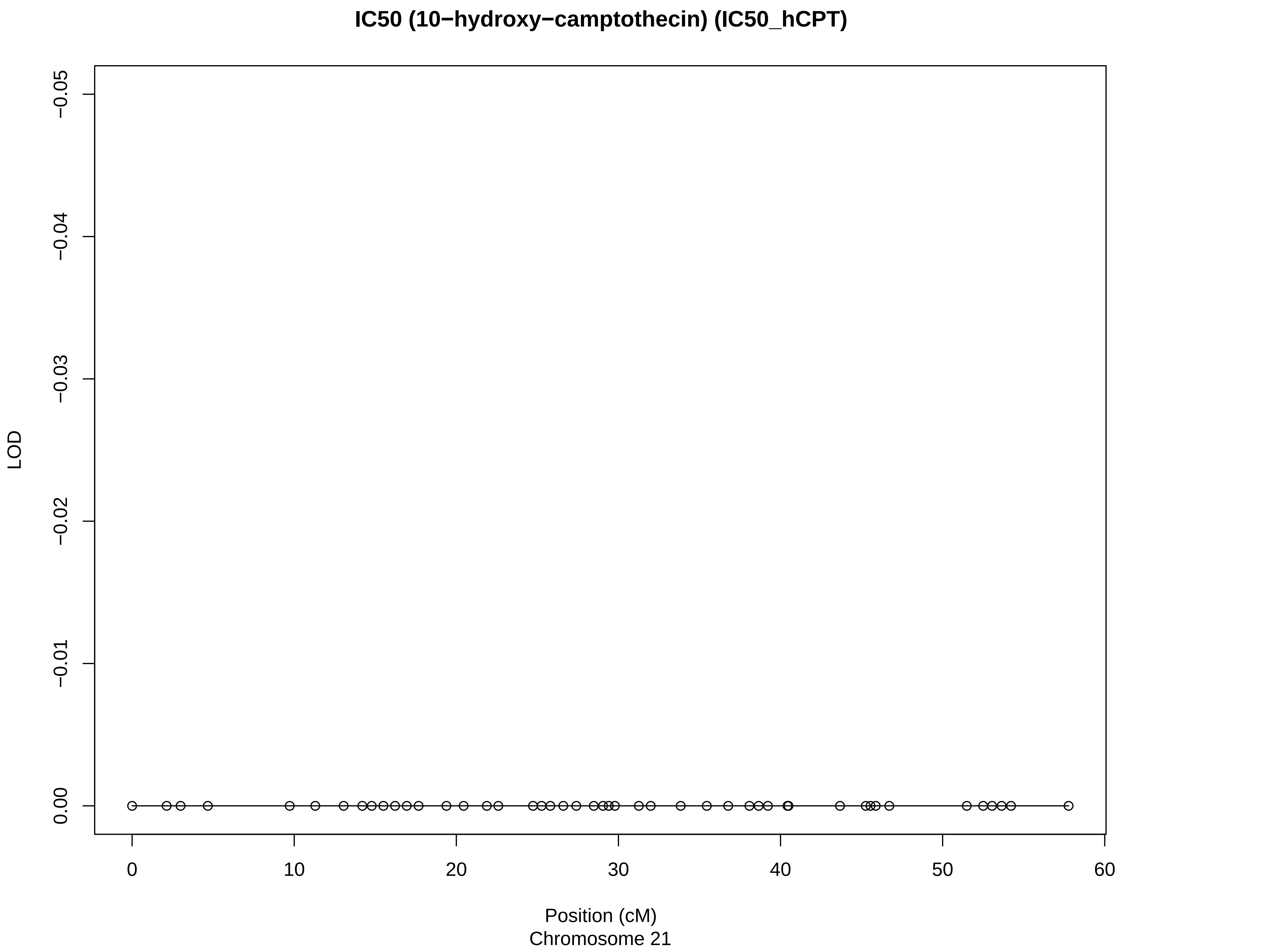
IC50 (10−hydroxy−camptothecin) (IC50_hCPT)
LOD
−0.05
−0.04
−0.03
−0.02
−0.01
0.00
0
10
20
30
40
50
60
Position (cM)
Chromosome 21

IC50 (10−hydroxy−camptothecin) (IC50_hCPT)

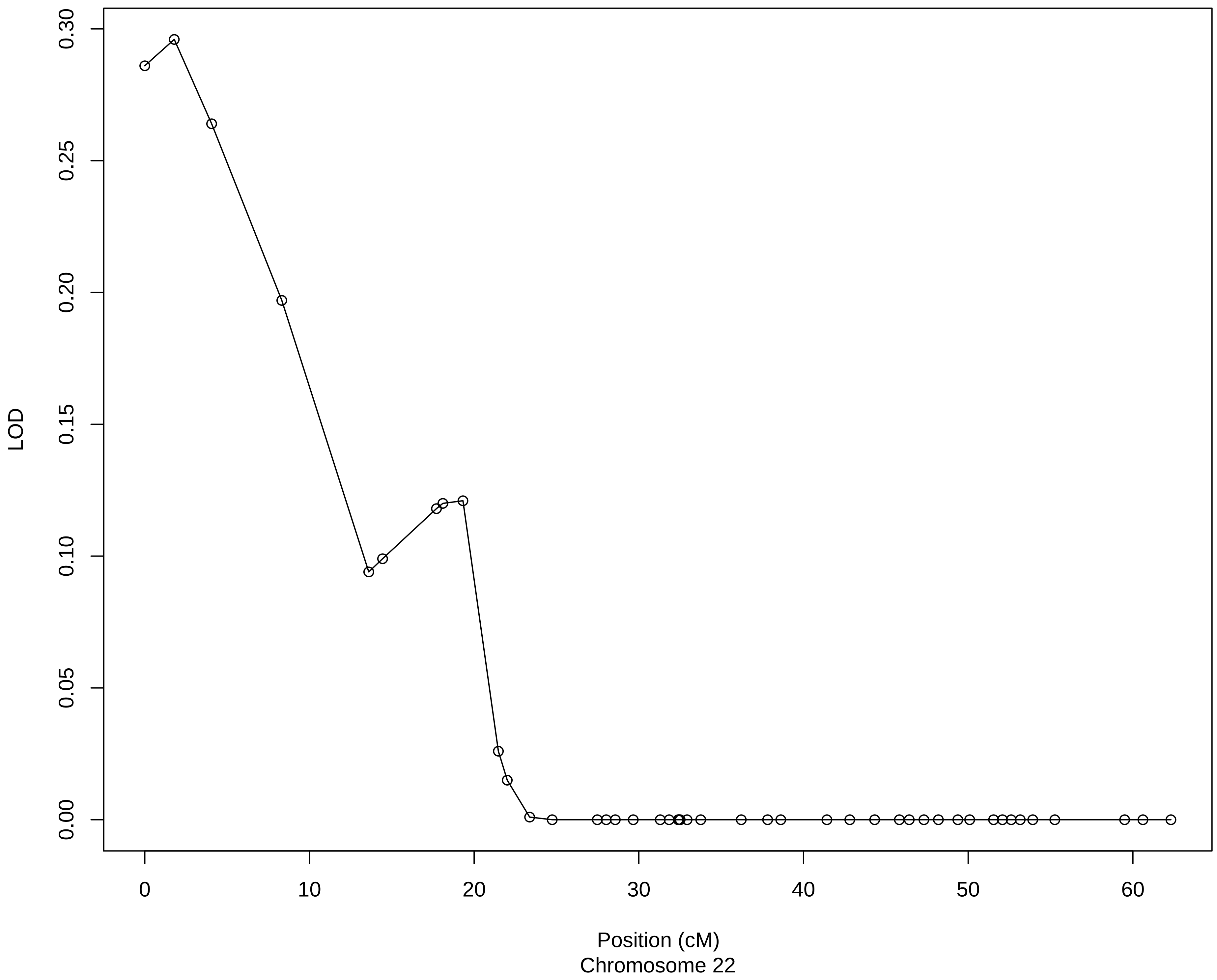

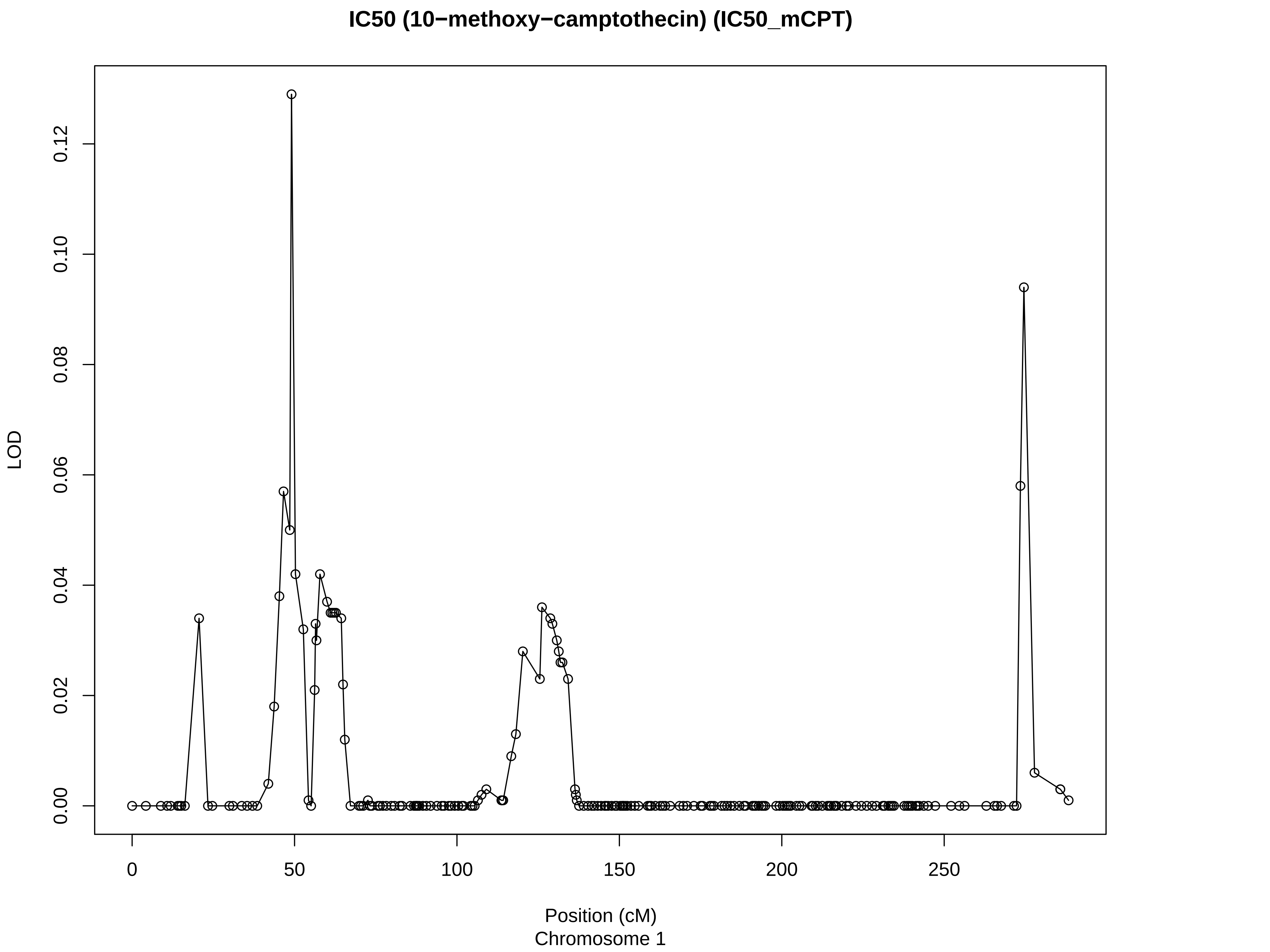
IC50 (10−methoxy−camptothecin) (IC50_mCPT)
LOD
0.00
0.02
0.04
0.06
0.08
0.10
0.12
0
50
100
150
200
250
Position (cM)
Chromosome 1

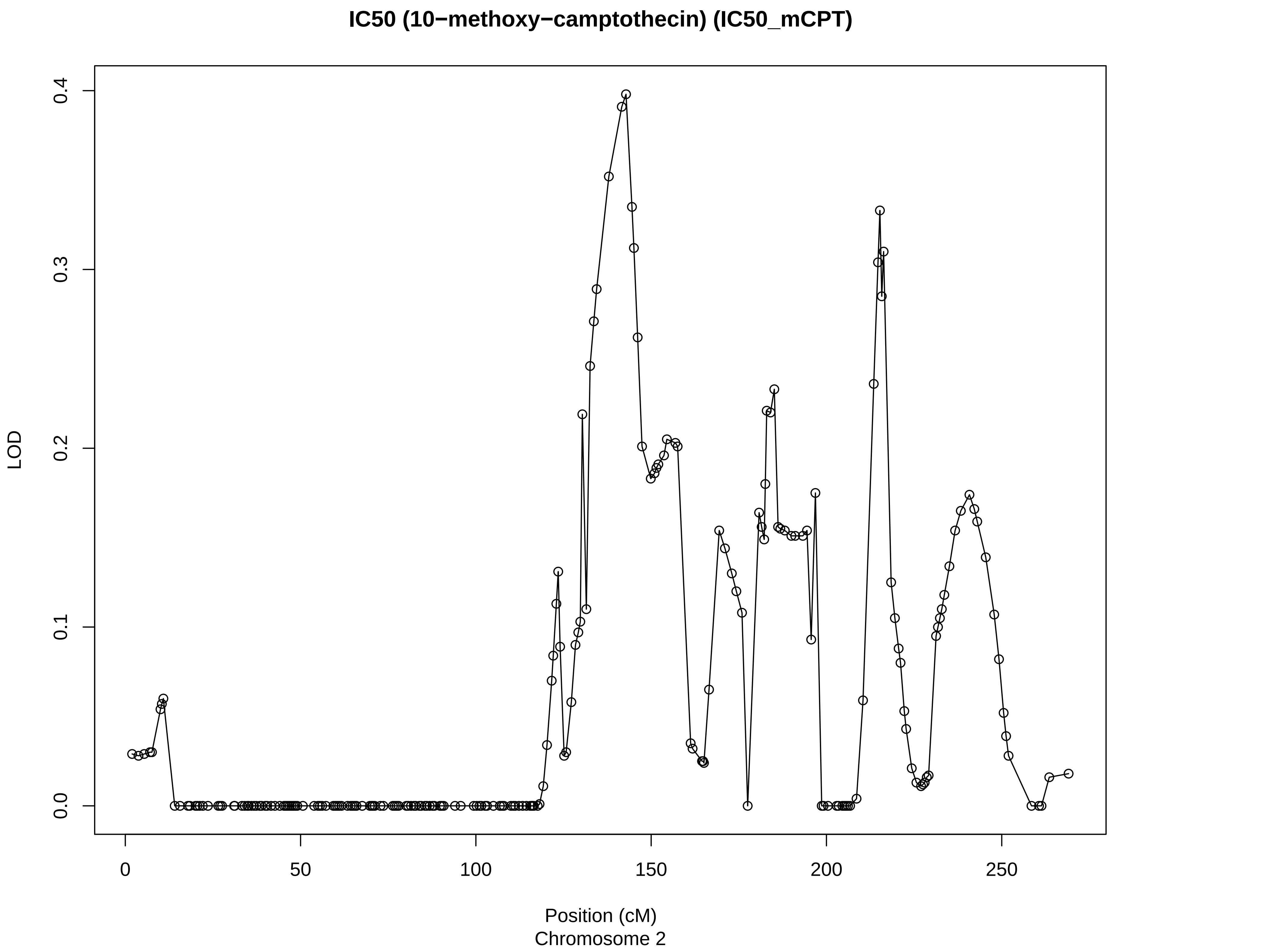

IC50 (10−methoxy−camptothecin) (IC50_mCPT)
LOD
0.0
0.1
0.2
0.3
0.4
0
50
100
150
200
250
Position (cM)
Chromosome 2

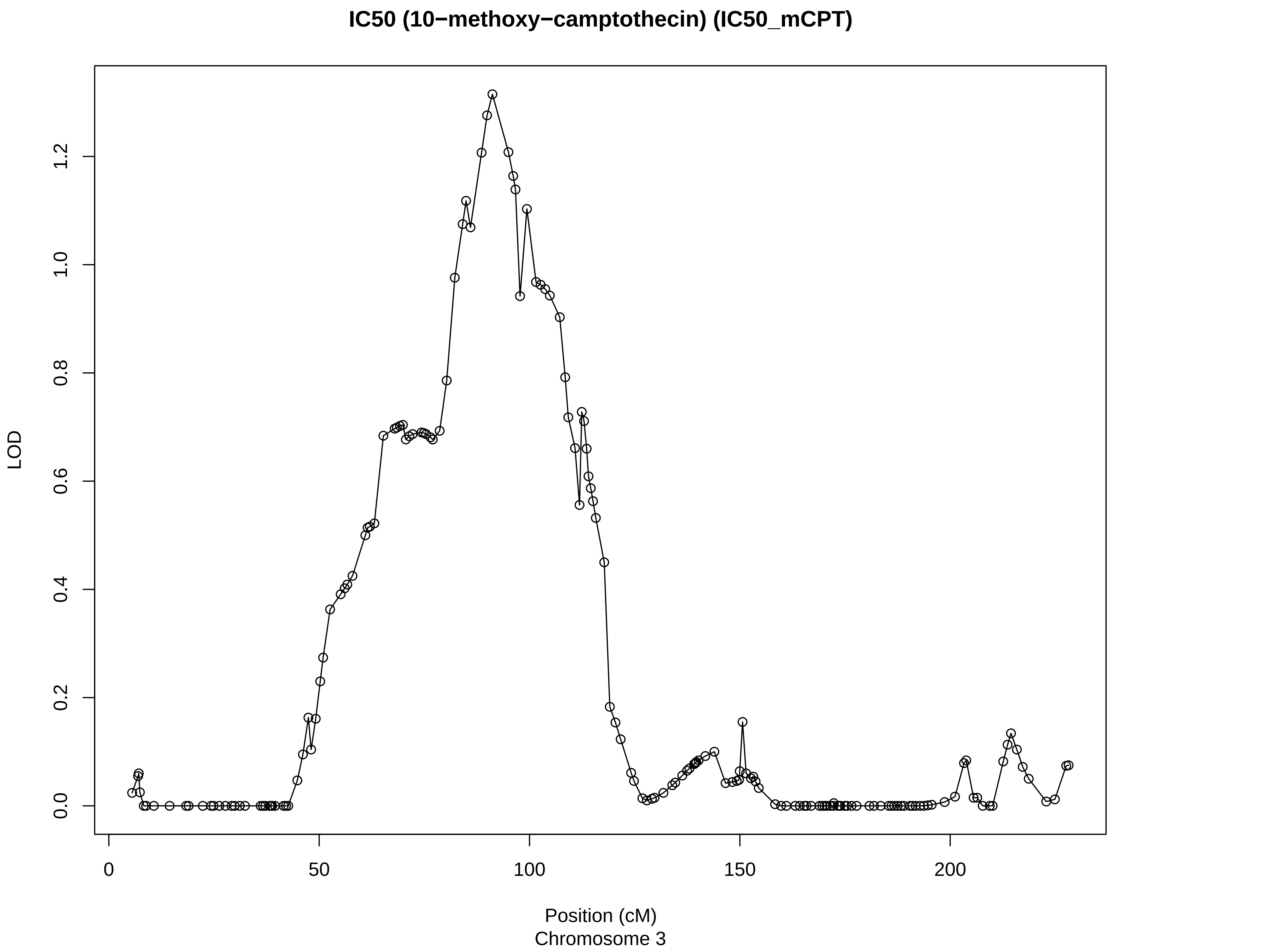

IC50 (10−methoxy−camptothecin) (IC50_mCPT)
LOD
0.0
0.2
0.4
0.6
0.8
1.0
1.2
0
50
100
150
200
Position (cM)
Chromosome 3

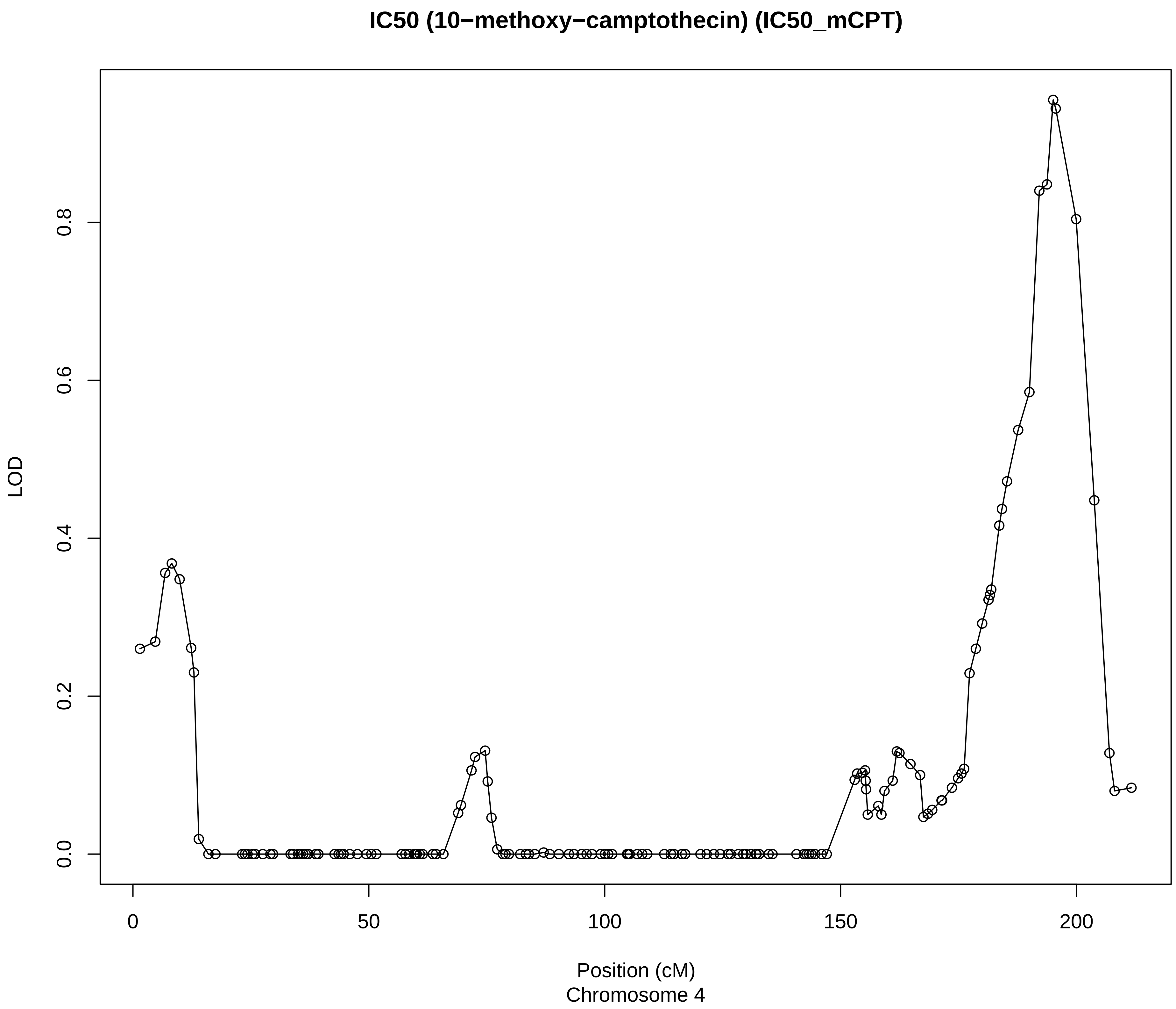

IC50 (10−methoxy−camptothecin) (IC50_mCPT)
LOD
0.0
0.2
0.4
0.6
0.8
0
50
100
150
200
Position (cM)
Chromosome 4

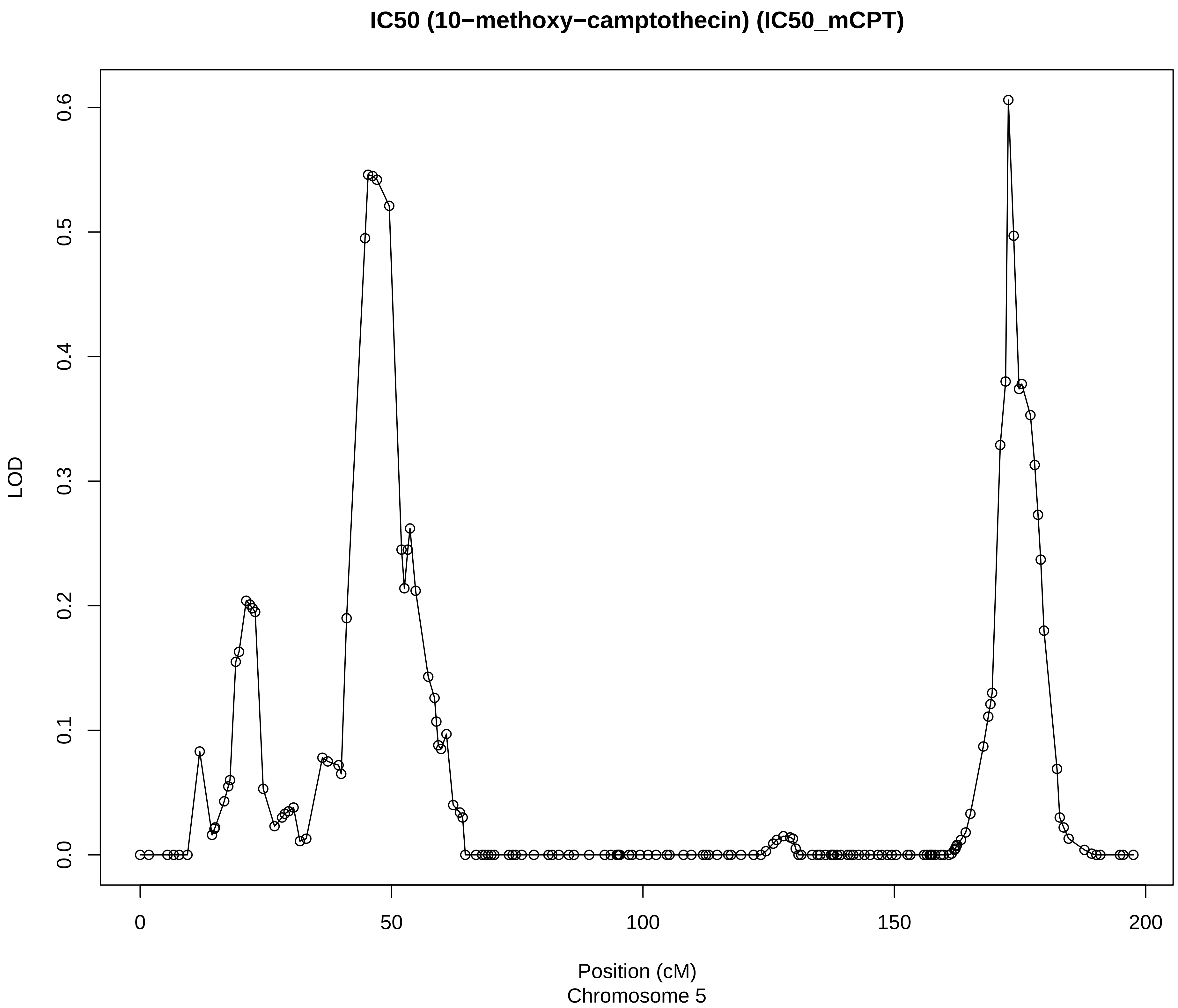
IC50 (10−methoxy−camptothecin) (IC50_mCPT)
LOD
0.0
0.1
0.2
0.3
0.4
0.5
0.6
0
50
100
150
200
Position (cM)
Chromosome 5

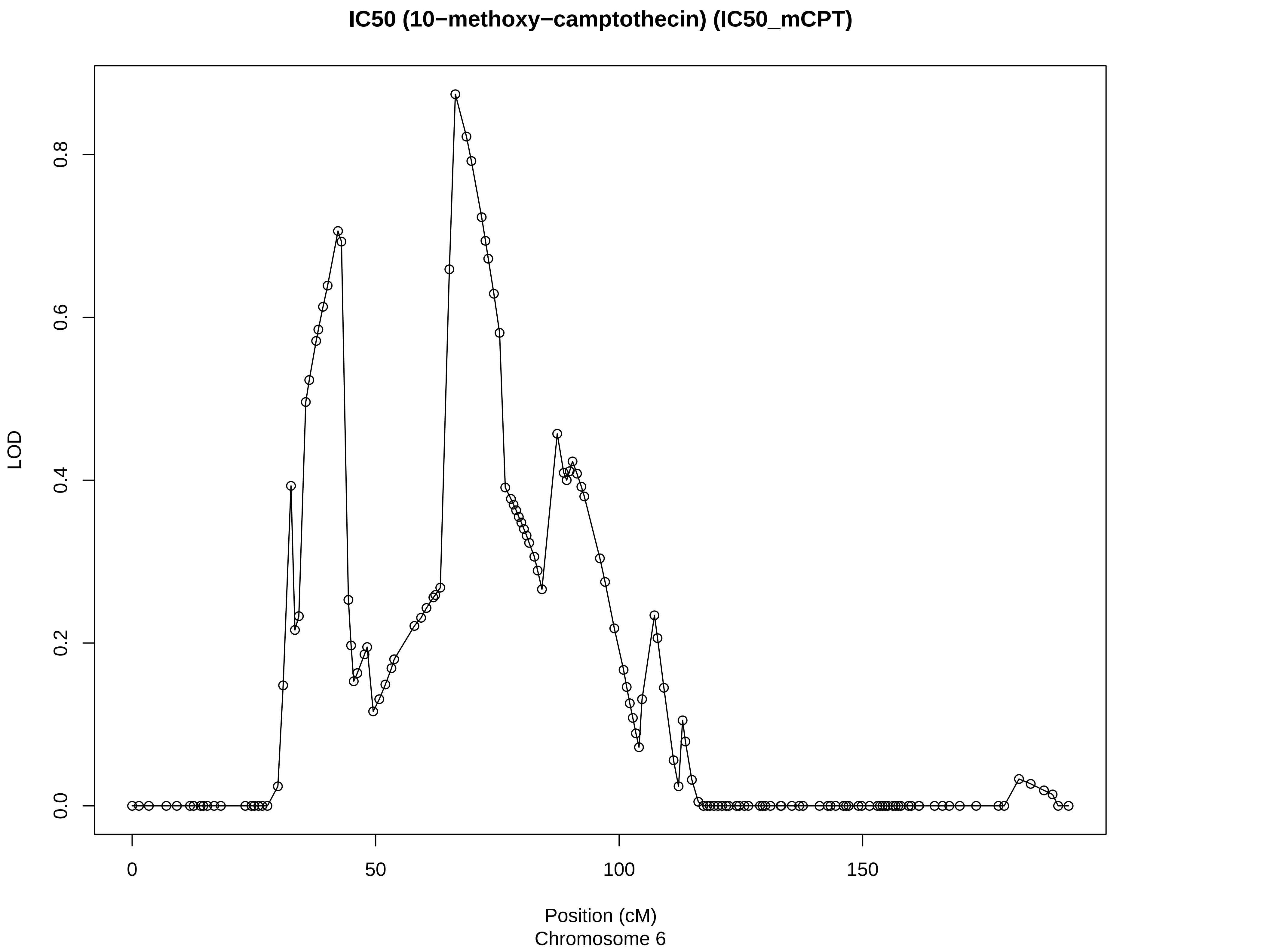
IC50 (10−methoxy−camptothecin) (IC50_mCPT)
LOD
0.0
0.2
0.4
0.6
0.8
0
50
100
150
Position (cM)
Chromosome 6

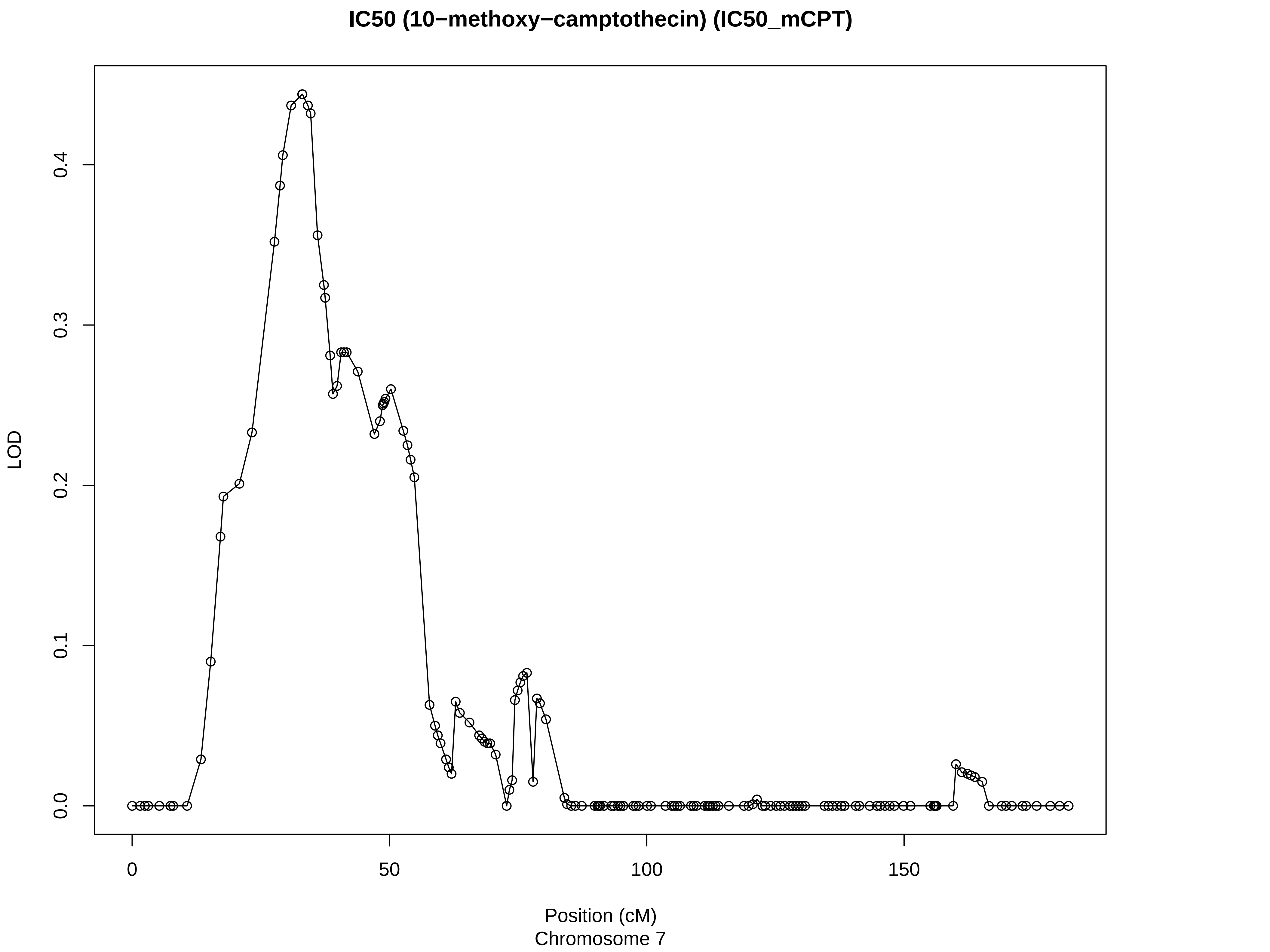
IC50 (10−methoxy−camptothecin) (IC50_mCPT)
LOD
0.0
0.1
0.2
0.3
0.4
0
50
100
150
Position (cM)
Chromosome 7

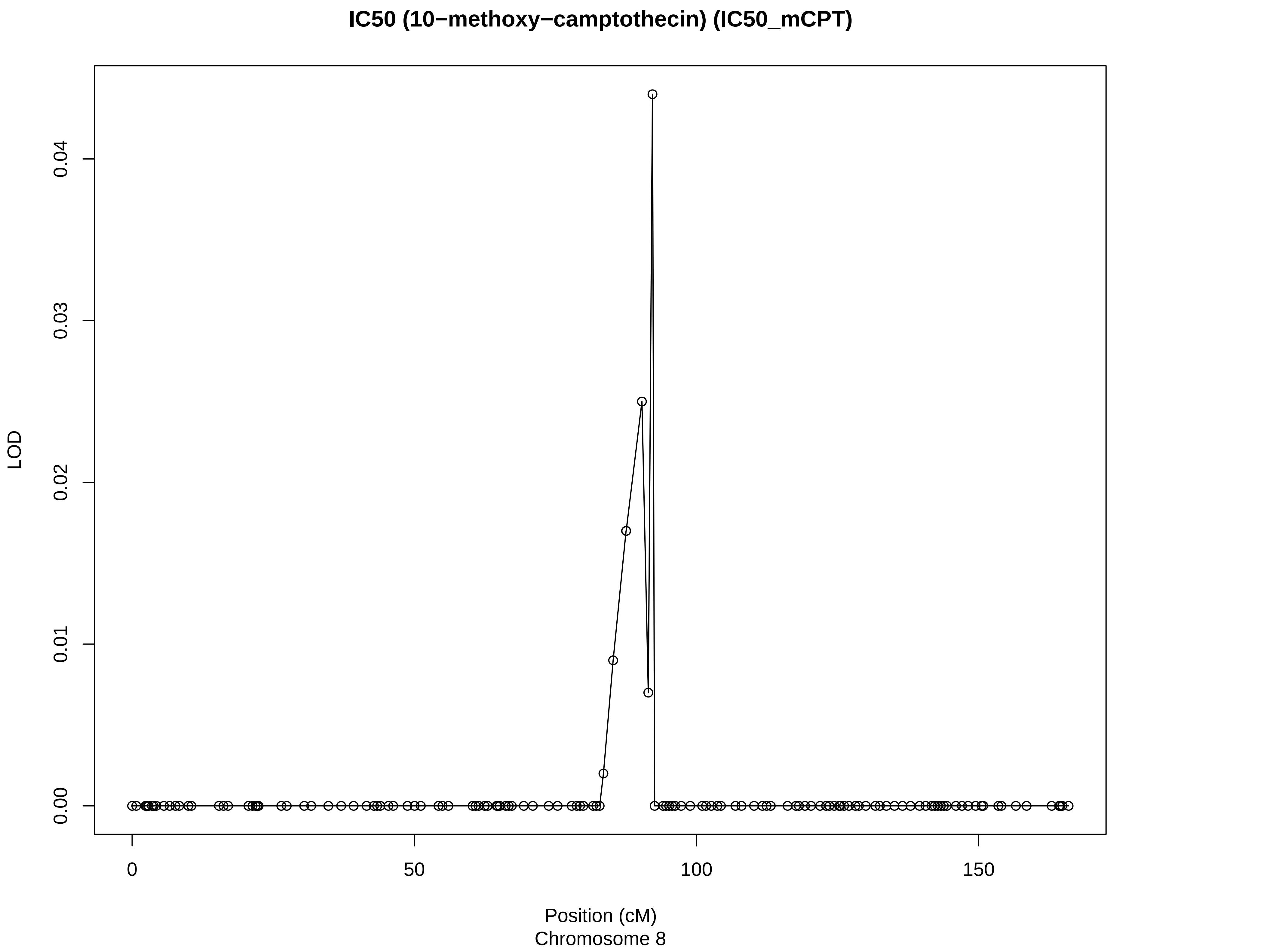
IC50 (10−methoxy−camptothecin) (IC50_mCPT)
LOD
0.00
0.01
0.02
0.03
0.04
0
50
100
150
Position (cM)
Chromosome 8

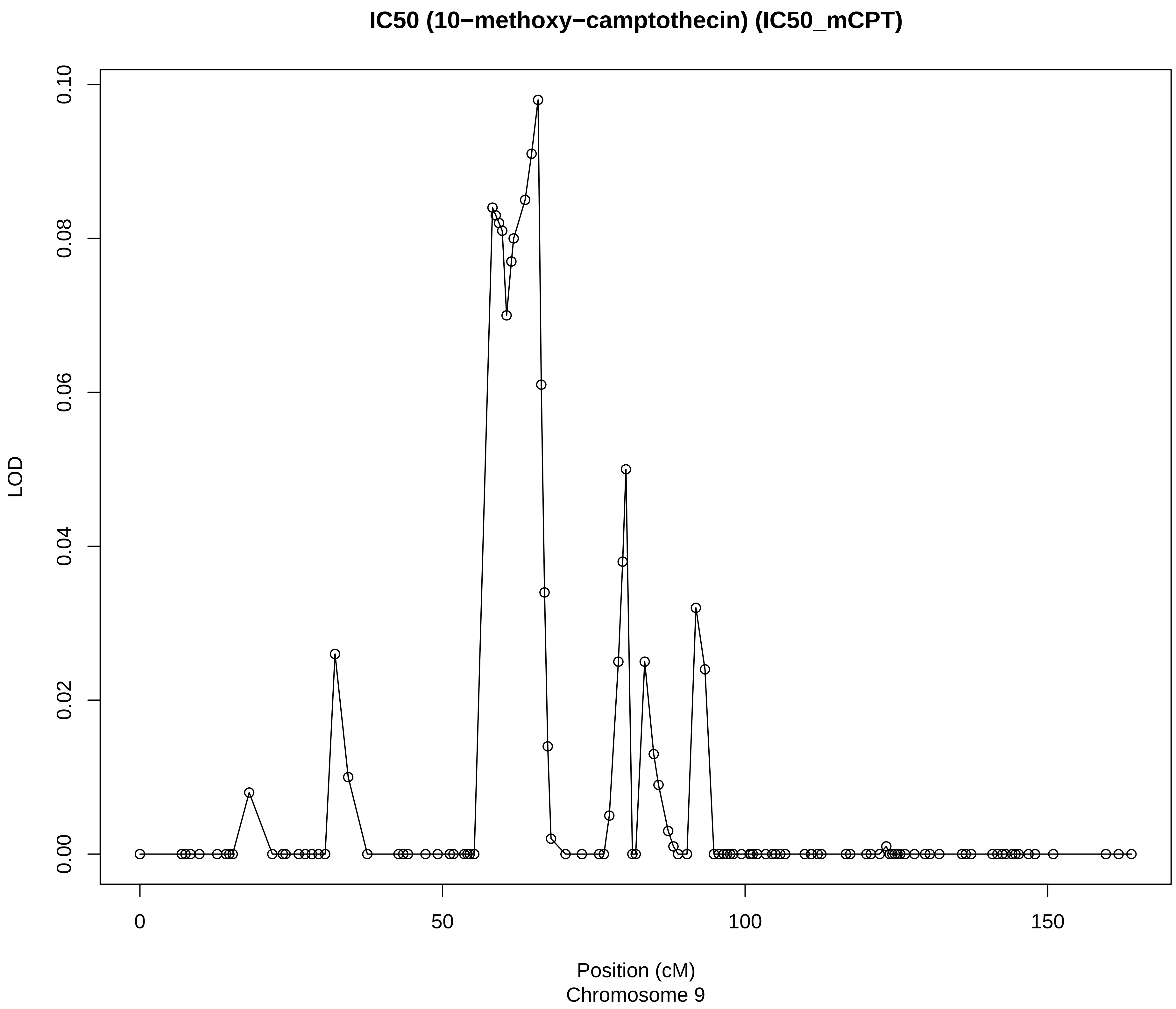
IC50 (10−methoxy−camptothecin) (IC50_mCPT)
LOD
0.00
0.02
0.04
0.06
0.08
0.10
0
50
100
150
Position (cM)
Chromosome 9

IC50 (10−methoxy−camptothecin) (IC50_mCPT)

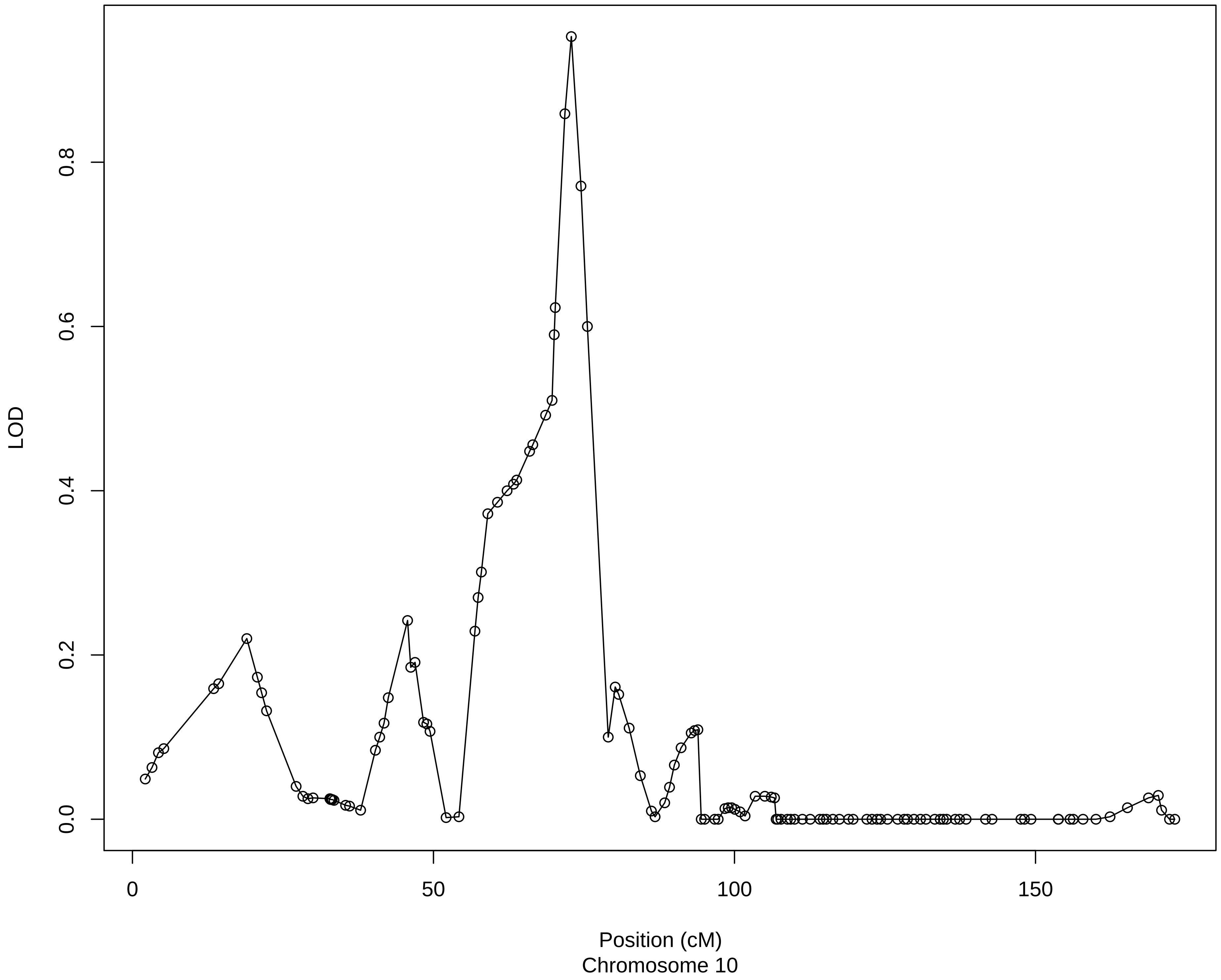

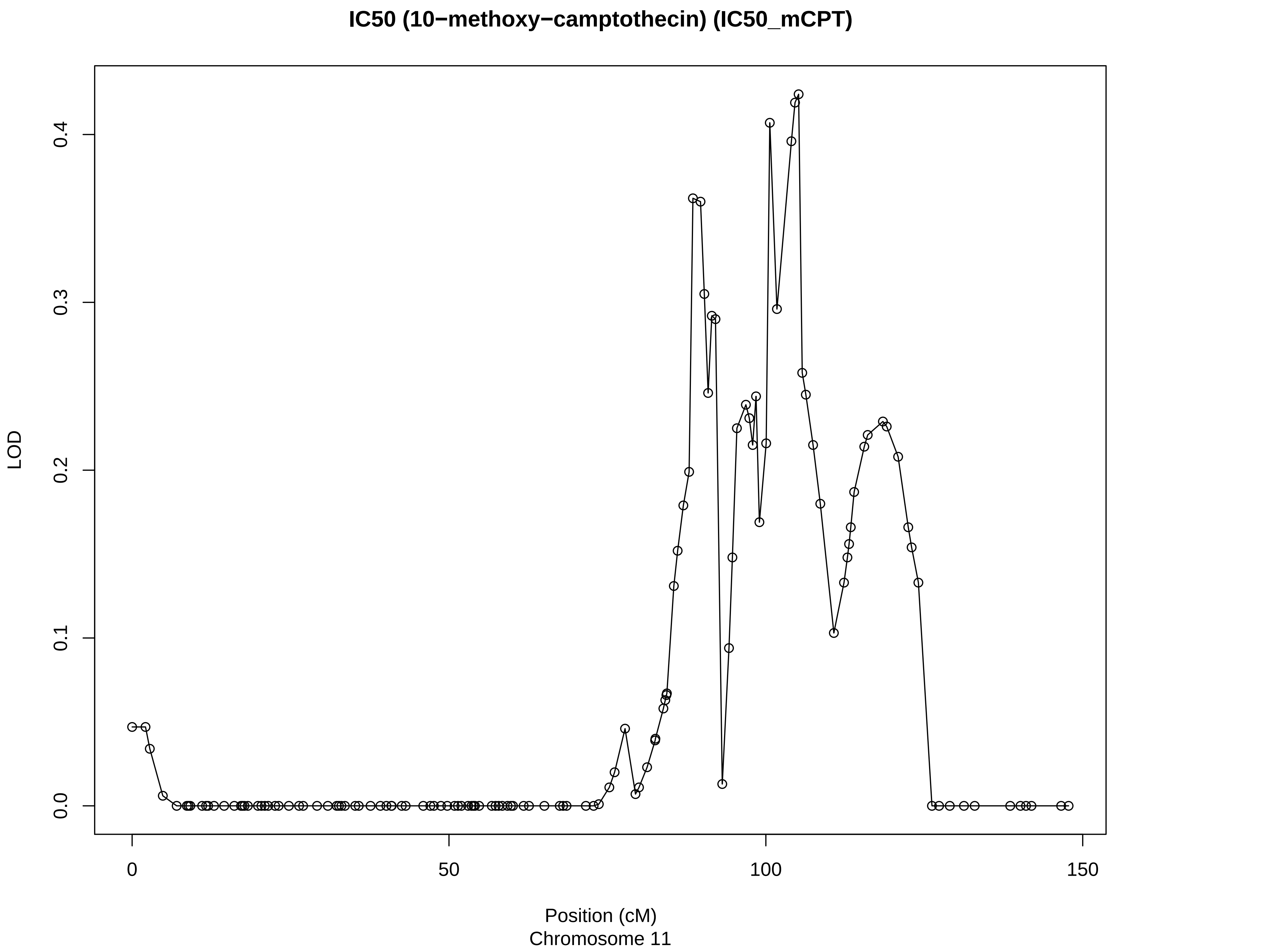

IC50 (10−methoxy−camptothecin) (IC50_mCPT)
LOD
0.0
0.1
0.2
0.3
0.4
0
50
100
150
Position (cM)
Chromosome 11

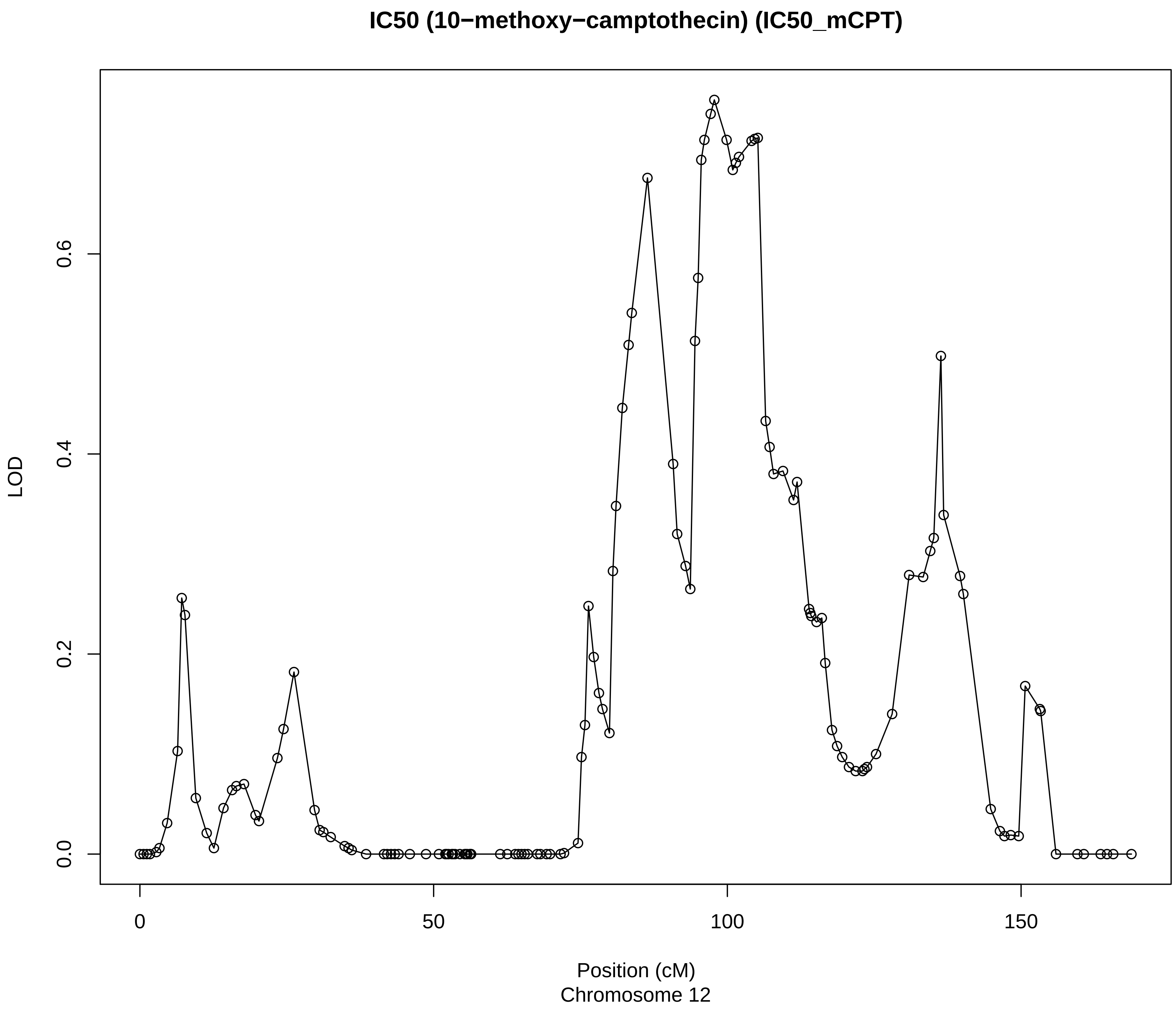
IC50 (10−methoxy−camptothecin) (IC50_mCPT)
LOD
0.0
0.2
0.4
0.6
0
50
100
150
Position (cM)
Chromosome 12

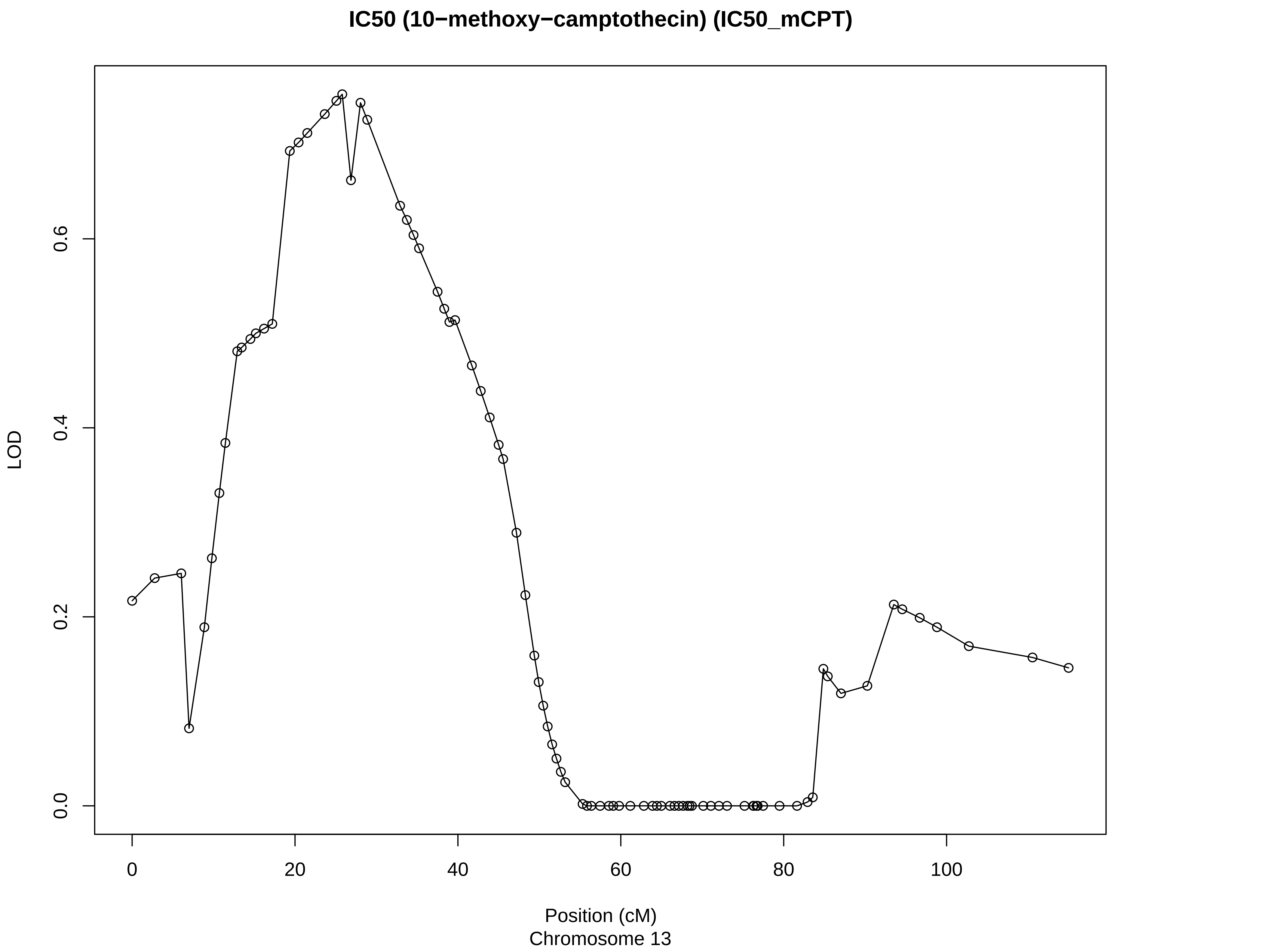
IC50 (10−methoxy−camptothecin) (IC50_mCPT)
LOD
0.0
0.2
0.4
0.6
0
20
40
60
80
100
Position (cM)
Chromosome 13

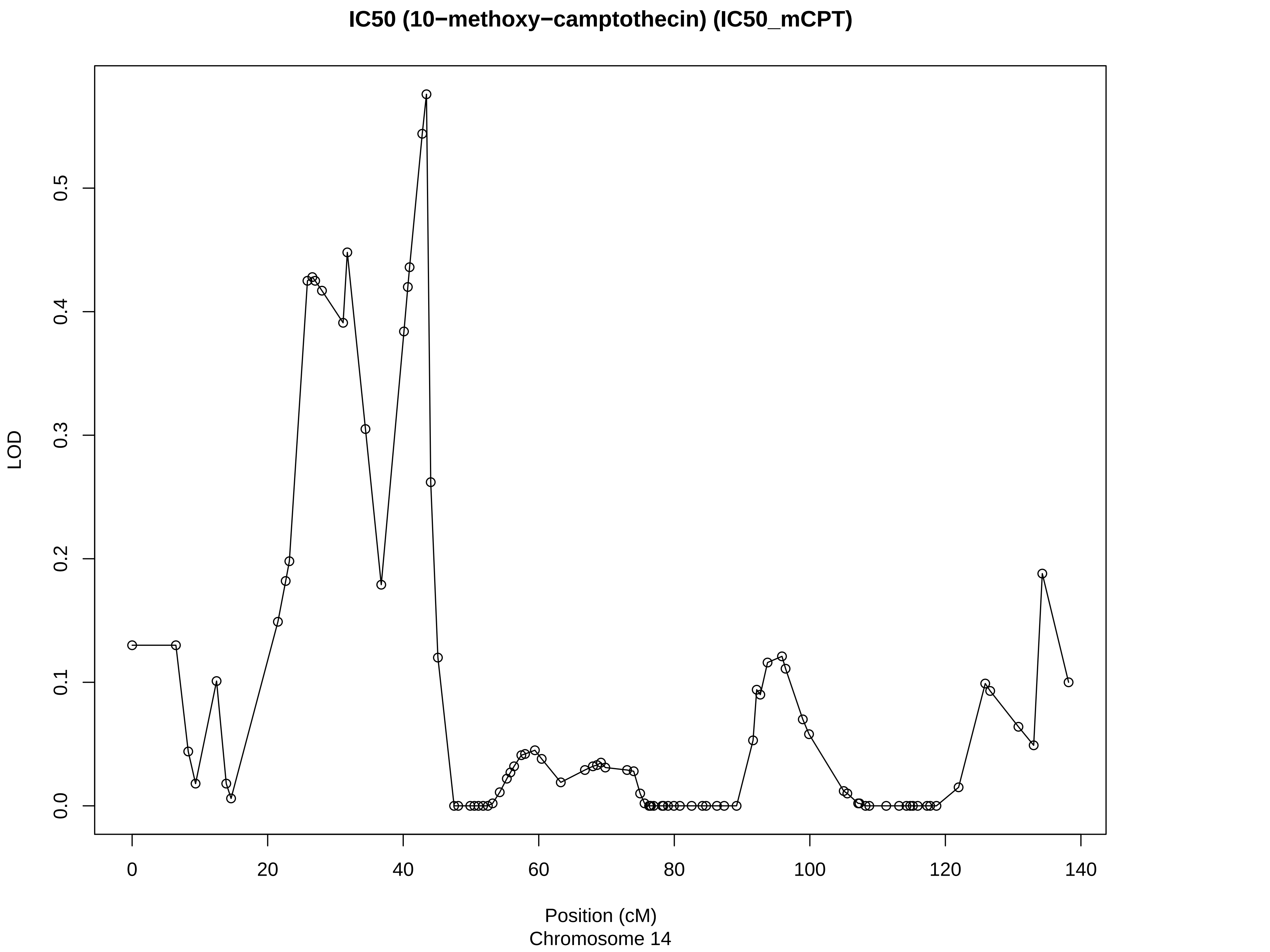

IC50 (10−methoxy−camptothecin) (IC50_mCPT)
LOD
0.0
0.1
0.2
0.3
0.4
0.5
0
20
40
60
80
100
120
140
Position (cM)
Chromosome 14

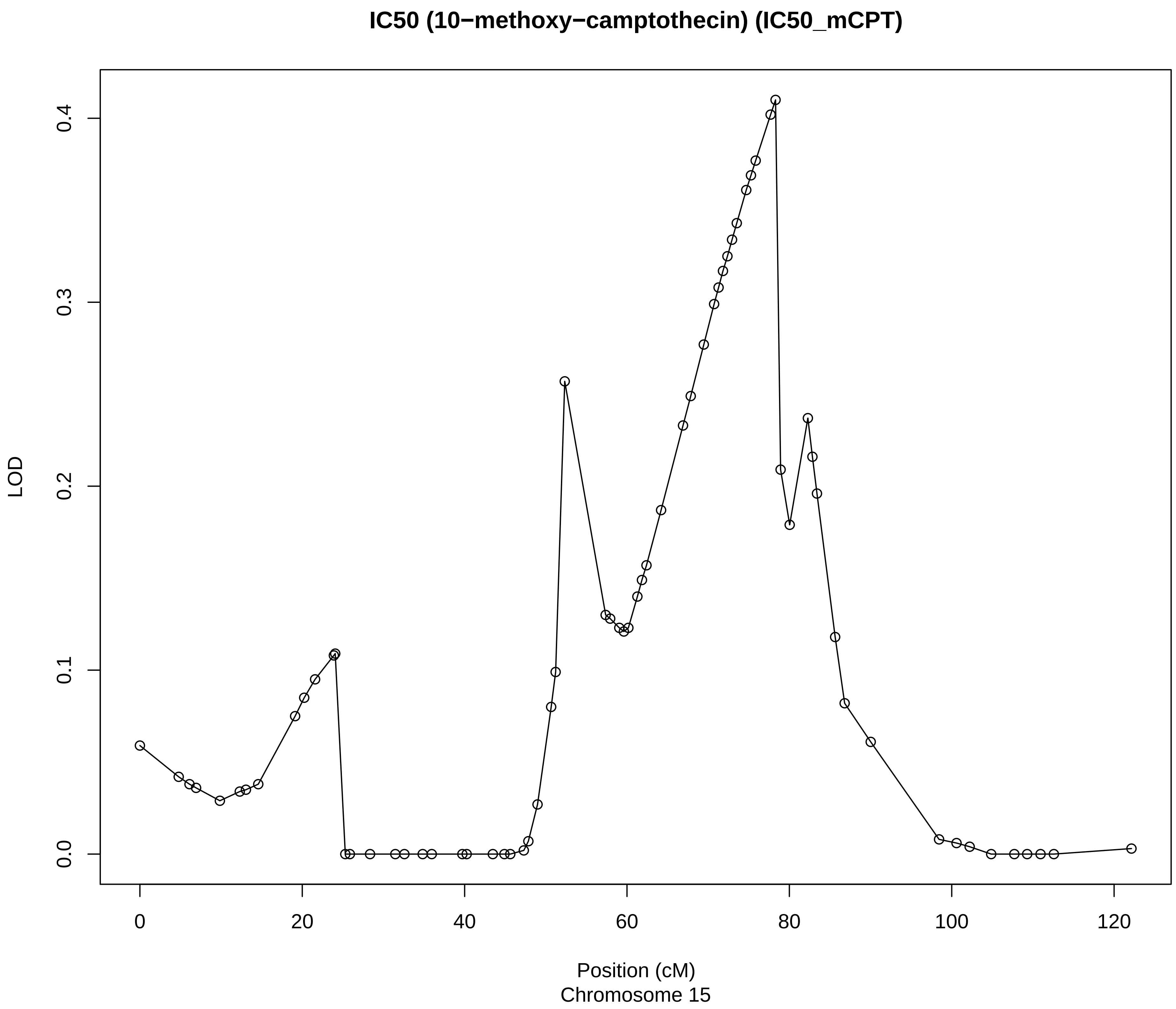
IC50 (10−methoxy−camptothecin) (IC50_mCPT)
LOD
0.0
0.1
0.2
0.3
0.4
0
20
40
60
80
100
120
Position (cM)
Chromosome 15

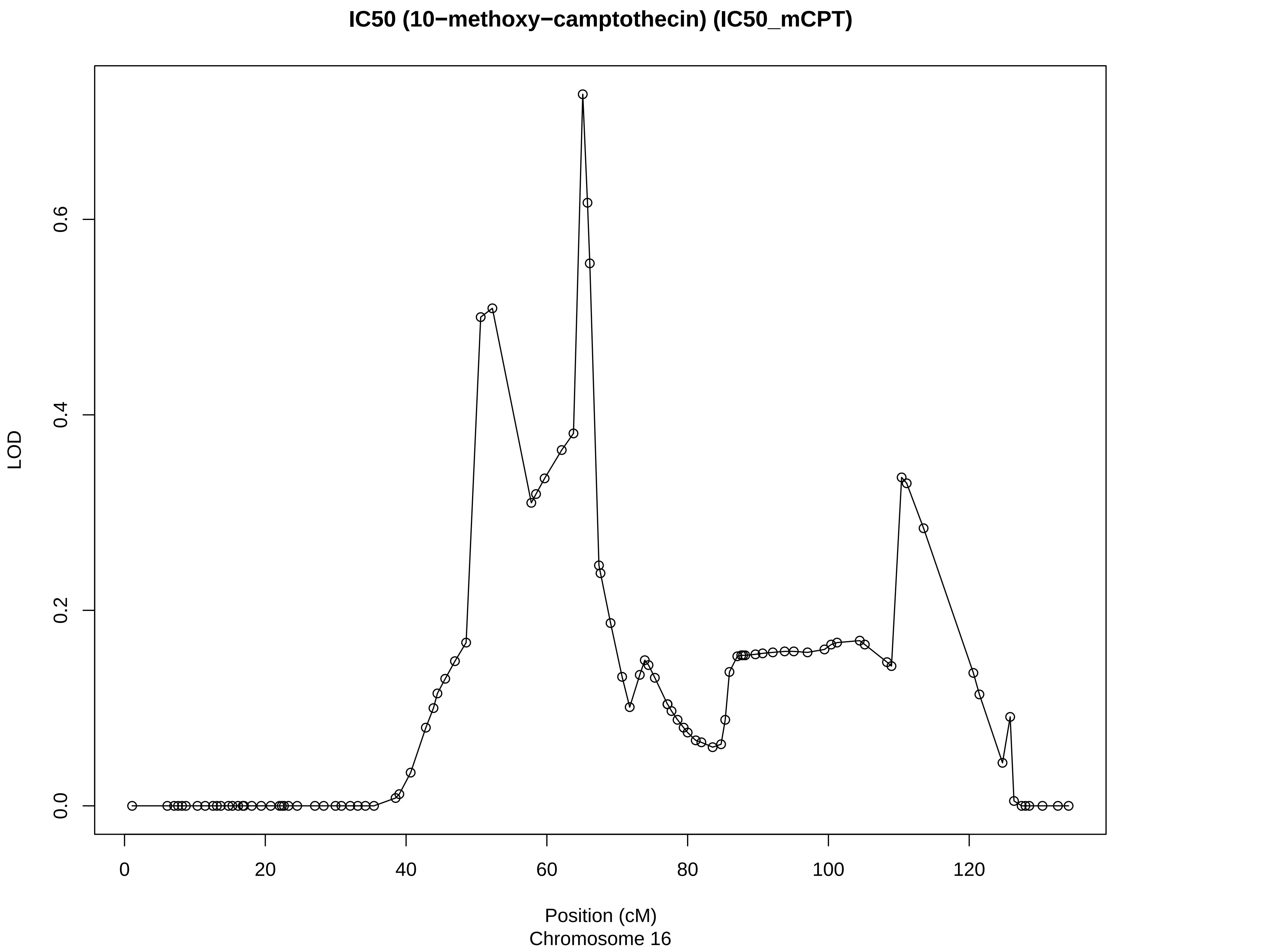

IC50 (10−methoxy−camptothecin) (IC50_mCPT)
LOD
0.0
0.2
0.4
0.6
0
20
40
60
80
100
120
Position (cM)
Chromosome 16

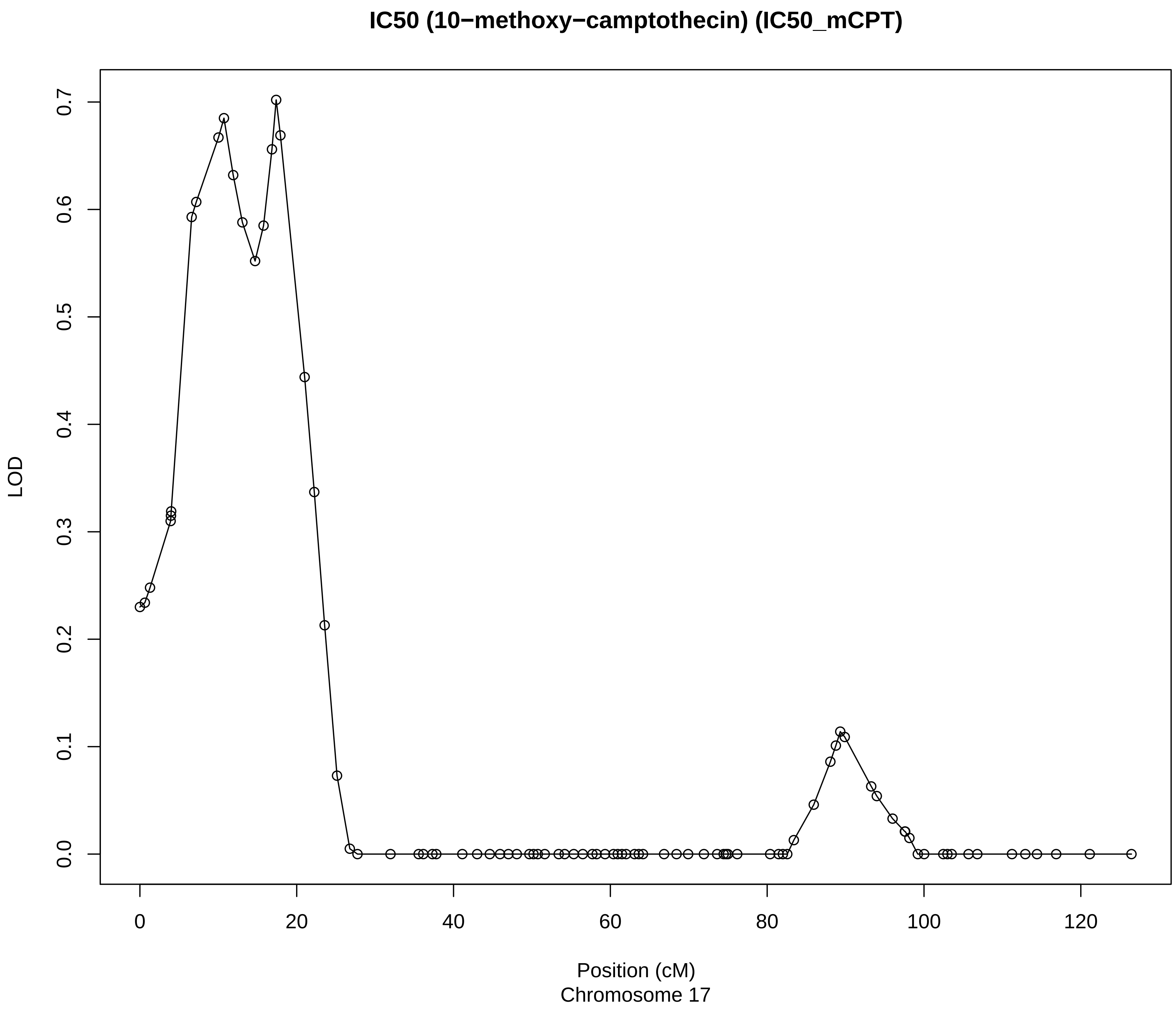
IC50 (10−methoxy−camptothecin) (IC50_mCPT)
LOD
0.0
0.1
0.2
0.3
0.4
0.5
0.6
0.7
0
20
40
60
80
100
120
Position (cM)
Chromosome 17

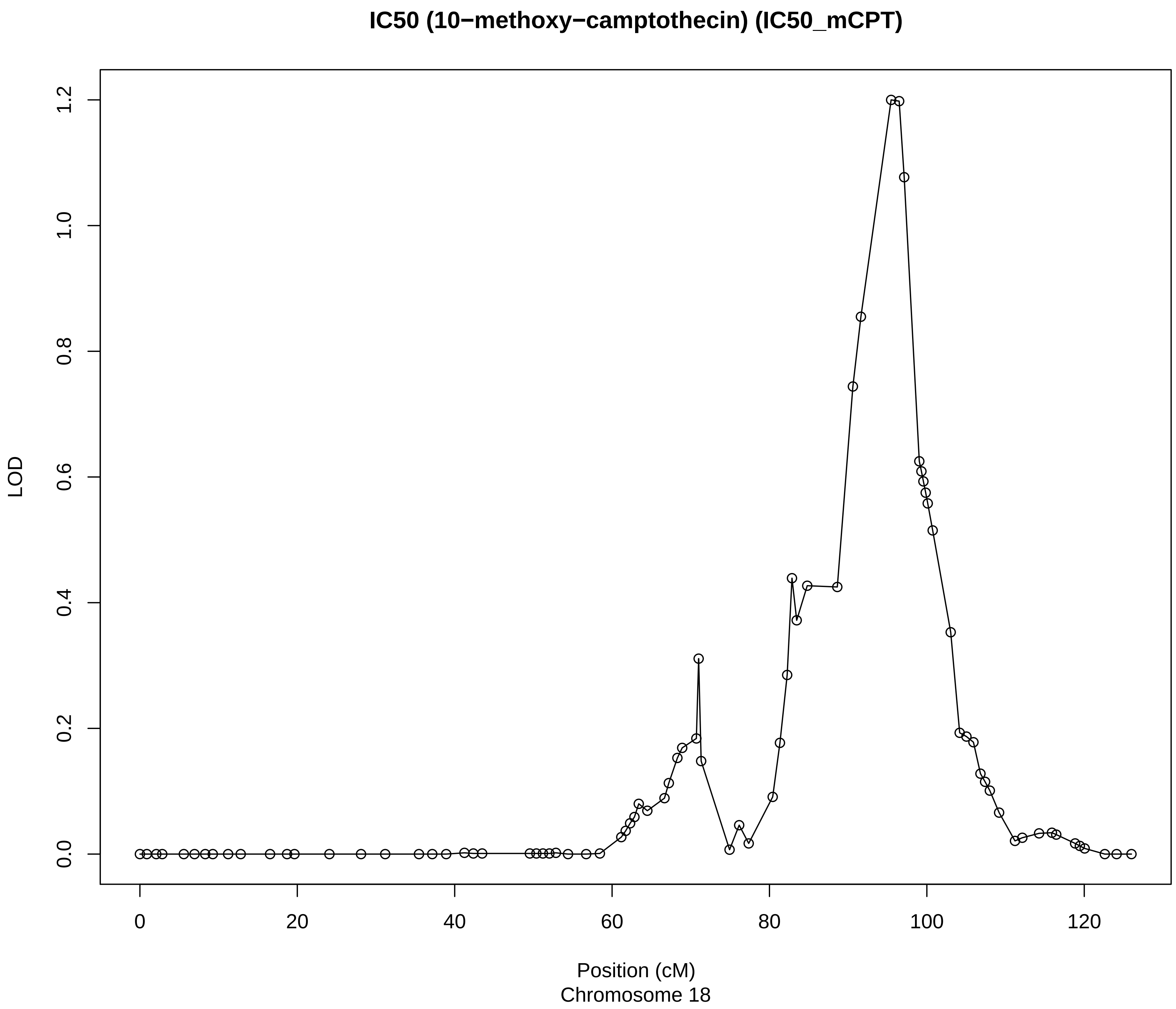
IC50 (10−methoxy−camptothecin) (IC50_mCPT)
LOD
0.0
0.2
0.4
0.6
0.8
1.0
1.2
0
20
40
60
80
100
120
Position (cM)
Chromosome 18

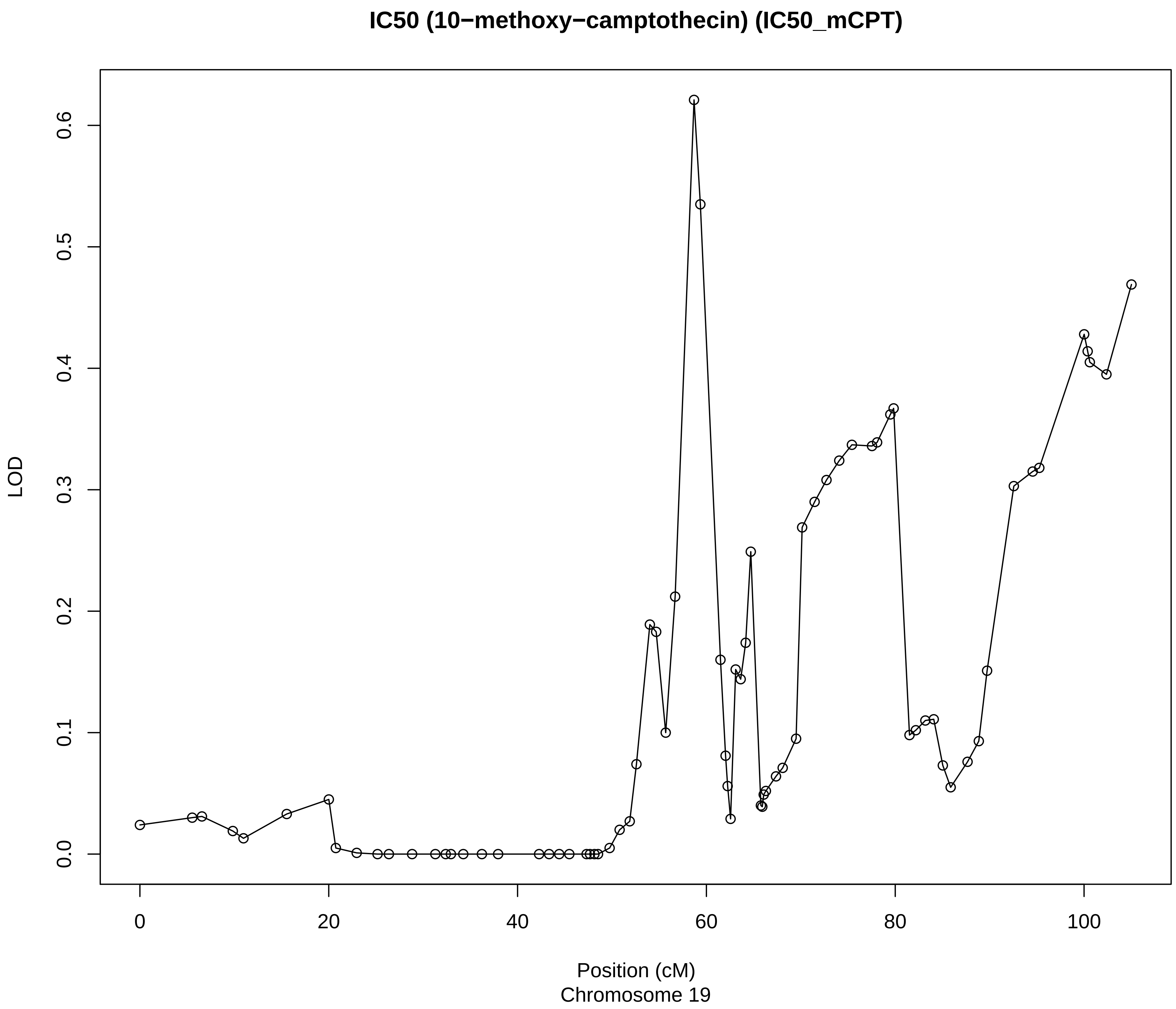
IC50 (10−methoxy−camptothecin) (IC50_mCPT)
LOD
0.0
0.1
0.2
0.3
0.4
0.5
0.6
0
20
40
60
80
100
Position (cM)
Chromosome 19

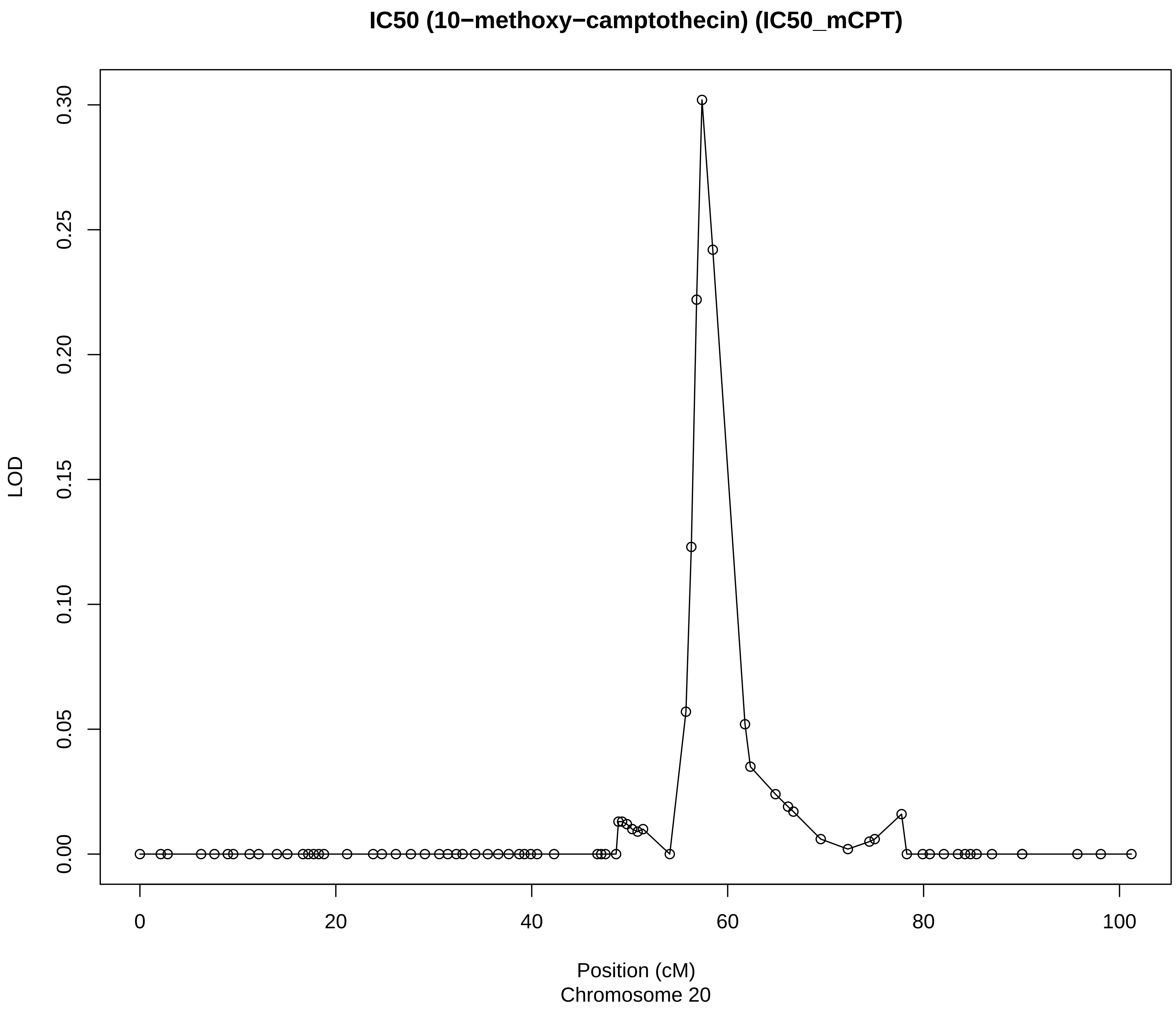

IC50 (10−methoxy−camptothecin) (IC50_mCPT)
LOD
0.00
0.05
0.10
0.15
0.20
0.25
0.30
0
20
40
60
80
100
Position (cM)
Chromosome 20

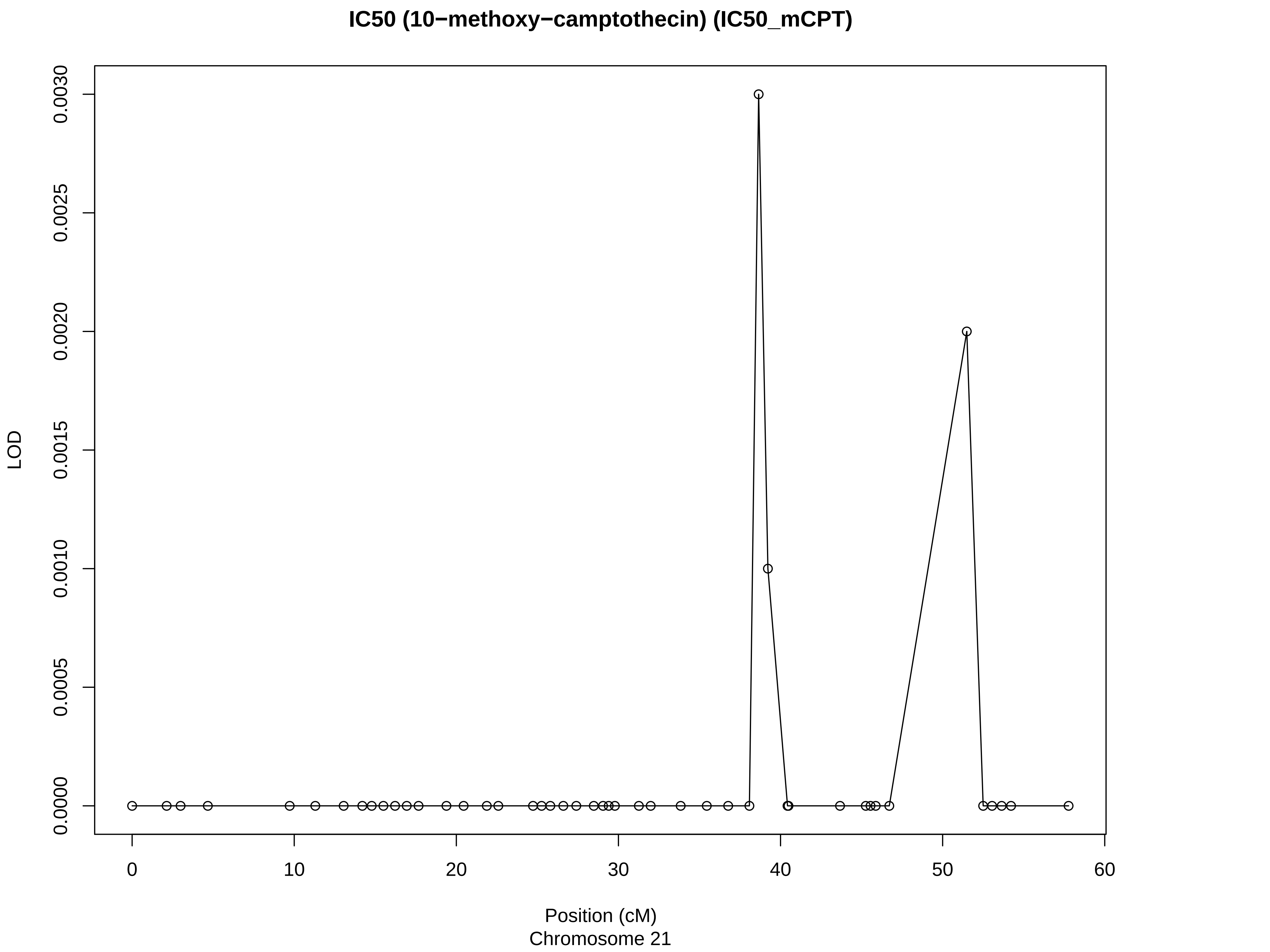

IC50 (10−methoxy−camptothecin) (IC50_mCPT)
LOD
0.0000
0.0005
0.0010
0.0015
0.0020
0.0025
0.0030
0
10
20
30
40
50
60
Position (cM)
Chromosome 21

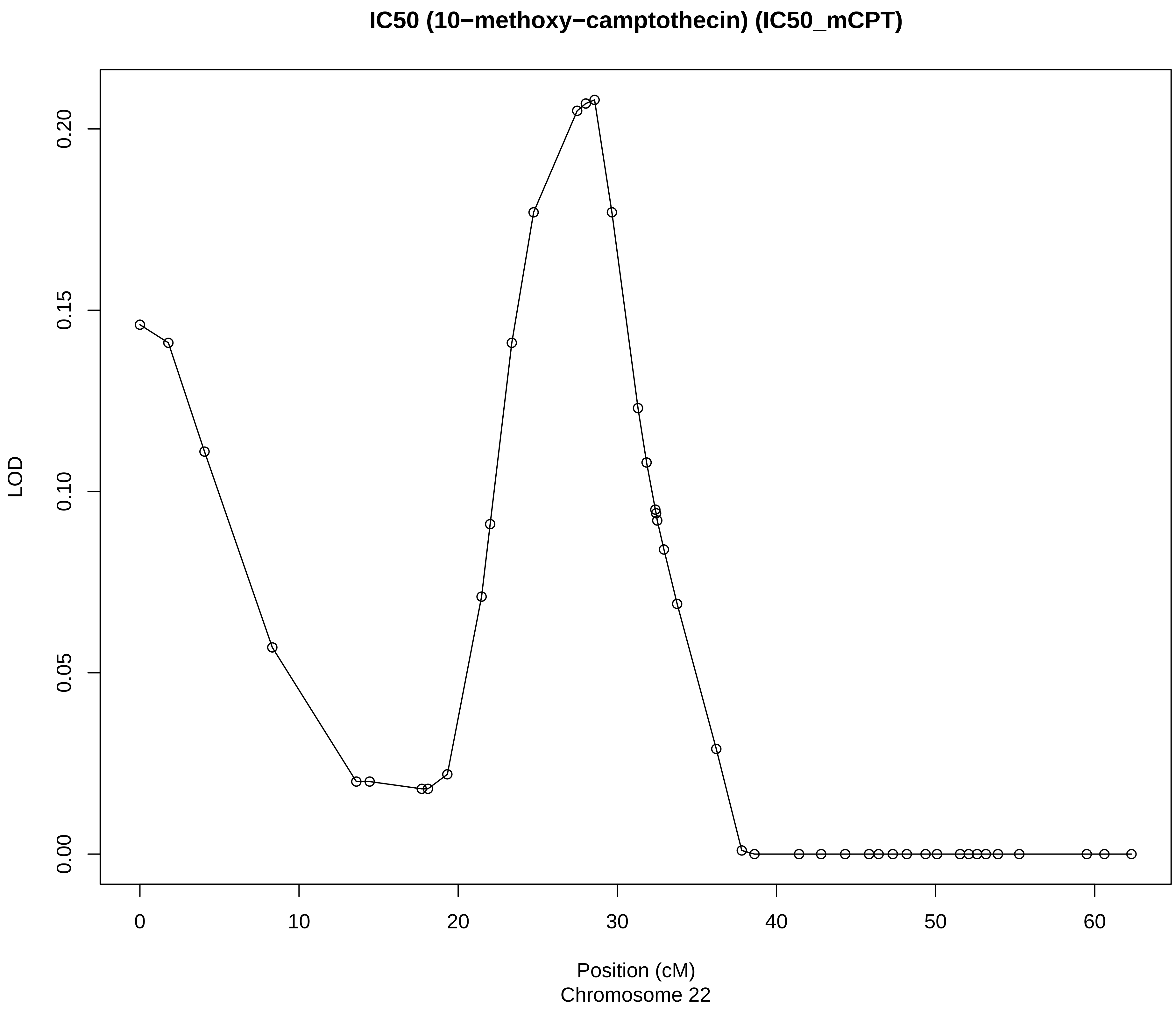
IC50 (10−methoxy−camptothecin) (IC50_mCPT)
LOD
0.00
0.05
0.10
0.15
0.20
0
10
20
30
40
50
60
Position (cM)
Chromosome 22

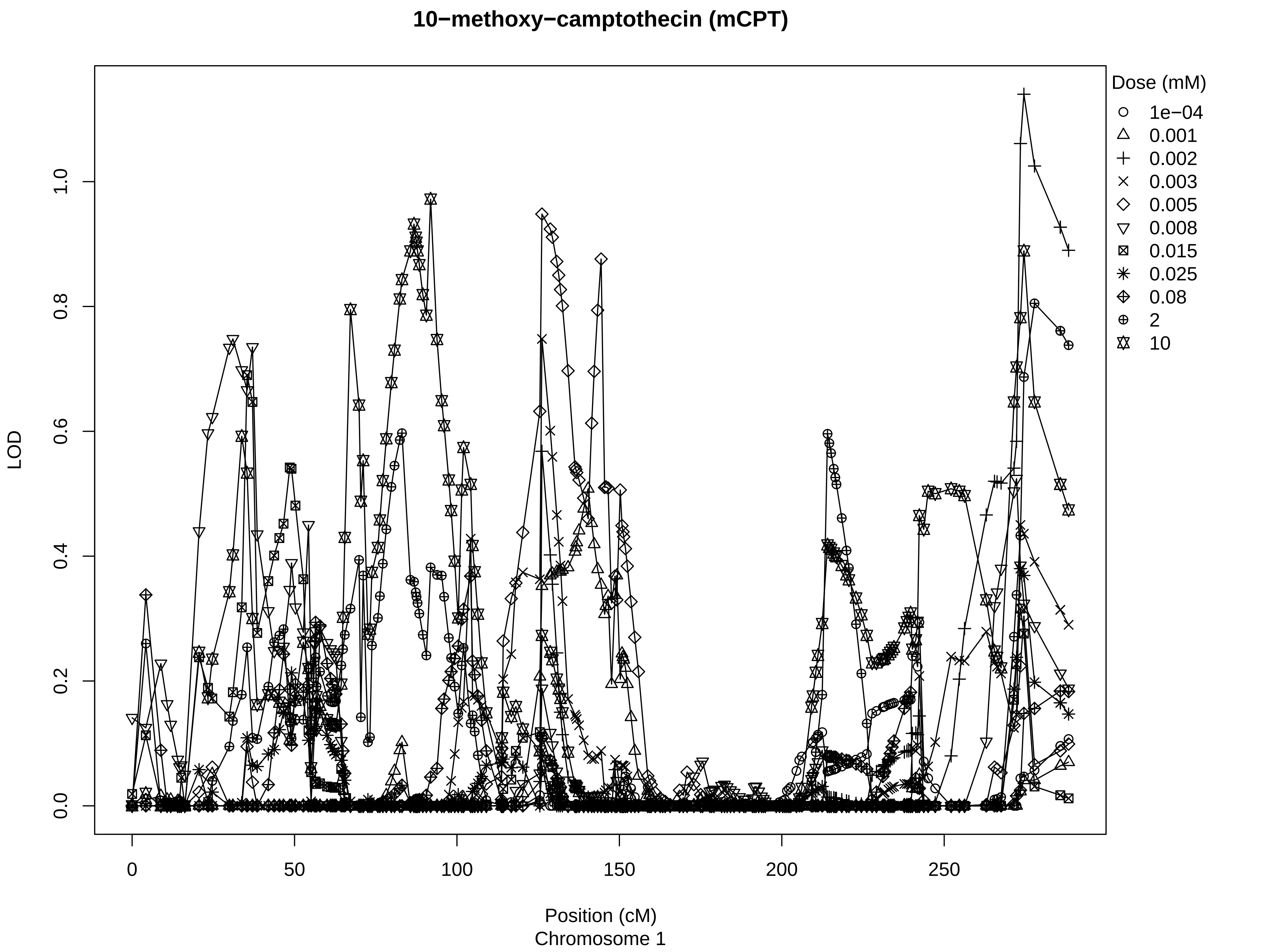

10−methoxy−camptothecin (mCPT)
LOD
0.0
0.2
0.4
0.6
0.8
1.0
0
50
100
150
200
250
Position (cM)
Chromosome 1
Dose (mM)
1e−04
0.001
0.002
0.003
0.005
0.008
0.015
0.025
0.08
2
10

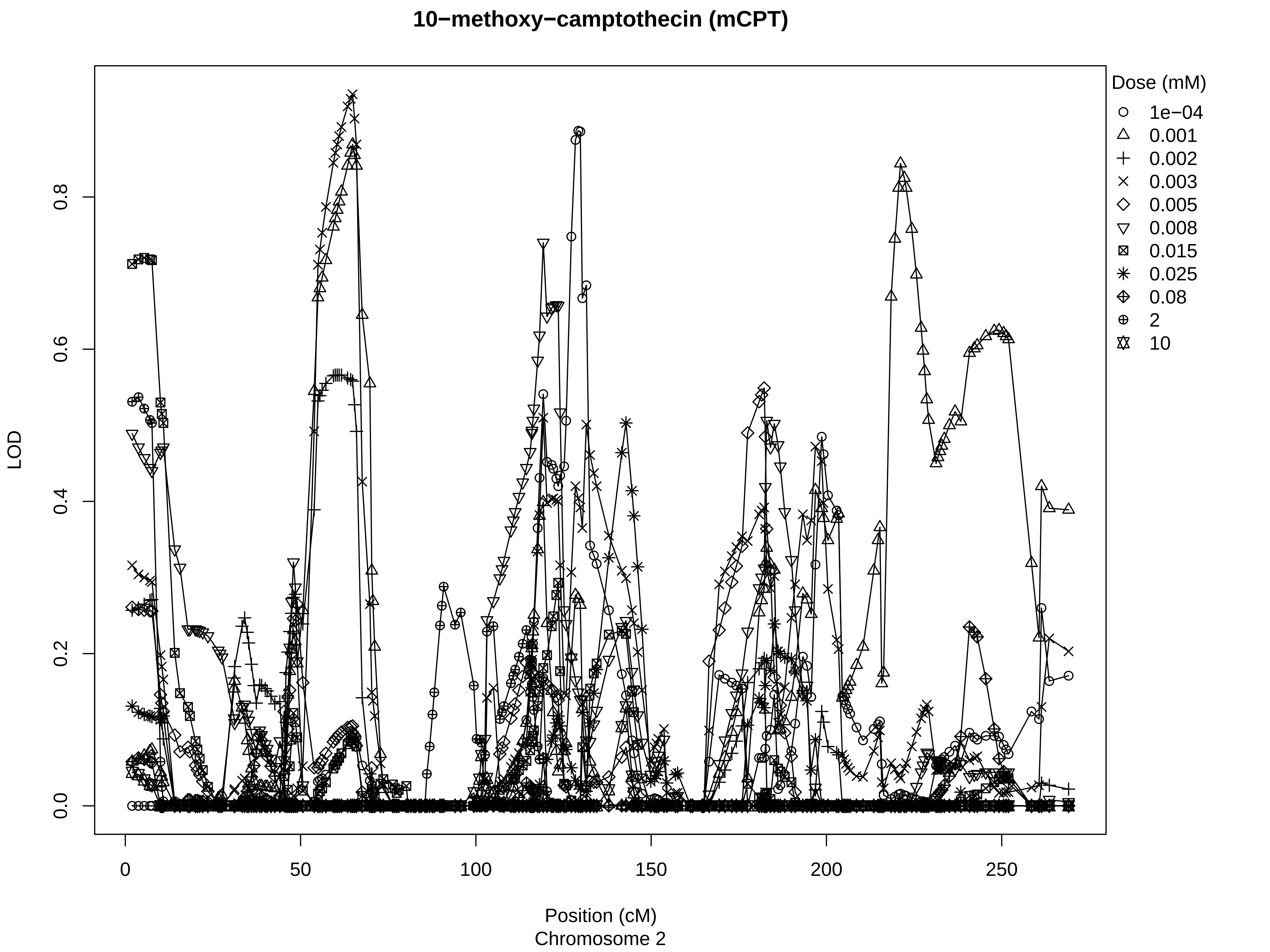
10−methoxy−camptothecin (mCPT)
Dose (mM)
1e−04
0.001
0.002
0.003
0.005
0.008
0.015
0.025
0.08
2
10
LOD
0.0
0.2
0.4
0.6
0.8
0
50
100
150
200
250
Position (cM)
Chromosome 2

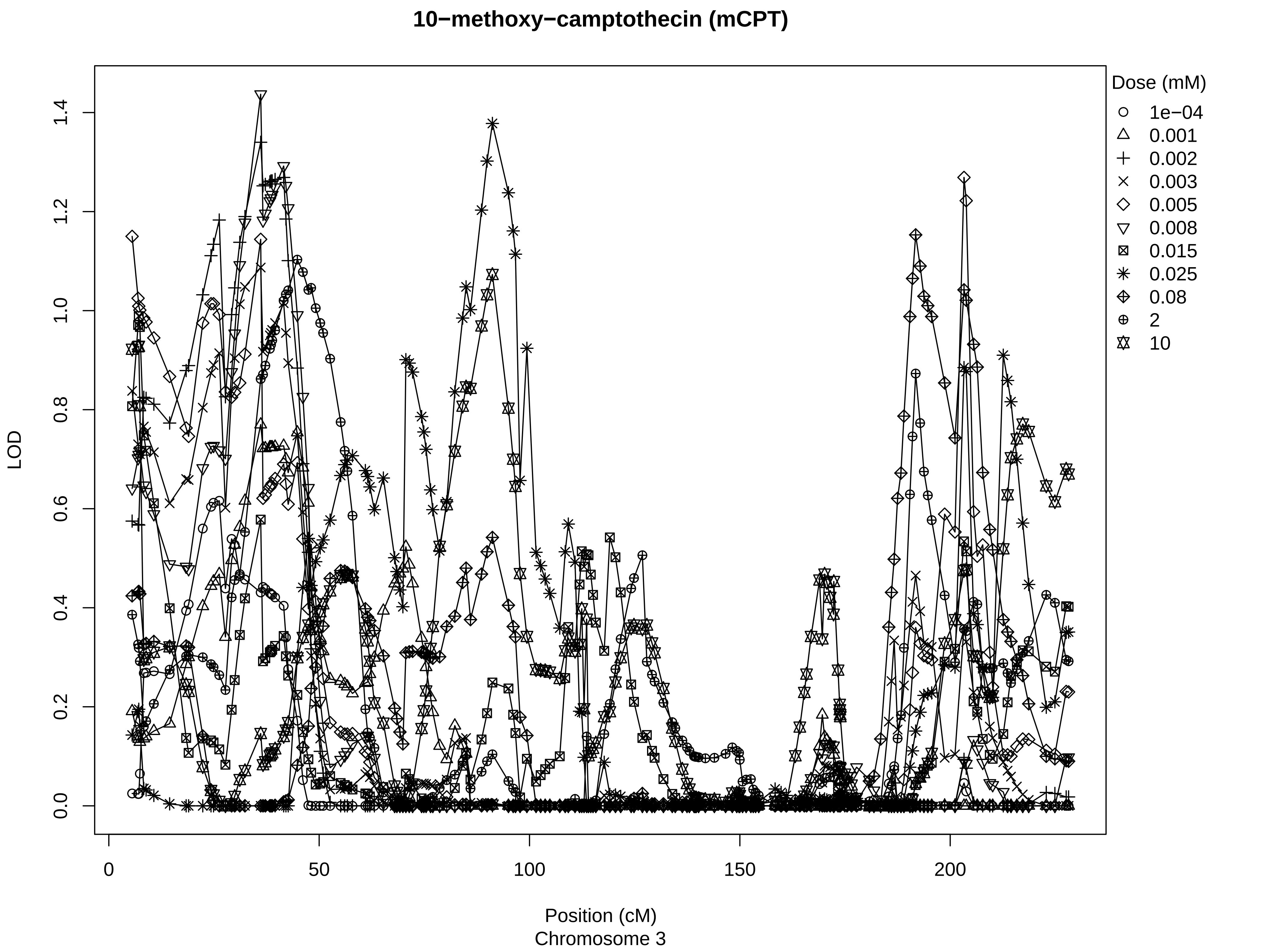
10−methoxy−camptothecin (mCPT)
LOD
Position (cM)
Chromosome 3
Dose (mM)
1e−04
0.001
0.002
0.003
0.005
0.008
0.015
0.025
0.08
2
10
0.0
0.2
0.4
0.6
0.8
1.0
1.2
1.4
0
50
100
150
200

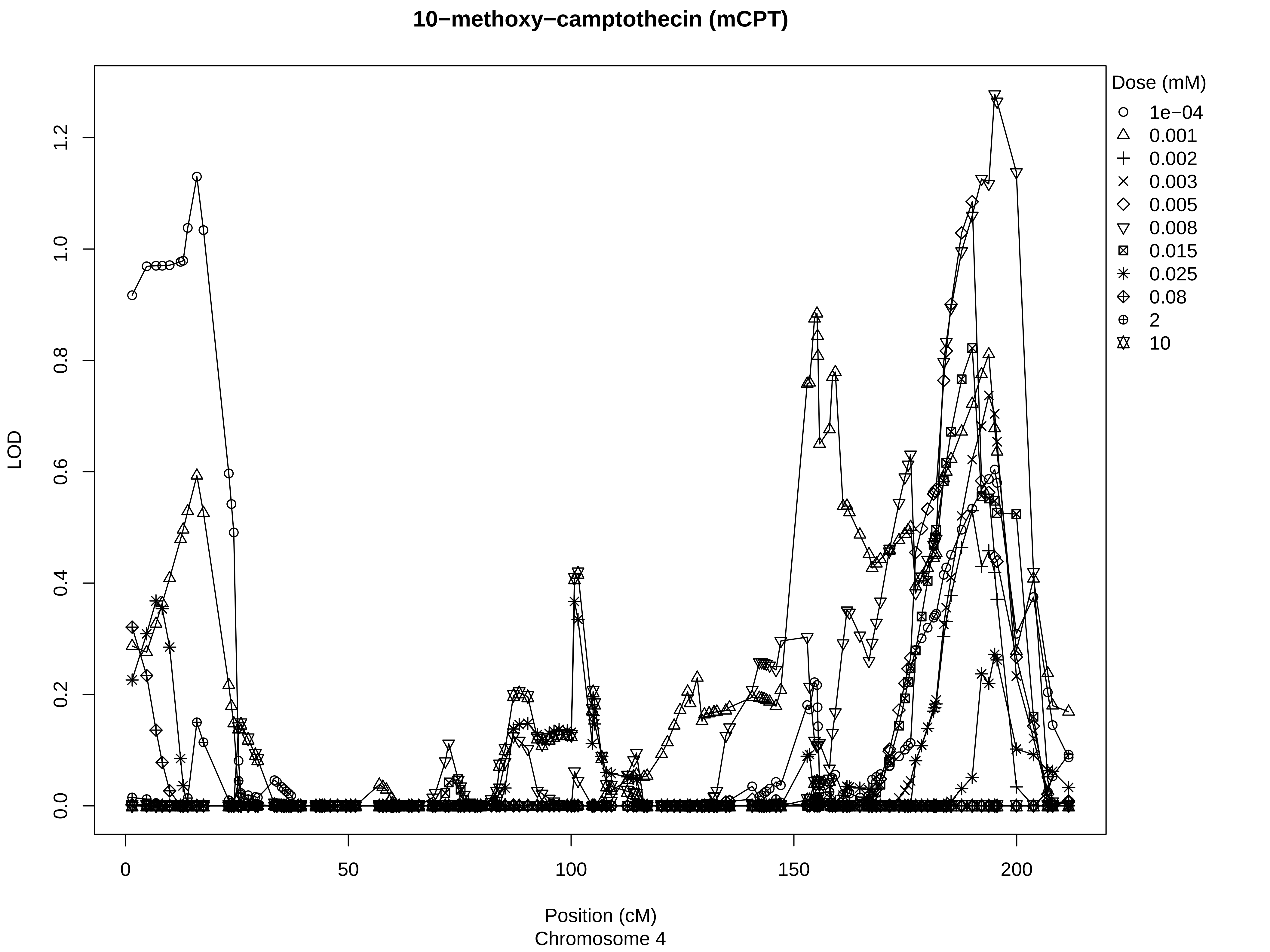
10−methoxy−camptothecin (mCPT)
LOD
0.0
0.2
0.4
0.6
0.8
1.0
1.2
Position (cM)
Chromosome 4
0
50
100
150
200
Dose (mM)
1e−04
0.001
0.002
0.003
0.005
0.008
0.015
0.025
0.08
2
10

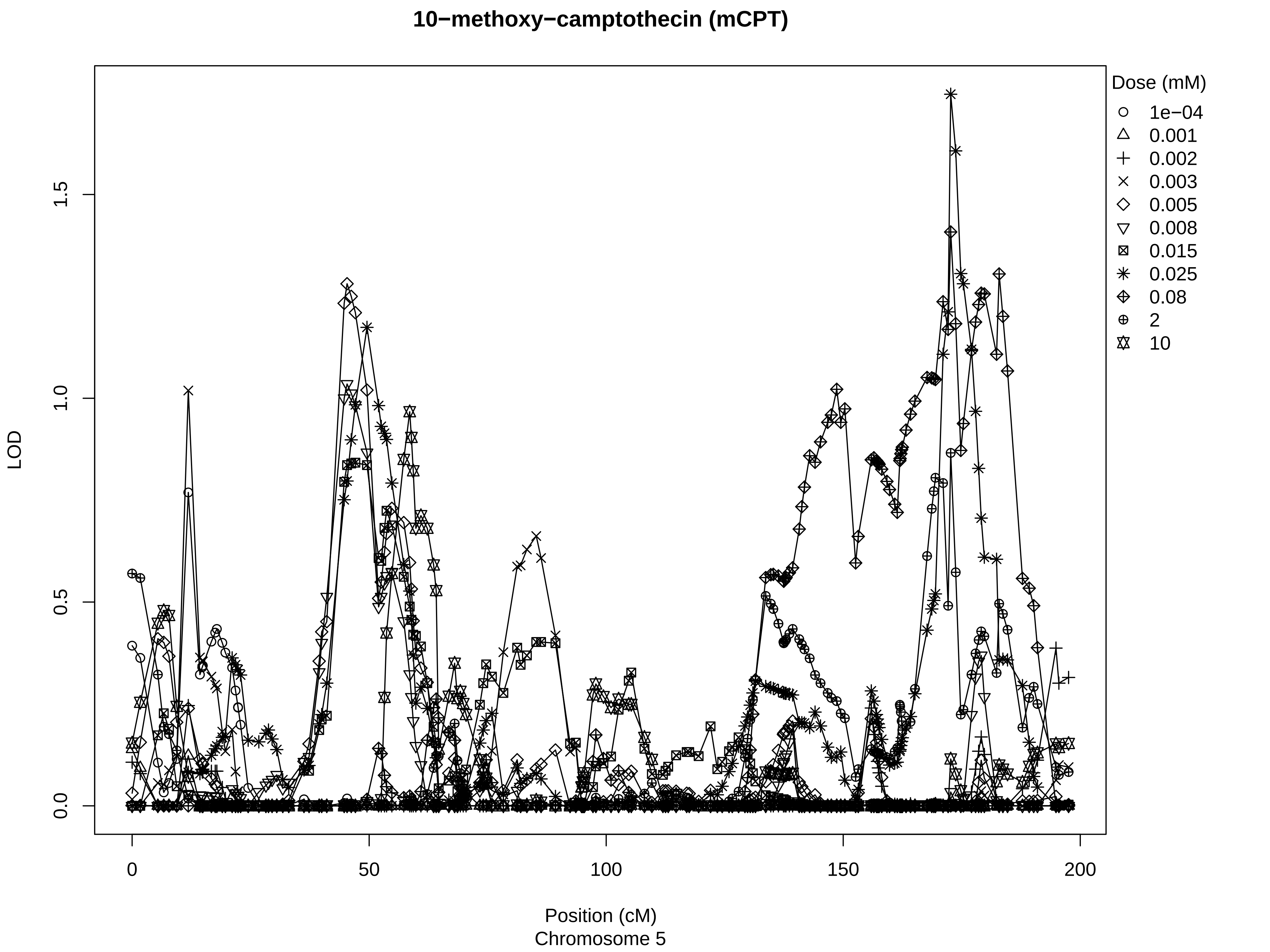
10−methoxy−camptothecin (mCPT)
Dose (mM)
1e−04
0.001
0.002
0.003
0.005
0.008
0.015
0.025
0.08
2
10
LOD
0.0
0.5
1.0
1.5
0
50
100
150
200
Position (cM)
Chromosome 5

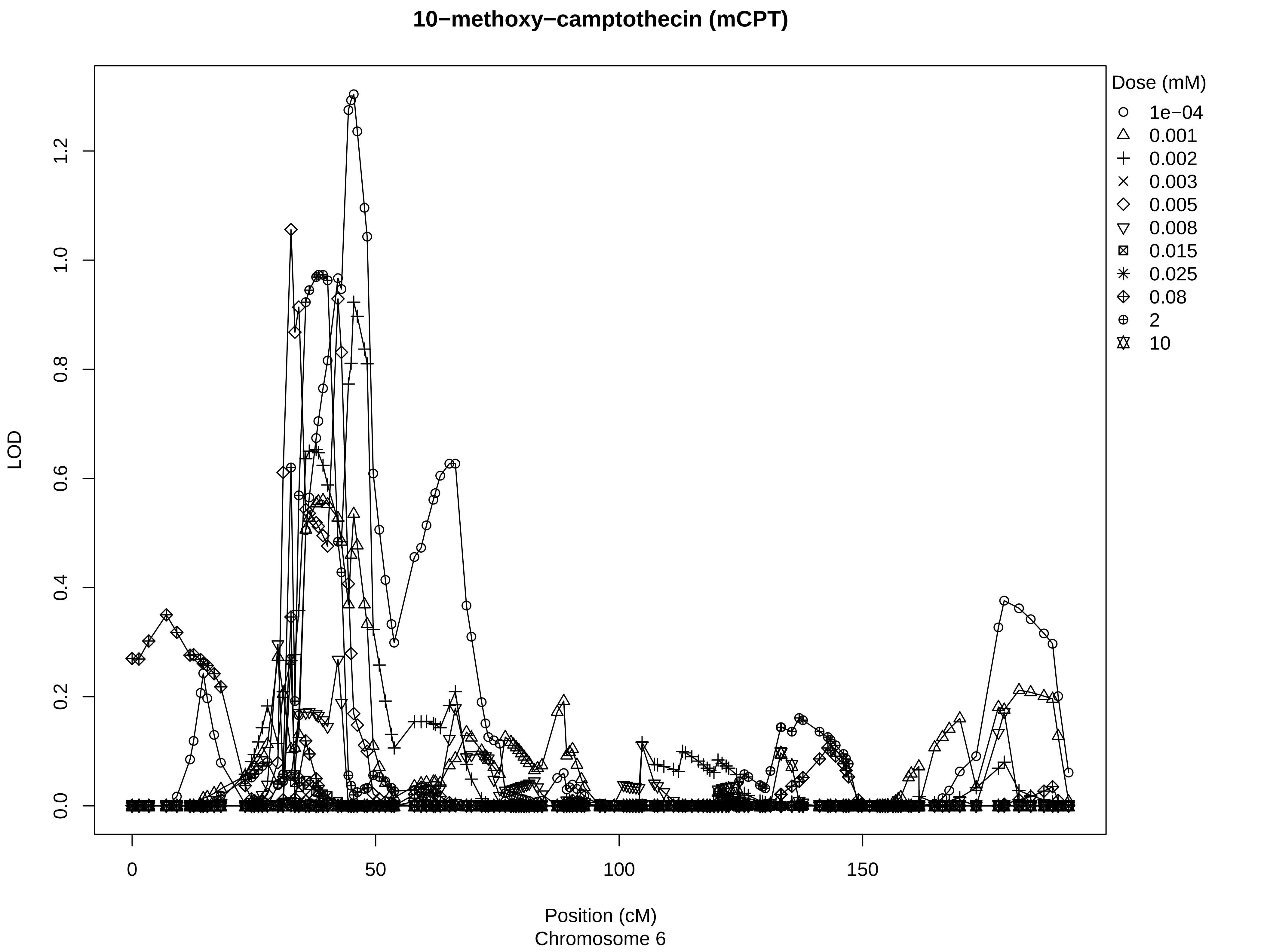
10−methoxy−camptothecin (mCPT)
LOD
Position (cM)
Chromosome 6
Dose (mM)
1e−04
0.001
0.002
0.003
0.005
0.008
0.015
0.025
0.08
2
10

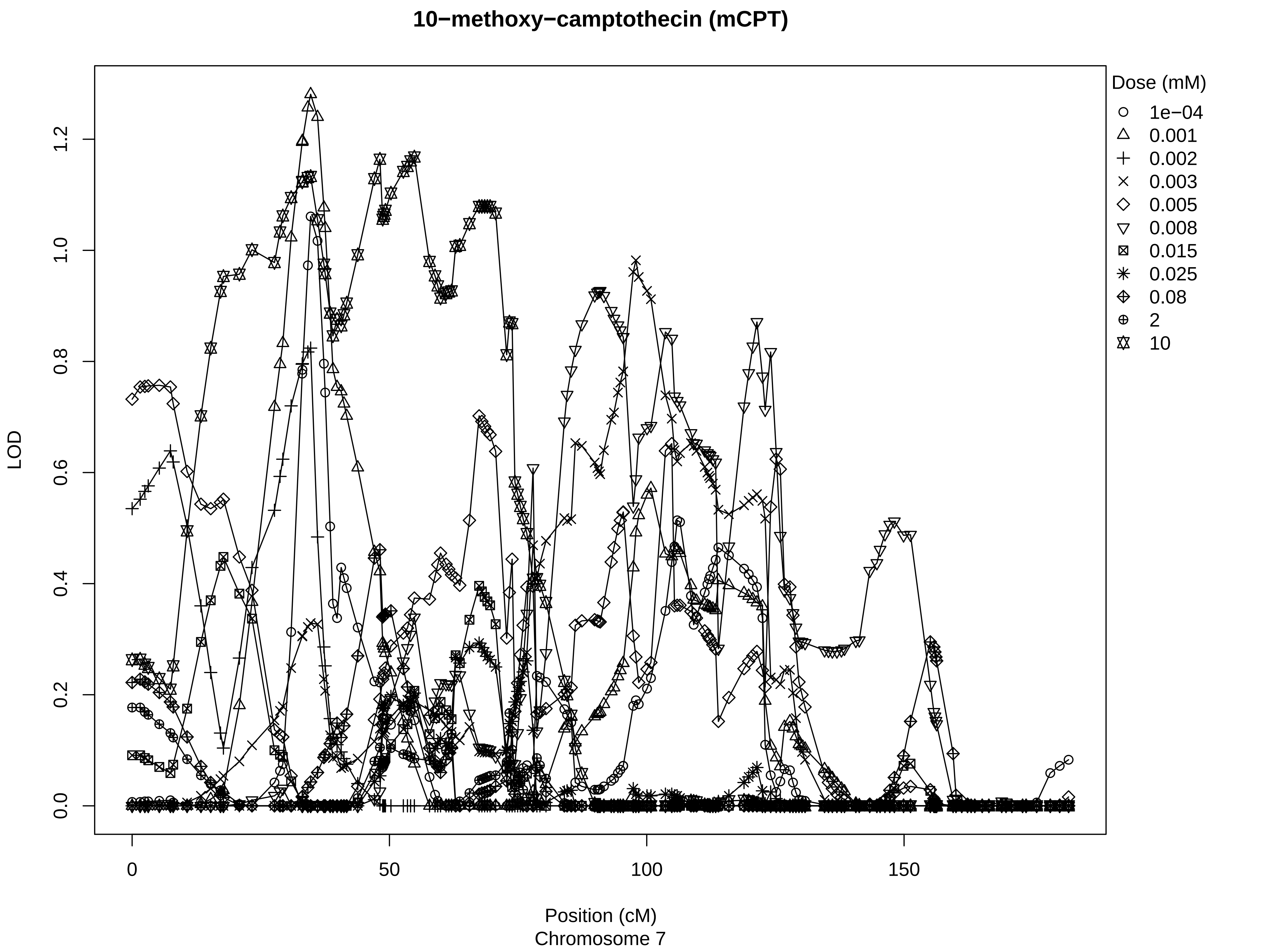

10−methoxy−camptothecin (mCPT)
LOD
Position (cM)
Chromosome 7
0.0
0.2
0.4
0.6
0.8
1.0
1.2
0
50
100
150
Dose (mM)
1e−04
0.001
0.002
0.003
0.005
0.008
0.015
0.025
0.08
2
10

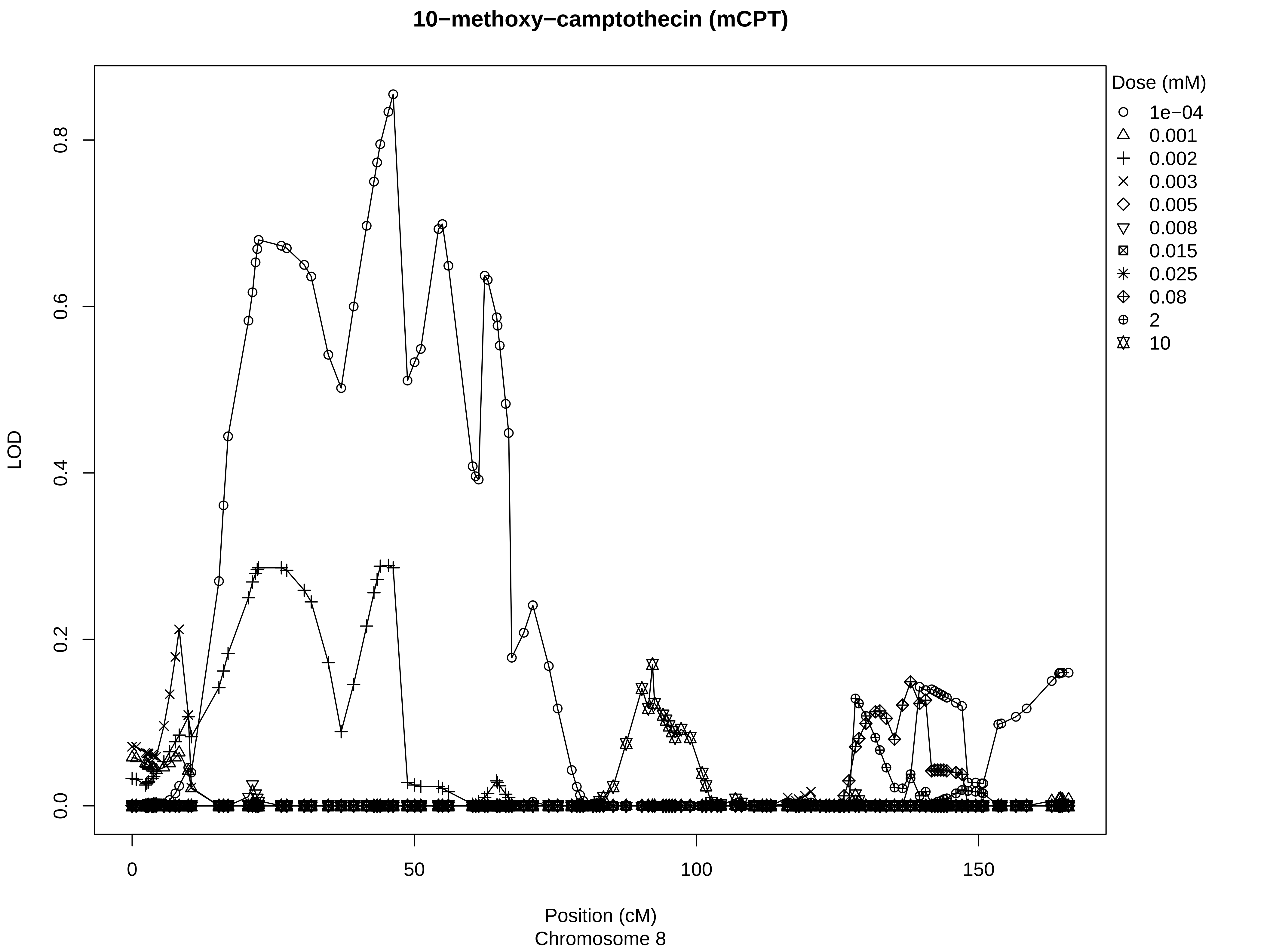
10−methoxy−camptothecin (mCPT)
LOD
0.0
0.2
0.4
0.6
0.8
0
50
100
150
Position (cM)
Chromosome 8
Dose (mM)
1e−04
0.001
0.002
0.003
0.005
0.008
0.015
0.025
0.08
2
10

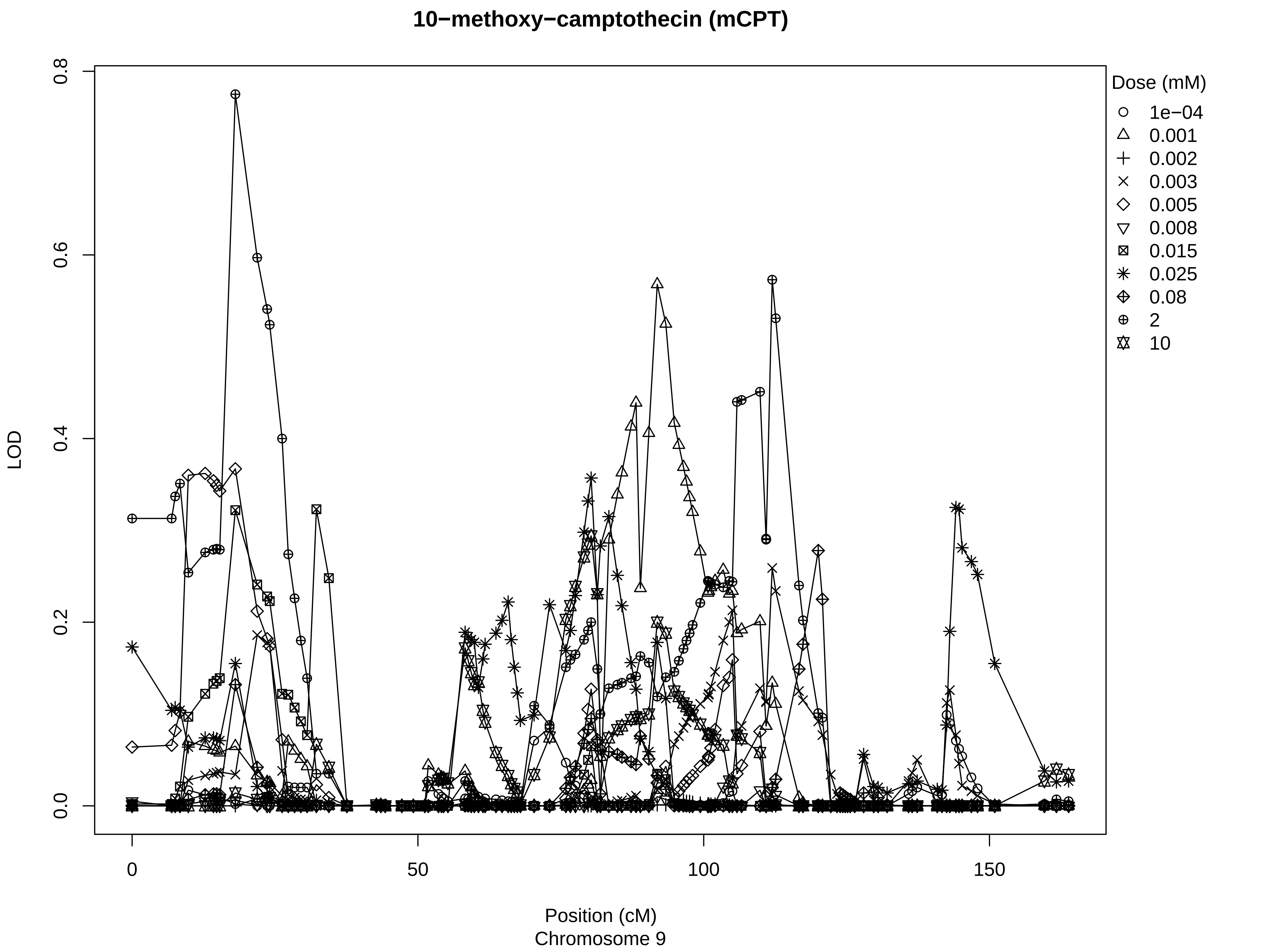

10−methoxy−camptothecin (mCPT)
LOD
0.0
0.2
0.4
0.6
0.8
0
50
100
150
Position (cM)
Chromosome 9
Dose (mM)
1e−04
0.001
0.002
0.003
0.005
0.008
0.015
0.025
0.08
2
10

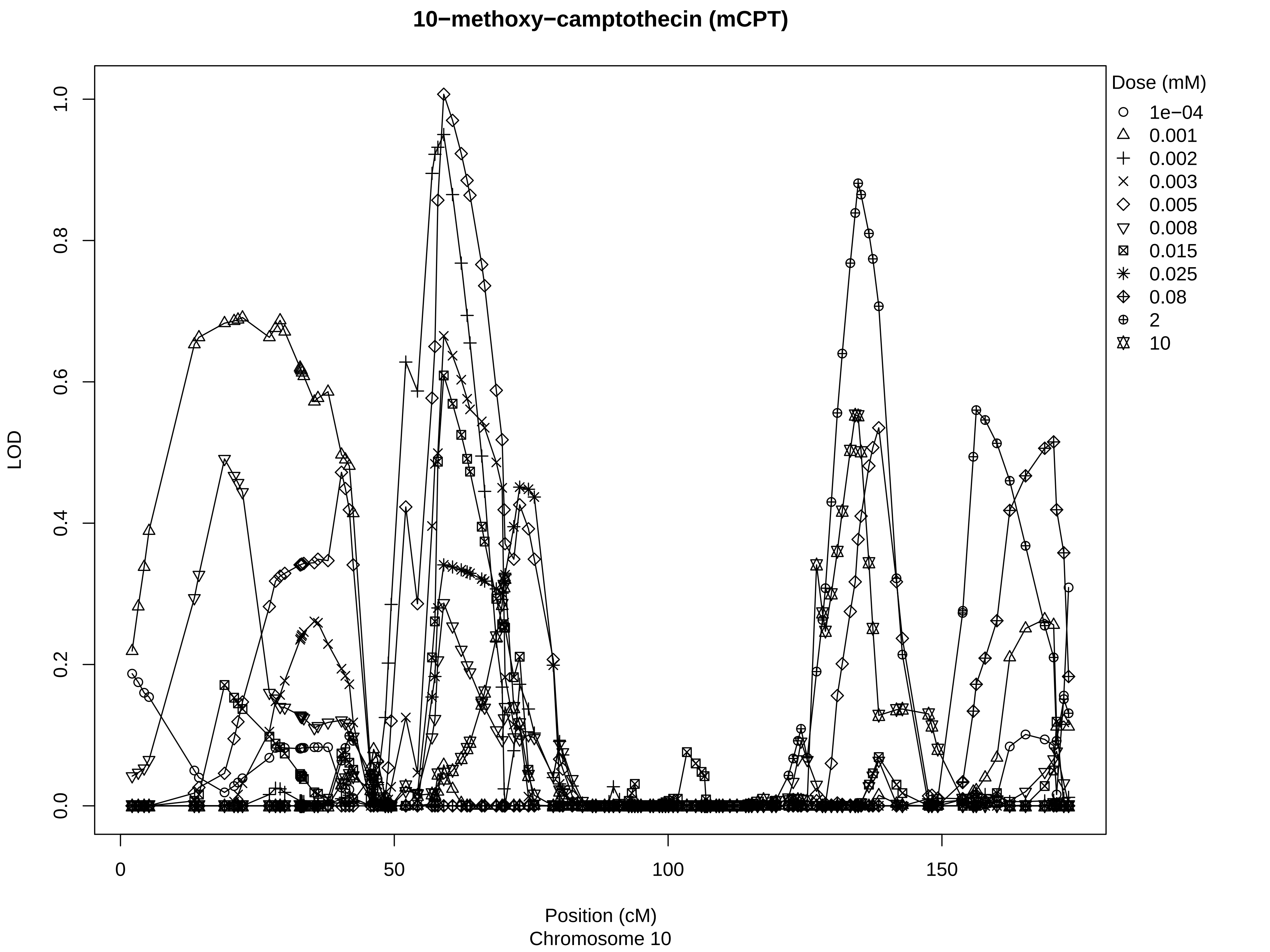
10−methoxy−camptothecin (mCPT)
LOD
0.0
0.2
0.4
0.6
0.8
1.0
0
50
100
150
Position (cM)
Chromosome 10
Dose (mM)
1e−04
0.001
0.002
0.003
0.005
0.008
0.015
0.025
0.08
2
10

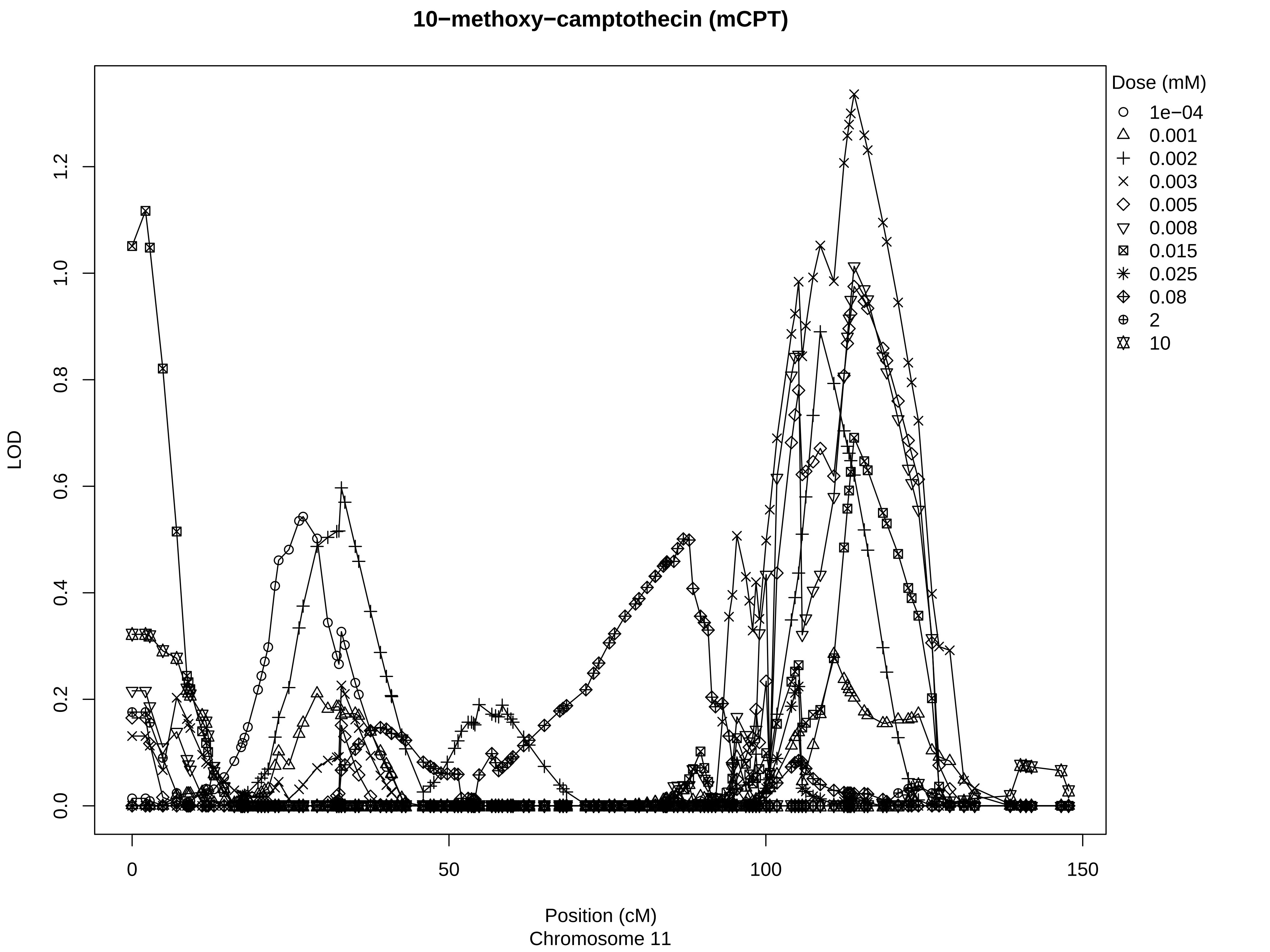

10−methoxy−camptothecin (mCPT)
LOD
0.0
0.2
0.4
0.6
0.8
1.0
1.2
0
50
100
150
Position (cM)
Chromosome 11
Dose (mM)
1e−04
0.001
0.002
0.003
0.005
0.008
0.015
0.025
0.08
2
10

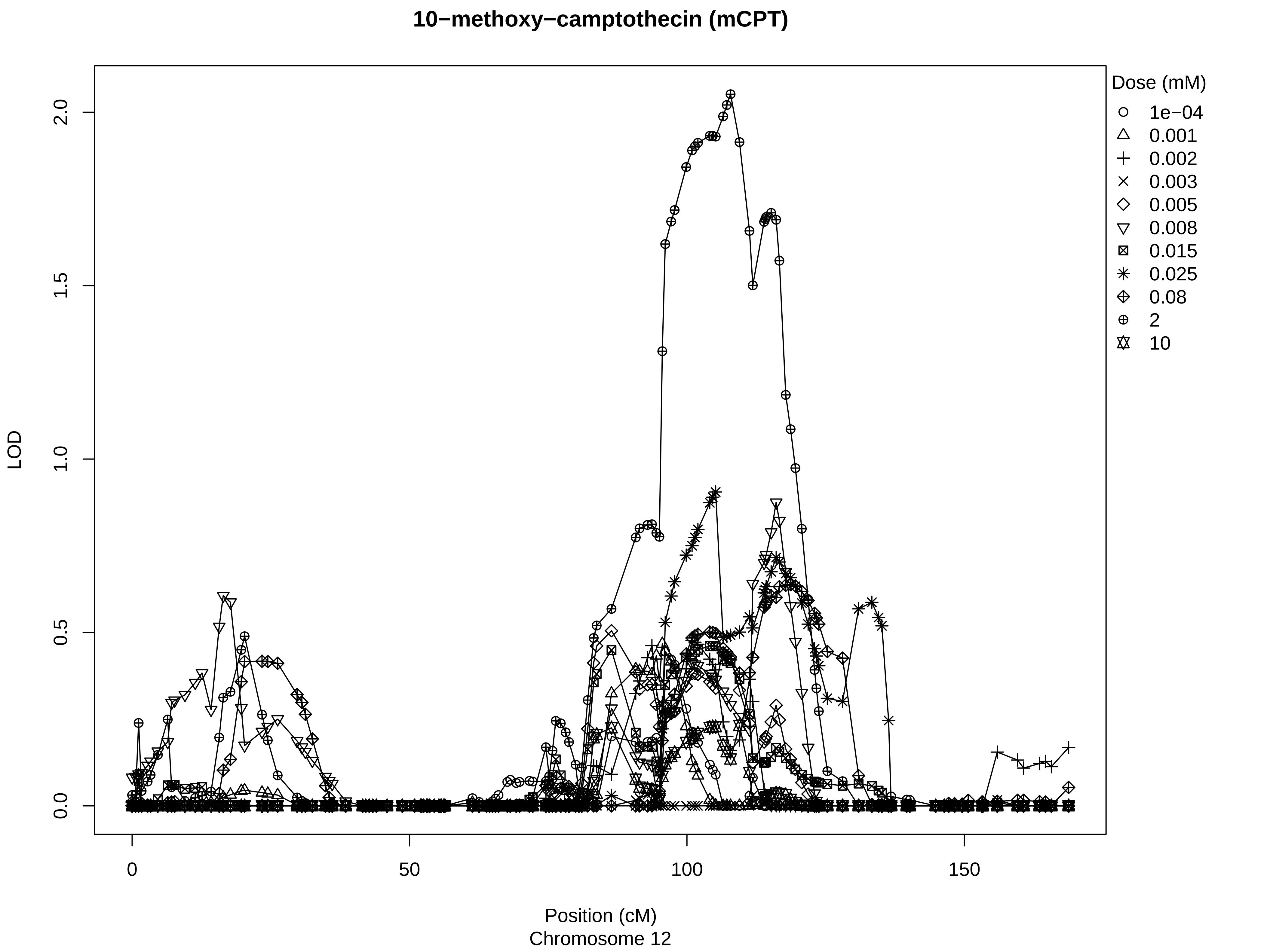
10−methoxy−camptothecin (mCPT)
LOD
0.0
0.5
1.0
1.5
2.0
0
50
100
150
Position (cM)
Chromosome 12
Dose (mM)
1e−04
0.001
0.002
0.003
0.005
0.008
0.015
0.025
0.08
2
10

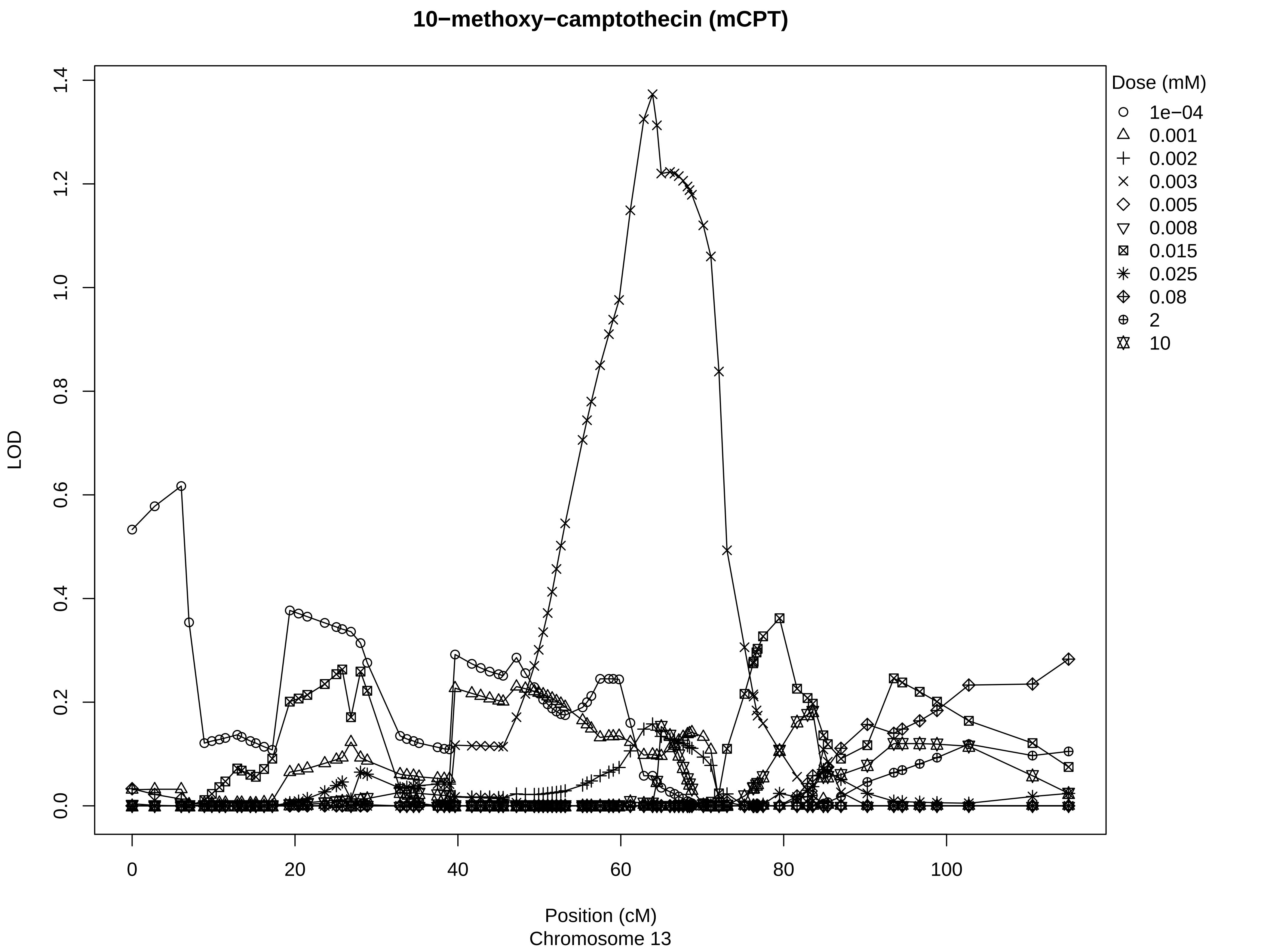
10−methoxy−camptothecin (mCPT)
Dose (mM)
1e−04
0.001
0.002
0.003
0.005
0.008
0.015
0.025
0.08
2
10
LOD
0.0
0.2
0.4
0.6
0.8
1.0
1.2
1.4
0
20
40
60
80
100
Position (cM)
Chromosome 13

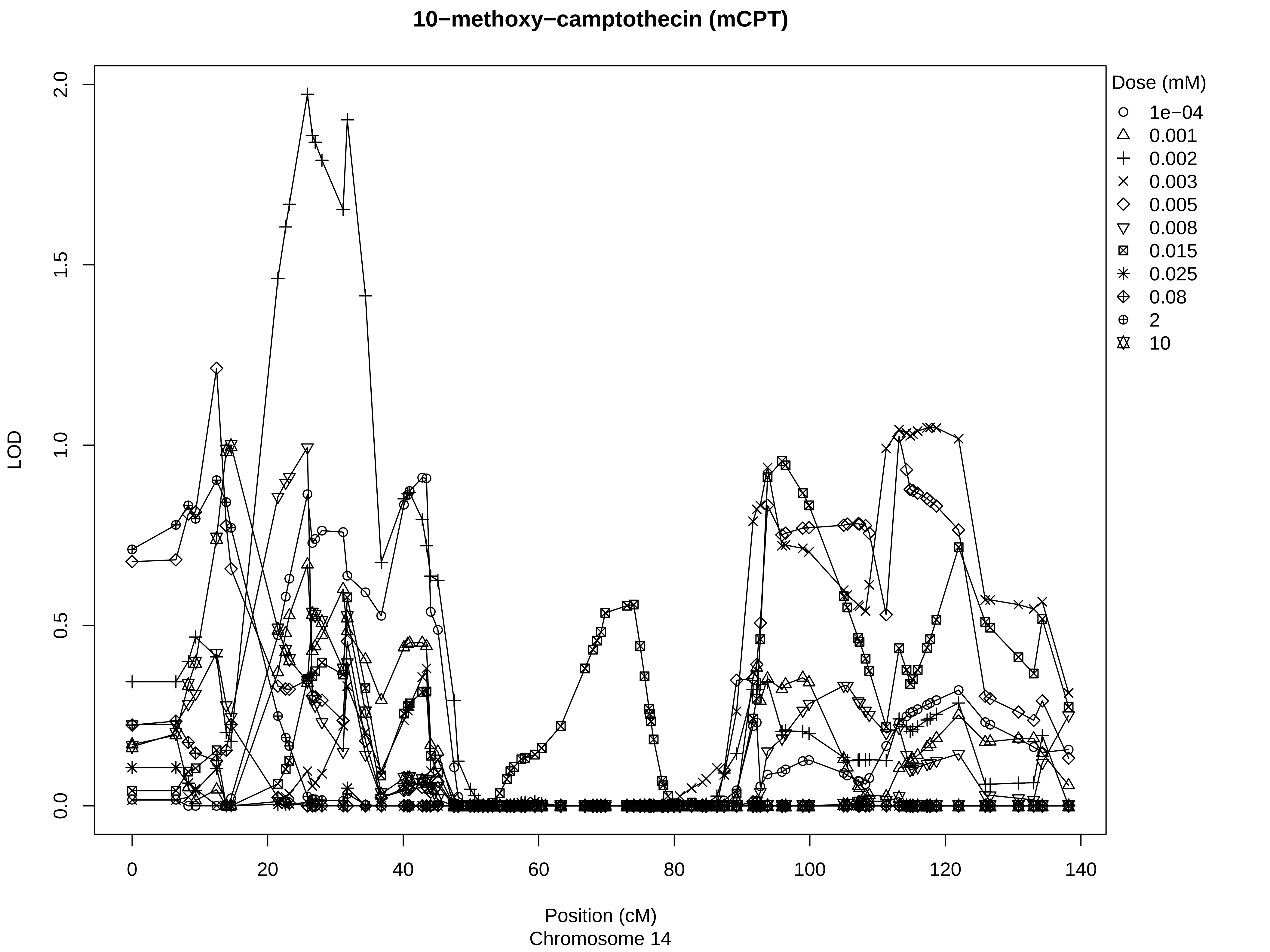

10−methoxy−camptothecin (mCPT)
LOD
0.0
0.5
1.0
1.5
2.0
Position (cM)
Chromosome 14
0
20
40
60
80
100
120
140
Dose (mM)
1e−04
0.001
0.002
0.003
0.005
0.008
0.015
0.025
0.08
2
10

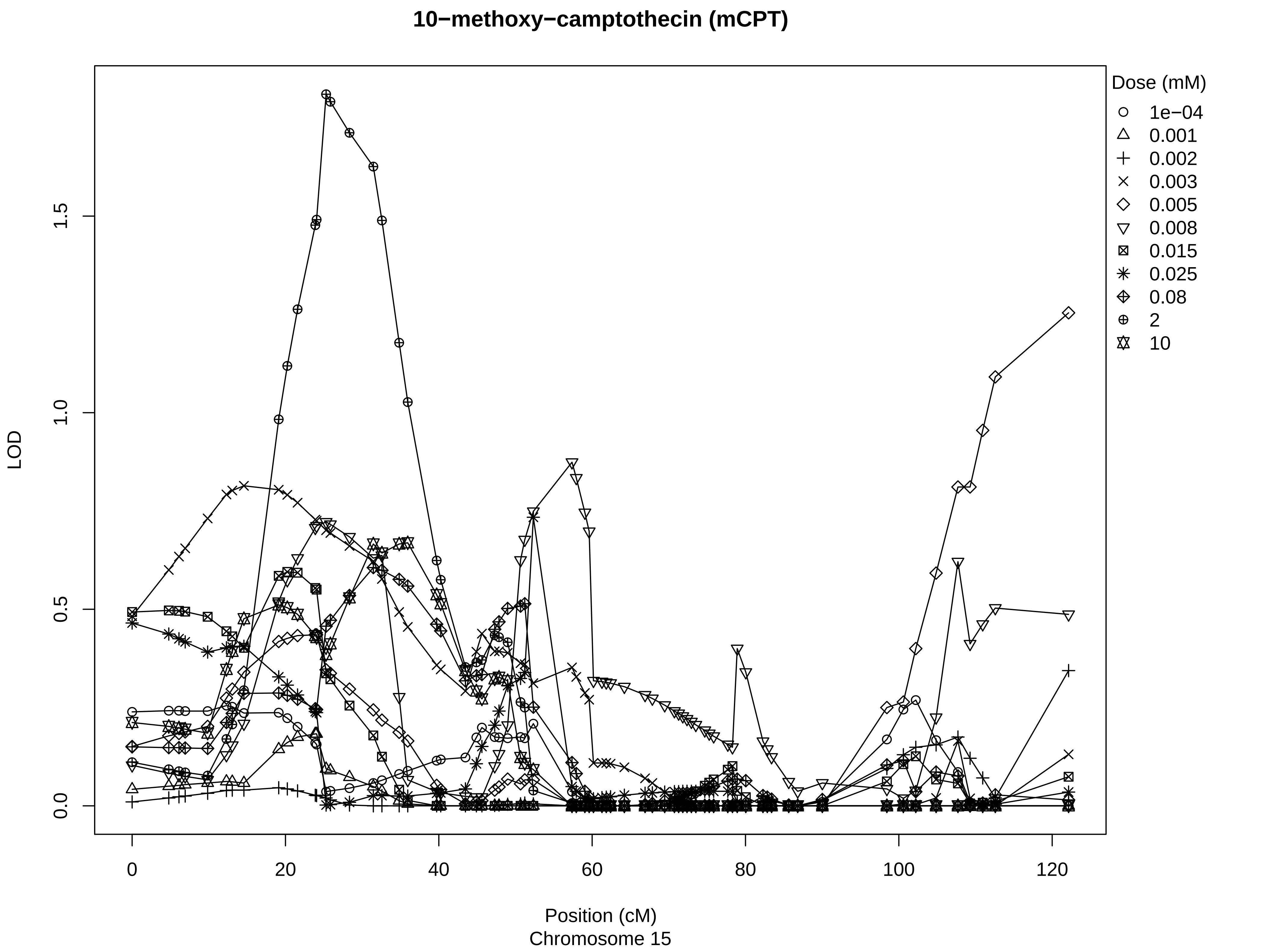

10−methoxy−camptothecin (mCPT)
Dose (mM)
1e−04
0.001
0.002
0.003
0.005
0.008
0.015
0.025
0.08
2
10
LOD
0.0
0.5
1.0
1.5
0
20
40
60
80
100
120
Position (cM)
Chromosome 15

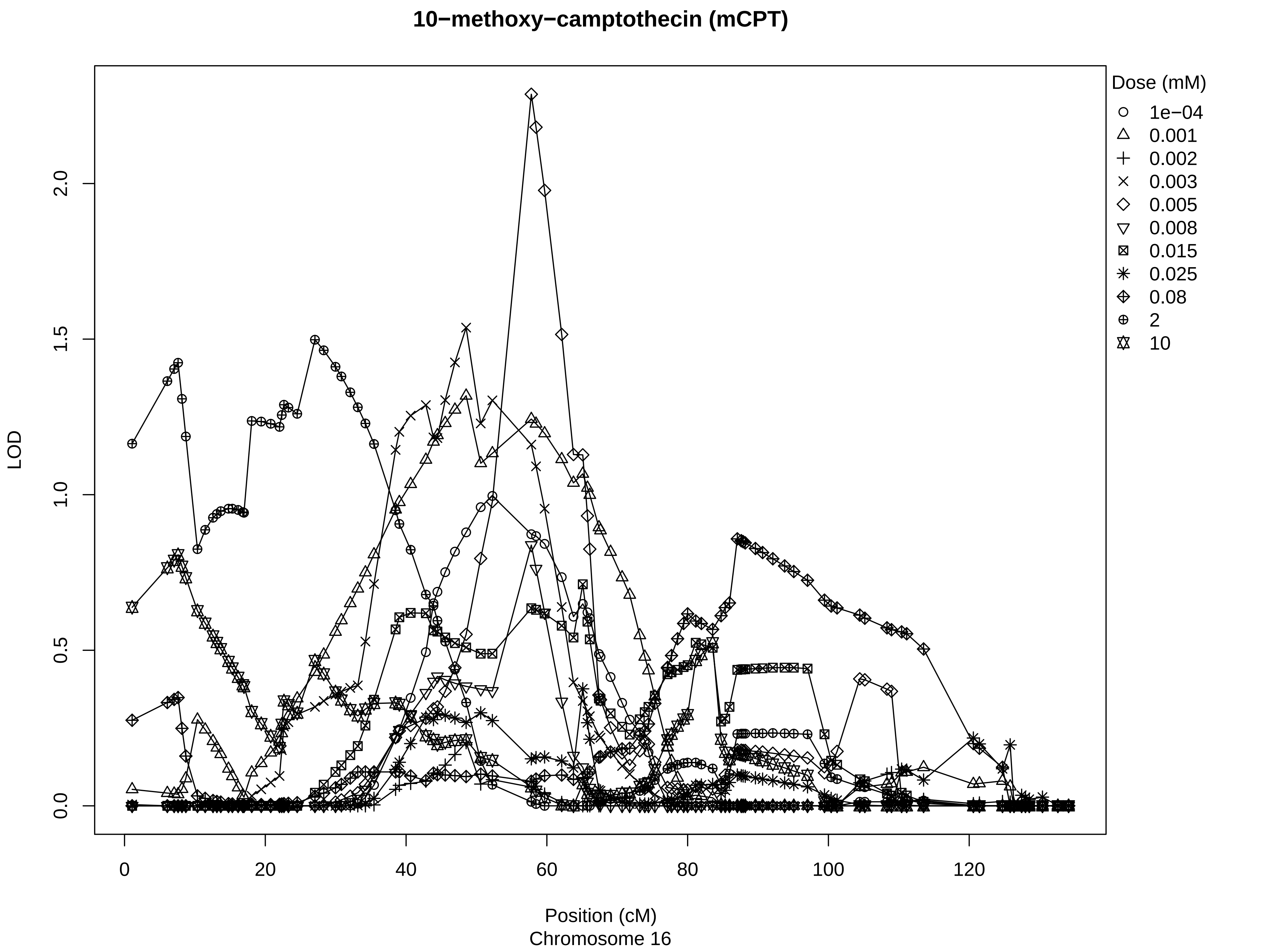

10−methoxy−camptothecin (mCPT)
Dose (mM)
1e−04
0.001
0.002
0.003
0.005
0.008
0.015
0.025
0.08
2
10
LOD
0.0
0.5
1.0
1.5
2.0
0
20
40
60
80
100
120
Position (cM)
Chromosome 16

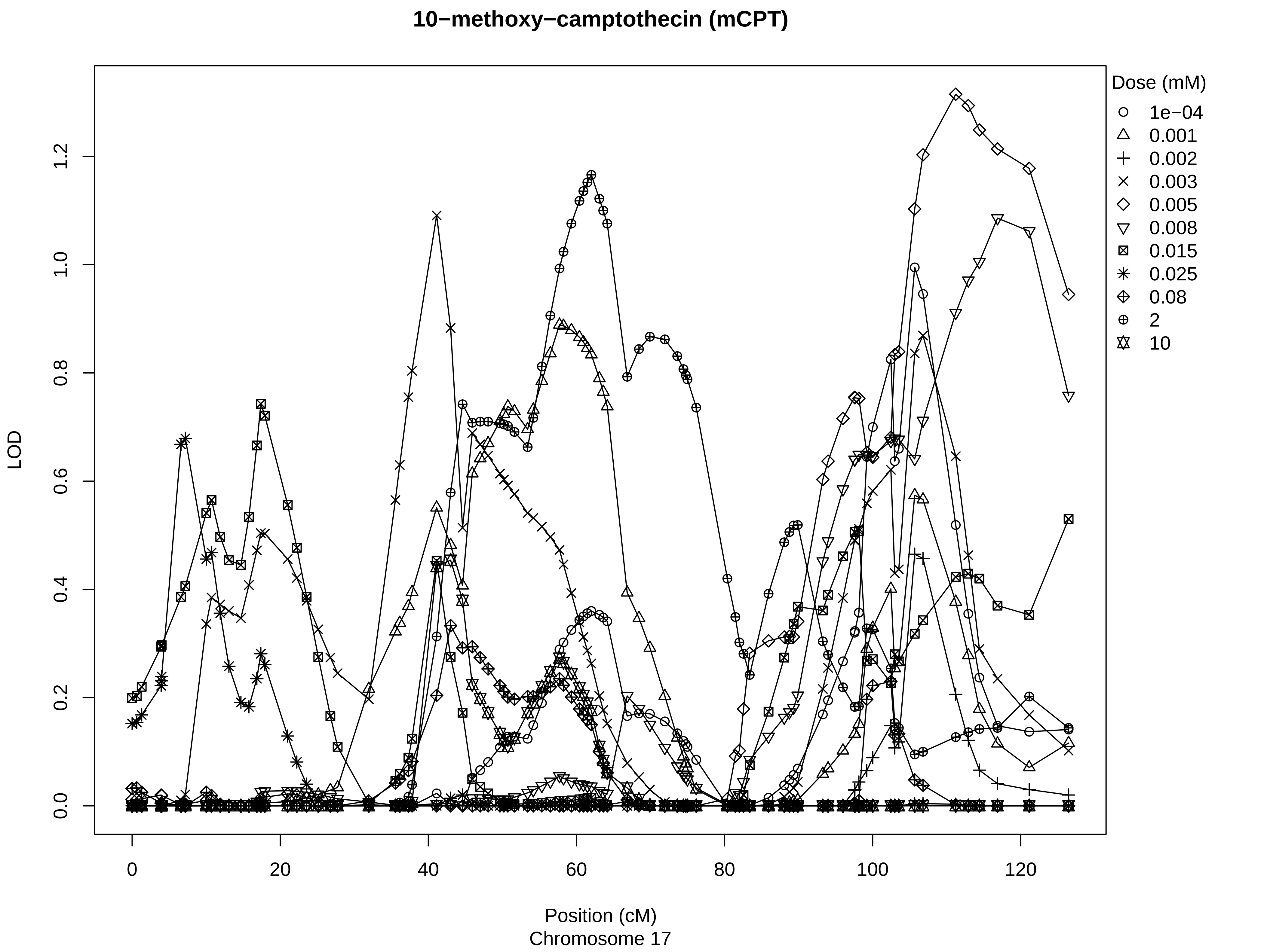
10−methoxy−camptothecin (mCPT)
LOD
0.0
0.2
0.4
0.6
0.8
1.0
1.2
0
20
40
60
80
100
120
Position (cM)
Chromosome 17
Dose (mM)
1e−04
0.001
0.002
0.003
0.005
0.008
0.015
0.025
0.08
2
10

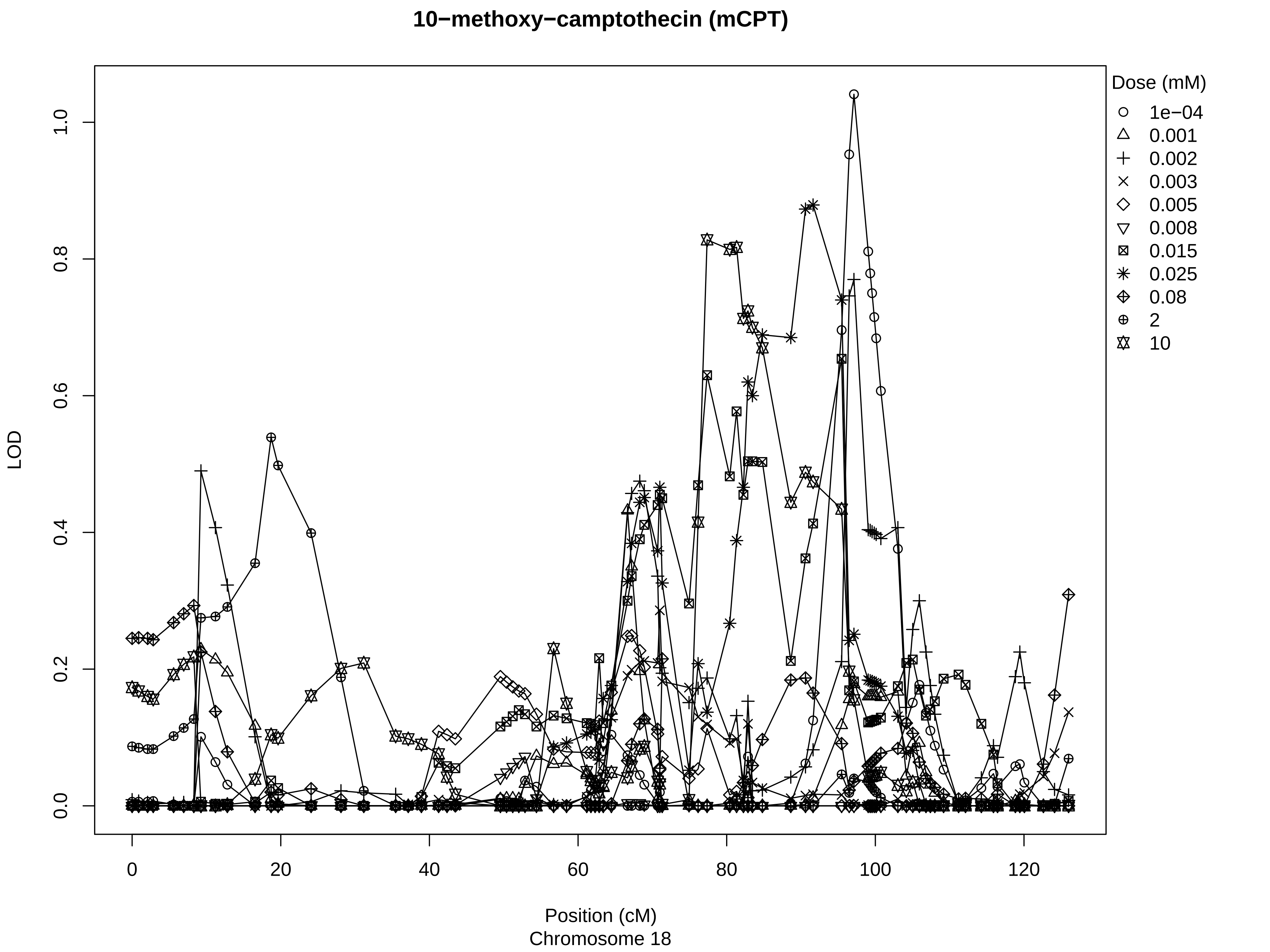
10−methoxy−camptothecin (mCPT)
LOD
0.0
0.2
0.4
0.6
0.8
1.0
0
20
40
60
80
100
120
Position (cM)
Chromosome 18
Dose (mM)
1e−04
0.001
0.002
0.003
0.005
0.008
0.015
0.025
0.08
2
10

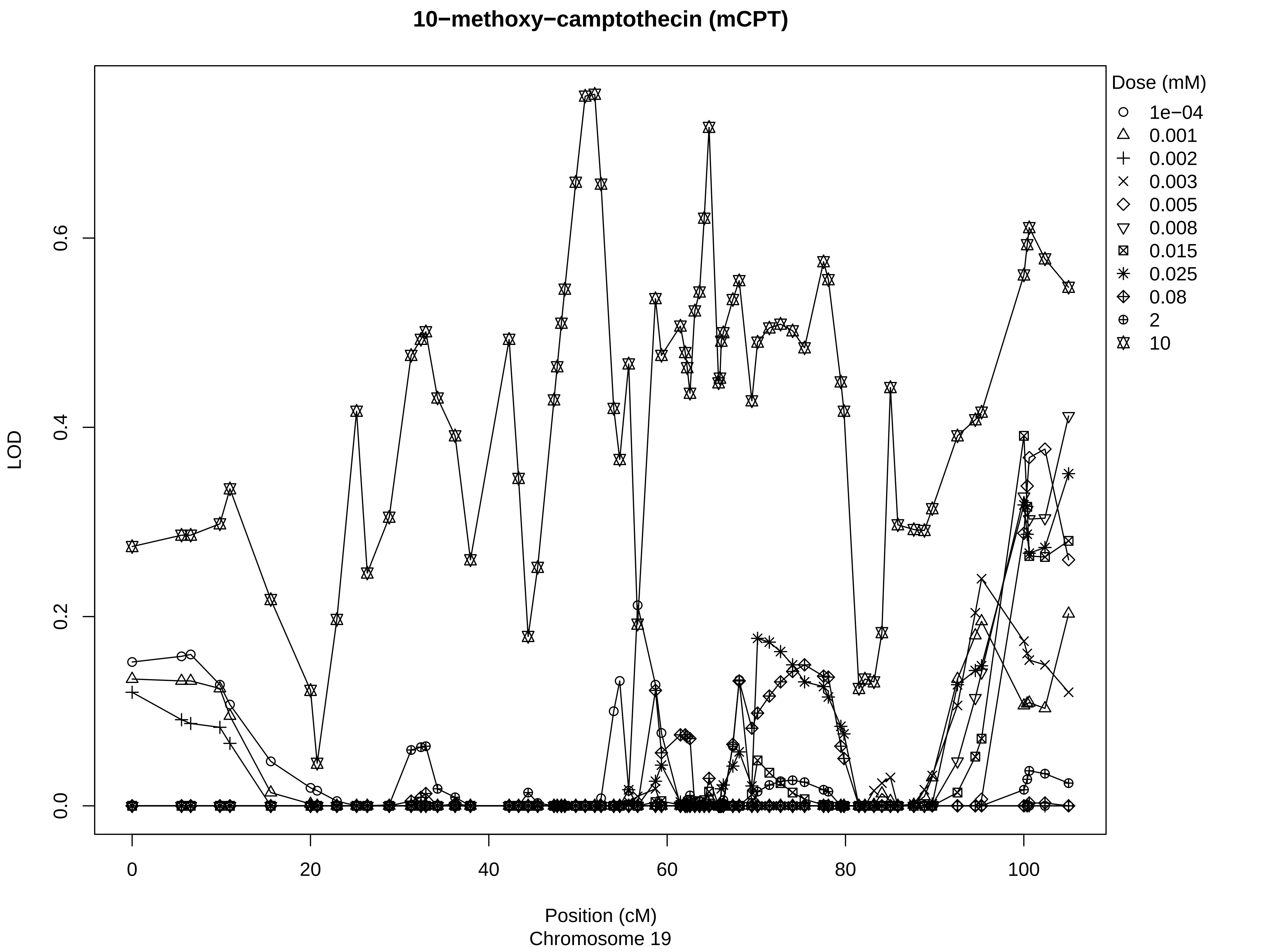

10−methoxy−camptothecin (mCPT)
Dose (mM)
1e−04
0.001
0.002
0.003
0.005
0.008
0.015
0.025
0.08
2
10
LOD
0.0
0.2
0.4
0.6
0
20
40
60
80
100
Position (cM)
Chromosome 19

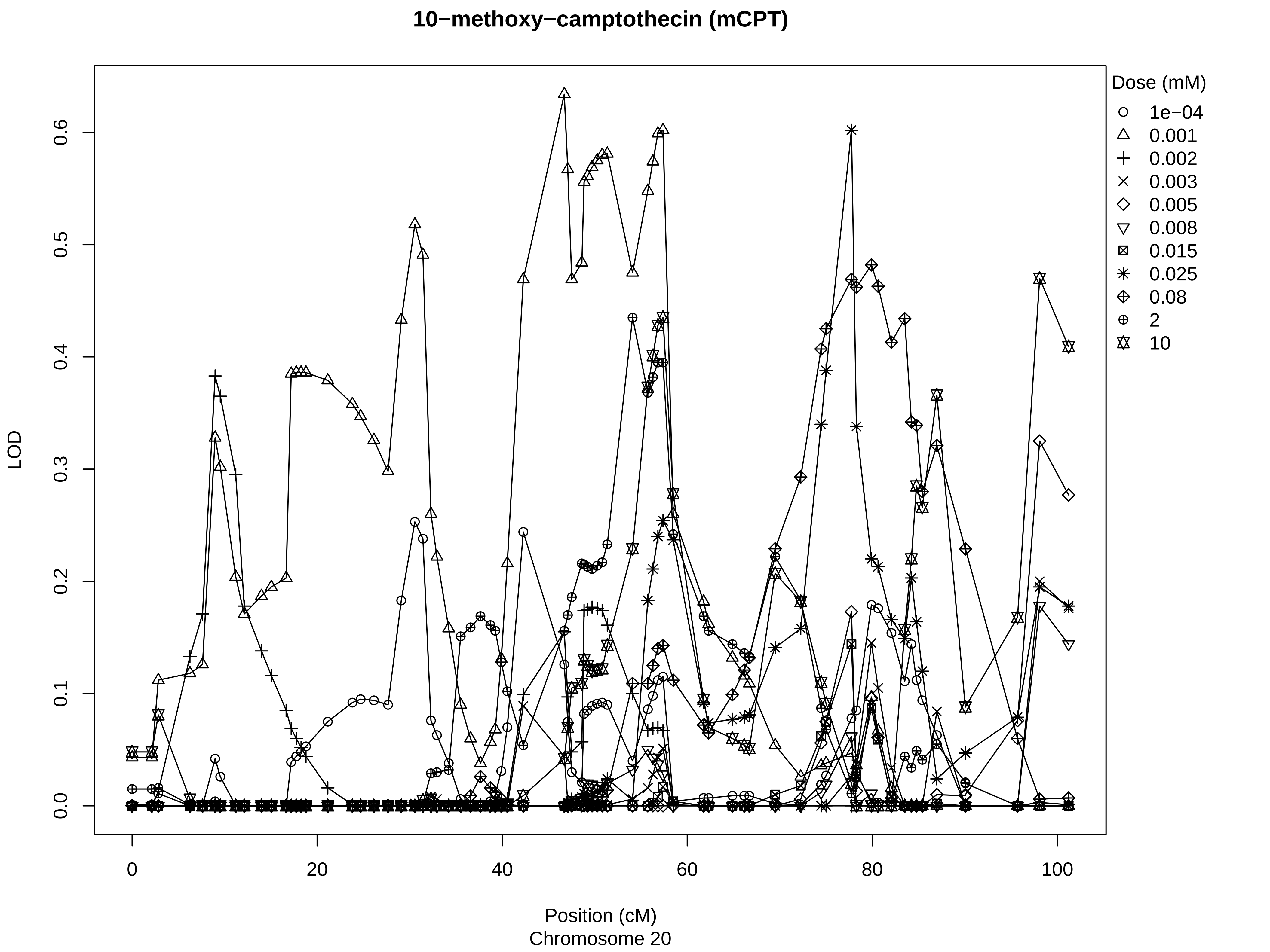

10−methoxy−camptothecin (mCPT)
LOD
0.0
0.1
0.2
0.3
0.4
0.5
0.6
0
20
40
60
80
100
Position (cM)
Chromosome 20
Dose (mM)
1e−04
0.001
0.002
0.003
0.005
0.008
0.015
0.025
0.08
2
10

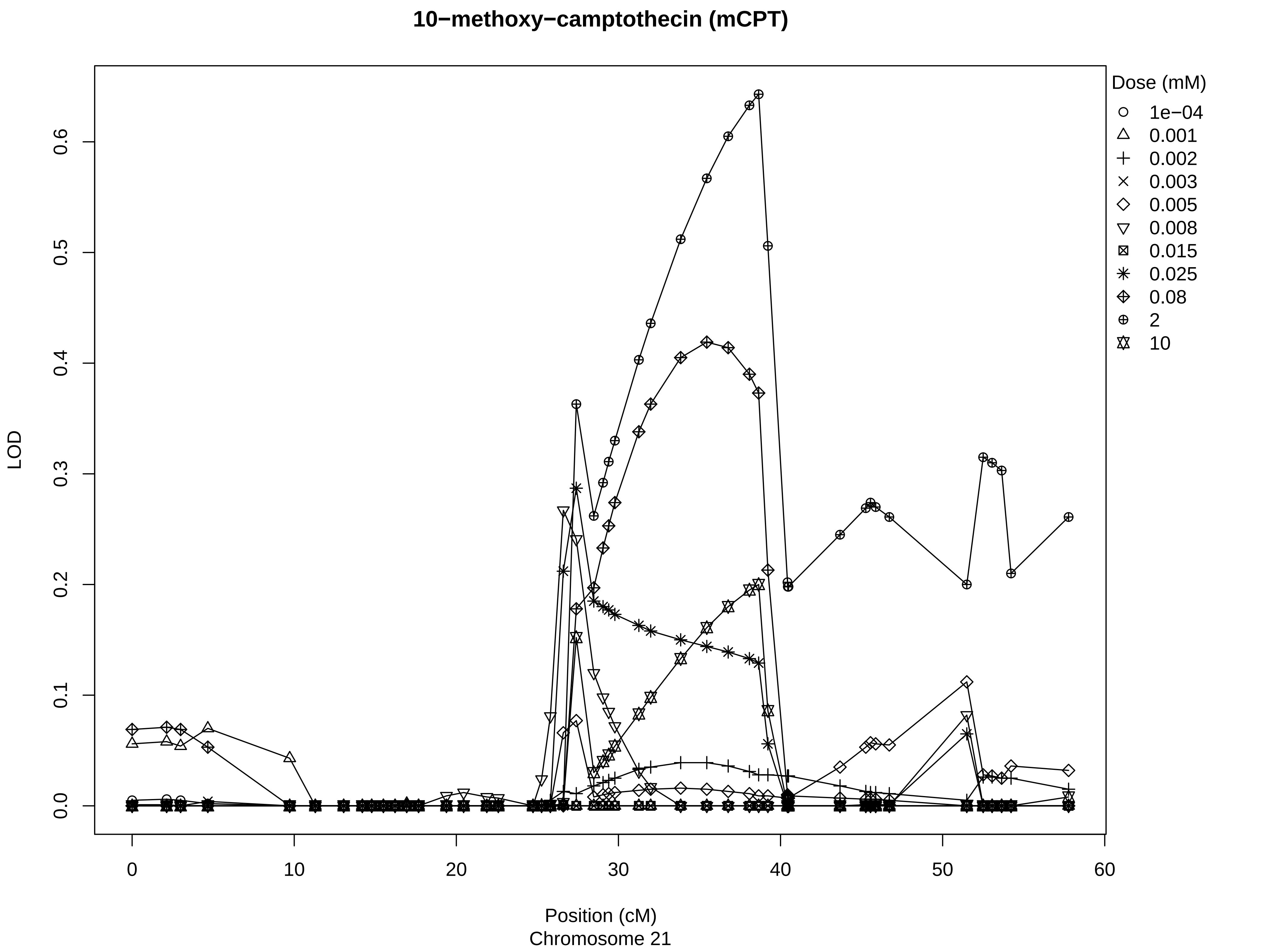
10−methoxy−camptothecin (mCPT)
LOD
0.0
0.1
0.2
0.3
0.4
0.5
0.6
0
10
20
30
40
50
60
Position (cM)
Chromosome 21
Dose (mM)
1e−04
0.001
0.002
0.003
0.005
0.008
0.015
0.025
0.08
2
10

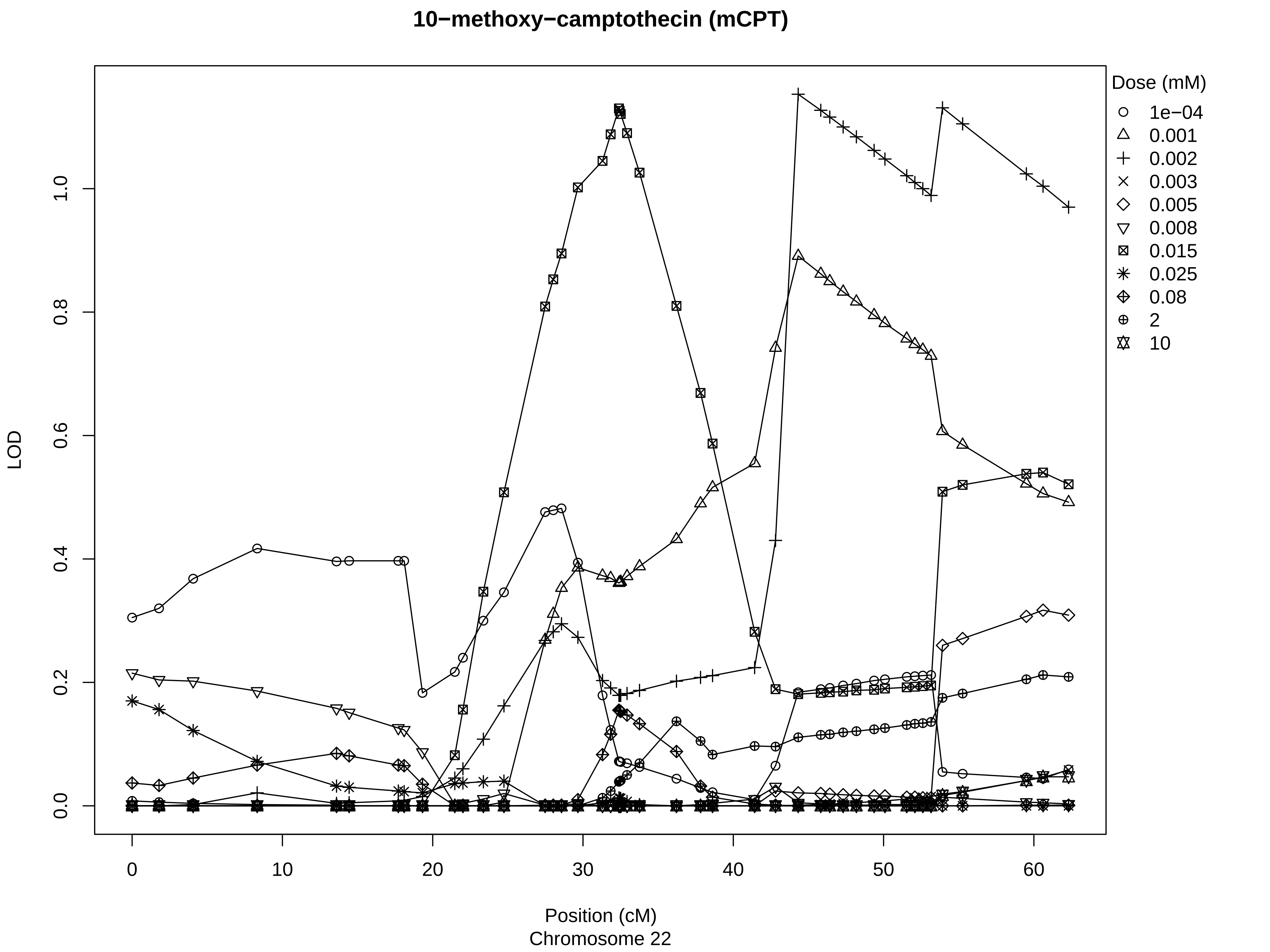
10−methoxy−camptothecin (mCPT)
LOD
Position (cM)
Chromosome 22
Dose (mM)
1e−04
0.001
0.002
0.003
0.005
0.008
0.015
0.025
0.08
2
10
0.0
0.2
0.4
0.6
0.8
1.0
0
10
20
30
40
50
60
